# Music recommendation algorithms based on knowledge graph and multi-task feature learning

**DOI:** 10.1038/s41598-024-52463-z

**Published:** 2024-01-24

**Authors:** Xinqiao Liu, Zhisheng Yang, Jinyong Cheng

**Affiliations:** 1 School of Music, Qufu Normal University, Rizhao, 276826 China; 2 Faculty of Computer Science and Technology, Qilu University of Technology (Shandong Academy of Sciences), Jinan, 250353 China

**Keywords:** Engineering, Mathematics and computing, Information theory and computation

## Abstract

During music recommendation scenarios, sparsity and cold start problems are inevitable. Auxiliary information has been utilized in music recommendation algorithms to provide users with more accurate music recommendation results. This study proposes an end-to-end framework, MMSS_MKR, that uses a knowledge graph as a source of auxiliary information to serve the information obtained from it to the recommendation module. The framework exploits Cross & Compression Units to bridge the knowledge graph embedding task with recommendation task modules. We can obtain more realistic triple information and exclude false triple information as much as possible, because our model obtains triple information through the music knowledge graph, and the information obtained through the recommendation module is used to determine the truth of the triple information; thus, the knowledge graph embedding task is used to perform the recommendation task. In the recommendation module, multiple predictions are adopted to predict the recommendation accuracy. In the knowledge graph embedding module, multiple calculations are used to calculate the score. Finally, the loss function of the model is improved to help us to obtain more useful information for music recommendations. The MMSS_MKR model achieved significant improvements in music recommendations compared with many existing recommendation models.

## Introduction

As artificial intelligence (AI) continues to advance, various types of music software applications have emerged, offering convenience to users^1^. The rapid growth in the music business has enriched existing music libraries and expanded music categories, leading to significant shifts in user preferences for music^2^. However, a major challenge in promoting the music business currently lies in efficiently extracting music matching users' preferences from an almost explosion-like music database and providing accurate personalized recommendations^3^. While music recommendation algorithms emphasize audio features, highlighting music styles and artists, film recommendation algorithms prioritize multiple data features, considering aspects such as film genres, directors, and actors. In contrast, book recommendation algorithms leverage text features, focusing on book themes and authors. These differences result in varying considerations of user preferences, where music recommendation algorithms excel in feature extraction and personalized recommendations, catering to diverse music preferences.

In recent years, there has been an explosion of audio, text, video, and image information^4^. However, this information explosion is both an opportunity and a challenge for people. Such an information explosion provides access to more information for analysis and use; the challenge is how this information can be accessed effectively for use and how the useful information that existed in a large amount of information can be extracted in a reasonable way^5^. Taking music recommendations as an example, a music library contains various categories, including themes, scenes, moods, eras, genres, and languages. Each of these categories are divided into various subcategories: for instance, themes include KTV gold songs, internet songs, love songs, and DJ songs; moods are divided into sad, happy, inspirational, healing, and frantic; this is also true for other categories. The same categories of scenes, eras, genres and languages also contain many smaller categories, each containing countless songs^6^. Selecting the right songs for users from these many categories to make accurate recommendations is challenging because of the existence of various music platforms, such as Free Music Archive, Musopen, SoundCloud CC0, NetEase Cloud Music, and Cool Music^7^. When one goes shopping in a mall, if the seller can provide an appropriate recommendation to the buyer, they would prefer to go to that seller. Similarly, if a music website can recommend the exact songs that a user listens to and the type of songs that the user likes, the music website will be bookmarked as a popular music website for the user^8^.

**Figure 1:**
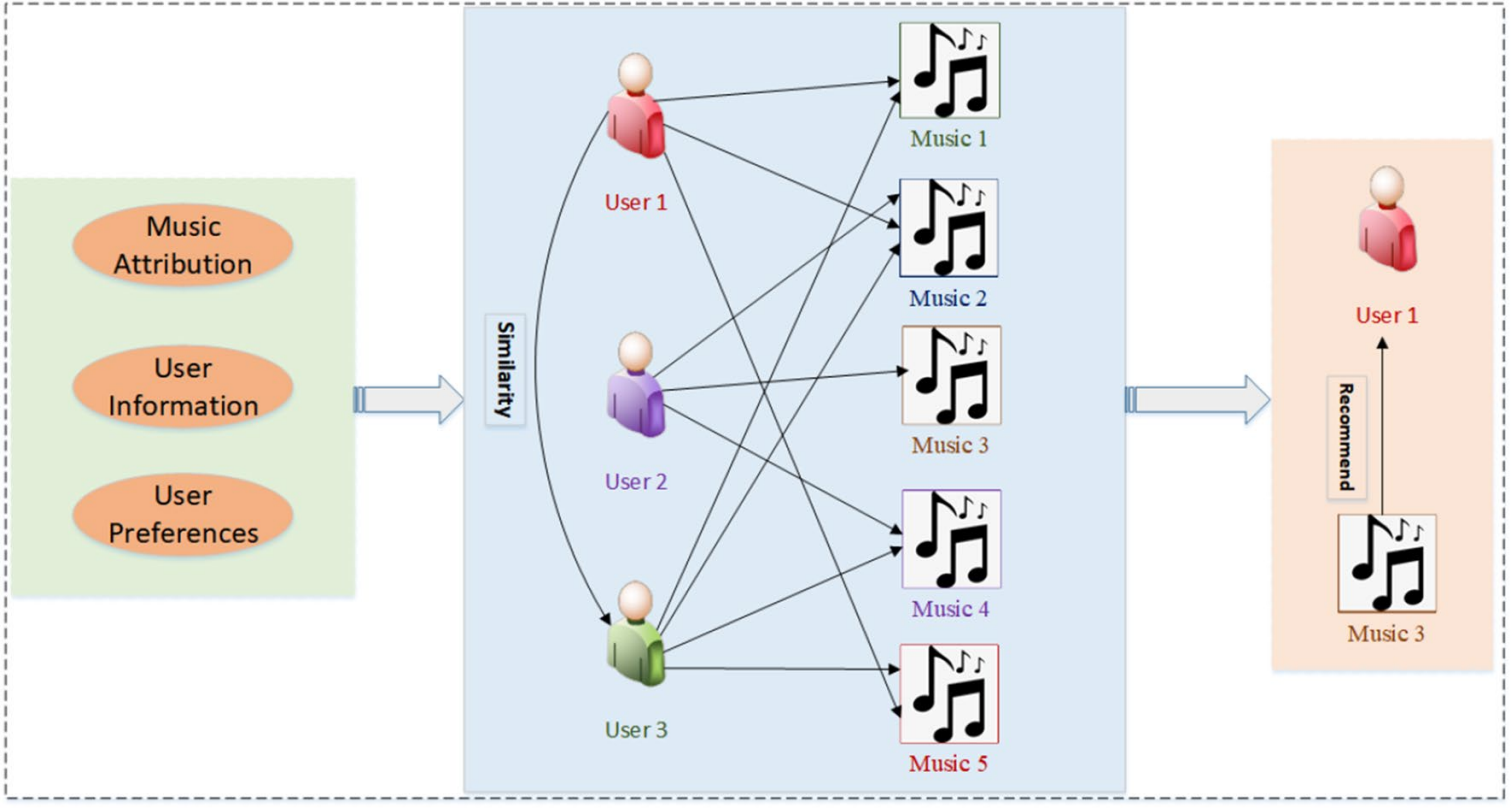
Collaborative filtering algorithm.

Researchers have introduced new auxiliary information to improve the accuracy of music recommendations. This information includes social network information, textual data, and external knowledge graphs, which can be used to make music-recommendation systems more personal. To obtain more auxiliary information, researchers have built various network architectures, including three types of neural networks–heterogeneous networks, attribute multiplexing heterogeneous networks, and knowledge graphs–to explore users' potential interests and make accurate music recommendations, as shown in Fig. 1^9–11^. Collaborative filtering is a common algorithm for music recommendation that is highly personalized and can automatically discover the potential interests of users^12,13^. Notably, the history includes Theuser's listening history, favorite history or search history, and even the information that the user fills- when signing up for a music account^14,15^. Most existing methods for predicting user interest in an item are relatively limited as they rely primarily on calculating the similarity between the user's item and those of others. This can result in different impacts for a single prediction across various application scenarios. In this study, we propose a novel approach that utilizes multiple prediction methods to assess whether a user is interested in an item. By employing a diverse set of prediction techniques, our model achieves greater stability than when relying solely on a single prediction method.

In music recommendation systems, auxiliary information is introduced into a recommendation algorithm to improve music recommendations for users^16^. Obtaining users' potential hobbies through various types of auxiliary information, such as listening history, is a solution to the data sparsity and cold-start problems that can occur in music recommendation systems^14,17^. In music recommendation, data cold starts and sparsity are unavoidable problems. The cold start problem refers to the problem of always encountering new users or items in a recommendation system, where the lack of historical behavioral or item association information for these new users makes it difficult to personalize recommendations^18^. Data sparsity refers to the problem of there being little to no interaction data between users and items, making it difficult for a model to establish an accurate association between them. Traditional music recommendation algorithms are typically implemented using one or more of the following approaches: content-based, collaborative filtering, hybrid, and popularity-based recommendations^19^. These approaches have been widely used to address the challenges of music recommendations and improve their accuracy and relevance^20^. Compared with traditional music recommendation models, knowledge graph recommendations has more data dimensions, deeper semantic information, and more personalized recommendation capabilities; therefore, the knowledge graph can better solve the problems of cold start and data sparsity in traditional music recommendations, and thereby provide a more accurate and richer music recommendation performance^21^.

In our music recommendation system, the multi-task, multi-channel and multi-loss music recommendation knowledge graph model (MMSS_MKR) model is a deep-learning-related multi-task learning framework. Because the music items in a music recommendation system overlap with the entities in the music knowledge graph, we can achieve this by utilizing Q-alternate learning between the music knowledge graph embedding and the recommendation system^22^. Because the knowledge graph embedding and recommendation tasks are both related and separated, the MMSS_MKR model can be used to obtain music-related information between the two modules. Because the entity information obtained from the knowledge graph contains items and their related information, there is a correspondence between the item entity information and the item information in the recommendation module. Therefore, the knowledge graph embedding module is closely related to the recommendation module, and the information extracted from it is highly relevant.

There has been a research gap in leveraging knowledge graph information effectively within music recommendation systems. Existing models have not adequately integrated the rich content of knowledge graphs, limiting a comprehensive understanding of music relations and features. Key issues include underutilization of knowledge graph content, isolating knowledge graph embeddings and recommendation tasks, and lacking an effective framework to integrate both. This limitation restricts the system's comprehension of music features, thus impacting recommendation accuracy. Therefore, establishing a recommendation framework that effectively integrates knowledge graph information is a pressing issue in current research.

This study proposes an end-to-end framework, MMSS_MKR, that uses a knowledge graph as an auxiliary information source to serve the information obtained from the knowledge graph to the recommendation module. The framework utilizes cross & compression units to bridge the knowledge graph embedding task and the recommendation task modules. Notably, our model obtains three-tuple information through the music knowledge graph; the information obtained through the recommendation module is used to determine the truth of the three-tuple information obtained from the knowledge graph, which can obtain as much true three-tuple information as possible and exclude false three-tuple information. The knowledge graph embedding task is used to serve the recommendation task. In the recommendation module, multiple predictions are used to determine recommendation accuracy. The multiple prediction method employed utilizes the dot product of three methods, namely, sigmoid, tanh, and softsign, to generate recommendations; the model generates recommendations to enhance the recommendation process by processing the dotted products of these methods. In the Knowledge Graph Embedding(KGE) module, multiple calculations are performed to calculate the score. The multiple calculation method refers to the utilization of various functions such as sigmoid, tanh, softsign, and softplus when computing the true and false triplet scores of a music triplet. These functions are employed to calculate the scores to enhance the accuracy and effectiveness of the calculation process. The loss function of the model can be improved to obtain useful information for music recommendations. Finally, we apply the MMSS_MKR model to music recommendation and find that the evaluation index Area Under the Curve(AUC) reaches 0.816, and the evaluation index Accuracy(ACC) reaches 0.763 for the Last.FM music dataset. Compared to the currently existing advanced recommendation models, the MMSS_MKR model achieves an AUC gain of 2.38–33.89% and an ACC gain of 1.46–30.30%, showing the effectiveness of the MMSS_MKR model in music recommendation systems based on its evaluation index and recommendation results.

## Related work

### Recommendation system

In the field of deep learning, the application of recommendation algorithms is developing rapidly. Not only is the information applied to recommendation rich, but also the algorithm model applied to recommendation has been improved step by step. The information used in recommendation is applied to the single application of user and project information, and then to increase the user project interaction information, increase the various types of attribute information of the user project, and then it is the knowledge graph as auxiliary information used in the field of recommendation. Its recommendation algorithm is also from a single collaborative filtering algorithm, slowly introduced deep neural networks, heterogeneous information networks, network embedding, knowledge graph embedding, Generative Adversarial Network(GAN)^23^, Graph Attention Network(GAT)^24^, and multi-task learning and other methods to improve the accuracy of the recommendation, derived from drinking more traps of the recommendation algorithm.

Traditional music recommendation methods usually apply item attribute information to recommendation algorithms; however, this application of item attributes cannot be effectively expanded, and supplementary information is difficult^25^. This study uses a knowledge graph as auxiliary information to apply item attribute information instead of directly applying item attribute information to the recommendation algorithm; this approach is adopted to realize the completion of the knowledge graph and supplement the recommended interaction information, thereby improving the effectiveness of recommendation performance^26^.

The personalized entity recommendation(PER) model represents the connectivity between users and items by extracting features based on meta-paths^27^. In this study, these meta-paths are "user-musician-genre-musician", "user-musician-country-musician" and "user-musician-age musician". The advantage of the PER model lies in providing personalized entity recommendations to users, yet its performance may be affected by data sparsity and cold-start issues. The signed heterogeneous information network embedding(SHINE) model uses auto encoders for user-item interactions and item profiles to predict click probabilities. The SHINE model's advantage lies in effectively capturing signed relations in complex heterogeneous networks, yet it might face challenges in scalability and handling large-scale networks due to increased computational complexity.

The deep knowledge-aware network(DKN) model treats entity embeddings and word embedding as multiple channels and combines them into a Convolutional Neural Network(CNN) for Click-Through Rate(CTR) prediction^28^. The DKN model excels in leveraging explicit knowledge graphs for improved recommendation accuracy, but its effectiveness may be limited by the availability and quality of knowledge graphs in certain domains. Collaborative knowledge-based embedding(CKE) combines Collaborative Filtering(CF) with structural, textual, and visual knowledge in a unified recommendation model^29^. The advantage of the CKE model lies in its ability to combine collaborative filtering with knowledge graph embedding to enhance recommendation effectiveness, yet its performance may be affected by incomplete or noisy knowledge graphs. Library for factorization machines(LibFM)^30^ is a feature-based decomposition model widely used in CTR scenarios. The advantage of the LibFM model lies in its capability to handle high-dimensional sparse data and flexibly learn feature interactions, yet it might face challenges in addressing nonlinear problems and handling large-scale data. Wide & Deep Learning for Recommender Systems(Wide&Deep) is a deep learning model that combines linear (wide) channels with non-linear (deep) channels. The (Wide & Deep) model combines memorization and generalization for effective recommendation, yet its performance might be affected by complex feature engineering and limited capability to handle cold-start scenarios.

To better represent the effect of the different models, we created a literature review table, as shown in Table 1.

**Table 1:** Literature review table.

Work	Cold-start/data sparsity	Ancillary information	Multiple forecasts	Main contributions
Steffen et.al., 2012	No	No	No	Provides a flexible and efficient recommendation algorithm framework for building personalized recommendation systems
Xiao et al., 2014	Yes	No	No	Using the structure and propagation process of the user-item graph, combined with user's personalized preferences, it provides a graph-based personalized recommendation algorithm to address the issues of cold-start and data sparsity
Cheng et al., 2016	Yes	Yes	No	By combining breadth and depth models, the accuracy and reach of personalized recommendations are balanced
Zhang et al., 2016	Yes	Yes	No	The fusion of structural information from knowledge graphs and user behavior data from collaborative filtering enables the effective combination of knowledge graphs and recommendation systems, improving recommendation accuracy and recommendation interpretation
Wang et al., 2018	Yes	Yes	No	Leveraging semantic information from knowledge graphs to enhance the representation learning capability of recommender systems
Wang et al., 2018	Yes	Yes	No	An end-to-end recommendation system that effectively captures user-item interactions by introducing interaction attention mechanisms and neighborhood propagation strategies
Wang et al., 2019a	Yes	Yes	No	Joint learning of multiple tasks and multiple relationships in a recommender system is achieved through multi-task learning and multi-relationship modelling
Wang et al., 2019b	Yes	Yes	No	Combining knowledge graphs and graph neural networks to improve knowledge representation learning and recommendation in recommender systems by means of label smoothing regularization
Ryosuke et al., 2020	Yes	Yes	No	Combining graph convolutional networks with knowledge graphs for effective modelling of user interests and item associations in recommender systems by learning the representation of nodes and the propagation of relationships
Our proposed method	Yes	Yes	Yes	The knowledge graph is used as auxiliary information in the recommendation system, while multi-task learning methods are combined to enrich the interactive information used for recommendation, multiple calculations are used to increase the proportion of true triad scores, and multiple prediction methods are used to enhance the accuracy of the prediction results

### Knowledge graph

A knowledge graph is a powerful network that connects diverse information and represents interactive relationships in a structured manner. It comprises nodes, which represent real-world entities, and edges, which represent the relationships between entities. Knowledge graphs can be categorized into general and industry-specific knowledge graphs, based on their depth and focus. General knowledge graphs are commonly used in applications such as search engines, question-answering systems, and recommendation engines, but lack deep industry-specific or professional content. Industry knowledge graphs are developed for specific fields or industries to provide comprehensive and in-depth representations of industry-specific knowledge; these are often utilized in analytical applications and decision support systems.

In the context of recommendation algorithms, the most commonly applied knowledge graphs are generic knowledge graphs, which can be classified into two types: user-item knowledge graphs and item knowledge graphs. User-item knowledge graphs contain information on users, items, and their attributes, whereas item knowledge graphs contain solely item and attribute information. Leveraging knowledge graphs in recommendation algorithms has significantly contributed to improving the accuracy of recommendation performance. Recommendation systems can generate more accurate and relevant recommendations by utilizing the relationships and attributes captured in the knowledge graph.

In addition, the knowledge graph can prevent the music recommendation system from overfitting, improve the generalization ability of the music recommendation system, and help eliminate local minima. This is because the entity and relationship information in the knowledge graph provides the model with rich knowledge of the music domain and contextual information, which can improve the ability of the music recommendation system to obtain relevant information about all users and music. Compared to traditional music recommendations, knowledge graphs can provide more comprehensive information to improve the generalization ability of the model and reduce its reliance on specific data, thus preventing overfitting. Simultaneously, as the music knowledge graph provides a wealth of music domain knowledge and rules, it provides additional constraints and guidance to the music recommendation system, helping the system go beyond local minima and find better recommendation strategies.

**Figure 2:**
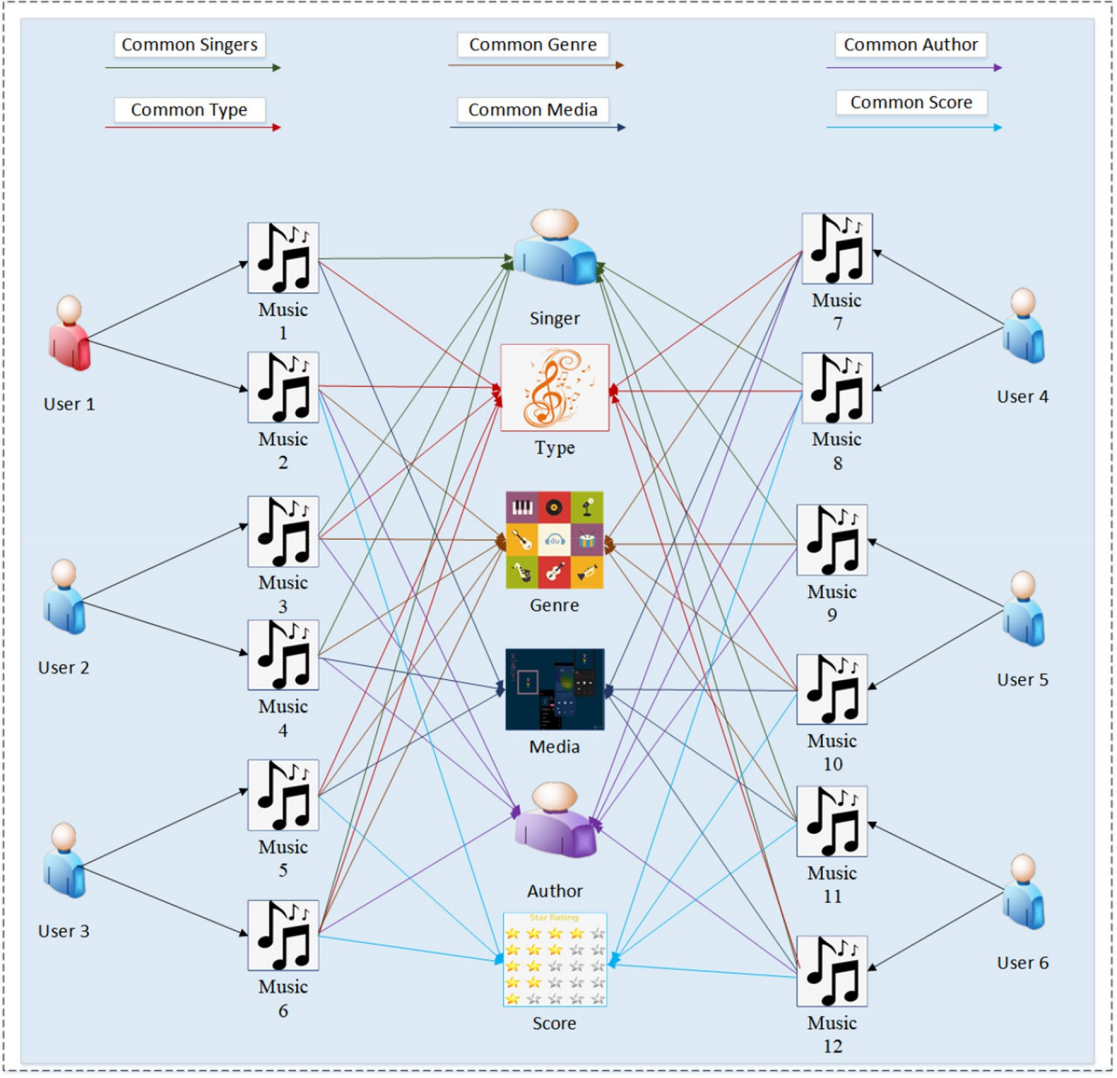
Music recommendation knowledge graph.

The music knowledge graph used in this paper is shown in Fig. 2, which demonstrates the correlation between music items and also the information related to entities and relationships. In a music knowledge graph, a triple is a data structure consisting of three elements used to describe relationships between entities within the music domain. These elements are:

Subject: Represents an entity, such as the name or identifier of a musician, band, album, or song.

Predicate: Denotes the relationship or attribute between the subject and object, such as "member," "release date," "genre," etc.

Object: Relates to the subject and can be another music entity, a date, genre, location, or any attribute associated with the subject.

Through this triple structure, it becomes possible to clearly depict relationships and attribute information among different music entities, aiding in the organization, comprehension, and utilization of the rich information within the music domain.

In order to apply the information contained in the knowledge graph usually use knowledge graph embedding methods to realize the information contained in it, and the commonly used knowledge graph embedding methods include Translating Embeddings for Modeling Multi-relational Data(TransE)^31^, Knowledge Graph Embedding by Translating on Hyperplanes(TransH), Learning Entity and Relation Embeddings for Knowledge Graph Completion(TransR), Multi-Layer Perceptron(MLP), and neural network models.

Currently, there have been numerous recommendation algorithms based on knowledge graphs. Table 1 presents various recommendation algorithms based on knowledge graphs. Propagating user preferences on the knowledge graph for recommender systems(RippleNet) makes recommendations for the knowledge graph by acquiring more information about three tuples, and thus mining more information about users' potential preferences^32^. The RippleNet model excels in leveraging user preferences across the knowledge graph for effective recommendations, yet it might face limitations in scalability and handling diverse user behaviors. Multitask feature learning for knowledge-graph-enhanced recommendation(MKR) is a deep end-to-end framework. MKR-1L is an MKR with an FM model with one layer of Cross&Compression Units. MKR-DCN is an EQ-based variant of the MKR corresponding to the DCN model^33^. MKR-stitch is another variant of MKR that corresponds to a cross-stitch network, where the transfer weights in EQ^34^ are replaced by four trainable scalars. The MKR model and its variants exhibit strong adaptability by jointly learning from knowledge graphs and recommendation tasks; however, they may encounter challenges in handling sparse or noisy knowledge graphs and large-scale data for effective recommendations.

Knowledge graph convolutional networks(KGCN)^35^ is a graph neural network model based on knowledge graphs which is designed to learn and represent the semantic correlations between entities and relations in the knowledge graph. The KGCN model excels in learning representations from knowledge graphs for recommendation, yet it might face challenges in handling large-scale and dynamically evolving knowledge graphs efficiently. Knowledge graph neural network with label smoothness(KGNN-LS)^36^ is a graph neural network model that can introduce label smoothing regularization into knowledge graph recommendation algorithms. The KGNN-LS model effectively incorporates label smoothness in knowledge graph embedding; however, its performance could be impacted by the quality and incompleteness of the knowledge graph data.

### Multi-task learning

Multi-task learning(MTL) is a machine-learning approach that aims to simultaneously learn multiple related tasks to enhance the model performance through information and representation sharing. In contrast to traditional single-task learning, MTL enables knowledge transfer and sharing among different tasks, thereby improving the generalization and efficiency of the model.

In multitask learning, a primary task typically exists alongside multiple secondary tasks. The primary task represents the main objective of the model, whereas the secondary tasks provide additional information and constraints that aid in the learning process of the primary task. By sharing the underlying representation or feature extractor, the model can learn shared features from multiple tasks and can be trained by minimizing the combined loss function of all tasks.

In the realm of recommendation algorithms, applying a multitask learning approach to models can enhance their generalization and efficiency by leveraging the information and representations shared among multiple related tasks. By sharing knowledge regarding user preferences and item characteristics across tasks, the model can gain a deeper understanding of the underlying patterns and dependencies; this in turn improves the accuracy and effectiveness of the recommendation system.

The application of multi-task learning in recommendation algorithms is widespread, allowing integration with knowledge graphs for recommendation^33^, heterogeneous networks for recommendation^37^, attention mechanisms for recommendation^38^, as well as combinations with various other methods to enhance recommendation systems^39,40^.

## Model

The MMSS_MKR model consists of three main components: the knowledge graph embedding module, the recommendation module, and the cross & compression units. It leverages knowledge graph data for music recommendations, embedding entity relation vectors, and generating personalized recommendation lists to improve accuracy and effectiveness. In this model, the role of the knowledge graph embedding task is to support the recommendation task by constructing a music recommendation knowledge graph and integrating it into the model. The foundation of this model relies on two key factors. Firstly, the knowledge graph serves as an auxiliary information source for the recommendation module. Secondly, the entity information within the knowledge graph correlates with the item attributes in the recommendation module. Through joint training of these two information sources, they complement each other, enriching the knowledge graph and enhancing the details of item attributes in the recommendation module. Thus, this method effectively harnesses the synergy between the knowledge graph and item attributes, ultimately enhancing the recommendation performance.

### Knowledge graph construction

Knowledge graph is a data structure used to represent relationships between entities, containing a plethora of entities (such as things, concepts, or people) and their connections or attributes. This graph-like structure is represented in the form of nodes and edges in graph theory, where nodes represent entities, and edges denote the relationships between entities.

As a source of supplementary information, the process of building the knowledge graph can be divided into two parts: knowledge acquisition and mapping construction. The knowledge acquisition process includes structured data extraction and cleaning. Structured data refer to data that exist in a predefined format with a fixed organization; unstructured data, however, lack a predefined organization and can take various forms; semi-structured data fall between structured and unstructured data, possessing some organizational features but without a rigid structure. Then, the mapping process is performed, which includes knowledge representation, triple histidization, entity fusion, and association edge building. The knowledge representation component represents information regarding a music item in a computer-processable manner. Triplisation is the process of transforming music-related entities and relationships in a music knowledge graph into a triad. Entity fusion is the process of merging duplicate entities in a music knowledge graph into a unique entity, with the goal of eliminating duplicate information and increasing the accuracy of the information available. Relationship edge establishment is the final step in the construction of the music knowledge graph; it defines the types of relationships and attributes between entities, thus completing the complete music knowledge graph network structure.

The music knowledge graphs built by us can be used in several aspects, including mining more information about users and music, exploring relationships between users and items, and exploring concepts–that is, directly accessing information about a user's favorite music. The knowledge graph can also be used for knowledge computing, knowledge inference, entity annotation, and concept annotation to better understand knowledge and gain more information for accurate music recommendations. Knowledge reasoning plays an important role in the recommendation domain by improving the accuracy, degree of personalization, and interpretation of recommendation systems. In particular, our music recommendation algorithm enables both the mining and extension of user interests and enhances personalized music recommendations. The information propagation process in a music knowledge graph can also be effective for enhancing recommendation interpretation and interpretability.

**Figure 3:**
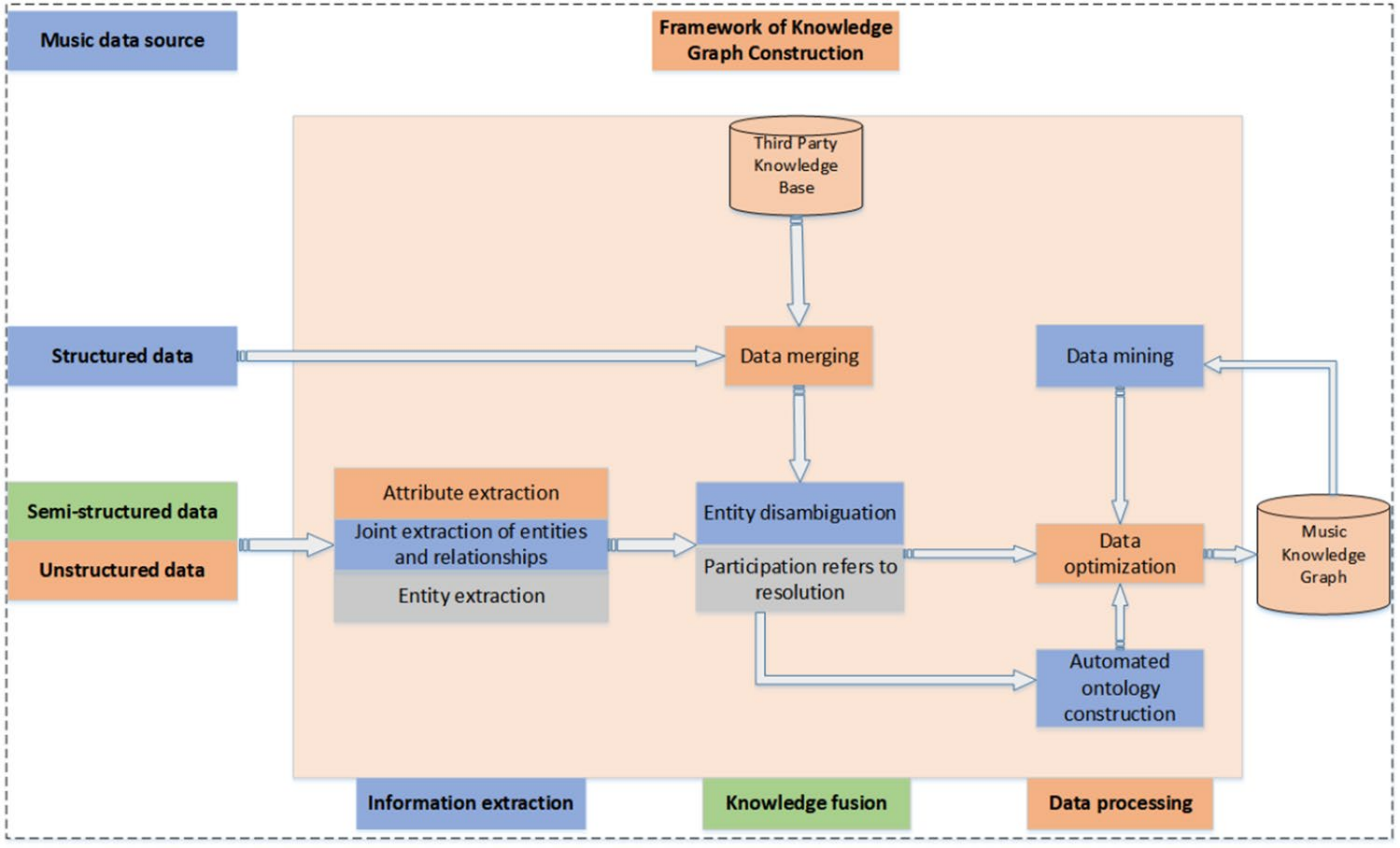
Detailed process of knowledge graph construction.

The construction of music knowledge graph is due to the model needs to apply the semantic relationship between the entities and the ternary information that exists in the knowledge graph, so this paper uses the open-domain FreeBase, of course, if the use of the dataset is less can be constructed on their own. To construct the music knowledge graph as comprehensively as possible, we must check the steps of information extraction, knowledge fusion, and data processing, as shown in Fig. 3. First, the data within the music data source are classified as structured, semi-structured, or unstructured according to their structural levels. Second, the semi-structured and unstructured data with unclear data structures are extracted through attribute extraction, entity extraction, and joint entity and relationship extraction. Knowledge fusion then integrates the information from the tripartite database of structured databases. Third-party databases and the information base are obtained through information extraction of semi-structured and unstructured data into one knowledge base. The main problem to be solved in the integration process is entity alignment, including entity disambiguation and co-reference disambiguation, which aims to integrate different descriptions of entities from different databases to obtain a complete description of the entities, i.e., the final knowledge base. Entity disambiguation can be accomplished through context-based methods, knowledge-based methods, and machine-learning techniques. Co-reference disambiguation, on the other hand, can be achieved using rule-based approaches, machine learning-based approaches, and end-to-end models. This is followed by a top-down approach to building the data organization framework of the knowledge graph, i.e. the data model. Subsequently, through knowledge inference (i.e., data mining), a logical system is used to obtain information regarding potential relationships between users and items based on inference rules^41^. When constructing music knowledge graph through data mining method, the neural network model is used to mine the associated information of music items, which includes singer, genre, genre, media, author and score, and different music items are associated with each other through these attribute information, and this process can obtain more and more accurate associated information of music items for the construction of the knowledge graph. Finally, the data is optimized by data cleaning and de-duplication methods to detect and correct errors, inconsistencies and missing values in the acquired music data, and at the same time removing duplicated music data to prevent duplicated information from appearing in the knowledge graph, and finally placing qualified data that meets our needs into the knowledge graph to complete the construction of the music knowledge graph. The construction process of the music knowledge graph is illustrated in Fig. 3.

In a music knowledge graph, it is possible to gather more information regarding the potential connections between user items. As different songs may have the same artist, genre, album type, media, author, and score and different users listen to different songs, we can use the knowledge graph to explore the potential interests of users through various pieces of information about music and recommend similar, suitable music to users based on the music they have previously listened to. In addition, knowledge graphs usually depict data in the form of triples, which are mainly represented in the form of (entity-relationship-entity) and (entity-attribute-entity) in the music knowledge graph in Fig. 2; we can use three tuples to uncover potential connections between users and music.

The middle part of Fig. 2 shows a simple item knowledge graph constructed from the item and item attribute information. In the figure, music (1–12) all represent entity information, and the attribute and relationship information include six types: common signers, common genre, common author, common type, common media, and common score. It can be observed that the more information there is about the relationship between entities, the stronger the similarity between the two entities; the reverse also holds true. When recommending music to users on the knowledge graph, the music items liked by the users can be connected through the number of entity relationships to recommend music items with higher similarity. As auxiliary information, the knowledge graph improves the accuracy of recommendation performance through information dissemination.

### MMSS_MKR Framework

**Figure 4:**
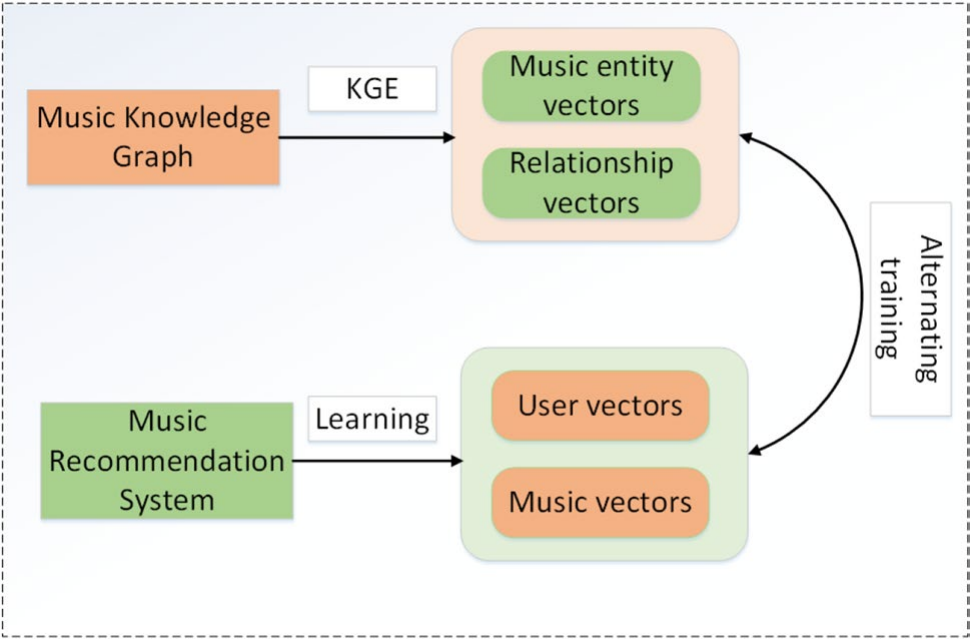
Structure of alternate training of models.

In our music recommendation system, the MMSS_MKR model serves as a multi-task learning framework rooted in deep learning. As the three-tuple information in the knowledge graph corresponds to the user-item data gathered in the recommendation module, we employ alternate learning between the features derived from the music knowledge graph and the recommendation system. This approach helps us acquire more precise insights into users and music items. Alternate learning denotes the practice of cyclically executing distinct tasks or optimizations during the training of a model. Specifically in recommendation algorithms, it involves cycling through the training of various modules or segments. In the context of the MMSS_MKR model, this method specifically focuses on combining knowledge graph embedding and recommendation tasks, allowing optimization between these two modules. The knowledge graph embedding task and the recommendation task are two separate modules, but they are perfectly linked by the existence of Cross&Compression Units. The cross-compression unit module is an effective feature extraction method that reduces redundancy and noise through feature crossover and compression operations, extracting features with greater inertia and thereby improving the effectiveness of the recommendation performance. Therefore, the MMSS_MKR model can be used to obtain music-related information. In addition, knowledge graphs can help music recommendation systems eliminate local minima, prevent overfitting and improve model generalization. Considering our music recommendation system as an example, the alternating training structure of the MMSS_MKR model is illustrated in Fig. 4.

In Fig. 4, the music knowledge graph provides the model with information on musical entities and relationships that contain potential interaction information linking different pieces of musical information. The Music Knowledge Graph can utilize the KGE model to capture the semantic associations between entities and relationships within it. It embeds these entities (such as musicians, bands, albums, songs) and relationships (like members, release times, genres) into a low-dimensional vector space. This embedding representation enables entities and relationships to be represented as continuous vectors in space, allowing computers to better understand and process this interconnected information. The goal of the KGE model is to map entities and relationships from the graph into a lower-dimensional vector space while preserving their semantic relationships, providing more effective data representations. These vector representations can then be leveraged by recommendation systems or other related tasks within the music domain. The music recommendation system provides the required user and item information for the model. It utilize embedding techniques from deep learning (such as MLP) to learn vector representations for users and music items. By training these models and leveraging vast amounts of user behavioral data and music features, the system generates low-dimensional vector representations for users and music items. However, the entity information in the music knowledge graph is related to the item information in the music recommendation system. The purpose of the alternate training of the two modules is to realize the mutual complementarity of the information of the two modules through the association of the information of the two modules; this is the inspiration for the MMSS_MKR model. In order to better represent the model, we have interpreted all the symbols as shown in Table 2.

**Table 2:** Symbol description.

Symbol	Description
*u*	User
*v*	Item
*M*	Number of users
*N*	Number of items
*G*	Knowledge graph
*(h,r,t)*	Three-tuple
*h, r, t*	Head entity, tail entity, relationship
θ	A parameter of the function f
Y	A user-item interaction matrix
W,${{\text{W}}}_{{\text{l}}}^{{\text{VV}}}$,${{\text{W}}}_{{\text{l}}}^{{\text{EV}}}$,${{\text{W}}}_{{\text{l}}}^{{\text{VE}}}$,${{\text{W}}}_{{\text{l}}}^{{\text{EE}}}$	Weight
*b*,${{\text{b}}}_{{\text{l}}}^{{\text{V}}}$,${{\text{b}}}_{{\text{l}}}^{{\text{E}}}$	Bias
S(v),S(u)	A set of entities associated with music item item v (user u )
$f_{{R{\text{S}}}}$	The prediction probability of the recommendation module
$\alpha ()$	The tanh function
$\sigma ()$	The Sigmoid function
$\beta ()$	The softsign function
$\delta ()$	The softplus function
$f_{{{\text{KG}}}}$	The probability prediction of the KGE module
*d*	The dimensions of the hidden layer
${{\text{C}}}_{{\text{l}}}$	The cross feature matrix of layer l
${{\text{v}}}_{{\text{l}}+1}$,${{\text{e}}}_{{\text{l}}+1}$	Item and entity feature vectors for layer l + 1 after cross-compression unit processing
λ1,λ2	The equilibrium parameters
*J*	The cross-entropy function

In the music-recommendation scenario, there are *M* users and *N* music items. The user-item interaction matrix is defined based on implicit feedback from users, in which each entity indicates that user *u* is involved in item *v*, such as the act of clicking, watching, browsing, or buying. Knowledge graph G, comprising three tuples, is structured in the form of entity-relationship-entity. Here, *h*, *r* and *t* denote the head, relationship, and tail of the three-tuple knowledge, respectively. In a music recommendation scenario, items may be associated with one or more entities in G. For example, if Jone listens to the 90s pop music *Listen To Your Heart*, the music recommendation matches with multiple entities in Knowledge Graph(KG) containing Jone, 90s, pop music, and *Listen To Your Heart*. Given a user-item interaction matrix *Y* and a knowledge graph G in a music recommendation system, the ultimate goal is to predict whether user u has a potential interest in a music item that they have not interacted with before; this goal can be achieved through a prediction function $\hat{y}_{uv} = F\left( {u,v\left| {\Theta ,Y,G} \right.} \right)$, where the probability that user *u* will participate in item *v* is $\mathop y\limits^{ \wedge }_{uv}$. *θ* is the model parameter of the function *f*.

**Figure 5:**
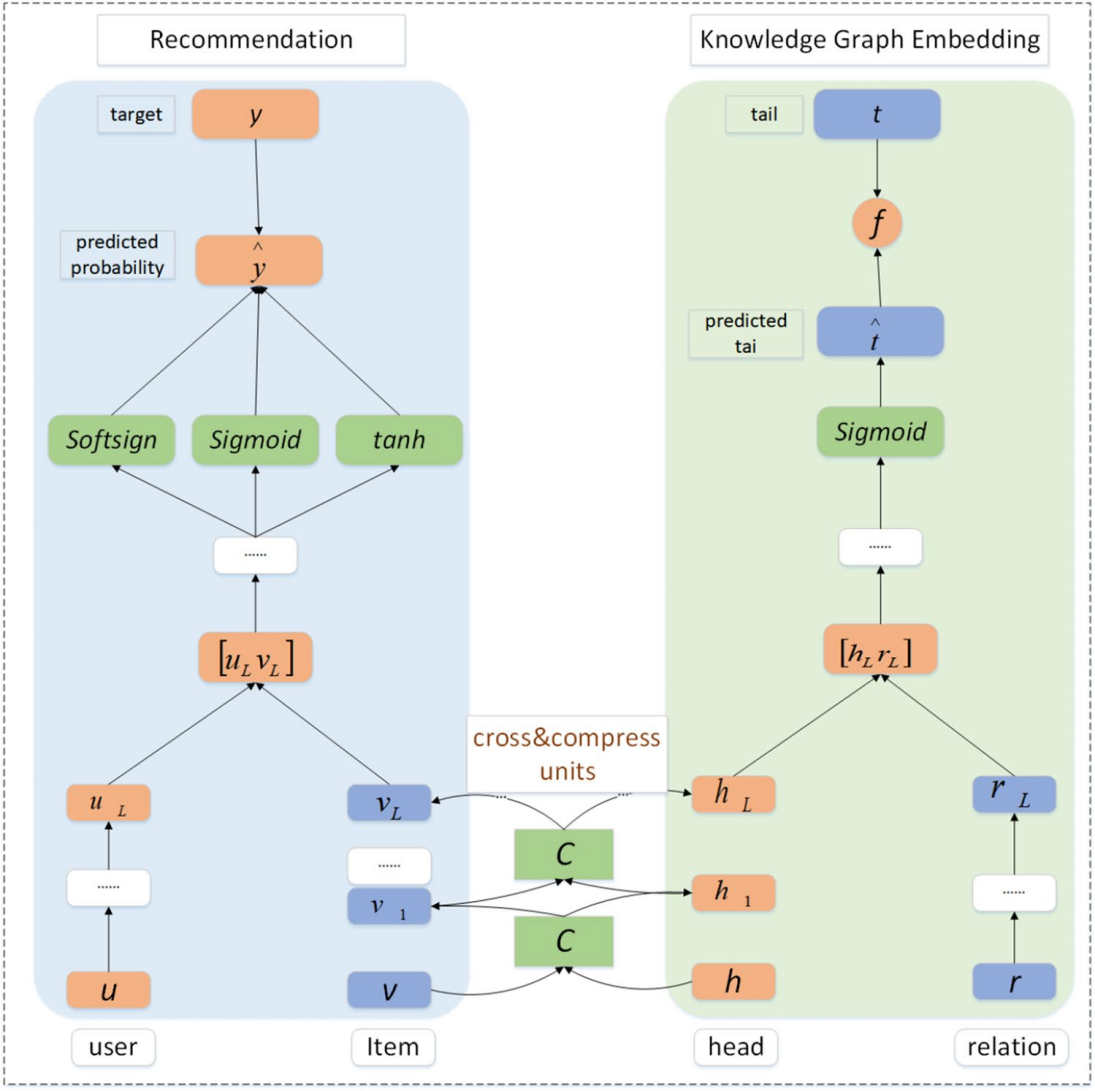
MMSS_MKR model framework: the left side of the figure depicts the recommended module structure, while the right side of the figure represents the KGE module; the bridging of these two modules is achieved by the Cross&Compression Unit.

The MMSS_MKR model consists of three modules: the recommendation module, the KGE module, and Cross&Compression unit, as shown in Fig. 5. In the recommendation module, because of its existence of music item information on the right side of the knowledge graph module have entity counterparts, and the attribute information of the music items in the recommendation module also have entity counterparts in the knowledge graph, and the attribute entities contained in the knowledge graph are more comprehensive, and at the same time, there will be some music items that contain attribute information that is more comprehensive than the music knowledge graph. It is because of the association between the music items in the recommendation module and the entities in the knowledge graph that the MMSS_MKR model can realize the mutual complementation of the information of the two modules when training alternately, and the complemented information not only enriches the knowledge graph, but also makes the information obtained by the recommendation module more comprehensive, which in turn improves the accuracy of the recommendation.

In the recommendation module, the user and the music item are used as inputs. Its input comprises the original feature vectors describing users u and items v, obtained from the user-item interaction matrix Y. Next, a multi-layer perceptron(MLP) is used to extract the short features of the user and the music item, whereas the dense features of the user and the item are obtained using Cross&Compression Units. Finally, the extracted segment features of the user and the music item, as well as the dense features, are sent to another MLP to obtain and output the predicted probabilities.

The inputs for the MMSS_MKR recommendation module are user *u* and item *v*. Given the user's original feature vector *u*, the latent features of *u* are extracted using an L-layer MLP.

1 $${{\text{u}}}_{{\text{L}}}={\text{M}}\left({\text{M}}\left(\cdots {\text{M}}\left({\text{u}}\right)\right)\right)={{\text{M}}}^{{\text{L}}}\left({\text{u}}\right)$$

where *M*(*x*) = σ(*Wx* + *b*) is a fully connected neural network layer with weight *W*; *b* denotes the bias; and σ() denotes the nonlinear activation function.

**Figure 6:**
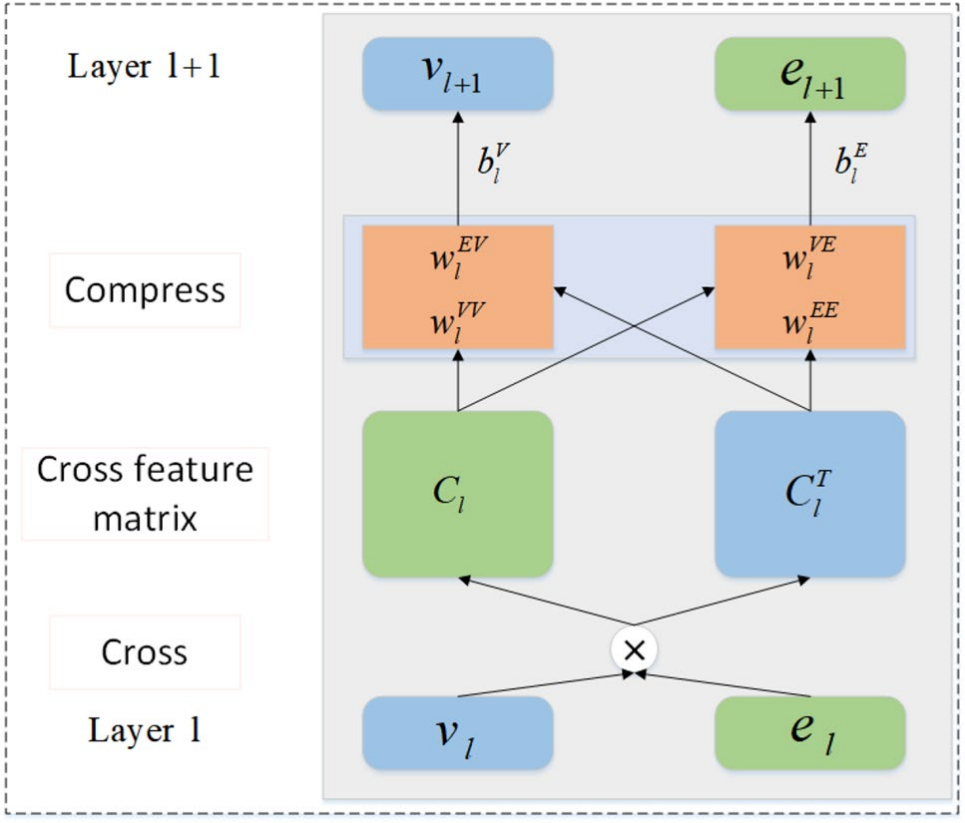
Structure of the Cross&Compression unit.

The structure of the Cross&Compression unit is shown in Fig. 6:

2 $${{\text{v}}}_{{\text{L}}}={{\text{E}}}_{{\text{e}}\sim {\text{S}}\left({\text{v}}\right)}\left[{{\text{C}}}^{{\text{L}}}\left({\text{v}},{\text{e}}\right)\left[{\text{v}}\right]\right]$$

$v_{L}$ denotes the information obtained after feature extraction using the L-layer cross-compression unit module, where S(v) denotes the set of entities associated with music item *v*.

In Fig. 5, ${{\text{u}}}_{{\text{L}}}$ corresponds to the user vectors processed through an L-layer MLP, while ${{\text{v}}}_{{\text{L}}}$ corresponds to the item vectors processed through an L-layer cross-compression unit. Because the knowledge graph used in the model is the Item-Entity KG constructed using the item and its attribute information, these entities correspond to the attribute information contained in the music item.

The potential features of the user and the music item are obtained using the MLP and the cross&compress unit, respectively. We use the inner product method in conjunction with the *sigmoid*, *tanh* and *softsign* functions to make the predictions. The obtained prediction function exhibits excellent performance, and the prediction results are clear. The dot product of *sigmoid*, *tanh* and *softsign* is processed to make the similarity more accurate; the sigmoid function is subsequently used to map the similarity index to an interval of 0–1 to better determine whether the user is interested in the music item. The final prediction probability of user participation in the music item is $f_{{R{\text{S}}}}$. In Eq. (3), $f_{{R{\text{S}}}}$ refers to the prediction probability of the recommendation module, typically using the latent features of user ${u}_{L}$ and item ${v}_{L}$ to predict the probability of whether user u will like item v through dot product and a multi-layer perceptron (MLP). In Eqs. (4)–(6), $\sigma ()$, $\alpha ()$, and $\beta ()$ denote the functions used to connect latent interactional information between user and item features. $\sigma ()$ denotes the Sigmoid function, $\alpha ()$ denotes the tanh function, and $\beta ()$ denotes the softsign function.

3 $$ \mathop y\limits^{ \wedge } = \sigma \left( {\frac{{\sigma \left( {f_{RS} \left( {u_{L} ,v_{L} } \right)} \right) + \alpha \left( {f_{RS} \left( {u_{L} ,v_{L} } \right)} \right) + \beta \left( {f_{RS} \left( {u_{L} ,v_{L} } \right)} \right)}}{3}} \right), $$

4 $$ \sigma \left( {f_{RS} \left( {u_{L} ,v_{L} } \right)} \right) = \frac{1}{{1 + e^{{ - f_{{RS\left( {u_{L} ,v_{L} } \right)}} }} }}, $$

5 $$ \alpha \left( {f_{RS} \left( {u_{L} ,v_{L} } \right)} \right) = \frac{{\exp \left( {f_{RS} \left( {u_{L} ,v_{L} } \right)} \right) - \exp \left( { - f_{RS} \left( {u_{L} ,v_{L} } \right)} \right)}}{{\exp \left( {f_{RS} \left( {u_{L} ,v_{L} } \right)} \right) + \exp \left( { - f_{RS} \left( {u_{L} ,v_{L} } \right)} \right)}}, $$

6 $$ \beta \left( {f_{RS} \left( {u_{L} ,v_{L} } \right)} \right) = \frac{{f_{RS} \left( {u_{L} ,v_{L} } \right)}}{{1 + \left| {f_{RS} \left( {u_{L} ,v_{L} } \right)} \right|}}. $$

In the music knowledge graph embedding module, MLP is used to obtain features in the head and the relations of the three tuples in the music knowledge graph.

**Figure 7:**
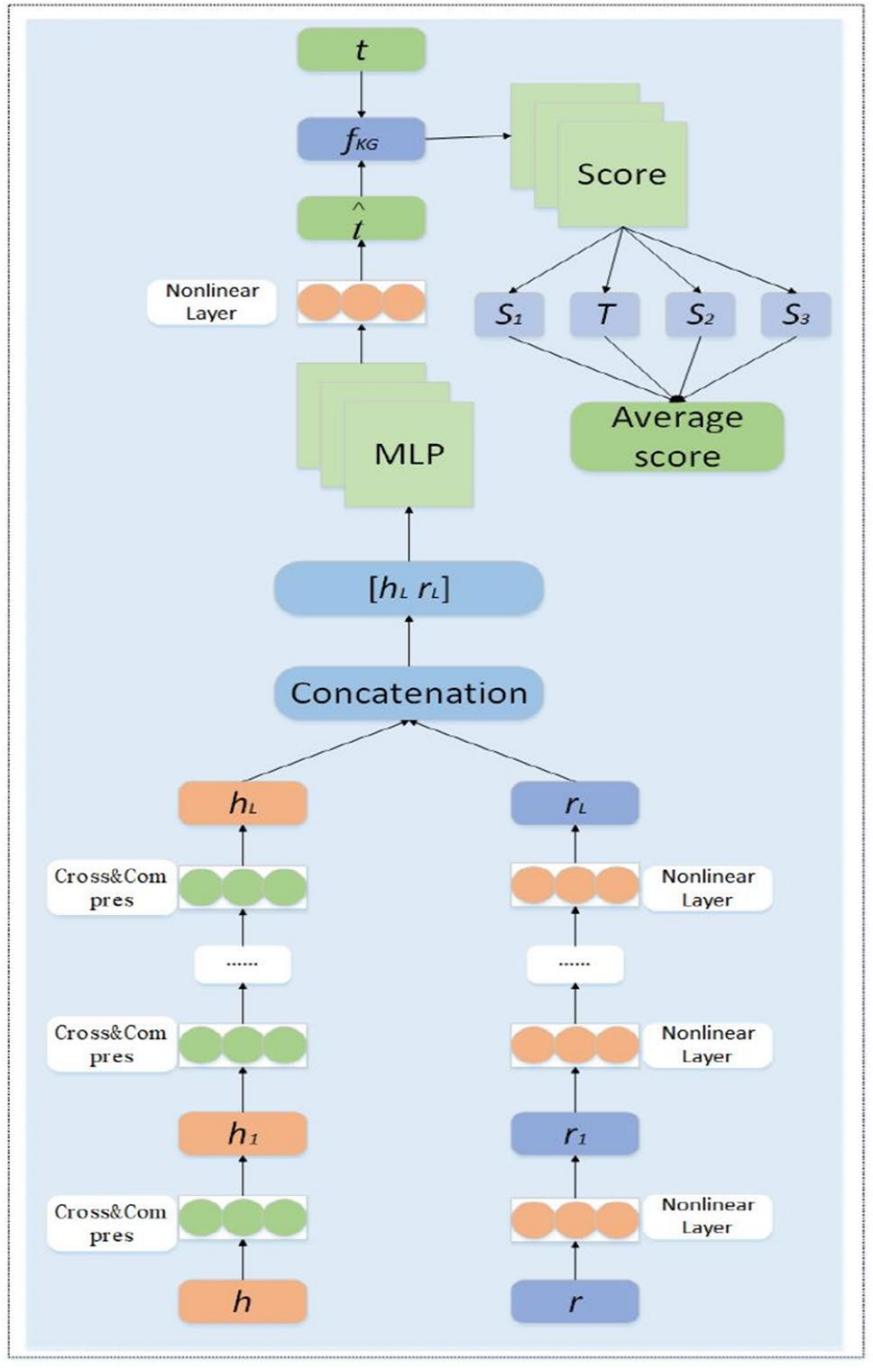
Knowledge graph embedding module analysis.

In the music knowledge graph embedding module, music entities and relations must be embedded in a continuous vector space. We adopt a semantic matching embedding framework for embedding representation of knowledge graph by connecting head entity node h and relation node t for predicting tail entity t,and then score() function to compute the score of the triplet. For a given music knowledge triplet, multiple Cross&Compression Units and a nonlinear layer are employed to process the head h and the relation r of the three tuples, link their potential features together, and connect a K-layer MLP to predict the tail t. The overall workflow of the music knowledge graph embedding module is illustrated in Fig. 7, where S1 denotes the sigmoid function, T denotes the tanh function, S2 denotes the softsign function, and S3 denotes the softplus function. The representation of the output prediction tails is also performed under the supervision of the function f and the real tails. The method for predicting the tail entity using the head entity and its relationship is as follows:

7 $${h}_{L}={E}_{v\sim S\left(h\right)}\left[{C}^{L}\left(v,h\right)\left[e\right]\right]$$

8 $${r}_{L}={M}^{L}\left(r\right)$$

9 $$ \mathop t\limits^{ \wedge } = M^{K} \left( {\left[ {\begin{array}{*{20}c} {h_{L} } \\ {r_{L} } \\ \end{array} } \right]} \right) $$

The Eq. (7) represents the handling of the head entity's feature information after l layers of MLP processing. The Eq. (8) denotes the processing of relation feature information after l layers of cross-compression units. The Eq. (9) signifies the computation involving the prediction of tail entities based on the head entity and relation. Where S(h) denotes the set of associated terms of entity *h* and *t* is the prediction vector of tail *t*. Finally, the score function is used to calculate the score of the three-tuple (*h*, *r*, *t*), whose formula is shown below. In the calculation of the music three-tuple score, we introduce the characteristics of the s*igmoid*, *tanh*, *softsign*, and *softplus* functions, through which the score is calculated not only to avoid the problem of too high or too low results caused by some special three-tuples but also to provide a good basis for accurate recommendations to users afterwards. The music three-tuple score is calculated as shown below.

10 $$ score\left( {h,r,t} \right) = \frac{{\sigma \left( {f_{KG} \left( {t^{T} ,\mathop t\limits^{ \wedge } } \right)} \right) + \alpha \left( {f_{KG} \left( {t^{T} ,\mathop t\limits^{ \wedge } } \right)} \right) + \beta \left( {f_{KG} \left( {t^{T} ,\mathop t\limits^{ \wedge } } \right)} \right) + \delta \left( {f_{KG} \left( {t^{T} ,\mathop t\limits^{ \wedge } } \right)} \right)}}{4} $$

11 $$ score\left( {h,r,t} \right) = \frac{{\sigma \left( {t^{T} \mathop t\limits^{ \wedge } } \right) + \alpha \left( {t^{T} \mathop t\limits^{ \wedge } } \right) + \beta \left( {t^{T} \mathop t\limits^{ \wedge } } \right) + \delta \left( {t^{T} \mathop t\limits^{ \wedge } } \right)}}{4} $$

12 $$ \sigma \left( {t^{T} \mathop t\limits^{ \wedge } } \right) = \frac{1}{{1 + e^{{ - t^{T} \mathop t\limits^{ \wedge } }} }} $$

13 $$ \alpha \left( {t^{T} \mathop t\limits^{ \wedge } } \right) = \frac{{\exp \left( {t^{T} \mathop t\limits^{ \wedge } } \right) - \exp \left( { - t^{T} \mathop t\limits^{ \wedge } } \right)}}{{\exp \left( {t^{T} \mathop t\limits^{ \wedge } } \right) + \exp \left( { - t^{T} \mathop t\limits^{ \wedge } } \right)}} $$

14 $$ \beta \left( {t^{T} \mathop t\limits^{ \wedge } } \right) = \frac{{t^{T} \mathop t\limits^{ \wedge } }}{{1 + \left| {t^{T} \mathop t\limits^{ \wedge } } \right|}} $$

15 $$ \delta \left( {t^{T} \mathop t\limits^{ \wedge } } \right) = \log \left( {1 + \exp \left( {t^{T} \mathop t\limits^{ \wedge } } \right)} \right) $$

The Eqs. (10)–(19) represent the process of predicting the tail entity t based on the head entity h and relation r. score() function is a scoring function used to calculate the score or evaluation of the predicted results by a model.

$f_{{{\text{KG}}}}$ denotes the probability prediction of the KGE module, typically calculated using dot product and MLP. $\sigma ()$ denotes the *sigmoid* function, $\alpha ()$ denotes the *tanh* function,$\beta ()$ denotes the *softsign* function, and $\delta ()$ denotes the *softplus* function.

The recommendation and KGE modules are separate but related modules that we bridge using a dedicated cross&compress unit. Our proposed unit not only automatically learns the interaction information of each music item present in the music recommendation system, but also obtains the interaction information of the music entities in KG. Finally, it aggregates the interaction information obtained from both parts to obtain the characteristic interaction information present in both parts, which contributes significantly to an accurate music recommendation for the user. As shown in Fig. 6, for music item v and its associated entity, we first construct their potential features from layer l and pairwise interactions:

16 $${C}_{l}={v}_{l}{e}_{l}^{T}=\left[\begin{array}{ccc}{v}_{l}^{\left(1\right)}{e}_{l}^{\left(1\right)}& \cdots & {v}_{l}^{\left(1\right)}{e}_{l}^{\left(d\right)}\\ \vdots & \ddots & \vdots \\ {v}_{l}^{\left(d\right)}{e}_{l}^{\left(1\right)}& \cdots & {v}_{l}^{\left(d\right)}{e}_{l}^{\left(d\right)}\end{array}\right]$$

The cross-compression unit comprises two processes: cross and compress. ${C}_{l}$ represents the cross-feature matrix of layer l and the dimensions of the hidden layer are represented by d. ${C}_{l}^{T}$ is a symmetric matrix of ${C}_{l}$, which is also the cross-compression matrix. This is called a crossover operation, because the interaction of features between item v and its associated entity e can be explicitly modelled in the cross-feature matrix. Finally, we output the feature vectors of the items and entities in the next layer by projecting the cross-feature matrix onto the potential representation space.

17 $${v}_{l+1}={C}_{l}{W}_{l}^{VV}+{C}_{l}^{T}{W}_{l}^{EV}+{b}_{l}^{V}={v}_{l}{e}_{l}^{T}{W}_{l}^{VV}+{e}_{l}{v}_{l}^{T}{W}_{l}^{EV}+{b}_{l}^{V}$$

18 $${e}_{l+1}={C}_{l}{W}_{l}^{VE}+{C}_{l}^{T}{W}_{l}^{EE}+{b}_{l}^{E}={v}_{l}{e}_{l}^{T}{W}_{l}^{VE}+{e}_{l}{v}_{l}^{T}{W}_{l}^{EE}+{b}_{l}^{E}$$

The compression operation projects the cross-compression matrix back to the feature space, generating corresponding item and entity feature information used for the recommendation module and the knowledge graph embedding module, where ${W}_{l}^{VV}$, ${W}_{l}^{EV}$,${W}_{l}^{VE}$ and ${W}_{l}^{EE}$ represent trainable weight, ${b}_{l}^{V}$ and ${b}_{l}^{E}$ represent bias vectors. Therefore, a cross-compression unit is shown below.

19 $$\left[{v}_{l+1},{e}_{l+1}\right]=C\left({v}_{l},{e}_{l}\right)$$

### Loss function

The complete loss function of MMSS_MKR is as follows:

$$ L = L_{RS} + L_{KG} + L_{REG} $$

20 $$ \begin{gathered} L = \sum\limits_{u \in U,v \in v} {J\left( {\mathop y\limits^{ \wedge }_{uv} ,y_{uv} } \right)} \hfill \\ \begin{array}{*{20}c} {} & {} \\ \end{array} - \lambda_{1} \left( {\sum\limits_{{\left( {h,r,t} \right) \in G}} {score\left( {h,r,t} \right) - \sum\limits_{{\left( {h{\prime} ,r,t{\prime} } \right) \notin G}} {score\left( {h^{\prime},r,t^{\prime}} \right)} } } \right) \hfill \\ \begin{array}{*{20}c} {} & {} \\ \end{array} + \lambda_{2} \left\| w \right\|_{2}^{2} \hfill \\ \end{gathered} $$

The first part denotes the metric loss in the recommendation module, where *u* and *v* traverse the sets of users and music items, respectively, and *J* is the cross-entropy function. The second part represents the loss in the KGE module, where we use the *sigmoid*, *softsign*, *tanh* and *softplus* functions to express the scores separately and then fuse the results, with the ultimate aim of maximizing the score of the true three-tuple and minimizing the score of the false three-tuple. Because we use a variety of computational methods to obtain the scores, our loss function is better able to increase the scores of all true triples, while decreasing the scores of false triples. The last part is a regular term to prevent overfitting, with λ1 and λ2 representing the equilibrium parameters. The parameters that need adjustment in Loss ${L}_{RS}$ of the recommendation module involve the weights and biases of the MLP and the cross & compression units. As for the parameters in Loss ${L}_{KG}$ of the knowledge graph embedding module, they are associated with the KGE model related parameters. Extensive experiments have shown that our loss function works well for our MMSS_MKR model in music recommendation systems. To provide a clearer representation of the workflow of the MMSS_MKR model, its learning algorithm is presented in Algorithm 1.

**Algorithm 1:**
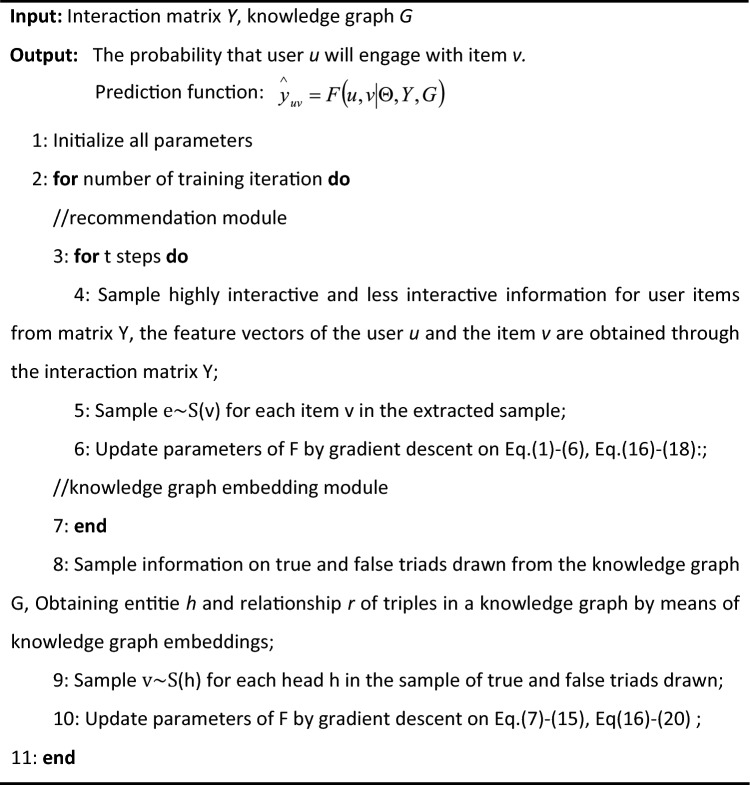
Music recommendation algorithm.

## Experiment

This section describes the results of the MMSS_MKR model compared to those of other mainstream advanced baseline recommendation models using a real music dataset and its analysis. First, the music dataset used in this experiment and the evaluation metrics are introduced. Then, the performance of the model is compared and analyzed from different perspectives. Finally, a detailed analysis of the recommendation results from the music dataset Last.FM using the MMSS_MKR model is presented.

### Data sets and assessment indices

This study uses the Last.FM dataset, which is a music information dataset containing musicians’ listening information on 2000 users of the Last.fm online music system, as shown in Table 3; these include various types of music information such as singer, genre, genre, media, author and score.

**Table 3:** DataSets.

Dataset	#users#	#items#	#interactions	#KGtriples
Last.FM	1872	3846	42,346	15,518
Book-Crossing	17,860	14,910	139,746	19,793
MovieLens-1 M	6036	2347	753,772	20,195

To better highlight the validity of the model, we validate it on the Book-Crossong dataset and the MovieLens-1M dataset, and the information contained in both datasets is shown in Table 2.

### Assessment index

In this study, two metrics, accuracy (ACC) and area under curve (AUC) of the ROC curve, were used to evaluate the MMSS_MKR model.

The ACC metric describes the proportion of classifications correctly predicted for the aggregate:

20 $$ ACC = \frac{{I_{r} }}{{I_{t} }} $$

where $I_{r}$ denotes the number of records correctly predicted, and $I_{t}$ denotes the total number of test data.

The area under the curve (AUC) quantifies the ROC curve. Because the ROC curve is not always smooth owing to the threshold value, it is difficult to determine the performance of the model; therefore, the AUC is chosen to evaluate the model, and the area formed by the ROC curve and the FPR axis is the value of the AUC.

### Comparison of model results

In the MMSS_MKR model, we determine the hyperparameters by optimizing the AUC; the hyperparameters are set as listed in Table 4.

**Table 4:** Hyperparameter settings.

Dataset	Haperparameters
Last.FM	L = 1 H = 1 dim = 128 n_epochs = 200 Batch_size = 256 Lr_rs = $2\times {10}^{-5}$ Lr_kge = $2\times {10}^{-5}$ l2_weight = $2\times {10}^{-5}$ kge_interval = 64

**Figure 8:**
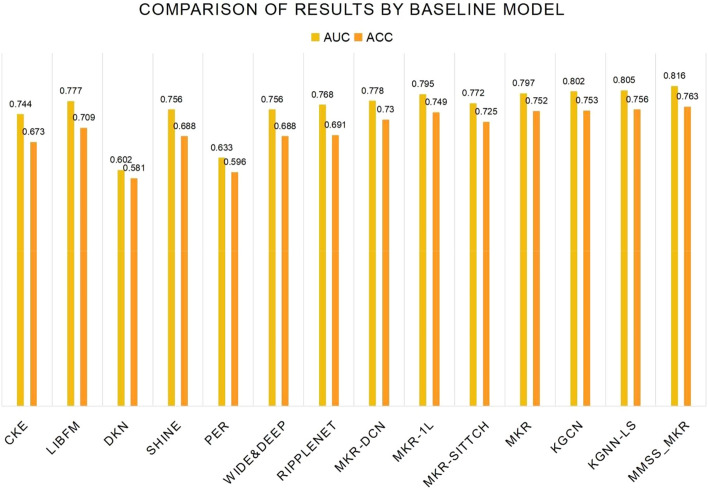
Histogram comparing results across baseline models.

We conducted a comparative analysis of our proposed method against several existing state-of-the-art recommendation algorithms, namely CKE, LibFM, DKN, SHINE, RippleNet, PER, Wide&Deep, MKR, KGCN, and KGNN-LS. The music dataset was divided into training, validation, and test sets using a 6:2:2 ratio, and multiple experiments were performed to derive the average performance. Table 5 illustrates the outcomes achieved by the MMSS_MKR model concerning evaluation metrics such as AUC and ACC^29^ when evaluated on the Last.FM dataset, alongside other methods. Figure 8 further visualizes a histogram depicting the evaluation indicators for each model. Notably, our MMSS_MKR model demonstrates superior music recommendation performance, boasting an AUC of 0.816 and an ACC of 0.763.

**Table 5:** Comparison of modeling results for music datasets.

Model	Last.FM	
	AUC	ACC
CKE	0.744	0.673
LibFM	0.777	0.709
DKN	0.602	0.581
SHINE	0.756	0.688
PER	0.633	0.596
Wide&Deep	0.756	0.688
RippleNet	0.768	0.691
MKR-DCN	0.778	0.730
MKR-1L	0.795	0.749
MKR-sittch	0.772	0.725
MKR	0.797	0.752
KGCN	0.802	0.753
KGNN-LS	0.805	0.756
MMSS_MKR	**0.816**	**0.763**

Significant values are in bold.

When compared to existing state-of-the-art recommendation algorithms such as CKE, LibFM, DKN, SHINE, RippleNet, PER, Wide&Deep, MKR, KGCN, and KGNN-LS, the MMSS_MKR model demonstrates several advantages. Firstly, it adopts comprehensive feature learning, enabling rich information extraction from the knowledge graph and facilitating the learning of comprehensive features for users and music items through multi-task learning, thereby enhancing recommendation accuracy and personalization. Secondly, the MMSS_MKR model utilizes cross-compression units to effectively capture the interaction information between the knowledge graph and the recommendation module, thus improving recommendation performance. Additionally, it possesses the ability for multi-task learning, concurrently handling knowledge graph embedding and recommendation tasks, fully leveraging knowledge graph information to enhance recommendation results. Through feature fusion, the MMSS_MKR model integrates information from different tasks and data sources to comprehensively describe users and music items, thereby improving recommendation precision and robustness. Overall evaluation metrics indicate that the MMSS_MKR model outperforms other models in music recommendation tasks, enhancing key evaluation metrics such as AUC and accuracy, demonstrating superior recommendation performance and effectiveness.

Among the current mainstream recommendation algorithms, the DKN model is the least effective for music recommendations, indicating that it is necessary to obtain more accurate preference information about users and items to obtain more accurate recommendation results. The proposed MMSS_MKR model proposed not only supplements the information null problem caused by data sparsity in the music dataset by constructing a music knowledge graph, but also obtains relevant information on users by constructing a recommendation module and a music knowledge graph embedding module and bridging the recommendation module and the KGE module by using a cross&compress unit to obtain the repeated interactions between users and music in both modules. This allows us to obtain more three-tuple information on the knowledge graph; because this information corresponds to the information obtained from the recommendation module, some three-tuple information about the music knowledge graph that interferes with accurate recommendations of users can be excluded to obtain truer three-tuple information and exclude as much false three-tuple information as possible that interferes with recommendations. The information obtained by our model is the result of the interaction between the recommendation and the music knowledge graph embedding modules. Thus, we can ensure that we mine more accurate user preference information and provide more accurate music to users. This also demonstrates the effectiveness of our model for music-recommendation systems.

MMSS_MKR not only achieves more accurate recommendations in music recommendation scenarios, it also achieves very accurate recommendations in book and movie recommendation scenarios. The accuracy of MMSS_MKR on Book-Crossing dataset and MovieLens-1 M dataset on MKR, KGCN, and KGNN-LS models are compared with MMSS_MKR model as shown in Table 6. Compared to the MKR, KGCN, and KGNN-LS models, the MMSS_MKR model achieved the best results in terms of evaluation metrics AUC and ACC, both on the MovieLens-1 M dataset and the Book-Crossing dataset.

**Table 6:** Comparison of modeling results for book and movie datasets.

Model	Book-crossing		MovieLens-1 M	
	AUC	ACC	AUC	ACC
MKR	0.719	0.679	0.917	0.830
KGCN	0.684	0.641	0.915	0.839
KGNN-LS	0.721	0.681	0.918	0.840
**MMSS_MKR**	**0.731**	**0.690**	**0.921**	**0.843**

Significant values are in bold.

### Multiple prediction experiment

To validate the effectiveness of multiple prediction methods in the MMSS_MKR model, an experiment was conducted using the Last.FM dataset. Three single prediction methods, three double prediction methods, and one triple prediction method were evaluated. The results are presented in Table 7. In the table, S1 denotes the sigmoid method, T represents the tanh method, and S2 represents the softsign method. The model utilizes the dot product of the sigmoid, tanh, and softsign functions to enhance the accuracy of the similarity computation, leading to improved recommendation performance.

**Table 7:** Comparison of experimental results of different prediction methods.

Method	S1	T	S2	S1 + T	S1 + S2	T + S2	S1 + T + S2
AUC	0.792	0.796	0.797	0.802	0.806	0.805	0.816
ACC	0.751	0.753	0.752	0.758	0.755	0.757	0.763

Table 7 clearly demonstrates that both the single and double prediction methods exhibited a significant gap in the evaluation indices AUC and ACC when compared to the triple prediction method employed in this study. Furthermore, the double prediction method showed a noticeable improvement over the single prediction method, and the triple prediction method showed further improvement over the double prediction method. Through repeated verification, it was evident that the triple prediction method was better suited for the MMSS_MKR model, resulting in a substantial enhancement in the accuracy of the recommendation performance.

### Hyperparameter experiments

Hyperparameters have a direct and important impact on the recommendation algorithm model and can directly affect the performance of the model and the accuracy of the recommendation results. In this section, we analyze the influence of two hyperparameters, L and H, on the MMSS_MKR model. In the MMSS_MKR model, L represents the lowest number of layers processed by the cross-compression units in each layer. This determines the depth of the model and the level of feature compression at each layer. Adjusting L can affect the model's ability to capture complex patterns and representations as well as its overall capacity to learn and generalize. H represents the highest number of layers in the MMSS_MKR model; it determines the maximum depth of the model and affects the level of abstraction and hierarchy in the feature representations. By adjusting H, the complexity of the model and its ability to capture high-level interactions and dependencies between features can be controlled.

The MMSS_MKR model was evaluated with the Last.FM dataset to verify the effects of the hyperparameters L and H, and the results are presented in Table 8. From the table, it can be observed that when L = 1 and H = 4, the MMSS_MKR model achieves the highest accuracy in terms of recommendation performance.

**Table 8:** Hyperparameter analysis.

Hyperparameter	Last.FM	
	AUC	ACC
L = 1,H = 1	0.801	0.751
L = 1,H = 2	0.809	0.756
L = 1,H = 3	0.812	0.759
L = 1,H = 4	0.816	0.763
L = 1,H = 5	0.813	0.761

### Ablation experiments

In the experiments performed in this study, to obtain rich feature information, we first set the hyperparameters for the entire model. The hyperparameter settings for each model are listed in Table 4.

In the ablation experiments, keeping the corresponding parameters unchanged, we performed an ablation study by removing or replacing the individual modules in the model. The original MKR model was implemented on the music dataset Last.FM, the model SM_MKR with a multiple prediction approach for predicting recommendation accuracy in the recommendation module, the model MS_MKR with a multiple calculation approach for calculating scores in the KGE module, the model SS_MKR with an improved loss function following our multiple calculation score approach, and the model MMSS_MKR incorporating the multiple.The other parameter settings remained unchanged.

As shown in Table 9, the differences between the three models, SM_MKR, MS_MKR, and SS_MKR, which are part of the experimental ablation model, are relatively small. However, these models demonstrate significant improvements compared to the baseline MKR model. This indicates that each of the proposed modules in the model offers distinct advantages in terms of music recommendation accuracy.

**Table 9:** Ablation studies on the Last.FM dataset.

Model	Last.FM	
	AUC	ACC
MKR	0.797	0.752
SM_MKR	0.809	0.759
MS_MKR	0.812	0.756
SS_MKR	0.811	0.760
MMSS_MKR	0.816	0.763

Each module plays a crucial role in enhancing the model's ability to gather comprehensive information regarding user items for recommendations, thereby improving the overall accuracy of the recommendations. Although the information obtained from the three modules differs, they complement each other by providing unique perspectives. When combined, the information gathered from all three modules offers a more comprehensive approach to music recommendation, resulting in more effective performance enhancement.

Hence, it is evident that each module involved in the ablation experiment is essential and contributes significantly to the success of the model in improving the recommendation performance.

**Figure 9:**
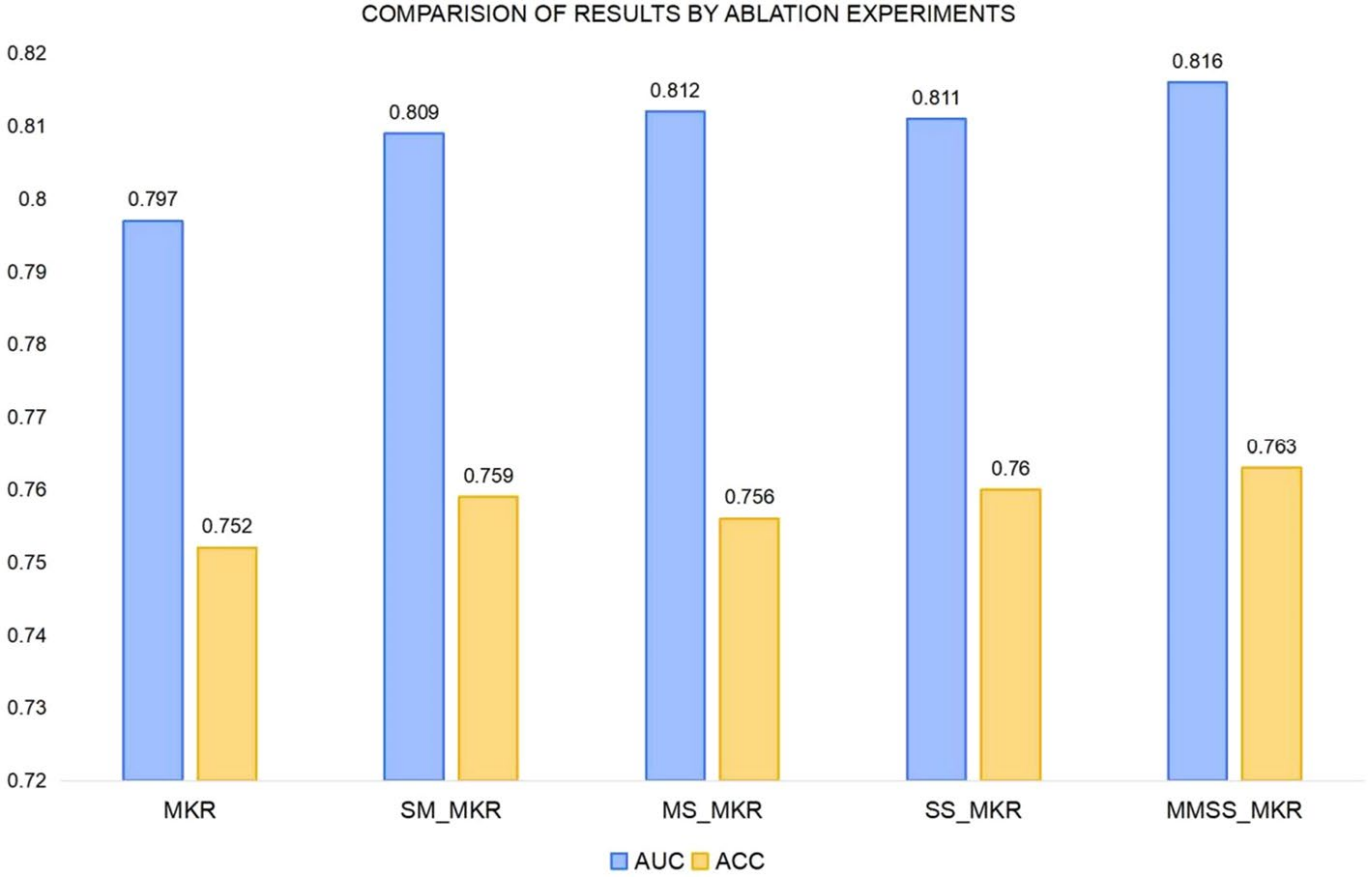
Comparison of results of ablation experiments.

The results of each of the ablation experiments in Table 9 and the histogram of the ablation experiments in Fig. 9 show that the multiple-prediction approach, multiple-computation approach, and improved loss function have a significant impact on the proposed MMSS_MKR model, which achieves a prediction accuracy of 0.816 and 0.763 in terms of AUC and ACC, respectively. Compared to the MKR model, the SM_MKR model, which incorporates only the multiple prediction approach, improves the assessment indices AUC and ACC by 1.51% and 0.93%, respectively. Compared with the MKR model, the MS_MKR model, which uses only the multiple calculation method, improves the evaluation indices AUC and ACC by 1.88% and 0.53%, respectively. Compared with the MKR model, the SS_MKR model with only our improved loss function improves the evaluation indices AUC and ACC by 1.76% and 1.06%, respectively. These results demonstrate the effectiveness of the model by dividing the music recommendation model into a music recommendation module and a music knowledge graph module using a cross&compress unit They also demonstrate the effectiveness of using multiple predictions in the recommendation module to predict the accuracy of the recommendation, multiple calculations in the KGE module to calculate the score, and our improved loss function. In turn, we demonstrate the effectiveness of our music recommendation model, MMSS_MKR, in accurately recommending music to users in the music dataset Last.FM.

## Conclusion

The proposed MMSS_MKR model obtains relevant information about users and music items through the recommendation module and acquires attribute information about music items through the knowledge graph module. The interaction and complementary nature between the recommendation and knowledge graph embedding modules contribute to a more comprehensive understanding of music items. The cross&compress unit not only facilitates information exchange between the two modules, but also optimizes the triples information within the knowledge graph, increasing the usability of genuine triples information. Multiple score calculation methods have been employed to derive scores when obtaining information about music triads, thereby enhancing the accuracy of real triples and reducing the influence of false triples. The utilization of a multichannel prediction method along with an improved loss function enables the model to provide more accurate music recommendations to users.

Our future work will focus on constructing a knowledge graph encompassing user items and addressing the deficiencies in user information within the graph. We aim to build cross-modules that offer more accurate complementary information to further enhance the accuracy of the overall model.

### Supplementary Information

Supplementary Information 1.

Supplementary Information 2.

Supplementary Information 3.

Supplementary Information 4.

## Data Availability

All data generated or analyzed in this study are included in the published article [and its supplementary information files].
