# Supplementary material for: Music recommendation algorithms based on knowledge graph and multi-task feature learning
Source: Sci Rep. 2024 Jan 24;14:2055. doi: 10.1038/s41598-024-52463-z (PMC10808181; doi:10.1038/s41598-024-52463-z)
Supplement: Supplementary file 1 — Supplementary Information 1. [file 41598_2024_52463_MOESM1_ESM.pdf]

2	0
3	1
4	2
6	3
9	4
11	5
13	6
15	7
18	8
23	9
24	10
26	11
28	12
30	13
31	14
34	15
40	16
41	17
48	18
50	19
52	20
57	21
58	22
62	23
64	24
82	25
85	26
90	27
91	28
92	29

93	30
94	31
95	32
113	33
152	34
162	35
163	36
171	37
175	38
178	39
183	40
184	41
188	42
189	43
200	44
204	45
207	46
209	47
211	48
213	49
214	50
219	51
220	52
222	53
223	54
228	55
231	56
237	57
239	58
243	59

245 60

246 61

248 62

256 63

259 64

264 65

265 66

271 67

275 68

277 69

278 70

284 71

296 72

308 73

310 74

311 75

313 76

317 77

324 78

325 79

328 80

332 81

336 82

339 83

341 84

343 85

345 86

349 87

350 88

357 89

359 90

362 91

365 92

369 93

373 94

376 95

377 96

380 97

386 98

393 99

407 100

409 101

412 102

415 103

423 104

424 105

426 106

432 107

435 108

436 109

437 110

440 111

443 112

452 113

453 114

456 115

467 116

485 117

486 118

489 119

491 120

495 121

508 122

509 123

510 124

514 125

518 126

525 127

527 128

529 129

531 130

532 131

535 132

538 133

542 134

543 135

547 136

548 137

549 138

550 139

552 140

554 141

571 142

573 143

574 144

575 145

581 146

583 147

586 148

587 149

588 150

592 151

593 152

597 153

602 154

603 155

616 156

619 157

621 158

622 159

625 160

627 161

628 162

629 163

631 164

632 165

633 166

637 167

639 168

642 169

643 170

646 171

649 172

651 173

654 174

658 175

659 176

660 177

661 178

667 179

671 180

672 181

673 182

677 183

681 184

686 185

702 186

703 187

705 188

711 189

716 190

717 191

722 192

736 193

739 194

747 195

748 196

755 197

756 198

768 199

782 200

783 201

787 202

791 203

795 204

796 205

797 206

801 207

805 208

806 209

808 210

812 211

823 212

826 213

829 214

832 215

834 216

837 217

839 218

840 219

842 220

851 221

853 222

858 223

867 224

872 225

876 226

880 227

881 228

882 229

886 230

887 231

892 232

896 233

897 234

898 235

899 236

906 237

909 238

912 239

921	240
922	241
923	242
924	243
925	244
936	245
938	246
939	247
941	248
950	249
957	250
958	251
962	252
968	253
972	254
974	255
996	256
997	257
1002	258
1003	259
1006	260
1009	261
1016	262
1021	263
1023	264
1028	265
1036	266
1047	267
1053	268
1057	269

1059	270
1064	271
1065	272
1069	273
1071	274
1073	275
1081	276
1083	277
1092	278
1095	279
1100	280
1102	281
1105	282
1108	283
1111	284
1113	285
1118	286
1124	287
1127	288
1134	289
1136	290
1142	291
1147	292
1151	293
1153	294
1155	295
1161	296
1164	297
1172	298
1176	299

1177	300
1183	301
1185	302
1188	303
1194	304
1201	305
1202	306
1203	307
1205	308
1207	309
1208	310
1211	311
1215	312
1218	313
1222	314
1229	315
1233	316
1234	317
1237	318
1252	319
1253	320
1256	321
1265	322
1266	323
1271	324
1274	325
1282	326
1287	327
1289	328
1295	329

1296	330
1297	331
1298	332
1299	333
1300	334
1301	335
1302	336
1303	337
1305	338
1318	339
1319	340
1329	341
1331	342
1332	343
1334	344
1335	345
1337	346
1338	347
1345	348
1354	349
1355	350
1367	351
1380	352
1383	353
1384	354
1385	355
1394	356
1397	357
1401	358
1403	359

1408	360
1409	361
1411	362
1428	363
1429	364
1430	365
1433	366
1434	367
1436	368
1437	369
1439	370
1440	371
1441	372
1450	373
1452	374
1454	375
1455	376
1459	377
1463	378
1466	379
1467	380
1478	381
1486	382
1494	383
1499	384
1505	385
1507	386
1509	387
1510	388
1511	389

1514	390
1515	391
1520	392
1521	393
1526	394
1533	395
1543	396
1544	397
1551	398
1556	399
1560	400
1561	401
1565	402
1569	403
1576	404
1589	405
1590	406
1591	407
1593	408
1599	409
1603	410
1606	411
1608	412
1609	413
1615	414
1616	415
1617	416
1619	417
1620	418
1622	419

1623	420
1625	421
1627	422
1628	423
1634	424
1635	425
1636	426
1650	427
1653	428
1663	429
1667	430
1674	431
1677	432
1688	433
1690	434
1691	435
1693	436
1698	437
1700	438
1701	439
1702	440
1704	441
1705	442
1706	443
1712	444
1713	445
1714	446
1715	447
1716	448
1720	449

1726	450
1727	451
1728	452
1732	453
1733	454
1734	455
1735	456
1738	457
1742	458
1743	459
1745	460
1746	461
1747	462
1748	463
1750	464
1752	465
1756	466
1761	467
1762	468
1767	469
1774	470
1779	471
1781	472
1782	473
1787	474
1789	475
1796	476
1797	477
1800	478
1801	479

1809	480
1812	481
1818	482
1824	483
1826	484
1827	485
1835	486
1836	487
1838	488
1840	489
1841	490
1845	491
1848	492
1859	493
1863	494
1875	495
1879	496
1882	497
1886	498
1892	499
1897	500
1899	501
1900	502
1902	503
1927	504
1931	505
1932	506
1941	507
1943	508
1945	509

1946	510
1947	511
1950	512
1953	513
1955	514
1959	515
1964	516
1965	517
1966	518
1969	519
1972	520
1973	521
1975	522
1977	523
1978	524
1982	525
1984	526
1985	527
1988	528
1989	529
1991	530
1994	531
1997	532
2001	533
2002	534
2009	535
2015	536
2017	537
2020	538
2022	539

2024	540
2026	541
2031	542
2032	543
2035	544
2038	545
2042	546
2046	547
2047	548
2048	549
2053	550
2059	551
2064	552
2066	553
2068	554
2069	555
2070	556
2071	557
2075	558
2078	559
2080	560
2084	561
2087	562
2095	563
2100	564
2105	565
2106	566
2124	567
2127	568
2129	569

2132	570
2134	571
2137	572
2140	573
2149	574
2150	575
2153	576
2154	577
2155	578
2162	579
2168	580
2169	581
2174	582
2177	583
2181	584
2188	585
2189	586
2197	587
2211	588
2214	589
2222	590
2223	591
2229	592
2230	593
2232	594
2235	595
2239	596
2244	597
2245	598
2246	599

2249	600
2252	601
2253	602
2254	603
2255	604
2260	605
2262	606
2264	607
2265	608
2266	609
2267	610
2270	611
2272	612
2276	613
2280	614
2281	615
2285	616
2289	617
2290	618
2293	619
2296	620
2297	621
2298	622
2299	623
2300	624
2301	625
2302	626
2303	627
2306	628
2307	629

2308	630
2310	631
2312	632
2313	633
2316	634
2317	635
2318	636
2320	637
2321	638
2322	639
2323	640
2325	641
2327	642
2328	643
2331	644
2332	645
2333	646
2349	647
2354	648
2355	649
2358	650
2361	651
2364	652
2366	653
2369	654
2370	655
2376	656
2381	657
2382	658
2383	659

2391	660
2393	661
2394	662
2399	663
2401	664
2404	665
2409	666
2410	667
2417	668
2420	669
2424	670
2427	671
2445	672
2446	673
2454	674
2465	675
2470	676
2471	677
2472	678
2474	679
2477	680
2478	681
2479	682
2485	683
2486	684
2487	685
2488	686
2492	687
2495	688
2496	689

2504	690
2511	691
2515	692
2518	693
2526	694
2532	695
2537	696
2539	697
2543	698
2545	699
2549	700
2563	701
2564	702
2572	703
2573	704
2575	705
2577	706
2578	707
2582	708
2588	709
2589	710
2592	711
2612	712
2617	713
2619	714
2627	715
2628	716
2635	717
2636	718
2640	719

2644	720
2645	721
2652	722
2657	723
2658	724
2659	725
2664	726
2668	727
2674	728
2681	729
2682	730
2683	731
2691	732
2693	733
2714	734
2716	735
2724	736
2727	737
2738	738
2740	739
2746	740
2749	741
2751	742
2752	743
2754	744
2755	745
2756	746
2757	747
2759	748
2761	749

2768	750
2769	751
2771	752
2779	753
2785	754
2789	755
2794	756
2795	757
2799	758
2803	759
2805	760
2806	761
2809	762
2811	763
2815	764
2821	765
2823	766
2824	767
2825	768
2828	769
2829	770
2835	771
2839	772
2841	773
2842	774
2848	775
2850	776
2852	777
2862	778
2865	779

2866	780
2872	781
2873	782
2876	783
2878	784
2894	785
2897	786
2900	787
2901	788
2903	789
2906	790
2909	791
2911	792
2914	793
2915	794
2921	795
2927	796
2929	797
2934	798
2938	799
2944	800
2951	801
2959	802
2966	803
2967	804
2973	805
2975	806
2976	807
2978	808
2980	809

2995	810
2996	811
2997	812
2999	813
3004	814
3009	815
3017	816
3020	817
3021	818
3022	819
3024	820
3026	821
3027	822
3031	823
3034	824
3037	825
3041	826
3043	827
3059	828
3069	829
3074	830
3076	831
3081	832
3082	833
3083	834
3087	835
3093	836
3099	837
3108	838
3114	839

3122	840
3124	841
3126	842
3131	843
3132	844
3134	845
3147	846
3150	847
3153	848
3155	849
3156	850
3157	851
3158	852
3163	853
3164	854
3165	855
3169	856
3184	857
3186	858
3187	859
3196	860
3198	861
3199	862
3200	863
3203	864
3208	865
3222	866
3230	867
3231	868
3234	869

3236	870
3239	871
3241	872
3242	873
3245	874
3248	875
3250	876
3257	877
3258	878
3264	879
3270	880
3271	881
3279	882
3280	883
3282	884
3283	885
3285	886
3289	887
3294	888
3295	889
3298	890
3300	891
3305	892
3307	893
3309	894
3310	895
3312	896
3313	897
3315	898
3316	899

3321	900
3327	901
3328	902
3334	903
3335	904
3339	905
3341	906
3344	907
3346	908
3347	909
3349	910
3351	911
3355	912
3359	913
3365	914
3367	915
3375	916
3376	917
3391	918
3397	919
3398	920
3402	921
3403	922
3404	923
3407	924
3410	925
3414	926
3415	927
3417	928
3420	929

3421	930
3429	931
3430	932
3431	933
3432	934
3433	935
3434	936
3435	937
3437	938
3440	939
3442	940
3444	941
3450	942
3452	943
3455	944
3456	945
3467	946
3472	947
3473	948
3478	949
3484	950
3489	951
3493	952
3494	953
3496	954
3499	955
3501	956
3502	957
3504	958
3505	959

3513	960
3528	961
3529	962
3530	963
3531	964
3532	965
3534	966
3541	967
3542	968
3543	969
3545	970
3551	971
3556	972
3558	973
3560	974
3562	975
3568	976
3569	977
3574	978
3583	979
3587	980
3591	981
3594	982
3595	983
3599	984
3604	985
3607	986
3609	987
3611	988
3621	989

3622	990
3624	991
3640	992
3645	993
3650	994
3657	995
3660	996
3668	997
3670	998
3671	999
3678	1000
3683	1001
3684	1002
3689	1003
3690	1004
3695	1005
3697	1006
3698	1007
3701	1008
3702	1009
3703	1010
3708	1011
3718	1012
3726	1013
3736	1014
3737	1015
3743	1016
3751	1017
3756	1018
3759	1019

3769	1020
3775	1021
3780	1022
3791	1023
3793	1024
3794	1025
3796	1026
3799	1027
3803	1028
3806	1029
3812	1030
3817	1031
3821	1032
3822	1033
3823	1034
3855	1035
3863	1036
3865	1037
3867	1038
3870	1039
3873	1040
3876	1041
3877	1042
3881	1043
3882	1044
3885	1045
3886	1046
3890	1047
3898	1048
3900	1049

3904	1050
3905	1051
3906	1052
3907	1053
3908	1054
3912	1055
3915	1056
3920	1057
3922	1058
3923	1059
3925	1060
3931	1061
3936	1062
3944	1063
3947	1064
3949	1065
3958	1066
3965	1067
3973	1068
3978	1069
3980	1070
3983	1071
3986	1072
3991	1073
3994	1074
3997	1075
4004	1076
4021	1077
4022	1078
4027	1079

4028	1080
4032	1081
4036	1082
4038	1083
4043	1084
4044	1085
4048	1086
4054	1087
4058	1088
4062	1089
4066	1090
4069	1091
4074	1092
4075	1093
4081	1094
4089	1095
4098	1096
4101	1097
4103	1098
4106	1099
4108	1100
4109	1101
4111	1102
4115	1103
4116	1104
4123	1105
4127	1106
4132	1107
4133	1108
4138	1109

4147	1110
4150	1111
4151	1112
4152	1113
4153	1114
4156	1115
4158	1116
4161	1117
4162	1118
4168	1119
4169	1120
4176	1121
4177	1122
4178	1123
4187	1124
4190	1125
4196	1126
4197	1127
4199	1128
4200	1129
4201	1130
4208	1131
4212	1132
4216	1133
4220	1134
4224	1135
4230	1136
4240	1137
4245	1138
4247	1139

4251	1140
4252	1141
4253	1142
4254	1143
4255	1144
4258	1145
4266	1146
4269	1147
4273	1148
4279	1149
4283	1150
4284	1151
4288	1152
4291	1153
4294	1154
4300	1155
4309	1156
4314	1157
4321	1158
4325	1159
4328	1160
4331	1161
4332	1162
4333	1163
4334	1164
4335	1165
4341	1166
4343	1167
4354	1168
4357	1169

4358	1170
4371	1171
4375	1172
4377	1173
4384	1174
4387	1175
4388	1176
4391	1177
4394	1178
4404	1179
4434	1180
4447	1181
4451	1182
4452	1183
4457	1184
4463	1185
4474	1186
4481	1187
4488	1188
4500	1189
4515	1190
4524	1191
4529	1192
4536	1193
4540	1194
4551	1195
4552	1196
4553	1197
4557	1198
4558	1199

4561	1200
4563	1201
4566	1202
4567	1203
4569	1204
4571	1205
4573	1206
4580	1207
4581	1208
4590	1209
4591	1210
4596	1211
4603	1212
4604	1213
4605	1214
4606	1215
4611	1216
4613	1217
4618	1218
4619	1219
4649	1220
4661	1221
4662	1222
4666	1223
4670	1224
4690	1225
4692	1226
4695	1227
4696	1228
4701	1229

4708	1230
4709	1231
4713	1232
4719	1233
4725	1234
4732	1235
4734	1236
4735	1237
4737	1238
4746	1239
4750	1240
4752	1241
4755	1242
4761	1243
4767	1244
4771	1245
4775	1246
4778	1247
4779	1248
4782	1249
4788	1250
4789	1251
4792	1252
4793	1253
4800	1254
4802	1255
4805	1256
4810	1257
4814	1258
4818	1259

4819	1260
4822	1261
4833	1262
4849	1263
4854	1264
4855	1265
4866	1266
4871	1267
4872	1268
4873	1269
4874	1270
4875	1271
4876	1272
4877	1273
4878	1274
4912	1275
4917	1276
4920	1277
4923	1278
4924	1279
4932	1280
4939	1281
4946	1282
4947	1283
4954	1284
4955	1285
4960	1286
4963	1287
4965	1288
4970	1289

4971	1290
4972	1291
4973	1292
4975	1293
4978	1294
4984	1295
4991	1296
4995	1297
4998	1298
5005	1299
5006	1300
5013	1301
5014	1302
5020	1303
5028	1304
5029	1305
5039	1306
5040	1307
5045	1308
5069	1309
5072	1310
5073	1311
5074	1312
5075	1313
5077	1314
5079	1315
5080	1316
5085	1317
5098	1318
5111	1319

5112	1320
5115	1321
5119	1322
5124	1323
5149	1324
5150	1325
5152	1326
5153	1327
5154	1328
5158	1329
5160	1330
5163	1331
5166	1332
5173	1333
5174	1334
5177	1335
5178	1336
5186	1337
5197	1338
5198	1339
5209	1340
5215	1341
5216	1342
5229	1343
5232	1344
5235	1345
5236	1346
5237	1347
5238	1348
5241	1349

5244	1350
5250	1351
5256	1352
5258	1353
5262	1354
5271	1355
5272	1356
5274	1357
5275	1358
5278	1359
5279	1360
5280	1361
5297	1362
5304	1363
5307	1364
5315	1365
5316	1366
5326	1367
5332	1368
5338	1369
5343	1370
5352	1371
5353	1372
5356	1373
5358	1374
5359	1375
5370	1376
5373	1377
5393	1378
5395	1379

5397	1380
5398	1381
5400	1382
5409	1383
5411	1384
5412	1385
5417	1386
5418	1387
5421	1388
5423	1389
5424	1390
5426	1391
5427	1392
5428	1393
5435	1394
5437	1395
5439	1396
5441	1397
5442	1398
5443	1399
5448	1400
5453	1401
5456	1402
5463	1403
5474	1404
5476	1405
5480	1406
5481	1407
5487	1408
5529	1409

5530	1410
5532	1411
5533	1412
5535	1413
5537	1414
5542	1415
5549	1416
5553	1417
5555	1418
5565	1419
5571	1420
5575	1421
5581	1422
5590	1423
5594	1424
5603	1425
5604	1426
5610	1427
5613	1428
5614	1429
5616	1430
5618	1431
5619	1432
5620	1433
5623	1434
5624	1435
5629	1436
5636	1437
5638	1438
5649	1439

5650	1440
5651	1441
5653	1442
5657	1443
5658	1444
5666	1445
5667	1446
5671	1447
5678	1448
5679	1449
5681	1450
5682	1451
5683	1452
5692	1453
5695	1454
5709	1455
5710	1456
5729	1457
5733	1458
5736	1459
5742	1460
5745	1461
5750	1462
5752	1463
5769	1464
5778	1465
5780	1466
5782	1467
5786	1468
5789	1469

5792	1470
5807	1471
5809	1472
5814	1473
5828	1474
5829	1475
5833	1476
5845	1477
5846	1478
5850	1479
5851	1480
5854	1481
5867	1482
5868	1483
5876	1484
5882	1485
5886	1486
5891	1487
5892	1488
5898	1489
5899	1490
5901	1491
5906	1492
5913	1493
5914	1494
5917	1495
5927	1496
5936	1497
5940	1498
5949	1499

5954	1500
5961	1501
5966	1502
5969	1503
5977	1504
5980	1505
5995	1506
6010	1507
6030	1508
6035	1509
6039	1510
6040	1511
6043	1512
6046	1513
6048	1514
6051	1515
6052	1516
6053	1517
6056	1518
6059	1519
6071	1520
6074	1521
6081	1522
6120	1523
6123	1524
6124	1525
6137	1526
6138	1527
6151	1528
6158	1529

6160	1530
6168	1531
6170	1532
6172	1533
6180	1534
6186	1535
6187	1536
6190	1537
6194	1538
6198	1539
6200	1540
6202	1541
6203	1542
6205	1543
6211	1544
6217	1545
6221	1546
6225	1547
6230	1548
6232	1549
6233	1550
6252	1551
6271	1552
6275	1553
6289	1554
6296	1555
6298	1556
6311	1557
6319	1558
6321	1559

6322	1560
6324	1561
6325	1562
6327	1563
6328	1564
6329	1565
6330	1566
6332	1567
6334	1568
6335	1569
6346	1570
6350	1571
6357	1572
6358	1573
6365	1574
6367	1575
6378	1576
6379	1577
6381	1578
6384	1579
6390	1580
6392	1581
6393	1582
6397	1583
6398	1584
6404	1585
6409	1586
6422	1587
6432	1588
6453	1589

6454	1590
6455	1591
6458	1592
6461	1593
6463	1594
6466	1595
6479	1596
6487	1597
6500	1598
6501	1599
6503	1600
6522	1601
6530	1602
6531	1603
6542	1604
6544	1605
6546	1606
6547	1607
6548	1608
6551	1609
6553	1610
6559	1611
6562	1612
6566	1613
6568	1614
6570	1615
6571	1616
6581	1617
6583	1618
6586	1619

6587	1620
6608	1621
6611	1622
6619	1623
6621	1624
6622	1625
6623	1626
6627	1627
6629	1628
6630	1629
6633	1630
6638	1631
6642	1632
6645	1633
6648	1634
6649	1635
6650	1636
6651	1637
6652	1638
6654	1639
6655	1640
6658	1641
6660	1642
6661	1643
6663	1644
6666	1645
6667	1646
6675	1647
6679	1648
6683	1649

6687	1650
6693	1651
6695	1652
6696	1653
6697	1654
6699	1655
6704	1656
6707	1657
6711	1658
6713	1659
6714	1660
6717	1661
6724	1662
6729	1663
6730	1664
6734	1665
6735	1666
6740	1667
6743	1668
6747	1669
6752	1670
6758	1671
6764	1672
6765	1673
6768	1674
6774	1675
6779	1676
6780	1677
6785	1678
6787	1679

6788	1680
6797	1681
6798	1682
6803	1683
6805	1684
6813	1685
6815	1686
6821	1687
6827	1688
6828	1689
6837	1690
6847	1691
6851	1692
6853	1693
6859	1694
6861	1695
6862	1696
6865	1697
6873	1698
6875	1699
6876	1700
6879	1701
6886	1702
6891	1703
6893	1704
6894	1705
6895	1706
6897	1707
6898	1708
6899	1709

6902	1710
6903	1711
6910	1712
6917	1713
6928	1714
6931	1715
6933	1716
6946	1717
6956	1718
6958	1719
6960	1720
6961	1721
6983	1722
6990	1723
6991	1724
7007	1725
7009	1726
7011	1727
7021	1728
7025	1729
7038	1730
7046	1731
7050	1732
7053	1733
7057	1734
7058	1735
7061	1736
7062	1737
7074	1738
7078	1739

7080	1740
7085	1741
7091	1742
7099	1743
7102	1744
7103	1745
7104	1746
7109	1747
7117	1748
7118	1749
7130	1750
7136	1751
7140	1752
7153	1753
7157	1754
7178	1755
7183	1756
7184	1757
7186	1758
7189	1759
7191	1760
7201	1761
7205	1762
7207	1763
7211	1764
7215	1765
7221	1766
7227	1767
7228	1768
7229	1769

7231	1770
7232	1771
7233	1772
7235	1773
7242	1774
7252	1775
7259	1776
7260	1777
7263	1778
7273	1779
7275	1780
7276	1781
7278	1782
7279	1783
7280	1784
7284	1785
7286	1786
7289	1787
7290	1788
7301	1789
7315	1790
7316	1791
7318	1792
7320	1793
7343	1794
7344	1795
7345	1796
7351	1797
7352	1798
7356	1799

7357	1800
7374	1801
7376	1802
7379	1803
7382	1804
7387	1805
7397	1806
7404	1807
7408	1808
7415	1809
7421	1810
7422	1811
7426	1812
7432	1813
7434	1814
7435	1815
7438	1816
7440	1817
7445	1818
7447	1819
7448	1820
7452	1821
7472	1822
7492	1823
7501	1824
7502	1825
7507	1826
7512	1827
7514	1828
7520	1829

7526	1830
7528	1831
7532	1832
7533	1833
7536	1834
7539	1835
7540	1836
7544	1837
7551	1838
7559	1839
7560	1840
7562	1841
7569	1842
7578	1843
7580	1844
7586	1845
7587	1846
7592	1847
7595	1848
7604	1849
7642	1850
7644	1851
7645	1852
7648	1853
7650	1854
7651	1855
7655	1856
7656	1857
7657	1858
7667	1859

7668	1860
7672	1861
7679	1862
7681	1863
7691	1864
7697	1865
7717	1866
7722	1867
7723	1868
7724	1869
7725	1870
7727	1871
7728	1872
7733	1873
7735	1874
7736	1875
7748	1876
7780	1877
7802	1878
7805	1879
7822	1880
7823	1881
7824	1882
7826	1883
7836	1884
7849	1885
7852	1886
7859	1887
7865	1888
7868	1889

7869	1890
7871	1891
7872	1892
7874	1893
7879	1894
7881	1895
7885	1896
7892	1897
7893	1898
7898	1899
7899	1900
7900	1901
7903	1902
7905	1903
7913	1904
7914	1905
7918	1906
7925	1907
7928	1908
7936	1909
7940	1910
7945	1911
7955	1912
7962	1913
7966	1914
7970	1915
8006	1916
8019	1917
8020	1918
8021	1919

8022	1920
8023	1921
8026	1922
8027	1923
8044	1924
8046	1925
8058	1926
8059	1927
8060	1928
8066	1929
8073	1930
8074	1931
8077	1932
8078	1933
8082	1934
8084	1935
8087	1936
8091	1937
8092	1938
8097	1939
8101	1940
8107	1941
8110	1942
8113	1943
8114	1944
8116	1945
8117	1946
8122	1947
8129	1948
8140	1949

8141	1950
8150	1951
8152	1952
8157	1953
8158	1954
8159	1955
8160	1956
8164	1957
8166	1958
8171	1959
8173	1960
8174	1961
8177	1962
8179	1963
8185	1964
8198	1965
8199	1966
8208	1967
8214	1968
8215	1969
8216	1970
8217	1971
8223	1972
8229	1973
8244	1974
8247	1975
8248	1976
8255	1977
8257	1978
8258	1979

8259	1980
8266	1981
8267	1982
8268	1983
8278	1984
8279	1985
8280	1986
8282	1987
8307	1988
8311	1989
8320	1990
8326	1991
8333	1992
8341	1993
8353	1994
8354	1995
8358	1996
8361	1997
8368	1998
8369	1999
8374	2000
8380	2001
8384	2002
8392	2003
8403	2004
8405	2005
8424	2006
8428	2007
8429	2008
8431	2009

8441	2010
8450	2011
8458	2012
8459	2013
8460	2014
8464	2015
8470	2016
8472	2017
8474	2018
8476	2019
8479	2020
8481	2021
8483	2022
8493	2023
8495	2024
8497	2025
8501	2026
8503	2027
8504	2028
8507	2029
8510	2030
8513	2031
8516	2032
8542	2033
8545	2034
8547	2035
8553	2036
8556	2037
8560	2038
8561	2039

8564	2040
8565	2041
8570	2042
8571	2043
8575	2044
8581	2045
8583	2046
8584	2047
8591	2048
8596	2049
8598	2050
8600	2051
8602	2052
8603	2053
8605	2054
8608	2055
8621	2056
8633	2057
8649	2058
8651	2059
8658	2060
8660	2061
8671	2062
8673	2063
8674	2064
8676	2065
8678	2066
8680	2067
8702	2068
8712	2069

8715	2070
8718	2071
8724	2072
8726	2073
8728	2074
8737	2075
8738	2076
8745	2077
8750	2078
8754	2079
8755	2080
8756	2081
8759	2082
8761	2083
8765	2084
8767	2085
8773	2086
8774	2087
8779	2088
8782	2089
8790	2090
8796	2091
8798	2092
8799	2093
8803	2094
8804	2095
8812	2096
8813	2097
8816	2098
8822	2099

8828	2100
8833	2101
8836	2102
8840	2103
8845	2104
8849	2105
8850	2106
8852	2107
8856	2108
8857	2109
8858	2110
8859	2111
8861	2112
8868	2113
8881	2114
8883	2115
8884	2116
8897	2117
8904	2118
8906	2119
8910	2120
8911	2121
8919	2122
8936	2123
8954	2124
8955	2125
8956	2126
8957	2127
8960	2128
8963	2129

8975	2130
8977	2131
8984	2132
8998	2133
9008	2134
9011	2135
9017	2136
9021	2137
9023	2138
9030	2139
9038	2140
9040	2141
9045	2142
9048	2143
9054	2144
9059	2145
9076	2146
9079	2147
9084	2148
9108	2149
9118	2150
9126	2151
9128	2152
9129	2153
9133	2154
9134	2155
9138	2156
9141	2157
9144	2158
9155	2159

9157	2160
9160	2161
9187	2162
9193	2163
9194	2164
9204	2165
9205	2166
9207	2167
9208	2168
9210	2169
9222	2170
9224	2171
9226	2172
9229	2173
9232	2174
9234	2175
9235	2176
9248	2177
9251	2178
9252	2179
9253	2180
9255	2181
9258	2182
9259	2183
9262	2184
9266	2185
9284	2186
9289	2187
9294	2188
9299	2189

9300	2190
9305	2191
9318	2192
9319	2193
9321	2194
9322	2195
9329	2196
9332	2197
9341	2198
9344	2199
9369	2200
9370	2201
9371	2202
9382	2203
9383	2204
9389	2205
9390	2206
9391	2207
9394	2208
9399	2209
9400	2210
9402	2211
9407	2212
9416	2213
9417	2214
9427	2215
9428	2216
9431	2217
9439	2218
9440	2219

9442	2220
9459	2221
9465	2222
9479	2223
9480	2224
9483	2225
9484	2226
9492	2227
9494	2228
9495	2229
9499	2230
9513	2231
9514	2232
9516	2233
9528	2234
9540	2235
9544	2236
9553	2237
9554	2238
9556	2239
9559	2240
9563	2241
9565	2242
9591	2243
9592	2244
9595	2245
9596	2246
9600	2247
9609	2248
9610	2249

9626	2250
9628	2251
9629	2252
9630	2253
9632	2254
9634	2255
9637	2256
9644	2257
9653	2258
9655	2259
9656	2260
9660	2261
9665	2262
9669	2263
9672	2264
9674	2265
9678	2266
9679	2267
9681	2268
9684	2269
9685	2270
9689	2271
9700	2272
9701	2273
9706	2274
9713	2275
9720	2276
9730	2277
9731	2278
9734	2279

9763	2280
9766	2281
9768	2282
9772	2283
9782	2284
9799	2285
9800	2286
9805	2287
9813	2288
9821	2289
9823	2290
9827	2291
9829	2292
9830	2293
9833	2294
9834	2295
9836	2296
9847	2297
9860	2298
9862	2299
9864	2300
9868	2301
9869	2302
9874	2303
9877	2304
9880	2305
9887	2306
9888	2307
9889	2308
9892	2309

9893	2310
9902	2311
9903	2312
9904	2313
9916	2314
9920	2315
9924	2316
9927	2317
9928	2318
9936	2319
9940	2320
9941	2321
9959	2322
9963	2323
9965	2324
9969	2325
9971	2326
9976	2327
9986	2328
9994	2329
9997	2330
9999	2331
10007	2332
10010	2333
10014	2334
10016	2335
10017	2336
10022	2337
10023	2338
10024	2339

10026	2340
10041	2341
10050	2342
10056	2343
10062	2344
10072	2345
10078	2346
10080	2347
10088	2348
10089	2349
10095	2350
10097	2351
10100	2352
10110	2353
10115	2354
10120	2355
10122	2356
10125	2357
10126	2358
10134	2359
10142	2360
10151	2361
10152	2362
10156	2363
10163	2364
10164	2365
10166	2366
10169	2367
10172	2368
10174	2369

10176	2370
10182	2371
10184	2372
10190	2373
10191	2374
10197	2375
10210	2376
10216	2377
10218	2378
10219	2379
10222	2380
10223	2381
10233	2382
10234	2383
10245	2384
10248	2385
10249	2386
10250	2387
10254	2388
10263	2389
10264	2390
10271	2391
10274	2392
10281	2393
10289	2394
10304	2395
10305	2396
10311	2397
10313	2398
10315	2399

10317	2400
10318	2401
10320	2402
10322	2403
10325	2404
10331	2405
10333	2406
10335	2407
10336	2408
10337	2409
10339	2410
10345	2411
10351	2412
10355	2413
10358	2414
10359	2415
10361	2416
10362	2417
10363	2418
10364	2419
10365	2420
10375	2421
10393	2422
10395	2423
10397	2424
10398	2425
10400	2426
10401	2427
10405	2428
10412	2429

10413	2430
10414	2431
10415	2432
10416	2433
10418	2434
10421	2435
10428	2436
10432	2437
10433	2438
10447	2439
10455	2440
10459	2441
10461	2442
10462	2443
10463	2444
10467	2445
10472	2446
10479	2447
10496	2448
10509	2449
10514	2450
10515	2451
10517	2452
10522	2453
10523	2454
10524	2455
10525	2456
10529	2457
10539	2458
10541	2459

10543	2460
10547	2461
10548	2462
10554	2463
10556	2464
10558	2465
10559	2466
10561	2467
10563	2468
10564	2469
10571	2470
10574	2471
10582	2472
10584	2473
10596	2474
10599	2475
10613	2476
10616	2477
10618	2478
10619	2479
10624	2480
10630	2481
10637	2482
10638	2483
10639	2484
10640	2485
10647	2486
10655	2487
10656	2488
10657	2489

10661	2490
10662	2491
10663	2492
10668	2493
10670	2494
10673	2495
10679	2496
10680	2497
10685	2498
10686	2499
10694	2500
10695	2501
10699	2502
10708	2503
10717	2504
10719	2505
10720	2506
10729	2507
10742	2508
10745	2509
10746	2510
10747	2511
10753	2512
10756	2513
10761	2514
10763	2515
10764	2516
10766	2517
10769	2518
10793	2519

10808	2520
10811	2521
10820	2522
10825	2523
10828	2524
10829	2525
10837	2526
10843	2527
10850	2528
10867	2529
10871	2530
10891	2531
10894	2532
10899	2533
10908	2534
10910	2535
10913	2536
10939	2537
10953	2538
10960	2539
10964	2540
10968	2541
10976	2542
10977	2543
10979	2544
10982	2545
10988	2546
10990	2547
10992	2548
10999	2549

11002	2550
11021	2551
11036	2552
11041	2553
11042	2554
11043	2555
11045	2556
11050	2557
11053	2558
11055	2559
11064	2560
11066	2561
11069	2562
11074	2563
11076	2564
11078	2565
11082	2566
11087	2567
11092	2568
11098	2569
11101	2570
11105	2571
11107	2572
11110	2573
11111	2574
11113	2575
11120	2576
11124	2577
11126	2578
11128	2579

11129	2580
11131	2581
11133	2582
11149	2583
11150	2584
11176	2585
11179	2586
11181	2587
11191	2588
11192	2589
11200	2590
11201	2591
11202	2592
11204	2593
11208	2594
11222	2595
11233	2596
11234	2597
11240	2598
11242	2599
11243	2600
11244	2601
11249	2602
11252	2603
11253	2604
11272	2605
11282	2606
11284	2607
11287	2608
11289	2609

11309	2610
11315	2611
11319	2612
11331	2613
11343	2614
11351	2615
11360	2616
11377	2617
11378	2618
11380	2619
11381	2620
11382	2621
11389	2622
11392	2623
11397	2624
11409	2625
11418	2626
11421	2627
11423	2628
11424	2629
11426	2630
11434	2631
11438	2632
11440	2633
11450	2634
11452	2635
11453	2636
11457	2637
11463	2638
11464	2639

11466	2640
11471	2641
11479	2642
11484	2643
11487	2644
11494	2645
11499	2646
11501	2647
11510	2648
11520	2649
11531	2650
11537	2651
11548	2652
11552	2653
11553	2654
11556	2655
11559	2656
11578	2657
11583	2658
11588	2659
11592	2660
11602	2661
11603	2662
11622	2663
11627	2664
11630	2665
11631	2666
11633	2667
11634	2668
11638	2669

11639	2670
11646	2671
11647	2672
11651	2673
11652	2674
11653	2675
11654	2676
11655	2677
11656	2678
11659	2679
11660	2680
11663	2681
11664	2682
11667	2683
11668	2684
11671	2685
11676	2686
11677	2687
11682	2688
11685	2689
11686	2690
11687	2691
11700	2692
11703	2693
11712	2694
11716	2695
11718	2696
11723	2697
11725	2698
11726	2699

11727	2700
11730	2701
11738	2702
11739	2703
11748	2704
11749	2705
11762	2706
11766	2707
11768	2708
11770	2709
11771	2710
11775	2711
11777	2712
11804	2713
11809	2714
11811	2715
11817	2716
11824	2717
11834	2718
11849	2719
11855	2720
11862	2721
11874	2722
11881	2723
11883	2724
11884	2725
11889	2726
11898	2727
11905	2728
11909	2729

11910	2730
11913	2731
11917	2732
11920	2733
11928	2734
11929	2735
11936	2736
11937	2737
11939	2738
11950	2739
11953	2740
11958	2741
11966	2742
11968	2743
11971	2744
11982	2745
11983	2746
11987	2747
11993	2748
11995	2749
12003	2750
12004	2751
12006	2752
12014	2753
12015	2754
12019	2755
12032	2756
12039	2757
12044	2758
12049	2759

12050	2760
12052	2761
12053	2762
12054	2763
12059	2764
12060	2765
12061	2766
12065	2767
12066	2768
12073	2769
12082	2770
12083	2771
12085	2772
12086	2773
12088	2774
12095	2775
12097	2776
12105	2777
12111	2778
12113	2779
12121	2780
12123	2781
12130	2782
12131	2783
12132	2784
12135	2785
12137	2786
12138	2787
12139	2788
12140	2789

12151	2790
12170	2791
12191	2792
12197	2793
12198	2794
12201	2795
12203	2796
12205	2797
12211	2798
12229	2799
12233	2800
12234	2801
12237	2802
12241	2803
12243	2804
12247	2805
12254	2806
12256	2807
12259	2808
12260	2809
12261	2810
12263	2811
12266	2812
12267	2813
12268	2814
12273	2815
12277	2816
12280	2817
12284	2818
12288	2819

12291	2820
12299	2821
12309	2822
12315	2823
12316	2824
12318	2825
12330	2826
12333	2827
12340	2828
12343	2829
12349	2830
12353	2831
12360	2832
12363	2833
12367	2834
12370	2835
12372	2836
12376	2837
12379	2838
12386	2839
12389	2840
12394	2841
12396	2842
12403	2843
12404	2844
12408	2845
12409	2846
12414	2847
12416	2848
12429	2849

12437	2850
12484	2851
12485	2852
12486	2853
12488	2854
12492	2855
12495	2856
12498	2857
12505	2858
12517	2859
12527	2860
12538	2861
12556	2862
12567	2863
12569	2864
12571	2865
12572	2866
12574	2867
12576	2868
12582	2869
12595	2870
12604	2871
12630	2872
12635	2873
12636	2874
12644	2875
12647	2876
12651	2877
12658	2878
12661	2879

12663	2880
12665	2881
12668	2882
12670	2883
12674	2884
12677	2885
12690	2886
12707	2887
12723	2888
12725	2889
12726	2890
12729	2891
12733	2892
12734	2893
12735	2894
12740	2895
12742	2896
12756	2897
12760	2898
12777	2899
12821	2900
12823	2901
12827	2902
12832	2903
12835	2904
12838	2905
12842	2906
12850	2907
12865	2908
12876	2909

12918	2910
12928	2911
12958	2912
12959	2913
12960	2914
12961	2915
12962	2916
12971	2917
12974	2918
12976	2919
12977	2920
12987	2921
12988	2922
12990	2923
12991	2924
12994	2925
12998	2926
13007	2927
13010	2928
13014	2929
13034	2930
13035	2931
13041	2932
13049	2933
13057	2934
13063	2935
13064	2936
13079	2937
13112	2938
13116	2939

13127	2940
13133	2941
13136	2942
13156	2943
13159	2944
13163	2945
13176	2946
13177	2947
13178	2948
13179	2949
13185	2950
13211	2951
13216	2952
13219	2953
13227	2954
13228	2955
13230	2956
13236	2957
13246	2958
13247	2959
13253	2960
13258	2961
13268	2962
13272	2963
13274	2964
13277	2965
13281	2966
13285	2967
13308	2968
13318	2969

13331	2970
13333	2971
13338	2972
13339	2973
13343	2974
13347	2975
13351	2976
13352	2977
13355	2978
13356	2979
13364	2980
13368	2981
13385	2982
13386	2983
13387	2984
13393	2985
13395	2986
13396	2987
13398	2988
13400	2989
13401	2990
13409	2991
13417	2992
13418	2993
13427	2994
13431	2995
13432	2996
13435	2997
13436	2998
13437	2999

13441	3000
13443	3001
13445	3002
13446	3003
13447	3004
13450	3005
13451	3006
13453	3007
13454	3008
13499	3009
13504	3010
13510	3011
13515	3012
13521	3013
13522	3014
13524	3015
13526	3016
13527	3017
13532	3018
13545	3019
13546	3020
13548	3021
13558	3022
13567	3023
13579	3024
13585	3025
13586	3026
13588	3027
13599	3028
13601	3029

13606	3030
13607	3031
13612	3032
13613	3033
13622	3034
13625	3035
13629	3036
13633	3037
13634	3038
13638	3039
13664	3040
13669	3041
13678	3042
13686	3043
13695	3044
13696	3045
13714	3046
13715	3047
13720	3048
13732	3049
13736	3050
13738	3051
13741	3052
13748	3053
13750	3054
13751	3055
13752	3056
13754	3057
13770	3058
13771	3059

13772	3060
13775	3061
13778	3062
13796	3063
13808	3064
13813	3065
13820	3066
13822	3067
13823	3068
13827	3069
13829	3070
13843	3071
13844	3072
13846	3073
13848	3074
13873	3075
13877	3076
13878	3077
13880	3078
13882	3079
13884	3080
13887	3081
13889	3082
13891	3083
13902	3084
13908	3085
13913	3086
13914	3087
13915	3088
13917	3089

13931	3090
13932	3091
13935	3092
13941	3093
13942	3094
13943	3095
13948	3096
13952	3097
13957	3098
13966	3099
13967	3100
13968	3101
13971	3102
13988	3103
13995	3104
14002	3105
14005	3106
14012	3107
14013	3108
14019	3109
14020	3110
14021	3111
14022	3112
14024	3113
14034	3114
14053	3115
14055	3116
14062	3117
14072	3118
14073	3119

14079	3120
14080	3121
14083	3122
14089	3123
14092	3124
14093	3125
14107	3126
14108	3127
14109	3128
14110	3129
14117	3130
14118	3131
14120	3132
14136	3133
14137	3134
14138	3135
14139	3136
14141	3137
14142	3138
14147	3139
14148	3140
14151	3141
14153	3142
14155	3143
14162	3144
14185	3145
14186	3146
14189	3147
14206	3148
14219	3149

14220	3150
14222	3151
14231	3152
14232	3153
14233	3154
14236	3155
14239	3156
14242	3157
14254	3158
14256	3159
14264	3160
14279	3161
14308	3162
14323	3163
14327	3164
14329	3165
14344	3166
14345	3167
14347	3168
14348	3169
14350	3170
14352	3171
14354	3172
14356	3173
14361	3174
14362	3175
14371	3176
14372	3177
14373	3178
14388	3179

14391	3180
14392	3181
14394	3182
14395	3183
14396	3184
14401	3185
14435	3186
14436	3187
14443	3188
14444	3189
14450	3190
14451	3191
14456	3192
14461	3193
14468	3194
14469	3195
14474	3196
14476	3197
14483	3198
14485	3199
14494	3200
14501	3201
14507	3202
14511	3203
14518	3204
14521	3205
14529	3206
14538	3207
14540	3208
14553	3209

14569	3210
14574	3211
14578	3212
14599	3213
14621	3214
14625	3215
14626	3216
14628	3217
14636	3218
14638	3219
14670	3220
14672	3221
14673	3222
14675	3223
14702	3224
14704	3225
14706	3226
14726	3227
14728	3228
14735	3229
14743	3230
14749	3231
14751	3232
14752	3233
14754	3234
14760	3235
14766	3236
14772	3237
14780	3238
14794	3239

14798	3240
14802	3241
14828	3242
14833	3243
14840	3244
14845	3245
14853	3246
14876	3247
14878	3248
14879	3249
14886	3250
14888	3251
14890	3252
14891	3253
14894	3254
14895	3255
14905	3256
14908	3257
14913	3258
14914	3259
14919	3260
14926	3261
14937	3262
14938	3263
14942	3264
14947	3265
14948	3266
14954	3267
14959	3268
14960	3269

14961	3270
14967	3271
14983	3272
14985	3273
15005	3274
15008	3275
15013	3276
15021	3277
15024	3278
15027	3279
15028	3280
15033	3281
15038	3282
15048	3283
15049	3284
15063	3285
15064	3286
15065	3287
15068	3288
15069	3289
15074	3290
15085	3291
15086	3292
15087	3293
15100	3294
15101	3295
15118	3296
15120	3297
15122	3298
15124	3299

15134	3300
15145	3301
15148	3302
15153	3303
15159	3304
15161	3305
15163	3306
15165	3307
15173	3308
15179	3309
15181	3310
15203	3311
15205	3312
15215	3313
15220	3314
15235	3315
15240	3316
15249	3317
15270	3318
15296	3319
15307	3320
15328	3321
15333	3322
15351	3323
15355	3324
15375	3325
15378	3326
15387	3327
15388	3328
15395	3329

15396	3330
15410	3331
15412	3332
15422	3333
15431	3334
15439	3335
15444	3336
15446	3337
15448	3338
15452	3339
15465	3340
15466	3341
15469	3342
15498	3343
15510	3344
15513	3345
15517	3346
15527	3347
15533	3348
15537	3349
15538	3350
15544	3351
15548	3352
15551	3353
15564	3354
15566	3355
15569	3356
15571	3357
15572	3358
15574	3359

15579	3360
15583	3361
15584	3362
15587	3363
15590	3364
15593	3365
15594	3366
15595	3367
15600	3368
15605	3369
15613	3370
15614	3371
15619	3372
15626	3373
15627	3374
15641	3375
15642	3376
15652	3377
15664	3378
15665	3379
15668	3380
15669	3381
15673	3382
15674	3383
15677	3384
15678	3385
15679	3386
15680	3387
15685	3388
15686	3389

15691	3390
15703	3391
15707	3392
15724	3393
15740	3394
15748	3395
15749	3396
15754	3397
15760	3398
15761	3399
15770	3400
15772	3401
15781	3402
15793	3403
15797	3404
15800	3405
15802	3406
15819	3407
15823	3408
15845	3409
15851	3410
15854	3411
15859	3412
15864	3413
15865	3414
15868	3415
15869	3416
15871	3417
15878	3418
15879	3419

15880	3420
15884	3421
15885	3422
15889	3423
15897	3424
15911	3425
15918	3426
15921	3427
15923	3428
15924	3429
15927	3430
15942	3431
15959	3432
15967	3433
15973	3434
15977	3435
15979	3436
15981	3437
15983	3438
15987	3439
15991	3440
15994	3441
15995	3442
16012	3443
16025	3444
16028	3445
16040	3446
16047	3447
16053	3448
16061	3449

16062	3450
16063	3451
16076	3452
16083	3453
16084	3454
16097	3455
16100	3456
16101	3457
16104	3458
16107	3459
16125	3460
16133	3461
16140	3462
16151	3463
16156	3464
16157	3465
16172	3466
16175	3467
16198	3468
16207	3469
16238	3470
16247	3471
16266	3472
16269	3473
16284	3474
16290	3475
16304	3476
16314	3477
16316	3478
16322	3479

16323	3480
16352	3481
16357	3482
16358	3483
16373	3484
16380	3485
16386	3486
16388	3487
16405	3488
16407	3489
16411	3490
16426	3491
16432	3492
16435	3493
16437	3494
16447	3495
16458	3496
16467	3497
16501	3498
16503	3499
16505	3500
16506	3501
16519	3502
16531	3503
16561	3504
16565	3505
16568	3506
16570	3507
16575	3508
16585	3509

16598	3510
16631	3511
16634	3512
16643	3513
16649	3514
16655	3515
16662	3516
16666	3517
16667	3518
16674	3519
16676	3520
16679	3521
16680	3522
16682	3523
16688	3524
16690	3525
16691	3526
16692	3527
16695	3528
16699	3529
16700	3530
16702	3531
16707	3532
16709	3533
16713	3534
16718	3535
16732	3536
16740	3537
16744	3538
16745	3539

16746	3540
16757	3541
16763	3542
16764	3543
16766	3544
16767	3545
16770	3546
16774	3547
16779	3548
16806	3549
16809	3550
16823	3551
16838	3552
16840	3553
16841	3554
16843	3555
16847	3556
16850	3557
16855	3558
16859	3559
16864	3560
16868	3561
16873	3562
16877	3563
16879	3564
16883	3565
16884	3566
16885	3567
16887	3568
16891	3569

16892	3570
16898	3571
16905	3572
16906	3573
16908	3574
16911	3575
16918	3576
16919	3577
16929	3578
16950	3579
16952	3580
16954	3581
16956	3582
16957	3583
16961	3584
17007	3585
17013	3586
17022	3587
17026	3588
17041	3589
17044	3590
17049	3591
17052	3592
17053	3593
17054	3594
17063	3595
17065	3596
17067	3597
17080	3598
17081	3599

17091	3600
17113	3601
17118	3602
17121	3603
17126	3604
17138	3605
17142	3606
17152	3607
17155	3608
17158	3609
17179	3610
17180	3611
17199	3612
17209	3613
17211	3614
17231	3615
17238	3616
17248	3617
17249	3618
17250	3619
17251	3620
17254	3621
17265	3622
17280	3623
17284	3624
17286	3625
17298	3626
17320	3627
17321	3628
17330	3629

17331	3630
17333	3631
17338	3632
17341	3633
17345	3634
17348	3635
17357	3636
17358	3637
17369	3638
17371	3639
17373	3640
17394	3641
17401	3642
17411	3643
17429	3644
17436	3645
17454	3646
17470	3647
17477	3648
17478	3649
17498	3650
17501	3651
17503	3652
17513	3653
17514	3654
17515	3655
17526	3656
17528	3657
17531	3658
17552	3659

17555	3660
17564	3661
17568	3662
17605	3663
17619	3664
17626	3665
17630	3666
17641	3667
17647	3668
17649	3669
17651	3670
17657	3671
17661	3672
17702	3673
17703	3674
17706	3675
17736	3676
17744	3677
17745	3678
17764	3679
17775	3680
17783	3681
17787	3682
17795	3683
17799	3684
17801	3685
17802	3686
17803	3687
17811	3688
17814	3689

17839	3690
17845	3691
17849	3692
17870	3693
17876	3694
17884	3695
17885	3696
17887	3697
17889	3698
17902	3699
17904	3700
17913	3701
17914	3702
17917	3703
17927	3704
17928	3705
17929	3706
17938	3707
17940	3708
17956	3709
17960	3710
17961	3711
17965	3712
17981	3713
17982	3714
17990	3715
17991	3716
17996	3717
18000	3718
18010	3719

18029	3720
18043	3721
18045	3722
18048	3723
18058	3724
18062	3725
18072	3726
18073	3727
18076	3728
18080	3729
18084	3730
18086	3731
18089	3732
18090	3733
18091	3734
18095	3735
18102	3736
18103	3737
18104	3738
18108	3739
18119	3740
18121	3741
18158	3742
18160	3743
18161	3744
18166	3745
18170	3746
18173	3747
18174	3748
18180	3749

18182	3750
18183	3751
18184	3752
18186	3753
18205	3754
18216	3755
18227	3756
18231	3757
18234	3758
18243	3759
18263	3760
18270	3761
18271	3762
18279	3763
18280	3764
18286	3765
18288	3766
18295	3767
18297	3768
18298	3769
18300	3770
18306	3771
18315	3772
18316	3773
18322	3774
18330	3775
18335	3776
18339	3777
18345	3778
18362	3779

18363	3780
18365	3781
18375	3782
18383	3783
18388	3784
18391	3785
18398	3786
18401	3787
18402	3788
18403	3789
18405	3790
18411	3791
18414	3792
18431	3793
18432	3794
18433	3795
18435	3796
18436	3797
18440	3798
18441	3799
18443	3800
18446	3801
18447	3802
18448	3803
18454	3804
18456	3805
18465	3806
18467	3807
18472	3808
18473	3809

18512	3810
18517	3811
18518	3812
18520	3813
18527	3814
18528	3815
18530	3816
18531	3817
18541	3818
18551	3819
18556	3820
18557	3821
18564	3822
18572	3823
18582	3824
18587	3825
18619	3826
18641	3827
18658	3828
18662	3829
18664	3830
18665	3831
18673	3832
18684	3833
18687	3834
18695	3835
18707	3836
18711	3837
18713	3838
18714	3839

18719	3840
18722	3841
18738	3842
18739	3843
18740	3844
18741	3845
