# Supplementary material for: Music recommendation algorithms based on knowledge graph and multi-task feature learning
Source: Sci Rep. 2024 Jan 24;14:2055. doi: 10.1038/s41598-024-52463-z (PMC10808181; doi:10.1038/s41598-024-52463-z)
Supplement: Supplementary file 2 — Supplementary Information 2. [file 41598_2024_52463_MOESM2_ESM.pdf]

2086 music.artist.origin 3846

1601 film.person_or_entity_appearing_in_film.film 3847

3355 film.actor.film 3848

3798 music.artist.origin 3849

427 music.artist.origin 3850

3177 music.musician.instruments_played 3851

79 film.actor.film 3852

715 film.actor.film 3853

1840 film.person_or_entity_appearing_in_film.film 3854

1733 music.artist.origin 3849

1276 people.person.place_of_birth 3855

484 film.actor.film 3856

3738 cvg.publisher.games_published 3857

3738 games.publisher.games_published 3858

613 music.artist.origin 3859

1865 music.artist.origin 3860

432 film.person_or_entity_appearing_in_film.film 3861

2241 film.distributor.film 3862

2241 film.production_company.film 3863

684 music.artist.origin 3864

643 film.person_or_entity_appearing_in_film.film 3865

1613 people.person.place_of_birth 3866

2741 film.person_or_entity_appearing_in_film.film 3867

1475 film.person_or_entity_appearing_in_film.film 3868

1059 film.actor.film 3869

1438 music.artist.origin 3870

1580 film.actor.film 3871

538 people.person.place_of_birth 3872

152 film.person_or_entity_appearing_in_film.film 3873

1443 music.artist.origin 3874

416 film.actor.film 3875

3390 film.person_or_entity_appearing_in_film.film 3876

3279 film.person_or_entity_appearing_in_film.film 3877

1479 film.actor.film 3878

68 film.person_or_entity_appearing_in_film.film 3879

68 film.person_or_entity_appearing_in_film.film 3880

383 film.actor.film 3881

2635 film.person_or_entity_appearing_in_film.film 3882

3831 music.artist.origin 3883

3384 film.person_or_entity_appearing_in_film.film 3884

1163 music.artist.origin 3885

3661 film.person_or_entity_appearing_in_film.film 3886

541 music.artist.origin 3887

829 music.artist.origin 3888

3827 film.actor.film 3889

1153 film.person_or_entity_appearing_in_film.film 3890

3444 music.artist.origin 3891

3425 music.artist.origin 3892

3317 film.actor.film 3893

2141 film.person_or_entity_appearing_in_film.film 3894

1324 people.person.place_of_birth 3895

2786 film.actor.film 3896

3539 music.artist.origin 3897

2359 film.actor.film 3898

2370 people.person.place_of_birth 3899

2125 film.actor.film 3900

3792 film.actor.film 3901

1473 film.actor.film 3902

3529 music.artist.album 3903

3784 film.writer.film 3904

749 music.artist.origin 3864

2890 film.actor.film 3905

789 film.person_or_entity_appearing_in_film.film 3906

2726 film.actor.film 3907

3109 film.person_or_entity_appearing_in_film.film 3908

3498 film.person_or_entity_appearing_in_film.film 3909

2913 film.actor.film 3910

3729 film.actor.film 3911

2622 film.actor.film 3912

628 film.actor.film 3913

472 people.person.place_of_birth 3914

70 film.actor.film 3915

447 music.artist.origin 3916

368 type.object.key 368

2636 film.person_or_entity_appearing_in_film.film 3917

147 film.person_or_entity_appearing_in_film.film 3918

1467 film.person_or_entity_appearing_in_film.film 3919

3166 film.person_or_entity_appearing_in_film.film 3920

2460 film.person_or_entity_appearing_in_film.film 3921

79 film.person_or_entity_appearing_in_film.film 3922

1840 film.actor.film 3923

2517 music.artist.origin 3897

484 film.person_or_entity_appearing_in_film.film 3924

3738 games.publisher.games_published 3925

3441 music.artist.origin 3926

119 music.artist.track 3927

3248 award.winner.awards_won 3928

2241 film.distributor.film 3929

2241 film.distributor.film 3930

795 film.actor.film 3931

1674 music.artist.origin 3932

591 film.actor.film 3933

1656 film.person_or_entity_appearing_in_film.film 3934

3031 film.actor.film 3935

2880 film.person_or_entity_appearing_in_film.film 3936

2275 film.actor.film 3937

643 film.person_or_entity_appearing_in_film.film 3938

2707 film.actor.film 3939

2493 film.person_or_entity_appearing_in_film.film 3940

538 film.person_or_entity_appearing_in_film.film 3941

2547 people.person.place_of_birth 3916

2008 music.artist.origin 3942

2843 film.actor.film 3943

444 music.artist.origin 3944

2051 people.person.place_of_birth 3945

2598 music.artist.origin 3946

176 music.artist.origin 3947

3544 film.person_or_entity_appearing_in_film.film 3948

1057 film.person_or_entity_appearing_in_film.film 3949

3822 film.person_or_entity_appearing_in_film.film 3950

997 film.person_or_entity_appearing_in_film.film 3951

1142 music.artist.origin 3952

3774 film.film.language 3953

1157 film.actor.film 3954

2621 film.person_or_entity_appearing_in_film.film 3955

3723 music.artist.origin 3956

540 film.actor.film 3957

1719 film.actor.film 3958

2725 music.musician.instruments_played 3959

3582 film.actor.film 3960

3759 music.artist.origin 3961

2953 film.actor.film 3962

3645 people.person.place_of_birth 3963

1325 award.competitor.awards_won 3964

3792 film.actor.film 3965

1529 people.person.place_of_birth 3966

136 film.actor.film 3967

2324 film.film.genre 3968

2924 film.person_or_entity_appearing_in_film.film 3969

2737 film.person_or_entity_appearing_in_film.film 3970

2564 people.person.place_of_birth 3971

3689 music.artist.origin 3972

539 film.actor.film 3973

539 film.actor.film 3974

2080 film.actor.film 3975

2214 film.actor.film 3886

508 film.actor.film 3976

3498 film.person_or_entity_appearing_in_film.film 3977

2913 film.person_or_entity_appearing_in_film.film 3978

458 music.artist.album 3979

628 film.person_or_entity_appearing_in_film.film 3980

3695 type.object.key 3695

319 film.film.genre 3981

1082 music.artist.origin 3982

2636 film.person_or_entity_appearing_in_film.film 3983

2688 people.person.place_of_birth 3984

484 film.actor.film 3985

2638 music.artist.origin 3986

901 film.actor.film 3987

2360 film.actor.film 3988

2241 film.distributor.film 3989

2241 film.production_company.film 3990

534 film.person_or_entity_appearing_in_film.film 3991

2741 music.artist.origin 3992

434 people.person.place_of_birth 3993

1664 people.person.place_of_birth 3994

2870 film.actor.film 3995

3049 film.person_or_entity_appearing_in_film.film 3996

3153 music.artist.origin 3997

3078 music.artist.origin 3897

3433 film.person_or_entity_appearing_in_film.film 3998

2051 film.person_or_entity_appearing_in_film.film 3999

1834 music.artist.origin 4000

3688 film.person_or_entity_appearing_in_film.film 4001

1057 film.actor.film 4002

1907 music.artist.origin 4003

2222 music.musician.instruments_played 3851

379 film.person_or_entity_appearing_in_film.film 4004

1633 film.actor.film 4005

623 film.actor.film 4006

1989 film.actor.film 4007

651 music.artist.origin 4008

3832 people.person.place_of_birth 4009

824 music.artist.origin 4010

492 film.person_or_entity_appearing_in_film.film 4011

1010 music.artist.album 4012

2359 people.person.place_of_birth 4013

1473 film.actor.film 4014

1412 film.person_or_entity_appearing_in_film.film 4015

1625 film.actor.film 4016

3109	film.person_or_entity_appearing_in_film.film	4017

80	music.producer.tracks_produced	4018

1744	film.actor.film	4019

447	music.artist.album	4020

1601	film.actor.film	4021

1601	film.person_or_entity_appearing_in_film.film	4022

1846	film.person_or_entity_appearing_in_film.film	3923

2636	film.person_or_entity_appearing_in_film.film	4023

3185	music.artist.origin	4024

79	film.person_or_entity_appearing_in_film.film	4025

2208	film.actor.film	4026

3738	cvg.publisher.games_published	4027

3738	cvg.publisher.games_published	4028

949	film.director.film	4029

2241	film.distributor.film	4030

2241	film.production_company.film	4031

2241	film.production_company.film	4032

2534	film.person_or_entity_appearing_in_film.film	4033

1762	film.actor.film	4034

2732	event.agent.performance	4035

1894	music.artist.album	4036

1656	film.person_or_entity_appearing_in_film.film	4037

123	music.artist.origin	4038

1806	film.actor.film	4039

2006	people.person.place_of_birth	4040

36	music.artist.track	4041

405	music.artist.origin	3864

2888	music.artist.origin	3992

1323	film.actor.film	4042

1786	film.person_or_entity_appearing_in_film.film	4043

3007 music.artist.origin 4044
3763 film.actor.film 4045
1738 music.artist.origin 4046
363 film.actor.film 4047
3199 film.actor.film 4048
2833 film.person_or_entity_appearing_in_film.film 4049
1569 people.person.place_of_birth 4050
2635 film.actor.film 4051
1157 film.writer.film 4052
2883 music.artist.origin 4053
1886 film.actor.film 4054
785 film.actor.film 4055
1179 film.film.star 4056
374 people.person.place_of_birth 4057
3367 music.artist.origin 4058
1153 people.person.place_of_birth 4059
1672 music.artist.album 4036
1891 film.person_or_entity_appearing_in_film.film 4060
149 film.actor.film 4061
3412 film.film.genre 4062
183 people.person.place_of_birth 4063
1473 film.person_or_entity_appearing_in_film.film 4064
181 film.actor.film 4065
895 film.person_or_entity_appearing_in_film.film 4066
1960 film.person_or_entity_appearing_in_film.film 4067
1983 film.actor.film 4068
3625 music.artist.origin 4013
2737 film.person_or_entity_appearing_in_film.film 4069
66 film.actor.film 4070
1238 music.artist.origin 4071

976 film.actor.film 4072

948 film.person_or_entity_appearing_in_film.film 4073

3729 film.person_or_entity_appearing_in_film.film 3911

646 film.actor.film 4074

1773 film.person_or_entity_appearing_in_film.film 3886

3120 film.person_or_entity_appearing_in_film.film 4075

319 film.film.language 4076

2121 music.artist.origin 4077

2070 film.actor.film 4078

1353 film.actor.film 4079

1308 film.actor.film 4080

2682 people.person.place_of_birth 4081

790 film.actor.film 4082

3738 cvg.publisher.games_published 4083

3738 games.publisher.games_published 4084

436 film.actor.film 4085

1154 film.person_or_entity_appearing_in_film.film 4086

1066 people.person.place_of_birth 4087

796 film.actor.film 3977

400 music.artist.origin 3972

2867 film.person_or_entity_appearing_in_film.film 4088

2241 film.distributor.film 4089

2241 film.production_company.film 4090

2241 film.production_company.film 4091

682 film.person_or_entity_appearing_in_film.film 4092

1347 people.person.place_of_birth 3947

3076 music.artist.origin 4093

2803 music.artist.origin 4094

2038 film.person_or_entity_appearing_in_film.film 4095

3486 film.actor.film 4096

643 film.actor.film 4097

1526 film.person_or_entity_appearing_in_film.film 3937

2802 music.artist.origin 4098

2906 event.agent.performance 4099

2211 film.actor.film 4100

3390 film.person_or_entity_appearing_in_film.film 4101

1323 film.person_or_entity_appearing_in_film.film 4102

3279 music.artist.origin 4103

2295 music.artist.origin 4094

3132 music.artist.album 4104

204 film.actor.film 4105

206 film.actor.film 4106

3381 film.actor.film 4107

3023 music.artist.origin 4108

2635 film.person_or_entity_appearing_in_film.film 4109

186 film.person_or_entity_appearing_in_film.film 4110

115 film.actor.film 4111

614 music.artist.album 4112

410 film.actor.film 4113

1179 film.film.writer 4114

1989 film.actor.film 4115

1748 film.actor.film 4116

2050 film.person_or_entity_appearing_in_film.film 4117

67 film.actor.film 4118

2125 film.actor.film 4119

1473 film.person_or_entity_appearing_in_film.film 4120

2467 people.person.place_of_birth 4121

3068 music.artist.album 4122

127 type.object.subject_key 127

39 people.person.place_of_birth 4123

279 film.actor.film 4124

279 film.person_or_entity_appearing_in_film.film 4125

1845 film.actor.film 4126

3282 film.actor.film 4127

3498 film.actor.film 4128

408 film.actor.film 4129

974 film.actor.film 4130

2636 film.person_or_entity_appearing_in_film.film 4131

2597 film.actor.film 4132

3821 people.person.place_of_birth 4133

781 people.person.place_of_birth 4134

2460 film.person_or_entity_appearing_in_film.film 4135

484 film.person_or_entity_appearing_in_film.film 4136

3738 cvg.publisher.games_published 4137

3738 cvg.publisher.games_published 4138

3738 games.publisher.games_published 4139

901 film.person_or_entity_appearing_in_film.film 4140

2241 film.distributor.film 4141

2241 film.distributor.film 4142

2241 film.distributor.film 4143

2241 film.distributor.film 4144

2241 film.production_company.film 4145

2779 music.artist.album 4146

1464 film.person_or_entity_appearing_in_film.film 4147

3440 music.artist.origin 4148

1624 film.actor.film 4149

1634 music.artist.origin 4150

2731 film.person_or_entity_appearing_in_film.film 4151

1475 film.actor.film 4152

2894 music.musician.instruments_played 3851

1241 film.film.writer 4153

1337 people.person.place_of_birth 4154

3742 film.person_or_entity_appearing_in_film.film 4155

3579 film.actor.film 4156

2598 people.person.place_of_birth 4157

3519 people.person.place_of_birth 4158

2658 film.actor.film 4159

1737 film.person_or_entity_appearing_in_film.film 4160

206 film.person_or_entity_appearing_in_film.film 4106

997 film.person_or_entity_appearing_in_film.film 4161

1776 film.actor.film 4162

3325 film.actor.film 4163

186 film.person_or_entity_appearing_in_film.film 4164

1720 film.writer.film 4165

1678 music.artist.origin 4166

1246 people.person.place_of_birth 4167

2724 film.actor.film 4168

1153 film.person_or_entity_appearing_in_film.film 4169

3746 music.artist.origin 4170

2039 music.artist.origin 4171

67 film.actor.film 4172

2804 people.person.place_of_birth 4094

2733 film.actor.film 4173

3087 people.person.place_of_birth 4174

181 people.person.place_of_birth 4175

895 film.person_or_entity_appearing_in_film.film 4176

1352 film.person_or_entity_appearing_in_film.film 4177

508 film.director.film 4178

1389 music.artist.album 4179

75 film.person_or_entity_appearing_in_film.film 4180

437 film.person_or_entity_appearing_in_film.film 4181

2636 film.actor.film 4182

3740 music.artist.origin 4183

294 film.person_or_entity_appearing_in_film.film 4184

602 music.producer.tracks_produced 4185

3738 cvg.publisher.games_published 4186

3738 cvg.publisher.games_published 4187

3738 games.publisher.games_published 4188

436 film.actor.film 4189

3452 film.person_or_entity_appearing_in_film.film 4190

1821 people.person.place_of_birth 4191

2241 film.distributor.film 4192

2241 film.distributor.film 4193

2241 film.production_company.film 4194

516 music.artist.origin 4195

1464 film.person_or_entity_appearing_in_film.film 4196

777 film.person_or_entity_appearing_in_film.film 4197

2514 music.artist.origin 3864

643 film.actor.film 4198

3191 music.artist.origin 4199

865 film.person_or_entity_appearing_in_film.film 4200

865 film.person_or_entity_appearing_in_film.film 4201

1806 film.person_or_entity_appearing_in_film.film 4202

1772 film.person_or_entity_appearing_in_film.film 4203

3665 people.person.place_of_birth 4204

434 film.person_or_entity_appearing_in_film.film 4205

3374 film.person_or_entity_appearing_in_film.film 4206

1496 film.actor.film 4207

3321 film.actor.film 4208

3458 film.person_or_entity_appearing_in_film.film 4209

1310 film.person_or_entity_appearing_in_film.film 4210

1183 music.artist.origin 4211

997 film.actor.film 4212

3802 music.artist.origin 4174

1228 film.actor.film 4213

2104 people.person.place_of_birth 3859

2515 music.artist.origin 4214

2494 film.actor.film 4215

2150 music.artist.origin 4216

2786 music.artist.origin 4191

149 people.deceased_person.place_of_death 4217

3788 film.actor.film 4218

67 film.person_or_entity_appearing_in_film.film 4219

2734 film.person_or_entity_appearing_in_film.film 4220

2079 people.person.profession 4221

3404 people.person.place_of_birth 4222

1910 film.film.rating 4223

2265 music.artist.origin 4224

2270 film.person_or_entity_appearing_in_film.film 4225

3598 film.actor.film 4226

30 film.person_or_entity_appearing_in_film.film 4227

1885 film.person_or_entity_appearing_in_film.film 4228

2338 film.actor.film 4229

3498 film.person_or_entity_appearing_in_film.film 4230

737 music.album.genre 4231

1974 music.artist.origin 4232

3676 film.person_or_entity_appearing_in_film.film 4233

1773 film.actor.film 4234

70 film.actor.film 4235

2636 film.person_or_entity_appearing_in_film.film 4236

147 film.person_or_entity_appearing_in_film.film 4237

2070 film.actor.film 4238

2070 film.person_or_entity_appearing_in_film.film 4239

529 film.person_or_entity_appearing_in_film.film 4240

79 film.actor.film 4241

484 film.actor.film 4242

3738 games.publisher.games_published 4243

1154 film.actor.film 4244

2241 film.distributor.film 4245

2241 film.production_company.film 4246

2241 film.production_company.film 4247

2241 film.production_company.film 4248

2241 film.production_company.film 4249

2241 film.production_company.film 4250

54 event.agent.performance 4251

777 film.actor.film 4252

3140 film.actor.film 4253

498 film.person_or_entity_appearing_in_film.film 4254

537 tv.program.genre 4255

1152 music.artist.album 4256

865 film.actor.film 4201

629 film.director.film 4257

538 film.person_or_entity_appearing_in_film.film 4258

677 music.artist.album 4259

3049 film.actor.film 4260

28 music.artist.origin 4261

1965 music.artist.origin 4262

2715 film.actor.film 4263

3026 film.person_or_entity_appearing_in_film.film 4264

1332 film.writer.film 4265

3648 film.actor.film 4266

2711 film.actor.film 4267

2919 film.actor.film 4268

3565 music.artist.origin 4269

601 film.actor.film 4270

383 film.actor.film 4271

1053 music.artist.origin 4272

2660 music.artist.origin 4013

2758 music.artist.origin 4058

1720 film.actor.film 4273

623 film.actor.film 4274

1044 type.object.subject_key 1044

2216 music.artist.track 4275

1473 film.actor.film 4276

2465 music.artist.origin 4277

977 film.actor.film 4278

1209 film.person_or_entity_appearing_in_film.film 4279

1847 music.album.genre 4280

604 film.person_or_entity_appearing_in_film.film 4281

557 film.person_or_entity_appearing_in_film.film 4282

386 film.actor.film 4283

809 film.actor.film 4284

2637 film.film.genre 4285

3280 film.actor.film 4286

2914 film.person_or_entity_appearing_in_film.film 4287

646 film.actor.film 4288

1500 film.person_or_entity_appearing_in_film.film 4289

628 film.actor.film 4290

408 film.person_or_entity_appearing_in_film.film 4129

147 film.person_or_entity_appearing_in_film.film 4291

1403 film.person_or_entity_appearing_in_film.film 4292

2070 film.actor.film 4239

2122 award.competitor.award_nominations 4293

1207 people.person.place_of_birth 3984

1831 people.person.place_of_birth 4294

1502 music.artist.origin 4217

3738 games.publisher.games_published 4295

2430 film.actor.film 4296

899 film.person_or_entity_appearing_in_film.film 4297

1115 music.artist.origin 3899

2241 film.production_company.film 4298

2241 film.production_company.film 4299

2241 film.production_company.film 4300

135 film.person_or_entity_appearing_in_film.film 4301

1762 film.person_or_entity_appearing_in_film.film 4302

2487 film.actor.film 4303

591 film.person_or_entity_appearing_in_film.film 4304

1767 film.actor.film 4305

1768 film.actor.film 4306

1051 film.person_or_entity_appearing_in_film.film 4307

1475 film.person_or_entity_appearing_in_film.film 4308

1695 film.actor.film 4309

284 music.artist.origin 3849

2926 music.artist.origin 3961

837 people.person.place_of_birth 4310

2221 music.artist.origin 3864

997 film.person_or_entity_appearing_in_film.film 4311

2402 film.actor.film 4312

2635 film.actor.film 4313

540 film.actor.film 3974

115 film.actor.film 4314

2494 film.person_or_entity_appearing_in_film.film 4315

410 film.actor.film 4316

374 film.person_or_entity_appearing_in_film.film 4317

58 film.person_or_entity_appearing_in_film.film 4318

1505 film.person_or_entity_appearing_in_film.film 4319

1153 film.actor.film 4320

2013 music.artist.origin 4217

235 music.artist.origin 4321

1910 film.film.actor 4322

2619 film.person_or_entity_appearing_in_film.film 4323

1960 film.actor.film 4324

2324 film.film.actor 4325

2998 film.actor.film 4326

2998 film.person_or_entity_appearing_in_film.film 4327

977 film.person_or_entity_appearing_in_film.film 4278

1497 film.person_or_entity_appearing_in_film.film 4328

678 music.artist.origin 3916

377 award.competitor.awards_won 4329

407 film.actor.film 4330

2217 film.actor.film 4331

1975 music.artist.origin 4332

2726 film.actor.film 4333

2338 film.person_or_entity_appearing_in_film.film 4334

386 film.person_or_entity_appearing_in_film.film 4335

809 film.actor.film 4336

80 music.artist.track 4018

2913 film.actor.film 4337

1846 film.person_or_entity_appearing_in_film.film 4168

308 music.artist.album 4338

2636 film.actor.film 4339

160 film.actor.film 4340

602 event.agent.performance 4251

1981 film.person_or_entity_appearing_in_film.film 4341

3738 cvg.publisher.games_published 4342

3738 cvg.publisher.games_published 4343

3738 games.publisher.games_published 4344

3738 games.publisher.games_published 4345

2034 film.person_or_entity_appearing_in_film.film 4346

2706 music.artist.origin 4347

2241 film.distributor.film 4348

1624 film.actor.film 3886

502 music.artist.origin 3860

2275 music.musician.instruments_played 4349

3486 film.person_or_entity_appearing_in_film.film 4350

1360 film.actor.film 4351

1553 music.artist.origin 3961

788 music.artist.origin 3866

194 film.actor.film 4352

2918 music.artist.origin 3916

253 film.person_or_entity_appearing_in_film.film 4353

3049 film.actor.film 4354

3742 film.actor.film 4355

3648 film.actor.film 4356

380 people.person.place_of_birth 4357

2711 film.person_or_entity_appearing_in_film.film 4358

1816 film.writer.film 4359

2449 film.person_or_entity_appearing_in_film.film 4360

1057 film.actor.film 4361

1724 music.artist.origin 4362

1280	film.actor.film	4363

997 film.actor.film	4364

2904	film.actor.film	4365

2402	film.person_or_entity_appearing_in_film.film	4366

735 music.artist.album	4367

2635	film.actor.film	4368

2964	music.artist.origin	4369

2507	music.artist.track 4370

1633	film.person_or_entity_appearing_in_film.film	4371

2961	music.artist.origin	4362

435 film.person_or_entity_appearing_in_film.film	4372

3788	film.person_or_entity_appearing_in_film.film	4373

117 film.person_or_entity_appearing_in_film.film	4374

2359	film.person_or_entity_appearing_in_film.film	4375

887 music.artist.origin	4376

887 music.artist.origin	4377

853 music.artist.origin	4094

1960	film.actor.film	4378

91	film.person_or_entity_appearing_in_film.film	4379

728 music.artist.origin	4380

30	film.actor.film	4227

3109	film.actor.film	4381

3498	film.actor.film	4382

3498	film.actor.film	4383

619 film.person_or_entity_appearing_in_film.film	4384

3512	film.actor.film	4385

80	music.artist.track 4386

2160	people.person.place_of_birth	4175

21	event.agent.performance	4387

128 film.person_or_entity_appearing_in_film.film	4388

2455 music.artist.origin 3864

3821 film.actor.film 4389

2688 film.person_or_entity_appearing_in_film.film 4390

1370 film.film.language 4076

1510 music.artist.origin 4391

2682 film.actor.film 3854

2682 film.person_or_entity_appearing_in_film.film 3854

3738 cvg.publisher.games_published 4392

3738 cvg.publisher.games_published 4393

3738 cvg.publisher.games_published 4394

901 film.actor.film 4395

3248 film.person_or_entity_appearing_in_film.film 4396

2867 film.person_or_entity_appearing_in_film.film 4397

2241 film.distributor.film 4398

2241 film.distributor.film 4399

2241 film.distributor.film 4400

2241 film.distributor.film 4401

2241 film.distributor.film 4402

2241 film.distributor.film 4403

2241 film.production_company.film 4404

156 music.artist.origin 4405

1618 film.actor.film 4406

1618 film.person_or_entity_appearing_in_film.film 4407

591 film.person_or_entity_appearing_in_film.film 4408

591 film.person_or_entity_appearing_in_film.film 4409

537 tv.program.genre 4410

2732 film.actor.film 4411

261 film.actor.film 4412

387 film.person_or_entity_appearing_in_film.film 4413

63 music.artist.origin 4191

469 film.person_or_entity_appearing_in_film.film 4414

3779 film.actor.film 4415

698 people.person.place_of_birth 4416

2057 music.artist.origin 4013

1913 music.album.genre 4417

696 film.actor.film 4418

3222 people.person.place_of_birth 4419

3321 film.actor.film 4420

3579 film.person_or_entity_appearing_in_film.film 4421

1786 people.person.place_of_birth 4422

2711 film.person_or_entity_appearing_in_film.film 4423

3132 film.actor.film 4424

2243 people.person.place_of_birth 4094

68 film.person_or_entity_appearing_in_film.film 4425

1985 music.musician.instruments_played 4426

2635 film.actor.film 4427

2635 film.actor.film 4428

2635 film.person_or_entity_appearing_in_film.film 4429

2789 music.musician.instruments_played 4430

379 film.actor.film 4431

818 film.person_or_entity_appearing_in_film.film 4432

3661 people.person.place_of_birth 3874

541 film.writer.film 4433

3582 film.person_or_entity_appearing_in_film.film 4434

1153 film.person_or_entity_appearing_in_film.film 4435

2141 film.actor.film 3894

905 music.artist.origin 3872

269 people.person.place_of_birth 4436

2337 film.person_or_entity_appearing_in_film.film 4437

895 film.actor.film 4438

550 music.artist.origin 4133

1706 film.actor.film 4439

192 music.producer.tracks_produced 4440

645 film.person_or_entity_appearing_in_film.film 4441

2227 music.artist.origin 4362

2462 music.artist.origin 4442

1849 music.artist.origin 4443

2234 film.person_or_entity_appearing_in_film.film 4444

3840 people.person.place_of_birth 4445

1097 music.artist.origin 4445

3363 film.person_or_entity_appearing_in_film.film 4446

2338 film.person_or_entity_appearing_in_film.film 4447

3387 film.actor.film 4448

118 music.album.genre 4449

675 film.person_or_entity_appearing_in_film.film 4450

3676 film.person_or_entity_appearing_in_film.film 4451

408 film.person_or_entity_appearing_in_film.film 4452

729 music.artist.origin 4453

2357 music.artist.origin 4454

1339 music.artist.origin 4454

424 music.artist.origin 4455

332 music.artist.origin 4456

366 film.actor.film 4457

2460 film.person_or_entity_appearing_in_film.film 4458

484 film.actor.film 4459

3193 music.artist.origin 4057

184 film.actor.film 4460

436 film.person_or_entity_appearing_in_film.film 4461

2293 film.actor.film 4462

1154 film.actor.film 4463

1249 film.actor.film 4464

1249 film.person_or_entity_appearing_in_film.film 4465

2159 film.person_or_entity_appearing_in_film.film 4466

2241 film.distributor.film 4467

2730 music.artist.origin 4046

2232 music.artist.origin 3846

135 film.actor.film 4468

451 award.nominee.award_nominations 4469

777 film.actor.film 4470

2606 music.artist.origin 4471

3547 tv.program.genre 4472

1059 film.actor.film 4473

1059 film.person_or_entity_appearing_in_film.film 4474

3331 film.person_or_entity_appearing_in_film.film 4475

1536 film.actor.film 4476

3742 film.person_or_entity_appearing_in_film.film 4477

2711 film.person_or_entity_appearing_in_film.film 4478

2409 people.person.place_of_birth 4479

2010 film.actor.film 4480

1358 film.person_or_entity_appearing_in_film.film 4481

422 people.deceased_person.place_of_death 4057

567 film.actor.film 4482

623 film.person_or_entity_appearing_in_film.film 4483

1153 film.person_or_entity_appearing_in_film.film 4484

1153 film.person_or_entity_appearing_in_film.film 4485

1891 music.producer.tracks_produced 4486

2050 film.person_or_entity_appearing_in_film.film 4487

1032 film.person_or_entity_appearing_in_film.film 4488

2147 film.actor.film 4489

2147 film.person_or_entity_appearing_in_film.film 4490

2125 film.actor.film 4491

2337 film.actor.film 4492

1711 film.person_or_entity_appearing_in_film.film 4493

71 film.person_or_entity_appearing_in_film.film 4494

604 music.artist.album 4495

2083 people.person.place_of_birth 4496

987 film.actor.film 4497

30 film.actor.film 4498

1035 people.person.place_of_birth 4499

893 film.actor.film 4500

3282 film.person_or_entity_appearing_in_film.film 4501

1638 music.artist.origin 4502

2497 film.person_or_entity_appearing_in_film.film 4503

675 film.actor.film 4504

2562 film.actor.film 4505

1355 film.person_or_entity_appearing_in_film.film 4506

2460 film.person_or_entity_appearing_in_film.film 4507

1307 film.person_or_entity_appearing_in_film.film 4508

3738 games.publisher.games_published 4509

1642 music.artist.origin 4454

1872 film.person_or_entity_appearing_in_film.film 4510

2241 film.distributor.film 4511

2241 film.distributor.film 4512

2241 film.production_company.film 4513

2241 film.production_company.film 4514

1873 film.actor.film 4515

894 film.actor.film 4516

3198 people.person.place_of_birth 4517

643 film.actor.film 4518

1806 film.person_or_entity_appearing_in_film.film 4519

3066 people.person.place_of_birth 4013

2100 people.deceased_person.place_of_death 4057

1357 film.person_or_entity_appearing_in_film.film 4520

3817 music.artist.origin 4521

2860 film.actor.film 4522

594 music.artist.album 4523

298 film.person_or_entity_appearing_in_film.film 3991

1558 film.actor.film 4524

1323 film.person_or_entity_appearing_in_film.film 4525

1130 music.artist.origin 4526

390 music.artist.origin 4527

2010 film.person_or_entity_appearing_in_film.film 4528

3132 film.person_or_entity_appearing_in_film.film 4073

1283 music.artist.origin 3916

1358 film.person_or_entity_appearing_in_film.film 4529

142 film.actor.film 4530

1279 film.actor.film 4531

102 music.album.track 4532

567 film.person_or_entity_appearing_in_film.film 4533

1110 music.artist.origin 4534

1157 film.person_or_entity_appearing_in_film.film 4535

3245 music.artist.origin 4150

3405 music.artist.origin 4536

2494 people.person.place_of_birth 4537

785 film.actor.film 4538

3454 music.artist.origin 3846

141 film.actor.film 4539

3035 film.person_or_entity_appearing_in_film.film 4540

395 film.actor.film 4541

149 film.person_or_entity_appearing_in_film.film 4061

633 film.person_or_entity_appearing_in_film.film 4542
2201 film.person_or_entity_appearing_in_film.film 4543
1473 film.actor.film 4544
895 film.person_or_entity_appearing_in_film.film 4545
2727 music.artist.origin 4057
3040 film.person_or_entity_appearing_in_film.film 4546
150 film.actor.film 4547
2398 event.agent.performance 4548
2737 film.person_or_entity_appearing_in_film.film 4549
2080 film.actor.film 4550
2726 film.person_or_entity_appearing_in_film.film 4551
1321 music.artist.origin 4454
3535 music.artist.origin 4552
1744 film.person_or_entity_appearing_in_film.film 4019
458 film.person_or_entity_appearing_in_film.film 4553
2113 people.person.place_of_birth 4232
1846 film.actor.film 4168
128 award.winner.awards_won 4554
2636 film.person_or_entity_appearing_in_film.film 4555
3355 film.person_or_entity_appearing_in_film.film 4556
3355 film.person_or_entity_appearing_in_film.film 4557
1206 film.actor.film 4558
2460 film.person_or_entity_appearing_in_film.film 4559
79 film.actor.film 4560
3349 film.person_or_entity_appearing_in_film.film 4561
3738 cvg.publisher.games_published 4562
1154 film.person_or_entity_appearing_in_film.film 4244
1827 music.artist.origin 3966
3562 music.artist.origin 3864
1993 music.artist.origin 4563

2867 film.actor.film 4564

1463 award.competitor.award_nominations 4565

2241 film.distributor.film 4566

2241 film.distributor.film 4567

2241 film.distributor.film 4568

2241 film.distributor.film 4569

2241 film.distributor.film 4570

2241 film.production_company.film 4571

2241 film.production_company.film 4572

2241 film.production_company.film 4573

1464 film.actor.film 4196

1433 music.artist.origin 4024

1624 film.person_or_entity_appearing_in_film.film 4574

777 film.actor.film 4575

2260 film.actor.film 4576

2260 music.artist.album 4577

3198 film.actor.film 4578

3143 tv.program.genre 4579

3486 film.person_or_entity_appearing_in_film.film 4580

1806 film.actor.film 4581

2741 film.actor.film 3867

2741 film.person_or_entity_appearing_in_film.film 4582

1146 music.artist.origin 3916

2745 music.artist.origin 4583

3331 film.person_or_entity_appearing_in_film.film 4584

629 film.director.film 4585

3321 film.actor.film 4586

3321 film.actor.film 3978

3754 music.artist.origin 4587

3390 film.actor.film 4588

3390 music.artist.album 4589

1285 people.deceased_person.place_of_death 3945

2296 film.actor.film 4590

3677 music.artist.origin 4058

2297 film.actor.film 4591

1157 film.person_or_entity_appearing_in_film.film 4592

1413 film.actor.film 4593

785 film.person_or_entity_appearing_in_film.film 4594

313 film.actor.film 4595

902 music.artist.origin 4008

3584 music.artist.origin 4596

3425 people.person.place_of_birth 4175

2953 film.actor.film 4597

3523 music.artist.origin 4057

633 film.person_or_entity_appearing_in_film.film 4598

2359 film.actor.film 4599

1960 film.actor.film 4600

3784 film.director.film 4601

3074 film.actor.film 4602

3040 event.agent.performance 4603

377 film.actor.film 4604

990 music.artist.album 4605

2890 music.artist.origin 4606

1661 film.person_or_entity_appearing_in_film.film 4607

458 film.actor.film 4608

70 film.person_or_entity_appearing_in_film.film 4609

2095 film.person_or_entity_appearing_in_film.film 4610

2095 film.person_or_entity_appearing_in_film.film 4611

2481 film.person_or_entity_appearing_in_film.film 4612

445 film.actor.film 4613

79 film.actor.film 4614

484 film.actor.film 4615

3794 music.artist.origin 4616

2208 music.artist.album 4617

3738 cvg.publisher.games_published 4618

2874 film.person_or_entity_appearing_in_film.film 4619

934 music.artist.origin 4057

3042 music.artist.origin 4003

3787 film.person_or_entity_appearing_in_film.film 4620

668 film.person_or_entity_appearing_in_film.film 4621

2631 film.actor.film 4622

2241 film.distributor.film 4623

2241 film.distributor.film 4624

2241 film.distributor.film 4625

2241 film.production_company.film 4626

2241 film.production_company.film 4627

2241 film.production_company.film 4628

988 film.person_or_entity_appearing_in_film.film 4629

1768 film.actor.film 4630

636 film.actor.film 4631

1772 music.artist.origin 3846

1729 film.person_or_entity_appearing_in_film.film 4632

434 film.person_or_entity_appearing_in_film.film 4633

3002 film.actor.film 4634

2656 people.person.place_of_birth 4635

3000 film.actor.film 4636

3231 film.person_or_entity_appearing_in_film.film 4637

898 film.actor.film 4638

3717 people.person.place_of_birth 4639

2573 music.artist.origin 4391

3026 film.actor.film 4640

2630 film.person_or_entity_appearing_in_film.film 4641

2711 film.actor.film 4642

1816 film.actor.film 4643

3132 film.person_or_entity_appearing_in_film.film 4644

2449 film.person_or_entity_appearing_in_film.film 4645

56 film.actor.film 4646

206 film.actor.film 4647

206 film.person_or_entity_appearing_in_film.film 4648

1358 film.person_or_entity_appearing_in_film.film 4649

383 film.actor.film 4650

2635 film.actor.film 4651

2142 music.artist.origin 3942

2494 film.actor.film 4652

1232 film.person_or_entity_appearing_in_film.film 4653

785 film.actor.film 4654

3827 film.person_or_entity_appearing_in_film.film 4655

58 film.actor.film 4656

492 film.person_or_entity_appearing_in_film.film 4657

2901 music.artist.origin 4010

662 people.person.place_of_birth 4658

1153 film.person_or_entity_appearing_in_film.film 4659

3788 film.person_or_entity_appearing_in_film.film 4660

299 music.artist.origin 4057

1473 film.actor.film 4661

722 music.artist.origin 3859

2337 film.person_or_entity_appearing_in_film.film 4662

1766 film.person_or_entity_appearing_in_film.film 4663

127 film.actor.film 4664

1792 music.artist.origin 4665

3598 film.actor.film 4666

3598 film.person_or_entity_appearing_in_film.film 4667

3086 film.actor.film 4668

1335 music.artist.origin 4362

508 film.actor.film 4669

2259 film.person_or_entity_appearing_in_film.film 4670

2913 film.person_or_entity_appearing_in_film.film 4671

2914 film.actor.film 4672

2093 music.artist.origin 4673

1601 film.actor.film 4674

2636 film.person_or_entity_appearing_in_film.film 4339

3071 music.artist.origin 4057

79 film.actor.film 3922

373 film.person_or_entity_appearing_in_film.film 3880

270 film.actor.film 4675

2167 music.artist.origin 4676

602 music.artist.track 4677

527 people.person.place_of_birth 3859

3738 games.publisher.games_published 4678

3738 games.publisher.games_published 4679

3738 games.publisher.games_published 4680

436 film.person_or_entity_appearing_in_film.film 4681

901 film.actor.film 4682

625 film.person_or_entity_appearing_in_film.film 4683

2867 film.person_or_entity_appearing_in_film.film 4684

2241 film.distributor.film 4685

2241 film.production_company.film 4686

2241 film.production_company.film 4687

2241 film.production_company.film 4688

2241 film.production_company.film 4689

2241	film.production_company.film	4684

2241	film.production_company.film	4690

2241	film.production_company.film	4691

745 music.artist.origin	4003

1676	film.person_or_entity_appearing_in_film.film	4692

215 music.artist.origin	4693

1768	film.person_or_entity_appearing_in_film.film	4694

1567	film.person_or_entity_appearing_in_film.film	4695

333 music.artist.origin	4376

474 music.artist.origin	3864

817 film.person_or_entity_appearing_in_film.film	4696

2493	film.person_or_entity_appearing_in_film.film	4697

757 film.person_or_entity_appearing_in_film.film	4360

3321	film.actor.film	4698

2711	film.person_or_entity_appearing_in_film.film	4699

3628	cvg.computer_videogame.publisher	4700

1576	music.artist.origin	4701

3006	music.artist.origin	4183

997 film.actor.film	4311

83	people.person.place_of_birth	4702

1662	film.actor.film	4703

411 music.artist.origin	4057

1293	music.artist.origin	3897

3384	film.person_or_entity_appearing_in_film.film	4704

1681	music.artist.origin	3849

1247	film.actor.film	4705

115 film.person_or_entity_appearing_in_film.film	4706

2494	film.actor.film	3868

718 music.artist.origin	4707

1044	award.competitor.award_nominations	4708

3827 film.actor.film 4709

492 film.person_or_entity_appearing_in_film.film 4710

492 people.deceased_person.place_of_death 4711

2371 film.person_or_entity_appearing_in_film.film 4712

847 film.person_or_entity_appearing_in_film.film 4713

1190 music.artist.origin 4714

136 people.person.place_of_birth 4077

1960 film.actor.film 4715

3784 film.producer.film 4716

127 film.actor.film 4717

604 music.artist.album 4718

987 film.actor.film 4719

1441 film.actor.film 4720

2080 film.actor.film 4721

3598 film.actor.film 4722

3396 people.deceased_person.place_of_death 4347

376 music.artist.origin 4723

3344 film.actor.film 4724

2713 film.person_or_entity_appearing_in_film.film 4725

322 music.artist.origin 4391

3498 film.actor.film 4726

2913 film.person_or_entity_appearing_in_film.film 4337

2914 film.actor.film 4287

741 music.artist.album 4256

1338 music.artist.origin 4174

3678 cvg.computer_videogame.publisher 4727

1836 music.artist.origin 4224

2070 film.person_or_entity_appearing_in_film.film 3957

3179 film.actor.film 4728

2689 event.agent.performance 4729

2460 film.actor.film 4730

3443 film.actor.film 4731

1363 music.artist.origin 4732

2241 film.distributor.film 4733

2241 film.distributor.film 4734

2241 film.distributor.film 4735

2241 film.distributor.film 4736

2241 film.distributor.film 4737

681 film.person_or_entity_appearing_in_film.film 4738

2453 music.artist.origin 3899

800 music.artist.origin 4739

2275 film.person_or_entity_appearing_in_film.film 4646

2200 film.actor.film 4740

2678 film.actor.film 4741

3156 music.artist.origin 3916

2206 film.writer.film 4742

2731 film.person_or_entity_appearing_in_film.film 4743

2100 film.person_or_entity_appearing_in_film.film 4744

2861 film.actor.film 4745

440 music.artist.origin 4746

3509 film.actor.film 4747

3321 film.person_or_entity_appearing_in_film.film 4748

81 film.person_or_entity_appearing_in_film.film 4749

2595 film.person_or_entity_appearing_in_film.film 4750

115 film.actor.film 4751

1439 film.actor.film 4752

785 film.actor.film 4753

1720 film.director.film4754

1720 film.person_or_entity_appearing_in_film.film 4755

3827 film.actor.film 4756

815 film.person_or_entity_appearing_in_film.film 4757

3014 music.artist.origin 3859

282 film.person_or_entity_appearing_in_film.film 4758

633 film.actor.film 4759

774 film.person_or_entity_appearing_in_film.film 4760

3115 music.artist.origin 4272

1473 film.person_or_entity_appearing_in_film.film 4761

2823 music.artist.origin 4174

407 film.actor.film 4762

2080 film.actor.film 4763

3086 film.person_or_entity_appearing_in_film.film 4668

30 people.person.place_of_birth 4764

1616 music.musician.instruments_played 3851

948 film.actor.film 4765

3498 film.actor.film 4766

672 film.actor.film 4767

1435 people.person.place_of_birth 4768

1793 music.artist.origin 3897

2914 film.person_or_entity_appearing_in_film.film 4769

1702 film.person_or_entity_appearing_in_film.film 4067

1007 event.agent.performance 4770

646 film.person_or_entity_appearing_in_film.film 4771

3695 type.object.subject_key 3695

437 film.actor.film 4772

2636 film.person_or_entity_appearing_in_film.film 4773

685 film.actor.film 4774

2674 people.deceased_person.place_of_death 4775

2597 film.person_or_entity_appearing_in_film.film 4776

855 people.person.place_of_birth 4094

3179 film.person_or_entity_appearing_in_film.film 4682

3166 film.person_or_entity_appearing_in_film.film 4777

1353 film.person_or_entity_appearing_in_film.film 4778

1173 film.person_or_entity_appearing_in_film.film 4779

3588 film.actor.film 4780

3738 games.publisher.games_published 4781

436 film.director.film 4782

901 award.competitor.awards_won 4783

3206 music.artist.origin 4784

796 film.actor.film 4230

796 people.person.place_of_birth 4785

2867 film.person_or_entity_appearing_in_film.film 4564

2241 film.distributor.film 4786

2241 film.distributor.film 4787

2241 film.production_company.film 4788

2241 film.production_company.film 4789

3297 film.person_or_entity_appearing_in_film.film 4281

1624 film.person_or_entity_appearing_in_film.film 4790

2099 film.actor.film 4791

382 film.person_or_entity_appearing_in_film.film 4792

1877 film.person_or_entity_appearing_in_film.film 4793

1656 film.actor.film 3934

2951 film.person_or_entity_appearing_in_film.film 4794

2643 film.actor.film 4795

2047 music.artist.origin 3944

3312 music.artist.origin 3874

1860 music.artist.origin 4796

1278 award.competitor.award_nominations 4797

2184 film.person_or_entity_appearing_in_film.film 4798

2970 film.person_or_entity_appearing_in_film.film 4799

1526 film.actor.film 4710

301 music.artist.origin 4800

3433 film.person_or_entity_appearing_in_film.film 4801

1954 film.actor.film 4802

2711 film.person_or_entity_appearing_in_film.film 4803

1803 music.artist.origin 4038

193 film.person_or_entity_appearing_in_film.film 4804

1362 music.artist.origin 4453

2130 people.person.place_of_birth 4077

1781 film.person_or_entity_appearing_in_film.film 4805

68 film.actor.film 4806

2840 music.artist.origin 4195

457 music.artist.origin 4807

2942 people.person.place_of_birth 4808

1157 film.person_or_entity_appearing_in_film.film 4809

3194 film.person_or_entity_appearing_in_film.film 4810

1044 film.actor.film 4811

2084 film.person_or_entity_appearing_in_film.film 4812

3035 people.deceased_person.place_of_death 4813

1891 film.person_or_entity_appearing_in_film.film 4814

2094 music.artist.origin 4057

2757 music.artist.origin 4815

2620 people.person.place_of_birth 4816

67 film.actor.film 4817

2216 music.producer.tracks_produced 4818

1473 film.actor.film 4819

1587 film.actor.film 4820

3469 music.artist.origin 4046

1193 film.actor.film 4821

3784 film.writer.film 4716

1187 film.actor.film 4822

3439 music.artist.origin 4199

545 music.artist.origin 4010

2217 film.actor.film 4823

3498 film.actor.film 4824

316 music.artist.origin 4058

2179 music.artist.origin 4825

3179 film.actor.film 4826

2460 film.actor.film 4827

2460 film.person_or_entity_appearing_in_film.film 4828

373 film.actor.film 4829

2458 film.person_or_entity_appearing_in_film.film 4830

602 music.producer.tracks_produced 4677

1981 film.actor.film 4831

3738 cvg.publisher.games_published 4832

3738 games.publisher.games_published 4833

184 film.person_or_entity_appearing_in_film.film 4834

3397 music.artist.origin 3966

625 film.person_or_entity_appearing_in_film.film 4835

3836 music.artist.album 4836

442 film.actor.film 4837

2360 film.actor.film 4838

2241 film.distributor.film 4839

2241 film.production_company.film 4840

1936 film.actor.film 4841

2779 film.person_or_entity_appearing_in_film.film 4842

1917 film.person_or_entity_appearing_in_film.film 4843

3644 music.artist.origin 4369

1618 music.artist.album 4844

3486 film.person_or_entity_appearing_in_film.film 4845

1567 film.person_or_entity_appearing_in_film.film 4846

643 film.actor.film 4847

2697 music.album.genre 4848

629 film.writer.film 4849

1378 film.person_or_entity_appearing_in_film.film 4850

2285 music.artist.origin 4851

3246 music.artist.origin 4852

3095 film.actor.film 4853

3048 film.actor.film 4854

1816 film.person_or_entity_appearing_in_film.film 4855

1057 film.actor.film 4856

3473 film.actor.film 4857

1358 film.actor.film 4858

2602 music.artist.origin 4211

2635 film.actor.film 3882

1633 film.person_or_entity_appearing_in_film.film 4859

1720 film.director.film4860

2109 people.person.place_of_birth 4861

1032 film.person_or_entity_appearing_in_film.film 4862

2734 film.person_or_entity_appearing_in_film.film 4863

1473 film.person_or_entity_appearing_in_film.film 4864

237 music.artist.origin 3864

3133 film.person_or_entity_appearing_in_film.film 4865

3133 film.person_or_entity_appearing_in_film.film 4866

1960 film.actor.film 4867

2421 people.person.place_of_birth 4868

2234 music.artist.origin 3961

3401 people.deceased_person.place_of_death 4869

77 film.actor.film 4870

1744 film.actor.film 4871

2636 film.actor.film 4872

2636 film.actor.film 4873

2597 film.person_or_entity_appearing_in_film.film 4874

2070 film.actor.film 4875

2351 people.person.place_of_birth 4876

160 people.person.place_of_birth 4877

1355 award.competitor.awards_won 4878

1353 film.person_or_entity_appearing_in_film.film 4879

484 film.actor.film 4880

3738 cvg.publisher.games_published 4881

3738 cvg.publisher.games_published 4882

3738 cvg.publisher.games_published 4883

3738 games.publisher.games_published 4884

2111 film.person_or_entity_appearing_in_film.film 4885

1154 film.actor.film 4886

2241 film.distributor.film 4887

2241 film.distributor.film 4888

2241 film.distributor.film 4889

2241 film.distributor.film 4890

2241 film.production_company.film 4891

2241 film.production_company.film 4892

988 music.artist.album 4893

1936 film.person_or_entity_appearing_in_film.film 4894

430 film.actor.film 4895

3646 location.location.contained_by 4896

207 music.artist.origin 4583

2189 film.person_or_entity_appearing_in_film.film 4897

979 music.artist.origin 4552

591 film.person_or_entity_appearing_in_film.film 4281

3234 event.agent.performance 4898

469 people.person.place_of_birth 4479

1880 music.artist.origin 3961

3002 people.person.place_of_birth 4013

3390 film.person_or_entity_appearing_in_film.film 4899

3390 film.person_or_entity_appearing_in_film.film 4900

2529 music.artist.origin 4901

1750 film.actor.film 4902

3542 film.actor.film 4822

2402 film.director.film 4366

2402 film.person_or_entity_appearing_in_film.film 4903

2106 music.artist.origin 4380

2635 film.actor.film 4904

2635 film.person_or_entity_appearing_in_film.film 4905

1157 film.actor.film 4906

2844 people.person.place_of_birth 4094

274 people.person.profession 4221

785 film.person_or_entity_appearing_in_film.film 4907

3827 film.actor.film 4908

1669 music.artist.origin 4909

3275 film.actor.film 4910

1400 film.actor.film 4911

1748 film.person_or_entity_appearing_in_film.film 4912

2050 film.person_or_entity_appearing_in_film.film 4913

67 film.person_or_entity_appearing_in_film.film 4914

67 film.person_or_entity_appearing_in_film.film 4915

2079 film.actor.film 4916

2370 film.actor.film 4917

2313 film.actor.film 4918

3364 music.artist.origin 4919

39 film.actor.film 4920

2737 film.actor.film 4921

3382 people.deceased_person.place_of_death 3846

1024 film.person_or_entity_appearing_in_film.film 4922

1407 film.person_or_entity_appearing_in_film.film 4923

249 people.person.place_of_birth 4191

2127 music.artist.origin 4183

283 music.artist.origin 4924

679 film.actor.film 4092

2781 film.actor.film 4753

133 award.competitor.award_nominations 4925

2460 film.person_or_entity_appearing_in_film.film 4926

1699 music.artist.origin 4927

484 film.person_or_entity_appearing_in_film.film 4928

3738 games.publisher.games_published 4929

901 film.person_or_entity_appearing_in_film.film 4930

3517 music.artist.origin 4931

2159 film.person_or_entity_appearing_in_film.film 4932

2867 film.actor.film 4933

2241 film.distributor.film 4934

2241 film.distributor.film 4935

2241 film.distributor.film 4936

2241 film.production_company.film 4937

2241 film.production_company.film 4938

988 film.actor.film 4939

2129 film.person_or_entity_appearing_in_film.film 4940

3350 music.artist.origin 4191

3430 film.actor.film 4941

1624 film.person_or_entity_appearing_in_film.film 4942

777 film.person_or_entity_appearing_in_film.film 4943

622 film.person_or_entity_appearing_in_film.film 4944

271 music.artist.origin 4454

3669 film.person_or_entity_appearing_in_film.film 4945

643 film.actor.film 3865

2731 film.actor.film 4946

538 film.person_or_entity_appearing_in_film.film 4947

3321 film.person_or_entity_appearing_in_film.film 4948

807 music.artist.origin 3866

2906 event.agent.performance 4949

2715 film.person_or_entity_appearing_in_film.film 4950

321 music.artist.origin 4454

2721 location.location.contained_by 4380

3544 film.actor.film 4951

3265 music.artist.origin 4952

997 film.actor.film 4953

726 music.artist.origin 3849

102 music.album.artist 3509

1157 film.actor.film 4954

3384 film.actor.film 4955

2633 film.person_or_entity_appearing_in_film.film 4956

2011 music.artist.origin 4957

785 film.person_or_entity_appearing_in_film.film 4922

3342 music.artist.origin 4058

3054 music.musician.instruments_played 4349

3608 location.location.contained_by 4454

2394 people.person.place_of_birth 4496

674 film.actor.film 4958

2683 film.actor.film 4959

2210 people.person.place_of_birth 4960

38 music.producer.tracks_produced 4961

3495 music.artist.origin 4150

1473 film.person_or_entity_appearing_in_film.film 4569

2337	film.actor.film	4962

189 people.person.place_of_birth	4963

895 film.actor.film	4964

895 film.person_or_entity_appearing_in_film.film	4965

3364	film.person_or_entity_appearing_in_film.film	4966

3204	film.actor.film	4967

66	film.actor.film	4968

3598	film.person_or_entity_appearing_in_film.film	4969

3109	film.person_or_entity_appearing_in_film.film	4970

3280	film.person_or_entity_appearing_in_film.film	4286

967 music.artist.album	4971

2562	film.person_or_entity_appearing_in_film.film	4972

2636	film.person_or_entity_appearing_in_film.film	4973

2437	film.actor.film	4974

3108	people.deceased_person.place_of_death	4975

2122	film.person_or_entity_appearing_in_film.film	4976

3016	music.artist.origin	4977

270 film.actor.film	4978

909 music.artist.origin	4977

2483	film.person_or_entity_appearing_in_film.film	4979

899 film.actor.film	4980

3673	music.producer.tracks_produced	4981

2867	film.person_or_entity_appearing_in_film.film	4982

2241	film.distributor.film	4983

2241	film.distributor.film	4984

2241	film.distributor.film	4985

2241	film.production_company.film	4986

2241	film.production_company.film	4987

2241	film.production_company.film	4988

530 music.artist.origin	4347

681 film.actor.film 4989

2692 people.person.profession 4990

581 music.artist.origin 4991

2245 film.actor.film 4992

3235 film.person_or_entity_appearing_in_film.film 4993

3002 film.actor.film 4994

538 film.person_or_entity_appearing_in_film.film 4995

3374 film.person_or_entity_appearing_in_film.film 4996

2207 film.person_or_entity_appearing_in_film.film 4997

3321 film.actor.film 4998

1332 people.person.place_of_birth 4999

2296 film.person_or_entity_appearing_in_film.film 5000

354 music.artist.origin 5001

3565 music.artist.origin 4013

3446 film.actor.film 5002

2130 film.actor.film 5003

68 film.person_or_entity_appearing_in_film.film 5004

231 music.artist.origin 3864

936 music.artist.origin 4380

997 film.person_or_entity_appearing_in_film.film 5005

1753 music.artist.origin 4232

1719 film.person_or_entity_appearing_in_film.film 3958

818 film.person_or_entity_appearing_in_film.film 5006

3827 film.actor.film 5007

492 film.actor.film 5008

871 music.artist.origin 4057

2811 music.artist.origin 5009

2050 film.person_or_entity_appearing_in_film.film 5010

3739 music.artist.origin 3866

205 film.actor.film 5011

1026 film.person_or_entity_appearing_in_film.film 5012

839 film.person_or_entity_appearing_in_film.film 5013

895 film.person_or_entity_appearing_in_film.film 4438

2603 film.person_or_entity_appearing_in_film.film 5014

2482 film.actor.film 5015

1706 film.person_or_entity_appearing_in_film.film 5016

2737 film.actor.film 5017

3123 music.album.genre 5018

325 music.artist.origin 5019

3344 film.actor.film 5020

2259 people.person.profession 5021

2913 film.actor.film 5022

151 film.person_or_entity_appearing_in_film.film 5023

1220 film.writer.film 5024

646 film.actor.film 5025

628 film.person_or_entity_appearing_in_film.film 5026

2636 film.actor.film 5027

2650 film.film.genre 4062

3663 film.person_or_entity_appearing_in_film.film 4407

1353 film.actor.film 4879

2460 film.actor.film 5028

79 film.actor.film 5029

1707 film.person_or_entity_appearing_in_film.film 4846

715 music.artist.origin 4310

3738 cvg.publisher.games_published 5030

1154 film.actor.film 5031

625 film.person_or_entity_appearing_in_film.film 5032

2577 people.person.place_of_birth 5033

2241 film.distributor.film 5034

2241 film.distributor.film 5035

2241 film.distributor.film 4840

2241 film.distributor.film 5036

2241 film.production_company.film 5037

2241 film.production_company.film 5038

2241 film.production_company.film 5039

3596 music.artist.album 5040

1600 music.artist.origin 4057

894 film.actor.film 5041

1767 film.person_or_entity_appearing_in_film.film 5042

1360 film.actor.film 5043

1360 film.actor.film 5044

1475 film.actor.film 5045

3312 film.person_or_entity_appearing_in_film.film 4385

2861 film.person_or_entity_appearing_in_film.film 5046

253 film.actor.film 5047

2244 film.film.producer 5048

1105 film.person_or_entity_appearing_in_film.film 5049

3742 film.director.film 5050

2658 film.person_or_entity_appearing_in_film.film 4706

917 music.artist.origin 5019

383 film.actor.film 5051

552 film.person_or_entity_appearing_in_film.film 5052

2635 film.actor.film 5053

1157 film.person_or_entity_appearing_in_film.film 5054

1633 film.person_or_entity_appearing_in_film.film 5055

1998 film.person_or_entity_appearing_in_film.film 5056

1833 film.person_or_entity_appearing_in_film.film 5057

2708 film.actor.film 5058

3788 film.person_or_entity_appearing_in_film.film 5059

67 film.person_or_entity_appearing_in_film.film 5060

2329	location.location.contained_by	5061
2337	film.person_or_entity_appearing_in_film.film	4492
181 film.person_or_entity_appearing_in_film.film	4065
2080	film.actor.film	5062
2713	film.actor.film	4725
672 film.person_or_entity_appearing_in_film.film	5063
3094	people.deceased_person.place_of_death	5064
2914	film.actor.film	5065
2830	film.actor.film	4168
628 film.person_or_entity_appearing_in_film.film	5066
2562	film.actor.film	5067
70	film.actor.film	5068
1453	film.actor.film	5069
368 people.person.place_of_birth	5070
2095	film.person_or_entity_appearing_in_film.film	5071
2636	film.person_or_entity_appearing_in_film.film	5072
445 film.actor.film	5073
1353	film.actor.film	4778
79	film.person_or_entity_appearing_in_film.film	4614
2682	music.artist.album	5074
3738	cvg.publisher.games_published	5075
3738	cvg.publisher.games_published	5076
625 film.person_or_entity_appearing_in_film.film	5077
1448	music.artist.origin	4294
1002	film.actor.film	5078
1249	film.actor.film	5079
442 music.artist.album	5080
3501	film.person_or_entity_appearing_in_film.film	5081
2241	film.distributor.film	5082
2241	film.distributor.film	5083

2241 film.production_company.film 4685

1942 film.person_or_entity_appearing_in_film.film 5084

451 people.person.profession 4221

2487 film.actor.film 5085

2311 film.actor.film 5086

14 music.artist.origin 4133

1567 film.actor.film 5087

360 music.artist.origin 4232

406 film.person_or_entity_appearing_in_film.film 5088

1806 film.actor.film 5089

371 film.actor.film 5090

1475 film.person_or_entity_appearing_in_film.film 4652

629 film.writer.film 4585

3458 film.person_or_entity_appearing_in_film.film 5091

57 film.person_or_entity_appearing_in_film.film 4476

3026 film.person_or_entity_appearing_in_film.film 5092

3650 film.actor.film 5093

3279 film.person_or_entity_appearing_in_film.film 5094

3132 film.person_or_entity_appearing_in_film.film 5095

873 film.actor.film 5096

2089 event.agent.performance 5097

997 film.person_or_entity_appearing_in_film.film 5098

102 music.album.track 5099

981 music.artist.origin 5100

1753 event.agent.performance 5101

3384 film.person_or_entity_appearing_in_film.film 5102

1045 film.actor.film 5103

3827 film.person_or_entity_appearing_in_film.film 4908

641 film.person_or_entity_appearing_in_film.film 5104

3317 music.artist.origin 5105

1300 music.artist.origin 5106

1111 music.artist.origin 4419

2737 film.person_or_entity_appearing_in_film.film 5107

3757 film.person_or_entity_appearing_in_film.film 5108

3396 film.person_or_entity_appearing_in_film.film 5109

976 film.person_or_entity_appearing_in_film.film 5110

3121 film.person_or_entity_appearing_in_film.film 5111

3167 film.actor.film 5112

2338 film.person_or_entity_appearing_in_film.film 5113

74 film.person_or_entity_appearing_in_film.film 5114

437 film.person_or_entity_appearing_in_film.film 5115

2636 film.person_or_entity_appearing_in_film.film 5116

160 film.actor.film 5117

3179 film.actor.film 5118

2460 film.person_or_entity_appearing_in_film.film 5119

602 film.actor.film 5120

2877 film.actor.film 5121

3738 cvg.publisher.games_published 5122

3738 cvg.publisher.games_published 5123

3738 games.publisher.games_published 4342

999 film.person_or_entity_appearing_in_film.film 5124

978 people.person.place_of_birth 5125

2577 film.actor.film 5126

1003 music.artist.origin 5127

2241 film.distributor.film 5128

2241 film.distributor.film 5129

2241 film.distributor.film 5130

2241 film.production_company.film 5131

2241 film.production_company.film 5132

2241 film.production_company.film 5133

2241 film.production_company.film 5134

2241 film.production_company.film 5135

2241 film.production_company.film 4737

2241 film.production_company.film 5136

2380 people.person.place_of_birth 4050

795 film.person_or_entity_appearing_in_film.film 5137

1936 film.person_or_entity_appearing_in_film.film 5138

451 people.person.place_of_birth 5139

2707 film.person_or_entity_appearing_in_film.film 5140

1357 film.person_or_entity_appearing_in_film.film 5141

2531 music.artist.origin 4057

2870 film.person_or_entity_appearing_in_film.film 5142

3321 film.person_or_entity_appearing_in_film.film 4998

2843 film.person_or_entity_appearing_in_film.film 5143

2573 music.artist.origin 5061

122 music.artist.origin 3963

2372 film.person_or_entity_appearing_in_film.film 5144

2593 music.artist.origin 4157

204 film.person_or_entity_appearing_in_film.film 5145

206 film.person_or_entity_appearing_in_film.film 5146

3264 music.artist.origin 4416

3375 music.artist.origin 4077

3640 music.artist.origin 4362

379 film.actor.film 5147

115 film.person_or_entity_appearing_in_film.film 5148

541 film.actor.film 5149

597 film.actor.film 5150

303 music.artist.origin 5151

1254 music.artist.origin 4191

1748 film.person_or_entity_appearing_in_film.film 5152

1488	award.nominee.award_nominations	5153
633 film.person_or_entity_appearing_in_film.film	5154
2712	film.actor.film	5155
774 film.person_or_entity_appearing_in_film.film	5156
38	music.artist.origin	4058
1473	film.actor.film	5157
2990	film.actor.film	5158
1629	music.artist.origin	4057
124 film.person_or_entity_appearing_in_film.film	5159
2699	music.artist.origin	5160
1043	music.musician.instruments_played	4349
145 people.person.place_of_birth	5161
2454	film.actor.film	5162
604 film.person_or_entity_appearing_in_film.film	4382
2080	film.actor.film	5163
3598	film.person_or_entity_appearing_in_film.film	5164
3080	music.artist.origin	3864
809 film.person_or_entity_appearing_in_film.film	5165
2913	film.actor.film	5166
2913	film.person_or_entity_appearing_in_film.film	5167
3830	music.artist.origin	5168
2772	music.artist.origin	4957
510 music.producer.tracks_produced	5169
70	film.actor.film	5170
2636	film.actor.film	5171
2510	music.artist.origin	4057
2460	film.actor.film	5172
79	film.actor.film	5173
724 tv.program.country_of_origin	4815
1840	music.musician.instruments_played	5174

602 film.actor.film 5175

2483 film.person_or_entity_appearing_in_film.film 5176

2877 film.actor.film 4072

3738 cvg.publisher.games_published 5177

3738 games.publisher.games_published 5178

3738 games.publisher.games_published 5179

901 award.nominee.award_nominations 5180

2864 people.person.place_of_birth 5181

1436 music.album.genre 5018

2241 film.distributor.film 5182

2241 film.production_company.film 5183

2241 film.production_company.film 4567

2241 film.production_company.film 5184

988 film.person_or_entity_appearing_in_film.film 5185

697 music.artist.origin 3864

2126 film.person_or_entity_appearing_in_film.film 5186

1567 film.person_or_entity_appearing_in_film.film 5087

643 film.person_or_entity_appearing_in_film.film 5187

1278 award.competitor.awards_won 5188

3242 film.actor.film 5189

434 film.person_or_entity_appearing_in_film.film 5190

1526 film.actor.film 5191

600 music.artist.album 4495

3321 film.actor.film 5192

2193 music.artist.origin 4232

1623 film.actor.film 5193

1149 music.artist.origin 4456

168 music.artist.origin 5194

244 people.person.place_of_birth 3972

2296 people.person.place_of_birth 4094

3369 people.deceased_person.place_of_death 4496

3520 music.artist.origin 3946

2449 film.actor.film 4645

68 film.actor.film 3879

2788 film.actor.film 5195

206 film.actor.film 5196

206 film.person_or_entity_appearing_in_film.film 5197

129 film.person_or_entity_appearing_in_film.film 5198

2402 film.actor.film 5199

3613 film.actor.film 5200

1092 event.agent.performance 4251

2605 music.album.genre 5201

379 film.person_or_entity_appearing_in_film.film 5202

2494 film.person_or_entity_appearing_in_film.film 3868

541 film.actor.film 5203

2724 film.actor.film 4136

1505 film.actor.film 4319

3317 film.actor.film 5204

499 music.artist.origin 3864

116 film.actor.film 5205

1032 film.actor.film 5206

67 film.person_or_entity_appearing_in_film.film 5207

304 music.artist.origin 5208

2750 film.film.writer 5209

2944 film.actor.film 5210

803 music.artist.origin 5211

2265 film.person_or_entity_appearing_in_film.film 5212

2608 music.artist.origin 5213

1227 music.artist.origin 4456

2637 film.film.director5214

361 music.artist.track 5215

646 film.person_or_entity_appearing_in_film.film 5216

1404 film.actor.film 5217

2070 film.person_or_entity_appearing_in_film.film 5218

1355 film.actor.film 5219

688 music.artist.origin 4121

1255 music.musician.instruments_played 4349

1353 film.person_or_entity_appearing_in_film.film 5220

1353 film.person_or_entity_appearing_in_film.film 5221

3738 cvg.publisher.games_published 5222

2537 music.artist.origin 4442

1249 film.person_or_entity_appearing_in_film.film 3935

1872 film.actor.film 4510

2241 film.distributor.film 5223

2241 film.distributor.film 4938

2241 film.production_company.film 5224

2241 film.production_company.film 5225

3172 music.artist.origin 4108

2144 people.deceased_person.place_of_death 3942

644 music.artist.origin 5226

816 people.person.place_of_birth 5227

1877 people.person.place_of_birth 5228

2126 film.actor.film 5229

3775 people.person.place_of_birth 5230

1860 people.person.place_of_birth 4796

629 film.director.film 4849

1734 music.artist.origin 4024

2592 people.person.place_of_birth 5231

3180 people.person.place_of_birth 5232

1623 film.person_or_entity_appearing_in_film.film 5233

3752 music.musician.instruments_played 5174

3360 people.person.profession 4990

916 film.actor.film 5234

2010 film.actor.film 5235

3660 music.artist.origin 4454

1280 people.person.place_of_birth 5236

3774 film.film.genre 4285

2635 film.actor.film 5237

1893 people.person.place_of_birth 5238

3111 people.deceased_person.place_of_death 4455

1153 film.person_or_entity_appearing_in_film.film 5239

358 type.object.subject_key 358

2337 film.actor.film 5240

861 film.person_or_entity_appearing_in_film.film 5241

1960 film.actor.film 5242

314 people.person.place_of_birth 4422

3762 music.artist.origin 5243

1625 film.person_or_entity_appearing_in_film.film 5244

3233 event.agent.performance 5097

3092 music.artist.origin 4852

1704 film.person_or_entity_appearing_in_film.film 5245

3691 music.artist.origin 5246

3159 people.person.place_of_birth 5247

1458 film.person_or_entity_appearing_in_film.film 5248

3663 people.person.place_of_birth 4013

3166 film.actor.film 4777

1353 film.person_or_entity_appearing_in_film.film 5249

2460 film.actor.film 5250

1308 music.artist.track 5251

3738 cvg.publisher.games_published 5252

436 film.actor.film 5253

2430 film.person_or_entity_appearing_in_film.film 5254

2241 film.distributor.film 5255

3200 music.artist.origin 5256

2487 film.actor.film 5257

3143 tv.program.country_of_origin 3961

3399 music.artist.origin 3916

1218 people.person.place_of_birth 5258

1159 music.artist.origin 5259

3242 film.producer.film 4379

1536 film.actor.film 4418

1526 film.person_or_entity_appearing_in_film.film 5260

2429 film.actor.film 5261

2847 music.artist.origin 4957

740 music.artist.origin 5262

3187 music.artist.origin 4732

2711 film.actor.film 5263

2711 film.actor.film 5264

586 music.artist.origin 4199

29 music.artist.origin 5265

2296 film.actor.film 4209

806 music.artist.origin 5266

68 film.actor.film 5267

68 film.person_or_entity_appearing_in_film.film 5268

2684 film.actor.film 5269

383 people.person.place_of_birth 5270

3381 film.person_or_entity_appearing_in_film.film 4107

567 film.actor.film 5271

1633 film.person_or_entity_appearing_in_film.film 4005

2792 music.artist.origin 5272

492 film.actor.film 4657

2441 film.actor.film 5273

53 event.agent.performance 4251

2786 film.actor.film 5274

2953 people.person.place_of_birth 5275

358 film.actor.film 5276

1525 film.actor.film 5277

67 film.actor.film 5278

67 film.person_or_entity_appearing_in_film.film 5279

67 film.person_or_entity_appearing_in_film.film 4802

2359 film.actor.film 5280

1473 film.actor.film 5281

1473 film.person_or_entity_appearing_in_film.film 5282

793 film.person_or_entity_appearing_in_film.film 4802

3128 music.artist.origin 4800

3040 film.director.film 5283

2737 film.person_or_entity_appearing_in_film.film 5284

407 film.person_or_entity_appearing_in_film.film 5285

695 film.actor.film 4413

2338 film.person_or_entity_appearing_in_film.film 5286

2718 music.artist.origin 3961

2460 film.producer.film 4559

1848 music.artist.origin 5287

3738 games.publisher.games_published 5288

1277 film.actor.film 5289

901 film.director.film 5290

1513 music.artist.album 5291

1249 film.person_or_entity_appearing_in_film.film 5292

2867 film.actor.film 5293

2241 film.distributor.film 4751

2241 film.distributor.film 5294

2241 film.distributor.film 5295

2241 film.distributor.film 5296

2241 film.distributor.film 5297

2241 film.production_company.film 5298

795 film.actor.film 5299

1824 people.person.place_of_birth 4784

3190 music.musician.instruments_played 3959

33 award.nominee.award_nominations 5300

2487 film.person_or_entity_appearing_in_film.film 5301

2692 music.musician.instruments_played 3959

1697 film.actor.film 4772

2272 film.person_or_entity_appearing_in_film.film 5302

2880 film.actor.film 5303

1651 people.person.place_of_birth 4362

2206 people.person.place_of_birth 4262

3547 tv.program.genre 4285

1059 film.actor.film 5304

2184 film.person_or_entity_appearing_in_film.film 5305

574 music.artist.origin 3855

2870 film.person_or_entity_appearing_in_film.film 3995

3321 film.actor.film 5306

2634 film.person_or_entity_appearing_in_film.film 5307

385 film.actor.film 5308

2509 film.person_or_entity_appearing_in_film.film 5309

2452 film.actor.film 5310

2584 film.person_or_entity_appearing_in_film.film 5311

1962 film.actor.film 5312

3273 people.person.place_of_birth 4010

2494 film.actor.film 4581

435 film.actor.film 4372

3020 film.person_or_entity_appearing_in_film.film 5313

67 film.actor.film 5314

2804 film.actor.film 5315

1473 film.actor.film 5316

78 music.artist.origin 4362

2552 music.artist.origin 3859

3784 film.writer.film 4601

3733 film.actor.film 5317

2341 film.person_or_entity_appearing_in_film.film 5318

2080 film.actor.film 5319

2913 film.actor.film 5320

2972 film.person_or_entity_appearing_in_film.film 5321

2710 music.artist.album 5322

2053 film.person_or_entity_appearing_in_film.film 5323

2095 film.person_or_entity_appearing_in_film.film 5324

21 film.person_or_entity_appearing_in_film.film 4646

3355 people.person.place_of_birth 4977

2597 film.actor.film 5325

2070 film.actor.film 5218

270 film.actor.film 5326

3738 cvg.publisher.games_published 5327

3738 cvg.publisher.games_published 5328

436 film.actor.film 4782

436 film.person_or_entity_appearing_in_film.film 5329

436 film.person_or_entity_appearing_in_film.film 5330

901 film.actor.film 4140

1694 film.actor.film 5331

1249 film.person_or_entity_appearing_in_film.film 5332

1249 film.person_or_entity_appearing_in_film.film 5333

2159 film.person_or_entity_appearing_in_film.film 5334

2241 film.distributor.film 3990

2241 film.distributor.film 5335

2241 film.distributor.film 5336

2241 film.production_company.film 5337

2241 film.production_company.film 5338

3297 film.actor.film 5339

681 people.person.place_of_birth 4057

1624 film.actor.film 5340

591 film.actor.film 4409

894 film.person_or_entity_appearing_in_film.film 5341

261 film.person_or_entity_appearing_in_film.film 4412

418 film.person_or_entity_appearing_in_film.film 5342

2100 film.actor.film 5343

2526 music.artist.origin 5344

3321 film.actor.film 5345

295 music.artist.origin 3864

898 film.person_or_entity_appearing_in_film.film 5346

2878 music.artist.origin 4277

814 film.person_or_entity_appearing_in_film.film 5347

3378 people.person.place_of_birth 4479

1323 people.person.place_of_birth 5348

1474 film.actor.film 5349

3528 music.artist.origin 4195

637 film.actor.film 5350

721 film.actor.film 5351

3475 film.actor.film 5352

350 music.artist.origin 3961

2402 film.actor.film 4903

115 film.actor.film 5353

2084 film.person_or_entity_appearing_in_film.film 5354

815 film.person_or_entity_appearing_in_film.film 5355

3054 film.actor.film 5356

404 music.artist.origin 5357

2359 film.person_or_entity_appearing_in_film.film 5280

1692 film.actor.film 5358

3364 film.actor.film 5359

3232 people.person.place_of_birth 4616

2893 film.person_or_entity_appearing_in_film.film 5360

645 film.person_or_entity_appearing_in_film.film 5361

1909 music.artist.origin 5362

789 film.actor.film 3906

675 film.actor.film 4267

46 film.actor.film 5363

628 film.person_or_entity_appearing_in_film.film 5364

2095 film.person_or_entity_appearing_in_film.film 5365

1846 film.person_or_entity_appearing_in_film.film 5366

2588 music.musician.instruments_played 5367

3179 film.person_or_entity_appearing_in_film.film 5368

3823 music.artist.origin 4445

2412 people.person.profession 4221

484 film.actor.film 5369

484 film.person_or_entity_appearing_in_film.film 5370

3738 cvg.publisher.games_published 5371

3738 cvg.publisher.games_published 4243

3738 games.publisher.games_published 5372

3738 games.publisher.games_published 5373

436 film.person_or_entity_appearing_in_film.film 5374

436 film.person_or_entity_appearing_in_film.film 5375

2111 film.actor.film 5376

901 film.actor.film 5377

625 film.actor.film 4483

551 people.person.place_of_birth 4158

791 film.person_or_entity_appearing_in_film.film 5378

1249 film.actor.film 3901

432 film.person_or_entity_appearing_in_film.film 5379

432 film.person_or_entity_appearing_in_film.film 5380

2867 film.person_or_entity_appearing_in_film.film 5381

3836 music.artist.origin 4174

2241 film.distributor.film 5382

2516 music.artist.origin 4851

2769 music.artist.origin 4174

252 music.artist.origin 5383

1360 film.person_or_entity_appearing_in_film.film 5384

2436 music.artist.origin 4174

2390 film.person_or_entity_appearing_in_film.film 5385

534 people.person.place_of_birth 3932

1567 film.actor.film 4915

2696 people.person.place_of_birth 5386

865 film.actor.film 4200

3607 film.actor.film 5387

341 film.film.actor 5388

1475 film.actor.film 4308

1580 people.person.profession 4221

538 film.actor.film 5389

3052 people.person.place_of_birth 3916

1810 music.artist.origin 5390

2279 film.person_or_entity_appearing_in_film.film 5391

3648 film.actor.film 5392

553 music.album.genre 4848

3132	film.person_or_entity_appearing_in_film.film	5393

2449	film.actor.film	5394

2343	music.artist.album	5395

2306	film.actor.film	5396

3446	music.artist.origin	5270

1955	film.person_or_entity_appearing_in_film.film	5397

1358	film.actor.film	4649

2833	film.person_or_entity_appearing_in_film.film	5398

2635	film.actor.film	5399

2635	film.person_or_entity_appearing_in_film.film	5400

3126	music.artist.origin	5401

1989	film.actor.film	5402

2174	music.artist.origin	5403

1505	film.actor.film	5404

504 music.artist.origin	5405

1582	people.person.place_of_birth	5236

2786	film.actor.film	5406

2708	film.actor.film	5407

3788	film.actor.film	5408

67	film.actor.film	5409

3412	film.film.language	4076

1473	film.actor.film	5410

3311	music.artist.origin	5411

2337	film.actor.film	5412

3567	music.artist.origin	5413

91	film.person_or_entity_appearing_in_film.film	5414

145 film.person_or_entity_appearing_in_film.film	4237

2454	music.musician.instruments_played	4349

2080	film.actor.film	5415

3094	film.actor.film	5416

1744 film.person_or_entity_appearing_in_film.film 5417

646 film.person_or_entity_appearing_in_film.film 5418

3120 people.deceased_person.place_of_death 4057

447 film.person_or_entity_appearing_in_film.film 5419

212 music.artist.origin 5420

603 music.artist.origin 5421

1846 event.agent.performance 4099

2636 film.actor.film 5422

2597 film.actor.film 4776

2070 film.person_or_entity_appearing_in_film.film 5423

1370 film.film.genre 4285

3417 music.artist.origin 4963

484 film.actor.film 5424

484 film.person_or_entity_appearing_in_film.film 5425

2483 people.person.place_of_birth 3984

3738 cvg.publisher.games_published 5426

3738 cvg.publisher.games_published 5427

3738 games.publisher.games_published 5428

901 film.person_or_entity_appearing_in_film.film 5429

3260 music.artist.origin 5430

3248 award.competitor.awards_won 5431

2867 film.actor.film 5432

2241 film.distributor.film 5433

2241 film.distributor.film 5434

2241 film.distributor.film 5435

2241 film.distributor.film 5436

2241 film.production_company.film 5437

54 music.artist.origin 3859

2487 film.person_or_entity_appearing_in_film.film 5438

534 music.musician.instruments_played 4349

159 music.artist.origin 4157

2741 people.person.place_of_birth 4171

25 film.actor.film 5439

2531 film.person_or_entity_appearing_in_film.film 5440

1089 film.actor.film 5441

195 music.artist.origin 4087

3390 film.actor.film 5442

2919 people.person.place_of_birth 4977

1632 film.actor.film 5443

2010 film.actor.film 5444

449 music.musician.instruments_played 3959

3599 film.actor.film 5445

2405 people.person.place_of_birth 4108

1720 film.director.film 5446

1989 film.person_or_entity_appearing_in_film.film 5447

913 music.artist.origin 4013

402 music.artist.origin 5106

2370 film.person_or_entity_appearing_in_film.film 5448

1473 film.person_or_entity_appearing_in_film.film 5449

895 film.person_or_entity_appearing_in_film.film 5450

2389 film.person_or_entity_appearing_in_film.film 5451

91 film.person_or_entity_appearing_in_film.film 5452

377 type.object.subject_key 377

1192 music.artist.origin 4013

3060 music.artist.origin 5453

1229 film.actor.film 5454

1895 film.actor.film 5455

2460 film.person_or_entity_appearing_in_film.film 5456

2460 film.producer.film 5457

2136 people.deceased_person.place_of_death 4496

2412 film.actor.film 5458

484 film.actor.film 5459

2208 music.artist.album 5460

3738 games.publisher.games_published 5461

3738 games.publisher.games_published 5462

3738 games.publisher.games_published 5463

3738 games.publisher.games_published 5464

436 film.person_or_entity_appearing_in_film.film 5465

1277 people.person.profession 4221

3518 music.artist.origin 3864

899 film.person_or_entity_appearing_in_film.film 5466

2241 film.distributor.film 5467

2241 film.distributor.film 5468

2241 film.production_company.film 4935

2241 film.production_company.film 5469

795 film.person_or_entity_appearing_in_film.film 5470

1936 film.actor.film 5138

135 film.actor.film 5471

1618 film.person_or_entity_appearing_in_film.film 4406

498 music.artist.track 5472

591 film.person_or_entity_appearing_in_film.film 5473

1656 film.actor.film 5474

3486 type.object.key 3486

1360 film.person_or_entity_appearing_in_film.film 5475

194 film.person_or_entity_appearing_in_film.film 5476

434 film.actor.film 5477

3002 film.person_or_entity_appearing_in_film.film 5478

1684 music.artist.origin 3849

696 film.actor.film 5479

1310 people.person.place_of_birth 3864

2655 music.artist.origin 3859

1931 music.artist.origin 4057

3648 film.person_or_entity_appearing_in_film.film 5480

3508 film.actor.film 4846

721 people.person.place_of_birth 5481

448 film.actor.film 5482

2205 film.person_or_entity_appearing_in_film.film 5483

997 film.actor.film 5484

997 film.person_or_entity_appearing_in_film.film 5484

1034 film.person_or_entity_appearing_in_film.film 5485

2833 film.actor.film 4740

396 film.actor.film 5486

1069 music.artist.origin 5403

2378 award.competitor.award_nominations 5487

540 film.actor.film 5488

2494 film.actor.film 5489

1525 film.actor.film 5490

2018 award.nominee.award_nominations 5491

2737 film.actor.film 5492

2893 film.actor.film 5360

976 film.actor.film 5493

1035 film.person_or_entity_appearing_in_film.film 5494

2338 film.person_or_entity_appearing_in_film.film 5495

3512 music.artist.origin 4362

1702 film.actor.film 4846

2044 music.artist.origin 4701

3183 people.person.place_of_birth 4768

308 music.artist.album 5496

2636 film.actor.film 5116

2561 music.artist.origin 5497

2819 people.person.profession 4221

769 music.artist.origin 4057

445 film.actor.film 5498

133 award.nominee.award_nominations 5499

1645 music.artist.origin 4087

1330 film.person_or_entity_appearing_in_film.film 5500

3738 cvg.publisher.games_published 5501

3738 cvg.publisher.games_published 5502

2111 film.actor.film 4885

339 people.person.place_of_birth 5503

897 music.artist.origin 5236

2241 film.distributor.film 5504

2241 film.distributor.film 5505

2487 film.person_or_entity_appearing_in_film.film 5506

2487 film.person_or_entity_appearing_in_film.film 5507

2078 film.person_or_entity_appearing_in_film.film 5508

2260 people.person.place_of_birth 5509

3354 award.nominee.award_nominations 5510

3669 film.actor.film 5511

2098 people.person.place_of_birth 5512

1806 film.actor.film 5513

2960 music.artist.origin 4071

434 film.person_or_entity_appearing_in_film.film 5514

3670 film.actor.film 5515

1270 music.artist.origin 4057

2277 music.artist.origin 5151

1376 music.artist.origin 4362

3321 film.actor.film 5516

3052 film.actor.film 5517

1597 film.person_or_entity_appearing_in_film.film 5518

2711 film.person_or_entity_appearing_in_film.film 5519

3763 film.person_or_entity_appearing_in_film.film 5520

1127 music.artist.origin 5521

2223 film.actor.film 5522

1985 people.person.place_of_birth 5523

1358 film.actor.film 5524

383 film.person_or_entity_appearing_in_film.film 4271

480 music.artist.origin 3961

3384 film.actor.film 3884

2362 film.actor.film 5525

785 film.person_or_entity_appearing_in_film.film 5526

492 people.person.place_of_birth 3984

2079 film.person_or_entity_appearing_in_film.film 5527

2712 film.actor.film 5528

774 film.actor.film 5156

38 music.artist.track 5529

450 music.artist.origin 3945

895 film.actor.film 4545

3364 film.actor.film 5530

2482 film.person_or_entity_appearing_in_film.film 5531

1209 film.actor.film 5532

109 film.actor.film 5533

3109 film.person_or_entity_appearing_in_film.film 4381

52 film.actor.film 5534

672 film.actor.film 5535

1091 music.artist.origin 3946

2636 film.actor.film 5536

2636 film.actor.film 5537

2636 film.person_or_entity_appearing_in_film.film 5538

250 music.artist.origin 3885

160 film.person_or_entity_appearing_in_film.film 5117

427 film.actor.film 5539

1355 film.person_or_entity_appearing_in_film.film 5219

1693 people.person.place_of_birth 3864

838 people.person.place_of_birth 4148

1840 film.actor.film 3854

3738 cvg.publisher.games_published 5540

3738 cvg.publisher.games_published 5541

436 film.actor.film 5375

1154 film.person_or_entity_appearing_in_film.film 5542

2400 film.actor.film 5543

223 film.person_or_entity_appearing_in_film.film 5544

2867 film.actor.film 5545

442 award.competitor.award_nominations 5546

2241 film.distributor.film 5547

2241 film.distributor.film 4573

2241 film.production_company.film 4798

1936 film.actor.film 5548

3706 film.actor.film 5549

956 film.actor.film 5550

1656 film.actor.film 5551

1620 music.artist.origin 3971

2206 film.person_or_entity_appearing_in_film.film 5552

719 film.person_or_entity_appearing_in_film.film 5553

2565 music.artist.origin 4362

3049 film.actor.film 5554

277 film.actor.film 5555

2836 people.person.place_of_birth 5556

2520 music.artist.origin 5557

2263 music.musician.instruments_played 5558

1654 people.person.place_of_birth 5559

3279 film.actor.film 5560

2770 film.actor.film 5561

2296 film.actor.film 5562

3508 film.actor.film 3931

3763 film.actor.film 5520

3199 film.person_or_entity_appearing_in_film.film 4048

735 music.artist.origin 5563

3381 film.person_or_entity_appearing_in_film.film 5564

567 film.person_or_entity_appearing_in_film.film 5271

375 film.person_or_entity_appearing_in_film.film 4001

1157 film.actor.film 5565

623 film.actor.film 5566

3111 film.actor.film 5567

1153 film.person_or_entity_appearing_in_film.film 5568

2978 people.person.place_of_birth 5569

171 music.artist.origin 4133

3317 film.actor.film 5570

1833 film.person_or_entity_appearing_in_film.film 5571

307 film.person_or_entity_appearing_in_film.film 5572

1383 film.actor.film 5573

2324 film.film.writer 5574

738 film.actor.film 5575

738 film.person_or_entity_appearing_in_film.film 5575

2983 film.person_or_entity_appearing_in_film.film 5576

3598 film.actor.film 5577

3498 film.person_or_entity_appearing_in_film.film 4382

619 film.actor.film 5578

809 film.person_or_entity_appearing_in_film.film 4336

672 film.actor.film 5579

3379 music.artist.origin 3945

2830 film.person_or_entity_appearing_in_film.film 5580

974 film.actor.film 5581

974 film.person_or_entity_appearing_in_film.film 5581

2070 film.actor.film 5582

2070 film.person_or_entity_appearing_in_film.film 5583

178 film.actor.film 5584

1458 film.actor.film 5585

3166 film.actor.film 3920

1353 film.person_or_entity_appearing_in_film.film 5586

1173 film.actor.film 4779

1579 film.person_or_entity_appearing_in_film.film 5587

1579 film.person_or_entity_appearing_in_film.film 5588

1154 film.person_or_entity_appearing_in_film.film 5589

3452 film.person_or_entity_appearing_in_film.film 5590

625 film.actor.film 5077

3400 film.actor.film 5591

2241 film.distributor.film 5592

2241 film.distributor.film 5593

2241 film.distributor.film 5594

2241 film.distributor.film 5595

2241 film.distributor.film 5596

2241 film.production_company.film 5597

2241 film.production_company.film 5598

2241 film.production_company.film 5599

1676 film.actor.film 4692

420 film.actor.film 5600

556 film.actor.film 5601

777 film.actor.film 5602

2126 film.person_or_entity_appearing_in_film.film 5603

356 music.artist.track 5604

113 music.artist.origin 5605

3354 film.actor.film 4792

2275 film.actor.film 5606

2206 film.actor.film 5607

3743 music.artist.origin 4456

2767 film.actor.film 5608

3049 film.actor.film 5609

3321 film.actor.film 5610

3557 film.film.executive_producer 5611

2711 film.actor.film 5612

1816 film.person_or_entity_appearing_in_film.film 5613

1348 film.writer.film 5614

1074 music.producer.tracks_produced 5615

2635 film.actor.film 5616

186 people.person.place_of_birth 5617

410 film.actor.film 5618

785 film.person_or_entity_appearing_in_film.film 4619

155 music.musician.instruments_played 5619

1891 film.person_or_entity_appearing_in_film.film 4976

847 music.artist.origin 4094

3046 film.person_or_entity_appearing_in_film.film 5620

1367 music.artist.origin 4010

2125 film.person_or_entity_appearing_in_film.film 5621

3792 film.person_or_entity_appearing_in_film.film 5622

2344 music.artist.origin 5623

3058 music.artist.origin 5453

550 people.person.place_of_birth 4133

66 film.actor.film 5624

789 film.person_or_entity_appearing_in_film.film 5625

585 music.artist.origin 4167

809 film.person_or_entity_appearing_in_film.film 5626

3419 music.artist.origin 5627

1661 film.person_or_entity_appearing_in_film.film 5628

1469 film.person_or_entity_appearing_in_film.film 4637

1601 film.actor.film 5629

1404 film.person_or_entity_appearing_in_film.film 5217

2636 film.actor.film 5630

2636 film.person_or_entity_appearing_in_film.film 5631

1573 film.person_or_entity_appearing_in_film.film 5632

529 film.actor.film 4240

2460 film.person_or_entity_appearing_in_film.film 5633

3738 cvg.publisher.games_published 5634

3738 games.publisher.games_published 5635

3738 games.publisher.games_published 5636

1154 film.person_or_entity_appearing_in_film.film 5543

1463 award.nominee.award_nominations 5637

1003 film.actor.film 5638

2241 film.distributor.film 4514

2232 music.musician.instruments_played 3959

3140 film.person_or_entity_appearing_in_film.film 4253

502 music.artist.origin 5639

1461 film.actor.film 4710

1767 film.actor.film 5640

261 music.producer.tracks_produced 5641

3486 film.actor.film 5642

3486 film.actor.film 5643

643 people.person.place_of_birth 3942

2358 music.artist.origin 3947

1042 music.artist.origin 3946

1399	film.actor.film	5644

1346	film.person_or_entity_appearing_in_film.film	4021

3578	music.artist.origin	4362

2551	music.artist.origin	3916

2207	film.actor.film	4846

3509	film.actor.film	5645

1046	film.actor.film	5646

157 music.producer.tracks_produced	5647

1105	film.person_or_entity_appearing_in_film.film	5648

3688	film.actor.film	5649

2449	film.actor.film	5650

2419	film.person_or_entity_appearing_in_film.film	5651

997 film.actor.film	5652

2635	film.person_or_entity_appearing_in_film.film	5653

464 film.director.film 5654

1157	film.actor.film	4535

115 film.person_or_entity_appearing_in_film.film	5655

2668	people.person.place_of_birth	3945

3582	film.actor.film	4434

3530	music.artist.origin	5656

3317	film.actor.film	5657

3788	film.person_or_entity_appearing_in_film.film	4218

3416	music.artist.origin	4134

1476	music.artist.album	5658

1473	film.person_or_entity_appearing_in_film.film	5659

1155	film.actor.film	4613

265 music.artist.origin	5660

2337	film.actor.film	5661

3040	film.person_or_entity_appearing_in_film.film	4355

1422	music.artist.origin	4057

2784 music.musician.instruments_played 5174

458 people.person.place_of_birth 5662

357 film.person_or_entity_appearing_in_film.film 5663

134 people.person.place_of_birth 5664

21 film.actor.film 4646

2636 film.actor.film 5665

1016 music.artist.origin 5666

3355 film.actor.film 5667

3449 music.artist.origin 4693

1353 film.actor.film 5668

838 music.artist.origin 4471

3738 games.publisher.games_published 5669

436 film.actor.film 5465

2241 film.distributor.film 5670

2241 film.distributor.film 5597

2241 film.production_company.film 5671

1942 film.actor.film 5084

1492 music.artist.origin 3961

1347 people.deceased_person.place_of_death 3947

1877 film.person_or_entity_appearing_in_film.film 5672

2126 film.actor.film 5673

3564 music.artist.origin 3946

2479 music.artist.origin 4003

3110 people.deceased_person.place_of_death 4134

157 event.agent.performance 4770

3231 film.person_or_entity_appearing_in_film.film 5674

3557 film.film.music 1044

794 film.person_or_entity_appearing_in_film.film 5675

843 people.person.place_of_birth 4098

3648 film.person_or_entity_appearing_in_film.film 5676

2658 film.actor.film 5677

3767 music.artist.origin 3864

687 film.person_or_entity_appearing_in_film.film 5678

3822 film.actor.film 5679

1358 film.actor.film 5680

2771 film.actor.film 5681

2833 film.actor.film 4494

2904 film.person_or_entity_appearing_in_film.film 5682

3599 film.person_or_entity_appearing_in_film.film 5683

1349 film.actor.film 5684

3332 people.person.place_of_birth 5685

540 film.person_or_entity_appearing_in_film.film 5686

115 film.person_or_entity_appearing_in_film.film 5687

1633 film.person_or_entity_appearing_in_film.film 5688

712 film.person_or_entity_appearing_in_film.film 5689

623 film.actor.film 5690

3827 film.actor.film 5691

3020 film.actor.film 5692

2043 music.artist.origin 4232

2050 film.person_or_entity_appearing_in_film.film 5693

1032 film.actor.film 4029

912 film.actor.film 5694

3721 people.person.place_of_birth 4183

1193 film.person_or_entity_appearing_in_film.film 4821

593 music.artist.origin 4391

2080 film.person_or_entity_appearing_in_film.film 5695

1352 film.actor.film 4177

948 film.person_or_entity_appearing_in_film.film 5696

809 people.person.place_of_birth 4232

1764 film.actor.film 5042

2913	film.actor.film	3978

2914	film.person_or_entity_appearing_in_film.film	5065

967 music.artist.album	5697

1031	music.artist.origin	5698

2437	film.person_or_entity_appearing_in_film.film	4974

2743	film.person_or_entity_appearing_in_film.film	5699

351 music.artist.origin	4852

3821	film.person_or_entity_appearing_in_film.film	5700

2122	film.actor.film	4976

2819	film.person_or_entity_appearing_in_film.film	5701

2936	people.person.place_of_birth	5702

2460	film.person_or_entity_appearing_in_film.film	4730

2412	award.competitor.award_nominations	5703

2877	film.actor.film	5493

2539	music.artist.origin	4454

3692	music.artist.origin	3859

2241	film.distributor.film	5704

2241	film.distributor.film	5705

2241	film.distributor.film	4645

2241	film.distributor.film	5706

2241	film.production_company.film	5707

2241	film.production_company.film	5708

795 film.person_or_entity_appearing_in_film.film	3931

520 film.person_or_entity_appearing_in_film.film	5709

681 film.actor.film	5710

777 film.person_or_entity_appearing_in_film.film	5711

777 film.person_or_entity_appearing_in_film.film	5712

514 music.artist.origin	4044

261 music.producer.tracks_produced	5713

429 music.artist.origin	4150

2275 film.person_or_entity_appearing_in_film.film 5714

865 film.person_or_entity_appearing_in_film.film 5715

2741 people.deceased_person.place_of_death 3992

2015 film.person_or_entity_appearing_in_film.film 5716

4 music.artist.origin 4195

1059 film.person_or_entity_appearing_in_film.film 4473

1278 award.winner.awards_won 5717

2184 film.person_or_entity_appearing_in_film.film 5718

3670 film.person_or_entity_appearing_in_film.film 5719

2387 film.person_or_entity_appearing_in_film.film 5720

3433 film.actor.film 3998

794 film.actor.film 4493

1427 music.artist.origin 4454

3026 film.actor.film 5721

3390 film.actor.film 5722

3390 film.person_or_entity_appearing_in_film.film 5723

3648 film.person_or_entity_appearing_in_film.film 5724

923 film.actor.film 5725

3132 film.actor.film 4073

68 film.actor.film 5004

1662 music.musician.instruments_played 4349

2635 people.person.place_of_birth 5726

1633 film.person_or_entity_appearing_in_film.film 5727

1179 film.film.genre 4062

2141 film.actor.film 5728

435 film.actor.film 4363

1833 music.producer.tracks_produced 5729

2963 film.person_or_entity_appearing_in_film.film 4823

67 film.person_or_entity_appearing_in_film.film 5409

774 film.person_or_entity_appearing_in_film.film 4538

2125 film.actor.film 5730

1473 film.actor.film 5731

235 event.agent.performance 5732

1960 film.person_or_entity_appearing_in_film.film 4600

84 film.person_or_entity_appearing_in_film.film 5733

2742 music.artist.album 5734

3079 film.person_or_entity_appearing_in_film.film 4048

3498 film.actor.film 5735

77 film.person_or_entity_appearing_in_film.film 5736

646 film.person_or_entity_appearing_in_film.film 5737

730 music.artist.origin 5639

437 film.person_or_entity_appearing_in_film.film 4772

3162 music.artist.origin 4332

2597 film.actor.film 4874

2993 music.artist.album 5738

3179 film.person_or_entity_appearing_in_film.film 5739

2460 film.person_or_entity_appearing_in_film.film 5740

602 music.artist.origin 4148

484 film.actor.film 4840

484 film.person_or_entity_appearing_in_film.film 3856

436 film.actor.film 5741

625 film.actor.film 5742

2557 music.musician.instruments_played 3851

668 film.person_or_entity_appearing_in_film.film 5743

2241 film.distributor.film 5598

2241 film.production_company.film 5744

2241 film.production_company.film 5745

795 music.artist.album 5746

2487 film.person_or_entity_appearing_in_film.film 5747

1819 film.actor.film 4330

537 tv.program.genre 4472

2563 music.artist.origin 4050

1562 music.artist.origin 4362

1957 film.person_or_entity_appearing_in_film.film 4240

3546 film.person_or_entity_appearing_in_film.film 5748

3321 film.person_or_entity_appearing_in_film.film 3975

1241 film.film.director4153

22 music.artist.origin 3864

2309 film.actor.film 5749

2211 film.person_or_entity_appearing_in_film.film 4100

2312 film.person_or_entity_appearing_in_film.film 5750

2343 film.person_or_entity_appearing_in_film.film 5751

1962 film.actor.film 5752

3473 film.person_or_entity_appearing_in_film.film 5753

841 music.artist.origin 4057

1662 film.person_or_entity_appearing_in_film.film 5310

2808 film.actor.film 5754

1157 film.actor.film 5755

379 people.person.place_of_birth 5756

3653 people.person.place_of_birth 4013

1617 music.artist.origin 3986

328 music.artist.origin 4454

2079 film.actor.film 5527

2359 film.actor.film 5757

793 film.person_or_entity_appearing_in_film.film 5758

2337 film.actor.film 5759

2337 film.person_or_entity_appearing_in_film.film 5760

3040 film.person_or_entity_appearing_in_film.film 5761

3757 film.person_or_entity_appearing_in_film.film 5762

3426 film.person_or_entity_appearing_in_film.film 4385

893 film.actor.film 5763

2679 film.actor.film 5764

2713 film.actor.film 5765

508 film.actor.film 5766

1095 music.artist.origin 4148

3211 film.person_or_entity_appearing_in_film.film 5767

2636 film.actor.film 5768

2597 film.person_or_entity_appearing_in_film.film 5325

359 people.person.place_of_birth 3846

781 event.agent.performance 4035

399 film.person_or_entity_appearing_in_film.film 5769

2460 film.person_or_entity_appearing_in_film.film 5770

3349 film.person_or_entity_appearing_in_film.film 5771

602 film.person_or_entity_appearing_in_film.film 5175

3738 cvg.publisher.games_published 5772

324 music.artist.origin 4013

1356 film.actor.film 5642

949 music.artist.origin 5773

2159 film.person_or_entity_appearing_in_film.film 5774

2867 film.actor.film 5775

2241 film.distributor.film 5776

2241 film.production_company.film 5777

2241 film.production_company.film 5778

2241 film.production_company.film 5779

2487 film.actor.film 5506

2311 music.artist.origin 4195

2189 film.person_or_entity_appearing_in_film.film 5780

387 film.actor.film 5479

387 film.person_or_entity_appearing_in_film.film 4073

3031 film.actor.film 5781

3607 film.person_or_entity_appearing_in_film.film 5782

3487 music.musician.instruments_played 3851

114 film.person_or_entity_appearing_in_film.film 5783

989 film.person_or_entity_appearing_in_film.film 5784

3813 music.artist.origin 5238

2387 film.person_or_entity_appearing_in_film.film 5785

951 type.object.key 951

1337 music.artist.origin 4232

81 film.person_or_entity_appearing_in_film.film 4870

2132 music.artist.origin 4057

2343 music.musician.instruments_played 3851

1259 film.actor.film 5786

706 music.artist.origin 4294

2761 music.artist.origin 4133

129 film.actor.film 5787

383 film.actor.film 5788

1221 people.person.place_of_birth 4008

3384 film.person_or_entity_appearing_in_film.film 5789

115 film.actor.film 5790

2668 film.actor.film 5791

623 film.person_or_entity_appearing_in_film.film 5792

2336 music.artist.origin 5793

815 film.actor.film 5794

1635 music.artist.origin 4852

1153 film.person_or_entity_appearing_in_film.film 5795

2786 film.person_or_entity_appearing_in_film.film 5274

67 film.actor.film 5182

2209 people.person.place_of_birth 4013

1960 film.person_or_entity_appearing_in_film.film 5242

2450 music.artist.album 5796

239 film.person_or_entity_appearing_in_film.film 5797

2080 film.person_or_entity_appearing_in_film.film 5798

3598 film.actor.film 5799

3598 film.actor.film 5483

3795 film.person_or_entity_appearing_in_film.film 5800

3093 people.person.place_of_birth 4191

3121 film.actor.film 5801

2214 music.musician.instruments_played 5802

1704 film.actor.film 5803

2913 film.actor.film 5804

2913 film.actor.film 5805

896 people.person.place_of_birth 3846

1208 film.actor.film 5806

70 film.person_or_entity_appearing_in_film.film 4600

1788 people.person.place_of_birth 5807

2597 film.actor.film 5808

1370 film.film.genre 5809

445 film.person_or_entity_appearing_in_film.film 5498

1707 film.person_or_entity_appearing_in_film.film 5810

1602 music.album.genre 5811

1277 film.person_or_entity_appearing_in_film.film 5812

625 film.person_or_entity_appearing_in_film.film 5813

1829 people.person.place_of_birth 5814

1066 music.artist.origin 4087

1333 film.actor.film 5815

779 film.actor.film 5816

1463 award.nominee.award_nominations 4565

2241 film.distributor.film 5817

2241 film.production_company.film 5818

2241 film.production_company.film 5819

2241	film.production_company.film	5820

1917	film.actor.film	4843

1614	film.actor.film	5821

681 film.person_or_entity_appearing_in_film.film	4250

1624	film.person_or_entity_appearing_in_film.film	5822

777 film.person_or_entity_appearing_in_film.film	5823

2487	film.actor.film	5824

591 film.actor.film	5825

1148	music.artist.origin	3860

2880	people.person.place_of_birth	3947

121 music.artist.origin	5826

2741	film.actor.film	5827

904 music.artist.origin	4013

1536	film.actor.film	4132

1340	people.person.place_of_birth	5828

3509	music.artist.track	5829

3052	film.person_or_entity_appearing_in_film.film	5830

3184	film.actor.film	5831

2615	people.deceased_person.place_of_death	4057

3390	film.person_or_entity_appearing_in_film.film	5832

166 music.musician.instruments_played	4430

1816	film.actor.film	4359

638 film.person_or_entity_appearing_in_film.film	5833

2774	music.artist.origin	3942

2904	film.actor.film	5834

2402	film.person_or_entity_appearing_in_film.film	5835

1751	people.deceased_person.place_of_death	4496

2633	people.person.place_of_birth	4272

2494	film.actor.film	5836

2670	film.person_or_entity_appearing_in_film.film	5837

1989 film.actor.film 5447
3827 film.person_or_entity_appearing_in_film.film 5838
3266 music.artist.origin 4616
1153 film.actor.film 5839
605 music.artist.origin 4057
2708 film.person_or_entity_appearing_in_film.film 5407
1525 film.person_or_entity_appearing_in_film.film 5840
2804 film.person_or_entity_appearing_in_film.film 5841
3133 music.artist.origin 3846
12 film.actor.film 5842
2750 film.film.genre 4062
3137 music.artist.origin 5161
136 film.actor.film 5843
1960 film.person_or_entity_appearing_in_film.film 5068
1187 film.actor.film 5844
2949 music.artist.origin 5845
2717 music.artist.origin 4057
192 music.producer.tracks_produced 5846
539 film.person_or_entity_appearing_in_film.film 3974
3228 film.actor.film 5847
46 music.artist.origin 5265
1453 film.actor.film 5848
1846 film.actor.film 5849
437 film.actor.film 5850
128 film.actor.film 5851
1559 film.actor.film 4494
2636 film.person_or_entity_appearing_in_film.film 5852
3179 film.person_or_entity_appearing_in_film.film 4826
366 film.actor.film 5853
3177 music.musician.instruments_played 5802

484 film.actor.film 5854

484 film.person_or_entity_appearing_in_film.film 5855

2208 people.person.place_of_birth 3866

3738 games.publisher.games_published 5856

3452 film.actor.film 4190

901 film.actor.film 5857

625 film.actor.film 5858

442 music.artist.album 5859

2241 film.distributor.film 5860

2241 film.production_company.film 5861

2241 film.production_company.film 5862

2241 film.production_company.film 5863

2241 film.production_company.film 4968

2241 film.production_company.film 5864

2487 film.actor.film 5865

1022 film.actor.film 4385

875 music.artist.origin 4171

2861 film.actor.film 5866

673 film.person_or_entity_appearing_in_film.film 5867

2207 film.person_or_entity_appearing_in_film.film 4846

152 film.actor.film 3873

1105 film.actor.film 5648

1273 music.artist.origin 5061

3026 film.actor.film 5868

1786 film.person_or_entity_appearing_in_film.film 5869

2770 film.actor.film 5870

2711 film.actor.film 5871

961 music.artist.origin 5872

2795 music.artist.origin 4174

969 music.artist.origin 5873

671 film.actor.film 5874

1157 film.actor.film 5875

3384 film.person_or_entity_appearing_in_film.film 5876

115 film.person_or_entity_appearing_in_film.film 4751

598 film.film.star 5877

1720 film.writer.film 5878

155 film.actor.film 5879

3827 film.person_or_entity_appearing_in_film.film 5880

3788 film.actor.film 5881

1881 film.person_or_entity_appearing_in_film.film 5882

2359 people.deceased_person.place_of_death 4057

3668 film.person_or_entity_appearing_in_film.film 5883

2723 music.musician.instruments_played 4349

1692 film.person_or_entity_appearing_in_film.film 5884

620 people.person.place_of_birth 3971

920 music.artist.origin 4057

279 film.person_or_entity_appearing_in_film.film 5885

645 film.person_or_entity_appearing_in_film.film 5886

893 film.actor.film 5887

3586 music.artist.origin 4380

458 film.person_or_entity_appearing_in_film.film 5888

1465 film.actor.film 5889

2562 film.person_or_entity_appearing_in_film.film 5890

974 film.actor.film 5891

1204 film.actor.film 5892

2636 film.person_or_entity_appearing_in_film.film 5422

2688 film.actor.film 4411

3009 film.actor.film 5893

3177 music.musician.instruments_played 4349

79 film.person_or_entity_appearing_in_film.film 5894

61 people.person.place_of_birth 5064

1864 film.actor.film 5895

3738 games.publisher.games_published 5896

3738 games.publisher.games_published 5897

3738 games.publisher.games_published 5898

436 film.person_or_entity_appearing_in_film.film 5899

2867 film.actor.film 4684

242 music.artist.origin 5900

2360 film.person_or_entity_appearing_in_film.film 5901

2241 film.distributor.film 5902

2241 film.distributor.film 5903

2241 film.production_company.film 5904

2516 music.artist.origin 4362

1107 music.artist.origin 4217

1656 film.person_or_entity_appearing_in_film.film 5551

387 film.person_or_entity_appearing_in_film.film 5905

3486 film.actor.film 5906

1806 film.person_or_entity_appearing_in_film.film 5907

2707 film.actor.film 5908

2741 film.actor.film 4582

629 film.writer.film 5909

3321 film.actor.film 4748

3321 film.person_or_entity_appearing_in_film.film 4586

883 music.artist.origin 5910

2148 film.actor.film 5911

2519 film.actor.film 5912

1421 music.artist.origin 4714

2402 film.actor.film 5913

202 people.person.place_of_birth 4133

3613 film.person_or_entity_appearing_in_film.film 5200

3460 film.person_or_entity_appearing_in_film.film 5042

379 film.actor.film 5593

1633 film.actor.film 5914

1044 award.nominee.award_nominations 4708

3496 film.film.language 4076

1521 music.artist.origin 4087

3778 music.musician.instruments_played 3851

2865 music.artist.origin 4232

2733 film.actor.film 5915

3391 music.artist.origin 5916

747 film.actor.film 5917

584 film.actor.film 5918

2913 film.person_or_entity_appearing_in_film.film 5919

197 music.artist.origin 5265

458 film.actor.film 4553

710 music.artist.origin 4800

199 music.artist.origin 5920

1204 film.actor.film 5921

2095 film.actor.film 5922

21 music.artist.origin 5213

2636 film.actor.film 5072

49 film.person_or_entity_appearing_in_film.film 5923

524 music.artist.origin 5924

1981 people.person.place_of_birth 3946

3738 cvg.publisher.games_published 5925

3738 games.publisher.games_published 5926

3738 games.publisher.games_published 5927

3738 games.publisher.games_published 5076

2430 film.person_or_entity_appearing_in_film.film 5928

901 film.person_or_entity_appearing_in_film.film 5929

1249 film.actor.film 5930

3161 music.artist.origin 4057

2241 film.distributor.film 4687

2241 film.production_company.film 5931

2241 film.production_company.film 5932

2241 film.production_company.film 5933

1710 music.artist.origin 5934

430 film.actor.film 5935

135 film.actor.film 5936

33 award.competitor.awards_won 5937

777 film.person_or_entity_appearing_in_film.film 5938

2487 film.person_or_entity_appearing_in_film.film 5939

2487 film.person_or_entity_appearing_in_film.film 5257

2951 film.actor.film 3991

719 people.person.place_of_birth 4587

194 music.musician.instruments_played 4349

1475 film.actor.film 4652

1059 film.person_or_entity_appearing_in_film.film 5940

1430 people.person.place_of_birth 3945

1135 music.artist.album 5941

3184 film.person_or_entity_appearing_in_film.film 5831

1606 music.artist.origin 3864

3390 film.actor.film 5942

1323 film.actor.film 5943

56 film.person_or_entity_appearing_in_film.film 5944

844 people.person.place_of_birth 5662

2076 people.person.place_of_birth 5945

379 film.person_or_entity_appearing_in_film.film 5147

3827 film.person_or_entity_appearing_in_film.film 5946

846 film.actor.film 5947

2313	film.person_or_entity_appearing_in_film.film	4918

1692	film.actor.film	5948

2389	film.director.film	5949

539	film.actor.film	5950

2085	people.person.place_of_birth	4496

3598	film.actor.film	5951

3598	film.person_or_entity_appearing_in_film.film	5951

3030	film.actor.film	5952

64	film.actor.film	5953

2913	film.person_or_entity_appearing_in_film.film	5804

2095	film.actor.film	5365

95	music.artist.origin	5845

1944	people.deceased_person.place_of_death	4357

2122	film.person_or_entity_appearing_in_film.film	5447

1707	film.actor.film	5810

715	film.person_or_entity_appearing_in_film.film	4720

660	film.person_or_entity_appearing_in_film.film	5052

3738	cvg.publisher.games_published	5954

3738	games.publisher.games_published	3857

184	film.person_or_entity_appearing_in_film.film	5955

436	film.actor.film	5956

901	film.actor.film	5957

625	film.person_or_entity_appearing_in_film.film	5958

1248	film.actor.film	5959

779	event.agent.performance	4035

2867	film.person_or_entity_appearing_in_film.film	5960

3161	film.actor.film	5961

2241	film.distributor.film	5861

2241	film.distributor.film	5962

2241	film.distributor.film	5963

2241 film.distributor.film 5964

2241 film.production_company.film 5083

2241 film.production_company.film 5965

1936 film.actor.film 5966

1936 film.person_or_entity_appearing_in_film.film 5967

1464 film.actor.film 5968

681 film.person_or_entity_appearing_in_film.film 5969

777 film.actor.film 5970

1014 music.musician.instruments_played 3851

1741 music.artist.origin 4211

643 film.person_or_entity_appearing_in_film.film 4097

2677 film.actor.film 5971

3526 film.actor.film 5972

434 film.actor.film 5190

538 film.person_or_entity_appearing_in_film.film 5973

1378 film.person_or_entity_appearing_in_film.film 5974

2686 film.person_or_entity_appearing_in_film.film 5975

2979 film.actor.film 5976

3290 music.artist.origin 5977

1328 music.artist.origin 3916

68 film.actor.film 3880

3599 film.actor.film 5683

2635 film.actor.film 5978

2161 film.actor.film 3957

1720 film.writer.film 5446

3411 music.artist.origin 4121

1748 film.actor.film 5979

3616 music.artist.origin 4471

3263 location.location.contained_by 5980

67 film.actor.film 4219

2712	film.person_or_entity_appearing_in_film.film	5155

2147	film.person_or_entity_appearing_in_film.film	5981

1473	film.person_or_entity_appearing_in_film.film	5982

463 music.artist.origin	5001

1960	film.person_or_entity_appearing_in_film.film	5983

3585	film.person_or_entity_appearing_in_film.film	5984

127 film.actor.film	5985

3725	film.person_or_entity_appearing_in_film.film	4802

192 music.artist.track 4440

645 film.actor.film	5986

2080	film.person_or_entity_appearing_in_film.film	5987

3498	film.actor.film	5988

361 award.competitor.award_nominations	5989

1928	music.artist.origin	4183

2892	film.person_or_entity_appearing_in_film.film	5990

308 music.artist.album	4259

2636	film.actor.film	5991

2636	film.person_or_entity_appearing_in_film.film	5992

373 film.actor.film	5993

484 film.actor.film	5994

484 music.musician.instruments_played	3959

184 film.person_or_entity_appearing_in_film.film	5918

1213	music.artist.origin	4183

901 film.person_or_entity_appearing_in_film.film	5995

1513	music.artist.album	5996

2915	people.person.place_of_birth	3972

3400	film.person_or_entity_appearing_in_film.film	5997

2241	film.distributor.film	5998

2241	film.distributor.film	5999

2241	film.distributor.film	6000

2241 film.production_company.film 6001

2241 film.production_company.film 6002

2241 film.production_company.film 6003

516 music.artist.album 6004

1624 film.actor.film 4802

1085 music.artist.origin 4502

816 film.person_or_entity_appearing_in_film.film 5801

643 film.person_or_entity_appearing_in_film.film 6005

788 film.actor.film 6006

3546 film.actor.film 6007

3546 film.person_or_entity_appearing_in_film.film 6008

2100 award.nominee.award_nominations 6009

1357 film.actor.film 6010

253 film.actor.film 6011

3538 music.artist.origin 3864

1560 music.artist.origin 6012

1623 film.person_or_entity_appearing_in_film.film 6013

3390 film.person_or_entity_appearing_in_film.film 6014

3648 film.actor.film 6015

1816 film.person_or_entity_appearing_in_film.film 6016

2658 film.person_or_entity_appearing_in_film.film 6017

3132 film.actor.film 6018

2449 film.actor.film 6006

3473 people.person.place_of_birth 4919

2252 people.person.place_of_birth 4707

3479 people.person.place_of_birth 6019

288 music.artist.origin 4008

2845 music.artist.origin 4094

2550 film.person_or_entity_appearing_in_film.film 5882

3602 music.artist.origin 5061

3384 film.actor.film 6020

115 film.person_or_entity_appearing_in_film.film 5790

598 film.film.genre 4410

3592 film.actor.film 6021

251 music.artist.origin 3849

3020 film.person_or_entity_appearing_in_film.film 5692

2186 music.artist.origin 4852

3603 film.person_or_entity_appearing_in_film.film 6022

3471 music.artist.origin 5213

1473 film.person_or_entity_appearing_in_film.film 5596

2337 film.person_or_entity_appearing_in_film.film 6023

2069 film.person_or_entity_appearing_in_film.film 6024

1079 film.cinematographer.film4669

3498 film.person_or_entity_appearing_in_film.film 6025

2562 film.actor.film 6026

70 film.actor.film 6027

1102 music.artist.origin 4852

2636 film.actor.film 5143

2650 film.film.actor 6028

3663 film.actor.film 4407

3179 film.actor.film 6029

3738 cvg.publisher.games_published 5373

2414 award.nominee.award_nominations 6030

1248 film.actor.film 4328

2241 film.distributor.film 5762

2241 film.distributor.film 4031

2241 film.distributor.film 6031

2241 film.production_company.film 6032

2241 film.production_company.film 6033

451 award.competitor.award_nominations 6034

1618 music.artist.album 6035

1240 music.artist.origin 6036

591 film.person_or_entity_appearing_in_film.film 6037

894 film.actor.film 5341

387 film.person_or_entity_appearing_in_film.film 6038

3546 film.person_or_entity_appearing_in_film.film 6007

538 film.actor.film 6039

3231 film.actor.film 6040

1140 music.artist.origin 3864

3752 film.actor.film 6041

2711 film.person_or_entity_appearing_in_film.film 6042

3048 film.actor.film 6043

1962 film.person_or_entity_appearing_in_film.film 6044

56 film.actor.film 5944

3702 people.person.place_of_birth 4077

1750 film.actor.film 6045

2022 music.artist.origin 4148

3822 film.actor.film 6046

3599 film.person_or_entity_appearing_in_film.film 6047

567 film.actor.film 6048

2635 film.person_or_entity_appearing_in_film.film 6049

3716 film.actor.film 6050

3114 people.person.place_of_birth 4214

3307 film.actor.film 6051

131 film.person_or_entity_appearing_in_film.film 6052

2177 music.album.genre 5811

2964 music.musician.instruments_played 4349

1006 award.competitor.award_nominations 6053

1633 film.actor.film 6054

623 film.person_or_entity_appearing_in_film.film 6055

1431 people.person.place_of_birth 6056

2141 film.actor.film 6057

3699 music.artist.origin 3864

1476 music.artist.album 5734

531 people.person.place_of_birth 6058

1449 music.artist.origin 4454

2990 film.person_or_entity_appearing_in_film.film 6059

3137 music.artist.album 6060

2983 film.person_or_entity_appearing_in_film.film 6061

3040 film.person_or_entity_appearing_in_film.film 6062

3598 film.person_or_entity_appearing_in_film.film 6063

386 film.person_or_entity_appearing_in_film.film 6064

2848 film.film.star 6065

1601 film.actor.film 6066

1904 music.artist.origin 4454

2636 film.actor.film 3917

3485 music.artist.origin 6067

1579 film.actor.film 5587

3738 cvg.publisher.games_published 6068

3738 games.publisher.games_published 5426

436 film.actor.film 5313

436 film.actor.film 4461

1154 film.actor.film 6069

3163 people.person.place_of_birth 4232

901 film.person_or_entity_appearing_in_film.film 6070

2706 people.person.place_of_birth 4347

3220 people.person.place_of_birth 3966

3248 music.artist.album 4146

2867 film.actor.film 4088

1463 award.winner.awards_won 6071

2241	film.distributor.film	5224

2241	film.distributor.film	4249

681 film.actor.film	4738

33	award.winner.awards_won	5937

894 film.person_or_entity_appearing_in_film.film	6072

572 event.agent.performance	4898

3486	film.actor.film	4845

1234	music.artist.origin	4391

3331	film.actor.film	6073

3002	film.actor.film	6074

3002	film.actor.film	6075

1536	people.person.place_of_birth	4204

983 music.musician.instruments_played	3959

2872	people.person.place_of_birth	6076

3043	music.artist.origin	4391

2368	music.artist.origin	6077

2556	film.person_or_entity_appearing_in_film.film	4323

2211	people.person.profession	4221

1332	film.actor.film	6078

2546	people.person.place_of_birth	4496

417 music.artist.origin	5421

1816	film.actor.film	6016

2010	film.actor.film	6079

306 music.artist.album	4104

3422	music.artist.origin	4174

56	film.actor.film	6080

206 film.person_or_entity_appearing_in_film.film	6081

3345	music.artist.origin	3961

735 film.actor.film	5532

2635	film.person_or_entity_appearing_in_film.film	6082

1584 people.deceased_person.place_of_death 4711

598 film.film.director6083

1720 film.person_or_entity_appearing_in_film.film 6084

326 music.artist.origin 4454

3610 music.artist.origin 3947

879 film.person_or_entity_appearing_in_film.film 6085

2146 people.deceased_person.place_of_death 3942

2422 film.person_or_entity_appearing_in_film.film 6086

912 film.person_or_entity_appearing_in_film.film 6087

2804 film.person_or_entity_appearing_in_film.film 6088

2079 film.person_or_entity_appearing_in_film.film 5764

3668 film.actor.film 5883

1805 film.actor.film 6062

2147 film.person_or_entity_appearing_in_film.film 6089

2125 film.person_or_entity_appearing_in_film.film 6090

1473 film.actor.film 6091

1473 film.actor.film 4250

1473 film.person_or_entity_appearing_in_film.film 6092

944 music.artist.origin 4008

2389 film.person_or_entity_appearing_in_film.film 6093

2998 film.actor.film 6094

1326 music.artist.origin 4046

1706 film.actor.film 5803

1685 music.artist.origin 6095

377 award.winner.awards_won 4329

3295 film.actor.film 6096

2080 film.actor.film 6097

3598 film.person_or_entity_appearing_in_film.film 6098

1973 film.person_or_entity_appearing_in_film.film 6099

893 film.person_or_entity_appearing_in_film.film 6100

2726	film.actor.film	4551

2913	film.actor.film	6101

2026	music.artist.origin	4008

1425	music.artist.origin	4362

827 music.artist.origin	6067

437 film.actor.film	6102

3821	film.actor.film	6103

2070	film.person_or_entity_appearing_in_film.film	6104

1353	film.actor.film	4692

3738	cvg.publisher.games_published	6105

3738	games.publisher.games_published	6068

1821	film.actor.film	6106

1416	film.person_or_entity_appearing_in_film.film	3905

2082	music.artist.origin	4053

1040	type.object.subject_key	1040

2867	film.person_or_entity_appearing_in_film.film	6107

2241	film.production_company.film	6108

1114	music.artist.origin	4183

2977	people.person.place_of_birth	3945

644 film.person_or_entity_appearing_in_film.film	6109

2126	film.person_or_entity_appearing_in_film.film	6110

441 music.artist.origin	6111

3772	film.film.writer	6112

3321	film.actor.film	6113

3631	music.musician.instruments_played	6114

2212	music.artist.origin	4217

1558	film.person_or_entity_appearing_in_film.film	4524

2987	music.artist.origin	4380

1385	music.artist.origin	4046

1705	film.person_or_entity_appearing_in_film.film	4846

3763 film.person_or_entity_appearing_in_film.film 6115

1750 film.person_or_entity_appearing_in_film.film 6116

997 film.actor.film 6117

3599 film.person_or_entity_appearing_in_film.film 6118

567 film.person_or_entity_appearing_in_film.film 6119

2635 film.actor.film 6120

1157 film.person_or_entity_appearing_in_film.film 6121

1157 film.person_or_entity_appearing_in_film.film 6122

2416 people.deceased_person.place_of_death 4057

1489 music.artist.origin 4391

1439 film.person_or_entity_appearing_in_film.film 4752

2724 people.person.place_of_birth 4357

1153 film.actor.film 6123

1153 film.actor.film 6124

1920 music.artist.origin 4852

3020 film.person_or_entity_appearing_in_film.film 6125

3494 music.artist.album 6126

67 film.person_or_entity_appearing_in_film.film 5378

1923 music.artist.origin 5139

1473 film.person_or_entity_appearing_in_film.film 6127

62 music.artist.origin 6128

2737 film.person_or_entity_appearing_in_film.film 6129

645 film.actor.film 6130

2080 film.person_or_entity_appearing_in_film.film 6131

3598 film.actor.film 6132

3598 film.person_or_entity_appearing_in_film.film 6133

3030 people.person.place_of_birth 4057

893 film.person_or_entity_appearing_in_film.film 4500

672 film.actor.film 6134

55 event.agent.performance 4035

2914 film.actor.film 6135

628 film.person_or_entity_appearing_in_film.film 6136

787 people.person.place_of_birth 6137

1796 film.person_or_entity_appearing_in_film.film 6138

852 film.actor.film 6139

1601 film.actor.film 3847

2636 film.actor.film 6140

2650 film.film.star 6028

2460 film.person_or_entity_appearing_in_film.film 6141

2412 award.competitor.award_nominations 6142

2661 film.actor.film 6143

484 film.person_or_entity_appearing_in_film.film 6144

1154 film.person_or_entity_appearing_in_film.film 6145

901 film.actor.film 6146

2138 music.artist.origin 5246

119 music.producer.tracks_produced 3927

1237 music.artist.origin 5181

2241 film.distributor.film 6001

2241 film.production_company.film 6147

2779 music.artist.album 6148

1917 film.actor.film 6149

1361 film.actor.film 5642

2738 people.person.place_of_birth 4471

1877 film.actor.film 4793

2677 film.person_or_entity_appearing_in_film.film 5971

1806 film.person_or_entity_appearing_in_film.film 4025

2100 film.person_or_entity_appearing_in_film.film 5343

3147 music.artist.origin 5161

696 film.person_or_entity_appearing_in_film.film 5479

101 people.person.place_of_birth 4442

1765 film.actor.film 6150

475 music.artist.origin 4093

1623 film.actor.film 6151

2051 people.person.profession 4221

385 film.person_or_entity_appearing_in_film.film 6152

2715 people.person.place_of_birth 6153

3390 film.person_or_entity_appearing_in_film.film 5942

2711 film.person_or_entity_appearing_in_film.film 4267

2919 film.actor.film 6154

916 people.person.place_of_birth 4357

3508 film.person_or_entity_appearing_in_film.film 6155

601 film.person_or_entity_appearing_in_film.film 5694

56 film.actor.film 6156

206 people.person.place_of_birth 4057

2402 film.person_or_entity_appearing_in_film.film 5913

2820 film.person_or_entity_appearing_in_film.film 6157

981 film.person_or_entity_appearing_in_film.film 4234

981 people.person.place_of_birth 5100

1157 film.person_or_entity_appearing_in_film.film 6158

3384 film.actor.film 6159

254 music.artist.origin 3963

3569 film.actor.film 6160

1924 music.artist.origin 4453

674 film.actor.film 6161

3792 film.person_or_entity_appearing_in_film.film 6162

2619 film.person_or_entity_appearing_in_film.film 6163

3364 film.actor.film 4966

1959 film.person_or_entity_appearing_in_film.film 6164

2998 film.person_or_entity_appearing_in_film.film 6165

2983 film.actor.film 5576

2069	film.person_or_entity_appearing_in_film.film	6166

2737	film.actor.film	6167

1973	music.artist.album	6168

3513	music.artist.origin	4024

3447	film.person_or_entity_appearing_in_film.film	5786

471 people.deceased_person.place_of_death	4191

1079	film.director.film	6169

334 music.artist.origin	4454

635 people.person.place_of_birth	3942

2913	film.person_or_entity_appearing_in_film.film	6170

510 event.agent.performance	4603

128 film.actor.film	4388

2636	film.actor.film	6171

2636	film.actor.film	6172

2636	film.person_or_entity_appearing_in_film.film	6173

2597	film.person_or_entity_appearing_in_film.film	6174

2070	film.actor.film	6175

3047	music.musician.instruments_played	3959

1211	film.person_or_entity_appearing_in_film.film	6176

3166	film.actor.film	6177

3738	cvg.publisher.games_published	6178

3738	games.publisher.games_published	6179

184 film.actor.film	4834

901 film.person_or_entity_appearing_in_film.film	6180

3462	film.person_or_entity_appearing_in_film.film	5010

2241	film.distributor.film	6181

3003	film.actor.film	4168

451 award.competitor.awards_won	6182

684 music.artist.album	6183

777 film.person_or_entity_appearing_in_film.film	6184

1317 music.artist.origin 6185

1360 film.actor.film 6186

1567 music.musician.instruments_played 5619

643 film.person_or_entity_appearing_in_film.film 6187

3085 people.person.place_of_birth 4272

2731 film.person_or_entity_appearing_in_film.film 4966

3779 film.person_or_entity_appearing_in_film.film 6022

1772 film.actor.film 6188

3547 tv.program.genre 6189

3648 film.person_or_entity_appearing_in_film.film 6015

2962 people.person.place_of_birth 6190

2658 film.actor.film 6191

1233 film.actor.film 6192

637 film.person_or_entity_appearing_in_film.film 6193

3508 film.person_or_entity_appearing_in_film.film 4846

3544 film.actor.film 6194

1260 people.person.place_of_birth 6195

454 music.artist.origin 3946

2306 people.deceased_person.place_of_death 4496

1962 film.person_or_entity_appearing_in_film.film 4802

2971 people.person.place_of_birth 5226

959 music.artist.album 6196

2904 film.person_or_entity_appearing_in_film.film 6197

1157 film.person_or_entity_appearing_in_film.film 6198

3635 music.artist.origin 4108

2618 music.artist.origin 4059

2494 film.actor.film 6199

626 music.artist.origin 6200

1720 people.person.place_of_birth 6201

3827 film.actor.film 6202

492 film.actor.film 6203

1790 music.artist.origin 5639

1153 film.person_or_entity_appearing_in_film.film 6204

3317 film.person_or_entity_appearing_in_film.film 6205

116 people.person.place_of_birth 3926

1128 music.artist.origin 3946

2708 film.person_or_entity_appearing_in_film.film 6206

67 film.person_or_entity_appearing_in_film.film 6207

67 film.person_or_entity_appearing_in_film.film 6208

1155 film.person_or_entity_appearing_in_film.film 4613

2619 people.person.place_of_birth 6209

2337 film.person_or_entity_appearing_in_film.film 6210

850 music.artist.album 6211

2175 music.artist.origin 4376

1151 music.album.genre 6212

74 film.actor.film 5114

80 music.producer.tracks_produced 4386

1661 music.artist.origin 4133

967 award.competitor.award_nominations 6213

70 film.actor.film 6214

2580 people.person.place_of_birth 6215

128 film.person_or_entity_appearing_in_film.film 6216

2481 music.artist.origin 4050

2674 film.actor.film 6217

519 film.person_or_entity_appearing_in_film.film 4328

49 film.director.film 6218

1353 film.actor.film 6219

2460 film.person_or_entity_appearing_in_film.film 6220

1707 music.artist.origin 4362

535 music.artist.origin 3864

2778 film.person_or_entity_appearing_in_film.film 6221

484 film.person_or_entity_appearing_in_film.film 6222

3738 games.publisher.games_published 6223

1154 film.actor.film 6224

933 music.artist.origin 3864

625 film.person_or_entity_appearing_in_film.film 6225

2138 film.actor.film 6226

2241 film.distributor.film 4404

2241 film.distributor.film 6227

2241 film.distributor.film 4194

2241 film.distributor.film 4090

2241 film.distributor.film 6228

2241 film.production_company.film 6229

2241 film.production_company.film 6230

682 film.person_or_entity_appearing_in_film.film 6231

1762 film.actor.film 6232

33 people.person.place_of_birth 4977

2487 film.person_or_entity_appearing_in_film.film 6233

3427 music.artist.origin 4369

622 film.person_or_entity_appearing_in_film.film 6234

2126 film.person_or_entity_appearing_in_film.film 6235

1767 film.person_or_entity_appearing_in_film.film 6236

1767 film.person_or_entity_appearing_in_film.film 6237

3669 film.actor.film 6238

3669 film.actor.film 6239

1360 film.person_or_entity_appearing_in_film.film 6240

643 film.actor.film 6241

1144 music.artist.origin 4013

2315 music.artist.origin 6242

3002 film.actor.film 6243

3002 film.actor.film 6244

3730 music.artist.origin 4232

1378 film.person_or_entity_appearing_in_film.film 6245

3321 film.actor.film 6246

3321 film.person_or_entity_appearing_in_film.film 6247

1241 film.film.art_director 6248

2399 music.artist.origin 6249

2634 film.actor.film 5307

3612 music.artist.origin 3946

81 film.actor.film 6250

3508 film.person_or_entity_appearing_in_film.film 6251

3508 film.person_or_entity_appearing_in_film.film 6087

56 event.agent.performance 4898

3473 film.person_or_entity_appearing_in_film.film 6252

422 people.person.place_of_birth 4347

3724 people.person.place_of_birth 3944

735 music.artist.album 4844

2635 film.person_or_entity_appearing_in_film.film 6253

985 film.person_or_entity_appearing_in_film.film 6254

1893 film.person_or_entity_appearing_in_film.film 6255

3384 film.person_or_entity_appearing_in_film.film 6256

1351 film.actor.film 6257

785 film.person_or_entity_appearing_in_film.film 4538

2576 music.artist.origin 4217

3713 music.artist.origin 4957

2359 film.person_or_entity_appearing_in_film.film 6258

3792 film.person_or_entity_appearing_in_film.film 3901

2619 film.actor.film 6163

2018 award.competitor.award_nominations 5491

3294 film.actor.film 5608

2338 film.person_or_entity_appearing_in_film.film 6259

40 music.artist.origin 3864

562 music.artist.origin 5662

2785 music.musician.instruments_played 6260

1469 film.person_or_entity_appearing_in_film.film 4281

646 film.person_or_entity_appearing_in_film.film 4288

910 music.artist.origin 4217

2009 people.person.place_of_birth 6261

2173 location.location.contained_by 6262

3682 music.artist.origin 6263

3561 people.person.place_of_birth 4455

2743 film.actor.film 5699

685 film.person_or_entity_appearing_in_film.film 4774

2481 film.person_or_entity_appearing_in_film.film 6264

2070 film.person_or_entity_appearing_in_film.film 6265

3179 film.person_or_entity_appearing_in_film.film 6029

2460 film.actor.film 4135

1315 film.person_or_entity_appearing_in_film.film 6266

3738 cvg.publisher.games_published 6267

184 film.person_or_entity_appearing_in_film.film 6268

2400 film.actor.film 6269

901 film.person_or_entity_appearing_in_film.film 6270

796 film.person_or_entity_appearing_in_film.film 5587

2360 people.person.place_of_birth 6271

2241 film.distributor.film 4246

2241 film.distributor.film 6272

2241 film.production_company.film 6273

2241 film.production_company.film 5964

3430 film.person_or_entity_appearing_in_film.film 6274

1464 film.actor.film 6275

2266 music.artist.origin 6276

1624 film.actor.film 6277

2189 film.actor.film 5780

1461 film.actor.film 6278

387 film.person_or_entity_appearing_in_film.film 6279

1697 people.person.place_of_birth 4044

955 people.person.place_of_birth 6280

1806 film.person_or_entity_appearing_in_film.film 6281

1806 film.person_or_entity_appearing_in_film.film 6282

3665 film.actor.film 6283

2741 film.person_or_entity_appearing_in_film.film 5827

434 film.actor.film 6284

2493 people.person.place_of_birth 4057

3374 film.person_or_entity_appearing_in_film.film 6285

1160 music.artist.origin 3849

2715 film.actor.film 6286

2711 film.person_or_entity_appearing_in_film.film 5871

1632 film.actor.film 6287

891 music.artist.origin 4701

3132 film.person_or_entity_appearing_in_film.film 6288

2938 film.person_or_entity_appearing_in_film.film 4421

567 film.person_or_entity_appearing_in_film.film 6289

2308 music.artist.origin 6290

2986 film.person_or_entity_appearing_in_film.film 5191

1719 film.actor.film 6291

2494 film.actor.film 6292

785 film.person_or_entity_appearing_in_film.film 4753

597 film.person_or_entity_appearing_in_film.film 5150

548 film.actor.film 6293

1044 award.competitor.award_nominations 6294

1998	film.actor.film	6295

3569	film.actor.film	6296

3395	film.person_or_entity_appearing_in_film.film	6297

2050	film.person_or_entity_appearing_in_film.film	6298

2422	film.person_or_entity_appearing_in_film.film	5312

912 film.actor.film	5470

2642	music.artist.origin	4380

67	film.person_or_entity_appearing_in_film.film	6299

2187	film.person_or_entity_appearing_in_film.film	6300

38	music.producer.tracks_produced	5529

2124	music.artist.origin	4502

2337	film.actor.film	6301

2337	film.actor.film	6302

2018	film.person_or_entity_appearing_in_film.film	4792

127 film.person_or_entity_appearing_in_film.film	4073

1813	film.person_or_entity_appearing_in_film.film	6303

2033	music.artist.origin	4499

2739	music.artist.origin	5265

3589	music.artist.origin	3897

46	award.nominee.award_nominations	6304

2095	film.actor.film	6305

2636	film.actor.film	5538

2070	film.person_or_entity_appearing_in_film.film	6306

2070	film.person_or_entity_appearing_in_film.film	6307

1392	people.person.place_of_birth	5019

373 film.actor.film	3880

2401	music.artist.origin	4960

229 music.artist.origin	4214

840 music.musician.instruments_played	6308

2208	film.person_or_entity_appearing_in_film.film	5821

901 film.person_or_entity_appearing_in_film.film 3987

2360 film.actor.film 6309

2241 film.distributor.film 6310

2241 film.distributor.film 4987

2241 film.distributor.film 6311

2241 film.production_company.film 6312

2241 film.production_company.film 6313

2241 film.production_company.film 6314

2241 film.production_company.film 4512

2685 music.musician.instruments_played 4349

2779 film.person_or_entity_appearing_in_film.film 6315

2897 music.artist.origin 5161

1966 music.artist.origin 3945

2126 people.person.place_of_birth 5916

261 music.artist.track 6316

2390 film.actor.film 6317

2410 people.deceased_person.place_of_death 4768

3331 film.actor.film 6318

2836 film.actor.film 6319

807 music.artist.album 5460

3579 film.person_or_entity_appearing_in_film.film 6320

3390 film.person_or_entity_appearing_in_film.film 5722

2711 film.person_or_entity_appearing_in_film.film 5264

731 music.artist.album 6321

2669 film.actor.film 6322

1785 people.person.place_of_birth 6076

2402 film.person_or_entity_appearing_in_film.film 4312

950 people.person.place_of_birth 6323

567 film.actor.film 6324

623 film.actor.film 6325

3708	music.artist.origin	4057

3514	music.artist.origin	3926

2720	music.artist.origin	4077

3548	film.person_or_entity_appearing_in_film.film	6326

2125	film.actor.film	6327

127	type.object.key	127

150	people.deceased_person.place_of_death	3997

2080	film.person_or_entity_appearing_in_film.film	6328

3498	film.actor.film	6329

3498	film.actor.film	3909

2052	people.person.place_of_birth	6067

3676	film.person_or_entity_appearing_in_film.film	3962

2622	film.person_or_entity_appearing_in_film.film	3912

1306	people.person.place_of_birth	4496

70	film.actor.film	4609

70	film.person_or_entity_appearing_in_film.film	5170

70	film.person_or_entity_appearing_in_film.film	6330

70	people.deceased_person.place_of_death	6331

2028	music.artist.origin	4077

2892	film.actor.film	6332

2070	film.actor.film	6333

790	film.actor.film	4802

3738	cvg.publisher.games_published	6334

1363	film.actor.film	6335

3791	people.person.place_of_birth	3846

1248	film.person_or_entity_appearing_in_film.film	4328

2867	film.person_or_entity_appearing_in_film.film	6336

65	people.person.place_of_birth	5569

2360	film.person_or_entity_appearing_in_film.film	6337

2241	film.distributor.film	6338

2241 film.distributor.film 6339

2241 film.distributor.film 6340

2241 film.distributor.film 6341

2241 film.production_company.film 6031

3350 people.person.place_of_birth 4191

3430 film.person_or_entity_appearing_in_film.film 4352

33 music.artist.origin 4057

777 film.actor.film 6342

1061 people.person.place_of_birth 6343

622 film.person_or_entity_appearing_in_film.film 6344

894 film.person_or_entity_appearing_in_film.film 6345

2231 music.artist.origin 6346

387 film.person_or_entity_appearing_in_film.film 6347

1360 film.person_or_entity_appearing_in_film.film 6348

3303 music.artist.origin 3972

882 music.artist.origin 6349

1357 film.person_or_entity_appearing_in_film.film 6350

3000 film.actor.film 6351

3321 film.actor.film 6352

2233 music.artist.origin 4195

3433 film.actor.film 4801

3433 film.person_or_entity_appearing_in_film.film 6353

2165 music.artist.origin 4380

81 film.person_or_entity_appearing_in_film.film 6250

2711 film.person_or_entity_appearing_in_film.film 6354

206 film.person_or_entity_appearing_in_film.film 6355

3774 film.film.genre 4062

2635 film.actor.film 6356

2635 film.actor.film 6357

1157 film.actor.film 4809

1828	music.artist.origin	4405

1351	people.deceased_person.place_of_death	3947

699	music.artist.origin	6358

3827	film.actor.film	6359

2724	film.person_or_entity_appearing_in_film.film	4136

2578	music.artist.origin	4195

662	film.actor.film	6360

1265	music.artist.origin	6361

286	music.artist.origin	4057

2560	music.artist.origin	4013

1488	event.agent.performance	4099

774	film.person_or_entity_appearing_in_film.film	6362

1982	film.person_or_entity_appearing_in_film.film	6363

127	film.actor.film	6364

3040	film.actor.film	6365

192	music.artist.track	6366

3250	film.actor.film	6367

2497	film.actor.film	4503

414	film.person_or_entity_appearing_in_film.film	4846

2562	film.person_or_entity_appearing_in_film.film	5067

408	film.actor.film	6368

47	film.actor.film	6369

3745	film.actor.film	6370

270	film.actor.film	6371

1579	film.actor.film	6372

3738	cvg.publisher.games_published	4139

3738	games.publisher.games_published	6373

2430	film.actor.film	5928

3272	people.person.place_of_birth	6374

2967	film.person_or_entity_appearing_in_film.film	6375

2335	people.person.place_of_birth	5662
589	music.artist.origin	4977
1015	film.person_or_entity_appearing_in_film.film	6376
2241	film.distributor.film	6377
2241	film.distributor.film	5134
2241	film.distributor.film	6378
2241	film.distributor.film	6379
2241	film.distributor.film	6380
2241	film.production_company.film	6381
2814	people.person.place_of_birth	4094
481	film.person_or_entity_appearing_in_film.film	6382
107	music.artist.origin	3864
2486	music.artist.origin	3916
2525	music.artist.origin	3849
114	film.actor.film	5783
2100	award.competitor.award_nominations	6009
3772	film.film.star	6383
1969	music.artist.origin	4419
3205	music.artist.origin	4150
1167	music.artist.origin	3849
702	music.album.genre	6384
1954	film.person_or_entity_appearing_in_film.film	4802
2519	film.person_or_entity_appearing_in_film.film	6385
298	film.person_or_entity_appearing_in_film.film	4794
3099	film.person_or_entity_appearing_in_film.film	6386
3279	film.actor.film	6387
923	film.actor.film	6388
3688	film.person_or_entity_appearing_in_film.film	6389
2348	music.artist.origin	4852
2010	film.person_or_entity_appearing_in_film.film	6390

3544 film.person_or_entity_appearing_in_film.film 6194

3763 film.person_or_entity_appearing_in_film.film 4045

206 film.person_or_entity_appearing_in_film.film 6391

997 film.person_or_entity_appearing_in_film.film 6392

3620 music.artist.origin 4583

202 film.person_or_entity_appearing_in_film.film 5052

2635 film.actor.film 6393

3384 people.person.place_of_birth 6185

540 film.person_or_entity_appearing_in_film.film 3957

115 film.person_or_entity_appearing_in_film.film 6394

785 film.actor.film 6395

1720 film.producer.film 6396

3496 film.film.actor 6397

141 film.person_or_entity_appearing_in_film.film 4539

1153 film.actor.film 6398

67 film.actor.film 6399

1013 music.artist.origin 6361

3814 music.artist.origin 4010

608 music.artist.origin 6400

2014 film.film.genre 6401

2080 film.actor.film 6402

584 film.person_or_entity_appearing_in_film.film 6403

3167 film.person_or_entity_appearing_in_film.film 5112

672 film.actor.film 6404

2332 music.artist.origin 6405

628 film.actor.film 5026

1465 film.person_or_entity_appearing_in_film.film 4283

1423 film.person_or_entity_appearing_in_film.film 6406

2096 film.actor.film 6407

2743 people.person.place_of_birth 6408

2597 film.person_or_entity_appearing_in_film.film 4132

665 people.person.place_of_birth 4232

466 film.person_or_entity_appearing_in_film.film 6409

2483 music.artist.album 6410

2208 film.person_or_entity_appearing_in_film.film 4026

436 film.person_or_entity_appearing_in_film.film 5313

2414 film.actor.film 6411

901 film.actor.film 6412

1249 film.person_or_entity_appearing_in_film.film 6413

2241 film.distributor.film 6414

2241 film.distributor.film 6415

2241 film.distributor.film 5338

2241 film.production_company.film 6416

556 music.artist.origin 6417

3297 film.person_or_entity_appearing_in_film.film 6418

3134 music.artist.origin 5161

1618 film.actor.film 4671

2995 music.artist.origin 5208

159 music.artist.origin 4347

1556 film.actor.film 6419

1278 award.competitor.award_nominations 6420

434 film.actor.film 6421

3049 film.actor.film 6422

3049 film.actor.film 6423

3557 film.film.director5611

1411 music.artist.origin 5125

2148 music.artist.origin 6242

705 music.artist.origin 4204

594 film.person_or_entity_appearing_in_film.film 6424

3095 film.person_or_entity_appearing_in_film.film 4853

533 people.person.place_of_birth 4868

601 music.artist.origin 6425

1372 film.person_or_entity_appearing_in_film.film 6426

148 film.person_or_entity_appearing_in_film.film 6427

2635 film.actor.film 4333

1719 film.person_or_entity_appearing_in_film.film 6428

2494 film.person_or_entity_appearing_in_film.film 6292

785 film.actor.film 4619

1153 film.actor.film 6429

1153 film.person_or_entity_appearing_in_film.film 6430

2963 people.person.place_of_birth 6431

995 film.person_or_entity_appearing_in_film.film 6432

67 film.actor.film 6433

633 music.artist.origin 5569

808 film.person_or_entity_appearing_in_film.film 6160

1473 film.actor.film 6127

1473 film.person_or_entity_appearing_in_film.film 5281

2337 film.actor.film 6434

1412 people.person.place_of_birth 6435

1706 film.person_or_entity_appearing_in_film.film 5758

1497 people.person.place_of_birth 3895

3040 film.actor.film 5283

2946 music.artist.origin 4391

3795 film.actor.film 5800

3568 music.artist.origin 3961

1181 film.person_or_entity_appearing_in_film.film 4874

2562 film.actor.film 4283

2095 film.actor.film 6436

2636 film.actor.film 6437

679 film.person_or_entity_appearing_in_film.film 4092

2070 film.actor.film 6307

1211 film.actor.film 6438

2460 film.person_or_entity_appearing_in_film.film 6439

1981 film.actor.film 6440

2539 music.artist.origin 3855

2867 film.actor.film 6441

2241 film.distributor.film 6442

2241 film.distributor.film 4032

2241 film.distributor.film 6443

2241 film.production_company.film 6442

2241 film.production_company.film 4733

2241 film.production_company.film 6444

2241 film.production_company.film 6445

2241 film.production_company.film 6446

490 film.person_or_entity_appearing_in_film.film 6447

1464 people.person.place_of_birth 6448

2624 music.artist.origin 3897

2078 film.person_or_entity_appearing_in_film.film 6449

998 film.person_or_entity_appearing_in_film.film 6450

894 film.person_or_entity_appearing_in_film.film 6451

1461 film.person_or_entity_appearing_in_film.film 6278

19 music.artist.origin 4003

655 music.artist.origin 4272

1511 music.artist.origin 4077

1580 award.nominee.award_nominations 6452

1817 film.person_or_entity_appearing_in_film.film 6453

1106 music.artist.origin 4199

3752 award.competitor.award_nominations 6454

3279 film.actor.film 6455

3279 film.person_or_entity_appearing_in_film.film 5560

3132 film.actor.film 6456

1658 people.person.place_of_birth 6457

2938 film.actor.film 5069

1358 film.person_or_entity_appearing_in_film.film 4858

997 film.actor.film 3951

1719 film.actor.film 6458

3287 film.actor.film 6459

623 film.person_or_entity_appearing_in_film.film 5690

1998 film.actor.film 5056

3020 film.actor.film 6460

358 film.person_or_entity_appearing_in_film.film 5276

67 film.actor.film 4914

1473 film.actor.film 4549

645 film.actor.film 6461

604 music.artist.origin 4816

377 film.person_or_entity_appearing_in_film.film 6462

377 people.person.place_of_birth 5617

3372 music.artist.origin 6463

386 film.actor.film 6064

3368 music.artist.origin 4058

980 people.person.place_of_birth 6463

2913 film.actor.film 6464

1744 film.actor.film 6465

1953 music.artist.origin 4496

974 film.actor.film 6466

128 film.actor.film 6467

2636 film.actor.film 4555

2636 film.actor.film 6468

3179 film.person_or_entity_appearing_in_film.film 6469

3166 film.person_or_entity_appearing_in_film.film 6177

1353 film.person_or_entity_appearing_in_film.film 4692

466 film.person_or_entity_appearing_in_film.film 6470

1981 film.person_or_entity_appearing_in_film.film 6471

343 music.artist.origin 4768

3738 games.publisher.games_published 5925

3738 games.publisher.games_published 4393

3738 games.publisher.games_published 6472

3193 music.musician.instruments_played 4349

2414 people.person.profession 4221

3309 music.artist.album 6473

455 music.artist.origin 3859

2241 film.distributor.film 6474

2241 film.production_company.film 4566

2241 film.production_company.film 6475

2241 film.production_company.film 6476

3720 music.artist.origin 3963

2779 music.artist.album 6477

2459 music.artist.origin 4133

1624 film.actor.film 6478

591 music.artist.album 6479

643 film.actor.film 6480

3546 type.object.key 3546

1918 people.person.place_of_birth 6019

1059 film.actor.film 5940

2383 music.artist.origin 5845

3049 film.actor.film 6481

3321 film.actor.film 6482

1241 film.film.language 4076

814 people.person.place_of_birth 4362

3752 film.person_or_entity_appearing_in_film.film 6041

385 film.actor.film 6483

3468 music.artist.album 6484

2211 film.person_or_entity_appearing_in_film.film 5990

1632 film.actor.film 3937

1814 film.film.country 4380

3475 film.person_or_entity_appearing_in_film.film 5352

786 people.person.place_of_birth 4357

2402 film.director.film 5835

1633 film.person_or_entity_appearing_in_film.film 6485

1633 film.person_or_entity_appearing_in_film.film 6486

578 music.artist.origin 6487

2494 film.person_or_entity_appearing_in_film.film 6199

3827 film.actor.film 6488

3827 film.person_or_entity_appearing_in_film.film 6489

3827 film.person_or_entity_appearing_in_film.film 6490

1891 film.actor.film 6038

1841 event.agent.performance 4898

1789 music.artist.origin 3895

1032 film.actor.film 6491

1473 film.actor.film 4064

1473 film.person_or_entity_appearing_in_film.film 6492

136 film.actor.film 6493

2389 music.artist.origin 3891

3604 people.person.place_of_birth 6494

3768 music.artist.origin 4536

893 film.person_or_entity_appearing_in_film.film 5763

3845 people.person.place_of_birth 4471

3498 film.person_or_entity_appearing_in_film.film 6495

2491 music.artist.track 6496

2913 film.person_or_entity_appearing_in_film.film 5670

736 film.person_or_entity_appearing_in_film.film 5847

1773 film.person_or_entity_appearing_in_film.film 6497

1601 film.person_or_entity_appearing_in_film.film 4021

408 film.person_or_entity_appearing_in_film.film 6498

1204 film.person_or_entity_appearing_in_film.film 6499

3821 film.person_or_entity_appearing_in_film.film 6103

3047 film.person_or_entity_appearing_in_film.film 6500

1458 film.person_or_entity_appearing_in_film.film 6501

1698 music.artist.origin 4380

359 music.artist.origin 6502

3166 film.actor.film 6503

1895 people.person.place_of_birth 6504

3017 music.artist.origin 4471

3238 music.artist.origin 6505

3373 music.artist.origin 4087

1840 people.person.profession 4221

484 film.actor.film 6506

1330 music.artist.origin 4057

3738 cvg.publisher.games_published 6507

3738 games.publisher.games_published 4562

3738 games.publisher.games_published 5177

3738 games.publisher.games_published 6508

834 music.artist.origin 6509

3452 film.actor.film 6510

901 film.person_or_entity_appearing_in_film.film 6511

901 film.person_or_entity_appearing_in_film.film 6512

2197 music.artist.origin 4583

2241 film.distributor.film 6513

2241 film.distributor.film 5333

2241 film.production_company.film 6227

2262 music.artist.origin 3864

1780 film.person_or_entity_appearing_in_film.film 6514

2487 film.actor.film 6515

2311 film.person_or_entity_appearing_in_film.film 5086

382 film.actor.film 4792

894 film.person_or_entity_appearing_in_film.film 6516

2098 music.artist.origin 5512

708 music.artist.origin 4204

2694 music.artist.origin 3897

757 film.actor.film 4645

3049 film.actor.film 6517

2847 music.artist.origin 6518

2634 film.actor.film 6519

1191 film.person_or_entity_appearing_in_film.film 6520

1323 film.person_or_entity_appearing_in_film.film 4042

417 music.artist.origin 3961

1814 film.film.genre 6401

1358 film.actor.film 4481

396 film.actor.film 6521

1365 film.person_or_entity_appearing_in_film.film 6335

2635 film.actor.film 6522

2635 film.person_or_entity_appearing_in_film.film 6523

2635 film.person_or_entity_appearing_in_film.film 6524

1633 film.actor.film 6525

2161 award.winner.awards_won 6526

3208 music.artist.origin 5070

1153 film.person_or_entity_appearing_in_film.film 6527

1891 film.actor.film 4814

1480 music.artist.album 4012

1141 film.actor.film 6528

2776	location.location.contained_by	4380
91	film.person_or_entity_appearing_in_film.film	6529
1151	music.album.genre	6530
2056	music.artist.origin	3916
3598	film.person_or_entity_appearing_in_film.film	6531
3795	film.person_or_entity_appearing_in_film.film	6532
112	music.artist.origin	3864
3344	film.actor.film	6533
508	award.competitor.award_nominations	6534
3797	film.actor.film	6535
2848	film.film.production_company	2241
414	film.actor.film	6536
2710	film.person_or_entity_appearing_in_film.film	6537
128	film.person_or_entity_appearing_in_film.film	6538
128	film.person_or_entity_appearing_in_film.film	6467
73	people.person.place_of_birth	4369
2743	film.actor.film	6539
2597	film.actor.film	6540
2070	film.actor.film	6541
1299	film.actor.film	6542
1353	film.person_or_entity_appearing_in_film.film	6543
3701	film.actor.film	6544
1682	music.artist.origin	6545
3738	cvg.publisher.games_published	6546
901	film.actor.film	6547
2241	film.distributor.film	6548
2241	film.distributor.film	6549
2241	film.distributor.film	6550
2241	film.production_company.film	6551
2241	film.production_company.film	4511

1936	film.actor.film	6552

161 music.artist.origin	6553

3083	music.artist.origin	6554

1595	music.artist.origin	6555

3486	film.person_or_entity_appearing_in_film.film	6556

1807	music.artist.origin	6557

1051	film.actor.film	4307

3321	film.person_or_entity_appearing_in_film.film	6482

743 music.artist.origin	4063

1599	music.artist.origin	4232

3027	music.musician.instruments_played	3959

889 music.artist.origin	3864

3026	film.person_or_entity_appearing_in_film.film	6558

3390	film.actor.film	6559

3390	people.person.place_of_birth	5270

3508	film.actor.film	6155

3637	film.actor.film	6560

2826	music.artist.origin	4471

2904	film.actor.film	6561

2904	film.person_or_entity_appearing_in_film.film	5834

567 film.person_or_entity_appearing_in_film.film	6562

2635	film.actor.film	6563

1930	music.artist.origin	6564

44	music.artist.origin	4362

115 film.actor.film	5148

2494	film.person_or_entity_appearing_in_film.film	5836

623 film.person_or_entity_appearing_in_film.film	6565

3317	film.actor.film	6566

756 film.person_or_entity_appearing_in_film.film	6567

2134	event.agent.performance	4770

67 film.actor.film 6329

1473 film.person_or_entity_appearing_in_film.film 6568

2990 film.person_or_entity_appearing_in_film.film 5158

1692 film.person_or_entity_appearing_in_film.film 6569

1412 film.person_or_entity_appearing_in_film.film 6570

39 film.actor.film 6571

2398 event.agent.performance 4099

2080 film.person_or_entity_appearing_in_film.film 5319

3598 film.person_or_entity_appearing_in_film.film 6132

3598 film.person_or_entity_appearing_in_film.film 6572

3470 people.person.place_of_birth 5662

2408 people.deceased_person.place_of_death 6242

3799 film.actor.film 6573

1796 film.person_or_entity_appearing_in_film.film 6574

408 film.actor.film 6575

974 film.person_or_entity_appearing_in_film.film 6576

2070 film.person_or_entity_appearing_in_film.film 6577

2016 music.artist.origin 4852

1353 film.actor.film 5221

2460 film.actor.film 4828

527 film.person_or_entity_appearing_in_film.film 6197

3600 music.artist.origin 3992

484 film.person_or_entity_appearing_in_film.film 6578

2483 film.actor.film 6579

3738 cvg.publisher.games_published 6580

3738 cvg.publisher.games_published 6581

3738 cvg.publisher.games_published 4345

436 film.person_or_entity_appearing_in_film.film 6582

2241 film.production_company.film 6583

33 award.competitor.award_nominations 5300

1347 music.musician.instruments_played 5619

894 film.actor.film 6584

2206 film.actor.film 4742

1051 people.person.place_of_birth 3888

296 music.artist.origin 5662

1062 music.artist.origin 6585

3388 music.musician.instruments_played 3959

538 film.actor.film 4947

538 film.person_or_entity_appearing_in_film.film 6350

3321 film.person_or_entity_appearing_in_film.film 6352

3732 people.person.place_of_birth 4332

1105 film.actor.film 6586

3132 film.actor.film 6587

2512 film.actor.film 6588

2312 film.actor.film 5750

1025 film.actor.film 5979

2130 film.actor.film 4823

683 film.actor.film 6589

3507 film.actor.film 6590

3125 music.artist.album 6591

1021 music.artist.origin 6555

1709 music.artist.origin 6564

1304 music.artist.origin 4058

2904 film.person_or_entity_appearing_in_film.film 6561

349 music.artist.origin 3850

3381 film.person_or_entity_appearing_in_film.film 6592

2635 film.person_or_entity_appearing_in_film.film 4647

2635 film.person_or_entity_appearing_in_film.film 5053

1072 music.artist.origin 4454

2222 music.musician.instruments_played 5802

1349	film.person_or_entity_appearing_in_film.film	6593
658	music.artist.origin	3986
3155	people.person.place_of_birth	6594
540	film.person_or_entity_appearing_in_film.film	6595
818	film.person_or_entity_appearing_in_film.film	6596
2494	film.person_or_entity_appearing_in_film.film	6597
1631	music.artist.origin	4191
58	people.person.place_of_birth	4455
662	film.person_or_entity_appearing_in_film.film	6598
3362	music.artist.origin	4294
3317	film.person_or_entity_appearing_in_film.film	6599
116	film.person_or_entity_appearing_in_film.film	5205
3559	film.actor.film	6600
2147	film.actor.film	6601
2603	people.person.place_of_birth	3895
136	film.person_or_entity_appearing_in_film.film	3967
2482	film.person_or_entity_appearing_in_film.film	6602
772	music.artist.origin	4521
3040	film.actor.film	4355
279	award.competitor.award_nominations	6603
3121	film.actor.film	5111
1079	film.writer.film	6169
619	film.actor.film	4384
80	music.artist.track	6604
2913	film.actor.film	4845
357	film.actor.film	5663
1211	type.object.key	1211
2819	music.artist.album	5859
2653	music.artist.origin	6012
1229	music.artist.origin	3956

79 film.person_or_entity_appearing_in_film.film 6605

3701 film.person_or_entity_appearing_in_film.film 6544

484 film.actor.film 6606

3738 cvg.publisher.games_published 6607

3738 cvg.publisher.games_published 6608

734 people.person.place_of_birth 6425

901 people.person.place_of_birth 5421

1829 music.artist.origin 5814

2867 film.person_or_entity_appearing_in_film.film 4132

442 film.person_or_entity_appearing_in_film.film 6609

2241 film.production_company.film 6610

2849 music.artist.origin 4957

2099 film.person_or_entity_appearing_in_film.film 4791

2019 music.artist.origin 3916

2275 film.person_or_entity_appearing_in_film.film 5606

2581 music.artist.origin 4380

1806 film.person_or_entity_appearing_in_film.film 6611

1806 film.person_or_entity_appearing_in_film.film 6612

3578 music.artist.origin 3961

3331 film.person_or_entity_appearing_in_film.film 6613

2184 film.person_or_entity_appearing_in_film.film 6614

2860 people.person.place_of_birth 5033

36 music.producer.tracks_produced 4041

2746 music.artist.origin 4013

3049 film.person_or_entity_appearing_in_film.film 6615

727 people.person.place_of_birth 4191

739 music.artist.origin 5814

3712 film.actor.film 6616

3579 film.person_or_entity_appearing_in_film.film 6617

2010 film.person_or_entity_appearing_in_film.film 5235

3829	music.artist.origin	6618

2787	music.artist.origin	6619

363 film.person_or_entity_appearing_in_film.film	4047

2130	film.person_or_entity_appearing_in_film.film	5003

2419	people.person.place_of_birth	4347

552 film.actor.film	5052

1839	film.person_or_entity_appearing_in_film.film	6620

2229	music.artist.origin	4362

1006	award.nominee.award_nominations	6621

1633	film.person_or_entity_appearing_in_film.film	6622

548 film.person_or_entity_appearing_in_film.film	6623

623 film.actor.film	6624

2698	music.artist.origin	4701

2978	film.person_or_entity_appearing_in_film.film	6625

307 film.person_or_entity_appearing_in_film.film	4595

2708	film.actor.film	6626

67	film.actor.film	6627

2370	film.person_or_entity_appearing_in_film.film	4917

2125	film.actor.film	6628

3656	people.deceased_person.place_of_death	4357

911 music.artist.origin	5662

314 music.artist.album	6321

3741	film.actor.film	6629

604 film.actor.film	6630

2080	film.actor.film	6328

2080	film.person_or_entity_appearing_in_film.film	6631

2217	film.person_or_entity_appearing_in_film.film	6632

357 music.artist.track	6633

646 film.actor.film	6634

646 film.person_or_entity_appearing_in_film.film	6635

477 music.artist.origin 6636

3355 film.actor.film 6637

1630 film.person_or_entity_appearing_in_film.film 6231

3166 film.person_or_entity_appearing_in_film.film 6638

2460 film.actor.film 5770

2466 music.artist.origin 4583

466 film.person_or_entity_appearing_in_film.film 6639

1981 film.person_or_entity_appearing_in_film.film 4831

3738 games.publisher.games_published 6640

3738 games.publisher.games_published 6641

1359 film.actor.film 5642

1463 award.competitor.awards_won 6071

2241 film.distributor.film 4627

2241 film.distributor.film 6642

2241 film.distributor.film 6643

832 film.person_or_entity_appearing_in_film.film 6644

3084 music.artist.origin 4217

3353 music.artist.origin 4167

777 film.person_or_entity_appearing_in_film.film 4470

1360 film.actor.film 6645

3607 film.person_or_entity_appearing_in_film.film 5447

3780 film.person_or_entity_appearing_in_film.film 6646

2245 film.person_or_entity_appearing_in_film.film 4992

159 people.person.place_of_birth 4347

246 music.artist.origin 5064

1475 film.person_or_entity_appearing_in_film.film 6647

3231 film.person_or_entity_appearing_in_film.film 6040

3648 film.actor.film 6648

2711 film.actor.film 4699

1094 music.artist.origin 4174

3271	music.artist.origin	6649
2595	film.actor.film	4750
2238	film.person_or_entity_appearing_in_film.film	6650
2635	film.actor.film	5400
2222	people.person.place_of_birth	4013
115	film.person_or_entity_appearing_in_film.film	6651
1397	people.person.place_of_birth	5605
3505	music.artist.origin	4057
2786	film.person_or_entity_appearing_in_film.film	3896
404	people.person.place_of_birth	5357
1833	film.person_or_entity_appearing_in_film.film	4623
674	film.actor.film	6652
153	music.artist.origin	4199
3364	film.actor.film	6653
3074	people.person.place_of_birth	4673
91	film.actor.film	6529
831	music.musician.instruments_played	4349
557	film.actor.film	6654
2925	music.artist.origin	4362
862	film.person_or_entity_appearing_in_film.film	6655
755	film.person_or_entity_appearing_in_film.film	6656
3159	film.actor.film	6657
2636	film.actor.film	5631
2636	film.person_or_entity_appearing_in_film.film	6658
489	music.artist.origin	4454
679	music.artist.album	6659
2070	film.actor.film	3957
1573	film.actor.film	5632
1353	film.actor.film	5220
3366	film.actor.film	4774

1276 music.artist.album 6660

602 film.actor.film 6661

1981 music.musician.instruments_played 5174

3738 cvg.publisher.games_published 6662

3738 games.publisher.games_published 6663

3738 games.publisher.games_published 6664

2967 people.person.place_of_birth 5569

901 film.actor.film 6665

2241 film.distributor.film 6666

2241 film.distributor.film 6667

2241 film.distributor.film 4789

516 film.person_or_entity_appearing_in_film.film 6668

3276 film.person_or_entity_appearing_in_film.film 6669

681 film.actor.film 6670

2487 film.person_or_entity_appearing_in_film.film 4303

2487 film.person_or_entity_appearing_in_film.film 5865

2319 music.artist.origin 4963

1767 film.person_or_entity_appearing_in_film.film 6671

1756 music.artist.origin 5411

1894 music.artist.album 6672

2355 music.artist.album 6673

1996 music.musician.instruments_played 5558

3486 film.actor.film 6674

1360 film.actor.film 6675

2261 people.person.place_of_birth 4975

2206 film.person_or_entity_appearing_in_film.film 4742

3526 film.person_or_entity_appearing_in_film.film 5972

3374 film.actor.film 6285

3374 people.person.place_of_birth 4583

253 film.person_or_entity_appearing_in_film.film 6676

1378	film.actor.film	6677

1378	film.person_or_entity_appearing_in_film.film	6678

600 music.artist.origin	4816

3049	film.person_or_entity_appearing_in_film.film	5554

2843	film.person_or_entity_appearing_in_film.film	6679

372 film.actor.film	6680

3579	film.actor.film	6681

2274	music.musician.instruments_played	6682

3132	film.person_or_entity_appearing_in_film.film	6335

1866	film.actor.film	6683

240 music.artist.origin	4521

2449	film.person_or_entity_appearing_in_film.film	6684

3428	film.person_or_entity_appearing_in_film.film	6685

193 film.actor.film	6686

2669	film.person_or_entity_appearing_in_film.film	6322

683 film.person_or_entity_appearing_in_film.film	6589

2684	film.person_or_entity_appearing_in_film.film	5269

959 music.artist.album	6687

1776	film.actor.film	4494

2635	film.person_or_entity_appearing_in_film.film	6688

2494	film.person_or_entity_appearing_in_film.film	4152

87	music.artist.origin	4057

94	film.actor.film	6689

1748	film.actor.film	6690

633 film.person_or_entity_appearing_in_film.film	6691

1473	film.actor.film	4569

1473	film.actor.film	6692

0	music.artist.origin	4454

595 film.actor.film	6693

16	music.artist.origin	6694

2737 film.person_or_entity_appearing_in_film.film 6695

30 film.person_or_entity_appearing_in_film.film 6696

2975 music.artist.origin 4471

508 film.person_or_entity_appearing_in_film.film 5766

2491 music.producer.tracks_produced 6496

2914 film.actor.film 6697

458 music.artist.album 6698

198 music.artist.origin 4454

2070 film.person_or_entity_appearing_in_film.film 6175

2689 award.competitor.awards_won 6699

3738 games.publisher.games_published 4137

3738 games.publisher.games_published 6700

2293 film.actor.film 5366

666 music.musician.instruments_played 4349

2138 film.person_or_entity_appearing_in_film.film 6226

796 film.actor.film 5587

2241 film.distributor.film 6701

2241 film.distributor.film 6702

2241 film.distributor.film 5062

2241 film.production_company.film 4934

2241 film.production_company.film 4936

2779 music.artist.album 6703

1061 film.person_or_entity_appearing_in_film.film 6704

2189 film.actor.film 6705

1819 film.person_or_entity_appearing_in_film.film 6706

591 film.person_or_entity_appearing_in_film.film 3933

894 film.actor.film 6451

2126 film.actor.film 5186

1360 film.person_or_entity_appearing_in_film.film 6707

470 music.musician.instruments_played 3959

958 people.person.place_of_birth 4927

636 film.actor.film 6708

1806 film.actor.film 4025

1234 people.person.place_of_birth 4391

2948 music.artist.origin 4732

1475 film.person_or_entity_appearing_in_film.film 6709

1378 film.actor.film 5974

298 film.actor.film 4794

2623 music.artist.origin 4232

2658 film.person_or_entity_appearing_in_film.film 6710

2010 film.person_or_entity_appearing_in_film.film 6711

1962 film.person_or_entity_appearing_in_film.film 6712

1962 film.person_or_entity_appearing_in_film.film 5752

687 film.actor.film 5678

98 music.artist.origin 4443

206 film.actor.film 6713

1358 film.person_or_entity_appearing_in_film.film 5642

1358 film.person_or_entity_appearing_in_film.film 5680

3774 film.film.star 6714

115 film.actor.film 6715

963 music.artist.origin 5265

548 film.actor.film 6716

1998 film.person_or_entity_appearing_in_film.film 6295

2134 event.agent.performance 4898

1473 film.person_or_entity_appearing_in_film.film 6717

895 film.actor.film 4066

3364 film.person_or_entity_appearing_in_film.film 5530

127 film.actor.film 4073

2513 music.artist.origin 3897

494 music.artist.origin 4057

66 film.person_or_entity_appearing_in_film.film 5624

30 people.person.profession 4221

557 film.actor.film 4282

2912 film.person_or_entity_appearing_in_film.film 6718

3109 people.deceased_person.place_of_death 6502

3498 film.person_or_entity_appearing_in_film.film 6719

3117 music.artist.origin 4701

3063 music.artist.origin 6720

2914 film.actor.film 4769

2830 people.person.place_of_birth 4217

1465 film.actor.film 6721

403 music.artist.origin 6722

2070 film.person_or_entity_appearing_in_film.film 4078

1211 film.actor.film 6037

2460 film.person_or_entity_appearing_in_film.film 5250

294 people.person.place_of_birth 6723

3738 games.publisher.games_published 6724

3338 music.artist.origin 6725

2478 music.artist.origin 4150

901 film.actor.film 6511

3248 award.competitor.awards_won 3928

2241 film.distributor.film 6726

3103 people.person.place_of_birth 3942

777 film.actor.film 6727

3706 film.person_or_entity_appearing_in_film.film 5549

622 film.actor.film 6344

1586 music.artist.origin 3850

387 film.actor.film 6347

1768 film.actor.film 6728

2678 film.person_or_entity_appearing_in_film.film 5764

2184 film.actor.film 6729

2861 film.person_or_entity_appearing_in_film.film 6730

1817 film.actor.film 6453

1646 music.artist.origin 4768

2870 film.actor.film 6731

3321 film.actor.film 6732

1468 film.person_or_entity_appearing_in_film.film 6733

1323 film.actor.film 4102

2163 music.artist.origin 5213

2658 film.person_or_entity_appearing_in_film.film 6734

3753 people.person.place_of_birth 6735

1962 film.person_or_entity_appearing_in_film.film 4653

3473 film.actor.film 6252

2816 music.artist.origin 6736

1358 film.actor.film 6737

2833 people.person.place_of_birth 5213

1157 film.person_or_entity_appearing_in_film.film 6738

2155 music.artist.origin 3947

1153 film.person_or_entity_appearing_in_film.film 6739

435 film.person_or_entity_appearing_in_film.film 6740

2050 film.actor.film 6298

2734 film.actor.film 6741

2359 film.person_or_entity_appearing_in_film.film 6742

1473 film.actor.film 6743

2020 music.artist.origin 4362

85 music.artist.origin 4454

1209 film.actor.film 6433

1209 film.actor.film 6744

893 film.person_or_entity_appearing_in_film.film 6745

1661 people.person.place_of_birth 4133

2830 film.actor.film 6746

646 film.person_or_entity_appearing_in_film.film 6634

70 film.actor.film 6330

2120 music.artist.origin 4347

3442 music.artist.origin 4053

3561 music.artist.origin 4455

1118 music.artist.origin 4454

2636 film.actor.film 4054

2597 film.actor.film 6347

2070 film.person_or_entity_appearing_in_film.film 6747

1840 film.person_or_entity_appearing_in_film.film 6748

3738 games.publisher.games_published 6749

782 film.actor.film 6750

2400 film.person_or_entity_appearing_in_film.film 5543

901 film.person_or_entity_appearing_in_film.film 5857

2241 film.distributor.film 6751

2241 film.distributor.film 6752

2241 film.distributor.film 6753

2241 film.distributor.film 4299

2241 film.distributor.film 6754

2241 film.production_company.film 6755

2468 music.artist.origin 6343

2078 film.actor.film 4476

2448 music.artist.origin 4174

2038 film.person_or_entity_appearing_in_film.film 6756

643 film.actor.film 3938

1806 film.actor.film 5918

1806 film.person_or_entity_appearing_in_film.film 6757

1772 film.actor.film 6758

1475 film.actor.film 6709

3242	film.person_or_entity_appearing_in_film.film	6759

3002	film.person_or_entity_appearing_in_film.film	4634

2037	music.artist.origin	6760

1241	film.film.executive_producer	6248

398 music.artist.origin	4057

2891	music.artist.origin	4057

1750	film.actor.film	6116

206 film.actor.film	5146

2635	film.actor.film	6761

1157	film.actor.film	6762

3384	film.actor.film	6256

2614	music.artist.origin	3942

1232	film.person_or_entity_appearing_in_film.film	6763

3111	film.actor.film	6764

1505	film.person_or_entity_appearing_in_film.film	6765

149 film.actor.film	6766

3100	music.artist.origin	4199

67	film.actor.film	6208

67	film.actor.film	6767

2370	film.person_or_entity_appearing_in_film.film	6768

2147	film.person_or_entity_appearing_in_film.film	6601

1473	film.actor.film	4761

1473	film.actor.film	6769

1473	film.person_or_entity_appearing_in_film.film	5731

1026	film.person_or_entity_appearing_in_film.film	6770

2337	film.writer.film	6771

895 film.actor.film	4176

1193	film.person_or_entity_appearing_in_film.film	4385

1209	film.actor.film	5398

645 film.actor.film	6772

2564	music.artist.origin	3971
3598	film.actor.film	6773
1434	film.person_or_entity_appearing_in_film.film	6774
74	film.actor.film	6775
2637	film.film.cinematographer	6776
2562	film.person_or_entity_appearing_in_film.film	4335
2636	film.actor.film	6777
2636	film.actor.film	6173
1211	film.person_or_entity_appearing_in_film.film	6778
3166	film.actor.film	5079
3166	film.person_or_entity_appearing_in_film.film	6413
445	film.person_or_entity_appearing_in_film.film	6779
79	film.actor.film	4025
79	film.person_or_entity_appearing_in_film.film	4241
3738	cvg.publisher.games_published	6780
3738	games.publisher.games_published	6781
3738	games.publisher.games_published	6782
436	film.actor.film	5374
2400	film.person_or_entity_appearing_in_film.film	6783
901	award.competitor.award_nominations	5180
901	film.actor.film	5929
1356	film.actor.film	6784
1011	film.person_or_entity_appearing_in_film.film	6785
2241	film.distributor.film	6786
2241	film.distributor.film	6787
2241	film.production_company.film	6788
1624	film.actor.film	6789
1624	film.person_or_entity_appearing_in_film.film	5340
2487	film.person_or_entity_appearing_in_film.film	6790
382	film.actor.film	6791

261 film.actor.film 6792

3669 film.actor.film 6793

2697 music.album.genre 6794

1475 film.person_or_entity_appearing_in_film.film 5045

696 film.person_or_entity_appearing_in_film.film 4418

1548 music.artist.origin 4108

2148 film.actor.film 6795

1623 film.actor.film 6796

1743 music.artist.origin 3849

1503 music.artist.origin 6797

3390 film.person_or_entity_appearing_in_film.film 6798

1303 music.artist.origin 3897

2010 film.person_or_entity_appearing_in_film.film 4480

3507 film.actor.film 6799

1100 music.artist.origin 3946

1662 film.person_or_entity_appearing_in_film.film 6800

1413 people.person.place_of_birth 4167

115 film.person_or_entity_appearing_in_film.film 6801

1720 film.director.film 6396

1720 film.writer.film 4273

1720 film.writer.film 4754

623 film.actor.film 6802

3827 film.person_or_entity_appearing_in_film.film 6803

1505 film.person_or_entity_appearing_in_film.film 6804

1153 film.actor.film 6805

1748 film.person_or_entity_appearing_in_film.film 5979

3788 film.person_or_entity_appearing_in_film.film 6806

2147 film.actor.film 6807

850 people.person.place_of_birth 5664

136 film.actor.film 6808

987 film.actor.film 6809

3295 music.artist.origin 4471

2080 film.actor.film 5987

976 film.person_or_entity_appearing_in_film.film 5493

151 people.person.profession 4221

2972 film.person_or_entity_appearing_in_film.film 6810

458 music.artist.album 4971

1119 music.artist.origin 4195

437 film.person_or_entity_appearing_in_film.film 6811

1605 music.artist.origin 4583

1570 people.person.place_of_birth 4013

3355 film.actor.film 4556

2650 film.film.writer 6812

2070 film.person_or_entity_appearing_in_film.film 6813

2460 film.person_or_entity_appearing_in_film.film 6814

1864 film.person_or_entity_appearing_in_film.film 5895

436 film.actor.film 6815

1837 music.artist.origin 4362

1463 film.actor.film 6816

2360 film.person_or_entity_appearing_in_film.film 4838

2241 film.distributor.film 6817

2241 film.production_company.film 6818

2241 film.production_company.film 6819

2586 music.artist.origin 5125

1624 film.actor.film 6820

2126 film.actor.film 5603

1767 film.person_or_entity_appearing_in_film.film 5640

3669 film.person_or_entity_appearing_in_film.film 6793

3486 film.actor.film 6821

538 film.actor.film 6822

1765 film.person_or_entity_appearing_in_film.film 6150

1954 film.person_or_entity_appearing_in_film.film 6823

810 film.person_or_entity_appearing_in_film.film 6824

3279 film.person_or_entity_appearing_in_film.film 4682

638 film.person_or_entity_appearing_in_film.film 6825

2312 film.person_or_entity_appearing_in_film.film 6826

3384 film.person_or_entity_appearing_in_film.film 6020

623 people.person.place_of_birth 6827

1044 award.nominee.award_nominations 6828

1197 music.artist.origin 6829

1881 film.actor.film 5882

236 people.person.place_of_birth 3916

2125 film.person_or_entity_appearing_in_film.film 3900

2188 music.artist.origin 6830

3580 music.artist.origin 6831

3209 music.artist.origin 6832

1754 film.person_or_entity_appearing_in_film.film 4314

751 music.artist.origin 6830

672 film.actor.film 6833

672 film.person_or_entity_appearing_in_film.film 6834

1435 film.actor.film 6835

1888 film.actor.film 6836

75 film.person_or_entity_appearing_in_film.film 6837

1453 film.person_or_entity_appearing_in_film.film 6617

3021 film.actor.film 6838

3819 music.artist.origin 6839

381 film.actor.film 6840

1299 film.person_or_entity_appearing_in_film.film 6841

3609 music.artist.origin 5106

3738 cvg.publisher.games_published 5926

3738 cvg.publisher.games_published 6842

1154 film.actor.film 6843

901 film.director.film 5429

1787 film.actor.film 6844

1463 award.winner.awards_won 6845

2241 film.distributor.film 6846

3297 film.person_or_entity_appearing_in_film.film 4846

1624 film.person_or_entity_appearing_in_film.film 6277

894 film.person_or_entity_appearing_in_film.film 6847

2126 film.person_or_entity_appearing_in_film.film 6848

1562 film.person_or_entity_appearing_in_film.film 4915

2648 music.artist.origin 4362

1133 film.person_or_entity_appearing_in_film.film 3934

371 film.person_or_entity_appearing_in_film.film 6849

2869 music.artist.origin 3855

341 film.film.star 5388

1417 film.person_or_entity_appearing_in_film.film 6850

1536 music.musician.instruments_played 3851

1496 film.person_or_entity_appearing_in_film.film 4207

1089 film.person_or_entity_appearing_in_film.film 5441

794 film.actor.film 5675

3648 film.actor.film 5480

388 music.artist.origin 4174

380 film.person_or_entity_appearing_in_film.film 6851

3048 film.person_or_entity_appearing_in_film.film 5774

204 film.actor.film 6852

206 film.actor.film 5197

1726 award.competitor.award_nominations 6853

1157 film.person_or_entity_appearing_in_film.film 6854

3194 music.artist.origin 6855

3801 music.artist.origin 6343

1247 people.deceased_person.place_of_death 4362

1555 music.artist.origin 3864

3827 film.actor.film 6856

2734 film.person_or_entity_appearing_in_film.film 6857

2359 film.actor.film 6858

1141 award.competitor.awards_won 6859

801 music.artist.origin 4087

1588 music.artist.origin 6860

2756 music.artist.origin 5287

1407 film.actor.film 4923

2848 film.film.music 1044

70 film.actor.film 4715

70 film.actor.film 6861

70 film.person_or_entity_appearing_in_film.film 4324

974 film.actor.film 6862

3005 music.artist.origin 4057

2070 film.actor.film 6863

2070 film.person_or_entity_appearing_in_film.film 6864

2460 film.person_or_entity_appearing_in_film.film 5028

602 film.actor.film 6865

3738 cvg.publisher.games_published 6373

1012 music.artist.origin 3932

999 film.actor.film 5124

625 film.actor.film 5032

791 film.actor.film 5378

2867 film.actor.film 6866

2360 film.actor.film 6337

2241 film.distributor.film 6867

2241 film.production_company.film 6311

2241 film.production_company.film 5062

419 film.actor.film 4915

681 film.person_or_entity_appearing_in_film.film 4549

2326 people.person.place_of_birth 6868

2275 film.person_or_entity_appearing_in_film.film 6869

2741 film.person_or_entity_appearing_in_film.film 4546

776 music.artist.origin 4521

1278 award.competitor.awards_won 5717

3838 music.artist.origin 5127

1378 film.actor.film 4850

1378 film.actor.film 6870

1378 film.person_or_entity_appearing_in_film.film 6870

3321 film.person_or_entity_appearing_in_film.film 6871

794 film.person_or_entity_appearing_in_film.film 6872

2556 film.person_or_entity_appearing_in_film.film 5694

2711 film.actor.film 6354

1816 film.actor.film 6873

2010 film.actor.film 6874

2891 people.person.place_of_birth 5557

3763 film.actor.film 6115

649 music.artist.origin 3849

575 music.artist.origin 3864

1138 music.artist.origin 3963

1955 film.actor.film 6875

3088 people.person.place_of_birth 4919

1365 music.artist.origin 4174

2635 film.actor.film 6876

2635 film.actor.film 6877

1309 music.artist.origin 6878

115 film.actor.film 4706

1719	film.person_or_entity_appearing_in_film.film	4907

410 film.actor.film	6879

623 film.person_or_entity_appearing_in_film.film	6880

623 film.person_or_entity_appearing_in_film.film	6881

2063	event.agent.performance	4603

2837	music.artist.origin	6504

2147	film.person_or_entity_appearing_in_film.film	6882

491 people.person.place_of_birth	4057

1473	film.person_or_entity_appearing_in_film.film	6414

189 film.person_or_entity_appearing_in_film.film	6883

2389	film.person_or_entity_appearing_in_film.film	5949

124 film.actor.film	5159

407 film.person_or_entity_appearing_in_film.film	6884

2080	film.person_or_entity_appearing_in_film.film	6402

3598	film.person_or_entity_appearing_in_film.film	6885

2071	award.winner.awards_won	6886

1764	film.actor.film	6887

1764	film.person_or_entity_appearing_in_film.film	5042

2913	film.actor.film	6888

3777	film.actor.film	6889

646 film.actor.film	6890

1610	music.artist.origin	4583

1082	people.person.place_of_birth	3982

2636	film.person_or_entity_appearing_in_film.film	6891

133 event.agent.performance	4251

2682	film.person_or_entity_appearing_in_film.film	6892

3738	cvg.publisher.games_published	4188

2400	film.actor.film	6893

999 film.person_or_entity_appearing_in_film.film	6894

3501	people.person.place_of_birth	4057

2241 film.distributor.film 6895

2241 film.distributor.film 5818

2241 film.distributor.film 5671

2241 film.production_company.film 6896

3430 film.person_or_entity_appearing_in_film.film 4941

3297 film.actor.film 6858

2487 film.actor.film 5438

2311 film.person_or_entity_appearing_in_film.film 4230

2189 film.person_or_entity_appearing_in_film.film 6705

3706 film.producer.film 6897

498 music.producer.tracks_produced 5472

3337 music.artist.origin 5421

1842 music.musician.instruments_played 4349

3486 film.person_or_entity_appearing_in_film.film 5906

1772 people.person.place_of_birth 6898

3670 film.actor.film 5719

36 music.producer.tracks_produced 6899

3321 film.person_or_entity_appearing_in_film.film 6900

57 film.person_or_entity_appearing_in_film.film 6901

3433 film.actor.film 6902

1954 film.person_or_entity_appearing_in_film.film 6897

3752 film.actor.film 6903

3026 film.person_or_entity_appearing_in_film.film 4640

810 people.person.place_of_birth 6343

2711 film.person_or_entity_appearing_in_film.film 6904

1814 film.film.star 6905

3475 film.actor.film 6906

2833 film.person_or_entity_appearing_in_film.film 6907

396 film.actor.film 4802

3613 music.artist.origin 6908

2792 film.actor.film 6909

2494 film.person_or_entity_appearing_in_film.film 6160

1747 film.person_or_entity_appearing_in_film.film 6539

2709 film.person_or_entity_appearing_in_film.film 6910

1748 music.artist.origin 6911

67 film.person_or_entity_appearing_in_film.film 6912

67 film.person_or_entity_appearing_in_film.film 6872

2712 film.person_or_entity_appearing_in_film.film 5528

760 people.person.place_of_birth 6913

3040 film.actor.film 4546

407 film.person_or_entity_appearing_in_film.film 4762

987 film.person_or_entity_appearing_in_film.film 6914

802 music.artist.origin 4977

3344 film.person_or_entity_appearing_in_film.film 6915

64 film.person_or_entity_appearing_in_film.film 5953

2236 music.artist.origin 6694

628 film.actor.film 5364

3576 music.artist.origin 3895

1888 film.person_or_entity_appearing_in_film.film 6836

2437 film.person_or_entity_appearing_in_film.film 6916

3185 film.person_or_entity_appearing_in_film.film 6917

1895 film.person_or_entity_appearing_in_film.film 5455

1353 film.actor.film 5249

294 music.artist.origin 6723

2483 film.person_or_entity_appearing_in_film.film 6918

625 film.person_or_entity_appearing_in_film.film 6919

796 film.actor.film 4323

1248 film.person_or_entity_appearing_in_film.film 5959

3248 film.person_or_entity_appearing_in_film.film 6920

1249 film.actor.film 4465

2241 film.distributor.film 6921

2241 film.distributor.film 4300

2241 film.production_company.film 6922

511 music.artist.origin 6923

3297 film.person_or_entity_appearing_in_film.film 5193

2487 film.actor.film 5747

2487 film.person_or_entity_appearing_in_film.film 6924

591 film.actor.film 5473

1148 film.actor.film 5842

2958 music.artist.album 5941

3486 film.person_or_entity_appearing_in_film.film 6925

3486 film.person_or_entity_appearing_in_film.film 5642

2648 film.person_or_entity_appearing_in_film.film 6926

825 music.artist.origin 4739

3779 film.actor.film 6022

1772 film.person_or_entity_appearing_in_film.film 6927

776 film.actor.film 6928

1475 film.person_or_entity_appearing_in_film.film 6929

1357 film.person_or_entity_appearing_in_film.film 6930

253 film.actor.film 4353

1755 music.artist.origin 4453

2843 people.person.place_of_birth 5227

3717 people.deceased_person.place_of_death 4639

1452 music.artist.origin 3864

1191 film.person_or_entity_appearing_in_film.film 6931

1474 film.actor.film 6932

3279 film.producer.film 4682

2711 film.person_or_entity_appearing_in_film.film 5263

327 music.artist.origin 4635

241 film.actor.film 6933

923 film.person_or_entity_appearing_in_film.film 6388

1233 film.person_or_entity_appearing_in_film.film 6934

2181 music.artist.origin 3864

2684 film.person_or_entity_appearing_in_film.film 6935

900 film.actor.film 6936

2635 film.actor.film 6937

2635 film.person_or_entity_appearing_in_film.film 6938

2635 film.producer.film 6939

3307 film.actor.film 6940

1157 film.actor.film 6941

1157 film.person_or_entity_appearing_in_film.film 3954

2222 music.musician.instruments_played 6942

541 film.director.film 4433

304 film.actor.film 6943

633 film.actor.film 4542

1552 music.artist.origin 4057

3404 music.artist.origin 4222

3484 music.producer.tracks_produced 6944

1473 film.person_or_entity_appearing_in_film.film 6945

3737 music.artist.origin 3866

1982 film.actor.film 6363

3364 film.person_or_entity_appearing_in_film.film 6653

3556 music.artist.album 6946

2276 people.person.place_of_birth 6947

1099 music.artist.origin 6948

192 music.producer.tracks_produced 6949

645 film.person_or_entity_appearing_in_film.film 6950

3757 film.actor.film 5762

3426 film.actor.film 4385

1718 music.artist.origin 3942

628 film.actor.film 6951

2790 music.artist.origin 3916

1601 film.actor.film 6952

154 people.person.place_of_birth 5411

2580 music.artist.origin 3961

2070 film.actor.film 6953

79 film.actor.film 6954

3504 film.actor.film 6955

484 film.person_or_entity_appearing_in_film.film 6956

3738 cvg.publisher.games_published 6957

3738 games.publisher.games_published 6958

796 film.actor.film 6959

2149 people.person.place_of_birth 5662

2241 film.distributor.film 6476

2241 film.distributor.film 4684

2241 film.production_company.film 6960

481 film.person_or_entity_appearing_in_film.film 6961

2099 music.artist.origin 6962

387 film.actor.film 4413

1120 music.artist.origin 4391

788 film.actor.film 6963

3049 film.actor.film 6964

57 music.musician.instruments_played 3851

2556 film.person_or_entity_appearing_in_film.film 6087

206 film.actor.film 4648

1709 film.person_or_entity_appearing_in_film.film 5323

997 film.person_or_entity_appearing_in_film.film 6965

2833 film.person_or_entity_appearing_in_film.film 4740

1279 film.person_or_entity_appearing_in_film.film 6966

2820 film.person_or_entity_appearing_in_film.film 6967

3381 film.actor.film 6968

2635 film.actor.film 6969

2789 people.person.place_of_birth 5208

2046 music.artist.origin 6137

3343 music.artist.origin 3846

492 film.actor.film 4710

2094 film.person_or_entity_appearing_in_film.film 4346

3317 film.actor.film 6970

1665 music.artist.origin 3864

633 film.actor.film 6971

2125 film.actor.film 6972

3484 music.artist.track 6944

1026 people.person.place_of_birth 4158

2337 film.actor.film 4662

1706 film.person_or_entity_appearing_in_film.film 6872

145 film.actor.film 6973

145 film.actor.film 4237

2454 film.person_or_entity_appearing_in_film.film 6974

645 film.person_or_entity_appearing_in_film.film 6975

3598 film.actor.film 6976

3129 music.artist.origin 4046

809 film.actor.film 6977

672 film.actor.film 6978

1812 film.actor.film 6979

2636 film.person_or_entity_appearing_in_film.film 5143

445 film.actor.film 6980

445 film.person_or_entity_appearing_in_film.film 5073

1258 people.person.place_of_birth 4673

2483 film.actor.film 4979

625 film.actor.film 6981

2241 film.distributor.film 6982

2241 film.production_company.film 6983

2241 film.production_company.film 6753

2241 film.production_company.film 6984

2241 film.production_company.film 3989

2241 film.production_company.film 5776

1464 film.person_or_entity_appearing_in_film.film 6985

13 music.artist.origin 4454

2487 film.actor.film 6986

2487 film.actor.film 5595

356 music.producer.tracks_produced 6987

1768 film.person_or_entity_appearing_in_film.film 4630

3486 film.person_or_entity_appearing_in_film.film 6988

1806 film.actor.film 6989

1806 film.actor.film 6145

3261 music.artist.origin 6990

3321 film.actor.film 6247

1191 music.artist.origin 4552

3579 film.person_or_entity_appearing_in_film.film 6991

3008 music.album.genre 6992

2211 film.person_or_entity_appearing_in_film.film 4102

1786 film.actor.film 6993

3279 film.actor.film 5094

3214 music.artist.origin 3864

1737 film.actor.film 4160

2449 film.person_or_entity_appearing_in_film.film 5650

1358 film.actor.film 6994

2461 music.artist.origin 4057

396 film.actor.film 6995

102 music.album.track 6996

1726	film.actor.film	5162

2107	film.actor.film	6997

479 music.artist.origin	4108

1157	film.editor.film	6998

818 film.person_or_entity_appearing_in_film.film	6999

1044	award.nominee.award_nominations	7000

3317	people.person.place_of_birth	5105

2939	film.actor.film	7001

2553	music.artist.origin	4103

2712	type.object.subject_key	2712

2359	film.person_or_entity_appearing_in_film.film	7002

2125	film.actor.film	7003

3558	music.artist.track	7004

1692	film.person_or_entity_appearing_in_film.film	7005

1193	film.person_or_entity_appearing_in_film.film	7006

2998	film.actor.film	6165

2482	film.actor.film	5531

1845	film.person_or_entity_appearing_in_film.film	7007

604 music.artist.album	7008

1537	music.artist.origin	3864

2080	people.person.place_of_birth	6012

1131	music.artist.origin	3971

1079	film.editor.film	4669

2491	music.artist.origin	4216

2549	music.artist.origin	5497

1500	film.actor.film	4289

945 music.artist.origin	4261

2273	people.person.place_of_birth	4174

1812	film.person_or_entity_appearing_in_film.film	7009

1601	film.person_or_entity_appearing_in_film.film	4674

974 film.actor.film 7010

2095 film.person_or_entity_appearing_in_film.film 5922

1404 film.actor.film 7011

2062 music.artist.origin 5401

1375 event.agent.performance 4387

1370 film.film.writer 7012

49 film.person_or_entity_appearing_in_film.film 6218

2412 award.nominee.award_nominations 6142

1268 music.artist.origin 4454

3738 cvg.publisher.games_published 6223

3738 cvg.publisher.games_published 7013

3738 games.publisher.games_published 6780

184 film.person_or_entity_appearing_in_film.film 7014

436 film.actor.film 7015

1154 film.actor.film 5589

2049 music.artist.origin 5231

2867 film.person_or_entity_appearing_in_film.film 7016

2241 film.distributor.film 7017

2241 film.production_company.film 5035

2241 film.production_company.film 5182

2241 film.production_company.film 7018

2241 film.production_company.film 7019

1624 film.person_or_entity_appearing_in_film.film 4149

777 film.person_or_entity_appearing_in_film.film 5970

1077 music.artist.origin 4391

1877 film.actor.film 5672

2126 film.actor.film 7020

788 film.person_or_entity_appearing_in_film.film 6006

1806 film.person_or_entity_appearing_in_film.film 4039

2354 music.artist.origin 4635

1357 film.actor.film 7021

1817 film.actor.film 6999

951 film.person_or_entity_appearing_in_film.film 5267

1493 music.artist.origin 4527

1599 people.person.place_of_birth 3846

2373 film.person_or_entity_appearing_in_film.film 7022

3648 film.person_or_entity_appearing_in_film.film 7023

2711 film.actor.film 5185

1657 film.film.language 4076

1074 film.person_or_entity_appearing_in_film.film 7024

1705 people.person.place_of_birth 4658

448 people.person.place_of_birth 4369

3428 film.actor.film 6685

1962 film.person_or_entity_appearing_in_film.film 7025

3633 film.person_or_entity_appearing_in_film.film 7026

2904 film.actor.film 7027

2635 film.person_or_entity_appearing_in_film.film 5237

1157 film.person_or_entity_appearing_in_film.film 5875

818 film.actor.film 5006

1633 film.actor.film 7028

2494 film.actor.film 4315

548 film.person_or_entity_appearing_in_film.film 7029

3827 film.actor.film 7030

1672 music.artist.album 6672

2359 film.actor.film 7031

2147 film.person_or_entity_appearing_in_film.film 7032

1155 music.artist.origin 4927

3656 people.person.place_of_birth 4057

136 film.person_or_entity_appearing_in_film.film 7033

267 music.artist.origin 3864

3784 film.producer.film 4601

1622 music.album.genre 5811

62 people.person.place_of_birth 6425

66 film.person_or_entity_appearing_in_film.film 7034

2762 people.deceased_person.place_of_death 4013

407 film.actor.film 7035

987 film.person_or_entity_appearing_in_film.film 7036

3598 film.person_or_entity_appearing_in_film.film 7037

325 film.person_or_entity_appearing_in_film.film 7038

2679 film.person_or_entity_appearing_in_film.film 5764

3498 film.person_or_entity_appearing_in_film.film 7039

2259 film.person_or_entity_appearing_in_film.film 7040

1778 music.artist.origin 5125

1204 film.actor.film 7041

1211 film.actor.film 6778

1579 film.actor.film 7042

3041 people.person.place_of_birth 7043

484 film.actor.film 5370

2474 music.artist.origin 4376

3738 games.publisher.games_published 7044

3738 games.publisher.games_published 7013

3786 music.artist.origin 4057

615 event.agent.performance 5732

2241 film.production_company.film 7045

2241 film.production_company.film 6378

2241 film.production_company.film 7046

1936 film.person_or_entity_appearing_in_film.film 6611

2779 film.person_or_entity_appearing_in_film.film 7047

3621 music.artist.origin 4174

599 film.person_or_entity_appearing_in_film.film 7048

2126 film.person_or_entity_appearing_in_film.film 7049

1767 film.actor.film 6236

3227 music.artist.origin 3932

3486 film.actor.film 7050

1475 film.person_or_entity_appearing_in_film.film 4152

1278 award.nominee.award_nominations 4797

2184 film.person_or_entity_appearing_in_film.film 6729

3242 film.writer.film 7051

434 film.actor.film 4633

2870 film.actor.film 7052

3321 film.actor.film 7053

3766 music.artist.origin 4376

2858 music.artist.origin 5061

34 film.person_or_entity_appearing_in_film.film 7054

2962 music.artist.origin 4362

3688 film.actor.film 6389

3688 people.person.place_of_birth 5664

1233 film.actor.film 6934

1952 film.person_or_entity_appearing_in_film.film 7055

1280 type.object.subject_key 1280

997 film.person_or_entity_appearing_in_film.film 7056

707 music.artist.origin 7057

2161 award.nominee.award_nominations 7058

1723 music.artist.origin 6555

2939 people.deceased_person.place_of_death 3944

67 film.actor.film 7059

67 people.person.place_of_birth 4050

2210 film.person_or_entity_appearing_in_film.film 6734

2671 people.deceased_person.place_of_death 5270

565 music.artist.origin 5845

37 film.actor.film 7060

3784 film.writer.film 7061

1754 film.actor.film 4314

3845 film.person_or_entity_appearing_in_film.film 7062

386 film.actor.film 7063

386 people.person.place_of_birth 3997

3498 film.person_or_entity_appearing_in_film.film 6251

2035 film.person_or_entity_appearing_in_film.film 7064

80 film.actor.film 7065

3274 people.person.place_of_birth 4058

2562 film.person_or_entity_appearing_in_film.film 7066

2892 film.person_or_entity_appearing_in_film.film 6503

2636 film.person_or_entity_appearing_in_film.film 5027

2612 music.artist.origin 5845

1458 film.actor.film 7067

1467 people.deceased_person.place_of_death 5161

3166 film.person_or_entity_appearing_in_film.film 5079

1180 music.artist.origin 6618

484 film.actor.film 7068

484 film.person_or_entity_appearing_in_film.film 7069

2208 film.actor.film 7070

3738 games.publisher.games_published 7071

436 film.person_or_entity_appearing_in_film.film 7072

2241 film.production_company.film 7073

2241 film.production_company.film 7017

2241 film.production_company.film 4570

2241 film.production_company.film 7074

3340 music.musician.instruments_played 3851

320 music.artist.album 7075

2487 film.actor.film 6924

622 film.person_or_entity_appearing_in_film.film 7076

3197 people.person.place_of_birth 4455

2678 film.person_or_entity_appearing_in_film.film 4741

1357 film.actor.film 7077

1357 film.actor.film 7078

3145 people.person.place_of_birth 7079

2431 music.artist.origin 3992

1395 music.artist.origin 4013

1578 music.artist.origin 3997

1348 film.actor.film 7080

1561 music.artist.origin 6425

721 film.person_or_entity_appearing_in_film.film 5351

2599 film.actor.film 7081

1122 film.actor.film 7082

1279 film.person_or_entity_appearing_in_film.film 7083

842 people.person.place_of_birth 3864

3208 people.person.place_of_birth 4050

82 film.actor.film 7084

286 film.actor.film 7085

2050 film.actor.film 4487

2422 music.artist.origin 4362

618 music.artist.origin 4108

331 music.artist.origin 4852

72 film.actor.film 4980

2210 film.person_or_entity_appearing_in_film.film 7086

2337 film.person_or_entity_appearing_in_film.film 5661

1882 people.person.place_of_birth 5664

789 film.person_or_entity_appearing_in_film.film 7087

3447 film.person_or_entity_appearing_in_film.film 7088

957 event.agent.performance 4898

348 music.artist.origin 4057

2784 film.person_or_entity_appearing_in_film.film 7089

3755 music.artist.origin 3932

1812 film.actor.film 7009

1204 film.person_or_entity_appearing_in_film.film 5921

1063 music.artist.origin 4380

860 music.artist.origin 4852

2636 film.actor.film 7090

1369 music.artist.origin 4232

1037 people.person.place_of_birth 7091

366 film.actor.film 6719

49 film.actor.film 7092

445 film.person_or_entity_appearing_in_film.film 7093

484 film.actor.film 7094

484 film.person_or_entity_appearing_in_film.film 4778

2877 people.person.place_of_birth 5168

1512 music.artist.origin 4183

2241 film.distributor.film 5437

2241 film.distributor.film 4798

2241 film.production_company.film 6443

180 people.person.place_of_birth 7095

1361 film.person_or_entity_appearing_in_film.film 5642

1624 film.actor.film 7096

859 music.artist.origin 4175

894 film.actor.film 6072

643 film.person_or_entity_appearing_in_film.film 4198

3546 film.person_or_entity_appearing_in_film.film 7097

1278 film.actor.film 7098

1295 people.person.place_of_birth 4702

3321 film.actor.film 7099

3180 music.artist.origin 5232

898 film.actor.film 7100

232 film.person_or_entity_appearing_in_film.film 7101

385 film.actor.film 7102

2274 people.person.place_of_birth 7103

3390 film.actor.film 5832

1479 film.person_or_entity_appearing_in_film.film 7104

1750 film.actor.film 7105

206 film.actor.film 6081

383 film.person_or_entity_appearing_in_film.film 4650

383 film.person_or_entity_appearing_in_film.film 7106

1519 people.person.place_of_birth 4010

1087 film.actor.film 7107

1662 people.person.place_of_birth 6425

908 music.artist.origin 4999

2635 film.person_or_entity_appearing_in_film.film 7108

1413 film.person_or_entity_appearing_in_film.film 4593

186 film.actor.film 4110

501 music.artist.origin 4108

3410 music.artist.origin 3864

815 film.actor.film 5355

1444 music.artist.origin 3961

3317 film.actor.film 6205

2939 people.person.place_of_birth 7109

3284 music.artist.origin 4046

1715 people.person.place_of_birth 4419

2359 film.actor.film 7110

1805 people.person.profession 4221

1483 music.artist.origin 4057

2270 film.person_or_entity_appearing_in_film.film 7111

1209	film.person_or_entity_appearing_in_film.film	5532

2567	music.artist.origin	4362

2014	film.film.star	7112

3295	film.person_or_entity_appearing_in_film.film	6096

3344	film.actor.film	7113

1616	music.musician.instruments_played	5802

3498	film.person_or_entity_appearing_in_film.film	4824

1704	film.actor.film	5245

2913	film.person_or_entity_appearing_in_film.film	7114

458 film.actor.film	7115

3533	music.artist.origin	4357

2367	people.person.place_of_birth	4057

780 people.person.place_of_birth	5348

437 film.person_or_entity_appearing_in_film.film	6102

2636	film.person_or_entity_appearing_in_film.film	6172

147 film.actor.film	3918

1172	music.artist.origin	6153

2936	music.artist.origin	5702

278 film.person_or_entity_appearing_in_film.film	5721

455 film.person_or_entity_appearing_in_film.film	7116

1652	music.artist.origin	4272

2241	film.distributor.film	4891

1936	film.person_or_entity_appearing_in_film.film	5548

1767	film.actor.film	7117

3031	film.person_or_entity_appearing_in_film.film	7118

159 film.person_or_entity_appearing_in_film.film	7119

341 film.film.rating	4223

3772	film.film.language	4076

3509	type.object.subject_key	3509

3049	film.person_or_entity_appearing_in_film.film	6481

1623 film.actor.film 7120

232 film.person_or_entity_appearing_in_film.film 7121

2556 film.person_or_entity_appearing_in_film.film 3931

3390 film.actor.film 7122

3048 film.actor.film 7123

2887 music.artist.origin 7124

3688 film.actor.film 4001

3581 people.deceased_person.place_of_death 5181

56 film.actor.film 7125

363 film.person_or_entity_appearing_in_film.film 7126

1259 film.person_or_entity_appearing_in_film.film 6024

1358 film.person_or_entity_appearing_in_film.film 6994

1222 music.artist.origin 4013

2833 film.person_or_entity_appearing_in_film.film 4494

32 music.artist.origin 4453

3599 film.person_or_entity_appearing_in_film.film 7127

2635 film.actor.film 7128

985 film.actor.film 6254

1020 music.artist.origin 4057

3384 film.actor.film 7129

785 event.agent.performance 4035

140 music.artist.origin 3864

2786 film.actor.film 7130

2050 film.person_or_entity_appearing_in_film.film 4976

2125 film.person_or_entity_appearing_in_film.film 6628

1141 film.person_or_entity_appearing_in_film.film 6539

1692 film.person_or_entity_appearing_in_film.film 5358

770 music.artist.origin 6797

2946 music.artist.origin 5061

2966 people.person.place_of_birth 3942

2080 film.actor.film 7131

3598 film.person_or_entity_appearing_in_film.film 7132

2423 film.actor.film 7133

3498 film.person_or_entity_appearing_in_film.film 7134

1176 people.person.place_of_birth 4150

80 music.producer.tracks_produced 7135

2913 film.actor.film 7136

675 film.person_or_entity_appearing_in_film.film 7137

70 film.person_or_entity_appearing_in_film.film 7138

147 film.actor.film 4291

2070 film.actor.film 5423

49 film.actor.film 7139

399 film.person_or_entity_appearing_in_film.film 7140

484 film.actor.film 7141

484 film.person_or_entity_appearing_in_film.film 7068

3738 games.publisher.games_published 4028

436 film.actor.film 7142

2430 film.actor.film 7143

625 film.person_or_entity_appearing_in_film.film 7144

2867 film.actor.film 7145

2867 film.actor.film 4982

2241 film.distributor.film 5633

2241 film.distributor.film 7146

2241 film.distributor.film 7147

2241 film.production_company.film 6310

2241 film.production_company.film 7148

135 film.person_or_entity_appearing_in_film.film 5471

777 film.person_or_entity_appearing_in_film.film 5602

2099 people.person.place_of_birth 6962

2311 film.actor.film 4230

894 film.person_or_entity_appearing_in_film.film 7149

2667 music.artist.origin 7150

955 music.artist.origin 6280

3136 music.artist.origin 4739

1462 people.person.place_of_birth 7151

3796 music.musician.instruments_played 3851

1932 music.artist.origin 5756

1357 film.actor.film 5141

3049 film.actor.film 7152

3321 film.actor.film 3975

2054 people.person.place_of_birth 4232

3773 music.artist.origin 3864

3579 film.actor.film 6320

858 music.artist.origin 4807

2330 music.artist.origin 3849

2711 film.person_or_entity_appearing_in_film.film 7153

24 event.agent.performance 4898

206 film.actor.film 6391

2058 film.person_or_entity_appearing_in_film.film 5310

2107 film.actor.film 7154

2635 film.person_or_entity_appearing_in_film.film 6877

2680 people.person.place_of_birth 3972

3182 film.writer.film 7155

435 film.actor.film 6740

846 film.person_or_entity_appearing_in_film.film 7156

3645 film.actor.film 7157

1473 film.actor.film 7158

1506 film.person_or_entity_appearing_in_film.film 7159

2737 film.actor.film 7160

645 film.person_or_entity_appearing_in_film.film 7161

377 award.competitor.awards_won 7162

1863 film.actor.film 7163

1754 film.person_or_entity_appearing_in_film.film 7164

2080 film.person_or_entity_appearing_in_film.film 7165

1875 music.artist.origin 7166

2940 music.artist.origin 4150

408 film.person_or_entity_appearing_in_film.film 6368

1091 film.person_or_entity_appearing_in_film.film 7167

1846 film.actor.film 5366

2481 film.actor.film 7168

2991 music.artist.origin 4262

1403 film.actor.film 7169

1570 film.person_or_entity_appearing_in_film.film 7170

1211 film.person_or_entity_appearing_in_film.film 5822

2688 film.actor.film 4637

2689 film.person_or_entity_appearing_in_film.film 5821

1725 music.artist.origin 4010

3738 games.publisher.games_published 7171

3673 music.producer.tracks_produced 7172

1249 film.person_or_entity_appearing_in_film.film 3901

2241 film.distributor.film 6032

2241 film.distributor.film 6416

2241 film.distributor.film 6003

2241 film.production_company.film 4030

320 music.artist.origin 4058

1022 film.person_or_entity_appearing_in_film.film 4385

3486 film.person_or_entity_appearing_in_film.film 6821

1806 film.person_or_entity_appearing_in_film.film 6145

1486 cvg.computer_videogame.publisher 7173

3235 music.artist.origin 5231

341 film.film.actor 7174

2894 music.artist.origin 3961

892 film.person_or_entity_appearing_in_film.film 7175

742 film.actor.film 7176

2749 music.artist.origin 6725

3390 film.person_or_entity_appearing_in_film.film 4384

2711 film.actor.film 5519

2711 film.actor.film 7177

3407 music.artist.origin 4391

997 film.person_or_entity_appearing_in_film.film 7178

2771 film.person_or_entity_appearing_in_film.film 5681

422 film.person_or_entity_appearing_in_film.film 5766

3351 film.actor.film 7179

102 music.album.genre 7180

370 music.artist.origin 4224

2107 film.actor.film 7181

2635 film.actor.film 7182

1157 film.actor.film 7183

642 music.artist.origin 4957

379 film.person_or_entity_appearing_in_film.film 4431

540 film.actor.film 5686

143 music.artist.origin 4347

428 film.person_or_entity_appearing_in_film.film 7184

3370 event.agent.performance 4387

67 film.person_or_entity_appearing_in_film.film 6399

1921 film.person_or_entity_appearing_in_film.film 7185

2733 film.person_or_entity_appearing_in_film.film 7186

1473 film.actor.film 6378

1473 film.actor.film 4684

1412 film.actor.film 4015

210 music.artist.origin 4013

2823 people.person.place_of_birth 7187

407 film.person_or_entity_appearing_in_film.film 7188

2080 film.person_or_entity_appearing_in_film.film 5163

1108 music.artist.origin 4775

928 music.artist.album 7189

2913 film.person_or_entity_appearing_in_film.film 4142

1702 film.actor.film 4067

70 film.person_or_entity_appearing_in_film.film 7190

1601 film.actor.film 7191

2636 film.person_or_entity_appearing_in_film.film 7192

144 film.actor.film 7193

3047 film.actor.film 6500

3335 music.artist.origin 3859

1811 music.artist.origin 7194

484 film.actor.film 4928

442 award.competitor.awards_won 7195

3501 film.actor.film 7196

2241 film.distributor.film 7197

2241 film.distributor.film 7198

2241 film.distributor.film 7199

2241 film.distributor.film 7200

2241 film.production_company.film 5706

2189 film.person_or_entity_appearing_in_film.film 7201

607 music.artist.origin 4391

591 music.artist.origin 4455

956 film.actor.film 7202

1957 film.actor.film 4240

1843 music.artist.origin 7203

170 people.person.place_of_birth 6425

1680	music.artist.origin	4815
434	film.person_or_entity_appearing_in_film.film	6421
3002	film.person_or_entity_appearing_in_film.film	6243
3049	film.person_or_entity_appearing_in_film.film	7204
3403	film.person_or_entity_appearing_in_film.film	7205
3812	music.album.genre	7206
2828	music.artist.origin	4058
3715	music.album.genre	6794
2711	film.person_or_entity_appearing_in_film.film	4642
2658	film.person_or_entity_appearing_in_film.film	7207
2599	film.person_or_entity_appearing_in_film.film	7081
1358	film.actor.film	7208
1388	music.artist.origin	4050
959	film.person_or_entity_appearing_in_film.film	7209
32	film.person_or_entity_appearing_in_film.film	4774
202	film.actor.film	5052
2635	film.actor.film	7210
2635	film.actor.film	7211
2986	people.deceased_person.place_of_death	4191
2494	film.person_or_entity_appearing_in_film.film	4215
3808	people.person.place_of_birth	7212
1505	film.person_or_entity_appearing_in_film.film	7213
2734	film.person_or_entity_appearing_in_film.film	7214
2712	film.person_or_entity_appearing_in_film.film	7215
3784	film.producer.film	7061
3733	film.actor.film	7216
645	film.actor.film	7161
3598	film.person_or_entity_appearing_in_film.film	7217
973	music.artist.origin	7218
3387	film.person_or_entity_appearing_in_film.film	4448

2914 film.actor.film 7219

1469 film.actor.film 7220

1469 film.person_or_entity_appearing_in_film.film 7221

1601 film.person_or_entity_appearing_in_film.film 7222

974 film.actor.film 7223

2095 film.person_or_entity_appearing_in_film.film 6305

1423 film.actor.film 6406

399 film.actor.film 7224

294 film.actor.film 7225

3738 games.publisher.games_published 7226

3738 games.publisher.games_published 7227

2853 music.artist.origin 5127

2241 film.distributor.film 4688

2241 film.distributor.film 7228

2241 film.distributor.film 5708

2241 film.production_company.film 7229

2241 film.production_company.film 7230

2241 film.production_company.film 7231

2364 music.artist.origin 5213

777 film.person_or_entity_appearing_in_film.film 7232

622 film.actor.film 4944

644 people.person.place_of_birth 5226

894 film.actor.film 7233

2275 film.actor.film 7234

1660 film.actor.film 7235

3049 film.actor.film 3996

3321 film.person_or_entity_appearing_in_film.film 7236

3433 film.person_or_entity_appearing_in_film.film 7237

720 film.actor.film 7238

2590 music.artist.origin 4963

2449 film.actor.film 7239

2343 film.actor.film 7240

2226 music.artist.origin 3864

1280 film.person_or_entity_appearing_in_film.film 4363

3479 people.deceased_person.place_of_death 4616

148 people.deceased_person.place_of_death 5227

163 music.artist.origin 3992

2818 music.artist.origin 4024

1157 film.actor.film 6121

3384 film.person_or_entity_appearing_in_film.film 7241

115 film.actor.film 7242

115 film.actor.film 7243

2695 music.musician.instruments_played 3959

1720 film.director.film 7244

819 people.person.place_of_birth 4517

3827 film.actor.film 6489

662 film.actor.film 4467

362 film.actor.film 7245

2994 music.artist.origin 4707

1748 film.person_or_entity_appearing_in_film.film 7246

2454 film.writer.film 6974

645 film.actor.film 7247

2080 film.person_or_entity_appearing_in_film.film 7248

2579 music.artist.origin 4010

77 film.actor.film 5736

361 music.producer.tracks_produced 7249

458 award.nominee.award_nominations 7250

2622 people.person.place_of_birth 4050

1465 film.person_or_entity_appearing_in_film.film 6915

2562 film.person_or_entity_appearing_in_film.film 7251

110 music.artist.origin 3946

1453 film.person_or_entity_appearing_in_film.film 7252

603 film.person_or_entity_appearing_in_film.film 7253

1211 type.object.subject_key 1211

2689 award.winner.awards_won 6699

1299 people.person.place_of_birth 4445

366 film.actor.film 7254

3738 cvg.publisher.games_published 6664

3738 games.publisher.games_published 7255

1154 film.person_or_entity_appearing_in_film.film 7256

1154 film.person_or_entity_appearing_in_film.film 4614

3248 film.actor.film 6920

2159 film.actor.film 4466

2867 film.actor.film 4397

2360 film.person_or_entity_appearing_in_film.film 7257

2241 film.distributor.film 4686

2241 film.distributor.film 5931

2241 film.production_company.film 7258

2241 film.production_company.film 7259

2241 film.production_company.film 7260

2241 film.production_company.film 7261

2779 film.actor.film 4842

682 film.actor.film 4092

2838 music.artist.origin 4217

2690 people.person.place_of_birth 7262

261 music.artist.track 5713

2200 music.musician.instruments_played 5367

643 film.person_or_entity_appearing_in_film.film 6480

3607 film.actor.film 5782

2862 people.person.place_of_birth 3947

1475 film.person_or_entity_appearing_in_film.film 7263

538 film.actor.film 7264

1817 film.person_or_entity_appearing_in_film.film 6999

1202 film.actor.film 7265

2583 music.artist.origin 4183

898 film.person_or_entity_appearing_in_film.film 7266

1158 music.artist.origin 5873

2715 film.person_or_entity_appearing_in_film.film 6286

2711 film.person_or_entity_appearing_in_film.film 7267

1816 film.actor.film 7268

3132 film.actor.film 7269

3727 music.artist.origin 4195

206 film.person_or_entity_appearing_in_film.film 7270

1985 film.person_or_entity_appearing_in_film.film 7271

2058 film.actor.film 5310

2635 film.actor.film 7272

2635 film.actor.film 5505

2808 people.person.place_of_birth 4094

1157 film.person_or_entity_appearing_in_film.film 5755

379 film.person_or_entity_appearing_in_film.film 7273

2633 music.artist.origin 4272

492 film.person_or_entity_appearing_in_film.film 7274

1998 people.person.place_of_birth 4150

1748 film.actor.film 7275

2139 music.artist.origin 4183

3788 film.person_or_entity_appearing_in_film.film 7276

67 film.actor.film 5485

67 film.person_or_entity_appearing_in_film.film 5068

2911 people.person.profession 4221

3133 film.actor.film 7277

1141 award.winner.awards_won 6859

3364 film.actor.film 7278

136 film.person_or_entity_appearing_in_film.film 6160

3215 film.film.production_company 2241

127 film.actor.film 7279

150 film.actor.film 7193

809 film.actor.film 5626

497 music.artist.origin 3916

458 people.person.profession 4221

646 film.person_or_entity_appearing_in_film.film 5025

70 film.person_or_entity_appearing_in_film.film 4235

437 film.person_or_entity_appearing_in_film.film 7280

2070 film.person_or_entity_appearing_in_film.film 7281

2122 film.actor.film 7282

1895 film.person_or_entity_appearing_in_film.film 7283

2180 music.artist.origin 3864

484 film.person_or_entity_appearing_in_film.film 3988

3738 cvg.publisher.games_published 7284

219 music.artist.origin 7285

2176 music.artist.origin 4635

1359 film.person_or_entity_appearing_in_film.film 7286

927 people.person.place_of_birth 3859

2241 film.distributor.film 4248

2241 film.distributor.film 7287

2241 film.production_company.film 7288

1936 film.person_or_entity_appearing_in_film.film 7289

481 film.actor.film 7290

1464 film.actor.film 7291

1618 film.actor.film 5532

894 film.actor.film 6847

3532	music.artist.origin	3864

1644	music.artist.origin	3864

3198	film.person_or_entity_appearing_in_film.film	7292

2525	people.person.place_of_birth	4272

3392	music.artist.origin	3945

1729	film.actor.film	4632

2861	film.person_or_entity_appearing_in_film.film	7293

2207	film.person_or_entity_appearing_in_film.film	5483

3509	music.artist.track	6996

3049	film.person_or_entity_appearing_in_film.film	7294

28	people.person.place_of_birth	4416

3742	film.person_or_entity_appearing_in_film.film	7295

3429	music.artist.origin	3961

2372	film.person_or_entity_appearing_in_film.film	7296

2715	film.person_or_entity_appearing_in_film.film	4263

3390	film.person_or_entity_appearing_in_film.film	7297

1786	film.person_or_entity_appearing_in_film.film	6993

1546	people.person.place_of_birth	5945

2711	film.person_or_entity_appearing_in_film.film	7298

1816	film.actor.film	7299

1632	film.actor.film	5928

1322	people.person.place_of_birth	4191

601	film.actor.film	7300

363	people.person.place_of_birth	4133

148	music.artist.origin	5227

735	music.musician.instruments_played	3851

2635	film.person_or_entity_appearing_in_film.film	6120

2635	film.person_or_entity_appearing_in_film.film	7301

1633	film.actor.film	5688

2183	music.artist.origin	6832

3477	people.person.place_of_birth	6056

1747	film.actor.film	6539

3483	music.artist.origin	4058

634	music.artist.origin	3942

1617	people.person.place_of_birth	7302

633	film.person_or_entity_appearing_in_film.film	7303

774	film.person_or_entity_appearing_in_film.film	7304

2359	film.person_or_entity_appearing_in_film.film	7305

2337	film.person_or_entity_appearing_in_film.film	7306

895	film.actor.film	7307

1959	film.actor.film	7308

2018	film.actor.film	4792

3359	music.artist.origin	7309

2080	film.person_or_entity_appearing_in_film.film	4672

2214	music.producer.tracks_produced	7310

393	film.person_or_entity_appearing_in_film.film	7311

672	film.actor.film	7312

672	film.person_or_entity_appearing_in_film.film	4767

357	film.person_or_entity_appearing_in_film.film	7313

628	film.person_or_entity_appearing_in_film.film	6951

3120	film.person_or_entity_appearing_in_film.film	7314

70	film.actor.film	7315

2095	film.actor.film	7316

445	film.person_or_entity_appearing_in_film.film	6980

294	film.actor.film	7317

484	film.person_or_entity_appearing_in_film.film	7318

3738	cvg.publisher.games_published	6663

3738	cvg.publisher.games_published	7171

3738	cvg.publisher.games_published	7319

3738	games.publisher.games_published	7320

3738 games.publisher.games_published 5327

2430 film.person_or_entity_appearing_in_film.film 4646

1002 people.person.place_of_birth 7321

188 music.artist.origin 5064

3400 film.actor.film 5997

2241 film.distributor.film 5707

1936 film.person_or_entity_appearing_in_film.film 7322

932 film.actor.film 7323

3297 film.person_or_entity_appearing_in_film.film 6858

998 film.actor.film 7324

1767 film.actor.film 7325

1583 people.person.place_of_birth 7326

2024 film.actor.film 7327

3772 film.film.rating 7328

2870 film.person_or_entity_appearing_in_film.film 7329

3027 film.person_or_entity_appearing_in_film.film 7330

892 film.person_or_entity_appearing_in_film.film 7331

3390 film.actor.film 4101

3390 film.person_or_entity_appearing_in_film.film 7332

1057 people.person.place_of_birth 7333

1868 music.artist.origin 5228

206 film.person_or_entity_appearing_in_film.film 7334

169 music.artist.origin 4676

2771 film.actor.film 7335

3599 film.person_or_entity_appearing_in_film.film 7336

2635 film.person_or_entity_appearing_in_film.film 7337

464 film.writer.film 5654

1157 film.actor.film 7338

1157 film.actor.film 4592

167 music.artist.origin 4896

2621	film.actor.film	3955

1889	film.actor.film	7339

155 film.person_or_entity_appearing_in_film.film	7340

1153	film.actor.film	4659

2842	music.artist.origin	7341

1838	people.person.place_of_birth	3947

1497	music.artist.origin	5127

1574	music.artist.origin	4008

3741	film.person_or_entity_appearing_in_film.film	7342

2726	film.person_or_entity_appearing_in_film.film	7343

672 film.person_or_entity_appearing_in_film.film	6833

1702	film.person_or_entity_appearing_in_film.film	4846

1469	film.person_or_entity_appearing_in_film.film	7344

2674	people.person.place_of_birth	4217

2650	film.film.director6812

3289	film.person_or_entity_appearing_in_film.film	5749

2689	film.actor.film	5821

1145	music.musician.instruments_played	4349

2460	film.person_or_entity_appearing_in_film.film	7345

3349	film.actor.film	5771

278 film.person_or_entity_appearing_in_film.film	7346

2192	music.artist.origin	5061

484 film.person_or_entity_appearing_in_film.film	4615

3738	games.publisher.games_published 7347

2400	film.person_or_entity_appearing_in_film.film	7014

901 film.actor.film	6512

69	film.person_or_entity_appearing_in_film.film	7348

2360	film.person_or_entity_appearing_in_film.film	7349

2241	film.distributor.film	6332

2241	film.distributor.film	7350

2241 film.distributor.film 5744

2241 film.distributor.film 7351

2241 film.production_company.film 7352

2241 film.production_company.film 7353

795 film.actor.film 4846

356 award.competitor.award_nominations 7354

418 music.artist.track 7355

371 film.person_or_entity_appearing_in_film.film 5090

1357 film.person_or_entity_appearing_in_film.film 7077

538 film.actor.film 7356

1615 music.artist.origin 4453

3458 people.person.place_of_birth 4094

232 film.actor.film 7101

1191 film.actor.film 6520

329 music.artist.origin 3864

1546 music.artist.origin 5521

1816 film.actor.film 4855

3132 film.person_or_entity_appearing_in_film.film 7357

3457 music.artist.origin 4094

2258 people.person.place_of_birth 7358

2807 people.person.place_of_birth 7359

2684 music.artist.album 7360

567 film.person_or_entity_appearing_in_film.film 6048

1157 film.person_or_entity_appearing_in_film.film 7338

2091 people.person.place_of_birth 7361

1720 film.producer.film 7362

3111 film.person_or_entity_appearing_in_film.film 6764

815 film.actor.film 7363

1826 film.person_or_entity_appearing_in_film.film 5990

433 music.artist.origin 4150

1383	film.actor.film	7364

2990	film.person_or_entity_appearing_in_film.film	7365

2944	film.actor.film	7366

3215	film.film.genre	6189

1870	music.artist.origin	5521

1029	music.artist.origin	4063

620 people.person.profession	7367

2265	film.actor.film	5212

377 film.person_or_entity_appearing_in_film.film	6695

84	film.actor.film	7368

1296	film.person_or_entity_appearing_in_film.film	7369

789 music.artist.origin	7370

3109	film.actor.film	3908

3250	film.person_or_entity_appearing_in_film.film	6367

1876	music.artist.origin	3864

672 film.actor.film	7371

2848	film.film.actor	6065

1544	music.artist.origin	4046

1601	film.actor.film	7372

3561	film.actor.film	7373

437 film.person_or_entity_appearing_in_film.film	7374

2437	film.actor.film	7375

679 film.person_or_entity_appearing_in_film.film	7376

2740	music.artist.origin	3866

2070	film.person_or_entity_appearing_in_film.film	7377

2688	film.person_or_entity_appearing_in_film.film	7378

3289	event.agent.performance	4387

79	film.person_or_entity_appearing_in_film.film	7379

79	film.person_or_entity_appearing_in_film.film	5173

272 music.artist.origin	4087

527 film.person_or_entity_appearing_in_film.film 7380

3511 music.musician.instruments_played 7381

3738 cvg.publisher.games_published 7227

3738 cvg.publisher.games_published 4884

3738 games.publisher.games_published 4027

3738 games.publisher.games_published 7382

901 film.actor.film 6180

625 film.actor.film 7383

1040 film.person_or_entity_appearing_in_film.film 7384

2241 film.production_company.film 7385

2241 film.production_company.film 6754

3354 award.competitor.award_nominations 7386

1278 people.person.place_of_birth 3846

3242 film.actor.film 7387

1580 film.actor.film 7388

3291 people.person.place_of_birth 6323

3509 film.person_or_entity_appearing_in_film.film 7389

1884 film.person_or_entity_appearing_in_film.film 7390

3226 film.actor.film 7391

892 film.person_or_entity_appearing_in_film.film 7392

385 film.actor.film 6152

3648 film.actor.film 7393

380 film.person_or_entity_appearing_in_film.film 7394

1816 film.person_or_entity_appearing_in_film.film 4643

683 music.artist.origin 4347

129 film.person_or_entity_appearing_in_film.film 5787

959 film.actor.film 7395

2635 film.actor.film 7396

2635 film.actor.film 7397

3464 music.artist.origin 5662

3114 film.actor.film 7398

480 music.artist.origin 7399

1157 film.person_or_entity_appearing_in_film.film 7400

953 music.album.artist 7401

1720 film.director.film 4273

1720 film.producer.film 7244

2432 music.artist.origin 4191

674 film.actor.film 7402

2359 film.actor.film 7403

183 film.actor.film 7404

1473 film.person_or_entity_appearing_in_film.film 7405

2292 film.actor.film 7406

1590 people.person.place_of_birth 7407

3656 music.artist.origin 4357

1269 music.artist.origin 3961

3595 music.artist.origin 4108

2324 film.film.language 4076

2482 film.actor.film 6602

3040 music.artist.album 7408

279 film.person_or_entity_appearing_in_film.film 4124

377 type.object.key 377

2080 film.actor.film 7409

177 film.person_or_entity_appearing_in_film.film 7410

2914 film.actor.film 7411

646 film.actor.film 7412

2562 film.person_or_entity_appearing_in_film.film 6026

2636 film.person_or_entity_appearing_in_film.film 7413

147 film.actor.film 7414

49 film.actor.film 7415

79 film.person_or_entity_appearing_in_film.film 4560

3738	cvg.publisher.games_published	5463
3738	games.publisher.games_published	4832
1154	people.person.place_of_birth	4050
2400	film.actor.film	7416
2342	music.artist.origin	7417
1821	film.person_or_entity_appearing_in_film.film	6106
2747	music.artist.album	7418
2241	film.distributor.film	4986
2241	film.distributor.film	7419
2241	film.distributor.film	7420
2241	film.production_company.film	4983
2241	film.production_company.film	6474
2241	film.production_company.film	7421
3430	people.person.place_of_birth	4191
2779	film.actor.film	7047
3098	people.person.place_of_birth	7422
777	film.person_or_entity_appearing_in_film.film	7423
816	people.deceased_person.place_of_death	5227
2126	film.actor.film	7424
2126	film.person_or_entity_appearing_in_film.film	7424
3486	film.person_or_entity_appearing_in_film.film	7425
558	music.artist.origin	4454
2810	type.object.key	2810
57	film.actor.film	4476
2945	film.actor.film	7426
3390	film.actor.film	7427
1345	music.musician.instruments_played	3959
854	music.artist.origin	4003
638	film.person_or_entity_appearing_in_film.film	7428
2635	film.actor.film	4647

2635 film.person_or_entity_appearing_in_film.film 7429

2845 people.person.place_of_birth 4094

115 film.actor.film 7430

2161 award.nominee.award_nominations 7431

1720 film.director.film 7362

3106 film.actor.film 7432

1156 music.artist.origin 7433

1010 music.artist.origin 4391

3317 film.person_or_entity_appearing_in_film.film 6970

1802 film.actor.film 5638

3788 film.person_or_entity_appearing_in_film.film 5408

2734 film.actor.film 6857

1473 film.actor.film 5982

2944 film.person_or_entity_appearing_in_film.film 5210

2324 film.film.director 5574

1740 music.artist.origin 3916

127 film.person_or_entity_appearing_in_film.film 4664

413 film.actor.film 7434

3833 people.deceased_person.place_of_death 4174

3295 film.actor.film 7435

1754 film.actor.film 7164

2080 film.actor.film 7436

3598 film.actor.film 6572

3396 film.actor.film 7437

3121 film.person_or_entity_appearing_in_film.film 6427

974 film.person_or_entity_appearing_in_film.film 4130

2095 film.actor.film 4611

2095 film.person_or_entity_appearing_in_film.film 7438

128 film.actor.film 7439

2437 film.actor.film 6916

2783	people.deceased_person.place_of_death	4362

49	film.person_or_entity_appearing_in_film.film	7440

79	film.person_or_entity_appearing_in_film.film	5146

270 film.person_or_entity_appearing_in_film.film	6371

1315	film.actor.film	6266

782 film.person_or_entity_appearing_in_film.film	6006

436 film.person_or_entity_appearing_in_film.film	7441

901 film.person_or_entity_appearing_in_film.film	7442

2283	music.artist.origin	7187

1359	film.person_or_entity_appearing_in_film.film	6784

1002	film.actor.film	7443

796 film.person_or_entity_appearing_in_film.film	3977

2867	film.person_or_entity_appearing_in_film.film	5293

2241	film.production_company.film	7444

2241	film.production_company.film	4245

2241	film.production_company.film	7445

2241	film.production_company.film	4787

2241	film.production_company.film	7446

2241	film.production_company.film	4144

2241	film.production_company.film	7447

1936	film.actor.film	7448

1624	film.actor.film	6661

2487	film.actor.film	5301

2206	film.actor.film	7449

1424	music.artist.origin	5627

1475	film.actor.film	6647

1475	people.person.place_of_birth	3864

1278	award.nominee.award_nominations	7450

1357	film.actor.film	7451

673 film.actor.film	5867

1660	film.actor.film	4073

3049	film.actor.film	6615

1046	film.person_or_entity_appearing_in_film.film	7452

1468	film.actor.film	7453

460 film.actor.film	7454

3279	film.person_or_entity_appearing_in_film.film	7455

1816	film.actor.film	7456

1348	film.person_or_entity_appearing_in_film.film	7457

1999	music.artist.origin	3961

3428	film.actor.film	7458

1057	film.person_or_entity_appearing_in_film.film	4856

496 music.artist.origin	4825

1122	film.person_or_entity_appearing_in_film.film	7082

3381	film.actor.film	7459

115 film.person_or_entity_appearing_in_film.film	7460

560 music.artist.origin	3972

1153	film.actor.film	5795

847 film.actor.film	4713

3816	music.artist.origin	4195

2359	film.person_or_entity_appearing_in_film.film	7461

1476	music.artist.origin	3897

2370	film.actor.film	5448

1412	music.artist.origin	6435

3288	music.artist.origin	4057

2737	people.person.place_of_birth	6797

1209	film.actor.film	7462

645 film.person_or_entity_appearing_in_film.film	7463

976 film.actor.film	7464

3121	event.agent.performance	4035

1851	people.person.place_of_birth	4150

1407	music.artist.origin	4693

3498	film.actor.film	6251

1212	music.artist.origin	4362

151 film.actor.film	7465

437 film.actor.film	7280

437 film.person_or_entity_appearing_in_film.film	7466

248 music.artist.origin	6262

2968	film.person_or_entity_appearing_in_film.film	7467

3738	cvg.publisher.games_published	4509

3738	cvg.publisher.games_published	5898

184 film.actor.film	7416

2199	music.artist.origin	3994

3141	music.artist.origin	4057

2996	music.artist.origin	4362

41	film.actor.film	7468

2577	music.musician.instruments_played	3851

2241	film.distributor.film	6896

2241	film.distributor.film	7353

2241	film.distributor.film	7469

2241	film.distributor.film	7470

2241	film.production_company.film	5082

3596	music.artist.album	7471

1464	film.actor.film	7472

1022	music.artist.origin	3866

894 film.actor.film	7473

643 film.actor.film	7474

2884	film.person_or_entity_appearing_in_film.film	7475

3380	music.artist.origin	4024

3546	film.actor.film	7476

3002	film.person_or_entity_appearing_in_film.film	6075

2118	music.artist.origin	7477

3049	film.person_or_entity_appearing_in_film.film	7478

3321	film.person_or_entity_appearing_in_film.film	6732

3321	film.person_or_entity_appearing_in_film.film	5306

1919	music.artist.origin	7479

3231	film.actor.film	6069

2153	music.artist.origin	3870

3052	film.actor.film	7480

2148	film.person_or_entity_appearing_in_film.film	6795

3474	film.person_or_entity_appearing_in_film.film	7481

1786	film.actor.film	4043

3048	film.actor.film	7482

1814	film.film.actor	7483

1124	music.artist.origin	4013

1358	film.actor.film	7484

1358	people.deceased_person.place_of_death	4058

997 film.person_or_entity_appearing_in_film.film	7485

2635	film.actor.film	7486

115 film.person_or_entity_appearing_in_film.film	7487

623 film.person_or_entity_appearing_in_film.film	6802

815 people.deceased_person.place_of_death	5227

1649	music.artist.origin	3946

1153	film.person_or_entity_appearing_in_film.film	4320

72	music.artist.origin	3864

1473	film.person_or_entity_appearing_in_film.film	7488

1225	music.artist.origin	6735

3521	music.artist.origin	7489

2603	film.person_or_entity_appearing_in_film.film	7490

1960	film.person_or_entity_appearing_in_film.film	4867

407 film.actor.film	7491

2217	people.person.place_of_birth	7492

64	film.person_or_entity_appearing_in_film.film	4915

809	film.person_or_entity_appearing_in_film.film	4284

3313	film.person_or_entity_appearing_in_film.film	5786

2636	film.actor.film	7493

2800	people.person.place_of_birth	4094

3738	cvg.publisher.games_published	6724

436	film.actor.film	6582

2293	film.person_or_entity_appearing_in_film.film	4462

1312	people.person.place_of_birth	4199

2867	film.actor.film	6107

2241	film.distributor.film	4626

2241	film.distributor.film	6818

2241	film.production_company.film	7494

681	film.person_or_entity_appearing_in_film.film	6670

1618	music.artist.album	4367

3706	film.actor.film	6897

214	music.artist.origin	3946

865	film.person_or_entity_appearing_in_film.film	7495

1357	film.actor.film	7496

3000	film.actor.film	7497

291	event.agent.performance	4729

3187	people.person.place_of_birth	5662

3099	people.person.place_of_birth	4063

1	music.artist.origin	7498

3650	film.person_or_entity_appearing_in_film.film	5093

1474	film.person_or_entity_appearing_in_film.film	5349

380	film.actor.film	7499

2956	tv.series_episode.writer	7500

2711	film.actor.film	7501

3048 film.actor.film 5774

1657 film.film.actor 7502

2010 film.actor.film 7503

3508 music.artist.origin 5258

566 music.artist.origin 4714

449 music.artist.origin 4454

383 film.actor.film 7106

3613 people.person.place_of_birth 6908

2635 film.actor.film 7504

2635 film.actor.film 7505

3592 people.deceased_person.place_of_death 4057

1153 film.person_or_entity_appearing_in_film.film 7506

3182 film.person_or_entity_appearing_in_film.film 7155

67 film.actor.film 7507

136 film.actor.film 6160

977 film.actor.film 7508

279 film.actor.film 5885

987 film.person_or_entity_appearing_in_film.film 4497

3598 film.actor.film 7509

1598 film.actor.film 5885

3498 film.actor.film 7510

2913 film.person_or_entity_appearing_in_film.film 7511

2913 film.person_or_entity_appearing_in_film.film 7512

2913 film.person_or_entity_appearing_in_film.film 7513

1661 film.actor.film 4607

2710 film.person_or_entity_appearing_in_film.film 7514

1812 people.deceased_person.place_of_death 7515

1403 people.person.place_of_birth 7516

2164 people.person.place_of_birth 5161

3366 film.person_or_entity_appearing_in_film.film 7517

3738 games.publisher.games_published 7518

436 film.actor.film 7072

436 film.actor.film 5330

1154 film.person_or_entity_appearing_in_film.film 7519

2241 film.distributor.film 7118

2241 film.production_company.film 7520

1464 film.person_or_entity_appearing_in_film.film 7521

1624 film.actor.film 4846

1995 film.person_or_entity_appearing_in_film.film 7522

1461 people.deceased_person.place_of_death 3972

1462 film.person_or_entity_appearing_in_film.film 7523

3744 music.artist.origin 4456

1475 film.person_or_entity_appearing_in_film.film 7524

1695 film.person_or_entity_appearing_in_film.film 7525

1496 film.actor.film 4124

2379 people.person.place_of_birth 6185

515 music.artist.origin 3961

1310 film.actor.film 7526

1310 music.artist.album 5080

740 film.actor.film 7527

892 film.actor.film 7392

892 film.person_or_entity_appearing_in_film.film 7528

2373 film.person_or_entity_appearing_in_film.film 7529

421 music.artist.origin 7530

3132 people.person.place_of_birth 7531

997 film.actor.film 7532

1279 film.actor.film 7083

567 film.person_or_entity_appearing_in_film.film 6324

1157 film.producer.film 4052

2647 film.person_or_entity_appearing_in_film.film 5848

280 film.actor.film 7533

785 film.actor.film 7534

785 film.actor.film 5526

2353 music.artist.origin 4046

3020 film.person_or_entity_appearing_in_film.film 4164

2613 music.artist.origin 4063

2422 film.actor.film 5312

1488 event.agent.performance 4548

2712 type.object.key 2712

3603 film.actor.film 6022

1449 people.person.place_of_birth 6276

203 people.person.place_of_birth 4150

2151 music.artist.origin 4216

2337 film.person_or_entity_appearing_in_film.film 7535

3204 people.person.place_of_birth 7095

2029 film.person_or_entity_appearing_in_film.film 7536

557 film.person_or_entity_appearing_in_film.film 6654

2914 film.actor.film 7537

646 film.actor.film 5418

2873 people.person.place_of_birth 4445

437 film.person_or_entity_appearing_in_film.film 5955

308 music.artist.origin 3897

2636 film.actor.film 7538

2636 film.actor.film 7539

763 music.artist.origin 4003

3745 film.actor.film 7540

1730 music.artist.origin 7541

2689 type.object.subject_key 2689

519 film.actor.film 4328

2164 film.actor.film 7542

1353 film.person_or_entity_appearing_in_film.film 7543

484 film.actor.film 3988

3738 games.publisher.games_published 7544

3408 people.person.place_of_birth 5019

500 music.artist.album 7545

2241 film.distributor.film 6381

2241 film.distributor.film 7546

451 music.artist.origin 6249

2495 music.musician.instruments_played 4349

1624 film.person_or_entity_appearing_in_film.film 6661

2487 film.actor.film 7547

1317 people.person.place_of_birth 6185

894 film.person_or_entity_appearing_in_film.film 7548

894 film.person_or_entity_appearing_in_film.film 7549

1656 people.person.profession 5021

2951 film.actor.film 7550

3486 film.person_or_entity_appearing_in_film.film 6674

1927 music.musician.instruments_played 3851

1093 music.artist.origin 4108

1992 music.artist.origin 7551

3321 film.person_or_entity_appearing_in_film.film 7552

3718 music.artist.origin 4639

701 film.person_or_entity_appearing_in_film.film 7553

3806 music.artist.origin 7554

1816 film.writer.film 4855

1962 film.person_or_entity_appearing_in_film.film 7555

1057 film.person_or_entity_appearing_in_film.film 7556

1627 music.artist.origin 4195

2599 film.person_or_entity_appearing_in_film.film 7557

1978 music.artist.origin 7558

2632 music.artist.origin 5125

313 film.person_or_entity_appearing_in_film.film 4595

369 music.artist.origin 4000

3769 music.artist.origin 4362

805 music.artist.origin 4057

2079 film.actor.film 5764

2301 music.artist.origin 3966

2911 people.person.place_of_birth 5662

1473 film.person_or_entity_appearing_in_film.film 5157

353 film.actor.film 7559

2750 film.film.language 4076

2944 film.person_or_entity_appearing_in_film.film 7560

3302 people.person.place_of_birth 6137

279 film.actor.film 7561

3733 film.person_or_entity_appearing_in_film.film 7562

987 film.person_or_entity_appearing_in_film.film 6809

1079 people.person.place_of_birth 3846

767 film.actor.film 7563

2637 film.film.genre 4410

80 music.producer.tracks_produced 7564

3318 people.person.place_of_birth 4063

3676 film.actor.film 4233

1744 film.person_or_entity_appearing_in_film.film 6471

70 film.person_or_entity_appearing_in_film.film 6861

437 film.person_or_entity_appearing_in_film.film 5065

2743 film.person_or_entity_appearing_in_film.film 7565

2070 film.person_or_entity_appearing_in_film.film 7566

505 music.artist.origin 3849

3179 film.actor.film 5739

1370 film.film.producer 7012

49 film.actor.film 7567

2208 film.person_or_entity_appearing_in_film.film 7070

3738 cvg.publisher.games_published 7568

1154 film.person_or_entity_appearing_in_film.film 7569

3219 people.person.place_of_birth 4003

3196 music.artist.origin 4050

438 film.person_or_entity_appearing_in_film.film 7570

432 type.object.key 432

2241 film.distributor.film 7571

2241 film.production_company.film 7572

135 film.actor.film 4301

783 people.person.place_of_birth 4362

549 film.person_or_entity_appearing_in_film.film 7573

2126 film.person_or_entity_appearing_in_film.film 7574

387 film.actor.film 6038

397 people.person.place_of_birth 5213

409 music.artist.origin 4673

1567 film.person_or_entity_appearing_in_film.film 6872

643 film.person_or_entity_appearing_in_film.film 4847

2731 film.actor.film 4151

2643 film.person_or_entity_appearing_in_film.film 7575

1918 music.artist.origin 6019

2100 film.actor.film 4744

2531 film.actor.film 7576

1496 music.artist.origin 6249

423 music.artist.origin 4050

2051 film.actor.film 3999

287 people.person.place_of_birth 3944

1294 music.artist.origin 4977

2584 film.person_or_entity_appearing_in_film.film 7577

3154 music.artist.origin 4391

1479 film.actor.film 5947

3660 people.person.place_of_birth 5403

3446 film.person_or_entity_appearing_in_film.film 7578

3251 film.person_or_entity_appearing_in_film.film 7579

1941 people.person.place_of_birth 4552

3543 people.person.place_of_birth 5270

394 music.artist.origin 3946

2635 film.person_or_entity_appearing_in_film.film 7580

2635 film.person_or_entity_appearing_in_film.film 7581

1157 film.actor.film 6854

3384 film.person_or_entity_appearing_in_film.film 7129

2905 music.artist.origin 3864

1748 film.person_or_entity_appearing_in_film.film 7582

2708 film.actor.film 6206

3788 film.actor.film 6511

774 film.actor.film 6332

2125 film.person_or_entity_appearing_in_film.film 4491

136 film.person_or_entity_appearing_in_film.film 7583

3784 film.producer.film 3904

91 film.actor.film 4379

71 film.person_or_entity_appearing_in_film.film 6087

2341 music.artist.origin 4347

2488 music.artist.origin 6564

2080 film.person_or_entity_appearing_in_film.film 6097

789 film.actor.film 7584

1079 film.writer.film 7585

809 film.person_or_entity_appearing_in_film.film 7586

675 film.actor.film 7587

3015 music.artist.origin 7588

1469 film.actor.film 7589

1453 film.person_or_entity_appearing_in_film.film 5848

437 film.actor.film 6811

2636 film.actor.film 4023

2636 film.actor.film 7590

2636 film.person_or_entity_appearing_in_film.film 4872

2535 film.person_or_entity_appearing_in_film.film 7591

1688 music.artist.origin 5213

1211 event.agent.performance 4729

1355 film.person_or_entity_appearing_in_film.film 7592

1355 type.object.key 1355

2460 film.actor.film 5740

2460 film.person_or_entity_appearing_in_film.film 5457

79 film.actor.film 5146

716 music.artist.origin 4422

3738 games.publisher.games_published 7593

625 film.person_or_entity_appearing_in_film.film 7594

1249 film.person_or_entity_appearing_in_film.film 7595

1071 music.artist.origin 4010

2631 music.artist.origin 3972

2241 film.distributor.film 7596

2241 film.production_company.film 7597

2241 film.production_company.film 7598

1464 film.actor.film 7599

3139 film.actor.film 5117

2722 music.artist.origin 6323

2897 film.actor.film 7600

591 film.person_or_entity_appearing_in_film.film 7601

2005 music.artist.origin 3944

488 music.artist.origin 4416

1583	film.person_or_entity_appearing_in_film.film	7602

956	film.person_or_entity_appearing_in_film.film	5550

2275	film.person_or_entity_appearing_in_film.film	7234

2677	music.artist.album	4122

2707	film.person_or_entity_appearing_in_film.film	3939

3546	film.person_or_entity_appearing_in_film.film	7476

1357	film.actor.film	4520

3321	film.person_or_entity_appearing_in_film.film	7603

157	music.artist.track	7604

3231	film.person_or_entity_appearing_in_film.film	6069

3027	film.actor.film	7330

748	music.artist.origin	4768

385	film.person_or_entity_appearing_in_film.film	6483

2089	event.agent.performance	4387

2402	film.person_or_entity_appearing_in_film.film	7605

379	film.person_or_entity_appearing_in_film.film	7606

143	music.artist.origin	4157

3020	film.person_or_entity_appearing_in_film.film	7607

2953	film.person_or_entity_appearing_in_film.film	7608

2438	music.artist.origin	3849

67	film.actor.film	4915

2384	music.artist.origin	4442

1209	film.person_or_entity_appearing_in_film.film	6433

645	film.person_or_entity_appearing_in_film.film	7609

1035	film.actor.film	7610

3719	music.artist.origin	3966

70	film.actor.film	4324

70	film.person_or_entity_appearing_in_film.film	6027

2095	film.person_or_entity_appearing_in_film.film	7611

1559	film.actor.film	5694

2425 music.artist.origin 4050

1366 music.artist.origin 6555

2128 music.artist.origin 4010

3179 film.actor.film 7612

133 award.winner.awards_won 7613

270 film.actor.film 7614

1531 people.deceased_person.place_of_death 7615

436 film.person_or_entity_appearing_in_film.film 5741

1002 film.person_or_entity_appearing_in_film.film 7443

1940 event.agent.performance 5101

2360 film.person_or_entity_appearing_in_film.film 7616

2241 film.distributor.film 7617

2241 film.distributor.film 7618

2241 film.distributor.film 6583

2241 film.distributor.film 7619

2241 film.distributor.film 7620

2241 film.production_company.film 5595

1464 film.actor.film 7621

382 film.person_or_entity_appearing_in_film.film 7622

2126 film.actor.film 7623

719 music.artist.origin 7624

2731 film.person_or_entity_appearing_in_film.film 7426

636 film.person_or_entity_appearing_in_film.film 7625

1806 film.person_or_entity_appearing_in_film.film 5513

2100 people.person.profession 4221

652 film.actor.film 7626

15 music.artist.origin 4456

2397 music.artist.origin 5270

2148 film.actor.film 4699

2945 people.deceased_person.place_of_death 4362

298 film.actor.film 3991

3390 film.person_or_entity_appearing_in_film.film 7627

1345 people.person.place_of_birth 3947

3648 film.person_or_entity_appearing_in_film.film 7628

3316 film.director.film 6574

2554 film.actor.film 4385

461 music.artist.origin 3864

851 people.person.place_of_birth 4094

68 film.person_or_entity_appearing_in_film.film 4806

2788 film.person_or_entity_appearing_in_film.film 5195

2771 film.person_or_entity_appearing_in_film.film 7335

3088 people.deceased_person.place_of_death 6448

2635 film.actor.film 7629

1751 people.person.place_of_birth 7630

506 music.artist.origin 5873

155 award.winner.awards_won 7631

3035 film.actor.film 7632

82 film.actor.film 7633

3046 film.actor.film 5620

3629 people.deceased_person.place_of_death 7634

1203 music.artist.origin 4053

67 film.actor.film 6872

2734 film.actor.film 7214

1383 film.actor.film 4720

1325 film.person_or_entity_appearing_in_film.film 4792

1473 film.actor.film 7635

1150 music.artist.origin 5019

850 music.artist.origin 5664

2341 film.actor.film 5318

539 film.actor.film 7636

2214 people.person.place_of_birth 7637

1616 film.actor.film 7638

3211 film.person_or_entity_appearing_in_film.film 7639

967 people.person.place_of_birth 7640

646 film.actor.film 7641

3394 people.person.place_of_birth 7262

447 film.person_or_entity_appearing_in_film.film 7642

2636 film.actor.film 5852

2636 film.person_or_entity_appearing_in_film.film 5536

3179 film.actor.film 6469

3166 film.actor.film 5292

1353 film.person_or_entity_appearing_in_film.film 5759

1353 film.person_or_entity_appearing_in_film.film 7643

2460 film.person_or_entity_appearing_in_film.film 7644

2412 award.nominee.award_nominations 5703

2192 music.artist.origin 4058

3738 cvg.publisher.games_published 7226

3738 cvg.publisher.games_published 4680

436 film.actor.film 4681

1787 film.actor.film 7645

3760 people.person.place_of_birth 7646

2714 music.artist.origin 4058

2867 film.person_or_entity_appearing_in_film.film 4933

2241 film.distributor.film 7647

2241 film.distributor.film 7648

2241 film.distributor.film 7649

2241 film.distributor.film 7650

2241 film.production_company.film 5382

1624 film.person_or_entity_appearing_in_film.film 6478

2078 film.person_or_entity_appearing_in_film.film 4132

2189 film.actor.film 7651

1567 film.person_or_entity_appearing_in_film.film 4323

2677 people.person.place_of_birth 4057

2861 film.actor.film 7293

2162 film.person_or_entity_appearing_in_film.film 7652

2970 film.actor.film 4799

2526 music.artist.album 4605

3772 film.film.genre 4410

2711 film.actor.film 4478

1816 film.person_or_entity_appearing_in_film.film 4359

683 music.artist.origin 3961

1839 film.person_or_entity_appearing_in_film.film 7369

1911 music.artist.origin 4362

1157 film.director.film 6121

1006 award.nominee.award_nominations 6053

2340 music.artist.origin 4057

3333 music.artist.origin 3864

1041 film.actor.film 7653

662 film.actor.film 7654

870 music.artist.origin 4991

67 film.actor.film 7655

67 film.actor.film 5068

2359 film.person_or_entity_appearing_in_film.film 4136

2359 film.person_or_entity_appearing_in_film.film 7656

3364 film.actor.film 7657

314 music.artist.origin 4422

3585 film.actor.film 5984

2398 event.agent.performance 4603

453 film.person_or_entity_appearing_in_film.film 6080

407 film.person_or_entity_appearing_in_film.film 7035

325 film.actor.film 7038

18 music.artist.origin 4183

3118 people.person.place_of_birth 5161

1896 music.artist.origin 4150

3513 music.artist.album 6126

3282 film.person_or_entity_appearing_in_film.film 7658

1564 music.artist.origin 4050

1702 music.artist.origin 4785

1001 music.artist.origin 6290

187 event.agent.performance 4898

1211 film.actor.film 6176

2968 film.actor.film 7659

1037 music.artist.origin 7091

1172 film.actor.film 7660

389 people.person.place_of_birth 7661

294 film.writer.film 7225

436 film.actor.film 5899

901 film.actor.film 4930

625 film.actor.film 4835

796 film.person_or_entity_appearing_in_film.film 4230

796 film.person_or_entity_appearing_in_film.film 6959

2159 film.actor.film 5334

2867 film.person_or_entity_appearing_in_film.film 6441

200 music.artist.origin 4087

2241 film.distributor.film 7229

1936 film.actor.film 5967

3669 film.person_or_entity_appearing_in_film.film 6239

1360 film.person_or_entity_appearing_in_film.film 6784

2207 film.actor.film 5323

3049 film.actor.film 7662

3321 film.person_or_entity_appearing_in_film.film 7663

2634 film.actor.film 7664

1474 film.person_or_entity_appearing_in_film.film 7665

380 award.competitor.award_nominations 7666

2235 music.artist.origin 4077

2407 people.person.place_of_birth 5151

2938 film.person_or_entity_appearing_in_film.film 7667

1763 music.artist.origin 4183

2635 film.person_or_entity_appearing_in_film.film 7668

111 film.person_or_entity_appearing_in_film.film 5638

3384 film.person_or_entity_appearing_in_film.film 7669

3661 film.actor.film 7670

1232 film.actor.film 5219

718 music.musician.instruments_played 3959

82 people.person.place_of_birth 4010

1946 music.musician.instruments_played 7381

183 film.person_or_entity_appearing_in_film.film 7404

2337 film.actor.film 7535

3364 film.person_or_entity_appearing_in_film.film 7657

1960 film.person_or_entity_appearing_in_film.film 4324

954 people.person.place_of_birth 4217

2029 film.actor.film 7536

1792 people.person.place_of_birth 4665

2080 film.actor.film 4672

2080 film.person_or_entity_appearing_in_film.film 4763

1079 film.director.film 7671

74 people.person.place_of_birth 7672

3228 film.person_or_entity_appearing_in_film.film 5847

2913 film.person_or_entity_appearing_in_film.film 7673

1469 film.actor.film 7674

458 film.person_or_entity_appearing_in_film.film 4608

2892 film.actor.film 3920

676 music.artist.origin 7675

3009 people.person.place_of_birth 6518

49 film.actor.film 7676

3166 film.actor.film 7677

580 music.artist.origin 5521

1774 film.person_or_entity_appearing_in_film.film 7678

3421 music.artist.origin 4526

527 film.person_or_entity_appearing_in_film.film 7679

484 film.actor.film 7680

3452 music.artist.origin 4552

3219 film.person_or_entity_appearing_in_film.film 6850

1359 film.person_or_entity_appearing_in_film.film 5642

2797 music.artist.origin 7681

2215 film.actor.film 7682

2574 music.artist.origin 5061

1249 film.actor.film 6413

528 music.artist.origin 7683

2241 film.distributor.film 7684

2241 film.distributor.film 7685

2380 film.person_or_entity_appearing_in_film.film 7182

1464 film.person_or_entity_appearing_in_film.film 7291

2780 music.artist.album 6477

2440 music.artist.origin 3864

1624 film.actor.film 7686

2280 music.artist.origin 6720

3296 film.actor.film 7687

894 film.actor.film 6345

1767 film.person_or_entity_appearing_in_film.film 7688

3143 tv.program.genre 4255

534 film.actor.film 4794

3772 film.film.actor 6383

6 music.artist.origin 4133

1526 film.person_or_entity_appearing_in_film.film 7689

661 music.album.genre 6384

1609 music.artist.origin 5061

2821 film.actor.film 7690

3433 film.actor.film 7691

1950 music.artist.origin 4362

3742 film.actor.film 4477

3099 music.artist.origin 4150

2202 music.artist.origin 5656

3132 film.actor.film 5393

2512 film.person_or_entity_appearing_in_film.film 6588

1952 film.person_or_entity_appearing_in_film.film 7692

1871 people.person.place_of_birth 5559

2635 film.actor.film 7693

2378 award.nominee.award_nominations 7694

115 film.person_or_entity_appearing_in_film.film 7695

623 film.actor.film 7696

2809 music.artist.origin 7359

2134 music.artist.origin 4183

633 film.person_or_entity_appearing_in_film.film 7697

2359 film.actor.film 6742

1805 film.person_or_entity_appearing_in_film.film 7698

1473 film.actor.film 7699

1473 film.person_or_entity_appearing_in_film.film 4684

127 film.actor.film 7700

2524 music.artist.origin 3897

1685 music.artist.origin 4332

2737 film.actor.film 5284

407 film.actor.film 5285

3598 film.actor.film 7217

1532 people.person.place_of_birth 4199

1554 music.artist.origin 4391

2886 people.person.place_of_birth 3859

2912 film.actor.film 6718

584 film.actor.film 7701

3282 film.actor.film 7658

361 film.actor.film 6792

2913 film.actor.film 7673

2801 music.artist.origin 7359

447 award.competitor.award_nominations 7702

165 music.artist.origin 4214

1299 film.person_or_entity_appearing_in_film.film 7703

445 film.actor.film 6779

2460 film.person_or_entity_appearing_in_film.film 7704

278 film.actor.film 7346

270 film.person_or_entity_appearing_in_film.film 5326

484 film.person_or_entity_appearing_in_film.film 7094

796 film.actor.film 6329

3400 people.person.place_of_birth 5924

2241 film.production_company.film 7705

2241 film.production_company.film 7706

1464 film.actor.film 6985

1762 film.actor.film 7707

3134 music.artist.album 6060

1804 film.actor.film 7708

591 people.person.place_of_birth 4057

387 people.person.profession 7367

3031 film.actor.film 5905

1301 music.artist.origin 4852

1360 film.actor.film 5475

1567 people.person.place_of_birth 4369

1772 film.person_or_entity_appearing_in_film.film 7709

1475 film.actor.film 7263

434 film.actor.film 4205

3321 film.person_or_entity_appearing_in_film.film 6113

1783 music.musician.instruments_played 4349

232 film.actor.film 7710

3135 music.artist.origin 5348

2556 film.actor.film 4323

2546 film.person_or_entity_appearing_in_film.film 7711

687 film.person_or_entity_appearing_in_film.film 7712

866 music.artist.origin 4852

2434 music.artist.origin 5664

2635 film.person_or_entity_appearing_in_film.film 4427

1911 people.person.place_of_birth 5213

1818 people.person.place_of_birth 7713

3170 music.artist.origin 4108

1633 film.person_or_entity_appearing_in_film.film 7714

2792 film.person_or_entity_appearing_in_film.film 6909

1179 film.film.director4114

3827 film.actor.film 6490

1188 people.person.place_of_birth 4362

1653 film.person_or_entity_appearing_in_film.film 7715

2371 film.actor.film 4712

1802 music.artist.origin 4166

2079 people.person.place_of_birth 4010

2313 people.person.place_of_birth 3984

1910 film.film.star 4322

2398 event.agent.performance 4898

2488 film.actor.film 7716

1035 film.actor.film 7717

3447 film.actor.film 7718

1181 film.person_or_entity_appearing_in_film.film 7719

675 film.actor.film 6520

675 film.person_or_entity_appearing_in_film.film 4267

675 film.person_or_entity_appearing_in_film.film 6520

357 music.producer.tracks_produced 6633

283 music.musician.instruments_played 6942

2636 film.person_or_entity_appearing_in_film.film 7720

1693 music.artist.origin 3864

79 film.person_or_entity_appearing_in_film.film 4886

821 music.artist.origin 4454

3738 cvg.publisher.games_published 7721

2293 film.person_or_entity_appearing_in_film.film 7722

1603 film.person_or_entity_appearing_in_film.film 7723

1356 film.person_or_entity_appearing_in_film.film 5642

3212 film.actor.film 7724

2867 film.person_or_entity_appearing_in_film.film 7147

2241 film.distributor.film 7148

2241 film.distributor.film 7725

2241 film.production_company.film 7726

2241 film.production_company.film 7727

2241 film.production_company.film 6867

988 film.actor.film 7728

777 film.actor.film 6631

644 film.actor.film 6109

261 award.winner.awards_won 7729

1567 film.person_or_entity_appearing_in_film.film 4915

643 film.person_or_entity_appearing_in_film.film 7474

418 music.producer.tracks_produced 7355

636 film.person_or_entity_appearing_in_film.film 7730

456 music.artist.origin 5139

2184 film.actor.film 5305

434 film.person_or_entity_appearing_in_film.film 7731

538 film.person_or_entity_appearing_in_film.film 7732

757 people.person.place_of_birth 6036

1884 film.actor.film 7733

232 film.actor.film 7121

3742 film.person_or_entity_appearing_in_film.film 4355

3390 film.person_or_entity_appearing_in_film.film 7734

2010 film.actor.film 4528

206 film.person_or_entity_appearing_in_film.film 7735

1358 film.actor.film 4529

383 film.person_or_entity_appearing_in_film.film 3881

2402 people.person.place_of_birth 5872

3307 people.person.place_of_birth 4768

1696 people.person.place_of_birth 7554

3492 music.artist.origin 7262

3827 film.person_or_entity_appearing_in_film.film 6359

1748 film.person_or_entity_appearing_in_film.film 7736

2050 film.actor.film 4976

3788 film.actor.film 4660

2734 film.actor.film 4863

2359 film.person_or_entity_appearing_in_film.film 7737

1383 film.person_or_entity_appearing_in_film.film 7738

793 film.person_or_entity_appearing_in_film.film 4281

1141	film.person_or_entity_appearing_in_film.film	7739

2616	music.artist.origin	7477

1706	film.person_or_entity_appearing_in_film.film	5323

2737	film.person_or_entity_appearing_in_film.film	7740

66	film.actor.film	7741

2080	film.actor.film	7742

557	film.actor.film	7743

557	film.person_or_entity_appearing_in_film.film	7744

893	film.actor.film	7745

74	film.person_or_entity_appearing_in_film.film	6157

2913	film.person_or_entity_appearing_in_film.film	7746

1788	event.agent.performance	4729

437	film.actor.film	5955

2317	people.person.place_of_birth	4496

2636	film.actor.film	7747

2636	film.person_or_entity_appearing_in_film.film	6468

2650	film.film.actor	6812

76	people.person.place_of_birth	7492

1211	music.artist.album	6479

1573	people.person.place_of_birth	7748

2689	film.actor.film	7749

781	film.person_or_entity_appearing_in_film.film	7750

133	music.artist.origin	4057

2460	film.person_or_entity_appearing_in_film.film	7751

79	film.person_or_entity_appearing_in_film.film	5031

771	film.actor.film	7752

184	film.actor.film	7014

1154	film.person_or_entity_appearing_in_film.film	6069

1808	people.person.place_of_birth	7753

2241	film.distributor.film	7754

2241 film.distributor.film 7755

419 film.person_or_entity_appearing_in_film.film 5885

681 film.actor.film 7756

1262 film.actor.film 7757

2189 film.actor.film 7201

894 film.actor.film 7758

2112 people.person.place_of_birth 7759

2098 music.musician.instruments_played 5558

3122 people.person.place_of_birth 4057

2184 film.actor.film 4073

3002 film.person_or_entity_appearing_in_film.film 4994

3374 film.actor.film 4996

253 film.actor.film 6676

157 event.agent.performance 4603

807 music.artist.album 4617

2309 film.person_or_entity_appearing_in_film.film 5749

2274 music.musician.instruments_played 4349

3390 film.actor.film 7332

1323 film.person_or_entity_appearing_in_film.film 5943

2711 film.actor.film 7760

3048 film.person_or_entity_appearing_in_film.film 7761

1814 film.film.rating 4223

3528 film.person_or_entity_appearing_in_film.film 7762

1479 film.actor.film 7763

2599 film.actor.film 7764

1358 film.actor.film 6784

997 film.actor.film 5098

2635 film.person_or_entity_appearing_in_film.film 4651

804 music.artist.origin 6868

1374 music.artist.origin 6649

1153	film.actor.film	4435

1153	film.actor.film	4181

1153	film.actor.film	4169

1891	film.actor.film	4060

3020	film.person_or_entity_appearing_in_film.film	7765

880 music.artist.origin	4952

886 music.artist.origin	4013

358 type.object.key	358

3423	film.actor.film	7766

756 music.artist.origin	5228

3548	film.person_or_entity_appearing_in_film.film	7767

2147	film.actor.film	6089

1473	film.actor.film	7768

645 film.actor.film	5361

645 film.person_or_entity_appearing_in_film.film	7769

645 film.person_or_entity_appearing_in_film.film	6130

3598	film.person_or_entity_appearing_in_film.film	4722

1885	film.actor.film	7770

106 music.artist.origin	4057

809 film.actor.film	5165

672 film.actor.film	6834

2636	film.person_or_entity_appearing_in_film.film	7771

2122	film.actor.film	7772

2688	film.person_or_entity_appearing_in_film.film	4411

1353	film.actor.film	7643

1840	film.person_or_entity_appearing_in_film.film	7773

3738	cvg.publisher.games_published	5428

1154	film.actor.film	4086

1249	film.person_or_entity_appearing_in_film.film	7774

2241	film.distributor.film	5970

2241 film.distributor.film 4513

2241 film.distributor.film 7775

2241 film.distributor.film 7776

2241 film.production_company.film 7777

1936 film.person_or_entity_appearing_in_film.film 7778

3003 people.person.place_of_birth 6797

520 music.artist.origin 4232

2582 people.person.place_of_birth 3846

1624 film.actor.film 4574

3709 music.artist.origin 3864

3789 film.actor.film 7779

356 film.actor.film 7780

1656 film.person_or_entity_appearing_in_film.film 7781

643 film.actor.film 6187

1357 film.actor.film 7782

401 music.artist.origin 4391

1825 people.person.place_of_birth 4583

3390 film.actor.film 4899

2764 people.deceased_person.place_of_death 4013

2711 film.actor.film 7153

2711 film.actor.film 7783

2658 film.person_or_entity_appearing_in_film.film 7784

1814 film.film.language 4076

2010 film.person_or_entity_appearing_in_film.film 7785

204 film.person_or_entity_appearing_in_film.film 4105

2599 film.person_or_entity_appearing_in_film.film 7764

2904 film.actor.film 6197

1279 people.person.place_of_birth 3947

567 film.actor.film 6562

2635 film.person_or_entity_appearing_in_film.film 4313

3307 film.actor.film 7786

623 film.actor.film 6881

1311 music.artist.origin 3864

1032 film.actor.film 4488

2620 film.actor.film 6897

756 film.actor.film 6567

1881 music.artist.origin 4191

1525 film.person_or_entity_appearing_in_film.film 7787

304 film.person_or_entity_appearing_in_film.film 6943

2359 film.person_or_entity_appearing_in_film.film 7788

3598 film.actor.film 6063

2105 people.person.place_of_birth 7789

2925 music.producer.tracks_produced 7790

70 film.actor.film 7138

2636 film.actor.film 5130

2743 music.artist.album 7791

1458 film.person_or_entity_appearing_in_film.film 7792

294 film.person_or_entity_appearing_in_film.film 7225

2400 film.actor.film 7014

2867 film.actor.film 7793

3025 film.actor.film 7794

2241 film.distributor.film 7795

2241 film.production_company.film 4887

2380 film.actor.film 5162

795 film.actor.film 5470

1936 film.actor.film 7796

1936 film.person_or_entity_appearing_in_film.film 4841

1464 film.person_or_entity_appearing_in_film.film 7797

451 award.winner.awards_won 6182

1061 film.actor.film 6704

3789	film.actor.film	7798

2098	film.actor.film	7799

2075	music.artist.origin	7624

989 film.actor.film	5784

2821	film.person_or_entity_appearing_in_film.film	7690

1323	film.person_or_entity_appearing_in_film.film	7800

2711	film.actor.film	7801

3256	film.actor.film	6792

1479	people.person.place_of_birth	4094

1750	film.person_or_entity_appearing_in_film.film	4902

1142	film.person_or_entity_appearing_in_film.film	7182

1662	film.actor.film	7802

375 film.actor.film	4001

3307	film.person_or_entity_appearing_in_film.film	7786

1157	film.actor.film	7803

1409	type.object.subject_key	1409

2851	music.artist.origin	7804

3384	film.actor.film	7805

3592	people.person.place_of_birth	4347

2146	music.musician.instruments_played	4349

2799	film.actor.film	5562

3645	film.person_or_entity_appearing_in_film.film	7157

2359	film.person_or_entity_appearing_in_film.film	7403

2370	film.actor.film	6768

1325	award.winner.awards_won	3964

1205	people.person.place_of_birth	3859

3598	film.actor.film	7806

1342	music.artist.origin	7807

893 film.person_or_entity_appearing_in_film.film	5887

3298	music.artist.origin	6137

672 film.person_or_entity_appearing_in_film.film 7312

80 film.actor.film 7526

675 film.person_or_entity_appearing_in_film.film 4504

2972 film.actor.film 7808

458 music.artist.album 7809

2339 music.album.genre 7810

755 film.actor.film 6656

2636 film.person_or_entity_appearing_in_film.film 6171

2636 film.person_or_entity_appearing_in_film.film 7811

2481 film.actor.film 6264

1408 film.person_or_entity_appearing_in_film.film 6499

1201 music.artist.origin 6128

2460 film.actor.film 7812

294 film.actor.film 7813

2483 music.musician.instruments_played 7814

3738 games.publisher.games_published 7815

436 film.actor.film 7816

1154 film.person_or_entity_appearing_in_film.film 7817

2874 film.actor.film 4619

2241 film.distributor.film 4572

2241 film.production_company.film 7818

2241 film.production_company.film 6549

2241 film.production_company.film 5504

2241 film.production_company.film 5129

795 film.person_or_entity_appearing_in_film.film 5694

2500 music.artist.origin 6405

777 film.actor.film 7819

2704 music.artist.origin 7820

1806 film.actor.film 4202

2707 film.actor.film 3937

1475	film.actor.film	7821

1580	film.person_or_entity_appearing_in_film.film	7822

3321	film.person_or_entity_appearing_in_film.film	7823

3534	music.artist.origin	5272

3052	film.person_or_entity_appearing_in_film.film	5517

1452	music.artist.origin	7824

3279	film.person_or_entity_appearing_in_film.film	7825

2711	film.actor.film	4358

2010	film.actor.film	7826

3132	film.person_or_entity_appearing_in_film.film	7827

2647	film.actor.film	5848

953 music.album.genre	7828

3384	film.actor.film	7829

115 film.actor.film	6612

2362	film.actor.film	7830

1045	film.person_or_entity_appearing_in_film.film	7831

1153	film.actor.film	7832

362 music.artist.origin	3864

1748	film.person_or_entity_appearing_in_film.film	7275

2050	film.person_or_entity_appearing_in_film.film	7833

2708	film.actor.film	7834

1032	film.actor.film	7835

2079	film.person_or_entity_appearing_in_film.film	7836

2359	film.actor.film	7656

3061	event.agent.performance	4387

2344	film.actor.film	5614

3087	film.person_or_entity_appearing_in_film.film	7837

2944	film.actor.film	7560

1193	music.artist.album	4893

2276	film.person_or_entity_appearing_in_film.film	7838

893 film.actor.film 7839

1487 music.artist.origin 5265

3498 film.actor.film 7840

2913 film.person_or_entity_appearing_in_film.film 5166

2914 film.person_or_entity_appearing_in_film.film 7411

896 film.actor.film 7841

1102 music.artist.origin 4150

1601 film.person_or_entity_appearing_in_film.film 6952

408 film.person_or_entity_appearing_in_film.film 7842

1204 film.actor.film 7843

3500 film.person_or_entity_appearing_in_film.film 7844

2636 film.actor.film 7845

2636 film.person_or_entity_appearing_in_film.film 7846

2689 music.artist.album 7847

715 film.person_or_entity_appearing_in_film.film 5719

3738 cvg.publisher.games_published 7848

2672 people.person.place_of_birth 4057

766 music.artist.origin 7849

523 music.artist.origin 3961

2241 film.distributor.film 6446

2241 film.production_company.film 5903

2241 film.production_company.film 7850

3157 people.person.place_of_birth 7851

1464 film.actor.film 7521

1464 film.person_or_entity_appearing_in_film.film 7852

310 music.artist.origin 4357

420 film.person_or_entity_appearing_in_film.film 5600

2780 people.person.place_of_birth 4171

1624 film.person_or_entity_appearing_in_film.film 7853

777 film.actor.film 4197

1618	film.person_or_entity_appearing_in_film.film	7854

2126	film.actor.film	7855

1767	film.actor.film	6671

3632	film.person_or_entity_appearing_in_film.film	7856

1768	people.deceased_person.place_of_death	7857

2275	film.person_or_entity_appearing_in_film.film	3937

2098	music.musician.instruments_played	3959

418 film.person_or_entity_appearing_in_film.film	7858

817 film.actor.film	7859

159 film.actor.film	7119

1310	film.actor.film	4210

3825	music.artist.origin	4927

1954	people.person.place_of_birth	7860

287 people.deceased_person.place_of_death	3944

2556	film.person_or_entity_appearing_in_film.film	6155

3390	film.actor.film	3876

3279	film.actor.film	4007

3648	film.actor.film	7861

3132	film.person_or_entity_appearing_in_film.film	7862

687 film.actor.film	7712

129 film.actor.film	5198

142 film.person_or_entity_appearing_in_film.film	4530

2635	film.person_or_entity_appearing_in_film.film	6357

2621	film.person_or_entity_appearing_in_film.film	7863

2876	music.musician.instruments_played	4349

58	film.actor.film	4318

1547	music.artist.origin	3932

627 film.actor.film	6109

1891	film.actor.film	7864

2359	film.person_or_entity_appearing_in_film.film	7865

1473 film.actor.film 7866

1026 film.actor.film 6770

811 music.artist.origin 4552

793 film.actor.film 5821

793 film.person_or_entity_appearing_in_film.film 5821

1236 music.artist.origin 4008

3040 film.actor.film 7867

620 people.deceased_person.place_of_death 4057

453 film.actor.film 7868

747 film.person_or_entity_appearing_in_film.film 5917

52 film.actor.film 7780

1181 film.person_or_entity_appearing_in_film.film 7869

3728 music.artist.origin 7302

70 film.person_or_entity_appearing_in_film.film 7315

2095 film.actor.film 7611

1404 film.person_or_entity_appearing_in_film.film 7011

1846 film.actor.film 3923

2636 film.actor.film 7870

2351 people.person.profession 4221

3179 film.person_or_entity_appearing_in_film.film 5118

2460 film.actor.film 7871

700 people.person.place_of_birth 5019

184 film.actor.film 5543

901 film.person_or_entity_appearing_in_film.film 5377

826 music.artist.origin 5556

2867 film.actor.film 6336

3501 film.person_or_entity_appearing_in_film.film 7196

2241 film.distributor.film 7872

2241 film.distributor.film 7873

2241 film.distributor.film 6960

2779	film.actor.film	6315

3297	film.actor.film	7874

823 music.artist.origin	4046

1058	music.artist.origin	5262

1302	music.artist.origin	3864

3218	music.artist.origin	4003

958 music.artist.album	6698

636 film.actor.film	7730

3743	music.musician.instruments_played	5174

3672	music.artist.origin	5662

1636	music.artist.origin	4183

36	music.artist.origin	3864

2870	film.person_or_entity_appearing_in_film.film	7875

2452	film.person_or_entity_appearing_in_film.film	7876

1632	people.person.place_of_birth	5664

1962	film.actor.film	3977

3304	music.artist.origin	7615

68	film.actor.film	7140

2684	film.actor.film	7877

2242	film.actor.film	7878

2156	music.artist.origin	4455

142 film.actor.film	7879

2528	film.actor.film	7880

1839	film.actor.film	7369

2635	film.person_or_entity_appearing_in_film.film	5399

2635	film.person_or_entity_appearing_in_film.film	7629

1930	people.person.place_of_birth	4583

3384	film.person_or_entity_appearing_in_film.film	7881

3384	film.person_or_entity_appearing_in_film.film	7829

2570	music.artist.origin	3864

540 film.person_or_entity_appearing_in_film.film 7882

1633 film.actor.film 5055

374 film.actor.film 4317

1174 music.artist.origin 4008

1153 film.person_or_entity_appearing_in_film.film 6382

3788 film.actor.film 7883

1525 film.person_or_entity_appearing_in_film.film 5490

993 film.actor.film 7884

2804 film.person_or_entity_appearing_in_film.film 7885

2879 music.artist.origin 4057

1473 film.actor.film 7886

1473 film.actor.film 5659

1137 music.artist.origin 7399

2337 film.actor.film 6210

2337 film.person_or_entity_appearing_in_film.film 5240

3364 film.person_or_entity_appearing_in_film.film 7887

1959 film.person_or_entity_appearing_in_film.film 7308

3302 film.person_or_entity_appearing_in_film.film 7888

342 music.artist.origin 4454

91 film.actor.film 5414

1187 film.person_or_entity_appearing_in_film.film 5844

2737 film.person_or_entity_appearing_in_film.film 7889

2338 film.actor.film 4334

2259 film.person_or_entity_appearing_in_film.film 7890

2913 film.person_or_entity_appearing_in_film.film 7891

1744 film.actor.film 5417

1469 film.actor.film 7221

1601 film.actor.film 7892

3185 film.person_or_entity_appearing_in_film.film 7893

3821 film.person_or_entity_appearing_in_film.film 7894

2122 film.person_or_entity_appearing_in_film.film 7772

79 film.actor.film 7895

715 film.person_or_entity_appearing_in_film.film 3853

1840 film.actor.film 7773

270 people.person.place_of_birth 6058

484 film.actor.film 7896

3738 cvg.publisher.games_published 5288

3738 games.publisher.games_published 7897

3738 games.publisher.games_published 7898

3738 games.publisher.games_published 7899

3738 games.publisher.games_published 4394

442 film.actor.film 6609

2241 film.distributor.film 7900

2241 film.distributor.film 4250

2241 film.production_company.film 7901

2241 film.production_company.film 7902

2241 film.production_company.film 7776

2077 people.person.place_of_birth 5569

2250 music.artist.origin 4094

1624 people.person.place_of_birth 4347

1360 film.person_or_entity_appearing_in_film.film 7903

958 music.musician.instruments_played 4349

1914 music.artist.origin 7551

3049 film.person_or_entity_appearing_in_film.film 7904

1371 music.artist.origin 3846

3742 film.actor.film 7295

2716 music.artist.origin 7358

2711 film.actor.film 7905

1814 film.film.writer 7906

638 film.actor.film 6825

3688	film.person_or_entity_appearing_in_film.film	5649
959 film.actor.film	7907
3542	film.person_or_entity_appearing_in_film.film	4822
1569	film.person_or_entity_appearing_in_film.film	4328
2635	film.actor.film	4109
1351	film.person_or_entity_appearing_in_film.film	6257
548 film.person_or_entity_appearing_in_film.film	7908
623 film.person_or_entity_appearing_in_film.film	7909
2521	music.artist.origin	5873
3317	film.person_or_entity_appearing_in_film.film	5657
120 music.artist.origin	4057
1833	film.person_or_entity_appearing_in_film.film	7910
3554	music.artist.origin	7911
3216	tv.program.country_of_origin	5061
525 music.artist.origin	7630
2700	film.person_or_entity_appearing_in_film.film	7912
2998	film.actor.film	7913
645 film.actor.film	5886
1601	film.person_or_entity_appearing_in_film.film	7914
974 film.person_or_entity_appearing_in_film.film	5891
1846	film.person_or_entity_appearing_in_film.film	4411
2294	music.artist.origin	7915
2391	people.person.place_of_birth	3942
3166	film.person_or_entity_appearing_in_film.film	5292
2460	film.actor.film	6220
3738	games.publisher.games_published	4882
3738	games.publisher.games_published	6842
184 film.person_or_entity_appearing_in_film.film	5543
436 film.person_or_entity_appearing_in_film.film	7916
2241	film.distributor.film	5133

2241 film.distributor.film 6444

2241 film.production_company.film 7917

988 film.person_or_entity_appearing_in_film.film 4823

1936 film.person_or_entity_appearing_in_film.film 7796

2331 music.artist.origin 7918

481 film.actor.film 7679

135 film.person_or_entity_appearing_in_film.film 7919

3583 film.person_or_entity_appearing_in_film.film 5990

2572 music.artist.origin 4376

3031 film.actor.film 7920

2275 film.actor.film 6869

1360 film.actor.film 6784

1967 music.artist.origin 4927

2810 music.artist.origin 5009

194 film.person_or_entity_appearing_in_film.film 7921

1475 film.actor.film 7886

1357 film.person_or_entity_appearing_in_film.film 4142

2531 film.actor.film 5440

2822 people.person.place_of_birth 5828

36 music.producer.tracks_produced 7922

3321 film.person_or_entity_appearing_in_film.film 5192

3315 music.artist.origin 5265

1096 music.artist.origin 4380

1954 film.actor.film 7923

1105 film.director.film 5648

1623 film.actor.film 4149

3579 film.actor.film 6617

3390 film.actor.film 7627

166 film.person_or_entity_appearing_in_film.film 7924

2711 film.actor.film 7925

640 film.actor.film 7926

2449 film.actor.film 4360

1196 music.artist.origin 4217

396 film.person_or_entity_appearing_in_film.film 4802

1263 music.artist.origin 3972

493 music.artist.origin 6911

2635 film.actor.film 7927

2635 film.person_or_entity_appearing_in_film.film 6393

1719 film.person_or_entity_appearing_in_film.film 7928

2695 music.artist.origin 6249

623 film.person_or_entity_appearing_in_film.film 6624

94 film.actor.film 7929

94 film.person_or_entity_appearing_in_film.film 6689

3539 music.artist.origin 3864

579 music.artist.origin 7930

3064 music.artist.origin 4057

2125 film.person_or_entity_appearing_in_film.film 4119

938 music.artist.origin 7672

1906 music.artist.origin 4093

2893 film.actor.film 7931

1845 people.person.place_of_birth 7932

2080 film.actor.film 7933

3598 film.actor.film 7934

3685 music.artist.origin 6431

2234 film.actor.film 4444

2637 film.film.executive_producer 5214

2914 film.person_or_entity_appearing_in_film.film 7935

1469 film.actor.film 7936

1453 film.person_or_entity_appearing_in_film.film 5069

437 film.person_or_entity_appearing_in_film.film 5850

1266	people.person.place_of_birth	4191
1467	film.actor.film	3919
1353	film.person_or_entity_appearing_in_film.film	5668
1320	location.location.time_zone	7937
2460	film.actor.film	7938
790	film.actor.film	6796
484	film.person_or_entity_appearing_in_film.film	7939
2877	film.person_or_entity_appearing_in_film.film	5121
3738	cvg.publisher.games_published	5461
3738	cvg.publisher.games_published	5636
3738	games.publisher.games_published	7940
3738	games.publisher.games_published	7941
2414	award.competitor.award_nominations	6030
2577	music.musician.instruments_played	5802
2241	film.distributor.film	6983
2241	film.distributor.film	7942
2241	film.distributor.film	7230
2241	film.production_company.film	7795
2241	film.production_company.film	7943
1464	film.person_or_entity_appearing_in_film.film	7621
2459	people.person.place_of_birth	7944
1101	music.artist.origin	5160
2078	film.actor.film	5508
1887	music.artist.origin	4217
1567	film.actor.film	4328
643	film.person_or_entity_appearing_in_film.film	7945
2640	music.artist.origin	5139
1806	film.person_or_entity_appearing_in_film.film	7946
3235	event.agent.performance	5097
1495	music.artist.origin	5265

385 film.person_or_entity_appearing_in_film.film 7102

3107 music.artist.origin 6012

3648 film.person_or_entity_appearing_in_film.film 4210

1705 film.actor.film 6521

2237 music.artist.origin 3864

997 film.actor.film 6392

142 people.deceased_person.place_of_death 6425

2635 film.person_or_entity_appearing_in_film.film 7947

274 people.person.place_of_birth 4362

1232 film.actor.film 7948

31 business.operation.industry 6189

548 film.person_or_entity_appearing_in_film.film 7949

262 music.artist.origin 4010

1044 award.nominee.award_nominations 7950

58 film.person_or_entity_appearing_in_film.film 4656

2578 music.musician.instruments_played 5367

2359 film.person_or_entity_appearing_in_film.film 7951

3559 music.artist.origin 7057

3215 film.film.actor 4114

2727 music.musician.instruments_played 5619

987 film.person_or_entity_appearing_in_film.film 7952

2392 music.artist.origin 4957

3109 people.person.place_of_birth 4077

386 film.person_or_entity_appearing_in_film.film 5067

3498 film.person_or_entity_appearing_in_film.film 5988

151 people.person.place_of_birth 4768

70 music.artist.album 5395

260 music.artist.origin 3864

974 film.person_or_entity_appearing_in_film.film 7953

437 film.person_or_entity_appearing_in_film.film 7537

2096 film.person_or_entity_appearing_in_film.film 7954

3811 music.artist.origin 7955

1211 award.competitor.award_nominations 7956

48 music.producer.tracks_produced 7957

389 music.artist.origin 7661

2460 film.actor.film 6814

79 film.actor.film 7958

782 film.actor.film 6006

436 film.actor.film 7959

3710 music.artist.origin 4347

3560 music.artist.origin 4024

1694 music.artist.origin 4563

10 music.artist.origin 4454

2241 film.distributor.film 5863

2241 film.distributor.film 7019

2241 film.distributor.film 7727

2241 film.distributor.film 7960

2241 film.production_company.film 6751

2241 film.production_company.film 7961

2241 film.production_company.film 7962

3297 film.actor.film 7963

3297 film.actor.film 4382

681 film.actor.film 5990

1624 film.person_or_entity_appearing_in_film.film 6789

2487 film.person_or_entity_appearing_in_film.film 4096

622 film.actor.film 5216

1586 music.artist.album 7964

3486 film.actor.film 6556

418 music.artist.track 7965

2884 film.actor.film 7475

1772 film.actor.film 7709

3186 film.actor.film 7966

1526 people.deceased_person.place_of_death 4362

3321 film.actor.film 7967

238 film.person_or_entity_appearing_in_film.film 7968

164 film.person_or_entity_appearing_in_film.film 7969

385 people.person.place_of_birth 4199

3648 film.actor.film 7628

2962 film.person_or_entity_appearing_in_film.film 6861

2540 music.artist.origin 7807

3507 film.actor.film 7970

997 film.person_or_entity_appearing_in_film.film 7971

2635 film.actor.film 7972

2621 film.actor.film 7973

3384 film.actor.film 5102

2805 people.person.place_of_birth 4094

818 film.actor.film 6596

509 music.artist.origin 4057

1153 film.actor.film 7974

1153 film.actor.film 4485

3020 film.actor.film 5677

2050 film.actor.film 4913

674 film.person_or_entity_appearing_in_film.film 6161

3412 film.film.producer 7975

37 film.person_or_entity_appearing_in_film.film 7060

2944 film.person_or_entity_appearing_in_film.film 7366

1935 film.person_or_entity_appearing_in_film.film 7976

453 film.actor.film 4730

1209 film.actor.film 4279

539 film.person_or_entity_appearing_in_film.film 7636

3598 film.actor.film 7132

3598 film.person_or_entity_appearing_in_film.film 7977

2752 music.artist.origin 5226

1235 music.artist.origin 4362

2925 music.artist.track 7790

1773 film.person_or_entity_appearing_in_film.film 7163

447 film.actor.film 7642

974 film.actor.film 7978

914 film.character.film 3930

2460 film.actor.film 5633

1735 people.person.place_of_birth 4003

2973 music.artist.origin 5559

602 film.person_or_entity_appearing_in_film.film 6693

3738 cvg.publisher.games_published 7979

2414 film.person_or_entity_appearing_in_film.film 6411

1277 people.person.place_of_birth 4057

2400 film.person_or_entity_appearing_in_film.film 6893

2747 music.artist.origin 4526

2241 film.distributor.film 7980

2241 film.distributor.film 6314

2241 film.distributor.film 5820

1936 film.person_or_entity_appearing_in_film.film 7981

2487 film.person_or_entity_appearing_in_film.film 7547

894 film.actor.film 7549

3669 people.person.place_of_birth 3942

3031 film.actor.film 5870

636 film.person_or_entity_appearing_in_film.film 7982

3097 people.deceased_person.place_of_death 4768

3186 film.person_or_entity_appearing_in_film.film 7966

1051 film.actor.film 7983

3450 music.artist.origin 6435

654 music.artist.origin 4050

538 film.person_or_entity_appearing_in_film.film 7984

3049 film.person_or_entity_appearing_in_film.film 4354

2148 film.person_or_entity_appearing_in_film.film 5911

2116 music.artist.origin 4013

561 music.artist.origin 4174

3390 film.actor.film 7985

380 film.person_or_entity_appearing_in_film.film 7986

1816 film.person_or_entity_appearing_in_film.film 7987

1210 music.artist.origin 4174

1067 film.person_or_entity_appearing_in_film.film 6106

1596 music.artist.origin 4191

1781 music.artist.origin 4121

1358 film.person_or_entity_appearing_in_film.film 7988

997 film.actor.film 7056

3088 film.actor.film 7989

2820 film.actor.film 6967

1293 music.artist.origin 4380

111 film.actor.film 5638

179 music.artist.origin 7990

1431 music.artist.origin 6056

3827 film.actor.film 5838

446 music.artist.origin 4454

1153 film.actor.film 7991

1153 film.actor.film 6739

2320 music.artist.origin 5662

2141 film.person_or_entity_appearing_in_film.film 7992

3020 film.person_or_entity_appearing_in_film.film 7993

3029 film.person_or_entity_appearing_in_film.film 7994

67	film.actor.film	5245

2216	music.producer.tracks_produced	7995

774	film.actor.film	7304

2147	film.actor.film	4490

1473	film.person_or_entity_appearing_in_film.film	7996

2178	people.person.place_of_birth	3855

3216	tv.program.country_of_origin	3961

1686	music.artist.origin	6545

3285	music.artist.origin	3864

2014	film.film.genre	4285

1030	music.artist.origin	4046

386	film.person_or_entity_appearing_in_film.film	4283

249	music.musician.instruments_played	4349

1661	film.actor.film	5628

1007	event.agent.performance	4603

646	film.actor.film	4771

646	film.actor.film	5737

2475	film.person_or_entity_appearing_in_film.film	7997

2460	film.person_or_entity_appearing_in_film.film	7998

79	film.actor.film	7999

1707	film.actor.film	4846

3738	cvg.publisher.games_published	8000

3738	games.publisher.games_published	6957

999	film.person_or_entity_appearing_in_film.film	8001

901	film.person_or_entity_appearing_in_film.film	6412

791	film.person_or_entity_appearing_in_film.film	4082

442	event.agent.performance	4898

2241	film.distributor.film	8002

2241	film.distributor.film	8003

2241	film.distributor.film	8004

2241 film.distributor.film 4730

2241 film.distributor.film 8005

2241 film.distributor.film 8006

2241 film.production_company.film 4549

2241 film.production_company.film 7775

2241 film.production_company.film 7886

1936 film.actor.film 8007

556 film.person_or_entity_appearing_in_film.film 5601

777 film.actor.film 8008

1697 film.person_or_entity_appearing_in_film.film 8009

2420 people.person.place_of_birth 5605

636 film.person_or_entity_appearing_in_film.film 8010

1772 film.person_or_entity_appearing_in_film.film 6188

3665 film.person_or_entity_appearing_in_film.film 8011

1417 people.person.place_of_birth 4552

1357 film.person_or_entity_appearing_in_film.film 8012

1660 film.actor.film 8013

1046 music.artist.origin 4050

3231 film.person_or_entity_appearing_in_film.film 8014

3631 people.deceased_person.place_of_death 4732

166 film.person_or_entity_appearing_in_film.film 8015

2751 music.artist.origin 3859

566 music.artist.origin 4442

2072 people.person.place_of_birth 5019

2300 music.artist.origin 4455

997 film.person_or_entity_appearing_in_film.film 4953

1142 film.actor.film 7182

2635 film.person_or_entity_appearing_in_film.film 6761

1157 film.actor.film 8016

115 film.person_or_entity_appearing_in_film.film 4314

818 film.actor.film 4432

2984 film.actor.film 8017

1439 award.nominee.award_nominations 8018

785 film.person_or_entity_appearing_in_film.film 6395

623 film.actor.film 7909

813 people.person.place_of_birth 6694

3827 film.person_or_entity_appearing_in_film.film 5691

3035 film.person_or_entity_appearing_in_film.film 8019

1153 film.actor.film 6382

1153 film.person_or_entity_appearing_in_film.film 6123

1153 film.person_or_entity_appearing_in_film.film 5839

1400 film.person_or_entity_appearing_in_film.film 4911

912 film.actor.film 6087

67 film.actor.film 6040

2359 film.actor.film 8020

3412 film.film.editor 7975

3559 film.person_or_entity_appearing_in_film.film 6600

1473 film.actor.film 6945

1473 film.person_or_entity_appearing_in_film.film 4276

3133 film.person_or_entity_appearing_in_film.film 8021

2119 music.artist.origin 7477

2750 film.film.director5209

407 film.actor.film 8022

3598 film.actor.film 8023

747 people.person.place_of_birth 7918

2071 award.competitor.awards_won 6886

741 music.artist.origin 4183

2053 film.actor.film 5323

974 film.person_or_entity_appearing_in_film.film 7978

2636 film.person_or_entity_appearing_in_film.film 7090

2096	film.person_or_entity_appearing_in_film.film	8024

2070	film.person_or_entity_appearing_in_film.film	8025

1252	music.artist.origin	3864

3009	film.person_or_entity_appearing_in_film.film	5893

399 film.actor.film	6010

399 film.actor.film	7559

1895	film.actor.film	8026

133 award.nominee.award_nominations	4925

3738	games.publisher.games_published	8027

3738	games.publisher.games_published	5122

1002	film.person_or_entity_appearing_in_film.film	5078

2241	film.distributor.film	8028

2241	film.distributor.film	8029

2241	film.distributor.film	8030

2241	film.production_company.film	8031

2241	film.production_company.film	7725

795 film.person_or_entity_appearing_in_film.film	6155

1936	film.person_or_entity_appearing_in_film.film	6552

1361	people.person.place_of_birth	4442

2780	music.artist.album	6148

894 film.actor.film	8032

894 film.person_or_entity_appearing_in_film.film	8032

356 film.person_or_entity_appearing_in_film.film	7780

3524	music.artist.origin	4362

3031	film.person_or_entity_appearing_in_film.film	5870

1360	film.person_or_entity_appearing_in_film.film	5043

1806	film.actor.film	5907

1843	people.person.place_of_birth	7203

1278	award.winner.awards_won	5188

264 music.artist.origin	4454

1956 music.artist.album 6673

3321 film.actor.film 8033

2630 film.person_or_entity_appearing_in_film.film 8034

2711 film.person_or_entity_appearing_in_film.film 7177

3132 film.actor.film 6335

3314 music.artist.origin 4123

2449 film.actor.film 8035

1358 film.person_or_entity_appearing_in_film.film 7984

997 film.person_or_entity_appearing_in_film.film 4212

671 film.person_or_entity_appearing_in_film.film 5874

1157 film.person_or_entity_appearing_in_film.film 5565

2222 music.musician.instruments_played 4349

2485 people.person.place_of_birth 5125

3237 music.album.genre 4449

2953 film.person_or_entity_appearing_in_film.film 4597

67 film.actor.film 8036

1473 film.actor.film 8037

1473 film.actor.film 8038

1587 film.person_or_entity_appearing_in_film.film 4820

192 music.artist.track 8039

1863 film.person_or_entity_appearing_in_film.film 7163

3693 music.artist.origin 7615

386 film.person_or_entity_appearing_in_film.film 7063

744 music.artist.origin 4150

2562 film.actor.film 4335

128 type.object.subject_key 128

2070 film.person_or_entity_appearing_in_film.film 4238

381 film.person_or_entity_appearing_in_film.film 8040

3179 film.person_or_entity_appearing_in_film.film 8041

305 music.artist.origin 7615

2460 film.person_or_entity_appearing_in_film.film 7812

484 film.actor.film 6578

3738 games.publisher.games_published 8042

3738 games.publisher.games_published 5502

3738 games.publisher.games_published 8043

901 film.actor.film 8044

3655 music.artist.origin 3864

2241 film.distributor.film 8045

2260 music.artist.origin 5509

1461 film.person_or_entity_appearing_in_film.film 4710

1394 music.artist.origin 5421

1360 film.actor.film 6348

636 film.actor.film 7982

636 film.person_or_entity_appearing_in_film.film 4631

1378 film.person_or_entity_appearing_in_film.film 6677

152 people.person.place_of_birth 4616

3321 film.person_or_entity_appearing_in_film.film 8033

2302 music.artist.origin 4852

693 people.person.place_of_birth 4496

2110 people.person.place_of_birth 6019

2658 film.person_or_entity_appearing_in_film.film 8046

638 film.actor.film 8047

1545 event.agent.performance 8048

3132 film.person_or_entity_appearing_in_film.film 8049

2449 music.musician.instruments_played 6942

3034 music.artist.origin 5430

1750 film.person_or_entity_appearing_in_film.film 8050

1358 film.person_or_entity_appearing_in_film.film 8051

1776 film.person_or_entity_appearing_in_film.film 4494

2703 music.artist.origin 4057

570 music.artist.origin 3864

1157 film.actor.film 6158

379 award.nominee.award_nominations 8052

379 film.actor.film 7606

1633 film.person_or_entity_appearing_in_film.film 8053

2362 film.person_or_entity_appearing_in_film.film 5525

1720 film.writer.film 7362

775 music.artist.origin 3972

662 film.person_or_entity_appearing_in_film.film 6360

2249 people.deceased_person.place_of_death 3932

51 film.person_or_entity_appearing_in_film.film 8054

2141 film.person_or_entity_appearing_in_film.film 8055

1833 film.actor.film 4623

67 film.actor.film 5193

205 film.person_or_entity_appearing_in_film.film 5011

2733 film.person_or_entity_appearing_in_film.film 8056

2125 film.person_or_entity_appearing_in_film.film 6327

1590 music.artist.origin 7407

1380 people.deceased_person.place_of_death 5243

2324 film.film.country 3961

1706 film.person_or_entity_appearing_in_film.film 8057

2544 music.artist.origin 4024

84 film.actor.film 5733

2080 film.person_or_entity_appearing_in_film.film 8058

3598 film.actor.film 7977

3598 film.person_or_entity_appearing_in_film.film 4226

3372 film.actor.film 6335

948 film.actor.film 5696

3318 music.artist.origin 4896

2913 film.person_or_entity_appearing_in_film.film 5320

357 music.musician.instruments_played 6942

1796 film.actor.film 6574

1601 film.person_or_entity_appearing_in_film.film 8059

2264 music.artist.origin 4975

2070 film.person_or_entity_appearing_in_film.film 8060

1211 film.person_or_entity_appearing_in_film.film 6438

2688 film.person_or_entity_appearing_in_film.film 4637

542 film.person_or_entity_appearing_in_film.film 8061

2460 film.actor.film 5911

466 film.person_or_entity_appearing_in_film.film 8062

1915 music.artist.origin 5125

3738 cvg.publisher.games_published 7044

3738 games.publisher.games_published 8063

901 film.actor.film 8064

3563 music.artist.origin 3859

2241 film.distributor.film 8065

2241 film.distributor.film 4129

2241 film.distributor.film 6445

2241 film.production_company.film 8066

2241 film.production_company.film 8067

2241 film.production_company.film 7420

2241 film.production_company.film 4890

3350 film.person_or_entity_appearing_in_film.film 4911

1777 film.actor.film 7767

261 film.person_or_entity_appearing_in_film.film 5198

387 film.actor.film 6279

2880 film.person_or_entity_appearing_in_film.film 8068

3451 music.artist.origin 3864

643 film.actor.film 8069

2678 film.person_or_entity_appearing_in_film.film 8070

3780	people.person.place_of_birth	6594

3242	film.producer.film	4569

2148	film.person_or_entity_appearing_in_film.film	4699

798 music.artist.origin	4376

1970	music.artist.origin	3846

2852	music.artist.origin	6487

20	music.artist.origin	3864

1662	film.person_or_entity_appearing_in_film.film	8071

3384	film.actor.film	8072

2934	music.artist.origin	4174

633 film.person_or_entity_appearing_in_film.film	4759

1473	film.person_or_entity_appearing_in_film.film	8073

1473	film.person_or_entity_appearing_in_film.film	6692

645 film.actor.film	8074

84	film.actor.film	8075

3129	music.artist.origin	4380

584 film.actor.film	8076

584 film.person_or_entity_appearing_in_film.film	5918

2813	film.person_or_entity_appearing_in_film.film	5754

1888	film.person_or_entity_appearing_in_film.film	8077

2318	music.artist.origin	4053

3262	music.artist.origin	8078

2636	film.actor.film	8079

2254	music.artist.origin	3864

2437	film.actor.film	8080

1211	film.person_or_entity_appearing_in_film.film	8081

3738	games.publisher.games_published	5427

1356	film.person_or_entity_appearing_in_film.film	6784

311 music.artist.origin	4635

1040	film.actor.film	7384

2241 film.distributor.film 8082

2241 film.distributor.film 8083

2241 film.distributor.film 5599

2241 film.production_company.film 6379

1936 film.person_or_entity_appearing_in_film.film 7448

2487 film.person_or_entity_appearing_in_film.film 8084

2200 film.person_or_entity_appearing_in_film.film 5310

1360 film.person_or_entity_appearing_in_film.film 6645

2731 film.actor.film 4743

406 film.actor.film 5088

673 film.person_or_entity_appearing_in_film.film 8085

521 film.person_or_entity_appearing_in_film.film 8086

3222 music.artist.origin 4419

3049 film.person_or_entity_appearing_in_film.film 6422

951 film.actor.film 5267

3078 music.artist.origin 3864

3027 music.artist.origin 4157

2154 music.artist.origin 5623

166 film.actor.film 8015

638 people.person.place_of_birth 6827

923 film.person_or_entity_appearing_in_film.film 5725

1527 music.artist.origin 4214

1750 film.person_or_entity_appearing_in_film.film 8087

1142 film.actor.film 8088

617 music.artist.origin 3864

2635 film.person_or_entity_appearing_in_film.film 7397

2611 music.artist.origin 3966

2494 film.actor.film 6247

1720 film.director.film4165

492 film.person_or_entity_appearing_in_film.film 8089

3569 film.person_or_entity_appearing_in_film.film 6160

2646 music.artist.origin 4362

3494 music.artist.origin 4183

1032 film.person_or_entity_appearing_in_film.film 5206

793 film.actor.film 6865

2998 film.actor.film 8090

1711 film.actor.film 8091

66 film.actor.film 8092

84 film.person_or_entity_appearing_in_film.film 8075

2625 music.artist.origin 7433

2214 film.actor.film 8093

1079 film.editor.film 7671

3498 film.person_or_entity_appearing_in_film.film 4766

2972 film.person_or_entity_appearing_in_film.film 7808

2073 music.artist.origin 4183

49 film.person_or_entity_appearing_in_film.film 7676

3301 people.person.place_of_birth 8094

79 film.actor.film 8095

3588 film.person_or_entity_appearing_in_film.film 4780

962 music.artist.origin 4456

2867 film.person_or_entity_appearing_in_film.film 8096

2241 film.distributor.film 8097

2241 film.distributor.film 8098

2241 film.production_company.film 7571

2241 film.production_company.film 7146

2241 film.production_company.film 6228

1624 film.person_or_entity_appearing_in_film.film 8099

2511 music.artist.origin 7683

2322 music.artist.origin 4391

387 film.actor.film 4073

434 film.actor.film 8100

3597 music.artist.origin 8101

3049 film.person_or_entity_appearing_in_film.film 8102

3579 film.actor.film 6991

2251 music.artist.origin 4094

3099 event.agent.performance 4898

2110 music.artist.origin 6019

923 film.actor.film 8103

2343 film.person_or_entity_appearing_in_film.film 8104

206 film.actor.film 5978

1985 film.actor.film 8105

2257 music.artist.origin 3982

735 people.person.place_of_birth 5563

567 film.person_or_entity_appearing_in_film.film 8106

2635 film.actor.film 6049

1157 film.director.film 4052

379 film.actor.film 4004

379 film.actor.film 8107

2362 film.person_or_entity_appearing_in_film.film 7830

785 film.person_or_entity_appearing_in_film.film 4168

1889 film.person_or_entity_appearing_in_film.film 7339

3827 film.actor.film 8108

1156 music.artist.origin 7479

1739 music.artist.origin 4456

3020 film.actor.film 4164

1833 music.producer.tracks_produced 8109

912 film.person_or_entity_appearing_in_film.film 5323

2990 people.person.place_of_birth 5208

2470 music.artist.origin 4537

1813 film.person_or_entity_appearing_in_film.film 8110

8 music.artist.origin 8111

2314 music.artist.origin 3944

3372 people.person.place_of_birth 6463

1704 film.person_or_entity_appearing_in_film.film 5016

2848 film.film.language 8112

1181 film.actor.film 7869

2636 film.actor.film 3983

2636 film.person_or_entity_appearing_in_film.film 8113

679 film.actor.film 6231

1252 film.person_or_entity_appearing_in_film.film 8114

373 film.person_or_entity_appearing_in_film.film 4829

484 film.actor.film 7939

3738 cvg.publisher.games_published 8115

867 music.artist.origin 4294

234 music.artist.origin 3864

2241 film.distributor.film 8116

2241 film.distributor.film 8117

2241 film.distributor.film 8118

2241 film.distributor.film 8119

2241 film.production_company.film 8117

2241 film.production_company.film 5468

2241 film.production_company.film 7596

3647 music.artist.origin 3895

451 award.competitor.award_nominations 4469

33 music.artist.genre 4280

1794 people.person.place_of_birth 3864

2471 music.artist.origin 4583

2126 film.person_or_entity_appearing_in_film.film 7020

3486 film.set_designer.film 7425

1360 film.actor.film 7903

1651 music.musician.instruments_played 4349

2677 film.actor.film 8120

2766 film.person_or_entity_appearing_in_film.film 8121

1817 film.actor.film 8122

3226 film.person_or_entity_appearing_in_film.film 7391

2999 film.person_or_entity_appearing_in_film.film 5360

2519 people.person.place_of_birth 8123

1931 people.person.place_of_birth 5227

2373 film.actor.film 7022

1474 film.actor.film 7665

2962 film.actor.film 8124

2658 film.actor.film 4706

2658 film.actor.film 8046

849 people.person.place_of_birth 8125

2312 film.actor.film 8126

56 film.person_or_entity_appearing_in_film.film 6156

206 film.person_or_entity_appearing_in_film.film 8127

997 film.person_or_entity_appearing_in_film.film 8128

1142 film.person_or_entity_appearing_in_film.film 8129

383 film.person_or_entity_appearing_in_film.film 5051

3351 film.person_or_entity_appearing_in_film.film 7179

3384 film.actor.film 7669

598 film.film.actor 5877

2097 film.person_or_entity_appearing_in_film.film 4168

3827 film.actor.film 8130

67 film.actor.film 8131

3548 film.person_or_entity_appearing_in_film.film 5959

1988 music.artist.origin 4471

2344 film.person_or_entity_appearing_in_film.film 8132

1910 film.film.genre 8133

3733 film.person_or_entity_appearing_in_film.film 5317

1079 film.cinematographer.film7671

1194 music.artist.origin 3849

1039 film.person_or_entity_appearing_in_film.film 6837

2636 film.actor.film 8134

160 film.actor.film 8135

3289 people.person.place_of_birth 4391

79 film.actor.film 6970

771 film.person_or_entity_appearing_in_film.film 7752

484 film.person_or_entity_appearing_in_film.film 8136

3738 games.publisher.games_published 8137

1436 music.album.genre 8138

1872 film.actor.film 8139

2241 film.distributor.film 3983

2241 film.distributor.film 8140

2241 film.distributor.film 6755

2241 film.distributor.film 8141

988 film.actor.film 4629

3348 music.artist.origin 4454

451 award.nominee.award_nominations 6034

2487 film.person_or_entity_appearing_in_film.film 8142

1459 music.artist.origin 5357

652 music.artist.origin 4852

3321 film.actor.film 8143

3321 film.person_or_entity_appearing_in_film.film 3868

582 music.artist.origin 4057

2373 film.actor.film 8144

3390 film.person_or_entity_appearing_in_film.film 8145

1474 film.actor.film 8146

2974 music.artist.origin 6056

3132 film.actor.film 8147

56 film.person_or_entity_appearing_in_film.film 7125

2130 film.person_or_entity_appearing_in_film.film 4823

206 film.person_or_entity_appearing_in_film.film 8148

1358 film.actor.film 8051

1358 film.person_or_entity_appearing_in_film.film 7208

2635 film.person_or_entity_appearing_in_film.film 8149

3831 people.person.place_of_birth 3883

3116 people.person.place_of_birth 4471

2040 film.actor.film 8150

2097 film.actor.film 4168

3827 film.actor.film 6803

992 music.artist.origin 4053

3317 film.person_or_entity_appearing_in_film.film 8151

3370 music.artist.origin 4057

435 people.person.place_of_birth 4693

3215 film.film.rating 7328

279 film.person_or_entity_appearing_in_film.film 8152

3295 film.person_or_entity_appearing_in_film.film 8153

2080 film.actor.film 8154

686 music.artist.origin 3864

789 film.person_or_entity_appearing_in_film.film 7584

928 music.artist.origin 4456

2913 film.person_or_entity_appearing_in_film.film 8155

1469 film.person_or_entity_appearing_in_film.film 3909

1469 film.person_or_entity_appearing_in_film.film 7674

414 music.artist.origin 4195

960 music.artist.origin 4380

70 film.actor.film 7190

1204 film.actor.film 6499

2095	film.actor.film	8156
2657	people.person.place_of_birth	4195
2636	film.actor.film	8157
753	music.artist.origin	3846
2122	film.producer.film	7282
1145	music.musician.instruments_played	3959
715	film.person_or_entity_appearing_in_film.film	8158
790	people.person.place_of_birth	4347
484	film.actor.film	8136
2377	film.director.film	4390
2867	film.actor.film	8159
2241	film.production_company.film	8160
2241	film.production_company.film	7649
2241	film.production_company.film	8161
795	film.actor.film	5137
340	music.artist.origin	3966
1762	film.person_or_entity_appearing_in_film.film	6232
1780	film.actor.film	8162
2126	film.actor.film	7574
2731	film.actor.film	4966
1806	film.actor.film	6612
3002	film.actor.film	5478
3509	music.artist.track	5099
2490	music.artist.origin	6564
996	music.artist.origin	3849
3095	people.person.place_of_birth	3972
81	people.person.place_of_birth	4195
1524	music.artist.origin	4191
601	film.person_or_entity_appearing_in_film.film	7300
1962	film.actor.film	6044

959 award.nominee.award_nominations 8163

735 music.artist.album 6035

1662 film.person_or_entity_appearing_in_film.film 8164

2417 people.deceased_person.place_of_death 4174

671 music.musician.instruments_played 5802

3307 film.actor.film 8165

1157 music.artist.origin 3945

2494 film.actor.film 8166

2418 people.person.place_of_birth 4362

2681 people.deceased_person.place_of_death 8094

548 film.actor.film 6623

3827 film.actor.film 5946

3317 film.person_or_entity_appearing_in_film.film 8167

2444 music.artist.origin 5403

1054 music.artist.origin 4272

2786 film.person_or_entity_appearing_in_film.film 7130

3054 people.person.place_of_birth 4195

2620 music.artist.origin 4057

67 film.person_or_entity_appearing_in_film.film 8168

2359 film.actor.film 8169

2603 film.actor.film 8170

1182 music.artist.origin 5227

512 music.artist.track 5169

1187 film.actor.film 8171

3396 film.person_or_entity_appearing_in_film.film 7437

1973 people.deceased_person.place_of_death 3947

976 film.person_or_entity_appearing_in_film.film 7464

2679 film.actor.film 8172

2913 film.actor.film 7513

1306 film.person_or_entity_appearing_in_film.film 8173

2636 film.actor.film 8174

2074 music.artist.origin 3972

2597 film.actor.film 6174

1211 film.actor.film 5822

3745 film.person_or_entity_appearing_in_film.film 6370

79 film.actor.film 7379

919 film.person_or_entity_appearing_in_film.film 8175

602 film.person_or_entity_appearing_in_film.film 4846

2411 film.person_or_entity_appearing_in_film.film 8176

1154 film.actor.film 7519

2566 music.artist.origin 4217

3243 film.film.language 4076

2867 film.person_or_entity_appearing_in_film.film 8159

2241 film.distributor.film 8177

2241 film.distributor.film 8178

2241 film.production_company.film 8179

2241 film.production_company.film 8180

2331 film.person_or_entity_appearing_in_film.film 5842

2232 film.actor.film 8181

723 music.artist.origin 4024

1624 film.person_or_entity_appearing_in_film.film 5751

1624 film.person_or_entity_appearing_in_film.film 7096

2487 film.actor.film 8142

2487 film.person_or_entity_appearing_in_film.film 6515

1768 film.person_or_entity_appearing_in_film.film 6728

1556 film.person_or_entity_appearing_in_film.film 6419

2184 film.actor.film 8182

2493 film.actor.film 8183

3000 film.actor.film 8184

3509 music.artist.track 4532

468 people.person.place_of_birth 7541

3321 film.actor.film 8185

742 film.person_or_entity_appearing_in_film.film 7176

2556 film.actor.film 5694

2630 film.actor.film 4641

3390 film.actor.film 4900

2711 film.actor.film 7267

2554 film.person_or_entity_appearing_in_film.film 4385

1260 film.actor.film 5416

3038 film.person_or_entity_appearing_in_film.film 8186

735 film.actor.film 7854

2635 film.person_or_entity_appearing_in_film.film 8187

3307 film.actor.film 8188

2930 music.artist.origin 3864

614 music.artist.origin 6797

818 film.actor.film 8189

3287 film.person_or_entity_appearing_in_film.film 6459

813 music.musician.instruments_played 4349

1041 film.person_or_entity_appearing_in_film.film 7653

3020 film.person_or_entity_appearing_in_film.film 5677

1748 film.actor.film 7246

3788 film.actor.film 5059

235 event.agent.performance 4949

3133 film.actor.film 4865

759 music.artist.origin 4714

263 music.artist.origin 4454

647 music.artist.origin 4057

66 film.person_or_entity_appearing_in_film.film 8092

2080 film.person_or_entity_appearing_in_film.film 8154

3598 film.actor.film 8190

3372 film.person_or_entity_appearing_in_film.film 8191

93 music.artist.origin 4442

893 film.person_or_entity_appearing_in_film.film 8192

3158 film.person_or_entity_appearing_in_film.film 5893

1616 music.musician.instruments_played 4349

3498 film.person_or_entity_appearing_in_film.film 4726

80 music.artist.track 7135

1773 film.actor.film 3886

3120 film.actor.film 7314

476 music.artist.origin 6405

1453 film.actor.film 7252

2095 film.person_or_entity_appearing_in_film.film 8156

437 film.person_or_entity_appearing_in_film.film 8193

3289 film.actor.film 4494

2460 film.person_or_entity_appearing_in_film.film 8194

2460 film.person_or_entity_appearing_in_film.film 7871

2246 film.actor.film 7406

3366 music.artist.origin 3864

484 film.person_or_entity_appearing_in_film.film 8195

1154 film.actor.film 6145

2559 music.artist.origin 4455

1787 film.person_or_entity_appearing_in_film.film 6844

2867 film.person_or_entity_appearing_in_film.film 5775

2241 film.distributor.film 8196

2241 film.distributor.film 7261

2241 film.production_company.film 8082

2241 film.production_company.film 4985

293 music.artist.origin 4294

2558 music.artist.origin 6095

622 film.actor.film 6234

2443 music.artist.origin 5238

3031 film.person_or_entity_appearing_in_film.film 3935

2472 music.artist.origin 5845

1806 film.actor.film 4519

2219 music.artist.origin 4455

2015 music.artist.origin 7615

1059 film.actor.film 8197

1357 film.person_or_entity_appearing_in_film.film 7496

3321 film.person_or_entity_appearing_in_film.film 7967

3321 film.person_or_entity_appearing_in_film.film 5345

1377 people.person.place_of_birth 5161

232 film.person_or_entity_appearing_in_film.film 7710

1816 film.actor.film 8198

2962 film.person_or_entity_appearing_in_film.film 8124

1637 music.artist.album 4338

851 people.deceased_person.place_of_death 4094

1358 film.actor.film 5642

717 people.person.profession 4221

1157 film.actor.film 8199

1409 type.object.key 1409

1943 people.person.place_of_birth 4552

115 film.person_or_entity_appearing_in_film.film 8200

2362 film.person_or_entity_appearing_in_film.film 4823

785 film.actor.film 4168

1989 film.person_or_entity_appearing_in_film.film 8201

1297 music.artist.origin 8202

3035 film.person_or_entity_appearing_in_film.film 7632

2050 film.actor.film 5693

2325 music.artist.origin 3864

1779 music.artist.origin 8203

3339 music.artist.origin 4362

1473 film.actor.film 8204

1473 film.person_or_entity_appearing_in_film.film 8205

1692 film.person_or_entity_appearing_in_film.film 5948

3690 music.artist.origin 4436

2737 film.actor.film 7889

1209 film.person_or_entity_appearing_in_film.film 4494

1239 music.artist.origin 5826

3030 film.person_or_entity_appearing_in_film.film 5952

789 film.person_or_entity_appearing_in_film.film 8206

893 people.person.place_of_birth 4768

458 award.competitor.award_nominations 7250

510 music.artist.origin 8207

2562 film.actor.film 8208

2562 film.actor.film 4972

75 film.actor.film 6837

47 film.person_or_entity_appearing_in_film.film 6369

2070 film.person_or_entity_appearing_in_film.film 8209

3108 people.person.place_of_birth 4171

3289 film.person_or_entity_appearing_in_film.film 4494

3166 people.person.place_of_birth 3864

2460 film.actor.film 7345

2460 film.actor.film 8210

3366 film.actor.film 7517

2682 film.actor.film 7773

484 film.actor.film 8211

484 film.person_or_entity_appearing_in_film.film 7680

3738 cvg.publisher.games_published 5856

436 film.actor.film 8212

436 film.person_or_entity_appearing_in_film.film 4189

2241	film.distributor.film	7396
2241	film.distributor.film	7520
2241	film.distributor.film	7524
3276	film.actor.film	6669
591 film.actor.film	4304
2200	film.actor.film	5310
2261	film.person_or_entity_appearing_in_film.film	8213
2861	film.person_or_entity_appearing_in_film.film	5847
3321	film.actor.film	8214
2372	music.artist.album	5322
1089	film.actor.film	8215
2770	film.person_or_entity_appearing_in_film.film	5870
3048	film.actor.film	7014
2089	music.artist.origin	5106
3822	film.actor.film	3950
2904	film.actor.film	7679
2635	film.person_or_entity_appearing_in_film.film	4428
2635	film.person_or_entity_appearing_in_film.film	8216
2635	film.person_or_entity_appearing_in_film.film	4904
186 film.actor.film	8217
1719	film.actor.film	4168
1720	film.writer.film	6084
768 music.artist.origin	3946
1505	film.person_or_entity_appearing_in_film.film	8218
1641	music.artist.origin	4150
3455	music.artist.origin	3864
3494	music.artist.album	8219
1525	film.actor.film	5840
67	film.actor.film	6207
2359	film.person_or_entity_appearing_in_film.film	8220

1473 film.actor.film 6492

3737 film.actor.film 8221

1587 music.artist.origin 7588

3215 film.film.actor 8222

3580 people.person.place_of_birth 6831

893 film.actor.film 8223

3498 film.actor.film 4735

3498 film.person_or_entity_appearing_in_film.film 7510

2913 film.person_or_entity_appearing_in_film.film 6888

2914 film.actor.film 8224

628 film.person_or_entity_appearing_in_film.film 8225

414 film.actor.film 4846

1761 music.artist.origin 7309

2562 film.person_or_entity_appearing_in_film.film 8226

1198 music.artist.origin 4357

1846 film.actor.film 7220

1299 film.person_or_entity_appearing_in_film.film 8227

2460 film.actor.film 8194

2460 film.actor.film 8228

2460 film.actor.film 8229

3738 cvg.publisher.games_published 7940

3738 games.publisher.games_published 6607

3738 games.publisher.games_published 4883

436 film.actor.film 7441

436 film.actor.film 7916

901 film.person_or_entity_appearing_in_film.film 8230

3248 film.person_or_entity_appearing_in_film.film 8231

2241 film.distributor.film 6147

2241 film.production_company.film 8232

2241 film.production_company.film 4624

2241 film.production_company.film 4193

2060 music.artist.origin 8233

797 music.artist.origin 4362

2806 film.actor.film 8234

777 film.person_or_entity_appearing_in_film.film 8235

2126 film.actor.film 8236

2126 film.actor.film 8237

3012 music.artist.origin 4362

3031 film.actor.film 8238

1806 film.person_or_entity_appearing_in_film.film 8239

1278 people.person.profession 4221

3448 music.artist.origin 5977

2970 people.person.profession 4221

241 film.person_or_entity_appearing_in_film.film 8240

2651 music.artist.origin 4380

2101 film.actor.film 8241

959 music.artist.album 7408

2833 film.actor.film 4049

540 film.person_or_entity_appearing_in_film.film 3974

2494 film.person_or_entity_appearing_in_film.film 8166

3827 film.actor.film 8242

2736 music.artist.origin 7285

26 music.artist.origin 3864

2683 film.actor.film 5424

633 film.actor.film 7303

1141 film.person_or_entity_appearing_in_film.film 8243

2389 film.actor.film 6093

3075 music.artist.origin 4380

2737 film.person_or_entity_appearing_in_film.film 7160

2737 film.person_or_entity_appearing_in_film.film 5492

3626	film.person_or_entity_appearing_in_film.film	6037

3598	film.person_or_entity_appearing_in_film.film	8244

976 people.person.place_of_birth	8245

2102	music.artist.origin	5139

789 film.actor.film	5625

2726	film.person_or_entity_appearing_in_film.film	3907

393 film.actor.film	7311

3094	film.actor.film	8246

2555	music.artist.album	8247

1208	film.person_or_entity_appearing_in_film.film	5806

187 event.agent.performance	4099

2636	film.person_or_entity_appearing_in_film.film	8174

3355	film.person_or_entity_appearing_in_film.film	5667

2070	film.person_or_entity_appearing_in_film.film	8248

1355	type.object.subject_key	1355

2819	film.actor.film	5701

3217	people.person.place_of_birth	4057

734 music.artist.origin	4362

1277	film.person_or_entity_appearing_in_film.film	5289

3452	film.person_or_entity_appearing_in_film.film	8249

2903	music.artist.origin	8250

223 film.actor.film	5544

3113	music.musician.instruments_played	8251

2360	film.actor.film	8252

1936	film.person_or_entity_appearing_in_film.film	8253

3583	people.person.place_of_birth	4391

2038	film.person_or_entity_appearing_in_film.film	8254

1859	music.artist.origin	4183

1613	music.artist.origin	4057

576 music.artist.origin	4010

2677	film.person_or_entity_appearing_in_film.film	8120

2766	people.person.place_of_birth	5161

3231	film.person_or_entity_appearing_in_film.film	8255

1954	film.actor.film	6823

164 film.actor.film	7969

385 film.person_or_entity_appearing_in_film.film	5308

3390	film.actor.film	8256

3390	film.actor.film	7734

166 film.actor.film	7924

1474	film.actor.film	7886

241 film.person_or_entity_appearing_in_film.film	8257

1428	cvg.computer_videogame.publisher	8258

3815	music.artist.origin	5420

533 film.actor.film	8259

900 film.actor.film	8260

422 film.actor.film	5766

2076	film.actor.film	8261

2635	film.actor.film	8262

2635	film.person_or_entity_appearing_in_film.film	8263

2635	film.person_or_entity_appearing_in_film.film	8264

1157	film.person_or_entity_appearing_in_film.film	8265

2123	music.artist.origin	3961

115 film.actor.film	7460

1044	film.person_or_entity_appearing_in_film.film	4811

1379	people.deceased_person.place_of_death	5243

2518	music.artist.origin	4057

946 music.artist.origin	4150

2359	film.actor.film	7865

3792	film.actor.film	5292

1129	music.artist.origin	4013

2337 film.person_or_entity_appearing_in_film.film 4962

2337 film.person_or_entity_appearing_in_film.film 8266

861 film.actor.film 5241

136 film.actor.film 7583

2135 music.artist.origin 3864

3229 film.person_or_entity_appearing_in_film.film 6335

103 people.person.place_of_birth 4596

3598 film.actor.film 4969

3598 film.person_or_entity_appearing_in_film.film 8267

1079 film.director.film 4669

3797 film.person_or_entity_appearing_in_film.film 6535

1566 film.actor.film 6335

357 film.actor.film 6792

1306 film.actor.film 8173

1846 film.person_or_entity_appearing_in_film.film 5849

437 film.actor.film 5065

1052 music.artist.origin 4272

3510 music.artist.origin 4909

3804 music.artist.origin 4876

2070 film.person_or_entity_appearing_in_film.film 6333

1299 film.actor.film 7703

2460 film.actor.film 5457

2460 film.person_or_entity_appearing_in_film.film 4827

184 film.actor.film 8268

3758 music.artist.origin 3947

901 award.winner.awards_won 8269

901 film.person_or_entity_appearing_in_film.film 8064

3673 music.artist.origin 6554

1249 film.person_or_entity_appearing_in_film.film 8270

1463 type.object.subject_key 1463

2403	event.agent.performance	4548

2241	film.distributor.film	8271

2241	film.distributor.film	7597

2241	film.distributor.film	8272

2241	film.distributor.film	8273

2241	film.production_company.film	6752

2241	film.production_company.film	6643

490 film.actor.film	6447

2849	music.artist.origin	6261

392 film.person_or_entity_appearing_in_film.film	4792

258 film.actor.film	7636

1360	film.person_or_entity_appearing_in_film.film	5044

1171	music.artist.origin	6487

1619	film.person_or_entity_appearing_in_film.film	8274

2274	people.deceased_person.place_of_death	4175

3648	film.actor.film	5724

3132	film.actor.film	7827

3240	music.artist.origin	4357

1955	film.actor.film	5397

383 film.actor.film	8275

2904	people.person.place_of_birth	3859

2635	film.actor.film	8276

2635	film.person_or_entity_appearing_in_film.film	8277

115 film.actor.film	8278

3661	film.actor.film	3886

813 people.deceased_person.place_of_death	4057

190 film.person_or_entity_appearing_in_film.film	8279

1897	music.artist.origin	4376

1032	film.person_or_entity_appearing_in_film.film	4029

67	film.person_or_entity_appearing_in_film.film	8036

1117	music.artist.origin	3849

1473	film.actor.film	8280

1766	film.actor.film	4663

3725	music.musician.instruments_played	5619

2959	music.album.genre	8281

645 film.person_or_entity_appearing_in_film.film	7247

645 film.person_or_entity_appearing_in_film.film	6461

377 film.actor.film	6695

1104	music.artist.origin	4963

3598	film.actor.film	8282

1434	film.actor.film	8283

1572	music.artist.origin	4332

3344	film.person_or_entity_appearing_in_film.film	8284

3344	film.person_or_entity_appearing_in_film.film	5020

1103	music.artist.origin	4199

2338	film.actor.film	5495

611 music.artist.origin	4214

2784	film.actor.film	4786

3320	people.person.place_of_birth	4053

628 film.actor.film	5066

2636	film.actor.film	8285

2743	film.person_or_entity_appearing_in_film.film	6539

1458	film.actor.film	5248

2760	music.artist.origin	3888

2719	music.artist.album	5738

484 film.person_or_entity_appearing_in_film.film	7896

3738	cvg.publisher.games_published	7255

2400	film.person_or_entity_appearing_in_film.film	5918

1003	film.person_or_entity_appearing_in_film.film	5638

2487	film.actor.film	4096

1892 music.musician.instruments_played 6308

2922 people.person.place_of_birth 4204

1767 film.actor.film 8286

1583 music.musician.instruments_played 3959

643 film.person_or_entity_appearing_in_film.film 6241

1472 music.artist.origin 5270

2741 film.actor.film 8287

757 film.person_or_entity_appearing_in_film.film 4645

702 music.album.genre 4848

2999 music.musician.instruments_played 8251

3579 film.actor.film 4421

206 film.person_or_entity_appearing_in_film.film 4647

997 film.actor.film 7178

383 film.actor.film 8288

142 film.person_or_entity_appearing_in_film.film 7879

2635 film.actor.film 6524

1157 film.person_or_entity_appearing_in_film.film 7183

115 film.actor.film 7487

2984 people.person.place_of_birth 3947

1886 film.person_or_entity_appearing_in_film.film 4054

1633 film.actor.film 8289

2609 music.artist.origin 4217

2451 music.artist.origin 6557

2371 film.actor.film 8290

1197 people.person.place_of_birth 6829

3317 film.actor.film 8291

257 music.artist.origin 5265

1833 film.person_or_entity_appearing_in_film.film 8292

2804 film.person_or_entity_appearing_in_film.film 5315

2359 film.actor.film 7461

2370	film.person_or_entity_appearing_in_film.film	8293

2467	music.artist.origin	4057

3737	film.person_or_entity_appearing_in_film.film	8221

2337	film.actor.film	5760

3725	film.actor.film	5485

2276	film.actor.film	7838

2014	film.film.language	8112

3295	film.person_or_entity_appearing_in_film.film	8294

3598	film.actor.film	8295

3598	film.actor.film	6531

1393	music.artist.origin	3864

2914	film.actor.film	3970

458 music.artist.album	8296

1465	film.person_or_entity_appearing_in_film.film	5889

1204	film.person_or_entity_appearing_in_film.film	7041

3355	film.person_or_entity_appearing_in_film.film	3848

1659	people.person.place_of_birth	4191

1308	film.person_or_entity_appearing_in_film.film	4080

3738	games.publisher.games_published	6178

1249	film.person_or_entity_appearing_in_film.film	4740

2867	film.actor.film	4569

3836	music.artist.album	8247

2241	film.distributor.film	8297

2241	film.distributor.film	4247

2241	film.distributor.film	8298

2241	film.production_company.film	6340

1762	film.actor.film	8299

2126	film.actor.film	6235

2126	film.person_or_entity_appearing_in_film.film	7623

778 music.artist.origin	5213

1767 people.person.place_of_birth 3846

1842 film.actor.film 8300

3765 music.artist.origin 4362

2648 music.musician.instruments_played 4349

2731 film.person_or_entity_appearing_in_film.film 8301

2643 film.actor.film 8302

1806 film.person_or_entity_appearing_in_film.film 4581

776 film.person_or_entity_appearing_in_film.film 6928

1284 music.artist.origin 4362

983 music.musician.instruments_played 4349

1378 film.actor.film 6245

157 type.object.subject_key 157

814 film.actor.film 8303

814 film.actor.film 8304

3052 film.person_or_entity_appearing_in_film.film 7480

3807 music.artist.origin 8305

2506 music.artist.origin 4453

3360 film.actor.film 8306

2711 film.actor.film 8307

3048 film.person_or_entity_appearing_in_film.film 7123

2296 film.person_or_entity_appearing_in_film.film 5562

2218 music.artist.origin 4121

2997 music.artist.album 8308

3088 film.actor.film 6407

2107 film.person_or_entity_appearing_in_film.film 7181

2635 film.person_or_entity_appearing_in_film.film 8309

2635 film.person_or_entity_appearing_in_film.film 8310

2808 film.actor.film 4462

3307 film.person_or_entity_appearing_in_film.film 8188

3011 music.artist.origin 4957

428 people.person.place_of_birth 3972

3778 film.actor.film 6322

2125 people.deceased_person.place_of_death 5916

793 film.actor.film 4802

124 music.artist.origin 4471

2324 film.film.writer 4325

91 film.actor.film 7051

3040 film.person_or_entity_appearing_in_film.film 6365

792 music.producer.tracks_produced 8311

1711 people.person.place_of_birth 4013

645 film.person_or_entity_appearing_in_film.film 8074

3249 music.artist.origin 4391

3498 film.actor.film 8312

672 film.person_or_entity_appearing_in_film.film 6404

672 film.person_or_entity_appearing_in_film.film 8313

2830 film.person_or_entity_appearing_in_film.film 8314

1469 music.producer.tracks_produced 8315

1773 film.actor.film 8316

1888 people.person.place_of_birth 4133

1341 music.artist.origin 3864

2475 film.actor.film 8317

335 music.artist.origin 4454

2070 film.actor.film 6813

160 film.person_or_entity_appearing_in_film.film 4340

3590 music.artist.origin 4416

1315 film.person_or_entity_appearing_in_film.film 8318

527 film.actor.film 6197

3738 cvg.publisher.games_published 7815

3738 games.publisher.games_published 7721

1154 film.person_or_entity_appearing_in_film.film 4886

2430 film.actor.film 5254

1356 film.person_or_entity_appearing_in_film.film 8319

2867 film.actor.film 4132

940 music.artist.origin 8320

2241 film.production_company.film 5434

2241 film.production_company.film 8297

2241 film.production_company.film 8321

419 film.actor.film 5885

3139 music.artist.origin 4013

1624 film.person_or_entity_appearing_in_film.film 8322

2144 people.person.place_of_birth 3942

894 film.actor.film 8323

2732 film.actor.film 4136

3031 film.actor.film 8324

2346 film.actor.film 8325

2184 film.actor.film 6614

2184 film.person_or_entity_appearing_in_film.film 8182

1536 film.person_or_entity_appearing_in_film.film 4418

3605 music.artist.origin 4362

2585 people.person.place_of_birth 4272

2906 music.artist.origin 5559

3390 music.artist.album 4577

166 film.person_or_entity_appearing_in_film.film 8326

3279 film.actor.film 4682

1540 music.artist.origin 3855

1781 film.actor.film 4805

3507 film.person_or_entity_appearing_in_film.film 6799

2328 people.person.profession 4221

2635 film.actor.film 8264

2635 film.producer.film 7429

1968 music.artist.origin 4471

2161 award.nominee.award_nominations 8327

2220 music.artist.origin 4057

67 film.person_or_entity_appearing_in_film.film 4118

2079 film.person_or_entity_appearing_in_film.film 4916

1625 film.person_or_entity_appearing_in_film.film 4016

2454 music.musician.instruments_played 6942

192 music.artist.track 6949

377 film.person_or_entity_appearing_in_film.film 4604

3598 film.actor.film 8244

2217 film.person_or_entity_appearing_in_film.film 4823

3121 film.actor.film 8328

3280 film.actor.film 8329

3280 film.person_or_entity_appearing_in_film.film 8330

1601 film.person_or_entity_appearing_in_film.film 7372

128 award.competitor.awards_won 4554

1256 music.artist.origin 4707

568 film.person_or_entity_appearing_in_film.film 8331

2674 film.actor.film 8332

2460 film.actor.film 4458

79 film.person_or_entity_appearing_in_film.film 8333

3738 games.publisher.games_published 8334

2430 film.person_or_entity_appearing_in_film.film 7143

625 film.actor.film 8335

899 people.person.place_of_birth 4094

1040 type.object.key 1040

3297 film.actor.film 4802

451 award.nominee.award_nominations 8336

2782 music.artist.origin 4010

3031 film.person_or_entity_appearing_in_film.film 7920

2346 music.artist.origin 4768

643 film.person_or_entity_appearing_in_film.film 8337

2442 music.artist.origin 6505

3771 music.artist.origin 4552

434 film.actor.film 8338

538 film.actor.film 8339

2686 people.person.place_of_birth 6201

3059 music.artist.origin 3966

3321 film.actor.film 7823

807 people.person.place_of_birth 5213

742 music.artist.origin 6649

81 film.person_or_entity_appearing_in_film.film 8340

2658 film.person_or_entity_appearing_in_film.film 6191

126 music.artist.origin 4635

1962 film.person_or_entity_appearing_in_film.film 3977

1358 film.person_or_entity_appearing_in_film.film 5524

997 film.person_or_entity_appearing_in_film.film 8341

315 music.artist.album 7189

1569 film.actor.film 4328

2635 film.person_or_entity_appearing_in_film.film 8342

2635 film.person_or_entity_appearing_in_film.film 7685

1157 film.actor.film 8343

115 film.actor.film 7022

2356 music.artist.origin 4050

746 music.artist.album 6484

3189 music.artist.origin 5383

2147 film.person_or_entity_appearing_in_film.film 6807

664 music.artist.album 4112

2337 film.actor.film 5078

2990 film.actor.film 7365

3604	music.artist.origin	6494

3626	music.artist.origin	4455

2080	film.person_or_entity_appearing_in_film.film	8344

2916	film.person_or_entity_appearing_in_film.film	8345

3770	people.person.place_of_birth	6056

52	film.actor.film	5533

2972	film.actor.film	5321

1573	film.actor.film	8346

49	film.person_or_entity_appearing_in_film.film	7415

2460	film.actor.film	4926

3701	film.person_or_entity_appearing_in_film.film	8347

484	film.actor.film	7529

2241	film.distributor.film	4689

2241	film.distributor.film	4788

2241	film.distributor.film	8348

2241	film.production_company.film	4568

764	music.artist.origin	8349

1624	film.person_or_entity_appearing_in_film.film	7686

2487	film.actor.film	8350

108	music.artist.origin	4195

3779	people.person.place_of_birth	8351

538	film.actor.film	3941

555	music.artist.origin	4635

3459	people.person.place_of_birth	8125

2919	film.person_or_entity_appearing_in_film.film	6154

3633	film.actor.film	7026

1770	people.person.place_of_birth	3846

3164	music.artist.origin	5662

1157	film.person_or_entity_appearing_in_film.film	6998

1221	music.artist.album	7360

1633 film.person_or_entity_appearing_in_film.film 8352

410 film.actor.film 8353

623 people.deceased_person.place_of_death 6200

82 music.artist.origin 4010

3317 film.person_or_entity_appearing_in_film.film 5204

3020 film.person_or_entity_appearing_in_film.film 8354

67 film.actor.film 4802

1473 film.actor.film 5282

3133 film.actor.film 4866

3598 film.person_or_entity_appearing_in_film.film 5799

508 film.writer.film 4178

2259 film.actor.film 8355

3280 film.actor.film 8330

974 film.person_or_entity_appearing_in_film.film 6466

1082 film.actor.film 8356

1403 film.person_or_entity_appearing_in_film.film 7169

2689 film.person_or_entity_appearing_in_film.film 7749

366 film.actor.film 8357

366 film.person_or_entity_appearing_in_film.film 6719

1353 film.person_or_entity_appearing_in_film.film 8358

1774 film.actor.film 7678

484 film.person_or_entity_appearing_in_film.film 8359

484 film.person_or_entity_appearing_in_film.film 8211

3738 games.publisher.games_published 4186

1154 film.actor.film 7256

1154 film.actor.film 7817

1356 film.actor.film 8319

2867 film.actor.film 8360

2867 film.person_or_entity_appearing_in_film.film 7145

1463 award.competitor.award_nominations 8361

2241 film.production_company.film 8362

988 film.actor.film 5185

481 film.person_or_entity_appearing_in_film.film 7380

2666 music.artist.origin 7150

591 film.actor.film 6037

2463 people.person.place_of_birth 5662

3031 film.person_or_entity_appearing_in_film.film 5781

2184 film.person_or_entity_appearing_in_film.film 4073

538 film.person_or_entity_appearing_in_film.film 8363

2207 film.person_or_entity_appearing_in_film.film 5323

277 music.artist.origin 3864

2190 music.artist.origin 4701

238 people.person.place_of_birth 4199

892 film.actor.film 7528

1508 film.producer.film 8364

380 film.actor.film 8365

1348 film.actor.film 7457

2658 film.actor.film 6734

1705 film.actor.film 4846

1479 film.person_or_entity_appearing_in_film.film 5947

997 film.actor.film 5005

383 film.person_or_entity_appearing_in_film.film 8288

2635 film.actor.film 8216

2635 film.actor.film 8187

2635 film.person_or_entity_appearing_in_film.film 8366

3022 music.artist.origin 4701

1157 film.director.film 4906

1681 music.artist.origin 7124

1232 people.person.place_of_birth 4347

546 music.artist.origin 4199

1998	film.person_or_entity_appearing_in_film.film	8367
662	film.actor.film	6598
1153	film.actor.film	6527
879	film.person_or_entity_appearing_in_film.film	8368
1833	film.actor.film	7910
2708	film.person_or_entity_appearing_in_film.film	5058
3548	film.actor.film	5959
633	film.actor.film	8129
89	film.actor.film	8369
2990	film.actor.film	6059
895	film.person_or_entity_appearing_in_film.film	8370
412	music.artist.origin	7860
2998	film.person_or_entity_appearing_in_film.film	7913
893	film.actor.film	8192
3363	music.artist.origin	5656
2338	film.actor.film	8371
3498	film.person_or_entity_appearing_in_film.film	4383
3676	film.actor.film	3962
458	film.actor.film	5888
1773	film.actor.film	6497
1799	music.artist.origin	3961
2674	film.person_or_entity_appearing_in_film.film	8372
919	film.actor.film	8175
1579	film.person_or_entity_appearing_in_film.film	8373
602	film.person_or_entity_appearing_in_film.film	6865
3738	games.publisher.games_published	6507
782	music.musician.instruments_played	4349
119	music.producer.tracks_produced	8374
2241	film.distributor.film	7380
2241	film.distributor.film	7074

2241	film.production_company.film	8375
988 film.actor.film	8376
1267	music.artist.origin	4454
681 film.actor.film	8377
382 people.person.place_of_birth	4217
387 film.actor.film	5905
3175	music.artist.origin	4419
1772	film.actor.film	8378
157 music.producer.tracks_produced	7604
1191	film.actor.film	6931
1332	film.person_or_entity_appearing_in_film.film	4265
3279	film.person_or_entity_appearing_in_film.film	4007
2345	music.artist.origin	4024
2010	film.person_or_entity_appearing_in_film.film	6874
3132	film.person_or_entity_appearing_in_film.film	5847
3152	music.artist.origin	3986
1087	people.person.place_of_birth	7492
2904	film.actor.film	5682
3013	music.artist.origin	4526
410 film.actor.film	8379
785 film.actor.film	4922
254 film.actor.film	8380
155 film.person_or_entity_appearing_in_film.film	5879
662 film.actor.film	8381
1473	film.actor.film	6568
1910	film.film.language	3953
91	people.person.place_of_birth	3859
2137	people.person.place_of_birth	5139
2913	film.actor.film	6170
3476	people.person.place_of_birth	3984

2455 music.artist.origin 3897

2070 film.actor.film 7281

3347 music.artist.origin 4422

2131 film.actor.film 4411

1299 film.actor.film 8227

484 film.person_or_entity_appearing_in_film.film 4459

3738 cvg.publisher.games_published 6179

3738 games.publisher.games_published 4881

3225 film.actor.film 8382

1416 film.actor.film 3905

1002 film.person_or_entity_appearing_in_film.film 8383

455 film.actor.film 7116

2241 film.distributor.film 8066

2241 film.distributor.film 6313

2241 film.production_company.film 6548

2241 film.production_company.film 8384

2241 film.production_company.film 6377

2241 film.production_company.film 8273

2241 film.production_company.film 8385

1068 music.artist.origin 4635

3782 people.person.place_of_birth 4013

3276 people.person.place_of_birth 5756

1109 music.artist.origin 6215

2311 film.actor.film 3977

3789 award.winner.awards_won 8386

1360 film.actor.film 6240

406 music.artist.origin 8387

1806 film.person_or_entity_appearing_in_film.film 5918

1772 film.person_or_entity_appearing_in_film.film 8388

434 film.person_or_entity_appearing_in_film.film 8100

1664 film.person_or_entity_appearing_in_film.film 8389

3049 film.person_or_entity_appearing_in_film.film 7662

2952 music.musician.instruments_played 4349

3433 film.actor.film 7237

2664 music.artist.origin 8390

2312 film.actor.film 6826

3038 people.person.place_of_birth 5125

3507 music.artist.origin 4050

3570 music.artist.origin 7492

1142 people.person.place_of_birth 3952

2508 music.artist.origin 4057

2635 film.actor.film 4905

379 film.person_or_entity_appearing_in_film.film 8391

2670 film.actor.film 8392

1961 music.artist.origin 6361

1720 film.person_or_entity_appearing_in_film.film 4273

1720 film.writer.film 8393

3594 music.artist.album 7471

1153 film.person_or_entity_appearing_in_film.film 4181

2094 film.actor.film 4346

3548 film.actor.film 6326

2125 film.person_or_entity_appearing_in_film.film 6972

1473 film.person_or_entity_appearing_in_film.film 8394

2292 music.artist.origin 4294

2344 film.person_or_entity_appearing_in_film.film 5614

2673 people.deceased_person.place_of_death 3846

2270 film.actor.film 4225

279 film.actor.film 4125

192 music.artist.track 8395

2080 film.actor.film 5695

1885 film.actor.film 4228

1598 film.actor.film 4804

393 music.artist.origin 4521

1181 film.actor.film 4874

1846 film.actor.film 4411

2437 film.person_or_entity_appearing_in_film.film 8396

3185 film.actor.film 6917

2070 film.person_or_entity_appearing_in_film.film 5582

1895 film.actor.film 7283

445 film.actor.film 8397

445 people.person.place_of_birth 8320

790 film.person_or_entity_appearing_in_film.film 4082

3738 cvg.publisher.games_published 8398

436 film.person_or_entity_appearing_in_film.film 8399

3358 music.artist.origin 4294

2031 music.artist.origin 7675

791 people.person.place_of_birth 4094

2241 film.distributor.film 5337

2241 film.distributor.film 4968

1762 film.person_or_entity_appearing_in_film.film 4034

3489 music.artist.origin 5559

36 film.person_or_entity_appearing_in_film.film 8400

2244 film.film.genre 4062

2950 music.artist.origin 3961

1332 film.person_or_entity_appearing_in_film.film 6078

1540 film.person_or_entity_appearing_in_film.film 8401

1331 music.artist.album 8402

3132 film.actor.film 8403

3132 film.person_or_entity_appearing_in_film.film 8147

3132 music.artist.album 8404

1017	music.artist.origin	3870

997 film.actor.film	8405

959 film.actor.film	7209

1655	people.person.place_of_birth	5236

2820	music.artist.origin	4232

3188	music.artist.origin	5211

1006	award.nominee.award_nominations	8406

1633	film.person_or_entity_appearing_in_film.film	7028

2161	award.nominee.award_nominations	8407

492 film.person_or_entity_appearing_in_film.film	4061

3606	music.artist.origin	8408

633 people.deceased_person.place_of_death	5569

1692	film.actor.film	8409

2349	film.film.language	3953

1706	film.actor.film	5016

820 people.deceased_person.place_of_death	4057

2737	film.actor.film	8410

2080	film.actor.film	6131

3768	people.person.place_of_birth	4536

74	film.person_or_entity_appearing_in_film.film	8411

2637	film.film.writer	5214

3211	film.actor.film	5767

3244	film.actor.film	8412

2026	music.artist.origin	4332

736 film.person_or_entity_appearing_in_film.film	8413

70	film.person_or_entity_appearing_in_film.film	8414

1601	film.person_or_entity_appearing_in_film.film	8415

2892	film.actor.film	5990

1204	film.actor.film	8416

2636	film.actor.film	4973

2437 film.person_or_entity_appearing_in_film.film 8417

750 music.artist.origin 4825

3179 film.person_or_entity_appearing_in_film.film 6666

2460 film.actor.film 8418

1579 music.artist.origin 5213

3286 music.artist.origin 3864

484 film.person_or_entity_appearing_in_film.film 4880

1981 film.actor.film 8419

1249 film.actor.film 6503

2241 film.distributor.film 7444

2241 film.distributor.film 7818

2241 film.distributor.film 8420

2241 film.distributor.film 5537

2241 film.distributor.film 7962

2241 film.distributor.film 8201

2241 film.production_company.film 8421

2241 film.production_company.film 8422

2241 film.production_company.film 7469

2241 film.production_company.film 8423

481 people.person.place_of_birth 7624

3837 music.musician.instruments_played 5619

2806 music.artist.origin 4094

1780 film.actor.film 6514

2311 people.person.place_of_birth 5411

894 film.person_or_entity_appearing_in_film.film 6584

894 film.person_or_entity_appearing_in_film.film 8424

3165 film.person_or_entity_appearing_in_film.film 8425

3486 film.writer.film 7425

3780 film.actor.film 6646

1772 film.person_or_entity_appearing_in_film.film 6758

1357 film.actor.film 8426

3509 event.agent.performance 4949

3049 film.actor.film 8427

1468 film.actor.film 6733

1089 film.person_or_entity_appearing_in_film.film 8428

2032 music.artist.origin 5662

3132 film.person_or_entity_appearing_in_film.film 4355

1305 music.artist.origin 4294

3453 music.artist.origin 4195

1067 film.actor.film 6106

3038 film.actor.film 8186

799 music.artist.origin 4701

3384 film.actor.film 7241

1247 film.person_or_entity_appearing_in_film.film 4705

540 film.person_or_entity_appearing_in_film.film 8429

3111 film.person_or_entity_appearing_in_film.film 5567

2084 film.person_or_entity_appearing_in_film.film 8430

2724 film.person_or_entity_appearing_in_film.film 4538

276 music.artist.origin 4087

2050 film.person_or_entity_appearing_in_film.film 8431

2927 music.artist.origin 4013

1910 film.film.genre 8432

839 film.actor.film 5013

1441 film.person_or_entity_appearing_in_film.film 4720

789 film.person_or_entity_appearing_in_film.film 4802

1598 people.deceased_person.place_of_death 4658

672 film.person_or_entity_appearing_in_film.film 5579

3591 music.artist.album 8433

3094 film.person_or_entity_appearing_in_film.film 8246

611 music.artist.album 7964

151 award.competitor.award_nominations 8434

128 film.person_or_entity_appearing_in_film.film 8435

2096 film.person_or_entity_appearing_in_film.film 6579

2070 film.person_or_entity_appearing_in_film.film 6953

2070 people.person.place_of_birth 6860

2923 people.person.place_of_birth 5125

484 film.person_or_entity_appearing_in_film.film 5459

3738 cvg.publisher.games_published 8436

3738 games.publisher.games_published 8437

436 film.actor.film 8399

2566 film.actor.film 4054

1011 music.artist.origin 8438

2867 film.person_or_entity_appearing_in_film.film 5545

2068 event.agent.performance 8439

2380 film.actor.film 7182

1361 film.actor.film 6784

2654 music.artist.origin 3932

1360 film.actor.film 8440

2731 film.person_or_entity_appearing_in_film.film 8441

3049 film.person_or_entity_appearing_in_film.film 3871

2843 film.actor.film 5143

2956 tv.series_episode.director 7500

3522 music.artist.origin 4150

228 music.artist.origin 7849

1142 film.person_or_entity_appearing_in_film.film 8442

148 people.person.place_of_birth 8443

2635 film.actor.film 7581

379 film.actor.film 5202

1633 film.actor.film 7714

2316 music.album.genre 8138

273 music.artist.origin 4479

1153 film.actor.film 8444

51 film.actor.film 8054

588 music.artist.origin 4471

117 film.actor.film 4374

3412 film.film.actor 8445

1473 film.actor.film 8394

1473 film.person_or_entity_appearing_in_film.film 8037

977 film.actor.film 8446

1706 film.actor.film 5758

1711 music.artist.track 8447

66 film.actor.film 8448

1551 people.person.place_of_birth 4057

1975 music.artist.origin 6095

2304 music.artist.origin 5383

2304 music.artist.origin 4852

2636 film.person_or_entity_appearing_in_film.film 4182

2597 people.person.place_of_birth 5265

1458 film.person_or_entity_appearing_in_film.film 8449

3289 event.agent.performance 4949

1735 music.artist.origin 4003

1579 film.actor.film 8450

3701 film.actor.film 8347

484 film.actor.film 8359

3738 cvg.publisher.games_published 8027

3738 games.publisher.games_published 7848

3738 games.publisher.games_published 7284

1154 film.person_or_entity_appearing_in_film.film 6843

2430 film.actor.film 4646

3452 film.actor.film 8249

779 film.person_or_entity_appearing_in_film.film 5816

668 film.actor.film 4621

592 people.person.place_of_birth 4816

2241 film.distributor.film 7073

2241 film.distributor.film 4904

2323 music.artist.origin 5920

1464 film.actor.film 8451

2976 music.artist.origin 3945

1850 music.artist.origin 3966

2189 film.actor.film 4897

943 film.person_or_entity_appearing_in_film.film 8452

1567 music.artist.origin 8453

1090 music.artist.origin 3864

425 people.person.place_of_birth 4357

1580 award.competitor.award_nominations 6452

538 film.actor.film 6350

3000 film.person_or_entity_appearing_in_film.film 7497

1608 music.artist.origin 4013

898 music.artist.origin 6343

1523 music.artist.origin 3972

1451 music.artist.origin 4195

3390 film.actor.film 4384

3279 film.actor.film 7825

3648 film.person_or_entity_appearing_in_film.film 4266

1348 film.director.film 5614

1074 music.artist.track 5615

1485 music.artist.origin 4701

1358 film.actor.film 7984

1365 film.person_or_entity_appearing_in_film.film 8454

2792 film.actor.film 8455

410 film.person_or_entity_appearing_in_film.film 8456

428 film.actor.film 7184

2094 film.actor.film 8457

846 music.artist.album 8433

2953 film.actor.film 8458

67 film.person_or_entity_appearing_in_film.film 6767

1473 film.person_or_entity_appearing_in_film.film 7886

3372 film.actor.film 8191

2813 people.person.place_of_birth 4094

2913 film.actor.film 8459

2914 film.actor.film 5320

650 music.artist.origin 3849

1601 film.person_or_entity_appearing_in_film.film 5629

3561 film.actor.film 8460

2636 film.person_or_entity_appearing_in_film.film 8461

2460 film.person_or_entity_appearing_in_film.film 5172

1308 film.person_or_entity_appearing_in_film.film 6052

2411 film.actor.film 8176

484 film.person_or_entity_appearing_in_film.film 8462

3738 games.publisher.games_published 8463

1550 music.artist.origin 3986

901 film.actor.film 8230

1448 film.actor.film 3967

1002 music.musician.instruments_played 4349

2159 film.actor.film 8464

3747 people.person.place_of_birth 4217

2241 film.distributor.film 8465

2241 film.production_company.film 8466

1936 film.actor.film 6611

1936 film.actor.film 8253

1344	people.person.place_of_birth	8408

599 film.person_or_entity_appearing_in_film.film	8467

3165	people.person.place_of_birth	8468

2355	people.person.place_of_birth	8443

2038	film.actor.film	6756

3486	type.object.subject_key	3486

958 music.artist.album	7809

2861	film.person_or_entity_appearing_in_film.film	8469

2288	music.artist.origin	3916

1580	film.actor.film	4837

2415	film.actor.film	8470

1884	film.person_or_entity_appearing_in_film.film	7733

2979	music.artist.origin	4876

2519	film.actor.film	6385

3752	award.nominee.award_nominations	6454

1089	film.actor.film	8428

3378	music.artist.origin	4479

3390	film.person_or_entity_appearing_in_film.film	5442

3048	film.person_or_entity_appearing_in_film.film	7482

3614	film.actor.film	4446

2402	film.actor.film	5835

2857	music.artist.origin	3961

2635	film.producer.film	8276

1157	film.actor.film	8471

2327	people.person.place_of_birth	3864

623 film.person_or_entity_appearing_in_film.film	5566

1281	music.artist.origin	6215

2359	film.person_or_entity_appearing_in_film.film	5757

2147	film.actor.film	5981

793 film.person_or_entity_appearing_in_film.film	6590

3784 film.director.film 7061

1935 film.actor.film 7976

71 film.actor.film 4494

539 film.actor.film 6595

3598 film.person_or_entity_appearing_in_film.film 8472

3491 music.artist.origin 4063

2830 film.person_or_entity_appearing_in_film.film 6746

2562 film.actor.film 7066

187 event.agent.performance 4949

2636 film.person_or_entity_appearing_in_film.film 8473

2650 film.film.genre 4285

2070 film.actor.film 6306

1458 film.actor.film 8449

3166 film.actor.film 8474

1353 film.actor.film 5759

2460 film.person_or_entity_appearing_in_film.film 8228

484 film.person_or_entity_appearing_in_film.film 8475

3738 cvg.publisher.games_published 5178

3738 cvg.publisher.games_published 6782

1333 music.artist.origin 3897

1249 film.actor.film 4740

2241 film.distributor.film 5862

2241 film.distributor.film 8476

2241 film.distributor.film 7726

2241 film.distributor.film 8477

2241 film.distributor.film 6300

2241 film.distributor.film 7259

2241 film.production_company.film 5128

2241 film.production_company.film 7470

2241 film.production_company.film 8478

2241 film.production_company.film 5296

1936 film.actor.film 7778

2129 film.actor.film 4940

345 music.artist.origin 3961

1780 film.actor.film 8479

1708 music.artist.origin 3961

1618 film.actor.film 7854

894 film.person_or_entity_appearing_in_film.film 8323

261 award.competitor.awards_won 7729

1656 film.actor.film 7781

1047 music.artist.origin 6076

3623 people.person.place_of_birth 4347

36 music.artist.track 6899

3321 film.actor.film 8480

573 music.artist.origin 3897

3557 film.film.actor 8481

1611 music.artist.origin 4108

1089 film.person_or_entity_appearing_in_film.film 8215

3026 film.actor.film 8482

2630 people.person.place_of_birth 8483

3390 film.actor.film 8484

3390 film.person_or_entity_appearing_in_film.film 8485

2243 music.artist.origin 7915

2599 film.actor.film 7557

1776 people.deceased_person.place_of_death 4362

2635 film.actor.film 8486

1349 film.person_or_entity_appearing_in_film.film 8487

2418 film.person_or_entity_appearing_in_film.film 8488

3292 music.artist.origin 5845

2842 people.person.place_of_birth 7341

282 music.artist.origin 7399

1779 people.person.place_of_birth 8203

1473 film.actor.film 8489

91 film.person_or_entity_appearing_in_film.film 8490

1223 film.actor.film 8491

990 music.artist.origin 5344

1538 music.artist.origin 7370

2742 music.musician.instruments_played 4349

3498 film.person_or_entity_appearing_in_film.film 7840

2902 music.artist.origin 8492

3799 film.actor.film 8493

1773 film.person_or_entity_appearing_in_film.film 8316

3500 people.person.place_of_birth 4975

2753 music.artist.origin 4174

2636 film.actor.film 8494

3047 music.musician.instruments_played 4349

715 film.actor.film 5719

1691 music.artist.origin 3864

602 film.person_or_entity_appearing_in_film.film 5120

3738 cvg.publisher.games_published 7320

3738 cvg.publisher.games_published 6508

666 people.person.place_of_birth 6923

1436 music.album.genre 6530

2241 film.distributor.film 8495

2241 film.distributor.film 3215

2241 film.production_company.film 5034

2241 film.production_company.film 5335

2241 film.production_company.film 7980

1936 film.person_or_entity_appearing_in_film.film 8007

520 film.actor.film 5709

1858	music.artist.origin	4057

2487	film.person_or_entity_appearing_in_film.film	8496

998	film.person_or_entity_appearing_in_film.film	8497

1767	film.person_or_entity_appearing_in_film.film	8286

643	film.actor.film	8337

2810	film.person_or_entity_appearing_in_film.film	8234

3780	film.actor.film	8498

434	film.person_or_entity_appearing_in_film.film	8499

3069	music.artist.origin	4057

3049	film.actor.film	7904

3748	music.artist.origin	4217

35	music.album.genre	5201

3390	film.actor.film	7573

2711	film.actor.film	8500

1700	music.artist.origin	4454

306	film.person_or_entity_appearing_in_film.film	6600

98	music.artist.origin	4454

1280	type.object.key	1280

1122	music.artist.origin	4471

1776	film.person_or_entity_appearing_in_film.film	4162

1726	film.actor.film	4837

2635	film.person_or_entity_appearing_in_film.film	8501

3307	film.person_or_entity_appearing_in_film.film	6940

115	film.actor.film	8502

2040	film.person_or_entity_appearing_in_film.film	8150

3293	music.artist.origin	5845

492	film.actor.film	8089

2371	film.person_or_entity_appearing_in_film.film	8503

2953	film.person_or_entity_appearing_in_film.film	8458

1852	music.artist.origin	4150

236 film.actor.film 7525

1473 film.actor.film 8205

189 film.actor.film 6883

3497 music.artist.origin 5845

2737 film.actor.film 3970

3757 music.artist.origin 4963

3598 film.person_or_entity_appearing_in_film.film 7934

1991 music.artist.origin 7554

2726 film.person_or_entity_appearing_in_film.film 4333

1079 film.writer.film 7671

809 film.person_or_entity_appearing_in_film.film 6977

2913 film.person_or_entity_appearing_in_film.film 8504

1469 film.actor.film 4281

70 film.person_or_entity_appearing_in_film.film 8505

974 film.actor.film 7953

2636 film.actor.film 8473

1355 film.person_or_entity_appearing_in_film.film 8506

48 music.artist.track 7957

137 award.nominee.award_nominations 8507

3091 music.artist.origin 4133

484 film.person_or_entity_appearing_in_film.film 5424

3738 games.publisher.games_published 7979

901 film.person_or_entity_appearing_in_film.film 8044

1249 film.person_or_entity_appearing_in_film.film 5930

2867 film.actor.film 5960

2241 film.distributor.film 5777

2241 film.production_company.film 8271

2241 film.production_company.film 8508

988 film.person_or_entity_appearing_in_film.film 8376

1426 music.album.genre 4417

1464 film.person_or_entity_appearing_in_film.film 8509

2239 people.person.place_of_birth 5916

1522 music.artist.origin 4583

3654 people.person.place_of_birth 4013

3354 award.nominee.award_nominations 7386

1690 music.artist.origin 3872

3376 music.album.genre 8510

1806 film.person_or_entity_appearing_in_film.film 8511

2184 film.actor.film 4798

701 film.actor.film 7553

166 people.person.place_of_birth 3872

3132 film.person_or_entity_appearing_in_film.film 8512

1479 film.person_or_entity_appearing_in_film.film 5562

206 film.actor.film 7334

3822 film.person_or_entity_appearing_in_film.film 8513

997 film.actor.film 6965

2076 film.person_or_entity_appearing_in_film.film 8514

281 music.artist.origin 3859

1662 film.person_or_entity_appearing_in_film.film 4703

3599 film.actor.film 7336

2297 film.person_or_entity_appearing_in_film.film 4591

2635 film.actor.film 8342

90 people.person.place_of_birth 4003

2876 event.agent.performance 4035

1607 music.artist.origin 4455

540 film.actor.film 6595

115 film.person_or_entity_appearing_in_film.film 7243

2494 film.actor.film 8515

220 music.artist.origin 4391

1505 film.person_or_entity_appearing_in_film.film 5404

627 film.person_or_entity_appearing_in_film.film 8516

362 music.artist.origin 3897

2050 film.person_or_entity_appearing_in_film.film 8517

3788 film.actor.film 8518

633 film.actor.film 7697

236 film.person_or_entity_appearing_in_film.film 7525

2125 film.person_or_entity_appearing_in_film.film 7003

1473 film.person_or_entity_appearing_in_film.film 4549

139 music.artist.origin 6425

645 film.person_or_entity_appearing_in_film.film 5986

3598 film.actor.film 8267

3598 film.person_or_entity_appearing_in_film.film 7806

130 people.person.place_of_birth 3864

3158 film.actor.film 8519

3094 film.person_or_entity_appearing_in_film.film 8520

2310 music.artist.origin 4057

319 film.film.star 8521

1601 film.actor.film 4022

2096 film.person_or_entity_appearing_in_film.film 8457

568 film.actor.film 8331

3355 film.actor.film 4557

1769 people.person.place_of_birth 7932

2688 film.actor.film 6021

366 film.person_or_entity_appearing_in_film.film 7254

771 people.person.place_of_birth 6619

2412 people.person.place_of_birth 3947

466 film.actor.film 6470

527 film.actor.film 8522

3738 cvg.publisher.games_published 8523

901 film.actor.film 8524

901 film.producer.film 8524

438 film.actor.film 7570

1883 music.artist.origin 7759

2241 film.distributor.film 4892

2241 film.distributor.film 5965

2241 film.production_company.film 8140

681 film.person_or_entity_appearing_in_film.film 8377

1784 music.artist.origin 8525

3051 music.artist.origin 4380

2487 film.person_or_entity_appearing_in_film.film 8526

894 film.actor.film 6516

2326 music.artist.origin 3961

1927 music.musician.instruments_played 4349

2648 music.artist.origin 4050

1651 music.musician.instruments_played 3851

2884 film.actor.film 8527

2643 film.actor.film 7575

1357 film.actor.film 7231

2334 music.artist.origin 6249

538 film.actor.film 4258

3781 music.artist.origin 3986

1817 film.person_or_entity_appearing_in_film.film 8122

1378 film.person_or_entity_appearing_in_film.film 8528

3205 people.person.place_of_birth 4150

2634 film.person_or_entity_appearing_in_film.film 7664

2211 film.actor.film 4102

3279 film.person_or_entity_appearing_in_film.film 8529

1632 film.person_or_entity_appearing_in_film.film 3937

2962 film.actor.film 6861

3154 music.artist.origin 5061

2242 film.person_or_entity_appearing_in_film.film 8530

1142 film.actor.film 8531

1683 music.artist.origin 4391

2161 award.competitor.award_nominations 8407

774 people.person.place_of_birth 4357

1473 film.person_or_entity_appearing_in_film.film 8532

1026 film.actor.film 8533

2603 film.actor.film 5014

2341 film.person_or_entity_appearing_in_film.film 8534

407 film.person_or_entity_appearing_in_film.film 4330

2742 music.artist.album 8535

3282 film.actor.film 4501

2881 film.person_or_entity_appearing_in_film.film 8536

1704 film.person_or_entity_appearing_in_film.film 5803

672 film.actor.film 8537

628 film.person_or_entity_appearing_in_film.film 8538

2562 film.person_or_entity_appearing_in_film.film 4505

2636 film.actor.film 8539

2636 film.person_or_entity_appearing_in_film.film 8540

2781 film.actor.film 4136

79 film.actor.film 5894

602 music.artist.track 8541

484 film.person_or_entity_appearing_in_film.film 8542

3738 cvg.publisher.games_published 5179

3738 cvg.publisher.games_published 8543

3738 cvg.publisher.games_published 8544

3738 games.publisher.games_published 6267

2335 film.actor.film 8545

899 film.person_or_entity_appearing_in_film.film 4980

1787 people.person.place_of_birth 4217

2152	music.artist.origin	5213

3248	film.person_or_entity_appearing_in_film.film	8546

432 film.actor.film	3861

2241	film.distributor.film	7258

2241	film.distributor.film	4628

2241	film.production_company.film	4734

1936	film.person_or_entity_appearing_in_film.film	5966

1589	music.artist.origin	4013

452 music.artist.origin	3864

1614	music.artist.origin	4521

777 film.actor.film	5938

2126	film.person_or_entity_appearing_in_film.film	5229

1360	film.person_or_entity_appearing_in_film.film	4351

1806	people.person.place_of_birth	5826

3526	people.person.place_of_birth	4166

538 film.person_or_entity_appearing_in_film.film	8547

1430	film.actor.film	8548

673 film.actor.film	8085

1023	music.artist.origin	4583

1202	film.person_or_entity_appearing_in_film.film	7265

2870	people.person.place_of_birth	8549

238 film.person_or_entity_appearing_in_film.film	8550

3187	music.artist.album	8551

243 music.artist.origin	3972

3752	film.person_or_entity_appearing_in_film.film	6903

1116	music.artist.origin	4010

3650	film.person_or_entity_appearing_in_film.film	8419

3390	film.person_or_entity_appearing_in_film.film	7573

2770	film.person_or_entity_appearing_in_film.film	5561

3077	music.artist.origin	4852

1816	film.writer.film	8552
3480	people.deceased_person.place_of_death	5270
1627	film.actor.film	7120
959	film.person_or_entity_appearing_in_film.film	7395
1662	film.person_or_entity_appearing_in_film.film	7802
1549	music.artist.origin	4852
2090	film.actor.film	4837
2494	film.person_or_entity_appearing_in_film.film	4581
785	film.actor.film	4594
785	film.person_or_entity_appearing_in_film.film	4654
926	music.artist.origin	5383
2040	film.person_or_entity_appearing_in_film.film	8553
3496	film.film.country	4380
662	film.person_or_entity_appearing_in_film.film	8381
1396	music.artist.origin	7672
2613	music.artist.origin	4896
2050	film.actor.film	8517
2963	film.actor.film	8554
67	film.person_or_entity_appearing_in_film.film	5485
633	people.person.place_of_birth	5569
2359	film.actor.film	8555
236	music.artist.origin	3916
1473	film.person_or_entity_appearing_in_film.film	4250
2824	music.artist.origin	3846
2337	film.actor.film	8266
1984	music.musician.instruments_played	4349
127	film.person_or_entity_appearing_in_film.film	3957
3725	music.musician.instruments_played	3851
2924	film.actor.film	3969
645	film.person_or_entity_appearing_in_film.film	8556

747 music.artist.origin 4456

893 film.person_or_entity_appearing_in_film.film 8223

3282 film.actor.film 8557

3845 film.actor.film 7062

672 film.person_or_entity_appearing_in_film.film 6134

361 award.nominee.award_nominations 5989

3056 music.artist.origin 3947

628 film.person_or_entity_appearing_in_film.film 3913

2021 music.artist.origin 6694

2070 film.person_or_entity_appearing_in_film.film 4750

2819 film.person_or_entity_appearing_in_film.film 8558

765 music.artist.origin 4552

3738 cvg.publisher.games_published 6749

2566 film.person_or_entity_appearing_in_film.film 4054

2639 music.artist.origin 7530

2400 film.actor.film 6783

2241 film.distributor.film 5797

2241 film.distributor.film 7494

2241 film.distributor.film 8559

988 film.person_or_entity_appearing_in_film.film 7728

988 people.person.place_of_birth 3887

659 music.artist.origin 6361

1624 film.actor.film 8099

2600 music.artist.origin 6137

2731 film.person_or_entity_appearing_in_film.film 8560

1806 film.person_or_entity_appearing_in_film.film 7014

159 film.actor.film 8561

2969 music.artist.origin 4183

440 music.musician.instruments_played 3959

3144 music.artist.origin 5247

2387 film.producer.film 5720

157 type.object.key 157

3557 film.film.writer 5611

380 film.person_or_entity_appearing_in_film.film 7499

2891 music.musician.instruments_played 5367

1260 music.artist.origin 6195

997 film.actor.film 4161

1776 film.person_or_entity_appearing_in_film.film 5000

2635 film.person_or_entity_appearing_in_film.film 7272

2635 film.person_or_entity_appearing_in_film.film 7182

2984 film.person_or_entity_appearing_in_film.film 8017

410 film.person_or_entity_appearing_in_film.film 4051

1891 film.person_or_entity_appearing_in_film.film 7864

1748 film.person_or_entity_appearing_in_film.film 4116

328 music.artist.origin 4222

912 film.person_or_entity_appearing_in_film.film 5470

2359 film.actor.film 7737

2370 film.actor.film 8293

2125 film.actor.film 8562

1141 film.actor.film 8243

895 film.person_or_entity_appearing_in_film.film 8563

1193 film.actor.film 7869

2482 film.person_or_entity_appearing_in_film.film 5015

377 film.actor.film 7868

3598 film.actor.film 6098

893 film.person_or_entity_appearing_in_film.film 7839

2726 film.actor.film 8003

3498 film.actor.film 6025

2604 music.artist.origin 4134

1601 film.actor.film 8415

3166 film.actor.film 5930

1353 film.actor.film 7543

1840 film.actor.film 8564

1579 music.artist.origin 3961

2208 film.actor.film 8565

3738 cvg.publisher.games_published 7898

3738 cvg.publisher.games_published 8566

3738 games.publisher.games_published 8567

901 film.actor.film 8339

901 film.person_or_entity_appearing_in_film.film 8568

1249 film.actor.film 3935

2241 film.distributor.film 4549

2241 film.distributor.film 8135

795 film.actor.film 4915

894 film.person_or_entity_appearing_in_film.film 7473

3031 film.person_or_entity_appearing_in_film.film 4910

2275 film.actor.film 3939

1772 film.actor.film 8388

2527 people.person.place_of_birth 5920

3002 film.person_or_entity_appearing_in_film.film 8569

1008 music.artist.origin 5213

1958 music.artist.origin 3846

81 film.actor.film 4870

3026 film.person_or_entity_appearing_in_film.film 5868

3390 film.actor.film 8570

3173 music.artist.origin 4108

2711 film.actor.film 6042

923 film.person_or_entity_appearing_in_film.film 6536

3466 music.artist.origin 3947

1701 music.artist.origin 5265

567 film.actor.film 8571

1157 film.person_or_entity_appearing_in_film.film 4954

155 award.competitor.awards_won 7631

1153 film.actor.film 8572

1010 people.person.place_of_birth 4058

307 film.actor.film 4595

2953 film.actor.film 8573

3263 location.location.contained_by 3961

2908 music.artist.origin 7079

774 film.actor.film 6362

1261 music.artist.origin 4445

2482 film.person_or_entity_appearing_in_film.film 8574

3733 film.actor.film 8575

2457 people.person.place_of_birth 8576

3598 film.person_or_entity_appearing_in_film.film 8282

619 people.person.profession 4990

980 film.actor.film 8577

2913 film.actor.film 7114

1469 film.person_or_entity_appearing_in_film.film 7589

3149 music.artist.origin 8578

1204 people.person.place_of_birth 6077

2636 film.actor.film 8579

2636 film.actor.film 6891

568 music.artist.origin 4057

2783 people.person.place_of_birth 3984

2688 film.person_or_entity_appearing_in_film.film 6221

49 film.actor.film 8580

49 film.actor.film 5923

2483 film.actor.film 5176

2748 music.artist.origin 4024

3738 cvg.publisher.games_published 3858

901 film.person_or_entity_appearing_in_film.film 6146

1363 film.actor.film 8191

1359 film.actor.film 6784

791 film.actor.film 4082

2241 film.distributor.film 5219

2241 film.distributor.film 6230

2241 film.distributor.film 8581

2241 film.distributor.film 8067

2241 film.distributor.film 8375

2241 film.production_company.film 5962

3409 music.artist.origin 4150

3430 film.actor.film 4352

1942 film.actor.film 7283

135 people.person.place_of_birth 5523

2311 film.person_or_entity_appearing_in_film.film 5821

23 music.artist.origin 7333

1768 film.actor.film 4694

2024 film.person_or_entity_appearing_in_film.film 7327

643 film.actor.film 5187

2678 music.artist.album 8582

817 people.deceased_person.place_of_death 4455

1536 film.person_or_entity_appearing_in_film.film 4476

2860 film.person_or_entity_appearing_in_film.film 4522

3049 film.person_or_entity_appearing_in_film.film 6964

1189 music.artist.origin 4362

3601 music.artist.origin 4852

2279 film.actor.film 8583

1468 film.person_or_entity_appearing_in_film.film 8584

2372 music.artist.origin 4552

1387 film.actor.film 8585

3026 music.artist.origin 8586

3048 film.actor.film 8587

1705 music.artist.origin 4369

2449 film.actor.film 6684

1962 film.actor.film 7555

193 film.person_or_entity_appearing_in_film.film 6686

773 music.artist.origin 8588

2635 film.actor.film 5653

2635 film.person_or_entity_appearing_in_film.film 6969

2550 film.actor.film 5882

1157 film.person_or_entity_appearing_in_film.film 8589

2366 music.artist.origin 5265

1633 film.actor.film 8590

1633 film.actor.film 6622

492 film.actor.film 8591

3436 music.artist.origin 8592

2963 film.actor.film 4823

1155 film.person_or_entity_appearing_in_film.film 6980

2552 film.actor.film 4385

2737 film.actor.film 4549

1209 music.artist.origin 4369

3598 film.person_or_entity_appearing_in_film.film 5577

2662 music.artist.origin 4214

3131 music.artist.origin 3864

584 film.actor.film 6403

672 film.person_or_entity_appearing_in_film.film 5535

2914 film.person_or_entity_appearing_in_film.film 5320

3777 film.person_or_entity_appearing_in_film.film 6889

653 music.artist.origin 7489

603 music.artist.album 8593

2636 film.actor.film 8594

2636 film.person_or_entity_appearing_in_film.film 8539

2016 music.artist.origin 5259

3738 cvg.publisher.games_published 8595

3738 cvg.publisher.games_published 8596

3431 people.person.place_of_birth 5357

2111 film.person_or_entity_appearing_in_film.film 5376

1703 music.artist.origin 5934

625 film.actor.film 6225

119 music.producer.tracks_produced 8597

1249 film.actor.film 5292

2867 film.person_or_entity_appearing_in_film.film 8598

2241 film.distributor.film 8599

2241 film.distributor.film 8600

2241 film.production_company.film 8601

2241 film.production_company.film 8477

2241 film.production_company.film 5295

2241 film.production_company.film 8602

2241 film.production_company.film 8006

3297 film.person_or_entity_appearing_in_film.film 4802

1780 film.person_or_entity_appearing_in_film.film 8479

1819 film.person_or_entity_appearing_in_film.film 4330

1842 film.person_or_entity_appearing_in_film.film 8300

3031 film.person_or_entity_appearing_in_film.film 8324

1567 film.actor.film 4323

2626 music.artist.origin 6215

486 music.artist.origin 3864

757 film.actor.film 4360

3052 film.actor.film 8603

3650 film.actor.film 8604

166 music.artist.album 6703

3279 film.actor.film 7455

1130 music.artist.album 8605

241 film.person_or_entity_appearing_in_film.film 8606

2658 film.actor.film 6710

3132 film.person_or_entity_appearing_in_film.film 6587

997 film.actor.film 7711

997 film.person_or_entity_appearing_in_film.film 8607

567 film.person_or_entity_appearing_in_film.film 4482

2528 film.person_or_entity_appearing_in_film.film 8608

540 film.actor.film 3973

2040 film.actor.film 8553

1153 film.person_or_entity_appearing_in_film.film 8609

3020 film.actor.film 7993

3788 film.person_or_entity_appearing_in_film.film 8610

2868 music.artist.origin 4175

1715 music.musician.instruments_played 6942

1476 music.artist.album 8535

1429 people.person.place_of_birth 6056

17 music.artist.origin 5228

1671 music.artist.origin 5139

2080 film.actor.film 7248

1086 music.artist.origin 4038

557 film.actor.film 7744

1352 people.person.profession 4221

64 film.actor.film 4915

3079 film.actor.film 4048

2784 people.person.place_of_birth 3874

967 music.artist.origin 7640

646 film.person_or_entity_appearing_in_film.film 8611

1601 film.person_or_entity_appearing_in_film.film 8612

974 film.actor.film 6576

2636 film.person_or_entity_appearing_in_film.film 4873

2437 film.person_or_entity_appearing_in_film.film 8080

2070 film.actor.film 5583

50 music.artist.origin 6797

2819 film.actor.film 8558

3179 film.actor.film 6666

3776 music.musician.instruments_played 3959

49 film.person_or_entity_appearing_in_film.film 8613

1172 film.person_or_entity_appearing_in_film.film 7660

133 award.competitor.award_nominations 8614

79 film.actor.film 4886

79 film.person_or_entity_appearing_in_film.film 5029

1579 film.person_or_entity_appearing_in_film.film 7042

3738 cvg.publisher.games_published 8615

3219 film.actor.film 6850

901 film.person_or_entity_appearing_in_film.film 8339

442 award.nominee.award_nominations 8616

2241 film.distributor.film 7385

2241 film.distributor.film 7572

2241 film.distributor.film 7850

2241 film.production_company.film 8177

2241 film.production_company.film 7228

2232 people.person.profession 4990

1762 film.actor.film 8617

1987 people.person.profession 4221

2126 film.actor.film 8618

1767 film.actor.film 5042

1437 film.actor.film 8619

2678 film.actor.film 8070

406 people.person.place_of_birth 8387

3546 film.actor.film 6008

3526 music.artist.origin 4050

2861 music.musician.instruments_played 5802

1580 film.person_or_entity_appearing_in_film.film 7388

2870 film.actor.film 7875

1783 music.musician.instruments_played 3959

2575 music.artist.origin 4455

238 film.actor.film 7968

3712 film.person_or_entity_appearing_in_film.film 6616

2715 film.actor.film 8620

2917 music.artist.origin 5934

794 film.person_or_entity_appearing_in_film.film 4493

2770 music.musician.instruments_played 3851

2711 film.person_or_entity_appearing_in_film.film 8307

3132 film.person_or_entity_appearing_in_film.film 4424

2312 film.person_or_entity_appearing_in_film.film 8126

2426 music.artist.origin 3864

3088 film.person_or_entity_appearing_in_film.film 6407

2827 music.album.genre 7810

1726 award.nominee.award_nominations 6853

1515 people.person.place_of_birth 4347

3460 film.person_or_entity_appearing_in_film.film 8621

2569 music.artist.origin 8349

540 film.actor.film 8429

115 film.person_or_entity_appearing_in_film.film 6612

3402 people.person.place_of_birth 4013

2494 film.actor.film 4152

1720 film.writer.film 4860

931 music.artist.origin 6111

155 music.musician.instruments_played 5367

1833 music.artist.track 8109

2045 music.artist.origin 4087

3412 film.film.actor 8622

907 music.artist.origin 4050

3404 film.actor.film 4225

977 film.person_or_entity_appearing_in_film.film 8446

1706 film.actor.film 8057

645 film.actor.film 7609

66 film.person_or_entity_appearing_in_film.film 8623

1223 music.musician.instruments_played 6260

3150 music.artist.origin 4963

2080 film.person_or_entity_appearing_in_film.film 5415

2705 film.actor.film 8624

1757 music.artist.origin 3864

128 people.person.place_of_birth 5227

3355 film.person_or_entity_appearing_in_film.film 8625

1366 music.artist.origin 4057

2070 film.person_or_entity_appearing_in_film.film 6863

1458 film.actor.film 6501

2594 music.artist.origin 3864

3750 people.person.place_of_birth 4536

445 film.actor.film 8626

2192 music.musician.instruments_played 4349

2778 people.person.place_of_birth 6343

790 film.person_or_entity_appearing_in_film.film 4802

3738 cvg.publisher.games_published 8334

3738 games.publisher.games_published 5328

119 music.artist.track 8374

2867 film.person_or_entity_appearing_in_film.film 4569

2241 film.distributor.film 8627

2241 film.distributor.film 8628

2241 film.distributor.film 8421

2241 film.distributor.film 5933

2241 film.distributor.film 8629

1056 music.artist.origin 3849

2951 film.actor.film 4794

3031 film.person_or_entity_appearing_in_film.film 8630

1360 film.actor.film 6707

2114 music.artist.origin 4852

788 film.person_or_entity_appearing_in_film.film 6963

2206 film.person_or_entity_appearing_in_film.film 8631

636 film.actor.film 7625

2643 film.person_or_entity_appearing_in_film.film 8302

538 film.person_or_entity_appearing_in_film.film 8632

372 people.person.place_of_birth 7789

298 film.person_or_entity_appearing_in_film.film 7550

3254 music.artist.origin 4199

1782 music.artist.origin 7399

1814 film.film.genre 4285

2010 film.actor.film 8633

606 music.artist.origin 4148

1866 film.person_or_entity_appearing_in_film.film 8634

2502 music.artist.origin 4183

2343 film.actor.film 8104

997 film.actor.film 8635

567 film.person_or_entity_appearing_in_film.film 8636

1633 film.actor.film 8637

2435 music.artist.origin 4057

1232 film.person_or_entity_appearing_in_film.film 5219

3827 film.actor.film 8638

2382 music.artist.origin 5845

1672 music.artist.album 8639

1891 film.actor.film 8640

3317 film.person_or_entity_appearing_in_film.film 3893

1748 film.actor.film 5152

2620 film.actor.film 5549

993 film.person_or_entity_appearing_in_film.film 7884

3412 film.film.art_director 8641

1805 film.person_or_entity_appearing_in_film.film 6062

2733 music.musician.instruments_played 4349

1473 film.actor.film 4120

1692 film.actor.film 6569

3328 music.musician.instruments_played 3851

2737 film.actor.film 6551

71 film.actor.film 6087

2080 film.person_or_entity_appearing_in_film.film 8642

789 film.actor.film 4802

3178 music.artist.origin 5924

408 film.person_or_entity_appearing_in_film.film 6575

1242 music.artist.origin 4195

3478 music.artist.origin 7433

2743 music.artist.origin 6408

1458 film.actor.film 8643

3663 film.actor.film 4406

366 film.person_or_entity_appearing_in_film.film 8357

49 film.writer.film 6218

399 film.person_or_entity_appearing_in_film.film 4411

2877 film.actor.film 8644

2877 film.person_or_entity_appearing_in_film.film 8644

3738 cvg.publisher.games_published 8645

3738 cvg.publisher.games_published 8646

3738 games.publisher.games_published 8596

1154 film.person_or_entity_appearing_in_film.film 8647

3010 people.person.place_of_birth 3942

2286 music.artist.origin 4058

615 music.artist.origin 4174

3161 music.artist.album 8648

3809 people.person.place_of_birth 4167

2241 film.distributor.film 8160

2241 film.distributor.film 5979

2241 film.production_company.film 8649

3680 film.character.film 8650

622 film.person_or_entity_appearing_in_film.film 5216

2677 people.deceased_person.place_of_death 4217

636 film.person_or_entity_appearing_in_film.film 6708

3374 film.actor.film 4206

3049 film.actor.film 8157

898 film.actor.film 5346

3432 people.person.place_of_birth 5270

3648 film.actor.film 8651

1632 film.person_or_entity_appearing_in_film.film 5443

923 film.actor.film 6536

1866 film.person_or_entity_appearing_in_film.film 6683

204 film.person_or_entity_appearing_in_film.film 6852

3822 film.actor.film 8513

1279 film.actor.film 6966

1243 music.artist.origin 8652

2986 film.actor.film 5191

3636 music.artist.origin 6898

1976 music.artist.origin 6735

1505 film.actor.film 7213

2050 film.actor.film 8653

1473 film.actor.film 8654

2292 film.person_or_entity_appearing_in_film.film 7406

2957 music.artist.origin 7930

2737 film.actor.film 5107

109 music.artist.track 4486

1078 music.artist.origin 3916

3598 film.actor.film 4667

3598 film.actor.film 8655

3396 film.actor.film 8121

1084 music.artist.origin 7789

1095 event.agent.performance 4099

945 film.actor.film 8656

3821 film.person_or_entity_appearing_in_film.film 8657

399 film.person_or_entity_appearing_in_film.film 6010

3443 film.person_or_entity_appearing_in_film.film 4731

1445 music.artist.origin 4008

3738 cvg.publisher.games_published 6958

3738 cvg.publisher.games_published 8658

3738 cvg.publisher.games_published 8659

3738 games.publisher.games_published 6662

796 film.person_or_entity_appearing_in_film.film 4323

2241 film.distributor.film 8660

2241 film.distributor.film 6273

2241 film.distributor.film 7919

2241 film.production_company.film 7197

2241 film.production_company.film 7647

2241 film.production_company.film 8004

2241 film.production_company.film 7199

2241 film.production_company.film 6726

481 film.person_or_entity_appearing_in_film.film 7679

3160 film.actor.film 8661

290 music.artist.origin 8351

777 film.person_or_entity_appearing_in_film.film 8008

3706 film.director.film 6897

2126 film.actor.film 8662

3669 film.person_or_entity_appearing_in_film.film 6238

2098 music.musician.instruments_played 8663

3306 music.album.artist 7401

1226 music.artist.origin 3846

2910 music.artist.origin 4167

194 film.actor.film 7921

3002 film.actor.film 8569

3049 film.actor.film 3871

3648 film.actor.film 5676

206 film.actor.film 4728

997 film.actor.film 8664

2904 film.person_or_entity_appearing_in_film.film 4365

230 music.artist.origin 4232

3460 music.artist.origin 3846

1505 film.actor.film 6765

1153 film.actor.film 5239

1153 film.actor.film 6204

2050 film.actor.film 5010

1488 award.nominee.award_nominations 8665

2359 film.person_or_entity_appearing_in_film.film 3898

3412 film.film.genre 8432

1473 film.person_or_entity_appearing_in_film.film 8666

793 film.actor.film 6590

2337 film.person_or_entity_appearing_in_film.film 6301

1820 people.person.place_of_birth 4087

145 film.actor.film 8667

279 award.nominee.award_nominations 6603

2966 film.actor.film 8668

645 film.actor.film 8337

66 film.person_or_entity_appearing_in_film.film 7741

109 film.person_or_entity_appearing_in_film.film 5533

2762 film.actor.film 4237

612 music.artist.origin 4927

2080 film.person_or_entity_appearing_in_film.film 7131

3598 film.person_or_entity_appearing_in_film.film 8669

217 music.artist.origin 5639

3447 film.actor.film 5786

1616 film.person_or_entity_appearing_in_film.film 7638

2913 film.actor.film 8155

2913 film.actor.film 7891

2710 film.actor.film 7514

1018 people.person.place_of_birth 7302

2636 film.actor.film 8670

2597 film.actor.film 8671

2688 film.actor.film 4786

2460 film.actor.film 8672

2460 film.actor.film 5456

3738 cvg.publisher.games_published 8673

2293 film.person_or_entity_appearing_in_film.film 5366

949 film.person_or_entity_appearing_in_film.film 8674

2159 film.actor.film 5774

725 music.artist.origin 3945

2241 film.distributor.film 8675

2241 film.distributor.film 6002

2241 film.distributor.film 8676

2241 film.production_company.film 4984

60 music.artist.origin 4094

3348 music.artist.origin 3855

2487 film.actor.film 8677

1618 music.artist.origin 4057

2897 film.person_or_entity_appearing_in_film.film 7600

1894 music.artist.album 8639

1697 film.actor.film 8678

2596 people.person.place_of_birth 6349

3331 film.person_or_entity_appearing_in_film.film 6073

1381 music.musician.instruments_played 4349

3049 film.person_or_entity_appearing_in_film.film 8679

3049 film.person_or_entity_appearing_in_film.film 8680

2519 music.artist.origin 8123

3360 film.person_or_entity_appearing_in_film.film 8306

3132 film.actor.film 8681

1771 music.artist.origin 3846

3686 music.artist.origin 6878

1963 music.artist.origin 8682

552 film.actor.film 8683

2635 film.actor.film 7337

953 music.album.genre 7206

379 film.actor.film 8684

186 film.person_or_entity_appearing_in_film.film 7130

2040 people.person.place_of_birth 4537

1675	music.artist.origin	3932

1505	film.actor.film	8685

1050	music.artist.origin	3892

3020	film.actor.film	8354

2963	film.person_or_entity_appearing_in_film.film	8554

3624	film.person_or_entity_appearing_in_film.film	5312

92	people.person.place_of_birth	8686

2359	film.actor.film	7305

3133	film.actor.film	8021

2398	event.agent.performance	8687

413	film.person_or_entity_appearing_in_film.film	8688

3516	people.person.place_of_birth	4170

1598	film.person_or_entity_appearing_in_film.film	5885

1389	music.artist.origin	4214

2913	film.actor.film	5670

2913	film.person_or_entity_appearing_in_film.film	6464

2941	people.deceased_person.place_of_death	5161

974	film.person_or_entity_appearing_in_film.film	7010

2636	film.person_or_entity_appearing_in_film.film	4054

1353	film.actor.film	6543

484	film.actor.film	8542

3024	people.person.place_of_birth	7370

3738	games.publisher.games_published	8689

184	film.actor.film	8690

3786	people.person.place_of_birth	4963

2241	film.production_company.film	8691

2241	film.production_company.film	5963

2241	film.production_company.film	7873

2241	film.production_company.film	8118

3430	film.actor.film	8692

986 music.artist.origin 3961

3515 film.actor.film 8693

3667 music.artist.origin 4707

591 film.actor.film 4408

1995 film.actor.film 7522

3461 people.person.place_of_birth 8694

3632 film.actor.film 7856

341 film.film.genre 8432

3331 film.person_or_entity_appearing_in_film.film 6318

1357 film.person_or_entity_appearing_in_film.film 8695

3458 music.artist.origin 4094

3231 film.actor.film 8460

2148 people.person.place_of_birth 6242

2 music.artist.origin 5845

380 film.actor.film 7986

1402 people.person.place_of_birth 3966

485 music.artist.origin 4453

2010 film.person_or_entity_appearing_in_film.film 8696

2449 film.person_or_entity_appearing_in_film.film 8035

1962 film.person_or_entity_appearing_in_film.film 8697

3822 film.actor.film 8698

959 film.actor.film 6370

632 people.deceased_person.place_of_death 3846

2161 award.competitor.award_nominations 8699

2641 music.artist.origin 4046

879 film.actor.film 8368

2050 film.actor.film 4117

846 film.person_or_entity_appearing_in_film.film 5947

67 film.person_or_entity_appearing_in_film.film 4172

67 film.person_or_entity_appearing_in_film.film 6040

2210	film.actor.film	7086

1383	film.actor.film	8700

595 film.actor.film	8099

2389	film.actor.film	5451

3577	people.person.place_of_birth	3916

1187	film.actor.film	8701

239 people.person.place_of_birth	4362

1625	film.actor.film	8702

1945	film.actor.film	7047

1862	music.artist.origin	8703

2080	film.person_or_entity_appearing_in_film.film	4550

3598	film.person_or_entity_appearing_in_film.film	8704

584 film.person_or_entity_appearing_in_film.film	7701

2607	music.artist.origin	4583

77	film.person_or_entity_appearing_in_film.film	6250

2913	film.actor.film	8705

70	film.person_or_entity_appearing_in_film.film	8706

147 film.actor.film	4237

3179	film.person_or_entity_appearing_in_film.film	4728

3776	music.musician.instruments_played	4349

1840	film.actor.film	6748

3664	music.artist.origin	7675

3738	cvg.publisher.games_published	8043

3738	games.publisher.games_published 8566

901 film.writer.film	5429

2241	film.distributor.film	8707

2241	film.distributor.film	8708

2241	film.distributor.film	4713

795 film.person_or_entity_appearing_in_film.film	8709

1563	music.artist.origin	4057

2629 music.artist.origin 4453

1925 music.artist.origin 4057

1464 film.actor.film 7797

681 film.actor.film 4549

777 film.person_or_entity_appearing_in_film.film 8710

1777 film.person_or_entity_appearing_in_film.film 7767

591 film.person_or_entity_appearing_in_film.film 4915

3330 film.actor.film 6753

1360 film.person_or_entity_appearing_in_film.film 8711

2861 film.actor.film 5847

3242 film.producer.film 7051

2686 film.actor.film 5975

1526 film.actor.film 5260

3393 music.artist.origin 8712

2870 film.actor.film 7329

1048 music.artist.origin 3892

1954 film.actor.film 5483

241 film.person_or_entity_appearing_in_film.film 6933

3446 film.actor.film 7578

68 film.person_or_entity_appearing_in_film.film 8713

396 film.actor.film 8714

567 film.actor.film 6119

3827 film.actor.film 8715

1036 music.artist.origin 5403

2084 film.actor.film 8430

67 film.actor.film 6299

2337 film.actor.film 6771

564 music.artist.origin 5845

3364 film.person_or_entity_appearing_in_film.film 5359

16 film.actor.film 8716

1706	film.person_or_entity_appearing_in_film.film	4439

3741	film.person_or_entity_appearing_in_film.film	6629

377	award.winner.awards_won	7162

583	film.person_or_entity_appearing_in_film.film	8717

2386	people.person.place_of_birth	6323

3079	music.artist.origin	8718

2338	film.actor.film	4447

74	film.person_or_entity_appearing_in_film.film	4717

75	film.actor.film	4180

447	film.actor.film	5419

2636	film.person_or_entity_appearing_in_film.film	6777

2166	people.person.place_of_birth	5265

2096	film.person_or_entity_appearing_in_film.film	4547

366	film.person_or_entity_appearing_in_film.film	4457

3258	music.artist.origin	5934

2460	film.person_or_entity_appearing_in_film.film	8229

3504	people.person.nationality	3961

1864	people.person.place_of_birth	3850

602	film.actor.film	4846

3738	cvg.publisher.games_published	8719

625	film.actor.film	8720

625	music.artist.origin	3942

3462	music.artist.origin	8682

432	film.actor.film	5379

2241	film.distributor.film	8721

2241	film.distributor.film	4690

556	people.person.place_of_birth	6417

2487	film.person_or_entity_appearing_in_film.film	5085

1085	film.actor.film	8722

3296	film.actor.film	8723

1767	film.person_or_entity_appearing_in_film.film	7117

157 event.agent.performance	8439

3705	people.person.place_of_birth	4013

1623	film.person_or_entity_appearing_in_film.film	4149

3643	location.location.contained_by	6249

81	film.person_or_entity_appearing_in_film.film	8724

2711	film.person_or_entity_appearing_in_film.film	8725

1816	film.actor.film	8726

638 film.actor.film	5833

3132	film.actor.film	4355

2684	people.person.place_of_birth	7218

1662	film.actor.film	8164

3381	film.person_or_entity_appearing_in_film.film	8727

2635	film.actor.film	7947

3307	film.person_or_entity_appearing_in_film.film	8728

115 film.person_or_entity_appearing_in_film.film	4111

1633	film.person_or_entity_appearing_in_film.film	8590

1351	people.person.place_of_birth	3947

410 film.person_or_entity_appearing_in_film.film	8353

82	film.person_or_entity_appearing_in_film.film	7633

2422	people.person.place_of_birth	4081

2953	film.actor.film	4051

1169	music.artist.origin	4362

1473	film.actor.film	7405

1026	film.person_or_entity_appearing_in_film.film	8729

3040	film.person_or_entity_appearing_in_film.film	8730

413 film.person_or_entity_appearing_in_film.film	7454

645 film.actor.film	8731

71	music.artist.origin	4057

1901	music.artist.origin	5845

2742 music.artist.album 5658

3282 film.person_or_entity_appearing_in_film.film 8557

2338 film.person_or_entity_appearing_in_film.film 8371

386 film.person_or_entity_appearing_in_film.film 8732

52 music.artist.origin 4057

2914 people.person.place_of_birth 3870

2428 music.artist.origin 5061

70 film.actor.film 4867

70 film.actor.film 8733

2230 music.artist.origin 3961

2892 film.actor.film 6503

2096 film.person_or_entity_appearing_in_film.film 8734

2070 film.person_or_entity_appearing_in_film.film 8735

1981 film.actor.film 4341

2483 film.person_or_entity_appearing_in_film.film 8736

3738 cvg.publisher.games_published 8737

3738 cvg.publisher.games_published 6700

625 film.actor.film 6919

1463 type.object.key 1463

2241 film.distributor.film 4145

2241 film.distributor.film 5469

2241 film.production_company.film 4143

1936 film.person_or_entity_appearing_in_film.film 8738

669 music.artist.origin 3992

777 film.actor.film 7232

2487 film.actor.film 8084

387 film.actor.film 8739

2275 film.actor.film 5786

3486 film.director.film 7425

2390 film.person_or_entity_appearing_in_film.film 6317

3546 film.person_or_entity_appearing_in_film.film 8740

2100 film.person_or_entity_appearing_in_film.film 8741

673 people.person.place_of_birth 4232

1046 film.person_or_entity_appearing_in_film.film 5646

1271 music.artist.origin 4332

3843 music.artist.origin 4701

233 music.artist.origin 3864

794 film.actor.film 6872

3650 film.actor.film 8742

3648 film.actor.film 5375

1866 film.actor.film 8743

2343 music.artist.origin 4148

3722 music.musician.instruments_played 4349

1251 film.person_or_entity_appearing_in_film.film 8744

2419 film.person_or_entity_appearing_in_film.film 5485

997 film.person_or_entity_appearing_in_film.film 8405

900 music.artist.origin 8745

2635 film.person_or_entity_appearing_in_film.film 8746

1157 film.editor.film 4906

395 film.person_or_entity_appearing_in_film.film 4541

633 film.person_or_entity_appearing_in_film.film 4736

1960 film.person_or_entity_appearing_in_film.film 4378

1193 film.actor.film 7006

2737 film.actor.film 4069

413 film.actor.film 7454

2454 film.actor.film 6974

1434 film.person_or_entity_appearing_in_film.film 8283

2423 film.person_or_entity_appearing_in_film.film 7133

77 film.actor.film 6250

3094 people.person.place_of_birth 5064

2902 people.person.place_of_birth 8492

736 film.actor.film 8413

1306 music.artist.origin 4496

1245 people.person.place_of_birth 4008

974 film.person_or_entity_appearing_in_film.film 8747

437 film.actor.film 7537

2636 film.person_or_entity_appearing_in_film.film 8748

3821 film.person_or_entity_appearing_in_film.film 8749

2070 film.actor.film 6577

2070 film.actor.film 6903

1752 people.person.place_of_birth 5213

133 award.nominee.award_nominations 8614

294 film.person_or_entity_appearing_in_film.film 7317

3504 film.person_or_entity_appearing_in_film.film 6955

1848 film.actor.film 7773

3738 cvg.publisher.games_published 8750

901 film.writer.film 5290

899 film.actor.film 8751

1292 music.artist.origin 8752

1249 film.actor.film 8753

1249 film.person_or_entity_appearing_in_film.film 8754

2241 film.distributor.film 8363

2241 film.distributor.film 8755

2241 film.distributor.film 8756

2241 film.production_company.film 4888

2241 film.production_company.film 8628

2241 film.production_company.film 3930

1936 film.actor.film 8738

986 music.artist.origin 4552

3297 film.person_or_entity_appearing_in_film.film 6277

2487	film.person_or_entity_appearing_in_film.film	8757

2487	people.person.place_of_birth	4369

2648	film.actor.film	6926

3346	music.artist.origin	3966

2030	music.artist.origin	4010

1798	film.actor.film	8758

3607	film.person_or_entity_appearing_in_film.film	5387

2493	film.actor.film	8759

2504	music.artist.origin	4808

1371	music.musician.instruments_played	5367

1898	music.artist.origin	7399

740 film.person_or_entity_appearing_in_film.film	8760

2945	film.person_or_entity_appearing_in_film.film	7426

3065	music.musician.instruments_played	3851

3199	music.artist.origin	3864

2771	film.person_or_entity_appearing_in_film.film	8761

2088	music.artist.origin	3966

2635	film.actor.film	8762

115 film.actor.film	8763

1633	film.actor.film	8764

104 people.person.place_of_birth	3864

3035	film.actor.film	8019

878 event.agent.performance	4949

1473	film.actor.film	8073

1141	film.actor.film	6539

2337	film.actor.film	6023

1960	film.actor.film	5068

1187	film.person_or_entity_appearing_in_film.film	8171

2069	people.person.place_of_birth	3972

3396	film.actor.film	5526

976 film.actor.film 5110

1079 film.writer.film 4669

3498 film.actor.film 8765

2813 film.actor.film 5754

1406 music.artist.origin 3897

2913 film.actor.film 4671

646 film.person_or_entity_appearing_in_film.film 7641

95 music.artist.album 6946

2636 film.actor.film 8766

1458 film.person_or_entity_appearing_in_film.film 8643

1211 film.person_or_entity_appearing_in_film.film 8767

445 film.person_or_entity_appearing_in_film.film 8768

3217 film.actor.film 6350

278 film.actor.film 5721

484 film.person_or_entity_appearing_in_film.film 7529

3738 cvg.publisher.games_published 6641

3738 cvg.publisher.games_published 8769

2414 people.deceased_person.place_of_death 4057

901 film.actor.film 5995

3673 music.artist.track 7172

2241 film.distributor.film 7901

2241 film.distributor.film 5779

2241 film.production_company.film 7618

2241 film.production_company.film 8676

2380 people.person.profession 4221

964 people.person.place_of_birth 5662

1464 film.person_or_entity_appearing_in_film.film 7472

3139 film.actor.film 8770

3139 people.deceased_person.place_of_death 4347

1591 people.person.place_of_birth 6585

3644	people.person.place_of_birth	4369

2260	film.person_or_entity_appearing_in_film.film	8771

591	film.actor.film	4915

1461	music.artist.album	8772

1019	music.artist.origin	4583

865	film.actor.film	5715

1806	film.person_or_entity_appearing_in_film.film	4826

194	film.person_or_entity_appearing_in_film.film	4352

3062	music.artist.origin	4087

3331	film.actor.film	4584

1357	film.person_or_entity_appearing_in_film.film	6010

1216	music.artist.album	8219

3049	film.person_or_entity_appearing_in_film.film	6517

942	music.artist.origin	4057

391	music.artist.origin	4963

1954	film.person_or_entity_appearing_in_film.film	8773

2999	film.actor.film	7931

2630	film.actor.film	8034

3048	film.person_or_entity_appearing_in_film.film	8587

3132	film.actor.film	8774

1057	film.person_or_entity_appearing_in_film.film	4361

900	film.person_or_entity_appearing_in_film.film	6936

2076	film.person_or_entity_appearing_in_film.film	8261

3774	film.film.writer	8775

396	film.person_or_entity_appearing_in_film.film	8714

3384	film.actor.film	5876

1633	film.actor.film	8053

2362	film.person_or_entity_appearing_in_film.film	7636

785	film.person_or_entity_appearing_in_film.film	7534

1505	film.person_or_entity_appearing_in_film.film	8685

286 music.artist.origin 6555

2896 music.artist.origin 4896

67 film.actor.film 6912

1175 music.artist.album 6004

633 film.actor.film 4736

2337 film.actor.film 8776

645 film.actor.film 7463

3598 film.person_or_entity_appearing_in_film.film 8655

2217 film.actor.film 6632

1296 film.actor.film 8777

1973 people.person.profession 4221

1230 music.artist.origin 5666

3498 film.actor.film 6719

1704 film.actor.film 8057

672 film.person_or_entity_appearing_in_film.film 6978

1773 film.actor.film 7163

2636 film.person_or_entity_appearing_in_film.film 7539

1065 music.artist.origin 4024

679 film.person_or_entity_appearing_in_film.film 6231

2070 film.person_or_entity_appearing_in_film.film 6541

2689 music.artist.origin 4195

1630 music.artist.origin 5161

294 film.actor.film 4184

1840 film.person_or_entity_appearing_in_film.film 8778

1000 music.artist.origin 4050

602 music.producer.tracks_produced 8541

1364 music.artist.origin 4057

821 music.artist.origin 4010

3738 cvg.publisher.games_published 8779

2241 film.distributor.film 8780

2241 film.production_company.film 5294

1936 film.actor.film 4894

1942 people.person.place_of_birth 4583

430 film.person_or_entity_appearing_in_film.film 5935

982 music.artist.origin 4058

1624 film.actor.film 7853

3438 music.artist.origin 3866

956 film.person_or_entity_appearing_in_film.film 8781

1656 film.person_or_entity_appearing_in_film.film 8782

1360 film.actor.film 8783

1357 film.actor.film 6930

253 film.person_or_entity_appearing_in_film.film 5047

677 music.artist.album 5496

1660 film.actor.film 8784

2244 film.film.star 5048

2248 music.artist.origin 7915

3390 film.actor.film 8485

3390 film.person_or_entity_appearing_in_film.film 8484

3390 film.person_or_entity_appearing_in_film.film 8785

1474 film.person_or_entity_appearing_in_film.film 6932

2424 music.artist.origin 4442

3573 music.artist.origin 4013

1816 film.person_or_entity_appearing_in_film.film 6873

1545 music.artist.origin 7212

2312 film.person_or_entity_appearing_in_film.film 8786

1705 film.person_or_entity_appearing_in_film.film 6521

3125 music.artist.origin 4471

997 film.person_or_entity_appearing_in_film.film 8664

396 film.person_or_entity_appearing_in_film.film 6521

2297 film.person_or_entity_appearing_in_film.film 8787

2635 film.person_or_entity_appearing_in_film.film 7504

2635 film.person_or_entity_appearing_in_film.film 8762

1719 film.person_or_entity_appearing_in_film.film 4168

1720 film.director.film 8393

3827 film.person_or_entity_appearing_in_film.film 4756

1759 music.artist.origin 4057

1153 film.actor.film 8788

1153 film.person_or_entity_appearing_in_film.film 8444

1153 film.person_or_entity_appearing_in_film.film 8789

2963 film.actor.film 8790

1383 film.person_or_entity_appearing_in_film.film 5573

3555 film.person_or_entity_appearing_in_film.film 3939

3792 film.person_or_entity_appearing_in_film.film 6503

1473 film.person_or_entity_appearing_in_film.film 4661

353 film.person_or_entity_appearing_in_film.film 7559

478 music.artist.origin 4170

2998 film.person_or_entity_appearing_in_film.film 6094

2768 music.album.genre 4231

1983 film.person_or_entity_appearing_in_film.film 4068

1209 film.person_or_entity_appearing_in_film.film 6744

2376 music.artist.origin 4057

192 music.artist.track 8791

226 music.artist.origin 3961

3295 film.actor.film 8792

3447 people.person.place_of_birth 3972

1079 film.editor.film 6169

2848 film.film.rating 4223

1517 people.deceased_person.place_of_death 3846

1208 film.actor.film 8793

2676 people.deceased_person.place_of_death 5270

2475 film.person_or_entity_appearing_in_film.film 8317

2674 music.artist.album 8794

1211 people.person.place_of_birth 8795

3179 film.actor.film 8041

657 music.artist.album 4179

1630 film.actor.film 6231

278 film.actor.film 8796

3377 people.person.place_of_birth 5160

436 film.actor.film 6246

2293 music.musician.instruments_played 4349

2241 film.distributor.film 8797

2241 film.distributor.film 8798

2241 film.distributor.film 7706

2241 film.production_company.film 8799

2241 film.production_company.film 8141

2241 film.production_company.film 8800

2241 film.production_company.film 7620

2333 music.artist.origin 3945

419 film.person_or_entity_appearing_in_film.film 4494

1464 film.actor.film 8801

1624 film.actor.film 8802

894 film.person_or_entity_appearing_in_film.film 8803

1767 people.deceased_person.place_of_death 3846

1437 film.person_or_entity_appearing_in_film.film 8619

643 film.person_or_entity_appearing_in_film.film 8069

341 film.film.genre 3968

3331 film.actor.film 6613

3331 film.actor.film 8804

1357 film.actor.film 8695

2933 music.artist.origin 4174

292 people.person.place_of_birth 8805

1496 music.artist.origin 5139

1202 music.artist.origin 5213

3321 film.actor.film 6900

3321 film.person_or_entity_appearing_in_film.film 8806

2841 music.artist.origin 4121

444 music.artist.origin 4454

2452 film.person_or_entity_appearing_in_film.film 5310

923 film.person_or_entity_appearing_in_film.film 8103

2312 film.director.film 8786

2036 music.artist.origin 4552

2449 film.person_or_entity_appearing_in_film.film 4385

3551 film.actor.film 8807

422 music.artist.album 5746

1365 film.actor.film 6335

567 film.person_or_entity_appearing_in_film.film 8808

671 music.artist.origin 7930

443 music.artist.origin 6555

2494 film.person_or_entity_appearing_in_film.film 5489

1720 film.writer.film 6396

709 music.artist.origin 6948

3317 film.person_or_entity_appearing_in_film.film 8291

2921 music.artist.origin 4502

2307 music.artist.origin 4536

2603 film.person_or_entity_appearing_in_film.film 8170

2998 film.person_or_entity_appearing_in_film.film 8809

2265 people.person.place_of_birth 4362

987 film.actor.film 7952

648 music.artist.origin 4272

1470 music.musician.instruments_played 3851

974 film.person_or_entity_appearing_in_film.film 8810

2636 film.actor.film 8811

2096 film.actor.film 7954

2597 film.person_or_entity_appearing_in_film.film 5808

1458 film.person_or_entity_appearing_in_film.film 7067

2663 music.artist.origin 4050

294 film.person_or_entity_appearing_in_film.film 8812

771 film.person_or_entity_appearing_in_film.film 5667

1840 film.person_or_entity_appearing_in_film.film 8564

484 film.person_or_entity_appearing_in_film.film 8813

3738 cvg.publisher.games_published 4678

3738 cvg.publisher.games_published 8814

3738 games.publisher.games_published 8769

184 film.actor.film 5955

436 film.person_or_entity_appearing_in_film.film 7816

2159 film.person_or_entity_appearing_in_film.film 5979

442 music.musician.instruments_played 3851

442 people.person.place_of_birth 3855

2241 film.production_company.film 8721

2203 music.artist.track 8447

1937 music.artist.origin 3961

1464 film.person_or_entity_appearing_in_film.film 8451

2897 film.actor.film 8815

2126 film.person_or_entity_appearing_in_film.film 5673

3031 film.actor.film 4910

2880 film.actor.film 8068

2677 film.person_or_entity_appearing_in_film.film 4786

3779 film.person_or_entity_appearing_in_film.film 4415

1059 film.person_or_entity_appearing_in_film.film 5304

434 film.person_or_entity_appearing_in_film.film 8338

2701 music.artist.origin 6435

3696 music.artist.origin 4058

2711 film.person_or_entity_appearing_in_film.film 8816

533 film.person_or_entity_appearing_in_film.film 8259

1479 film.person_or_entity_appearing_in_film.film 8817

2669 people.person.place_of_birth 4010

997 film.actor.film 8818

115 film.actor.film 5687

125 music.artist.origin 7370

1886 music.artist.origin 4896

1720 film.writer.film 8819

3367 music.artist.origin 5061

1891 film.person_or_entity_appearing_in_film.film 8640

674 film.person_or_entity_appearing_in_film.film 6652

674 film.person_or_entity_appearing_in_film.film 7402

2359 film.person_or_entity_appearing_in_film.film 8555

1473 film.person_or_entity_appearing_in_film.film 6769

595 film.person_or_entity_appearing_in_film.film 6693

1994 music.artist.origin 4087

1706 film.actor.film 5323

39 film.person_or_entity_appearing_in_film.film 4920

1885 film.actor.film 8820

557 film.person_or_entity_appearing_in_film.film 7743

1902 music.artist.origin 4272

646 film.actor.film 5216

2562 film.person_or_entity_appearing_in_film.film 8821

70 film.person_or_entity_appearing_in_film.film 8822

3053 music.artist.origin 4963

3044 film.person_or_entity_appearing_in_film.film 8823

2070 film.actor.film 8025

519 people.person.place_of_birth 3895

49 film.person_or_entity_appearing_in_film.film 7092

3166 film.actor.film 6638

3041 music.musician.instruments_played 4349

3738 games.publisher.games_published 4343

184 film.actor.film 8824

96 film.person_or_entity_appearing_in_film.film 3848

442 music.artist.album 8825

2241 film.distributor.film 8826

2241 film.production_company.film 8827

3019 people.person.place_of_birth 4094

2779 film.actor.film 8828

2794 music.artist.origin 8111

2487 film.actor.film 8757

2487 film.person_or_entity_appearing_in_film.film 6986

2311 film.person_or_entity_appearing_in_film.film 8829

2126 film.actor.film 7049

1360 film.person_or_entity_appearing_in_film.film 6186

1567 film.actor.film 4695

2065 music.artist.origin 4050

2678 film.actor.film 8830

2860 film.person_or_entity_appearing_in_film.film 8831

3321 film.actor.film 6871

1135 music.artist.origin 8805

81 film.actor.film 8832

81 film.actor.film 4749

1816 film.actor.film 5613

24 music.artist.origin 6457

1004 people.person.profession 4221

3132 film.actor.film 5847

959 music.artist.album 8833

3774 film.film.actor 6714

3774 film.film.director 8775

2635 film.actor.film 8834

2635 film.person_or_entity_appearing_in_film.film 8835

1157 film.person_or_entity_appearing_in_film.film 8471

1415 music.artist.origin 4852

1153 film.actor.film 3890

1672 people.person.place_of_birth 4057

190 film.actor.film 8279

1032 film.person_or_entity_appearing_in_film.film 6491

38 music.artist.track 4961

3555 film.actor.film 3939

1473 film.person_or_entity_appearing_in_film.film 6743

1473 film.person_or_entity_appearing_in_film.film 7768

235 event.agent.performance 4099

2337 film.actor.film 8836

850 film.person_or_entity_appearing_in_film.film 6217

2484 people.person.place_of_birth 8837

3784 film.editor.film 4601

792 music.artist.track 8311

3598 film.actor.film 5164

508 award.nominee.award_nominations 8838

980 film.person_or_entity_appearing_in_film.film 8577

2913 film.actor.film 5919

2914 film.actor.film 5929

3641 people.person.place_of_birth 5826

1601 film.actor.film 8839

1559 music.artist.track 8840

2636 film.actor.film 4131

2636 film.person_or_entity_appearing_in_film.film 8841

45 film.actor.film 4411

568 film.person_or_entity_appearing_in_film.film 8842

147 film.actor.film 7928

1403 film.actor.film 8843

2070 film.actor.film 6104

1211 award.nominee.award_nominations 7956

2122 film.actor.film 8844

173 music.artist.origin 5194

49 film.actor.film 8845

1840 film.person_or_entity_appearing_in_film.film 3923

3738 cvg.publisher.games_published 8846

100 people.person.profession 4221

2400 type.object.key 2400

999 film.actor.film 8001

2215 film.person_or_entity_appearing_in_film.film 7682

2360 film.actor.film 5901

2241 film.production_company.film 8627

364 people.person.place_of_birth 8847

2266 people.person.place_of_birth 6276

681 film.actor.film 5969

681 film.actor.film 4250

387 film.person_or_entity_appearing_in_film.film 8848

2206 film.actor.film 8631

3547 tv.program.country_of_origin 6249

1164 music.artist.origin 8694

2363 people.person.place_of_birth 5208

3458 film.actor.film 5091

1623 people.person.place_of_birth 4347

3065 people.person.place_of_birth 3870

2711	film.person_or_entity_appearing_in_film.film	8500

2711	film.person_or_entity_appearing_in_film.film	8849

1816	film.writer.film	6873

306 film.actor.film	6600

2306	film.person_or_entity_appearing_in_film.film	5396

2938	music.artist.album	8850

2635	film.actor.film	8851

3384	film.person_or_entity_appearing_in_film.film	7805

2668	film.person_or_entity_appearing_in_film.film	5791

548 film.actor.film	6559

3827	film.person_or_entity_appearing_in_film.film	8852

1224	music.artist.origin	4087

1476	music.artist.album	3979

3061	people.person.place_of_birth	4077

3230	music.artist.origin	8586

2998	film.person_or_entity_appearing_in_film.film	8853

127 film.actor.film	3957

3598	film.actor.film	8854

3598	people.person.place_of_birth	3866

2214	music.producer.tracks_produced	8855

3844	music.artist.origin	3899

3512	film.person_or_entity_appearing_in_film.film	4385

3094	film.person_or_entity_appearing_in_film.film	5416

128 film.actor.film	8435

2636	film.person_or_entity_appearing_in_film.film	6140

2636	film.person_or_entity_appearing_in_film.film	5991

2096	film.actor.film	8660

2070	film.actor.film	8856

1206	film.person_or_entity_appearing_in_film.film	4558

2213	music.artist.origin	4013

2744 music.artist.origin 4199

3366 film.person_or_entity_appearing_in_film.film 4774

3366 music.musician.instruments_played 6942

466 film.actor.film 6639

602 film.person_or_entity_appearing_in_film.film 6661

3738 games.publisher.games_published 5030

3738 games.publisher.games_published 5772

901 film.actor.film 5429

2867 film.person_or_entity_appearing_in_film.film 8857

2241 film.production_company.film 8858

2241 film.production_company.film 5433

2241 film.production_company.film 8708

2267 music.artist.origin 8686

451 award.competitor.award_nominations 8336

681 people.person.profession 4221

1990 music.artist.origin 6361

382 film.actor.film 7622

894 film.person_or_entity_appearing_in_film.film 5041

1697 film.actor.film 8009

2880 film.actor.film 8859

1360 film.person_or_entity_appearing_in_film.film 8860

1948 music.artist.origin 4171

341 film.film.language 4076

3000 film.person_or_entity_appearing_in_film.film 8184

3049 film.person_or_entity_appearing_in_film.film 5609

3049 film.person_or_entity_appearing_in_film.film 8861

2064 event.agent.performance 4548

3694 music.artist.origin 4217

1954 film.actor.film 8773

3742 film.actor.film 5050

1387	film.person_or_entity_appearing_in_film.film	8585

1323	film.actor.film	7800

3648	film.actor.film	8862

241 film.person_or_entity_appearing_in_film.film	8863

465 film.person_or_entity_appearing_in_film.film	8864

3507	film.person_or_entity_appearing_in_film.film	7970

906 music.artist.origin	5521

997 film.person_or_entity_appearing_in_film.film	8865

3490	music.artist.origin	8866

2695	music.musician.instruments_played	3851

2161	award.competitor.awards_won	6526

2725	people.deceased_person.place_of_death	4013

492 film.person_or_entity_appearing_in_film.film	8591

3496	film.film.genre	4255

3594	music.artist.album	5040

808 music.artist.origin	6555

1473	film.actor.film	5596

136 film.actor.film	8867

3215	film.film.star	8222

1935	people.person.place_of_birth	8795

1209	film.actor.film	4494

3372	film.person_or_entity_appearing_in_film.film	6335

767 film.person_or_entity_appearing_in_film.film	7563

2913	film.actor.film	7512

1208	film.person_or_entity_appearing_in_film.film	8793

2871	music.artist.origin	3946

2650	film.film.country	3961

2070	film.person_or_entity_appearing_in_film.film	8868

2171	music.artist.origin	5019

1458	film.actor.film	8869

270 film.person_or_entity_appearing_in_film.film 4675

3666 music.artist.origin 4454

1330 film.actor.film 5500

3738 cvg.publisher.games_published 8042

3738 cvg.publisher.games_published 8870

3452 film.actor.film 8871

2867 film.person_or_entity_appearing_in_film.film 8360

2241 film.distributor.film 6091

2241 film.distributor.film 5881

2241 film.distributor.film 5778

2241 film.distributor.film 7447

182 music.artist.origin 4896

1713 people.person.place_of_birth 6095

2350 music.artist.origin 5127

894 film.person_or_entity_appearing_in_film.film 8872

3546 film.actor.film 7097

864 music.artist.origin 4214

2531 type.object.key 2531

2860 film.actor.film 8831

2143 people.deceased_person.place_of_death 4391

1526 film.actor.film 3937

3659 people.person.place_of_birth 4357

157 film.actor.film 5533

2979 film.person_or_entity_appearing_in_film.film 5976

1468 film.actor.film 8584

1623 film.person_or_entity_appearing_in_film.film 5193

3032 music.artist.origin 5161

2372 people.person.place_of_birth 4552

81 film.actor.film 8340

3432 music.artist.origin 5270

2295	music.artist.origin	7915
2711	film.person_or_entity_appearing_in_film.film	7783
2658	film.actor.film	6017
1814	film.film.director7906
3763	film.person_or_entity_appearing_in_film.film	8873
2904	film.person_or_entity_appearing_in_film.film	7027
3599	film.actor.film	6047
1092	film.actor.film	5310
2550	people.person.place_of_birth	5605
480 film.person_or_entity_appearing_in_film.film	8874
1045	film.person_or_entity_appearing_in_film.film	5103
2825	music.artist.origin	4057
3827	film.person_or_entity_appearing_in_film.film	5007
3035	film.actor.film	8875
2172	music.artist.origin	7554
912 people.deceased_person.place_of_death	4362
633 film.actor.film	6691
1155	film.actor.film	6980
2337	film.person_or_entity_appearing_in_film.film	8836
1264	music.artist.album	8876
2737	film.person_or_entity_appearing_in_film.film	8410
1845	film.actor.film	7007
2080	film.person_or_entity_appearing_in_film.film	7436
2080	film.person_or_entity_appearing_in_film.film	7409
3840	music.artist.origin	4732
948 people.person.place_of_birth	8877
2259	film.person_or_entity_appearing_in_film.film	8355
2848	film.film.genre	4062
675 film.actor.film	4450
967 award.nominee.award_nominations	6213

1773 film.person_or_entity_appearing_in_film.film 4234

3192 music.artist.origin 4050

2636 film.actor.film 7771

2636 film.person_or_entity_appearing_in_film.film 8878

2070 film.actor.film 8879

445 film.person_or_entity_appearing_in_film.film 4613

3738 games.publisher.games_published 8846

2882 music.artist.origin 4454

3248 film.actor.film 4396

1249 film.person_or_entity_appearing_in_film.film 6638

1463 film.person_or_entity_appearing_in_film.film 6816

2241 film.distributor.film 8880

2241 film.distributor.film 5864

2241 film.production_company.film 8881

2241 film.production_company.film 6895

2203 music.artist.origin 8882

1464 film.person_or_entity_appearing_in_film.film 8883

1464 film.person_or_entity_appearing_in_film.film 6275

3297 film.person_or_entity_appearing_in_film.film 7874

1624 film.actor.film 5751

1618 film.actor.film 8884

1240 people.person.place_of_birth 6036

894 film.person_or_entity_appearing_in_film.film 7233

2732 music.artist.origin 8885

3354 film.person_or_entity_appearing_in_film.film 5485

1656 film.actor.film 8886

2055 music.artist.origin 4991

3671 music.artist.origin 5845

2766 people.deceased_person.place_of_death 3846

3388 people.person.place_of_birth 4616

538 film.actor.film 8887

36 music.artist.track 7922

3049 film.actor.film 8679

157 film.person_or_entity_appearing_in_film.film 5533

3231 film.actor.film 5674

740 film.actor.film 8760

1105 film.director.film 6586

298 film.actor.film 7550

547 film.person_or_entity_appearing_in_film.film 8888

3793 music.artist.origin 7091

2223 music.artist.origin 4552

68 film.actor.film 8713

2635 film.person_or_entity_appearing_in_film.film 6356

1535 music.artist.origin 8889

1157 film.writer.film 6998

379 film.actor.film 8391

1232 film.actor.film 4653

2875 film.person_or_entity_appearing_in_film.film 8890

3106 film.person_or_entity_appearing_in_film.film 7432

1153 film.actor.film 7275

2141 film.person_or_entity_appearing_in_film.film 5728

67 film.actor.film 8168

2804 film.actor.film 8891

3216 tv.program.regular_cast 8892

136 film.person_or_entity_appearing_in_film.film 6808

3328 music.musician.instruments_played 4349

1960 film.actor.film 4067

1813 film.actor.film 6303

1935 film.actor.film 8893

604 film.actor.film 4281

245 music.artist.origin 3961

1442 music.artist.origin 3961

80 film.person_or_entity_appearing_in_film.film 7065

80 music.artist.track 7564

2913 film.actor.film 4142

1469 film.actor.film 7344

458 music.artist.album 5697

1846 film.person_or_entity_appearing_in_film.film 8894

1211 film.actor.film 8767

49 film.person_or_entity_appearing_in_film.film 8895

2460 film.actor.film 4507

79 film.person_or_entity_appearing_in_film.film 6970

484 film.actor.film 4778

2414 award.nominee.award_nominations 8896

2400 film.actor.film 8897

2400 film.actor.film 5918

432 type.object.subject_key 432

2241 film.distributor.film 8601

2241 film.distributor.film 8384

2241 film.production_company.film 8898

2241 film.production_company.film 6341

795 film.actor.film 8709

3195 music.artist.origin 4362

2487 film.person_or_entity_appearing_in_film.film 8899

1618 film.actor.film 4407

1618 music.musician.instruments_played 5619

3090 music.artist.origin 4010

3789 award.competitor.awards_won 8386

3486 people.person.place_of_birth 7911

1360 film.actor.film 5384

2206	film.person_or_entity_appearing_in_film.film	8900

2731	film.actor.film	8441

1806	film.actor.film	4826

1475	film.person_or_entity_appearing_in_film.film	7886

2861	film.actor.film	5046

3242	film.person_or_entity_appearing_in_film.film	8901

434 film.actor.film	8499

3537	music.artist.origin	4454

1695	film.actor.film	7525

2207	film.actor.film	5483

1105	people.person.place_of_birth	6948

378 music.artist.origin	8902

3026	film.actor.film	6558

3390	film.person_or_entity_appearing_in_film.film	8256

3390	film.person_or_entity_appearing_in_film.film	6559

3648	film.actor.film	8903

2711	film.person_or_entity_appearing_in_film.film	5612

3475	film.person_or_entity_appearing_in_film.film	6906

2306	people.person.place_of_birth	4496

2833	film.actor.film	6907

2635	film.person_or_entity_appearing_in_film.film	8904

480 film.actor.film	8874

888 music.artist.origin	7759

115 film.person_or_entity_appearing_in_film.film	8502

115 film.person_or_entity_appearing_in_film.film	4543

818 people.person.place_of_birth	4362

1633	film.person_or_entity_appearing_in_film.film	8905

2792	film.person_or_entity_appearing_in_film.film	8455

3827	film.person_or_entity_appearing_in_film.film	8704

446 film.actor.film	8906

662 film.person_or_entity_appearing_in_film.film 4467

1153 film.actor.film 8907

3054 film.person_or_entity_appearing_in_film.film 5356

67 film.person_or_entity_appearing_in_film.film 7655

633 film.person_or_entity_appearing_in_film.film 6971

2359 film.actor.film 7951

1117 music.artist.origin 6545

3087 music.musician.instruments_played 4349

2337 film.person_or_entity_appearing_in_film.film 6302

3785 music.artist.origin 3864

127 film.person_or_entity_appearing_in_film.film 4717

3725 music.artist.origin 4050

2831 people.person.place_of_birth 4191

453 film.person_or_entity_appearing_in_film.film 7868

1845 film.actor.film 8908

84 people.person.place_of_birth 3944

3540 music.artist.origin 8233

1035 film.person_or_entity_appearing_in_film.film 7717

52 event.agent.performance 4099

2866 music.album.genre 8909

2913 film.actor.film 7746

2913 film.person_or_entity_appearing_in_film.film 5157

151 film.actor.film 5023

2830 film.actor.film 8314

316 music.artist.origin 5061

1198 music.artist.origin 5977

1570 film.actor.film 7170

2597 film.person_or_entity_appearing_in_film.film 6540

160 music.artist.album 8582

2819 people.person.place_of_birth 4057

527 film.actor.film 7380

484 film.actor.film 8813

484 film.actor.film 6956

2877 film.person_or_entity_appearing_in_film.film 5493

1244 people.person.place_of_birth 4008

901 film.person_or_entity_appearing_in_film.film 8910

1787 film.person_or_entity_appearing_in_film.film 7645

2159 people.person.place_of_birth 4133

2241 film.distributor.film 4571

2241 film.production_company.film 5336

469 people.person.profession 5021

1772 film.actor.film 6927

1800 music.artist.origin 4362

3665 people.deceased_person.place_of_death 3864

1475 film.person_or_entity_appearing_in_film.film 8911

1417 film.person_or_entity_appearing_in_film.film 8912

1113 people.person.place_of_birth 4170

3045 film.actor.film 8913

814 film.person_or_entity_appearing_in_film.film 8303

1571 music.artist.origin 6095

2092 music.artist.origin 5405

2010 film.actor.film 8696

1479 film.actor.film 7104

1962 film.actor.film 4653

2281 music.artist.origin 3972

735 film.person_or_entity_appearing_in_film.film 7854

111 music.artist.origin 6343

379 film.person_or_entity_appearing_in_film.film 8684

540 film.person_or_entity_appearing_in_film.film 3973

2473 film.person_or_entity_appearing_in_film.film 8431

541 film.person_or_entity_appearing_in_film.film 5203

3827 film.person_or_entity_appearing_in_film.film 8914

1153 film.person_or_entity_appearing_in_film.film 8788

912 film.actor.film 5323

656 people.person.place_of_birth 6545

3412 film.film.star 8622

2829 people.person.place_of_birth 3860

2125 film.actor.film 6090

2313 music.artist.origin 5213

1473 film.actor.film 5449

2324 film.film.editor 5574

1187 film.person_or_entity_appearing_in_film.film 8701

279 film.actor.film 6347

1885 film.person_or_entity_appearing_in_film.film 7770

3244 music.artist.origin 4010

1469 film.person_or_entity_appearing_in_film.film 7936

646 film.person_or_entity_appearing_in_film.film 7412

3149 people.person.place_of_birth 8578

868 music.artist.origin 4357

2892 film.actor.film 4423

2096 film.actor.film 8457

3255 music.artist.origin 4739

1098 music.artist.origin 3932

445 film.person_or_entity_appearing_in_film.film 8397

278 film.person_or_entity_appearing_in_film.film 8915

2665 people.deceased_person.place_of_death 4927

3738 cvg.publisher.games_published 6781

2693 music.artist.origin 5756

899 film.person_or_entity_appearing_in_film.film 8751

2241 film.distributor.film 8161

2241	film.distributor.film	8916

2241	film.distributor.film	8423

2241	film.production_company.film	5435

1936	film.actor.film	7289

364 music.artist.origin	8847

1464	film.actor.film	8917

777 film.person_or_entity_appearing_in_film.film	6342

2487	film.actor.film	8649

1767	film.person_or_entity_appearing_in_film.film	4305

2880	film.person_or_entity_appearing_in_film.film	5303

2900	music.artist.origin	4050

2707	film.person_or_entity_appearing_in_film.film	5908

1278	film.person_or_entity_appearing_in_film.film	7098

1357	film.actor.film	8012

2184	film.actor.film	5718

2861	film.actor.film	6730

434 film.person_or_entity_appearing_in_film.film	8918

2493	film.person_or_entity_appearing_in_film.film	8183

3323	music.artist.origin	4852

577 music.artist.origin	6361

2207	film.actor.film	4997

2207	film.person_or_entity_appearing_in_film.film	8919

2945	people.person.place_of_birth	4499

81	film.actor.film	8724

3026	film.actor.film	4264

3132	film.actor.film	6288

637 film.actor.film	6193

3544	film.person_or_entity_appearing_in_film.film	8920

384 people.person.place_of_birth	5660

1962	film.actor.film	8921

687 music.artist.origin 6374

997 film.person_or_entity_appearing_in_film.film 8922

959 award.competitor.award_nominations 8163

2904 film.actor.film 8923

459 people.person.profession 4990

567 film.actor.film 4533

2695 film.writer.film 8924

2507 music.producer.tracks_produced 4370

410 film.actor.film 8925

541 film.person_or_entity_appearing_in_film.film 5149

483 music.artist.origin 3864

1153 film.actor.film 5568

1153 film.person_or_entity_appearing_in_film.film 8926

846 film.actor.film 7156

1833 film.person_or_entity_appearing_in_film.film 8927

2359 film.actor.film 7788

1136 music.artist.origin 6425

1026 film.person_or_entity_appearing_in_film.film 8533

3133 film.person_or_entity_appearing_in_film.film 7277

2832 people.person.profession 8928

136 film.actor.film 8929

2700 film.person_or_entity_appearing_in_film.film 8930

3119 music.musician.instruments_played 7814

3733 film.person_or_entity_appearing_in_film.film 8931

539 film.person_or_entity_appearing_in_film.film 3973

3598 film.actor.film 8472

508 film.person_or_entity_appearing_in_film.film 4669

77 film.person_or_entity_appearing_in_film.film 8932

361 film.person_or_entity_appearing_in_film.film 6792

1744 film.person_or_entity_appearing_in_film.film 4871

472 music.artist.origin 3914

2636 film.actor.film 7811

3044 film.actor.film 8823

2597 film.director.film 5879

2122 film.actor.film 5447

445 film.actor.film 8768

1320 location.location.contained_by 3961

1307 film.actor.film 8933

2483 film.person_or_entity_appearing_in_film.film 8934

3738 games.publisher.games_published 7319

2898 music.artist.origin 5807

999 film.actor.film 6894

826 music.artist.origin 7930

1249 film.actor.film 7774

587 fictional_universe.character.creator 7500

3001 people.person.place_of_birth 6457

2241 film.distributor.film 5132

2241 film.distributor.film 6551

2241 film.production_company.film 6550

2241 film.production_company.film 8935

2241 film.production_company.film 8936

3139 film.person_or_entity_appearing_in_film.film 5117

2780 film.person_or_entity_appearing_in_film.film 8937

2487 film.actor.film 8938

2078 film.actor.film 6449

894 film.actor.film 8424

670 music.artist.origin 4058

3607 film.actor.film 5447

2247 music.artist.origin 8125

3525 people.person.place_of_birth 8939

1241 film.film.editor 4153

3180 people.person.profession 4221

3184 people.person.place_of_birth 8940

892 film.actor.film 7175

2658 film.person_or_entity_appearing_in_film.film 4159

1004 people.person.place_of_birth 7516

1405 event.agent.performance 4949

206 film.person_or_entity_appearing_in_film.film 8941

3566 music.artist.origin 3982

1720 film.director.film 5878

1989 film.person_or_entity_appearing_in_film.film 4115

2709 film.actor.film 6910

1153 film.actor.film 8609

3020 film.actor.film 7765

2359 film.actor.film 8942

3412 film.film.director 8445

3068 people.deceased_person.place_of_death 4013

3364 film.actor.film 7887

1625 film.actor.film 3912

1575 music.artist.origin 3961

3109 film.actor.film 8943

1812 music.musician.instruments_played 3959

1601 film.person_or_entity_appearing_in_film.film 7191

2636 film.person_or_entity_appearing_in_film.film 8811

2096 film.actor.film 6579

3047 music.artist.origin 5403

7 music.artist.origin 4003

2548 music.artist.origin 4150

2293 film.actor.film 7722

1154 film.person_or_entity_appearing_in_film.film 8944

1603	music.artist.origin	8945

1002	film.actor.film	8383

3248	film.actor.film	8231

609 music.artist.origin	3946

3467	people.deceased_person.place_of_death	8946

2241	film.distributor.film	8947

2241	film.distributor.film	7045

2241	film.production_company.film	6702

2241	film.production_company.film	8005

2241	film.production_company.film	5297

1936	film.actor.film	8948

481 film.actor.film	7380

1784	people.person.place_of_birth	8525

777 film.actor.film	5712

2487	film.person_or_entity_appearing_in_film.film	8949

610 music.artist.origin	4013

1085	film.person_or_entity_appearing_in_film.film	8722

622 film.actor.film	7076

816 film.actor.film	5801

2126	film.person_or_entity_appearing_in_film.film	7855

1767	film.actor.film	8950

1768	people.person.place_of_birth	7857

3031	film.person_or_entity_appearing_in_film.film	8238

3221	people.person.profession	4221

2861	film.person_or_entity_appearing_in_film.film	8951

1664	film.actor.film	8389

3231	film.actor.film	8255

2191	music.artist.origin	3926

2711	film.person_or_entity_appearing_in_film.film	5185

638 film.person_or_entity_appearing_in_film.film	8952

884 music.artist.origin 3864

3551 film.actor.film 8953

68 film.actor.film 5268

2938 film.actor.film 4421

3800 music.artist.origin 3846

2635 film.person_or_entity_appearing_in_film.film 4051

2635 film.producer.film 8364

115 film.actor.film 8200

2161 event.agent.performance 8687

1391 music.artist.origin 4471

1232 film.actor.film 6763

785 film.actor.film 4907

548 film.person_or_entity_appearing_in_film.film 6293

2724 film.person_or_entity_appearing_in_film.film 4907

3317 film.actor.film 6599

3463 music.artist.origin 4380

878 event.agent.performance 4548

571 people.person.place_of_birth 4362

2370 film.actor.film 8954

3792 film.actor.film 6503

1473 film.actor.film 7996

2450 people.person.place_of_birth 4496

2271 music.artist.origin 7955

672 film.actor.film 5063

2555 film.actor.film 6590

628 film.actor.film 8538

3120 film.actor.film 4075

3575 music.artist.origin 3986

1453 film.actor.film 6617

1601 film.person_or_entity_appearing_in_film.film 8955

3703	location.location.contained_by	6405
2649	music.artist.origin	4796
1320	location.location.contained_by	8956
1840	film.actor.film	8778
3176	film.person_or_entity_appearing_in_film.film	8957
3738	cvg.publisher.games_published	5372
3738	games.publisher.games_published	8958
2400	type.object.subject_key	2400
901	film.person_or_entity_appearing_in_film.film	5957
899	film.actor.film	4297
2241	film.distributor.film	8959
2241	film.production_company.film	8960
2859	music.artist.origin	5569
1618	film.person_or_entity_appearing_in_film.film	4671
2126	film.person_or_entity_appearing_in_film.film	8237
1697	film.person_or_entity_appearing_in_film.film	8678
1177	music.artist.origin	4454
534	film.person_or_entity_appearing_in_film.film	4794
1112	music.musician.instruments_played	3851
1357	film.person_or_entity_appearing_in_film.film	7782
2812	music.artist.origin	4094
1496	film.person_or_entity_appearing_in_film.film	4124
2504	people.person.place_of_birth	4808
1919	music.artist.origin	7433
57	film.actor.film	6901
2279	people.person.place_of_birth	3942
2372	film.actor.film	7296
3360	people.person.place_of_birth	6077
720	film.person_or_entity_appearing_in_film.film	7238
460	film.person_or_entity_appearing_in_film.film	7454

2373 film.person_or_entity_appearing_in_film.film 8144

105 music.artist.origin 4362

380 film.actor.film 6851

1816 film.actor.film 7987

640 music.artist.origin 3942

241 film.actor.film 8863

29 music.artist.origin 3897

1074 film.actor.film 7024

2195 music.artist.origin 3864

3508 film.actor.film 6251

1479 film.actor.film 7240

193 film.actor.film 4804

1251 film.actor.film 8744

3381 film.person_or_entity_appearing_in_film.film 7459

1460 music.artist.origin 8961

2378 award.nominee.award_nominations 5487

1157 film.actor.film 6198

3384 film.actor.film 4704

1257 music.artist.origin 4046

410 people.person.place_of_birth 8962

548 film.person_or_entity_appearing_in_film.film 8963

548 film.person_or_entity_appearing_in_film.film 6716

623 film.actor.film 6055

1044 award.competitor.award_nominations 7000

3714 film.actor.film 7926

1833 film.person_or_entity_appearing_in_film.film 8964

946 music.artist.origin 4852

2734 film.actor.film 4220

993 people.person.place_of_birth 4496

117 film.person_or_entity_appearing_in_film.film 8255

2359 film.person_or_entity_appearing_in_film.film 8020

1516 music.artist.origin 4057

808 film.actor.film 6160

2552 film.person_or_entity_appearing_in_film.film 4385

2603 film.actor.film 7490

136 film.person_or_entity_appearing_in_film.film 8867

145 film.person_or_entity_appearing_in_film.film 8667

604 film.actor.film 4382

3295 film.person_or_entity_appearing_in_film.film 8965

869 music.artist.origin 4050

213 music.artist.origin 4057

2637 film.film.actor 5214

3379 people.person.place_of_birth 3945

3028 music.artist.origin 4362

2636 film.actor.film 8966

2636 film.person_or_entity_appearing_in_film.film 8157

2674 film.person_or_entity_appearing_in_film.film 6217

1408 film.actor.film 6499

1355 film.actor.film 8967

1353 film.person_or_entity_appearing_in_film.film 8968

2460 award.nominee.award_nominations 8969

294 film.actor.film 8970

2719 music.artist.origin 7675

527 film.person_or_entity_appearing_in_film.film 8522

484 film.person_or_entity_appearing_in_film.film 4242

3738 cvg.publisher.games_published 4781

3424 people.person.place_of_birth 6990

3400 film.person_or_entity_appearing_in_film.film 5591

2241 film.distributor.film 8691

2241 film.distributor.film 5037

2241 film.distributor.film 5819

2241 film.distributor.film 6033

2241 film.production_company.film 6921

2241 film.production_company.film 7755

3350 film.actor.film 8971

1917 music.artist.origin 4057

1464 film.actor.film 8972

3334 music.artist.origin 6504

1656 film.actor.film 4037

2678 film.actor.film 5117

2206 film.person_or_entity_appearing_in_film.film 5607

3049 film.actor.film 8973

3742 film.director.film 4477

3579 film.person_or_entity_appearing_in_film.film 4156

1451 people.person.place_of_birth 6128

601 film.person_or_entity_appearing_in_film.film 4270

1057 film.actor.film 7556

997 film.person_or_entity_appearing_in_film.film 5652

2635 film.person_or_entity_appearing_in_film.film 8834

2635 film.person_or_entity_appearing_in_film.film 4333

3325 film.person_or_entity_appearing_in_film.film 4163

540 film.actor.film 8974

1006 award.competitor.award_nominations 6621

254 film.person_or_entity_appearing_in_film.film 8380

3827 film.actor.film 8704

815 film.actor.film 5117

3514 film.actor.film 8975

2708 film.person_or_entity_appearing_in_film.film 6626

3788 film.actor.film 8610

487 music.artist.origin 3864

1541 music.artist.origin 3864

1598 film.person_or_entity_appearing_in_film.film 4804

2338 film.actor.film 5113

3094 film.person_or_entity_appearing_in_film.film 8976

70 film.actor.film 8414

2710 film.actor.film 6537

1601 film.person_or_entity_appearing_in_film.film 8977

2096 film.person_or_entity_appearing_in_film.film 6407

2674 film.actor.film 8372

2070 film.actor.film 8978

445 film.person_or_entity_appearing_in_film.film 8979

389 award.competitor.award_nominations 8980

2460 award.competitor.award_nominations 8969

2460 film.actor.film 7704

278 film.actor.film 8915

3552 music.artist.origin 3961

484 film.actor.film 8981

3738 games.publisher.games_published 6334

184 film.actor.film 8982

1356 people.person.place_of_birth 4057

2241 film.distributor.film 8466

2241 film.production_company.film 6667

3398 music.musician.instruments_played 4349

1464 film.actor.film 7852

3572 music.artist.origin 4380

681 film.person_or_entity_appearing_in_film.film 4989

1618 film.person_or_entity_appearing_in_film.film 8884

2126 music.artist.origin 5916

1697 music.artist.origin 4815

2951 film.person_or_entity_appearing_in_film.film 3991

2643 film.person_or_entity_appearing_in_film.film 4795

3242 film.actor.film 8901

3259 music.artist.origin 6346

673 people.person.profession 4221

2207 film.actor.film 8919

3049 film.person_or_entity_appearing_in_film.film 7152

2543 music.artist.origin 4057

3557 film.film.star 8481

2955 music.artist.origin 7672

3390 film.actor.film 7297

3279 film.person_or_entity_appearing_in_film.film 6387

873 film.person_or_entity_appearing_in_film.film 5096

2469 music.artist.origin 4583

1100 people.person.place_of_birth 8956

3822 film.person_or_entity_appearing_in_film.film 8698

1358 film.actor.film 4675

175 tv.program.country_of_origin 5623

2652 music.artist.origin 3961

410 film.person_or_entity_appearing_in_film.film 4113

410 film.person_or_entity_appearing_in_film.film 5618

2161 film.person_or_entity_appearing_in_film.film 5485

1179 film.film.actor 4056

3582 people.person.place_of_birth 3932

1933 music.artist.origin 4050

1833 film.actor.film 5571

2953 film.person_or_entity_appearing_in_film.film 8573

3087 film.actor.film 7837

1209 film.person_or_entity_appearing_in_film.film 5398

3295 film.actor.film 8153

2080 film.person_or_entity_appearing_in_film.film 8983

2679 people.deceased_person.place_of_death 4057

2637 film.film.producer 5214

646 film.person_or_entity_appearing_in_film.film 6890

3120 film.person_or_entity_appearing_in_film.film 8984

1601 film.person_or_entity_appearing_in_film.film 6066

2981 music.artist.origin 3897

2117 music.artist.origin 7479

665 music.artist.origin 4232

1856 people.person.place_of_birth 7212

2460 film.person_or_entity_appearing_in_film.film 8418

2412 film.actor.film 8985

1912 music.artist.origin 4195

3738 cvg.publisher.games_published 4929

3738 games.publisher.games_published 8115

1249 film.actor.film 8270

1290 music.artist.origin 4901

2241 film.distributor.film 8986

2241 film.distributor.film 5039

2241 film.production_company.film 8298

2241 film.production_company.film 4402

1464 film.person_or_entity_appearing_in_film.film 8987

1455 music.artist.origin 4024

894 film.actor.film 8872

3198 film.actor.film 7292

1656 film.person_or_entity_appearing_in_film.film 8886

3327 music.artist.origin 8939

538 film.person_or_entity_appearing_in_film.film 8339

2387 film.actor.film 5720

2775 music.artist.origin 5623

843 people.deceased_person.place_of_death 4094

1816 film.person_or_entity_appearing_in_film.film 8726

2194 music.artist.origin 4380

3545 film.actor.film 4822

552 film.person_or_entity_appearing_in_film.film 8988

1157 film.person_or_entity_appearing_in_film.film 4906

712 film.actor.film 5689

1989 film.actor.film 8201

3827 film.person_or_entity_appearing_in_film.film 4709

1153 film.actor.film 8989

2269 people.person.place_of_birth 8588

2186 film.actor.film 4210

1473 film.person_or_entity_appearing_in_film.film 8990

3061 music.artist.origin 4347

895 film.person_or_entity_appearing_in_film.film 7307

2464 music.artist.origin 6564

1187 film.person_or_entity_appearing_in_film.film 4822

3356 music.artist.origin 4148

2069 film.actor.film 6166

377 film.person_or_entity_appearing_in_film.film 7868

1125 music.artist.origin 4050

1434 film.actor.film 6774

885 music.artist.origin 7661

357 film.actor.film 7313

945 film.person_or_entity_appearing_in_film.film 8656

1980 music.artist.origin 4357

1453 music.artist.album 8850

974 film.actor.film 8747

1423 film.actor.film 8991

437 film.actor.film 8992

2460 film.person_or_entity_appearing_in_film.film 5911

2661 film.person_or_entity_appearing_in_film.film 6143

436 film.person_or_entity_appearing_in_film.film 5253

1143 people.person.place_of_birth 8993

1011 film.actor.film 6785

1249 film.person_or_entity_appearing_in_film.film 4464

2867 film.person_or_entity_appearing_in_film.film 7793

2241 film.production_company.film 8559

2241 film.production_company.film 8994

924 music.artist.origin 4768

2798 people.person.place_of_birth 5523

3297 film.actor.film 4846

382 film.person_or_entity_appearing_in_film.film 6791

2189 film.person_or_entity_appearing_in_film.film 7651

2732 film.person_or_entity_appearing_in_film.film 4411

1656 film.actor.film 8995

3031 film.person_or_entity_appearing_in_film.film 8996

2200 people.person.place_of_birth 8997

1360 film.person_or_entity_appearing_in_film.film 6675

817 film.actor.film 4696

2765 music.artist.origin 4133

3312 film.actor.film 4385

1417 music.artist.origin 4057

3242 film.actor.film 6759

2415 people.deceased_person.place_of_death 8998

3321 film.person_or_entity_appearing_in_film.film 4152

3321 film.person_or_entity_appearing_in_film.film 8143

3095 film.actor.film 5416

3390 music.artist.album 8308

3648 film.actor.film 7023

3132 film.actor.film 7357

56 film.person_or_entity_appearing_in_film.film 6080

206 film.person_or_entity_appearing_in_film.film 7053

997 film.actor.film 8999

2402 film.person_or_entity_appearing_in_film.film 9000

1662 film.actor.film 5310

567 film.person_or_entity_appearing_in_film.film 9001

642 people.person.place_of_birth 6261

2362 film.actor.film 7636

3127 music.artist.origin 4707

2494 film.actor.film 9002

374 film.person_or_entity_appearing_in_film.film 9003

155 award.competitor.awards_won 9004

1916 music.artist.origin 3963

563 music.musician.instruments_played 3851

395 film.person_or_entity_appearing_in_film.film 7311

1162 music.artist.origin 4008

2147 film.person_or_entity_appearing_in_film.film 4489

2042 music.artist.origin 4701

1473 film.actor.film 9005

3733 film.person_or_entity_appearing_in_film.film 9006

3587 music.artist.origin 4454

2271 people.person.place_of_birth 5523

3109 film.person_or_entity_appearing_in_film.film 8943

2913 film.actor.film 9007

2913 film.actor.film 5157

2914 film.person_or_entity_appearing_in_film.film 6697

3799 film.person_or_entity_appearing_in_film.film 6573

1453 film.person_or_entity_appearing_in_film.film 7667

568 film.actor.film 8842

1450 people.person.place_of_birth 4013

2597 film.actor.film 8152

2993 music.artist.origin 7675

3289 film.actor.film 5749

1353 film.actor.film 8358

133 award.competitor.award_nominations 5499

2460 film.actor.film 6439

2412 film.person_or_entity_appearing_in_film.film 5458

790 film.person_or_entity_appearing_in_film.film 6796

484 film.actor.film 8475

3738 cvg.publisher.games_published 8463

1154 film.person_or_entity_appearing_in_film.film 4463

1530 music.artist.origin 4058

174 music.artist.origin 4896

2241 film.distributor.film 3863

2241 film.production_company.film 8119

1824 film.person_or_entity_appearing_in_film.film 9008

3430 film.actor.film 6274

1942 film.person_or_entity_appearing_in_film.film 7283

419 music.artist.origin 3932

777 film.person_or_entity_appearing_in_film.film 4252

2487 film.actor.film 9009

1855 people.person.place_of_birth 7498

2126 film.person_or_entity_appearing_in_film.film 8618

3486 film.actor.film 5017

2206 film.actor.film 8900

1798 people.person.place_of_birth 3992

371 film.actor.film 6849

1440 music.artist.origin 6215

247 music.artist.origin 6262

1357 film.person_or_entity_appearing_in_film.film 7451

434 film.actor.film 5514

292 people.person.profession 4221

538 film.actor.film 7732

1585 people.person.place_of_birth 5873

3321 film.actor.film 9010

2979 film.person_or_entity_appearing_in_film.film 9011

898 film.person_or_entity_appearing_in_film.film 4638

1468 film.person_or_entity_appearing_in_film.film 7453

2556 film.person_or_entity_appearing_in_film.film 5470

3390 film.person_or_entity_appearing_in_film.film 7122

638 film.person_or_entity_appearing_in_film.film 9012

2312 film.actor.film 8786

637 film.person_or_entity_appearing_in_film.film 5350

2928 music.artist.origin 4963

3488 music.artist.origin 4053

997 film.actor.film 8607

422 music.producer.tracks_produced 8840

1854 music.artist.origin 4010

1372 people.person.place_of_birth 4094

148 film.actor.film 6427

567 film.actor.film 8636

2808 film.person_or_entity_appearing_in_film.film 5754

3384 film.actor.film 7881

2161 film.actor.film 5485

2817 music.artist.origin 9013

492 film.actor.film 7274

3317 film.person_or_entity_appearing_in_film.film 6566

2811 people.person.place_of_birth 5009

2186 film.person_or_entity_appearing_in_film.film 4210

2620 film.person_or_entity_appearing_in_film.film 5549

2804 film.person_or_entity_appearing_in_film.film 8891

205 music.artist.origin 8961

2017 music.artist.origin 4057

713 music.artist.origin 4471

2157 music.artist.origin 3961

2454 film.producer.film 6974

645 film.person_or_entity_appearing_in_film.film 8337

1844 film.character.film 4987

508 award.nominee.award_nominations 6534

386 film.actor.film 9014

2913 film.person_or_entity_appearing_in_film.film 8459

1220 film.person_or_entity_appearing_in_film.film 9015

628 film.actor.film 3980

209 music.artist.origin 7558

1846 film.person_or_entity_appearing_in_film.film 7220

437 film.actor.film 7466

2636 film.actor.film 7720

1458 film.actor.film 7792

2225 music.artist.origin 4369

49 film.person_or_entity_appearing_in_film.film 7139

3738 games.publisher.games_published 9016

184 film.actor.film 9017

2400 film.person_or_entity_appearing_in_film.film 7416

625 film.actor.film 5813

2404 music.artist.origin 4807

2241 film.distributor.film 8960

2241 film.production_company.film 4132

2241 film.production_company.film 5817

2241 film.production_company.film 9018

2241 film.production_company.film 8675

2241 film.production_company.film 7350

2241 film.production_company.film 4889

3160 film.person_or_entity_appearing_in_film.film 8661

3297 film.actor.film 6418

1762 film.actor.film 9019

1762 film.person_or_entity_appearing_in_film.film 9020

758 music.artist.origin 4442

356 music.artist.track 6987

538 film.actor.film 9021

600 music.artist.album 4718

3049 film.actor.film 7204

3321 film.person_or_entity_appearing_in_film.film 4208

157 film.producer.film 4915

2051 people.deceased_person.place_of_death 4768

3648 film.person_or_entity_appearing_in_film.film 9022

2711 film.actor.film 8816

601 film.actor.film 5694

687 film.actor.film 9023

2298 film.person_or_entity_appearing_in_film.film 9024

297 music.artist.origin 4583

68 film.actor.film 4425

997 film.actor.film 8922

997 film.person_or_entity_appearing_in_film.film 8999

2635 film.actor.film 7685

2635 film.person_or_entity_appearing_in_film.film 8849

2789 film.person_or_entity_appearing_in_film.film 8830

857 music.artist.origin 4927

3174 people.deceased_person.place_of_death 3966

3827 film.person_or_entity_appearing_in_film.film 8130

1174 music.artist.origin 4332

1401	music.artist.origin	5064
830 music.artist.origin	4191
3263	location.location.time_zone	7937
2963	film.actor.film	9025
2359	film.actor.film	8220
2865	film.person_or_entity_appearing_in_film.film	5869
1742	music.artist.origin	5627
3364	people.person.place_of_birth	4919
2700	film.actor.film	8930
407 film.actor.film	9026
3344	film.person_or_entity_appearing_in_film.film	7113
1079	film.director.film 6139
3211	people.person.place_of_birth	3972
1928	people.person.place_of_birth	4183
70	film.person_or_entity_appearing_in_film.film	4715
408 film.actor.film	7842
1204	film.actor.film	9027
2636	film.person_or_entity_appearing_in_film.film	8079
147 film.actor.film	9028
3021	film.person_or_entity_appearing_in_film.film	3939
1211	film.person_or_entity_appearing_in_film.film	9029
2246	people.deceased_person.place_of_death	3864
2412	film.person_or_entity_appearing_in_film.film	8985
484 film.person_or_entity_appearing_in_film.film	8981
1154	film.actor.film	5543
2400	film.person_or_entity_appearing_in_film.film	8897
1038	music.artist.origin	4453
2360	film.actor.film	6258
2241	film.production_company.film	4192
2282	music.artist.origin	8703

419 film.person_or_entity_appearing_in_film.film 4915

3267 music.artist.origin 4768

3841 music.artist.origin 4453

3527 music.artist.origin 4362

1777 film.person_or_entity_appearing_in_film.film 9030

534 film.actor.film 3991

2731 film.actor.film 7426

2627 music.artist.origin 4050

1357 film.person_or_entity_appearing_in_film.film 7021

3242 film.person_or_entity_appearing_in_film.film 7387

2415 film.person_or_entity_appearing_in_film.film 8470

3231 film.actor.film 8014

898 film.actor.film 7266

1954 film.person_or_entity_appearing_in_film.film 5483

3742 film.person_or_entity_appearing_in_film.film 6858

2509 film.actor.film 5309

3648 film.person_or_entity_appearing_in_film.film 8862

3648 film.person_or_entity_appearing_in_film.film 6648

547 people.person.place_of_birth 9031

1348 film.director.film 8132

923 film.actor.film 9032

1866 film.actor.film 8634

2343 film.person_or_entity_appearing_in_film.film 7240

1142 film.person_or_entity_appearing_in_film.film 9033

900 film.person_or_entity_appearing_in_film.film 8260

2635 film.person_or_entity_appearing_in_film.film 6937

2789 film.actor.film 8830

1157 film.person_or_entity_appearing_in_film.film 6762

3386 location.location.contained_by 4676

2366 film.actor.film 6138

2161	film.person_or_entity_appearing_in_film.film	3957

2040	film.person_or_entity_appearing_in_film.film	4823

548 film.person_or_entity_appearing_in_film.film	6559

3594	film.actor.film	9034

2359	film.person_or_entity_appearing_in_film.film	8942

1473	film.actor.film	9035

2204	music.artist.origin	5213

2893	film.person_or_entity_appearing_in_film.film	7931

3146	people.person.place_of_birth	5228

987 film.person_or_entity_appearing_in_film.film	4719

1885	film.person_or_entity_appearing_in_film.film	8820

893 film.actor.film	9036

3250	people.person.profession	4221

3676	film.actor.film	4451

3676	people.person.place_of_birth	5125

3503	music.artist.origin	3916

1601	film.person_or_entity_appearing_in_film.film	9037

2437	film.actor.film	8396

2597	film.person_or_entity_appearing_in_film.film	8671

3652	music.artist.origin	4057

2070	film.person_or_entity_appearing_in_film.film	9038

3179	film.person_or_entity_appearing_in_film.film	7612

1370	film.film.director7012

771 film.actor.film	5667

270 film.person_or_entity_appearing_in_film.film	4978

2411	people.person.place_of_birth	4768

1249	film.actor.film	5333

2241	film.production_company.film	9039

2241	film.production_company.film	8465

1936	film.person_or_entity_appearing_in_film.film	8948

3276	people.deceased_person.place_of_death	4013

3031	film.person_or_entity_appearing_in_film.film	7679

2275	film.actor.film	4646

2200	music.musician.instruments_played	5802

3486	film.person_or_entity_appearing_in_film.film	5017

1360	film.person_or_entity_appearing_in_film.film	9040

418 music.artist.origin	4195

469 film.person_or_entity_appearing_in_film.film	9041

2677	film.actor.film	9042

1806	film.person_or_entity_appearing_in_film.film	5739

194 people.person.place_of_birth	4191

3546	film.actor.film	9043

3665	film.actor.film	8011

1357	film.actor.film	6350

538 film.actor.film	7984

232 music.artist.origin	4357

3650	film.actor.film	8419

3390	film.actor.film	6798

3648	film.actor.film	9022

99	music.artist.origin	4442

2305	music.artist.origin	6249

1479	film.person_or_entity_appearing_in_film.film	7763

1962	film.person_or_entity_appearing_in_film.film	7059

3055	music.artist.origin	4046

3039	music.artist.origin	4496

997 film.actor.film	7485

997 film.person_or_entity_appearing_in_film.film	8818

735 music.musician.instruments_played	5619

3599	film.actor.film	9044

2635	film.actor.film	6688

1157 film.person_or_entity_appearing_in_film.film 8199

379 award.competitor.award_nominations 8052

186 film.actor.film 7130

3617 music.artist.origin 7370

509 people.person.place_of_birth 6076

1153 film.person_or_entity_appearing_in_film.film 8572

312 music.artist.origin 5639

2953 film.person_or_entity_appearing_in_film.film 3962

1951 music.artist.origin 4108

1473 film.person_or_entity_appearing_in_film.film 7635

12 film.actor.film 9045

127 film.person_or_entity_appearing_in_film.film 7279

3733 film.person_or_entity_appearing_in_film.film 7216

539 film.person_or_entity_appearing_in_film.film 6595

3795 film.actor.film 6532

1352 people.person.place_of_birth 5246

347 music.artist.origin 4087

2081 music.artist.origin 5623

1744 film.person_or_entity_appearing_in_film.film 6465

1509 music.artist.origin 4552

3185 film.actor.film 7893

2688 music.musician.instruments_played 6260

2460 film.actor.film 7998

2208 film.person_or_entity_appearing_in_film.film 8565

3619 music.artist.origin 4391

2241 film.production_company.film 7942

2241 film.production_company.film 6817

2241 film.production_company.film 4398

2241 film.production_company.film 9046

795 film.person_or_entity_appearing_in_film.film 4846

1186 people.person.place_of_birth 5627

2501 film.person_or_entity_appearing_in_film.film 9047

1501 music.artist.origin 3916

1762 film.person_or_entity_appearing_in_film.film 8299

777 film.actor.film 5711

2487 film.actor.film 6233

1777 film.person_or_entity_appearing_in_film.film 7660

2759 music.artist.origin 3947

3486 film.actor.film 6988

2731 film.person_or_entity_appearing_in_film.film 4946

3546 film.actor.film 5748

3796 music.artist.origin 4077

1417 film.actor.film 6850

3538 music.artist.origin 3897

1332 film.actor.film 4265

2919 film.person_or_entity_appearing_in_film.film 4268

1070 music.artist.origin 4150

1955 film.person_or_entity_appearing_in_film.film 6875

1185 music.artist.origin 8483

2635 film.person_or_entity_appearing_in_film.film 7396

2635 film.person_or_entity_appearing_in_film.film 8276

2635 film.person_or_entity_appearing_in_film.film 6876

115 film.person_or_entity_appearing_in_film.film 6715

517 event.agent.performance 4898

3352 music.artist.origin 7753

1748 film.actor.film 4912

2939 film.person_or_entity_appearing_in_film.film 7001

426 music.artist.origin 4217

3788 film.actor.film 6806

674 film.person_or_entity_appearing_in_film.film 4958

67 film.actor.film 5378

67 film.person_or_entity_appearing_in_film.film 5245

136 film.person_or_entity_appearing_in_film.film 6493

2361 music.artist.origin 4195

1209 film.person_or_entity_appearing_in_film.film 4802

2080 film.actor.film 8642

74 film.person_or_entity_appearing_in_film.film 6775

2972 film.actor.film 6810

1797 music.artist.origin 4183

1601 film.person_or_entity_appearing_in_film.film 7892

2636 film.person_or_entity_appearing_in_film.film 8594

2437 film.person_or_entity_appearing_in_film.film 9048

1403 film.actor.film 4292

3663 film.person_or_entity_appearing_in_film.film 4406

3179 film.actor.film 8098

3166 film.person_or_entity_appearing_in_film.film 8474

1353 film.actor.film 5586

2460 film.person_or_entity_appearing_in_film.film 8672

3738 games.publisher.games_published 4187

3738 games.publisher.games_published 8544

2293 film.actor.film 7220

2360 film.actor.film 7257

2241 film.distributor.film 9049

2241 film.distributor.film 8935

2241 film.distributor.film 8180

2241 film.production_company.film 3929

2241 film.production_company.film 9050

2241 film.production_company.film 6339

2241 film.production_company.film 9051

2241 film.production_company.film 8178

681 film.person_or_entity_appearing_in_film.film 5710

1894 music.artist.album 9052

1997 music.artist.origin 6911

469 film.actor.film 9041

1357 film.person_or_entity_appearing_in_film.film 7231

218 music.artist.origin 4455

3321 film.person_or_entity_appearing_in_film.film 8214

1167 music.artist.origin 7554

221 music.artist.origin 4924

1922 music.artist.origin 4416

616 music.artist.origin 3872

1479 film.actor.film 8817

667 music.artist.origin 4422

1930 music.artist.origin 4851

3716 film.person_or_entity_appearing_in_film.film 6050

115 film.person_or_entity_appearing_in_film.film 7430

1351 film.actor.film 9053

931 music.artist.origin 4454

3778 music.artist.origin 4010

733 music.artist.origin 4009

1488 award.competitor.award_nominations 5153

1334 music.artist.origin 4175

3216 tv.program.genre 8133

407 film.person_or_entity_appearing_in_film.film 9026

84 film.actor.film 9054

3598 film.person_or_entity_appearing_in_film.film 8023

789 film.actor.film 9055

3344 film.actor.film 6915

3250 people.deceased_person.place_of_death 4057

2913 film.person_or_entity_appearing_in_film.film 7136

974 film.person_or_entity_appearing_in_film.film 6862

754 music.artist.origin 4057

211 music.artist.origin 4852

1154 film.actor.film 8944

901 film.person_or_entity_appearing_in_film.film 6665

625 film.person_or_entity_appearing_in_film.film 8720

266 music.artist.origin 4977

1940 people.person.place_of_birth 8592

2867 film.actor.film 7147

2747 music.artist.album 8605

592 film.person_or_entity_appearing_in_film.film 9056

2241 film.distributor.film 7018

2241 film.distributor.film 7445

481 film.actor.film 6961

2897 film.person_or_entity_appearing_in_film.film 8815

894 film.person_or_entity_appearing_in_film.film 9057

261 film.actor.film 5198

534 film.person_or_entity_appearing_in_film.film 7550

2810 film.actor.film 8234

2731 film.actor.film 8301

1806 film.actor.film 7946

3779 music.artist.origin 8351

3242 film.producer.film 7635

1695 film.person_or_entity_appearing_in_film.film 4309

1660 film.person_or_entity_appearing_in_film.film 8784

2387 film.actor.film 5785

1241 film.film.executive_producer 4153

3223 music.artist.origin 4010

3528 film.actor.film 7762

1941 event.agent.performance 5101

2833 film.person_or_entity_appearing_in_film.film 9058

2635 film.person_or_entity_appearing_in_film.film 7128

2808 film.person_or_entity_appearing_in_film.film 4462

115 film.actor.film 9059

818 film.actor.film 9060

1720 film.actor.film 4755

1581 music.artist.origin 7370

1505 film.actor.film 9061

1494 music.artist.origin 4362

1939 music.artist.origin 3984

2505 music.artist.origin 4583

2953 film.actor.film 7608

3624 film.actor.film 5312

850 music.artist.album 9062

3598 film.actor.film 8669

3598 film.person_or_entity_appearing_in_film.film 9063

2839 music.artist.origin 3986

2914 film.person_or_entity_appearing_in_film.film 7280

1329 people.person.place_of_birth 4294

510 film.person_or_entity_appearing_in_film.film 5766

2562 film.actor.film 5890

472 film.person_or_entity_appearing_in_film.film 4498

70 film.person_or_entity_appearing_in_film.film 3915

2636 film.person_or_entity_appearing_in_film.film 8670

2437 film.person_or_entity_appearing_in_film.film 9064

79 film.person_or_entity_appearing_in_film.film 8095

3738 cvg.publisher.games_published 9065

1154 film.person_or_entity_appearing_in_film.film 9066

2414 award.competitor.award_nominations 8896

1749 music.artist.origin 4170

901 award.winner.awards_won 4783

901 film.actor.film 7442

1359 film.person_or_entity_appearing_in_film.film 9067

2867 film.person_or_entity_appearing_in_film.film 6414

1463 award.competitor.awards_won 6845

2920 music.artist.origin 3946

2241 film.distributor.film 8799

2241 film.production_company.film 9068

2241 film.production_company.film 6181

2241 film.production_company.film 9069

2241 film.production_company.film 6846

337 music.artist.origin 3859

3350 film.person_or_entity_appearing_in_film.film 9070

3297 film.actor.film 4281

894 film.person_or_entity_appearing_in_film.film 7758

1583 music.artist.origin 8078

261 music.producer.tracks_produced 6316

2200 music.artist.origin 8997

3657 music.artist.origin 4217

3000 film.actor.film 9071

3049 film.actor.film 7478

1241 film.film.producer 4153

2542 music.artist.origin 4024

3742 film.actor.film 6858

1387 film.person_or_entity_appearing_in_film.film 9072

1612 music.artist.origin 4057

2658 film.actor.film 7207

3639 music.artist.origin 6201

1454 music.album.genre 6212

997 film.actor.film 8865

1662	film.actor.film	8071

1905	music.artist.origin	4024

3307	film.person_or_entity_appearing_in_film.film	8165

1157	film.actor.film	6122

623 film.person_or_entity_appearing_in_film.film	4274

3827	film.actor.film	5880

3778	music.musician.instruments_played	5367

774 film.person_or_entity_appearing_in_film.film	9073

1473	film.actor.film	9074

1473	film.person_or_entity_appearing_in_film.film	6378

3529	music.musician.instruments_played	5619

3741	film.actor.film	7342

239 film.person_or_entity_appearing_in_film.film	5162

1209	film.person_or_entity_appearing_in_film.film	7462

1184	music.artist.origin	4456

407 film.actor.film	7188

3598	film.person_or_entity_appearing_in_film.film	9075

3004	people.person.place_of_birth	4077

3121	film.actor.film	6427

1979	music.artist.origin	3944

386 film.person_or_entity_appearing_in_film.film	9014

2637	film.film.editor	6776

1208	music.musician.instruments_played	6942

1447	music.artist.origin	6760

2562	film.person_or_entity_appearing_in_film.film	8208

128 type.object.key	128

2535	people.person.place_of_birth	9076

466 film.actor.film	6409

2208	film.actor.film	5821

3738	games.publisher.games_published 6546

3738 games.publisher.games_published 8645

1363 film.person_or_entity_appearing_in_film.film 8191

1694 people.person.place_of_birth 4563

3760 music.artist.origin 7646

1249 film.actor.film 5332

2159 film.person_or_entity_appearing_in_film.film 8464

196 music.artist.origin 4707

2403 music.artist.origin 7477

2241 film.distributor.film 5183

2241 film.distributor.film 5135

2241 film.distributor.film 9018

2241 film.distributor.film 7046

2241 film.distributor.film 7917

2241 film.production_company.film 8420

462 film.person_or_entity_appearing_in_film.film 7454

3761 music.artist.origin 9077

1624 film.actor.film 5822

2487 film.actor.film 8899

591 film.actor.film 4281

261 film.person_or_entity_appearing_in_film.film 6792

406 film.actor.film 4706

1059 people.person.place_of_birth 4272

1874 music.artist.origin 7166

2493 film.actor.film 4697

3045 film.person_or_entity_appearing_in_film.film 8913

1660 film.actor.film 9078

3321 film.person_or_entity_appearing_in_film.film 7053

2279 film.actor.film 5391

1623 film.actor.film 6013

3269 music.artist.origin 6262

638 film.person_or_entity_appearing_in_film.film 8047

354 film.actor.film 5815

3727 film.person_or_entity_appearing_in_film.film 9079

1962 film.actor.film 9080

1075 music.artist.origin 4013

2402 film.actor.film 4366

131 people.person.place_of_birth 5828

2728 music.artist.origin 4391

984 music.musician.instruments_played 5558

379 film.person_or_entity_appearing_in_film.film 5593

1633 film.actor.film 6486

2381 people.person.place_of_birth 4714

2875 film.actor.film 8890

2422 film.actor.film 6086

2734 film.person_or_entity_appearing_in_film.film 6741

3548 film.actor.film 7767

3548 film.actor.film 4802

2398 event.agent.performance 5732

3733 film.person_or_entity_appearing_in_film.film 5684

3598 film.actor.film 6133

2396 music.artist.origin 5270

1559 film.person_or_entity_appearing_in_film.film 5694

3036 music.artist.origin 4024

2131 film.person_or_entity_appearing_in_film.film 4411

1355 film.person_or_entity_appearing_in_film.film 9081

366 people.person.place_of_birth 5070

3177 people.person.place_of_birth 4321

466 film.actor.film 8062

3738 cvg.publisher.games_published 7544

3738 cvg.publisher.games_published 7593

3738 games.publisher.games_published 8595

3738 games.publisher.games_published 8779

3738 games.publisher.games_published 4392

3738 games.publisher.games_published 8719

1603 film.actor.film 7723

554 people.person.profession 4990

2335 film.person_or_entity_appearing_in_film.film 8545

2867 film.actor.film 8097

2241 film.distributor.film 9068

2241 film.distributor.film 8858

2241 film.distributor.film 9082

2241 film.production_company.film 9083

663 music.artist.origin 4391

3751 people.person.place_of_birth 4003

537 tv.program.genre 4062

1462 film.actor.film 7523

406 film.person_or_entity_appearing_in_film.film 4706

2643 music.artist.origin 4191

2184 film.person_or_entity_appearing_in_film.film 3957

2162 music.artist.origin 4199

3002 film.person_or_entity_appearing_in_film.film 6074

538 film.person_or_entity_appearing_in_film.film 9084

2870 film.actor.film 5142

3321 film.actor.film 8806

3357 music.artist.origin 5569

1310 film.actor.film 9085

88 music.artist.origin 4454

2711 film.actor.film 4803

1816 film.person_or_entity_appearing_in_film.film 7268

1816 film.person_or_entity_appearing_in_film.film 8198

1814 film.film.actor 6905

687 film.person_or_entity_appearing_in_film.film 9086

2238 music.artist.origin 4362

142 people.person.place_of_birth 4013

2635 film.actor.film 7108

44 film.actor.film 9087

1157 film.actor.film 6738

540 film.person_or_entity_appearing_in_film.film 8974

2536 people.person.place_of_birth 4232

662 film.person_or_entity_appearing_in_film.film 7654

3020 film.actor.film 7607

1833 film.actor.film 9088

878 event.agent.performance 9089

774 film.actor.film 4538

3792 people.person.profession 8928

895 film.actor.film 8370

279 film.person_or_entity_appearing_in_film.film 4418

1625 film.person_or_entity_appearing_in_film.film 3912

645 film.actor.film 8556

1594 music.artist.origin 4195

893 film.actor.film 6745

2497 people.person.place_of_birth 4217

809 film.actor.film 9090

675 film.actor.film 4168

675 film.person_or_entity_appearing_in_film.film 7587

3799 film.person_or_entity_appearing_in_film.film 8493

70 film.actor.film 8706

2095 film.actor.film 5071

3021 people.person.place_of_birth 9091

2597 film.person_or_entity_appearing_in_film.film 8152

1139 music.artist.origin 3864

2968 film.actor.film 7467

49 film.person_or_entity_appearing_in_film.film 9092

1647 music.artist.origin 4852

415 music.artist.origin 4347

344 music.artist.origin 3859

484 film.person_or_entity_appearing_in_film.film 8546

630 film.actor.film 9093

3499 music.artist.origin 4376

2159 film.actor.film 5979

2867 film.actor.film 5381

1463 award.competitor.award_nominations 9094

2001 music.artist.origin 4000

2241 film.distributor.film 5298

2241 film.distributor.film 9046

2241 film.production_company.film 5860

2241 film.production_company.film 6380

2241 film.production_company.film 6787

2241 type.object.subject_key 2241

599 film.director.film 8467

777 film.actor.film 9095

777 film.person_or_entity_appearing_in_film.film 7819

2487 film.person_or_entity_appearing_in_film.film 5595

2126 film.actor.film 6848

261 music.artist.track 5641

3481 music.artist.origin 3916

3031 film.actor.film 8630

3031 music.artist.album 8402

3486 film.person_or_entity_appearing_in_film.film 6784

1360 film.actor.film 8860

643 film.actor.film 6005

3546 film.actor.film 8740

3546 film.person_or_entity_appearing_in_film.film 8513

1357 film.person_or_entity_appearing_in_film.film 7078

629 film.director.film 5909

835 music.artist.origin 4768

1643 music.artist.origin 3946

3279 film.actor.film 9096

1285 people.person.place_of_birth 6019

2711 film.actor.film 8348

3727 film.actor.film 9079

687 film.actor.film 9086

1287 music.artist.origin 8752

68 film.person_or_entity_appearing_in_film.film 7140

206 film.actor.film 9097

2076 music.artist.origin 5945

2904 film.actor.film 7380

1670 music.artist.origin 7683

375 people.person.place_of_birth 7151

3460 film.actor.film 5042

1633 film.actor.film 5727

1179 film.film.language 9098

1720 film.writer.film 9099

191 film.person_or_entity_appearing_in_film.film 9100

623 film.actor.film 4483

2953 film.person_or_entity_appearing_in_film.film 4968

67 film.person_or_entity_appearing_in_film.film 6433

1383 film.person_or_entity_appearing_in_film.film 8700

1473 film.actor.film 4864

2619 film.actor.film 5193

2337 film.person_or_entity_appearing_in_film.film 5759

2832 people.person.place_of_birth 5421

3364 film.person_or_entity_appearing_in_film.film 7278

1722 music.artist.origin 4454

3733 film.actor.film 9006

3626 film.actor.film 6037

2762 film.person_or_entity_appearing_in_film.film 4237

1214 music.artist.origin 4272

1616 film.actor.film 4477

1592 music.artist.origin 4362

1039 film.actor.film 6837

1601 film.actor.film 7222

2636 film.actor.film 5992

1736 music.artist.origin 4191

3821 film.person_or_entity_appearing_in_film.film 4389

2070 film.actor.film 7377

2070 film.person_or_entity_appearing_in_film.film 9101

1835 music.artist.origin 4369

781 film.actor.film 8890

373 film.person_or_entity_appearing_in_film.film 5993

484 people.deceased_person.place_of_death 5161

3738 cvg.publisher.games_published 6640

3738 games.publisher.games_published 8658

3673 music.artist.track 4981

1249 film.person_or_entity_appearing_in_film.film 6503

1639 music.artist.origin 4502

2241 film.distributor.film 4091

2241 film.distributor.film 7421

2241 film.production_company.film 7619

2456 music.artist.origin 4057

2793 music.artist.origin 7681

681 film.person_or_entity_appearing_in_film.film 7756

1624 film.person_or_entity_appearing_in_film.film 9102

2311 film.actor.film 8829

2895 music.artist.album 9103

1126 music.artist.origin 3971

1806 film.actor.film 6611

3331 film.actor.film 4475

1731 music.artist.origin 7361

3321 film.person_or_entity_appearing_in_film.film 5610

1105 film.person_or_entity_appearing_in_film.film 6586

2279 film.person_or_entity_appearing_in_film.film 8583

1623 film.actor.film 5233

2715 film.actor.film 4950

2373 film.actor.film 7529

380 film.person_or_entity_appearing_in_film.film 8365

241 film.actor.film 8606

2023 music.artist.origin 4087

2010 film.actor.film 6390

1233 people.person.place_of_birth 3972

3508 film.actor.film 9104

2595 film.actor.film 9105

959 film.person_or_entity_appearing_in_film.film 6370

396 people.person.place_of_birth 8961

2321 music.artist.origin 5421

3381 film.actor.film 9106

2635 film.person_or_entity_appearing_in_film.film 7972

3241 music.artist.origin 3966

1557 film.person_or_entity_appearing_in_film.film 5562

3384 film.actor.film 9107

1719 film.person_or_entity_appearing_in_film.film 6291

2515 film.actor.film 4385

374 film.person_or_entity_appearing_in_film.film 9108

3827 film.person_or_entity_appearing_in_film.film 8108

3418 music.artist.origin 5980

3778 film.person_or_entity_appearing_in_film.film 6322

1833 film.actor.film 8292

2216 music.producer.tracks_produced 4275

150 film.person_or_entity_appearing_in_film.film 7193

150 film.person_or_entity_appearing_in_film.film 4547

1983 music.artist.origin 7944

604 film.person_or_entity_appearing_in_film.film 6630

2080 film.person_or_entity_appearing_in_film.film 7933

3598 film.person_or_entity_appearing_in_film.film 5483

1079 film.director.film 7585

948 film.person_or_entity_appearing_in_film.film 9109

3498 film.actor.film 3977

74 film.actor.film 8411

74 film.actor.film 6157

55 music.artist.origin 4552

621 music.artist.origin 4232

631 people.person.place_of_birth 6200

1559 music.artist.origin 4057

2636 film.actor.film 4051

2357 music.artist.origin 6374

2982 music.artist.origin 6832

2674 type.object.subject_key 2674

1458 film.person_or_entity_appearing_in_film.film 5585

1370 film.film.writer 9110

3217 music.artist.origin 4552

484 film.actor.film 6222

3738 cvg.publisher.games_published 5896

3738 cvg.publisher.games_published 8437

901 film.actor.film 9111

1359 film.actor.film 9112

442 music.artist.album 6660

2241 film.distributor.film 9113

2241 film.distributor.film 9114

2241 film.distributor.film 8758

11 music.artist.origin 4183

135 film.person_or_entity_appearing_in_film.film 5936

1762 film.actor.film 4302

2066 music.artist.origin 9115

2810 type.object.subject_key 2810

1491 music.artist.origin 4422

3235 film.actor.film 4993

1772 film.actor.film 4203

473 people.deceased_person.place_of_death 4362

1526 film.person_or_entity_appearing_in_film.film 4710

2870 film.person_or_entity_appearing_in_film.film 6731

3403 film.actor.film 7205

1825 music.musician.instruments_played 3959

2711 film.actor.film 8725

2711 film.actor.film 4423

3132 film.actor.film 7862

3132 film.actor.film 5095

543 music.artist.origin 4454

1962 film.person_or_entity_appearing_in_film.film 9080

363 film.person_or_entity_appearing_in_film.film 9116

997 film.person_or_entity_appearing_in_film.film 9117

735 event.agent.performance 4770

2161 award.competitor.award_nominations 9118

2473 film.actor.film 8431

822 music.artist.origin 4171

1617 music.artist.album 8648

53 music.artist.origin 8889

1153 film.person_or_entity_appearing_in_film.film 9119

67 film.actor.film 5279

67 film.person_or_entity_appearing_in_film.film 7059

1473 film.person_or_entity_appearing_in_film.film 8489

1982 film.person_or_entity_appearing_in_film.film 3905

3733 film.actor.film 5684

1035 film.person_or_entity_appearing_in_film.film 7610

619 film.person_or_entity_appearing_in_film.film 7538

52 film.person_or_entity_appearing_in_film.film 5534

809 film.person_or_entity_appearing_in_film.film 7883

672 film.actor.film 8313

1384 music.artist.album 8876

1435 film.person_or_entity_appearing_in_film.film 6835

458 film.person_or_entity_appearing_in_film.film 7115

3561 film.person_or_entity_appearing_in_film.film 8460

3021 film.person_or_entity_appearing_in_film.film 6838

2674 film.person_or_entity_appearing_in_film.film 8332

2070 film.person_or_entity_appearing_in_film.film 6903

49 film.person_or_entity_appearing_in_film.film 7567

1981 film.person_or_entity_appearing_in_film.film 6440

3738 cvg.publisher.games_published 5462

3738 games.publisher.games_published 9120

3738 games.publisher.games_published 6580

3104 music.artist.origin 4272

2241	film.distributor.film	7352

2241	film.distributor.film	8179

2241	film.distributor.film	6922

2241	film.production_company.film	8797

2241	film.production_company.film	8495

2241	film.production_company.film	7419

682 music.artist.album	6659

135 film.person_or_entity_appearing_in_film.film	5406

3789	film.person_or_entity_appearing_in_film.film	7798

194 film.actor.film	5476

1475	film.actor.film	7524

1059	film.actor.film	4474

784 people.person.place_of_birth	4357

434 film.person_or_entity_appearing_in_film.film	5477

3002	film.person_or_entity_appearing_in_film.film	6244

1536	music.musician.instruments_played	4349

3321	film.person_or_entity_appearing_in_film.film	5516

372 film.person_or_entity_appearing_in_film.film	6680

2630	film.actor.film	6470

3508	film.person_or_entity_appearing_in_film.film	3931

2595	people.person.place_of_birth	5243

465 film.actor.film	8864

1750	film.person_or_entity_appearing_in_film.film	7105

2684	music.artist.album	5074

206 film.actor.film	8127

1157	film.editor.film	4052

3622	music.album.genre	8281

3783	music.artist.origin	3963

818 people.deceased_person.place_of_death	4471

2792	people.person.place_of_birth	4057

1466 music.artist.origin 4174

548 film.person_or_entity_appearing_in_film.film 7741

2097 film.actor.film 9121

3035 film.actor.film 4540

1153 film.person_or_entity_appearing_in_film.film 8907

3020 film.person_or_entity_appearing_in_film.film 6460

89 film.person_or_entity_appearing_in_film.film 8369

3484 music.artist.origin 3864

595 film.person_or_entity_appearing_in_film.film 8099

1960 film.actor.film 6438

3675 music.artist.origin 8718

977 film.person_or_entity_appearing_in_film.film 7508

3784 film.actor.film 3904

138 music.artist.origin 7624

761 music.artist.origin 4442

2253 music.artist.origin 4057

3300 music.artist.origin 4957

1711 film.person_or_entity_appearing_in_film.film 8091

3757 film.actor.film 5108

3598 film.actor.film 9122

3598 film.person_or_entity_appearing_in_film.film 9123

2035 film.actor.film 7064

1744 film.actor.film 6471

2562 film.writer.film 7251

70 film.actor.film 4399

2636 film.actor.film 4236

2981 film.person_or_entity_appearing_in_film.film 9124

208 music.artist.album 9125

79 film.actor.film 9017

2000 people.person.place_of_birth 8946

3217 film.person_or_entity_appearing_in_film.film 6350

782 film.person_or_entity_appearing_in_film.film 6750

436 film.actor.film 7243

96 film.actor.film 3848

877 music.artist.origin 4008

2241 film.production_company.film 6338

2241 film.production_company.film 6513

1464 film.actor.film 4147

3297 film.actor.film 5087

135 film.person_or_entity_appearing_in_film.film 4468

1780 film.person_or_entity_appearing_in_film.film 8162

3406 music.artist.origin 4010

2126 film.person_or_entity_appearing_in_film.film 9126

356 music.producer.tracks_produced 5604

1583 people.person.profession 4221

3031 film.person_or_entity_appearing_in_film.film 9127

2958 music.artist.origin 7422

643 film.person_or_entity_appearing_in_film.film 4518

469 film.person_or_entity_appearing_in_film.film 9128

2395 people.person.place_of_birth 5756

2861 film.actor.film 8469

2531 type.object.subject_key 2531

43 music.artist.origin 4272

2711 film.actor.film 9129

3048 film.actor.film 7761

3048 film.person_or_entity_appearing_in_film.film 7374

1428 games.game.publisher 8258

1534 people.person.place_of_birth 4167

1627 film.person_or_entity_appearing_in_film.film 7120

102 music.album.track 5829

3381 film.person_or_entity_appearing_in_film.film 9130

2635 film.actor.film 6082

2635 film.person_or_entity_appearing_in_film.film 9131

2494 film.person_or_entity_appearing_in_film.film 9002

2735 music.artist.origin 7194

785 film.person_or_entity_appearing_in_film.film 4055

3827 film.person_or_entity_appearing_in_film.film 9132

492 film.actor.film 4011

1153 film.actor.film 8926

1153 film.person_or_entity_appearing_in_film.film 8989

3395 film.actor.film 6297

2786 film.person_or_entity_appearing_in_film.film 5406

3020 film.person_or_entity_appearing_in_film.film 9133

2804 film.actor.film 9134

1473 film.person_or_entity_appearing_in_film.film 9135

1960 film.person_or_entity_appearing_in_film.film 6438

3611 film.character.film 8375

3295 film.person_or_entity_appearing_in_film.film 8792

3396 film.person_or_entity_appearing_in_film.film 5526

893 film.actor.film 9136

536 music.artist.origin 9137

1758 music.artist.origin 5265

3109 film.actor.film 8856

856 music.artist.origin 6405

2562 film.actor.film 7251

2854 music.artist.origin 9115

2289 film.person_or_entity_appearing_in_film.film 4494

128 film.actor.film 6538

2636 film.actor.film 7846

2743 film.actor.film 7565

137 award.competitor.award_nominations 8507
2483 film.person_or_entity_appearing_in_film.film 6579
2430 film.person_or_entity_appearing_in_film.film 4296
9 music.artist.origin 4456
2241 film.distributor.film 9078
2241 film.production_company.film 5223
2241 film.production_company.film 9003
1464 film.person_or_entity_appearing_in_film.film 7599
3297 film.person_or_entity_appearing_in_film.film 9138
1614 film.person_or_entity_appearing_in_film.film 5821
1762 film.actor.film 9020
1624 film.actor.film 4790
3354 film.actor.film 5485
3669 film.actor.film 4945
1806 film.actor.film 8239
1278 award.competitor.award_nominations 7450
2531 film.person_or_entity_appearing_in_film.film 9139
652 film.person_or_entity_appearing_in_film.film 7626
59 people.deceased_person.place_of_death 3846
157 music.artist.track 5647
1310 film.person_or_entity_appearing_in_film.film 9085
898 film.person_or_entity_appearing_in_film.film 7100
3557 film.film.language 9140
1105 film.actor.film 5049
1468 people.person.place_of_birth 4768
1623 film.person_or_entity_appearing_in_film.film 7120
3752 people.person.place_of_birth 5161
892 film.actor.film 7331
3390 film.person_or_entity_appearing_in_film.film 4588
3803 music.artist.origin 4057

3434	film.actor.film	9141

2419	film.actor.film	5485

2635	film.person_or_entity_appearing_in_film.film	6563

225 music.artist.origin	4496

115 film.actor.film	6651

115 film.actor.film	7695

410 film.person_or_entity_appearing_in_film.film	6879

548 film.actor.film	7908

3502	people.person.place_of_birth	5243

1044	award.competitor.award_nominations	6828

1044	film.writer.film	9142

815 film.person_or_entity_appearing_in_film.film	5794

815 film.person_or_entity_appearing_in_film.film	9143

1891	film.person_or_entity_appearing_in_film.film	6038

2954	music.artist.origin	6878

2963	film.person_or_entity_appearing_in_film.film	8790

2683	people.deceased_person.place_of_death	5236

117 film.actor.film	8255

1972	music.artist.origin	4455

2125	film.person_or_entity_appearing_in_film.film	5730

3792	film.person_or_entity_appearing_in_film.film	3965

1473	film.person_or_entity_appearing_in_film.film	3902

3445	music.album.genre	6992

839 people.person.place_of_birth	4269

2998	film.person_or_entity_appearing_in_film.film	8090

1706	film.actor.film	6872

3725	film.actor.film	4802

3067	music.artist.origin	4057

1209	film.actor.film	4802

1845	film.person_or_entity_appearing_in_film.film	9144

604 people.person.place_of_birth 4816

3598 film.person_or_entity_appearing_in_film.film 9122

2374 people.person.place_of_birth 4347

3498 film.actor.film 9145

2913 film.person_or_entity_appearing_in_film.film 9007

1601 film.actor.film 7914

437 film.actor.film 5115

2636 film.person_or_entity_appearing_in_film.film 7870

2981 film.actor.film 9124

86 people.person.place_of_birth 3855

2943 people.person.place_of_birth 6553

48 music.artist.origin 3946

79 film.actor.film 6605

890 music.artist.origin 6249

2877 film.actor.film 5110

3738 games.publisher.games_published 5541

436 film.person_or_entity_appearing_in_film.film 4782

2241 film.distributor.film 6790

3350 film.actor.film 4911

1464 film.actor.film 9146

1676 people.person.place_of_birth 4357

3297 film.actor.film 6277

1327 music.album.genre 4231

1618 film.actor.film 9147

935 music.artist.origin 8718

1927 people.person.place_of_birth 5685

929 music.artist.origin 3897

3235 event.agent.performance 4898

3549 people.person.place_of_birth 5238

2184 film.actor.film 3957

3321 film.person_or_entity_appearing_in_film.film 8480

2278 people.person.place_of_birth 4150

3550 people.person.place_of_birth 5403

3290 music.artist.origin 4214

594 film.actor.film 6424

876 music.artist.origin 9115

3687 music.artist.origin 7212

2556 music.artist.origin 4050

3279 film.person_or_entity_appearing_in_film.film 9148

2658 film.actor.film 9149

3132 film.person_or_entity_appearing_in_film.film 9150

306 music.artist.album 8404

2669 music.artist.origin 4010

1750 film.actor.film 8087

206 film.person_or_entity_appearing_in_film.film 9097

2827 music.album.genre 7828

1157 film.actor.film 9151

3630 music.artist.origin 3897

1006 award.competitor.award_nominations 8406

623 film.person_or_entity_appearing_in_film.film 9152

3324 people.person.place_of_birth 8866

627 film.person_or_entity_appearing_in_film.film 6109

1153 film.actor.film 7506

1525 film.actor.film 7787

2683 people.person.place_of_birth 4521

67 film.actor.film 9153

2147 film.actor.film 6882

793 film.person_or_entity_appearing_in_film.film 6865

1506 film.actor.film 7159

2337 film.director.film 6771

2983 film.actor.film 9154

453 film.person_or_entity_appearing_in_film.film 4730

3158 music.artist.origin 5230

3498 film.actor.film 4230

619 film.actor.film 7538

2914 film.actor.film 7935

357 film.person_or_entity_appearing_in_film.film 6792

2562 film.producer.film 7251

2289 film.actor.film 4494

3310 music.artist.origin 4362

2636 film.actor.film 8841

1390 music.artist.origin 4455

2070 film.person_or_entity_appearing_in_film.film 8856

3776 music.musician.instruments_played 6308

1299 film.person_or_entity_appearing_in_film.film 6542

2460 film.person_or_entity_appearing_in_film.film 8210

2460 people.person.place_of_birth 5070

1308 music.artist.origin 3864

3634 people.person.place_of_birth 4050

1307 music.artist.origin 4050

484 film.actor.film 9155

3738 games.publisher.games_published 5540

436 film.person_or_entity_appearing_in_film.film 7959

2430 music.artist.origin 3972

796 film.actor.film 9156

1249 film.actor.film 7595

3465 music.artist.origin 3961

2159 film.actor.film 9157

2241 film.distributor.film 7705

2241 film.distributor.film 8373

2241 film.distributor.film 9158

2241 film.production_company.film 5467

2241 film.production_company.film 8029

3430 film.person_or_entity_appearing_in_film.film 9159

1464 film.actor.film 8509

3297 film.person_or_entity_appearing_in_film.film 5339

356 film.person_or_entity_appearing_in_film.film 9160

1047 music.artist.origin 6555

1159 music.artist.origin 4852

1059 film.person_or_entity_appearing_in_film.film 8197

951 people.person.place_of_birth 4977

2054 film.actor.film 9161

1861 music.artist.origin 4807

3650 film.person_or_entity_appearing_in_film.film 7716

2373 music.artist.origin 3932

1323 film.actor.film 4525

3279 film.actor.film 8529

1231 people.person.place_of_birth 9162

3508 film.person_or_entity_appearing_in_film.film 9104

44 film.person_or_entity_appearing_in_film.film 9087

1157 film.person_or_entity_appearing_in_film.film 7803

3384 film.person_or_entity_appearing_in_film.film 8072

2362 film.actor.film 4823

2040 film.actor.film 4823

548 film.actor.film 7949

2097 film.person_or_entity_appearing_in_film.film 9121

1044 award.competitor.award_nominations 9163

2591 music.artist.origin 4010

3594 film.person_or_entity_appearing_in_film.film 9034

94 film.person_or_entity_appearing_in_film.film 7929

1748 film.person_or_entity_appearing_in_film.film 6456

2359 film.actor.film 4136

2017 film.actor.film 5766

3792 film.actor.film 5622

2337 film.actor.film 4437

1692 film.person_or_entity_appearing_in_film.film 8409

3204 film.person_or_entity_appearing_in_film.film 9164

413 film.actor.film 8688

279 film.person_or_entity_appearing_in_film.film 6347

645 film.person_or_entity_appearing_in_film.film 6772

3540 music.artist.origin 3897

386 film.actor.film 5067

510 film.actor.film 5766

1601 film.actor.film 8612

187 event.agent.performance 9089

1648 film.writer.film 5157

1211 film.actor.film 9165

2196 music.artist.origin 8586

3349 film.actor.film 4561

484 film.actor.film 8195

901 film.person_or_entity_appearing_in_film.film 4682

592 film.actor.film 9056

1463 award.competitor.award_nominations 5637

2241 film.distributor.film 8478

2241 film.production_company.film 8947

2241 film.production_company.film 6982

1343 music.artist.origin 3864

777 film.actor.film 6184

498 film.actor.film 4254

2126 film.actor.film 6110

1767 film.actor.film 9166

3031 film.actor.film 7118

3031 film.person_or_entity_appearing_in_film.film 9167

3486 film.actor.film 4580

1806 film.actor.film 5739

3487 music.musician.instruments_played 5802

2766 film.actor.film 9168

1977 music.artist.origin 6505

1884 film.actor.film 7390

3321 film.person_or_entity_appearing_in_film.film 6246

1623 film.person_or_entity_appearing_in_film.film 6796

2658 film.person_or_entity_appearing_in_film.film 5677

2010 film.person_or_entity_appearing_in_film.film 7826

3544 film.person_or_entity_appearing_in_film.film 4951

1689 music.artist.origin 4174

735 film.person_or_entity_appearing_in_film.film 5532

227 music.artist.origin 5656

2494 film.actor.film 6160

1439 award.competitor.award_nominations 8018

1313 music.artist.origin 4013

492 film.actor.film 4061

3569 film.person_or_entity_appearing_in_film.film 6296

3514 film.actor.film 9169

1153 film.person_or_entity_appearing_in_film.film 9170

1175 music.artist.origin 6948

2619 film.actor.film 5758

189 film.person_or_entity_appearing_in_film.film 9171

895 film.actor.film 8563

3364 film.actor.film 9172

1960 people.deceased_person.place_of_death 4057

3784 film.director.film 4716

3725 film.person_or_entity_appearing_in_film.film 5485

3169 music.artist.origin 6137

66 film.person_or_entity_appearing_in_film.film 8448

1754 music.artist.origin 4154

3344 film.actor.film 9173

3498 film.actor.film 7039

2830 film.person_or_entity_appearing_in_film.film 4168

408 people.person.place_of_birth 5125

1091 film.actor.film 7167

2437 film.person_or_entity_appearing_in_film.film 9174

2437 film.person_or_entity_appearing_in_film.film 7375

1830 music.artist.origin 4000

2070 film.actor.film 6265

133 award.competitor.awards_won 7613

146 people.deceased_person.place_of_death 4013

3738 games.publisher.games_published 8543

1277 film.actor.film 5812

625 film.actor.film 4683

3365 music.artist.origin 7187

355 music.artist.origin 6839

2241 film.distributor.film 4132

2241 film.distributor.film 8031

2241 film.production_company.film 6272

2241 film.production_company.film 9175

2241 film.production_company.film 8030

3297 film.person_or_entity_appearing_in_film.film 4382

135 film.actor.film 7919

356 music.producer.tracks_produced 9176

1767 film.actor.film 7688

956 music.artist.origin 4183

387 film.person_or_entity_appearing_in_film.film 5479

534 music.artist.album 9177

865 film.actor.film 5990

2568 music.artist.origin 3961

3749 people.person.place_of_birth 4003

3487 music.musician.instruments_played 4349

1357 film.actor.film 9178

2861 film.person_or_entity_appearing_in_film.film 4745

1926 film.person_or_entity_appearing_in_film.film 9179

2387 film.person_or_entity_appearing_in_film.film 9180

3180 music.musician.instruments_played 3959

2634 film.person_or_entity_appearing_in_film.film 6519

3650 film.actor.film 7716

3390 film.person_or_entity_appearing_in_film.film 7913

1323 film.actor.film 9181

241 film.person_or_entity_appearing_in_film.film 9182

997 film.person_or_entity_appearing_in_film.film 7532

1142 film.actor.film 8129

1142 film.actor.film 8442

2635 film.person_or_entity_appearing_in_film.film 8486

1157 film.director.film 6998

2523 music.artist.origin 3874

2507 music.artist.origin 3916

815 film.person_or_entity_appearing_in_film.film 7363

811 people.deceased_person.place_of_death 9183

1663 music.artist.origin 7789

267 music.artist.origin 4380

453 film.actor.film 6080

1625 music.artist.origin 6128

624 music.artist.origin 3942

2217 film.person_or_entity_appearing_in_film.film 4331

1035 people.person.profession 7367

2679 film.person_or_entity_appearing_in_film.film 8172

584 film.person_or_entity_appearing_in_film.film 8076

3344 film.person_or_entity_appearing_in_film.film 9173

3447 film.person_or_entity_appearing_in_film.film 6024

1890 people.person.place_of_birth 7789

3589 music.artist.origin 3864

1846 film.actor.film 9184

3159 film.person_or_entity_appearing_in_film.film 6657

2636 film.actor.film 8540

2636 film.person_or_entity_appearing_in_film.film 6437

3821 film.actor.film 5700

1211 film.actor.film 4281

2164 film.person_or_entity_appearing_in_film.film 7542

79 film.person_or_entity_appearing_in_film.film 7958

294 film.person_or_entity_appearing_in_film.film 8970

3176 film.actor.film 8957

602 music.artist.track 4185

630 film.person_or_entity_appearing_in_film.film 9093

2835 music.artist.origin 5845

184 film.person_or_entity_appearing_in_film.film 9017

1363 film.person_or_entity_appearing_in_film.film 6335

1900 music.artist.origin 5900

69 film.actor.film 7348

3467 people.person.place_of_birth 3947

2867 film.person_or_entity_appearing_in_film.film 7635

2241 film.distributor.film 9185

2241 film.distributor.film 6229

2241 film.distributor.film 8898

2241 film.distributor.film 7886

2241 film.production_company.film 6642

2241 film.production_company.film 8600

2241 film.production_company.film 8916

2459 music.musician.instruments_played 4349

1624 film.actor.film 9102

777 film.person_or_entity_appearing_in_film.film 4575

894 film.person_or_entity_appearing_in_film.film 4516

3486 film.person_or_entity_appearing_in_film.film 5643

1957 music.artist.origin 7630

1475 film.person_or_entity_appearing_in_film.film 9186

1081 music.artist.origin 3946

2861 film.person_or_entity_appearing_in_film.film 9187

434 film.person_or_entity_appearing_in_film.film 6284

661 music.album.genre 5811

3049 film.person_or_entity_appearing_in_film.film 8157

3321 film.person_or_entity_appearing_in_film.film 9188

3433 people.person.place_of_birth 6555

2010 film.actor.film 7785

2684 film.actor.film 6935

1358 film.person_or_entity_appearing_in_film.film 6737

997 film.person_or_entity_appearing_in_film.film 4364

396 film.person_or_entity_appearing_in_film.film 6995

1092 music.artist.origin 4057

1157 film.person_or_entity_appearing_in_film.film 9189

2695 film.actor.film 8924

548 film.actor.film 9190

623 film.actor.film 6880

1748 film.actor.film 6456

774 film.actor.film 4760

145 film.actor.film 9191

2737 film.actor.film 6695

604 music.artist.album 8593

2014 film.film.actor 7112

1009 people.person.place_of_birth 9192

74 film.actor.film 4717

646 film.person_or_entity_appearing_in_film.film 9193

2636 film.actor.film 8748

178 people.person.place_of_birth 6947

2122 film.actor.film 4837

3179 film.actor.film 4682

1145 music.artist.origin 8993

445 film.actor.film 7093

2675 people.person.place_of_birth 4658

484 film.person_or_entity_appearing_in_film.film 9194

3738 cvg.publisher.games_published 9195

3738 games.publisher.games_published 8870

625 film.person_or_entity_appearing_in_film.film 5858

2867 film.actor.film 8598

2989 music.artist.origin 4046

2241 film.distributor.film 7461

2241 film.distributor.film 4947

2241 film.distributor.film 5184

2241 film.production_company.film 8065

988 music.artist.album 6410

3200 people.person.place_of_birth 5256

3268 music.artist.origin 4676

1987 music.musician.instruments_played 3959

3198 film.person_or_entity_appearing_in_film.film 4578

261 film.actor.film 9196

1656 film.person_or_entity_appearing_in_film.film 8995

1133 film.actor.film 3934

2678 music.artist.album 6168

25 film.actor.film 8152

434 film.actor.film 4045

2856 music.artist.origin 4174

1074 people.person.place_of_birth 4222

3132 film.person_or_entity_appearing_in_film.film 7269

2533 music.artist.origin 4053

2427 music.artist.origin 4058

56 film.person_or_entity_appearing_in_film.film 4646

56 people.person.place_of_birth 3866

1419 music.artist.origin 4714

2194 music.artist.origin 3897

2242 film.actor.film 8530

997 film.actor.film 8128

2402 film.actor.film 7605

3599 film.actor.film 7127

2635 film.actor.film 6253

1542 music.artist.origin 4167

3460 film.person_or_entity_appearing_in_film.film 9197

2621 film.person_or_entity_appearing_in_film.film 7973

1815 music.artist.origin 4008

598 film.film.writer 6083

2418 music.musician.instruments_played 3959

815 film.actor.film 4757

3020 film.actor.film 6125

774 film.actor.film 9073

2359 film.actor.film 7002

2359 film.actor.film 4375

2201 people.person.place_of_birth 6012

3364 film.actor.film 9198

620 film.actor.film 9199

3735 music.artist.origin 5125

2762 music.artist.origin 5161

2080 film.person_or_entity_appearing_in_film.film 7742

2080 film.person_or_entity_appearing_in_film.film 3975

3158 film.person_or_entity_appearing_in_film.film 8519

3109 film.actor.film 4017

809 film.actor.film 7586

672 film.person_or_entity_appearing_in_film.film 9200

3094 film.actor.film 8520

2892 film.person_or_entity_appearing_in_film.film 4423

1204 film.person_or_entity_appearing_in_film.film 7843

3561 film.actor.film 5674

2636 film.person_or_entity_appearing_in_film.film 9201

147 film.person_or_entity_appearing_in_film.film 7414

2650 film.film.language 4076

1206 people.person.place_of_birth 4496

399 film.actor.film 7140

399 film.person_or_entity_appearing_in_film.film 9202

79 film.person_or_entity_appearing_in_film.film 9017

484 film.actor.film 4136

484 film.actor.film 9194

3151 music.artist.origin 4272

3738 cvg.publisher.games_published 6472

3738 games.publisher.games_published 9203

184 film.person_or_entity_appearing_in_film.film 8824

436 film.person_or_entity_appearing_in_film.film 4085

2400 film.person_or_entity_appearing_in_film.film 6269

2967 film.actor.film 6375

1249 film.actor.film 7677

2241 film.distributor.film 4397

2241 film.production_company.film 4089

2241 film.production_company.film 9204

2241 film.production_company.film 5036

3350 film.person_or_entity_appearing_in_film.film 8971

3430 film.actor.film 9159

964 music.artist.album 3903

1464 film.person_or_entity_appearing_in_film.film 5968

1123 music.artist.origin 5266

777 film.person_or_entity_appearing_in_film.film 6727

1319 music.artist.origin 4057

108 film.person_or_entity_appearing_in_film.film 6668

3031 film.person_or_entity_appearing_in_film.film 5905

1073 music.artist.origin 4456

159 film.person_or_entity_appearing_in_film.film 8561

1059 film.person_or_entity_appearing_in_film.film 3869

1414 music.artist.origin 4606

3052 film.actor.film 5830

3650 music.artist.origin 4071

3390 film.actor.film 9205

380 award.nominee.award_nominations 7666

380 film.actor.film 7394

495 music.artist.origin 5125

3544 film.actor.film 3948

2791 music.artist.origin 8837

1358 film.actor.film 7988

881 people.person.place_of_birth 3945

396 music.artist.origin 6564

2402 film.person_or_entity_appearing_in_film.film 5199

735 music.artist.album 8551

3381 film.actor.film 8727

3307 film.actor.film 8728

186 film.person_or_entity_appearing_in_film.film 8217

623 film.person_or_entity_appearing_in_film.film 9206

3827 film.person_or_entity_appearing_in_film.film 9207

3514 film.person_or_entity_appearing_in_film.film 9169

1153 film.person_or_entity_appearing_in_film.film 6429

3317 film.actor.film 8167

2256 music.artist.origin 4454

3778 people.person.place_of_birth 4010

2708 film.person_or_entity_appearing_in_film.film 7834

1032 film.actor.film 4862

282 film.actor.film 4758

633 film.actor.film 4598

2359 film.actor.film 9208

1386 music.artist.origin 5662

1960 film.actor.film 5983

3040 film.actor.film 6062

947 music.artist.origin 6056

30 film.person_or_entity_appearing_in_film.film 4498

513 music.artist.origin 3846

1033 music.artist.origin 7748

386 film.actor.film 8732

1181 film.actor.film 7719

361 music.producer.tracks_produced 5215

2913 film.actor.film 5167

1298 music.artist.origin 6860

2070	film.person_or_entity_appearing_in_film.film	8978

3179	film.person_or_entity_appearing_in_film.film	9209

49	film.person_or_entity_appearing_in_film.film	8580

660	film.actor.film	5052

484	film.actor.film	7069

3738	games.publisher.games_published	5371

2255	music.artist.origin	3966

436	film.person_or_entity_appearing_in_film.film	7142

625	film.actor.film	7144

2241	film.distributor.film	4691

2241	film.production_company.film	7900

2241	film.production_company.film	8707

2241	film.production_company.film	7960

2610	people.person.place_of_birth	3846

1161	music.artist.origin	4502

2487	film.person_or_entity_appearing_in_film.film	8938

1618	film.person_or_entity_appearing_in_film.film	9147

2346	film.person_or_entity_appearing_in_film.film	8325

1504	people.person.place_of_birth	5828

1278	film.actor.film	4837

3321	film.person_or_entity_appearing_in_film.film	8185

1783	film.person_or_entity_appearing_in_film.film	9210

287	film.actor.film	9211

3048	film.actor.film	5918

3132	film.actor.film	4644

2584	film.actor.film	7577

1057	film.actor.film	8186

1067	film.person_or_entity_appearing_in_film.film	6934

3681	music.artist.origin	7262

68	film.person_or_entity_appearing_in_film.film	5267

206 film.actor.film 5955

1291 film.actor.film 9212

567 film.actor.film 9001

567 film.actor.film 8106

1839 film.actor.film 6620

1006 award.winner.awards_won 9213

280 film.person_or_entity_appearing_in_film.film 7533

2494 film.actor.film 6597

994 music.artist.origin 4053

1760 music.artist.origin 4635

3827 film.person_or_entity_appearing_in_film.film 7030

1998 film.actor.film 8367

67 film.person_or_entity_appearing_in_film.film 5193

3412 film.film.producer 8641

2733 film.actor.film 7186

1473 film.actor.film 7488

37 film.actor.film 8739

2337 film.person_or_entity_appearing_in_film.film 8776

3040 film.actor.film 7698

145 film.actor.film 4136

2737 film.actor.film 6129

2270 music.artist.origin 3966

279 film.actor.film 5479

3733 film.actor.film 8931

1677 music.artist.origin 4963

645 film.person_or_entity_appearing_in_film.film 9214

645 film.person_or_entity_appearing_in_film.film 8731

3341 music.artist.origin 5497

2726 film.person_or_entity_appearing_in_film.film 8003

3109 film.person_or_entity_appearing_in_film.film 8856

2115 people.person.place_of_birth 4496

1704 film.person_or_entity_appearing_in_film.film 8057

151 film.person_or_entity_appearing_in_film.film 7465

2096 people.person.place_of_birth 5227

679 film.actor.film 7376

2688 film.actor.film 6221

3179 film.person_or_entity_appearing_in_film.film 9215

3179 film.person_or_entity_appearing_in_film.film 9216

366 film.actor.film 9217

3166 film.actor.film 6413

1276 music.artist.origin 3855

2778 music.artist.album 8833

484 film.actor.film 7318

3738 cvg.publisher.games_published 9218

901 film.person_or_entity_appearing_in_film.film 6547

625 film.actor.film 5958

1121 music.artist.origin 4217

1809 music.artist.origin 4454

2867 film.person_or_entity_appearing_in_film.film 8097

2241 film.distributor.film 8881

2241 film.distributor.film 6788

2241 film.distributor.film 5932

2241 film.production_company.film 8003

1908 music.artist.origin 6012

932 music.artist.origin 5421

2287 music.album.genre 8909

1822 music.artist.origin 6215

3031 film.actor.film 8996

2098 film.person_or_entity_appearing_in_film.film 7799

865 film.actor.film 7495

1526 film.person_or_entity_appearing_in_film.film 5191

2429 film.person_or_entity_appearing_in_film.film 5261

3557 film.film.genre 4062

2999 film.actor.film 5360

3132 film.actor.film 8512

3434 film.person_or_entity_appearing_in_film.film 9141

1716 music.artist.origin 4479

959 film.person_or_entity_appearing_in_film.film 7907

1776 people.person.place_of_birth 4013

2833 film.actor.film 5398

2076 event.agent.performance 4035

2635 film.actor.film 7429

3460 film.actor.film 9197

3224 people.person.place_of_birth 7955

1893 music.artist.origin 5238

3384 film.actor.film 9219

1006 people.person.place_of_birth 4800

1633 film.person_or_entity_appearing_in_film.film 8637

410 film.actor.film 4051

1889 people.person.place_of_birth 4347

1044 award.nominee.award_nominations 6294

3827 film.actor.film 9207

3253 music.artist.origin 6504

2953 film.actor.film 4968

2683 film.person_or_entity_appearing_in_film.film 4959

2804 film.actor.film 7885

1473 film.actor.film 6717

1193 film.actor.film 4385

2983 film.actor.film 6061

1666 music.artist.origin 4380

239 film.actor.film 5162

1845 film.person_or_entity_appearing_in_film.film 8908

809 film.person_or_entity_appearing_in_film.film 9090

80 music.producer.tracks_produced 6604

544 film.actor.film 4837

896 film.person_or_entity_appearing_in_film.film 7841

46 award.competitor.award_nominations 6304

2352 music.artist.origin 4057

1601 film.person_or_entity_appearing_in_film.film 8839

2095 film.actor.film 5324

1846 film.actor.film 8894

1846 film.person_or_entity_appearing_in_film.film 9184

2636 film.person_or_entity_appearing_in_film.film 8494

2437 film.actor.film 9220

679 music.artist.origin 3961

3138 music.artist.origin 3859

381 film.actor.film 8040

1211 film.person_or_entity_appearing_in_film.film 6037

2968 film.person_or_entity_appearing_in_film.film 7659

366 film.person_or_entity_appearing_in_film.film 5853

49 film.actor.film 9092

2682 film.person_or_entity_appearing_in_film.film 7773

484 film.actor.film 6144

3738 games.publisher.games_published 4618

184 film.person_or_entity_appearing_in_film.film 7416

1808 film.person_or_entity_appearing_in_film.film 9221

1477 music.artist.origin 4496

2241 film.distributor.film 9050

2241 film.distributor.film 8994

2241 film.production_company.film 4129

2487	film.person_or_entity_appearing_in_film.film	8350

2979	film.actor.film	9011

2701	music.artist.origin	5390

3026	film.person_or_entity_appearing_in_film.film	8482

3026	film.person_or_entity_appearing_in_film.film	5721

3279	film.person_or_entity_appearing_in_film.film	6455

2658	film.actor.film	7784

638 film.actor.film	9012

2296	film.person_or_entity_appearing_in_film.film	4590

3551	film.person_or_entity_appearing_in_film.film	8953

3637	film.person_or_entity_appearing_in_film.film	6560

2268	people.person.place_of_birth	6111

2461	people.person.place_of_birth	4057

1087	film.person_or_entity_appearing_in_film.film	7107

567 film.person_or_entity_appearing_in_film.film	9222

2635	film.actor.film	8277

2635	film.actor.film	7668

2635	film.person_or_entity_appearing_in_film.film	8851

1092	film.person_or_entity_appearing_in_film.film	5310

379 film.person_or_entity_appearing_in_film.film	8107

1584	people.person.place_of_birth	4057

540 film.person_or_entity_appearing_in_film.film	5488

2161	award.nominee.award_nominations	9223

2670	film.actor.film	5837

2336	people.person.place_of_birth	5793

937 music.artist.origin	4057

492 film.person_or_entity_appearing_in_film.film	5008

845 music.artist.origin	4094

82	film.person_or_entity_appearing_in_film.film	7084

1153	film.person_or_entity_appearing_in_film.film	6398

879 film.actor.film 6085

3020 people.person.place_of_birth 8390

3788 film.person_or_entity_appearing_in_film.film 9224

2804 film.actor.film 6088

1473 film.person_or_entity_appearing_in_film.film 8654

1141 award.nominee.award_nominations 9225

991 music.artist.origin 7630

172 music.artist.origin 5270

91 film.person_or_entity_appearing_in_film.film 7051

3828 music.artist.origin 7302

3598 film.actor.film 8704

3598 film.person_or_entity_appearing_in_film.film 8190

3598 film.person_or_entity_appearing_in_film.film 6773

1604 music.artist.origin 4199

3282 film.person_or_entity_appearing_in_film.film 9226

590 music.artist.origin 4077

361 music.artist.track 7249

2913 film.person_or_entity_appearing_in_film.film 3910

70 film.actor.film 4600

2636 film.person_or_entity_appearing_in_film.film 5537

2460 film.actor.film 6141

3805 music.artist.origin 4040

1981 film.actor.film 6471

3738 games.publisher.games_published 8737

436 film.actor.film 5329

2224 music.artist.origin 7804

901 film.director.film 8524

3025 film.person_or_entity_appearing_in_film.film 7794

2241 film.production_company.film 8476

2241 film.production_company.film 3862

795 film.actor.film 6087

795 film.person_or_entity_appearing_in_film.film 4915

795 film.person_or_entity_appearing_in_film.film 5299

1936 film.actor.film 7322

1762 film.person_or_entity_appearing_in_film.film 9019

2443 music.artist.origin 4454

2038 film.actor.film 8254

3669 film.person_or_entity_appearing_in_film.film 5511

1360 film.person_or_entity_appearing_in_film.film 8440

643 film.actor.film 9227

2677 film.person_or_entity_appearing_in_film.film 9042

636 film.actor.film 8010

1357 film.person_or_entity_appearing_in_film.film 8426

434 film.person_or_entity_appearing_in_film.film 8873

1580 people.person.place_of_birth 7218

1526 film.actor.film 7689

3049 film.person_or_entity_appearing_in_film.film 6423

3321 film.person_or_entity_appearing_in_film.film 9228

1954 film.person_or_entity_appearing_in_film.film 7923

3742 film.writer.film 5050

3081 music.artist.origin 4380

3390 film.actor.film 9229

3390 film.actor.film 9230

1368 music.artist.origin 5662

1816 film.actor.film 8552

2962 people.deceased_person.place_of_death 4362

638 film.actor.film 7428

1962 film.person_or_entity_appearing_in_film.film 8921

3826 music.artist.origin 3864

2252 film.person_or_entity_appearing_in_film.film 9231

2530 music.artist.origin 6636

206 film.actor.film 8941

206 film.person_or_entity_appearing_in_film.film 9232

900 people.person.place_of_birth 8745

2402 film.director.film 5913

2635 film.actor.film 4429

1157 film.actor.film 9233

1157 film.person_or_entity_appearing_in_film.film 9151

3384 film.person_or_entity_appearing_in_film.film 9219

941 music.artist.origin 4174

2050 film.actor.film 8431

863 music.album.track 7004

3029 film.actor.film 7994

72 film.person_or_entity_appearing_in_film.film 4980

3061 event.agent.performance 5097

12 film.person_or_entity_appearing_in_film.film 5842

3725 people.person.place_of_birth 3866

1223 film.person_or_entity_appearing_in_film.film 8491

893 film.actor.film 6100

1005 music.artist.origin 4380

1764 film.person_or_entity_appearing_in_film.film 6887

646 film.person_or_entity_appearing_in_film.film 9234

628 film.person_or_entity_appearing_in_film.film 4290

447 people.person.place_of_birth 3916

3355 film.actor.film 8625

2070 film.actor.film 6747

2070 film.actor.film 9101

3179 film.actor.film 9209

1370 film.film.editor 9110

49 film.writer.film 7440

2460 film.person_or_entity_appearing_in_film.film 9235

79 film.person_or_entity_appearing_in_film.film 6429

294 film.actor.film 8812

3634 music.musician.instruments_played 6114

2877 film.person_or_entity_appearing_in_film.film 5110

3738 games.publisher.games_published 8814

625 film.person_or_entity_appearing_in_film.film 8335

2342 music.artist.origin 4053

119 music.artist.track 8597

2241 film.production_company.film 7754

2241 film.production_company.film 6000

1936 people.person.place_of_birth 4013

3297 film.person_or_entity_appearing_in_film.film 7963

599 film.actor.film 7048

2487 film.person_or_entity_appearing_in_film.film 8677

2965 music.artist.origin 4010

2126 film.person_or_entity_appearing_in_film.film 8662

3330 film.person_or_entity_appearing_in_film.film 6753

2275 people.person.place_of_birth 4191

1567 film.person_or_entity_appearing_in_film.film 4328

2707 film.actor.film 5140

1772 film.person_or_entity_appearing_in_film.film 8378

3420 music.artist.origin 3864

2162 film.actor.film 7652

538 film.actor.film 8547

538 film.person_or_entity_appearing_in_film.film 6039

3049 film.person_or_entity_appearing_in_film.film 9236

238 film.actor.film 8550

1568 music.artist.origin 3932

3650 film.person_or_entity_appearing_in_film.film 8742

3390 film.person_or_entity_appearing_in_film.film 7985

569 music.artist.origin 6509

1593 music.artist.origin 4148

1816 film.person_or_entity_appearing_in_film.film 7299

1816 film.writer.film 4643

241 film.actor.film 9237

1478 music.artist.origin 4496

3132 film.person_or_entity_appearing_in_film.film 9238

3545 film.person_or_entity_appearing_in_film.film 4822

2820 film.actor.film 6157

567 film.actor.film 6289

2499 music.artist.origin 9239

2635 film.actor.film 6523

3384 film.person_or_entity_appearing_in_film.film 9240

2668 film.person_or_entity_appearing_in_film.film 9241

548 film.actor.film 8963

1505 film.actor.film 8218

641 film.actor.film 5104

362 film.person_or_entity_appearing_in_film.film 7245

2708 people.deceased_person.place_of_death 9242

3792 film.person_or_entity_appearing_in_film.film 9243

1141 film.actor.film 9244

939 film.actor.film 9245

2616 people.person.place_of_birth 5213

1935 film.person_or_entity_appearing_in_film.film 8893

2048 music.artist.origin 9246

1845 film.actor.film 9144

66 film.person_or_entity_appearing_in_film.film 9247

2488 film.person_or_entity_appearing_in_film.film 7716

3396 film.actor.film 5109

3344 film.person_or_entity_appearing_in_film.film 6533

3447 film.actor.film 6024

2914 film.person_or_entity_appearing_in_film.film 3970

2914 film.person_or_entity_appearing_in_film.film 5929

319 film.film.actor 8521

437 film.actor.film 8193

128 film.person_or_entity_appearing_in_film.film 7439

3531 music.artist.origin 3916

2674 type.object.key 2674

1355 film.actor.film 4506

3179 film.actor.film 5368

2185 music.artist.origin 6137

1353 film.person_or_entity_appearing_in_film.film 9248

3738 cvg.publisher.games_published 5669

3738 cvg.publisher.games_published 9249

901 film.actor.film 6270

3161 film.person_or_entity_appearing_in_film.film 5961

2241 film.distributor.film 6819

2241 film.production_company.film 7872

2241 film.production_company.film 6701

2241 film.production_company.film 4625

1762 film.person_or_entity_appearing_in_film.film 8617

392 film.actor.film 4792

998 event.agent.performance 4387

1656 film.person_or_entity_appearing_in_film.film 5474

1656 film.person_or_entity_appearing_in_film.film 9250

1768 film.actor.film 9251

1533 music.artist.origin 4454

3486 film.actor.film 7425

825 music.artist.origin 4852

3665 film.person_or_entity_appearing_in_film.film 6283

1536 film.person_or_entity_appearing_in_film.film 4132

3049 film.actor.film 8102

2387 film.actor.film 9180

2399 film.actor.film 4124

2836 film.person_or_entity_appearing_in_film.film 6319

1619 film.actor.film 9252

3057 music.artist.origin 4013

2509 music.artist.origin 4057

216 music.artist.origin 6331

3203 music.artist.origin 5265

241 film.actor.film 9253

3132 film.actor.film 9150

3551 music.artist.origin 4552

1985 film.actor.film 7271

1358 film.person_or_entity_appearing_in_film.film 6784

2904 film.person_or_entity_appearing_in_film.film 7380

2107 film.actor.film 9254

3460 film.actor.film 8621

3270 music.artist.origin 3864

818 film.person_or_entity_appearing_in_film.film 9060

1633 film.actor.film 8905

2494 film.person_or_entity_appearing_in_film.film 8515

623 film.actor.film 5792

2084 film.actor.film 5354

3018 music.artist.origin 4707

2698 music.artist.origin 3897

1153 film.actor.film 6430

3068 people.person.place_of_birth 4013

3364 film.person_or_entity_appearing_in_film.film 9172

2998 film.person_or_entity_appearing_in_film.film 4326

3784 film.editor.film 7061

39 film.person_or_entity_appearing_in_film.film 6571

2069 film.actor.film 6024

66 film.actor.film 9247

2589 music.artist.origin 4502

3598 film.person_or_entity_appearing_in_film.film 9255

3447 film.actor.film 7088

2338 film.actor.film 5286

1878 music.artist.origin 3945

2848 film.film.country 4380

70 film.person_or_entity_appearing_in_film.film 8733

437 film.person_or_entity_appearing_in_film.film 8992

2636 film.actor.film 9256

2636 film.actor.film 7413

2437 film.actor.film 9048

2122 film.person_or_entity_appearing_in_film.film 8844

2819 music.artist.album 8825

49 film.actor.film 8895

3738 games.publisher.games_published 8646

3738 games.publisher.games_published 8659

703 music.artist.origin 3859

3248 film.actor.film 9257

2867 film.person_or_entity_appearing_in_film.film 5432

2004 music.artist.origin 4362

3190 music.artist.origin 4077

1614 people.person.place_of_birth 4217

522 people.person.place_of_birth 5924

2487 film.person_or_entity_appearing_in_film.film 9009

356 film.actor.film 9160

1562	film.actor.film	4915
2678	film.person_or_entity_appearing_in_film.film	8830
719	film.actor.film	5553
1798	film.person_or_entity_appearing_in_film.film	8758
2707	film.person_or_entity_appearing_in_film.film	3937
1712	music.artist.origin	4362
25	film.person_or_entity_appearing_in_film.film	8152
3509	film.actor.film	7389
996	music.artist.origin	4272
467	music.artist.origin	4191
3579	film.person_or_entity_appearing_in_film.film	6681
810	film.actor.film	6824
1514	music.artist.origin	4391
3648	film.person_or_entity_appearing_in_film.film	7393
68	film.actor.film	7958
2833	film.actor.film	9058
2904	film.person_or_entity_appearing_in_film.film	8923
186	film.person_or_entity_appearing_in_film.film	9258
1006	award.competitor.awards_won	9213
1633	film.person_or_entity_appearing_in_film.film	6054
2494	film.person_or_entity_appearing_in_film.film	4652
785	music.musician.instruments_played	4349
1577	music.artist.origin	5228
3827	film.person_or_entity_appearing_in_film.film	6202
395	film.actor.film	7311
1833	film.person_or_entity_appearing_in_film.film	9088
67	film.actor.film	5457
67	film.person_or_entity_appearing_in_film.film	5278
67	film.person_or_entity_appearing_in_film.film	8131
3472	music.artist.origin	4583

2359 film.person_or_entity_appearing_in_film.film 9208

2125 film.actor.film 5621

1473 film.actor.film 6092

793 film.actor.film 5758

3040 film.actor.film 5761

620 film.person_or_entity_appearing_in_film.film 9199

192 music.artist.track 5846

2702 music.artist.origin 4214

3295 film.actor.film 8294

3598 film.actor.film 7037

3396 film.person_or_entity_appearing_in_film.film 8121

2637 film.film.genre 5809

3211 film.actor.film 7639

80 people.person.place_of_birth 3846

2555 film.person_or_entity_appearing_in_film.film 6590

3313 film.actor.film 5786

628 film.actor.film 8225

603 film.actor.film 7253

2644 music.artist.origin 4362

2636 film.actor.film 8113

2437 film.actor.film 9174

2475 film.actor.film 7997

781 film.person_or_entity_appearing_in_film.film 8890

1308 music.producer.tracks_produced 5251

1307 film.actor.film 4508

484 film.person_or_entity_appearing_in_film.film 7141

3738 games.publisher.games_published 6105

184 film.actor.film 5918

2360 film.person_or_entity_appearing_in_film.film 6309

2360 music.artist.album 4589

2331 film.actor.film 5842

1624 film.actor.film 4942

777 people.person.place_of_birth 3864

1148 film.person_or_entity_appearing_in_film.film 5842

97 music.artist.origin 4380

2012 music.artist.origin 4764

3486 film.actor.film 4350

3486 film.actor.film 9259

3486 film.person_or_entity_appearing_in_film.film 7050

2206 film.actor.film 5552

2884 film.person_or_entity_appearing_in_film.film 8527

3672 music.artist.origin 5845

3321 film.person_or_entity_appearing_in_film.film 9010

814 film.actor.film 5347

416 film.person_or_entity_appearing_in_film.film 3875

1332 film.producer.film 4265

3048 film.person_or_entity_appearing_in_film.film 4854

2449 film.person_or_entity_appearing_in_film.film 7239

2076 film.actor.film 8514

1662 film.actor.film 6800

3381 film.actor.film 9130

3381 film.person_or_entity_appearing_in_film.film 6968

2297 film.actor.film 8787

2297 film.person_or_entity_appearing_in_film.film 9260

2476 music.artist.origin 3859

2668 film.actor.film 9241

1732 music.album.genre 6212

191 film.actor.film 9100

623 film.person_or_entity_appearing_in_film.film 7696

3827 film.actor.film 9261

1153 film.person_or_entity_appearing_in_film.film 7832

3792 film.person_or_entity_appearing_in_film.film 5292

1473 film.actor.film 8990

3204 film.actor.film 9164

3229 film.actor.film 6335

145 film.person_or_entity_appearing_in_film.film 9191

975 music.artist.origin 6557

2726 film.actor.film 7343

1616 film.person_or_entity_appearing_in_film.film 4477

948 film.person_or_entity_appearing_in_film.film 9262

1393 film.actor.film 9263

367 music.artist.origin 6860

2369 music.artist.origin 4199

2830 film.actor.film 9264

1469 music.artist.track 8315

2562 film.person_or_entity_appearing_in_film.film 8732

1812 film.person_or_entity_appearing_in_film.film 6979

1082 film.person_or_entity_appearing_in_film.film 8356

3148 music.artist.origin 7399

184 film.actor.film 6268

1249 film.actor.film 6638

1463 award.nominee.award_nominations 8361

2241 film.distributor.film 7036

795 film.actor.film 5694

2617 music.artist.origin 3864

943 film.actor.film 8452

1767 film.person_or_entity_appearing_in_film.film 9166

3031 film.actor.film 9167

365 people.person.place_of_birth 4133

1278 award.nominee.award_nominations 6420

1684 music.artist.origin 7361

291 music.artist.origin 4081

3299 music.artist.origin 4204

1387 film.actor.film 9265

3390 film.person_or_entity_appearing_in_film.film 9230

3048 film.person_or_entity_appearing_in_film.film 7014

241 film.actor.film 8257

2296 people.deceased_person.place_of_death 3864

2010 people.deceased_person.place_of_death 8586

3132 film.actor.film 8049

1251 film.person_or_entity_appearing_in_film.film 9266

1985 film.person_or_entity_appearing_in_film.film 8105

2635 film.actor.film 8835

2635 film.person_or_entity_appearing_in_film.film 5616

1157 film.person_or_entity_appearing_in_film.film 6941

3460 music.musician.instruments_played 3959

186 film.actor.film 9258

3642 music.artist.origin 4183

548 film.actor.film 7741

1044 award.competitor.award_nominations 7950

517 music.musician.instruments_played 3959

1170 music.artist.origin 4013

286 film.person_or_entity_appearing_in_film.film 7085

1833 film.actor.film 8964

1833 music.artist.track 5729

2865 film.actor.film 5869

1490 music.artist.origin 4977

1910 film.film.country 4676

3215 film.film.language 4076

2482 film.actor.film 8574

3207 people.person.place_of_birth 4133

2737 film.person_or_entity_appearing_in_film.film 4921

239 award.competitor.award_nominations 9267

66 film.actor.film 8623

948 film.actor.film 9109

2259 film.actor.film 7040

408 film.actor.film 6498

1204 film.person_or_entity_appearing_in_film.film 5892

3050 music.artist.origin 3864

2636 film.actor.film 4773

2636 film.person_or_entity_appearing_in_film.film 7538

1355 film.actor.film 7592

399 film.person_or_entity_appearing_in_film.film 7559

484 film.actor.film 5855

3738 cvg.publisher.games_published 7347

3738 games.publisher.games_published 5075

352 music.artist.origin 3866

949 people.person.place_of_birth 5773

2001 people.person.place_of_birth 4000

2360 film.person_or_entity_appearing_in_film.film 3988

2241 film.distributor.film 5131

2241 film.production_company.film 5436

1464 film.actor.film 8987

2487 film.actor.film 5939

2041 music.artist.origin 4057

3354 film.actor.film 9268

1360 film.actor.film 9040

1360 film.person_or_entity_appearing_in_film.film 8783

3546 type.object.subject_key 3546

538 film.person_or_entity_appearing_in_film.film 5389

2870 film.person_or_entity_appearing_in_film.film 7052

3321 film.person_or_entity_appearing_in_film.film 7099

1623 film.person_or_entity_appearing_in_film.film 6151

2413 people.person.place_of_birth 6502

547 film.actor.film 8888

3508 film.actor.film 6087

1867 music.artist.origin 4496

2938 film.person_or_entity_appearing_in_film.film 5069

2635 film.actor.film 8149

1157 film.actor.film 4052

1157 film.writer.film 4906

1633 film.actor.film 4859

689 music.artist.origin 4000

1044 people.person.profession 4221

3827 film.actor.film 9132

2050 film.actor.film 7833

67 film.actor.film 5207

1473 film.actor.film 9269

1473 film.person_or_entity_appearing_in_film.film 5410

1813 people.deceased_person.place_of_death 3864

1497 film.actor.film 4328

761 music.artist.origin 4714

2545 music.artist.origin 4272

3498 film.person_or_entity_appearing_in_film.film 5193

472 film.actor.film 4498

852 film.person_or_entity_appearing_in_film.film 6139

2179 music.musician.instruments_played 6942

1204 film.person_or_entity_appearing_in_film.film 9270

1934 music.artist.origin 4171

201 music.artist.origin 3864

2674 music.artist.album 9062

178 film.person_or_entity_appearing_in_film.film 5584

427 film.person_or_entity_appearing_in_film.film 5539

49 film.actor.film 8613

3166 people.deceased_person.place_of_death 4977

484 film.person_or_entity_appearing_in_film.film 9155

484 film.person_or_entity_appearing_in_film.film 3985

3738 cvg.publisher.games_published 7382

3738 cvg.publisher.games_published 8689

934 music.artist.origin 3961

625 film.person_or_entity_appearing_in_film.film 6981

1872 film.person_or_entity_appearing_in_film.film 8139

2241 film.distributor.film 9175

2241 film.production_company.film 8756

2241 film.production_company.film 9082

2779 music.artist.album 5796

1917 film.person_or_entity_appearing_in_film.film 6149

3160 people.person.place_of_birth 5270

1464 film.person_or_entity_appearing_in_film.film 8917

932 film.person_or_entity_appearing_in_film.film 7323

1624 film.actor.film 8322

777 film.person_or_entity_appearing_in_film.film 9095

1777 film.actor.film 9030

1621 music.artist.origin 4960

3296 film.person_or_entity_appearing_in_film.film 8723

3105 music.artist.origin 5756

2731 film.actor.film 8560

3780 film.person_or_entity_appearing_in_film.film 8498

1475 film.actor.film 3868

1475 film.person_or_entity_appearing_in_film.film 9271

538 film.person_or_entity_appearing_in_film.film 8887

652 music.artist.origin 4150

1956 people.person.place_of_birth 3866

3321 film.actor.film 7663

3433 film.person_or_entity_appearing_in_film.film 7691

3742 music.artist.album 6196

2519 film.person_or_entity_appearing_in_film.film 5912

532 music.artist.origin 4087

3390 film.actor.film 5723

158 music.artist.origin 4471

3279 film.actor.film 7648

241 film.writer.film 9237

923 film.person_or_entity_appearing_in_film.film 9032

3132 film.person_or_entity_appearing_in_film.film 8681

2312 people.person.place_of_birth 4347

3763 film.actor.film 8873

3822 film.person_or_entity_appearing_in_film.film 6046

1358 film.person_or_entity_appearing_in_film.film 4675

997 film.person_or_entity_appearing_in_film.film 8635

3599 people.person.place_of_birth 4000

2635 film.actor.film 9131

1528 music.artist.origin 4214

131 film.actor.film 6052

186 film.actor.film 4164

1719 film.actor.film 6428

1633 film.person_or_entity_appearing_in_film.film 5914

623 film.actor.film 6565

2724 film.actor.film 4538

3319 music.artist.origin 4010

1153 film.actor.film 9170

2786	people.person.profession	4990

995 film.actor.film	6432

2079	film.person_or_entity_appearing_in_film.film	9272

2712	film.actor.film	7215

2733	people.deceased_person.place_of_death	5161

2619	film.actor.film	4323

2337	film.person_or_entity_appearing_in_film.film	5412

3741	people.person.place_of_birth	7109

1272	music.artist.origin	4454

2480	people.person.place_of_birth	4013

407 film.person_or_entity_appearing_in_film.film	9273

2907	music.artist.origin	3864

3835	people.deceased_person.place_of_death	4658

1885	film.person_or_entity_appearing_in_film.film	9274

2027	music.artist.origin	8712

2913	film.person_or_entity_appearing_in_film.film	4845

151 award.nominee.award_nominations	8434

3790	people.person.place_of_birth	4008

357 film.actor.film	9275

974 film.actor.film	8810

368 type.object.subject_key	368

2743	film.actor.film	9276

3821	film.actor.film	7894

2070	film.actor.film	8735

1211	film.person_or_entity_appearing_in_film.film	4281

1944	people.person.place_of_birth	7637

2003	music.artist.origin	5845

2460	film.actor.film	4559

2460	film.writer.film	5457

79	film.actor.film	6429

3738 games.publisher.games_published 8523

3738 games.publisher.games_published 5501

3738 games.publisher.games_published 9195

2215 music.artist.origin 3961

2241 film.distributor.film 7777

2241 film.distributor.film 6579

2241 film.distributor.film 8321

2241 film.production_company.film 7546

1464 film.actor.film 8883

3515 film.person_or_entity_appearing_in_film.film 8693

3726 music.artist.origin 4362

812 people.deceased_person.place_of_death 5258

1461 people.person.place_of_birth 5064

3165 film.actor.film 8425

2880 film.person_or_entity_appearing_in_film.film 8859

2200 film.actor.film 7772

3486 film.person_or_entity_appearing_in_film.film 9277

2969 music.musician.instruments_played 5174

1357 film.person_or_entity_appearing_in_film.film 9178

1378 film.actor.film 8528

1926 film.actor.film 9179

1869 music.artist.origin 7646

1954 film.actor.film 6897

2375 music.artist.origin 3864

1382 music.musician.instruments_played 4349

3390 film.person_or_entity_appearing_in_film.film 9278

158 music.artist.album 7791

3279 film.person_or_entity_appearing_in_film.film 9096

2711 film.person_or_entity_appearing_in_film.film 7501

2711 film.person_or_entity_appearing_in_film.film 7801

2449	film.actor.film	4385

900 music.musician.instruments_played	3851

1279	film.actor.film	9279

32	film.actor.film	4774

3381	film.person_or_entity_appearing_in_film.film	9106

2635	film.actor.film	8263

2635	film.actor.film	8849

2378	people.person.place_of_birth	7515

1349	film.actor.film	8487

379 film.actor.film	4826

1633	film.person_or_entity_appearing_in_film.film	6525

548 film.actor.film	7029

3827	film.person_or_entity_appearing_in_film.film	6856

2371	film.person_or_entity_appearing_in_film.film	8290

67	film.person_or_entity_appearing_in_film.film	7507

2216	music.artist.track 4818

645 film.actor.film	6975

3574	music.artist.origin	4332

3598	film.producer.film	7132

1410	music.artist.origin	7824

330 music.artist.origin	4454

3158	film.actor.film	5893

3280	people.person.place_of_birth	4347

646 film.person_or_entity_appearing_in_film.film	9280

1601	film.actor.film	9037

2636	film.actor.film	8461

1355	award.winner.awards_won	4878

1579	film.actor.film	8373

484 film.actor.film	8546

484 film.person_or_entity_appearing_in_film.film	9281

3738 games.publisher.games_published 5634

436 film.person_or_entity_appearing_in_film.film 7015

966 music.artist.origin 4391

625 film.person_or_entity_appearing_in_film.film 7383

2559 film.actor.film 9282

2241 film.distributor.film 9003

2241 film.distributor.film 5038

2241 film.production_company.film 8272

1624 film.person_or_entity_appearing_in_film.film 3886

777 film.actor.film 8710

3486 film.actor.film 9277

3105 music.artist.origin 3961

1567 film.actor.film 4846

2206 film.person_or_entity_appearing_in_film.film 9283

2493 film.actor.film 3940

538 film.person_or_entity_appearing_in_film.film 7356

1430 film.person_or_entity_appearing_in_film.film 8548

3772 film.film.genre 4062

3000 people.person.place_of_birth 4133

3049 film.actor.film 8861

2054 film.person_or_entity_appearing_in_film.film 9161

378 people.person.place_of_birth 8902

1398 film.actor.film 4911

3279 film.person_or_entity_appearing_in_film.film 7648

3648 film.person_or_entity_appearing_in_film.film 5392

3048 film.person_or_entity_appearing_in_film.film 5918

1816 film.person_or_entity_appearing_in_film.film 7456

241 film.actor.film 8240

2240 film.actor.film 9284

2240 film.person_or_entity_appearing_in_film.film 9284

2584 film.person_or_entity_appearing_in_film.film 9285

275 music.artist.origin 3899

1358 film.person_or_entity_appearing_in_film.film 7484

3088 film.person_or_entity_appearing_in_film.film 7989

396 film.person_or_entity_appearing_in_film.film 5486

3194 film.person_or_entity_appearing_in_film.film 9286

1943 film.actor.film 9287

1989 film.actor.film 7214

492 film.person_or_entity_appearing_in_film.film 6203

2050 people.person.place_of_birth 4174

3788 film.actor.film 4373

1383 film.person_or_entity_appearing_in_film.film 7364

3792 film.actor.film 9243

1141 award.competitor.award_nominations 9225

2619 film.person_or_entity_appearing_in_film.film 5758

939 film.person_or_entity_appearing_in_film.film 9245

3074 film.person_or_entity_appearing_in_film.film 4602

1200 people.person.place_of_birth 7433

711 music.artist.origin 7326

2341 film.actor.film 8534

66 film.person_or_entity_appearing_in_film.film 4070

3277 music.artist.origin 5557

893 film.person_or_entity_appearing_in_film.film 9036

2899 music.artist.origin 5756

106 film.actor.film 8716

2259 film.actor.film 7890

1702 film.person_or_entity_appearing_in_film.film 9288

2303 music.artist.origin 5139

319 film.film.genre 4062

437 film.actor.film 4181

2636 film.person_or_entity_appearing_in_film.film 8966

2636 film.person_or_entity_appearing_in_film.film 8134

2437 film.actor.film 9064

2674 music.artist.album 6211

2597 film.person_or_entity_appearing_in_film.film 6347

5 music.artist.origin 4010

1355 film.person_or_entity_appearing_in_film.film 8967

2198 music.artist.origin 4453

3308 music.artist.origin 3966

1353 film.person_or_entity_appearing_in_film.film 6219

79 film.actor.film 8333

294 film.person_or_entity_appearing_in_film.film 7813

278 film.person_or_entity_appearing_in_film.film 8796

484 film.actor.film 9289

3738 cvg.publisher.games_published 4344

3738 cvg.publisher.games_published 4679

2967 film.actor.film 6625

2477 music.artist.origin 4050

1746 music.artist.origin 3947

899 film.actor.film 5466

3501 film.actor.film 5081

2360 film.person_or_entity_appearing_in_film.film 8252

2241 film.distributor.film 8232

2241 film.distributor.film 9039

2241 film.distributor.film 9051

2241 film.distributor.film 7635

2241 film.production_company.film 8599

2241 film.production_company.film 9290

2779 film.person_or_entity_appearing_in_film.film 8828

2732 film.person_or_entity_appearing_in_film.film 4136

3354 award.nominee.award_nominations 9291

2206 music.artist.origin 4262

865 film.person_or_entity_appearing_in_film.film 5990

3049 film.actor.film 7294

1046 film.actor.film 7452

2634 people.person.place_of_birth 4191

1717 music.artist.origin 4957

2711 film.person_or_entity_appearing_in_film.film 7760

241 film.person_or_entity_appearing_in_film.film 9237

916 film.person_or_entity_appearing_in_film.film 5234

692 people.person.place_of_birth 5362

2449 film.person_or_entity_appearing_in_film.film 5394

1479 film.actor.film 5562

3551 people.person.place_of_birth 4552

1750 film.actor.film 8050

997 film.actor.film 9117

1349 people.person.place_of_birth 3947

3101 music.artist.origin 3961

115 film.person_or_entity_appearing_in_film.film 7022

2494 film.person_or_entity_appearing_in_film.film 6247

3842 music.artist.origin 3864

1748 film.actor.film 7736

1748 film.person_or_entity_appearing_in_film.film 6690

2210 film.actor.film 6734

2370 film.person_or_entity_appearing_in_film.film 9292

1473 film.person_or_entity_appearing_in_film.film 8038

1473 film.person_or_entity_appearing_in_film.film 9293

1026 film.actor.film 5012

3364 film.person_or_entity_appearing_in_film.film 9198

2145 people.person.place_of_birth 9239

1199 music.artist.origin 4442

377 film.actor.film 9294

987 film.actor.film 6914

1219 people.person.place_of_birth 4217

1296 film.actor.film 7369

2214 film.person_or_entity_appearing_in_film.film 3886

3282 film.person_or_entity_appearing_in_film.film 4127

2881 film.actor.film 8536

1079 film.cinematographer.film6169

3498 film.person_or_entity_appearing_in_film.film 8312

2784 film.actor.film 7089

361 award.competitor.award_nominations 9295

2914 film.actor.film 9296

1702 film.actor.film 9288

2367 music.artist.album 6183

147 film.person_or_entity_appearing_in_film.film 9028

2743 film.person_or_entity_appearing_in_film.film 9276

2122 award.nominee.award_nominations 4293

399 film.actor.film 9202

399 film.person_or_entity_appearing_in_film.film 7224

445 film.actor.film 8979

1667 music.artist.origin 3897

779 music.musician.instruments_played 4349

2241 film.production_company.film 6790

2780 film.actor.film 8937

777 film.actor.film 8235

2005 music.artist.origin 4454

3198 film.person_or_entity_appearing_in_film.film 9297

1772 music.musician.instruments_played 4349

2861 film.actor.film 9187

2148 music.musician.instruments_played 4349

1498 music.artist.origin 5228

3390 film.person_or_entity_appearing_in_film.film 9298

3279 film.actor.film 3877

1962 film.person_or_entity_appearing_in_film.film 5312

3473 people.deceased_person.place_of_death 6736

2402 film.actor.film 9000

2635 film.actor.film 7580

2635 film.person_or_entity_appearing_in_film.film 7927

1893 film.actor.film 6255

598 film.film.genre 4062

1696 music.artist.origin 7554

623 film.actor.film 9152

1989 film.person_or_entity_appearing_in_film.film 4007

762 people.person.place_of_birth 4362

3210 people.person.place_of_birth 5019

1153 film.actor.film 9119

1153 film.person_or_entity_appearing_in_film.film 6805

3788 film.person_or_entity_appearing_in_film.film 6511

2017 film.person_or_entity_appearing_in_film.film 5766

2337 film.person_or_entity_appearing_in_film.film 5078

2998 film.actor.film 4327

3598 film.person_or_entity_appearing_in_film.film 4666

893 film.person_or_entity_appearing_in_film.film 9136

3498 film.actor.film 5193

672 film.person_or_entity_appearing_in_film.film 7371

3244 film.person_or_entity_appearing_in_film.film 8412

3593 music.artist.origin 7194

70 film.person_or_entity_appearing_in_film.film 5068

2481 film.person_or_entity_appearing_in_film.film 7168

3821	film.actor.film	8749

2070	film.actor.film	9299

2070	film.actor.film	8868

1211	film.actor.film	9029

1299	film.actor.film	6841

484 film.person_or_entity_appearing_in_film.film	4840

3738	cvg.publisher.games_published	9300

2571	games.game.publisher	9301

462 film.actor.film	7454

1857	music.artist.origin	4150

3297	film.actor.film	9138

2487	film.person_or_entity_appearing_in_film.film	8649

998 music.artist.origin	4813

2112	film.actor.film	9302

1767	film.actor.film	6237

3031	film.actor.film	9303

2390	film.actor.film	5385

1806	film.actor.film	7014

1806	film.actor.film	6757

2932	music.artist.origin	3859

434 film.actor.film	7731

434 film.person_or_entity_appearing_in_film.film	4045

538 film.person_or_entity_appearing_in_film.film	7264

3772	film.film.genre	6189

1660	film.actor.film	9304

1046	film.producer.film	7831

2587	music.artist.origin	4362

3390	film.actor.film	9298

3390	film.person_or_entity_appearing_in_film.film	8570

2546	film.actor.film	7711

2711 film.actor.film 8849

1348 film.person_or_entity_appearing_in_film.film 7080

241 film.person_or_entity_appearing_in_film.film 9253

1168 music.artist.origin 3872

2538 music.artist.origin 4087

1358 film.person_or_entity_appearing_in_film.film 9305

1372 film.actor.film 6426

2402 film.actor.film 9306

1557 music.artist.origin 4094

410 film.person_or_entity_appearing_in_film.film 8925

191 music.artist.origin 4294

548 film.person_or_entity_appearing_in_film.film 9190

623 film.actor.film 9206

155 people.person.place_of_birth 4123

1505 film.actor.film 6804

67 film.person_or_entity_appearing_in_film.film 5314

2734 people.person.place_of_birth 6343

1921 film.actor.film 7185

3558 music.artist.origin 3972

2337 film.producer.film 6771

3040 film.person_or_entity_appearing_in_film.film 7867

1711 film.actor.film 4213

2080 film.actor.film 5798

2080 film.person_or_entity_appearing_in_film.film 5062

1035 film.actor.film 5494

789 film.person_or_entity_appearing_in_film.film 9055

903 music.artist.album 5291

3401 people.person.place_of_birth 6209

2848 film.film.language 9140

80 film.actor.film 9307

361 award.nominee.award_nominations 9295

2913 film.person_or_entity_appearing_in_film.film 5805

1418 people.person.place_of_birth 4693

967 music.artist.album 8296

628 film.actor.film 9308

2688 film.actor.film 7378

1355 film.actor.film 8506

2689 type.object.key 2689

3738 cvg.publisher.games_published 4084

1154 film.actor.film 7569

1446 music.artist.origin 4391

901 award.competitor.awards_won 8269

901 film.actor.film 8568

625 film.person_or_entity_appearing_in_film.film 4483

3212 people.person.place_of_birth 9162

1249 film.person_or_entity_appearing_in_film.film 4777

3202 people.deceased_person.place_of_death 9242

2360 film.person_or_entity_appearing_in_film.film 6258

2241 film.distributor.film 7288

2241 film.distributor.film 8422

2241 film.production_company.film 7617

490 people.person.place_of_birth 4217

481 film.actor.film 9309

682 film.actor.film 6231

419 film.actor.film 4494

2534 film.actor.film 4033

2806 film.person_or_entity_appearing_in_film.film 8234

2487 film.person_or_entity_appearing_in_film.film 9310

3583 film.actor.film 5990

356 music.artist.track 9176

2038	film.actor.film	4095
956 film.person_or_entity_appearing_in_film.film	7202
3486	film.actor.film	6925
2810	people.person.place_of_birth	4094
1051	film.person_or_entity_appearing_in_film.film	7983
3321	film.actor.film	9228
3321	film.person_or_entity_appearing_in_film.film	4698
157 award.winner.awards_won	9311
3231	people.person.place_of_birth	4195
1619	film.person_or_entity_appearing_in_film.film	9252
1345	music.musician.instruments_played	4349
132 music.artist.origin	3864
3132	film.person_or_entity_appearing_in_film.film	6018
1479	film.person_or_entity_appearing_in_film.film	3878
3446	film.person_or_entity_appearing_in_film.film	5002
1507	music.artist.origin	3864
1034	film.actor.film	5485
735 event.agent.performance	4898
2635	film.person_or_entity_appearing_in_film.film	7211
2299	music.artist.origin	8962
2169	music.artist.origin	7124
67	film.person_or_entity_appearing_in_film.film	9312
861 music.artist.origin	4362
1193	people.person.place_of_birth	4552
1024	film.actor.film	4922
2338	film.actor.film	6259
386 film.actor.film	4335
2914	film.actor.film	7280
2914	film.person_or_entity_appearing_in_film.film	6135
1888	film.actor.film	8077

1453 film.actor.film 7667

1601 film.actor.film 8059

974 film.person_or_entity_appearing_in_film.film 7223

3561 film.person_or_entity_appearing_in_film.film 5674

2636 film.person_or_entity_appearing_in_film.film 9256

2636 film.person_or_entity_appearing_in_film.film 8766

2481 film.actor.film 4612

144 film.person_or_entity_appearing_in_film.film 7193

2475 film.actor.film 9313

1211 film.person_or_entity_appearing_in_film.film 9165

2815 music.artist.origin 6736

79 film.actor.film 9314

278 music.musician.instruments_played 4349

484 film.person_or_entity_appearing_in_film.film 5369

3738 cvg.publisher.games_published 9120

1154 film.person_or_entity_appearing_in_film.film 6224

625 film.actor.film 7594

2863 music.artist.origin 7820

1015 film.actor.film 6376

2241 film.distributor.film 9315

2241 film.production_company.film 8002

2241 film.production_company.film 9114

681 film.actor.film 4837

1275 music.artist.origin 4454

1262 music.artist.origin 4999

3751 music.artist.origin 4456

894 film.actor.film 8803

3354 award.competitor.award_nominations 5510

3354 film.person_or_entity_appearing_in_film.film 9268

1656 film.actor.film 8782

2272	film.actor.film	5302
534 film.actor.film	7550
1806	film.actor.film	6281
1806	film.person_or_entity_appearing_in_film.film	6989
2741	film.person_or_entity_appearing_in_film.film	8287
2100	film.actor.film	8741
1278	award.competitor.award_nominations	9316
434 film.actor.film	8918
538 film.actor.film	4995
3321	film.actor.film	7603
3321	film.person_or_entity_appearing_in_film.film	4420
2064	music.artist.origin	4825
2843	film.actor.film	6679
1387	film.person_or_entity_appearing_in_film.film	9265
1028	music.artist.origin	4852
2711	film.person_or_entity_appearing_in_film.film	7905
2711	film.person_or_entity_appearing_in_film.film	9129
1962	film.actor.film	7059
1962	film.actor.film	4802
2252	film.actor.film	9231
1259	film.person_or_entity_appearing_in_film.film	5786
1952	film.actor.film	7692
206 film.person_or_entity_appearing_in_film.film	5978
1282	music.artist.origin	9183
552 music.artist.origin	7944
570 music.artist.origin	7824
1633	film.actor.film	8352
1633	people.person.place_of_birth	5662
3827	film.actor.film	8914
3182	film.actor.film	7155

3788 film.person_or_entity_appearing_in_film.film 5881

2359 film.person_or_entity_appearing_in_film.film 7031

2370 film.person_or_entity_appearing_in_film.film 8954

1325 film.actor.film 4792

3700 people.person.place_of_birth 4391

1539 music.artist.origin 3966

3215 film.film.genre 4062

2998 film.actor.film 8809

1200 music.artist.origin 7433

925 music.artist.origin 4502

2080 film.person_or_entity_appearing_in_film.film 4721

3598 film.actor.film 9075

1296 film.person_or_entity_appearing_in_film.film 8777

789 film.actor.film 8206

3344 film.actor.film 8284

948 film.actor.film 9262

672 film.actor.film 9200

2913 film.person_or_entity_appearing_in_film.film 9317

736 film.actor.film 5847

1253 music.artist.origin 5064

458 music.artist.album 9318

646 film.actor.film 9193

3361 music.artist.origin 5977

3627 music.artist.origin 4057

2892 film.person_or_entity_appearing_in_film.film 6332

431 music.artist.origin 4977

2636 film.person_or_entity_appearing_in_film.film 7493

1403 film.person_or_entity_appearing_in_film.film 8843

1353 film.actor.film 8968

79 film.actor.film 5031

1502	film.actor.film	7533

484 film.person_or_entity_appearing_in_film.film	5854

1981	film.person_or_entity_appearing_in_film.film	8419

3738	cvg.publisher.games_published	5464

3738	games.publisher.games_published 4083

901 film.person_or_entity_appearing_in_film.film	4395

2241	film.distributor.film	6108

2241	film.production_company.film	4401

2241	film.production_company.film	7200

2571	cvg.computer_videogame.publisher	9301

3486	film.actor.film	6784

3546	film.actor.film	8513

1475	film.actor.film	4569

253 film.person_or_entity_appearing_in_film.film	6011

3670	film.person_or_entity_appearing_in_film.film	5515

3509	film.actor.film	9319

3321	film.actor.film	9188

3390	film.actor.film	9320

2711	film.actor.film	7298

2010	film.actor.film	9321

3132	film.person_or_entity_appearing_in_film.film	8403

2777	music.artist.origin	3846

2584	film.actor.film	5311

2684	film.person_or_entity_appearing_in_film.film	7877

206 film.actor.film	6355

2107	film.person_or_entity_appearing_in_film.film	6997

1157	film.person_or_entity_appearing_in_film.film	9322

1719	film.actor.film	7928

3661	film.person_or_entity_appearing_in_film.film	7670

2446	music.artist.origin	4852

1505 film.person_or_entity_appearing_in_film.film 9061

2094 film.person_or_entity_appearing_in_film.film 8457

2889 music.artist.origin 5125

952 people.person.place_of_birth 4877

1473 film.actor.film 6414

1473 film.person_or_entity_appearing_in_film.film 4014

836 music.artist.origin 4150

2087 music.artist.origin 4195

1193 film.person_or_entity_appearing_in_film.film 7869

3215 film.film.country 3961

2398 event.agent.performance 4387

1845 film.person_or_entity_appearing_in_film.film 4126

2080 film.actor.film 6631

30 film.actor.film 6696

2890 film.person_or_entity_appearing_in_film.film 3905

2439 music.artist.origin 5503

903 music.artist.album 5996

1005 people.person.place_of_birth 3864

77 people.person.place_of_birth 4723

3387 music.artist.origin 4768

2562 people.person.place_of_birth 4057

1601 film.actor.film 8977

187 event.agent.performance 5732

3810 music.artist.origin 5238

2636 film.actor.film 7192

2636 film.person_or_entity_appearing_in_film.film 7845

1211 music.artist.album 9323

2122 film.writer.film 7282

270 film.person_or_entity_appearing_in_film.film 7614

1307 film.person_or_entity_appearing_in_film.film 8933

484 film.actor.film 5425

484 film.person_or_entity_appearing_in_film.film 6506

3738 games.publisher.games_published 8000

3738 games.publisher.games_published 7568

3452 film.person_or_entity_appearing_in_film.film 6510

3452 film.person_or_entity_appearing_in_film.film 8871

901 film.person_or_entity_appearing_in_film.film 9324

3697 music.artist.origin 4232

3834 film.person_or_entity_appearing_in_film.film 9325

1359 film.actor.film 7286

1801 music.artist.origin 7804

2241 film.distributor.film 6984

2241 film.production_company.film 9113

3139 film.person_or_entity_appearing_in_film.film 8770

2935 music.musician.instruments_played 4349

1762 film.person_or_entity_appearing_in_film.film 7707

2487 film.person_or_entity_appearing_in_film.film 5824

778 music.musician.instruments_played 4349

3486 film.person_or_entity_appearing_in_film.film 4096

1938 people.person.place_of_birth 4057

3049 film.person_or_entity_appearing_in_film.film 8973

3435 music.artist.origin 5945

3130 music.artist.origin 6343

3390 film.person_or_entity_appearing_in_film.film 9320

3648 film.person_or_entity_appearing_in_film.film 8651

3316 film.producer.film 6574

3628 games.game.publisher 4700

2296 film.actor.film 5000

3082 film.film.language 9098

383 film.person_or_entity_appearing_in_film.film 5788

3599	film.person_or_entity_appearing_in_film.film	9044

552 film.person_or_entity_appearing_in_film.film	8683

567 film.actor.film	9326

2528	film.actor.film	8608

1349	film.actor.film	6593

2161	award.competitor.award_nominations	7431

2670	film.person_or_entity_appearing_in_film.film	8392

3582	film.person_or_entity_appearing_in_film.film	3960

3247	music.artist.origin	4057

239 award.nominee.award_nominations	9267

3282	film.actor.film	9226

948 film.actor.film	4073

3498	film.person_or_entity_appearing_in_film.film	4735

1393	film.person_or_entity_appearing_in_film.film	9263

646 film.actor.film	6635

3678	games.game.publisher	4727

2636	film.actor.film	8878

3821	film.actor.film	8657

49	film.actor.film	7440

715 people.person.place_of_birth	4310

3041	music.artist.origin	7043

256 music.album.genre	7810

1011	people.person.place_of_birth	8438

1694	film.person_or_entity_appearing_in_film.film	5331

119 music.artist.origin	4171

2867	film.actor.film	8096

2241	film.distributor.film	7961

2241	film.production_company.film	5902

2241	film.production_company.film	8986

2241	film.production_company.film	4348

2241 film.production_company.film 7700

2380 film.person_or_entity_appearing_in_film.film 5162

599 film.actor.film 8467

777 film.actor.film 4943

998 film.actor.film 8497

1056 music.artist.origin 4272

1806 film.actor.film 8511

629 film.writer.film 4257

1992 music.artist.origin 4635

3321 film.actor.film 4152

1310 film.person_or_entity_appearing_in_film.film 7526

1597 film.actor.film 5518

2999 film.person_or_entity_appearing_in_film.film 7931

1474 film.person_or_entity_appearing_in_film.film 8146

3256 film.person_or_entity_appearing_in_film.film 6792

1816 film.person_or_entity_appearing_in_film.film 8552

1632 film.person_or_entity_appearing_in_film.film 6287

916 film.actor.film 8472

3132 film.person_or_entity_appearing_in_film.film 8774

1025 film.person_or_entity_appearing_in_film.film 5979

3551 film.person_or_entity_appearing_in_film.film 8807

1057 film.person_or_entity_appearing_in_film.film 4002

2268 music.artist.origin 4957

68 people.person.place_of_birth 5411

1726 film.person_or_entity_appearing_in_film.film 5162

2107 film.person_or_entity_appearing_in_film.film 9254

2635 film.actor.film 8746

2635 film.person_or_entity_appearing_in_film.film 8262

2090 music.musician.instruments_played 5802

3213 people.person.profession 5021

1633 film.person_or_entity_appearing_in_film.film 8764

598 film.film.country 4454

410 film.person_or_entity_appearing_in_film.film 8379

2161 award.competitor.award_nominations 9223

2735 music.musician.instruments_played 3851

623 film.person_or_entity_appearing_in_film.film 9327

307 music.artist.origin 4058

1250 music.artist.origin 4175

12 film.person_or_entity_appearing_in_film.film 9045

2337 film.actor.film 7306

189 film.actor.film 9171

895 film.person_or_entity_appearing_in_film.film 4964

1706 film.person_or_entity_appearing_in_film.film 5803

145 film.person_or_entity_appearing_in_film.film 6973

279 film.person_or_entity_appearing_in_film.film 7561

645 film.actor.film 9214

3295 film.actor.film 8965

903 people.person.place_of_birth 6215

2214 music.artist.track 8855

3498 film.actor.film 7134

3498 film.person_or_entity_appearing_in_film.film 4128

2830 film.actor.film 5580

70 film.actor.film 8505

437 film.actor.film 7374

3385 music.artist.origin 4183

1570 music.artist.origin 4195

3738 cvg.publisher.games_published 8958

3738 cvg.publisher.games_published 8137

436 film.person_or_entity_appearing_in_film.film 6815

1853 music.artist.origin 3966

1049	music.artist.origin	4175

432 film.actor.film	5380

2577	music.musician.instruments_played	4349

2631	film.person_or_entity_appearing_in_film.film	4622

2241	film.distributor.film	6610

2241	film.production_company.film	8780

2241	film.production_company.film	7287

2629	music.artist.album	9103

3003	film.person_or_entity_appearing_in_film.film	4168

956 film.actor.film	8781

2067	music.artist.origin	6948

2275	film.actor.film	5714

2678	music.artist.album	8794

2677	film.actor.film	4786

3487	music.musician.instruments_played	6942

259 music.artist.origin	3864

3374	music.artist.origin	8453

740 film.person_or_entity_appearing_in_film.film	7527

3433	film.actor.film	6353

2980	people.person.place_of_birth	3855

3650	film.person_or_entity_appearing_in_film.film	8604

3390	film.person_or_entity_appearing_in_film.film	7427

1474	film.person_or_entity_appearing_in_film.film	7886

2711	film.person_or_entity_appearing_in_film.film	8348

2393	people.person.place_of_birth	6261

448 film.person_or_entity_appearing_in_film.film	5482

2659	music.artist.origin	4183

1776	film.actor.film	5000

968 music.artist.origin	4768

2388	people.deceased_person.place_of_death	6200

1871 music.artist.origin 4876

1157 film.writer.film 5755

3384 film.actor.film 5789

3384 film.person_or_entity_appearing_in_film.film 4955

2161 award.nominee.award_nominations 9118

1720 film.producer.film 4860

1518 music.artist.origin 6331

374 film.actor.film 9003

3827 film.person_or_entity_appearing_in_film.film 9261

1153 film.person_or_entity_appearing_in_film.film 7275

3020 film.actor.film 9133

3788 film.person_or_entity_appearing_in_film.film 8518

67 film.person_or_entity_appearing_in_film.film 9153

2733 film.actor.film 8056

2125 film.person_or_entity_appearing_in_film.film 8562

3061 event.agent.performance 4099

1692 people.person.place_of_birth 3947

91 film.actor.film 5452

127 film.person_or_entity_appearing_in_film.film 7700

84 film.person_or_entity_appearing_in_film.film 7368

728 music.artist.origin 5390

1640 music.artist.origin 4852

386 film.actor.film 8208

3414 music.artist.origin 4174

80 film.person_or_entity_appearing_in_film.film 9307

2081 people.deceased_person.place_of_death 3947

2914 film.person_or_entity_appearing_in_film.film 7537

646 film.actor.film 9280

628 film.actor.film 6136

408 film.actor.film 9328

2095 film.person_or_entity_appearing_in_film.film 7316

49 film.actor.film 6218

2460 film.actor.film 3921

79 film.person_or_entity_appearing_in_film.film 7895

270 tv.actor.starring_roles 8892

1579 film.actor.film 5588

1315 film.actor.film 8318

484 film.person_or_entity_appearing_in_film.film 5994

3248 award.winner.awards_won 5431

2867 film.actor.film 8857

1015 music.artist.origin 4999

3124 music.artist.origin 4150

2241 film.distributor.film 9329

2241 film.production_company.film 7650

2203 people.person.place_of_birth 8882

2078 film.actor.film 4132

387 film.actor.film 8848

2200 film.person_or_entity_appearing_in_film.film 4740

1399 film.person_or_entity_appearing_in_film.film 5644

1357 film.actor.film 4142

434 film.person_or_entity_appearing_in_film.film 9330

3509 film.person_or_entity_appearing_in_film.film 4747

1660 film.person_or_entity_appearing_in_film.film 4073

1619 film.actor.film 8274

848 film.person_or_entity_appearing_in_film.film 9331

1387 film.actor.film 9072

2630 film.person_or_entity_appearing_in_film.film 6470

2010 film.person_or_entity_appearing_in_film.film 9321

1233 film.person_or_entity_appearing_in_film.film 6192

1866 film.person_or_entity_appearing_in_film.film 8743

2595	film.person_or_entity_appearing_in_film.film	9105

3437	music.artist.origin	4919

1142	film.person_or_entity_appearing_in_film.film	8088

2528	film.person_or_entity_appearing_in_film.film	7880

1719	film.actor.film	4907

2695	film.person_or_entity_appearing_in_film.film	8924

819 people.deceased_person.place_of_death	4310

3638	music.artist.origin	4040

224 music.artist.origin	3916

2763	people.deceased_person.place_of_death	4013

2141	film.actor.film	8055

912 film.person_or_entity_appearing_in_film.film	5694

1286	music.artist.origin	3849

3548	film.person_or_entity_appearing_in_film.film	4802

1473	film.person_or_entity_appearing_in_film.film	9074

1141	film.actor.film	7739

895 film.actor.film	5450

127 film.person_or_entity_appearing_in_film.film	5985

2271	music.musician.instruments_played	4426

3598	film.actor.film	9255

508 film.person_or_entity_appearing_in_film.film	3976

3236	music.artist.origin	6509

70	film.person_or_entity_appearing_in_film.film	4867

2636	film.actor.film	9201

294 film.director.film	7317

3738	cvg.publisher.games_published	5897

3738	games.publisher.games_published	8398

3738	games.publisher.games_published	6608

436 film.person_or_entity_appearing_in_film.film	5956

1248	event.agent.performance	8048

2241 film.distributor.film 9083

2241 film.distributor.film 9290

1484 music.artist.origin 3850

3615 music.artist.origin 4191

538 film.actor.film 8632

1378 film.actor.film 6678

1783 film.actor.film 9210

1149 music.artist.origin 4003

2211 film.actor.film 5990

3390 film.actor.film 7913

2010 film.person_or_entity_appearing_in_film.film 6079

1316 music.artist.origin 5662

1067 film.actor.film 6934

2298 film.actor.film 9024

3599 film.actor.film 6118

567 film.actor.film 8808

1076 music.artist.origin 7634

2635 film.person_or_entity_appearing_in_film.film 4467

2635 film.person_or_entity_appearing_in_film.film 9332

3307 film.person_or_entity_appearing_in_film.film 6051

1157 film.person_or_entity_appearing_in_film.film 8343

379 film.person_or_entity_appearing_in_film.film 4826

2418 film.actor.film 8488

222 music.artist.origin 5390

3106 people.person.place_of_birth 9333

3629 people.person.place_of_birth 4232

2953 film.person_or_entity_appearing_in_film.film 4051

67 film.person_or_entity_appearing_in_film.film 4817

1473 film.person_or_entity_appearing_in_film.film 5316

1982 film.actor.film 3905

2619	film.person_or_entity_appearing_in_film.film	5193

850 film.actor.film	6217

91	film.actor.film	8490

3598	film.person_or_entity_appearing_in_film.film	8854

3072	music.artist.origin	4825

752 music.artist.origin	4013

2562	film.actor.film	8821

2562	film.actor.film	8226

408 film.actor.film	4452

2781	film.person_or_entity_appearing_in_film.film	4136

1355	film.actor.film	9081

970 music.artist.origin	4174

1229	film.person_or_entity_appearing_in_film.film	5454

2689	event.agent.performance	4898

1299	music.artist.origin	4445

399 film.actor.film	5769

2246	film.person_or_entity_appearing_in_film.film	7406

484 film.actor.film	3924

484 film.actor.film	9281

484 film.person_or_entity_appearing_in_film.film	9289

3738	cvg.publisher.games_published	5927

3738	cvg.publisher.games_published	4295

2639	music.artist.origin	4362

1456	music.artist.origin	4502

949 film.actor.film	8674

300 music.artist.origin	4852

442 award.competitor.award_nominations	8616

2241	film.distributor.film	8508

2241	film.distributor.film	7231

2241	film.production_company.film	9049

2241 film.production_company.film 4141

2241 film.production_company.film 9158

2241 film.production_company.film 9329

1464 film.person_or_entity_appearing_in_film.film 8972

3297 film.actor.film 5193

1583 film.actor.film 7602

1768 film.person_or_entity_appearing_in_film.film 9251

2206 film.person_or_entity_appearing_in_film.film 7449

439 music.artist.origin 6555

1217 music.artist.origin 4876

2015 film.actor.film 5716

1475 film.actor.film 9186

1417 film.actor.film 8912

1336 music.artist.origin 3864

1926 people.person.place_of_birth 4217

521 film.actor.film 8086

3509 film.person_or_entity_appearing_in_film.film 5645

2909 people.person.place_of_birth 5698

3049 film.actor.film 9236

2244 film.film.actor 5048

3474 film.actor.film 7481

2489 music.artist.origin 4057

3070 people.person.place_of_birth 6215

2452 film.actor.film 7876

2007 people.person.place_of_birth 3855

2584 film.actor.film 9285

465 music.artist.album 9334

2449 film.person_or_entity_appearing_in_film.film 6006

3722 music.musician.instruments_played 3851

206 film.person_or_entity_appearing_in_film.film 5196

997 film.person_or_entity_appearing_in_film.film 7711

2729 music.artist.origin 9013

552 film.actor.film 8988

2635 film.actor.film 8310

2635 film.director.film 6939

2378 award.competitor.award_nominations 7694

2133 music.artist.origin 4868

2507 music.artist.track 9335

3126 music.artist.album 6473

2161 award.nominee.award_nominations 8699

1720 film.actor.film 6084

2371 music.artist.origin 6636

1153 film.person_or_entity_appearing_in_film.film 7974

1383 film.actor.film 7738

3792 film.actor.film 6162

1155 people.person.place_of_birth 4927

16 film.person_or_entity_appearing_in_film.film 8716

145 film.person_or_entity_appearing_in_film.film 4136

2737 film.actor.film 7740

1973 film.actor.film 6099

1791 music.artist.origin 4635

2913 film.person_or_entity_appearing_in_film.film 5022

2914 film.person_or_entity_appearing_in_film.film 7219

2914 film.person_or_entity_appearing_in_film.film 9296

70 film.person_or_entity_appearing_in_film.film 6214

862 film.actor.film 6655

2636 film.person_or_entity_appearing_in_film.film 8579

2437 film.actor.film 8417

1626 people.person.place_of_birth 4013

2846 music.artist.origin 4217

1353	film.person_or_entity_appearing_in_film.film	4079

79	film.person_or_entity_appearing_in_film.film	7999

79	film.person_or_entity_appearing_in_film.film	6954

2176	people.person.place_of_birth	7789

503	music.artist.origin	3864

1002	music.musician.instruments_played	3959

587	film.character.film	4840

2159	film.actor.film	4932

41	film.person_or_entity_appearing_in_film.film	7468

1314	people.person.place_of_birth	7851

2241	film.distributor.film	6312

2241	film.production_company.film	7198

2241	film.production_company.film	9315

2241	film.production_company.film	8045

988	film.actor.film	4823

1464	film.actor.film	9336

1055	people.person.place_of_birth	5211

135	film.actor.film	9337

1624	film.person_or_entity_appearing_in_film.film	4846

591	music.artist.album	9323

1806	film.person_or_entity_appearing_in_film.film	5089

170	music.artist.origin	7990

1971	music.artist.origin	4010

3049	film.actor.film	8680

3458	film.actor.film	4209

338	music.artist.origin	3885

3579	music.musician.instruments_played	4349

2556	film.actor.film	6087

3026	film.actor.film	5092

3390	film.person_or_entity_appearing_in_film.film	9338

3648	film.person_or_entity_appearing_in_film.film	7861

3065	music.musician.instruments_played	3959

638 film.actor.film	8952

2010	film.person_or_entity_appearing_in_film.film	8633

1962	film.actor.film	6712

1057	film.person_or_entity_appearing_in_film.film	8186

1658	music.artist.origin	6457

206 film.person_or_entity_appearing_in_film.film	4728

2942	music.artist.origin	4808

2635	film.actor.film	6938

1157	film.person_or_entity_appearing_in_film.film	9233

115 film.person_or_entity_appearing_in_film.film	7242

1633	film.actor.film	4371

2724	film.actor.film	4907

3413	music.artist.origin	4732

3389	music.artist.origin	3945

3020	film.actor.film	5313

1802	film.person_or_entity_appearing_in_film.film	5638

2370	film.actor.film	9292

1473	film.person_or_entity_appearing_in_film.film	9339

255 people.person.place_of_birth	3864

3529	people.person.place_of_birth	4010

1813	film.actor.film	8110

1043	music.musician.instruments_played	3959

2270	film.actor.film	7111

2457	music.artist.origin	8576

3371	people.deceased_person.place_of_death	4057

987 film.actor.film	7036

539 film.person_or_entity_appearing_in_film.film	5950

2080	film.actor.film	8344

1195	music.artist.origin	3864

70	film.person_or_entity_appearing_in_film.film	4399

2294	music.artist.origin	4094

1145	people.person.place_of_birth	8993

3738	cvg.publisher.games_published	8567

2967	film.person_or_entity_appearing_in_film.film	6625

1359	people.person.place_of_birth	4357

2867	film.person_or_entity_appearing_in_film.film	6866

2241	film.distributor.film	7598

2241	film.production_company.film	8028

2241	film.production_company.film	8581

481	film.person_or_entity_appearing_in_film.film	9340

3160	film.person_or_entity_appearing_in_film.film	9341

894	film.actor.film	9057

537	tv.program.genre	4579

2732	people.person.place_of_birth	8885

302	music.artist.origin	6012

1656	film.actor.film	9250

3486	film.person_or_entity_appearing_in_film.film	9259

3242	film.person_or_entity_appearing_in_film.film	5189

434	film.actor.film	8873

440	people.person.place_of_birth	4746

538	film.person_or_entity_appearing_in_film.film	6822

1660	film.person_or_entity_appearing_in_film.film	9078

3390	film.person_or_entity_appearing_in_film.film	9205

3648	film.actor.film	4210

3048	film.actor.film	7374

363	film.actor.film	9116

1952	film.actor.film	7055

1750	music.artist.origin	4362

2025 music.artist.origin 4454

1358 people.person.place_of_birth 4321

997 film.person_or_entity_appearing_in_film.film 9342

3384 film.person_or_entity_appearing_in_film.film 6159

115 film.actor.film 4543

1633 film.person_or_entity_appearing_in_film.film 8289

2633 film.actor.film 4956

3679 film.character.film 8650

410 film.person_or_entity_appearing_in_film.film 9343

155 film.actor.film 7340

2929 music.album.genre 8909

1891 film.actor.film 4976

3788 film.actor.film 7276

3788 film.person_or_entity_appearing_in_film.film 7883

67 film.actor.film 9312

760 music.artist.origin 6913

2359 film.person_or_entity_appearing_in_film.film 8169

38 music.artist.album 7075

2147 film.actor.film 7032

2284 music.artist.origin 4471

136 film.person_or_entity_appearing_in_film.film 5843

3784 film.director.film 3904

279 film.actor.film 8152

1625 film.person_or_entity_appearing_in_film.film 8702

3733 film.person_or_entity_appearing_in_film.film 8575

407 film.actor.film 9273

2214 film.person_or_entity_appearing_in_film.film 8093

2713 film.person_or_entity_appearing_in_film.film 5765

64 people.person.place_of_birth 4310

77 film.actor.film 8932

2913 film.actor.film 9317

510 people.person.place_of_birth 8207

1796 film.actor.film 6138

45 film.person_or_entity_appearing_in_film.film 4411

922 people.person.place_of_birth 3947

3683 music.artist.origin 5662

49 film.director.film 7440

2458 film.actor.film 4830

527 film.actor.film 7679

3738 cvg.publisher.games_published 9016

901 film.actor.film 8910

796 film.person_or_entity_appearing_in_film.film 9156

2241 film.distributor.film 7273

2241 film.distributor.film 7260

2241 film.distributor.film 8602

2241 film.distributor.film 7446

2241 film.production_company.film 8755

2241 film.production_company.film 5705

3706 film.person_or_entity_appearing_in_film.film 6897

1697 film.person_or_entity_appearing_in_film.film 4772

3031 film.actor.film 7679

2766 film.actor.film 8121

2522 music.artist.origin 4963

2531 film.person_or_entity_appearing_in_film.film 7576

538 film.actor.film 5973

3321 film.actor.film 7236

1398 film.person_or_entity_appearing_in_film.film 4911

3390 film.actor.film 9278

2855 music.artist.origin 4217

1057 film.actor.film 3949

2503 music.artist.origin 5481

206 film.person_or_entity_appearing_in_film.film 5955

567 film.person_or_entity_appearing_in_film.film 8571

2635 film.actor.film 8366

1157 film.actor.film 8589

1157 film.actor.film 8265

3194 film.actor.film 9286

1557 film.actor.film 5562

3384 film.person_or_entity_appearing_in_film.film 9107

115 film.person_or_entity_appearing_in_film.film 8278

115 film.person_or_entity_appearing_in_film.film 9344

2418 people.deceased_person.place_of_death 5523

845 people.person.place_of_birth 4094

639 people.person.place_of_birth 6261

307 film.actor.film 5572

878 music.artist.origin 8101

3404 film.person_or_entity_appearing_in_film.film 4225

1473 film.person_or_entity_appearing_in_film.film 7699

1473 film.person_or_entity_appearing_in_film.film 7158

793 music.artist.origin 4869

1692 film.actor.film 5884

1959 film.actor.film 6164

559 people.person.place_of_birth 4133

3733 film.actor.film 7562

2966 film.person_or_entity_appearing_in_film.film 8668

192 music.producer.tracks_produced 6366

377 film.actor.film 6462

407 film.person_or_entity_appearing_in_film.film 8022

3598 film.actor.film 9063

2214 music.artist.track 7310

2338 film.person_or_entity_appearing_in_film.film 4229

177 film.actor.film 7410

948 film.person_or_entity_appearing_in_film.film 4765

2913 film.actor.film 8504

3280 film.person_or_entity_appearing_in_film.film 8329

3820 music.artist.origin 7944

646 film.actor.film 8611

408 film.person_or_entity_appearing_in_film.film 9328

2892 people.person.place_of_birth 4044

2095 film.actor.film 7438

2317 people.deceased_person.place_of_death 5362

1256 music.artist.origin 4277

381 film.person_or_entity_appearing_in_film.film 6840

542 film.actor.film 8061

133 event.agent.performance 4035

2460 film.actor.film 9235

2682 film.actor.film 6892

2778 music.artist.album 6687

3738 cvg.publisher.games_published 4833

436 film.person_or_entity_appearing_in_film.film 7243

1154 film.actor.film 5542

901 film.person_or_entity_appearing_in_film.film 9111

2559 film.person_or_entity_appearing_in_film.film 9282

223 event.agent.performance 4603

949 film.writer.film 4029

2360 film.actor.film 7349

2241 film.distributor.film 8649

2241 film.distributor.film 9345

2241 film.distributor.film 8936

2241 film.production_company.film 5999

2241 film.production_company.film 9346

918 people.person.place_of_birth 9091

2487 film.actor.film 9310

1777 music.artist.origin 4057

1767 film.person_or_entity_appearing_in_film.film 7325

1432 music.artist.origin 3945

1373 music.artist.origin 5262

677 music.artist.origin 4380

1660 film.person_or_entity_appearing_in_film.film 8013

3049 film.person_or_entity_appearing_in_film.film 4260

814 film.person_or_entity_appearing_in_film.film 8304

972 music.artist.origin 4057

2556 film.actor.film 3931

1027 music.artist.origin 6343

3322 music.artist.origin 8549

3048 film.person_or_entity_appearing_in_film.film 6043

3544 film.actor.film 8920

1962 film.actor.film 7025

3599 film.person_or_entity_appearing_in_film.film 5445

1157 film.actor.film 9189

379 film.actor.film 7273

115 film.person_or_entity_appearing_in_film.film 8763

3 music.artist.origin 9347

1633 film.actor.film 6485

2498 music.artist.origin 4010

155 award.winner.awards_won 9004

3827 film.person_or_entity_appearing_in_film.film 8638

446 film.person_or_entity_appearing_in_film.film 8906

1153 film.actor.film 4484

1672 music.artist.album 9052

2050 film.writer.film 8653

1833 film.actor.film 8927

1715 music.artist.origin 4419

2079 film.actor.film 7836

3412 film.film.producer 8445

3412 film.film.writer 8445

2733 film.person_or_entity_appearing_in_film.film 5915

1473 film.actor.film 9348

1473 film.person_or_entity_appearing_in_film.film 9005

1473 film.person_or_entity_appearing_in_film.film 8280

1692 film.actor.film 7005

136 film.person_or_entity_appearing_in_film.film 8929

239 film.actor.film 5797

2705 film.person_or_entity_appearing_in_film.film 8624

789 film.actor.film 7087

893 film.person_or_entity_appearing_in_film.film 7745

2406 people.person.place_of_birth 6056

3797 people.deceased_person.place_of_death 4768

1520 people.person.place_of_birth 7640

1220 film.actor.film 9015

1220 film.director.film 5024

1795 music.artist.origin 3966

2535 film.actor.film 7591

2688 film.actor.film 4390

781 film.actor.film 7750

2778 film.actor.film 6221

3738 cvg.publisher.games_published 3925

3738 cvg.publisher.games_published 7899

3738 games.publisher.games_published 5252

3738 games.publisher.games_published 5222

1154 film.actor.film 9066

3248 film.person_or_entity_appearing_in_film.film 9257

2241 film.distributor.film 4298

2241 film.distributor.film 5225

2241 film.production_company.film 8196

2241 film.production_company.film 6415

2241 film.production_company.film 3215

777 film.actor.film 7423

1804 film.person_or_entity_appearing_in_film.film 7708

2126 film.actor.film 9126

1060 people.person.place_of_birth 4272

3221 people.person.place_of_birth 3846

833 music.artist.origin 4552

1580 film.actor.film 7822

2244 film.film.director5048

2399 film.person_or_entity_appearing_in_film.film 4124

3099 film.actor.film 6386

2715 film.person_or_entity_appearing_in_film.film 8620

3390 film.actor.film 6014

3390 film.actor.film 9338

482 music.artist.origin 4454

3428 people.person.place_of_birth 3986

2205 film.actor.film 5483

3251 film.actor.film 7579

3507 film.person_or_entity_appearing_in_film.film 6590

2833 film.person_or_entity_appearing_in_film.film 4328

2297 film.actor.film 9260

2635 film.actor.film 9332

2635 film.person_or_entity_appearing_in_film.film 7210

680 music.artist.origin 3864

1221 music.musician.instruments_played 8663

3239 music.artist.origin 5401

2161 award.competitor.award_nominations 7058

1720 film.writer.film 7244

3824 music.artist.origin 7804

2755 music.artist.origin 3859

623 film.person_or_entity_appearing_in_film.film 4006

1747 people.person.place_of_birth 4442

395 music.artist.album 7418

1833 film.actor.film 5057

878 event.agent.performance 8048

2359 film.actor.film 6258

1473 film.actor.film 9135

1687 music.artist.origin 4852

583 film.person_or_entity_appearing_in_film.film 9349

3598 film.person_or_entity_appearing_in_film.film 8295

376 people.person.place_of_birth 4310

3278 music.artist.origin 3866

1181 film.actor.film 9350

2914 film.person_or_entity_appearing_in_film.film 4672

2649 music.artist.origin 4635

2437 film.person_or_entity_appearing_in_film.film 9220

162 people.person.place_of_birth 4195

2070 film.person_or_entity_appearing_in_film.film 4875

3179 film.actor.film 9215

49 film.person_or_entity_appearing_in_film.film 8845

399 film.actor.film 4411

445 film.actor.film 9351

445 film.person_or_entity_appearing_in_film.film 8626

445 film.person_or_entity_appearing_in_film.film 9351

3738 games.publisher.games_published 9218

3738 games.publisher.games_published 9249

184 film.person_or_entity_appearing_in_film.film 8690

1154 film.person_or_entity_appearing_in_film.film 5031

1673 music.artist.origin 5127

2867 film.actor.film 7635

2241 film.distributor.film 5745

2241 film.production_company.film 9185

2241 film.production_company.film 7351

2532 music.artist.origin 9333

998 film.person_or_entity_appearing_in_film.film 7324

3789 film.person_or_entity_appearing_in_film.film 7779

2200 film.person_or_entity_appearing_in_film.film 7772

643 film.person_or_entity_appearing_in_film.film 9227

3433 film.person_or_entity_appearing_in_film.film 6902

1089 people.deceased_person.place_of_death 9076

1543 music.artist.origin 3864

1323 film.person_or_entity_appearing_in_film.film 9181

2601 music.artist.origin 4199

2449 people.person.place_of_birth 3859

1420 music.artist.origin 5845

507 music.artist.origin 4195

1907 music.artist.origin 4456

1259 film.actor.film 6024

2223 film.person_or_entity_appearing_in_film.film 5522

2997 people.person.place_of_birth 4419

1157 film.person_or_entity_appearing_in_film.film 4052

3194 film.actor.film 4810

115 film.actor.film 6394

410 film.actor.film 8456

623 film.actor.film 9327

2084 film.actor.film 4812

3035 film.person_or_entity_appearing_in_film.film 8875

1672 music.artist.album 7545

1721 music.artist.origin 3942

3073 music.artist.origin 6797

3651 music.artist.origin 4183

136 film.actor.film 7033

930 music.artist.origin 3897

192 music.producer.tracks_produced 8039

2061 music.artist.origin 4852

3121 film.person_or_entity_appearing_in_film.film 5801

3344 people.person.place_of_birth 6722

3704 music.artist.origin 8682

77 film.person_or_entity_appearing_in_film.film 4870

809 film.actor.film 7883

2913 film.actor.film 7511

675 film.person_or_entity_appearing_in_film.film 4168

2541 people.deceased_person.place_of_death 4768

3756 music.artist.origin 5106

2070 film.actor.film 8060

3745 film.person_or_entity_appearing_in_film.film 7540

1573 film.person_or_entity_appearing_in_film.film 8346

289 music.artist.origin 3946

3738 games.publisher.games_published 9065

1808 film.actor.film 9221

3309 music.artist.origin 4362

3787 film.actor.film 4620

2159 film.person_or_entity_appearing_in_film.film 9157

2867 film.actor.film 9352

2645	music.artist.origin	9031
2241	film.distributor.film	5409
2241	film.distributor.film	9204
2241	film.production_company.film	4403
1361	film.person_or_entity_appearing_in_film.film	6784
2985	music.artist.origin	4057
3761	people.person.place_of_birth	9077
1624	film.person_or_entity_appearing_in_film.film	6820
1804	music.artist.origin	4008
1832	music.artist.origin	3947
2078	film.person_or_entity_appearing_in_film.film	4476
2260	film.actor.film	8771
2275	film.person_or_entity_appearing_in_film.film	5786
3674	music.artist.origin	5270
2206	film.actor.film	9283
1806	film.actor.film	6282
3252	music.artist.origin	4057
1475	film.person_or_entity_appearing_in_film.film	4569
3000	film.person_or_entity_appearing_in_film.film	9071
3509	film.person_or_entity_appearing_in_film.film	9319
1660	film.person_or_entity_appearing_in_film.film	9304
3049	film.person_or_entity_appearing_in_film.film	8427
3321	film.actor.film	3868
921 music.artist.origin	4195
2372	film.actor.film	5144
3390	film.actor.film	8145
2296	film.person_or_entity_appearing_in_film.film	4209
2010	film.actor.film	6711
1866	music.artist.origin	3961
1132	music.artist.origin	3961

2130	people.deceased_person.place_of_death	9192

2419	film.actor.film	5651

997	film.actor.film	9342

567	film.actor.film	9222

2635	film.person_or_entity_appearing_in_film.film	7693

1351	film.person_or_entity_appearing_in_film.film	9053

410	film.person_or_entity_appearing_in_film.film	4316

1653	film.actor.film	7715

190	music.artist.origin	4108

3317	film.person_or_entity_appearing_in_film.film	5570

2953	music.artist.origin	5275

2963	film.person_or_entity_appearing_in_film.film	9025

633	film.actor.film	5154

1473	film.actor.film	8666

2465	music.artist.origin	4963

3391	people.person.place_of_birth	5916

136	film.actor.film	9353

1412	film.actor.film	6570

2737	film.person_or_entity_appearing_in_film.film	6167

645	film.actor.film	7769

3295	film.person_or_entity_appearing_in_film.film	7435

583	film.actor.film	8717

2080	film.actor.film	7165

789	people.person.place_of_birth	4369

1178	music.artist.origin	6555

52	film.person_or_entity_appearing_in_film.film	7780

2834	music.artist.origin	4471

3676	film.person_or_entity_appearing_in_film.film	9354

2830	film.person_or_entity_appearing_in_film.film	9264

357	film.person_or_entity_appearing_in_film.film	9275

2562 film.actor.film 8732

447 award.nominee.award_nominations 7702

1947 music.artist.origin 3897

2070 film.actor.film 8209

1173 people.person.place_of_birth 4057

3738 cvg.publisher.games_published 7518

184 people.person.place_of_birth 8101

3248 film.actor.film 8546

2241 film.distributor.film 7902

2241 film.distributor.film 5136

2241 film.distributor.film 4988

2241 film.production_company.film 6786

2241 film.production_company.film 8880

2241 film.production_company.film 8629

988 film.person_or_entity_appearing_in_film.film 4939

1873 film.person_or_entity_appearing_in_film.film 4515

481 film.person_or_entity_appearing_in_film.film 9309

135 film.person_or_entity_appearing_in_film.film 9337

3296 film.person_or_entity_appearing_in_film.film 7687

894 film.actor.film 7149

2291 music.artist.origin 4963

3198 film.actor.film 9297

2098 music.musician.instruments_played 9355

341 film.film.director7174

1475 film.actor.film 8911

3283 music.artist.origin 4057

538 film.actor.film 8363

3772 film.film.genre 3981

405 music.artist.origin 4380

3281 music.artist.origin 4471

2630 film.actor.film 9356

1147 music.artist.origin 4454

166 film.actor.film 8326

3648 film.person_or_entity_appearing_in_film.film 5375

3648 people.person.place_of_birth 4195

2010 film.person_or_entity_appearing_in_film.film 5444

1260 film.person_or_entity_appearing_in_film.film 5416

1962 film.actor.film 8697

1709 film.actor.film 5323

968 music.artist.origin 4024

1291 film.person_or_entity_appearing_in_film.film 9212

2297 music.artist.origin 4232

567 film.person_or_entity_appearing_in_film.film 9326

2107 film.person_or_entity_appearing_in_film.film 7154

1719 film.person_or_entity_appearing_in_film.film 6458

3839 music.artist.origin 4013

1748 film.actor.film 7582

1727 music.artist.origin 3961

1525 film.person_or_entity_appearing_in_film.film 5277

67 film.actor.film 5060

117 people.person.place_of_birth 6564

1473 film.person_or_entity_appearing_in_film.film 7866

1473 film.person_or_entity_appearing_in_film.film 9035

1745 music.artist.origin 3849

793 film.actor.film 4281

2018 event.agent.performance 4770

3040 film.actor.film 8730

3040 film.person_or_entity_appearing_in_film.film 7698

413 film.person_or_entity_appearing_in_film.film 7434

3336 music.artist.origin 5390

109 music.artist.origin 4013

3094 film.actor.film 8976

2059 music.artist.origin 4442

2784 film.person_or_entity_appearing_in_film.film 4786

2914 film.person_or_entity_appearing_in_film.film 8224

3676 film.actor.film 9354

3355 film.person_or_entity_appearing_in_film.film 6637

2968 people.person.place_of_birth 5362

2460 film.person_or_entity_appearing_in_film.film 7938

1502 music.artist.origin 3961

715 film.actor.film 8158

2000 music.artist.origin 8946

3738 cvg.publisher.games_published 7897

3738 cvg.publisher.games_published 8063

3738 cvg.publisher.games_published 9357

3738 games.publisher.games_published 8673

436 film.person_or_entity_appearing_in_film.film 8212

3383 people.person.place_of_birth 4768

2241 film.distributor.film 4937

1903 music.musician.instruments_played 3959

481 film.person_or_entity_appearing_in_film.film 7290

3181 music.artist.origin 3864

1165 music.artist.origin 4024

2487 film.actor.film 8526

1618 film.person_or_entity_appearing_in_film.film 5532

998 film.actor.film 6450

356 music.artist.origin 4453

1996 music.artist.origin 4471

3487 music.musician.instruments_played 9355

1346 film.actor.film 4021

538 film.actor.film 9084

951 type.object.subject_key 951

1565 music.artist.origin 8202

3557 film.film.genre 4410

1457 music.artist.origin 4272

2754 music.artist.origin 4391

2274 music.artist.origin 7103

2630 film.person_or_entity_appearing_in_film.film 9356

1632 film.person_or_entity_appearing_in_film.film 5928

2290 music.artist.origin 5105

2023 music.artist.origin 4852

2885 people.deceased_person.place_of_death 6056

1481 music.artist.origin 4077

1650 music.artist.origin 4391

27 music.artist.origin 4416

2402 film.person_or_entity_appearing_in_film.film 9306

1365 film.actor.film 8454

981 film.actor.film 4234

2635 film.actor.film 4467

2635 film.actor.film 8904

2635 film.person_or_entity_appearing_in_film.film 5505

3818 music.artist.origin 4416

3493 music.artist.origin 5421

115 film.person_or_entity_appearing_in_film.film 5353

323 music.artist.origin 4150

3827 film.person_or_entity_appearing_in_film.film 3889

596 people.person.place_of_birth 4455

1153 film.person_or_entity_appearing_in_film.film 7991

2141 film.person_or_entity_appearing_in_film.film 6057

3788 film.actor.film 9224

1728 music.artist.origin 3846

774 film.person_or_entity_appearing_in_film.film 6332

2983 film.person_or_entity_appearing_in_film.film 9154

1043 people.person.place_of_birth 6457

2454 film.person_or_entity_appearing_in_film.film 5162

2071 people.person.place_of_birth 7212

3109 film.actor.film 4970

3498 film.actor.film 6495

3498 film.person_or_entity_appearing_in_film.film 8765

619 film.person_or_entity_appearing_in_film.film 5578

2913 film.person_or_entity_appearing_in_film.film 8705

2913 film.person_or_entity_appearing_in_film.film 6101

1469 film.person_or_entity_appearing_in_film.film 7220

1208 music.musician.instruments_played 4349

1465 film.actor.film 6915

1465 film.actor.film 4283

1465 film.person_or_entity_appearing_in_film.film 6721

2140 people.person.place_of_birth 7417

2636 film.person_or_entity_appearing_in_film.film 7747

2096 film.actor.film 4547

1570 film.actor.film 3880

3541 music.artist.origin 4050

2070 film.actor.film 6864

2070 film.actor.film 4750

1252 film.actor.film 8114

2460 film.actor.film 7644

2460 film.actor.film 7751

317 music.artist.origin 3864

1579 film.person_or_entity_appearing_in_film.film 6372

527 people.deceased_person.place_of_death 4347

484 film.actor.film 8462

3738 games.publisher.games_published 9357

436 film.person_or_entity_appearing_in_film.film 6246

3096 people.deceased_person.place_of_death 7417

1359 film.actor.film 9067

1463 music.artist.origin 3864

2241 film.distributor.film 7700

2241 film.production_company.film 8116

2241 film.production_company.film 9345

2241 film.production_company.film 8083

795 film.person_or_entity_appearing_in_film.film 6087

3398 music.musician.instruments_played 3959

2501 people.person.place_of_birth 7713

2260 film.person_or_entity_appearing_in_film.film 4576

704 music.artist.origin 7588

591 film.actor.film 7601

1064 people.person.place_of_birth 9358

2126 film.person_or_entity_appearing_in_film.film 8236

268 music.artist.origin 4496

3546 film.person_or_entity_appearing_in_film.film 9043

25 film.person_or_entity_appearing_in_film.film 5439

287 film.person_or_entity_appearing_in_film.film 9211

3614 film.person_or_entity_appearing_in_film.film 4446

3132 film.person_or_entity_appearing_in_film.film 6456

2205 music.artist.origin 5161

3722 music.musician.instruments_played 6682

1358 film.actor.film 9305

383 film.person_or_entity_appearing_in_film.film 8275

2635 film.person_or_entity_appearing_in_film.film 4368

1349 film.person_or_entity_appearing_in_film.film 5684

3384 film.actor.film 9240

3827 film.actor.film 4655

3827 film.person_or_entity_appearing_in_film.film 8715

3275 film.person_or_entity_appearing_in_film.film 4910

42 music.artist.origin 4150

808 music.artist.origin 4057

1026 film.actor.film 8729

3764 people.person.place_of_birth 4768

3784 film.person_or_entity_appearing_in_film.film 3904

127 film.person_or_entity_appearing_in_film.film 6364

2433 music.artist.origin 8939

407 film.actor.film 6884

407 film.person_or_entity_appearing_in_film.film 7491

583 film.actor.film 9349

3363 film.actor.film 4446

2259 film.actor.film 4670

55 music.artist.origin 9246

1423 film.person_or_entity_appearing_in_film.film 8991

1668 music.artist.origin 6349

2636 film.actor.film 6658

1570 film.person_or_entity_appearing_in_film.film 3880

2070 film.actor.film 7566

2781 people.deceased_person.place_of_death 4013

366 film.person_or_entity_appearing_in_film.film 9217

294 film.writer.film 7317

2973 music.artist.origin 4876

3738 cvg.publisher.games_published 7071

2293 film.person_or_entity_appearing_in_film.film 7220

2241 film.distributor.film 8827

2241 film.production_company.film 8826

2241 film.production_company.film 4400

2241 film.production_company.film 8758

795 music.artist.origin 5258

1964 music.artist.origin 5161

3350 film.actor.film 9070

3662 music.musician.instruments_played 4349

3698 music.artist.origin 4232

516 film.actor.film 6668

2773 people.person.place_of_birth 6261

777 film.actor.film 5823

2487 film.actor.film 8496

2487 film.actor.film 5507

258 film.person_or_entity_appearing_in_film.film 7636

3354 award.competitor.award_nominations 9291

185 music.artist.origin 6868

2103 music.artist.origin 3864

2741 film.actor.film 4546

434 film.actor.film 9330

3000 film.person_or_entity_appearing_in_film.film 4636

3000 film.person_or_entity_appearing_in_film.film 6351

157 award.competitor.awards_won 9311

3742 film.actor.film 4155

3648 film.person_or_entity_appearing_in_film.film 4356

2658 film.person_or_entity_appearing_in_film.film 9149

2243 people.deceased_person.place_of_death 3895

2101 film.person_or_entity_appearing_in_film.film 8241

1637 music.artist.origin 3961

1251 film.actor.film 9266

206 film.actor.film 7053

1955 music.artist.album 4523

1775 people.person.place_of_birth 4191

2635 film.person_or_entity_appearing_in_film.film 7505

1157 film.actor.film 7400

540 film.actor.film 7882

818 film.person_or_entity_appearing_in_film.film 8189

1232 film.person_or_entity_appearing_in_film.film 7948

3317 film.actor.film 8151

3555 people.person.place_of_birth 5019

1473 film.person_or_entity_appearing_in_film.film 9269

1080 music.artist.origin 4391

279 film.actor.film 4418

645 film.actor.film 4441

3598 film.person_or_entity_appearing_in_film.film 7509

3598 film.person_or_entity_appearing_in_film.film 9359

3447 film.person_or_entity_appearing_in_film.film 7718

619 music.musician.instruments_played 3959

2941 people.person.place_of_birth 5161

70 film.actor.film 8822

1601 film.actor.film 8955

2095 film.person_or_entity_appearing_in_film.film 6436

2688 film.person_or_entity_appearing_in_film.film 4786

529 music.artist.origin 7417

389 award.nominee.award_nominations 8980

1353 film.actor.film 9248

3738 games.publisher.games_published 9300

1154 film.producer.film 7519

915 music.artist.origin 4010

3452 film.actor.film 5590

625 film.actor.film 9360

625 film.person_or_entity_appearing_in_film.film 9360

3834 film.actor.film 9325

1249 film.person_or_entity_appearing_in_film.film 8753

2241 film.distributor.film 6475

2241 film.distributor.film 8385

2241 film.production_company.film 8798

2241 film.production_company.film 5704

2241 film.production_company.film 8959

2685 people.deceased_person.place_of_death 8586

832 film.actor.film 6644

2931 people.person.place_of_birth 4044

430 film.person_or_entity_appearing_in_film.film 4895

3297 film.person_or_entity_appearing_in_film.film 5087

681 film.person_or_entity_appearing_in_film.film 5990

1624 film.person_or_entity_appearing_in_film.film 8802

1262 film.person_or_entity_appearing_in_film.film 7757

1777 film.actor.film 7660

549 film.actor.film 7573

998 people.person.place_of_birth 8998

2678 film.person_or_entity_appearing_in_film.film 5117

469 film.actor.film 4414

425 music.artist.origin 4057

2531 film.actor.film 9139

3684 music.artist.origin 4380

2947 people.person.place_of_birth 3846

1657 film.film.star 7502

3089 music.artist.origin 4362

3731 music.artist.origin 4362

3473 film.person_or_entity_appearing_in_film.film 4857

3536 people.person.place_of_birth 4362

1482 music.artist.origin 3846

3822 film.person_or_entity_appearing_in_film.film 5679

459 people.person.place_of_birth 4479

1157 film.actor.film 6998

1157 film.person_or_entity_appearing_in_film.film 8016

1045 film.actor.film 7831

1720 film.director.film 9099

2796 music.artist.origin 7079

1989 film.person_or_entity_appearing_in_film.film 7214

2492 music.artist.origin 7915

2365 people.deceased_person.place_of_death 4453

2422 music.artist.album 4836

2359 film.person_or_entity_appearing_in_film.film 7110

3736 music.artist.origin 7531

1960 film.person_or_entity_appearing_in_film.film 4715

1625 film.actor.film 5244

377 film.person_or_entity_appearing_in_film.film 9294

2080 film.actor.film 9361

3598 film.person_or_entity_appearing_in_film.film 6976

690 music.artist.origin 5662

518 music.artist.origin 9242

52 film.person_or_entity_appearing_in_film.film 5533

672 film.person_or_entity_appearing_in_film.film 8537

1181 film.person_or_entity_appearing_in_film.film 9350

134 film.person_or_entity_appearing_in_film.film 9362

2636 film.person_or_entity_appearing_in_film.film 5768

2096 film.actor.film 8024

505 music.artist.origin 4901

965 music.artist.origin 3897

79 film.person_or_entity_appearing_in_film.film 9314

1679 music.artist.origin 8939

484 film.person_or_entity_appearing_in_film.film 6606

2483 film.actor.film 6918

1463 award.nominee.award_nominations 9094

442 music.musician.instruments_played 3959

2241 film.distributor.film 5343

481 film.actor.film 6382

1464 film.person_or_entity_appearing_in_film.film 9146

3506 music.artist.origin 4044

2500 people.person.place_of_birth 3963

2447 music.artist.origin 4635

777 film.person_or_entity_appearing_in_film.film 6631

215 music.artist.album 9125

3789 people.person.place_of_birth 4347

1499 music.artist.origin 4013

2628 music.artist.origin 4217

2261 film.actor.film 8213

2678 film.actor.film 5764

1714 people.person.place_of_birth 4534

3257 music.artist.origin 5106

3772 film.film.country 4815

3772 film.film.director 6112

3145 music.artist.origin 7079

3231 film.person_or_entity_appearing_in_film.film 8460

2992 music.artist.origin 3916

298 music.artist.album 9177

81 film.person_or_entity_appearing_in_film.film 8832

2556 film.actor.film 6155

460 music.artist.album 9334

241 film.actor.film 9182

526 music.artist.origin 7630

2298 people.person.place_of_birth 4013

1750 film.person_or_entity_appearing_in_film.film 6045

2238 film.actor.film 6650

206 film.actor.film 7270

1157 film.actor.film 5054

115 film.actor.film 9344

2494 film.person_or_entity_appearing_in_film.film 7684

699 people.person.place_of_birth 6358

815 film.person_or_entity_appearing_in_film.film 5117

1153 film.person_or_entity_appearing_in_film.film 6124

2978 film.actor.film 6625

435 film.person_or_entity_appearing_in_film.film 4363

2799 film.person_or_entity_appearing_in_film.film 5562

67 film.person_or_entity_appearing_in_film.film 5182

1175 music.artist.origin 3897

2804 film.person_or_entity_appearing_in_film.film 9134

1383 film.actor.film 8330

1141 film.person_or_entity_appearing_in_film.film 9244

2750 film.film.language 9140

2998 film.actor.film 8853

828 music.artist.origin 3888

2737 film.person_or_entity_appearing_in_film.film 5017

2737 film.person_or_entity_appearing_in_film.film 6551

279 film.person_or_entity_appearing_in_film.film 5479

66 film.actor.film 7034

3598 film.actor.film 9123

2937 music.artist.origin 8250

1469 film.actor.film 3909

46 film.person_or_entity_appearing_in_film.film 5363

1204 film.person_or_entity_appearing_in_film.film 8416

128 film.person_or_entity_appearing_in_film.film 5851

1559 film.person_or_entity_appearing_in_film.film 4494

2636 film.person_or_entity_appearing_in_film.film 5665

2070 film.person_or_entity_appearing_in_film.film 8879

1211 film.actor.film 8081

160 film.person_or_entity_appearing_in_film.film 8135

1355 people.person.place_of_birth 6271

3166 film.person_or_entity_appearing_in_film.film 6503

3738 cvg.publisher.games_published 9203

3738 cvg.publisher.games_published 5635

3738 games.publisher.games_published 8750

2347 music.artist.origin 3864

901 film.actor.film 6070

285 music.artist.origin 4635

3201 music.artist.origin 4010

3225 film.person_or_entity_appearing_in_film.film 8382

1249 film.person_or_entity_appearing_in_film.film 5079

2850 music.artist.origin 4957

442 people.person.profession 4221

2241 film.production_company.film 8783

1464 film.person_or_entity_appearing_in_film.film 8801

1464 film.person_or_entity_appearing_in_film.film 9336

3142 music.artist.origin 3916

3649 music.artist.origin 3864

1624 film.person_or_entity_appearing_in_film.film 4802

2487 film.actor.film 8949

1064 music.artist.origin 9358

356 award.nominee.award_nominations 7354

3354 film.person_or_entity_appearing_in_film.film 4792

2861 film.actor.film 8951

2493 film.person_or_entity_appearing_in_film.film 8759

694 music.artist.origin 4272

538 film.person_or_entity_appearing_in_film.film 9021

36 film.actor.film 8400

3321 film.person_or_entity_appearing_in_film.film 3978

3231 film.actor.film 4637

2843 film.person_or_entity_appearing_in_film.film 3943

810 people.deceased_person.place_of_death 6343

3390 film.person_or_entity_appearing_in_film.film 9229

2108 people.person.place_of_birth 6694

2711 film.actor.film 6904

3305 music.artist.origin 4108

1814 film.film.writer 7483

916 film.person_or_entity_appearing_in_film.film 8472

2833 film.actor.film 4328

2635 film.actor.film 8309

3114 film.person_or_entity_appearing_in_film.film 7398

598 film.film.genre 8432

410 film.actor.film 9343

714 music.album.genre 8510

3827 film.person_or_entity_appearing_in_film.film 6488

815 film.actor.film 9143

2441 people.person.place_of_birth 3972

3444 music.musician.instruments_played 3851

1823 music.artist.origin 5061

1473 film.person_or_entity_appearing_in_film.film 6091

1473 film.person_or_entity_appearing_in_film.film 9348

346 music.artist.origin 4362

3204 film.person_or_entity_appearing_in_film.film 4967

2002 music.artist.origin 4768

1711 film.actor.film 4493

3598 film.actor.film 9359

3093 music.artist.origin 4191

386 film.person_or_entity_appearing_in_film.film 8208

1566 film.person_or_entity_appearing_in_film.film 6335

646 film.person_or_entity_appearing_in_film.film 4074

447 people.person.profession 4221

2636 film.person_or_entity_appearing_in_film.film 4051

147 film.person_or_entity_appearing_in_film.film 7928

3553 music.artist.origin 6555

2070 film.person_or_entity_appearing_in_film.film 9299

3179 film.actor.film 9216

3166 film.person_or_entity_appearing_in_film.film 5930

1502 film.person_or_entity_appearing_in_film.film 7533

715 film.actor.film 4720

3738 games.publisher.games_published 8615

3738 games.publisher.games_published 4138

184 film.person_or_entity_appearing_in_film.film 8982

3033 people.person.place_of_birth 8877

668 music.artist.origin 6343

2867 film.actor.film 6414

2867 film.actor.film 7016

2241 film.distributor.film 4962

2241 film.distributor.film 9069

1288 music.artist.origin 4294

2501 film.actor.film 9047

3160 film.actor.film 9341

2232 film.person_or_entity_appearing_in_film.film 8181

2445 music.artist.origin 4991

2311 film.person_or_entity_appearing_in_film.film 3977

591 film.person_or_entity_appearing_in_film.film 5825

3031 film.person_or_entity_appearing_in_film.film 9303

1567 film.actor.film 6872

418 film.actor.film 7858

958 music.artist.album 9318

1486 games.game.publisher 7173

1660 film.person_or_entity_appearing_in_film.film 7235

600 music.artist.album 7008

3052 film.person_or_entity_appearing_in_film.film 8603

3279 film.actor.film 9148

3648 film.person_or_entity_appearing_in_film.film 8903

2010 film.person_or_entity_appearing_in_film.film 7503

2343 film.actor.film 5751

997 film.person_or_entity_appearing_in_film.film 6117

2904 film.person_or_entity_appearing_in_film.film 7679

1949 music.artist.origin 8349

115 film.person_or_entity_appearing_in_film.film 9059

2515 film.person_or_entity_appearing_in_film.film 4385

2507 music.producer.tracks_produced 9335

2161 award.competitor.award_nominations 8327

627 film.actor.film 8516

1826 film.actor.film 5990

149 film.person_or_entity_appearing_in_film.film 6766

2134 event.agent.performance 4387

2733 film.person_or_entity_appearing_in_film.film 4173

2147 people.person.place_of_birth 3993

1473 film.actor.film 9339

2182 people.person.place_of_birth 6723

1141 film.person_or_entity_appearing_in_film.film 6528

895 film.actor.film 4965

2080 film.actor.film 8983

675 film.actor.film 7137

1007 event.agent.performance 4898

628 film.person_or_entity_appearing_in_film.film 9308

3711 music.artist.origin 4796

3500 film.actor.film 7844

3021 film.actor.film 3939

2475 film.person_or_entity_appearing_in_film.film 9313

3738 games.publisher.games_published 6581

3738 games.publisher.games_published 5123

1154 film.actor.film 4614

3618 music.artist.origin 6736

1359 film.person_or_entity_appearing_in_film.film 9112

3462 film.actor.film 5010

1249 film.actor.film 4777

668 film.actor.film 5743

2241 film.distributor.film 8362

2241 film.distributor.film 8800

2241 film.production_company.film 5594

481 film.actor.film 9340

2311 film.actor.film 5821

1819 film.actor.film 6706

261 film.person_or_entity_appearing_in_film.film 9196

1360 film.actor.film 8711

643 film.actor.film 7945

418 film.actor.film 5342

418 music.producer.tracks_produced 7965

1475 film.person_or_entity_appearing_in_film.film 7821

3037 music.artist.origin 4057

277 film.person_or_entity_appearing_in_film.film 5555

2064 event.agent.performance 4251

2687 people.person.place_of_birth 4217

238 film.writer.film 7968

848 film.actor.film 9331

3742 film.person_or_entity_appearing_in_film.film 5050

2556 film.actor.film 5470

3428 film.person_or_entity_appearing_in_film.film 7458

3473 film.actor.film 5753

971 music.artist.origin 3961

68 film.person_or_entity_appearing_in_film.film 7958

206 film.actor.film 7735

2938 film.actor.film 7667

997 film.actor.film 8341

1279 film.person_or_entity_appearing_in_film.film 9279

2621 film.actor.film 7863

1720 film.director.film 8819

1989 film.person_or_entity_appearing_in_film.film 5402

1044 film.director.film 9142

3496 film.film.star 6397

2441 film.person_or_entity_appearing_in_film.film 5273

1505 people.person.place_of_birth 5662

1318 music.artist.origin 4377

1153 film.actor.film 8789

2141 film.actor.film 7992

3482 music.artist.origin 5807

2187 film.actor.film 6300

2216 music.artist.track 7995

2359 film.person_or_entity_appearing_in_film.film 6858

1473 film.actor.film 9293

1473 film.person_or_entity_appearing_in_film.film 4819

1473 film.person_or_entity_appearing_in_film.film 4544

1471 people.person.profession 4221

2389 film.actor.film 5949

2700 film.actor.film 7912

1986 music.artist.origin 5061

3112 music.artist.origin 3961

192 music.producer.tracks_produced 8395

645 film.actor.film 6950

2385 music.artist.origin 4150

3498 film.person_or_entity_appearing_in_film.film 5735

619 people.person.place_of_birth 4764

2848 film.film.language 4076

1384 music.artist.origin 5873

3729 music.artist.origin 4058

1204 film.person_or_entity_appearing_in_film.film 9363

2636 film.person_or_entity_appearing_in_film.film 8285

2096 film.actor.film 8734

2070 film.actor.film 8248

874 music.artist.origin 7370

1895 film.person_or_entity_appearing_in_film.film 8026

2460 film.actor.film 5119

79 film.person_or_entity_appearing_in_film.film 3852

3738 cvg.publisher.games_published 7941

3738 games.publisher.games_published 8436

184 film.person_or_entity_appearing_in_film.film 4460

184 film.person_or_entity_appearing_in_film.film 8268

2293 film.person_or_entity_appearing_in_film.film 9364

1350 music.artist.origin 4808

901 film.actor.film 9324

2241 film.distributor.film 7943

2241 film.production_company.film 5255

2241 film.production_company.film 9365

894 film.actor.film 7548

2112 film.person_or_entity_appearing_in_film.film 9302

108 film.actor.film 6668

3306 music.album.genre 7180

1274 music.artist.origin 4442

3321 film.actor.film 4948

1387 people.person.place_of_birth 8349

3390 film.actor.film 8785

1786 film.actor.film 5869

1899 music.artist.origin 5386

872 music.artist.origin 4362

601 music.artist.origin 4362

997 film.actor.film 7971

959 people.person.place_of_birth 4362

2635 film.person_or_entity_appearing_in_film.film 5978

309 music.artist.origin 7915

2691 music.artist.origin 4442

2724 film.person_or_entity_appearing_in_film.film 4168

3514 film.person_or_entity_appearing_in_film.film 8975

1032 film.person_or_entity_appearing_in_film.film 7835

3423 film.person_or_entity_appearing_in_film.film 7766

67 film.person_or_entity_appearing_in_film.film 6627

2804 film.actor.film 5841

192 music.producer.tracks_produced 8791

84 film.person_or_entity_appearing_in_film.film 9054

3598 film.actor.film 6885

1885 film.actor.film 9274

106 film.person_or_entity_appearing_in_film.film 8716

1024 music.artist.origin 4217

3498 film.person_or_entity_appearing_in_film.film 9145

1704 film.actor.film 5016

732 music.artist.origin 5227

646 film.actor.film 9234

3120 film.actor.film 8984

2636 film.person_or_entity_appearing_in_film.film 5630

2096 film.person_or_entity_appearing_in_film.film 8660

1458 film.person_or_entity_appearing_in_film.film 8869

1308 film.actor.film 6052

2483 film.actor.film 8736

1154 film.actor.film 8647

2034 film.actor.film 4346

625 film.person_or_entity_appearing_in_film.film 5742

318 music.artist.origin 8245

1002 music.artist.origin 7321

1249 film.actor.film 8754

2577 film.person_or_entity_appearing_in_film.film 5126

442 award.nominee.award_nominations 5546

442 award.winner.awards_won 7195

2241 film.production_company.film 5547

2241 film.production_company.film 4839

3340 people.person.place_of_birth 8468

135 film.actor.film 5406

2487 film.actor.film 6790

1767 film.person_or_entity_appearing_in_film.film 8950

3744 people.person.place_of_birth 4861

469 film.actor.film 9128

817 film.person_or_entity_appearing_in_film.film 7859

1354 music.artist.origin 6400

1475	film.actor.film	9271
3331	film.person_or_entity_appearing_in_film.film	8804
1278	award.nominee.award_nominations	9316
2861	film.person_or_entity_appearing_in_film.film	5866
1526	music.artist.album	8772
3321	film.actor.film	7552
3095	film.person_or_entity_appearing_in_film.film	5416
3048	people.person.place_of_birth	6331
204 film.actor.film	5145
2228	people.person.place_of_birth	4050
1929	music.artist.origin	7804
687 film.person_or_entity_appearing_in_film.film	9023
363 film.actor.film	7126
350 music.artist.origin	8652
206 film.actor.film	9232
2771	film.actor.film	8761
2635	film.actor.film	7301
3194	people.person.place_of_birth	6855
2366	film.person_or_entity_appearing_in_film.film	6138
2170	music.artist.origin	8305
1720	film.director.film 7712
3111	music.musician.instruments_played	4349
3827	film.person_or_entity_appearing_in_film.film	8242
3827	people.person.place_of_birth	4222
3171	music.artist.origin	4963
3329	people.person.place_of_birth	8945
1488	award.competitor.award_nominations	8665
633 film.person_or_entity_appearing_in_film.film	8129
1805	film.actor.film	7698
2201	film.actor.film	4543

1383 film.person_or_entity_appearing_in_film.film 8330

1473 film.actor.film 8532

1473 film.person_or_entity_appearing_in_film.film 8204

136 film.person_or_entity_appearing_in_film.film 9353

3302 film.actor.film 7888

2673 people.person.place_of_birth 8586

1879 music.artist.origin 5623

1083 music.artist.origin 4199

3168 music.artist.origin 4108

3658 music.artist.origin 4851

2271 people.deceased_person.place_of_death 5523

2916 film.actor.film 8345

3734 music.artist.origin 9347

3121 film.person_or_entity_appearing_in_film.film 8328

3344 film.person_or_entity_appearing_in_film.film 4724

2338 people.person.place_of_birth 4825

2168 music.artist.origin 8940

1469 film.actor.film 4637

414 film.person_or_entity_appearing_in_film.film 6536

2562 film.person_or_entity_appearing_in_film.film 4283

1204 film.actor.film 9270

1204 film.person_or_entity_appearing_in_film.film 9027

2636 film.person_or_entity_appearing_in_film.film 7590

2636 film.person_or_entity_appearing_in_film.film 5171

3179 film.person_or_entity_appearing_in_film.film 8098

3738 games.publisher.games_published 5954

3212 film.person_or_entity_appearing_in_film.film 7724

3113 music.artist.origin 4013

2867 film.person_or_entity_appearing_in_film.film 9352

442 music.artist.album 4020

2360 film.actor.film 7616

2241 film.distributor.film 9365

2241 film.distributor.film 9346

2241 film.production_company.film 5998

795 film.actor.film 6155

1936 film.actor.film 7981

3430 film.person_or_entity_appearing_in_film.film 8692

3326 music.artist.origin 3864

691 music.artist.origin 4852

1768 film.person_or_entity_appearing_in_film.film 4306

2951 film.person_or_entity_appearing_in_film.film 7550

3031 film.actor.film 9127

1475 film.actor.film 6929

1284 music.artist.album 6591

1241 film.film.country 3961

1310 music.musician.instruments_played 5367

2711 film.person_or_entity_appearing_in_film.film 7925

1479 film.person_or_entity_appearing_in_film.film 7240

2158 music.artist.origin 4174

206 film.person_or_entity_appearing_in_film.film 6713

3381 film.actor.film 6592

2635 film.actor.film 8501

2635 film.person_or_entity_appearing_in_film.film 6522

115 film.actor.film 5655

3707 music.artist.origin 4471

623 film.person_or_entity_appearing_in_film.film 6325

1989 people.person.place_of_birth 4999

1044 award.nominee.award_nominations 9163

3827 film.actor.film 8852

141 music.artist.origin 3864

2371 film.actor.film 8503

1134 music.artist.origin 9137

1383 film.person_or_entity_appearing_in_film.film 4720

1289 music.artist.origin 4093

3571 music.artist.origin 4977

66 film.person_or_entity_appearing_in_film.film 4968

1945 film.person_or_entity_appearing_in_film.film 7047

695 music.artist.origin 6137

508 award.competitor.award_nominations 8838

134 film.actor.film 9362

1204 film.actor.film 9363

2095 film.actor.film 4610

3561 film.person_or_entity_appearing_in_film.film 7373

128 film.actor.film 6216

2636 film.person_or_entity_appearing_in_film.film 5130

3162 music.artist.origin 4852

2496 people.person.place_of_birth 4526

2070 film.actor.film 9038

1579 film.person_or_entity_appearing_in_film.film 8450

602 film.actor.film 6693

2483 film.actor.film 8934

2293 film.actor.film 9364

3517 people.person.place_of_birth 4931

2241 film.distributor.film 5904

2241 film.distributor.film 8360

2241 film.production_company.film 5592

1824 film.actor.film 9008

336 music.artist.origin 5726

1780 people.person.place_of_birth 6263

1894 people.person.place_of_birth 5910

2880 film.actor.film 3936

2988 music.artist.origin 4050

2275 film.person_or_entity_appearing_in_film.film 3939

2766 film.person_or_entity_appearing_in_film.film 9168

1215 music.artist.origin 4800

1580 film.person_or_entity_appearing_in_film.film 3871

1088 music.artist.origin 4174

1451 music.artist.album 7847

34 film.actor.film 7054

1540 film.actor.film 8401

1814 film.film.country 3961

3132 film.actor.film 9238

206 film.actor.film 8148

2242 film.person_or_entity_appearing_in_film.film 7878

1142 film.actor.film 9033

1142 film.person_or_entity_appearing_in_film.film 8531

3456 people.deceased_person.place_of_death 6190

1279 film.person_or_entity_appearing_in_film.film 4531

288 music.artist.origin 4332

3381 film.actor.film 5564

2635 film.person_or_entity_appearing_in_film.film 7486

1157 film.actor.film 9322

1943 film.person_or_entity_appearing_in_film.film 9287

115 film.actor.film 6801

818 film.actor.film 6999

2494 film.actor.film 7684

374 film.actor.film 9108

1166 music.artist.origin 4454

1628 music.artist.origin 5258

2079 film.actor.film 9272

2359	film.person_or_entity_appearing_in_film.film	4599
2344	film.actor.film	8132
2337	film.person_or_entity_appearing_in_film.film	6434
3415	music.artist.origin	5413
3232	music.artist.origin	4616
2080	film.actor.film	8058
2080	film.person_or_entity_appearing_in_film.film	9361
3102	music.artist.origin	5213
