# Supplementary material for: Music recommendation algorithms based on knowledge graph and multi-task feature learning
Source: Sci Rep. 2024 Jan 24;14:2055. doi: 10.1038/s41598-024-52463-z (PMC10808181; doi:10.1038/s41598-024-52463-z)
Supplement: Supplementary file 3 — Supplementary Information 3. [file 41598_2024_52463_MOESM3_ESM.pdf]

0	32	1
0	20	1
0	21	1
0	22	1
0	23	1
0	24	1
0	25	1
0	26	1
0	27	1
0	28	1
0	29	1
0	30	1
0	31	1
0	259	0
0	963	0
0	2432	0
0	2398	0
0	3669	0
0	1406	0
0	1962	0
0	3724	0
0	2584	0
0	1400	0
0	3239	0
0	3796	0
0	2995	0
1	33	1
1	1115	0
2	34	1
2	35	1

2	36	1
2	37	1
2	38	1
2	39	1
2	40	1
2	41	1
2	42	1
2	43	1
2	24	1
2	593	0
2	1073	0
2	2716	0
2	919	0
2	1970	0
2	272	0
2	3548	0
2	2437	0
2	1009	0
2	2188	0
2	3183	0
3	44	1
3	45	1
3	46	1
3	47	1
3	48	1
3	49	1
3	50	1
3	51	1
3	52	1
3	53	1

3	54	1
3	55	1
3	56	1
3	57	1
3	1090	0
3	1121	0
3	2044	0
3	594	0
3	697	0
3	2081	0
3	1263	0
3	1178	0
3	437	0
3	2089	0
3	1298	0
3	294	0
3	3809	0
3	3213	0
4	64	1
4	65	1
4	66	1
4	67	1
4	68	1
4	69	1
4	70	1
4	71	1
4	58	1
4	59	1
4	60	1
4	61	1

4	62	1
4	63	1
4	2443	0
4	2380	0
4	1778	0
4	289	0
4	3312	0
4	194	0
4	2129	0
4	2636	0
4	2246	0
4	2836	0
4	2540	0
4	468	0
4	2693	0
4	1278	0
5	72	1
5	73	1
5	74	1
5	75	1
5	76	1
5	77	1
5	78	1
5	79	1
5	80	1
5	81	1
5	2181	0
5	1392	0
5	2979	0
5	1239	0

5	2891	0
5	1818	0
5	3769	0
5	1186	0
5	3126	0
5	2232	0
6	66	1
6	72	1
6	75	1
6	82	1
6	83	1
6	84	1
6	85	1
6	86	1
6	87	1
6	88	1
6	89	1
6	90	1
6	91	1
6	92	1
6	1796	0
6	2445	0
6	278	0
6	268	0
6	2551	0
6	2459	0
6	2183	0
6	1726	0
6	805	0
6	3689	0

6	1584	0
6	1719	0
6	583	0
6	1369	0
7	96	1
7	97	1
7	98	1
7	99	1
7	100	1
7	101	1
7	102	1
7	103	1
7	93	1
7	94	1
7	95	1
7	1661	0
7	735	0
7	1682	0
7	1852	0
7	2556	0
7	273	0
7	2924	0
7	2605	0
7	2903	0
7	1503	0
7	3563	0
8	104	1
8	105	1
8	106	1
8	107	1

8	44	1
8	108	1
8	109	1
8	110	1
8	111	1
8	112	1
8	113	1
8	51	1
8	114	1
8	55	1
8	1890	0
8	3397	0
8	536	0
8	1224	0
8	783	0
8	1472	0
8	2453	0
8	1063	0
8	3538	0
8	3083	0
8	1685	0
8	2561	0
8	1893	0
8	907	0
9	79	1
9	115	1
9	116	1
9	117	1
9	87	1
9	1576	0

9	1137	0
9	3581	0
9	2373	0
9	1335	0
10	96	1
10	100	1
10	43	1
10	118	1
10	119	1
10	120	1
10	121	1
10	122	1
10	123	1
10	124	1
10	125	1
10	126	1
10	3657	0
10	1644	0
10	241	0
10	1029	0
10	3753	0
10	3288	0
10	3735	0
10	2672	0
10	2624	0
10	809	0
10	3653	0
10	3798	0
11	128	1
11	129	1

11	130	1
11	131	1
11	132	1
11	133	1
11	134	1
11	135	1
11	136	1
11	137	1
11	74	1
11	79	1
11	80	1
11	55	1
11	87	1
11	127	1
11	3039	0
11	2212	0
11	230	0
11	2636	0
11	3630	0
11	3485	0
11	3789	0
11	3382	0
11	3165	0
11	1234	0
11	1539	0
11	3223	0
11	408	0
11	58	0
11	301	0
11	1743	0

12	138	1
12	139	1
12	140	1
12	141	1
12	142	1
12	143	1
12	144	1
12	145	1
12	146	1
12	147	1
12	148	1
12	149	1
12	150	1
12	151	1
12	152	1
12	153	1
12	250	0
12	3580	0
12	1579	0
12	689	0
12	2840	0
12	1131	0
12	1382	0
12	2939	0
12	1142	0
12	2113	0
12	910	0
12	1013	0
12	1978	0
12	1503	0

12	569	0
12	1673	0
13	160	1
13	161	1
13	162	1
13	163	1
13	164	1
13	165	1
13	166	1
13	53	1
13	154	1
13	155	1
13	156	1
13	157	1
13	158	1
13	159	1
13	1478	0
13	3812	0
13	2801	0
13	555	0
13	3600	0
13	466	0
13	2076	0
13	827	0
13	291	0
13	2051	0
13	3479	0
13	3763	0
13	2581	0
13	181	0

14	131	1
14	167	1
14	168	1
14	169	1
14	170	1
14	171	1
14	172	1
14	173	1
14	174	1
14	175	1
14	176	1
14	177	1
14	178	1
14	179	1
14	180	1
14	181	1
14	182	1
14	183	1
14	98	1
14	3474	0
14	3832	0
14	1148	0
14	383	0
14	1464	0
14	742	0
14	11	0
14	3121	0
14	1107	0
14	1577	0
14	477	0

14	201	0
14	1717	0
14	39	0
14	1193	0
14	1068	0
14	3034	0
14	60	0
14	3368	0
15	133	1
15	137	1
15	78	1
15	79	1
15	184	1
15	185	1
15	186	1
15	187	1
15	1155	0
15	2080	0
15	3454	0
15	1244	0
15	2440	0
15	1240	0
15	3197	0
15	3715	0
16	192	1
16	193	1
16	194	1
16	36	1
16	52	1
16	188	1

16	189	1
16	190	1
16	191	1
16	2270	0
16	3239	0
16	3077	0
16	2708	0
16	785	0
16	132	0
16	2962	0
16	2887	0
16	876	0
17	195	1
17	196	1
17	36	1
17	197	1
17	198	1
17	199	1
17	200	1
17	201	1
17	372	0
17	1652	0
17	830	0
17	1634	0
17	3198	0
17	1401	0
17	2629	0
17	3444	0
18	130	1
18	132	1

18	135	1
18	74	1
18	202	1
18	203	1
18	204	1
18	205	1
18	79	1
18	206	1
18	177	1
18	207	1
18	184	1
18	185	1
18	2723	0
18	389	0
18	3345	0
18	1751	0
18	3196	0
18	505	0
18	384	0
18	3608	0
18	2300	0
18	2720	0
18	582	0
18	2528	0
18	481	0
18	151	0
19	102	1
19	208	1
19	209	1
19	210	1

19	211	1
19	212	1
19	213	1
19	214	1
19	215	1
19	216	1
19	217	1
19	218	1
19	219	1
19	220	1
19	125	1
19	2083	0
19	3011	0
19	3842	0
19	3476	0
19	2351	0
19	114	0
19	382	0
19	203	0
19	1889	0
19	2221	0
19	2717	0
19	3704	0
19	655	0
19	2865	0
19	3739	0
20	224	1
20	225	1
20	34	1
20	226	1

20	36	1
20	227	1
20	228	1
20	229	1
20	109	1
20	52	1
20	221	1
20	222	1
20	223	1
20	1075	0
20	2391	0
20	2313	0
20	3502	0
20	3804	0
20	841	0
20	1703	0
20	322	0
20	2496	0
20	1537	0
20	628	0
20	170	0
20	3371	0
21	230	1
21	231	1
21	232	1
21	233	1
21	74	1
21	234	1
21	235	1
21	236	1

21	110	1
21	3370	0
21	1678	0
21	3193	0
21	3809	0
21	1797	0
21	208	0
21	954	0
21	2758	0
21	1834	0
22	130	1
22	135	1
22	72	1
22	73	1
22	74	1
22	75	1
22	237	1
22	238	1
22	79	1
22	80	1
22	239	1
22	87	1
22	88	1
22	641	0
22	3673	0
22	3414	0
22	296	0
22	2441	0
22	2152	0
22	1819	0

22	1662	0
22	2166	0
22	1667	0
22	2741	0
22	3044	0
22	3589	0
23	3	1
23	240	1
23	241	1
23	242	1
23	243	1
23	244	1
23	245	1
23	246	1
23	247	1
23	248	1
23	249	1
23	250	1
23	251	1
23	191	0
23	1170	0
23	3216	0
23	3340	0
23	2794	0
23	2839	0
23	171	0
23	354	0
23	1780	0
23	1341	0
23	2978	0

23	3616	0
23	514	0
24	34	1
24	40	1
24	137	1
24	104	1
24	80	1
24	187	1
24	252	1
24	253	1
24	254	1
24	255	1
24	2559	0
24	741	0
24	3401	0
24	1954	0
24	453	0
24	3253	0
24	3447	0
24	3174	0
24	1595	0
24	2816	0
25	256	1
25	257	1
25	258	1
25	259	1
25	260	1
25	261	1
25	262	1
25	263	1

25	264	1
25	265	1
25	26	1
25	28	1
25	3033	0
25	1956	0
25	2582	0
25	3540	0
25	2752	0
25	2224	0
25	1461	0
25	1138	0
25	1140	0
25	1435	0
25	3632	0
25	2579	0
26	130	1
26	135	1
26	266	1
26	77	1
26	47	1
26	80	1
26	185	1
26	1359	0
26	1224	0
26	481	0
26	3700	0
26	2178	0
26	3434	0
26	2086	0

27	133	1
27	171	1
27	267	1
27	268	1
27	269	1
27	79	1
27	270	1
27	115	1
27	55	1
27	1155	0
27	3014	0
27	1623	0
27	1239	0
27	2560	0
27	812	0
27	3624	0
27	826	0
27	750	0
28	227	1
28	265	1
28	13	1
28	271	1
28	272	1
28	273	1
28	274	1
28	275	1
28	276	1
28	277	1
28	155	1
28	254	1

28	1877	0
28	3717	0
28	961	0
28	1613	0
28	1106	0
28	3314	0
28	3688	0
28	2047	0
28	3506	0
28	1312	0
28	2321	0
28	1351	0
29	96	1
29	44	1
29	210	1
29	52	1
29	278	1
29	279	1
29	280	1
29	281	1
29	282	1
29	283	1
29	221	1
29	2805	0
29	1787	0
29	3473	0
29	259	0
29	1960	0
29	691	0
29	229	0

29	3758	0
29	539	0
29	2793	0
29	3330	0
30	288	1
30	289	1
30	34	1
30	290	1
30	291	1
30	165	1
30	40	1
30	42	1
30	285	1
30	284	1
30	253	1
30	286	1
30	287	1
30	3079	0
30	256	0
30	2793	0
30	2938	0
30	879	0
30	1498	0
30	3510	0
30	167	0
30	3194	0
30	1432	0
30	39	0
30	2983	0
30	1319	0

31	159	1
31	36	1
31	292	1
31	293	1
31	294	1
31	295	1
31	296	1
31	297	1
31	52	1
31	221	1
31	191	1
31	3500	0
31	364	0
31	1631	0
31	74	0
31	3432	0
31	3646	0
31	465	0
31	2662	0
31	1973	0
31	466	0
31	1103	0
32	298	1
32	299	1
32	300	1
32	2311	0
32	2285	0
32	173	0
33	199	1
33	267	1

33 301 1

33 302 1

33 47 1

33 208 1

33 303 1

33 210 1

33 304 1

33 305 1

33 3056 0

33 3631 0

33 3110 0

33 26 0

33 1670 0

33 197 0

33 3718 0

33 166 0

33 1181 0

33 3044 0

34 164 1

34 306 1

34 307 1

34 308 1

34 309 1

34 310 1

34 311 1

34 312 1

34 313 1

34 314 1

34 315 1

34 316 1

34	317	1
34	318	1
34	3063	0
34	611	0
34	3178	0
34	1945	0
34	1504	0
34	2610	0
34	282	0
34	3067	0
34	1338	0
34	1047	0
34	3028	0
34	3368	0
34	2857	0
34	1093	0
35	119	1
35	293	1
35	118	1
35	55	1
35	871	0
35	2320	0
35	107	0
35	2166	0
36	320	1
36	321	1
36	322	1
36	323	1
36	324	1
36	325	1

36	6	1
36	326	1
36	327	1
36	328	1
36	319	1
36	159	0
36	350	0
36	1363	0
36	2580	0
36	459	0
36	2901	0
36	2239	0
36	1815	0
36	1552	0
36	2688	0
36	558	0
37	329	1
37	330	1
37	331	1
37	332	1
37	333	1
37	334	1
37	15	1
37	335	1
37	336	1
37	337	1
37	338	1
37	339	1
37	340	1
37	341	1

37	342	1
37	344	1
37	345	1
37	343	1
37	3496	0
37	1597	0
37	1081	0
37	3285	0
37	3543	0
37	2901	0
37	2983	0
37	1492	0
37	724	0
37	1051	0
37	2289	0
37	813	0
37	2914	0
37	1037	0
37	280	0
37	1606	0
37	199	0
37	3759	0
38	346	1
38	241	1
38	250	1
38	347	1
38	348	1
38	349	1
38	350	1
38	351	1

38	3112	0
38	1316	0
38	2268	0
38	357	0
38	1992	0
38	3784	0
38	2129	0
38	1332	0
39	96	1
39	352	1
39	353	1
39	354	1
39	355	1
39	133	1
39	41	1
39	45	1
39	55	1
39	252	1
39	3780	0
39	2118	0
39	2504	0
39	1847	0
39	3156	0
39	884	0
39	3022	0
39	3026	0
39	3375	0
39	848	0
40	96	1
40	196	1

40	261	1
40	70	1
40	356	1
40	357	1
40	358	1
40	359	1
40	360	1
40	361	1
40	46	1
40	80	1
40	22	1
40	118	1
40	24	1
40	155	1
40	3110	0
40	366	0
40	2781	0
40	2865	0
40	36	0
40	1360	0
40	2224	0
40	2024	0
40	2951	0
40	2957	0
40	738	0
40	1	0
40	1752	0
40	3823	0
40	2336	0
40	1511	0

41	34	1
41	36	1
41	7	1
41	362	1
41	109	1
41	250	1
41	191	1
41	2957	0
41	1450	0
41	1592	0
41	238	0
41	2828	0
41	779	0
41	354	0
42	135	1
42	74	1
42	171	1
42	202	1
42	204	1
42	363	1
42	79	1
42	364	1
42	81	1
42	365	1
42	366	1
42	367	1
42	368	1
42	369	1
42	87	1
42	370	1

42	371	1
42	372	1
42	3819	0
42	1488	0
42	1996	0
42	3568	0
42	1870	0
42	3414	0
42	3178	0
42	2875	0
42	3154	0
42	1225	0
42	3551	0
42	1566	0
42	1648	0
42	2127	0
42	447	0
42	38	0
42	1541	0
42	2523	0
43	376	1
43	134	1
43	171	1
43	363	1
43	79	1
43	80	1
43	373	1
43	374	1
43	87	1
43	184	1

43	185	1
43	375	1
43	1125	0
43	597	0
43	161	0
43	3245	0
43	547	0
43	2710	0
43	3040	0
43	859	0
43	2881	0
43	136	0
43	2504	0
43	2856	0
44	377	1
44	74	1
44	77	1
44	79	1
44	115	1
44	184	1
44	185	1
44	378	1
44	254	1
44	127	1
44	46	0
44	3318	0
44	1427	0
44	1355	0
44	2882	0
44	2240	0

44	1220	0
44	3537	0
44	131	0
44	2859	0
45	66	1
45	145	1
45	381	1
45	52	1
45	379	1
45	380	1
45	61	1
45	254	1
45	1184	0
45	3583	0
45	1820	0
45	2352	0
45	676	0
45	2727	0
45	408	0
45	2222	0
46	384	1
46	385	1
46	386	1
46	73	1
46	74	1
46	78	1
46	16	1
46	54	1
46	382	1
46	383	1

46	1226	0
46	1288	0
46	754	0
46	2499	0
46	460	0
46	3608	0
46	3298	0
46	3769	0
46	3216	0
46	1316	0
47	387	1
47	36	1
47	37	1
47	293	1
47	356	1
47	388	1
47	105	1
47	389	1
47	390	1
47	391	1
47	392	1
47	46	1
47	52	1
47	55	1
47	187	1
47	21	0
47	1369	0
47	2131	0
47	2444	0
47	1844	0

47	1983	0
47	1113	0
47	2625	0
47	2682	0
47	2933	0
47	1979	0
47	674	0
47	3116	0
47	2796	0
47	2683	0
48	36	1
48	325	1
48	393	1
48	42	1
48	394	1
48	395	1
48	209	1
48	242	1
48	346	1
48	2091	0
48	1694	0
48	982	0
48	595	0
48	1383	0
48	1201	0
48	739	0
48	2925	0
48	2865	0
49	325	1
49	396	1

49	397	1
49	398	1
49	399	1
49	208	1
49	400	1
49	401	1
49	402	1
49	403	1
49	119	1
49	218	1
49	83	0
49	3729	0
49	950	0
49	2330	0
49	495	0
49	3657	0
49	3597	0
49	3224	0
49	3202	0
49	3823	0
49	3126	0
49	195	0
50	98	1
50	130	1
50	132	1
50	72	1
50	74	1
50	404	1
50	87	1
50	120	1

50	127	1
50	914	0
50	1558	0
50	174	0
50	1027	0
50	3407	0
50	3695	0
50	3811	0
50	1870	0
50	3376	0
51	407	1
51	132	1
51	133	1
51	72	1
51	74	1
51	237	1
51	405	1
51	406	1
51	87	1
51	408	1
51	409	1
51	410	1
51	127	1
51	2915	0
51	1730	0
51	2394	0
51	3471	0
51	2335	0
51	3566	0
51	3388	0

51	34	0
51	2142	0
51	3246	0
51	1476	0
51	2888	0
51	3410	0
52	411	1
52	412	1
52	413	1
52	414	1
52	415	1
52	416	1
52	417	1
52	418	1
52	419	1
52	420	1
52	421	1
52	422	1
52	423	1
52	424	1
52	425	1
52	426	1
52	52	1
52	70	1
52	376	1
52	289	0
52	176	0
52	3826	0
52	2214	0
52	189	0

52	2274	0
52	919	0
52	2374	0
52	1847	0
52	2722	0
52	1382	0
52	124	0
52	159	0
52	2139	0
52	241	0
52	1846	0
52	2535	0
52	2964	0
52	303	0
53	37	1
53	391	1
53	105	1
53	427	1
53	428	1
53	109	1
53	46	1
53	429	1
53	430	1
53	55	1
53	1121	0
53	3627	0
53	1138	0
53	3158	0
53	231	0
53	1634	0

53	2679	0
53	1094	0
53	695	0
53	220	0
54	96	1
54	105	1
54	364	1
54	46	1
54	302	1
54	303	1
54	431	1
54	432	1
54	118	1
54	185	1
54	2679	0
54	2959	0
54	1097	0
54	2641	0
54	1074	0
54	1846	0
54	975	0
54	1352	0
54	1193	0
54	3613	0
55	98	1
55	232	1
55	377	1
55	206	1
55	79	1
55	433	1

55	434	1
55	435	1
55	436	1
55	437	1
55	184	1
55	185	1
55	1	0
55	2897	0
55	1778	0
55	2017	0
55	3367	0
55	554	0
55	1183	0
55	1904	0
55	2093	0
55	1687	0
55	1774	0
55	294	0
56	96	1
56	448	1
56	100	1
56	293	1
56	446	1
56	72	1
56	443	1
56	438	1
56	439	1
56	440	1
56	441	1
56	442	1

56	155	1
56	444	1
56	445	1
56	286	1
56	447	1
56	2487	0
56	1412	0
56	1720	0
56	823	0
56	3072	0
56	2722	0
56	2406	0
56	1900	0
56	3093	0
56	1971	0
56	1386	0
56	1355	0
56	3550	0
56	1829	0
56	900	0
56	2154	0
56	137	0
57	158	1
57	33	1
57	45	1
57	449	1
57	450	1
57	451	1
57	452	1
57	197	1

57	453	1
57	454	1
57	455	1
57	456	1
57	457	1
57	458	1
57	459	1
57	460	1
57	461	1
57	462	1
57	463	1
57	464	1
57	465	1
57	3003	0
57	1528	0
57	3294	0
57	899	0
57	1387	0
57	1815	0
57	19	0
57	2267	0
57	2600	0
57	2252	0
57	3737	0
57	1460	0
57	1680	0
57	1522	0
57	58	0
57	2646	0
57	1381	0

57	3403	0
57	1293	0
57	1427	0
57	1038	0
58	36	1
58	165	1
58	466	1
58	467	1
58	468	1
58	469	1
58	470	1
58	471	1
58	472	1
58	473	1
58	61	1
58	1265	0
58	1464	0
58	3618	0
58	640	0
58	223	0
58	2905	0
58	2107	0
58	2236	0
58	3290	0
58	1880	0
58	2450	0
59	477	1
59	354	1
59	74	1
59	43	1

59	52	1
59	24	1
59	474	1
59	475	1
59	476	1
59	445	1
59	478	1
59	479	1
59	3786	0
59	1745	0
59	3586	0
59	3167	0
59	1128	0
59	3161	0
59	2599	0
59	468	0
59	2929	0
59	2585	0
59	2000	0
59	297	0
60	480	1
60	481	1
60	36	1
60	40	1
60	141	1
60	242	1
60	2143	0
60	1994	0
60	1791	0
60	1741	0

60	871	0
60	985	0
61	482	1
61	67	1
61	356	1
61	484	1
61	483	1
61	485	1
61	486	1
61	487	1
61	488	1
61	489	1
61	490	1
61	491	1
61	492	1
61	2156	0
61	3430	0
61	897	0
61	3349	0
61	3542	0
61	3299	0
61	300	0
61	2797	0
61	748	0
61	469	0
61	1166	0
61	2473	0
61	2672	0
62	96	1
62	493	1

62	494	1
62	47	1
62	208	1
62	495	1
62	210	1
62	119	1
62	3500	0
62	83	0
62	310	0
62	1791	0
62	1173	0
62	3619	0
62	1772	0
62	953	0
63	257	1
63	227	1
63	260	1
63	503	1
63	487	1
63	496	1
63	497	1
63	498	1
63	275	1
63	499	1
63	500	1
63	501	1
63	502	1
63	26	1
63	1344	0
63	3654	0

63	3544	0
63	1715	0
63	3309	0
63	2782	0
63	320	0
63	675	0
63	3653	0
63	1158	0
63	149	0
63	1367	0
63	2280	0
63	1599	0
64	128	1
64	130	1
64	135	1
64	73	1
64	77	1
64	80	1
64	81	1
64	87	1
64	58	1
64	507	1
64	2691	0
64	3297	0
64	2483	0
64	1532	0
64	3349	0
64	440	0
64	899	0
64	1102	0

64	25	0
64	273	0
65	128	1
65	512	1
65	513	1
65	514	1
65	515	1
65	516	1
65	517	1
65	518	1
65	519	1
65	520	1
65	521	1
65	155	1
65	156	1
65	419	1
65	422	1
65	67	1
65	508	1
65	509	1
65	510	1
65	511	1
65	3255	0
65	3344	0
65	998	0
65	3737	0
65	3833	0
65	2312	0
65	3808	0
65	1274	0

65 2001 0

65 2461 0

65 1134 0

65 2394 0

65 1849 0

65 3264 0

65 1027 0

65 3219 0

65 2806 0

65 675 0

65 3340 0

65 3012 0

66 36 1

66 295 1

66 392 1

66 522 1

66 107 1

66 523 1

66 524 1

66 46 1

66 525 1

66 526 1

66 527 1

66 528 1

66 529 1

66 530 1

66 55 1

66 60 1

66 785 0

66 1426 0

66	3045	0
66	1184	0
66	2924	0
66	124	0
66	2548	0
66	940	0
66	3113	0
66	2075	0
66	620	0
66	1819	0
66	3753	0
66	1895	0
66	2909	0
66	50	0
67	535	1
67	42	1
67	241	1
67	242	1
67	531	1
67	532	1
67	245	1
67	533	1
67	534	1
67	536	1
67	537	1
67	250	1
67	285	1
67	1241	0
67	3335	0
67	388	0

67	3749	0
67	1755	0
67	2862	0
67	3365	0
67	2919	0
67	3352	0
67	911	0
67	3330	0
67	1212	0
67	1802	0
68	66	1
68	98	1
68	72	1
68	74	1
68	80	1
68	117	1
68	22	1
68	185	1
68	538	1
68	539	1
68	540	1
68	254	1
68	127	1
68	1954	0
68	1598	0
68	1009	0
68	2176	0
68	3291	0
68	1447	0
68	1707	0

68	278	0
68	892	0
68	3260	0
68	384	0
68	2943	0
68	2412	0
69	544	1
69	257	1
69	541	1
69	545	1
69	546	1
69	542	1
69	331	1
69	441	1
69	13	1
69	275	1
69	25	1
69	28	1
69	221	1
69	446	1
69	543	1
69	2482	0
69	1524	0
69	2612	0
69	1021	0
69	1726	0
69	2321	0
69	2488	0
69	29	0
69	3101	0

69	3106	0
69	3	0
69	3556	0
69	2602	0
69	51	0
69	649	0
70	547	1
70	548	1
70	549	1
70	550	1
70	551	1
70	552	1
70	553	1
70	554	1
70	171	1
70	555	1
70	556	1
70	557	1
70	558	1
70	559	1
70	126	1
70	949	0
70	705	0
70	3397	0
70	2362	0
70	1629	0
70	2744	0
70	2937	0
70	2858	0
70	3517	0

70	2444	0
70	3030	0
70	3533	0
70	1151	0
70	3308	0
70	644	0
71	96	1
71	130	1
71	133	1
71	102	1
71	135	1
71	171	1
71	47	1
71	560	1
71	561	1
71	374	1
71	376	1
71	254	1
71	2801	0
71	2881	0
71	15	0
71	557	0
71	1234	0
71	3126	0
71	3140	0
71	1275	0
71	553	0
71	759	0
71	2030	0
71	702	0

72	99	1
72	562	1
72	563	1
72	564	1
72	565	1
72	566	1
72	93	1
72	95	1
72	744	0
72	1759	0
72	3354	0
72	1398	0
72	419	0
72	3825	0
72	3198	0
72	853	0
73	133	1
73	391	1
73	40	1
73	105	1
73	136	1
73	107	1
73	46	1
73	528	1
73	567	1
73	568	1
73	569	1
73	570	1
73	571	1
73	252	1

73 573 1

73 572 1

73 2989 0

73 3100 0

73 2181 0

73 1596 0

73 2152 0

73 2930 0

73 1348 0

73 2057 0

73 1266 0

73 109 0

73 1818 0

73 3186 0

73 3333 0

73 3726 0

73 3804 0

73 1193 0

74 576 1

74 577 1

74 578 1

74 579 1

74 580 1

74 581 1

74 582 1

74 460 1

74 119 1

74 314 1

74 155 1

74 220 1

74	574	1
74	575	1
74	2	0
74	2557	0
74	2027	0
74	952	0
74	2316	0
74	1869	0
74	810	0
74	1270	0
74	2028	0
74	3308	0
74	3551	0
74	1604	0
74	3768	0
74	2682	0
75	134	1
75	583	1
75	584	1
75	75	1
75	76	1
75	206	1
75	79	1
75	399	1
75	55	1
75	2392	0
75	1305	0
75	1477	0
75	645	0
75	2790	0

75	3652	0
75	2667	0
75	2459	0
75	1030	0
76	199	1
76	585	1
76	586	1
76	587	1
76	588	1
76	589	1
76	303	1
76	208	1
76	212	1
76	2779	0
76	2055	0
76	1762	0
76	397	0
76	1875	0
76	198	0
76	270	0
76	923	0
76	3711	0
77	70	1
77	590	1
77	591	1
77	592	1
77	593	1
77	594	1
77	595	1
77	596	1

77	597	1
77	598	1
77	599	1
77	600	1
77	601	1
77	602	1
77	603	1
77	604	1
77	605	1
77	606	1
77	1982	0
77	2589	0
77	1150	0
77	377	0
77	2508	0
77	3207	0
77	1797	0
77	1409	0
77	475	0
77	2555	0
77	3765	0
77	1076	0
77	3790	0
77	29	0
77	3000	0
77	2156	0
77	3719	0
77	1968	0
78	159	1
78	607	1

78	608	1
78	609	1
78	610	1
78	611	1
78	612	1
78	613	1
78	102	1
78	614	1
78	615	1
78	616	1
78	106	1
78	617	1
78	618	1
78	619	1
78	620	1
78	111	1
78	621	1
78	1677	0
78	2748	0
78	3343	0
78	2469	0
78	795	0
78	3325	0
78	2538	0
78	695	0
78	3213	0
78	3718	0
78	791	0
78	284	0
78	391	0

78	2437	0
78	1292	0
78	2853	0
78	1104	0
78	2998	0
78	3062	0
79	640	1
79	641	1
79	642	1
79	643	1
79	644	1
79	645	1
79	646	1
79	36	1
79	44	1
79	622	1
79	623	1
79	624	1
79	625	1
79	626	1
79	627	1
79	628	1
79	629	1
79	630	1
79	631	1
79	632	1
79	633	1
79	634	1
79	635	1
79	636	1

79	637	1
79	638	1
79	639	1
79	3110	0
79	3278	0
79	3439	0
79	1113	0
79	3248	0
79	3610	0
79	2060	0
79	2323	0
79	2535	0
79	118	0
79	3243	0
79	1073	0
79	2778	0
79	2420	0
79	50	0
79	1201	0
79	2263	0
79	2507	0
79	3454	0
79	1661	0
79	3791	0
79	1103	0
79	1744	0
79	1937	0
79	1865	0
79	1800	0
79	754	0

80	36	1
80	325	1
80	647	1
80	242	1
80	52	1
80	347	1
80	190	1
80	2014	0
80	2642	0
80	256	0
80	3339	0
80	2865	0
80	3657	0
80	2519	0
81	34	1
81	648	1
81	649	1
81	650	1
81	651	1
81	652	1
81	653	1
81	654	1
81	655	1
81	656	1
81	210	1
81	242	1
81	474	1
81	252	1
81	221	1
81	2255	0

81	2114	0
81	3039	0
81	2550	0
81	2826	0
81	2944	0
81	3084	0
81	582	0
81	3254	0
81	2087	0
81	2063	0
81	1658	0
81	2897	0
81	219	0
81	1696	0
82	96	1
82	208	1
82	432	1
82	657	1
82	658	1
82	212	1
82	213	1
82	659	1
82	218	1
82	220	1
82	125	1
82	2738	0
82	222	0
82	2594	0
82	3220	0
82	3020	0

82	1042	0
82	521	0
82	1343	0
82	981	0
82	3696	0
82	1822	0
83	66	1
83	132	1
83	74	1
83	81	1
83	115	1
83	372	1
83	660	1
83	661	1
83	87	1
83	662	1
83	185	1
83	127	1
83	204	0
83	1276	0
83	1537	0
83	2015	0
83	1489	0
83	3436	0
83	2301	0
83	1583	0
83	2121	0
83	2964	0
83	89	0
83	42	0

84	163	1
84	453	1
84	614	1
84	663	1
84	511	1
84	45	1
84	111	1
84	48	1
84	54	1
84	151	1
84	664	1
84	665	1
84	666	1
84	667	1
84	668	1
84	669	1
84	670	1
84	159	1
84	2716	0
84	2383	0
84	596	0
84	1910	0
84	3045	0
84	3275	0
84	3680	0
84	3245	0
84	2290	0
84	1623	0
84	3264	0
84	2526	0

84	3107	0
84	3323	0
84	2096	0
84	190	0
84	2966	0
84	3159	0
85	96	1
85	267	1
85	301	1
85	46	1
85	47	1
85	303	1
85	671	1
85	118	1
85	55	1
85	91	1
85	127	1
85	2805	0
85	340	0
85	128	0
85	1671	0
85	3552	0
85	2159	0
85	3009	0
85	3461	0
85	2824	0
85	123	0
85	3134	0
86	672	1
86	673	1

86 674 1

86 675 1

86 133 1

86 3210 0

86 2899 0

86 2308 0

86 379 0

86 3677 0

87 275 1

87 541 1

87 546 1

87 164 1

87 676 1

87 677 1

87 678 1

87 679 1

87 680 1

87 681 1

87 682 1

87 683 1

87 684 1

87 685 1

87 686 1

87 687 1

87 688 1

87 689 1

87 307 1

87 224 1

87 2993 0

87 2616 0

87	3727	0
87	1351	0
87	3596	0
87	2651	0
87	301	0
87	3019	0
87	1141	0
87	107	0
87	3404	0
87	516	0
87	2874	0
87	96	0
87	738	0
87	3636	0
87	1485	0
87	2840	0
87	604	0
87	2057	0
88	98	1
88	72	1
88	74	1
88	80	1
88	690	1
88	691	1
88	692	1
88	693	1
88	535	1
88	184	1
88	18	0
88	1256	0

88	78	0
88	3750	0
88	2996	0
88	1297	0
88	3649	0
88	818	0
88	3322	0
88	83	0
89	516	1
89	105	1
89	111	1
89	53	1
89	695	1
89	696	1
89	697	1
89	670	1
89	223	1
89	3696	0
89	661	0
89	3620	0
89	3673	0
89	1983	0
89	2517	0
89	1598	0
89	161	0
89	1709	0
90	700	1
90	134	1
90	135	1
90	74	1

90	77	1
90	206	1
90	118	1
90	87	1
90	184	1
90	185	1
90	698	1
90	699	1
90	572	1
90	127	1
90	2367	0
90	539	0
90	1063	0
90	2819	0
90	49	0
90	868	0
90	179	0
90	2958	0
90	2617	0
90	1817	0
90	3190	0
90	477	0
90	205	0
90	2290	0
91	133	1
91	74	1
91	399	1
91	81	1
91	52	1
91	87	1

91	26	1
91	701	1
91	702	1
91	443	0
91	1785	0
91	2371	0
91	1916	0
91	815	0
91	2073	0
91	3324	0
91	1698	0
91	1757	0
92	704	1
92	705	1
92	610	1
92	706	1
92	707	1
92	197	1
92	165	1
92	708	1
92	709	1
92	710	1
92	711	1
92	107	1
92	523	1
92	46	1
92	208	1
92	703	1
92	575	1
92	3594	0

92	504	0
92	2204	0
92	777	0
92	10	0
92	2160	0
92	865	0
92	835	0
92	1947	0
92	3702	0
92	1298	0
92	1305	0
92	1991	0
92	2360	0
92	1419	0
92	210	0
92	270	0
93	78	1
93	16	1
93	80	1
93	87	1
93	538	1
93	127	1
93	2707	0
93	1401	0
93	3718	0
93	2142	0
93	3505	0
93	3494	0
94	96	1
94	354	1

94	708	1
94	357	1
94	105	1
94	45	1
94	112	1
94	55	1
94	187	1
94	572	1
94	445	1
94	2988	0
94	457	0
94	1286	0
94	3347	0
94	604	0
94	3635	0
94	3368	0
94	2996	0
94	1283	0
94	3346	0
94	2061	0
95	354	1
95	133	1
95	37	1
95	70	1
95	712	1
95	686	1
95	55	1
95	221	1
95	1284	0
95	2611	0

95	2648	0
95	1199	0
95	2422	0
95	2204	0
95	2341	0
95	2771	0
96	96	1
96	102	1
96	199	1
96	713	1
96	714	1
96	267	1
96	78	1
96	47	1
96	303	1
96	52	1
96	184	1
96	2166	0
96	2686	0
96	3320	0
96	2695	0
96	2746	0
96	1177	0
96	791	0
96	1126	0
96	1725	0
96	630	0
96	1520	0
97	608	1
97	67	1

97	715	1
97	716	1
97	717	1
97	718	1
97	719	1
97	154	1
97	670	1
97	214	0
97	1750	0
97	3538	0
97	1036	0
97	2756	0
97	3316	0
97	2888	0
97	3813	0
97	1571	0
98	96	1
98	133	1
98	46	1
98	720	1
98	721	1
98	498	1
98	275	1
98	55	1
98	24	1
98	445	1
98	287	1
98	3492	0
98	755	0
98	142	0

98	3436	0
98	2372	0
98	2720	0
98	811	0
98	1554	0
98	667	0
98	1619	0
98	115	0
99	325	1
99	391	1
99	42	1
99	302	1
99	78	1
99	305	1
99	722	1
99	723	1
99	724	1
99	725	1
99	726	1
99	188	1
99	3794	0
99	416	0
99	239	0
99	743	0
99	1823	0
99	2307	0
99	1867	0
99	1881	0
99	3398	0
99	111	0

99 1191 0

99 191 0

100 732 1

100 196 1

100 197 1

100 198 1

100 519 1

100 582 1

100 733 1

100 460 1

100 689 1

100 731 1

100 727 1

100 728 1

100 729 1

100 314 1

100 155 1

100 156 1

100 509 1

100 730 1

100 1821 0

100 3706 0

100 3623 0

100 3739 0

100 52 0

100 1339 0

100 1315 0

100 168 0

100 1279 0

100 2601 0

100 1349 0

100 602 0

100 1293 0

100 2612 0

100 2979 0

100 2446 0

100 1040 0

100 1854 0

101 736 1

101 737 1

101 291 1

101 102 1

101 391 1

101 155 1

101 733 1

101 734 1

101 735 1

101 2445 0

101 561 0

101 1715 0

101 1786 0

101 2805 0

101 1923 0

101 1416 0

101 224 0

101 3204 0

102 97 1

102 545 1

102 738 1

102 739 1

102 325 1

102 740 1

102 242 1

102 52 1

102 151 1

102 250 1

102 475 1

102 3050 0

102 2707 0

102 2750 0

102 88 0

102 1757 0

102 996 0

102 3544 0

102 237 0

102 2140 0

102 2186 0

102 3186 0

103 7 1

103 393 1

103 12 1

103 34 1

103 42 1

103 315 1

103 325 1

103 741 1

103 742 1

103 743 1

103 744 1

103 745 1

103 746 1

103 747 1

103 748 1

103 749 1

103 750 1

103 751 1

103 752 1

103 753 1

103 1863 0

103 573 0

103 2025 0

103 1584 0

103 3310 0

103 1372 0

103 329 0

103 660 0

103 2531 0

103 2637 0

103 2602 0

103 2059 0

103 406 0

103 2723 0

103 2661 0

103 97 0

103 1112 0

103 3631 0

103 3131 0

103 2899 0

104 105 1

104 528 1

104 754 1

104 755 1

104 756 1

104 757 1

104 614 0

104 102 0

104 3461 0

104 864 0

104 3442 0

104 886 0

105 96 1

105 66 1

105 99 1

105 101 1

105 759 1

105 78 1

105 79 1

105 760 1

105 566 1

105 87 1

105 758 1

105 761 1

105 762 1

105 93 1

105 3685 0

105 952 0

105 3831 0

105 1979 0

105 3118 0

105 2114 0

105 2087 0

105 700 0

105 1041 0

105 1217 0

105 3842 0

105 1849 0

105 3738 0

105 2649 0

106 768 1

106 769 1

106 770 1

106 100 1

106 107 1

106 46 1

106 528 1

106 767 1

106 155 1

106 764 1

106 765 1

106 766 1

106 763 1

106 2172 0

106 3218 0

106 3686 0

106 2889 0

106 257 0

106 2358 0

106 2555 0

106 3681 0

106 94 0

106 2312 0

106 1172 0

106 969 0

106 2080 0

107 96 1

107 771 1

107 772 1

107 773 1

107 774 1

107 775 1

107 776 1

107 52 1

107 245 1

107 250 1

107 124 1

107 285 1

107 2308 0

107 261 0

107 2810 0

107 2323 0

107 85 0

107 2566 0

107 2453 0

107 940 0

107 374 0

107 3150 0

107 3331 0

107 1791 0

108 36 1

108 40 1

108 105 1

108 777 1

108 191 1

108 3787 0

108 3445 0

108 1767 0

108 1751 0

108 377 0

109 774 1

109 778 1

109 779 1

109 780 1

109 781 1

109 782 1

109 783 1

109 784 1

109 785 1

109 786 1

109 787 1

109 788 1

109 3430 0

109 3292 0

109 2961 0

109 2861 0

109 3351 0

109 2999 0

109 3209 0

109 1057 0

109 1862 0

109 26 0

109 510 0

109 3028 0

110 128 1

110 795 1

110 418 1

110 419 1

110 794 1

110 797 1

110 789 1

110 790 1

110 791 1

110 792 1

110 601 1

110 793 1

110 507 1

110 796 1

110 157 1

110 62 1

110 3272 0

110 3585 0

110 3361 0

110 3243 0

110 2476 0

110 2417 0

110 155 0

110 0 0

110 2490 0

110 349 0

110 2817 0

110 59 0

110 2809 0

110 2991 0

110 1757 0

110 2088 0

111 96 1

111 257 1

111 259 1

111 798 1

111 499 1

111 116 1

111 799 1

111 28 1

111 542 1

111 191 1

111 1854 0

111 3376 0

111 2300 0

111 3133 0

111 2630 0

111 3822 0

111 180 0

111 3523 0

111 99 0

111 771 0

112 96 1

112 800 1

112 801 1

112 802 1

112 493 1

112 216 1

112 477 1

112 1768 0

112 376 0

112 180 0

112 521 0

112 1436 0

112 2884 0

112 541 0

113 289 1

113 34 1

113 803 1

113 804 1

113 805 1

113 806 1

113 807 1

113 42 1

113 459 1

113 588 1

113 655 1

113 209 1

113 242 1

113 211 1

113 531 1

113 245 1

113 285 1

113 1691 0

113 2303 0

113 3608 0

113 371 0

113 2542 0

113 2096 0

113 1141 0

113 3181 0

113 195 0

113 888 0

113 3216 0

113 1031 0

113 1355 0

113 1675 0

113 852 0

113 819 0

113 3056 0

114 96 1

114 352 1

114 135 1

114 808 1

114 137 1

114 809 1

114 270 1

114 399 1

114 81 1

114 118 1

114 377 1

114 2714 0

114 3154 0

114 1853 0

114 2129 0

114 3577 0

114 3530 0

114 3623 0

114 2328 0

114 565 0

114 287 0

114 1960 0

115 96 1

115 134 1

115 171 1

115 364 1

115 560 1

115 126 1

115 2520 0

115 2157 0

115 1055 0

115 3473 0

115 3740 0

115 3748 0

116 160 1

116 810 1

116 811 1

116 812 1

116 813 1

116 814 1

116 815 1

116 816 1

116 817 1

116 818 1

116 819 1

116 148 1

116 820 1

116 150 1

116 145 0

116 1622 0

116 1107 0

116 1198 0

116 2721 0

116 2361 0

116 3663 0

116 1553 0

116 3711 0

116 3491 0

116 2892 0

116 3282 0

116 520 0

116 1455 0

117 528 1

117 24 1

117 821 1

117 822 1

117 823 1

117 824 1

117 825 1

117 826 1

117 827 1

117 187 1

117 703 1

117 708 1

117 199 1

117 208 1

117 210 1

117 361 1

117 111 1

117 119 1

117 767 1

117 1629 0

117 1518 0

117 1247 0

117 2859 0

117 3402 0

117 3048 0

117 686 0

117 3 0

117 124 0

117 865 0

117 1196 0

117 2953 0

117 1612 0

117 3285 0

117 2890 0

117 2900 0

117 2624 0

117 1421 0

117 397 0

118 96 1

118 100 1

118 133 1

118 493 1

118 118 1

118 119 1

118 476 1

118 445 1

118 1122 0
118 462 0
118 2395 0
118 3125 0
118 894 0
118 2607 0
118 2917 0
118 1726 0
119 96 1
119 829 1
119 828 1
119 133 1
119 1345 0
119 1725 0
119 1338 0
119 2613 0
120 832 1
120 41 1
120 79 1
120 80 1
120 115 1
120 831 1
120 118 1
120 188 1
120 830 1
120 63 1
120 2060 0
120 1355 0
120 3629 0
120 2057 0

120 362 0
120 2236 0
120 2717 0
120 29 0
120 22 0
120 1214 0
121 833 1
121 834 1
121 835 1
121 836 1
121 754 1
121 756 1
121 118 1
121 504 1
121 201 0
121 2501 0
121 1462 0
121 413 0
121 3695 0
121 1905 0
121 1082 0
121 737 0
122 837 1
122 838 1
122 839 1
122 840 1
122 715 1
122 48 1
122 114 1
122 252 1

122	542	1
122	159	1
122	1850	0
122	2101	0
122	1607	0
122	897	0
122	2983	0
122	3541	0
122	183	0
122	217	0
122	2302	0
122	2367	0
123	66	1
123	72	1
123	104	1
123	137	1
123	377	1
123	841	1
123	237	1
123	842	1
123	79	1
123	185	1
123	378	1
123	91	1
123	254	1
123	127	1
123	1895	0
123	3387	0
123	2655	0
123	2343	0

123 1364 0

123 570 0

123 1467 0

123 846 0

123 2574 0

123 858 0

123 928 0

123 1837 0

123 1157 0

123 628 0

124 66 1

124 131 1

124 132 1

124 75 1

124 237 1

124 372 1

124 373 1

124 87 1

124 378 1

124 91 1

124 254 1

124 127 1

124 2981 0

124 988 0

124 910 0

124 3307 0

124 1258 0

124 2111 0

124 281 0

124 390 0

124 2779 0

124 1150 0

124 1299 0

124 1094 0

125 855 1

125 843 1

125 844 1

125 845 1

125 846 1

125 847 1

125 848 1

125 849 1

125 850 1

125 851 1

125 852 1

125 597 1

125 309 1

125 853 1

125 854 1

125 856 1

125 510 1

125 1539 0

125 650 0

125 700 0

125 2289 0

125 3182 0

125 1165 0

125 2133 0

125 1163 0

125 618 0

125 2843 0
125 522 0
125 2649 0
125 1609 0
125 488 0
125 3837 0
125 917 0
125 2653 0
126 130 1
126 131 1
126 376 1
126 261 1
126 134 1
126 135 1
126 74 1
126 206 1
126 79 1
126 87 1
126 184 1
126 185 1
126 127 1
126 1739 0
126 2349 0
126 107 0
126 1362 0
126 2284 0
126 583 0
126 3076 0
126 2397 0
126 1313 0

126 882 0

126 3634 0

126 1207 0

126 1194 0

127 130 1

127 135 1

127 75 1

127 171 1

127 364 1

127 206 1

127 79 1

127 81 1

127 118 1

127 87 1

127 184 1

127 377 1

127 254 1

127 865 0

127 31 0

127 2468 0

127 1506 0

127 1970 0

127 1787 0

127 82 0

127 330 0

127 1625 0

127 2970 0

127 1464 0

127 1558 0

127 2736 0

128	98	1
128	261	1
128	135	1
128	73	1
128	77	1
128	79	1
128	373	1
128	374	1
128	87	1
128	185	1
128	698	1
128	254	1
128	2200	0
128	2179	0
128	1896	0
128	2327	0
128	811	0
128	558	0
128	167	0
128	3707	0
128	305	0
128	1968	0
128	3435	0
128	3501	0
129	0	1
129	98	1
129	4	1
129	446	1
129	72	1
129	13	1

129 237 1

129 334 1

129 254 1

129 857 1

129 185 1

129 858 1

129 859 1

129 860 1

129 861 1

129 126 1

129 1167 0

129 3490 0

129 1928 0

129 866 0

129 3323 0

129 1246 0

129 3167 0

129 2050 0

129 3503 0

129 2902 0

129 1297 0

129 678 0

129 3679 0

129 2489 0

129 2502 0

129 3199 0

130 98 1

130 132 1

130 133 1

130 74 1

130 79 1

130 80 1

130 115 1

130 87 1

130 377 1

130 539 1

130 862 1

130 2207 0

130 2241 0

130 219 0

130 3057 0

130 1888 0

130 3594 0

130 898 0

130 3086 0

130 263 0

130 824 0

130 1389 0

131 864 1

131 472 1

131 399 1

131 22 1

131 863 1

131 24 1

131 61 1

131 127 1

131 2891 0

131 3187 0

131 3536 0

131 3328 0

131 591 0

131 2632 0

131 2319 0

131 2585 0

132 96 1

132 870 1

132 871 1

132 872 1

132 873 1

132 874 1

132 427 1

132 242 1

132 55 1

132 118 1

132 119 1

132 126 1

132 863 0

132 3664 0

132 2782 0

132 2927 0

132 1326 0

132 2736 0

132 1393 0

132 2317 0

132 1163 0

132 1398 0

132 2444 0

132 3417 0

133 516 1

133 293 1

133 105 1

133 875 1

133 876 1

133 877 1

133 46 1

133 878 1

133 48 1

133 54 1

133 57 1

133 154 1

133 159 1

133 3719 0

133 2504 0

133 33 0

133 1294 0

133 1791 0

133 1725 0

133 632 0

133 3749 0

133 2816 0

133 3337 0

133 2578 0

133 353 0

133 805 0

134 450 1

134 36 1

134 879 1

134 880 1

134 881 1

134 221 1

134 743 0

134 2619 0

134 844 0

134 624 0

134 3460 0

134 1553 0

135 161 1

135 257 1

135 227 1

135 617 1

135 686 1

135 887 1

135 882 1

135 307 1

135 883 1

135 277 1

135 884 1

135 885 1

135 886 1

135 888 1

135 890 1

135 891 1

135 889 1

135 799 1

135 821 0

135 3446 0

135 1709 0

135 2758 0

135 1286 0

135 1188 0

135 877 0

135 2472 0

135 2246 0

135 133 0

135 2905 0

135 1489 0

135 2306 0

135 2387 0

135 1358 0

135 2702 0

135 2999 0

135 3806 0

136 896 1

136 895 1

136 90 1

136 892 1

136 893 1

136 894 1

136 191 1

136 1078 0

136 330 0

136 1747 0

136 3003 0

136 989 0

136 2693 0

136 2394 0

137 897 1

137 898 1

137 899 1

137 77 1

137 80 1

137 81 1

137 87 1

137 127 1

137 3373 0

137 3241 0

137 1276 0

137 1951 0

137 1753 0

137 3837 0

137 1933 0

137 2400 0

138 832 1

138 900 1

138 389 1

138 137 1

138 777 1

138 75 1

138 76 1

138 45 1

138 78 1

138 267 1

138 115 1

138 2624 0

138 578 0

138 3333 0

138 3009 0

138 1251 0

138 1639 0

138 1570 0

138 1369 0

138 2593 0

138 61 0

138 3045 0

139 98 1

139 135 1

139 75 1

139 759 1

139 79 1

139 373 1

139 566 1

139 87 1

139 758 1

139 2381 0

139 2726 0

139 1701 0

139 1431 0

139 3254 0

139 1851 0

139 3653 0

139 2104 0

139 3048 0

140 133 1

140 134 1

140 901 1

140 902 1

140 74 1

140 237 1

140 79 1

140 47 1

140 87 1

140 254 1

140 127 1

140 1579 0

140 158 0

140 2512 0

140 554 0

140 360 0

140 3783 0

140 73 0

140 561 0

140 1577 0

140 1767 0

140 3607 0

141 903 1

141 904 1

141 905 1

141 906 1

141 907 1

141 908 1

141 909 1

141 910 1

141 911 1

141 912 1

141 913 1

141 914 1

141 157 1

141 286 1

141 165 1

141 733 1

141 361 1

141 109 1

141 508 1

141 564 0

141 2892 0

141 2473 0

141 3803 0

141 3444 0

141 448 0

141 2899 0

141 3013 0

141 2498 0

141 1526 0

141 3131 0

141 3636 0

141 1496 0

141 1363 0

141 2259 0

141 163 0

141 3482 0

141 739 0

141 849 0

142 772 1

142 109 1

142 915 1

142 118 1

142 254 1

142 699 1

142 126 1

142 2693 0

142 603 0
142 1655 0
142 1103 0
142 2484 0
142 35 0
142 173 0
143 66 1
143 171 1
143 79 1
143 916 1
143 917 1
143 87 1
143 377 1
143 699 1
143 2331 0
143 1518 0
143 3795 0
143 904 0
143 2794 0
143 3709 0
143 2630 0
143 350 0
144 134 1
144 41 1
144 45 1
144 80 1
144 560 1
144 917 1
144 918 1
144 699 1

144 188 1

144 2744 0

144 3462 0

144 2203 0

144 21 0

144 711 0

144 3002 0

144 293 0

144 130 0

144 2500 0

145 772 1

145 102 1

145 78 1

145 46 1

145 79 1

145 206 1

145 52 1

145 919 1

145 920 1

145 921 1

145 922 1

145 923 1

145 2955 0

145 1999 0

145 108 0

145 1493 0

145 2608 0

145 1657 0

145 3789 0

145 791 0

145 2393 0

145 359 0

145 1206 0

145 3214 0

146 928 1

146 929 1

146 930 1

146 35 1

146 293 1

146 104 1

146 42 1

146 43 1

146 655 1

146 926 1

146 756 1

146 54 1

146 55 1

146 27 1

146 924 1

146 925 1

146 126 1

146 927 1

146 2869 0

146 103 0

146 3473 0

146 1477 0

146 3665 0

146 3083 0

146 2859 0

146 2389 0

146 3488 0
146 767 0
146 3248 0
146 2500 0
146 1791 0
146 2657 0
146 3070 0
146 2489 0
146 26 0
146 2821 0
147 931 1
147 102 1
147 7 1
147 8 1
147 871 1
147 302 1
147 47 1
147 17 1
147 242 1
147 564 1
147 118 1
147 126 1
147 1508 0
147 2923 0
147 892 0
147 1625 0
147 1480 0
147 1318 0
147 491 0
147 1796 0

147 668 0

147 2893 0

147 385 0

147 2941 0

148 932 1

148 933 1

148 934 1

148 935 1

148 936 1

148 937 1

148 938 1

148 939 1

148 940 1

148 941 1

148 942 1

148 943 1

148 944 1

148 945 1

148 277 1

148 2327 0

148 333 0

148 3022 0

148 652 0

148 3483 0

148 426 0

148 774 0

148 3261 0

148 775 0

148 1683 0

148 881 0

148 718 0

148 404 0

148 826 0

148 1814 0

149 36 1

149 37 1

149 133 1

149 391 1

149 109 1

149 46 1

149 946 1

149 52 1

149 469 0

149 392 0

149 2223 0

149 2864 0

149 645 0

149 1781 0

149 137 0

149 1432 0

150 36 1

150 805 1

150 947 1

150 948 1

150 949 1

150 55 1

150 1725 0

150 2787 0

150 2256 0

150 2126 0

150 787 0

150 325 0

151 832 1

151 954 1

151 98 1

151 900 1

151 955 1

151 953 1

151 137 1

151 75 1

151 206 1

151 79 1

151 238 1

151 373 1

151 950 1

151 951 1

151 184 1

151 185 1

151 90 1

151 952 1

151 1663 0

151 1929 0

151 3373 0

151 1005 0

151 3542 0

151 3705 0

151 3706 0

151 2013 0

151 2618 0

151 1543 0

151 1160 0

151 1368 0

151 2053 0

151 2010 0

151 1241 0

151 1243 0

151 3338 0

151 1896 0

152 96 1

152 352 1

152 66 1

152 385 1

152 72 1

152 137 1

152 74 1

152 43 1

152 268 1

152 237 1

152 81 1

152 52 1

152 116 1

152 24 1

152 27 1

152 956 1

152 957 1

152 3473 0

152 703 0

152 1352 0

152 3605 0

152 441 0

152 2464 0

152 2401 0

152 2073 0

152 3339 0

152 2380 0

152 1187 0

152 2303 0

152 1205 0

152 2344 0

152 2146 0

152 1040 0

152 3720 0

153 835 1

153 100 1

153 870 1

153 968 1

153 969 1

153 970 1

153 971 1

153 972 1

153 973 1

153 654 1

153 974 1

153 975 1

153 17 1

153 211 1

153 468 0

153 195 0

153 1020 0

153 2562 0

153 2200	0

153 819 0

153 1000	0

153 3248	0

153 3354	0

153 777 0

153 1811	0

153 3842	0

153 109 0

153 1085	0

154 900 1

154 134 1

154 79	1

154 976 1

154 721 1

154 977 1

154 978 1

154 472 1

154 863 1

154 1218	0

154 1900	0

154 1700	0

154 3709	0

154 2988	0

154 1697	0

154 2768	0

154 2561	0

154 3787	0

155 674 1

155 232 1

155 988 1

155 979 1

155 980 1

155 981 1

155 982 1

155 983 1

155 984 1

155 377 1

155 986 1

155 987 1

155 985 1

155 3056 0

155 2056 0

155 2696 0

155 999 0

155 1600 0

155 604 0

155 2311 0

155 2113 0

155 2758 0

155 1954 0

155 1023 0

155 1049 0

155 1783 0

156 34 1

156 42 1

156 45 1

156 495 1

156 990 1

156 154 1

156 989 1

156 286 1

156 159 1

156 2873 0

156 3082 0

156 701 0

156 1734 0

156 997 0

156 796 0

156 2797 0

156 750 0

156 2458 0

157 997 1

157 998 1

157 999 1

157 1000 1

157 44 1

157 527 1

157 879 1

157 529 1

157 54 1

157 475 1

157 221 1

157 2290 0

157 2944 0

157 3426 0

157 3624 0

157 2829 0

157 421 0

157 2966 0

157 1475 0

157 2039 0

157 1398 0

157 3663 0

158 513 1

158 516 1

158 517 1

158 159 1

158 163 1

158 422 1

158 187 1

158 196 1

158 708 1

158 1003 1

158 1004 1

158 1005 1

158 1006 1

158 111 1

158 1007 1

158 1008 1

158 1009 1

158 1010 1

158 508 1

158 509 1

158 2488 0

158 1595 0

158 1485 0

158 1592 0

158 2645 0

158 183 0

158 2311 0

158 1965 0

158 865 0

158 2281 0

158 975 0

158 3830 0

158 3602 0

158 3478 0

158 1022 0

158 2672 0

158 2696 0

158 1777 0

158 3582 0

158 2378 0

159 481 1

159 199 1

159 393 1

159 654 1

159 401 1

159 242 1

159 1012 1

159 1013 1

159 220 1

159 713 0

159 3575 0

159 590 0

159 312 0

159 385 0

159 1226 0

159 2169 0

159 1475 0

159 770 0

160 708 1

160 133 1

160 102 1

160 199 1

160 136 1

160 46 1

160 111 1

160 52 1

160 55 1

160 57 1

160 187 1

160 572 1

160 413 1

160 38 0

160 79 0

160 3384 0

160 436 0

160 947 0

160 3122 0

160 764 0

160 3563 0

160 1108 0

160 2791 0

160 3490 0

160 2021 0

160 308 0

161 608 1

161 36 1

161 356 1

161 357 1

161 199 1

161 72 1

161 105 1

161 361 1

161 107 1

161 46 1

161 1015 1

161 119 1

161 1014 1

161 55 1

161 187 1

161 765 1

161 190 1

161 226 0

161 624 0

161 2195 0

161 434 0

161 1857 0

161 1318 0

161 3462 0

161 1960 0

161 862 0

161 842 0

161 2643 0

161 2107 0

161 3262 0

161 3478 0

161 1085 0

161 1248 0

161 3736 0

162 36 1

162 102 1

162 105 1

162 109 1

162 46 1

162 302 1

162 918 1

162 1016 1

162 188 1

162 230 0

162 3080 0

162 2659 0

162 2547 0

162 2859 0

162 3751 0

162 187 0

162 350 0

162 3799 0

163 41 1

163 45 1

163 52 1

163 1017 1

163 1018 1

163 1019 1

163 2965 0

163 1050 0

163 2043 0

163 3535 0

163 503 0

163 340 0

164 674 1

164 66 1

164 473 1

164 979 1

164 377 1

164 2145 0

164 972 0

164 1692 0

164 174 0

164 2062 0

165 196 1

165 105 1

165 107 1

165 878 1

165 111 1

165 16 1

165 498 1

165 54 1

165 55 1

165 1020 1

165 799 1

165 504 0

165 3402 0

165 64 0

165 1627 0

165 1824 0

165 3330 0

165 1932 0

165 2233 0

165 758 0

165 2789 0

165 3705 0

166 289 1

166 34 1

166 930 1

166 100 1

166 36 1

166 42 1

166 362 1

166 109 1

166 654 1

166 657 1

166 1021 1

166 52 1

166 245 1

166 250 1

166 507 1

166 285 1

166 2884 0

166 656 0

166 2641 0

166 1385 0

166 1566 0

166 1143 0

166 966 0

166 1128 0

166 2971 0

166 648 0

166 2113 0

166 3206 0

166 2944 0

166 1239 0

166 268 0

166 347 0

167 1024 1

167 289 1

167 740 1

167 996 1

167 774 1

167 42 1

167 395 1

167 941 1

167 655 1

167 879 1

167 945 1

167 211 1

167 531 1

167 217 1

167 1022 1

167 1023 1

167 2983 0

167 1658 0

167 1834 0

167 3780 0

167 1877 0

167 1154 0

167 3093 0

167 2818 0

167 3715 0

167 2787 0

167 528 0

167 2161 0

167 3314 0

167 1244 0

167 2367 0

167 2390 0

168 96 1

168 1025 1

168 1026 1

168 808 1

168 528 1

168 118 1

168 89 1

168 765 1

168 2423 0

168 1625 0

168 164 0

168 3030 0

168 2143 0

168 1653 0

168 3732 0

168 2660 0

169 928 1

169 1027 1

169 35 1

169 197 1

169 1028 1

169 1029 1

169 1030 1
169 1031 1
169 1032 1
169 1033 1
169 1034 1
169 308 1
169 1864 0
169 3099 0
169 3298 0
169 2644 0
169 1557 0
169 2575 0
169 2935 0
169 1446 0
169 2695 0
169 3694 0
169 2750 0
169 3374 0
170 476 1
170 60 1
170 477 1
170 2147 0
170 2139 0
170 3126 0
171 548 1
171 1035 1
171 1036 1
171 1037 1
171 1038 1
171 1039 1

171 1040 1
171 1041 1
171 1042 1
171 1043 1
171 1044 1
171 893 1
171 1657 0
171 1893 0
171 1046 0
171 1121 0
171 3003 0
171 3328 0
171 2819 0
171 3592 0
171 2721 0
171 159 0
171 3471 0
171 3360 0
172 354 1
172 36 1
172 145 1
172 883 1
172 52 1
172 277 1
172 1045 1
172 1046 1
172 1047 1
172 252 1
172 445 1
172 286 1

172 3826 0

172 1287 0

172 1592 0

172 3569 0

172 1863 0

172 635 0

172 23 0

172 2797 0

172 1840 0

172 2449 0

172 1489 0

172 2792 0

173 34 1

173 36 1

173 104 1

173 883 1

173 1049 1

173 24 1

173 1048 1

173 1050 1

173 1051 1

173 126 1

173 3347 0

173 3792 0

173 3513 0

173 615 0

173 2152 0

173 1021 0

173 820 0

173 2776 0

173 927 0

173 2746 0

174 1056 1

174 257 1

174 1057 1

174 1058 1

174 1059 1

174 1060 1

174 650 1

174 271 1

174 1053 1

174 118 1

174 24 1

174 1052 1

174 221 1

174 1054 1

174 1055 1

174 1482 0

174 380 0

174 648 0

174 3291 0

174 1195 0

174 376 0

174 3106 0

174 3459 0

174 2613 0

174 51 0

174 3209 0

174 2089 0

174 432 0

174 280 0

174 114 0

175 96 1

175 1025 1

175 376 1

175 772 1

175 102 1

175 1061 1

175 841 1

175 714 1

175 52 1

175 184 1

175 126 1

175 127 1

175 2105 0

175 1081 0

175 3386 0

175 1765 0

175 1059 0

175 3305 0

175 3784 0

175 692 0

175 32 0

175 1417 0

175 1831 0

175 3715 0

176 257 1

176 34 1

176 483 1

176 36 1

176 1057 1

176 1062 1

176 879 1

176 498 1

176 23 1

176 28 1

176 191 1

176 190 0

176 2400 0

176 2792 0

176 2296 0

176 2662 0

176 1344 0

176 3213 0

176 1259 0

176 1043 0

176 3618 0

176 3837 0

177 96 1

177 1063 1

177 712 1

177 109 1

177 989 1

177 210 1

177 118 1

177 119 1

177 221 1

177 125 1

177 286 1

177 499 0

177 1745 0

177 1792 0

177 1220 0

177 3549 0

177 2549 0

177 610 0

177 2041 0

177 759 0

177 233 0

177 2714 0

178 1064 1

178 1065 1

178 1066 1

178 1067 1

178 1068 1

178 1069 1

178 1070 1

178 1071 1

178 276 1

178 798 1

178 1934 0

178 137 0

178 401 0

178 720 0

178 1876 0

178 1837 0

178 1039 0

178 3189 0

178 3844 0

178 2699 0

179 37 1
179 332 1
179 1072 1
179 1073 1
179 1074 1
179 20 1
179 245 1
179 52 1
179 474 1
179 956 1
179 445 1
179 2169 0
179 3143 0
179 212 0
179 3731 0
179 3792 0
179 480 0
179 194 0
179 1125 0
179 2919 0
179 1930 0
179 3844 0
180 1075 1
180 35 1
180 1076 1
180 1077 1
180 90 0
180 3118 0
180 2268 0
180 3715 0

181 131 1
181 36 1
181 357 1
181 1082 1
181 137 1
181 852 1
181 1078 1
181 1079 1
181 24 1
181 1081 1
181 1080 1
181 1083 1
181 1084 1
181 2699 0
181 3551 0
181 2186 0
181 3444 0
181 3315 0
181 1025 0
181 1618 0
181 752 0
181 2808 0
181 1607 0
181 1952 0
181 2530 0
181 762 0
182 36 1
182 456 1
182 462 1
182 1039 1

182 1087 1
182 22 1
182 854 1
182 56 1
182 474 1
182 1085 1
182 1086 1
182 223 1
182 2595 0
182 2855 0
182 2472 0
182 1925 0
182 263 0
182 3535 0
182 2755 0
182 1779 0
182 3501 0
182 1244 0
182 238 0
182 2343 0
183 1088 1
183 1089 1
183 1090 1
183 1091 1
183 36 1
183 388 1
183 105 1
183 141 1
183 52 1
183 151 1

183 2188 0

183 3229 0

183 3711 0

183 2573 0

183 2192 0

183 1151 0

183 1807 0

183 153 0

183 2744 0

183 2927 0

184 1092 1

184 133 1

184 293 1

184 1093 1

184 105 1

184 523 1

184 109 1

184 110 1

184 46 1

184 52 1

184 118 1

184 765 1

184 190 1

184 427 0

184 789 0

184 1084 0

184 416 0

184 2149 0

184 2844 0

184 2363 0

184 686 0
184 3449 0
184 2006 0
184 362 0
184 2801 0
184 3738 0
185 770 1
185 325 1
185 776 1
185 1096 1
185 1097 1
185 1098 1
185 1099 1
185 46 1
185 210 1
185 212 1
185 823 1
185 219 1
185 124 1
185 286 1
185 813 0
185 774 0
185 2420 0
185 87 0
185 2110 0
185 1330 0
185 467 0
185 695 0
185 2803 0
185 2695 0

185 729 0

185 1345 0

185 978 0

185 2552 0

186 36 1

186 357 1

186 1105 1

186 1106 1

186 1107 1

186 1108 1

186 55 1

186 442 1

186 30 1

186 191 1

186 169 0

186 1948 0

186 302 0

186 3119 0

186 3734 0

186 2586 0

186 3237 0

186 12 0

186 1996 0

186 1802 0

187 555 1

187 300 1

187 1109 1

187 1110 1

187 1111 1

187 1112 1

187 1113	1
187 1114	1
187 252 1
187 126 1
187 1428	0
187 346 0
187 1410	0
187 3020	0
187 1527	0
187 521 0
187 2146	0
187 926 0
187 3397	0
187 79	0
188 740 1
188 1115	1
188 505 1
188 242 1
188 217 1
188 219 1
188 1116	1
188 1117	1
188 446 1
188 1118	1
188 978 0
188 2188	0
188 1155	0
188 2857	0
188 3738	0
188 1396	0

188 1626 0

188 687 0

188 1284 0

188 3331 0

189 1120 1

189 1121 1

189 1122 1

189 1123 1

189 776 1

189 303 1

189 208 1

189 214 1

189 118 1

189 215 1

189 1119 1

189 1887 0

189 1380 0

189 3679 0

189 2874 0

189 2007 0

189 1345 0

189 246 0

189 521 0

189 1853 0

189 2165 0

189 548 0

190 1124 1

190 1125 1

190 1126 1

190 1127 1

190 1128 1

190 1129 1

190 1130 1

190 1131 1

190 1132 1

190 1133 1

190 1134 1

190 1135 1

190 305 1

190 1015 1

190 219 1

190 1377 0

190 3544 0

190 2868 0

190 472 0

190 2717 0

190 1855 0

190 642 0

190 739 0

190 147 0

190 3649 0

190 2239 0

190 3093 0

190 2812 0

190 801 0

190 814 0

191 97 1

191 577 1

191 770 1

191 199 1

191 1100 1

191 47 1

191 303 1

191 1136 1

191 1137 1

191 659 1

191 118 1

191 254 1

191 1205 0

191 437 0

191 844 0

191 2900 0

191 2754 0

191 2002 0

191 1436 0

191 2942 0

191 2753 0

191 2601 0

191 1822 0

191 273 0

192 46 1

192 1138 1

192 1139 1

192 1140 1

192 863 1

192 474 1

192 187 1

192 252 1

192 413 1

192 223 1

192 2170 0

192 586 0

192 227 0

192 341 0

192 1263 0

192 2863 0

192 1778 0

192 2454 0

192 2740 0

192 1891 0

193 382 1

193 72 1

193 906 1

193 302 1

193 378 1

193 305 1

193 1141 1

193 1142 1

193 1143 1

193 280 1

193 1145 1

193 90 1

193 1144 1

193 254 1

193 574 0

193 1513 0

193 3489 0

193 2021 0

193 2987 0

193 2444 0

193 1758 0

193 1208 0

193 369 0

193 2947 0

193 1649 0

193 869 0

193 2458 0

193 3663 0

194 1152 1

194 545 1

194 3 1

194 325 1

194 7 1

194 393 1

194 395 1

194 12 1

194 1146 1

194 1147 1

194 1148 1

194 1149 1

194 1150 1

194 1151 1

194 1943 0

194 2441 0

194 3781 0

194 3383 0

194 71 0

194 2979 0

194 1229 0

194 3666 0

194 2010 0
194 3591 0
194 732 0
194 3080 0
194 917 0
194 1122 0
195 96 1
195 1153 1
195 1154 1
195 133 1
195 74 1
195 75 1
195 171 1
195 79 1
195 47 1
195 81 1
195 560 1
195 115 1
195 118 1
195 87 1
195 127 1
195 1810 0
195 2449 0
195 3450 0
195 23 0
195 2454 0
195 1630 0
195 7 0
195 2084 0
195 2997 0

195 1703 0

195 2518 0

195 1208 0

195 1026 0

195 3592 0

195 3788 0

196 192 1

196 1155 1

196 1156 1

196 52 1

196 445 1

196 254 1

196 2913 0

196 1559 0

196 2823 0

196 518 0

196 667 0

196 974 0

197 129 1

197 4 1

197 1157 1

197 392 1

197 41 1

197 44 1

197 13 1

197 528 1

197 52 1

197 118 1

197 569 1

197 445 1

197 1081 0

197 3542 0

197 3188 0

197 3110 0

197 1993 0

197 1665 0

197 482 0

197 802 0

197 1819 0

197 1678 0

197 3756 0

197 641 0

198 1158 1

198 1159 1

198 1160 1

198 1161 1

198 1162 1

198 1163 1

198 1164 1

198 1165 1

198 1166 1

198 1167 1

198 532 1

198 535 1

198 536 1

198 806 1

198 347 1

198 242 1

198 243 1

198 245 1

198 250 1

198 942 0

198 2340 0

198 2055 0

198 1268 0

198 153 0

198 1752 0

198 1859 0

198 3006 0

198 763 0

198 2164 0

198 2736 0

198 3472 0

198 3282 0

198 1745 0

198 544 0

198 3194 0

198 1614 0

198 522 0

198 3454 0

199 96 1

199 36 1

199 581 1

199 70 1

199 102 1

199 46 1

199 52 1

199 119 1

199 212 1

199 55 1

199 597 1

199 60 1

199 156 0

199 498 0

199 2541 0

199 2096 0

199 1481 0

199 3416 0

199 677 0

199 111 0

199 1389 0

199 409 0

199 342 0

199 3504 0

200 897 1

200 171 1

200 87 1

200 1016 1

200 538 1

200 380 1

200 3121 0

200 2055 0

200 3730 0

200 913 0

200 3736 0

200 3604 0

201 1168 1

201 1169 1

201 1170 1

201 1171 1

201 1172 1

201 1173 1

201 1023 1

201 3252 0

201 2821 0

201 1644 0

201 486 0

201 1269 0

201 2728 0

201 291 0

202 359 1

202 295 1

202 231 1

202 957 1

202 80 1

202 498 1

202 275 1

202 1174 1

202 1175 1

202 1176 1

202 1177 1

202 1178 1

202 891 1

202 445 1

202 767 1

202 3453 0

202 1544 0

202 3645 0

202 1921 0

202 196 0

202 1072 0

202 1248 0

202 2536 0

202 1129 0

202 1784 0

202 3801 0

202 2906 0

202 3579 0

202 2029 0

202 1870 0

203 932 1

203 1094 1

203 53 1

203 1179 1

203 541 1

203 956 0

203 633 0

203 2125 0

203 441 0

203 2866 0

204 224 1

204 771 1

204 36 1

204 427 1

204 303 1

204 445 1

204 758 0

204 450 0

204 1153 0

204 34 0

204 645 0

204 1990 0

205 96 1

205 772 1

205 41 1

205 714 1

205 575 1

205 78 1

205 79 1

205 114 1

205 754 1

205 118 1

205 89 1

205 1180 1

205 286 1

205 95 1

205 1792 0

205 1116 0

205 2190 0

205 2859 0

205 2342 0

205 2891 0

205 2183 0

205 2240 0

205 31 0

205 2046 0

205 3263 0

205 456 0

205 2502 0

205 973 0

206 1183 1

206 1092 1

206 105 1

206 109 1

206 46 1

206 55 1

206 154 1

206 1181 1

206 1182 1

206 607 1

206 1072 0

206 747 0

206 916 0

206 632 0

206 2460 0

206 624 0

206 3459 0

206 2818 0

206 2053 0

206 3581 0

207 832 1

207 1188 1

207 75 1

207 237 1

207 79 1

207 951 1

207 87 1

207 184 1

207 91 1

207 2885 0

207 1122 0

207 2532 0

207 1387 0

207 2853 0

207 3704 0

207 964 0

207 3476 0

207 2371 0

208 608 1

208 257 1

208 498 1

208 883 1

208 277 1

208 85 1

208 17 0

208 2111 0

208 2500 0

208 2821 0

208 55 0

208 2137 0

209 800 1

209 581 1

209 1189 1

209 395 1

209 1077 1

209 218 1

209 220 1

209 1697 0

209 82 0

209 2759 0

209 1840 0

209 2818 0

209 697 0

209 2580 0

210 376 1

210 134 1

210 135 1

210 74 1

210 79 1

210 87 1

210 184 1

210 185 1

210 254 1

210 1091 0

210 1051 0

210 2908 0

210 3719 0

210 2659 0

210 3450 0

210 2388 0

210 3769 0

210 2115 0

211 98 1

211 99 1

211 101 1

211 1190 1

211 135 1

211 759 1

211 761 1

211 566 1

211 87 1

211 184 1

211 377 1

211 758 1

211 127 1

211 1369 0

211 3343 0

211 3433 0

211 3529 0

211 1573 0

211 34 0

211 3057 0

211 215 0

211 918 0

211 956 0

211 751 0

211 2977 0

211 549 0

212 196 1

212 1191 1

212 1192 1

212 297 1

212 1193 1

212 1194 1

212 719 1

212 51 1

212 53 1

212 1950 0

212 2722 0

212 1587 0

212 2735 0

212 3203 0

212 3102 0

212 433 0

212 3207 0

212 1504 0

213 128 1

213 802 1

213 325 1

213 968 1

213 1195 1

213 1196 1

213 77 1

213 1197 1

213 47 1

213 87 1

213 540 1

213 126 1

213 223 1

213 1498 0

213 3751 0

213 3198 0

213 3561 0

213 1220 0

213 2378 0

213 509 0

213 2882 0

213 2484 0

213 1226 0

213 1027 0

213 3731 0
213 3036 0
214 267 1
214 78 1
214 1198 1
214 1199 1
214 115 1
214 87 1
214 126 1
214 335 0
214 2656 0
214 512 0
214 3670 0
214 476 0
214 3078 0
214 1030 0
215 98 1
215 132 1
215 135 1
215 72 1
215 77 1
215 80 1
215 81 1
215 1200 1
215 1201 1
215 87 1
215 1070 0
215 597 0
215 2874 0
215 2972 0

215 670 0

215 1134 0

215 2397 0

215 3808 0

215 264 0

215 1896 0

216 73 1

216 74 1

216 77 1

216 81 1

216 1202 1

216 1203 1

216 692 1

216 1204 1

216 1205 1

216 87 1

216 1206 1

216 3335 0

216 930 0

216 3802 0

216 473 0

216 2003 0

216 767 0

216 3312 0

216 1886 0

216 1159 0

216 3805 0

216 1137 0

217 96 1

217 267 1

217 1208 1
217 55 1
217 376 1
217 1207 1
217 698 1
217 861 1
217 254 1
217 127 1
217 380 0
217 2516 0
217 250 0
217 1880 0
217 3044 0
217 2834 0
217 705 0
217 1383 0
217 2149 0
217 2971 0
218 96 1
218 67 1
218 1209 1
218 74 1
218 107 1
218 426 1
218 601 1
218 1210 1
218 1211 1
218 1212 1
218 735 1
218 1740 0

218 1019 0

218 916 0

218 2173 0

218 3151 0

218 2814 0

218 2170 0

218 2987 0

218 1300 0

218 2886 0

218 3275 0

219 1216 1

219 289 1

219 34 1

219 1215 1

219 36 1

219 1217 1

219 806 1

219 7 1

219 42 1

219 655 1

219 211 1

219 250 1

219 1213 1

219 1214 1

219 927 1

219 3146 0

219 3476 0

219 3489 0

219 3190 0

219 3526 0

219 2129 0

219 373 0

219 2842 0

219 734 0

219 3250 0

219 3057 0

219 777 0

219 2189 0

219 2990 0

219 2209 0

220 386 1

220 1218 1

220 1219 1

220 389 1

220 136 1

220 45 1

220 52 1

220 694 1

220 55 1

220 126 1

220 983 0

220 1298 0

220 2549 0

220 1960 0

220 34 0

220 629 0

220 3006 0

220 2712 0

220 1189 0

220 2362 0

221 354 1

221 36 1

221 105 1

221 109 1

221 46 1

221 111 1

221 53 1

221 57 1

221 571 1

221 221 1

221 159 1

221 662 0

221 35 0

221 2144 0

221 1069 0

221 51 0

221 1153 0

221 2478 0

221 2247 0

221 28 0

221 3253 0

221 3445 0

222 450 1

222 1220 1

222 1221 1

222 1222 1

222 1223 1

222 362 1

222 109 1

222 947 1

222 2557 0
222 3154 0
222 1451 0
222 3231 0
222 2313 0
222 1228 0
222 676 0
222 2777 0
223 98 1
223 80 1
223 404 1
223 118 1
223 376 1
223 3075 0
223 1312 0
223 1727 0
223 3333 0
223 164 0
224 34 1
224 3 1
224 1225 1
224 42 1
224 1226 1
224 1227 1
224 109 1
224 654 1
224 655 1
224 755 1
224 1118 1
224 1238 0

224 3560 0

224 2127 0

224 3001 0

224 3714 0

224 1746 0

224 1981 0

224 3101 0

224 2335 0

224 2308 0

224 151 0

225 73 1

225 74 1

225 75 1

225 202 1

225 77 1

225 1228 1

225 1229 1

225 115 1

225 372 1

225 87 1

225 1254 0

225 2160 0

225 2389 0

225 871 0

225 2781 0

225 1704 0

225 24 0

225 2953 0

225 2932 0

225 3427 0

226 131 1

226 483 1

226 72 1

226 104 1

226 137 1

226 237 1

226 957 1

226 117 1

226 22 1

226 24 1

226 252 1

226 413 1

226 3291 0

226 2754 0

226 3006 0

226 3489 0

226 2760 0

226 1487 0

226 2003 0

226 2343 0

226 451 0

226 2726 0

226 2457 0

226 1810 0

227 1153 1

227 133 1

227 105 1

227 46 1

227 47 1

227 1230 1

227 1231 1
227 52 1
227 125 1
227 118 1
227 55 1
227 920 1
227 61 1
227 190 1
227 2930 0
227 1706 0
227 2024 0
227 2602 0
227 28 0
227 3394 0
227 2179 0
227 1302 0
227 156 0
227 177 0
227 1951 0
227 2176 0
227 2420 0
227 2489 0
228 808 1
228 1040 1
228 1238 1
228 1239 1
228 1240 1
228 1241 1
228 1242 1
228 1243 1

228 575 1

228 152 0

228 192 0

228 1308 0

228 2374 0

228 462 0

228 1430 0

228 3468 0

228 421 0

228 1847 0

229 98 1

229 131 1

229 135 1

229 72 1

229 73 1

229 263 1

229 78 1

229 79 1

229 919 1

229 81 1

229 274 1

229 87 1

229 698 1

229 185 1

229 538 1

229 1244 1

229 1245 1

229 3260 0

229 1094 0

229 2060 0

229 1473 0

229 3092 0

229 134 0

229 2116 0

229 1954 0

229 377 0

229 3619 0

229 2860 0

229 392 0

229 1490 0

229 552 0

229 1358 0

229 17 0

229 896 0

230 96 1

230 802 1

230 772 1

230 902 1

230 776 1

230 808 1

230 714 1

230 79 1

230 47 1

230 303 1

230 210 1

230 118 1

230 1082 1

230 253 1

230 1246 1

230 1742 0

230 618 0

230 3768 0

230 1622 0

230 1768 0

230 339 0

230 590 0

230 2610 0

230 3809 0

230 2966 0

230 1037 0

230 819 0

230 421 0

230 1125 0

230 1636 0

231 1248 1

231 1249 1

231 1247 1

231 1250 1

231 36 1

231 1251 1

231 936 1

231 42 1

231 362 1

231 426 1

231 941 1

231 53 1

231 250 1

231 191 1

231 2032 0

231 2232 0

231 3709 0

231 1914 0

231 3363 0

231 3255 0

231 938 0

231 624 0

231 905 0

231 1239 0

231 3570 0

231 171 0

231 1773 0

231 911 0

232 1252 1

232 325 1

232 1253 1

232 1254 1

232 1255 1

232 105 1

232 1256 1

232 46 1

232 1183 1

232 1782 0

232 1485 0

232 3018 0

232 2791 0

232 753 0

232 2488 0

232 1007 0

232 3339 0

232 2607 0

233 832 1

233 385 1

233 98 1

233 131 1

233 132 1

233 1218 1

233 137 1

233 361 1

233 1257 1

233 1258 1

233 79 1

233 951 1

233 184 1

233 185 1

233 2301 0

233 2421 0

233 1842 0

233 2988 0

233 504 0

233 2076 0

233 3350 0

233 3398 0

233 140 0

233 192 0

233 1823 0

233 2148 0

233 2177 0

233 1527 0

234 859 1

234 134 1

234 104 1

234 74 1

234 171 1

234 364 1

234 237 1

234 1259 1

234 1260 1

234 52 1

234 918 1

234 87 1

234 88 1

234 539 1

234 91 1

234 61 1

234 63 1

234 1464 0

234 3728 0

234 3684 0

234 2758 0

234 2420 0

234 2796 0

234 1756 0

234 2814 0

234 3363 0

234 763 0

234 2021 0

234 3288 0

234 3320 0

234 1002 0

234 1860 0

234 2244 0

234 1419 0

235 102 1

235 1261 1

235 1262 1

235 1263 1

235 210 1

235 1172 1

235 920 1

235 219 1

235 1180 1

235 2772 0

235 3369 0

235 41 0

235 3522 0

235 3419 0

235 1880 0

235 2711 0

235 2035 0

235 1448 0

236 482 1

236 707 1

236 486 1

236 582 1

236 460 1

236 1264 1

236 465 1

236 1265 1

236 52 1

236 475 1

236 727 1

236 314 1

236 155 1

236 477 1

236 1306 0

236 3649 0

236 438 0

236 2116 0

236 1846 0

236 1765 0

236 2816 0

236 3588 0

236 3734 0

236 470 0

236 1850 0

236 1058 0

236 502 0

236 2764 0

237 41 1

237 75 1

237 1259 1

237 45 1

237 1260 1

237 80 1

237 1200 1

237 1266 1

237 917 1

237 87 1

237 61 1

237 63 1

237 3286 0

237 3473 0

237 1970 0

237 2627 0

237 1147 0

237 880 0

237 2602 0

237 3323 0

237 2472 0

237 2563 0

237 3151 0

237 3220 0

238 0 1

238 4 1

238 8 1

238 10 1

238 13 1

238 15 1

238 17 1

238 307 1

238 329 1

238 330 1

238 331 1

238 332 1

238 333 1

238 971 1

238 336 1

238 337 1

238 343 1

238 344 1

238 345 1

238 1267 1

238 1268 1

238 1269 1

238 1270 1

238 1271 1

238 1272 1

238 1273 1

238 2970 0

238 1168 0

238 2449 0

238 2479 0

238 3830 0

238 2847 0

238 1620 0

238 665 0

238 692 0

238 1682 0

238 2415 0

238 1019 0

238 3506 0

238 3255 0

238 3755 0

238 3175 0

238 616 0

238 3483 0

238 1931 0

238 737 0

238 156 0

238 908 0

238 2753 0

238 2050 0

238 3742 0

238 1810 0

239 133 1

239 73 1

239 74 1

239 206 1

239 79 1

239 919 1

239 915 1

239 437 1

239 87 1

239 184 1

239 185 1

239 1274 1

239 254 1

239 3494 0

239 533 0

239 3348 0

239 1682 0

239 2750 0

239 3812 0

239 3535 0

239 3602 0

239 675 0

239 1045 0

239 649 0

239 125 0

239 3275 0

240 1280 1

240 673 1

240 130 1

240 897 1

240 558 1

240 176 1

240 438 1

240 1278 1

240 442 1

240 1275 1

240 1276 1

240 1277 1

240 126 1

240 1279 1

240 3278 0

240 649 0

240 1348 0

240 789 0

240 2461 0

240 2297 0

240 2997 0

240 3665 0

240 449 0

240 899 0

240 2390 0

240 3731 0

240 1070 0

240 1697 0

241 96 1

241 1281 1

241 1282 1

241 1283 1

241 102 1

241 136 1

241 906 1

241 1098 1

241 494 1

241 303 1

241 208 1

241 376 1

241 2730 0

241 3734 0

241 1046 0

241 1715 0

241 202 0

241 1536 0

241 2372 0

241 3519 0

241 536 0

241 1705 0

241 1939 0

241 453 0

242 450 1

242 1220 1

242 1029 1

242 1284 1

242 1285 1

242 1286 1

242 1287 1

242 1288 1

242 1289 1

242 1290 1

242 1291 1

242 1292 1

242 947 1

242 1147 1

242 924 1

242 286 1

242 1630 0

242 3508 0

242 377 0

242 2060 0

242 1068 0

242 1603 0

242 33 0

242 299 0

242 3191 0

242 298 0

242 641 0

242 272 0

242 30 0

242 3623 0

242 2215 0

242 779 0

243 96 1

243 257 1

243 544 1

243 1057 1

243 1293 1

243 1294 1

243 879 1

243 442 1

243 499 1

243 276 1

243 1278 1

243 26 1

243 28 1

243 542 1

243 1418 0

243 1015 0

243 2364 0

243 1483 0

243 1556 0

243 3709 0

243 1475 0

243 2002 0

243 64 0

243 1873 0

243 1101 0

243 763 0

243 2723 0

243 2955 0

244 192 1

244 261 1

244 38 1

244 1093 1

244 1295 1

244 1296 1

244 883 1

244 118 1

244 3042 0

244 2441 0

244 2843 0

244 2903 0

244 2288 0

244 3613 0

244 948 0

244 3803 0

245 1283 1

245 165 1

245 102 1

245 776 1

245 1297 1

245 1012 1

245 119 1

245 280 1

245 286 1

245 3800 0

245 949 0

245 88 0

245 3798 0

245 738 0

245 1364 0

245 1732 0

245 3171 0

245 2 0

246 353 1

246 354 1

246 355 1

246 385 1

246 37 1

246 133 1

246 41 1

246 1298 1

246 499 1

246 252 1

246 221 1

246 190 1

246 3157 0

246 98 0

246 3758 0

246 3512 0

246 1474 0

246 3320 0

246 2446 0

246 1093 0

246 1733 0

246 2790 0

246 427 0

246 3085 0

247 130 1

247 137 1

247 377 1

247 110 1

247 78 1

247 79 1

247 115 1

247 184 1

247 185 1

247 1018 1

247 91 1

247 642 0

247 2427 0

247 1243 0

247 935 0

247 2481 0

247 2647 0

247 3438 0

247 2675 0

247 1536 0

247 631 0

247 2908 0

248 740 1

248 805 1

248 1299 1

248 1300 1

248 1301 1

248 1302 1

248 542 1

248 1682 0

248 2196 0

248 298 0

248 2891 0

248 1 0

248 1894 0

248 1593 0

249 96 1

249 738 1

249 36 1

249 41 1

249 659 1

249 1303 1

249 60 1

249 253 1

249 542 1

249 3229 0

249 677 0

249 1001 0

249 1662 0

249 989 0

249 139 0

249 1467 0

249 639 0

249 2619 0

250 770 1

250 107 1

250 1304 1

250 1305 1

250 764 1

250 130 0

250 1272 0

250 1294 0

250 2137 0

250 1798 0

251 135 1

251 73 1

251 171 1

251 363 1

251 206 1

251 79 1

251 1201 1

251 1202 1

251 87 1

251 184 1

251 377 1

251 496 0

251 2416 0

251 2543 0

251 3427 0

251 1005 0

251 1477 0

251 1088 0

251 2428 0

251 550 0

251 1736 0

251 641 0

252 96 1

252 128 1

252 802 1

252 1312 1

252 1313 1

252 1030 1

252 589 1

252 302 1

252 406 1

252 119 1

252 1309 1

252 1310 1

252 1311 1

252 129 0

252 1226 0

252 1597 0

252 2653 0

252 1820 0

252 1413 0

252 1650 0

252 3727 0

252 1472 0

252 2178 0

252 2764 0

252 3463 0

252 2635 0

253 66 1

253 1314 1

253 1315 1

253 1316 1

253 1317 1

253 1199 1

253 953 1

253 445 1

253 2109 0

253 2811 0

253 1452 0

253 37 0

253 2353 0

253 1828 0

253 2241 0

253 2540 0

254 257 1

254 1318 1

254	109	1
254	13	1
254	498	1
254	499	1
254	445	1
254	789	0
254	1202	0
254	2151	0
254	1283	0
254	3016	0
254	917	0
254	2199	0
255	35	1
255	197	1
255	6	1
255	7	1
255	934	1
255	1319	1
255	1320	1
255	11	1
255	331	1
255	13	1
255	14	1
255	333	1
255	1321	1
255	52	1
255	857	1
255	57	1
255	2311	0
255	256	0

255 3386 0

255 1403 0

255 1891 0

255 3172 0

255 3431 0

255 3412 0

255 5 0

255 3552 0

255 1561 0

255 2111 0

255 1678 0

255 2561 0

255 587 0

255 2081 0

256 96 1

256 98 1

256 1322 1

256 1323 1

256 399 1

256 373 1

256 377 1

256 254 1

256 241 0

256 894 0

256 208 0

256 1907 0

256 3163 0

256 3293 0

256 453 0

256 1325 0

257 1261 1

257 302 1

257 303 1

257 398 1

257 212 1

257 1172 1

257 118 1

257 218 1

257 1119 1

257 1253 0

257 1691 0

257 752 0

257 2014 0

257 2243 0

257 1364 0

257 1106 0

257 1580 0

257 3114 0

258 36 1

258 358 1

258 1324 1

258 1325 1

258 110 1

258 1326 1

258 1327 1

258 1328 1

258 275 1

258 507 1

258 22 1

258 438 1

258 187 1

258 863 1

258 2046 0

258 3259 0

258 208 0

258 2240 0

258 2228 0

258 1316 0

258 3412 0

258 3087 0

258 3408 0

258 3833 0

258 2630 0

258 3534 0

258 3471 0

258 2144 0

259 96 1

259 805 1

259 241 1

259 1329 1

259 1330 1

259 1331 1

259 1332 1

259 190 1

259 1003 0

259 572 0

259 3747 0

259 3504 0

259 667 0

259 1380 0

259 3560 0

259 2264 0

260 673 1

260 34 1

260 1249 1

260 228 1

260 36 1

260 362 1

260 109 1

260 52 1

260 1333 1

260 1334 1

260 599 0

260 2839 0

260 243 0

260 2999 0

260 2857 0

260 1566 0

260 2681 0

260 3063 0

260 3351 0

260 2206 0

261 483 1

261 261 1

261 844 1

261 1338 1

261 80 1

261 20 1

261 21 1

261 854 1

261 1335 1

261 1336 1

261 1337 1

261 474 1

261 1339 1

261 479 1

261 77 0

261 1784 0

261 2600 0

261 2566 0

261 2776 0

261 1009 0

261 3337 0

261 2492 0

261 2616 0

261 3407 0

261 870 0

261 872 0

261 2930 0

261 907 0

262 1342 1

262 1063 1

262 531 1

262 756 1

262 119 1

262 1340 1

262 1341 1

262 286 1

262 575 1

262 1959 0

262 2824 0

262 907 0

262 580 0

262 3152 0

262 590 0

262 2063 0

262 1174 0

262 1543 0

263 96 1

263 133 1

263 134 1

263 102 1

263 998 1

263 52 1

263 250 1

263 1022 1

263 100 0

263 3133 0

263 947 0

263 434 0

263 2030 0

263 3243 0

263 2639 0

263 1637 0

264 66 1

264 98 1

264 133 1

264 74 1

264 77 1

264 399 1

264 115 1

264 117 1

264 373 1

264 87 1

264 127 1

264 2823 0

264 3112 0

264 2459 0

264 94 0

264 2304 0

264 3326 0

264 880 0

264 2351 0

264 1458 0

264 898 0

264 3111 0

265 1345 1

265 1346 1

265 1347 1

265 36 1

265 1348 1

265 102 1

265 1349 1

265 1350 1

265 1351 1

265 109 1

265 52 1

265 278 1

265 286 1

265 1001 0

265 1928 0

265 150 0

265 3055 0

265 2903 0

265 660 0

265 3054 0

265 2782 0

265 41 0

265 993 0

265 836 0

265 1535 0

265 3777 0

266 96 1

266 36 1

266 133 1

266 102 1

266 136 1

266 105 1

266 107 1

266 235 1

266 78 1

266 46 1

266 80 1

266 52 1

266 118 1

266 55 1

266 920 1

266 128 0

266 2780 0

266 453 0

266 1354	0

266 231 0

266 3472	0

266 2239	0

266 826 0

266 2607	0

266 2507	0

266 1653	0

266 3298	0

266 3796	0

266 1507	0

266 3573	0

267 1057	1

267 1352	1

267 361 1

267 1353	1

267 1354	1

267 276 1

267 25	1

267 3042	0

267 3326	0

267 1074	0

267 3418	0

267 2573	0

267 2415	0

267 667 0

268 377 1

268 1355	1

268 1356	1

268 1357	1

268 1358 1
268 79 1
268 1359 1
268 1360 1
268 1361 1
268 184 1
268 185 1
268 538 1
268 382 1
268 1861 0
268 2641 0
268 2683 0
268 219 0
268 866 0
268 422 0
268 1631 0
268 1830 0
268 3652 0
268 1512 0
268 1 0
268 3513 0
268 442 0
269 34 1
269 36 1
269 362 1
269 109 1
269 1362 1
269 2824 0
269 2594 0
269 1384 0

269 647 0

269 2127 0

270 102 1

270 681 1

270 1363 1

270 1364 1

270 1365 1

270 1366 1

270 3715 0

270 2457 0

270 3399 0

270 1598 0

270 2618 0

270 210 0

271 200 1

271 52 1

271 1333 1

271 406 1

271 1367 1

271 1368 1

271 1369 1

271 1370 1

271 1371 1

271 1372 1

271 1373 1

271 1374 1

271 1375 1

271 1901 0

271 3771 0

271 2889 0

271 2135 0
271 2968 0
271 231 0
271 158 0
271 820 0
271 2559 0
271 1104 0
271 3011 0
271 3473 0
271 2840 0
272 36 1
272 392 1
272 941 1
272 241 1
272 242 1
272 52 1
272 347 1
272 1148 1
272 554 0
272 1547 0
272 2531 0
272 2640 0
272 2122 0
272 3208 0
272 1064 0
272 3100 0
273 1376 1
273 1377 1
273 1378 1
273 1379 1

273 1380 1
273 1381 1
273 1382 1
273 1383 1
273 3436 0
273 2716 0
273 2266 0
273 600 0
273 847 0
273 752 0
273 1075 0
273 3242 0
274 223 1
274 163 1
274 453 1
274 1253 1
274 615 1
274 1384 1
274 1385 1
274 1386 1
274 1387 1
274 1388 1
274 141 1
274 1389 1
274 1390 1
274 53 1
274 863 1
274 695 1
274 56 1
274 159 1

274 1490 0

274 1814 0

274 3437 0

274 3473 0

274 3145 0

274 2843 0

274 3578 0

274 515 0

274 769 0

274 3512 0

274 3181 0

274 293 0

274 2297 0

274 366 0

274 298 0

274 2991 0

274 2938 0

274 3604 0

275 78 1

275 46 1

275 47 1

275 1391 1

275 1392 1

275 1393 1

275 1140 1

275 1394 1

275 1395 1

275 1396 1

275 280 1

275 61 1

275 863 1

275 1315 0

275 3348 0

275 3144 0

275 2775 0

275 3369 0

275 1581 0

275 3791 0

275 3061 0

275 3 0

275 1445 0

275 1402 0

275 1905 0

275 3085 0

276 133 1

276 391 1

276 41 1

276 105 1

276 1259 1

276 46 1

276 399 1

276 1397 1

276 1398 1

276 1399 1

276 1400 1

276 413 1

276 190 1

276 863 1

276 744 0

276 3635 0

276 1631 0

276 3732 0

276 1502 0

276 2726 0

276 214 0

276 2810 0

276 1488 0

276 308 0

276 487 0

276 2754 0

276 3380 0

276 1937 0

277 1253 1

277 43 1

277 1397 1

277 1401 1

277 1402 1

277 347 1

277 188 1

277 190 1

277 316 0

277 3435 0

277 2282 0

277 1685 0

277 1951 0

277 1856 0

277 1834 0

277 3597 0

278 194 1

278 171 1

278 235 1

278 528 1

278 469 1

278 863 1

278 55 1

278 188 1

278 127 1

278 973 0

278 967 0

278 1670 0

278 2149 0

278 2007 0

278 247 0

278 2584 0

278 1873 0

278 2724 0

279 66 1

279 131 1

279 1315 1

279 135 1

279 73 1

279 1258 1

279 75 1

279 1403 1

279 126 1

279 360 0

279 986 0

279 2681 0

279 2770 0

279 3019 0

279 2195 0

279 3299 0

279 3254 0

279 1346 0

280 36 1

280 325 1

280 102 1

280 138 1

280 1103 1

280 788 1

280 55 1

280 920 1

280 187 1

280 2632 0

280 2250 0

280 402 0

280 3496 0

280 1881 0

280 1564 0

280 682 0

280 2586 0

280 2472 0

281 354 1

281 483 1

281 36 1

281 37 1

281 1315 1

281 136 1

281 109 1

281 878 1

281 1405 1
281 277 1
281 1404 1
281 956 1
281 445 1
281 1406 1
281 1407 1
281 663 0
281 3399 0
281 3764 0
281 3339 0
281 3049 0
281 790 0
281 3229 0
281 434 0
281 3440 0
281 1052 0
281 1336 0
281 2520 0
281 996 0
281 2171 0
281 3317 0
282 96 1
282 98 1
282 133 1
282 135 1
282 74 1
282 75 1
282 80 1
282 118 1

282 55 1

282 185 1

282 1417 0

282 1291 0

282 2671 0

282 2306 0

282 3767 0

282 3192 0

282 2605 0

282 2772 0

282 801 0

282 2477 0

283 96 1

283 1408 1

283 227 1

283 1001 1

283 332 1

283 13 1

283 116 1

283 118 1

283 445 1

283 1255 0

283 1279 0

283 1304 0

283 3668 0

283 770 0

283 455 0

283 880 0

283 2853 0

283 3072 0

284 200 1

284 1234 1

284 21 1

284 1374 1

284 1311 1

284 3604 0

284 3800 0

284 1378 0

284 3621 0

284 950 0

285 36 1

285 1224 1

285 210 1

285 242 1

285 243 1

285 217 1

285 1434 0

285 1570 0

285 397 0

285 2731 0

285 1369 0

285 1673 0

286 128 1

286 1409 1

286 130 1

286 1410 1

286 1411 1

286 1412 1

286 1413 1

286 135 1

286 72 1

286 137 1

286 361 1

286 1414 1

286 77 1

286 237 1

286 80 1

286 376 1

286 378 1

286 1529 0

286 1745 0

286 1718 0

286 3364 0

286 995 0

286 2337 0

286 30 0

286 778 0

286 3631 0

286 2755 0

286 3560 0

286 1672 0

286 3544 0

286 3724 0

286 279 0

286 2849 0

286 3018 0

287 1218 1

287 838 1

287 1415 1

287 1416 1

287 1417 1
287 1418 1
287 235 1
287 821 1
287 22 1
287 918 1
287 572 1
287 1083 0
287 1986 0
287 1680 0
287 2194 0
287 3401 0
287 3090 0
287 2914 0
287 2727 0
287 2790 0
287 460 0
287 1149 0
288 99 1
288 1316 1
288 1419 1
288 1420 1
288 1421 1
288 78 1
288 1199 1
288 566 1
288 758 1
288 93 1
288 1143 0
288 416 0

288 796 0

288 2641 0

288 1262 0

288 768 0

288 1040 0

288 640 0

288 3488 0

288 1181 0

289 4 1

289 5 1

289 806 1

289 7 1

289 1029 1

289 333 1

289 1422 1

289 15 1

289 241 1

289 245 1

289 217 1

289 1117 1

289 2012 0

289 3297 0

289 272 0

289 2969 0

289 2437 0

289 1744 0

289 2775 0

289 58 0

289 1260 0

289 2054 0

289 688 0

289 117 0

290 1185 1

290 1122 1

290 1186 1

290 41 1

290 1423 1

290 1424 1

290 52 1

290 118 1

290 184 1

290 699 1

290 700 1

290 919 0

290 99 0

290 3313 0

290 1853 0

290 2770 0

290 3827 0

290 3219 0

290 1228 0

290 703 0

290 1259 0

290 891 0

291 96 1

291 105 1

291 1259 1

291 46 1

291 47 1

291 80 1

291 52 1

291 118 1

291 55 1

291 863 1

291 127 1

291 845 0

291 725 0

291 1679 0

291 3836 0

291 2237 0

291 287 0

291 1185 0

291 3657 0

291 165 0

291 3671 0

291 3742 0

292 96 1

292 545 1

292 738 1

292 771 1

292 1184 1

292 46 1

292 655 1

292 1425 1

292 1426 1

292 1427 1

292 1428 1

292 119 1

292 125 1

292 543 1

292 3540 0

292 957 0

292 1844 0

292 3340 0

292 3836 0

292 1014 0

292 232 0

292 3623 0

292 3743 0

292 1983 0

292 2258 0

292 2956 0

292 3182 0

292 3185 0

293 1431 1

293 36 1

293 556 1

293 947 1

293 52 1

293 949 1

293 1429 1

293 1430 1

293 1432 1

293 1433 1

293 1434 1

293 1435 1

293 841 0

293 2294 0

293 762 0

293 3760 0

293 2439 0
293 3032 0
293 3247 0
293 1402 0
293 712 0
293 313 0
293 876 0
293 848 0
294 128 1
294 356 1
294 136 1
294 235 1
294 267 1
294 979 1
294 87 1
294 1436 1
294 127 1
294 324 0
294 1795 0
294 3706 0
294 1065 0
294 1792 0
294 2201 0
294 3047 0
294 119 0
294 813 0
295 1131 1
295 46 1
295 1262 1
295 210 1

295 826 1

295 827 1

295 1437 1

295 1438 1

295 320 0

295 2873 0

295 3143 0

295 830 0

295 2218 0

295 180 0

295 1430 0

295 290 0

296 902 1

296 8 1

296 147 1

296 21 1

296 22 1

296 1439 1

296 1440 1

296 1441 1

296 1442 1

296 1443 1

296 1444 1

296 681 1

296 939 1

296 307 1

296 53 1

296 184 1

296 329 1

296 332 1

296 988 1

296 110 1

296 886 1

296 2913 0

296 2752 0

296 3023 0

296 3222 0

296 1389 0

296 3569 0

296 2601 0

296 718 0

296 2374 0

296 3071 0

296 1213 0

296 3842 0

296 2789 0

296 1870 0

296 890 0

296 641 0

296 3197 0

296 3802 0

296 3075 0

296 1858 0

296 3216 0

297 1445 1

297 325 1

297 1446 1

297 1447 1

297 1167 1

297 216 1

297 126 1

297 2854 0

297 1147 0

297 933 0

297 2234 0

297 3374 0

297 2200 0

297 2126 0

298 128 1

298 1344 1

298 136 1

298 137 1

298 1448 1

298 1449 1

298 1450 1

298 77 1

298 206 1

298 1451 1

298 80 1

298 1452 1

298 210 1

298 118 1

298 376 1

298 1057 0

298 397 0

298 715 0

298 1199 0

298 2400 0

298 2735 0

298 1656 0

298 289 0

298 547 0

298 2818 0

298 531 0

298 1118 0

298 2539 0

298 2280 0

298 3056 0

299 928 1

299 289 1

299 1216 1

299 36 1

299 1445 1

299 42 1

299 747 1

299 1453 1

299 1454 1

299 241 1

299 348 1

299 250 1

299 284 1

299 133 0

299 697 0

299 3686 0

299 2964 0

299 3196 0

299 1118 0

299 852 0

299 2241 0

299 739 0

299 3232 0

299 3394 0

299 1336 0

299 2619 0

300 0 1

300 5 1

300 6 1

300 331 1

300 460 1

300 1422 1

300 1455 1

300 1456 1

300 341 1

300 1275 1

300 732 1

300 478 1

300 1106 0

300 709 0

300 1427 0

300 377 0

300 1864 0

300 1836 0

300 3082 0

300 738 0

300 1411 0

300 818 0

300 1928 0

300 2165 0

301 96 1

301 134 1

301 135 1

301 79 1

301 399 1

301 560 1

301 976 1

301 917 1

301 87 1

301 127 1

301 3089 0

301 3355 0

301 1870 0

301 241 0

301 3180 0

301 660 0

301 1599 0

301 2291 0

301 441 0

301 3205 0

302 1408 1

302 98 1

302 130 1

302 137 1

302 1258 1

302 268 1

302 269 1

302 270 1

302 81 1

302 1457 1

302 692 1

302 185 1

302 1390 0
302 227 0
302 1696 0
302 3024 0
302 2062 0
302 3537 0
302 1129 0
302 1445 0
302 3593 0
302 2748 0
302 871 0
302 2208 0
303 96 1
303 708 1
303 105 1
303 46 1
303 1458 1
303 1459 1
303 1460 1
303 280 1
303 859 1
303 254 1
303 1218 0
303 1427 0
303 345 0
303 1409 0
303 3546 0
303 1311 0
303 930 0
303 1789 0

303 2476 0

303 523 0

304 385 1

304 132 1

304 72 1

304 1257 1

304 74 1

304 236 1

304 237 1

304 46 1

304 52 1

304 117 1

304 1461 1

304 55 1

304 252 1

304 61 1

304 127 1

304 2646 0

304 3409 0

304 2334 0

304 2158 0

304 1844 0

304 2954 0

304 3376 0

304 3077 0

304 3436 0

304 1639 0

304 753 0

304 3739 0

304 2609 0

304 1554 0
304 2537 0
305 171 1
305 78 1
305 79 1
305 1199 1
305 437 1
305 87 1
305 184 1
305 185 1
305 538 1
305 1246 0
305 1231 0
305 2932 0
305 865 0
305 3633 0
305 3598 0
305 1983 0
305 501 0
305 3460 0
306 1463 1
306 98 1
306 923 1
306 267 1
306 1230 1
306 399 1
306 372 1
306 1462 1
306 919 1
306 91 1

306 254 1

306 1837 0

306 2356 0

306 61 0

306 1163 0

306 2945 0

306 3539 0

306 3033 0

306 1398 0

306 1367 0

306 3290 0

306 2652 0

307 134 1

307 75 1

307 77 1

307 399 1

307 863 1

307 55 1

307 1464 1

307 377 1

307 63 1

307 2318 0

307 1238 0

307 268 0

307 1134 0

307 1632 0

307 977 0

307 779 0

307 1873 0

307 1804 0

308 96 1

308 1185 1

308 66 1

308 98 1

308 1026 1

308 78 1

308 87 1

308 1465 1

308 1466 1

308 959 0

308 1308 0

308 984 0

308 2952 0

308 683 0

308 1691 0

308 1025 0

308 2511 0

308 2582 0

309 96 1

309 740 1

309 392 1

309 105 1

309 523 1

309 652 1

309 46 1

309 1103 1

309 946 1

309 52 1

309 250 1

309 1467 1

309 190 1

309 1136 0

309 1481 0

309 2176 0

309 97 0

309 344 0

309 1042 0

309 1199 0

309 260 0

309 582 0

309 3111 0

309 244 0

309 3302 0

309 693 0

310 551 1

310 41 1

310 171 1

310 45 1

310 145 1

310 433 1

310 115 1

310 118 1

310 87 1

310 1468 1

310 1469 1

310 126 1

310 3293 0

310 3183 0

310 1748 0

310 3759 0

310 2367 0

310 1226 0

310 3733 0

310 428 0

310 3387 0

310 3508 0

310 2013 0

310 2718 0

311 96 1

311 673 1

311 35 1

311 196 1

311 197 1

311 771 1

311 752 1

311 118 1

311 438 1

311 24 1

311 729 1

311 474 1

311 187 1

311 253 1

311 1470 1

311 1471 1

311 1060 0

311 1578 0

311 2620 0

311 2421 0

311 939 0

311 3802 0

311 3344 0

311 3036 0

311 1831 0

311 3073 0

311 3321 0

311 3331 0

311 284 0

311 2498 0

311 1053 0

311 3366 0

312 1472 1

312 1473 1

312 1474 1

312 3 1

312 1475 1

312 1150 1

312 2299 0

312 473 0

312 694 0

312 2219 0

312 442 0

312 2441 0

313 1330 1

313 1476 1

313 36 1

313 805 1

313 1855 0

313 1778 0

313 1236 0

313 1230 0

314 98 1

314 130 1

314 133 1

314 134 1

314 135 1

314 73 1

314 77 1

314 79 1

314 80 1

314 81 1

314 87 1

314 185 1

314 538 1

314 2227 0

314 2292 0

314 816 0

314 108 0

314 3013 0

314 2368 0

314 721 0

314 3483 0

314 1112 0

314 2643 0

314 2060 0

314 3393 0

314 304 0

315 1026 1

315 1477 1

315 1478 1

315 1479 1

315 80 1

315 1404 1

315 126 1

315 127 1

315 382 0

315 1518 0

315 41 0

315 2143 0

315 2042 0

315 2521 0

315 2180 0

315 1897 0

316 962 1

316 1480 1

316 1481 1

316 1482 1

316 1483 1

316 1484 1

316 1485 1

316 1486 1

316 1010 1

316 314 1

316 763 1

316 508 1

316 510 1

316 1696 0

316 2109 0

316 82 0

316 2397 0

316 2144 0

316 184 0

316 1724 0

316 3290 0

316 1189 0

316 641 0

316 3008 0

316 1444 0

316 869 0

317 36 1

317 295 1

317 765 1

317 107 1

317 46 1

317 1487 1

317 1488 1

317 52 1

317 24 1

317 474 1

317 956 1

317 221 1

317 190 1

317 2853 0

317 3162 0

317 1880 0

317 2501 0

317 293 0

317 3495 0

317 2123 0

317 1226 0

317 1064 0

317 3178 0

317 1630 0

317 2350 0

317 3046 0

318 241 1

318 210 1

318 1491 1

318 1489 1

318 1490 1

318 2620 0

318 3193 0

318 325 0

318 2780 0

318 99 0

319 1495 1

319 293 1

319 104 1

319 109 1

319 1492 1

319 277 1

319 1493 1

319 1494 1

319 3661 0

319 342 0

319 213 0

319 90 0

319 1698 0

319 1596 0

319 2325 0

319 70 0

320 708 1

320 359 1

320 141 1

320 46 1

320 1138 1

320 1496 1

320 187 1

320 572 1

320 413 1

320 3394 0

320 2793 0

320 1938 0

320 592 0

320 415 0

320 414 0

320 3110 0

320 2359 0

320 2792 0

321 102 1

321 134 1

321 295 1

321 105 1

321 392 1

321 46 1

321 47 1

321 528 1

321 114 1

321 1174 1

321 55 1

321 861 1

321 863 1

321 595 0

321 2553 0

321 64 0

321 3324 0

321 2661 0

321 1853 0

321 609 0

321 176 0

321 1136 0

321 1997 0

321 653 0

321 3489 0

321 384 0

322 96 1

322 1248 1

322 102 1

322 118 1

322 376 1

322 1497 1

322 1498 1

322 216 1

322 362 0

322 1896 0

322 32 0

322 3309 0

322 1264 0

322 1938 0

322 626 0

322 768 0

323	115	1
323	863	1
323	87	1
323	184	1
323	1499	1
323	1500	1
323	127	1
323	1958	0
323	2311	0
323	939	0
323	2479	0
323	3013	0
323	216	0
323	3583	0
324	708	1
324	1097	1
324	1503	1
324	460	1
324	589	1
324	303	1
324	111	1
324	1175	1
324	1501	1
324	1502	1
324	863	1
324	3666	0
324	3635	0
324	2131	0
324	2387	0
324	3344	0

324 806 0

324 3252 0

324 2693 0

324 2219 0

324 2654 0

324 1046 0

325 385 1

325 66 1

325 72 1

325 74 1

325 171 1

325 237 1

325 79 1

325 87 1

325 88 1

325 539 1

325 540 1

325 3820 0

325 1526 0

325 1749 0

325 753 0

325 1444 0

325 3246 0

325 2780 0

325 463 0

325 1844 0

325 848 0

325 933 0

326 1504 1

326 1409 1

326 133 1

326 714 1

326 267 1

326 1258 1

326 301 1

326 78 1

326 79 1

326 47 1

326 434 1

326 184 1

326 923 1

326 582 0

326 1636 0

326 2407 0

326 1201 0

326 2752 0

326 2980 0

326 2661 0

326 3401 0

326 1058 0

326 1135 0

326 2504 0

326 785 0

326 964 0

327 1505 1

327 131 1

327 133 1

327 135 1

327 109 1

327 238 1

327 80 1

327 725 1

327 22 1

327 184 1

327 377 1

327 538 1

327 1460 0

327 3195 0

327 917 0

327 687 0

327 927 0

327 979 0

327 156 0

327 2009 0

327 2058 0

327 832 0

327 1800 0

327 107 0

328 134 1

328 136 1

328 79 1

328 81 1

328 374 1

328 87 1

328 184 1

328 185 1

328 3306 0

328 3109 0

328 580 0

328 467 0

328 3314 0

328 147 0

328 117 0

328 2258 0

329 96 1

329 98 1

329 1506 1

329 77 1

329 79 1

329 1200 1

329 87 1

329 185 1

329 127 1

329 116 0

329 3148 0

329 1582 0

329 950 0

329 2141 0

329 683 0

329 1493 0

329 2644 0

329 1045 0

330 96 1

330 137 1

330 80 1

330 118 1

330 55 1

330 538 1

330 3111 0

330 2010 0

330 2436	0
330 1192	0
330 1822	0
330 825 0
331 1507	1
331 199 1
331 303 1
331 304 1
331 215 1
331 187 1
331 1503	1
331 3110	0
331 1530	0
331 1932	0
331 1802	0
331 3797	0
331 2055	0
331 2212	0
332 1508	1
332 293 1
332 517 1
332 295 1
332 104 1
332 1509	1
332 1510	1
332 1511	1
332 460 1
332 187 1
332 1138	1
332 1237	1

332 57 1

332 155 1

332 957 1

332 2764 0

332 1591 0

332 1307 0

332 170 0

332 2489 0

332 1071 0

332 1050 0

332 527 0

332 2099 0

332 1360 0

332 3472 0

332 2237 0

332 2059 0

332 895 0

332 2298 0

333 35 1

333 708 1

333 486 1

333 360 1

333 1512 1

333 1513 1

333 1514 1

333 1100 1

333 1515 1

333 46 1

333 1516 1

333 1136 1

333 1517 1

333 1496 1

333 442 1

333 155 1

333 158 1

333 2370 0

333 1062 0

333 2559 0

333 1503 0

333 963 0

333 1056 0

333 903 0

333 2926 0

333 2236 0

333 3519 0

333 2000 0

333 1472 0

333 2681 0

333 790 0

333 1821 0

333 1033 0

333 1768 0

334 1352 1

334 1293 1

334 1518 1

334 1519 1

334 1520 1

334 497 1

334 498 1

334 275 1

334 500 1

334 1521 1

334 1277 1

334 30 1

334 799 1

334 1238 0

334 43 0

334 2669 0

334 1695 0

334 2435 0

334 155 0

334 358 0

334 33 0

334 1466 0

334 1219 0

334 3170 0

334 1101 0

334 1624 0

335 442 1

335 1522 1

335 1623 0

335 3788 0

336 133 1

336 134 1

336 73 1

336 74 1

336 171 1

336 87 1

336 377 1

336 127 1

336 1582	0

336 3446	0

336 1000	0

336 3110	0

336 3405	0

336 2525	0

336 1618	0

336 1880	0

337 102 1

337 267 1

337 78 1

337 560 1

337 1523	1

337 1524	1

337 1525	1

337 118 1

337 55 1

337 3435	0

337 1864	0

337 3234	0

337 1003	0

337 2337	0

337 2111	0

337 3101	0

337 3831	0

337 2515	0

338 1342	1

338 738 1

338 392 1

338 208 1

338 210 1

338 830 1

338 1535 0

338 3353 0

338 1429 0

338 1635 0

338 2807 0

338 3249 0

339 608 1

339 864 1

339 35 1

339 36 1

339 1527 1

339 297 1

339 141 1

339 909 1

339 48 1

339 1526 1

339 471 1

339 451 0

339 1319 0

339 2151 0

339 1963 0

339 990 0

339 1434 0

339 240 0

339 1035 0

339 3160 0

339 1387 0

339 1655 0

340 1374 1
340 867 1
340 1534 1
340 1514 1
340 1105 1
340 1010 1
340 30 1
340 1528 1
340 1529 1
340 1530 1
340 1531 1
340 1532 1
340 1533 1
340 958 1
340 153 0
340 2096 0
340 1918 0
340 1284 0
340 592 0
340 2783 0
340 3468 0
340 3179 0
340 3250 0
340 1540 0
340 2298 0
340 1345 0
340 2933 0
340 2075 0
341 1536 1
341 1537 1

341 196 1

341 36 1

341 516 1

341 615 1

341 1416 1

341 362 1

341 1514 1

341 1535 1

341 53 1

341 888 1

341 155 1

341 156 1

341 735 1

341 1859 0

341 528 0

341 216 0

341 1992 0

341 1632 0

341 3233 0

341 1979 0

341 802 0

341 545 0

341 866 0

341 1585 0

341 2635 0

341 503 0

341 875 0

341 1851 0

342 833 1

342 1538 1

342 1539 1

342 1540 1

342 102 1

342 906 1

342 78 1

342 46 1

342 47 1

342 528 1

342 119 1

342 920 1

342 2772 0

342 904 0

342 3242 0

342 974 0

342 3197 0

342 1547 0

342 2498 0

342 1899 0

342 1313 0

342 3344 0

342 764 0

342 1721 0

343 224 1

343 1541 1

343 1542 1

343 1543 1

343 104 1

343 1544 1

343 1545 1

343 1546 1

343 1004 1
343 1325 1
343 462 1
343 883 1
343 1174 1
343 1047 1
343 223 1
343 1633 0
343 3686 0
343 885 0
343 93 0
343 3835 0
343 652 0
343 2374 0
343 3199 0
343 3249 0
343 1934 0
343 3426 0
343 3540 0
343 1438 0
343 2148 0
343 3211 0
344 105 1
344 106 1
344 1257 1
344 1547 1
344 1548 1
344 878 1
344 46 1
344 1396 1

344 764 1

344 449 0

344 3366 0

344 3482 0

344 1238 0

344 1578 0

344 1337 0

344 1490 0

344 3723 0

344 3832 0

345 353 1

345 37 1

345 133 1

345 1098 1

345 235 1

345 1549 1

345 1550 1

345 80 1

345 118 1

345 22 1

345 90 1

345 1409 0

345 1626 0

345 1761 0

345 1198 0

345 2163 0

345 2464 0

345 226 0

345 3626 0

345 3447 0

345 1815 0

345 1114 0

346 577 1

346 754 1

346 659 1

346 476 1

346 445 1

346 735 0

346 3258 0

346 1429 0

346 282 0

346 2645 0

347 96 1

347 100 1

347 133 1

347 38 1

347 1551 1

347 756 1

347 53 1

347 505 1

347 60 1

347 2585 0

347 673 0

347 3286 0

347 1276 0

347 3206 0

347 1151 0

347 913 0

347 3729 0

347 610 0

348 26 1

348 146 0

349 674 1

349 232 1

349 377 1

349 79 1

349 399 1

349 22 1

349 185 1

349 538 1

349 799 1

349 3160 0

349 3547 0

349 2966 0

349 886 0

349 2408 0

349 2838 0

349 2997 0

349 694 0

349 3346 0

350 36 1

350 325 1

350 1552 1

350 241 1

350 1553 1

350 476 1

350 1088 0

350 370 0

350 1602 0

350 1779 0

350 2039 0

350 149 0

351 1248 1

351 535 1

351 7 1

351 1224 1

351 42 1

351 300 1

351 657 1

351 242 1

351 211 1

351 1554 1

351 245 1

351 55 1

351 1175 1

351 250 1

351 347 1

351 989 1

351 3463 0

351 992 0

351 1432 0

351 1963 0

351 3566 0

351 1761 0

351 1073 0

351 1637 0

351 683 0

351 1129 0

351 2903 0

351 3142 0

351 1585 0
351 3641 0
351 2667 0
351 1922 0
352 96 1
352 897 1
352 36 1
352 145 1
352 435 1
352 52 1
352 118 1
352 542 1
352 516 0
352 1676 0
352 328 0
352 1291 0
352 308 0
352 522 0
352 772 0
352 2471 0
353 135 1
353 72 1
353 73 1
353 77 1
353 79 1
353 81 1
353 115 1
353 1557 1
353 87 1
353 127 1

353 1122 0

353 536 0

353 606 0

353 1250 0

353 1478 0

353 1809 0

353 1859 0

353 2813 0

353 3408 0

353 1796 0

354 520 1

354 20 1

354 1559 1

354 1560 1

354 1561 1

354 665 1

354 1562 1

354 1563 1

354 1565 1

354 1564 1

354 1567 1

354 157 1

354 417 1

354 1566 1

354 419 1

354 1568 1

354 1569 1

354 357 1

354 1000 1

354 1516 1

354 1407 1
354 2301 0
354 3504 0
354 1989 0
354 1933 0
354 537 0
354 631 0
354 2236 0
354 1478 0
354 442 0
354 1973 0
354 2269 0
354 2740 0
354 2290 0
354 226 0
354 2314 0
354 3210 0
354 69 0
354 3350 0
354 1382 0
354 2695 0
354 1687 0
355 859 1
355 1570 1
355 37 1
355 133 1
355 392 1
355 41 1
355 75 1
355 235 1

355 1324 1

355 270 1

355 366 1

355 57 1

355 91 1

355 252 1

355 254 1

355 673 0

355 845 0

355 2534 0

355 543 0

355 394 0

355 3179 0

355 1303 0

355 3045 0

355 1620 0

355 3340 0

355 349 0

355 2651 0

355 3447 0

355 397 0

355 2248 0

356 96 1

356 98 1

356 557 1

356 78 1

356 79 1

356 118 1

356 55 1

356 184 1

356 185 1

356 126 1

356 2903 0

356 342 0

356 3241 0

356 1704 0

356 2573 0

356 1021 0

356 834 0

356 942 0

356 630 0

356 1773 0

357 1571 1

357 36 1

357 1445 1

357 102 1

357 1572 1

357 1573 1

357 1574 1

357 1575 1

357 651 1

357 236 1

357 1486 0

357 2354 0

357 3084 0

357 995 0

357 288 0

357 2563 0

357 1053 0

357 9 0

357 462 0

357 3793 0

358 418 1

358 295 1

358 1576 1

358 137 1

358 361 1

358 1577 1

358 1578 1

358 45 1

358 110 1

358 1579 1

358 1235 1

358 1337 1

358 1469 1

358 746 0

358 839 0

358 1667 0

358 524 0

358 3808 0

358 3057 0

358 3287 0

358 154 0

358 2794 0

358 1419 0

358 410 0

358 654 0

358 1962 0

359 1551 1

359 544 1

359 673 1

359 800 1

359 1580 1

359 1581 1

359 1582 1

359 1583 1

359 1584 1

359 55 1

359 442 1

359 1471 1

359 217 1

359 96 1

359 100 1

359 740 1

359 1518 1

359 1276 1

359 1277 1

359 126 1

359 2712 0

359 3475 0

359 2571 0

359 1654 0

359 1460 0

359 3194 0

359 1032 0

359 3798 0

359 1143 0

359 1249 0

359 3108 0

359 1694 0

359 1057 0

359 253 0

359 2515 0

359 2824 0

359 741 0

359 2039 0

359 215 0

359 2211 0

360 98 1

360 135 1

360 72 1

360 73 1

360 171 1

360 77 1

360 80 1

360 81 1

360 2778 0

360 686 0

360 2344 0

360 241 0

360 3644 0

360 1262 0

360 3170 0

360 2200 0

361 96 1

361 577 1

361 304 1

361 1588 1

361 118 1

361 60 1

361 2634 0

361 575 0

361 3210 0

361 3024 0

361 2262 0

361 2528 0

362 102 1

362 232 1

362 45 1

362 78 1

362 276 1

362 1589 1

362 1590 1

362 1591 1

362 377 1

362 91 1

362 955 0

362 1431 0

362 3286 0

362 259 0

362 2401 0

362 1789 0

362 2453 0

362 1319 0

362 2334 0

362 1449 0

363 1095 1

363 1592 1

363 141 1

363 46 1

363 47 1

363 302 1

363 276 1

363 118 1

363 280 1

363 1593 1

363 1594 1

363 252 1

363 1503 1

363 2728 0

363 2486 0

363 1989 0

363 2817 0

363 3003 0

363 478 0

363 1892 0

363 330 0

363 2251 0

363 178 0

363 1240 0

363 316 0

363 898 0

364 1185 1

364 36 1

364 1254 1

364 1322 1

364 1424 1

364 1399 1

364 1016 1

364 1595 1

364 60 1

364 1597 1

364 1596 1

364 1822 0

364 142 0

364 1361 0

364 2179 0

364 109 0

364 1836 0

364 1035 0

364 532 0

364 2369 0

364 3282 0

364 58 0

365 96 1

365 325 1

365 1189 1

365 393 1

365 395 1

365 52 1

365 216 1

365 1149 1

365 2079 0

365 2081 0

365 1384 0

365 3830 0

365 1284 0

365 2940 0

365 756 0

365 1059 0

366 192 1

366 1600 1

366 227 1

366 356 1

366 357 1

366 13 1

366 275 1

366 883 1

366 1598 1

366 1599 1

366 3760 0

366 1914 0

366 921 0

366 1411 0

366 961 0

366 3509 0

366 2320 0

366 1772 0

366 2728 0

366 2878 0

367 0 1

367 320 1

367 1601 1

367 1347 1

367 4 1

367 1348 1

367 13 1

367 334 1

367 654 1

367 1303 1

367 887 1

367 857 1

367 858 1

367 1051 1

367 860 1

367 445 1

367 254 1

367 1369 0

367 544 0

367 2613 0

367 453 0

367 1856 0

367 3839 0

367 3013 0

367 2619 0

367 1884 0

367 1197 0

367 1844 0

367 3410 0

367 3293 0

367 3475 0

367 1435 0

367 81 0

367 2596 0

368 418 1

368 36 1

368 356 1

368 523 1

368 46 1

368 114 1

368 467 1

368 850 1

368 279 1

368 57 1

368 155 1

368 3088 0

368 3275 0

368 1622 0

368 1003 0

368 2626 0

368 1736 0

368 2586 0

368 2859 0

368 2214 0

368 3676 0

368 2387 0

369 832 1

369 134 1

369 377 1

369 74 1

369 75 1

369 363 1

369 47 1

369 560 1

369 434 1

369 115 1

369 185 1

369 254 1

369 127 1

369 2902 0

369 1080 0

369 685 0

369 1753 0

369 812 0

369 3486 0

369 2668 0

369 1475 0

369 2046 0

369 249 0

369 3387 0

369 1688 0

369 320 0

370 130 1

370 1602 1

370 361 1

370 74 1

370 363 1

370 79 1

370 434 1

370 919 1

370 184 1

370 185 1

370 859 1

370 254 1

370 1034 0

370 547 0

370 2888 0

370 1789 0

370 488 0

370 2805 0

370 3771 0

370 3437 0

370 1058 0

370 730 0

370 2534 0

370 586 0

371 802 1

371 1603 1

371 102 1

371 776 1

371 52 1

371 87 1

371 2643 0

371 2514 0

371 1270 0

371 721 0

371 3362 0

371 2593 0

372 393 1

372 36 1

372 325 1

372 1276 1

372 365 0

372 3362 0

372 52 0

372 2092 0

373 98 1

373 1315 1

373 4 1

373 1604 1

373 1605 1
373 1606 1
373 136 1
373 1607 1
373 1608 1
373 1609 1
373 972 1
373 973 1
373 47 1
373 80 1
373 185 1
373 557 0
373 3617 0
373 3818 0
373 3810 0
373 3390 0
373 2111 0
373 494 0
373 1647 0
373 241 0
373 1786 0
373 438 0
373 877 0
373 612 0
373 3600 0
373 2808 0
374 96 1
374 102 1
374 1610 1
374 1611 1

374 1261 1

374 494 1

374 47 1

374 1262 1

374 210 1

374 1363 1

374 1172 1

374 1589 1

374 1115 1

374 1293 0

374 2545 0

374 3416 0

374 2475 0

374 1684 0

374 2403 0

374 611 0

374 628 0

374 547 0

374 2323 0

374 328 0

374 2096 0

374 3070 0

375 128 1

375 135 1

375 72 1

375 74 1

375 1258 1

375 1612 1

375 77 1

375 1613 1

375 79 1

375 1232 1

375 1614 1

375 1202 1

375 117 1

375 1557 1

375 87 1

375 88 1

375 127 1

375 2295 0

375 2872 0

375 2377 0

375 3758 0

375 2661 0

375 2167 0

375 2790 0

375 3205 0

375 543 0

375 1138 0

375 3267 0

375 2885 0

375 1420 0

375 1270 0

375 2350 0

375 742 0

375 1093 0

376 34 1

376 930 1

376 36 1

376 1476 1

376 42 1

376 362 1

376 879 1

376 1615 1

376 1616 1

376 531 1

376 1447 0

376 1467 0

376 676 0

376 3360 0

376 1097 0

376 1210 0

376 1293 0

376 3177 0

376 1033 0

376 50 0

377 131 1

377 356 1

377 484 1

377 611 1

377 295 1

377 107 1

377 1517 1

377 1617 1

377 1586 1

377 275 1

377 852 1

377 1618 1

377 1619 1

377 1079 1

377 24 1

377 1620 1

377 1080 1

377 2839 0

377 2514 0

377 1704 0

377 1197 0

377 1621 0

377 3836 0

377 1289 0

377 259 0

377 1116 0

377 431 0

377 1608 0

377 3419 0

377 2584 0

377 1034 0

377 2090 0

377 3718 0

377 672 0

378 388 1

378 523 1

378 46 1

378 55 1

378 697 1

378 1340 0

378 2394 0

378 2608 0

378 1638 0

378 340 0

379 389 1

379 136 1

379 41 1

379 1448 1

379 117 1

379 118 1

379 55 1

379 126 1

379 127 1

379 1152 0

379 2555 0

379 109 0

379 1217 0

379 2989 0

379 3396 0

379 3545 0

379 1551 0

379 3516 0

380 266 1

380 301 1

380 305 1

380 1621 1

380 1622 1

380 3241 0

380 2299 0

380 1369 0

380 113 0

380 3628 0

381 96 1

381 128 1

381 70 1

381 1623 1

381 1624 1

381 1625 1

381 87 1

381 120 1

381 601 1

381 1626 1

381 1627 1

381 1628 1

381 1629 1

381 1630 1

381 2025 0

381 2439 0

381 570 0

381 2533 0

381 887 0

381 466 0

381 1617 0

381 1711 0

381 2395 0

381 2214 0

381 1097 0

381 1345 0

381 542 0

381 241 0

382 1632 1

382 134 1

382 267 1

382 46 1

382 1230 1

382 1231 1

382 1631 1

382 435 1

382 467 1

382 895 1

382 863 1

382 471 1

382 1461 1

382 127 1

382 1455 0

382 1210 0

382 655 0

382 1932 0

382 1692 0

382 1266 0

382 2284 0

382 1605 0

382 2044 0

382 3121 0

382 2460 0

382 1062 0

382 3296 0

382 1815 0

383 96 1

383 1633 1

383 136 1

383 74 1

383 75 1

383 560 1

383 1011 1
383 52 1
383 376 1
383 127 1
383 3133 0
383 479 0
383 826 0
383 3434 0
383 3004 0
383 3696 0
383 3370 0
383 2778 0
383 1444 0
383 1153 0
384 98 1
384 133 1
384 134 1
384 171 1
384 76 1
384 79 1
384 374 1
384 87 1
384 863 1
384 185 1
384 63 1
384 1119 0
384 2609 0
384 2486 0
384 2021 0
384 2075 0

384 3663 0

384 2726 0

384 722 0

384 1199 0

384 2624 0

384 1816 0

385 98 1

385 131 1

385 134 1

385 171 1

385 78 1

385 184 1

385 185 1

385 538 1

385 923 1

385 254 1

385 127 1

385 880 0

385 2251 0

385 1814 0

385 3571 0

385 3013 0

385 633 0

385 119 0

385 631 0

385 297 0

385 2583 0

385 1690 0

386 776 1

386 155 1

386 928 1

386 35 1

386 677 1

386 308 1

386 196 1

386 727 1

386 729 1

386 1502 1

386 1634 1

386 1635 1

386 1636 1

386 1637 1

386 1638 1

386 1639 1

386 1640 1

386 1641 1

386 1642 1

386 1643 1

386 1644 1

386 1645 1

386 1646 1

386 1647 1

386 1010 1

386 514 0

386 1387 0

386 2121 0

386 1005 0

386 1675 0

386 1963 0

386 613 0

386 2523 0

386 2271 0

386 2226 0

386 188 0

386 1785 0

386 3808 0

386 2944 0

386 2716 0

386 2323 0

386 3639 0

386 1835 0

386 1291 0

386 485 0

386 2729 0

386 71 0

386 2273 0

386 2213 0

386 862 0

387 353 1

387 37 1

387 72 1

387 106 1

387 75 1

387 237 1

387 87 1

387 2810 0

387 1735 0

387 1910 0

387 1664 0

387 192 0

387 803 0

387 3341 0

388 163 1

388 1128 1

388 106 1

388 235 1

388 236 1

388 511 1

388 878 1

388 48 1

388 1648 1

388 1649 1

388 1650 1

388 1556 1

388 282 1

388 190 1

388 159 1

388 3786 0

388 687 0

388 3534 0

388 3179 0

388 1944 0

388 117 0

388 1281 0

388 3012 0

388 141 0

388 3076 0

388 3031 0

388 274 0

388 2791 0

388 342 0

388 2019 0

389 192 1

389 1025 1

389 130 1

389 73 1

389 1258 1

389 77 1

389 81 1

389 1202 1

389 1651 1

389 1652 1

389 373 1

389 87 1

389 538 1

389 253 1

389 2010 0

389 3207 0

389 1561 0

389 1080 0

389 527 0

389 907 0

389 2108 0

389 2852 0

389 2131 0

389 1936 0

389 1519 0

389 3075 0

389 3508 0

389 596 0

390 1057 1

390 37 1

390 267 1

390 1653 1

390 1654 1

390 1655 1

390 1656 1

390 1657 1

390 1658 1

390 1659 1

390 1660 1

390 1661 1

390 2520 0

390 3201 0

390 1381 0

390 55 0

390 1959 0

390 2281 0

390 3157 0

390 3126 0

390 12 0

390 3037 0

390 657 0

390 3611 0

391 385 1

391 1091 1

391 134 1

391 135 1

391 74 1

391 75 1

391 87 1

391 127 1

391 3833 0

391 938 0

391 29 0

391 603 0

391 549 0

391 2051 0

391 37 0

391 1406 0

392 257 1

392 1665 1

392 1666 1

392 1667 1

392 133 1

392 1668 1

392 26 1

392 445 1

392 189 1

392 191 1

392 1767 0

392 1615 0

392 609 0

392 547 0

392 2637 0

392 1303 0

392 743 0

392 3839 0

392 2822 0

392 2346 0

393 325 1

393 1669 1

393 7 1

393 40 1

393 1670 1

393 654 1

393 442 1

393 756 1

393 213 1

393 119 1

393 250 1

393 347 1

393 3461 0

393 3604 0

393 2873 0

393 289 0

393 1580 0

393 2699 0

393 2332 0

393 3729 0

393 222 0

393 3403 0

393 128 0

393 1482 0

394 98 1

394 376 1

394 75 1

394 171 1

394 206 1

394 79 1

394 80 1

394 118 1

394 87 1

394 184 1

394 185 1

394 538 1

394 861 0

394 1182 0

394 433 0

394 602 0

394 2815 0

394 3737 0

394 2682 0

394 2106 0

394 1102 0

394 1100 0

394 3424 0

394 343 0

395 1671 1

395 1672 1

395 24 1

395 155 1

395 158 1

395 928 1

395 35 1

395 165 1

395 38 1

395 195 1

395 196 1

395 198 1

395 200 1

395 462 1

395 478 1

395 353 1

395 483 1

395 1136 1

395 1528 1

395 1530 1

395 3615 0

395 3511 0

395 603 0

395 438 0

395 2992 0

395 1094 0

395 263 0

395 1282 0

395 684 0

395 1896 0

395 2206 0

395 823 0

395 1984 0

395 2392 0

395 2758 0

395 3619 0

395 543 0

395 1499 0

395 1705 0

395 264 0

396 128 1

396 102 1

396 136 1

396 1673 1

396 1674 1

396 1547 1

396 1675 1

396 1589 1

396 280 1

396 507 1

396 1502 1

396 1118 0

396 2165 0

396 726 0

396 2562 0

396 375 0

396 3090 0

396 3331 0

396 361 0

396 1413 0

396 3637 0

396 2796 0

397 161 1

397 257 1

397 265 1

397 1353 1

397 1676 1

397 1677 1

397 499 1

397 26 1

397 1660 1

397 292 0

397 1195 0

397 2105 0

397 1819 0

397 1736 0

397 2313 0

397 2515 0

397 1430 0

397 2336 0

398 257 1

398 1476 1

398 1678 1

398 1679 1

398 496 1

398 1680 1

398 799 1

398 534 0

398 1401 0

398 134 0

398 2260 0

398 3718 0

398 339 0

398 2999 0

399 96 1

399 102 1

399 105 1

399 267 1

399 46 1

399 1103 1

399 114 1

399 242 1

399 52 1

399 756 1

399 55 1

399 188 1

399 3336 0

399 2785 0

399 2414 0

399 1487 0

399 1261 0

399 181 0

399 1770 0

399 3683 0

399 1750 0

399 3580 0

399 2749 0

399 1541 0

400 96 1

400 771 1

400 133 1

400 427 1

400 210 1

400 52 1

400 118 1

400 119 1

400 920 1

400 2392 0

400 1808 0

400 2194 0

400 1526 0

400 414 0

400 1609 0

400 2267 0

400 917 0

400 1642 0

401 7 1

401 1687 1

401 9 1

401 1681 1

401 242 1

401 1554 1

401 1682 1

401 125 1

401 1683 1

401 1684 1

401 1685 1

401 1686 1

401 1148 1

401 477 1

401 430 0

401 2883 0

401 3504 0

401 779 0

401 1689 0

401 3551 0

401 3194 0

401 84 0

401 3548 0

401 2474 0

401 1047 0

401 48 0

401 3158 0
401 867 0
402 163 1
402 196 1
402 676 1
402 1543 1
402 617 1
402 362 1
402 1326 1
402 51 1
402 886 1
402 1688 1
402 1689 1
402 1467 1
402 2189 0
402 1405 0
402 3034 0
402 47 0
402 219 0
402 3297 0
402 1978 0
402 1387 0
402 2201 0
402 104 0
402 2771 0
402 1380 0
403 708 1
403 197 1
403 199 1
403 776 1

403 1503 1

403 302 1

403 303 1

403 304 1

403 494 1

403 822 1

403 280 1

403 1690 1

403 1691 1

403 863 1

403 1657 0

403 3607 0

403 2600 0

403 2400 0

403 1662 0

403 3790 0

403 459 0

403 3533 0

403 1863 0

403 2364 0

403 817 0

403 1824 0

403 2225 0

403 839 0

404 1345 1

404 354 1

404 105 1

404 1327 1

404 55 1

404 567 1

404 922 1

404 1692 1

404 1693 1

404 2580 0

404 2193 0

404 1794 0

404 3782 0

404 10 0

404 2144 0

404 1641 0

404 2437 0

404 1364 0

405 1504 1

405 1696 1

405 98 1

405 1697 1

405 238 1

405 79 1

405 915 1

405 88 1

405 1694 1

405 1695 1

405 2711 0

405 1202 0

405 2806 0

405 2051 0

405 2476 0

405 2659 0

405 2631 0

405 2157 0

405 1775 0

405 1060 0

406 4 1

406 6 1

406 10 1

406 11 1

406 17 1

406 799 1

406 1698 1

406 1699 1

406 1700 1

406 1701 1

406 60 1

406 1348 1

406 971 1

406 333 1

406 337 1

406 343 1

406 498 1

406 1267 1

406 1269 1

406 1272 1

406 1273 1

406 3226 0

406 1614 0

406 2639 0

406 3242 0

406 1073 0

406 3769 0

406 267 0

406 1628 0

406 1064 0

406 1381 0

406 79 0

406 1181 0

406 1660 0

406 2613 0

406 2253 0

406 3084 0

406 1425 0

406 1619 0

406 2982 0

406 2878 0

406 3639 0

407 128 1

407 790 1

407 794 1

407 795 1

407 796 1

407 1702 1

407 1703 1

407 1704 1

407 1705 1

407 426 1

407 1706 1

407 1707 1

407 1708 1

407 1709 1

407 1710 1

407 1711 1

407 67 1
407 70 1
407 1623 1
407 601 1
407 1339 0
407 211 0
407 3621 0
407 223 0
407 1916 0
407 2978 0
407 1598 0
407 1581 0
407 221 0
407 1046 0
407 626 0
407 1626 0
407 2404 0
407 3497 0
407 2810 0
407 3245 0
407 3546 0
407 334 0
407 1090 0
407 1277 0
408 516 1
408 517 1
408 154 1
408 155 1
408 158 1
408 295 1

408 1712 1
408 1586 1
408 53 1
408 54 1
408 56 1
408 703 1
408 704 1
408 1088 1
408 196 1
408 358 1
408 616 1
408 508 1
408 878 1
408 111 1
408 764 1
408 1521 0
408 2929 0
408 2289 0
408 1974 0
408 94 0
408 201 0
408 385 0
408 3605 0
408 2780 0
408 1016 0
408 2426 0
408 2015 0
408 3720 0
408 58 0
408 199 0

408 2233 0
408 3656 0
408 3047 0
408 243 0
408 1998 0
408 3285 0
409 131 1
409 356 1
409 293 1
409 1445 1
409 1574 1
409 1713 1
409 852 1
409 1236 1
409 22 1
409 55 1
409 24 1
409 507 1
409 221 1
409 766 1
409 870 0
409 3551 0
409 3595 0
409 3687 0
409 977 0
409 3021 0
409 698 0
409 3200 0
409 3779 0
409 1971 0

409 466 0

409 3056 0

409 868 0

409 1587 0

410 74 1

410 13 1

410 270 1

410 1133 1

410 1714 1

410 1715 1

410 1716 1

410 477 1

410 445 1

410 94 0

410 2659 0

410 2465 0

410 1411 0

410 1465 0

410 3302 0

410 2717 0

410 315 0

410 672 0

411 640 1

411 34 1

411 36 1

411 1719 1

411 42 1

411 1720 1

411 624 1

411 21 1

411 1717 1

411 1718 1

411 632 1

411 1721 1

411 635 1

411 3190 0

411 1022 0

411 2469 0

411 2958 0

411 3200 0

411 775 0

411 3402 0

411 2550 0

411 1911 0

411 3706 0

411 558 0

411 2541 0

411 409 0

412 961 1

412 676 1

412 4 1

412 964 1

412 1639 1

412 1640 1

412 393 1

412 1722 1

412 315 1

412 1724 1

412 1723 1

412 3022 0

412 467 0

412 2167 0

412 2695 0

412 2869 0

412 2724 0

412 147 0

412 460 0

412 500 0

412 1732 0

412 1842 0

413 224 1

413 354 1

413 36 1

413 1725 1

413 499 1

413 1045 1

413 438 1

413 1046 1

413 445 1

413 1726 1

413 2185 0

413 1577 0

413 751 0

413 1476 0

413 297 0

413 3744 0

413 767 0

413 555 0

413 3798 0

413 2081 0

414 1728 1
414 33 1
414 1729 1
414 195 1
414 967 1
414 200 1
414 508 1
414 1727 1
414 1397 0
414 546 0
414 1422 0
414 2021 0
414 1009 0
414 9 0
414 291 0
414 2029 0
415 928 1
415 97 1
415 803 1
415 36 1
415 1731 1
415 7 1
415 42 1
415 1148 1
415 1214 1
415 1442 0
415 927 0
415 940 0
415 1872 0
415 215 0

415 2686 0
415 1103 0
415 112 0
415 1254 0
416 929 1
416 34 1
416 451 1
416 228 1
416 36 1
416 1732 1
416 1733 1
416 1734 1
416 1673 1
416 42 1
416 362 1
416 1735 1
416 1140 1
416 2378 0
416 1452 0
416 107 0
416 146 0
416 3103 0
416 1829 0
416 1984 0
416 3147 0
416 3124 0
416 1933 0
416 2934 0
416 1737 0
416 1396 0

417 96 1

417 1736 1

417 267 1

417 78 1

417 79 1

417 80 1

417 87 1

417 184 1

417 185 1

417 538 1

417 417 0

417 764 0

417 2369 0

417 2627 0

417 2880 0

417 960 0

417 306 0

417 3195 0

417 2692 0

417 652 0

418 257 1

418 36 1

418 1737 1

418 141 1

418 1293 1

418 276 1

418 788 1

418 26 1

418 30 1

418 191 1

418 96 0

418 1563 0

418 1778 0

418 1809 0

418 1301 0

418 901 0

418 1839 0

418 3321 0

418 2709 0

418 2598 0

419 354 1

419 1738 1

419 109 1

419 141 1

419 863 1

419 277 0

419 2922 0

419 2779 0

419 2652 0

419 512 0

420 3842 1

420 3843 1

420 3844 1

420 3845 1

420 2572 1

420 541 1

420 546 1

420 164 1

420 676 1

420 678 1

420 933 1

420 2858 1

420 307 1

420 320 1

420 960 1

420 224 1

420 226 1

420 227 1

420 356 1

420 3057 1

420 883 1

420 1269 1

420 887 1

420 1271 1

420 2785 0

420 346 0

420 3432 0

420 3172 0

420 1724 0

420 1389 0

420 1930 0

420 2893 0

420 3344 0

420 1463 0

420 405 0

420 674 0

420 1214 0

420 1361 0

420 3189 0

420 1843 0

420 3385 0

420 2387 0

420 551 0

420 1223 0

420 685 0

420 1302 0

420 68 0

420 419 0

421 35 1

421 196 1

421 100 1

421 1739 1

421 460 1

421 684 1

421 654 1

421 975 1

421 1740 1

421 498 1

421 307 1

421 886 1

421 24 1

421 1338 1

421 155 1

421 732 1

421 286 1

421 1229 0

421 3162 0

421 3507 0

421 1176 0

421 244 0

421 2886 0

421 2280 0

421 3196 0

421 2192 0

421 2936 0

421 327 0

421 1886 0

421 67 0

421 3739 0

421 1536 0

421 3446 0

421 178 0

422 1185 1

422 1186 1

422 134 1

422 1736 1

422 171 1

422 1741 1

422 1742 1

422 560 1

422 1424 1

422 3095 0

422 149 0

422 2884 0

422 2649 0

422 1558 0

422 2697 0

422 1493 0

422 1445 0

422 75 0

423 97 1
423 681 1
423 42 1
423 777 1
423 1743 1
423 442 1
423 1744 1
423 1745 1
423 243 1
423 244 1
423 1746 1
423 1747 1
423 250 1
423 126 1
423 164 0
423 2154 0
423 1326 0
423 226 0
423 951 0
423 1330 0
423 3525 0
423 1142 0
423 2490 0
423 2519 0
423 1366 0
423 4 0
423 2054 0
423 984 0
424 36 1
424 1502 1

424 107 1

424 46 1

424 47 1

424 114 1

424 118 1

424 155 1

424 989 1

424 126 1

424 3309 0

424 2690 0

424 364 0

424 3028 0

424 2189 0

424 3480 0

424 173 0

424 1656 0

424 1385 0

424 1888 0

425 771 1

425 805 1

425 7 1

425 242 1

425 788 1

425 1748 1

425 1749 1

425 2034 0

425 2295 0

425 487 0

425 2114 0

425 2101 0

425 478 0

425 1935 0

426 356 1

426 196 1

426 391 1

426 1036 1

426 109 1

426 46 1

426 493 1

426 438 1

426 155 1

426 1342 1

426 1311 1

426 672 0

426 2516 0

426 1506 0

426 2905 0

426 1885 0

426 1566 0

426 562 0

426 106 0

426 1492 0

426 503 0

426 2036 0

427 133 1

427 1751 1

427 787 1

427 20 1

427 52 1

427 1750 1

427 727 1

427 1752 1

427 252 1

427 583 0

427 3296 0

427 2705 0

427 283 0

427 312 0

427 1840 0

427 3237 0

427 3451 0

427 500 0

428 1416 1

428 681 1

428 557 1

428 719 1

428 48 1

428 51 1

428 54 1

428 1753 1

428 670 1

428 3415 0

428 34 0

428 3459 0

428 1104 0

428 1611 0

428 1007 0

428 427 0

428 2455 0

428 3250 0

429 195 1

429 554 1

429 1754 1

429 1583 1

429 895 1

429 729 1

429 442 1

429 60 1

429 1277 1

429 478 1

429 447 1

429 3490 0

429 696 0

429 3336 0

429 3222 0

429 742 0

429 1516 0

429 2240 0

429 3042 0

429 3593 0

429 3495 0

429 944 0

430 1756 1

430 105 1

430 1758 1

430 46 1

430 697 1

430 55 1

430 1755 1

430 571 1

430 187 1

430 764 1

430 1757 1

430 190 1

430 1759 1

430 1072 0

430 3310 0

430 2273 0

430 221 0

430 1902 0

430 1788 0

430 636 0

430 3627 0

430 704 0

430 3122 0

430 2399 0

430 185 0

430 588 0

431 354 1

431 388 1

431 293 1

431 389 1

431 516 1

431 392 1

431 105 1

431 109 1

431 46 1

431 766 1

431 112 1

431 1103 1

431 52 1

431 54 1

431 55 1

431 190 1

431 1335 0

431 3503 0

431 2891 0

431 287 0

431 2146 0

431 553 0

431 345 0

431 1296 0

431 1969 0

431 1782 0

431 2658 0

431 1572 0

431 397 0

431 2681 0

431 3076 0

431 2355 0

432 1760 1

432 98 1

432 134 1

432 80 1

432 1200 1

432 114 1

432 254 1

432 87 1

432 472 1

432 539 1

432 126 1

432 3774 0

432 430 0

432 3111 0

432 212 0

432 3317 0

432 3654 0

432 2882 0

432 1164 0

432 2015 0

432 652 0

432 767 0

433 1761 1

433 1762 1

433 1763 1

433 1764 1

433 1765 1

433 1766 1

433 1767 1

433 1768 1

433 1769 1

433 1770 1

433 1771 1

433 1772 1

433 1773 1

433 1774 1

433 49 1

433 3253 0

433 2056 0

433 1579 0

433 2789 0
433 1074 0
433 2269 0
433 3433 0
433 2875 0
433 3126 0
433 1638 0
433 729 0
433 1933 0
433 3516 0
433 1757 0
433 879 0
434 608 1
434 353 1
434 681 1
434 554 1
434 1775 1
434 468 1
434 799 1
434 1713 0
434 2297 0
434 2850 0
434 2875 0
434 1343 0
434 1843 0
434 2024 0
435 36 1
435 42 1
435 1166 1
435 1776 1

435 1777 1

435 1778 1

435 211 1

435 1395 1

435 250 1

435 285 1

435 126 1

435 286 1

435 627 0

435 1116 0

435 1407 0

435 424 0

435 1193 0

435 262 0

435 231 0

435 1056 0

435 671 0

435 3092 0

435 1213 0

435 2918 0

436 377 1

436 74 1

436 171 1

436 79 1

436 919 1

436 185 1

436 542 1

436 127 1

436 2148 0

436 1033 0

436	1420	0
436	949	0
436	3270	0
436	49	0
436	3253	0
436	2526	0
437	964	1
437	36	1
437	111	1
437	2176	0
437	3550	0
437	2626	0
438	1185	1
438	98	1
438	130	1
438	135	1
438	1258	1
438	80	1
438	118	1
438	55	1
438	376	1
438	185	1
438	471	0
438	2755	0
438	3540	0
438	2872	0
438	1430	0
438	2169	0
438	1708	0
438	1901	0

438 838 0

438 982 0

439 385 1

439 134 1

439 809 1

439 1418 1

439 75 1

439 1783 1

439 1779 1

439 1780 1

439 1781 1

439 1590 1

439 1782 1

439 1784 1

439 1785 1

439 1786 1

439 91 1

439 1788 1

439 1787 1

439 127 1

439 1311 0

439 1776 0

439 170 0

439 1695 0

439 2443 0

439 2314 0

439 1396 0

439 1620 0

439 3433 0

439 908 0

439 970 0

439 1925 0

439 3249 0

439 2471 0

439 1606 0

439 1111 0

439 428 0

439 365 0

440 96 1

440 1312 1

440 100 1

440 261 1

440 356 1

440 358 1

440 1789 1

440 80 1

440 1234 1

440 253 1

440 1790 1

440 1791 1

440 3635 0

440 3100 0

440 2469 0

440 2308 0

440 3317 0

440 2399 0

440 114 0

440 1076 0

440 1660 0

440 1272 0

440 2816 0
440 1140 0
441 1792 1
441 1793 1
441 293 1
441 389 1
441 391 1
441 712 1
441 105 1
441 45 1
441 46 1
441 528 1
441 118 1
441 55 1
441 56 1
441 188 1
441 190 1
441 177 0
441 2598 0
441 2326 0
441 2845 0
441 2941 0
441 11 0
441 2874 0
441 2349 0
441 2129 0
441 3611 0
441 876 0
441 3270 0
441 985 0

441 1408 0

441 3673 0

442 96 1

442 738 1

442 293 1

442 105 1

442 107 1

442 46 1

442 47 1

442 696 1

442 55 1

442 120 1

442 571 1

442 1259 0

442 257 0

442 713 0

442 1761 0

442 2759 0

442 75 0

442 3513 0

442 1834 0

442 1780 0

442 2158 0

442 1202 0

443 66 1

443 131 1

443 77 1

443 47 1

443 80 1

443 433 1

443 87 1

443 126 1

443 127 1

443 213 0

443 2926 0

443 1513 0

443 3606 0

443 28 0

443 2058 0

443 2537 0

443 2323 0

443 1443 0

444 1253 1

444 102 1

444 105 1

444 941 1

444 1133 1

444 1261 1

444 1172 1

444 920 1

444 219 1

444 876 0

444 8 0

444 2270 0

444 1767 0

444 2984 0

444 1488 0

444 1121 0

444 1758 0

444 2588 0

445 897 1

445 133 1

445 135 1

445 81 1

445 118 1

445 87 1

445 3146 0

445 3075 0

445 2593 0

445 3621 0

445 2481 0

445 2217 0

446 257 1

446 1794 1

446 1795 1

446 260 1

446 1796 1

446 1529 1

446 686 1

446 1105 1

446 498 1

446 275 1

446 500 1

446 25 1

446 3068 0

446 738 0

446 2379 0

446 3122 0

446 2617 0

446 2912 0

446 2808 0

446 1432 0

446 2771 0

446 104 0

446 2110 0

446 3247 0

447 131 1

447 104 1

447 137 1

447 235 1

447 78 1

447 469 1

447 863 1

447 187 1

447 413 1

447 254 1

447 127 1

447 545 0

447 3656 0

447 1556 0

447 1694 0

447 3808 0

447 478 0

447 986 0

447 1484 0

447 2478 0

447 418 0

447 623 0

448 1797 1

448 102 1

448 1798 1
448 118 1
448 55 1
448 473 1
448 858 1
448 190 1
448 346 0
448 3709 0
448 233 0
448 3171 0
448 2189 0
448 1538 0
448 2442 0
448 3189 0
449 288 1
449 738 1
449 100 1
449 20 1
449 52 1
449 729 1
449 155 1
449 476 1
449 254 1
449 2700 0
449 1129 0
449 1861 0
449 3241 0
449 427 0
449 2003 0
449 1042 0

449 2333 0

449 1219 0

450 229 1

450 325 1

450 197 1

450 1801 1

450 1514 1

450 1802 1

450 1803 1

450 1389 1

450 1804 1

450 1805 1

450 52 1

450 1662 1

450 1632 0

450 1405 0

450 1849 0

450 527 0

450 1830 0

450 1882 0

450 2269 0

450 3370 0

450 3260 0

450 1969 0

450 1914 0

450 3776 0

451 135 1

451 66 1

451 1244 1

451 87 1

451 522 0

451 3182 0

451 385 0

451 1981 0

452 66 1

452 98 1

452 1806 1

452 115 1

452 406 1

452 951 1

452 184 1

452 377 1

452 1251 0

452 238 0

452 526 0

452 2059 0

452 24 0

452 369 0

452 2408 0

452 3263 0

453 608 1

453 391 1

453 105 1

453 523 1

453 109 1

453 46 1

453 1807 1

453 60 1

453 190 1

453 286 1

453 2602 0

453 2242 0

453 616 0

453 2218 0

453 3037 0

453 2031 0

453 1484 0

453 2793 0

453 2224 0

453 1249 0

454 36 1

454 105 1

454 141 1

454 1808 1

454 1617 1

454 1140 1

454 2727 0

454 85 0

454 1940 0

454 777 0

454 2424 0

454 803 0

455 545 1

455 104 1

455 105 1

455 1608 1

455 78 1

455 46 1

455 47 1

455 1809 1

455 915 1

455 118 1

455 55 1

455 126 1

455 863 1

455 2180 0

455 1585 0

455 2696 0

455 1778 0

455 727 0

455 2999 0

455 653 0

455 1545 0

455 2274 0

455 324 0

455 3416 0

455 2834 0

455 1638 0

456 34 1

456 36 1

456 522 1

456 1810 1

456 147 1

456 788 1

456 1811 1

456 346 1

456 991 1

456 1174 0

456 71 0

456 2109 0

456 3507 0
456 1906 0
456 1910 0
456 2798 0
456 1277 0
456 523 0
457 715 1
457 1816 1
457 1812 1
457 1813 1
457 1814 1
457 1815 1
457 56 1
457 1817 1
457 511 1
457 798 0
457 1200 0
457 1438 0
457 2625 0
457 3636 0
457 2348 0
457 1548 0
457 3409 0
457 2938 0
458 96 1
458 227 1
458 1818 1
458 445 1
458 30 1
458 61 0

458 1562 0
458 3242 0
458 532 0
458 2167 0
459 132 1
459 72 1
459 137 1
459 74 1
459 237 1
459 238 1
459 79 1
459 406 1
459 951 1
459 184 1
459 185 1
459 1819 1
459 1820 1
459 95 0
459 1743 0
459 776 0
459 193 0
459 3086 0
459 1918 0
459 2198 0
459 1451 0
459 2524 0
459 1036 0
459 1135 0
459 2484 0
459 2376 0

460 66 1

460 206 1

460 79 1

460 560 1

460 87 1

460 1821 1

460 830 1

460 3388 0

460 894 0

460 3476 0

460 3572 0

460 3272 0

460 3508 0

460 441 0

461 3 1

461 4 1

461 36 1

461 329 1

461 42 1

461 332 1

461 13 1

461 334 1

461 273 1

461 337 1

461 1268 1

461 277 1

461 1303 1

461 126 1

461 1852 0

461 962 0

461 652 0

461 952 0

461 2102 0

461 308 0

461 2967 0

461 3438 0

461 886 0

461 168 0

461 2270 0

461 2547 0

461 2287 0

461 2214 0

462 131 1

462 134 1

462 74 1

462 171 1

462 79 1

462 538 1

462 91 1

462 1708 0

462 790 0

462 1404 0

462 2254 0

462 102 0

462 829 0

462 2838 0

463 41 1

463 74 1

463 75 1

463 77 1

463 590 1
463 80 1
463 81 1
463 117 1
463 87 1
463 58 1
463 127 1
463 461 0
463 829 0
463 1663 0
463 1075 0
463 3281 0
463 1098 0
463 419 0
463 2893 0
463 2000 0
463 1606 0
463 1070 0
464 1315 1
464 446 1
464 109 1
464 499 1
464 245 1
464 445 1
464 126 1
464 127 1
464 2573 0
464 2892 0
464 2478 0
464 787 0

464 764 0

464 2447 0

464 2320 0

464 3360 0

465 800 1

465 196 1

465 1487 1

465 1823 1

465 314 1

465 155 1

465 156 1

465 1822 1

465 479 1

465 3672 0

465 682 0

465 2195 0

465 814 0

465 584 0

465 756 0

465 3302 0

465 1468 0

465 1606 0

466 832 1

466 41 1

466 171 1

466 267 1

466 79 1

466 399 1

466 1461 1

466 87 1

466 184 1

466 254 1

466 127 1

466 3464 0

466 2219 0

466 830 0

466 1199 0

466 3682 0

466 3398 0

466 3174 0

466 3391 0

466 1055 0

466 2577 0

466 1655 0

467 1824 1

467 66 1

467 1412 1

467 135 1

467 72 1

467 237 1

467 87 1

467 539 1

467 679 0

467 1047 0

467 1315 0

467 2930 0

467 1739 0

467 2626 0

467 3407 0

467 701 0

468 1825 1
468 1412 1
468 361 1
468 171 1
468 78 1
468 79 1
468 538 1
468 87 1
468 376 1
468 442 1
468 254 1
468 127 1
468 2474 0
468 3796 0
468 2306 0
468 612 0
468 2126 0
468 2052 0
468 1610 0
468 2179 0
468 2853 0
468 3295 0
468 3801 0
468 3660 0
469 36 1
469 376 1
469 102 1
469 78 1
469 47 1
469 304 1

469 184 1

469 185 1

469 1226 0

469 962 0

469 1219 0

469 335 0

469 929 0

469 1165 0

469 3338 0

469 2330 0

470 227 1

470 4 1

470 1699 1

470 1827 1

470 1828 1

470 104 1

470 1829 1

470 46 1

470 15 1

470 1422 1

470 118 1

470 343 1

470 1691 1

470 3210 0

470 2595 0

470 1502 0

470 3089 0

470 1412 0

470 1837 0

470 1366 0

470 3754 0

470 3424 0

470 972 0

470 3131 0

470 3639 0

470 1040 0

471 1345 1

471 1346 1

471 1347 1

471 1348 1

471 1830 1

471 1831 1

471 1832 1

471 1833 1

471 1834 1

471 1835 1

471 1836 1

471 1837 1

471 46 1

471 1838 1

471 1051 1

471 1469 1

471 2530 0

471 490 0

471 830 0

471 3698 0

471 2273 0

471 3172 0

471 3110 0

471 1299 0

471 1158 0

471 1817 0

471 228 0

471 3035 0

471 2651 0

471 2206 0

471 1078 0

471 2583 0

472 1089 1

472 290 1

472 1839 1

472 1840 1

472 1841 1

472 1842 1

472 1843 1

472 1844 1

472 1845 1

472 1846 1

472 1847 1

472 1848 1

472 2545 0

472 612 0

472 7 0

472 2038 0

472 1585 0

472 3291 0

472 3199 0

472 3814 0

472 451 0

472 3700 0

472 3284 0
472 218 0
473 96 1
473 209 1
473 210 1
473 1297 1
473 52 1
473 118 1
473 726 1
473 184 1
473 1849 1
473 1183 0
473 2572 0
473 3754 0
473 722 0
473 1709 0
473 1420 0
473 3650 0
473 2861 0
473 672 0
474 96 1
474 2 1
474 1346 1
474 1419 1
474 118 1
474 507 1
474 3046 0
474 2186 0
474 3234 0
474 2815 0

474 3159 0

474 2217 0

475 1536 1

475 36 1

475 228 1

475 105 1

475 109 1

475 111 1

475 210 1

475 55 1

475 57 1

475 442 1

475 765 1

475 2741 0

475 917 0

475 40 0

475 2174 0

475 1589 0

475 605 0

475 1343 0

475 1062 0

475 3618 0

475 2281 0

475 3301 0

476 800 1

476 100 1

476 43 1

476 653 1

476 1262 1

476 210 1

476 211 1

476 217 1

476 860 1

476 3537 0

476 2568 0

476 325 0

476 2903 0

476 3561 0

476 2412 0

476 3580 0

476 2538 0

476 3272 0

477 1851 1

477 388 1

477 1853 1

477 1855 1

477 46 1

477 528 1

477 1854 1

477 53 1

477 54 1

477 55 1

477 24 1

477 118 1

477 1850 1

477 187 1

477 1852 1

477 413 1

477 766 1

477 767 1

477 112 0

477 1871 0

477 300 0

477 3270 0

477 3758 0

477 539 0

477 2454 0

477 3508 0

477 890 0

477 3101 0

477 3621 0

477 3831 0

477 1596 0

477 218 0

477 3635 0

477 3523 0

477 3802 0

477 757 0

478 1856 1

478 1857 1

478 1858 1

478 473 1

478 79 1

478 47 1

478 399 1

478 916 1

478 118 1

478 87 1

478 184 1

478 185 1

478 378 1

478 406 1

478 860 1

478 254 1

478 5 0

478 1237 0

478 3308 0

478 1377 0

478 1661 0

478 877 0

478 1590 0

478 564 0

478 1805 0

478 2956 0

478 1095 0

478 2513 0

478 3219 0

478 73 0

478 3828 0

478 367 0

479 512 1

479 1859 1

479 1860 1

479 1861 1

479 422 1

479 105 1

479 695 1

479 715 1

479 46 1

479 111 1

479 878 1

479 1393 1

479 1678 1

479 23 1

479 55 1

479 508 1

479 2523 0

479 801 0

479 1869 0

479 2022 0

479 1847 0

479 2157 0

479 82 0

479 2414 0

479 2106 0

479 3829 0

479 1149 0

479 1389 0

479 1960 0

479 3472 0

479 1266 0

479 47 0

480 1315 1

480 933 1

480 102 1

480 902 1

480 1862 1

480 1863 1

480 304 1

480 915 1

480 277 1

480 254 1

480 126 1

480 2834 0

480 3040 0

480 1737 0

480 3784 0

480 2018 0

480 1480 0

480 3504 0

480 2799 0

480 2632 0

480 582 0

480 1154 0

481 163 1

481 422 1

481 295 1

481 1864 1

481 1865 1

481 852 1

481 821 1

481 56 1

481 1081 1

481 187 1

481 508 1

481 510 1

481 1403 0

481 304 0

481 2110 0

481 3757 0

481 3352 0

481 26 0

481 2046 0

481 2014 0

481 1500 0

481 1186 0

481 635 0

481 1382 0

482 66 1

482 130 1

482 135 1

482 206 1

482 560 1

482 87 1

482 185 1

482 672 0

482 139 0

482 241 0

482 952 0

482 2194 0

482 3157 0

482 1420 0

483 96 1

483 477 1

483 6 1

483 40 1

483 1224 1

483 1321 1

483 241 1

483 245 1

483 285 1
483 2756 0
483 1178 0
483 3513 0
483 1358 0
483 145 0
483 719 0
483 3165 0
483 1187 0
483 510 0
484 227 1
484 771 1
484 1828 1
484 1866 1
484 43 1
484 1867 1
484 13 1
484 498 1
484 915 1
484 24 1
484 442 1
484 1341 1
484 445 1
484 3370 0
484 1499 0
484 1833 0
484 1362 0
484 2436 0
484 2066 0
484 1404 0

484 3385 0

484 228 0

484 163 0

484 1973 0

484 231 0

484 3240 0

485 96 1

485 776 1

485 105 1

485 714 1

485 1868 1

485 45 1

485 46 1

485 47 1

485 301 1

485 1869 1

485 1870 1

485 1172 1

485 118 1

485 282 1

485 219 1

485 1180 1

485 1985 0

485 538 0

485 386 0

485 3188 0

485 3461 0

485 930 0

485 2392 0

485 2545 0

485 673 0
485 2703 0
485 54 0
485 1514 0
485 391 0
485 2981 0
485 978 0
485 1931 0
486 608 1
486 36 1
486 105 1
486 235 1
486 141 1
486 46 1
486 112 1
486 1874 1
486 1875 1
486 1876 1
486 764 1
486 1456 0
486 2878 0
486 2981 0
486 600 0
486 3181 0
486 3221 0
486 1971 0
486 2512 0
486 3129 0
486 1922 0
486 3079 0

487 352 1

487 802 1

487 199 1

487 714 1

487 302 1

487 303 1

487 47 1

487 210 1

487 756 1

487 785 0

487 2141 0

487 2929 0

487 3282 0

487 1035 0

487 3514 0

487 1022 0

487 3637 0

487 2250 0

488 128 1

488 771 1

488 899 1

488 102 1

488 998 1

488 105 1

488 755 1

488 52 1

488 1877 1

488 357 0

488 1901 0

488 242 0

488 530 0

488 1049 0

488 2030 0

488 2366 0

488 3628 0

488 1759 0

489 66 1

489 135 1

489 137 1

489 233 1

489 1258 1

489 1325 1

489 117 1

489 374 1

489 698 1

489 923 1

489 957 1

489 3507 0

489 1823 0

489 2601 0

489 1233 0

489 3186 0

489 2250 0

489 3127 0

489 3803 0

489 1728 0

489 715 0

489 3771 0

490 36 1

490 1734 1

490 362 1

490 879 1

490 1362 1

490 275 1

490 1491 1

490 1878 1

490 1879 1

490 1533 1

490 1694 0

490 3448 0

490 2389 0

490 1369 0

490 914 0

490 1998 0

490 3434 0

490 965 0

490 211 0

490 420 0

491 192 1

491 1883 1

491 137 1

491 1385 1

491 1545 1

491 1821 1

491 1880 1

491 854 1

491 55 1

491 918 1

491 1881 1

491 1882 1

491 187 1

491 1725 1

491 75 0

491 772 0

491 145 0

491 1 0

491 1755 0

491 1905 0

491 965 0

491 2410 0

491 2106 0

491 1911 0

491 3359 0

491 1043 0

491 1901 0

491 3244 0

492 135 1

492 267 1

492 1324 1

492 78 1

492 79 1

492 206 1

492 184 1

492 185 1

492 538 1

492 698 1

492 127 1

492 761 0

492 3759 0

492 1178 0

492 2865	0
492 2108	0
492 1170	0
492 1059	0
492 2992	0
492 1094	0
492 1632	0
492 3731	0
493 102 1
493 713 1
493 266 1
493 714 1
493 301 1
493 46	1
493 302 1
493 303 1
493 305 1
493 1297	1
493 52	1
493 920 1
493 967 0
493 917 0
493 906 0
493 622 0
493 1858	0
493 313 0
493 3254	0
493 1813	0
493 3279	0
493 1571	0

493 2452 0

493 2056 0

494 1601 1

494 66 1

494 547 1

494 551 1

494 1885 1

494 558 1

494 662 1

494 1142 1

494 1884 1

494 893 1

494 1886 1

494 383 1

494 3208 0

494 3100 0

494 3409 0

494 1310 0

494 1541 0

494 1702 0

494 1961 0

494 2043 0

494 2186 0

494 3599 0

494 81 0

494 3157 0

495 132 1

495 135 1

495 72 1

495 73 1

495 80 1

495 87 1

495 445 1

495 2353 0

495 1425 0

495 1029 0

495 1995 0

495 1752 0

495 3626 0

495 2101 0

496 832 1

496 74 1

496 1230 1

496 399 1

496 80 1

496 830 1

496 437 1

496 917 1

496 1397 1

496 377 1

496 538 1

496 126 1

496 1887 1

496 2192 0

496 3366 0

496 51 0

496 154 0

496 1933 0

496 3148 0

496 3525 0

496 2493	0
496 2724	0
496 1772	0
496 3217	0
496 2263	0
496 976 0
497 36	1
497 548 1
497 41	1
497 364 1
497 52	1
497 542 1
497 2406	0
497 2906	0
497 657 0
497 1575	0
497 323 0
497 2045	0
498 1888	1
498 841 1
498 363 1
498 79	1
498 1199	1
498 52	1
498 758 1
498 698 1
498 2986	0
498 1127	0
498 1945	0
498 1134	0

498 3438 0

498 245 0

498 689 0

498 2636 0

499 1889 1

499 130 1

499 1890 1

499 135 1

499 377 1

499 74 1

499 80 1

499 374 1

499 376 1

499 185 1

499 538 1

499 3461 0

499 1804 0

499 1602 0

499 3122 0

499 941 0

499 938 0

499 1686 0

499 3755 0

499 1589 0

499 1882 0

499 2680 0

500 96 1

500 1057 1

500 66 1

500 1411 1

500 1891 1

500 1892 1

500 1893 1

500 40 1

500 109 1

500 47 1

500 118 1

500 120 1

500 474 1

500 445 1

500 2509 0

500 2717 0

500 2518 0

500 3030 0

500 386 0

500 1245 0

500 999 0

500 2275 0

500 1635 0

500 2405 0

500 3814 0

500 1488 0

500 2219 0

500 1335 0

501 1894 1

501 104 1

501 882 1

501 499 1

501 883 1

501 277 1

501 22 1

501 1174 1

501 1493 1

501 25 1

501 889 1

501 799 1

501 2343 0

501 834 0

501 837 0

501 951 0

501 724 0

501 2479 0

501 2074 0

501 808 0

501 3379 0

501 2335 0

501 1877 0

501 2140 0

502 34 1

502 36 1

502 774 1

502 1895 1

502 362 1

502 20 1

502 1397 1

502 512 0

502 2796 0

502 3264 0

502 2913 0

502 2992 0

502 1812 0

502 2663 0

503 35 1

503 293 1

503 40 1

503 104 1

503 1896 1

503 46 1

503 114 1

503 245 1

503 22 1

503 24 1

503 286 1

503 2366 0

503 2946 0

503 2387 0

503 2245 0

503 1816 0

503 1640 0

503 3485 0

503 1566 0

503 3314 0

503 2991 0

503 3477 0

504 355 1

504 1031 1

504 1897 1

504 1898 1

504 1804 1

504 213 1

504 1078 1
504 1496 1
504 1020 1
504 1021 1
504 926 1
504 1056 0
504 3068 0
504 1601 0
504 92 0
504 1812 0
504 763 0
504 2056 0
504 2341 0
504 59 0
504 610 0
504 1012 0
505 96 1
505 1122 1
505 133 1
505 43 1
505 1899 1
505 45 1
505 494 1
505 47 1
505 1651 1
505 118 1
505 126 1
505 1570 0
505 1315 0
505 187 0

505 379 0

505 3085 0

505 998 0

505 2022 0

505 1298 0

505 104 0

505 1621 0

505 1501 0

506 34 1

506 36 1

506 740 1

506 9 1

506 42 1

506 1290 1

506 1900 1

506 1901 1

506 1902 1

506 1903 1

506 242 1

506 211 1

506 883 1

506 444 1

506 1797 0

506 596 0

506 2092 0

506 3762 0

506 3182 0

506 302 0

506 3627 0

506 1344 0

506 2089 0

506 3704 0

506 1179 0

506 846 0

506 3119 0

506 679 0

507 1664 1

507 36 1

507 228 1

507 388 1

507 105 1

507 765 1

507 939 1

507 109 1

507 1105 1

507 306 1

507 157 1

507 223 1

507 40 0

507 1425 0

507 2761 0

507 2533 0

507 548 0

507 2059 0

507 2832 0

507 1148 0

507 134 0

507 3335 0

507 1621 0

507 517 0

508 1 1

508 3 1

508 996 1

508 1029 1

508 9 1

508 42 1

508 12 1

508 749 1

508 1904 1

508 1905 1

508 18 1

508 1906 1

508 1907 1

508 1146 1

508 1148 1

508 185 0

508 2775 0

508 2033 0

508 626 0

508 310 0

508 1552 0

508 2939 0

508 511 0

508 2173 0

508 1915 0

508 3078 0

508 22 0

508 2001 0

508 240 0

508 3438 0

509 608 1

509 867 1

509 36 1

509 1105 1

509 1812 1

509 1908 1

509 1909 1

509 1214 1

509 1197 0

509 3610 0

509 3117 0

509 2755 0

509 300 0

509 2278 0

509 1868 0

509 385 0

510 325 1

510 1445 1

510 42 1

510 44 1

510 242 1

510 788 1

510 1910 1

510 216 1

510 3355 0

510 2317 0

510 3602 0

510 3140 0

510 3033 0

510 1410 0

510 2498 0

510 3211 0

511 47 1

511 1168 1

511 1777 1

511 210 1

511 1911 1

511 118 1

511 55 1

511 154 1

511 1020 1

511 778 0

511 2716 0

511 2063 0

511 2564 0

511 3657 0

511 2997 0

511 1393 0

511 83 0

511 2467 0

512 576 1

512 1912 1

512 1913 1

512 1914 1

512 1915 1

512 3307 0

512 1248 0

512 2994 0

512 2067 0

512 217 0

513 66 1

513 132 1

513 177 1

513 404 1

513 87 1

513 1916 1

513 1613 0

513 1095 0

513 414 0

513 1489 0

513 3682 0

513 1060 0

514 1920 1

514 1921 1

514 98 1

514 72 1

514 237 1

514 1549 1

514 81 1

514 88 1

514 1917 1

514 1918 1

514 1919 1

514 1943 0

514 1416 0

514 3706 0

514 672 0

514 1456 0

514 1210 0

514 1032 0

514 1819 0

514 3086 0

514 1427 0

514 1868 0

515 1922 1

515 1923 1

515 708 1

515 261 1

515 165 1

515 358 1

515 41 1

515 1197 1

515 50 1

515 438 1

515 442 1

515 2329 0

515 2541 0

515 1442 0

515 3179 0

515 1937 0

515 2981 0

515 489 0

515 2443 0

515 3593 0

515 97 0

515 1792 0

516 1924 1

516 1925 1

516 8 1

516 15 1

516 1311 1
516 675 1
516 1315 1
516 443 1
516 1608 1
516 329 1
516 332 1
516 972 1
516 334 1
516 335 1
516 973 1
516 337 1
516 1374 1
516 1270 1
516 126 1
516 1053 0
516 3433 0
516 572 0
516 1852 0
516 905 0
516 2988 0
516 2051 0
516 2966 0
516 1707 0
516 2573 0
516 632 0
516 894 0
516 952 0
516 1704 0
516 100 0

516 729 0

516 42 0

516 208 0

516 3393 0

517 755 1

517 132 1

517 3287 0

517 2161 0

518 608 1

518 36 1

518 1926 1

518 1927 1

518 1928 1

518 52 1

518 1662 1

518 1698 0

518 318 0

518 2632 0

518 2329 0

518 3124 0

518 819 0

518 211 0

519 0 1

519 257 1

519 4 1

519 356 1

519 295 1

519 1929 1

519 107 1

519 45 1

519 883 1

519 277 1

519 1175 1

519 190 1

519 223 1

519 2360 0

519 3792 0

519 3428 0

519 3546 0

519 2754 0

519 2070 0

519 663 0

519 1993 0

519 647 0

519 366 0

519 1023 0

519 1086 0

519 2281 0

520 1930 1

520 1931 1

520 1932 1

520 1933 1

520 1934 1

520 1935 1

520 1936 1

520 1937 1

520 1938 1

520 1939 1

520 419 1

520 1711 1

520 1202 1
520 1207 1
520 58 1
520 192 1
520 1228 1
520 77 1
520 1613 1
520 1232 1
520 171 0
520 1236 0
520 136 0
520 2490 0
520 2942 0
520 815 0
520 1500 0
520 2566 0
520 1189 0
520 741 0
520 2868 0
520 853 0
520 3481 0
520 590 0
520 3277 0
520 1394 0
520 2658 0
520 510 0
520 2918 0
520 164 0
521 672 1
521 232 1

521 1325 1

521 1940 1

521 1941 1

521 1439 1

521 1712 0

521 3698 0

521 895 0

521 2197 0

521 1010 0

521 3716 0

522 1191 1

522 145 1

522 146 1

522 147 1

522 820 1

522 785 1

522 1942 1

522 1943 1

522 1944 1

522 1945 1

522 1946 1

522 1947 1

522 1948 1

522 1349 0

522 553 0

522 3282 0

522 1699 0

522 2356 0

522 224 0

522 1381 0

522 2720 0

522 2419 0

522 3718 0

522 2010 0

522 1464 0

522 2957 0

523 161 1

523 257 1

523 293 1

523 1257 1

523 1949 1

523 858 1

523 275 1

523 22 1

523 24 1

523 26 1

523 221 1

523 1950 1

523 445 1

523 2121 0

523 1182 0

523 2424 0

523 289 0

523 696 0

523 1474 0

523 1006 0

523 1018 0

523 433 0

523 288 0

523 715 0

523 215 0

523 788 0

524 354 1

524 8 1

524 329 1

524 1807 1

524 336 1

524 337 1

524 1267 1

524 1493 1

524 1272 1

524 345 1

524 1951 1

524 3830 0

524 1713 0

524 1985 0

524 465 0

524 732 0

524 1757 0

524 1989 0

524 2283 0

524 2556 0

524 2579 0

524 500 0

525 106 1

525 511 1

525 878 1

525 111 1

525 16 1

525 48 1

525 1650 1

525 1556 1

525 53 1

525 282 1

525 159 1

525 2168 0

525 36 0

525 55 0

525 1214 0

525 1438 0

525 3380 0

525 1669 0

525 2607 0

525 2855 0

525 3589 0

525 314 0

526 1952 1

526 161 1

526 353 1

526 72 1

526 74 1

526 492 1

526 46 1

526 52 1

526 55 1

526 221 1

526 2356 0

526 2050 0

526 8 0

526 2169 0

526 772 0

526 2428 0

526 2126 0

526 3332 0

526 2965 0

526 978 0

527 66 1

527 133 1

527 1259 1

527 1231 1

527 80 1

527 467 1

527 87 1

527 830 1

527 281 0

527 3291 0

527 361 0

527 3386 0

527 2613 0

527 803 0

527 1545 0

527 3196 0

528 128 1

528 1953 1

528 1954 1

528 1955 1

528 1956 1

528 1957 1

528 70 1

528 807 1

528 1958 1

528 1624 1

528 590 1

528 80 1

528 790 1

528 376 1

528 1627 1

528 1629 1

528 2026 0

528 3454 0

528 874 0

528 3133 0

528 3237 0

528 3241 0

528 2749 0

528 682 0

528 2980 0

528 1843 0

528 3623 0

528 1509 0

528 1778 0

528 755 0

528 2706 0

528 3653 0

529 133 1

529 230 1

529 74 1

529 202 1

529 204 1

529 957 1

529 116 1

529 117 1

529 22 1

529 87 1

529 861 1

529 862 1

529 127 1

529 3491 0

529 3177 0

529 1116 0

529 1670 0

529 2983 0

529 3029 0

529 107 0

529 3378 0

529 1255 0

529 2651 0

529 2376 0

529 960 0

529 3499 0

530 132 1

530 1959 1

530 137 1

530 74 1

530 171 1

530 361 1

530 237 1

530 79 1

530 919 1

530 2232 0

530 822 0

530 2883 0

530 2057 0

530 3070 0

530 1875 0

530 2267 0

530 2584 0

530 2641 0

531 70 1

531 1960 1

531 1961 1

531 1706 1

531 1962 1

531 1963 1

531 1964 1

531 790 1

531 791 1

531 1624 1

531 602 1

531 795 1

531 1628 1

531 3759 0

531 2186 0

531 21 0

531 824 0

531 1362 0

531 1854 0

531 2501 0

531 1179 0

531 3299 0

531 2039 0

531 2457 0

531 2022 0

531 1970 0

532 96 1

532 560 1

532 1523 1

532 52 1

532 118 1

532 119 1

532 1829 0

532 1419 0

532 42 0

532 230 0

532 3442 0

532 192 0

533 133 1

533 358 1

533 1965 1

533 46 1

533 80 1

533 118 1

533 87 1

533 442 1

533 445 1

533 3468 0

533 2967 0

533 2445 0

533 3195 0

533 298 0

533 3358 0

533 925 0

533 65 0

533 3017 0

534 228 1

534 1966 1

534 143 1

534 1967 1

534 1968 1

534 1042 1

534 1139 1

534 1236 1

534 1969 1

534 1970 1

534 727 1

534 1496 1

534 25 1

534 474 1

534 1971 1

534 1972 1

534 223 1

534 2157 0

534 2206 0

534 2665 0

534 3658 0

534 3706 0

534 3775 0

534 2945 0

534 3394 0

534 85 0

534 321 0

534 2997 0

534 2956 0

534 540 0

534 697 0

534 2393 0

534 2851 0

534 282 0

535 227 1

535 36 1

535 459 1

535 275 1

535 1973 1

535 55 1

535 474 1

535 445 1

535 644 0

535 2642 0

535 2704 0

535 1599 0

535 3844 0

535 1046 0

535 2733 0

535 1717 0

536 376 1

536 713 1

536 266 1

536 714 1

536 301 1

536 78 1

536 79 1

536 47 1

536 494 1

536 1974 1

536 184 1

536 185 1

536 902 0

536 479 0

536 1875 0

536 2498 0

536 2344 0

536 767 0

536 236 0

536 755 0

536 3332 0

536 3318 0

536 544 0

536 2168 0

537 1123 1

537 325 1

537 7 1

537 1554 1

537 242 1

537 1975 1

537 1976 1

537 1977 1

537 1978 1

537 1979 1

537 1980 1

537 1882 0

537 3546 0

537 2738 0

537 3482 0

537 3117 0

537 63 0

537 2609 0

537 2518 0

537 3633 0

537 2369 0

537 3276 0

538 0 1

538 192 1

538 1984 1

538 1985 1

538 1986 1

538 998 1

538 1350 1

538 712 1

538 42 1

538 45 1

538 1583 1

538 208 1

538 210 1

538 1982 1

538 55 1

538 1981 1

538 1022 1

538 1983 1

538 3120 0

538 2475 0

538 2157 0

538 3797 0

538 3525 0

538 1388 0

538 1614 0

538 2533 0

538 1432 0

538 2656 0

538 1712 0

538 1723 0

538 1203 0

538 3622 0

538 701 0

538 2540 0

538 1045 0

538 144 0

539 66 1

539 1987 1

539 325 1

539 167 1

539 74 1

539 1771 1

539 52 1

539 1276 1

539 956 1

539 3762 0

539 2052 0

539 1505 0

539 3192 0

539 1062 0

539 405 0

539 2152 0

539 1115 0

539 44 0

540 199 1

540 398 1

540 208 1

540 1075 1

540 212 1

540 1076 1

540 215 1

540 218 1

540 220 1

540 1501 1

540 780 0

540 181 0

540 1484 0

540 2313 0

540 1225 0

540 1896 0

540 2152 0

540 936 0

540 2035 0

540 2493 0

541 802 1

541 35 1

541 1988 1

541 199 1

541 1031 1

541 210 1

541 1589 1

541 438 1

541 118 1

541 530 0

541 1386 0

541 545 0

541 609 0

541 1824 0

541 2712 0

541 658 0

541 280 0

541 158 0

542 1808 1

542 377 1

542 979 1

542 1989 1

542 2024 0

542 2933 0

542 951 0

542 1158 0

543 391 1

543 3047 1

543 41 1

543 522 1

543 107 1

543 268 1

543 45 1

543 46 1

543 554 1

543 1001 1

543 1203 1
543 52 1
543 692 1
543 1204 1
543 55 1
543 2394 1
543 675 0
543 1745 0
543 2837 0
543 679 0
543 1260 0
543 3478 0
543 542 0
543 3149 0
543 3744 0
543 3594 0
543 2455 0
543 558 0
543 1275 0
543 523 0
543 2754 0
543 946 0
544 771 1
544 1990 1
544 1991 1
544 1992 1
544 1993 1
544 12 1
544 242 1
544 1147 1

544 1603 0

544 2492 0

544 2862 0

544 1731 0

544 1614 0

544 2051 0

544 2667 0

544 1221 0

545 96 1

545 34 1

545 36 1

545 42 1

545 1994 1

545 1995 1

545 208 1

545 209 1

545 212 1

545 124 1

545 125 1

545 1662 1

545 3656 0

545 2134 0

545 458 0

545 2049 0

545 1453 0

545 2854 0

545 3107 0

545 878 0

545 2802 0

545 2897 0

545 2419 0
545 1650 0
546 1888 1
546 1186 1
546 202 1
546 171 1
546 363 1
546 364 1
546 206 1
546 79 1
546 365 1
546 560 1
546 115 1
546 376 1
546 185 1
546 2256 0
546 1008 0
546 2365 0
546 2934 0
546 3252 0
546 2009 0
546 716 0
546 1267 0
546 2374 0
546 2997 0
546 571 0
546 1718 0
546 3009 0
547 96 1
547 1153 1

547 130 1

547 1996 1

547 1997 1

547 206 1

547 118 1

547 87 1

547 2640 0

547 736 0

547 3771 0

547 495 0

547 506 0

547 282 0

547 3 0

547 303 0

548 7 1

548 237 1

548 1998 1

548 1999 1

548 80 1

548 176 1

548 727 1

548 2203 0

548 1334 0

548 979 0

548 3425 0

548 2647 0

548 1956 0

548 3287 0

549 1154 1

549 206 1

549 368 1

549 2000 1

549 2001 1

549 115 1

549 437 1

549 184 1

549 185 1

549 2568 0

549 2401 0

549 280 0

549 1829 0

549 1017 0

549 2953 0

549 629 0

549 2412 0

549 3840 0

550 96 1

550 36 1

550 199 1

550 40 1

550 776 1

550 302 1

550 47 1

550 304 1

550 494 1

550 2002 1

550 52 1

550 55 1

550 2562 0

550 1242 0

550 3083 0

550 3073 0

550 1800 0

550 2249 0

550 2473 0

550 2966 0

550 1631 0

550 235 0

550 1926 0

550 2656 0

551 36 1

551 325 1

551 346 1

551 241 1

551 242 1

551 243 1

551 245 1

551 250 1

551 347 1

551 350 1

551 3834 0

551 1497 0

551 424 0

551 2017 0

551 3524 0

551 1050 0

551 2805 0

551 3051 0

551 2183 0

551 1127 0

552 1794 1

552 676 1

552 933 1

552 329 1

552 1353 1

552 13 1

552 498 1

552 275 1

552 277 1

552 25 1

552 799 1

552 852 0

552 2866 0

552 1076 0

552 1009 0

552 1187 0

552 1474 0

552 1213 0

552 2268 0

552 2255 0

552 268 0

552 3056 0

553 544 1

553 73 1

553 557 1

553 78 1

553 80 1

553 87 1

553 472 1

553 185 1

553 1050 1
553 413 1
553 1278 1
553 447 0
553 2515 0
553 1343 0
553 3390 0
553 425 0
553 2367 0
553 117 0
553 2930 0
553 1336 0
553 1420 0
553 610 0
554 1312 1
554 2010 1
554 2011 1
554 459 1
554 1042 1
554 629 1
554 2006 1
554 2007 1
554 632 1
554 1721 1
554 442 1
554 2008 1
554 2009 1
554 2745 0
554 1391 0
554 290 0

554 2853 0

554 578 0

554 3644 0

554 492 0

554 91 0

554 192 0

554 1140 0

554 2601 0

554 497 0

554 2818 0

555 2016 1

555 2017 1

555 34 1

555 35 1

555 2018 1

555 613 1

555 102 1

555 2019 1

555 904 1

555 109 1

555 1389 1

555 657 1

555 826 1

555 2012 1

555 2013 1

555 2014 1

555 2015 1

555 3809 0

555 404 0

555 2501 0

555 352 0

555 2076 0

555 342 0

555 2666 0

555 1822 0

555 2721 0

555 2438 0

555 2609 0

555 2428 0

555 1591 0

555 3760 0

555 1739 0

555 435 0

555 1717 0

556 1443 1

556 388 1

556 2020 1

556 2021 1

556 2022 1

556 2023 1

556 528 1

556 1328 1

556 52 1

556 438 1

556 765 1

556 190 1

556 3275 0

556 2368 0

556 2334 0

556 1518 0

556 1343 0

556 412 0

556 1249 0

556 1022 0

556 2930 0

556 1612 0

556 1061 0

556 1760 0

557 192 1

557 66 1

557 72 1

557 2024 1

557 74 1

557 75 1

557 2025 1

557 87 1

557 151 1

557 1277 1

557 126 1

557 127 1

557 1451 0

557 839 0

557 2229 0

557 167 0

557 177 0

557 265 0

557 76 0

557 1442 0

557 2634 0

557 2106 0

557 895 0

557 1772 0

558 36 1

558 102 1

558 2026 1

558 651 1

558 2027 1

558 2028 1

558 46 1

558 2029 1

558 1713 1

558 52 1

558 1174 1

558 60 1

558 1540 0

558 3547 0

558 3544 0

558 782 0

558 2248 0

558 1941 0

558 890 0

558 1559 0

558 2665 0

558 1591 0

558 2010 0

558 3690 0

559 34 1

559 163 1

559 483 1

559 1347 1

559 42 1

559 109 1

559 2030 1

559 2031 1

559 20 1

559 472 1

559 57 1

559 220 1

559 2845 0

559 3651 0

559 3680 0

559 388 0

559 2783 0

559 1118 0

559 418 0

559 3542 0

559 958 0

559 2059 0

559 3385 0

559 2653 0

560 287 1

560 741 1

560 2032 1

560 2033 1

560 1722 1

560 767 1

560 1700 0

560 2001 0

560 860 0

560 1469 0

560 1753 0
560 1616 0
561 867 1
561 2034 1
561 2035 1
561 2036 1
561 2037 1
561 2038 1
561 2039 1
561 2040 1
561 2041 1
561 2042 1
561 2043 1
561 2044 1
561 191 1
561 1677 0
561 1711 0
561 3497 0
561 133 0
561 2566 0
561 2780 0
561 1307 0
561 195 0
561 798 0
561 96 0
561 2735 0
561 1790 0
561 2699 0
562 293 1
562 107 1

562 115 1

562 117 1

562 55 1

562 187 1

562 572 1

562 413 1

562 2053 0

562 852 0

562 1419 0

562 3386 0

562 3296 0

562 3553 0

562 3730 0

562 1871 0

563 257 1

563 131 1

563 22 1

563 413 1

563 293 1

563 295 1

563 1083 1

563 187 1

563 457 1

563 1234 1

563 1235 1

563 1237 1

563 361 1

563 107 1

563 112 1

563 1138 1

563 2045 1

563 2046 1

563 2047 1

563 3131 0

563 20 0

563 3243 0

563 3636 0

563 567 0

563 688 0

563 1534 0

563 1375 0

563 2338 0

563 942 0

563 1499 0

563 1000 0

563 2619 0

563 1338 0

563 396 0

563 3525 0

563 3580 0

563 582 0

563 875 0

564 353 1

564 131 1

564 132 1

564 230 1

564 1095 1

564 105 1

564 361 1

564 237 1

564 46 1

564 957 1

564 22 1

564 55 1

564 24 1

564 572 1

564 413 1

564 1406 1

564 1254 0

564 122 0

564 430 0

564 3589 0

564 3688 0

564 2389 0

564 1659 0

564 2804 0

564 3469 0

564 791 0

564 1602 0

564 1660 0

564 3362 0

564 1838 0

564 1066 0

564 3042 0

565 2048 1

565 45 1

565 495 1

565 55 1

565 154 1

565 2074 0

565 2138 0

565 936 0

565 700 0

565 3659 0

566 2049 1

566 776 1

566 1098 1

566 46 1

566 47 1

566 154 1

566 1587 0

566 1945 0

566 1809 0

566 1493 0

566 799 0

566 3686 0

567 2050 1

567 2051 1

567 740 1

567 325 1

567 394 1

567 756 1

567 216 1

567 217 1

567 3005 0

567 1241 0

567 2726 0

567 1001 0

567 1833 0

567 2905 0

567 645 0
567 1553 0
568 2052 1
568 2053 1
568 2054 1
568 135 1
568 1576 1
568 1931 1
568 1932 1
568 77 1
568 1232 1
568 81 1
568 1202 1
568 87 1
568 58 1
568 326 0
568 1910 0
568 3692 0
568 1203 0
568 984 0
568 1857 0
568 1419 0
568 1103 0
568 3008 0
568 2099 0
568 2514 0
568 140 0
568 3009 0
569 2055 1
569 2056 1

569 2057 1

569 396 1

569 1497 1

569 827 1

569 1358 0

569 2863 0

569 2924 0

569 3281 0

569 558 0

569 289 0

570 163 1

570 102 1

570 392 1

570 105 1

570 107 1

570 46 1

570 528 1

570 55 1

570 764 1

570 2952 0

570 1223 0

570 1446 0

570 2221 0

570 2805 0

570 535 0

570 2207 0

570 553 0

570 191 0

571 97 1

571 389 1

571 134 1

571 681 1

571 2058 1

571 47 1

571 118 1

571 857 1

571 250 1

571 126 1

571 316 0

571 2242 0

571 1477 0

571 882 0

571 1016 0

571 2623 0

571 2772 0

571 1364 0

571 1999 0

571 3221 0

572 96 1

572 353 1

572 544 1

572 99 1

572 714 1

572 75 1

572 2059 1

572 237 1

572 1278 1

572 118 1

572 87 1

572 184 1

572 1277 1
572 442 1
572 93 1
572 126 1
572 3183 0
572 16 0
572 3744 0
572 30 0
572 1867 0
572 1567 0
572 465 0
572 758 0
572 1075 0
572 961 0
572 2538 0
572 3428 0
572 2038 0
572 511 0
572 3709 0
572 1270 0
573 34 1
573 36 1
573 2060 1
573 109 1
573 111 1
573 959 1
573 155 1
573 156 1
573 221 1
573 159 1

573 3796 0

573 2356 0

573 2834 0

573 3055 0

573 1614 0

573 2758 0

573 734 0

573 257 0

573 2049 0

573 1984 0

574 34 1

574 867 1

574 36 1

574 42 1

574 362 1

574 109 1

574 2061 1

574 114 1

574 1362 1

574 1526 1

574 286 1

574 207 0

574 3845 0

574 2830 0

574 3185 0

574 685 0

574 381 0

574 2032 0

574 2649 0

574 1828 0

574 1277 0

574 3129 0

575 96 1

575 1312 1

575 36 1

575 329 1

575 45 1

575 2062 1

575 438 1

575 1046 1

575 1341 1

575 1211 0

575 2096 0

575 18 0

575 125 0

575 263 0

575 773 0

575 2135 0

575 539 0

575 916 0

576 516 1

576 1545 1

576 2063 1

576 2064 1

576 2065 1

576 2066 1

576 2067 1

576 1174 1

576 165 1

576 297 1

576 1343 1

576 196 1

576 708 1

576 1738 1

576 356 1

576 235 1

576 111 1

576 888 1

576 511 1

576 174 0

576 590 0

576 254 0

576 1853 0

576 1199 0

576 3362 0

576 2119 0

576 1193 0

576 564 0

576 1913 0

576 2421 0

576 700 0

576 1086 0

576 768 0

576 2684 0

576 3573 0

576 3100 0

576 3708 0

576 804 0

577 673 1

577 1346 1

577 1601 1
577 36 1
577 487 1
577 1402 1
577 243 1
577 250 1
577 61 1
577 2679 0
577 659 0
577 3642 0
577 3002 0
577 2753 0
577 3597 0
577 2545 0
577 2114 0
577 554 0
578 516 1
578 41 1
578 105 1
578 107 1
578 1545 1
578 109 1
578 46 1
578 528 1
578 1521 1
578 2068 1
578 821 1
578 24 1
578 3735 0
578 2900 0

578 3763	0

578 2138	0

578 697 0

578 1716	0

578 3351	0

578 1504	0

578 2196	0

578 3230	0

578 1917	0

578 2612	0

579 36	1

579 1253	1

579 105 1

579 523 1

579 652 1

579 109 1

579 46	1

579 141 1

579 53	1

579 918 1

579 55	1

579 2069	1

579 188 1

579 3493	0

579 2545	0

579 288 0

579 3698	0

579 1408	0

579 349 0

579 2625	0

579 2518 0
579 702 0
579 794 0
579 1317 0
579 3479 0
579 532 0
580 535 1
580 391 1
580 41 1
580 105 1
580 46 1
580 528 1
580 241 1
580 55 1
580 347 1
580 190 1
580 2032 0
580 1529 0
580 3134 0
580 2271 0
580 3635 0
580 2387 0
580 2761 0
580 2795 0
580 1204 0
580 2018 0
581 133 1
581 72 1
581 74 1
581 75 1

581 46 1

581 79 1

581 863 1

581 87 1

581 184 1

581 956 1

581 254 1

581 127 1

581 1479 0

581 2300 0

581 561 0

581 801 0

581 3303 0

581 2065 0

581 1990 0

581 3465 0

581 2481 0

581 1573 0

581 3330 0

581 3363 0

582 96 1

582 1122 1

582 2074 1

582 105 1

582 1322 1

582 47 1

582 188 1

582 210 1

582 52 1

582 212 1

582 917 1

582 1523 1

582 2072 1

582 2073 1

582 826 1

582 124 1

582 640 0

582 1623 0

582 383 0

582 2477 0

582 3139 0

582 2579 0

582 2705 0

582 2471 0

582 365 0

582 3805 0

582 1352 0

582 309 0

582 568 0

582 2364 0

582 40 0

582 2878 0

583 41 1

583 75 1

583 377 1

583 2075 1

583 2076 1

583 2077 1

583 3248 0

583 1091 0

583	1024	0
583	1413	0
583	1981	0
583	3750	0
584	1536	1
584	2080	1
584	2081	1
584	1923	1
584	1349	1
584	774	1
584	777	1
584	110	1
584	399	1
584	46	1
584	278	1
584	57	1
584	2078	1
584	2079	1
584	1511	0
584	554	0
584	1940	0
584	1330	0
584	222	0
584	2041	0
584	3368	0
584	1720	0
584	3091	0
584	79	0
584	1292	0
584	3352	0

584 3386 0

584 2761 0

585 66 1

585 674 1

585 2082 1

585 2083 1

585 2084 1

585 2085 1

585 72 1

585 74 1

585 238 1

585 1206 1

585 87 1

585 378 1

585 2885 0

585 2985 0

585 3400 0

585 1111 0

585 3560 0

585 2989 0

585 3185 0

585 2230 0

585 2997 0

585 1699 0

585 798 0

585 1476 0

586 2086 1

586 1767 1

586 2087 1

586 754 1

586 55 1

586 223 1

586 3109 0

586 1549 0

586 3759 0

586 3646 0

586 1121 0

586 1169 0

587 133 1

587 135 1

587 72 1

587 78 1

587 79 1

587 87 1

587 1501 0

587 3626 0

587 3243 0

587 1781 0

587 3353 0

587 432 0

588 192 1

588 129 1

588 98 1

588 353 1

588 261 1

588 358 1

588 263 1

588 2088 1

588 2089 1

588 2090 1

588 80 1

588 24 1

588 1082 1

588 253 1

588 1310 1

588 1473 0

588 1272 0

588 1997 0

588 782 0

588 728 0

588 3329 0

588 3039 0

588 28 0

588 2562 0

588 20 0

588 3187 0

588 985 0

588 3744 0

588 420 0

588 1265 0

589 36 1

589 548 1

589 362 1

589 141 1

589 52 1

589 788 1

589 949 1

589 1140 1

589 188 1

589 2904 0

589 3086 0

589 254 0

589 1358 0

589 216 0

589 3153 0

589 2459 0

589 849 0

589 3758 0

590 650 1

590 267 1

590 916 1

590 1694 1

590 2091 1

590 2092 1

590 2093 1

590 47 1

590 1457 1

590 1589 1

590 694 1

590 55 1

590 184 1

590 575 1

590 78 1

590 96 1

590 352 1

590 1002 1

590 239 1

590 1278 1

590 2716 0

590 2068 0

590 2960 0
590 2952 0
590 1903 0
590 2419 0
590 823 0
590 1512 0
590 1335 0
590 2369 0
590 533 0
590 3133 0
590 1508 0
590 3601 0
590 1414 0
590 2725 0
590 3206 0
590 2935 0
590 2751 0
590 243 0
591 66 1
591 1987 1
591 774 1
591 2094 1
591 2095 1
591 144 1
591 145 1
591 146 1
591 147 1
591 785 1
591 2096 1
591 2097 1

591 1719 1

591 2098 1

591 2099 1

591 191 1

591 2503 0

591 1016 0

591 1951 0

591 285 0

591 3446 0

591 640 0

591 226 0

591 2073 0

591 1186 0

591 1054 0

591 910 0

591 1616 0

591 1179 0

591 625 0

591 2271 0

591 1293 0

592 2051 1

592 1476 1

592 390 1

592 1542 1

592 297 1

592 109 1

592 110 1

592 141 1

592 882 1

592 2100 1

592 1174 1
592 1047 1
592 568 1
592 2070 1
592 957 1
592 2583 0
592 972 0
592 2969 0
592 737 0
592 414 0
592 60 0
592 1505 0
592 578 0
592 2927 0
592 126 0
592 3299 0
592 2470 0
592 3504 0
592 36 0
592 3547 0
593 1345 1
593 34 1
593 36 1
593 133 1
593 165 1
593 469 1
593 1973 1
593 2101 1
593 1048 1
593 2102 1

593 2103 1
593 1051 1
593 286 1
593 3592 0
593 2377 0
593 1708 0
593 2902 0
593 1304 0
593 858 0
593 1932 0
593 2406 0
593 576 0
593 3590 0
593 2073 0
593 2003 0
593 3404 0
594 1952 1
594 2112 1
594 66 1
594 547 1
594 548 1
594 551 1
594 2110 1
594 2107 1
594 145 1
594 1461 1
594 2104 1
594 2105 1
594 2106 1
594 1435 1

594 2108 1

594 2109 1

594 478 1

594 2111 1

594 2637 0

594 1196 0

594 2055 0

594 1532 0

594 996 0

594 3785 0

594 1534 0

594 443 0

594 1104 0

594 2472 0

594 703 0

594 618 0

594 388 0

594 1620 0

594 842 0

594 3504 0

594 3527 0

594 572 0

595 96 1

595 376 1

595 133 1

595 267 1

595 206 1

595 79 1

595 80 1

595 115 1

595 374 1

595 184 1

595 185 1

595 2105 0

595 3069 0

595 3276 0

595 787 0

595 1812 0

595 1360 0

595 3287 0

595 2825 0

595 3312 0

595 1688 0

595 2135 0

596 1088 1

596 2113 1

596 35 1

596 732 1

596 165 1

596 617 1

596 1545 1

596 460 1

596 109 1

596 883 1

596 22 1

596 252 1

596 158 1

596 3605 0

596 1726 0

596 2945 0

596 1020 0

596 2745 0

596 33 0

596 3138 0

596 3595 0

596 3324 0

596 3170 0

596 3624 0

596 3522 0

596 3469 0

597 96 1

597 2114 1

597 803 1

597 1123 1

597 1290 1

597 209 1

597 210 1

597 211 1

597 52 1

597 756 1

597 535 1

597 217 1

597 250 1

597 2094 0

597 2341 0

597 1405 0

597 734 0

597 3213 0

597 2069 0

597 1559 0

597 1048 0
597 2120 0
597 967 0
597 1279 0
597 2332 0
597 955 0
598 96 1
598 128 1
598 130 1
598 2115 1
598 2116 1
598 77 1
598 301 1
598 118 1
598 55 1
598 442 1
598 445 1
598 190 1
598 2251 0
598 1079 0
598 1591 0
598 1309 0
598 2838 0
598 2674 0
598 1008 0
598 1420 0
598 1138 0
598 3536 0
598 177 0
598 1273 0

599 2117	1

599 74	1

599 1084	1

599 24	1

599 1818	1

599 956 1

599 957 1

599 254 1

599 127 1

599 1838	0

599 3249	0

599 2351	0

599 3676	0

599 2533	0

599 400 0

599 158 0

599 462 0

599 2792	0

600 96	1

600 2118	1

600 2119	1

600 2120	1

600 2121	1

600 2122	1

600 307 1

600 439 1

600 286 1

600 2434	0

600 480 0

600 508 0

600 1728 0

600 2351 0

600 2043 0

600 71 0

600 601 0

600 2400 0

601 673 1

601 36 1

601 42 1

601 362 1

601 2124 1

601 2125 1

601 2126 1

601 2127 1

601 442 1

601 2128 1

601 2129 1

601 438 1

601 151 1

601 472 1

601 126 1

601 1276 1

601 286 1

601 3654 0

601 888 0

601 3650 0

601 168 0

601 2652 0

601 876 0

601 3438 0

601 2544 0

601 1957 0

601 2192 0

601 167 0

601 2337 0

601 3083 0

601 1946 0

601 3038 0

601 2523 0

601 538 0

602 133 1

602 74 1

602 1202 1

602 2130 1

602 2131 1

602 2132 1

602 87 1

602 127 1

602 3430 0

602 3718 0

602 319 0

602 2208 0

602 2713 0

602 1829 0

602 2388 0

602 852 0

603 956 1

603 2133 1

603 271 1

603 2882 0

603 1322 0

603 970 0

604 96 1

604 577 1

604 2090 1

604 107 1

604 46 1

604 1551 1

604 659 1

604 118 1

604 438 1

604 566 1

604 761 1

604 475 1

604 254 1

604 976 0

604 3136 0

604 2877 0

604 3745 0

604 2415 0

604 2767 0

604 1720 0

604 2034 0

604 3410 0

604 267 0

604 1192 0

604 393 0

604 548 0

605 132 1

605 295 1

605 361 1

605 457 1

605 1328 1

605 1138 1

605 1079 1

605 22 1

605 1078 1

605 24 1

605 2134 1

605 1080 1

605 571 1

605 2135 1

605 445 1

605 1950 1

605 3255 0

605 1036 0

605 2946 0

605 2751 0

605 2498 0

605 39 0

605 3173 0

605 1415 0

605 2382 0

605 1290 0

605 2873 0

605 2900 0

605 3258 0

605 1897 0

605 2672 0

605 746 0

606 135 1

606 202 1

606 75 1

606 171 1

606 237 1

606 46 1

606 1583 1

606 528 1

606 656 1

606 115 1

606 116 1

606 118 1

606 87 1

606 377 1

606 1285 0

606 3312 0

606 2387 0

606 2249 0

606 1065 0

606 709 0

606 3186 0

606 11 0

606 504 0

606 644 0

606 1496 0

606 2775 0

606 1552 0

606 3813 0

607 1156 1

607 2084 1

607 269 1

607 692 1

607 2071 1

607 1654 1

607 1751 1

607 2136 1

607 2137 1

607 2138 1

607 2139 1

607 2140 1

607 2141 1

607 2453 0

607 571 0

607 1695 0

607 2846 0

607 192 0

607 1782 0

607 1821 0

607 588 0

607 142 0

607 971 0

607 2135 0

607 2434 0

607 3193 0

608 2144 1

608 1761 1

608 2145 1

608 2146 1

608 634 1

608 2011 1

608 2142 1
608 2143 1
608 1142 0
608 2266 0
608 2426 0
608 2039 0
608 1219 0
608 3592 0
608 3316 0
608 3639 0
609 1122 1
609 2147 1
609 1188 1
609 37 1
609 2148 1
609 1899 1
609 1621 1
609 1018 1
609 91 1
609 119 0
609 1685 0
609 2732 0
609 3594 0
609 2365 0
609 3208 0
609 762 0
609 3124 0
609 2404 0
610 2149 1
610 245 1

610 126 1

610 3528 0

610 3668 0

610 1809 0

611 1088 1

611 280 1

611 1248 1

611 165 1

611 453 1

611 2150 1

611 2151 1

611 2152 1

611 2153 1

611 111 1

611 1650 1

611 1396 1

611 56 1

611 1497 1

611 159 1

611 944 0

611 3266 0

611 2678 0

611 919 0

611 131 0

611 170 0

611 1136 0

611 1581 0

611 1628 0

611 1113 0

611 2783 0

611 3809 0

611 3204 0

611 2172 0

611 3841 0

612 354 1

612 1347 1

612 356 1

612 357 1

612 105 1

612 2154 1

612 2155 1

612 109 1

612 275 1

612 445 1

612 574 1

612 3011 0

612 3328 0

612 198 0

612 1882 0

612 2232 0

612 3834 0

612 2905 0

612 1361 0

612 3374 0

612 3318 0

612 2026 0

613 224 1

613 1315 1

613 1444 1

613 934 1

613 2156 1

613 2157 1

613 2158 1

613 883 1

613 1493 1

613 314 1

613 128 0

613 3718 0

613 178 0

613 2595 0

613 118 0

613 2890 0

613 225 0

613 635 0

613 1710 0

613 1014 0

614 1889 1

614 130 1

614 133 1

614 78 1

614 79 1

614 80 1

614 87 1

614 127 1

614 2785 0

614 1436 0

614 3297 0

614 3780 0

614 1051 0

614 3292 0

614 1694 0

614 506 0

615 1185 1

615 1253 1

615 102 1

615 560 1

615 243 1

615 52 1

615 1523 1

615 250 1

615 188 1

615 543 1

615 2820 0

615 1214 0

615 3457 0

615 3024 0

615 2777 0

615 3206 0

615 2203 0

615 2665 0

615 363 0

615 987 0

616 923 1

616 134 1

616 79 1

616 560 1

616 830 1

616 2159 1

616 2160 1

616 118 1

616 919 1

616 859 1

616 254 1

616 835 0

616 3080 0

616 3285 0

616 1162 0

616 2369 0

616 3176 0

616 3775 0

616 2289 0

616 3387 0

616 1112 0

616 3204 0

617 382 1

617 132 1

617 708 1

617 295 1

617 361 1

617 74 1

617 1818 1

617 525 1

617 46 1

617 1393 1

617 2161 1

617 1308 1

617 55 1

617 570 1

617 507 1

617 572 1

617 190 1

617 2048 0

617 2796 0

617 2441 0

617 291 0

617 2204 0

617 24 0

617 2352 0

617 1067 0

617 1057 0

617 875 0

617 3659 0

617 1836 0

617 2688 0

617 3487 0

617 3736 0

617 3258 0

617 1659 0

618 1185 1

618 1186 1

618 99 1

618 133 1

618 1736 1

618 171 1

618 560 1

618 118 1

618 3449 0

618 2915 0

618 26 0

618 3144 0

618 1121 0

618 3314 0

618 726 0

618 1026 0

619 770 1

619 802 1

619 102 1

619 358 1

619 713 1

619 1836 1

619 589 1

619 302 1

619 303 1

619 210 1

619 52 1

619 280 1

619 1593 1

619 1501 1

619 2708 0

619 3075 0

619 3174 0

619 2225 0

619 291 0

619 3732 0

619 3687 0

619 489 0

619 2043 0

619 374 0

619 2674 0

619 1087 0

619 3375 0

619 532 0

620 102 1

620 554 1

620 399 1

620 1392 1

620 2162 1

620 2163 1

620 2164 1

620 2074 1

620 1312 0

620 3403 0

620 3251 0

620 2711 0

620 3144 0

620 3269 0

620 1523 0

620 3358 0

621 98 1

621 131 1

621 1414 1

621 135 1

621 1258 1

621 1200 1

621 374 1

621 758 1

621 185 1

621 538 1

621 254 1

621 127 1

621 1993 0
621 842 0
621 372 0
621 2059 0
621 612 0
621 2314 0
621 1980 0
621 2763 0
621 1587 0
621 271 0
621 136 0
621 1335 0
622 131 1
622 132 1
622 261 1
622 230 1
622 72 1
622 104 1
622 137 1
622 1818 1
622 237 1
622 87 1
622 698 1
622 187 1
622 1441 0
622 1217 0
622 2297 0
622 145 0
622 1657 0
622 2923 0

622 1594 0

622 3362 0

622 576 0

622 1233 0

622 752 0

622 1473 0

623 325 1

623 395 1

623 109 1

623 654 1

623 306 1

623 2165 1

623 2166 1

623 3359 0

623 1745 0

623 1152 0

623 962 0

623 3431 0

623 218 0

623 3113 0

624 289 1

624 740 1

624 325 1

624 1146 1

624 2170 1

624 393 1

624 74 1

624 395 1

624 1641 1

624 1906 1

624	2167	1
624	2168	1
624	2169	1
624	250	1
624	2171	1
624	2172	1
624	3478	0
624	2066	0
624	1512	0
624	2776	0
624	1828	0
624	3325	0
624	2702	0
624	3589	0
624	3720	0
624	972	0
624	929	0
624	2466	0
624	874	0
624	3105	0
624	2588	0
624	2161	0
625	102	1
625	777	1
625	1263	1
625	52	1
625	188	1
625	2173	1
625	63	1
625	1328	0

625	913	0
625	469	0
625	2961	0
625	576	0
625	2463	0
625	3636	0
626	385	1
626	98	1
626	130	1
626	132	1
626	74	1
626	203	1
626	1258	1
626	79	1
626	1201	1
626	2071	1
626	117	1
626	87	1
626	2174	1
626	127	1
626	2458	0
626	2107	0
626	1499	0
626	3366	0
626	1962	0
626	2659	0
626	3140	0
626	2636	0
626	1635	0
626	315	0

626 1137 0

626 3551 0

626 1400 0

626 833 0

627 96 1

627 2176 1

627 185 1

627 1820 1

627 263 1

627 237 1

627 206 1

627 79 1

627 80 1

627 89 1

627 1115 1

627 1244 1

627 477 1

627 2175 1

627 2741 0

627 196 0

627 498 0

627 390 0

627 3660 0

627 3156 0

627 3219 0

627 2319 0

627 2478 0

627 1670 0

627 3366 0

627 3325 0

627 949 0

627 147 0

628 865 1

628 2 1

628 2177 1

628 199 1

628 39 1

628 776 1

628 1098 1

628 47 1

628 118 1

628 764 1

628 477 1

628 1503 1

628 1132 0

628 1559 0

628 3319 0

628 2312 0

628 3300 0

628 623 0

628 2862 0

628 1361 0

628 2352 0

628 443 0

628 3175 0

628 2606 0

629 130 1

629 133 1

629 363 1

629 78 1

629 206 1

629 80 1

629 115 1

629 118 1

629 374 1

629 184 1

629 185 1

629 699 1

629 63 1

629 1403 0

629 3567 0

629 3208 0

629 2501 0

629 478 0

629 3456 0

629 2819 0

629 3106 0

629 1212 0

629 1810 0

629 656 0

629 2988 0

629 2469 0

630 257 1

630 1057 1

630 1794 1

630 1476 1

630 933 1

630 2178 1

630 2179 1

630 2180 1

630 2181 1

630 497 1

630 498 1

630 499 1

630 500 1

630 277 1

630 883 1

630 1495 1

630 26 1

630 2868 0

630 8 0

630 1273 0

630 46 0

630 3509 0

630 3018 0

630 2378 0

630 2397 0

630 1836 0

630 3578 0

630 2725 0

630 544 0

630 3200 0

630 1755 0

630 316 0

630 915 0

630 2894 0

631 2182 1

631 135 1

631 72 1

631 2183 1

631 1258 1

631 79 1

631 80 1

631 81 1

631 919 1

631 87 1

631 185 1

631 538 1

631 254 1

631 127 1

631 2178 0

631 1196 0

631 3492 0

631 1581 0

631 2130 0

631 1463 0

631 1684 0

631 1915 0

631 3675 0

631 2986 0

631 3252 0

631 3739 0

631 1327 0

631 2888 0

632 70 1

632 871 1

632 1446 1

632 42 1

632 141 1

632 47 1

632 52 1

632 695 1

632 2107 0

632 2015 0

632 3358 0

632 2819 0

632 2169 0

632 2618 0

632 1986 0

632 2283 0

633 257 1

633 227 1

633 4 1

633 37 1

633 293 1

633 2184 1

633 329 1

633 118 1

633 22 1

633 28 1

633 445 1

633 343 1

633 693 0

633 2111 0

633 2967 0

633 3738 0

633 3264 0

633 2551 0

633 97 0

633 1805 0

633 2476 0

633 2988 0

633 1503 0

633 1109 0

634 224 1

634 388 1

634 933 1

634 934 1

634 937 1

634 2185 1

634 686 1

634 498 1

634 882 1

634 308 1

634 277 1

634 2259 0

634 181 0

634 2198 0

634 1081 0

634 1802 0

634 2903 0

634 1313 0

634 0 0

634 3115 0

634 2847 0

634 604 0

635 32 1

635 96 1

635 102 1

635 41 1

635	105	1
635	1259	1
635	46	1
635	47	1
635	52	1
635	87	1
635	55	1
635	184	1
635	91	1
635	1318	0
635	3287	0
635	3304	0
635	1143	0
635	2580	0
635	1929	0
635	2256	0
635	851	0
635	3040	0
635	3602	0
635	501	0
635	771	0
635	763	0
636	130	1
636	547	1
636	134	1
636	135	1
636	75	1
636	77	1
636	79	1
636	80	1

636 118 1

636 374 1

636 1275 1

636 2244 0

636 1997 0

636 3131 0

636 1500 0

636 753 0

636 2777 0

636 495 0

636 1503 0

636 33 0

636 2897 0

636 723 0

637 132 1

637 1412 1

637 135 1

637 74 1

637 202 1

637 237 1

637 22 1

637 185 1

637 127 1

637 1000 0

637 1143 0

637 3506 0

637 683 0

637 1373 0

637 2625 0

637 1006 0

637 3482 0

637 2346 0

638 192 1

638 137 1

638 2186 1

638 45 1

638 1459 1

638 22 1

638 55 1

638 24 1

638 1114 1

638 187 1

638 3778 0

638 3344 0

638 2937 0

638 2107 0

638 1446 0

638 117 0

638 1088 0

638 3042 0

638 1435 0

638 3796 0

639 897 1

639 257 1

639 258 1

639 2187 1

639 2188 1

639 2189 1

639 2190 1

639 2096 1

639 2034 1

639 275 1

639 169 0

639 1760 0

639 719 0

639 2435 0

639 2393 0

639 3637 0

639 280 0

639 3142 0

639 1307 0

639 2698 0

640 131 1

640 1092 1

640 102 1

640 230 1

640 106 1

640 235 1

640 2191 1

640 2192 1

640 2193 1

640 882 1

640 1587 1

640 53 1

640 54 1

640 55 1

640 1174 1

640 607 1

640 3268 0

640 806 0

640 99 0

640 2657 0

640 1778 0

640 301 0

640 840 0

640 2590 0

640 2259 0

640 3273 0

640 1070 0

640 3256 0

640 2015 0

640 1834 0

640 1437 0

640 494 0

641 2120 1

641 332 1

641 13 1

641 2194 1

641 1235 1

641 2195 1

641 2196 1

641 2197 1

641 799 1

641 2877 0

641 2970 0

641 3562 0

641 832 0

641 626 0

641 1523 0

641 1474 0

641	2273	0
641	1717	0
642	481	1
642	354	1
642	35	1
642	36	1
642	37	1
642	165	1
642	196	1
642	198	1
642	805	1
642	1330	1
642	2198	1
642	2199	1
642	221	1
642	158	1
642	703	1
642	3679	0
642	3094	0
642	412	0
642	2624	0
642	2228	0
642	1017	0
642	2729	0
642	864	0
642	1862	0
642	1451	0
642	881	0
642	2043	0
642	1876	0

642 1777 0

642 2889 0

643 755 1

643 52 1

643 1303 1

643 191 1

643 2393 0

643 3307 0

643 3493 0

643 3783 0

644 96 1

644 1374 1

644 418 1

644 195 1

644 196 1

644 38 1

644 200 1

644 1036 1

644 2031 1

644 20 1

644 21 1

644 24 1

644 732 1

644 1310 1

644 1003 0

644 97 0

644 1545 0

644 2273 0

644 3723 0

644 2872 0

644 3469 0

644 23 0

644 976 0

644 2543 0

644 1936 0

644 883 0

644 1345 0

644 3631 0

645 96 1

645 66 1

645 548 1

645 41 1

645 459 1

645 1516 1

645 117 1

645 254 1

645 2200 1

645 2201 1

645 442 1

645 1275 1

645 126 1

645 3403 0

645 2135 0

645 2030 0

645 3379 0

645 3169 0

645 2589 0

645 3145 0

645 292 0

645 3128 0

645 334 0

645 680 0

645 2683 0

645 373 0

646 257 1

646 275 1

646 788 1

646 501 1

646 499 1

646 3273 0

646 1947 0

646 1120 0

646 3314 0

646 1483 0

647 96 1

647 128 1

647 130 1

647 133 1

647 78 1

647 79 1

647 80 1

647 55 1

647 87 1

647 376 1

647 2266 0

647 688 0

647 1046 0

647 1952 0

647 855 0

647 2682 0

647 1729 0

647 932 0

647 100 0

647 3204 0

648 74 1

648 77 1

648 79 1

648 80 1

648 81 1

648 115 1

648 87 1

648 2202 1

648 127 1

648 845 0

648 1753 0

648 787 0

648 1169 0

648 2456 0

648 3784 0

648 1179 0

648 1256 0

648 2781 0

649 1185 1

649 135 1

649 80 1

649 374 1

649 87 1

649 376 1

649 698 1

649 61 1

649 2701 0

649 1215 0

649 2219 0

649 233 0

649 217 0

649 586 0

649 255 0

649 3117 0

650 128 1

650 2207 1

650 2208 1

650 1956 1

650 70 1

650 1624 1

650 790 1

650 1623 1

650 376 1

650 2203 1

650 2204 1

650 2205 1

650 2206 1

650 1599 1

650 1916 0

650 220 0

650 2116 0

650 3311 0

650 537 0

650 1117 0

650 2614 0

650 526 0

650 634 0

650 2595 0

650 391 0

650 664 0

650 264 0

650 3771 0

651 96 1

651 673 1

651 2209 1

651 2210 1

651 2211 1

651 681 1

651 557 1

651 1997 1

651 239 1

651 1583 1

651 305 1

651 87 1

651 1240 1

651 377 1

651 442 1

651 1277 1

651 1278 1

651 313 0

651 2492 0

651 1183 0

651 1725 0

651 2918 0

651 848 0

651 3256 0

651 3 0
651 532 0
651 1802 0
651 2876 0
651 1523 0
651 2395 0
651 3570 0
651 923 0
651 3218 0
651 3800 0
652 2212 1
652 325 1
652 2213 1
652 7 1
652 47 1
652 655 1
652 756 1
652 118 1
652 250 1
652 166 0
652 2071 0
652 230 0
652 3233 0
652 1741 0
652 2414 0
652 664 0
652 3542 0
652 3310 0
653 832 1
653 1890 1

653 2214 1

653 2215 1

653 2216 1

653 2217 1

653 377 1

653 191 1

653 2996 0

653 86 0

653 2299 0

653 2059 0

653 2612 0

653 3527 0

653 2282 0

653 2283 0

654 964 1

654 1094 1

654 1802 1

654 2218 1

654 2219 1

654 941 1

654 2220 1

654 911 1

654 306 1

654 315 1

654 959 1

654 2469 0

654 1340 0

654 1849 0

654 1454 0

654 1330 0

654 3716 0
654 446 0
654 2750 0
654 2531 0
654 164 0
654 2612 0
655 2221 1
655 2222 1
655 2559 0
655 1594 0
656 941 1
656 109 1
656 882 1
656 277 1
656 2197 1
656 2199 1
656 799 1
656 3393 0
656 2741 0
656 959 0
656 2218 0
656 2025 0
656 1452 0
656 3389 0
657 96 1
657 834 1
657 36 1
657 871 1
657 788 1
657 119 1

657 347 1

657 2355 0

657 2722 0

657 2229 0

657 1620 0

657 3706 0

657 2647 0

657 2787 0

658 391 1

658 332 1

658 45 1

658 2223 1

658 2224 1

658 2225 1

658 50 1

658 2226 1

658 2227 1

658 413 1

658 568 1

658 221 1

658 190 1

658 1915 0

658 2797 0

658 2551 0

658 1939 0

658 3415 0

658 250 0

658 197 0

658 2123 0

658 167 0

658 1810	0

658 1311	0

658 3543	0

658 580 0

659 135 1

659 74	1

659 81	1

659 115 1

659 2228	1

659 117 1

659 2229	1

659 87	1

659 127 1

659 2112	0

659 210 0

659 882 0

659 3367	0

659 3376	0

659 1562	0

659 3100	0

659 2078	0

659 2004	0

660 257 1

660 16	1

660 277 1

660 1691	1

660 799 1

660 934 1

660 1455	1

660 2230	1

660 1083 1

660 1091 1

660 1999 1

660 1235 1

660 1495 1

660 474 1

660 858 1

660 860 1

660 498 1

660 500 1

660 1020 1

660 878 0

660 2050 0

660 1596 0

660 630 0

660 1713 0

660 403 0

660 1901 0

660 3295 0

660 814 0

660 3777 0

660 1784 0

660 2773 0

660 2827 0

660 650 0

660 3238 0

660 3797 0

660 673 0

660 1339 0

660 2804 0

661 36 1

661 805 1

661 7 1

661 1161 1

661 1166 1

661 241 1

661 2231 1

661 2232 1

661 250 1

661 347 1

661 2710 0

661 3134 0

661 2858 0

661 1307 0

661 1067 0

661 2212 0

661 1927 0

661 1151 0

661 1177 0

661 2268 0

662 96 1

662 802 1

662 102 1

662 1031 1

662 776 1

662 714 1

662 47 1

662 208 1

662 210 1

662 212 1

662 118 1

662 2233 1

662 2015 1

662 3461 0

662 2600 0

662 584 0

662 3424 0

662 2430 0

662 2100 0

662 1281 0

662 182 0

662 3402 0

662 925 0

662 2731 0

662 1197 0

662 2708 0

663 96 1

663 134 1

663 74 1

663 75 1

663 237 1

663 79 1

663 399 1

663 373 1

663 87 1

663 254 1

663 127 1

663 1645 0

663 236 0

663 888 0

663 2292 0

663 3693 0

663 66 0

663 3753 0

663 1786 0

663 2311 0

663 2586 0

663 3396 0

664 231 1

664 295 1

664 519 1

664 617 1

664 860 1

664 460 1

664 46 1

664 465 1

664 1969 1

664 1235 1

664 22 1

664 24 1

664 57 1

664 858 1

664 155 1

664 156 1

664 863 1

664 3027 0

664 220 0

664 730 0

664 2286 0

664 1909 0

664	2834	0
664	3370	0
664	3194	0
664	2380	0
664	1710	0
664	832	0
664	422	0
664	3510	0
664	3519	0
664	1808	0
664	97	0
664	1767	0
665	1249	1
665	356	1
665	36	1
665	1453	1
665	2234	1
665	1067	0
665	3691	0
665	1825	0
665	1559	0
665	1457	0
666	608	1
666	289	1
666	35	1
666	36	1
666	42	1
666	250	1
666	191	1
666	2052	0

666 1123 0

666 644 0

666 1458 0

666 1019 0

666 3081 0

666 1984 0

667 453 1

667 106 1

667 511 1

667 109 1

667 110 1

667 719 1

667 48 1

667 878 1

667 52 1

667 53 1

667 2235 1

667 2236 1

667 159 1

667 3550 0

667 444 0

667 2956 0

667 2734 0

667 3122 0

667 2122 0

667 3113 0

667 2425 0

667 93 0

667 487 0

667 2694 0

667 1784 0

667 3602 0

668 98 1

668 74 1

668 78 1

668 79 1

668 52 1

668 87 1

668 254 1

668 127 1

668 3008 0

668 3098 0

668 996 0

668 2022 0

668 519 0

668 812 0

668 1802 0

668 395 0

669 130 1

669 1218 1

669 41 1

669 74 1

669 76 1

669 78 1

669 80 1

669 81 1

669 87 1

669 760 1

669 377 1

669 330 0

669 649 0

669 3101 0

669 293 0

669 305 0

669 2525 0

669 436 0

669 1329 0

669 3059 0

669 2856 0

669 3778 0

670 2240 1

670 2239 1

670 2241 1

670 2242 1

670 100 1

670 36 1

670 681 1

670 2187 1

670 275 1

670 1046 1

670 151 1

670 442 1

670 2237 1

670 2238 1

670 1311 1

670 3443 0

670 1377 0

670 671 0

670 3592 0

670 1033 0

670 2100 0
670 3688 0
670 3534 0
670 1894 0
670 827 0
670 2639 0
670 3250 0
670 835 0
670 1612 0
670 3584 0
671 320 1
671 72 1
671 1199 1
671 1073 1
671 956 1
671 1502 0
671 2301 0
671 808 0
671 530 0
671 2367 0
672 995 1
672 2243 1
672 2244 1
672 2245 1
672 2246 1
672 2247 1
672 1193 1
672 2248 1
672 2249 1
672 2250 1

672 2251 1
672 846 1
672 309 1
672 60 1
672 3744 0
672 2661 0
672 3252 0
672 1731 0
672 1512 0
672 1544 0
672 522 0
672 3510 0
672 713 0
672 1202 0
672 79 0
672 2858 0
672 1566 0
672 1049 0
673 1088 1
673 1664 1
673 1091 1
673 388 1
673 293 1
673 297 1
673 1738 1
673 2252 1
673 109 1
673 46 1
673 527 1
673 2253 1

673 2254 1

673 498 1

673 1078 1

673 474 1

673 1182 1

673 1240 0

673 2600 0

673 40 0

673 836 0

673 631 0

673 779 0

673 3579 0

673 1145 0

673 787 0

673 2513 0

673 1129 0

673 2267 0

673 575 0

673 996 0

673 3474 0

673 342 0

673 2915 0

674 545 1

674 8 1

674 329 1

674 332 1

674 13 1

674 333 1

674 2255 1

674 2256 1

674 2257 1

674 19 1

674 1268 1

674 55 1

674 857 1

674 441 1

674 378 1

674 895 1

674 2899 0

674 1307 0

674 495 0

674 3274 0

674 2785 0

674 580 0

674 2218 0

674 242 0

674 3735 0

674 132 0

674 3133 0

674 1333 0

674 1633 0

674 1449 0

674 2628 0

674 3590 0

675 36 1

675 74 1

675 47 1

675 1105 1

675 694 1

675 89 1

675 1812 0

675 1361 0

675 2832 0

675 95 0

675 3696 0

675 2305 0

676 34 1

676 36 1

676 133 1

676 42 1

676 653 1

676 655 1

676 209 1

676 242 1

676 756 1

676 442 1

676 0 0

676 1509 0

676 1487 0

676 1558 0

676 180 0

676 2377 0

676 3505 0

676 248 0

676 626 0

676 445 0

677 66 1

677 167 1

677 956 1

677 974 1

677 957 1

677 1073 1

677 2258 1

677 2259 1

677 895 1

677 2260 1

677 118 1

677 183 1

677 252 1

677 861 1

677 254 1

677 127 1

677 1860 0

677 2132 0

677 2764 0

677 2194 0

677 2744 0

677 2479 0

677 1344 0

677 2224 0

677 166 0

677 2550 0

677 2725 0

677 500 0

677 870 0

677 2461 0

677 2322 0

677 2378 0

678 267 1

678 1230 1

678 78 1

678 723 1

678 1172 1

678 1621 1

678 2261 1

678 584 0

678 97 0

678 777 0

678 1562 0

678 2573 0

678 402 0

678 551 0

679 35 1

679 1923 1

679 165 1

679 102 1

679 1514 1

679 715 1

679 875 1

679 1131 1

679 1136 1

679 52 1

679 1556 1

679 2262 1

679 2263 1

679 2264 1

679 1594 1

679 2013 1

679 1662 1

679 159 1

679 117 0

679 1716 0

679 626 0

679 3084 0

679 3456 0

679 2450 0

679 2282 0

679 1475 0

679 1734 0

679 2610 0

679 2991 0

679 1038 0

679 3304 0

679 2792 0

679 825 0

679 2082 0

679 3697 0

679 2530 0

680 241 1

680 52 1

680 2265 1

680 250 1

680 126 1

680 1703 0

680 755 0

680 61 0

680 3272 0

680 1183 0

681 96 1

681 2051 1

681 1893 1
681 70 1
681 2267 1
681 432 1
681 694 1
681 87 1
681 824 1
681 2266 1
681 1627 1
681 2268 1
681 2269 1
681 2270 1
681 2271 1
681 3569 0
681 1150 0
681 3827 0
681 2153 0
681 1104 0
681 655 0
681 3473 0
681 578 0
681 956 0
681 1056 0
681 3494 0
681 1691 0
681 2091 0
681 1341 0
681 3271 0
682 102 1
682 712 1

682 105 1

682 109 1

682 141 1

682 52 1

682 119 1

682 55 1

682 2532 0

682 3636 0

682 2969 0

682 2963 0

682 1332 0

682 1307 0

682 1308 0

682 3267 0

683 740 1

683 36 1

683 325 1

683 996 1

683 2170 1

683 535 1

683 250 1

683 1154 0

683 3133 0

683 1216 0

683 1466 0

683 3412 0

683 120 0

683 3749 0

684 2272 1

684 2081 1

684 1218 1

684 2273 1

684 2274 1

684 2275 1

684 134 1

684 232 1

684 1229 1

684 466 1

684 469 1

684 1397 1

684 1461 1

684 378 1

684 188 1

684 3317 0

684 1519 0

684 2547 0

684 1486 0

684 1709 0

684 946 0

684 1758 0

684 2836 0

684 1968 0

684 3063 0

684 3163 0

684 3167 0

684 3691 0

684 3704 0

684 316 0

685 96 1

685 355 1

685 1126 1
685 78 1
685 47 1
685 210 1
685 1589 1
685 118 1
685 120 1
685 1963 0
685 999 0
685 20 0
685 2784 0
685 3310 0
685 1847 0
685 293 0
685 2311 0
685 1771 0
686 2276 1
686 2277 1
686 2278 1
686 137 1
686 1325 1
686 656 1
686 2064 1
686 55 1
686 572 1
686 413 1
686 382 1
686 1318 0
686 3206 0
686 3594 0

686 2795 0

686 3550 0

686 2880 0

686 2059 0

686 2141 0

686 1145 0

686 2649 0

686 2589 0

687 355 1

687 261 1

687 2279 1

687 235 1

687 1772 1

687 1037 1

687 1165 1

687 1421 1

687 2028 1

687 956 1

687 3411 0

687 1515 0

687 107 0

687 2624 0

687 2647 0

687 393 0

687 2363 0

687 929 0

687 3766 0

687 2389 0

688 96 1

688 192 1

688 1408 1
688 2083 1
688 1156 1
688 73 1
688 714 1
688 269 1
688 558 1
688 245 1
688 118 1
688 254 1
688 347 1
688 253 1
688 126 1
688 3454 0
688 1077 0
688 789 0
688 3423 0
688 1162 0
688 1185 0
688 785 0
688 173 0
688 275 0
688 2630 0
688 385 0
688 1271 0
688 1404 0
688 2147 0
688 1566 0
689 196 1
689 2280 1

689 2281 1

689 460 1

689 209 1

689 1236 1

689 507 1

689 125 1

689 2197 0

689 873 0

689 1363 0

689 3144 0

689 2806 0

689 2113 0

689 2912 0

689 1088 0

690 1120 1

690 353 1

690 199 1

690 776 1

690 2282 1

690 2283 1

690 302 1

690 303 1

690 214 1

690 280 1

690 2015 1

690 1020 1

690 989 1

690 1503 1

690 2120 0

690 3071 0

690 2125 0

690 1852 0

690 1178 0

690 3199 0

690 59 0

690 1527 0

690 1100 0

690 982 0

690 2025 0

690 759 0

690 2466 0

690 2374 0

691 78 1

691 46 1

691 79 1

691 919 1

691 87 1

691 377 1

691 181 0

691 1473 0

691 513 0

691 3353 0

691 2825 0

691 1289 0

692 196 1

692 708 1

692 678 1

692 105 1

692 2284 1

692 46 1

692 307 1

692 155 1

692 221 1

692 286 1

692 2876 0

692 459 0

692 3627 0

692 3067 0

692 3742 0

692 1201 0

692 954 0

692 3271 0

692 439 0

692 1195 0

693 96 1

693 102 1

693 70 1

693 109 1

693 47 1

693 210 1

693 1172 1

693 118 1

693 122 1

693 3315 0

693 1336 0

693 797 0

693 3714 0

693 530 0

693 566 0

693 2063 0

693 2479 0

693 23 0

694 102 1

694 2285 1

694 2286 1

694 2287 1

694 208 1

694 2288 1

694 212 1

694 822 1

694 280 1

694 2105 0

694 1802 0

694 1774 0

694 1868 0

694 1050 0

694 547 0

694 1399 0

694 1989 0

694 84 0

695 550 1

695 80 1

695 2289 1

695 948 1

695 1558 1

695 1669 0

695 3613 0

695 602 0

695 2241 0

695 1738 0

696 1798 1

696 141 1

696 145 1

696 882 1

696 2290 1

696 52 1

696 2291 1

696 55 1

696 221 1

696 3750 0

696 315 0

696 2159 0

696 2644 0

696 2808 0

696 482 0

696 3585 0

696 2735 0

696 1116 0

697 1890 1

697 105 1

697 141 1

697 46 1

697 882 1

697 52 1

697 53 1

697 55 1

697 190 1

697 1389 0

697 925 0

697 2477 0

697 1268 0

697 3517 0

697 2529 0

697 2720 0

697 3312 0

697 3292 0

698 66 1

698 130 1

698 133 1

698 41 1

698 115 1

698 118 1

698 374 1

698 376 1

698 2297 1

698 3237 0

698 1193 0

698 2684 0

698 247 0

698 999 0

698 1985 0

698 748 0

698 3671 0

698 619 0

699 261 1

699 104 1

699 137 1

699 878 1

699 50 1

699 118 1

699 184 1

699 282 1

699 187 1

699 413 1

699 2001 0

699 3420 0

699 2424 0

699 3767 0

699 245 0

699 627 0

699 912 0

699 2939 0

699 361 0

699 3720 0

700 132 1

700 133 1

700 293 1

700 104 1

700 137 1

700 110 1

700 46 1

700 469 1

700 1781 1

700 57 1

700 187 1

700 2675 0

700 2240 0

700 1210 0

700 741 0

700 2860 0

700 841 0

700 3243 0

700 3530 0

700 2168 0

700 1087 0

700 3639 0

701 96 1

701 134 1

701 775 1

701 1259 1

701 47 1

701 560 1

701 210 1

701 1523 1

701 52 1

701 1524 1

701 188 1

701 2173 1

701 2636 0

701 809 0

701 1777 0

701 116 0

701 3551 0

701 3827 0

701 570 0

701 3633 0

701 572 0

701 1378 0

701 1357 0

701 120 0

702 257 1

702 356 1

702 485 1

702 1353 1

702 2090 1

702 686 1

702 975 1

702 498 1

702 1235 1

702 277 1

702 22 1

702 886 1

702 2298 1

702 2299 1

702 1308 1

702 3558 0

702 1103 0

702 965 0

702 481 0

702 2088 0

702 1369 0

702 636 0

702 1277 0

702 1558 0

702 2944 0

702 666 0

702 546 0

702 3566 0

702 1896 0

702 3631 0

703 13 1

703 275 1

703 52 1

703 1268 1

703 118 1

703 1812 1

703 2302 1

703 2301 1

703 2300 1

703 445 1

703 446 1

703 1558 0

703 1615 0

703 2267 0

703 2687 0

703 2335 0

703 2146 0

703 872 0

703 2061 0

703 150 0

703 1467 0

703 3413 0

704 96 1

704 493 1

704 494 1

704 80 1

704 210 1

704 915 1

704 118 1

704 119 1

704 477 1

704 126 1

704 2881 0

704 2221 0

704 3264 0

704 1113 0

704 3806 0

704 1527 0

704 658 0

704 846 0

704 1129 0

704 3268 0

705 608 1

705 235 1

705 109 1

705 46 1

705 1102 1

705 53 1

705 887 1

705 765 1

705 286 1

705 1565 0

705 3818 0

705 369 0

705 2535 0

705 3510 0

705 2119 0

705 598 0

705 1424 0

705 2376 0

706 2304 1
706 2305 1
706 2303 1
706 165 1
706 422 1
706 295 1
706 456 1
706 111 1
706 821 1
706 1496 1
706 187 1
706 508 1
706 157 1
706 158 1
706 159 1
706 2204 0
706 834 0
706 565 0
706 1513 0
706 3682 0
706 326 0
706 2301 0
706 1014 0
706 2975 0
706 2895 0
706 1939 0
706 3674 0
706 2668 0
706 3727 0
706 2218 0

707 1408 1
707 2306 1
707 2307 1
707 2308 1
707 2309 1
707 268 1
707 13 1
707 1042 1
707 47 1
707 52 1
707 694 1
707 445 1
707 192 1
707 74 1
707 332 1
707 80 1
707 87 1
707 1115 1
707 2140 1
707 96 1
707 227 1
707 3000 0
707 251 0
707 3792 0
707 187 0
707 2291 0
707 1141 0
707 3612 0
707 1732 0
707 1157 0

707 3231 0

707 1484 0

707 2464 0

707 2469 0

707 682 0

707 241 0

707 2713 0

707 189 0

707 2490 0

707 2174 0

707 3539 0

707 2500 0

708 192 1

708 1154 1

708 376 1

708 2310 1

708 377 1

708 267 1

708 206 1

708 79 1

708 184 1

708 185 1

708 3727 0

708 3142 0

708 1200 0

708 1188 0

708 3436 0

708 1775 0

708 1778 0

708 330 0

708 1864 0

708 374 0

709 35 1

709 389 1

709 41 1

709 107 1

709 46 1

709 118 1

709 1750 1

709 1718 0

709 150 0

709 2953 0

709 923 0

709 3279 0

709 1919 0

709 2596 0

710 96 1

710 98 1

710 899 1

710 133 1

710 261 1

710 73 1

710 74 1

710 494 1

710 80 1

710 254 1

710 127 1

710 2913 0

710 2372 0

710 2779 0

710 2973 0

710 3523 0

710 295 0

710 1442 0

710 1089 0

710 2933 0

710 1159 0

710 928 0

711 128 1

711 418 1

711 67 1

711 70 1

711 2311 1

711 136 1

711 2312 1

711 490 1

711 2313 1

711 1228 1

711 399 1

711 1776 1

711 55 1

711 793 1

711 795 1

711 60 1

711 2587 0

711 2676 0

711 994 0

711 2007 0

711 1573 0

711 1457 0

711 2523 0

711 96 0

711 3316 0

711 1479 0

711 2895 0

711 1565 0

711 321 0

711 3690 0

711 116 0

711 108 0

712 2314 1

712 2315 1

712 2316 1

712 1454 1

712 1979 1

712 361 0

712 1672 0

712 303 0

712 797 0

712 596 0

713 224 1

713 1730 1

713 36 1

713 712 1

713 109 1

713 526 1

713 47 1

713 2317 1

713 2318 1

713 52 1

713 1812 1
713 2907 0
713 2194 0
713 3561 0
713 690 0
713 1700 0
713 906 0
713 2990 0
713 2150 0
713 70 0
713 940 0
713 2637 0
714 738 1
714 133 1
714 102 1
714 588 1
714 1133 1
714 2323 1
714 52 1
714 2324 1
714 119 1
714 220 1
714 1844 0
714 1693 0
714 1420 0
714 2384 0
714 1137 0
714 3424 0
714 44 0
714 546 0

714 1342 0

714 137 0

715 1634 1

715 835 1

715 132 1

715 5 1

715 356 1

715 1321 1

715 331 1

715 13 1

715 654 1

715 1646 1

715 273 1

715 277 1

715 2325 1

715 2326 1

715 729 1

715 1083 1

715 861 1

715 2785 0

715 3696 0

715 2586 0

715 683 0

715 2197 0

715 1059 0

715 2109 0

715 2094 0

715 388 0

715 1435 0

715 12 0

715 1984 0
715 732 0
715 1556 0
715 2466 0
715 3621 0
715 1635 0
716 832 1
716 66 1
716 1219 1
716 133 1
716 137 1
716 75 1
716 79 1
716 47 1
716 1202 1
716 117 1
716 87 1
716 154 1
716 2327 1
716 2441 0
716 3582 0
716 2434 0
716 420 0
716 1545 0
716 2337 0
716 1540 0
716 376 0
716 1429 0
716 2908 0
716 1385 0

716 3429 0

716 501 0

717 192 1

717 1145 1

717 36 1

717 1540 1

717 378 1

717 1809 1

717 1267 1

717 502 1

717 2328 1

717 441 1

717 250 1

717 1275 1

717 189 1

717 126 1

717 2968 0

717 1475 0

717 2987 0

717 3574 0

717 845 0

717 927 0

717 679 0

717 3121 0

717 1900 0

717 2043 0

717 3662 0

717 1526 0

717 2289 0

717 172 0

718 257 1

718 131 1

718 36 1

718 1352 1

718 361 1

718 78 1

718 275 1

718 22 1

718 2046 1

718 24 1

718 571 1

718 28 1

718 254 1

718 127 1

718 431 0

718 1825 0

718 1748 0

718 746 0

718 2032 0

718 2461 0

718 669 0

718 3461 0

718 3340 0

718 1187 0

718 1606 0

718 729 0

718 2259 0

718 1360 0

719 98 1

719 133 1

719 135 1

719 72 1

719 73 1

719 77 1

719 206 1

719 79 1

719 80 1

719 87 1

719 184 1

719 185 1

719 2925 0

719 3396 0

719 480 0

719 3647 0

719 640 0

719 1645 0

719 2553 0

719 3108 0

719 375 0

719 558 0

719 2854 0

719 2758 0

720 1728 1

720 195 1

720 196 1

720 2330 1

720 462 1

720 2331 1

720 1496 1

720 2329 1

720 314 1

720 155 1

720 2332 1

720 2333 1

720 2334 1

720 1351 0

720 2914 0

720 216 0

720 3741 0

720 746 0

720 535 0

720 1445 0

720 1487 0

720 2814 0

720 113 0

720 1196 0

720 1849 0

720 3731 0

721 664 1

721 154 1

721 670 1

721 159 1

721 163 1

721 2339 1

721 2340 1

721 297 1

721 48 1

721 1712 1

721 187 1

721 196 1

721 453 1

721 715 1

721 1105 1

721 989 1

721 878 1

721 111 1

721 511 1

721 3587 0

721 3323 0

721 3557 0

721 2342 0

721 3741 0

721 2891 0

721 1590 0

721 921 0

721 1256 0

721 3637 0

721 1865 0

721 1027 0

721 3067 0

721 3751 0

721 1504 0

721 2011 0

721 1338 0

721 1807 0

721 3135 0

722 2272 1

722 1253 1

722 392 1

722 105 1

722 1260 1
722 109 1
722 52 1
722 2234 1
722 1899 0
722 3555 0
722 1376 0
722 512 0
722 3593 0
722 2732 0
722 3162 0
722 1564 0
723 2114 1
723 707 1
723 261 1
723 136 1
723 52 1
723 475 1
723 253 1
723 3069 0
723 985 0
723 3085 0
723 1448 0
723 2366 0
723 686 0
723 3006 0
724 96 1
724 833 1
724 708 1
724 199 1

724 105 1

724 107 1

724 301 1

724 46 1

724 2319 1

724 1297 1

724 210 1

724 118 1

724 376 1

724 1020 1

724 669 0

724 2557 0

724 2107 0

724 620 0

724 415 0

724 3053 0

724 3548 0

724 3239 0

724 618 0

724 2317 0

724 2734 0

724 2024 0

724 2794 0

724 3049 0

725 948 1

725 60 1

725 2341 1

725 3208 0

725 1342 0

725 3278 0

726 96 1

726 1408 1

726 70 1

726 2342 1

726 2343 1

726 522 1

726 529 1

726 52 1

726 2512 0

726 583 0

726 2667 0

726 2807 0

726 2706 0

726 1514 0

726 345 0

726 2480 0

727 96 1

727 2081 1

727 1051 1

727 377 1

727 2344 1

727 79 1

727 239 1

727 87 1

727 184 1

727 185 1

727 91 1

727 1278 1

727 127 1

727 2071 0

727 759 0

727 2781 0

727 215 0

727 390 0

727 640 0

727 3033 0

727 3797 0

727 3631 0

727 399 0

727 67 0

727 2043 0

727 2115 0

728 1476 1

728 293 1

728 808 1

728 105 1

728 498 1

728 118 1

728 572 1

728 190 1

728 799 1

728 2877 0

728 633 0

728 540 0

728 1679 0

728 1368 0

728 295 0

728 2891 0

728 154 0

728 2624 0

729 96 1

729 1538 1

729 771 1

729 804 1

729 102 1

729 199 1

729 2345 1

729 107 1

729 47 1

729 118 1

729 1178 1

729 989 1

729 745 0

729 3411 0

729 297 0

729 1220 0

729 2787 0

729 951 0

729 130 0

729 2774 0

729 1612 0

729 1017 0

729 2863 0

729 2567 0

730 66 1

730 131 1

730 132 1

730 133 1

730 230 1

730 72 1

730 361 1

730 74 1

730 1325 1

730 46 1

730 80 1

730 87 1

730 55 1

730 957 1

730 1446 0

730 1190 0

730 3086 0

730 3202 0

730 2927 0

730 38 0

730 1080 0

730 1512 0

730 1776 0

730 2834 0

730 3483 0

730 2596 0

730 64 0

730 1244 0

731 354 1

731 1315 1

731 105 1

731 777 1

731 2346 1

731 46 1

731 399 1

731 755 1

731 55 1

731 190 1

731 2531 0

731 2744 0

731 2407 0

731 2761 0

731 3074 0

731 2669 0

731 1152 0

731 1704 0

731 3663 0

731 3688 0

732 96 1

732 135 1

732 72 1

732 74 1

732 80 1

732 87 1

732 185 1

732 956 1

732 254 1

732 69 0

732 3596 0

732 2381 0

732 1413 0

732 539 0

732 364 0

732 248 0

732 1872 0

732 2038 0

733 673 1

733 803 1

733 325 1

733 741 1

733 1867 1

733 588 1

733 2348 1

733 2349 1

733 751 1

733 2350 1

733 2351 1

733 2352 1

733 217 1

733 1471 1

733 1533 0

733 902 0

733 38 0

733 3618 0

733 1315 0

733 2475 0

733 2448 0

733 320 0

733 1077 0

733 2308 0

733 1909 0

733 152 0

733 1047 0

733 1265 0

734 2065 1

734 2353 1

734 883 1

734 275 1

734 277 1

734 2354 1

734 2355 1

734 888 1

734 2356 1

734 2300 1

734 1355 0

734 2803 0

734 2028 0

734 970 0

734 665 0

734 2954 0

734 341 0

734 355 0

734 291 0

734 1232 0

735 36 1

735 241 1

735 243 1

735 788 1

735 917 1

735 2357 1

735 2358 1

735 250 1

735 2448 0

735 3299 0

735 2463 0

735 820 0

735 503 0
735 2214 0
735 3568 0
735 3345 0
736 32 1
736 145 1
736 2130 1
736 499 1
736 1655 1
736 2360 1
736 2361 1
736 2362 1
736 2363 1
736 2359 1
736 1310 0
736 3256 0
736 1765 0
736 3647 0
736 2432 0
736 316 0
736 1332 0
736 1698 0
736 3305 0
736 2291 0
737 13 1
737 275 1
737 27 1
737 541 1
737 546 1
737 164 1

737 676 1

737 684 1

737 2364 1

737 2365 1

737 2366 1

737 2367 1

737 2368 1

737 2369 1

737 963 1

737 965 1

737 474 1

737 1635 1

737 356 1

737 3173 0

737 2521 0

737 1808 0

737 2084 0

737 2469 0

737 2899 0

737 1571 0

737 2427 0

737 1212 0

737 631 0

737 637 0

737 1709 0

737 2433 0

737 3659 0

737 2462 0

737 2645 0

737 1353 0

737 1060 0

737 3453 0

738 2373 1

738 2374 1

738 74 1

738 237 1

738 79 1

738 115 1

738 87 1

738 127 1

738 522 0

738 1768 0

738 176 0

738 1858 0

738 2924 0

738 3299 0

738 370 0

738 2524 0

739 353 1

739 196 1

739 36 1

739 198 1

739 2375 1

739 55 1

739 1528 1

739 156 1

739 158 1

739 2329 0

739 3147 0

739 2975 0

739 2282 0

739 2960 0

739 1736 0

739 2206 0

739 705 0

739 71 0

740 96 1

740 2376 1

740 2377 1

740 2378 1

740 2379 1

740 2380 1

740 109 1

740 2381 1

740 498 1

740 883 1

740 22 1

740 151 1

740 442 1

740 861 1

740 286 1

740 895 1

740 2456 0

740 3259 0

740 485 0

740 2908 0

740 3843 0

740 646 0

740 3362 0

740 2113 0

740 1292 0

740 2716 0

740 3807 0

740 324 0

740 3066 0

740 1507 0

740 2576 0

740 436 0

741 102 1

741 2382 1

741 655 1

741 2383 1

741 2384 1

741 210 1

741 563 1

741 253 1

741 2503 0

741 3446 0

741 414 0

741 710 0

741 2104 0

741 622 0

741 1430 0

741 562 0

742 134 1

742 267 1

742 110 1

742 80 1

742 2385 1

742 2386 1

742 854 1

742 863 1

742 538 1

742 252 1

742 127 1

742 37 0

742 3532 0

742 1879 0

742 149 0

742 2891 0

742 3822 0

742 808 0

742 3165 0

742 314 0

742 1913 0

742 675 0

743 96 1

743 130 1

743 133 1

743 134 1

743 135 1

743 364 1

743 79 1

743 80 1

743 118 1

743 87 1

743 374 1

743 1403 1

743 127 1

743 1347 0

743 495 0

743 2254 0

743 3639 0

743 2007 0

743 571 0

743 3456 0

743 3712 0

743 2421 0

743 3162 0

743 370 0

743 859 0

743 3590 0

744 96 1

744 2392 1

744 2393 1

744 622 1

744 2387 1

744 2388 1

744 2389 1

744 630 1

744 2390 1

744 632 1

744 2391 1

744 1754 1

744 637 1

744 254 1

744 369 0

744 1494 0

744 2226 0

744 2073 0

744 3653 0

744 102 0

744 742 0

744 3728 0

744 2931 0

744 1130 0

744 2786 0

744 3621 0

744 2486 0

744 3523 0

745 96 1

745 869 1

745 870 1

745 199 1

745 47 1

745 371 1

745 212 1

745 2396 1

745 215 1

745 218 1

745 2395 1

745 220 1

745 2397 1

745 867 0

745 2387 0

745 1890 0

745 1327 0

745 2456 0

745 3460 0

745 3179 0

745 487 0

745 1061 0

745 2241 0

745 2243 0

745 2922 0

745 1508 0

746 128 1

746 2398 1

746 382 1

746 483 1

746 196 1

746 1496 1

746 456 1

746 105 1

746 107 1

746 1586 1

746 890 1

746 22 1

746 55 1

746 24 1

746 56 1

746 413 1

746 158 1

746 2399 1

746 449 0

746 1491 0

746 1844 0

746 2468 0

746 1580 0

746 2448 0

746 1 0

746 1695 0

746 3265 0

746 2578 0

746 2993 0

746 2969 0

746 3269 0

746 1286 0

746 3204 0

746 1093 0

746 2515 0

746 195 0

747 2400 1

747 2401 1

747 102 1

747 1258 1

747 171 1

747 267 1

747 270 1

747 79 1

747 560 1

747 55 1

747 184 1

747 124 0

747 853 0

747 1933 0

747 3769 0

747 2421 0

747 1500 0

747 2141 0

747 1676 0
747 1443 0
747 2975 0
747 626 0
748 133 1
748 74 1
748 75 1
748 78 1
748 79 1
748 373 1
748 87 1
748 538 1
748 127 1
748 2202 0
748 2766 0
748 1774 0
748 1990 0
748 1577 0
748 1875 0
748 1932 0
748 1111 0
748 579 0
749 2402 1
749 899 1
749 135 1
749 72 1
749 74 1
749 1258 1
749 79 1
749 919 1

749 372 1

749 87 1

749 185 1

749 698 1

749 127 1

749 1810 0

749 1176 0

749 959 0

749 3563 0

749 2605 0

749 2339 0

749 2049 0

749 3654 0

749 2502 0

749 200 0

749 661 0

749 1158 0

749 944 0

750 2401 1

750 1122 1

750 1756 1

750 2403 1

750 1445 1

750 391 1

750 137 1

750 651 1

750 78 1

750 764 1

750 697 0

750 2948 0

750 3358 0

750 3808 0

750 973 0

750 1431 0

750 3355 0

750 722 0

750 3502 0

750 1488 0

751 96 1

751 2404 1

751 293 1

751 102 1

751 518 1

751 1126 1

751 46 1

751 47 1

751 1777 1

751 210 1

751 52 1

751 119 1

751 190 1

751 876 0

751 2545 0

751 896 0

751 3784 0

751 2184 0

751 2370 0

751 1640 0

751 587 0

751 3097 0

751 3524 0

751 92 0

751 2139 0

751 1045 0

752 96 1

752 66 1

752 79 1

752 87 1

752 1465 1

752 127 1

752 2299 0

752 3198 0

752 1041 0

752 323 0

752 2374 0

752 1113 0

753 2405 1

753 2406 1

753 2407 1

753 2408 1

753 2409 1

753 2410 1

753 2411 1

753 2412 1

753 2413 1

753 2414 1

753 2415 1

753 2416 1

753 2417 1

753 2418 1

753 2419 1
753 2420 1
753 2421 1
753 1371 1
753 940 0
753 2599 0
753 3475 0
753 2166 0
753 2336 0
753 1445 0
753 3077 0
753 1892 0
753 1523 0
753 459 0
753 333 0
753 935 0
753 2146 0
753 3342 0
753 2338 0
753 267 0
753 2111 0
753 787 0
754 516 1
754 109 1
754 46 1
754 1389 1
754 433 1
754 52 1
754 118 1
754 55 1

754 474 1

754 1854 1

754 2573 0

754 611 0

754 2059 0

754 1733 0

754 2052 0

754 1109 0

754 2168 0

754 3221 0

754 0 0

754 3028 0

755 1248 1

755 418 1

755 67 1

755 510 1

755 426 1

755 396 1

755 1100 1

755 1617 1

755 2422 1

755 2423 1

755 2424 1

755 2425 1

755 2426 1

755 60 1

755 318 1

755 735 1

755 2830 0

755 1712 0

755 1760 0
755 1677 0
755 3758 0
755 2237 0
755 2625 0
755 3843 0
755 2877 0
755 1688 0
755 2483 0
755 971 0
755 86 0
755 25 0
755 916 0
755 1778 0
756 224 1
756 902 1
756 199 1
756 873 1
756 906 1
756 427 1
756 47 1
756 944 1
756 2427 1
756 2428 1
756 989 1
756 1854 1
756 575 1
756 2347 0
756 3281 0
756 396 0

756 2049 0

756 142 0

756 1003 0

756 1136 0

756 2688 0

756 2727 0

756 740 0

756 1839 0

756 715 0

756 2945 0

757 36 1

757 391 1

757 105 1

757 109 1

757 46 1

757 141 1

757 878 1

757 53 1

757 55 1

757 2429 1

757 190 1

757 677 0

757 707 0

757 2956 0

757 971 0

757 2446 0

757 536 0

757 712 0

757 1374 0

757 621 0

757 2805 0

757 1670 0

758 2432 1

758 2433 1

758 2430 1

758 163 1

758 2434 1

758 2435 1

758 2436 1

758 295 1

758 40 1

758 2431 1

758 46 1

758 2224 1

758 467 1

758 309 1

758 1526 1

758 664 1

758 2046 1

758 511 1

758 1019 0

758 3304 0

758 1889 0

758 3843 0

758 2876 0

758 828 0

758 1553 0

758 35 0

758 2855 0

758 602 0

758 2033 0

758 2800 0

758 1065 0

758 2146 0

758 76 0

758 1872 0

758 1487 0

758 1758 0

759 96 1

759 535 1

759 2437 1

759 2438 1

759 242 1

759 276 1

759 118 1

759 374 1

759 378 1

759 347 1

759 126 1

759 1305 0

759 1139 0

759 3223 0

759 657 0

759 1655 0

759 349 0

759 1684 0

759 2967 0

759 975 0

759 3677 0

759 1484 0

760 1184 1
760 2439 1
760 652 1
760 945 1
760 757 1
760 1302 1
760 3350 0
760 2491 0
760 447 0
760 1619 0
760 620 0
760 2289 0
761 34 1
761 36 1
761 2440 1
761 2219 1
761 1901 1
761 879 1
761 788 1
761 347 1
761 221 1
761 205 0
761 1608 0
761 50 0
761 2844 0
761 1941 0
761 962 0
761 973 0
761 3010 0
761 1555 0

762 353 1

762 223 1

762 196 1

762 105 1

762 106 1

762 511 1

762 878 1

762 111 1

762 53 1

762 1494 1

762 55 1

762 154 1

762 858 1

762 190 1

762 159 1

762 2219 0

762 3129 0

762 3149 0

762 496 0

762 809 0

762 2104 0

762 2311 0

762 959 0

762 996 0

762 3078 0

762 1109 0

762 2678 0

762 490 0

762 2015 0

762 1919 0

763 96 1

763 134 1

763 2441 1

763 171 1

763 363 1

763 364 1

763 365 1

763 80 1

763 1200 1

763 1523 1

763 87 1

763 592 0

763 71 0

763 2360 0

763 1339 0

763 1716 0

763 885 0

763 20 0

763 1283 0

763 2828 0

763 157 0

763 2385 0

764 2370 1

764 2307 1

764 2371 1

764 1893 1

764 2442 1

764 2443 1

764 1036 1

764 2444 1

764 2445 1
764 2270 1
764 21 1
764 1754 1
764 378 1
764 1115 1
764 893 1
764 126 1
764 625 0
764 3544 0
764 3333 0
764 3800 0
764 1992 0
764 3658 0
764 1026 0
764 3352 0
764 2420 0
764 987 0
764 3111 0
764 2927 0
764 784 0
764 2565 0
764 1210 0
764 465 0
765 34 1
765 806 1
765 42 1
765 2446 1
765 245 1
765 285 1

765 221 1

765 126 1

765 372 0

765 2012 0

765 2059 0

765 3136 0

765 1278 0

765 1552 0

765 3018 0

765 660 0

766 102 1

766 871 1

766 199 1

766 969 1

766 427 1

766 2448 1

766 210 1

766 253 0

766 1355 0

766 2851 0

766 2442 0

766 3466 0

766 2868 0

766 474 0

767 96 1

767 36 1

767 2449 1

767 499 1

767 118 1

767 3224 0

767 2890 0

767 206 0

767 1191 0

767 2515 0

768 66 1

768 1219 1

768 104 1

768 2450 1

768 22 1

768 91 1

768 1277 1

768 127 1

768 1733 0

768 3508 0

768 1568 0

768 979 0

768 1480 0

768 3599 0

768 1692 0

768 3303 0

769 517 1

769 1804 1

769 2451 1

769 2452 1

769 155 1

769 156 1

769 158 1

769 35 1

769 165 1

769 57 1

769	314	1
769	196	1
769	197	1
769	198	1
769	460	1
769	1484	1
769	462	1
769	727	1
769	472	1
769	474	1
769	735	1
769	486	1
769	1512	1
769	763	1
769	2866	0
769	3013	0
769	2128	0
769	2523	0
769	2914	0
769	3746	0
769	2796	0
769	2496	0
769	3051	0
769	3029	0
769	2264	0
769	312	0
769	2629	0
769	225	0
769	3426	0
769	2423	0

769	3241	0
769	2958	0
769	1871	0
769	3299	0
769	292	0
769	3817	0
769	2905	0
769	2047	0
770	34	1
770	1091	1
770	196	1
770	100	1
770	356	1
770	388	1
770	517	1
770	457	1
770	474	1
770	1236	1
770	2453	1
770	2454	1
770	2455	1
770	57	1
770	442	1
770	155	1
770	286	1
770	402	0
770	1867	0
770	2171	0
770	254	0
770	2647	0

770 3352 0

770 2117 0

770 2172 0

770 987 0

770 900 0

770 2394 0

770 323 0

770 2440 0

770 520 0

770 3006 0

770 2951 0

770 889 0

771 96 1

771 800 1

771 2456 1

771 1739 1

771 493 1

771 209 1

771 210 1

771 118 1

771 216 1

771 125 1

771 3209 0

771 3427 0

771 486 0

771 3842 0

771 212 0

771 1809 0

771 3215 0

771 913 0

771 2638 0

771 3683 0

772 673 1

772 1346 1

772 36 1

772 2457 1

772 52 1

772 278 1

772 57 1

772 2458 1

772 2459 1

772 286 1

772 225 0

772 2135 0

772 3443 0

772 2543 0

772 123 0

772 2108 0

772 471 0

772 1056 0

772 2720 0

772 206 0

773 352 1

773 1730 1

773 133 1

773 41 1

773 2281 1

773 2318 1

773 1523 1

773 2460 1

773 188 1

773 1854 1

773 2940 0

773 3158 0

773 1943 0

773 3562 0

773 2545 0

773 510 0

773 2139 0

773 3820 0

773 1044 0

773 1785 0

774 512 1

774 513 1

774 515 1

774 519 1

774 1419 1

774 791 1

774 795 1

774 155 1

774 157 1

774 2461 1

774 2462 1

774 2463 1

774 2464 1

774 2465 1

774 419 1

774 2466 1

774 2467 1

774 422 1

774 1325 1
774 570 1
774 1483 1
774 844 1
774 727 1
774 1248 1
774 1513 1
774 508 1
774 509 1
774 510 1
774 1759 0
774 2756 0
774 113 0
774 2497 0
774 2739 0
774 1287 0
774 1261 0
774 3562 0
774 3528 0
774 1677 0
774 1306 0
774 3504 0
774 2253 0
774 2655 0
774 721 0
774 77 0
774 1310 0
774 1709 0
774 2744 0
774 2556 0

774 2687 0
774 500 0
774 1477 0
774 1386 0
774 1993 0
774 1019 0
774 944 0
774 1961 0
775 2468 1
775 2469 1
775 102 1
775 2470 1
775 2471 1
775 1132 1
775 1365 1
775 1015 1
775 827 1
775 1444 0
775 213 0
775 2259 0
775 3509 0
775 7 0
775 884 0
775 3176 0
775 2313 0
775 3019 0
776 802 1
776 134 1
776 199 1
776 776 1

776 137 1

776 2472 1

776 1324 1

776 1869 1

776 302 1

776 47 1

776 16 1

776 303 1

776 560 1

776 915 1

776 118 1

776 859 1

776 254 1

776 2711 0

776 3456 0

776 482 0

776 3281 0

776 2051 0

776 1365 0

776 3619 0

776 2434 0

776 399 0

776 2713 0

776 2410 0

776 1192 0

776 1396 0

776 2431 0

776 1756 0

776 3497 0

776 38 0

777 325 1

777 2473 1

777 42 1

777 362 1

777 211 1

777 152 1

777 2696 0

777 2717 0

777 1028 0

777 2085 0

777 748 0

777 1263 0

778 1794 1

778 164 1

778 676 1

778 677 1

778 933 1

778 934 1

778 2474 1

778 684 1

778 940 1

778 945 1

778 306 1

778 308 1

778 314 1

778 2365 1

778 2332 1

778 541 1

778 1830 0

778 2544 0

778 3002	0
778 2626	0
778 3793	0
778 299	0
778 3099	0
778 3415	0
778 2961	0
778 1655	0
778 2559	0
778 2834	0
778 839	0
778 253	0
778 2775	0
778 3197	0
779 1889	1
779 386	1
779 899	1
779 1122	1
779 136	1
779 73	1
779 137	1
779 1258	1
779 2475	1
779 77	1
779 923	1
779 2913	0
779 926	0
779 2413	0
779 986	0
779 2793	0

779 2909 0

779 2629 0

779 551 0

779 3413 0

779 1343 0

779 3718 0

780 163 1

780 932 1

780 165 1

780 453 1

780 235 1

780 2476 1

780 909 1

780 2477 1

780 111 1

780 1712 1

780 2452 1

780 53 1

780 1528 1

780 159 1

780 532 0

780 2275 0

780 2221 0

780 2552 0

780 3296 0

780 3783 0

780 3505 0

780 1649 0

780 538 0

780 993 0

780 2374 0

780 375 0

780 580 0

780 2315 0

781 1184 1

781 2016 1

781 228 1

781 1253 1

781 392 1

781 105 1

781 107 1

781 652 1

781 46 1

781 2478 1

781 2479 1

781 55 1

781 190 1

781 418 0

781 812 0

781 3662 0

781 770 0

781 577 0

781 3800 0

781 1365 0

781 1583 0

781 3032 0

781 2873 0

781 1448 0

781 483 0

781 3606 0

782 1549 1

782 13 1

782 2189 1

782 2480 1

782 2481 1

782 2482 1

782 2483 1

782 1268 1

782 25 1

782 956 1

782 2302 1

782 1404 0

782 1590 0

782 1916 0

782 3524 0

782 816 0

782 1752 0

782 2148 0

782 74 0

782 1066 0

782 3523 0

782 1451 0

783 98 1

783 1959 1

783 72 1

783 74 1

783 75 1

783 77 1

783 78 1

783 79 1

783 372 1

783 2484 1

783 2485 1

783 184 1

783 185 1

783 254 1

783 3796 0

783 217 0

783 3244 0

783 3276 0

783 597 0

783 311 0

783 1622 0

783 1777 0

783 30 0

783 985 0

783 2816 0

783 417 0

783 1400 0

783 3166 0

784 1412 1

784 135 1

784 72 1

784 377 1

784 75 1

784 77 1

784 237 1

784 79 1

784 87 1

784 185 1

784 127 1

784 558 0

784 702 0

784 3652 0

784 3731 0

784 60 0

784 764 0

784 104 0

784 2215 0

784 2014 0

784 2967 0

784 988 0

785 32 1

785 385 1

785 674 1

785 132 1

785 41 1

785 75 1

785 238 1

785 1043 1

785 151 1

785 377 1

785 378 1

785 539 1

785 126 1

785 255 1

785 128 0

785 1945 0

785 1314 0

785 942 0

785 422 0

785 3654 0

785 2437 0

785 419 0

785 680 0

785 2294 0

785 808 0

785 1393 0

785 87 0

785 2223 0

786 580 1

786 70 1

786 874 1

786 303 1

786 208 1

786 212 1

786 2486 1

786 119 1

786 2456 1

786 125 1

786 1739 0

786 650 0

786 1399 0

786 3331 0

786 2090 0

786 3031 0

786 1379 0

786 824 0

786 827 0

786 1267 0

787 224 1

787 512 1

787 2051 1

787 708 1

787 390 1

787 391 1

787 510 1

787 46 1

787 112 1

787 2487 1

787 2488 1

787 2489 1

787 2490 1

787 1307 1

787 190 1

787 62 1

787 2841 0

787 3139 0

787 1970 0

787 671 0

787 585 0

787 1567 0

787 3474 0

787 3802 0

787 228 0

787 3047 0

787 1059 0

787 3093 0

787 569 0

787 31 0

787 1704 0
787 2499 0
788 36 1
788 105 1
788 235 1
788 523 1
788 109 1
788 46 1
788 941 1
788 114 1
788 52 1
788 2491 1
788 2492 1
788 286 1
788 3182 0
788 2976 0
788 308 0
788 1195 0
788 1209 0
788 3171 0
788 2220 0
788 2087 0
788 2789 0
788 1961 0
788 2764 0
788 938 0
789 192 1
789 1889 1
789 130 1
789 133 1

789 358 1

789 361 1

789 376 1

789 57 1

789 538 1

789 187 1

789 2494 1

789 509 0

789 1644 0

789 964 0

789 3567 0

789 814 0

789 536 0

789 1194 0

789 2053 0

789 1298 0

789 1455 0

789 3735 0

790 96 1

790 129 1

790 131 1

790 392 1

790 1352 1

790 1452 1

790 80 1

790 178 1

790 2454 1

790 697 1

790 538 1

790 1084 1

790 1048 0

790 1796 0

790 3061 0

790 3387 0

790 2274 0

790 2584 0

790 3649 0

790 1697 0

790 635 0

790 1616 0

790 3780 0

790 315 0

791 928 1

791 769 1

791 34 1

791 961 1

791 229 1

791 741 1

791 1289 1

791 362 1

791 2219 1

791 752 1

791 306 1

791 1010 1

791 151 1

791 1528 1

791 315 1

791 1437 1

791 2495 1

791 3482 0

791 363 0

791 1726 0

791 1006 0

791 943 0

791 490 0

791 3373 0

791 625 0

791 1232 0

791 3653 0

791 1143 0

791 2967 0

791 1425 0

791 534 0

791 1698 0

791 3216 0

791 1488 0

792 832 1

792 2496 1

792 2497 1

792 2147 1

792 772 1

792 1188 1

792 134 1

792 102 1

792 75 1

792 1550 1

792 47 1

792 951 1

792 378 1

792 1820 1

792 255 1
792 3722 0
792 3270 0
792 466 0
792 2737 0
792 3467 0
792 682 0
792 1853 0
792 1365 0
792 3733 0
792 3691 0
792 2090 0
792 2115 0
792 2587 0
792 219 0
792 2178 0
793 353 1
793 131 1
793 132 1
793 923 1
793 137 1
793 1324 1
793 206 1
793 433 1
793 87 1
793 539 1
793 540 1
793 254 1
793 727 0
793 2685 0

793 1622 0

793 851 0

793 2429 0

793 2528 0

793 2475 0

793 1445 0

793 3285 0

793 1460 0

793 3629 0

793 2397 0

794 2498 1

794 231 1

794 360 1

794 105 1

794 107 1

794 46 1

794 1235 1

794 55 1

794 858 1

794 190 1

794 863 1

794 2477 0

794 2169 0

794 713 0

794 1370 0

794 1569 0

794 1078 0

794 1543 0

794 920 0

794 1740 0

794 1073 0

794 915 0

795 105 1

795 109 1

795 46 1

795 114 1

795 52 1

795 55 1

795 890 1

795 187 1

795 863 1

795 2726 0

795 3268 0

795 1099 0

795 2642 0

795 2446 0

795 1459 0

795 327 0

795 1843 0

795 2566 0

796 754 1

796 1748 1

796 1300 1

796 2499 1

796 1047 0

796 3808 0

796 2864 0

796 2909 0

797 129 1

797 771 1

797 261 1

797 1036 1

797 2064 1

797 548 1

797 1082 1

797 192 1

797 2500 1

797 2501 1

797 2502 1

797 2503 1

797 338 1

797 476 1

797 118 1

797 253 1

797 1789 1

797 126 1

797 1791 1

797 1910 0

797 1561 0

797 1422 0

797 3354 0

797 2218 0

797 284 0

797 3574 0

797 2777 0

797 2716 0

797 2537 0

797 2252 0

797 2186 0

797 2834 0

797 115 0
797 1481 0
797 1238 0
797 1227 0
797 3166 0
797 2772 0
798 242 1
798 991 1
798 1029 1
798 535 1
798 1278 0
798 1393 0
798 410 0
798 2126 0
799 36 1
799 102 1
799 134 1
799 775 1
799 755 1
799 52 1
799 1523 1
799 188 1
799 2462 0
799 1765 0
799 1113 0
799 2208 0
799 1086 0
799 3571 0
799 3627 0
799 3415 0

800 34 1

800 354 1

800 36 1

800 708 1

800 1346 1

800 1347 1

800 1350 1

800 2504 1

800 42 1

800 109 1

800 654 1

800 1327 1

800 1051 1

800 286 1

800 2709 0

800 3649 0

800 2506 0

800 38 0

800 1040 0

800 900 0

800 214 0

800 1223 0

800 2379 0

800 3026 0

800 2116 0

800 329 0

800 2788 0

800 294 0

801 96 1

801 135 1

801 72 1

801 73 1

801 74 1

801 75 1

801 77 1

801 79 1

801 81 1

801 87 1

801 1423 0

801 3335 0

801 317 0

801 138 0

801 758 0

801 2216 0

801 2133 0

801 781 0

801 2906 0

801 885 0

802 835 1

802 37 1

802 1739 1

802 332 1

802 109 1

802 334 1

802 460 1

802 528 1

802 2027 1

802 1085 1

802 1236 1

802 1427 1

802 118 1

802 1144 1

802 857 1

802 413 1

802 1118 1

802 676 0

802 1107 0

802 73 0

802 438 0

802 808 0

802 2541 0

802 1709 0

802 216 0

802 2381 0

802 1258 0

802 2583 0

802 1940 0

802 2500 0

802 99 0

802 1161 0

802 62 0

802 3407 0

803 130 1

803 1414 1

803 135 1

803 171 1

803 267 1

803 363 1

803 364 1

803 79 1

803 118 1

803 87 1

803 184 1

803 185 1

803 1403 1

803 3431 0

803 3621 0

803 1534 0

803 2657 0

803 3686 0

803 3105 0

803 435 0

803 235 0

803 1597 0

803 957 0

803 3038 0

803 3384 0

803 1710 0

804 932 1

804 2508 1

804 2509 1

804 141 1

804 2510 1

804 2511 1

804 2512 1

804 2513 1

804 2514 1

804 2515 1

804 277 1

804 2516 1

804 2517 1

804 2711 0

804 3278 0

804 3641 0

804 460 0

804 2597 0

804 1860 0

804 1161 0

804 2746 0

804 797 0

804 1224 0

804 486 0

804 3744 0

804 2902 0

805 1800 1

805 346 1

805 2518 1

805 1022 1

805 1959 0

805 1682 0

805 2987 0

805 1239 0

806 130 1

806 263 1

806 73 1

806 77 1

806 206 1

806 79 1

806 374 1

806 87 1

806 184 1

806 185 1

806 2659 0

806 626 0

806 2961 0

806 1954 0

806 226 0

806 1536 0

806 1459 0

806 2241 0

806 1927 0

806 1395 0

807 102 1

807 105 1

807 467 1

807 52 1

807 1172 1

807 1523 1

807 1180 1

807 61 1

807 863 1

807 2025 0

807 2627 0

807 2142 0

807 24 0

807 381 0

807 1211 0

807 2280 0

807 833 0

807 1220 0

808 98 1

808 101 1

808 435 1

808 1011 1

808 2003 1

808 2520 1

808 2521 1

808 506 1

808 2126 0

808 2629 0

808 2293 0

808 2917 0

808 1357 0

808 1025 0

808 1910 0

808 83 0

809 134 1

809 135 1

809 74 1

809 171 1

809 363 1

809 77 1

809 79 1

809 81 1

809 115 1

809 87 1

809 2746 0

809 1852 0

809 1297 0

809 3665 0

809 3767	0

809 1236	0

809 1733	0

809 1111	0

809 1528	0

809 1326	0

810 135 1

810 74	1

810 77	1

810 79	1

810 80	1

810 919 1

810 951 1

810 115 1

810 405 1

810 87	1

810 127 1

810 2293	0

810 1630	0

810 386 0

810 120 0

810 788 0

810 2481	0

810 2856	0

810 239 0

810 1806	0

810 573 0

810 680 0

811 96	1

811 66	1

811 36 1

811 134 1

811 841 1

811 109 1

811 47 1

811 560 1

811 118 1

811 55 1

811 2523 1

811 126 1

811 2073 0

811 1247 0

811 0 0

811 451 0

811 2293 0

811 487 0

811 1443 0

811 238 0

811 1275 0

811 260 0

811 801 0

811 1487 0

812 928 1

812 2525 1

812 34 1

812 930 1

812 36 1

812 42 1

812 747 1

812 2060 1

812 109 1

812 1902 1

812 1615 1

812 531 1

812 2524 1

812 285 1

812 940 0

812 809 0

812 568 0

812 3399 0

812 786 0

812 2672 0

812 3064 0

812 2390 0

812 1172 0

812 1662 0

812 559 0

812 307 0

812 71 0

812 1186 0

813 261 1

813 35 1

813 37 1

813 38 1

813 43 1

813 558 1

813 1082 1

813 192 1

813 478 1

813 2526 1

813 2527 1

813 1122 1

813 100 1

813 356 1

813 1515 1

813 1518 1

813 118 1

813 253 1

813 126 1

813 2776 0

813 1708 0

813 1798 0

813 3712 0

813 3439 0

813 3756 0

813 3114 0

813 2120 0

813 1979 0

813 2596 0

813 1552 0

813 119 0

813 493 0

813 104 0

813 3051 0

813 1803 0

813 134 0

813 3776 0

813 2429 0

814 512 1

814 2528 1

814 163 1

814 36 1

814 2463 1

814 105 1

814 1663 1

814 2004 1

814 53 1

814 221 1

814 511 1

814 1818 0

814 3175 0

814 1566 0

814 395 0

814 1062 0

814 840 0

814 2338 0

814 2517 0

814 2209 0

814 1156 0

814 2080 0

815 754 1

815 1748 1

815 36 1

815 910 0

815 2958 0

815 1201 0

816 196 1

816 329 1

816 1580 1

816 657 1

816 307 1

816 1268 1

816 439 1

816 123 1

816 285 1

816 446 1

816 3278 0

816 137 0

816 882 0

816 2461 0

816 3354 0

816 2145 0

816 2966 0

816 1198 0

816 3646 0

816 2026 0

817 1312 1

817 129 1

817 2530 1

817 2438 1

817 1449 1

817 46 1

817 80 1

817 119 1

817 2267 1

817 2268 1

817 445 1

817 1935 0

817 1617 0

817 229 0

817 97 0

817 2982 0

817 1849 0

817 2970 0

817 1993 0

817 1273 0

817 1473 0

817 2112 0

818 34 1

818 3 1

818 36 1

818 740 1

818 1477 1

818 2533 1

818 362 1

818 523 1

818 2317 1

818 526 1

818 529 1

818 1362 1

818 788 1

818 119 1

818 504 0

818 1611 0

818 631 0

818 1537 0

818 471 0

818 2796 0

818 3659 0

818 2582 0

818 843 0

818 1595 0

818 2010 0

818 3748 0

818 884 0

818 2281 0

819 100 1

819 105 1

819 844 1

819 302 1

819 1136 1

819 21 1

819 55 1

819 155 1

819 735 1

819 1410 0

819 2214 0

819 2241 0

819 968 0

819 751 0

819 2235 0

819 1307 0

819 2941 0

819 3639 0

820 289 1

820 34 1

820 483 1

820 36 1

820 165 1

820 325 1

820 1217 1

820 40 1

820 2404 1

820 42 1

820 2534 1

820 2535 1

820 2536 1

820 462 1

820 1970 1

820 285 1

820 3697 0

820 2269 0

820 2649 0

820 1713 0

820 2917 0

820 1283 0

820 1117 0

820 163 0

820 3724 0

820 1874 0

820 3407 0

820 2818 0

820 1486 0

820 3519 0

820 1541 0

820 2249 0

821 132 1

821 74 1

821 237 1

821 80 1

821 81 1
821 87 1
821 24 1
821 127 1
821 31 1
821 1092 0
821 1634 0
821 2585 0
821 1107 0
821 1851 0
821 321 0
821 2712 0
821 2699 0
821 1675 0
822 2401 1
822 301 1
822 78 1
822 431 1
822 2448 1
822 305 1
822 1589 1
822 1621 1
822 1499 1
822 3777 0
822 1383 0
822 1059 0
822 917 0
822 194 0
822 1983 0
822 218 0

822 3569 0

822 345 0

823 1513 1

823 2537 1

823 1419 1

823 758 1

823 2462 1

823 2946 0

823 1420 0

823 2194 0

823 1820 0

823 2232 0

824 738 1

824 1123 1

824 1445 1

824 969 1

824 1290 1

824 2538 1

824 209 1

824 242 1

824 210 1

824 211 1

824 657 1

824 1554 1

824 1974 0

824 560 0

824 1706 0

824 2724 0

824 2357 0

824 3461 0

824 24 0

824 2087 0

824 977 0

824 1241 0

824 2130 0

824 3338 0

825 96 1

825 192 1

825 224 1

825 352 1

825 2539 1

825 1327 1

825 499 1

825 52 1

825 118 1

825 55 1

825 376 1

825 1278 1

825 474 1

825 124 1

825 253 1

825 126 1

825 2902 0

825 2406 0

825 1354 0

825 3603 0

825 3710 0

825 612 0

825 1947 0

825 3780 0

825 680 0

825 634 0

825 135 0

825 2374 0

825 2500 0

825 3711 0

825 2780 0

825 2860 0

826 102 1

826 2470 1

826 74 1

826 267 1

826 118 1

826 87 1

826 376 1

826 863 1

826 127 1

826 3491 0

826 2301 0

826 1763 0

826 1084 0

826 924 0

826 692 0

826 1776 0

826 2918 0

826 2671 0

827 133 1

827 134 1

827 871 1

827 267 1

827 2540 1

827 494 1

827 79 1

827 80 1

827 431 1

827 560 1

827 1364 1

827 87 1

827 42 0

827 3346 0

827 2767 0

827 1728 0

827 3065 0

827 645 0

827 327 0

827 2204 0

827 3002 0

827 2644 0

827 3451 0

827 2857 0

828 608 1

828 36 1

828 2541 1

828 141 1

828 2429 1

828 1572 0

828 956 0

828 1542 0

828 2538 0

828 3063 0

829 4 1

829 19 1

829 931 1

829 804 1

829 1315 1

829 1604 1

829 1608 1

829 329 1

829 332 1

829 973 1

829 334 1

829 857 1

829 860 1

829 1118 1

829 2542 1

829 2543 1

829 2544 1

829 2545 1

829 126 1

829 3366 0

829 174 0

829 1489 0

829 947 0

829 217 0

829 2878 0

829 1580 0

829 364 0

829 1076 0

829 2752 0

829 237 0

829 982 0

829 859 0

829 2184 0

829 2873 0

829 159 0

829 587 0

829 3768 0

829 2312 0

830 96 1

830 771 1

830 102 1

830 78 1

830 47 1

830 915 1

830 118 1

830 694 1

830 726 1

830 446 1

830 2360 0

830 2313 0

830 2233 0

830 1311 0

830 2908 0

830 2247 0

830 1297 0

830 1985 0

830 1694 0

830 1354 0

831 66 1

831 132 1

831 2084 1

831 2054 1

831 135 1

831 72 1

831 73 1

831 137 1

831 75 1

831 237 1

831 80 1

831 81 1

831 1201 1

831 1202 1

831 2546 1

831 2547 1

831 22 1

831 87 1

831 1009 0

831 1954 0

831 2042 0

831 3131 0

831 3146 0

831 2043 0

831 3533 0

831 3611 0

831 854 0

831 785 0

831 2753 0

831 1172 0

831 2598 0

831 1737 0

831 3815 0

831 3140 0

831 2895 0

831 3809 0

832 1156 1

832 261 1

832 73 1

832 74 1

832 78 1

832 80 1

832 178 1

832 2548 1

832 117 1

832 87 1

832 538 1

832 253 1

832 382 1

832 1011 0

832 965 0

832 3179 0

832 2747 0

832 1254 0

832 494 0

832 268 0

832 2854 0

832 1919 0

832 1007 0

832 396 0

832 3149 0

832 1617 0

833 545 1

833 36 1

833 996 1

833 2358 1

833 188 1

833 1799 0

833 116 0

833 1605 0

833 3241 0

833 2819 0

834 96 1

834 764 1

834 37 1

834 293 1

834 391 1

834 109 1

834 46 1

834 52 1

834 118 1

834 119 1

834 286 1

834 221 1

834 190 1

834 127 1

834 2112 0

834 1825 0

834 3828 0

834 858 0

834 3842 0

834 2904 0

834 3699 0

834 231 0

834 487 0

834 1661 0

834 3287 0

834 1557 0

834 371 0

834 2499 0

835 1698 1

835 1123 1

835 37 1

835 5 1

835 42 1

835 333 1

835 14 1

835 655 1

835 973 1

835 17 1

835 242 1

835 211 1

835 24 1

835 3540 0

835 3145 0

835 3342 0

835 2447 0

835 1296 0

835 3470 0

835 253 0

835 2057 0

835 2222 0

835 3710 0

835 2219 0

835 3529 0

835 228 0

836 325 1

836 358 1

836 1254 1

836 776 1

836 46 1

836 114 1

836 52 1

836 2549 1

836 2550 1

836 1016 1

836 219 1

836 1102 0

836 3492 0

836 2188 0

836 3745 0

836 2627 0

836 2325 0

836 3691 0

836 967 0

836 3512 0

836 2338 0

836 3212 0

837 1154 1

837 75 1

837 79 1

837 399 1

837 115 1

837 87 1

837 184 1

837 185 1

837 2999 0

837 463 0

837 602 0

837 2899 0

837 435 0

837 1856 0

837 656 0

837 3490 0

838 388 1

838 105 1

838 109 1

838 110 1

838 1327 1

838 114 1

838 210 1

838 882 1

838 469 1

838 2197 1

838 1438 1

838 799 1

838 2268 0

838 3346 0

838 207 0

838 399 0

838 1501 0

838 964 0

838 186 0

838 3364 0

838 2681 0

838 2667 0

838 2106 0

838 3400 0

839 1536 1

839 1923 1

839 132 1

839 165 1

839 197 1

839 1092 1

839 41 1

839 107 1

839 460 1

839 46 1

839 465 1

839 22 1

839 57 1

839 187 1

839 956 1

839 1906 0

839 1046 0

839 2884 0

839 2357 0

839 3025 0

839 2026 0

839 1162 0

839 2607 0

839 538 0

839 3643 0

839 1407 0

839 1812 0

839 2061 0

839 860 0

839 1315 0

840 96 1

840 102 1

840 871 1

840 776 1

840 427 1

840 589 1

840 302 1

840 47 1

840 304 1

840 494 1

840 216 1

840 1430 0

840 1672 0

840 3534 0

840 2813 0

840 3638 0

840 1214 0

840 459 0

840 1592 0

840 1701 0

840 2915 0

840 1986 0

841 70 1

841 392 1

841 2508 1
841 2285 1
841 590 1
841 2556 1
841 912 1
841 306 1
841 1022 1
841 2552 1
841 2553 1
841 2554 1
841 2555 1
841 1212 1
841 510 1
841 3389 0
841 1469 0
841 2184 0
841 3435 0
841 1002 0
841 3005 0
841 910 0
841 2940 0
841 3037 0
841 1668 0
841 1173 0
841 340 0
841 223 0
841 0 0
841 485 0
842 32 1
842 2558 1

842 1415 1
842 40 1
842 456 1
842 1804 1
842 2447 1
842 1136 1
842 883 1
842 21 1
842 22 1
842 854 1
842 57 1
842 474 1
842 1851 1
842 2557 1
842 478 1
842 223 1
842 1581 0
842 1711 0
842 2772 0
842 3228 0
842 2282 0
842 3336 0
842 1372 0
842 1016 0
842 1161 0
842 1536 0
842 763 0
842 1633 0
842 1154 0
842 3112 0

842 88 0

842 3517 0

842 495 0

842 2599 0

843 2560 1

843 2561 1

843 386 1

843 2562 1

843 2563 1

843 2564 1

843 2565 1

843 2566 1

843 2313 1

843 1579 1

843 397 1

843 58 1

843 3777 0

843 2541 0

843 2568 0

843 1284 0

843 1485 0

843 2058 0

843 3507 0

843 2704 0

843 3554 0

843 1099 0

843 2583 0

843 1342 0

844 1345 1

844 1346 1

844 1347 1

844 2276 1

844 550 1

844 202 1

844 1547 1

844 45 1

844 527 1

844 278 1

844 1337 1

844 286 1

844 780 0

844 466 0

844 64 0

844 2418 0

844 1660 0

844 3262 0

844 1991 0

844 544 0

844 556 0

844 3761 0

844 651 0

844 1170 0

845 356 1

845 1700 1

845 4 1

845 1604 1

845 2567 1

845 2568 1

845 2569 1

845 2570 1

845 2571 1

845 2572 1

845 2573 1

845 2574 1

845 2575 1

845 887 1

845 2330 1

845 1366 0

845 1336 0

845 2431 0

845 3810 0

845 3347 0

845 1329 0

845 3552 0

845 2026 0

845 314 0

845 1367 0

845 427 0

845 2466 0

845 789 0

845 3734 0

845 1025 0

846 1607 1

846 588 1

846 47 1

846 2576 1

846 210 1

846 242 1

846 52 1

846 1593 1

846 443 1

846 1020 1

846 2015 1

846 1000 0

846 828 0

846 3316 0

846 134 0

846 3233 0

846 1611 0

846 2960 0

846 3532 0

846 73 0

846 808 0

846 224 0

847 517 1

847 519 1

847 2577 1

847 2578 1

847 2579 1

847 2452 1

847 2580 1

847 2581 1

847 155 1

847 156 1

847 314 1

847 448 1

847 196 1

847 198 1

847 582 1

847 460 1

847 727 1

847 472 1

847 729 1

847 732 1

847 486 1

847 930 0

847 1193 0

847 490 0

847 2572 0

847 670 0

847 3445 0

847 2648 0

847 2260 0

847 3596 0

847 0 0

847 1086 0

847 2980 0

847 2396 0

847 2477 0

847 2002 0

847 2930 0

847 983 0

847 3065 0

847 498 0

847 120 0

847 601 0

848 1984 1

848 261 1

848 2309 1

848 2535 1

848 41 1

848 80 1

848 242 1

848 2582 1

848 2583 1

848 2584 1

848 154 1

848 1789 1

848 126 1

848 665 0

848 320 0

848 3674 0

848 2834 0

848 3788 0

848 2890 0

848 729 0

848 2189 0

848 3021 0

848 2659 0

848 2004 0

848 1508 0

848 2555 0

849 2147 1

849 772 1

849 171 1

849 184 1

849 185 1

849 538 1

849 380 1

849 3085 0

849 2373 0
849 3248 0
849 912 0
849 2735 0
849 1138 0
849 2924 0
850 1282 1
850 325 1
850 362 1
850 394 1
850 395 1
850 346 1
850 1514 1
850 1822 1
850 1722 1
850 218 1
850 1307 1
850 766 1
850 2074 0
850 1207 0
850 976 0
850 739 0
850 2414 0
850 1448 0
850 3149 0
850 3224 0
850 91 0
850 1559 0
850 1212 0
850 358 0

851 263 1

851 1069 1

851 558 1

851 276 1

851 1750 1

851 254 1

851 2585 1

851 956 1

851 542 1

851 2990 0

851 2195 0

851 161 0

851 1343 0

851 2516 0

851 3417 0

851 1675 0

851 1459 0

851 2025 0

852 764 1

852 293 1

852 105 1

852 107 1

852 46 1

852 1174 1

852 55 1

852 2586 1

852 2587 1

852 252 1

852 190 1

852 1183 1

852 594 0
852 1692 0
852 1252 0
852 1691 0
852 1842 0
852 1719 0
852 2369 0
852 3281 0
852 1136 0
852 3546 0
852 2120 0
852 2768 0
853 36 1
853 141 1
853 46 1
853 766 1
853 878 1
853 657 1
853 1168 1
853 670 1
853 1324 0
853 1221 0
853 2303 0
853 1005 0
853 1449 0
853 2891 0
853 220 0
853 477 0
854 0 1
854 321 1

854 2589 1

854 4 1

854 6 1

854 358 1

854 329 1

854 13 1

854 334 1

854 337 1

854 1267 1

854 342 1

854 1270 1

854 1272 1

854 2230 1

854 124 1

854 2588 1

854 637 0

854 338 0

854 3640 0

854 526 0

854 2074 0

854 2159 0

854 767 0

854 782 0

854 1569 0

854 2054 0

854 2027 0

854 2651 0

854 1622 0

854 1506 0

854 659 0

854 3465 0

854 947 0

855 517 1

855 519 1

855 2579 1

855 155 1

855 156 1

855 2590 1

855 2591 1

855 2592 1

855 2593 1

855 2594 1

855 314 1

855 574 1

855 196 1

855 198 1

855 582 1

855 460 1

855 727 1

855 728 1

855 729 1

855 732 1

855 2281 1

855 508 1

855 509 1

855 3186 0

855 765 0

855 1606 0

855 1774 0

855 457 0

855 750 0

855 997 0

855 3666 0

855 2973 0

855 133 0

855 2013 0

855 1544 0

855 2415 0

855 3111 0

855 1551 0

855 3112 0

855 725 0

855 1414 0

855 173 0

855 3768 0

855 2651 0

855 3084 0

855 2705 0

856 96 1

856 1760 1

856 2595 1

856 841 1

856 74 1

856 77 1

856 2162 1

856 2163 1

856 254 1

856 376 1

856 1243 1

856 126 1

856 518 0

856 1144 0

856 1779 0

856 65 0

856 2946 0

856 1727 0

856 1602 0

856 2882 0

856 1559 0

856 2395 0

856 3402 0

856 1043 0

857 836 1

857 1445 1

857 714 1

857 970 1

857 77 1

857 47 1

857 303 1

857 212 1

857 118 1

857 215 1

857 989 1

857 1042 0

857 3355 0

857 1333 0

857 440 0

857 3648 0

857 3102 0

857 1392 0

857 661 0

857 2884 0

857 113 0

857 272 0

858 354 1

858 388 1

858 389 1

858 1541 1

858 2596 1

858 2597 1

858 2347 1

858 2254 1

858 2103 1

858 55 1

858 696 1

858 221 1

858 2669 0

858 2513 0

858 149 0

858 2772 0

858 410 0

858 2548 0

858 61 0

858 3569 0

858 2110 0

858 3663 0

858 2297 0

858 862 0

859 353 1

859 708 1

859 2598 1

859 2599 1

859 2600 1

859 1385 1

859 2601 1

859 76 1

859 47 1

859 1911 1

859 2064 1

859 54 1

859 55 1

859 1020 1

859 3353 0

859 3785 0

859 1138 0

859 3060 0

859 945 0

859 2088 0

859 2590 0

859 345 0

859 3420 0

859 1245 0

859 2171 0

859 2692 0

859 220 0

859 2216 0

860 2562 1

860 131 1

860 135 1

860 2602 1

860 2603 1
860 919 1
860 127 1
860 1857 0
860 2116 0
860 1709 0
860 2139 0
860 1361 0
860 3238 0
860 3816 0
861 128 1
861 353 1
861 36 1
861 2469 1
861 102 1
861 105 1
861 906 1
861 523 1
861 1034 1
861 2604 1
861 46 1
861 52 1
861 54 1
861 318 1
861 2096 0
861 860 0
861 1887 0
861 1493 0
861 3661 0
861 3318 0

861 1069 0
861 1175 0
861 2114 0
861 667 0
861 3099 0
861 194 0
861 1364 0
861 600 0
862 96 1
862 2401 1
862 102 1
862 267 1
862 301 1
862 78 1
862 302 1
862 303 1
862 305 1
862 1261 1
862 1523 1
862 2048 0
862 1828 0
862 894 0
862 1902 0
862 1113 0
862 1683 0
862 3694 0
862 824 0
862 1715 0
862 1445 0
862 224 0

863 34 1

863 163 1

863 1543 1

863 1128 1

863 297 1

863 1003 1

863 1389 1

863 2606 1

863 1039 1

863 1615 1

863 2607 1

863 946 1

863 2451 1

863 2608 1

863 2609 1

863 1437 1

863 1438 1

863 738 0

863 3665 0

863 1653 0

863 1322 0

863 804 0

863 1802 0

863 3748 0

863 519 0

863 1743 0

863 3839 0

863 2385 0

863 2859 0

863 2530 0

863 3625 0

863 3270 0

863 1021 0

863 82 0

864 776 1

864 713 1

864 589 1

864 302 1

864 303 1

864 2002 1

864 280 1

864 2233 1

864 1119 1

864 561 0

864 649 0

864 2672 0

864 298 0

864 652 0

864 1453 0

864 1756 0

864 2834 0

864 1775 0

865 480 1

865 36 1

865 805 1

865 7 1

865 2222 1

865 241 1

865 273 1

865 68 0

865 1909 0

865 1934 0

865 3139 0

865 2396 0

865 2408 0

865 53 0

866 448 1

866 100 1

866 329 1

866 330 1

866 331 1

866 2505 1

866 335 1

866 337 1

866 2610 1

866 2611 1

866 2612 1

866 438 1

866 1502 1

866 2736 0

866 2762 0

866 2295 0

866 2791 0

866 731 0

866 1770 0

866 1691 0

866 2519 0

866 217 0

866 2485 0

866 1058 0

866 860 0

866 137 0

867 133 1

867 391 1

867 235 1

867 46 1

867 542 1

867 127 1

867 285 0

867 3208 0

867 3504 0

867 1542 0

867 2901 0

867 1586 0

868 163 1

868 295 1

868 267 1

868 78 1

868 46 1

868 55 1

868 863 1

868 342 0

868 2792 0

868 371 0

868 3070 0

868 3561 0

868 3032 0

868 1908 0

869 227 1

869 132 1

869 331 1

869 332 1

869 13 1

869 1138 1

869 275 1

869 2613 1

869 887 1

869 956 1

869 445 1

869 1512 0

869 863 0

869 2449 0

869 2450 0

869 2203 0

869 3043 0

869 1019 0

869 3458 0

869 845 0

869 225 0

869 1784 0

870 1280 1

870 897 1

870 484 1

870 2133 1

870 2614 1

870 2615 1

870 93 1

870 190 1

870 1564 0

870 3715 0

870 2318 0

870 139 0

870 28 0

870 2375 0

870 1470 0

870 2053 0

871 2051 1

871 1957 1

871 70 1

871 2622 1

871 522 1

871 1625 1

871 1623 1

871 1624 1

871 601 1

871 1306 1

871 2619 1

871 1628 1

871 2621 1

871 2620 1

871 2305 0

871 40 0

871 841 0

871 1261 0

871 3110 0

871 3179 0

871 3820 0

871 2407 0

871 3598 0

871 149 0

871 1682 0

871 3751 0

871 599 0

871 2804 0

872 772 1

872 713 1

872 2476 1

872 77 1

872 2285 1

872 2623 1

872 1556 1

872 58 1

872 2075 1

872 2076 1

872 1439 1

872 3165 0

872 487 0

872 3680 0

872 2953 0

872 495 0

872 2767 0

872 666 0

872 3819 0

872 1368 0

872 2988 0

872 2942 0

873 2624 1

873 354 1

873 1406 1

873 551 1

873 2254 1
873 498 1
873 499 1
873 500 1
873 22 1
873 254 1
873 223 1
873 2454 0
873 1870 0
873 1399 0
873 2584 0
873 2654 0
873 1986 0
873 42 0
873 3843 0
873 2769 0
873 1045 0
873 3248 0
874 96 1
874 2625 1
874 100 1
874 1156 1
874 422 1
874 1414 1
874 136 1
874 77 1
874 80 1
874 178 1
874 404 1
874 2328 1

874 253 1

874 2356 0

874 1421 0

874 3796 0

874 3547 0

874 884 0

874 1094 0

874 2604 0

874 990 0

874 2166 0

874 802 0

874 2460 0

874 3698 0

874 2764 0

875 2626 1

875 2627 1

875 2628 1

875 838 1

875 297 1

875 106 1

875 235 1

875 2478 1

875 48 1

875 1587 1

875 511 1

875 1316 0

875 3131 0

875 441 0

875 784 0

875 3764 0

875 975 0

875 2620 0

875 381 0

875 3636 0

875 1973 0

875 2920 0

876 160 1

876 36 1

876 2629 1

876 2630 1

876 2631 1

876 2632 1

876 2633 1

876 362 1

876 1362 1

876 2430 1

876 3285 0

876 1206 0

876 813 0

876 3601 0

876 3047 0

876 1434 0

876 1361 0

876 1601 0

876 1971 0

876 3845 0

877 2634 1

877 2635 1

877 2636 1

877 1549 1

877 2414 1

877 499 1

877 507 1

877 1821 1

877 2089 0

877 19 0

877 2402 0

877 3826 0

877 2976 0

877 422 0

877 1398 0

877 1753 0

878 66 1

878 36 1

878 548 1

878 102 1

878 105 1

878 267 1

878 46 1

878 47 1

878 1589 1

878 118 1

878 55 1

878 838 0

878 3184 0

878 1576 0

878 168 0

878 8 0

878 3285 0

878 2502 0

878 960 0

878 2358 0

878 1301 0

878 440 0

879 79 1

879 87 1

879 185 1

879 538 1

879 127 1

879 3072 0

879 3469 0

879 3791 0

879 2775 0

879 1683 0

880 1018 1

880 135 1

880 105 1

880 74 1

880 77 1

880 80 1

880 55 1

880 117 1

880 87 1

880 538 1

880 923 1

880 3719 0

880 3115 0

880 1480 0

880 2391 0

880 1841 0

880 1326 0

880 2262 0

880 608 0

880 2127 0

880 144 0

880 463 0

881 518 1

881 1130 1

881 2637 1

881 210 1

881 1523 1

881 1016 1

881 219 1

881 124 1

881 1023 1

881 3613 0

881 1684 0

881 835 0

881 2367 0

881 2752 0

881 3801 0

881 1383 0

881 2196 0

881 2300 0

882 1922 1

882 163 1

882 516 1

882 105 1

882 1545 1

882 107 1

882 2638 1

882 111 1

882 2639 1

882 2640 1

882 24 1

882 187 1

882 186 0

882 3256 0

882 3680 0

882 445 0

882 1669 0

882 1145 0

882 61 0

882 2555 0

882 2895 0

882 2583 0

882 1200 0

882 297 0

883 673 1

883 36 1

883 134 1

883 39 1

883 46 1

883 1143 1

883 406 1

883 863 1

883 187 1

883 254 1

883 63 1

883 2828 0

883 944 0

883 3102 0

883 942 0

883 847 0

883 2120 0

883 3030 0

883 3039 0

883 420 0

883 1294 0

883 2629 0

884 96 1

884 102 1

884 267 1

884 47 1

884 560 1

884 2641 1

884 1523 1

884 52 1

884 118 1

884 2074 1

884 188 1

884 478 0

884 933 0

884 1245 0

884 3623 0

884 952 0

884 2898 0

884 2257 0

884 407 0

884 641 0

884 3481 0

884 2979 0

885 1536 1

885 355 1

885 36 1

885 197 1

885 165 1

885 486 1

885 1514 1

885 1804 1

885 1105 1

885 2642 1

885 2643 1

885 1397 1

885 438 1

885 472 1

885 30 1

885 2757 0

885 3486 0

885 3581 0

885 2362 0

885 3787 0

885 466 0

885 1549 0

885 287 0

885 2251 0

885 2318 0

885 308 0

885 3284 0

885 1103 0

885 2292 0

885 6 0

886 96 1

886 133 1

886 102 1

886 74 1

886 47 1

886 859 1

886 254 1

886 37 0

886 263 0

886 1627 0

886 1294 0

886 1326 0

886 1267 0

886 2779 0

887 37 1

887 493 1

887 2646 1

887 2647 1

887 2493 1

887 1288 0

887 1523 0

887 263 0

887 19 0

887 1097 0

888 96 1

888 608 1

888 523 1

888 1777 1

888 52 1

888 53 1

888 1108 1

888 55 1

888 155 1

888 1662 1

888 223 1

888 138 0

888 996 0

888 3779 0

888 2692 0

888 561 0

888 487 0

888 2023 0

888 2503 0

888 1122 0

888 556 0

888 1920 0

889 704 1

889 289 1

889 34 1

889 35 1

889 928 1

889 165 1

889 329 1

889 42 1

889 459 1

889 1673 1

889 655 1

889 242 1

889 211 1

889 243 1

889 531 1

889 2648 1

889 285 1

889 158 1

889 1057 0

889 551 0

889 2269 0

889 2191 0

889 3238 0

889 3176 0

889 3061 0

889 3403 0

889 2808 0

889 2765 0

889 2523 0

889 1012 0

889 3249 0

889 1132 0

889 2750 0

889 327 0

889 3184 0

889 3755 0

890 96 1

890 133 1

890 681 1

890 1388 1

890 654 1

890 2158 1

890 2543 1
890 1710 0
890 675 0
890 1889 0
890 2944 0
890 2631 0
890 1009 0
890 1764 0
891 1728 1
891 195 1
891 200 1
891 2650 1
891 2649 1
891 1370 1
891 2651 1
891 1375 1
891 1683 0
891 456 0
891 3003 0
891 987 0
891 2230 0
891 1822 0
891 511 0
891 340 0
892 2653 1
892 1868 1
892 78 1
892 2655 1
892 1556 1
892 53 1

892 54 1

892 1175 1

892 1594 1

892 154 1

892 2652 1

892 2013 1

892 2654 1

892 511 1

892 79 0

892 359 0

892 2525 0

892 609 0

892 2883 0

892 3804 0

892 1178 0

892 3582 0

892 157 0

892 563 0

892 313 0

892 2563 0

892 141 0

892 2814 0

893 1312 1

893 2656 1

893 36 1

893 199 1

893 1516 1

893 1010 1

893 1044 1

893 790 1

893 727 1

893 1080 1

893 442 1

893 253 1

893 1246 1

893 559 0

893 381 0

893 2300 0

893 294 0

893 472 0

893 3090 0

893 546 0

893 3248 0

893 1504 0

893 3533 0

893 2844 0

893 2791 0

893 2856 0

894 96 1

894 2657 1

894 98 1

894 136 1

894 1258 1

894 77 1

894 80 1

894 372 1

894 404 1

894 118 1

894 374 1

894 376 1

894 2269 1
894 126 1
894 2707 0
894 1628 0
894 2418 0
894 1138 0
894 1371 0
894 2198 0
894 3574 0
894 3370 0
894 3814 0
894 2378 0
894 1622 0
894 1980 0
894 1567 0
894 1455 0
895 96 1
895 897 1
895 66 1
895 133 1
895 134 1
895 74 1
895 79 1
895 80 1
895 87 1
895 254 1
895 127 1
895 2519 0
895 1501 0
895 1883 0

895 3195 0

895 442 0

895 608 0

895 2935 0

895 2348 0

895 1184 0

895 152 0

895 106 0

896 96 1

896 102 1

896 714 1

896 589 1

896 78 1

896 47 1

896 2321 1

896 756 1

896 1589 1

896 3636 0

896 1206 0

896 2341 0

896 358 0

896 2319 0

896 1258 0

896 2948 0

896 1686 0

896 1214 0

897 288 1

897 2658 1

897 133 1

897 135 1

897 73 1

897 77 1

897 1325 1

897 79 1

897 919 1

897 374 1

897 87 1

897 377 1

897 382 1

897 127 1

897 2167 0

897 32 0

897 1967 0

897 1074 0

897 2307 0

897 3482 0

897 2342 0

897 821 0

897 2916 0

897 1964 0

897 2200 0

897 2924 0

897 462 0

897 963 0

898 2432 1

898 223 1

898 163 1

898 2659 1

898 295 1

898 456 1

898 105 1

898 1545 1

898 1325 1

898 1006 1

898 111 1

898 2334 1

898 159 1

898 3062 0

898 3772 0

898 1337 0

898 384 0

898 733 0

898 1725 0

898 3777 0

898 489 0

898 3150 0

898 2655 0

898 1427 0

898 2541 0

898 3693 0

899 865 1

899 1923 1

899 2660 1

899 293 1

899 1545 1

899 109 1

899 45 1

899 2066 1

899 275 1

899 1048 1

899 57 1

899 155 1

899 732 1

899 221 1

899 1822 0

899 3550 0

899 3707 0

899 3186 0

899 2817 0

899 151 0

899 1455 0

899 2354 0

899 1356 0

899 1438 0

899 1522 0

899 3833 0

899 349 0

899 770 0

900 96 1

900 34 1

900 354 1

900 36 1

900 41 1

900 107 1

900 180 0

900 89 0

900 3047 0

900 2355 0

900 3707 0

900 3064 0

901 708 1

901 102 1

901 2663 1

901 136 1

901 2664 1

901 1148 1

901 1150 1

901 1680 0

901 1497 0

901 2934 0

901 1544 0

901 1558 0

901 630 0

901 3605 0

902 450 1

902 1923 1

902 36 1

902 1220 1

902 2665 1

902 2666 1

902 2667 1

902 2668 1

902 2669 1

902 558 1

902 2670 1

902 275 1

902 1047 1

902 22 1

902 87 1

902 1878 1

902 1841 0
902 3673 0
902 2561 0
902 3239 0
902 1926 0
902 490 0
902 1970 0
902 2003 0
902 2124 0
902 3422 0
902 2973 0
902 1184 0
902 408 0
902 1637 0
902 717 0
902 3119 0
903 135 1
903 363 1
903 206 1
903 80 1
903 434 1
903 374 1
903 376 1
903 185 1
903 3796 0
903 1979 0
903 1686 0
903 844 0
903 3442 0
903 1571 0

903 1251	0
903 2063	0
904 2688	1
904 1942	1
904 160 1	
904 814 1	
904 2681	1
904 2671	1
904 2672	1
904 2673	1
904 2674	1
904 2675	1
904 2676	1
904 2677	1
904 1142	1
904 2679	1
904 2680	1
904 2678	1
904 2682	1
904 2683	1
904 2684	1
904 2685	1
904 2686	1
904 2687	1
904 2086	0
904 1246	0
904 2799	0
904 3440	0
904 2456	0
904 3533	0

904 2979 0

904 714 0

904 1466 0

904 3237 0

904 219 0

904 3025 0

904 1968 0

904 1818 0

904 300 0

904 3101 0

904 3673 0

904 1475 0

904 457 0

904 3295 0

904 800 0

904 295 0

905 128 1

905 512 1

905 2689 1

905 163 1

905 516 1

905 2690 1

905 109 1

905 48 1

905 1467 1

905 280 1

905 795 1

905 508 1

905 286 1

905 159 1

905 1681 0
905 838 0
905 836 0
905 1864 0
905 2924 0
905 1544 0
905 3841 0
905 1221 0
905 3049 0
905 927 0
905 3821 0
905 580 0
905 1750 0
905 1224 0
906 2691 1
906 2692 1
906 101 1
906 235 1
906 1421 1
906 47 1
906 566 1
906 93 1
906 1278 1
906 2263 0
906 1397 0
906 3795 0
906 2956 0
906 3309 0
906 3399 0
906 1310 0

906 233 0
906 3658 0
907 96 1
907 928 1
907 34 1
907 133 1
907 197 1
907 2693 1
907 42 1
907 298 1
907 2446 1
907 118 1
907 184 1
907 1049 1
907 442 1
907 286 1
907 1506 0
907 1553 0
907 3266 0
907 2962 0
907 1808 0
907 3328 0
907 2547 0
907 1654 0
907 161 0
907 1727 0
907 3716 0
907 596 0
907 3550 0
907 2112 0

908 1792 1
908 2696 1
908 267 1
908 951 1
908 184 1
908 377 1
908 126 1
908 575 1
908 1522 0
908 2989 0
908 1865 0
908 996 0
908 3300 0
908 2682 0
908 2477 0
908 2208 0
909 192 1
909 132 1
909 38 1
909 72 1
909 264 1
909 74 1
909 2697 1
909 77 1
909 237 1
909 238 1
909 80 1
909 81 1
909 1202 1
909 87 1

909 58 1

909 3432 0

909 2656 0

909 1603 0

909 2394 0

909 2957 0

909 2787 0

909 3146 0

909 3433 0

909 3298 0

909 1615 0

909 996 0

909 869 0

909 3124 0

909 70 0

909 1692 0

910 257 1

910 897 1

910 137 1

910 2698 1

910 2699 1

910 2700 1

910 237 1

910 2701 1

910 22 1

910 55 1

910 1660 1

910 1658 0

910 1693 0

910 2145 0

910 3803 0

910 900 0

910 2474 0

910 1081 0

910 3285 0

910 2970 0

910 1404 0

910 132 0

911 2304 1

911 617 1

911 1385 1

911 141 1

911 2702 1

911 2703 1

911 2704 1

911 2705 1

911 1650 1

911 1174 1

911 511 1

911 3661 0

911 1881 0

911 2627 0

911 2019 0

911 3740 0

911 254 0

911 3004 0

911 296 0

911 2922 0

911 3699 0

911 3564 0

912 512 1

912 136 1

912 522 1

912 2706 1

912 157 1

912 418 1

912 419 1

912 422 1

912 295 1

912 46 1

912 570 1

912 67 1

912 1617 1

912 735 1

912 2018 1

912 2425 1

912 507 1

912 508 1

912 510 1

912 2863 0

912 3430 0

912 684 0

912 2031 0

912 633 0

912 2064 0

912 2296 0

912 3694 0

912 2972 0

912 921 0

912 1058 0

912 3577 0

912 2825 0

912 3039 0

912 3005 0

912 783 0

912 3178 0

912 58 0

912 3054 0

913 2272 1

913 356 1

913 37 1

913 109 1

913 466 1

913 2707 1

913 1461 1

913 1526 1

913 56 1

913 61 1

913 674 0

913 2402 0

913 287 0

913 1208 0

913 1979 0

913 3223 0

913 542 0

913 3722 0

913 369 0

913 3493 0

914 672 1

914 674 1

914 2708 1

914 2709 1

914 2710 1

914 2711 1

914 2712 1

914 377 1

914 987 1

914 1353 0

914 1197 0

914 3394 0

914 1139 0

914 341 0

914 3019 0

914 3135 0

914 2398 0

914 1079 0

915 928 1

915 2017 1

915 2018 1

915 708 1

915 1672 1

915 1289 1

915 1471 1

915 1005 1

915 462 1

915 2013 1

915 1010 1

915 1012 1

915 438 1

915 157 1

915 511 1

915 416 0

915 2008 0

915 3349 0

915 864 0

915 3756 0

915 2748 0

915 3475 0

915 1453 0

915 3135 0

915 3802 0

915 2555 0

915 3004 0

915 2717 0

915 2888 0

915 3395 0

916 96 1

916 1122 1

916 102 1

916 105 1

916 332 1

916 47 1

916 118 1

916 1450 0

916 29 0

916 2538 0

916 148 0

916 1350 0

916 1952 0

916 1921 0

917 257 1

917 805 1

917 43 1

917 499 1

917 694 1

917 26 1

917 1385 0

917 359 0

917 3842 0

917 2983 0

917 845 0

917 1153 0

918 96 1

918 802 1

918 713 1

918 78 1

918 47 1

918 302 1

918 304 1

918 494 1

918 1395 1

918 406 1

918 91 1

918 126 1

918 1530 0

918 1274 0

918 3341 0

918 734 0

918 1852 0

918 513 0

918 3226 0

918 2853 0

918 1111 0

918 3545 0

918 16 0

918 3214 0

919 773 1

919 102 1

919 1319 1

919 47 1

919 304 1

919 1396 1

919 1589 1

919 118 1

919 55 1

919 766 1

919 549 0

919 45 0

919 2780 0

919 3465 0

919 1792 0

919 948 0

919 3648 0

919 266 0

919 2900 0

919 2039 0

920 128 1

920 80 1

920 374 1

920 376 1

920 185 1

920 1601 0

920 3537 0

920 2658 0

920 3758 0

920 3005 0

921 353 1

921 1122 1

921 998 1

921 74 1

921 878 1

921 1556 1

921 2714 1

921 2713 1

921 154 1

921 733 1

921 1406 1

921 159 1

921 2137 0

921 26 0

921 538 0

921 1496 0

921 1202 0

921 1936 0

921 3099 0

921 2941 0

921 2697 0

921 3440 0

921 3135 0

921 1553 0

922 165 1

922 102 1

922 615 1

922 106 1

922 906 1

922 716 1

922 878 1

922 48 1

922 52 1

922 53 1

922 920 1

922 154 1

922 2715 1

922 158 1

922 159 1

922 2231 0

922 2037 0

922 1200 0

922 2929 0

922 832 0

922 1173 0

922 1128 0

922 2730 0

922 2266 0

922 2589 0

922 3351 0

922 2179 0

922 1480 0

922 223 0

922 3643 0

923 407 1

923 66 1

923 98 1

923 135 1

923 72 1

923 167 1

923 74 1

923 75 1

923 238 1

923 79 1

923 80 1

923 919 1

923 87 1

923 377 1

923 489 0

923 1846 0

923 1780 0

923 1131 0

923 285 0

923 1222 0

923 1648 0

923 1080 0

923 1715 0

923 2421 0

923 3325 0

923 1562 0

923 1876 0

923 3096 0

924 36 1

924 1446 1

924 7 1

924 42 1

924 655 1

924 211 1

924 1076 1

924 216 1

924 2716 1

924 201 0

924 1489 0

924 3389 0

924 3653 0

924 2344 0

924 2882 0

924 1328 0

924 1267 0

924 258 0

925 704 1

925 613 1

925 102 1

925 105 1

925 906 1

925 1130 1

925 918 1

925 1180 1

925 863 1

925 2670 0

925 1243 0

925 2865 0

925 1620 0

925 1426 0

925 2301 0

925 2344 0

925 2536 0

925 2537 0

926 96 1

926 802 1

926 267 1

926 79 1

926 47 1

926 184 1

926 3276 0

926 1670 0

926 1106 0

926 1251 0

926 385 0

926 171 0

927 36 1

927 1767 1

927 109 1

927 141 1

927 112 1

927 52 1

927 55 1

927 223 1

927 232 0

927 2742 0

927 976 0

927 1022 0

927 2022 0

927 1404 0

927 1513 0

927 1229 0

928 1056 1

928 2472 1

928 41 1

928 301 1

928 47 1

928 1052 1

928 766 1

928 2117 0

928 2925 0

928 28 0

928 3621 0

928 3797 0

928 627 0

928 3238 0

929 385 1

929 131 1

929 230 1

929 74 1

929 237 1

929 206 1

929 52 1

929 118 1

929 87 1

929 88 1

929 698 1

929 442 1

929 863 1

929 918 1

929 127 1

929 1353 0

929 707 0

929 1174 0

929 1239 0

929 121 0

929 84 0

929 300 0

929 1702 0

929 3284 0

929 1164 0

929 3753 0

929 2066 0

929 3365 0

929 3626 0

929 1276 0

930 96 1

930 773 1

930 2717 1

930 135 1

930 493 1

930 78 1

930 915 1

930 118 1

930 1117 1

930 446 1

930 1277 0

930 3388 0

930 2332 0

930 1316 0

930 1193 0

930 714 0

930 2164 0

930 2228 0

930 3231 0

930 1270 0

931 608 1

931 36 1

931 1477 1

931 362 1

931 2718 1

931 46 1

931 52 1

931 1140 1

931 1812 1

931 60 1

931 1662 1

931 2088 0

931 2270 0

931 860 0

931 3305 0

931 3153 0

931 1697 0

931 2118 0

931 904 0

931 1250 0

931 118 0

931 407 0

932 1315 1

932 804 1

932 9 1
932 655 1
932 209 1
932 657 1
932 378 1
932 477 1
932 254 1
932 691 0
932 3123 0
932 3640 0
932 930 0
932 2940 0
932 1027 0
932 906 0
932 2729 0
932 267 0
933 320 1
933 135 1
933 104 1
933 1228 1
933 77 1
933 1612 1
933 1202 1
933 115 1
933 58 1
933 3515 0
933 11 0
933 3068 0
933 2674 0
933 2184 0

933 3 0

933 202 0

933 219 0

933 2670 0

934 96 1

934 1505 1

934 841 1

934 47 1

934 2320 1

934 435 1

934 1395 1

934 118 1

934 438 1

934 442 1

934 1341 1

934 126 1

934 3510 0

934 132 0

934 893 0

934 1789 0

934 3500 0

934 2392 0

934 16 0

934 3819 0

934 836 0

934 2435 0

934 3506 0

934 3229 0

935 257 1

935 36 1

935 356 1

935 2661 1

935 2194 1

935 275 1

935 20 1

935 499 1

935 60 1

935 221 1

935 2719 1

935 3583 0

935 3494 0

935 2002 0

935 1703 0

935 3650 0

935 3257 0

935 22 0

935 939 0

935 3379 0

935 1512 0

935 2743 0

936 2720 1

936 2721 1

936 1027 1

936 1445 1

936 2120 1

936 522 1

936 2287 1

936 208 1

936 210 1

936 218 1

936 220 1

936 2360 0

936 1582 0

936 1868 0

936 1246 0

936 3231 0

936 457 0

936 3700 0

936 3712 0

936 40 0

936 3325 0

936 3636 0

937 2723 1

937 2724 1

937 2725 1

937 774 1

937 782 1

937 785 1

937 146 1

937 147 1

937 788 1

937 820 1

937 1942 1

937 1947 1

937 3770 0

937 3375 0

937 2627 0

937 472 0

937 1284 0

937 2916 0

937 2020 0

937 2321 0

937 2933 0

937 732 0

937 2473 0

937 3085 0

938 1889 1

938 66 1

938 2726 1

938 74 1

938 2635 1

938 1244 1

938 117 1

938 87 1

938 376 1

938 378 1

938 540 1

938 2133 0

938 1832 0

938 1115 0

938 1862 0

938 781 0

938 1900 0

938 1030 0

938 1732 0

938 3267 0

938 422 0

938 494 0

939 96 1

939 99 1

939 101 1

939 72 1

939 74 1

939 77 1

939 115 1

939 118 1

939 1623 1

939 376 1

939 538 1

939 1393 0

939 839 0

939 747 0

939 1729 0

939 1084 0

939 998 0

939 2857 0

939 1922 0

939 702 0

939 2389 0

939 945 0

940 388 1

940 165 1

940 1444 1

940 1543 1

940 2727 1

940 617 1

940 2728 1

940 883 1

940 20 1

940 277 1

940 22 1

940 884 1

940 1047 1

940 1109 1

940 2067 1

940 313 0

940 2613 0

940 3121 0

940 1435 0

940 3014 0

940 3226 0

940 832 0

940 1643 0

940 1187 0

940 1840 0

940 1127 0

940 204 0

940 3658 0

940 2209 0

940 1210 0

941 354 1

941 1668 1

941 1541 1

941 2729 1

941 2730 1

941 2347 1

941 2252 1

941 2731 1

941 2290 1

941 2103 1

941 221 1
941 2513 0
941 2750 0
941 2338 0
941 1416 0
941 3763 0
941 2281 0
941 2700 0
941 1505 0
941 1609 0
941 2170 0
941 3787 0
942 774 1
942 2732 1
942 2733 1
942 785 1
942 147 1
942 1140 1
942 788 1
942 1719 1
942 1777 0
942 2767 0
942 1786 0
942 1496 0
942 1396 0
942 634 0
942 1615 0
942 2878 0
943 257 1
943 1794 1

943 260 1

943 2180 1

943 2181 1

943 1896 1

943 2734 1

943 2735 1

943 498 1

943 499 1

943 500 1

943 25 1

943 28 1

943 1725 0

943 727 0

943 1002 0

943 595 0

943 3069 0

943 247 0

943 1325 0

943 1618 0

943 2513 0

943 802 0

943 3772 0

943 3815 0

943 2133 0

944 96 1

944 128 1

944 386 1

944 807 1

944 136 1

944 1579 1

944 494 1

944 80 1

944 376 1

944 1630 1

944 2316 0

944 1608 0

944 2675 0

944 1475 0

944 2629 0

944 825 0

944 1748 0

944 1940 0

944 2541 0

944 186 0

945 1184 1

945 133 1

945 102 1

945 776 1

945 652 1

945 302 1

945 47 1

945 210 1

945 242 1

945 118 1

945 1438 1

945 831 0

945 3142 0

945 1648 0

945 1257 0

945 2179 0

945 1413 0

945 396 0

945 2670 0

945 2744 0

945 1402 0

945 2087 0

946 96 1

946 47 1

946 2736 1

946 2737 1

946 2738 1

946 52 1

946 250 1

946 347 1

946 476 1

946 3081 0

946 1046 0

946 1779 0

946 2953 0

946 2391 0

946 3816 0

946 3044 0

946 2393 0

946 3358 0

947 2147 1

947 78 1

947 79 1

947 80 1

947 527 1

947 55 1

947 87 1

947 376 1

947 185 1

947 187 1

947 693 0

947 2591 0

947 933 0

947 1371 0

947 1161 0

947 3441 0

947 1075 0

947 811 0

947 3539 0

947 664 0

948 676 1

948 453 1

948 677 1

948 615 1

948 932 1

948 111 1

948 911 1

948 306 1

948 2740 1

948 2741 1

948 2742 1

948 2743 1

948 56 1

948 1957 0

948 2343 0

948 1069 0

948 2539 0

948 2505 0

948 2880 0

948 2037 0

948 47 0

948 425 0

948 343 0

948 3587 0

948 3529 0

948 2148 0

949 130 1

949 77 1

949 206 1

949 79 1

949 1200 1

949 561 1

949 374 1

949 87 1

949 184 1

949 185 1

949 538 1

949 922 0

949 3145 0

949 2827 0

949 498 0

949 2866 0

949 1864 0

949 128 0

949 3537 0

949 740 0

949 2963 0

949 1533 0

950 1514 1

950 54 1

950 1015 1

950 2746 1

950 2745 1

950 826 1

950 2747 1

950 2748 1

950 159 1

950 677 0

950 3118 0

950 3442 0

950 206 0

950 1719 0

950 3423 0

950 1263 0

950 942 0

950 2942 0

951 109 1

951 46 1

951 277 1

951 2455 1

951 1691 1

951 190 1

951 223 1

951 484 0

951 3769 0

951 3756 0

951 3689 0

951 140 0

951 878 0

951 2790 0

952 704 1

952 163 1

952 196 1

952 516 1

952 519 1

952 615 1

952 1416 1

952 1545 1

952 511 1

952 2750 1

952 111 1

952 1712 1

952 2751 1

952 1010 1

952 1528 1

952 958 1

952 159 1

952 1089 0

952 2012 0

952 2087 0

952 3779 0

952 2320 0

952 3412 0

952 3265 0

952 1543 0

952 1039 0

952 2734 0

952 152 0

952 2399 0

952 3829 0

952 2794 0

952 1085 0

952 1701 0

952 2488 0

953 2752 1

953 105 1

953 46 1

953 47 1

953 243 1

953 52 1

953 755 1

953 55 1

953 248 1

953 522 0

953 262 0

953 2698 0

953 951 0

953 2746 0

953 1349 0

953 3620 0

953 3518 0

953 2319 0

954 2753 1

954 2754 1

954 100 1

954 293 1

954 109 1

954 1389 1

954 882 1

954 946 1

954 118 1

954 55 1

954 1174 1

954 1850 1

954 187 1

954 3097 0

954 699 0

954 1302 0

954 3071 0

954 2137 0

954 2025 0

954 242 0

954 1374 0

954 1740 0

954 434 0

954 2279 0

954 1880 0

954 3349 0

955 1889 1

955 98 1

955 130 1

955 2435 1

955 133 1

955 361 1

955 1258 1

955 78 1

955 79 1
955 376 1
955 185 1
955 572 1
955 1917 1
955 2439 0
955 3 0
955 2235 0
955 1074 0
955 1306 0
955 355 0
955 2011 0
955 1874 0
955 730 0
955 1248 0
955 65 0
955 1351 0
955 1958 0
956 1923 1
956 516 1
956 137 1
956 1545 1
956 2452 1
956 926 1
956 1825 1
956 295 1
956 2476 1
956 438 1
956 57 1
956 187 1

956 61 1

956 451 1

956 2755 1

956 2505 1

956 460 1

956 462 1

956 2639 1

956 607 1

956 1136 1

956 1526 1

956 360 0

956 1099 0

956 321 0

956 3374 0

956 23 0

956 2501 0

956 722 0

956 366 0

956 2603 0

956 835 0

956 3402 0

956 2116 0

956 2537 0

956 3120 0

956 3378 0

956 1752 0

956 1647 0

956 1875 0

956 658 0

956 583 0

956 2710 0

956 712 0

957 480 1

957 1122 1

957 2756 1

957 134 1

957 493 1

957 1392 1

957 1524 1

957 917 1

957 1240 1

957 2072 1

957 2074 1

957 3186 0

957 1515 0

957 2716 0

957 772 0

957 3309 0

957 642 0

957 812 0

957 1646 0

957 2861 0

957 1487 0

957 2715 0

958 1186 1

958 171 1

958 364 1

958 363 1

958 365 1

958 184 1

958 185 1

958 859 1

958 2057 0

958 3689 0

958 1086 0

958 1334 0

958 2360 0

958 3483 0

958 14 0

958 986 0

959 832 1

959 66 1

959 135 1

959 74 1

959 43 1

959 171 1

959 47 1

959 372 1

959 377 1

959 91 1

959 126 1

959 1781 0

959 1529 0

959 3615 0

959 3612 0

959 1139 0

959 1729 0

959 258 0

959 2583 0

959 2384 0

959 804 0

959 2899 0

960 2757 1

960 73 1

960 377 1

960 78 1

960 80 1

960 81 1

960 560 1

960 1200 1

960 184 1

960 185 1

960 1732 0

960 3401 0

960 3319 0

960 47 0

960 932 0

960 1731 0

960 243 0

960 1412 0

960 3582 0

960 1565 0

961 908 1

961 1559 1

961 1689 1

961 411 1

961 35 1

961 36 1

961 165 1

961 42 1

961 52 1

961 2618 1

961 318 1

961 703 1

961 2758 1

961 1100 1

961 1502 1

961 97 1

961 228 1

961 1513 1

961 1901 1

961 878 1

961 508 1

961 2183 0

961 37 0

961 2031 0

961 3801 0

961 1953 0

961 3173 0

961 2426 0

961 79 0

961 1661 0

961 1124 0

961 1691 0

961 1731 0

961 894 0

961 2445 0

961 1659 0

961 1723 0

961 3825 0

961 1498 0

961 795 0

961 290 0

961 917 0

962 774 1

962 782 1

962 785 1

962 146 1

962 147 1

962 1944 1

962 2340 1

962 1832 1

962 41 1

962 52 1

962 820 1

962 1719 1

962 2759 1

962 2760 1

962 2761 1

962 2762 1

962 2763 1

962 2764 1

962 2765 1

962 2766 1

962 1777 1

962 403 0

962 495 0

962 2946 0

962 372 0

962 3091 0

962 508 0

962 2027 0

962 554 0

962 2836 0

962 3355 0

962 2040 0

962 2492 0

962 755 0

962 1615 0

962 2572 0

962 2494 0

962 2431 0

962 3216 0

962 2442 0

962 2530 0

962 3322 0

963 1664 1

963 939 1

963 2767 1

963 2768 1

963 52 1

963 21 1

963 854 1

963 695 1

963 2492 1

963 1662 1

963 495 0

963 835 0

963 2884 0

963 3638 0

963 3530 0

963 1475 0

963 931 0

963 1276 0

963 2840 0

963 2212 0

964 1799 1

964 295 1

964 2769 1

964 275 1

964 890 1

964 2047 1

964 863 1

964 1214 0

964 584 0

964 3184 0

964 3657 0

964 1759 0

964 19 0

964 278 0

965 2770 1

965 2771 1

965 36 1

965 191 1

965 3524 0

965 1029 0

965 1803 0

965 2111 0

966 96 1

966 477 1

966 261 1

966 75 1

966 957 1

966 80 1

966 2772 1

966 2773 1

966 22 1

966 87 1

966 2774 1

966 635 1

966 413 1

966 126 1

966 127 1

966 2673 0

966 2243 0

966 644 0

966 240 0

966 1739 0

966 3747 0

966 2312 0

966 59 0

966 3687 0

966 1593 0

966 400 0

966 3262 0

966 938 0

966 1277 0

966 2613 0

967 36 1

967 932 1

967 102 1

967 1254 1

967 105 1

967 13 1

967 46 1

967 941 1

967 560 1

967 945 1

967 1523 1

967 277 1

967 799 1

967 869 0

967 1853 0

967 227 0

967 1834 0

967 392 0

967 3734 0

967 1248 0

967 3675 0

967 3219 0

967 1540 0

967 792 0

967 1227 0

967 3403 0

968 738 1

968 102 1

968 2775 1

968 427 1

968 940 1

968 941 1

968 1261 1
968 47 1
968 1262 1
968 945 1
968 210 1
968 2512 1
968 52 1
968 2358 1
968 119 1
968 2776 1
968 219 1
968 1717 0
968 620 0
968 3144 0
968 740 0
968 2859 0
968 3284 0
968 1574 0
968 2441 0
968 2074 0
968 3008 0
968 1017 0
968 1113 0
968 2843 0
968 1902 0
968 722 0
968 1735 0
968 2876 0
969 261 1
969 134 1

969 74 1

969 1871 1

969 80 1

969 368 1

969 560 1

969 115 1

969 52 1

969 917 1

969 1523 1

969 87 1

969 2001 1

969 861 1

969 830 1

969 127 1

969 3401 0

969 2006 0

969 809 0

969 1347 0

969 3340 0

969 2318 0

969 3541 0

969 838 0

969 1215 0

969 2992 0

969 3147 0

969 632 0

969 1775 0

969 2774 0

969 3758 0

969 1138 0

970 102 1

970 46 1

970 47 1

970 210 1

970 118 1

970 55 1

970 920 1

970 2777 1

970 3322 0

970 2905 0

970 3705 0

970 1372 0

970 903 0

970 3177 0

970 2602 0

970 2455 0

971 98 1

971 102 1

971 2281 1

971 74 1

971 75 1

971 79 1

971 80 1

971 87 1

971 55 1

971 254 1

971 127 1

971 1308 0

971 1141 0

971 3184 0

971 593 0

971 1698 0

971 1647 0

971 1200 0

971 3764 0

971 179 0

971 3389 0

971 134 0

972 1061 1

972 134 1

972 72 1

972 79 1

972 87 1

972 377 1

972 2179 0

972 1703 0

972 2229 0

972 326 0

972 1158 0

972 322 0

973 2531 1

973 2780 1

973 1989 1

973 2674 1

973 54 1

973 2778 1

973 2779 1

973 60 1

973 2781 1

973 191 1

973 820 0

973 472 0

973 1492 0

973 2090 0

973 1771 0

973 127 0

973 231 0

973 1239 0

973 1441 0

973 1096 0

974 32 1

974 36 1

974 110 1

974 830 1

974 2161 1

974 499 1

974 469 1

974 221 1

974 190 1

974 799 1

974 3501 0

974 3702 0

974 2797 0

974 1020 0

974 399 0

974 2409 0

974 3505 0

974 1653 0

974 2674 0

974 875 0

975 2304	1

975 891 1

975 293 1

975 295 1

975 104 1

975 361 1

975 1896	1

975 1324	1

975 45	1

975 46	1

975 117 1

975 55	1

975 24	1

975 187 1

975 2782	1

975 863 1

975 321 0

975 952 0

975 1471	0

975 1065	0

975 2549	0

975 2918	0

975 697 0

975 115 0

975 2014	0

975 732 0

975 2807	0

975 260 0

975 2552	0

975 3766	0

975 867 0

975 220 0

976 2688 1

976 2784 1

976 2785 1

976 67 1

976 2786 1

976 133 1

976 422 1

976 774 1

976 2787 1

976 2788 1

976 2789 1

976 814 1

976 1295 1

976 2674 1

976 2677 1

976 157 1

976 2783 1

976 206 0

976 3015 0

976 1088 0

976 555 0

976 2381 0

976 2467 0

976 1637 0

976 1585 0

976 2834 0

976 647 0

976 179 0

976 3765 0

976 2633 0

976 2950 0

976 2300 0

976 3217 0

976 3717 0

977 243 1

977 36 1

977 2790 1

977 1054 0

977 2892 0

977 1716 0

978 1088 1

978 1091 1

978 902 1

978 235 1

978 332 1

978 494 1

978 47 1

978 944 1

978 1589 1

978 1174 1

978 858 1

978 2427 1

978 221 1

978 462 0

978 2590 0

978 3509 0

978 73 0

978 1318 0

978 2853	0

978 3536	0

978 2460	0

978 511 0

978 1635	0

978 3642	0

978 3155	0

978 2079	0

979 96	1

979 135 1

979 171 1

979 267 1

979 363 1

979 364 1

979 79	1

979 1231	1

979 87	1

979 184 1

979 185 1

979 188 1

979 1415	0

979 197 0

979 910 0

979 919 0

979 2666	0

979 959 0

979 3800	0

979 3083	0

979 130 0

979 327 0

979 1137 0

979 18 0

980 389 1

980 391 1

980 2791 1

980 105 1

980 46 1

980 47 1

980 1742 1

980 55 1

980 1880 1

980 572 1

980 765 1

980 2730 0

980 2789 0

980 3613 0

980 624 0

980 3247 0

980 2298 0

980 196 0

980 1233 0

980 1337 0

980 2982 0

980 367 0

981 67 1

981 2149 1

981 1385 1

981 110 1

981 46 1

981 950 1

981 1278 1
981 1678 0
981 1212 0
981 2363 0
981 1413 0
981 2461 0
981 2550 0
981 1298 0
982 385 1
982 1122 1
982 1218 1
982 2792 1
982 41 1
982 235 1
982 236 1
982 45 1
982 47 1
982 671 1
982 1590 1
982 951 1
982 920 1
982 95 1
982 587 0
982 2333 0
982 2570 0
982 2399 0
982 2579 0
982 975 0
982 303 0
982 2320 0

982 2951 0

982 972 0

982 3679 0

982 292 0

982 3517 0

982 1826 0

983 36 1

983 356 1

983 104 1

983 107 1

983 46 1

983 275 1

983 1139 1

983 24 1

983 984 0

983 1672 0

983 1530 0

983 395 0

983 507 0

983 1786 0

983 66 0

983 202 0

984 704 1

984 705 1

984 165 1

984 613 1

984 616 1

984 2793 1

984 2794 1

984 2795 1

984 1804 1

984 2796 1

984 462 1

984 2797 1

984 703 1

984 2743 1

984 154 1

984 158 1

984 159 1

984 2415 0

984 251 0

984 250 0

984 2377 0

984 1657 0

984 2671 0

984 1896 0

984 1811 0

984 1640 0

984 2814 0

984 2209 0

984 2658 0

984 2542 0

984 2489 0

984 3324 0

984 1155 0

984 1181 0

985 608 1

985 353 1

985 1786 1

985 2148 1

985 2214 1
985 2216 1
985 141 1
985 1198 1
985 2798 1
985 916 1
985 282 1
985 3706 0
985 1776 0
985 1045 0
985 1587 0
985 494 0
985 2488 0
985 1125 0
985 1969 0
985 2253 0
985 1940 0
985 1355 0
986 1220 1
986 105 1
986 107 1
986 46 1
986 112 1
986 55 1
986 2071 0
986 2240 0
986 420 0
986 3473 0
986 1017 0
986 1170 0

987 102 1

987 235 1

987 302 1

987 560 1

987 305 1

987 54 1

987 190 1

987 294 0

987 1428 0

987 2529 0

987 3024 0

987 1861 0

987 1308 0

987 2561 0

988 4 1

988 5 1

988 6 1

988 329 1

988 330 1

988 332 1

988 13 1

988 14 1

988 1422 1

988 273 1

988 498 1

988 307 1

988 308 1

988 1109 1

988 1267 1

988 445 1

988 223 1

988 1124 0

988 743 0

988 2669 0

988 2650 0

988 3189 0

988 87 0

988 1877 0

988 269 0

988 2815 0

988 3712 0

988 1107 0

988 1701 0

988 1640 0

988 613 0

988 2611 0

988 2272 0

988 3820 0

989 2563 1

989 597 1

989 1372 1

989 2799 1

989 2800 1

989 2801 1

989 2802 1

989 2803 1

989 2804 1

989 2805 1

989 2806 1

989 2807 1

989 2808 1
989 2809 1
989 2810 1
989 2811 1
989 2812 1
989 2813 1
989 2814 1
989 2396 0
989 2254 0
989 1642 0
989 2475 0
989 2324 0
989 300 0
989 516 0
989 3622 0
989 1407 0
989 295 0
989 2830 0
989 3738 0
989 2215 0
989 2397 0
989 3439 0
989 1652 0
989 3271 0
989 17 0
989 3558 0
990 96 1
990 2816 1
990 74 1
990 106 1

990 235 1
990 1899 1
990 2815 1
990 118 1
990 124 1
990 126 1
990 255 1
990 430 0
990 2560 0
990 799 0
990 3642 0
990 3001 0
990 1239 0
990 234 0
990 3183 0
990 1476 0
990 2512 0
990 1378 0
991 832 1
991 353 1
991 2817 1
991 75 1
991 77 1
991 1325 1
991 117 1
991 24 1
991 3057 0
991 263 0
991 1570 0
991 3363 0

991 2222 0

991 65 0

991 983 0

991 192 0

992 673 1

992 2818 1

992 1091 1

992 36 1

992 740 1

992 996 1

992 7 1

992 2819 1

992 554 1

992 43 1

992 300 1

992 217 1

992 250 1

992 347 1

992 285 1

992 126 1

992 287 1

992 399 0

992 1518 0

992 3227 0

992 1051 0

992 3834 0

992 406 0

992 3311 0

992 3762 0

992 3732 0

992 3455 0

992 2458 0

992 2081 0

992 2153 0

992 3124 0

992 3615 0

992 1240 0

992 2802 0

993 355 1

993 2020 1

993 293 1

993 37 1

993 2375 1

993 2535 1

993 2820 1

993 495 1

993 1807 1

993 2451 1

993 52 1

993 55 1

993 572 1

993 221 1

993 1280 0

993 1964 0

993 335 0

993 3 0

993 1289 0

993 969 0

993 1915 0

993 553 0

993 419 0

993 3530 0

993 1330 0

993 1397 0

993 787 0

993 1222 0

994 192 1

994 832 1

994 2822 1

994 39 1

994 104 1

994 378 1

994 1871 1

994 2175 1

994 22 1

994 254 1

994 377 1

994 90 1

994 91 1

994 1754 1

994 126 1

994 863 1

994 1537 0

994 2664 0

994 3464 0

994 3017 0

994 780 0

994 829 0

994 3688 0

994 2432 0

994 1339 0

994 472 0

994 517 0

994 3518 0

994 3659 0

994 2458 0

994 933 0

994 3366 0

995 832 1

995 353 1

995 928 1

995 483 1

995 932 1

995 198 1

995 1798 1

995 2823 1

995 2158 1

995 21 1

995 22 1

995 310 1

995 473 1

995 1338 1

995 155 1

995 30 1

995 610 0

995 2594 0

995 647 0

995 3531 0

995 2492 0

995 207 0

995 2167 0

995 3752 0

995 398 0

995 475 0

995 2095 0

995 1670 0

995 432 0

995 1352 0

995 1810 0

995 867 0

996 2336 1

996 36 1

996 542 1

996 40 1

996 2824 1

996 788 1

996 852 1

996 22 1

996 445 1

996 190 1

996 1442 0

996 3161 0

996 1863 0

996 2745 0

996 2303 0

996 3307 0

996 3618 0

996 154 0

996 3367 0

996 1158 0

997 928 1

997 1664 1

997 678 1

997 1094 1

997 2118 1

997 2120 1

997 2825 1

997 2826 1

997 654 1

997 306 1

997 531 1

997 2741 1

997 541 1

997 3067 0

997 1762 0

997 2853 0

997 2249 0

997 3805 0

997 2892 0

997 807 0

997 423 0

997 1088 0

997 2930 0

997 210 0

997 1337 0

997 3338 0

998 34 1

998 732 1

998 196 1

998 519 1

998 2827 1

998 460 1

998 2828 1

998 2829 1

998 1617 1

998 307 1

998 2581 1

998 727 1

998 1530 1

998 155 1

998 156 1

998 2588 1

998 204 0

998 2502 0

998 777 0

998 315 0

998 3720 0

998 504 0

998 2651 0

998 2031 0

998 2589 0

998 2667 0

998 3674 0

998 1865 0

998 1771 0

998 53 0

998 1665 0

998 3064 0

999 131 1

999 132 1

999 133 1

999 22 1

999 24 1

999 538 1

999 295 1

999 1080 1

999 192 1

999 74 1

999 80 1

999 1234 1

999 1235 1

999 469 1

999 861 1

999 356 1

999 230 1

999 361 1

999 237 1

999 116 1

999 382 1

999 127 1

999 1764 0

999 2740 0

999 3637 0

999 2930 0

999 339 0

999 3254 0

999 3305 0

999 2468 0

999 1449 0

999 2377 0

999 310 0

999 945 0

999 1762 0

999 751 0

999 2117 0

999 155 0

999 1973 0

999 2820 0

999 2948 0

999 2460 0

999 60 0

999 2758 0

1000 4 1

1000 2443 1

1000 2830 1

1000 24 1

1000 1828 1

1000 55 1

1000 441 1

1000 445 1

1000 2371 1

1000 329 1

1000 330 1

1000 334 1

1000 335 1

1000 89 1

1000 90 1

1000 91 1

1000 1893 1

1000 1272 1

1000	2302	1
1000	767 1	
1000	3567	0
1000	2259	0
1000	2483	0
1000	282 0	
1000	763 0	
1000	2268	0
1000	392 0	
1000	173 0	
1000	516 0	
1000	1857	0
1000	3071	0
1000	2969	0
1000	384 0	
1000	2288	0
1000	2372	0
1000	1118	0
1000	2587	0
1000	254 0	
1000	3794	0
1000	1801	0
1001	66 1	
1001	276 1	
1001	2831	1
1001	2812	0
1001	474 0	
1001	3393	0
1002	232 1	
1002	377 1	

1002	66	1
1002	130	1
1002	2255	0
1002	14	0
1002	2291	0
1002	1045	0
1003	833	1
1003	1098	1
1003	301	1
1003	47	1
1003	209	1
1003	210	1
1003	52	1
1003	1979	1
1003	446	1
1003	2057	0
1003	653	0
1003	339	0
1003	685	0
1003	1845	0
1003	1651	0
1003	3152	0
1003	2004	0
1003	88	0
1004	517	1
1004	519	1
1004	2832	1
1004	2833	1
1004	2834	1
1004	2835	1

1004	2836	1
1004	2837	1
1004	795 1	
1004	928 1	
1004	417 1	
1004	2464	1
1004	296 1	
1004	1712	1
1004	196 1	
1004	453 1	
1004	966 1	
1004	732 1	
1004	1384	1
1004	763 1	
1004	508 1	
1004	779 0	
1004	2112	0
1004	1530	0
1004	2008	0
1004	1831	0
1004	1556	0
1004	2784	0
1004	1603	0
1004	625 0	
1004	2227	0
1004	1726	0
1004	2510	0
1004	3325	0
1004	131 0	
1004	1509	0

1004	3055	0
1004	3840	0
1004	294	0
1004	2597	0
1004	3752	0
1004	1192	0
1005	66	1
1005	74	1
1005	75	1
1005	171	1
1005	79	1
1005	117	1
1005	2838	1
1005	87	1
1005	90	1
1005	254	1
1005	127	1
1005	3621	0
1005	2371	0
1005	420	0
1005	1938	0
1005	371	0
1005	1708	0
1005	380	0
1005	734	0
1005	410	0
1005	1153	0
1005	1180	0
1006	96	1
1006	803	1

1006	1446	1
1006	776	1
1006	1224	1
1006	42	1
1006	1518	1
1006	209	1
1006	211	1
1006	119	1
1006	895	0
1006	923	0
1006	1584	0
1006	752	0
1006	3386	0
1006	2083	0
1006	2272	0
1006	2885	0
1006	1456	0
1006	412	0
1007	802	1
1007	713	1
1007	714	1
1007	589	1
1007	302	1
1007	303	1
1007	212	1
1007	1622	1
1007	2839	1
1007	280	1
1007	1119	1
1007	3156	0

1007	1680	0
1007	2117	0
1007	3004	0
1007	3660	0
1007	887	0
1007	1674	0
1007	1438	0
1007	960	0
1007	3606	0
1007	2409	0
1008	704	1
1008	34	1
1008	35	1
1008	165	1
1008	197	1
1008	1063	1
1008	2821	1
1008	1615	1
1008	1136	1
1008	52	1
1008	53	1
1008	2840	1
1008	1594	1
1008	575	1
1008	274	0
1008	2443	0
1008	3508	0
1008	578	0
1008	1566	0
1008	1652	0

1008	1782	0
1008	3019	0
1008	3482	0
1008	2868	0
1008	2850	0
1008	2451	0
1008	3064	0
1008	306 0	
1009	386 1	
1009	2562	1
1009	1228	1
1009	77 1	
1009	397 1	
1009	1612	1
1009	81 1	
1009	1202	1
1009	87 1	
1009	2841	1
1009	58 1	
1009	2842	1
1009	2966	0
1009	1115	0
1009	667 0	
1009	3407	0
1009	3441	0
1009	2702	0
1009	1891	0
1009	3564	0
1009	324 0	
1009	817 0	

1009	178	0
1009	3570	0
1010	96	1
1010	832	1
1010	1832	1
1010	105	1
1010	361	1
1010	109	1
1010	46	1
1010	52	1
1010	87	1
1010	572	1
1010	2258	0
1010	3250	0
1010	417	0
1010	3764	0
1010	2304	0
1010	2517	0
1010	1342	0
1010	811	0
1010	2443	0
1010	968	0
1011	2560	1
1011	2561	1
1011	2562	1
1011	2689	1
1011	2564	1
1011	2313	1
1011	397	1
1011	2843	1

1011	2844	1
1011	2845	1
1011	2846	1
1011	1579	1
1011	58	1
1011	1228	1
1011	77	1
1011	1612	1
1011	490	1
1011	2804	1
1011	2810	1
1011	2814	1
1011	2608	0
1011	2336	0
1011	872	0
1011	187	0
1011	3317	0
1011	175	0
1011	394	0
1011	2445	0
1011	1438	0
1011	1096	0
1011	1953	0
1011	2044	0
1011	36	0
1011	458	0
1011	575	0
1011	3741	0
1011	2011	0
1011	3291	0

1011	2250	0
1011	1987	0
1012	96	1
1012	800	1
1012	199	1
1012	1031	1
1012	1319	1
1012	242	1
1012	52	1
1012	119	1
1012	2494	0
1012	3715	0
1012	2199	0
1012	1747	0
1012	2854	0
1012	1884	0
1012	673	0
1012	2040	0
1013	2848	1
1013	2849	1
1013	41	1
1013	714	1
1013	52	1
1013	376	1
1013	1277	1
1013	2847	1
1013	3827	0
1013	789	0
1013	1273	0
1013	488	0

1013	3658	0
1013	1616	0
1013	1925	0
1013	1596	0
1014	132	1
1014	230	1
1014	72	1
1014	105	1
1014	74	1
1014	75	1
1014	361	1
1014	237	1
1014	81	1
1014	1202	1
1014	117	1
1014	87	1
1014	254	1
1014	127	1
1014	1308	0
1014	2125	0
1014	2354	0
1014	2133	0
1014	938	0
1014	1302	0
1014	2177	0
1014	3638	0
1014	2545	0
1014	2455	0
1014	932	0
1014	196	0

1014	2012	0
1014	2290	0
1015	184	1
1015	2850	1
1015	1547	0
1015	1343	0
1016	128	1
1016	130	1
1016	1570	1
1016	773	1
1016	137	1
1016	2058	1
1016	78	1
1016	79	1
1016	184	1
1016	185	1
1016	2652	0
1016	2853	0
1016	3683	0
1016	1204	0
1016	3108	0
1016	1883	0
1016	2901	0
1016	2995	0
1016	1072	0
1016	1225	0
1017	610	1
1017	2851	1
1017	2852	1
1017	453	1

1017	165	1
1017	102	1
1017	1416	1
1017	1801	1
1017	2853	1
1017	2854	1
1017	1484	1
1017	2855	1
1017	2856	1
1017	111	1
1017	2857	1
1017	53	1
1017	2014	1
1017	703	1
1017	1717	0
1017	291	0
1017	137	0
1017	299	0
1017	585	0
1017	1551	0
1017	3430	0
1017	3682	0
1017	246	0
1017	700	0
1017	1713	0
1017	2038	0
1017	2097	0
1017	19	0
1017	3578	0
1017	3371	0

1017	1776	0
1017	2236	0
1018	131	1
1018	134	1
1018	74	1
1018	75	1
1018	79	1
1018	254	1
1018	87	1
1018	185	1
1018	91	1
1018	861	1
1018	126	1
1018	863	1
1018	627	0
1018	870	0
1018	3233	0
1018	2770	0
1018	1147	0
1018	1714	0
1018	582	0
1018	1580	0
1018	3497	0
1018	754	0
1018	2851	0
1018	4	0
1019	2567	1
1019	10	1
1019	13	1
1019	15	1

1019	17	1
1019	277	1
1019	2858	1
1019	307	1
1019	320	1
1019	963	1
1019	329	1
1019	971	1
1019	334	1
1019	226	1
1019	227	1
1019	498	1
1019	1267	1
1019	887	1
1019	1271	1
1019	1907	0
1019	2156	0
1019	2773	0
1019	398	0
1019	1300	0
1019	2775	0
1019	1827	0
1019	2464	0
1019	1349	0
1019	664	0
1019	2703	0
1019	1674	0
1019	3450	0
1019	493	0
1019	2776	0

1019	3289	0
1019	2870	0
1019	3279	0
1019	3745	0
1020	102	1
1020	873	1
1020	2859	1
1020	2860	1
1020	47	1
1020	210	1
1020	242	1
1020	52	1
1020	1523	1
1020	119	1
1020	219	1
1020	952	0
1020	167	0
1020	2985	0
1020	850	0
1020	584	0
1020	2583	0
1020	3732	0
1020	3003	0
1020	1529	0
1020	1624	0
1020	2800	0
1021	1856	1
1021	2147	1
1021	772	1
1021	235	1

1021	1143	1
1021	406	1
1021	118	1
1021	376	1
1021	538	1
1021	413	1
1021	127	1
1021	125	0
1021	1163	0
1021	2131	0
1021	831	0
1021	1987	0
1021	3718	0
1021	1772	0
1021	181	0
1021	951	0
1021	3427	0
1021	480	0
1022	192	1
1022	1443	1
1022	356	1
1022	104	1
1022	1047	1
1022	277	1
1022	118	1
1022	55	1
1022	378	1
1022	187	1
1022	286	1
1022	1226	0

1022	2223	0
1022	826	0
1022	3822	0
1022	1688	0
1022	1942	0
1022	919	0
1022	2073	0
1022	1880	0
1022	285	0
1022	131	0
1023	1153	1
1023	202	1
1023	171	1
1023	204	1
1023	78	1
1023	206	1
1023	2862	1
1023	115	1
1023	184	1
1023	185	1
1023	2053	0
1023	3512	0
1023	1731	0
1023	358	0
1023	1751	0
1023	2277	0
1023	1800	0
1023	1262	0
1023	520	0
1023	3093	0

1024	133	1
1024	75	1
1024	267	1
1024	78	1
1024	79	1
1024	373	1
1024	87	1
1024	1018	1
1024	3032	0
1024	3083	0
1024	1978	0
1024	1082	0
1024	2757	0
1024	524	0
1024	3726	0
1024	276	0
1025	36	1
1025	105	1
1025	1322	1
1025	107	1
1025	46	1
1025	507	1
1025	61	1
1025	3707	0
1025	3513	0
1025	1443	0
1025	2226	0
1025	2357	0
1025	1921	0
1025	3712	0

1026	2526	1
1026	40	1
1026	74	1
1026	2863	1
1026	2864	1
1026	2865	1
1026	2866	1
1026	916	1
1026	951	1
1026	282	1
1026	124	1
1026	1310	1
1026	2207	0
1026	3449	0
1026	1349	0
1026	915	0
1026	2412	0
1026	1005	0
1026	928	0
1026	1762	0
1026	109	0
1026	2001	0
1026	408	0
1026	2891	0
1027	1153	1
1027	66	1
1027	134	1
1027	72	1
1027	74	1
1027	171	1

1027	79	1
1027	2867	1
1027	52	1
1027	374	1
1027	127	1
1027	1882	0
1027	2446	0
1027	3523	0
1027	1412	0
1027	48	0
1027	3451	0
1027	3537	0
1027	288	0
1027	379	0
1027	18	0
1027	1282	0
1028	132	1
1028	1258	1
1028	1324	1
1028	237	1
1028	78	1
1028	270	1
1028	185	1
1028	698	1
1028	923	1
1028	852	0
1028	2724	0
1028	1636	0
1028	947	0
1028	1959	0

1028	1306	0
1028	351	0
1028	2373	0
1028	1080	0
1029	129	1
1029	385	1
1029	899	1
1029	132	1
1029	261	1
1029	1037	1
1029	1040	1
1029	660	1
1029	1310	1
1029	40	1
1029	552	1
1029	2868	1
1029	192	1
1029	66	1
1029	74	1
1029	202	1
1029	87	1
1029	127	1
1029	253	1
1029	2175	1
1029	483	0
1029	1661	0
1029	3208	0
1029	510	0
1029	983	0
1029	1068	0

1029	1532	0
1029	1303	0
1029	2583	0
1029	1374	0
1029	179	0
1029	1139	0
1029	2015	0
1029	2993	0
1029	2396	0
1029	1850	0
1029	888	0
1029	1862	0
1029	626	0
1029	900	0
1030	224	1
1030	289	1
1030	34	1
1030	928	1
1030	36	1
1030	1664	1
1030	42	1
1030	2795	1
1030	2253	1
1030	2869	1
1030	1182	1
1030	1171	0
1030	2596	0
1030	835	0
1030	656	0
1030	856	0

1030	138	0
1030	173	0
1030	1859	0
1030	3171	0
1030	883	0
1030	615	0
1031	96	1
1031	264	1
1031	171	1
1031	115	1
1031	2868	1
1031	2870	1
1031	87	1
1031	254	1
1031	678	0
1031	3524	0
1031	1530	0
1031	35	0
1031	3152	0
1031	3363	0
1031	1429	0
1031	1920	0
1032	163	1
1032	516	1
1032	295	1
1032	617	1
1032	460	1
1032	141	1
1032	2702	1
1032	111	1

1032	1586	1
1032	2871	1
1032	187	1
1032	668	1
1032	510	1
1032	1290	0
1032	801	0
1032	790	0
1032	1590	0
1032	1741	0
1032	3645	0
1032	2246	0
1032	758	0
1032	3767	0
1032	2764	0
1032	1567	0
1032	1602	0
1032	647	0
1033	544	1
1033	100	1
1033	133	1
1033	557	1
1033	977	1
1033	115	1
1033	916	1
1033	762	1
1033	380	1
1033	1277	1
1033	844	0
1033	27	0

1033	17	0
1033	3262	0
1033	3334	0
1033	2978	0
1033	3400	0
1033	758	0
1033	154	0
1033	3270	0
1034	774	1
1034	782	1
1034	785	1
1034	147	1
1034	788	1
1034	2771	1
1034	2872	1
1034	2873	1
1034	2874	1
1034	2875	1
1034	2876	1
1034	2454	0
1034	2895	0
1034	2617	0
1034	66	0
1034	950	0
1034	89	0
1034	123	0
1034	1775	0
1034	759	0
1034	1878	0
1034	3014	0

1035	377	1
1035	267	1
1035	206	1
1035	79	1
1035	115	1
1035	435	1
1035	1590	1
1035	184	1
1035	185	1
1035	647	0
1035	2394	0
1035	1177	0
1035	485	0
1035	781	0
1035	2468	0
1035	1986	0
1035	3836	0
1035	2622	0
1036	832	1
1036	1186	1
1036	134	1
1036	109	1
1036	1742	1
1036	976	1
1036	54	1
1036	377	1
1036	91	1
1036	2877	1
1036	126	1
1036	286	1

1036	1661	0
1036	3433	0
1036	2899	0
1036	1619	0
1036	3516	0
1036	1200	0
1036	1910	0
1036	1654	0
1036	1732	0
1036	861	0
1036	3626	0
1036	2104	0
1037	96	1
1037	800	1
1037	2880	1
1037	2881	1
1037	806	1
1037	7	1
1037	42	1
1037	1266	1
1037	211	1
1037	532	1
1037	250	1
1037	2878	1
1037	2879	1
1037	3348	0
1037	3668	0
1037	2485	0
1037	2202	0
1037	1243	0

1037	2737	0
1037	2874	0
1037	2238	0
1037	148	0
1037	3602	0
1037	1171	0
1037	2376	0
1037	1847	0
1038	2882	1
1038	2883	1
1038	36	1
1038	447	1
1038	334	1
1038	558	1
1038	879	1
1038	275	1
1038	883	1
1038	889	1
1038	442	1
1038	2235	1
1038	956	1
1038	477	1
1038	30	1
1038	191	1
1038	759	0
1038	1204	0
1038	494	0
1038	3441	0
1038	260	0
1038	911	0

1038	1876	0
1038	1675	0
1038	2413	0
1038	536	0
1038	3293	0
1038	2531	0
1038	61	0
1038	1114	0
1038	1294	0
1038	2788	0
1039	450	1
1039	2884	1
1039	2885	1
1039	109	1
1039	2254	1
1039	527	1
1039	277	1
1039	1046	1
1039	221	1
1039	302	0
1039	3341	0
1039	3460	0
1039	791	0
1039	3437	0
1039	2348	0
1039	3560	0
1039	1805	0
1039	717	0
1040	2886	1
1040	232	1

1040	237 1	
1040	979 1	
1040	184 1	
1040	377 1	
1040	987 1	
1040	1478	0
1040	2478	0
1040	3806	0
1040	1362	0
1040	1554	0
1040	1767	0
1040	3460	0
1041	480 1	
1041	1122	1
1041	493 1	
1041	1869	1
1041	722 1	
1041	2162	1
1041	1172	1
1041	1877	1
1041	1974	1
1041	121 1	
1041	124 1	
1041	3165	0
1041	3237	0
1041	2457	0
1041	1598	0
1041	257 0	
1041	1263	0
1041	2616	0

1041	2839	0
1041	2822	0
1041	2341	0
1041	654	0
1042	388	1
1042	2694	1
1042	2887	1
1042	2347	1
1042	1100	1
1042	1136	1
1042	2067	1
1042	221	1
1042	2382	0
1042	3608	0
1042	232	0
1042	2158	0
1042	442	0
1042	2270	0
1042	45	0
1042	3369	0
1043	37	1
1043	2888	1
1043	2889	1
1043	2890	1
1043	2252	1
1043	46	1
1043	55	1
1043	1797	0
1043	1403	0
1043	104	0

1043	682	0
1043	2437	0
1043	2547	0
1043	212	0
1044	195	1
1044	2819	1
1044	478	1
1044	38	1
1044	200	1
1044	1352	1
1044	2891	1
1044	2892	1
1044	2893	1
1044	2894	1
1044	2895	1
1044	2896	1
1044	1105	1
1044	502	1
1044	151	1
1044	442	1
1044	1723	1
1044	30	1
1044	2656	0
1044	3514	0
1044	8	0
1044	1617	0
1044	1703	0
1044	1802	0
1044	2510	0
1044	613	0

1044	3216	0
1044	3712	0
1044	554	0
1044	975	0
1044	1088	0
1044	154	0
1044	2188	0
1044	2719	0
1044	362	0
1044	3573	0
1045	512	1
1045	2465	1
1045	516	1
1045	422	1
1045	2855	1
1045	904	1
1045	1005	1
1045	111	1
1045	1009	1
1045	2897	1
1045	883	1
1045	2898	1
1045	53	1
1045	795	1
1045	508	1
1045	3292	0
1045	1971	0
1045	745	0
1045	1304	0
1045	395	0

1045	333	0
1045	1455	0
1045	574	0
1045	17	0
1045	2426	0
1045	1384	0
1045	3725	0
1045	3562	0
1045	1195	0
1045	274	0
1046	1122	1
1046	35	1
1046	100	1
1046	165	1
1046	261	1
1046	903	1
1046	1502	1
1046	1513	1
1046	926	1
1046	118	1
1046	445	1
1046	158	1
1046	191	1
1046	3389	0
1046	882	0
1046	3327	0
1046	3729	0
1046	2463	0
1046	723	0
1046	2477	0

1046	177	0
1046	541	0
1046	1012	0
1046	636	0
1046	2991	0
1046	1646	0
1047	874	1
1047	441	1
1047	493	1
1047	2861	1
1047	210	1
1047	2899	1
1047	118	1
1047	2073	1
1047	89	1
1047	1979	1
1047	125	1
1047	446	1
1047	1242	0
1047	785	0
1047	3121	0
1047	2975	0
1047	807	0
1047	2538	0
1047	529	0
1047	130	0
1047	491	0
1047	1599	0
1047	377	0
1047	3396	0

1048	96	1
1048	352	1
1048	802	1
1048	133	1
1048	1098	1
1048	782	1
1048	47	1
1048	1593	1
1048	505	1
1048	1118	1
1048	2748	0
1048	41	0
1048	15	0
1048	3321	0
1048	295	0
1048	1305	0
1048	3719	0
1048	454	0
1048	1291	0
1048	390	0
1049	96	1
1049	352	1
1049	34	1
1049	1122	1
1049	36	1
1049	42	1
1049	210	1
1049	534	1
1049	217	1
1049	286	1

1049	597	0
1049	2448	0
1049	548	0
1049	589	0
1049	3739	0
1049	1366	0
1049	3220	0
1049	471	0
1049	2395	0
1049	3568	0
1050	102	1
1050	776	1
1050	302	1
1050	47	1
1050	2448	1
1050	305	1
1050	1523	1
1050	52	1
1050	1524	1
1050	863	1
1050	3368	0
1050	973	0
1050	1934	0
1050	3163	0
1050	3140	0
1050	1176	0
1050	22	0
1050	1561	0
1050	3697	0
1050	2235	0

1051	37	1
1051	2472	1
1051	364	1
1051	564	1
1051	95	1
1051	2735	0
1051	950	0
1051	2182	0
1051	17	0
1051	2220	0
1052	227	1
1052	36	1
1052	13	1
1052	275	1
1052	499	1
1052	788	1
1052	2133	1
1052	2325	1
1052	445	1
1052	138	0
1052	529	0
1052	626	0
1052	1246	0
1052	1971	0
1052	3719	0
1052	533	0
1052	549	0
1052	94	0
1053	272	1
1053	24	1

1053	1949	1
1053	30	1
1053	161	1
1053	676	1
1053	684	1
1053	1324	1
1053	2613	1
1053	1081	1
1053	957	1
1053	72	1
1053	2900	1
1053	2901	1
1053	2902	1
1053	858	1
1053	1896	1
1053	105	1
1053	2665	1
1053	2669	1
1053	2608	0
1053	2227	0
1053	787	0
1053	3700	0
1053	2469	0
1053	438	0
1053	877	0
1053	2947	0
1053	3845	0
1053	1602	0
1053	3686	0
1053	1663	0

1053	2386	0
1053	2163	0
1053	3714	0
1053	455	0
1053	1922	0
1053	2815	0
1053	1263	0
1053	2504	0
1054	109	1
1054	2903	1
1054	52	1
1054	55	1
1054	2904	1
1054	26	1
1054	445	1
1054	1662	1
1054	2981	0
1054	3778	0
1054	281	0
1054	284	0
1054	334	0
1054	1580	0
1054	3039	0
1054	1161	0
1055	576	1
1055	199	1
1055	52	1
1055	1912	1
1055	2905	1
1055	2906	1

1055	2907	1
1055	2908	1
1055	3208	0
1055	306 0	
1055	3028	0
1055	2284	0
1055	1573	0
1055	3419	0
1055	2602	0
1055	721 0	
1056	1728	1
1056	195 1	
1056	196 1	
1056	200 1	
1056	105 1	
1056	392 1	
1056	1036	1
1056	1528	1
1056	2909	1
1056	3089	0
1056	52 0	
1056	2455	0
1056	275 0	
1056	1080	0
1056	3290	0
1056	2573	0
1056	3228	0
1056	174 0	
1057	391 1	
1057	105 1	

1057	235	1
1057	110	1
1057	46	1
1057	399	1
1057	528	1
1057	1396	1
1057	55	1
1057	764	1
1057	2910	1
1057	863	1
1057	357	0
1057	2095	0
1057	141	0
1057	3809	0
1057	3255	0
1057	1032	0
1057	3468	0
1057	2351	0
1057	2858	0
1057	3764	0
1057	679	0
1057	94	0
1058	202	1
1058	371	1
1058	550	1
1058	970	0
1058	621	0
1058	868	0
1059	1760	1
1059	36	1

1059	261	1
1059	548	1
1059	806	1
1059	1161	1
1059	1162	1
1059	1166	1
1059	80	1
1059	243	1
1059	245	1
1059	126	1
1059	2911	1
1059	2609	0
1059	990	0
1059	1851	0
1059	358	0
1059	1369	0
1059	1075	0
1059	1806	0
1059	3801	0
1059	629	0
1059	30	0
1059	712	0
1059	1491	0
1059	3765	0
1060	161	1
1060	196	1
1060	485	1
1060	516	1
1060	295	1
1060	685	1

1060	111	1
1060	882	1
1060	275	1
1060	883	1
1060	1528	1
1060	155	1
1060	3669	0
1060	314	0
1060	1590	0
1060	1264	0
1060	3285	0
1060	354	0
1060	1798	0
1060	1186	0
1060	484	0
1060	1363	0
1060	33	0
1060	921	0
1061	754	1
1061	1748	1
1061	1300	1
1061	1155	0
1061	1542	0
1061	3321	0
1062	96	1
1062	98	1
1062	1445	1
1062	102	1
1062	47	1
1062	209	1

1062	210	1
1062	242	1
1062	212	1
1062	216	1
1062	3810	0
1062	3789	0
1062	3081	0
1062	3275	0
1062	2551	0
1062	2011	0
1062	2636	0
1062	282	0
1062	1851	0
1062	823	0
1063	1122	1
1063	2210	1
1063	493	1
1063	1997	1
1063	722	1
1063	2162	1
1063	1974	1
1063	1239	1
1063	1240	1
1063	2072	1
1063	2074	1
1063	124	1
1063	101	0
1063	182	0
1063	491	0
1063	2869	0

1063	1526	0
1063	1706	0
1063	1578	0
1063	2512	0
1063	354	0
1063	945	0
1063	2158	0
1063	2086	0
1064	2912	1
1064	1697	1
1064	1186	1
1064	2913	1
1064	2914	1
1064	389	1
1064	2915	1
1064	391	1
1064	2916	1
1064	105	1
1064	267	1
1064	46	1
1064	469	1
1064	87	1
1064	3543	0
1064	2308	0
1064	2321	0
1064	1242	0
1064	2638	0
1064	3652	0
1064	1858	0
1064	824	0

1064	252	0
1064	2236	0
1064	3035	0
1064	1556	0
1064	3040	0
1064	546	0
1065	1889	1
1065	387	1
1065	133	1
1065	73	1
1065	80	1
1065	560	1
1065	52	1
1065	55	1
1065	281	0
1065	2389	0
1065	3359	0
1065	2647	0
1065	207	0
1065	3593	0
1065	3740	0
1065	1799	0
1066	128	1
1066	130	1
1066	899	1
1066	2917	1
1066	1960	1
1066	361	1
1066	74	1
1066	1710	1

1066	80	1
1066	55	1
1066	570	1
1066	1823	0
1066	791	0
1066	410	0
1066	1307	0
1066	1320	0
1066	1131	0
1066	3075	0
1066	43	0
1066	1853	0
1066	1409	0
1066	2163	0
1067	516	1
1067	1545	1
1067	2826	1
1067	2067	1
1067	1951	1
1067	164	1
1067	936	1
1067	297	1
1067	2856	1
1067	684	1
1067	1712	1
1067	2364	1
1067	320	1
1067	196	1
1067	223	1
1067	2660	1

1067	2918	1
1067	2919	1
1067	2920	1
1067	498 1	
1067	883 1	
1067	252 1	
1067	1765	0
1067	2104	0
1067	1028	0
1067	974 0	
1067	178 0	
1067	446 0	
1067	110 0	
1067	2653	0
1067	3363	0
1067	3192	0
1067	3345	0
1067	1102	0
1067	123 0	
1067	1444	0
1067	2127	0
1067	2667	0
1067	1532	0
1067	3213	0
1067	2803	0
1067	930 0	
1067	2504	0
1067	923 0	
1068	1536	1
1068	155 1	

1068	156	1
1068	286	1
1068	160	1
1068	35	1
1068	165	1
1068	2089	1
1068	299	1
1068	57	1
1068	448	1
1068	196	1
1068	582	1
1068	2013	1
1068	1502	1
1068	735	1
1068	358	1
1068	1514	1
1068	110	1
1068	508	1
1068	2029	0
1068	1211	0
1068	744	0
1068	489	0
1068	3461	0
1068	3396	0
1068	1509	0
1068	2481	0
1068	2288	0
1068	2725	0
1068	2353	0
1068	3577	0

1068	2675	0
1068	3430	0
1068	803	0
1068	1523	0
1068	218	0
1068	1035	0
1068	1757	0
1068	3763	0
1069	802	1
1069	325	1
1069	102	1
1069	776	1
1069	1261	1
1069	302	1
1069	560	1
1069	118	1
1069	2021	0
1069	3041	0
1069	1256	0
1069	828	0
1069	3635	0
1069	3838	0
1069	1561	0
1069	2124	0
1070	131	1
1070	230	1
1070	2921	1
1070	74	1
1070	80	1
1070	117	1

1070	22	1
1070	895	1
1070	922	1
1070	829	1
1070	127	1
1070	1283	0
1070	2442	0
1070	1358	0
1070	2143	0
1070	3485	0
1070	1882	0
1070	2496	0
1070	3055	0
1070	132	0
1070	773	0
1070	1426	0
1071	230	1
1071	104	1
1071	137	1
1071	233	1
1071	2922	1
1071	2923	1
1071	2924	1
1071	2925	1
1071	957	1
1071	1950	1
1071	20	1
1071	22	1
1071	55	1
1071	24	1

1071	572	1
1071	413	1
1071	574	1
1071	294	0
1071	1693	0
1071	365	0
1071	2884	0
1071	3312	0
1071	2680	0
1071	3594	0
1071	2106	0
1071	2831	0
1071	148	0
1071	3186	0
1071	2970	0
1071	237	0
1071	875	0
1071	1773	0
1071	1037	0
1071	631	0
1072	130	1
1072	132	1
1072	72	1
1072	137	1
1072	74	1
1072	75	1
1072	237	1
1072	117	1
1072	1141	1
1072	87	1

1072	3276	0
1072	3298	0
1072	188 0	
1072	2293	0
1072	3704	0
1072	2129	0
1072	3211	0
1072	2670	0
1072	303 0	
1072	1514	0
1073	608 1	
1073	865 1	
1073	1088	1
1073	163 1	
1073	36 1	
1073	613 1	
1073	614 1	
1073	1416	1
1073	617 1	
1073	141 1	
1073	2926	1
1073	111 1	
1073	1586	1
1073	664 1	
1073	558 0	
1073	2793	0
1073	1130	0
1073	2506	0
1073	3802	0
1073	2987	0

1073	1909	0
1073	941	0
1073	2269	0
1073	3605	0
1073	3013	0
1073	2699	0
1073	2758	0
1073	269	0
1074	131	1
1074	104	1
1074	41	1
1074	137	1
1074	107	1
1074	361	1
1074	1324	1
1074	46	1
1074	957	1
1074	2927	1
1074	1589	1
1074	24	1
1074	187	1
1074	413	1
1074	863	1
1074	3058	0
1074	3799	0
1074	2041	0
1074	351	0
1074	1554	0
1074	2206	0
1074	3520	0

1074	3646	0
1074	3774	0
1074	1516	0
1074	3004	0
1074	2313	0
1074	391	0
1074	1786	0
1074	1283	0
1075	130	1
1075	132	1
1075	134	1
1075	135	1
1075	74	1
1075	267	1
1075	1325	1
1075	206	1
1075	79	1
1075	80	1
1075	117	1
1075	374	1
1075	87	1
1075	185	1
1075	127	1
1075	892	0
1075	626	0
1075	326	0
1075	2036	0
1075	2188	0
1075	2837	0
1075	3227	0

1075	1952	0
1075	2687	0
1075	629	0
1075	2139	0
1075	244	0
1075	2299	0
1075	2158	0
1075	1753	0
1076	516	1
1076	708	1
1076	295	1
1076	361	1
1076	107	1
1076	111	1
1076	2928	1
1076	1969	1
1076	1078	1
1076	2135	1
1076	56	1
1076	1083	1
1076	1971	0
1076	1610	0
1076	1453	0
1076	2889	0
1076	1352	0
1076	2243	0
1076	2401	0
1076	2906	0
1076	1202	0
1076	227	0

1076	3135	0
1076	1612	0
1077	352	1
1077	356	1
1077	36	1
1077	357	1
1077	487	1
1077	2604	1
1077	2929	1
1077	276	1
1077	52	1
1077	788	1
1077	1310	1
1077	8	0
1077	2865	0
1077	2527	0
1077	1688	0
1077	445	0
1077	3158	0
1077	673	0
1077	1656	0
1077	1595	0
1077	3198	0
1077	2562	0
1078	483	1
1078	36	1
1078	105	1
1078	109	1
1078	527	1
1078	114	1

1078	52	1
1078	2199	1
1078	1662	1
1078	895	1
1078	521	0
1078	1923	0
1078	3479	0
1078	3036	0
1078	1015	0
1078	1399	0
1078	2294	0
1078	43	0
1078	2080	0
1078	718	0
1079	992	1
1079	994	1
1079	1730	1
1079	1256	1
1079	522	1
1079	427	1
1079	524	1
1079	526	1
1079	119	1
1079	1115	1
1079	1668	0
1079	3325	0
1079	2362	0
1079	964	0
1079	3140	0
1079	1863	0

1079	1424	0
1079	1171	0
1079	894	0
1079	377	0
1080	673	1
1080	2819	1
1080	104	1
1080	681	1
1080	235	1
1080	2610	1
1080	2930	1
1080	1236	1
1080	2931	1
1080	55	1
1080	24	1
1080	442	1
1080	508	1
1080	1277	1
1080	1278	1
1080	223	1
1080	460	0
1080	1566	0
1080	3346	0
1080	1941	0
1080	2735	0
1080	2282	0
1080	42	0
1080	1253	0
1080	2410	0
1080	63	0

1080	3199	0
1080	2699	0
1080	2509	0
1080	1780	0
1080	1630	0
1080	1401	0
1081	516 1	
1081	422 1	
1081	2638	1
1081	2932	1
1081	2933	1
1081	790 1	
1081	2934	1
1081	2759	0
1081	1916	0
1081	1342	0
1081	3284	0
1081	1414	0
1081	244 0	
1081	2057	0
1082	102 1	
1082	134 1	
1082	527 1	
1082	114 1	
1082	188 1	
1082	52 1	
1082	756 1	
1082	119 1	
1082	2199	1
1082	1401	1

1082	1466	1
1082	124	1
1082	311	0
1082	2141	0
1082	3655	0
1082	1048	0
1082	609	0
1082	1949	0
1082	335	0
1082	240	0
1082	267	0
1082	1192	0
1082	3439	0
1082	3221	0
1083	1145	1
1083	1188	1
1083	901	1
1083	377	1
1083	2189	1
1083	206	1
1083	79	1
1083	399	1
1083	2935	1
1083	115	1
1083	2936	1
1083	437	1
1083	406	1
1083	919	1
1083	184	1
1083	185	1

1083	1143	1
1083	2485	1
1083	3564	0
1083	550	0
1083	3495	0
1083	2651	0
1083	3031	0
1083	2406	0
1083	3570	0
1083	3761	0
1083	254	0
1083	1566	0
1083	1132	0
1083	3612	0
1083	1860	0
1083	2123	0
1083	3523	0
1083	3782	0
1083	264	0
1083	1504	0
1084	98	1
1084	131	1
1084	137	1
1084	78	1
1084	79	1
1084	80	1
1084	46	1
1084	47	1
1084	117	1
1084	185	1

1084	923	1
1084	572	1
1084	2008	0
1084	3434	0
1084	1727	0
1084	3544	0
1084	3070	0
1084	974	0
1084	3074	0
1084	3390	0
1084	2984	0
1084	315	0
1084	3725	0
1084	375	0
1085	36	1
1085	1989	1
1085	391	1
1085	141	1
1085	718	1
1085	2096	1
1085	670	1
1085	191	1
1085	2720	0
1085	80	0
1085	2703	0
1085	1956	0
1085	1891	0
1085	296	0
1085	2198	0
1085	1770	0

1086	97	1
1086	36	1
1086	294	1
1086	52	1
1086	55	1
1086	221	1
1086	2937	1
1086	189	1
1086	1662	1
1086	1573	0
1086	2542	0
1086	3291	0
1086	1713	0
1086	1656	0
1086	3313	0
1086	3564	0
1086	3066	0
1086	3317	0
1087	161	1
1087	293	1
1087	22	1
1087	887	1
1087	155	1
1087	1181	1
1087	449	0
1087	2718	0
1087	1600	0
1087	1807	0
1087	2171	0
1087	2686	0

1088	795	1
1088	2593	1
1088	418	1
1088	419	1
1088	196	1
1088	422	1
1088	519	1
1088	843	1
1088	846	1
1088	53	1
1088	1372	1
1088	1528	1
1088	155	1
1088	156	1
1088	510	1
1088	514	0
1088	1753	0
1088	1213	0
1088	1584	0
1088	1498	0
1088	1545	0
1088	2716	0
1088	3065	0
1088	3748	0
1088	1675	0
1088	2698	0
1088	1424	0
1088	756	0
1088	1691	0
1088	2580	0

1089	608	1
1089	36	1
1089	141	1
1089	558	1
1089	1453	1
1089	306	1
1089	250	1
1089	2939	1
1089	2938	1
1089	432	0
1089	3249	0
1089	1655	0
1089	364	0
1089	3450	0
1089	2749	0
1089	2484	0
1089	1769	0
1089	69	0
1090	102	1
1090	2504	1
1090	298	1
1090	243	1
1090	52	1
1090	2940	1
1090	2941	1
1090	2942	1
1090	3339	0
1090	1888	0
1090	436	0
1090	3239	0

1090	266	0
1090	811	0
1090	1510	0
1090	3827	0
1091	2944	1
1091	1057	1
1091	1667	1
1091	40	1
1091	275	1
1091	501	1
1091	26	1
1091	28	1
1091	2943	1
1091	2860	0
1091	2561	0
1091	1017	0
1091	3826	0
1091	1779	0
1091	1325	0
1091	146	0
1091	3465	0
1091	1899	0
1092	256	1
1092	257	1
1092	1057	1
1092	1571	1
1092	499	1
1092	883	1
1092	26	1
1092	1436	0

1092	584	0
1092	616	0
1092	2468	0
1092	1526	0
1092	964	0
1092	1724	0
1093	257	1
1093	2945	1
1093	2946	1
1093	932	1
1093	2947	1
1093	2948	1
1093	1677	1
1093	882	1
1093	883	1
1093	277	1
1093	1047	1
1093	2649	1
1093	2980	0
1093	3203	0
1093	1409	0
1093	852	0
1093	1403	0
1093	1947	0
1093	1797	0
1093	821	0
1093	3075	0
1093	2553	0
1093	3816	0
1093	658	0

1094	36	1
1094	2949	1
1094	2463	1
1094	235	1
1094	109	1
1094	48	1
1094	52	1
1094	53	1
1094	22	1
1094	1144	1
1094	670	1
1094	1535	1
1094	1501	0
1094	2817	0
1094	3268	0
1094	487	0
1094	394	0
1094	703	0
1094	1669	0
1094	429	0
1094	2215	0
1094	3447	0
1094	1071	0
1094	2841	0
1095	2950	1
1095	329	1
1095	265	1
1095	2506	1
1095	332	1
1095	13	1

1095	191 1	
1095	1150	0
1095	3040	0
1095	3631	0
1095	2206	0
1095	3553	0
1095	3094	0
1095	1550	0
1096	2114	1
1096	804 1	
1096	871 1	
1096	2951	1
1096	1523	1
1096	216 1	
1096	1980	1
1096	96 0	
1096	3189	0
1096	2722	0
1096	824 0	
1096	2104	0
1096	1126	0
1096	1859	0
1097	832 1	
1097	1218	1
1097	389 1	
1097	2598	1
1097	2952	1
1097	233 1	
1097	106 1	
1097	2953	1

1097	2131	1
1097	1141	1
1097	2327	1
1097	252 1	
1097	1469	1
1097	2470	0
1097	1078	0
1097	351 0	
1097	2191	0
1097	669 0	
1097	3420	0
1097	3506	0
1097	1107	0
1097	1977	0
1097	814 0	
1097	1847	0
1097	569 0	
1097	3277	0
1098	2116	1
1098	102 1	
1098	78 1	
1098	47 1	
1098	1594	1
1098	1589	1
1098	118 1	
1098	55 1	
1098	1144	1
1098	282 1	
1098	575 1	
1098	621 0	

1098	1709	0
1098	140 0	
1098	2322	0
1098	3615	0
1098	411 0	
1098	3321	0
1098	135 0	
1098	3371	0
1098	1469	0
1098	2653	0
1099	1440	1
1099	353 1	
1099	1218	1
1099	1851	1
1099	2020	1
1099	2627	1
1099	1257	1
1099	2954	1
1099	235 1	
1099	2955	1
1099	2956	1
1099	878 1	
1099	2065	1
1099	1395	1
1099	1587	1
1099	378 1	
1099	91 1	
1099	1152	0
1099	339 0	
1099	686 0	

1099	3641	0
1099	278	0
1099	815	0
1099	136	0
1099	1534	0
1099	3425	0
1099	1306	0
1099	1064	0
1099	2415	0
1099	1081	0
1099	393	0
1099	1299	0
1099	1169	0
1099	10	0
1100	192	1
1100	385	1
1100	132	1
1100	41	1
1100	46	1
1100	2064	1
1100	53	1
1100	22	1
1100	252	1
1100	3347	0
1100	3732	0
1100	1204	0
1100	1013	0
1100	170	0
1100	2336	0
1100	610	0

1100	3550	0
1100	617	0
1101	261	1
1101	230	1
1101	40	1
1101	361	1
1101	107	1
1101	109	1
1101	110	1
1101	399	1
1101	1325	1
1101	22	1
1101	55	1
1101	24	1
1101	1174	1
1101	1308	1
1101	957	1
1101	3450	0
1101	750	0
1101	873	0
1101	2755	0
1101	321	0
1101	369	0
1101	2681	0
1101	207	0
1101	200	0
1101	1224	0
1101	2282	0
1101	2927	0
1101	3421	0

1101	1235	0
1101	2518	0
1102	102	1
1102	2855	1
1102	2957	1
1102	2958	1
1102	1135	1
1102	2959	1
1102	2960	1
1102	2961	1
1102	2654	1
1102	2583	0
1102	37	0
1102	3125	0
1102	1605	0
1102	1901	0
1102	793	0
1102	1502	0
1102	1415	0
1102	3650	0
1103	72	1
1103	2698	1
1103	237	1
1103	80	1
1103	115	1
1103	116	1
1103	276	1
1103	87	1
1103	88	1
1103	377	1

1103	539	1
1103	2933	0
1103	596	0
1103	1723	0
1103	666	0
1103	3156	0
1103	3185	0
1103	2053	0
1103	3647	0
1103	979	0
1103	3052	0
1103	430	0
1104	2307	1
1104	36	1
1104	1325	1
1104	1854	1
1104	465	1
1104	2577	1
1104	147	1
1104	788	1
1104	1982	1
1104	2962	1
1104	55	1
1104	30	1
1104	1260	0
1104	3549	0
1104	3390	0
1104	865	0
1104	3585	0
1104	679	0

1104	1985	0
1104	845 0	
1104	2995	0
1104	215 0	
1104	1109	0
1104	3075	0
1105	774 1	
1105	2732	1
1105	2764	1
1105	785 1	
1105	146 1	
1105	147 1	
1105	788 1	
1105	1140	1
1105	2963	1
1105	1719	1
1105	1944	1
1105	2964	1
1105	2781	1
1105	2982	0
1105	2700	0
1105	2269	0
1105	1026	0
1105	591 0	
1105	3336	0
1105	1631	0
1105	1742	0
1105	186 0	
1105	2478	0
1105	286 0	

1105	16	0
1105	929	0
1106	20	1
1106	2965	1
1106	24	1
1106	221	1
1106	126	1
1106	445	1
1106	3341	0
1106	3371	0
1106	352	0
1106	2689	0
1106	715	0
1106	1603	0
1107	96	1
1107	66	1
1107	548	1
1107	134	1
1107	74	1
1107	2967	1
1107	2966	1
1107	183	1
1107	426	0
1107	1540	0
1107	1464	0
1107	759	0
1107	2614	0
1107	1686	0
1107	3031	0
1107	521	0

1108	928	1
1108	961	1
1108	34	1
1108	195	1
1108	100	1
1108	36	1
1108	228	1
1108	362	1
1108	1514	1
1108	315	1
1108	735	1
1108	52	1
1108	1528	1
1108	1530	1
1108	155	1
1108	511	1
1108	191	0
1108	3183	0
1108	1835	0
1108	2687	0
1108	2811	0
1108	874	0
1108	2074	0
1108	2756	0
1108	2690	0
1108	143	0
1108	1588	0
1108	3144	0
1108	1839	0
1108	1836	0

1108	1705	0
1108	1702	0
1109	129	1
1109	899	1
1109	261	1
1109	2504	1
1109	366	1
1109	1558	1
1109	253	1
1109	254	1
1109	691	0
1109	1489	0
1109	1253	0
1109	3023	0
1109	3359	0
1109	3204	0
1109	2627	0
1109	672	0
1110	1672	1
1110	459	1
1110	51	1
1110	468	1
1110	2968	1
1110	2969	1
1110	2970	1
1110	2971	1
1110	2972	1
1110	959	1
1110	3102	0
1110	1723	0

1110	1006	0
1110	2472	0
1110	596	0
1110	1963	0
1110	358	0
1110	1904	0
1110	2785	0
1110	3820	0
1111	2274	1
1111	36	1
1111	788	1
1111	30	1
1111	2973	1
1111	478	1
1111	1311	1
1111	1597	0
1111	1055	0
1111	2421	0
1111	2660	0
1111	1777	0
1111	3013	0
1111	3782	0
1112	1220	1
1112	293	1
1112	1292	1
1112	13	1
1112	498	1
1112	883	1
1112	799	1
1112	277	1

1112	947	1
1112	1492	1
1112	1493	1
1112	2975	1
1112	445	1
1112	2974	1
1112	223	1
1112	1736	0
1112	774	0
1112	465	0
1112	2281	0
1112	3744	0
1112	771	0
1112	485	0
1112	797	0
1112	3630	0
1112	1074	0
1112	350	0
1112	2686	0
1112	1650	0
1112	2994	0
1112	1370	0
1113	2976	1
1113	2977	1
1113	98	1
1113	131	1
1113	132	1
1113	1336	1
1113	230	1
1113	2978	1

1113	2979	1
1113	361	1
1113	74	1
1113	2155	1
1113	237	1
1113	80	1
1113	919	1
1113	24	1
1113	1114	1
1113	1813	0
1113	1925	0
1113	3489	0
1113	3763	0
1113	1989	0
1113	386	0
1113	902	0
1113	2504	0
1113	2875	0
1113	3183	0
1113	1782	0
1113	473	0
1113	3117	0
1113	3079	0
1113	1407	0
1113	9	0
1113	866	0
1114	2980	1
1114	38	1
1114	499	1
1114	915	1

1114	2260	1
1114	118	1
1114	956	1
1114	254	1
1114	2608	0
1114	3218	0
1114	1221	0
1114	2952	0
1114	2413	0
1114	3161	0
1114	1044	0
1114	2805	0
1115	96	1
1115	1826	1
1115	36	1
1115	2981	1
1115	2823	1
1115	1353	1
1115	1777	1
1115	151	1
1115	2648	1
1115	155	1
1115	3491	0
1115	3056	0
1115	866	0
1115	2199	0
1115	3807	0
1115	1530	0
1115	1357	0
1115	1820	0

1115	2888	0
1115	2478	0
1116	96	1
1116	358	1
1116	679	1
1116	136	1
1116	361	1
1116	682	1
1116	653	1
1116	1328	1
1116	210	1
1116	114	1
1116	211	1
1116	242	1
1116	2551	1
1116	286	1
1116	2630	0
1116	2051	0
1116	2725	0
1116	1296	0
1116	1399	0
1116	3595	0
1116	2283	0
1116	406	0
1116	2189	0
1116	2436	0
1116	3224	0
1116	1659	0
1116	988	0
1116	1654	0

1117	389	1
1117	294	1
1117	295	1
1117	2982	1
1117	2983	1
1117	74	1
1117	46	1
1117	53	1
1117	2937	1
1117	1933	0
1117	2912	0
1117	971	0
1117	3458	0
1117	2320	0
1117	385	0
1117	3608	0
1117	1957	0
1117	2500	0
1118	1345	1
1118	1347	1
1118	36	1
1118	2245	1
1118	2984	1
1118	2058	1
1118	109	1
1118	110	1
1118	143	1
1118	55	1
1118	895	1
1118	2029	0

1118	2727	0
1118	2706	0
1118	2689	0
1118	1641	0
1118	3518	0
1118	1206	0
1118	1278	0
1118	1104	0
1118	991	0
1118	1744	0
1119	34	1
1119	2434	1
1119	36	1
1119	37	1
1119	105	1
1119	42	1
1119	2985	1
1119	2986	1
1119	45	1
1119	109	1
1119	52	1
1119	469	1
1119	55	1
1119	252	1
1119	1662	1
1119	1584	0
1119	2871	0
1119	1685	0
1119	1765	0
1119	2569	0

1119	1074	0
1119	2098	0
1119	2341	0
1119	2841	0
1119	1588	0
1119	656	0
1119	2234	0
1119	2298	0
1119	2496	0
1119	2745	0
1120	769	1
1120	388	1
1120	1802	1
1120	141	1
1120	1805	1
1120	35	1
1120	36	1
1120	165	1
1120	2597	1
1120	2987	1
1120	2988	1
1120	2989	1
1120	46	1
1120	2990	1
1120	2741	1
1120	438	1
1120	695	1
1120	1514	1
1120	107	1
1120	882	1

1120	79	0
1120	858	0
1120	248	0
1120	911	0
1120	2706	0
1120	519	0
1120	2787	0
1120	3791	0
1120	116	0
1120	972	0
1120	2131	0
1120	3433	0
1120	1729	0
1120	3505	0
1120	2991	0
1120	969	0
1120	3307	0
1120	2673	0
1120	3573	0
1120	3531	0
1121	771	1
1121	388	1
1121	36	1
1121	446	1
1121	1992	1
1121	2120	1
1121	45	1
1121	654	1
1121	2991	1
1121	1042	1

1121	52	1
1121	438	1
1121	2648	1
1121	286	1
1121	1712	0
1121	3567	0
1121	817	0
1121	3233	0
1121	3354	0
1121	2632	0
1121	1633	0
1121	3350	0
1121	400	0
1121	665	0
1121	3	0
1121	910	0
1121	2014	0
1121	531	0
1122	1342	1
1122	102	1
1122	1832	1
1122	105	1
1122	109	1
1122	1133	1
1122	114	1
1122	2199	1
1122	477	1
1122	286	1
1122	394	0
1122	837	0

1122	930	0
1122	1356	0
1122	3320	0
1122	3555	0
1122	2726	0
1122	360	0
1122	1223	0
1122	417	0
1123	2597	1
1123	2890	1
1123	46	1
1123	2719	1
1123	2992	1
1123	2993	1
1123	1235	1
1123	2994	1
1123	2517	1
1123	221	1
1123	1343	1
1123	395	0
1123	3168	0
1123	3127	0
1123	1441	0
1123	485	0
1123	3107	0
1123	186	0
1123	481	0
1123	3434	0
1123	1227	0
1123	881	0

1124	833	1
1124	2116	1
1124	906	1
1124	1020	1
1124	47	1
1124	305	1
1124	2995	1
1124	1556	1
1124	2996	1
1124	118	1
1124	55	1
1124	1144	1
1124	282	1
1124	764	1
1124	1995	0
1124	1541	0
1124	1121	0
1124	2330	0
1124	2371	0
1124	1718	0
1124	1715	0
1124	1842	0
1124	431	0
1124	52	0
1124	2253	0
1124	2156	0
1124	3468	0
1124	511	0
1125	673	1
1125	132	1

1125	41	1
1125	2893	1
1125	558	1
1125	2414	1
1125	1073	1
1125	2997	1
1125	2998	1
1125	2999	1
1125	24	1
1125	3000	1
1125	3001	1
1125	659	0
1125	1763	0
1125	492	0
1125	2382	0
1125	3742	0
1125	758	0
1125	928	0
1125	2045	0
1125	2336	0
1125	2614	0
1125	2017	0
1125	2553	0
1125	1477	0
1126	66	1
1126	98	1
1126	130	1
1126	74	1
1126	79	1
1126	80	1

1126	919	1
1126	438	1
1126	87	1
1126	184	1
1126	185	1
1126	3002	1
1126	1277	1
1126	263	0
1126	3772	0
1126	1184	0
1126	1773	0
1126	549	0
1126	3272	0
1126	1160	0
1126	3147	0
1126	3333	0
1126	2025	0
1126	3504	0
1126	994	0
1126	3414	0
1127	832	1
1127	1026	1
1127	68	1
1127	900	1
1127	2598	1
1127	75	1
1127	399	1
1127	145	1
1127	3003	1
1127	3004	1

1127	1469	1
1127	108 0	
1127	1258	0
1127	674 0	
1127	999 0	
1127	3204	0
1127	2885	0
1127	2344	0
1127	260 0	
1127	312 0	
1127	1666	0
1127	3844	0
1128	897 1	
1128	2194	1
1128	277 1	
1128	36 1	
1128	941 1	
1128	307 1	
1128	53 1	
1128	60 1	
1128	3005	1
1128	3006	1
1128	3007	1
1128	3008	1
1128	959 1	
1128	590 1	
1128	2771	1
1128	227 1	
1128	867 1	
1128	2804	1

1128	887	1
1128	1557	0
1128	3781	0
1128	3061	0
1128	229	0
1128	1759	0
1128	1503	0
1128	1781	0
1128	374	0
1128	3705	0
1128	2549	0
1128	831	0
1128	2574	0
1128	1074	0
1128	3732	0
1128	1305	0
1128	3503	0
1128	764	0
1128	1897	0
1128	1869	0
1129	1186	1
1129	134	1
1129	135	1
1129	171	1
1129	267	1
1129	1230	1
1129	79	1
1129	560	1
1129	1424	1
1129	87	1

1129	2126	0
1129	255 0	
1129	3025	0
1129	2022	0
1129	3834	0
1129	3658	0
1129	3350	0
1129	3460	0
1129	1578	0
1129	362 0	
1130	928 1	
1130	34 1	
1130	35 1	
1130	42 1	
1130	1721	1
1130	2492	1
1130	1217	1
1130	3009	1
1130	3010	1
1130	196 1	
1130	3011	1
1130	2505	1
1130	211 1	
1130	2009	1
1130	735 1	
1130	622 1	
1130	623 1	
1130	624 1	
1130	630 1	
1130	1659	0

1130	753	0
1130	1702	0
1130	2083	0
1130	2576	0
1130	2945	0
1130	2218	0
1130	2687	0
1130	2398	0
1130	3193	0
1130	33	0
1130	86	0
1130	1794	0
1130	3764	0
1130	2674	0
1130	2475	0
1130	3413	0
1130	2366	0
1130	2825	0
1131	104	1
1131	107	1
1131	109	1
1131	45	1
1131	110	1
1131	111	1
1131	863	1
1131	252	1
1131	127	1
1131	291	0
1131	924	0
1131	243	0

1131	2221	0
1131	1476	0
1131	370 0	
1131	2263	0
1131	598 0	
1131	1537	0
1132	1440	1
1132	3012	1
1132	613 1	
1132	3013	1
1132	3014	1
1132	904 1	
1132	3015	1
1132	1130	1
1132	3016	1
1132	3017	1
1132	989 1	
1132	3700	0
1132	198 0	
1132	1967	0
1132	998 0	
1132	2984	0
1132	3411	0
1132	2512	0
1132	430 0	
1132	2873	0
1132	3224	0
1132	2431	0
1133	224 1	
1133	164 1	

1133	932	1
1133	3018	1
1133	941	1
1133	686	1
1133	496	1
1133	277	1
1133	1115	1
1133	221	1
1133	799	1
1133	3710	0
1133	2851	0
1133	2182	0
1133	131	0
1133	1473	0
1133	1518	0
1133	3614	0
1133	2540	0
1133	1215	0
1133	271	0
1133	1544	0
1134	1538	1
1134	47	1
1134	1395	1
1134	756	1
1134	118	1
1134	504	1
1134	3226	0
1134	2100	0
1134	3546	0
1134	2472	0

1134	1352	0
1134	2666	0
1135	2507	1
1135	45	1
1135	469	1
1135	22	1
1135	55	1
1135	56	1
1135	57	1
1135	1461	1
1135	187	1
1135	61	1
1135	159	1
1135	3371	0
1135	1410	0
1135	2444	0
1135	3276	0
1135	1302	0
1135	3476	0
1135	475	0
1135	3658	0
1135	1257	0
1135	625	0
1135	2304	0
1136	899	1
1136	3019	1
1136	3020	1
1136	2804	1
1136	437	1
1136	2806	1

1136	2810	1
1136	1372	1
1136	2814	1
1136	447	0
1136	3358	0
1136	1120	0
1136	610	0
1136	1921	0
1136	2135	0
1136	1468	0
1136	1998	0
1136	3637	0
1137	96	1
1137	167	1
1137	203	1
1137	3021	1
1137	78	1
1137	79	1
1137	1040	1
1137	721	1
1137	1231	1
1137	2071	1
1137	1820	1
1137	1821	1
1137	878	0
1137	2812	0
1137	1260	0
1137	61	0
1137	1927	0
1137	819	0

1137	2383	0
1137	1220	0
1137	496	0
1137	2670	0
1137	985	0
1137	1150	0
1138	191	1
1138	36	1
1138	46	1
1138	52	1
1138	1210	0
1138	3123	0
1138	127	0
1138	1400	0
1139	35	1
1139	708	1
1139	165	1
1139	197	1
1139	732	1
1139	1545	1
1139	110	1
1139	46	1
1139	462	1
1139	1969	1
1139	3022	1
1139	24	1
1139	1114	1
1139	572	1
1139	626	0
1139	3825	0

1139	3101	0
1139	2914	0
1139	357	0
1139	2149	0
1139	1605	0
1139	586	0
1139	1227	0
1139	883	0
1139	3140	0
1139	2571	0
1139	319	0
1139	1341	0
1140	36	1
1140	102	1
1140	42	1
1140	47	1
1140	3023	1
1140	243	1
1140	1523	1
1140	250	1
1140	1860	0
1140	467	0
1140	535	0
1140	1656	0
1140	3013	0
1140	2108	0
1140	722	0
1140	987	0
1141	128	1
1141	353	1

1141	133	1
1141	102	1
1141	998	1
1141	776	1
1141	41	1
1141	3024	1
1141	1589	1
1141	280	1
1141	282	1
1141	989	1
1141	517	0
1141	3573	0
1141	958	0
1141	1137	0
1141	3546	0
1141	1094	0
1141	3162	0
1141	3268	0
1141	393	0
1141	2119	0
1141	484	0
1141	2723	0
1142	608	1
1142	2671	1
1142	1105	1
1142	3025	1
1142	3026	1
1142	3027	1
1142	3028	1
1142	1754	1

1142	1851	1
1142	2686	1
1142	3538	0
1142	132 0	
1142	1570	0
1142	1435	0
1142	542 0	
1142	3592	0
1142	2332	0
1142	2519	0
1142	1298	0
1142	3450	0
1143	133 1	
1143	102 1	
1143	171 1	
1143	267 1	
1143	78 1	
1143	47 1	
1143	118 1	
1143	55 1	
1143	184 1	
1143	185 1	
1143	181 0	
1143	1781	0
1143	678 0	
1143	626 0	
1143	2609	0
1143	1648	0
1143	1485	0
1143	1290	0

1143	1022	0
1143	1423	0
1144	545	1
1144	355	1
1144	100	1
1144	4	1
1144	197	1
1144	446	1
1144	1700	1
1144	334	1
1144	1583	1
1144	1809	1
1144	3029	1
1144	55	1
1144	857	1
1144	858	1
1144	254	1
1144	383	1
1144	166	0
1144	186	0
1144	2807	0
1144	2258	0
1144	352	0
1144	1043	0
1144	174	0
1144	3491	0
1144	2622	0
1144	3711	0
1144	3608	0
1144	3118	0

1144	1943	0
1144	658	0
1144	3239	0
1144	3042	0
1145	36	1
1145	1989	1
1145	788	1
1145	52	1
1145	3030	1
1145	2050	0
1145	2695	0
1145	1097	0
1145	2408	0
1145	115	0
1146	97	1
1146	36	1
1146	104	1
1146	105	1
1146	141	1
1146	46	1
1146	110	1
1146	2703	1
1146	850	1
1146	3031	1
1146	22	1
1146	55	1
1146	1174	1
1146	2528	0
1146	3481	0
1146	332	0

1146	1204	0
1146	1350	0
1146	109 0	
1146	2858	0
1146	1354	0
1146	2648	0
1146	474 0	
1146	2342	0
1146	768 0	
1146	2260	0
1147	705 1	
1147	102 1	
1147	3035	1
1147	3036	1
1147	3032	1
1147	3033	1
1147	3034	1
1147	827 1	
1147	2748	1
1147	511 1	
1147	3844	0
1147	57 0	
1147	2448	0
1147	87 0	
1147	2721	0
1147	1932	0
1147	1778	0
1147	361 0	
1147	3543	0
1147	3361	0

1148	133	1
1148	41	1
1148	105	1
1148	235	1
1148	236	1
1148	267	1
1148	46	1
1148	878	1
1148	1103	1
1148	114	1
1148	2163	1
1148	52	1
1148	53	1
1148	951	1
1148	3532	0
1148	1280	0
1148	3338	0
1148	3115	0
1148	3034	0
1148	1199	0
1148	667	0
1148	291	0
1148	1168	0
1148	1477	0
1148	1756	0
1148	3535	0
1148	2319	0
1148	3105	0
1149	1889	1
1149	130	1

1149	1410	1
1149	2083	1
1149	261	1
1149	522	1
1149	269	1
1149	79	1
1149	80	1
1149	81	1
1149	561	1
1149	376	1
1149	2269	1
1149	2561	0
1149	3232	0
1149	2533	0
1149	911	0
1149	3221	0
1149	258	0
1149	3066	0
1149	3277	0
1149	1334	0
1149	460	0
1149	103	0
1149	1724	0
1149	1829	0
1150	1218	1
1150	197	1
1150	165	1
1150	1031	1
1150	456	1
1150	41	1

1150	554	1
1150	46	1
1150	462	1
1150	2102	1
1150	1496	1
1150	3037	1
1150	170	0
1150	2730	0
1150	1001	0
1150	2553	0
1150	596	0
1150	2247	0
1150	1888	0
1150	491	0
1150	3787	0
1150	213	0
1150	3306	0
1150	161	0
1151	132	1
1151	2347	1
1151	237	1
1151	16	1
1151	116	1
1151	53	1
1151	22	1
1151	407	1
1151	3038	1
1151	221	1
1151	190	1
1151	1813	0

1151	3104	0
1151	414 0	
1151	2506	0
1151	1672	0
1151	636 0	
1151	918 0	
1151	2838	0
1151	3049	0
1151	2693	0
1151	1701	0
1152	34 1	
1152	42 1	
1152	756 1	
1152	439 1	
1152	2199	1
1152	286 1	
1152	3039	1
1152	2709	0
1152	789 0	
1152	3758	0
1152	2528	0
1152	1439	0
1152	160 0	
1152	1002	0
1153	353 1	
1153	136 1	
1153	2030	1
1153	498 1	
1153	1018	1
1153	891 1	

1153	445	1
1153	382	1
1153	2012	0
1153	2207	0
1153	1971	0
1153	2020	0
1153	3062	0
1153	1492	0
1153	2255	0
1153	1068	0
1154	377	1
1154	75	1
1154	78	1
1154	79	1
1154	115	1
1154	406	1
1154	87	1
1154	184	1
1154	185	1
1154	91	1
1154	3689	0
1154	2513	0
1154	2925	0
1154	1039	0
1154	3683	0
1154	571	0
1154	2157	0
1154	1335	0
1154	3494	0
1154	3711	0

1155	2113	1
1155	732	1
1155	196	1
1155	519	1
1155	200	1
1155	1384	1
1155	690	1
1155	275	1
1155	2579	1
1155	727	1
1155	314	1
1155	155	1
1155	156	1
1155	2126	0
1155	1711	0
1155	170	0
1155	652	0
1155	2524	0
1155	2351	0
1155	1267	0
1155	577	0
1155	1768	0
1155	2665	0
1155	291	0
1155	3292	0
1155	1966	0
1156	3041	1
1156	516	1
1156	293	1
1156	1416	1

1156	46	1
1156	882	1
1156	883	1
1156	888	1
1156	2587	1
1156	280	0
1156	220	0
1156	3228	0
1156	471	0
1156	2161	0
1156	3749	0
1156	3171	0
1156	2485	0
1156	302	0
1157	1	1
1157	3042	1
1157	3	1
1157	771	1
1157	1446	1
1157	7	1
1157	871	1
1157	393	1
1157	746	1
1157	2172	1
1157	242	1
1157	18	1
1157	1554	1
1157	1907	1
1157	2235	1
1157	1148	1

1157	92	0
1157	729	0
1157	2014	0
1157	2764	0
1157	3830	0
1157	1403	0
1157	1179	0
1157	531	0
1157	3436	0
1157	3026	0
1157	2110	0
1157	428	0
1157	1968	0
1157	2871	0
1157	3167	0
1157	2868	0
1158	608	1
1158	899	1
1158	3043	1
1158	3044	1
1158	3045	1
1158	520	1
1158	41	1
1158	46	1
1158	54	1
1158	2744	1
1158	507	1
1158	60	1
1158	1705	0
1158	308	0

1158	477	0
1158	1755	0
1158	1572	0
1158	1531	0
1158	1249	0
1158	506	0
1158	382	0
1158	1228	0
1158	2023	0
1158	3310	0
1159	512	1
1159	610	1
1159	196	1
1159	165	1
1159	516	1
1159	517	1
1159	511	1
1159	111	1
1159	1712	1
1159	1586	1
1159	53	1
1159	56	1
1159	57	1
1159	158	1
1159	159	1
1159	1591	0
1159	3178	0
1159	2124	0
1159	245	0
1159	2846	0

1159	116	0
1159	3534	0
1159	215	0
1159	3608	0
1159	3017	0
1159	27	0
1159	781	0
1159	1335	0
1159	1411	0
1159	1399	0
1160	832	1
1160	353	1
1160	3046	1
1160	106	1
1160	1494	1
1160	1590	1
1160	91	1
1160	1181	1
1160	1299	0
1160	1046	0
1160	457	0
1160	238	0
1160	1560	0
1160	3344	0
1160	1921	0
1160	2237	0
1161	32	1
1161	66	1
1161	3047	1
1161	40	1

1161	1325	1
1161	1231	1
1161	243 1	
1161	276 1	
1161	1461	1
1161	187 1	
1161	252 1	
1161	681 0	
1161	3324	0
1161	1212	0
1161	2178	0
1161	230 0	
1161	1920	0
1161	3155	0
1161	2096	0
1161	616 0	
1161	3416	0
1161	310 0	
1162	1153	1
1162	1154	1
1162	901 1	
1162	3048	1
1162	270 1	
1162	79 1	
1162	115 1	
1162	406 1	
1162	2935	1
1162	184 1	
1162	3726	0
1162	1272	0

1162	2634	0
1162	1161	0
1162	1897	0
1162	3751	0
1162	861	0
1162	1109	0
1162	3666	0
1162	2	0
1163	802	1
1163	1315	1
1163	902	1
1163	494	1
1163	47	1
1163	754	1
1163	55	1
1163	1144	1
1163	126	1
1163	1829	0
1163	1159	0
1163	3801	0
1163	3535	0
1163	3432	0
1163	113	0
1163	1632	0
1163	2835	0
1163	1369	0
1164	1153	1
1164	583	1
1164	76	1
1164	78	1

1164	47	1
1164	2159	1
1164	184	1
1164	185	1
1164	127	1
1164	2586	0
1164	1624	0
1164	3634	0
1164	1350	0
1164	2694	0
1164	847	0
1164	3622	0
1164	2734	0
1164	3284	0
1165	608	1
1165	2434	1
1165	35	1
1165	36	1
1165	165	1
1165	102	1
1165	391	1
1165	518	1
1165	105	1
1165	46	1
1165	187	1
1165	1704	0
1165	236	0
1165	161	0
1165	358	0
1165	1444	0

1165	3559	0
1165	640 0	
1165	1298	0
1165	3363	0
1165	1738	0
1165	3581	0
1166	34 1	
1166	803 1	
1166	36 1	
1166	7 1	
1166	362 1	
1166	1290	1
1166	655 1	
1166	211 1	
1166	216 1	
1166	250 1	
1166	347 1	
1166	1214	1
1166	286 1	
1166	2074	0
1166	3458	0
1166	3711	0
1166	1935	0
1166	3475	0
1166	805 0	
1166	3681	0
1166	2048	0
1166	1359	0
1166	1444	0
1166	3601	0

1166	1523	0
1166	2662	0
1167	1889	1
1167	98	1
1167	2297	1
1167	377	1
1167	74	1
1167	77	1
1167	237	1
1167	1651	1
1167	405	1
1167	919	1
1167	185	1
1167	2854	0
1167	1515	0
1167	925	0
1167	1802	0
1167	625	0
1167	3286	0
1167	2972	0
1167	3514	0
1167	3198	0
1167	1506	0
1167	828	0
1168	1696	1
1168	132	1
1168	36	1
1168	134	1
1168	3049	1
1168	171	1

1168	238	1
1168	126	1
1168	510	0
1168	2993	0
1168	3784	0
1168	1332	0
1168	2047	0
1168	964	0
1168	2761	0
1168	530	0
1169	72	1
1169	137	1
1169	3050	1
1169	237	1
1169	399	1
1169	87	1
1169	539	1
1169	540	1
1169	254	1
1169	127	1
1169	3126	0
1169	1866	0
1169	952	0
1169	2978	0
1169	2306	0
1169	3418	0
1169	1873	0
1169	987	0
1169	3043	0
1169	3261	0

1170	66	1
1170	132	1
1170	237	1
1170	79	1
1170	863	1
1170	87	1
1170	539	1
1170	540	1
1170	254	1
1170	127	1
1170	3057	0
1170	3208	0
1170	3412	0
1170	928	0
1170	1435	0
1170	1738	0
1170	1124	0
1170	1207	0
1170	2895	0
1170	3567	0
1171	165	1
1171	389	1
1171	391	1
1171	105	1
1171	45	1
1171	399	1
1171	467	1
1171	1396	1
1171	190	1
1171	223	1

1171	3400	0
1171	2618	0
1171	3178	0
1171	1488	0
1171	2550	0
1171	339	0
1171	3487	0
1171	3601	0
1171	299	0
1171	3772	0
1172	471	1
1172	35	1
1172	388	1
1172	165	1
1172	391	1
1172	105	1
1172	3051	1
1172	109	1
1172	46	1
1172	51	1
1172	1174	1
1172	55	1
1172	154	1
1172	2763	0
1172	1013	0
1172	2885	0
1172	3113	0
1172	3495	0
1172	959	0
1172	3012	0

1172	1753	0
1172	1796	0
1172	376	0
1172	1693	0
1172	718	0
1172	2477	0
1173	98	1
1173	135	1
1173	171	1
1173	363	1
1173	364	1
1173	365	1
1173	3052	1
1173	81	1
1173	374	1
1173	185	1
1173	538	1
1173	695	0
1173	3666	0
1173	469	0
1173	1039	0
1173	2285	0
1173	576	0
1173	733	0
1173	3812	0
1173	2831	0
1173	559	0
1173	2131	0
1174	135	1
1174	73	1

1174	75	1
1174	77	1
1174	81	1
1174	145	1
1174	374	1
1174	87	1
1174	185	1
1174	152	0
1174	791	0
1174	2408	0
1174	1454	0
1174	2087	0
1174	2369	0
1174	3087	0
1174	1401	0
1174	1933	0
1175	66	1
1175	72	1
1175	75	1
1175	79	1
1175	87	1
1175	377	1
1175	91	1
1175	540	1
1175	126	1
1175	127	1
1175	3249	0
1175	240	0
1175	1628	0
1175	504	0

1175	807	0
1175	325	0
1175	559	0
1175	3584	0
1175	1411	0
1175	2401	0
1176	224	1
1176	1088	1
1176	1091	1
1176	228	1
1176	36	1
1176	1416	1
1176	523	1
1176	109	1
1176	766	1
1176	3054	1
1176	3055	1
1176	3056	1
1176	306	1
1176	3057	1
1176	52	1
1176	277	1
1176	286	1
1176	2609	0
1176	519	0
1176	2345	0
1176	2469	0
1176	2007	0
1176	1652	0
1176	552	0

1176	3471	0
1176	2256	0
1176	2912	0
1176	3675	0
1176	3396	0
1176	3225	0
1176	3305	0
1176	3583	0
1176	925 0	
1176	2235	0
1177	608 1	
1177	1952	1
1177	2372	1
1177	613 1	
1177	933 1	
1177	232 1	
1177	74 1	
1177	363 1	
1177	568 1	
1177	1875	1
1177	184 1	
1177	764 1	
1177	127 1	
1177	1599	0
1177	2359	0
1177	2661	0
1177	3024	0
1177	1457	0
1177	2889	0
1177	79 0	

1177	3582	0
1177	2090	0
1177	2155	0
1177	567	0
1177	3031	0
1177	3020	0
1178	2084	1
1178	3003	1
1178	2566	1
1178	1612	1
1178	77	1
1178	81	1
1178	1202	1
1178	2546	1
1178	692	1
1178	117	1
1178	661	1
1178	2843	1
1178	3361	0
1178	829	0
1178	1353	0
1178	1742	0
1178	1830	0
1178	3191	0
1178	3465	0
1178	921	0
1178	1049	0
1178	1277	0
1178	1412	0
1178	906	0

1179	771	1
1179	302	1
1179	47	1
1179	1872	1
1179	210	1
1179	3058	1
1179	3059	1
1179	3060	1
1179	3061	1
1179	120	1
1179	282	1
1179	1503	1
1179	1828	0
1179	1324	0
1179	1897	0
1179	908	0
1179	1500	0
1179	82	0
1179	1526	0
1179	1809	0
1179	2781	0
1179	520	0
1179	2606	0
1179	3610	0
1180	769	1
1180	516	1
1180	24	1
1180	2587	1
1180	670	1
1180	159	1

1180	34	1
1180	35	1
1180	165	1
1180	2988	1
1180	48	1
1180	54	1
1180	3008	1
1180	196	1
1180	708	1
1180	2888	1
1180	719	1
1180	223	1
1180	615	1
1180	1389	1
1180	111	1
1180	752	1
1180	3062	1
1180	21	0
1180	1567	0
1180	2703	0
1180	3724	0
1180	431	0
1180	3811	0
1180	1241	0
1180	3809	0
1180	838	0
1180	762	0
1180	1734	0
1180	51	0
1180	3386	0

1180	3635	0
1180	1231	0
1180	3367	0
1180	393 0	
1180	2604	0
1180	723 0	
1180	2383	0
1180	1988	0
1180	3731	0
1180	148 0	
1181	2341	1
1181	491 1	
1181	1776	1
1181	2130	1
1181	2419	1
1181	3070	1
1181	3063	1
1181	3064	1
1181	3065	1
1181	3066	1
1181	3067	1
1181	3068	1
1181	3069	1
1181	1822	1
1181	1194	0
1181	1934	0
1181	2623	0
1181	3615	0
1181	3356	0
1181	1110	0

1181	641	0
1181	1119	0
1181	1427	0
1181	2225	0
1181	248	0
1181	697	0
1181	3116	0
1181	2590	0
1182	184	1
1182	221	1
1182	141	1
1182	237	1
1182	2180	0
1182	2268	0
1182	33	0
1182	1697	0
1183	131	1
1183	134	1
1183	1065	1
1183	171	1
1183	267	1
1183	78	1
1183	47	1
1183	55	1
1183	382	1
1183	2974	0
1183	1371	0
1183	1108	0
1183	1768	0
1183	2964	0

1183	1505	0
1183	1477	0
1183	1058	0
1183	1655	0
1184	1056	1
1184	392	1
1184	46	1
1184	47	1
1184	1589	1
1184	55	1
1184	1178	1
1184	91	1
1184	1709	0
1184	683	0
1184	2774	0
1184	3253	0
1184	448	0
1184	489	0
1184	1409	0
1184	2522	0
1185	3072	1
1185	388	1
1185	133	1
1185	614	1
1185	392	1
1185	105	1
1185	652	1
1185	46	1
1185	399	1
1185	528	1

1185	1103	1
1185	755	1
1185	1140	1
1185	55	1
1185	187	1
1185	765	1
1185	3071	1
1185	276	0
1185	2099	0
1185	2021	0
1185	2006	0
1185	1681	0
1185	1803	0
1185	2875	0
1185	301	0
1185	629	0
1185	632	0
1185	461	0
1185	3067	0
1185	3521	0
1185	2411	0
1185	3295	0
1185	2327	0
1185	3769	0
1186	32	1
1186	928	1
1186	961	1
1186	3073	1
1186	196	1
1186	3074	1

1186	358	1
1186	40	1
1186	42	1
1186	844	1
1186	307	1
1186	2787	0
1186	1126	0
1186	1789	0
1186	964	0
1186	801	0
1186	5	0
1186	2297	0
1186	1628	0
1186	193	0
1186	645	0
1186	1397	0
1187	577	1
1187	2328	1
1187	1507	1
1187	197	1
1187	102	1
1187	906	1
1187	47	1
1187	118	1
1187	920	1
1187	1276	1
1187	3253	0
1187	463	0
1187	1606	0
1187	737	0

1187	320	0
1187	2999	0
1187	3203	0
1187	3114	0
1187	3286	0
1187	3792	0
1188	928	1
1188	1216	1
1188	3075	1
1188	36	1
1188	741	1
1188	3076	1
1188	3077	1
1188	1288	1
1188	393	1
1188	1641	1
1188	395	1
1188	1993	1
1188	1165	1
1188	242	1
1188	1214	1
1188	953	0
1188	419	0
1188	2018	0
1188	2327	0
1188	2337	0
1188	852	0
1188	3109	0
1188	659	0
1188	3264	0

1188	2883	0
1188	2406	0
1188	3118	0
1188	3417	0
1188	349 0	
1188	197 0	
1189	1664	1
1189	771 1	
1189	3078	1
1189	3079	1
1189	3080	1
1189	3081	1
1189	2314	1
1189	2315	1
1189	3082	1
1189	3083	1
1189	2199	1
1189	35 1	
1189	165 1	
1189	2987	1
1189	306 1	
1189	1738	1
1189	222 1	
1189	224 1	
1189	228 1	
1189	3717	0
1189	1227	0
1189	3774	0
1189	2602	0
1189	3647	0

1189	275	0
1189	913	0
1189	1825	0
1189	726	0
1189	2604	0
1189	575	0
1189	3640	0
1189	3165	0
1189	3049	0
1189	783	0
1189	1020	0
1189	1550	0
1189	620	0
1189	1463	0
1190	32	1
1190	257	1
1190	260	1
1190	2181	1
1190	38	1
1190	1353	1
1190	1294	1
1190	498	1
1190	499	1
1190	28	1
1190	2002	0
1190	450	0
1190	2355	0
1190	1206	0
1190	693	0
1190	1293	0

1190	1922	0
1190	3089	0
1190	149	0
1190	1620	0
1191	1798	1
1191	391	1
1191	716	1
1191	3085	1
1191	878	1
1191	2992	1
1191	51	1
1191	764	1
1191	1254	0
1191	1802	0
1191	187	0
1191	537	0
1191	2145	0
1191	414	0
1191	1810	0
1191	734	0
1192	513	1
1192	2786	1
1192	484	1
1192	2406	1
1192	2408	1
1192	1322	1
1192	3086	1
1192	3087	1
1192	1776	1
1192	3088	1

1192	2418	1
1192	3089	1
1192	52	1
1192	3090	1
1192	3091	1
1192	3065	1
1192	411	1
1192	510	1
1192	2415	0
1192	1006	0
1192	2190	0
1192	3133	0
1192	505	0
1192	2451	0
1192	1749	0
1192	2030	0
1192	3393	0
1192	3132	0
1192	337	0
1192	915	0
1192	336	0
1192	2986	0
1192	220	0
1192	895	0
1192	113	0
1192	2052	0
1193	2272	1
1193	929	1
1193	34	1
1193	1315	1

1193	36	1
1193	2275	1
1193	2434	1
1193	2631	1
1193	1513	1
1193	106	1
1193	109	1
1193	1325	1
1193	3092	1
1193	3093	1
1193	3094	1
1193	3095	1
1193	318	1
1193	3044	0
1193	3516	0
1193	2189	0
1193	3519	0
1193	1514	0
1193	2497	0
1193	1566	0
1193	1515	0
1193	185	0
1193	253	0
1193	47	0
1193	1014	0
1193	2244	0
1193	692	0
1193	3613	0
1193	3045	0
1193	2271	0

1194	899	1
1194	73	1
1194	1258	1
1194	366	1
1194	79	1
1194	80	1
1194	117	1
1194	87	1
1194	185	1
1194	43	0
1194	1562	0
1194	1038	0
1194	957	0
1194	3622	0
1194	1697	0
1194	379	0
1194	2703	0
1194	1189	0
1195	163	1
1195	1541	1
1195	295	1
1195	2023	1
1195	107	1
1195	235	1
1195	109	1
1195	46	1
1195	1103	1
1195	2290	1
1195	1396	1
1195	53	1

1195	55	1
1195	696	1
1195	190	1
1195	159	1
1195	611	0
1195	1093	0
1195	2976	0
1195	2803	0
1195	1877	0
1195	2244	0
1195	1430	0
1195	658	0
1195	365	0
1195	1986	0
1195	229	0
1195	2639	0
1195	1937	0
1195	1174	0
1195	2225	0
1195	3364	0
1196	135	1
1196	3146	0
1197	160	1
1197	961	1
1197	3098	1
1197	196	1
1197	198	1
1197	1798	1
1197	297	1
1197	2194	1

1197	1531	1
1197	1528	1
1197	3096	1
1197	314	1
1197	155	1
1197	156	1
1197	509	1
1197	3097	1
1197	742	0
1197	2263	0
1197	599	0
1197	772	0
1197	2237	0
1197	2304	0
1197	2986	0
1197	3362	0
1197	3489	0
1197	622	0
1197	3456	0
1197	1748	0
1197	563	0
1197	324	0
1197	195	0
1197	1676	0
1198	96	1
1198	994	1
1198	35	1
1198	36	1
1198	197	1
1198	1091	1

1198	1730	1
1198	1001	1
1198	526	1
1198	52	1
1198	277	1
1198	445	1
1198	1056	0
1198	550	0
1198	1918	0
1198	1950	0
1198	2865	0
1198	2909	0
1198	2460	0
1198	1008	0
1198	2136	0
1198	2675	0
1198	485	0
1198	710	0
1199	1605	1
1199	105	1
1199	361	1
1199	46	1
1199	878	1
1199	528	1
1199	1235	1
1199	858	1
1199	252	1
1199	190	1
1199	863	1
1199	1220	0

1199	2389	0
1199	3457	0
1199	988	0
1199	2830	0
1199	2740	0
1199	1654	0
1199	3696	0
1199	1374	0
1199	3141	0
1199	1169	0
1200	96	1
1200	354	1
1200	133	1
1200	776	1
1200	105	1
1200	2281	1
1200	46	1
1200	80	1
1200	304	1
1200	52	1
1200	55	1
1200	3099	1
1200	253	1
1200	1569	0
1200	3342	0
1200	3622	0
1200	3173	0
1200	2057	0
1200	2427	0
1200	522	0

1200	3712	0
1200	328	0
1200	3140	0
1200	82	0
1200	736	0
1200	2722	0
1201	1132	1
1201	716	1
1201	941	1
1201	3101	1
1201	2491	1
1201	219	1
1201	3100	1
1201	1437	1
1201	3102	1
1201	3012	0
1201	1916	0
1201	1372	0
1201	2995	0
1201	3044	0
1201	3809	0
1201	3508	0
1201	1910	0
1201	1976	0
1202	608	1
1202	803	1
1202	36	1
1202	677	1
1202	362	1
1202	1290	1

1202	462	1
1202	209	1
1202	1362	1
1202	632	1
1202	1721	1
1202	2009	1
1202	2142	1
1202	3103	1
1202	1846	0
1202	1889	0
1202	335	0
1202	876	0
1202	2107	0
1202	1060	0
1202	3098	0
1202	3323	0
1202	473	0
1202	1480	0
1202	1640	0
1202	812	0
1202	1774	0
1202	3133	0
1203	3104	1
1203	109	1
1203	755	1
1203	245	1
1203	445	1
1203	446	1
1203	1601	0
1203	2126	0

1203	1377	0
1203	160	0
1203	2174	0
1203	944	0
1204	3105	1
1204	3106	1
1204	362	1
1204	941	1
1204	2360	1
1204	287	1
1204	3112	0
1204	2438	0
1204	1681	0
1204	3068	0
1204	1173	0
1204	3820	0
1205	96	1
1205	130	1
1205	3107	1
1205	714	1
1205	76	1
1205	301	1
1205	118	1
1205	376	1
1205	441	1
1205	2269	1
1205	548	0
1205	1309	0
1205	360	0
1205	1947	0

1205	2014	0
1205	1382	0
1205	2508	0
1205	969	0
1205	70	0
1205	3478	0
1206	3108	1
1206	3109	1
1206	3110	1
1206	3111	1
1206	3112	1
1206	3113	1
1206	3114	1
1206	2766	1
1206	785	1
1206	147	1
1206	820	1
1206	1883	1
1206	2781	1
1206	206	0
1206	2347	0
1206	2340	0
1206	1612	0
1206	1238	0
1206	3504	0
1206	1867	0
1206	1071	0
1206	3050	0
1206	2494	0
1206	2604	0

1206	420	0
1206	216	0
1207	96	1
1207	98	1
1207	951	1
1207	184	1
1207	377	1
1207	2285	0
1207	3308	0
1207	2059	0
1207	3144	0
1207	2082	0
1208	96	1
1208	1536	1
1208	133	1
1208	210	1
1208	52	1
1208	118	1
1208	119	1
1208	438	1
1208	57	1
1208	1756	1
1208	2078	1
1208	3214	0
1208	2215	0
1208	1999	0
1208	2626	0
1208	3458	0
1208	368	0
1208	801	0

1208	1628	0
1208	3392	0
1208	658	0
1208	1511	0
1209	36	1
1209	356	1
1209	3115	1
1209	241	1
1209	243	1
1209	52	1
1209	347	1
1209	3416	0
1209	2508	0
1209	1237	0
1209	2901	0
1209	3138	0
1209	1184	0
1209	846	0
1210	1889	1
1210	130	1
1210	134	1
1210	1258	1
1210	3116	1
1210	79	1
1210	1525	1
1210	118	1
1210	376	1
1210	538	1
1210	923	1
1210	700	1

1210	1464	0
1210	349	0
1210	649	0
1210	926	0
1210	899	0
1210	2770	0
1210	1455	0
1210	1860	0
1210	3308	0
1210	1051	0
1210	1295	0
1210	3346	0
1211	1027	1
1211	325	1
1211	2120	1
1211	3117	1
1211	16	1
1211	401	1
1211	1854	1
1211	1075	1
1211	53	1
1211	55	1
1211	190	1
1211	3382	0
1211	3084	0
1211	880	0
1211	310	0
1211	2254	0
1211	2357	0
1211	2187	0

1211	1637	0
1211	2393	0
1211	2902	0
1211	978	0
1212	96	1
1212	1283	1
1212	102	1
1212	806	1
1212	776	1
1212	589	1
1212	494	1
1212	303	1
1212	210	1
1212	118	1
1212	216	1
1212	217	1
1212	2418	0
1212	1569	0
1212	2341	0
1212	1141	0
1212	2514	0
1212	3032	0
1212	1804	0
1212	3027	0
1212	2251	0
1212	32	0
1212	1850	0
1212	2466	0
1213	708	1
1213	1095	1

1213	105	1
1213	107	1
1213	141	1
1213	46	1
1213	1103	1
1213	2064	1
1213	2703	1
1213	882	1
1213	53	1
1213	54	1
1213	55	1
1213	572	1
1213	670	1
1213	3757	0
1213	857	0
1213	151	0
1213	3754	0
1213	12	0
1213	2248	0
1213	2691	0
1213	1362	0
1213	1645	0
1213	3808	0
1213	2841	0
1213	1342	0
1213	2964	0
1213	2074	0
1213	3639	0
1214	1088	1
1214	1091	1

1214	1543	1
1214	1580	1
1214	45	1
1214	878	1
1214	48	1
1214	852	1
1214	54	1
1214	154	1
1214	670	1
1214	159	1
1214	2360	0
1214	684	0
1214	199	0
1214	452	0
1214	1597	0
1214	1522	0
1214	2416	0
1214	364	0
1214	3036	0
1214	2296	0
1214	704	0
1214	1671	0
1215	98	1
1215	1060	1
1215	135	1
1215	73	1
1215	1258	1
1215	75	1
1215	79	1
1215	919	1

1215	372	1
1215	87	1
1215	2083	0
1215	1498	0
1215	1462	0
1215	1657	0
1215	416	0
1215	431	0
1215	2178	0
1215	2199	0
1215	1731	0
1215	1139	0
1216	3110	1
1216	813	1
1216	2414	1
1216	3118	1
1216	144	1
1216	2096	1
1216	1042	1
1216	147	1
1216	3119	1
1216	3120	1
1216	150	1
1216	151	1
1216	1592	1
1216	3121	1
1216	3122	1
1216	3123	1
1216	1854	0
1216	410	0

1216	3792	0
1216	2529	0
1216	2155	0
1216	46	0
1216	832	0
1216	2777	0
1216	371	0
1216	505	0
1216	1244	0
1216	3312	0
1216	141	0
1216	1258	0
1216	2619	0
1216	883	0
1217	289	1
1217	34	1
1217	35	1
1217	100	1
1217	102	1
1217	393	1
1217	42	1
1217	395	1
1217	655	1
1217	1425	1
1217	18	1
1217	3124	1
1217	3125	1
1217	2376	0
1217	1201	0
1217	941	0

1217	2416	0
1217	3820	0
1217	2984	0
1217	2053	0
1217	31	0
1217	2705	0
1217	1174	0
1217	2258	0
1217	3444	0
1217	1246	0
1218	1416	1
1218	1545	1
1218	1556	1
1218	282	1
1218	670	1
1218	158	1
1218	159	1
1218	163	1
1218	165	1
1218	46	1
1218	48	1
1218	52	1
1218	53	1
1218	54	1
1218	695	1
1218	196	1
1218	2628	1
1218	715	1
1218	2653	1
1218	483	1

1218	105	1
1218	878	1
1218	111	1
1218	1395	1
1218	1867	0
1218	1505	0
1218	1631	0
1218	3474	0
1218	2330	0
1218	578	0
1218	3353	0
1218	3668	0
1218	3059	0
1218	1482	0
1218	286	0
1218	2790	0
1218	3546	0
1218	2204	0
1218	1289	0
1218	197	0
1218	3468	0
1218	3340	0
1218	2469	0
1218	1117	0
1218	2933	0
1218	2578	0
1218	3483	0
1218	3406	0
1219	96	1
1219	3130	1

1219	3128	1
1219	936	1
1219	1354	1
1219	427	1
1219	940	1
1219	941	1
1219	210	1
1219	245	1
1219	3126	1
1219	1015	1
1219	3127	1
1219	3129	1
1219	2554	1
1219	3131	1
1219	3132	1
1219	1437	1
1219	537	0
1219	3817	0
1219	2478	0
1219	2455	0
1219	3617	0
1219	2017	0
1219	2644	0
1219	1140	0
1219	1299	0
1219	2461	0
1219	3669	0
1219	6	0
1219	2975	0
1219	1108	0

1219	3366	0
1219	1930	0
1219	2404	0
1219	1430	0
1220	102	1
1220	137	1
1220	267	1
1220	301	1
1220	78	1
1220	47	1
1220	302	1
1220	303	1
1220	245	1
1220	118	1
1220	1589	1
1220	187	1
1220	1180	1
1220	253	1
1220	126	1
1220	3290	0
1220	236	0
1220	3110	0
1220	326	0
1220	1122	0
1220	3370	0
1220	1212	0
1220	1673	0
1220	2504	0
1220	3675	0
1220	1305	0

1220	81	0
1220	3103	0
1220	452	0
1220	2839	0
1221	774	1
1221	1995	1
1221	300	1
1221	141	1
1221	782	1
1221	1750	1
1221	3077	0
1221	632	0
1221	364	0
1221	1397	0
1221	2223	0
1221	3142	0
1222	1409	1
1222	135	1
1222	377	1
1222	74	1
1222	75	1
1222	79	1
1222	80	1
1222	117	1
1222	185	1
1222	127	1
1222	282	0
1222	1807	0
1222	1244	0
1222	3105	0

1222	502	0
1222	3260	0
1222	2096	0
1222	2844	0
1222	3771	0
1222	3073	0
1223	96	1
1223	1091	1
1223	357	1
1223	389	1
1223	520	1
1223	361	1
1223	78	1
1223	80	1
1223	371	1
1223	118	1
1223	299	0
1223	1467	0
1223	3273	0
1223	2728	0
1223	1273	0
1223	1308	0
1223	2653	0
1223	884	0
1223	2427	0
1223	2362	0
1224	128	1
1224	3136	1
1224	3137	1
1224	3138	1

1224	676	1
1224	3139	1
1224	3140	1
1224	617	1
1224	235	1
1224	2638	1
1224	3133	1
1224	1982	1
1224	151	1
1224	3134	1
1224	541	1
1224	1630	1
1224	3135	1
1224	2624	0
1224	522	0
1224	2276	0
1224	1633	0
1224	1270	0
1224	2735	0
1224	2120	0
1224	878	0
1224	35	0
1224	1426	0
1224	1841	0
1224	3697	0
1224	1970	0
1224	1961	0
1224	1753	0
1224	749	0
1224	839	0

1225	96	1
1225	673	1
1225	36	1
1225	3141	1
1225	47	1
1225	118	1
1225	87	1
1225	438	1
1225	286	1
1225	1897	0
1225	3319	0
1225	3840	0
1225	1023	0
1225	2582	0
1225	611	0
1225	441	0
1225	3070	0
1225	630	0
1226	96	1
1226	133	1
1226	3142	1
1226	3048	1
1226	78	1
1226	79	1
1226	118	1
1226	184	1
1226	185	1
1226	538	1
1226	1244	1
1226	832	0

1226	2979	0
1226	64	0
1226	1013	0
1226	308	0
1226	3585	0
1226	2491	0
1226	3755	0
1226	2892	0
1226	1603	0
1226	820	0
1227	197	1
1227	1030	1
1227	199	1
1227	3143	1
1227	3144	1
1227	752	1
1227	1010	1
1227	1015	1
1227	318	1
1227	2514	0
1227	662	0
1227	2642	0
1227	1489	0
1227	413	0
1227	1316	0
1227	2164	0
1227	3316	0
1227	37	0
1228	446	1
1228	105	1

1228	107	1
1228	46	1
1228	118	1
1228	475	1
1228	572	1
1228	190	1
1228	966	0
1228	82	0
1228	1627	0
1228	2954	0
1228	922	0
1228	35	0
1228	1126	0
1228	2152	0
1229	1440	1
1229	1122	1
1229	2595	1
1229	841	1
1229	3145	1
1229	3146	1
1229	3147	1
1229	2162	1
1229	243	1
1229	244	1
1229	1461	1
1229	2074	1
1229	190	1
1229	1053	0
1229	1935	0
1229	2616	0

1229	2977	0
1229	1561	0
1229	1187	0
1229	2540	0
1229	3832	0
1229	3237	0
1229	3588	0
1229	179	0
1229	2282	0
1229	3529	0
1230	802	1
1230	1820	1
1230	102	1
1230	46	1
1230	55	1
1230	119	1
1230	286	1
1230	126	1
1230	127	1
1230	866	0
1230	323	0
1230	1135	0
1230	256	0
1230	2658	0
1230	3402	0
1230	1121	0
1230	1980	0
1230	827	0
1231	1984	1
1231	705	1

1231	34	1
1231	35	1
1231	36	1
1231	1446	1
1231	2476	1
1231	208	1
1231	703	1
1231	2375	0
1231	3437	0
1231	1054	0
1231	1801	0
1231	3257	0
1231	3129	0
1231	1774	0
1231	895	0
1231	3685	0
1232	133	1
1232	102	1
1232	1130	1
1232	1324	1
1232	46	1
1232	47	1
1232	302	1
1232	431	1
1232	1391	1
1232	52	1
1232	118	1
1232	1144	1
1232	1593	1
1232	126	1

1232	362	0
1232	3132	0
1232	489	0
1232	645	0
1232	658	0
1232	1329	0
1232	1308	0
1232	3812	0
1232	1214	0
1232	1919	0
1232	2196	0
1232	1023	0
1232	3046	0
1232	978	0
1233	102	1
1233	776	1
1233	1673	1
1233	427	1
1233	3148	1
1233	1869	1
1233	47	1
1233	210	1
1233	1015	1
1233	280	1
1233	1594	1
1233	2559	1
1233	1927	0
1233	2378	0
1233	1037	0
1233	2444	0

1233	532	0
1233	3675	0
1233	641	0
1233	2136	0
1233	1095	0
1233	754	0
1233	2268	0
1233	166	0
1234	1248	1
1234	1569	1
1234	196	1
1234	36	1
1234	519	1
1234	616	1
1234	681	1
1234	945	1
1234	882	1
1234	1586	1
1234	895	1
1234	56	1
1234	154	1
1234	157	1
1234	735	1
1234	393	0
1234	2214	0
1234	2921	0
1234	973	0
1234	1174	0
1234	3222	0
1234	1406	0

1234	1249	0
1234	3349	0
1234	2087	0
1234	3253	0
1234	744	0
1234	50	0
1234	3350	0
1234	1412	0
1235	96	1
1235	832	1
1235	132	1
1235	901	1
1235	1188	1
1235	137	1
1235	377	1
1235	75	1
1235	3149	1
1235	3150	1
1235	79	1
1235	115	1
1235	951	1
1235	184	1
1235	185	1
1235	254	1
1235	368	0
1235	2250	0
1235	1180	0
1235	2571	0
1235	1645	0
1235	1137	0

1235	3690	0
1235	1698	0
1235	2335	0
1235	1660	0
1235	1119	0
1235	677	0
1235	2994	0
1235	3205	0
1235	1495	0
1235	3544	0
1236	102	1
1236	41	1
1236	105	1
1236	46	1
1236	302	1
1236	303	1
1236	754	1
1236	52	1
1236	118	1
1236	55	1
1236	822	1
1236	575	1
1236	322	0
1236	1243	0
1236	1666	0
1236	2330	0
1236	1037	0
1236	1918	0
1236	2584	0
1236	2083	0

1236	2479	0
1236	1837	0
1236	1116	0
1236	3165	0
1237	196	1
1237	165	1
1237	2695	1
1237	104	1
1237	233	1
1237	91	1
1237	462	1
1237	3151	1
1237	433	1
1237	1496	1
1237	154	1
1237	187	1
1237	860	1
1237	765	1
1237	1406	1
1237	895	1
1237	3704	0
1237	319	0
1237	2271	0
1237	1818	0
1237	456	0
1237	2876	0
1237	90	0
1237	542	0
1237	2362	0
1237	1085	0

1237	3586	0
1237	18	0
1237	1829	0
1237	2085	0
1237	3570	0
1237	3206	0
1238	64	1
1238	512	1
1238	418	1
1238	419	1
1238	515	1
1238	2464	1
1238	70	1
1238	422	1
1238	136	1
1238	912	1
1238	3152	1
1238	3153	1
1238	1559	1
1238	2616	1
1238	601	1
1238	795	1
1238	508	1
1238	510	1
1238	2081	0
1238	1597	0
1238	3560	0
1238	919	0
1238	765	0
1238	2940	0

1238	727	0
1238	3081	0
1238	139	0
1238	3390	0
1238	3626	0
1238	1439	0
1238	3315	0
1238	3840	0
1238	295	0
1238	1388	0
1238	1603	0
1238	788	0
1239	36	1
1239	104	1
1239	46	1
1239	2065	1
1239	3154	1
1239	3155	1
1239	22	1
1239	55	1
1239	57	1
1239	187	1
1239	252	1
1239	1406	1
1239	3037	0
1239	1729	0
1239	2316	0
1239	1945	0
1239	2210	0
1239	2566	0

1239	2390	0
1239	113	0
1239	527	0
1239	797	0
1239	3073	0
1239	3399	0
1240	477	1
1240	385	1
1240	674	1
1240	39	1
1240	2953	1
1240	427	1
1240	1395	1
1240	52	1
1240	2815	1
1240	863	1
1240	3156	1
1240	24	1
1240	1145	1
1240	1178	1
1240	221	1
1240	127	1
1240	2403	0
1240	3199	0
1240	2020	0
1240	118	0
1240	959	0
1240	3430	0
1240	746	0
1240	298	0

1240	3585	0
1240	2508	0
1240	996	0
1240	3202	0
1240	3484	0
1240	1459	0
1240	3093	0
1240	1254	0
1241	832	1
1241	2817	1
1241	133	1
1241	137	1
1241	1419	1
1241	76	1
1241	1899	1
1241	206	1
1241	916	1
1241	3157	1
1241	184	1
1241	185	1
1241	923	1
1241	202	0
1241	197	0
1241	1362	0
1241	1248	0
1241	1991	0
1241	3233	0
1241	643	0
1241	68	0
1241	391	0

1241	2880	0
1241	2257	0
1241	1100	0
1241	2127	0
1242	645	1
1242	39	1
1242	622	1
1242	1494	1
1242	567	1
1242	1112	1
1242	3158	1
1242	3159	1
1242	772	0
1242	3272	0
1242	2029	0
1242	2570	0
1242	607	0
1242	1699	0
1242	334	0
1242	485	0
1243	353	1
1243	130	1
1243	35	1
1243	708	1
1243	293	1
1243	1406	1
1243	1319	1
1243	104	1
1243	137	1
1243	712	1

1243	118	1
1243	254	1
1243	183	0
1243	3741	0
1243	2065	0
1243	3296	0
1243	3032	0
1243	2736	0
1243	1953	0
1243	2442	0
1243	1649	0
1243	1785	0
1243	3008	0
1243	1993	0
1244	130	1
1244	700	1
1244	376	1
1244	1080	1
1244	135	1
1244	1324	1
1244	78	1
1244	46	1
1244	47	1
1244	560	1
1244	561	1
1244	374	1
1244	184	1
1244	185	1
1244	538	1
1244	572	1

1244	863	1
1244	1908	0
1244	111	0
1244	39	0
1244	1950	0
1244	696	0
1244	40	0
1244	168	0
1244	793	0
1244	3550	0
1244	3584	0
1244	2254	0
1244	2920	0
1244	424	0
1244	2863	0
1244	132	0
1244	482	0
1244	1851	0
1245	1345	1
1245	2081	1
1245	1347	1
1245	105	1
1245	107	1
1245	109	1
1245	46	1
1245	111	1
1245	56	1
1245	3160	1
1245	159	1
1245	1319	0

1245	2709	0
1245	868	0
1245	684	0
1245	164	0
1245	1467	0
1245	3127	0
1245	1065	0
1245	3511	0
1245	1787	0
1245	633	0
1246	36	1
1246	391	1
1246	1255	1
1246	105	1
1246	109	1
1246	46	1
1246	47	1
1246	110	1
1246	112	1
1246	1020	1
1246	382	1
1246	863	1
1246	2343	0
1246	1663	0
1246	1475	0
1246	2342	0
1246	2619	0
1246	365	0
1246	2740	0
1246	1693	0

1246	591	0
1246	485	0
1246	2960	0
1246	723	0
1247	320	1
1247	1698	1
1247	835	1
1247	1827	1
1247	331	1
1247	1739	1
1247	14	1
1247	16	1
1247	17	1
1247	307	1
1247	2613	1
1247	1270	1
1247	1271	1
1247	860	1
1247	1463	0
1247	3454	0
1247	3374	0
1247	459	0
1247	208	0
1247	998	0
1247	1916	0
1247	2570	0
1247	373	0
1247	737	0
1247	3686	0
1247	3404	0

1247	434	0
1247	2345	0
1248	3047	1
1248	74	1
1248	46	1
1248	55	1
1248	87	1
1248	378	1
1248	187	1
1248	2438	0
1248	2606	0
1248	1399	0
1248	747	0
1248	219	0
1248	3529	0
1248	1116	0
1249	795	1
1249	163	1
1249	422	1
1249	615	1
1249	617	1
1249	1325	1
1249	111	1
1249	187	1
1249	508	1
1249	526	0
1249	546	0
1249	3548	0
1249	2595	0
1249	702	0

1249	1164	0
1249	1490	0
1249	770	0
1249	2035	0
1250	36	1
1250	394	1
1250	109	1
1250	1810	1
1250	1811	1
1250	2199	1
1250	346	1
1250	1022	1
1250	821	0
1250	2314	0
1250	2794	0
1250	1528	0
1250	1668	0
1250	1367	0
1250	3662	0
1250	2973	0
1251	1163	1
1251	1166	1
1251	948	1
1251	2357	1
1251	3162	1
1251	416	0
1251	2678	0
1251	2037	0
1251	1466	0
1251	2882	0

1252	96	1
1252	1122	1
1252	1316	1
1252	133	1
1252	3163	1
1252	3164	1
1252	3165	1
1252	2291	0
1252	3449	0
1252	175	0
1252	514	0
1252	2751	0
1252	3482	0
1252	2582	0
1253	130	1
1253	134	1
1253	135	1
1253	1258	1
1253	78	1
1253	79	1
1253	206	1
1253	373	1
1253	87	1
1253	184	1
1253	185	1
1253	538	1
1253	698	1
1253	583	0
1253	3796	0
1253	2215	0

1253	1074	0
1253	3535	0
1253	3101	0
1253	2946	0
1253	190	0
1253	3803	0
1253	3309	0
1253	3474	0
1253	3727	0
1253	2243	0
1254	96	1
1254	1311	1
1254	807	1
1254	366	1
1254	80	1
1254	276	1
1254	117	1
1254	87	1
1254	127	1
1254	476	0
1254	135	0
1254	2986	0
1254	1669	0
1254	2252	0
1254	2621	0
1254	480	0
1254	2259	0
1254	955	0
1255	224	1
1255	36	1

1255	109	1
1255	685	1
1255	52	1
1255	2197	1
1255	3166	1
1255	3167	1
1255	218	0
1255	1790	0
1255	2243	0
1255	3736	0
1255	3350	0
1255	2804	0
1255	2076	0
1255	1443	0
1256	96	1
1256	133	1
1256	102	1
1256	199	1
1256	391	1
1256	267	1
1256	46	1
1256	47	1
1256	305	1
1256	2161	1
1256	190	1
1256	1352	0
1256	3566	0
1256	2679	0
1256	1496	0
1256	2145	0

1256	2927	0
1256	3411	0
1256	2030	0
1256	1161	0
1256	3731	0
1256	1174	0
1257	224	1
1257	165	1
1257	615	1
1257	141	1
1257	878	1
1257	111	1
1257	48	1
1257	719	1
1257	51	1
1257	54	1
1257	154	1
1257	670	1
1257	159	1
1257	522	0
1257	3644	0
1257	966	0
1257	3455	0
1257	2562	0
1257	1739	0
1257	77	0
1257	2268	0
1257	774	0
1257	462	0
1257	1156	0

1257	2563	0
1257	1387	0
1258	163	1
1258	297	1
1258	670	1
1258	1004	1
1258	1325	1
1258	46	1
1258	111	1
1258	48	1
1258	719	1
1258	878	1
1258	51	1
1258	53	1
1258	154	1
1258	507	1
1258	190	1
1258	159	1
1258	3511	0
1258	3587	0
1258	1469	0
1258	3573	0
1258	1427	0
1258	2737	0
1258	675	0
1258	1183	0
1258	3470	0
1258	1871	0
1258	692	0
1258	2031	0

1258	1839	0
1258	2641	0
1258	291	0
1258	716	0
1259	3168	1
1259	3169	1
1259	3170	1
1259	3171	1
1259	708	1
1259	229	1
1259	3172	1
1259	3173	1
1259	715	1
1259	1003	1
1259	1004	1
1259	55	1
1259	57	1
1259	187	1
1259	223	1
1259	1069	0
1259	2872	0
1259	3641	0
1259	2966	0
1259	2391	0
1259	1425	0
1259	466	0
1259	2308	0
1259	1166	0
1259	1199	0
1259	3021	0

1259	986	0
1259	115	0
1259	3167	0
1259	583	0
1260	354	1
1260	387	1
1260	37	1
1260	293	1
1260	41	1
1260	105	1
1260	141	1
1260	685	1
1260	1870	1
1260	2290	1
1260	55	1
1260	697	1
1260	221	1
1260	3547	0
1260	1163	0
1260	976	0
1260	361	0
1260	2312	0
1260	2888	0
1260	1856	0
1260	3469	0
1260	1808	0
1260	2814	0
1260	1527	0
1260	2462	0
1260	484	0

1261	1730	1
1261	3174	1
1261	3175	1
1261	904	1
1261	3176	1
1261	3177	1
1261	3178	1
1261	2477	1
1261	1136	1
1261	1624	1
1261	735	1
1261	3373	0
1261	3678	0
1261	1669	0
1261	484	0
1261	658	0
1261	1119	0
1261	1101	0
1261	962	0
1261	2036	0
1261	240	0
1261	3460	0
1262	96	1
1262	673	1
1262	185	1
1262	377	1
1262	1545	1
1262	3179	1
1262	206	1
1262	239	1

1262	2910	1
1262	1781	1
1262	184	1
1262	57	1
1262	413	1
1262	1278	1
1262	1816	0
1262	503	0
1262	3396	0
1262	3210	0
1262	177	0
1262	1752	0
1262	2707	0
1262	1399	0
1262	138	0
1262	817	0
1262	2026	0
1262	937	0
1262	2454	0
1262	121	0
1263	1032	1
1263	1435	1
1263	36	1
1263	41	1
1263	556	1
1263	949	1
1263	1973	1
1263	196	1
1263	1220	1
1263	459	1

1263	478	1
1263	2786	1
1263	3180	1
1263	2541	1
1263	3181	1
1263	3182	1
1263	3183	1
1263	3184	1
1263	3185	1
1263	68	0
1263	3369	0
1263	3580	0
1263	1073	0
1263	1455	0
1263	31	0
1263	2601	0
1263	2200	0
1263	886	0
1263	3172	0
1263	3506	0
1263	2116	0
1263	3804	0
1263	1239	0
1263	1341	0
1263	3025	0
1263	3669	0
1263	2822	0
1263	1140	0
1264	96	1
1264	130	1

1264	135	1
1264	47	1
1264	80	1
1264	81	1
1264	1200	1
1264	1871	1
1264	374	1
1264	376	1
1264	185	1
1264	127	1
1264	2254	0
1264	3143	0
1264	71	0
1264	3728	0
1264	631	0
1264	690	0
1264	3406	0
1264	3693	0
1264	3815	0
1264	3297	0
1264	83	0
1264	348	0
1265	96	1
1265	770	1
1265	1756	1
1265	1092	1
1265	776	1
1265	109	1
1265	210	1
1265	52	1

1265	118	1
1265	55	1
1265	1020	1
1265	989	1
1265	1023	0
1265	1176	0
1265	51	0
1265	2014	0
1265	2667	0
1265	144	0
1265	3397	0
1265	3797	0
1265	3420	0
1265	2383	0
1265	3838	0
1265	1661	0
1266	37	1
1266	391	1
1266	105	1
1266	523	1
1266	141	1
1266	46	1
1266	1103	1
1266	1396	1
1266	2103	1
1266	55	1
1266	56	1
1266	187	1
1266	159	1
1266	1723	0

1266	436	0
1266	2717	0
1266	3472	0
1266	1257	0
1266	1176	0
1266	2773	0
1266	3274	0
1266	557	0
1266	515	0
1266	2001	0
1266	3684	0
1266	1745	0
1267	96	1
1267	102	1
1267	199	1
1267	426	1
1267	302	1
1267	79	1
1267	303	1
1267	3188	1
1267	3189	1
1267	118	1
1267	376	1
1267	1501	1
1267	3104	0
1267	1838	0
1267	1895	0
1267	3764	0
1267	738	0
1267	516	0

1267	1758	0
1267	1500	0
1267	3342	0
1267	1143	0
1267	2328	0
1267	3421	0
1268	96	1
1268	133	1
1268	43	1
1268	715	1
1268	2163	1
1268	3191	1
1268	694	1
1268	119	1
1268	3190	1
1268	1018	1
1268	91	1
1268	126	1
1268	1407	1
1268	3601	0
1268	78	0
1268	497	0
1268	2212	0
1268	3176	0
1268	1191	0
1268	1026	0
1268	2775	0
1268	1783	0
1268	422	0
1268	3493	0

1268	3776	0
1268	3091	0
1269	356	1
1269	1253	1
1269	1797	1
1269	1260	1
1269	46	1
1269	111	1
1269	3151	1
1269	55	1
1269	223	1
1269	2812	0
1269	2331	0
1269	3674	0
1269	28	0
1269	1162	0
1269	1347	0
1269	2878	0
1269	2624	0
1269	130	0
1270	352	1
1270	1664	1
1270	44	1
1270	109	1
1270	2604	1
1270	1168	1
1270	3192	1
1270	3193	1
1270	3194	1
1270	3195	1

1270	1182	1
1270	159	1
1270	1689	0
1270	3550	0
1270	3103	0
1270	2601	0
1270	573	0
1270	1474	0
1270	978	0
1270	1149	0
1270	3311	0
1270	2416	0
1270	647	0
1270	2646	0
1271	354	1
1271	708	1
1271	1092	1
1271	102	1
1271	199	1
1271	712	1
1271	1516	1
1271	109	1
1271	301	1
1271	52	1
1271	920	1
1271	54	1
1271	55	1
1271	118	1
1271	575	1
1271	84	0

1271	850	0
1271	2072	0
1271	671	0
1271	1449	0
1271	3187	0
1271	1064	0
1271	957	0
1271	76	0
1271	3559	0
1271	3739	0
1271	3493	0
1271	3571	0
1271	643	0
1271	941	0
1272	98	1
1272	1413	1
1272	74	1
1272	267	1
1272	79	1
1272	368	1
1272	721	1
1272	184	1
1272	185	1
1272	665	0
1272	584	0
1272	2674	0
1272	1824	0
1272	1625	0
1272	883	0
1272	819	0

1272	1324	0
1272	893	0
1273	36	1
1273	293	1
1273	776	1
1273	105	1
1273	302	1
1273	467	1
1273	212	1
1273	52	1
1273	821	1
1273	1243	0
1273	872	0
1273	495	0
1273	1046	0
1273	1085	0
1273	3026	0
1273	820	0
1273	1050	0
1273	844	0
1274	131	1
1274	923	1
1274	104	1
1274	137	1
1274	2058	1
1274	1393	1
1274	2065	1
1274	117	1
1274	1141	1
1274	3197	1

1274	187	1
1274	3196	1
1274	413	1
1274	1950	1
1274	2719	1
1274	554	0
1274	3306	0
1274	2303	0
1274	1112	0
1274	821	0
1274	2808	0
1274	3240	0
1274	273	0
1274	1084	0
1274	1003	0
1274	1143	0
1274	2167	0
1274	667	0
1274	1766	0
1274	506	0
1275	3200	1
1275	3201	1
1275	3202	1
1275	1863	1
1275	232	1
1275	1773	1
1275	3118	1
1275	2481	1
1275	3123	1
1275	2038	1

1275	2460	1
1275	988 1	
1275	3198	1
1275	3199	1
1275	3492	0
1275	108 0	
1275	3354	0
1275	3436	0
1275	2914	0
1275	3280	0
1275	3750	0
1275	142 0	
1275	2169	0
1275	191 0	
1275	1111	0
1275	779 0	
1275	1121	0
1275	2782	0
1276	320 1	
1276	257 1	
1276	1795	1
1276	164 1	
1276	3203	1
1276	332 1	
1276	13 1	
1276	1036	1
1276	498 1	
1276	275 1	
1276	499 1	
1276	887 1	

1276	155	1
1276	3006	1
1276	446	0
1276	1206	0
1276	2194	0
1276	1418	0
1276	497	0
1276	3099	0
1276	1038	0
1276	2241	0
1276	3261	0
1276	3110	0
1276	2817	0
1276	2008	0
1276	2295	0
1276	2458	0
1277	325	1
1277	134	1
1277	393	1
1277	46	1
1277	243	1
1277	52	1
1277	726	1
1277	1115	1
1277	60	0
1277	672	0
1277	705	0
1277	2196	0
1277	1214	0
1277	2460	0

1277	3718	0
1277	3643	0
1278	96	1
1278	135	1
1278	75	1
1278	238	1
1278	79	1
1278	87	1
1278	184	1
1278	2922	0
1278	2564	0
1278	899	0
1278	327	0
1278	2629	0
1278	3346	0
1278	2154	0
1279	900	1
1279	1988	1
1279	102	1
1279	392	1
1279	1868	1
1279	1389	1
1279	1395	1
1279	1556	1
1279	55	1
1279	282	1
1279	219	1
1279	190	1
1279	1242	0
1279	1942	0

1279	2100	0
1279	3284	0
1279	316	0
1279	2015	0
1279	880	0
1279	997	0
1279	3060	0
1279	949	0
1279	1658	0
1279	2922	0
1280	385	1
1280	66	1
1280	2337	1
1280	2915	1
1280	3204	1
1280	3205	1
1280	203	1
1280	893	1
1280	558	1
1280	540	1
1280	61	1
1280	2921	0
1280	1133	0
1280	408	0
1280	1970	0
1280	3031	0
1280	2245	0
1280	1681	0
1280	2199	0
1280	1184	0

1280	2587	0
1280	3011	0
1281	1949	1
1281	21	1
1281	1310	1
1281	1761	0
1281	3506	0
1281	2252	0
1282	1856	1
1282	66	1
1282	356	1
1282	3206	1
1282	1064	1
1282	2921	1
1282	1549	1
1282	46	1
1282	1073	1
1282	726	1
1282	1264	0
1282	3530	0
1282	2057	0
1282	1033	0
1282	2272	0
1282	3146	0
1282	2262	0
1282	2336	0
1282	312	0
1282	3785	0
1283	2913	1
1283	98	1

1283	261	1
1283	3207	1
1283	3208	1
1283	74	1
1283	364	1
1283	254	1
1283	3162	0
1283	3723	0
1283	1679	0
1283	1171	0
1283	1057	0
1283	63	0
1283	1802	0
1283	2861	0
1284	66	1
1284	74	1
1284	1228	1
1284	1229	1
1284	145	1
1284	127	1
1284	630	0
1284	3316	0
1284	1284	0
1284	3785	0
1284	400	0
1284	1221	0
1285	96	1
1285	1186	1
1285	134	1
1285	3209	1

1285	364	1
1285	80	1
1285	1200	1
1285	2548	1
1285	374	1
1285	87	1
1285	376	1
1285	185	1
1285	3358	0
1285	1722	0
1285	2570	0
1285	3015	0
1285	3154	0
1285	2971	0
1285	1937	0
1285	2551	0
1285	3365	0
1285	643	0
1285	2300	0
1285	2848	0
1286	105	1
1286	45	1
1286	110	1
1286	113	1
1286	1397	1
1286	55	1
1286	56	1
1286	1401	1
1286	187	1
1286	188	1

1286	103	0
1286	475	0
1286	1848	0
1286	3348	0
1286	2818	0
1286	1125	0
1286	2	0
1286	2874	0
1286	3606	0
1286	3009	0
1287	608	1
1287	1342	1
1287	34	1
1287	2433	1
1287	232	1
1287	1662	1
1287	109	1
1287	1752	1
1287	286	1
1287	2100	0
1287	1648	0
1287	1574	0
1287	1070	0
1287	1937	0
1287	2179	0
1287	3464	0
1287	1724	0
1287	2803	0
1288	66	1
1288	2441	1

1288	1821	1
1288	2602	1
1288	3210	1
1288	3211	1
1288	3212	1
1288	917	1
1288	1659	1
1288	699	1
1288	1491	0
1288	1154	0
1288	3332	0
1288	599	0
1288	3365	0
1288	2752	0
1288	1280	0
1288	491	0
1288	3773	0
1288	1198	0
1289	608	1
1289	391	1
1289	105	1
1289	107	1
1289	110	1
1289	46	1
1289	1139	1
1289	2489	1
1289	571	1
1289	765	1
1289	2856	0
1289	512	0

1289	1198	0
1289	2742	0
1289	1915	0
1289	360	0
1289	517	0
1289	1941	0
1289	3373	0
1289	2440	0
1290	199	1
1290	776	1
1290	1098	1
1290	525	1
1290	208	1
1290	209	1
1290	210	1
1290	212	1
1290	118	1
1290	215	1
1290	822	1
1290	1502	1
1290	840	0
1290	1495	0
1290	3206	0
1290	1594	0
1290	2589	0
1290	1380	0
1290	3465	0
1290	3103	0
1290	1958	0
1290	41	0

1290	99	0
1290	3128	0
1291	608	1
1291	36	1
1291	775	1
1291	2058	1
1291	243	1
1291	188	1
1291	600	0
1291	3296	0
1291	1793	0
1291	3750	0
1291	2046	0
1291	1193	0
1292	102	1
1292	776	1
1292	560	1
1292	305	1
1292	920	1
1292	2074	1
1292	871	0
1292	1314	0
1292	3291	0
1292	953	0
1292	90	0
1292	2924	0
1293	99	1
1293	1190	1
1293	759	1
1293	79	1

1293	47	1
1293	305	1
1293	399	1
1293	1199	1
1293	87	1
1293	93	1
1293	862	1
1293	1196	0
1293	1197	0
1293	1490	0
1293	2906	0
1293	950	0
1293	3410	0
1293	1673	0
1293	2473	0
1293	1067	0
1293	360	0
1293	1115	0
1294	130	1
1294	133	1
1294	1324	1
1294	3213	1
1294	78	1
1294	206	1
1294	239	1
1294	52	1
1294	118	1
1294	184	1
1294	185	1
1294	1668	0

1294	780	0
1294	193	0
1294	2643	0
1294	2263	0
1294	1747	0
1294	822	0
1294	3603	0
1294	2838	0
1294	3577	0
1294	187	0
1295	130	1
1295	1186	1
1295	110	1
1295	79	1
1295	560	1
1295	561	1
1295	538	1
1295	923	1
1295	1775	0
1295	1223	0
1295	3514	0
1295	1169	0
1295	62	0
1295	3057	0
1295	3675	0
1295	476	0
1296	608	1
1296	354	1
1296	998	1
1296	48	1

1296	1947	1
1296	1693	1
1296	2271	1
1296	1494	0
1296	2774	0
1296	438	0
1296	681	0
1296	868	0
1296	342	0
1296	1518	0
1297	2116	1
1297	1868	1
1297	302	1
1297	78	1
1297	560	1
1297	46	1
1297	305	1
1297	1391	1
1297	3214	1
1297	1589	1
1297	1503	1
1297	1162	0
1297	802	0
1297	3073	0
1297	3523	0
1297	1682	0
1297	1436	0
1297	2283	0
1297	2628	0
1297	3758	0

1297	3130	0
1297	1524	0
1298	832	1
1298	75	1
1298	76	1
1298	3215	1
1298	3216	1
1298	951	1
1298	473	1
1298	1469	1
1298	1943	0
1298	3662	0
1298	12	0
1298	770	0
1298	3087	0
1298	1505	0
1298	3723	0
1298	1719	0
1299	1315	1
1299	134	1
1299	714	1
1299	267	1
1299	76	1
1299	301	1
1299	78	1
1299	79	1
1299	47	1
1299	206	1
1299	3217	1
1299	118	1

1299	919	1
1299	184	1
1299	3051	0
1299	1902	0
1299	945	0
1299	1564	0
1299	156	0
1299	1606	0
1299	110	0
1299	3671	0
1299	870	0
1299	2544	0
1299	827	0
1299	3118	0
1299	1435	0
1299	3729	0
1300	98	1
1300	132	1
1300	135	1
1300	72	1
1300	73	1
1300	1258	1
1300	78	1
1300	79	1
1300	80	1
1300	81	1
1300	919	1
1300	692	1
1300	87	1
1300	184	1

1300	185	1
1300	538	1
1300	1199	0
1300	2017	0
1300	2545	0
1300	386	0
1300	3643	0
1300	1242	0
1300	2665	0
1300	453	0
1300	3405	0
1300	1096	0
1300	1327	0
1300	1057	0
1300	2589	0
1300	2939	0
1300	3714	0
1300	2614	0
1301	32	1
1301	192	1
1301	256	1
1301	257	1
1301	275	1
1301	52	1
1301	499	1
1301	26	1
1301	30	1
1301	2773	0
1301	2966	0
1301	2058	0

1301	3256	0
1301	2480	0
1301	1475	0
1301	1358	0
1301	3378	0
1301	790	0
1302	96	1
1302	100	1
1302	72	1
1302	136	1
1302	74	1
1302	75	1
1302	269	1
1302	1651	1
1302	87	1
1302	120	1
1302	377	1
1302	916	0
1302	3500	0
1302	3466	0
1302	1222	0
1302	196	0
1302	3680	0
1302	127	0
1302	3750	0
1302	206	0
1302	2279	0
1302	1550	0
1303	353	1
1303	385	1

1303	1959	1
1303	238	1
1303	239	1
1303	497	1
1303	114	1
1303	3218	1
1303	3219	1
1303	2997	1
1303	24	1
1303	1855	1
1303	782	0
1303	2177	0
1303	1352	0
1303	1829	0
1303	1772	0
1303	1101	0
1303	148	0
1303	887	0
1303	1321	0
1303	852	0
1303	2693	0
1303	2445	0
1304	96	1
1304	130	1
1304	74	1
1304	714	1
1304	76	1
1304	77	1
1304	78	1
1304	79	1

1304	184	1
1304	185	1
1304	3104	0
1304	161	0
1304	2362	0
1304	38	0
1304	331	0
1304	3040	0
1304	1750	0
1304	1082	0
1304	3655	0
1304	1725	0
1305	134	1
1305	263	1
1305	41	1
1305	267	1
1305	560	1
1305	1424	1
1305	115	1
1305	118	1
1305	58	1
1305	2695	0
1305	3818	0
1305	2967	0
1305	293	0
1305	417	0
1305	1019	0
1305	3643	0
1305	1437	0
1305	1047	0

1306	1664	1
1306	2144	1
1306	3008	1
1306	36	1
1306	2826	1
1306	878	1
1306	624	1
1306	50	1
1306	1717	1
1306	54	1
1306	3158	1
1306	24	1
1306	378	1
1306	635	1
1306	3103	1
1306	637	1
1306	895	1
1306	1596	0
1306	1411	0
1306	3425	0
1306	3029	0
1306	3645	0
1306	2821	0
1306	78	0
1306	2705	0
1306	804	0
1306	2432	0
1306	2289	0
1306	3423	0
1306	3660	0

1306	1363	0
1306	1595	0
1306	973	0
1306	819	0
1307	96	1
1307	98	1
1307	102	1
1307	267	1
1307	79	1
1307	47	1
1307	184	1
1307	1756	1
1307	127	1
1307	194	0
1307	3782	0
1307	2304	0
1307	533	0
1307	2710	0
1307	2163	0
1307	3304	0
1307	377	0
1307	3523	0
1308	109	1
1308	46	1
1308	47	1
1308	276	1
1308	858	1
1308	63	1
1308	4	0
1308	1068	0

1308	3102	0
1308	174 0	
1308	1561	0
1308	2324	0
1309	295 1	
1309	40 1	
1309	50 1	
1309	1235	1
1309	500 1	
1309	1237	1
1309	2066	1
1309	3220	1
1309	3221	1
1309	3222	1
1309	2298	1
1309	507 1	
1309	3223	1
1309	3397	0
1309	2143	0
1309	2974	0
1309	1410	0
1309	2039	0
1309	312 0	
1309	3639	0
1309	1447	0
1309	248 0	
1309	471 0	
1309	1643	0
1309	146 0	
1309	2906	0

1310	97	1
1310	771	1
1310	164	1
1310	197	1
1310	1979	1
1310	1039	1
1310	118	1
1310	727	1
1310	24	1
1310	443	1
1310	286	1
1310	3294	0
1310	2211	0
1310	205	0
1310	3686	0
1310	3321	0
1310	520	0
1310	1057	0
1310	2292	0
1310	1486	0
1310	2618	0
1310	876	0
1311	96	1
1311	192	1
1311	1697	1
1311	133	1
1311	263	1
1311	41	1
1311	74	1
1311	80	1

1311	210	1
1311	916	1
1311	2175	1
1311	831	1
1311	2722	0
1311	3506	0
1311	3228	0
1311	2791	0
1311	2286	0
1311	759	0
1311	2048	0
1311	248	0
1311	33	0
1311	649	0
1311	371	0
1311	2462	0
1312	98	1
1312	72	1
1312	75	1
1312	237	1
1312	79	1
1312	1202	1
1312	115	1
1312	87	1
1312	184	1
1312	377	1
1312	127	1
1312	1703	0
1312	3179	0
1312	2297	0

1312	447	0
1312	3024	0
1312	2695	0
1312	538	0
1312	109	0
1312	2120	0
1312	565	0
1312	134	0
1313	96	1
1313	1122	1
1313	99	1
1313	3226	1
1313	2877	1
1313	493	1
1313	976	1
1313	3224	1
1313	120	1
1313	3225	1
1313	2074	1
1313	124	1
1313	2717	1
1313	344	0
1313	1790	0
1313	2192	0
1313	441	0
1313	3663	0
1313	1250	0
1313	3838	0
1313	562	0
1313	32	0

1313	402	0
1313	1874	0
1313	1372	0
1313	2721	0
1314	96	1
1314	36	1
1314	133	1
1314	105	1
1314	46	1
1314	47	1
1314	52	1
1314	118	1
1314	1023	0
1314	885	0
1314	1599	0
1314	3511	0
1314	2577	0
1314	2336	0
1314	548	0
1314	756	0
1315	1536	1
1315	516	1
1315	453	1
1315	295	1
1315	104	1
1315	108	1
1315	111	1
1315	48	1
1315	56	1
1315	57	1

1315	159	1
1315	3201	0
1315	1928	0
1315	1326	0
1315	2530	0
1315	2296	0
1315	780	0
1315	3319	0
1315	911	0
1315	3529	0
1315	1431	0
1315	752	0
1316	96	1
1316	132	1
1316	133	1
1316	134	1
1316	171	1
1316	80	1
1316	87	1
1316	185	1
1316	1659	1
1316	63	1
1316	1440	0
1316	22	0
1316	1110	0
1316	2349	0
1316	1088	0
1316	3124	0
1316	3280	0
1316	1787	0

1316	2654	0
1316	2433	0
1317	96	1
1317	34	1
1317	771	1
1317	100	1
1317	293	1
1317	199	1
1317	2027	1
1317	3227	1
1317	47	1
1317	16	1
1317	52	1
1317	2841	1
1317	1691	1
1317	3228	1
1317	126	1
1317	286	1
1317	1463	0
1317	2805	0
1317	342	0
1317	3767	0
1317	1655	0
1317	2490	0
1317	2480	0
1317	1444	0
1317	705	0
1317	2518	0
1317	3454	0
1317	1255	0

1317	1515	0
1317	2675	0
1317	2688	0
1317	959	0
1318	128	1
1318	773	1
1318	136	1
1318	104	1
1318	2540	1
1318	78	1
1318	80	1
1318	1011	1
1318	3229	1
1318	376	1
1318	570	1
1318	2269	1
1318	413	1
1318	382	1
1318	849	0
1318	2496	0
1318	2721	0
1318	3312	0
1318	1023	0
1318	2419	0
1318	1618	0
1318	153	0
1318	231	0
1318	3761	0
1318	2799	0
1318	2225	0

1318	919	0
1318	3140	0
1319	196	1
1319	517	1
1319	582	1
1319	154	1
1319	40	1
1319	998	1
1319	1496	1
1319	843	1
1319	460	1
1319	2281	1
1319	2030	1
1319	2590	1
1319	21	1
1319	24	1
1319	56	1
1319	3230	1
1319	732	1
1319	574	1
1319	735	0
1319	2656	0
1319	748	0
1319	16	0
1319	1832	0
1319	1029	0
1319	2394	0
1319	1578	0
1319	584	0
1319	3112	0

1319	3363	0
1319	326	0
1319	65	0
1319	2897	0
1319	2639	0
1319	1644	0
1319	3502	0
1319	187	0
1320	1408	1
1320	899	1
1320	74	1
1320	554	1
1320	1034	1
1320	1325	1
1320	80	1
1320	81	1
1320	371	1
1320	508	1
1320	1342	1
1320	3231	1
1320	2597	0
1320	1573	0
1320	2101	0
1320	2272	0
1320	2850	0
1320	265	0
1320	1651	0
1320	3050	0
1320	2822	0
1320	3015	0

1320	690	0
1320	1880	0
1321	96	1
1321	130	1
1321	376	1
1321	1448	1
1321	267	1
1321	78	1
1321	79	1
1321	47	1
1321	1651	1
1321	118	1
1321	184	1
1321	538	1
1321	1676	0
1321	1933	0
1321	2504	0
1321	72	0
1321	1853	0
1321	464	0
1321	2493	0
1321	2874	0
1321	1415	0
1321	2309	0
1321	1464	0
1321	1700	0
1322	2592	1
1322	3232	1
1322	3233	1
1322	3234	1

1322	3235	1
1322	3236	1
1322	3237	1
1322	3238	1
1322	574	1
1322	2281	1
1322	1236	1
1322	2590	1
1322	767	1
1322	268	0
1322	2708	0
1322	2353	0
1322	2712	0
1322	906	0
1322	2134	0
1322	1714	0
1322	1404	0
1322	605	0
1322	2013	0
1322	402	0
1322	3559	0
1322	761	0
1323	98	1
1323	131	1
1323	104	1
1323	137	1
1323	78	1
1323	79	1
1323	81	1
1323	118	1

1323	184	1
1323	185	1
1323	538	1
1323	2453	0
1323	1774	0
1323	3512	0
1323	109	0
1323	2257	0
1323	3501	0
1323	3133	0
1323	2415	0
1323	161	0
1323	1910	0
1323	3026	0
1324	102	1
1324	295	1
1324	136	1
1324	3239	1
1324	208	1
1324	2323	1
1324	52	1
1324	212	1
1324	213	1
1324	119	1
1324	382	0
1324	122	0
1324	2690	0
1324	3741	0
1324	1715	0
1324	2043	0

1324	3507	0
1324	570	0
1324	3731	0
1324	2936	0
1325	96	1
1325	353	1
1325	1889	1
1325	3046	1
1325	3240	1
1325	110	1
1325	78	1
1325	47	1
1325	494	1
1325	1781	1
1325	1020	1
1325	413	1
1325	254	1
1325	3086	0
1325	3092	0
1325	10	0
1325	2500	0
1325	2757	0
1325	3565	0
1325	3462	0
1325	2626	0
1325	1898	0
1325	798	0
1325	3776	0
1325	333	0
1325	2793	0

1326	96	1
1326	2307	1
1326	102	1
1326	391	1
1326	392	1
1326	105	1
1326	1126	1
1326	427	1
1326	3241	1
1326	46	1
1326	47	1
1326	528	1
1326	1103	1
1326	55	1
1326	1854	1
1326	1119	0
1326	3553	0
1326	2981	0
1326	72	0
1326	679	0
1326	1274	0
1326	3513	0
1326	728	0
1326	118	0
1326	2652	0
1326	420	0
1326	3205	0
1326	1353	0
1326	1745	0
1326	2709	0

1327	96	1
1327	133	1
1327	171	1
1327	267	1
1327	47	1
1327	303	1
1327	118	1
1327	247	1
1327	3310	0
1327	646	0
1327	1227	0
1327	2470	0
1327	2577	0
1327	3809	0
1327	2681	0
1327	3626	0
1328	96	1
1328	827	1
1328	2402	1
1328	3242	1
1328	3243	1
1328	3244	1
1328	208	1
1328	1777	1
1328	210	1
1328	824	1
1328	219	1
1328	1438	1
1328	2523	0
1328	1386	0

1328	961	0
1328	1982	0
1328	2052	0
1328	3579	0
1328	773	0
1328	3081	0
1328	1866	0
1328	532	0
1328	214	0
1328	2416	0
1329	296	1
1329	429	1
1329	46	1
1329	399	1
1329	1231	1
1329	3245	1
1329	1397	1
1329	572	1
1329	895	1
1329	486	0
1329	633	0
1329	3409	0
1329	3366	0
1329	3757	0
1329	3251	0
1329	2489	0
1329	2030	0
1329	2260	0
1330	96	1
1330	34	1

1330	391	1
1330	105	1
1330	107	1
1330	46	1
1330	52	1
1330	277	1
1330	26	1
1330	221	1
1330	190	1
1330	3307	0
1330	2527	0
1330	3632	0
1330	643	0
1330	3801	0
1330	635	0
1330	1580	0
1330	2203	0
1330	299	0
1330	730	0
1330	212	0
1331	36	1
1331	325	1
1331	2951	1
1331	42	1
1331	298	1
1331	3247	1
1331	3248	1
1331	3249	1
1331	3250	1
1331	533	1

1331	249	1
1331	2371	0
1331	3181	0
1331	3555	0
1331	891	0
1331	1430	0
1331	1415	0
1331	2831	0
1331	2256	0
1331	2996	0
1331	1536	0
1331	3292	0
1332	96	1
1332	66	1
1332	1122	1
1332	72	1
1332	74	1
1332	238	1
1332	47	1
1332	1040	1
1332	3251	1
1332	118	1
1332	87	1
1332	120	1
1332	1788	1
1332	126	1
1332	3241	0
1332	1056	0
1332	2360	0
1332	2888	0

1332	2027	0
1332	2409	0
1332	2308	0
1332	656	0
1332	3022	0
1332	1678	0
1332	2353	0
1332	298	0
1332	2635	0
1332	3824	0
1333	96	1
1333	3255	1
1333	1029	1
1333	969	1
1333	208	1
1333	209	1
1333	210	1
1333	242	1
1333	212	1
1333	3252	1
1333	3253	1
1333	535	1
1333	216	1
1333	3254	1
1333	347	1
1333	2878	1
1333	214	0
1333	3467	0
1333	1231	0
1333	2316	0

1333	3155	0
1333	649	0
1333	1134	0
1333	2961	0
1333	2854	0
1333	2064	0
1333	2051	0
1333	932	0
1333	2738	0
1333	2072	0
1333	333	0
1333	621	0
1334	361	1
1334	107	1
1334	524	1
1334	1324	1
1334	46	1
1334	465	1
1334	55	1
1334	733	1
1334	127	1
1334	3396	0
1334	3823	0
1334	1906	0
1334	2767	0
1334	78	0
1334	645	0
1334	2566	0
1334	2456	0
1334	998	0

1335	2083	1
1335	923	1
1335	261	1
1335	269	1
1335	2548	1
1335	118	1
1335	3256	1
1335	91	1
1335	253	1
1335	127	1
1335	893	0
1335	1017	0
1335	2750	0
1335	1504	0
1335	3381	0
1335	170	0
1335	1279	0
1335	1413	0
1335	2386	0
1335	1516	0
1336	928	1
1336	673	1
1336	35	1
1336	1288	1
1336	3258	1
1336	42	1
1336	2317	1
1336	398	1
1336	15	1
1336	2736	1

1336	401	1
1336	402	1
1336	3259	1
1336	3257	1
1336	1722	1
1336	1307	1
1336	3260	1
1336	223	0
1336	2027	0
1336	2478	0
1336	133	0
1336	1829	0
1336	665	0
1336	165	0
1336	1280	0
1336	833	0
1336	977	0
1336	56	0
1336	3579	0
1336	3695	0
1336	3052	0
1336	3187	0
1336	3756	0
1336	1063	0
1337	1346	1
1337	392	1
1337	105	1
1337	552	1
1337	2504	1
1337	46	1

1337	47	1
1337	87	1
1337	3261	1
1337	190	1
1337	127	1
1337	732	0
1337	3168	0
1337	2994	0
1337	880	0
1337	143	0
1337	3745	0
1337	857	0
1337	327	0
1337	2947	0
1337	1439	0
1337	1	0
1338	3040	1
1338	769	1
1338	3264	1
1338	35	1
1338	2660	1
1338	165	1
1338	2057	1
1338	1514	1
1338	1803	1
1338	2067	1
1338	888	1
1338	3262	1
1338	3263	1
1338	3243	0

1338	988	0
1338	427	0
1338	176	0
1338	2868	0
1338	1447	0
1338	237	0
1338	3792	0
1338	1823	0
1338	211	0
1338	2642	0
1338	2945	0
1338	3570	0
1339	354	1
1339	293	1
1339	295	1
1339	876	1
1339	109	1
1339	112	1
1339	275	1
1339	52	1
1339	187	1
1339	732	1
1339	445	1
1339	190	1
1339	909	0
1339	1386	0
1339	3384	0
1339	3788	0
1339	2967	0
1339	324	0

1339	3210	0
1339	996 0	
1339	3427	0
1339	507 0	
1339	2884	0
1339	170 0	
1340	3265	1
1340	3266	1
1340	3267	1
1340	3268	1
1340	3269	1
1340	1734	1
1340	3270	1
1340	3271	1
1340	362 1	
1340	1973	1
1340	2501	0
1340	742 0	
1340	1682	0
1340	906 0	
1340	1761	0
1340	871 0	
1340	2246	0
1340	1629	0
1340	2829	0
1340	2605	0
1341	224 1	
1341	1057	1
1341	2661	1
1341	3272	1

1341	1833	1
1341	3273	1
1341	883 1	
1341	277 1	
1341	1754	1
1341	300 0	
1341	1099	0
1341	598 0	
1341	3795	0
1341	2363	0
1341	2635	0
1341	581 0	
1341	3346	0
1341	733 0	
1342	3274	1
1342	3275	1
1342	3276	1
1342	3277	1
1342	3278	1
1342	3279	1
1342	3280	1
1342	3165	0
1342	3092	0
1342	676 0	
1342	2280	0
1342	2095	0
1342	3787	0
1342	2932	0
1343	608 1	
1343	2882	1

1343	4	1
1343	1605	1
1343	38	1
1343	3079	1
1343	8	1
1343	329	1
1343	332	1
1343	337	1
1343	3281	1
1343	3282	1
1343	1754	1
1343	2487	0
1343	1629	0
1343	2000	0
1343	1234	0
1343	2241	0
1343	3026	0
1343	2363	0
1343	1657	0
1343	2358	0
1343	2240	0
1343	3233	0
1343	3537	0
1343	1844	0
1344	208	1
1344	658	1
1344	1075	1
1344	1076	1
1344	2995	1
1344	214	1

1344	3283	1
1344	3284	1
1344	218 1	
1344	220 1	
1344	3295	0
1344	1080	0
1344	2907	0
1344	3327	0
1344	288 0	
1344	3203	0
1344	1415	0
1344	387 0	
1344	162 0	
1344	1190	0
1345	1312	1
1345	3287	1
1345	295 1	
1345	104 1	
1345	3079	1
1345	2298	1
1345	703 1	
1345	1042	1
1345	1618	1
1345	21 1	
1345	3285	1
1345	3286	1
1345	1114	1
1345	187 1	
1345	957 1	
1345	223 1	

1345	1834	0
1345	1709	0
1345	817	0
1345	2107	0
1345	437	0
1345	954	0
1345	1683	0
1345	3437	0
1345	2066	0
1345	3066	0
1345	640	0
1345	3755	0
1345	3501	0
1345	1205	0
1345	2389	0
1345	3513	0
1346	136	1
1346	367	1
1346	119	1
1346	3288	1
1346	3289	1
1346	3290	1
1346	155	1
1346	124	1
1346	286	1
1346	1311	1
1346	1885	0
1346	409	0
1346	176	0
1346	3044	0

1346	1988	0
1346	2937	0
1346	132	0
1346	3666	0
1346	1980	0
1346	892	0
1347	2113	1
1347	483	1
1347	356	1
1347	357	1
1347	519	1
1347	361	1
1347	457	1
1347	107	1
1347	1513	1
1347	2090	1
1347	3291	1
1347	3292	1
1347	24	1
1347	155	1
1347	156	1
1347	3293	1
1347	863	1
1347	2119	0
1347	827	0
1347	3427	0
1347	2049	0
1347	151	0
1347	1105	0
1347	1179	0

1347	1608	0
1347	131 0	
1347	1276	0
1347	644 0	
1347	2098	0
1347	3437	0
1347	514 0	
1347	1155	0
1347	3581	0
1347	2472	0
1348	96 1	
1348	2371	1
1348	3235	1
1348	499 1	
1348	119 1	
1348	24 1	
1348	474 1	
1348	445 1	
1348	126 1	
1348	3796	0
1348	1642	0
1348	3170	0
1348	2088	0
1348	3562	0
1348	289 0	
1348	1747	0
1348	3001	0
1348	315 0	
1349	293 1	
1349	107 1	

1349	111	1
1349	53	1
1349	57	1
1349	221	1
1349	3294	1
1349	223	1
1349	3322	0
1349	1894	0
1349	3775	0
1349	2548	0
1349	1996	0
1349	366	0
1349	115	0
1349	3471	0
1350	3296	1
1350	3297	1
1350	70	1
1350	3208	1
1350	1229	1
1350	789	1
1350	60	1
1350	157	1
1350	3295	1
1350	1145	0
1350	354	0
1350	3464	0
1350	2766	0
1350	3788	0
1350	2368	0
1350	2573	0

1350	2750	0
1350	1987	0
1351	36	1
1351	133	1
1351	105	1
1351	109	1
1351	46	1
1351	114	1
1351	863	1
1351	1573	0
1351	594	0
1351	2043	0
1351	3842	0
1351	826	0
1351	1059	0
1351	291	0
1352	96	1
1352	1185	1
1352	1186	1
1352	771	1
1352	133	1
1352	134	1
1352	46	1
1352	80	1
1352	560	1
1352	1424	1
1352	52	1
1352	531	0
1352	1657	0
1352	638	0

1352	1825	0
1352	577	0
1352	2000	0
1352	434	0
1352	3287	0
1352	2940	0
1352	1997	0
1352	1317	0
1353	98	1
1353	133	1
1353	134	1
1353	135	1
1353	171	1
1353	2603	1
1353	206	1
1353	115	1
1353	118	1
1353	87	1
1353	374	1
1353	377	1
1353	1265	0
1353	2248	0
1353	2975	0
1353	926	0
1353	609	0
1353	853	0
1353	3428	0
1353	2747	0
1353	2573	0
1353	1838	0

1353	3523	0
1353	362	0
1354	352	1
1354	738	1
1354	891	1
1354	1255	1
1354	681	1
1354	110	1
1354	1039	1
1354	475	1
1354	221	1
1354	1102	0
1354	252	0
1354	1486	0
1354	3498	0
1354	2049	0
1354	3163	0
1354	3137	0
1354	2676	0
1354	2079	0
1355	257	1
1355	227	1
1355	36	1
1355	38	1
1355	487	1
1355	887	1
1355	498	1
1355	499	1
1355	22	1
1355	502	1

1355	155	1
1355	221	1
1355	30	1
1355	1311	1
1355	2013	0
1355	1084	0
1355	2136	0
1355	3824	0
1355	1306	0
1355	445	0
1355	3292	0
1355	3207	0
1355	2885	0
1355	146	0
1355	3226	0
1355	2101	0
1355	1086	0
1355	246	0
1356	2912	1
1356	1186	1
1356	66	1
1356	1413	1
1356	3301	1
1356	135	1
1356	3302	1
1356	3303	1
1356	3052	1
1356	237	1
1356	1463	1
1356	1659	1

1356	1471	0
1356	3261	0
1356	1196	0
1356	1933	0
1356	1665	0
1356	2324	0
1356	2573	0
1356	3694	0
1356	3363	0
1356	3016	0
1356	1437	0
1356	323 0	
1357	41 1	
1357	830 1	
1357	52 1	
1357	55 1	
1357	118 1	
1357	87 1	
1357	1524	1
1357	1821	1
1357	542 1	
1357	2082	0
1357	1416	0
1357	835 0	
1357	1776	0
1357	2211	0
1357	1724	0
1357	734 0	
1357	2135	0
1357	1187	0

1358	1219	1
1358	90	1
1358	133	1
1358	137	1
1358	74	1
1358	75	1
1358	399	1
1358	81	1
1358	117	1
1358	55	1
1358	377	1
1358	58	1
1358	3341	0
1358	1400	0
1358	2476	0
1358	2302	0
1358	894	0
1358	3426	0
1358	561	0
1358	3350	0
1358	914	0
1358	2369	0
1358	3811	0
1358	666	0
1359	224	1
1359	608	1
1359	3304	1
1359	3305	1
1359	883	1
1359	53	1

1359	221	1
1359	3294	1
1359	511	1
1359	3758	0
1359	210	0
1359	3060	0
1359	1809	0
1359	2670	0
1359	2334	0
1359	574	0
1359	3748	0
1359	796	0
1360	96	1
1360	354	1
1360	802	1
1360	105	1
1360	267	1
1360	46	1
1360	47	1
1360	302	1
1360	915	1
1360	118	1
1360	254	1
1360	703	0
1360	134	0
1360	1994	0
1360	1795	0
1360	966	0
1360	176	0
1360	2835	0

1360	2028	0
1360	2571	0
1360	3036	0
1360	2780	0
1361	354	1
1361	131	1
1361	3306	1
1361	3307	1
1361	46	1
1361	47	1
1361	1135	1
1361	945	1
1361	22	1
1361	279	1
1361	764	1
1361	1038	0
1361	3264	0
1361	3449	0
1361	1691	0
1361	3086	0
1361	2383	0
1361	1474	0
1361	1410	0
1361	2331	0
1361	2177	0
1361	1555	0
1362	2520	1
1362	1258	1
1362	75	1
1362	184	1

1362	538	1
1362	3644	0
1362	73	0
1362	29	0
1362	1002	0
1362	2193	0
1363	36	1
1363	295	1
1363	457	1
1363	3308	1
1363	109	1
1363	46	1
1363	111	1
1363	210	1
1363	2066	1
1363	52	1
1363	1175	1
1363	1080	1
1363	2609	0
1363	2834	0
1363	3811	0
1363	1712	0
1363	1500	0
1363	2819	0
1363	1349	0
1363	69	0
1363	2230	0
1363	1426	0
1363	664	0
1363	3448	0

1364	16	1
1364	282	1
1364	155	1
1364	36	1
1364	2469	1
1364	44	1
1364	52	1
1364	53	1
1364	716	1
1364	863	1
1364	102	1
1364	230	1
1364	1126	1
1364	105	1
1364	1131	1
1364	3309	1
1364	1262	1
1364	3310	1
1364	1015	1
1364	1673	0
1364	1444	0
1364	3254	0
1364	582	0
1364	3536	0
1364	503	0
1364	813	0
1364	3182	0
1364	1024	0
1364	768	0
1364	3152	0

1364	3756	0
1364	1581	0
1364	1855	0
1364	3441	0
1364	642	0
1364	2450	0
1364	2505	0
1364	3444	0
1365	2400	1
1365	98	1
1365	185	1
1365	3048	1
1365	74	1
1365	75	1
1365	206	1
1365	79	1
1365	2128	1
1365	437	1
1365	184	1
1365	89	1
1365	1244	1
1365	395	0
1365	1889	0
1365	2064	0
1365	2933	0
1365	674	0
1365	826	0
1365	2058	0
1365	629	0
1365	433	0

1365	1292	0
1365	904	0
1365	3260	0
1365	2748	0
1366	3171	1
1366	764	1
1366	107	1
1366	46	1
1366	304	1
1366	528	1
1366	722	1
1366	1396	1
1366	1621	1
1366	54	1
1366	55	1
1366	1305	1
1366	572	1
1366	2910	1
1366	236	0
1366	1451	0
1366	1675	0
1366	1003	0
1366	242	0
1366	1342	0
1366	3717	0
1366	3698	0
1366	358	0
1366	3117	0
1366	3679	0
1366	3802	0

1366	2332	0
1366	748	0
1367	130	1
1367	134	1
1367	135	1
1367	171	1
1367	364	1
1367	77	1
1367	46	1
1367	206	1
1367	2603	1
1367	81	1
1367	117	1
1367	374	1
1367	87	1
1367	185	1
1367	698	1
1367	197	0
1367	2914	0
1367	47	0
1367	985	0
1367	1127	0
1367	3509	0
1367	2536	0
1367	184	0
1367	1957	0
1367	1518	0
1367	417	0
1367	1163	0
1367	3490	0

1367	1364	0
1367	1061	0
1368	738	1
1368	36	1
1368	102	1
1368	1254	1
1368	392	1
1368	105	1
1368	1256	1
1368	109	1
1368	941	1
1368	52	1
1368	2519	1
1368	60	1
1368	326	0
1368	1893	0
1368	3793	0
1368	1472	0
1368	3669	0
1368	3127	0
1368	1865	0
1368	1378	0
1368	2210	0
1368	2262	0
1368	388	0
1368	3029	0
1369	928	1
1369	393	1
1369	937	1
1369	1388	1

1369	12	1
1369	2508	1
1369	3311	1
1369	3312	1
1369	3102	1
1369	282	0
1369	3498	0
1369	2631	0
1369	166	0
1369	1099	0
1369	3232	0
1369	2734	0
1369	3702	0
1369	2266	0
1370	132	1
1370	134	1
1370	74	1
1370	75	1
1370	399	1
1370	81	1
1370	863	1
1370	87	1
1370	378	1
1370	254	1
1370	127	1
1370	2872	0
1370	2343	0
1370	3537	0
1370	2128	0
1370	2063	0

1370	280	0
1370	3357	0
1370	3394	0
1370	2148	0
1370	1208	0
1370	3612	0
1371	66	1
1371	2564	1
1371	133	1
1371	232	1
1371	1228	1
1371	1325	1
1371	81	1
1371	145	1
1371	3313	1
1371	377	1
1371	58	1
1371	208	0
1371	3286	0
1371	3266	0
1371	2098	0
1371	931	0
1371	3843	0
1371	623	0
1371	759	0
1371	916	0
1371	262	0
1371	989	0
1372	96	1
1372	34	1

1372	2819	1
1372	36	1
1372	199	1
1372	776	1
1372	43	1
1372	45	1
1372	654	1
1372	22	1
1372	118	1
1372	442	1
1372	60	1
1372	989	1
1372	2564	0
1372	3041	0
1372	1379	0
1372	73	0
1372	1775	0
1372	427	0
1372	235	0
1372	3473	0
1372	593	0
1372	1470	0
1372	3536	0
1372	1616	0
1372	1305	0
1372	1524	0
1373	832	1
1373	2401	1
1373	131	1
1373	102	1

1373	777	1
1373	1257	1
1373	267	1
1373	78	1
1373	47	1
1373	1583	1
1373	3314	1
1373	406	1
1373	185	1
1373	2881	0
1373	1258	0
1373	1741	0
1373	2151	0
1373	3596	0
1373	1913	0
1373	909	0
1373	3631	0
1373	3078	0
1373	1935	0
1373	3672	0
1373	1546	0
1373	691	0
1374	771	1
1374	2499	1
1374	7	1
1374	393	1
1374	241	1
1374	52	1
1374	991	1
1374	396	0

1374	2096	0
1374	3708	0
1374	391	0
1374	3097	0
1374	1828	0
1374	139	0
1375	772	1
1375	133	1
1375	102	1
1375	773	1
1375	41	1
1375	1322	1
1375	267	1
1375	1259	1
1375	1231	1
1375	1397	1
1375	1589	1
1375	188	1
1375	1268	0
1375	399	0
1375	1480	0
1375	3661	0
1375	974	0
1375	2160	0
1375	1975	0
1375	717	0
1375	1619	0
1375	3321	0
1375	1754	0
1375	2465	0

1376	640	1
1376	36	1
1376	293	1
1376	102	1
1376	105	1
1376	511	1
1376	118	1
1376	55	1
1376	154	1
1376	221	1
1376	286	1
1376	223	1
1376	108	0
1376	2928	0
1376	2024	0
1376	1213	0
1376	899	0
1376	3094	0
1376	1859	0
1376	573	0
1376	711	0
1376	2962	0
1376	351	0
1376	2432	0
1377	1153	1
1377	391	1
1377	105	1
1377	110	1
1377	46	1
1377	528	1

1377	3315	1
1377	190	1
1377	765	1
1377	382	1
1377	863	1
1377	1676	0
1377	1670	0
1377	2220	0
1377	1202	0
1377	3164	0
1377	2591	0
1377	3602	0
1377	2068	0
1377	994	0
1377	860	0
1377	3445	0
1378	800	1
1378	131	1
1378	132	1
1378	230	1
1378	202	1
1378	237	1
1378	78	1
1378	47	1
1378	1020	1
1378	2749	1
1378	126	1
1378	937	0
1378	1013	0
1378	285	0

1378	3833	0
1378	2968	0
1378	577	0
1378	919	0
1378	3534	0
1378	2836	0
1378	3478	0
1378	2653	0
1379	161	1
1379	257	1
1379	131	1
1379	1542	1
1379	1896	1
1379	265	1
1379	457	1
1379	111	1
1379	16	1
1379	498	1
1379	1235	1
1379	3316	1
1379	2613	1
1379	22	1
1379	2302	1
1379	858	1
1379	2046	1
1379	799	1
1379	1965	0
1379	2823	0
1379	1462	0
1379	829	0

1379	589	0
1379	2263	0
1379	693	0
1379	2422	0
1379	1104	0
1379	3330	0
1379	3132	0
1379	3829	0
1379	593	0
1379	1598	0
1379	1418	0
1379	2076	0
1379	3731	0
1379	3238	0
1380	257	1
1380	259	1
1380	36	1
1380	53	1
1380	501	1
1380	3317	1
1380	542	1
1380	1399	0
1380	464	0
1380	3765	0
1380	983	0
1380	1022	0
1380	2067	0
1380	3802	0
1381	2752	1
1381	245	1

1381	1311	1
1381	232	0
1381	1743	0
1381	2493	0
1382	96	1
1382	1315	1
1382	1799	1
1382	40	1
1382	841	1
1382	303	1
1382	830	1
1382	915	1
1382	61	1
1382	254	1
1382	2129	0
1382	2382	0
1382	2437	0
1382	1608	0
1382	615	0
1382	200	0
1382	722	0
1382	3631	0
1382	477	0
1382	2849	0
1383	1315	1
1383	105	1
1383	44	1
1383	1388	1
1383	1104	1
1383	1777	1

1383	1526	1
1383	219	1
1383	188	1
1383	221	1
1383	190	1
1383	799	1
1383	3572	0
1383	1620	0
1383	1065	0
1383	1386	0
1383	2192	0
1383	957	0
1383	2840	0
1383	2697	0
1383	1568	0
1383	3391	0
1383	1865	0
1383	827	0
1384	764	1
1384	136	1
1384	105	1
1384	109	1
1384	46	1
1384	528	1
1384	2995	1
1384	1364	1
1384	54	1
1384	254	1
1384	219	1
1384	124	1

1384	190	1
1384	1254	0
1384	1831	0
1384	1489	0
1384	3327	0
1384	1010	0
1384	19	0
1384	597	0
1384	662	0
1384	218	0
1384	1403	0
1384	3755	0
1384	1814	0
1384	245	0
1385	96	1
1385	354	1
1385	1091	1
1385	388	1
1385	293	1
1385	1347	1
1385	199	1
1385	107	1
1385	235	1
1385	46	1
1385	111	1
1385	1103	1
1385	1502	1
1385	1736	0
1385	508	0
1385	1446	0

1385	2530	0
1385	3111	0
1385	3700	0
1385	2434	0
1385	446	0
1385	1182	0
1385	291	0
1385	2082	0
1385	1941	0
1385	3304	0
1386	36	1
1386	37	1
1386	391	1
1386	392	1
1386	105	1
1386	46	1
1386	1103	1
1386	145	1
1386	530	1
1386	1042	1
1386	3318	1
1386	572	1
1386	766	1
1386	863	1
1386	2410	0
1386	3489	0
1386	1006	0
1386	3302	0
1386	1270	0
1386	3019	0

1386	491	0
1386	2284	0
1386	1168	0
1386	1952	0
1386	1286	0
1386	2175	0
1386	1631	0
1386	3771	0
1387	0	1
1387	4	1
1387	8	1
1387	654	1
1387	36	1
1387	307	1
1387	52	1
1387	2118	1
1387	1607	1
1387	2120	1
1387	329	1
1387	1739	1
1387	332	1
1387	334	1
1387	975	1
1387	337	1
1387	1499	1
1387	109	1
1387	498	1
1387	1267	1
1387	1929	0
1387	1404	0

1387	3106	0
1387	1031	0
1387	316	0
1387	509	0
1387	3093	0
1387	27	0
1387	3838	0
1387	55	0
1387	3100	0
1387	3273	0
1387	1110	0
1387	3074	0
1387	3499	0
1387	2742	0
1387	2985	0
1387	1449	0
1387	1030	0
1387	2219	0
1388	171	1
1388	3223	0
1389	96	1
1389	2557	1
1389	1315	1
1389	133	1
1389	1318	1
1389	104	1
1389	681	1
1389	334	1
1389	442	1
1389	1395	1

1389	438	1
1389	3319	1
1389	857	1
1389	378	1
1389	1244	1
1389	1085	1
1389	3530	0
1389	1957	0
1389	3711	0
1389	689	0
1389	1062	0
1389	1771	0
1389	1441	0
1389	1565	0
1389	1631	0
1389	3659	0
1389	3123	0
1389	1662	0
1389	1088	0
1389	2360	0
1389	2225	0
1389	2972	0
1390	36	1
1390	2884	1
1390	141	1
1390	46	1
1390	850	1
1390	1140	1
1390	1526	1
1390	2323	0

1390	3825	0
1390	3117	0
1390	1113	0
1390	3764	0
1390	3294	0
1390	3292	0
1391	1188	1
1391	72	1
1391	74	1
1391	75	1
1391	1258	1
1391	80	1
1391	830	1
1391	87	1
1391	254	1
1391	127	1
1391	3503	0
1391	184	0
1391	81	0
1391	252	0
1391	1276	0
1391	360	0
1391	3101	0
1391	3444	0
1391	908	0
1391	3196	0
1392	928	1
1392	289	1
1392	34	1
1392	1217	1

1392	36	1
1392	2118	1
1392	2504	1
1392	42	1
1392	109	1
1392	366	1
1392	879	1
1392	1878	1
1392	285	1
1392	2133	0
1392	954	0
1392	424	0
1392	791	0
1392	181	0
1392	1672	0
1392	2557	0
1392	3663	0
1392	1833	0
1392	2465	0
1392	1316	0
1392	4	0
1392	2566	0
1393	196	1
1393	517	1
1393	198	1
1393	486	1
1393	2281	1
1393	460	1
1393	2030	1
1393	2578	1

1393	727	1
1393	3320	1
1393	314	1
1393	155	1
1393	156	1
1393	1099	0
1393	2512	0
1393	979	0
1393	3627	0
1393	3246	0
1393	687	0
1393	2107	0
1393	3699	0
1393	683	0
1393	1892	0
1393	383	0
1393	2637	0
1393	3264	0
1394	96	1
1394	37	1
1394	105	1
1394	714	1
1394	432	1
1394	723	1
1394	118	1
1394	55	1
1394	124	1
1394	254	1
1394	2807	0
1394	3327	0

1394	3558	0
1394	788	0
1394	240	0
1394	1977	0
1394	930	0
1394	149	0
1394	1685	0
1394	713	0
1395	96	1
1395	354	1
1395	1186	1
1395	133	1
1395	102	1
1395	776	1
1395	41	1
1395	105	1
1395	46	1
1395	80	1
1395	304	1
1395	52	1
1395	55	1
1395	188	1
1395	2156	0
1395	1823	0
1395	980	0
1395	1833	0
1395	3689	0
1395	3162	0
1395	2789	0
1395	2713	0

1395	2313	0
1395	840	0
1395	3014	0
1395	3384	0
1395	933	0
1395	3236	0
1396	130	1
1396	868	1
1396	134	1
1396	560	1
1396	376	1
1396	1016	1
1396	1020	1
1396	63	1
1396	159	1
1396	3738	0
1396	3714	0
1396	2879	0
1396	2074	0
1396	1968	0
1396	3311	0
1396	3421	0
1396	1933	0
1396	3068	0
1397	96	1
1397	46	1
1397	52	1
1397	3321	1
1397	863	1
1397	1466	0

1397	1085	0
1397	3280	0
1397	631	0
1397	914	0
1398	3041	1
1398	1348	1
1398	2504	1
1398	202	1
1398	368	1
1398	371	1
1398	662	1
1398	3322	1
1398	3263	0
1398	2198	0
1398	3701	0
1398	1008	0
1398	3169	0
1398	843	0
1398	1602	0
1398	2350	0
1399	130	1
1399	135	1
1399	171	1
1399	363	1
1399	79	1
1399	80	1
1399	87	1
1399	184	1
1399	185	1
1399	538	1

1399	2164	0
1399	778	0
1399	3727	0
1399	1660	0
1399	136	0
1399	589	0
1399	437	0
1399	1310	0
1399	1554	0
1399	1696	0
1400	163	1
1400	388	1
1400	293	1
1400	391	1
1400	1095	1
1400	105	1
1400	46	1
1400	112	1
1400	55	1
1400	3129	0
1400	608	0
1400	2030	0
1400	969	0
1400	3144	0
1400	1402	0
1400	68	0
1400	2584	0
1400	3835	0
1401	708	1
1401	102	1

1401	136	1
1401	105	1
1401	107	1
1401	46	1
1401	111	1
1401	55	1
1401	571	1
1401	187	1
1401	572	1
1401	863	1
1401	924	0
1401	1051	0
1401	174	0
1401	3741	0
1401	878	0
1401	1572	0
1401	918	0
1401	241	0
1401	267	0
1401	1939	0
1401	3645	0
1401	950	0
1402	1408	1
1402	98	1
1402	132	1
1402	1477	1
1402	269	1
1402	177	1
1402	721	1
1402	116	1

1402	87	1
1402	185	1
1402	3323	1
1402	126	1
1402	605	0
1402	1150	0
1402	258	0
1402	1187	0
1402	1856	0
1402	2385	0
1402	1826	0
1402	1663	0
1402	1791	0
1402	138	0
1402	3784	0
1402	1753	0
1403	1186	1
1403	1156	1
1403	134	1
1403	171	1
1403	365	1
1403	1742	1
1403	79	1
1403	47	1
1403	560	1
1403	118	1
1403	1462	1
1403	3324	1
1403	2958	0
1403	625	0

1403	1040	0
1403	2966	0
1403	3051	0
1403	684	0
1403	793	0
1403	2467	0
1403	2104	0
1403	3091	0
1403	1776	0
1403	3506	0
1404	353	1
1404	1315	1
1404	36	1
1404	137	1
1404	75	1
1404	235	1
1404	110	1
1404	399	1
1404	468	1
1404	2070	1
1404	184	1
1404	3325	1
1404	187	1
1404	413	1
1404	895	1
1404	1605	0
1404	914	0
1404	1812	0
1404	737	0
1404	809	0

1404	1532	0
1404	3017	0
1404	2216	0
1404	3267	0
1404	687	0
1404	3454	0
1404	2958	0
1404	1749	0
1404	3124	0
1404	2847	0
1405	72	1
1405	74	1
1405	171	1
1405	237	1
1405	79	1
1405	87	1
1405	119	0
1405	3036	0
1405	3681	0
1405	3251	0
1405	3714	0
1405	2165	0
1406	391	1
1406	392	1
1406	105	1
1406	429	1
1406	110	1
1406	46	1
1406	1363	1
1406	52	1

1406	2777	1
1406	3326	1
1406	190	1
1406	98	0
1406	1372	0
1406	2842	0
1406	508	0
1406	977	0
1406	1823	0
1406	2024	0
1406	3271	0
1406	2113	0
1406	2312	0
1406	218	0
1407	96	1
1407	992	1
1407	738	1
1407	1477	1
1407	2342	1
1407	1095	1
1407	392	1
1407	522	1
1407	524	1
1407	525	1
1407	46	1
1407	529	1
1407	52	1
1407	119	1
1407	1020	1
1407	190	1

1407	2473	0
1407	1264	0
1407	1985	0
1407	2575	0
1407	3294	0
1407	2796	0
1407	1326	0
1407	836	0
1407	3119	0
1407	1625	0
1407	2719	0
1407	2858	0
1407	581	0
1407	2439	0
1407	2801	0
1407	3516	0
1408	3328	1
1408	2436	1
1408	617	1
1408	1801	1
1408	141	1
1408	695	1
1408	2299	1
1408	2046	1
1408	3327	1
1408	1919	0
1408	3305	0
1408	1010	0
1408	3427	0
1408	70	0

1408	2421	0
1408	1532	0
1408	1712	0
1408	2918	0
1409	3329	1
1409	3330	1
1409	3331	1
1409	3332	1
1409	133	1
1409	102	1
1409	3027	1
1409	277	1
1409	473	1
1409	2228	0
1409	1365	0
1409	3739	0
1409	1490	0
1409	3123	0
1409	3484	0
1409	1924	0
1409	861	0
1409	108	0
1410	96	1
1410	1857	1
1410	98	1
1410	130	1
1410	1889	1
1410	177	1
1410	2548	1
1410	2261	1

1410	87	1
1410	254	1
1410	127	1
1410	3816	0
1410	53	0
1410	305	0
1410	178	0
1410	196	0
1410	1828	0
1410	932	0
1410	2725	0
1410	3430	0
1410	3681	0
1410	2635	0
1411	98	1
1411	132	1
1411	135	1
1411	72	1
1411	73	1
1411	237	1
1411	81	1
1411	87	1
1411	569	0
1411	571	0
1411	1624	0
1411	2302	0
1411	3145	0
1411	2775	0
1411	3700	0
1411	679	0

1412	67	1
1412	1764	1
1412	391	1
1412	136	1
1412	105	1
1412	1255	1
1412	46	1
1412	1678	1
1412	863	1
1412	55	1
1412	413	1
1412	766	1
1412	703	1
1412	3749	0
1412	2689	0
1412	1664	0
1412	2160	0
1412	3116	0
1412	955	0
1412	3300	0
1412	627	0
1412	1940	0
1412	2747	0
1412	3841	0
1412	3699	0
1412	1011	0
1413	96	1
1413	131	1
1413	133	1
1413	3333	1

1413	72	1
1413	377	1
1413	74	1
1413	75	1
1413	203	1
1413	237	1
1413	79	1
1413	116	1
1413	87	1
1413	184	1
1413	185	1
1413	546	0
1413	258	0
1413	148	0
1413	2098	0
1413	3673	0
1413	3111	0
1413	1972	0
1413	2906	0
1413	1982	0
1413	2249	0
1413	3216	0
1413	2051	0
1413	761	0
1413	3570	0
1413	1366	0
1414	353	1
1414	2020	1
1414	1416	1
1414	235	1

1414	2643	1
1414	52	1
1414	53	1
1414	863	1
1414	55	1
1414	1240	1
1414	1396	1
1414	858	1
1414	219	1
1414	223	1
1414	1444	0
1414	780	0
1414	3618	0
1414	267	0
1414	2496	0
1414	3197	0
1414	3021	0
1414	2164	0
1414	3793	0
1414	3296	0
1414	2921	0
1414	1221	0
1414	2680	0
1414	1114	0
1415	388	1
1415	3012	1
1415	3334	1
1415	3335	1
1415	1130	1
1415	1261	1

1415	1389	1
1415	47	1
1415	282	1
1415	1180	1
1415	1502	1
1415	2652	1
1415	2170	0
1415	3112	0
1415	128	0
1415	3021	0
1415	3166	0
1415	458	0
1415	2360	0
1415	1625	0
1415	2268	0
1415	1357	0
1415	22	0
1415	2336	0
1416	165	1
1416	293	1
1416	3336	1
1416	3053	1
1416	526	1
1416	118	1
1416	55	1
1416	472	1
1416	57	1
1416	1341	1
1416	221	1
1416	2656	0

1416	981	0
1416	2391	0
1416	1606	0
1416	1175	0
1416	1442	0
1416	2276	0
1416	3305	0
1416	2565	0
1416	882	0
1416	861	0
1417	96	1
1417	833	1
1417	102	1
1417	1126	1
1417	3337	1
1417	3338	1
1417	301	1
1417	47	1
1417	304	1
1417	210	1
1417	118	1
1417	1015	1
1417	1144	1
1417	569	1
1417	190	1
1417	988	0
1417	3324	0
1417	2256	0
1417	966	0
1417	3356	0

1417	1501	0
1417	1265	0
1417	3043	0
1417	1486	0
1417	3789	0
1417	2783	0
1417	1257	0
1417	953	0
1417	3120	0
1417	643	0
1418	3339	1
1418	196	1
1418	261	1
1418	1310	1
1418	1864	0
1418	1096	0
1418	3510	0
1418	1292	0
1419	992	1
1419	393	1
1419	210	1
1419	52	1
1419	346	1
1419	991	1
1419	2946	0
1419	605	0
1419	401	0
1419	3835	0
1419	355	0
1419	3809	0

1420	133	1
1420	1551	1
1420	20	1
1420	286	1
1420	34	1
1420	36	1
1420	37	1
1420	293	1
1420	40	1
1420	54	1
1420	956	1
1420	452	1
1420	1220	1
1420	854	1
1420	355	1
1420	356	1
1420	1891	1
1420	998	1
1420	617	1
1420	253	1
1420	3467	0
1420	1038	0
1420	2051	0
1420	1527	0
1420	2606	0
1420	3259	0
1420	3349	0
1420	560	0
1420	1820	0
1420	2874	0

1420	1980	0
1420	1374	0
1420	319	0
1420	2570	0
1420	3834	0
1420	171	0
1420	2667	0
1420	1375	0
1420	3244	0
1420	834	0
1421	672	1
1421	1575	1
1421	979	1
1421	788	1
1421	377	1
1421	1439	1
1421	1647	0
1421	3250	0
1421	619	0
1421	1048	0
1421	2922	0
1421	1588	0
1422	1216	1
1422	165	1
1422	395	1
1422	300	1
1422	3340	1
1422	398	1
1422	3341	1
1422	2736	1

1422	210	1
1422	1075	1
1422	212	1
1422	216	1
1422	3257	1
1422	218	1
1422	253	1
1422	126	1
1422	1973	0
1422	2767	0
1422	3568	0
1422	207	0
1422	1346	0
1422	507	0
1422	742	0
1422	730	0
1422	1917	0
1422	2688	0
1422	872	0
1422	2275	0
1422	698	0
1422	2302	0
1422	1062	0
1422	347	0
1423	131	1
1423	72	1
1423	74	1
1423	861	1
1423	77	1
1423	237	1

1423	957	1
1423	3151	1
1423	3342	1
1423	117	1
1423	87	1
1423	24	1
1423	858	1
1423	88	1
1423	413	1
1423	127	1
1423	530	0
1423	3808	0
1423	85	0
1423	820	0
1423	2127	0
1423	866	0
1423	2820	0
1423	3166	0
1423	1641	0
1423	1585	0
1423	1111	0
1423	2976	0
1423	742	0
1423	1323	0
1423	3750	0
1423	3734	0
1424	1570	1
1424	74	1
1424	79	1
1424	80	1

1424	372	1
1424	87	1
1424	127	1
1424	605	0
1424	850	0
1424	3009	0
1424	1289	0
1424	237	0
1424	61	0
1424	3627	0
1425	96	1
1425	652	1
1425	1040	1
1425	52	1
1425	55	1
1425	119	1
1425	2468	0
1425	2445	0
1425	1760	0
1425	3049	0
1425	3381	0
1425	562	0
1426	128	1
1426	1570	1
1426	2562	1
1426	2054	1
1426	1258	1
1426	77	1
1426	397	1
1426	1232	1

1426	81	1
1426	58	1
1426	3675	0
1426	1742	0
1426	283	0
1426	1728	0
1426	515	0
1426	2215	0
1426	1294	0
1426	821	0
1426	2203	0
1426	2605	0
1427	96	1
1427	1253	1
1427	102	1
1427	105	1
1427	523	1
1427	46	1
1427	114	1
1427	91	1
1427	188	1
1427	286	1
1427	2200	0
1427	1463	0
1427	3648	0
1427	2948	0
1427	3188	0
1427	89	0
1427	1004	0
1427	3475	0

1427	2447	0
1427	1766	0
1428	164	1
1428	36	1
1428	2180	1
1428	879	1
1428	499	1
1428	500	1
1428	2325	1
1428	55	1
1428	2231	1
1428	191	1
1428	1285	0
1428	2855	0
1428	3442	0
1428	2861	0
1428	2821	0
1428	1252	0
1428	2781	0
1428	476	0
1428	3169	0
1428	719	0
1429	36	1
1429	523	1
1429	141	1
1429	52	1
1429	568	1
1429	3266	0
1429	3130	0
1429	1709	0

1429	2869	0
1429	197	0
1430	1315	1
1430	36	1
1430	105	1
1430	681	1
1430	2069	1
1430	127	1
1430	692	0
1430	600	0
1430	2897	0
1430	3609	0
1430	905	0
1430	516	0
1431	102	1
1431	3246	1
1431	3343	1
1431	304	1
1431	1523	1
1431	212	1
1431	219	1
1431	3697	0
1431	370	0
1431	1797	0
1431	1719	0
1431	270	0
1431	2422	0
1431	2716	0
1432	131	1
1432	132	1

1432	230	1
1432	137	1
1432	1789	1
1432	79	1
1432	80	1
1432	81	1
1432	919	1
1432	3344	1
1432	117	1
1432	87	1
1432	957	1
1432	1268	0
1432	714	0
1432	3638	0
1432	2718	0
1432	3116	0
1432	3254	0
1432	3468	0
1432	2352	0
1432	3489	0
1432	1237	0
1432	1479	0
1432	3115	0
1432	1579	0
1433	0	1
1433	224	1
1433	2882	1
1433	933	1
1433	934	1
1433	935	1

1433	937	1
1433	1999	1
1433	273	1
1433	3345	1
1433	3346	1
1433	277	1
1433	856	0
1433	2200	0
1433	1256	0
1433	2741	0
1433	138	0
1433	2144	0
1433	2963	0
1433	1969	0
1433	1426	0
1433	3598	0
1433	2484	0
1433	1667	0
1434	0	1
1434	2529	1
1434	835	1
1434	6	1
1434	43	1
1434	334	1
1434	655	1
1434	2256	1
1434	209	1
1434	337	1
1434	276	1
1434	3348	1

1434	118	1
1434	1272	1
1434	1118	1
1434	1082	1
1434	253	1
1434	446	1
1434	2717	0
1434	1095	0
1434	1634	0
1434	3183	0
1434	2520	0
1434	3588	0
1434	2583	0
1434	2964	0
1434	1633	0
1434	1985	0
1434	126	0
1434	836	0
1434	1776	0
1434	1715	0
1434	507	0
1434	2441	0
1434	250	0
1434	320	0
1435	1344	1
1435	130	1
1435	137	1
1435	74	1
1435	107	1
1435	77	1

1435	80	1
1435	1200	1
1435	2064	1
1435	118	1
1435	378	1
1435	253	1
1435	2089	0
1435	601	0
1435	3538	0
1435	2702	0
1435	3782	0
1435	1467	0
1435	2079	0
1435	2810	0
1435	2460	0
1435	1014	0
1435	684	0
1435	139	0
1436	137	1
1436	171	1
1436	79	1
1436	55	1
1436	469	1
1436	87	1
1436	184	1
1436	185	1
1436	1114	0
1436	1135	0
1436	2582	0
1436	1495	0

1436	3839	0
1436	2554	0
1436	2605	0
1436	1347	0
1437	98	1
1437	130	1
1437	376	1
1437	135	1
1437	363	1
1437	78	1
1437	79	1
1437	206	1
1437	118	1
1437	184	1
1437	185	1
1437	538	1
1437	3602	0
1437	322	0
1437	3037	0
1437	588	0
1437	2244	0
1437	1886	0
1437	2152	0
1437	776	0
1437	1503	0
1437	1349	0
1437	1635	0
1437	1671	0
1438	133	1
1438	230	1

1438	391	1
1438	105	1
1438	107	1
1438	46	1
1438	114	1
1438	115	1
1438	3349	1
1438	3350	1
1438	55	1
1438	1080	1
1438	190	1
1438	863	1
1438	2048	0
1438	1457	0
1438	3092	0
1438	1310	0
1438	3450	0
1438	5	0
1438	1508	0
1438	3252	0
1438	1916	0
1438	2740	0
1438	1304	0
1438	1253	0
1438	2494	0
1438	3103	0
1439	358	1
1439	494	1
1439	47	1
1439	210	1

1439	118	1
1439	3354	1
1439	124	1
1439	155	0
1439	1632	0
1439	862	0
1439	2933	0
1439	473	0
1439	2834	0
1439	712	0
1440	96	1
1440	131	1
1440	3359	1
1440	808	1
1440	1322	1
1440	107	1
1440	3358	1
1440	495	1
1440	80	1
1440	2484	1
1440	3356	1
1440	474	1
1440	3355	1
1440	1404	1
1440	3357	1
1440	286	1
1440	863	1
1440	377	0
1440	1413	0
1440	2162	0

1440	2017	0
1440	2974	0
1440	6	0
1440	1065	0
1440	438	0
1440	2349	0
1440	581	0
1440	2094	0
1440	3159	0
1440	508	0
1440	1370	0
1440	1643	0
1440	3309	0
1440	924	0
1441	738	1
1441	102	1
1441	906	1
1441	109	1
1441	47	1
1441	210	1
1441	52	1
1441	118	1
1441	989	1
1441	1168	0
1441	3380	0
1441	2030	0
1441	845	0
1441	1162	0
1441	3553	0
1441	2218	0

1441	3117	0
1441	1411	0
1442	3360	1
1442	66	1
1442	72	1
1442	87	1
1442	126	1
1442	539	1
1442	254	1
1442	51	0
1442	2459	0
1442	2414	0
1442	774	0
1442	1903	0
1442	2252	0
1442	2879	0
1443	1057	1
1443	3361	1
1443	3362	1
1443	3363	1
1443	3364	1
1443	141	1
1443	2577	1
1443	882	1
1443	2943	1
1443	1592	1
1443	799	1
1443	2371	0
1443	609	0
1443	223	0

1443	274	0
1443	2669	0
1443	3240	0
1443	2261	0
1443	1852	0
1443	2938	0
1443	2194	0
1443	965	0
1444	3203	1
1444	524	1
1444	527	1
1444	1046	1
1444	2085	1
1444	3365	1
1444	3366	1
1444	3367	1
1444	3368	1
1444	2474	1
1444	3369	1
1444	308	1
1444	320	1
1444	852	1
1444	1493	1
1444	1495	1
1444	1636	1
1444	500	1
1444	502	1
1444	887	1
1444	1662	1
1444	1407	0

1444	3052	0
1444	1547	0
1444	2616	0
1444	1421	0
1444	89	0
1444	520	0
1444	346	0
1444	2691	0
1444	307	0
1444	3063	0
1444	2871	0
1444	2446	0
1444	1296	0
1444	774	0
1444	245	0
1444	1969	0
1444	2801	0
1444	359	0
1444	74	0
1444	132	0
1445	96	1
1445	34	1
1445	3	1
1445	902	1
1445	7	1
1445	42	1
1445	109	1
1445	944	1
1445	209	1
1445	215	1

1445	154	1
1445	446	1
1445	1341	0
1445	2754	0
1445	655	0
1445	1395	0
1445	1953	0
1445	3493	0
1445	3221	0
1445	1644	0
1445	2167	0
1445	872	0
1445	698	0
1445	90	0
1446	128	1
1446	866	1
1446	1570	1
1446	196	1
1446	165	1
1446	102	1
1446	197	1
1446	1031	1
1446	137	1
1446	3370	1
1446	459	1
1446	1100	1
1446	1131	1
1446	3371	1
1446	3372	1
1446	1363	1

1446	3354	1
1446	1921	0
1446	2366	0
1446	261	0
1446	1395	0
1446	2285	0
1446	3441	0
1446	2674	0
1446	548	0
1446	1339	0
1446	877	0
1446	448	0
1446	2098	0
1446	1587	0
1446	711	0
1446	3515	0
1446	2668	0
1446	1091	0
1447	353	1
1447	321	1
1447	1605	1
1447	38	1
1447	2823	1
1447	2950	1
1447	2157	1
1447	3373	1
1447	3374	1
1447	465	1
1447	1267	1
1447	22	1

1447	24	1
1447	912	0
1447	2620	0
1447	2953	0
1447	3378	0
1447	3385	0
1447	99	0
1447	3510	0
1447	3503	0
1447	3765	0
1447	1326	0
1447	507	0
1447	3026	0
1447	1156	0
1448	128	1
1448	1088	1
1448	422	1
1448	2406	1
1448	2666	1
1448	141	1
1448	2063	1
1448	1776	1
1448	3375	1
1448	3376	1
1448	883	1
1448	1493	1
1448	727	1
1448	889	1
1448	2330	1
1448	799	1

1448	2753	0
1448	170	0
1448	1693	0
1448	1483	0
1448	2167	0
1448	1519	0
1448	1045	0
1448	1224	0
1448	1807	0
1448	2218	0
1448	2056	0
1448	2123	0
1448	2156	0
1448	1203	0
1448	1127	0
1448	3681	0
1449	929	1
1449	34	1
1449	42	1
1449	1662	1
1449	314	1
1449	1554	1
1449	211	1
1449	756	1
1449	1397	1
1449	3092	1
1449	90	1
1449	507	1
1449	285	1
1449	286	1

1449	1052	0
1449	2949	0
1449	2577	0
1449	2532	0
1449	1049	0
1449	3658	0
1449	108	0
1449	2642	0
1449	2564	0
1449	2939	0
1449	2596	0
1449	130	0
1449	1797	0
1449	2779	0
1450	159	1
1450	3175	1
1450	1546	1
1450	2506	1
1450	1004	1
1450	1326	1
1450	3377	1
1450	1555	1
1450	2645	1
1450	24	1
1450	56	1
1450	413	1
1450	223	1
1450	347	0
1450	787	0
1450	1615	0

1450	155 0	
1450	715 0	
1450	1197	0
1450	1723	0
1450	2699	0
1450	3564	0
1450	3369	0
1450	123 0	
1450	2414	0
1450	2546	0
1451	1429	1
1451	2336	1
1451	549 1	
1451	2098	1
1451	3378	1
1451	3379	1
1451	3380	1
1451	3381	1
1451	3382	1
1451	3384	1
1451	3385	1
1451	3386	1
1451	3383	1
1451	3387	1
1451	3388	1
1451	3389	1
1451	1220	1
1451	478 1	
1451	3183	1
1451	880 1	

1451	893	1
1451	254	1
1451	633	0
1451	2024	0
1451	1925	0
1451	3627	0
1451	179	0
1451	2348	0
1451	3584	0
1451	2654	0
1451	2617	0
1451	2804	0
1451	421	0
1451	1360	0
1451	2651	0
1451	43	0
1451	1640	0
1451	2232	0
1451	1068	0
1451	2828	0
1451	326	0
1451	1151	0
1451	1142	0
1451	1601	0
1452	36	1
1452	774	1
1452	359	1
1452	2760	1
1452	788	1
1452	21	1

1452	1236	1
1452	1526	1
1452	57	1
1452	60	1
1452	3390	1
1452	447	1
1452	2465	0
1452	3371	0
1452	913	0
1452	1162	0
1452	3536	0
1452	1767	0
1452	2129	0
1452	2116	0
1452	1079	0
1452	1220	0
1452	346	0
1452	1616	0
1453	608	1
1453	36	1
1453	2318	1
1453	1140	1
1453	346	1
1453	264	0
1453	2015	0
1453	110	0
1453	2676	0
1453	2848	0
1454	135	1
1454	137	1

1454	1258	1
1454	1324	1
1454	77	1
1454	206	1
1454	80	1
1454	117	1
1454	87	1
1454	185	1
1454	698	1
1454	923	1
1454	3667	0
1454	753	0
1454	251	0
1454	3730	0
1454	879	0
1454	1413	0
1454	3280	0
1454	2016	0
1454	3505	0
1454	3571	0
1454	182	0
1454	3311	0
1455	96	1
1455	3392	1
1455	36	1
1455	1041	1
1455	151	1
1455	442	1
1455	3391	1
1455	3685	0

1455	3337	0
1455	3130	0
1455	576	0
1455	3382	0
1455	2387	0
1455	0	0
1456	3393	1
1456	388	1
1456	104	1
1456	877	1
1456	24	1
1456	187	1
1456	223	1
1456	1220	0
1456	3103	0
1456	3429	0
1456	1345	0
1456	394	0
1456	1332	0
1456	1574	0
1457	66	1
1457	98	1
1457	130	1
1457	1186	1
1457	3394	1
1457	135	1
1457	73	1
1457	2602	1
1457	364	1
1457	79	1

1457	80	1
1457	560	1
1457	376	1
1457	699	1
1457	1527	0
1457	316	0
1457	3164	0
1457	2183	0
1457	575	0
1457	3387	0
1457	1043	0
1457	1491	0
1457	3764	0
1457	113	0
1457	1835	0
1457	2468	0
1457	290	0
1457	151	0
1458	899	1
1458	132	1
1458	2758	1
1458	135	1
1458	74	1
1458	80	1
1458	469	1
1458	87	1
1458	2234	1
1458	127	1
1458	1220	0
1458	2861	0

1458	3033	0
1458	2165	0
1458	2903	0
1458	2143	0
1458	1862	0
1458	2650	0
1458	1547	0
1458	846	0
1459	3395	1
1459	3396	1
1459	3533	0
1459	677	0
1460	354	1
1460	36	1
1460	388	1
1460	105	1
1460	297	1
1460	109	1
1460	1389	1
1460	2254	1
1460	52	1
1460	1396	1
1460	118	1
1460	55	1
1460	286	1
1460	703	1
1460	2085	0
1460	2705	0
1460	865	0
1460	2037	0

1460	3842	0
1460	1499	0
1460	3121	0
1460	3188	0
1460	3566	0
1460	2585	0
1460	555	0
1460	3676	0
1460	3382	0
1460	1701	0
1461	96	1
1461	131	1
1461	356	1
1461	134	1
1461	41	1
1461	74	1
1461	267	1
1461	80	1
1461	560	1
1461	54	1
1461	22	1
1461	863	1
1461	458	0
1461	2670	0
1461	2875	0
1461	2084	0
1461	696	0
1461	1830	0
1461	758	0
1461	2673	0

1461	1004	0
1461	2388	0
1461	3	0
1461	2805	0
1462	388	1
1462	104	1
1462	106	1
1462	876	1
1462	110	1
1462	111	1
1462	2290	1
1462	55	1
1462	54	1
1462	22	1
1462	252	1
1462	1109	0
1462	278	0
1462	3325	0
1462	1988	0
1462	533	0
1462	1390	0
1462	3607	0
1462	2032	0
1462	3107	0
1462	2086	0
1462	4	0
1463	928	1
1463	165	1
1463	3269	1
1463	3397	1

1463	3398	1
1463	3399	1
1463	362	1
1463	1514	1
1463	1010	1
1463	1528	1
1463	315	1
1463	2638	0
1463	3797	0
1463	1984	0
1463	3783	0
1463	1866	0
1463	3282	0
1463	2435	0
1463	855	0
1463	716	0
1463	2015	0
1463	301	0
1464	608	1
1464	97	1
1464	36	1
1464	43	1
1464	189	1
1464	191	1
1464	766	0
1464	1886	0
1464	503	0
1464	1788	0
1464	769	0
1464	1367	0

1465	3400	1
1465	526	1
1465	241	1
1465	242	1
1465	756	1
1465	726	1
1465	118	1
1465	991	1
1465	1693	0
1465	2078	0
1465	776	0
1465	655	0
1465	2967	0
1465	36	0
1465	1105	0
1465	1139	0
1466	161	1
1466	163	1
1466	484	1
1466	296	1
1466	3401	1
1466	491	1
1466	492	1
1466	2412	1
1466	2414	1
1466	3086	1
1466	3088	1
1466	145	1
1466	2418	1
1466	499	1

1466	21	1
1466	2359	1
1466	2719	1
1466	2130	0
1466	1344	0
1466	367	0
1466	3316	0
1466	3236	0
1466	1778	0
1466	1438	0
1466	661	0
1466	1372	0
1466	1173	0
1466	556	0
1466	1487	0
1466	2071	0
1466	2558	0
1466	1286	0
1466	2353	0
1466	579	0
1467	129	1
1467	3233	1
1467	261	1
1467	3238	1
1467	3402	1
1467	3403	1
1467	3404	1
1467	3405	1
1467	3406	1
1467	3223	1

1467	2649	1
1467	445	1
1467	574	1
1467	767	1
1467	3454	0
1467	1684	0
1467	120	0
1467	1539	0
1467	3680	0
1467	3174	0
1467	1707	0
1467	1579	0
1467	154	0
1467	2828	0
1467	2950	0
1467	1771	0
1467	3608	0
1467	1498	0
1468	32	1
1468	72	1
1468	267	1
1468	2700	1
1468	237	1
1468	557	1
1468	207	1
1468	2065	1
1468	254	1
1468	1061	0
1468	1529	0
1468	420	0

1468	2932	0
1468	584 0	
1468	1490	0
1468	1939	0
1468	1607	0
1468	1666	0
1469	3104	1
1469	740 1	
1469	36 1	
1469	2414	1
1469	145 1	
1469	2098	1
1469	276 1	
1469	3351	1
1469	2040	1
1469	1117	1
1469	3508	0
1469	1643	0
1469	1931	0
1469	523 0	
1469	3637	0
1469	3702	0
1469	2410	0
1469	986 0	
1469	1773	0
1469	1286	0
1470	132 1	
1470	389 1	
1470	104 1	
1470	137 1	

1470	74	1
1470	233	1
1470	842	1
1470	2923	1
1470	399	1
1470	145	1
1470	433	1
1470	469	1
1470	1141	1
1470	55	1
1470	378	1
1470	957	1
1470	1918	1
1470	3354	0
1470	1433	0
1470	269	0
1470	2779	0
1470	1791	0
1470	2090	0
1470	775	0
1470	3643	0
1470	919	0
1470	994	0
1470	2387	0
1470	3390	0
1470	1685	0
1470	1570	0
1470	1316	0
1470	871	0
1470	3493	0

1471	96	1
1471	522	1
1471	3020	1
1471	79	1
1471	80	1
1471	239	1
1471	115	1
1471	1278	1
1471	118	1
1471	87	1
1471	120	1
1471	377	1
1471	184	1
1471	1479	0
1471	2580	0
1471	1285	0
1471	2955	0
1471	3644	0
1471	2249	0
1471	1368	0
1471	3624	0
1471	3783	0
1471	2524	0
1471	3648	0
1471	65	0
1471	3841	0
1472	66	1
1472	354	1
1472	1923	1
1472	1325	1

1472	110	1
1472	1392	1
1472	3408	1
1472	117	1
1472	53	0
1472	373	0
1472	848	0
1472	1742	0
1472	1323	0
1472	77	0
1472	766	0
1472	629	0
1473	96	1
1473	74	1
1473	43	1
1473	302	1
1473	118	1
1473	126	1
1473	434	0
1473	3416	0
1473	1373	0
1473	1092	0
1473	3736	0
1473	3024	0
1474	1416	1
1474	105	1
1474	1801	1
1474	2512	1
1474	1777	1
1474	476	1

1474	1023	1
1474	894	0
1474	3639	0
1474	2514	0
1474	3316	0
1474	3013	0
1474	491	0
1474	172	0
1475	96	1
1475	2877	1
1475	46	1
1475	976	1
1475	115	1
1475	52	1
1475	119	1
1475	118	1
1475	87	1
1475	1277	1
1475	2056	0
1475	1117	0
1475	917	0
1475	504	0
1475	731	0
1475	147	0
1475	316	0
1475	2424	0
1475	2982	0
1475	2708	0
1476	1347	1
1476	104	1

1476	681	1
1476	13	1
1476	275	1
1476	277	1
1476	1973	1
1476	1278	1
1476	223	1
1476	829	0
1476	3778	0
1476	1870	0
1476	1185	0
1476	691	0
1476	3003	0
1476	3672	0
1476	1839	0
1476	2691	0
1477	419	1
1477	3299	1
1477	511	1
1477	719	1
1477	3410	1
1477	3411	1
1477	3412	1
1477	3413	1
1477	3414	1
1477	3415	1
1477	3416	1
1477	3417	1
1477	670	1
1477	1343	1

1477	3604	0
1477	2211	0
1477	3709	0
1477	2700	0
1477	1972	0
1477	3047	0
1477	3768	0
1477	3126	0
1477	2851	0
1477	2953	0
1477	840	0
1477	2176	0
1477	1606	0
1477	2660	0
1478	289	1
1478	34	1
1478	36	1
1478	40	1
1478	1225	1
1478	42	1
1478	43	1
1478	1673	1
1478	45	1
1478	2060	1
1478	3418	1
1478	250	1
1478	3419	1
1478	2889	0
1478	141	0
1478	2699	0

1478	2637	0
1478	2693	0
1478	2818	0
1478	1620	0
1478	2086	0
1478	48	0
1478	2398	0
1478	1177	0
1478	988	0
1478	3028	0
1479	2273	1
1479	387	1
1479	772	1
1479	137	1
1479	696	1
1479	1048	1
1479	187	1
1479	572	1
1479	190	1
1479	1268	0
1479	207	0
1479	1578	0
1479	2520	0
1479	1528	0
1479	3282	0
1479	2063	0
1479	3143	0
1479	1842	0
1480	196	1
1480	36	1

1480	198	1
1480	486	1
1480	519	1
1480	939	1
1480	3083	1
1480	1617	1
1480	727	1
1480	3161	1
1480	154	1
1480	3420	1
1480	3421	1
1480	3422	1
1480	3423	1
1480	3646	0
1480	890	0
1480	1463	0
1480	583	0
1480	682	0
1480	3163	0
1480	3770	0
1480	711	0
1480	2387	0
1480	1067	0
1480	1459	0
1480	2842	0
1480	3805	0
1480	796	0
1480	2314	0
1481	3424	1
1481	1153	1

1481	171	1
1481	302	1
1481	55	1
1481	184	1
1481	859	1
1481	863	1
1481	1970	0
1481	2025	0
1481	603	0
1481	2378	0
1481	1173	0
1481	790	0
1481	1833	0
1481	3679	0
1482	1825	1
1482	3425	1
1482	167	1
1482	104	1
1482	202	1
1482	204	1
1482	79	1
1482	80	1
1482	2868	1
1482	3196	1
1482	413	1
1482	3136	0
1482	1951	0
1482	2452	0
1482	1177	0
1482	1074	0

1482	3191	0
1482	568	0
1482	2356	0
1482	1586	0
1482	408	0
1482	28	0
1483	928	1
1483	3426	1
1483	164	1
1483	36	1
1483	229	1
1483	932	1
1483	1222	1
1483	395	1
1483	306	1
1483	52	1
1483	2554	1
1483	541	1
1483	1022	1
1483	268	0
1483	2858	0
1483	1134	0
1483	3131	0
1483	2830	0
1483	2276	0
1483	1579	0
1483	864	0
1483	3287	0
1483	2788	0
1483	771	0

1483	1415	0
1483	2506	0
1484	2147	1
1484	133	1
1484	134	1
1484	137	1
1484	267	1
1484	46	1
1484	55	1
1484	184	1
1484	217	0
1484	2323	0
1484	760	0
1484	3801	0
1484	1401	0
1484	2702	0
1484	1909	0
1484	448	0
1485	418	1
1485	3427	1
1485	3428	1
1485	2565	1
1485	70	1
1485	3429	1
1485	1960	1
1485	590	1
1485	538	0
1485	1792	0
1485	628	0
1485	1643	0

1485	1177	0
1485	2963	0
1485	2609	0
1485	1599	0
1486	66	1
1486	72	1
1486	238	1
1486	79	1
1486	87	1
1486	539	1
1486	540	1
1486	706	0
1486	2382	0
1486	2079	0
1486	29	0
1486	2139	0
1486	1425	0
1486	2240	0
1487	96	1
1487	480	1
1487	1122	1
1487	772	1
1487	3430	1
1487	46	1
1487	1775	1
1487	1392	1
1487	305	1
1487	243	1
1487	2995	1
1487	118	1

1487	216	1
1487	2879	1
1487	3278	0
1487	1296	0
1487	1266	0
1487	1210	0
1487	1901	0
1487	2291	0
1487	947	0
1487	3756	0
1487	1288	0
1487	1804	0
1487	644	0
1487	3122	0
1487	331	0
1487	2055	0
1488	96	1
1488	3431	1
1488	77	1
1488	373	1
1488	124	1
1488	1663	0
1488	2681	0
1488	568	0
1488	195	0
1488	1728	0
1489	135	1
1489	72	1
1489	73	1
1489	75	1

1489	78	1
1489	79	1
1489	270	1
1489	81	1
1489	87	1
1489	127	1
1489	3590	0
1489	552	0
1489	1912	0
1489	138	0
1489	3768	0
1489	1945	0
1489	1199	0
1489	3788	0
1489	3126	0
1489	1859	0
1490	3432	1
1490	254	1
1490	546	0
1490	3357	0
1491	35	1
1491	154	1
1491	3433	1
1491	3434	1
1491	720	1
1491	24	1
1491	250	1
1491	3037	1
1491	886	0
1491	948	0

1491	2825	0
1491	1739	0
1491	1917	0
1491	2693	0
1491	3728	0
1491	1809	0
1492	96	1
1492	3435	1
1492	3436	1
1492	3437	1
1492	527	1
1492	756	1
1492	1238	1
1492	2834	0
1492	733	0
1492	2909	0
1492	493	0
1492	1514	0
1492	863	0
1492	1251	0
1493	325	1
1493	395	1
1493	3438	1
1493	3439	1
1493	2516	1
1493	1148	1
1493	2484	0
1493	411	0
1493	2156	0
1493	398	0

1493	3290	0
1493	536	0
1494	224	1
1494	297	1
1494	3440	1
1494	2066	1
1494	883	1
1494	3186	1
1494	1493	1
1494	1046	1
1494	55	1
1494	56	1
1494	187	1
1494	765	1
1494	223	1
1494	1958	0
1494	2845	0
1494	450	0
1494	1597	0
1494	1863	0
1494	2832	0
1494	3237	0
1494	1136	0
1494	287	0
1494	2478	0
1494	561	0
1494	110	0
1494	3100	0
1495	131	1
1495	133	1

1495	105	1
1495	2602	1
1495	107	1
1495	78	1
1495	46	1
1495	47	1
1495	3441	1
1495	114	1
1495	3442	1
1495	863	1
1495	63	1
1495	465	0
1495	1972	0
1495	1751	0
1495	3084	0
1495	2586	0
1495	2414	0
1495	132	0
1495	2644	0
1495	2226	0
1495	917	0
1495	759	0
1495	3598	0
1495	565	0
1496	992	1
1496	197	1
1496	524	1
1496	46	1
1496	526	1
1496	20	1

1496	52	1
1496	1204	1
1496	1662	1
1496	223	1
1496	2972	0
1496	2823	0
1496	2438	0
1496	510	0
1496	2334	0
1496	3743	0
1496	3103	0
1496	2707	0
1496	2	0
1496	358	0
1497	96	1
1497	385	1
1497	770	1
1497	1283	1
1497	494	1
1497	2323	1
1497	2264	1
1497	124	1
1497	3113	0
1497	1073	0
1497	2616	0
1497	2105	0
1497	1587	0
1497	927	0
1497	48	0
1497	2970	0

1498	96	1
1498	134	1
1498	171	1
1498	364	1
1498	79	1
1498	80	1
1498	560	1
1498	118	1
1498	87	1
1498	184	1
1498	185	1
1498	538	1
1498	830	1
1498	2063	0
1498	1419	0
1498	1451	0
1498	1299	0
1498	1685	0
1498	3354	0
1498	2865	0
1498	876	0
1498	1265	0
1498	2794	0
1498	492	0
1498	2735	0
1498	3238	0
1499	0	1
1499	96	1
1499	835	1
1499	1315	1

1499	199	1
1499	8	1
1499	1739	1
1499	332	1
1499	337	1
1499	915	1
1499	3443	1
1499	3356	1
1499	118	1
1499	1020	1
1499	936	0
1499	3020	0
1499	1595	0
1499	404	0
1499	1488	0
1499	1929	0
1499	3083	0
1499	2900	0
1499	2826	0
1499	1314	0
1499	2690	0
1499	2005	0
1499	1714	0
1499	2991	0
1500	928	1
1500	36	1
1500	133	1
1500	1798	1
1500	2632	1
1500	624	1

1500	2772	1
1500	3349	1
1500	1181	1
1500	3444	1
1500	3445	1
1500	1754	1
1500	637	1
1500	929	0
1500	2353	0
1500	3431	0
1500	1286	0
1500	2441	0
1500	2412	0
1500	2537	0
1500	3536	0
1500	1990	0
1500	3308	0
1500	1810	0
1500	686	0
1500	2176	0
1501	1122	1
1501	772	1
1501	235	1
1501	1868	1
1501	301	1
1501	305	1
1501	118	1
1501	55	1
1501	1226	0
1501	2596	0

1501	2999	0
1501	2860	0
1501	3313	0
1501	3800	0
1501	694	0
1501	134	0
1502	2434	1
1502	36	1
1502	165	1
1502	1259	1
1502	2028	1
1502	45	1
1502	46	1
1502	1231	1
1502	3446	1
1502	1016	1
1502	863	1
1502	205	0
1502	1562	0
1502	1832	0
1502	2407	0
1502	1894	0
1502	3188	0
1502	805	0
1502	1335	0
1502	214	0
1502	985	0
1502	2029	0
1503	133	1
1503	102	1

1503	41	1
1503	171	1
1503	560	1
1503	976	1
1503	87	1
1503	2232	1
1503	3447	1
1503	567	0
1503	1277	0
1503	3609	0
1503	2575	0
1503	399	0
1503	3458	0
1503	3314	0
1503	1025	0
1503	1094	0
1504	353	1
1504	105	1
1504	107	1
1504	267	1
1504	109	1
1504	46	1
1504	114	1
1504	1235	1
1504	52	1
1504	1396	1
1504	55	1
1504	2877	1
1504	1662	1
1504	863	1

1504	870	0
1504	2124	0
1504	1726	0
1504	761	0
1504	1567	0
1504	2229	0
1504	682	0
1504	3417	0
1504	1384	0
1504	50	0
1504	3034	0
1504	1607	0
1504	2178	0
1504	1562	0
1505	96	1
1505	133	1
1505	102	1
1505	105	1
1505	267	1
1505	78	1
1505	46	1
1505	47	1
1505	305	1
1505	118	1
1505	190	1
1505	1968	0
1505	552	0
1505	1056	0
1505	3515	0
1505	1381	0

1505	3158	0
1505	1967	0
1505	1854	0
1505	2491	0
1505	3402	0
1505	2859	0
1506	130	1
1506	1186	1
1506	80	1
1506	372	1
1506	374	1
1506	87	1
1506	376	1
1506	1310	0
1506	2626	0
1506	3513	0
1506	146	0
1506	445	0
1506	591	0
1506	2478	0
1507	353	1
1507	104	1
1507	137	1
1507	45	1
1507	187	1
1507	2226	1
1507	20	1
1507	1494	1
1507	413	1
1507	472	1

1507	57	1
1507	1114	1
1507	91	1
1507	61	1
1507	382	1
1507	159	1
1507	3468	0
1507	1288	0
1507	734	0
1507	2601	0
1507	2810	0
1507	369	0
1507	1010	0
1507	1407	0
1507	3028	0
1507	3255	0
1507	1865	0
1507	6	0
1507	834	0
1507	1273	0
1507	166	0
1507	1948	0
1508	2593	1
1508	196	1
1508	516	1
1508	422	1
1508	295	1
1508	519	1
1508	1545	1
1508	3448	1

1508	187	1
1508	621	1
1508	1006	1
1508	888	1
1508	155	1
1508	508	1
1508	3262	1
1508	3712	0
1508	1428	0
1508	559	0
1508	1683	0
1508	820	0
1508	1076	0
1508	2727	0
1508	995	0
1508	810	0
1508	954	0
1508	3603	0
1508	1254	0
1508	1776	0
1508	3477	0
1508	863	0
1509	131	1
1509	132	1
1509	1412	1
1509	230	1
1509	137	1
1509	3449	1
1509	237	1
1509	110	1

1509	22	1
1509	472	1
1509	2777	1
1509	187	1
1509	861	1
1509	127	1
1509	1845	0
1509	477	0
1509	1094	0
1509	43	0
1509	3580	0
1509	1105	0
1509	3022	0
1509	3355	0
1509	1262	0
1509	3505	0
1509	2495	0
1509	2219	0
1509	1887	0
1509	668	0
1510	356	1
1510	357	1
1510	360	1
1510	105	1
1510	457	1
1510	107	1
1510	1517	1
1510	46	1
1510	1234	1
1510	275	1

1510	52	1
1510	1079	1
1510	1308	1
1510	55	1
1510	24	1
1510	3450	1
1510	3451	1
1510	572	1
1510	358	0
1510	1408	0
1510	3322	0
1510	188	0
1510	1131	0
1510	1504	0
1510	858	0
1510	1384	0
1510	317	0
1510	325	0
1510	1103	0
1510	3546	0
1510	2249	0
1510	3680	0
1510	3352	0
1510	3646	0
1510	2098	0
1510	715	0
1511	353	1
1511	133	1
1511	1542	1
1511	74	1

1511	235	1
1511	78	1
1511	1395	1
1511	116	1
1511	276	1
1511	55	1
1511	1369	1
1511	1754	1
1511	1756	1
1511	158	0
1511	1617	0
1511	598	0
1511	728	0
1511	2940	0
1511	2757	0
1511	231	0
1511	2450	0
1511	2285	0
1511	1729	0
1511	3729	0
1511	2279	0
1511	2418	0
1512	96	1
1512	135	1
1512	78	1
1512	79	1
1512	87	1
1512	185	1
1512	923	1
1512	254	1

1512	127	1
1512	822	0
1512	2711	0
1512	1169	0
1512	718	0
1512	2147	0
1512	166	0
1512	2664	0
1512	908	0
1512	3180	0
1513	128	1
1513	67	1
1513	70	1
1513	71	1
1513	136	1
1513	908	1
1513	3084	1
1513	3187	1
1513	3453	1
1513	3452	1
1513	1565	1
1513	3454	1
1513	398	0
1513	931	0
1513	1666	0
1513	1001	0
1513	2087	0
1513	2537	0
1513	3558	0
1513	554	0

1513	1550	0
1513	953	0
1513	1988	0
1513	11	0
1514	387	1
1514	36	1
1514	2020	1
1514	391	1
1514	105	1
1514	1545	1
1514	523	1
1514	109	1
1514	46	1
1514	1103	1
1514	528	1
1514	821	1
1514	55	1
1514	572	1
1514	898	0
1514	2803	0
1514	1708	0
1514	1612	0
1514	856	0
1514	512	0
1514	2693	0
1514	2168	0
1514	2015	0
1514	1565	0
1514	3290	0
1514	2166	0

1514	1504	0
1514	1530	0
1515	34	1
1515	36	1
1515	325	1
1515	1189	1
1515	42	1
1515	362	1
1515	241	1
1515	1843	0
1515	274	0
1515	2092	0
1515	3367	0
1515	2717	0
1515	254	0
1515	1636	0
1516	3456	1
1516	3457	1
1516	3458	1
1516	3459	1
1516	36	1
1516	309	1
1516	2492	1
1516	60	1
1516	1089	1
1516	2246	1
1516	1479	1
1516	843	1
1516	845	1
1516	846	1

1516	853 1	
1516	1372	1
1516	2292	1
1516	2804	1
1516	2294	1
1516	2295	1
1516	2296	1
1516	3455	1
1516	2580	0
1516	196 0	
1516	2971	0
1516	3248	0
1516	1829	0
1516	1522	0
1516	1220	0
1516	3203	0
1516	3005	0
1516	276 0	
1516	3637	0
1516	1827	0
1516	1340	0
1516	2417	0
1516	1221	0
1516	1203	0
1516	3808	0
1516	2479	0
1516	290 0	
1516	1673	0
1516	3677	0
1516	210 0	

1517	1570	1
1517	70	1
1517	135	1
1517	72	1
1517	73	1
1517	807	1
1517	1623	1
1517	87	1
1517	601	1
1517	378	1
1517	127	1
1517	3423	0
1517	1245	0
1517	1119	0
1517	3279	0
1517	1473	0
1517	2551	0
1517	235	0
1517	3559	0
1517	2116	0
1517	1411	0
1517	2423	0
1518	35	1
1518	36	1
1518	165	1
1518	486	1
1518	2531	1
1518	3460	1
1518	3461	1
1518	2674	1

1518	20	1
1518	52	1
1518	1236	1
1518	1526	1
1518	2969	1
1518	2683	1
1518	252	1
1518	735	1
1518	549	0
1518	287	0
1518	3837	0
1518	2605	0
1518	448	0
1518	3574	0
1518	89	0
1518	158	0
1518	638	0
1518	521	0
1518	1024	0
1518	3433	0
1518	3560	0
1518	1053	0
1518	236	0
1518	489	0
1519	1986	1
1519	197	1
1519	102	1
1519	1126	1
1519	906	1
1519	875	1

1519	109	1
1519	53	1
1519	118	1
1519	438	1
1519	751	0
1519	2568	0
1519	3452	0
1519	3610	0
1519	2104	0
1519	711	0
1519	1797	0
1519	3032	0
1519	727	0
1519	1548	0
1520	608	1
1520	36	1
1520	362	1
1520	141	1
1520	1140	1
1520	2647	1
1520	568	1
1520	1704	0
1520	3325	0
1520	2232	0
1520	1384	0
1520	1968	0
1520	3762	0
1520	49	0
1521	96	1
1521	834	1

1521	3462	1
1521	75	1
1521	43	1
1521	493	1
1521	47	1
1521	52	1
1521	118	1
1521	126	1
1521	3468	0
1521	1470	0
1521	1023	0
1521	367	0
1521	2511	0
1521	359	0
1521	3289	0
1521	3776	0
1521	2433	0
1521	1626	0
1522	3463	1
1522	72	1
1522	237	1
1522	238	1
1522	915	1
1522	254	1
1522	799	0
1522	695	0
1522	3795	0
1522	3311	0
1522	1699	0
1522	792	0

1523	2627	1
1523	708	1
1523	1253	1
1523	392	1
1523	105	1
1523	1389	1
1523	46	1
1523	528	1
1523	55	1
1523	764	1
1523	73	0
1523	548	0
1523	657	0
1523	772	0
1523	374	0
1523	2532	0
1523	2147	0
1523	1602	0
1523	3595	0
1523	2695	0
1524	261	1
1524	357	1
1524	358	1
1524	3464	1
1524	361	1
1524	3465	1
1524	3244	1
1524	80	1
1524	1368	1
1524	1082	1

1524	1308	1
1524	253	1
1524	863	1
1524	1888	0
1524	2180	0
1524	250	0
1524	3031	0
1524	496	0
1524	2660	0
1524	813	0
1524	3651	0
1524	1772	0
1524	3300	0
1524	2922	0
1524	96	0
1524	378	0
1525	109	1
1525	46	1
1525	527	1
1525	141	1
1525	1140	1
1525	460	0
1525	796	0
1525	1574	0
1525	2286	0
1525	1009	0
1526	1091	1
1526	2597	1
1526	295	1
1526	106	1

1526	47	1
1526	696	1
1526	442	1
1526	2655	0
1526	685	0
1526	2157	0
1526	440	0
1526	3690	0
1526	78	0
1526	3269	0
1527	1347	1
1527	36	1
1527	1349	1
1527	166	1
1527	3300	1
1527	3466	1
1527	3467	1
1527	662	1
1527	1303	1
1527	541	1
1527	191	1
1527	1711	0
1527	2485	0
1527	362	0
1527	1535	0
1527	3500	0
1527	1817	0
1527	1214	0
1527	2775	0
1527	3386	0

1527	281	0
1527	2999	0
1528	961	1
1528	100	1
1528	964	1
1528	746	1
1528	3468	1
1528	438	1
1528	1046	1
1528	287	1
1528	2282	0
1528	3399	0
1528	1041	0
1528	3211	0
1528	3302	0
1528	743	0
1528	2738	0
1528	2870	0
1529	163	1
1529	1315	1
1529	2341	1
1529	230	1
1529	297	1
1529	1257	1
1529	2252	1
1529	3469	1
1529	2514	1
1529	1972	1
1529	1048	0
1529	2177	0

1529	3293	0
1529	1482	0
1529	614	0
1529	2092	0
1529	966	0
1529	2896	0
1529	2911	0
1529	818	0
1530	1664	1
1530	481	1
1530	354	1
1530	36	1
1530	1662	1
1530	109	1
1530	55	1
1530	2199	1
1530	286	1
1530	174	0
1530	736	0
1530	180	0
1530	1410	0
1530	986	0
1530	3498	0
1530	477	0
1530	1748	0
1530	2857	0
1531	1504	1
1531	131	1
1531	133	1
1531	134	1

1531	104	1
1531	137	1
1531	233	1
1531	2056	1
1531	1231	1
1531	374	1
1531	1913	0
1531	258	0
1531	2810	0
1531	1669	0
1531	1113	0
1531	2358	0
1531	3659	0
1531	2186	0
1531	1487	0
1531	2528	0
1532	96	1
1532	832	1
1532	130	1
1532	133	1
1532	72	1
1532	74	1
1532	75	1
1532	237	1
1532	115	1
1532	951	1
1532	127	1
1532	3645	0
1532	2299	0
1532	1702	0

1532	2108	0
1532	3073	0
1532	3732	0
1532	1006	0
1532	93	0
1532	2716	0
1532	2303	0
1532	2771	0
1533	133	1
1533	712	1
1533	233	1
1533	842	1
1533	110	1
1533	145	1
1533	1469	1
1533	635	0
1533	3083	0
1533	3113	0
1533	353	0
1533	2386	0
1533	2797	0
1533	1081	0
1534	2722	1
1534	1352	1
1534	617	1
1534	3470	1
1534	3471	1
1534	3409	1
1534	156	1
1534	2461	1

1534	3307	0
1534	1654	0
1534	3448	0
1534	2784	0
1534	3165	0
1534	637	0
1534	2415	0
1534	1564	0
1535	102	1
1535	1127	1
1535	136	1
1535	2512	1
1535	3472	1
1535	3473	1
1535	53	1
1535	3127	1
1535	2554	1
1535	187	1
1535	22	0
1535	899	0
1535	2588	0
1535	2454	0
1535	431	0
1535	729	0
1535	458	0
1535	1029	0
1535	2144	0
1535	3045	0
1536	2243	1
1536	1386	1

1536	3474	1
1536	3475	1
1536	2036	1
1536	3476	1
1536	1057	0
1536	833	0
1536	1228	0
1536	1267	0
1536	3812	0
1536	1216	0
1537	385	1
1537	132	1
1537	133	1
1537	74	1
1537	80	1
1537	81	1
1537	1918	1
1537	3379	1
1537	117	1
1537	3477	1
1537	3478	1
1537	254	1
1537	3392	0
1537	1274	0
1537	926	0
1537	261	0
1537	2890	0
1537	1742	0
1537	3258	0
1537	1340	0

1537	450	0
1537	3249	0
1537	2089	0
1537	1457	0
1538	196	1
1538	3480	1
1538	3174	1
1538	462	1
1538	21	1
1538	3479	1
1538	1528	1
1538	3164	0
1538	2446	0
1538	1743	0
1538	47	0
1538	1513	0
1538	2399	0
1538	3477	0
1539	196	1
1539	2884	1
1539	40	1
1539	1961	1
1539	107	1
1539	46	1
1539	530	1
1539	20	1
1539	956	1
1539	735	1
1539	2326	0
1539	360	0

1539	897	0
1539	1045	0
1539	259	0
1539	2538	0
1539	2958	0
1539	1717	0
1539	3216	0
1539	1350	0
1540	2020	1
1540	72	1
1540	137	1
1540	75	1
1540	77	1
1540	78	1
1540	79	1
1540	237	1
1540	1359	1
1540	2485	1
1540	55	1
1540	88	1
1540	538	1
1540	3180	0
1540	2254	0
1540	196	0
1540	204	0
1540	286	0
1540	735	0
1540	536	0
1540	336	0
1540	1006	0

1540	1551	0
1540	2398	0
1540	829	0
1540	1992	0
1541	128	1
1541	130	1
1541	133	1
1541	80	1
1541	87	1
1541	538	1
1541	127	1
1541	3051	0
1541	2428	0
1541	316	0
1541	2552	0
1541	223	0
1541	909	0
1541	2782	0
1542	96	1
1542	293	1
1542	295	1
1542	391	1
1542	1095	1
1542	2601	1
1542	107	1
1542	46	1
1542	878	1
1542	528	1
1542	114	1
1542	53	1

1542	55	1
1542	3481	1
1542	764	1
1542	190	1
1542	1624	0
1542	1362	0
1542	1377	0
1542	363	0
1542	1027	0
1542	1015	0
1542	11	0
1542	2035	0
1542	38	0
1542	852	0
1542	3045	0
1542	2794	0
1542	3254	0
1542	622	0
1542	210	0
1542	2737	0
1543	257	1
1543	354	1
1543	227	1
1543	487	1
1543	104	1
1543	275	1
1543	20	1
1543	883	1
1543	729	1
1543	445	1

1543	223	1
1543	2647	0
1543	501	0
1543	3709	0
1543	1905	0
1543	2626	0
1543	1786	0
1543	1730	0
1543	828	0
1543	1604	0
1543	1833	0
1543	3	0
1544	1088	1
1544	1091	1
1544	516	1
1544	3171	1
1544	109	1
1544	3352	1
1544	3482	1
1544	3483	1
1544	511	1
1544	2528	0
1544	3769	0
1544	3011	0
1544	1049	0
1544	1320	0
1544	1176	0
1544	39	0
1544	2586	0
1544	1910	0

1545	133	1
1545	134	1
1545	104	1
1545	915	1
1545	564	1
1545	22	1
1545	477	1
1545	1746	0
1545	1542	0
1545	471	0
1545	1947	0
1545	45	0
1545	3375	0
1545	2404	0
1546	608	1
1546	1672	1
1546	2031	1
1546	20	1
1546	22	1
1546	1528	1
1546	3484	1
1546	155	1
1546	2492	1
1546	479	1
1546	1481	0
1546	3495	0
1546	1899	0
1546	1632	0
1546	1169	0
1546	1031	0

1546	629	0
1546	3164	0
1546	3534	0
1546	2406	0
1547	1115	1
1547	427	1
1547	209	1
1547	242	1
1547	210	1
1547	1172	1
1547	250	1
1547	219	1
1547	1980	1
1547	3485	1
1547	1854	1
1547	543	1
1547	3603	0
1547	2399	0
1547	886	0
1547	730	0
1547	732	0
1547	357	0
1547	2689	0
1547	3059	0
1547	3306	0
1547	991	0
1547	856	0
1547	3756	0
1548	171	1
1548	239	1

1548	81	1
1548	116	1
1548	55	1
1548	87	1
1548	184	1
1548	185	1
1548	2051	0
1548	1148	0
1548	1070	0
1548	1068	0
1548	1506	0
1548	3303	0
1548	679	0
1548	2388	0
1549	385	1
1549	130	1
1549	1889	1
1549	2953	1
1549	78	1
1549	145	1
1549	276	1
1549	2071	1
1549	252	1
1549	3486	1
1549	63	1
1549	3620	0
1549	3683	0
1549	3669	0
1549	1835	0
1549	3290	0

1549	588	0
1549	2449	0
1549	1787	0
1549	1954	0
1549	2254	0
1549	1002	0
1550	1442	1
1550	35	1
1550	2340	1
1550	197	1
1550	1502	1
1550	1031	1
1550	40	1
1550	1979	1
1550	727	1
1550	653	1
1550	1327	1
1550	52	1
1550	439	1
1550	280	1
1550	443	1
1550	1309	1
1550	286	1
1550	3487	1
1550	3693	0
1550	2214	0
1550	1266	0
1550	2848	0
1550	802	0
1550	2139	0

1550	1648	0
1550	2933	0
1550	882	0
1550	1149	0
1550	1753	0
1550	2672	0
1550	2083	0
1550	1064	0
1550	373	0
1550	161	0
1550	2133	0
1550	1922	0
1551	519	1
1551	522	1
1551	155	1
1551	1308	1
1551	3488	1
1551	3489	1
1551	163	1
1551	46	1
1551	314	1
1551	957	1
1551	70	1
1551	582	1
1551	844	1
1551	1617	1
1551	854	1
1551	1114	1
1551	356	1
1551	361	1

1551	875 1	
1551	763 1	
1551	3282	0
1551	2659	0
1551	1244	0
1551	1689	0
1551	3267	0
1551	2002	0
1551	3661	0
1551	3073	0
1551	433 0	
1551	2455	0
1551	1611	0
1551	1514	0
1551	2145	0
1551	2812	0
1551	781 0	
1551	2006	0
1551	2620	0
1551	1307	0
1551	2902	0
1551	2189	0
1552	128 1	
1552	389 1	
1552	199 1	
1552	136 1	
1552	105 1	
1552	361 1	
1552	107 1	
1552	776 1	

1552	1324	1
1552	46	1
1552	1583	1
1552	242	1
1552	118	1
1552	1278	1
1552	571	1
1552	764	1
1552	190	1
1552	863	1
1552	3634	0
1552	2230	0
1552	3612	0
1552	924	0
1552	993	0
1552	3124	0
1552	259	0
1552	3088	0
1552	3213	0
1552	3710	0
1552	1207	0
1552	1212	0
1552	3624	0
1552	432	0
1552	1621	0
1552	1652	0
1552	2668	0
1552	3733	0
1553	1409	1
1553	2147	1

1553	1413	1
1553	105	1
1553	137	1
1553	171	1
1553	364	1
1553	79	1
1553	698	1
1553	863	1
1553	3687	0
1553	1663	0
1553	529	0
1553	1144	0
1553	3845	0
1553	1381	0
1553	2159	0
1553	3162	0
1553	2023	0
1553	1157	0
1554	776	1
1554	398	1
1554	1297	1
1554	802	1
1554	3490	1
1554	804	1
1554	302	1
1554	47	1
1554	303	1
1554	199	1
1554	713	1
1554	714	1

1554	589	1
1554	208	1
1554	210	1
1554	1503	1
1554	1119	1
1554	3188	1
1554	3189	1
1554	3580	0
1554	2615	0
1554	2692	0
1554	2557	0
1554	1334	0
1554	615	0
1554	2922	0
1554	70	0
1554	101	0
1554	1513	0
1554	1986	0
1554	728	0
1554	1016	0
1554	1268	0
1554	838	0
1554	257	0
1554	202	0
1554	1310	0
1554	3652	0
1555	2890	1
1555	1101	1
1555	1887	0
1555	707	0

1556	1122	1
1556	301	1
1556	45	1
1556	212	1
1556	54	1
1556	55	1
1556	2015	1
1556	2013	1
1556	703	1
1556	1192	0
1556	339	0
1556	1109	0
1556	1789	0
1556	720	0
1556	1343	0
1556	3378	0
1556	197	0
1556	798	0
1557	354	1
1557	523	1
1557	46	1
1557	2290	1
1557	52	1
1557	22	1
1557	55	1
1557	957	1
1557	190	1
1557	780	0
1557	3061	0
1557	945	0

1557	248	0
1557	2069	0
1557	1395	0
1557	3553	0
1557	2311	0
1557	2174	0
1558	449	1
1558	418	1
1558	707	1
1558	164	1
1558	676	1
1558	2829	1
1558	2030	1
1558	2299	1
1558	465	1
1558	57	1
1558	2330	1
1558	155	1
1558	158	1
1558	959	1
1558	3565	0
1558	3520	0
1558	1342	0
1558	1620	0
1558	3811	0
1558	791	0
1558	3529	0
1558	1239	0
1558	333	0
1558	2461	0

1558	2137	0
1558	1238	0
1558	2619	0
1558	1442	0
1559	96	1
1559	801	1
1559	1760	1
1559	3491	1
1559	137	1
1559	841	1
1559	1452	1
1559	301	1
1559	3217	1
1559	184	1
1559	185	1
1559	126	1
1559	255	1
1559	1093	0
1559	2279	0
1559	238	0
1559	1190	0
1559	1379	0
1559	2701	0
1559	612	0
1559	2040	0
1559	2537	0
1559	1796	0
1559	838	0
1559	1546	0
1559	3113	0

1560	804	1
1560	932	1
1560	902	1
1560	3492	1
1560	873	1
1560	2058	1
1560	235	1
1560	941	1
1560	945	1
1560	210	1
1560	914	1
1560	52	1
1560	1168	0
1560	1741	0
1560	2936	0
1560	1098	0
1560	2818	0
1560	3392	0
1560	3811	0
1560	755	0
1560	2479	0
1560	3236	0
1560	301	0
1560	2610	0
1561	704	1
1561	289	1
1561	1216	1
1561	35	1
1561	165	1
1561	2694	1

1561	3494	1
1561	1514	1
1561	752	1
1561	2012	1
1561	713	0
1561	1126	0
1561	1076	0
1561	3827	0
1561	1609	0
1561	1124	0
1561	758	0
1561	3787	0
1561	2186	0
1561	378	0
1562	131	1
1562	2501	1
1562	1448	1
1562	239	1
1562	433	1
1562	821	1
1562	22	1
1562	472	1
1562	442	1
1562	187	1
1562	1278	1
1562	3637	0
1562	3625	0
1562	247	0
1562	2759	0
1562	1816	0

1562	560	0
1562	950	0
1562	2637	0
1562	148	0
1562	3744	0
1562	3067	0
1563	96	1
1563	1889	1
1563	98	1
1563	130	1
1563	261	1
1563	135	1
1563	74	1
1563	267	1
1563	1258	1
1563	80	1
1563	81	1
1563	919	1
1563	117	1
1563	118	1
1563	55	1
1563	1351	0
1563	3003	0
1563	3742	0
1563	3114	0
1563	2328	0
1563	1298	0
1563	3076	0
1563	188	0
1563	1318	0

1563	2017	0
1563	2874	0
1563	1090	0
1563	549	0
1563	1230	0
1563	2850	0
1564	1342	1
1564	36	1
1564	325	1
1564	3495	1
1564	1225	1
1564	42	1
1564	211	1
1564	632	1
1564	2009	1
1564	442	1
1564	286	1
1564	1971	0
1564	3262	0
1564	3701	0
1564	446	0
1564	3593	0
1564	820	0
1564	21	0
1564	2803	0
1564	754	0
1564	3000	0
1564	1820	0
1565	512	1
1565	615	1

1565	519	1
1565	617	1
1565	3496	1
1565	395	1
1565	3497	1
1565	2415	1
1565	3375	1
1565	1010	1
1565	1586	1
1565	3220	1
1565	1079	1
1565	2365	1
1565	541	1
1565	2423	1
1565	2424	0
1565	433	0
1565	1801	0
1565	1287	0
1565	3716	0
1565	2782	0
1565	1177	0
1565	398	0
1565	1418	0
1565	3762	0
1565	3014	0
1565	1167	0
1565	2552	0
1565	1683	0
1565	995	0
1565	840	0

1566	96	1
1566	131	1
1566	1414	1
1566	72	1
1566	3241	1
1566	1258	1
1566	1324	1
1566	80	1
1566	1200	1
1566	1364	1
1566	694	1
1566	87	1
1566	127	1
1566	2925	0
1566	3050	0
1566	2814	0
1566	279	0
1566	3409	0
1566	387	0
1566	1482	0
1566	805	0
1566	1029	0
1566	3612	0
1566	3417	0
1566	370	0
1566	3328	0
1567	96	1
1567	807	1
1567	776	1
1567	3498	1

1567	1624	1
1567	2289	1
1567	119	1
1567	120	1
1567	601	1
1567	1307	1
1567	124	1
1567	125	1
1567	735	1
1567	2530	0
1567	3310	0
1567	2586	0
1567	2322	0
1567	752	0
1567	3645	0
1567	1287	0
1567	398	0
1567	2310	0
1567	3471	0
1567	2268	0
1567	2830	0
1567	1061	0
1568	96	1
1568	291	1
1568	36	1
1568	199	1
1568	361	1
1568	74	1
1568	3499	1
1568	941	1

1568	1969	1
1568	210 1	
1568	242 1	
1568	2554	1
1568	277 1	
1568	1078	1
1568	26 1	
1568	2663	0
1568	820 0	
1568	3841	0
1568	2066	0
1568	2133	0
1568	3806	0
1568	1611	0
1568	2155	0
1568	684 0	
1568	1156	0
1568	810 0	
1568	2649	0
1568	2272	0
1568	382 0	
1568	774 0	
1569	2786	1
1569	492 1	
1569	3501	1
1569	3500	1
1569	3502	1
1569	2360	1
1569	1879	0
1569	399 0	

1569	720	0
1569	3463	0
1569	2858	0
1569	2673	0
1570	1345	1
1570	131	1
1570	41	1
1570	105	1
1570	46	1
1570	78	1
1570	366	1
1570	527	1
1570	87	1
1570	3742	0
1570	3426	0
1570	1679	0
1570	212	0
1570	988	0
1570	220	0
1570	2406	0
1570	2466	0
1570	1826	0
1571	128	1
1571	513	1
1571	104	1
1571	297	1
1571	2058	1
1571	108	1
1571	111	1
1571	1712	1

1571	1009	1
1571	3186	1
1571	3503	1
1571	2644	1
1571	56	1
1571	154	1
1571	187	1
1571	159	1
1571	74	0
1571	1319	0
1571	2602	0
1571	2066	0
1571	186	0
1571	1843	0
1571	1084	0
1571	3778	0
1571	211	0
1571	543	0
1571	544	0
1571	2468	0
1571	1325	0
1571	625	0
1571	1275	0
1571	2867	0
1572	96	1
1572	770	1
1572	708	1
1572	295	1
1572	137	1
1572	361	1

1572	107	1
1572	237	1
1572	55	1
1572	187	1
1572	572	1
1572	1388	0
1572	2024	0
1572	2307	0
1572	635	0
1572	405	0
1572	1877	0
1572	2018	0
1572	2339	0
1572	30	0
1572	1045	0
1572	1408	0
1573	230	1
1573	2054	1
1573	74	1
1573	75	1
1573	237	1
1573	80	1
1573	373	1
1573	87	1
1573	184	1
1573	1020	1
1573	127	1
1573	3343	0
1573	3037	0
1573	3430	0

1573	461	0
1573	2064	0
1573	1814	0
1573	2936	0
1573	1568	0
1573	2197	0
1573	758	0
1573	1703	0
1574	134	1
1574	171	1
1574	364	1
1574	78	1
1574	46	1
1574	80	1
1574	79	1
1574	206	1
1574	3504	1
1574	118	1
1574	374	1
1574	863	1
1574	133	0
1574	1253	0
1574	2642	0
1574	3805	0
1574	3836	0
1574	3449	0
1574	1011	0
1574	2420	0
1574	1351	0
1574	561	0

1574	1289	0
1574	2776	0
1575	96	1
1575	131	1
1575	102	1
1575	105	1
1575	171	1
1575	267	1
1575	1324	1
1575	47	1
1575	3505	1
1575	3506	1
1575	87	1
1575	2269	1
1575	254	1
1575	863	1
1575	1827	0
1575	1158	0
1575	3702	0
1575	1199	0
1575	3038	0
1575	2959	0
1575	3755	0
1575	2255	0
1575	2523	0
1575	1896	0
1575	3159	0
1575	3298	0
1575	3398	0
1575	935	0

1576	291	1
1576	1030	1
1576	1321	1
1576	426	1
1576	1449	1
1576	2089	1
1576	912	1
1576	178	1
1576	3507	1
1576	3508	1
1576	795	1
1576	157	1
1576	1455	0
1576	1024	0
1576	1632	0
1576	3258	0
1576	1419	0
1576	3582	0
1576	3361	0
1576	623	0
1576	3183	0
1576	1244	0
1576	2347	0
1576	338	0
1577	128	1
1577	1153	1
1577	133	1
1577	102	1
1577	115	1
1577	3509	1

1577	87	1
1577	3094	0
1577	2174	0
1577	1712	0
1577	2811	0
1577	2807	0
1577	3259	0
1577	1683	0
1578	2593	1
1578	738	1
1578	102	1
1578	2184	1
1578	105	1
1578	681	1
1578	235	1
1578	523	1
1578	715	1
1578	114	1
1578	52	1
1578	1079	1
1578	55	1
1578	1342	1
1578	935	0
1578	2569	0
1578	3373	0
1578	1356	0
1578	3370	0
1578	714	0
1578	2055	0
1578	502	0

1578	2512	0
1578	3610	0
1578	147	0
1578	1725	0
1578	2102	0
1578	1430	0
1579	80	1
1579	74	1
1579	133	1
1579	819	0
1579	2692	0
1579	262	0
1580	224	1
1580	164	1
1580	4	1
1580	871	1
1580	456	1
1580	2858	1
1580	427	1
1580	971	1
1580	1262	1
1580	47	1
1580	305	1
1580	242	1
1580	337	1
1580	3510	1
1580	280	1
1580	155	1
1580	2538	0
1580	1911	0

1580	1799	0
1580	145 0	
1580	3537	0
1580	2368	0
1580	604 0	
1580	2712	0
1580	161 0	
1580	3717	0
1580	1127	0
1580	1446	0
1580	806 0	
1580	1400	0
1580	3639	0
1580	366 0	
1581	96 1	
1581	257 1	
1581	1058	1
1581	650 1	
1581	271 1	
1581	1457	1
1581	754 1	
1581	118 1	
1581	505 1	
1581	1052	1
1581	1053	1
1581	2302	1
1581	1055	1
1581	3757	0
1581	3349	0
1581	1298	0

1581	658	0
1581	2964	0
1581	2095	0
1581	62	0
1581	1877	0
1581	836	0
1581	1158	0
1581	3124	0
1581	1069	0
1581	289	0
1582	96	1
1582	131	1
1582	808	1
1582	137	1
1582	1258	1
1582	267	1
1582	363	1
1582	77	1
1582	206	1
1582	79	1
1582	433	1
1582	185	1
1582	538	1
1582	923	1
1582	863	1
1582	940	0
1582	2074	0
1582	2013	0
1582	591	0
1582	621	0

1582	2348	0
1582	2007	0
1582	2302	0
1582	2252	0
1582	1010	0
1582	1234	0
1582	3076	0
1582	2390	0
1582	1711	0
1582	2039	0
1583	265	1
1583	2950	1
1583	382	1
1583	2302	1
1583	1935	0
1583	1714	0
1583	1817	0
1583	171	0
1584	96	1
1584	34	1
1584	37	1
1584	293	1
1584	391	1
1584	392	1
1584	105	1
1584	107	1
1584	46	1
1584	2161	1
1584	52	1
1584	1303	1

1584	57	1
1584	764	1
1584	221	1
1584	190	1
1584	1581	0
1584	1847	0
1584	816	0
1584	1023	0
1584	2179	0
1584	880	0
1584	2697	0
1584	1538	0
1584	1854	0
1584	3607	0
1584	1645	0
1584	446	0
1584	2789	0
1584	1403	0
1584	1492	0
1584	421	0
1585	704	1
1585	289	1
1585	928	1
1585	1216	1
1585	100	1
1585	197	1
1585	3494	1
1585	1289	1
1585	945	1
1585	3511	1

1585	3512	1
1585	3513	1
1585	2522	1
1585	1022	1
1585	2744	0
1585	3393	0
1585	1582	0
1585	3066	0
1585	438	0
1585	2567	0
1585	1835	0
1585	1487	0
1585	969	0
1585	2021	0
1585	1226	0
1585	753	0
1585	2421	0
1585	2612	0
1586	131	1
1586	135	1
1586	77	1
1586	206	1
1586	79	1
1586	919	1
1586	117	1
1586	373	1
1586	87	1
1586	184	1
1586	185	1
1586	645	0

1586	3331	0
1586	3121	0
1586	1818	0
1586	2577	0
1586	1960	0
1586	575	0
1586	629	0
1586	2167	0
1586	2490	0
1586	724	0
1587	774	1
1587	2890	1
1587	715	1
1587	2123	1
1587	2194	1
1587	147	1
1587	1140	1
1587	52	1
1587	3514	1
1587	3515	1
1587	3741	0
1587	3119	0
1587	2160	0
1587	1256	0
1587	1785	0
1587	643	0
1587	2403	0
1587	3578	0
1587	2241	0
1587	2504	0

1588	1216	1
1588	129	1
1588	132	1
1588	325	1
1588	453	1
1588	7	1
1588	1029	1
1588	2887	1
1588	1388	1
1588	272	1
1588	209	1
1588	735	1
1588	1073	1
1588	507	1
1588	3516	1
1588	957	1
1588	2175	1
1588	63	1
1588	3636	0
1588	1128	0
1588	582	0
1588	3553	0
1588	3797	0
1588	2306	0
1588	356	0
1588	3102	0
1588	3566	0
1588	874	0
1588	3335	0
1588	3687	0

1588	1926	0
1588	726	0
1588	3127	0
1588	2621	0
1588	3227	0
1588	3603	0
1589	36	1
1589	105	1
1589	46	1
1589	114	1
1589	243	1
1589	52	1
1589	55	1
1589	188	1
1589	599	0
1589	1182	0
1589	2791	0
1589	360	0
1589	1073	0
1589	1125	0
1589	447	0
1589	1931	0
1590	133	1
1590	105	1
1590	1259	1
1590	78	1
1590	46	1
1590	305	1
1590	1589	1
1590	1016	1

1590	282	1
1590	61	1
1590	2483	0
1590	3384	0
1590	206	0
1590	2178	0
1590	1159	0
1590	2327	0
1590	2345	0
1590	2823	0
1590	2647	0
1590	1001	0
1591	903	1
1591	21	1
1591	24	1
1591	155	1
1591	157	1
1591	419	1
1591	36	1
1591	46	1
1591	55	1
1591	57	1
1591	187	1
1591	572	1
1591	3517	1
1591	3518	1
1591	457	1
1591	221	1
1591	1374	1
1591	483	1

1591	105	1
1591	372	0
1591	2726	0
1591	1337	0
1591	1414	0
1591	3533	0
1591	662	0
1591	1688	0
1591	738	0
1591	1867	0
1591	739	0
1591	684	0
1591	1660	0
1591	675	0
1591	3305	0
1591	1538	0
1591	1948	0
1591	3093	0
1591	1171	0
1591	821	0
1592	554	1
1592	77	1
1592	2064	1
1592	923	1
1592	3519	1
1592	3680	0
1592	1131	0
1592	672	0
1592	1851	0
1592	522	0

1593	3520	1
1593	109	1
1593	1103	1
1593	688	1
1593	306	1
1593	882	1
1593	52	1
1593	222	1
1593	3496	0
1593	2634	0
1593	585	0
1593	1517	0
1593	2258	0
1593	2157	0
1593	1935	0
1593	263	0
1594	1463	1
1594	1186	1
1594	171	1
1594	267	1
1594	363	1
1594	79	1
1594	560	1
1594	118	1
1594	87	1
1594	184	1
1594	185	1
1594	538	1
1594	254	1
1594	1218	0

1594	2827	0
1594	1835	0
1594	577 0	
1594	3658	0
1594	3414	0
1594	2496	0
1594	50 0	
1594	2590	0
1594	2073	0
1594	3637	0
1594	696 0	
1594	1537	0
1595	2048	1
1595	3521	1
1595	3522	1
1595	1859	1
1595	3523	1
1595	3524	1
1595	614 1	
1595	1543	1
1595	2023	1
1595	1545	1
1595	878 1	
1595	2290	1
1595	799 1	
1595	373 0	
1595	3079	0
1595	2359	0
1595	1426	0
1595	2223	0

1595	1934	0
1595	365	0
1595	338	0
1595	839	0
1595	232	0
1595	2078	0
1595	2413	0
1595	2727	0
1596	223	1
1596	3525	1
1596	3526	1
1596	3527	1
1596	105	1
1596	235	1
1596	45	1
1596	110	1
1596	1469	1
1596	1526	1
1596	154	1
1596	1181	1
1596	159	1
1596	1488	0
1596	2229	0
1596	959	0
1596	1692	0
1596	878	0
1596	2719	0
1596	307	0
1596	2846	0
1596	3710	0

1596	3390	0
1596	1432	0
1596	18	0
1596	3706	0
1597	66	1
1597	674	1
1597	2566	1
1597	232	1
1597	73	1
1597	74	1
1597	75	1
1597	399	1
1597	81	1
1597	951	1
1597	1202	1
1597	87	1
1597	377	1
1597	58	1
1597	127	1
1597	695	0
1597	659	0
1597	2951	0
1597	2697	0
1597	3272	0
1597	706	0
1597	1220	0
1597	3113	0
1597	642	0
1597	76	0
1597	1407	0

1597	973	0
1597	2254	0
1597	135	0
1597	977	0
1598	1140	1
1598	147	1
1598	36	1
1598	115	0
1598	1637	0
1598	2026	0
1599	899	1
1599	1955	1
1599	1956	1
1599	3428	1
1599	807	1
1599	136	1
1599	3528	1
1599	1322	1
1599	3529	1
1599	3244	1
1599	590	1
1599	366	1
1599	1487	1
1599	254	1
1599	3647	0
1599	2582	0
1599	2812	0
1599	2506	0
1599	660	0
1599	2922	0

1599	1832	0
1599	1531	0
1599	3132	0
1599	93	0
1599	1285	0
1599	2028	0
1599	3346	0
1599	1343	0
1600	96	1
1600	2116	1
1600	1126	1
1600	3338	1
1600	3530	1
1600	3531	1
1600	653	1
1600	3532	1
1600	2319	1
1600	3533	1
1600	3534	1
1600	916	1
1600	3481	1
1600	21	0
1600	314	0
1600	1511	0
1600	109	0
1600	2413	0
1600	272	0
1600	1368	0
1600	2618	0
1600	2445	0

1600	3817	0
1600	3148	0
1600	1526	0
1600	3355	0
1601	96	1
1601	66	1
1601	131	1
1601	72	1
1601	137	1
1601	74	1
1601	237	1
1601	1202	1
1601	117	1
1601	185	1
1601	788	0
1601	2947	0
1601	1854	0
1601	3716	0
1601	3566	0
1601	1024	0
1601	2240	0
1601	3	0
1601	3162	0
1601	3227	0
1602	1092	1
1602	295	1
1602	266	1
1602	78	1
1602	46	1
1602	47	1

1602	3535	1
1602	1589	1
1602	55	1
1602	3805	0
1602	52	0
1602	2245	0
1602	2425	0
1602	1882	0
1602	3744	0
1602	3601	0
1602	1935	0
1602	3581	0
1603	96	1
1603	36	1
1603	528	1
1603	118	1
1603	55	1
1603	187	1
1603	190	1
1603	159	1
1603	10	0
1603	426	0
1603	1138	0
1603	666	0
1603	1089	0
1603	903	0
1603	1040	0
1603	2708	0
1604	96	1
1604	385	1

1604	36	1
1604	293	1
1604	356	1
1604	388	1
1604	1318	1
1604	957	1
1604	499	1
1604	729	1
1604	245	1
1604	55	1
1604	57	1
1604	474	1
1604	445	1
1604	1546	0
1604	3454	0
1604	1151	0
1604	2080	0
1604	393	0
1604	2597	0
1604	3421	0
1604	3541	0
1604	2515	0
1604	2171	0
1604	3397	0
1604	1727	0
1604	3052	0
1604	2208	0
1604	3373	0
1605	130	1
1605	73	1

1605	1612	1
1605	77	1
1605	80	1
1605	117	1
1605	87	1
1605	58	1
1605	824	0
1605	867	0
1605	3539	0
1605	3832	0
1605	714	0
1605	697	0
1605	2090	0
1605	2043	0
1606	131	1
1606	358	1
1606	1607	1
1606	1324	1
1606	1678	1
1606	3536	1
1606	87	1
1606	253	1
1606	1082	1
1606	1083	1
1606	445	1
1606	1310	1
1606	2471	0
1606	1256	0
1606	417	0
1606	2109	0

1606	3191	0
1606	3655	0
1606	323 0	
1606	1557	0
1606	1943	0
1606	1316	0
1606	1261	0
1606	1938	0
1607	483 1	
1607	517 1	
1607	486 1	
1607	1005	1
1607	1136	1
1607	1585	1
1607	3537	1
1607	3538	1
1607	3539	1
1607	3540	1
1607	2454	1
1607	727 1	
1607	728 1	
1607	3541	1
1607	155 1	
1607	732 1	
1607	509 1	
1607	3766	0
1607	3708	0
1607	956 0	
1607	2694	0
1607	3784	0

1607	103	0
1607	445	0
1607	2147	0
1607	3438	0
1607	2452	0
1607	1647	0
1607	1158	0
1607	1634	0
1607	3676	0
1607	3115	0
1607	1945	0
1607	644	0
1608	102	1
1608	906	1
1608	715	1
1608	878	1
1608	1395	1
1608	52	1
1608	1556	1
1608	282	1
1608	159	1
1608	1130	0
1608	2527	0
1608	1191	0
1608	3820	0
1608	3699	0
1608	316	0
1608	1813	0
1608	2276	0
1608	815	0

1609	674	1
1609	1187	1
1609	3543	1
1609	3544	1
1609	203	1
1609	3545	1
1609	919	1
1609	3546	1
1609	3542	1
1609	183	1
1609	184	1
1609	185	1
1609	2106	1
1609	1659	1
1609	540	1
1609	3547	1
1609	862	1
1609	1844	0
1609	2052	0
1609	3042	0
1609	3339	0
1609	3288	0
1609	894	0
1609	2585	0
1609	949	0
1609	658	0
1609	3316	0
1609	719	0
1609	260	0
1609	997	0

1609	3250	0
1609	2493	0
1609	1078	0
1609	1524	0
1610	96	1
1610	102	1
1610	105	1
1610	46	1
1610	47	1
1610	560	1
1610	1523	1
1610	52	1
1610	118	1
1610	55	1
1610	863	1
1610	729	0
1610	3467	0
1610	1888	0
1610	2799	0
1610	1189	0
1610	3151	0
1610	2109	0
1610	2144	0
1610	2617	0
1610	1576	0
1610	2681	0
1611	36	1
1611	613	1
1611	45	1
1611	109	1

1611	47	1
1611	141	1
1611	53	1
1611	56	1
1611	57	1
1611	3548	1
1611	1437	1
1611	223	1
1611	1850	0
1611	990	0
1611	3133	0
1611	456	0
1611	1019	0
1611	623	0
1611	675	0
1611	3539	0
1611	3500	0
1611	1134	0
1611	64	0
1611	54	0
1612	128	1
1612	1923	1
1612	37	1
1612	453	1
1612	136	1
1612	235	1
1612	462	1
1612	1555	1
1612	118	1
1612	1528	1

1612	57	1
1612	541	1
1612	159	1
1612	1433	0
1612	3727	0
1612	652	0
1612	2191	0
1612	527	0
1612	2576	0
1612	1929	0
1612	1016	0
1612	1170	0
1612	23	0
1612	3143	0
1612	2127	0
1612	998	0
1613	708	1
1613	613	1
1613	165	1
1613	3036	1
1613	1502	1
1613	2948	0
1613	742	0
1613	3541	0
1613	250	0
1613	2845	0
1614	96	1
1614	133	1
1614	135	1
1614	75	1

1614	79	1
1614	80	1
1614	115	1
1614	87	1
1614	377	1
1614	1995	0
1614	707	0
1614	1489	0
1614	2499	0
1614	523	0
1614	3266	0
1614	141	0
1614	1832	0
1614	3819	0
1615	96	1
1615	325	1
1615	102	1
1615	427	1
1615	243	1
1615	244	1
1615	119	1
1615	247	1
1615	124	1
1615	126	1
1615	2508	0
1615	2893	0
1615	344	0
1615	3084	0
1615	118	0
1615	2524	0

1615	1554	0
1615	253	0
1615	3557	0
1615	3026	0
1616	372	1
1616	2214	1
1616	548	1
1616	87	1
1616	1231	0
1616	3693	0
1616	3060	0
1616	1546	0
1617	3550	1
1617	134	1
1617	135	1
1617	171	1
1617	81	1
1617	1233	1
1617	87	1
1617	88	1
1617	3549	1
1617	254	1
1617	3218	0
1617	1547	0
1617	722	0
1617	137	0
1617	1757	0
1617	1331	0
1617	190	0
1617	578	0

1617	3258	0
1617	1037	0
1618	98	1
1618	131	1
1618	376	1
1618	135	1
1618	137	1
1618	1325	1
1618	81	1
1618	723	1
1618	87	1
1618	184	1
1618	185	1
1618	698	1
1618	3519	0
1618	624	0
1618	3121	0
1618	2118	0
1618	3300	0
1618	3595	0
1618	1754	0
1618	3668	0
1618	1908	0
1618	793	0
1618	146	0
1618	2638	0
1619	3072	1
1619	1253	1
1619	391	1
1619	295	1

1619	105	1
1619	392	1
1619	46	1
1619	755	1
1619	190	1
1619	799	1
1619	2875	0
1619	970	0
1619	319	0
1619	2808	0
1619	644	0
1619	652	0
1619	1841	0
1619	404	0
1619	2967	0
1619	536	0
1620	2048	1
1620	3552	1
1620	66	1
1620	354	1
1620	36	1
1620	54	1
1620	3745	0
1620	1364	0
1620	3801	0
1620	1058	0
1620	3714	0
1620	3047	0
1621	261	1
1621	946	1

1621	52	1
1621	1595	1
1621	2643	1
1621	1240	1
1621	3553	1
1621	482	1
1621	3554	1
1621	356	1
1621	3555	1
1621	998	1
1621	107	1
1621	1131	1
1621	113	1
1621	2292	1
1621	1397	1
1621	1526	1
1621	378	1
1621	124	1
1621	2485	0
1621	1548	0
1621	3458	0
1621	2013	0
1621	1426	0
1621	1026	0
1621	1381	0
1621	20	0
1621	497	0
1621	2832	0
1621	1214	0
1621	1332	0

1621	680	0
1621	1827	0
1621	942	0
1621	2839	0
1621	2438	0
1621	1755	0
1621	2191	0
1621	1561	0
1622	96	1
1622	3556	1
1622	3557	1
1622	3558	1
1622	472	1
1622	40	1
1622	104	1
1622	3371	1
1622	957	1
1622	433	1
1622	2835	1
1622	276	1
1622	1044	1
1622	24	1
1622	413	1
1622	3635	0
1622	1195	0
1622	853	0
1622	968	0
1622	1928	0
1622	2867	0
1622	1809	0

1622	587	0
1622	1024	0
1622	145	0
1622	2289	0
1622	2746	0
1622	1958	0
1622	1898	0
1622	2609	0
1623	26	1
1623	3559	1
1623	3560	1
1623	105	1
1623	1466	1
1623	190	1
1623	3096	0
1623	3254	0
1623	117	0
1623	3085	0
1623	2919	0
1623	845	0
1624	3561	1
1624	2285	1
1624	366	1
1624	1198	1
1624	270	1
1624	3024	1
1624	2806	1
1624	2488	1
1624	2947	0
1624	3220	0

1624	2978	0
1624	564	0
1624	3466	0
1624	62	0
1624	2919	0
1624	3333	0
1625	80	1
1625	130	1
1625	78	1
1625	919	1
1625	3198	0
1625	2752	0
1625	103	0
1625	2527	0
1626	130	1
1626	131	1
1626	132	1
1626	78	1
1626	79	1
1626	80	1
1626	372	1
1626	185	1
1626	538	1
1626	254	1
1626	962	0
1626	716	0
1626	1010	0
1626	2168	0
1626	776	0
1626	2917	0

1626	2259	0
1626	1583	0
1626	3726	0
1626	1264	0
1627	1184	1
1627	3493	1
1627	1127	1
1627	392 1	
1627	3562	1
1627	3563	1
1627	3564	1
1627	3565	1
1627	3566	1
1627	1135	1
1627	944 1	
1627	2959	1
1627	2960	1
1627	3567	1
1627	3568	1
1627	222 1	
1627	2627	0
1627	1924	0
1627	2016	0
1627	1219	0
1627	2915	0
1627	336 0	
1627	1095	0
1627	1140	0
1627	2513	0
1627	1846	0

1627	182	0
1627	1997	0
1627	1113	0
1627	1008	0
1627	1759	0
1627	1162	0
1628	96	1
1628	1122	1
1628	2074	1
1628	134	1
1628	3569	1
1628	2162	1
1628	1523	1
1628	1754	1
1628	3764	0
1628	385	0
1628	417	0
1628	1528	0
1628	187	0
1628	1319	0
1628	1298	0
1628	545	0
1629	1218	1
1629	392	1
1629	105	1
1629	878	1
1629	1103	1
1629	3441	1
1629	3570	1
1629	1140	1

1629	53	1
1629	1078	1
1629	1556	1
1629	1752	1
1629	154	1
1629	891	1
1629	2815	1
1629	70	0
1629	3706	0
1629	2753	0
1629	2832	0
1629	1523	0
1629	2788	0
1629	2688	0
1629	2492	0
1629	1258	0
1629	2394	0
1629	1351	0
1629	746	0
1629	709	0
1629	2424	0
1629	2777	0
1630	130	1
1630	102	1
1630	871	1
1630	902	1
1630	427	1
1630	941	1
1630	302	1
1630	303	1

1630	657	1
1630	210	1
1630	216	1
1630	377	1
1630	2554	1
1630	3188	0
1630	3177	0
1630	982	0
1630	3695	0
1630	2880	0
1630	1540	0
1630	336	0
1630	3356	0
1630	1852	0
1630	445	0
1630	2832	0
1630	869	0
1630	3373	0
1631	134	1
1631	329	1
1631	528	1
1631	114	1
1631	1397	1
1631	118	1
1631	55	1
1631	863	1
1631	858	1
1631	1020	1
1631	861	1
1631	126	1

1631	127	1
1631	2642	0
1631	919	0
1631	1901	0
1631	193	0
1631	2496	0
1631	3589	0
1631	242	0
1631	3062	0
1631	3142	0
1631	261	0
1631	1250	0
1631	2554	0
1631	241	0
1632	320	1
1632	3575	1
1632	4	1
1632	5	1
1632	678	1
1632	1604	1
1632	1608	1
1632	971	1
1632	1740	1
1632	333	1
1632	307	1
1632	3571	1
1632	3572	1
1632	3573	1
1632	1271	1
1632	1272	1

1632	3574	1
1632	314	1
1632	553	0
1632	1194	0
1632	871	0
1632	3032	0
1632	2453	0
1632	3668	0
1632	2164	0
1632	710	0
1632	181	0
1632	3039	0
1632	1033	0
1632	1055	0
1632	918	0
1632	453	0
1632	3507	0
1632	1501	0
1632	1954	0
1632	10	0
1633	133	1
1633	361	1
1633	74	1
1633	75	1
1633	79	1
1633	80	1
1633	87	1
1633	127	1
1633	444	0
1633	1712	0

1633	743	0
1633	1295	0
1633	215	0
1633	1493	0
1633	3806	0
1633	541	0
1634	96	1
1634	131	1
1634	134	1
1634	104	1
1634	267	1
1634	47	1
1634	433	1
1634	118	1
1634	185	1
1634	413	1
1634	2806	0
1634	310	0
1634	2176	0
1634	1002	0
1634	138	0
1634	8	0
1634	3005	0
1634	1798	0
1634	1869	0
1634	3803	0
1635	832	1
1635	2434	1
1635	133	1
1635	1255	1

1635	105	1
1635	109	1
1635	46	1
1635	52	1
1635	55	1
1635	91	1
1635	126	0
1635	2553	0
1635	758	0
1635	1805	0
1635	1875	0
1635	3403	0
1635	2779	0
1635	135	0
1635	3792	0
1635	755	0
1636	163	1
1636	1253	1
1636	1797	1
1636	391	1
1636	1255	1
1636	105	1
1636	235	1
1636	109	1
1636	46	1
1636	55	1
1636	863	1
1636	1370	0
1636	521	0
1636	1417	0

1636	2402	0
1636	2953	0
1636	1524	0
1636	1343	0
1636	694	0
1636	2176	0
1636	2331	0
1636	1703	0
1637	223	1
1637	36	1
1637	104	1
1637	105	1
1637	235	1
1637	46	1
1637	110	1
1637	52	1
1637	53	1
1637	55	1
1637	154	1
1637	382	1
1637	159	1
1637	1860	0
1637	2359	0
1637	3695	0
1637	189	0
1637	3621	0
1637	2937	0
1637	1886	0
1637	542	0
1637	561	0

1637	426	0
1637	3540	0
1637	1985	0
1637	1048	0
1638	1890	1
1638	135	1
1638	73	1
1638	41	1
1638	75	1
1638	366	1
1638	80	1
1638	371	1
1638	373	1
1638	1141	1
1638	87	1
1638	3576	1
1638	1788	1
1638	127	1
1638	843	0
1638	893	0
1638	2393	0
1638	2047	0
1638	1781	0
1638	2755	0
1638	1618	0
1638	341	0
1638	272	0
1638	461	0
1638	1651	0
1638	762	0

1638	3839	0
1638	3023	0
1639	1697	1
1639	74	1
1639	3020	1
1639	3577	1
1639	206	1
1639	79	1
1639	270	1
1639	1806	1
1639	406	1
1639	87	1
1639	184	1
1639	185	1
1639	3578	1
1639	2717	0
1639	1124	0
1639	1026	0
1639	1852	0
1639	798	0
1639	2740	0
1639	2236	0
1639	781	0
1639	1297	0
1639	1482	0
1639	2404	0
1639	2581	0
1639	2771	0
1640	227	1
1640	293	1

1640	765	1
1640	187	1
1640	557	1
1640	500	1
1640	438	1
1640	474	1
1640	27	1
1640	445	1
1640	1387	0
1640	1817	0
1640	267	0
1640	2046	0
1640	1943	0
1640	198	0
1640	669	0
1640	2685	0
1640	1352	0
1640	1596	0
1641	1095	1
1641	712	1
1641	105	1
1641	2028	1
1641	301	1
1641	46	1
1641	2719	1
1641	305	1
1641	114	1
1641	498	1
1641	1874	1
1641	1875	1

1641	159	1
1641	2798	0
1641	1642	0
1641	1270	0
1641	2278	0
1641	2232	0
1641	2406	0
1641	2493	0
1641	3660	0
1641	1167	0
1641	3629	0
1641	3131	0
1641	2724	0
1641	670	0
1642	483	1
1642	74	1
1642	21	1
1642	22	1
1642	159	1
1642	3271	0
1642	3526	0
1642	234	0
1642	787	0
1642	2963	0
1643	228	1
1643	1732	1
1643	782	1
1643	241	1
1643	788	1
1643	1140	1

1643	3579	1
1643	250	1
1643	347	1
1643	60	1
1643	2875	0
1643	3519	0
1643	1738	0
1643	600	0
1643	1805	0
1643	2742	0
1643	2244	0
1643	3554	0
1643	2215	0
1643	3488	0
1644	832	1
1644	66	1
1644	675	1
1644	292	1
1644	3582	1
1644	1580	1
1644	1583	1
1644	3580	1
1644	442	1
1644	3452	1
1644	3581	1
1644	542	1
1644	3583	1
1644	2228	0
1644	1147	0
1644	2385	0

1644	1451	0
1644	1056	0
1644	2890	0
1644	2891	0
1644	640	0
1644	483	0
1644	2405	0
1644	3033	0
1644	728	0
1644	2941	0
1645	3584	1
1645	1699	1
1645	6	1
1645	934	1
1645	968	1
1645	10	1
1645	331	1
1645	971	1
1645	333	1
1645	334	1
1645	15	1
1645	2158	1
1645	273	1
1645	19	1
1645	2235	1
1645	2589	1
1645	1717	0
1645	2490	0
1645	3033	0
1645	3553	0

1645	3600	0
1645	844	0
1645	2476	0
1645	3412	0
1645	3138	0
1645	3418	0
1645	3372	0
1645	1909	0
1645	3046	0
1645	3363	0
1645	1387	0
1645	40	0
1646	96	1
1646	1547	1
1646	1388	1
1646	653	1
1646	756	1
1646	860	1
1646	575	1
1646	898	0
1646	1816	0
1646	392	0
1646	1849	0
1646	1075	0
1646	3459	0
1646	2516	0
1647	105	1
1647	3585	1
1647	863	1
1647	223	1

1647	867	0
1647	2321	0
1647	227	0
1647	192	0
1648	1057	1
1648	34	1
1648	98	1
1648	2522	1
1648	55	1
1648	26	1
1648	1303	1
1648	2719	1
1648	1825	0
1648	1820	0
1648	1461	0
1648	3342	0
1648	323	0
1648	1462	0
1648	3627	0
1648	2393	0
1649	608	1
1649	98	1
1649	473	1
1649	110	1
1649	78	1
1649	47	1
1649	184	1
1649	377	1
1649	1277	1
1649	191	1

1649	906 0	
1649	3600	0
1649	2321	0
1649	1979	0
1649	1735	0
1649	2240	0
1649	1176	0
1649	3529	0
1649	3394	0
1649	2314	0
1650	3586	1
1650	3587	1
1650	3588	1
1650	3589	1
1650	3590	1
1650	3591	1
1650	3592	1
1650	1558	0
1650	829 0	
1650	1224	0
1650	349 0	
1650	3657	0
1650	2777	0
1650	1601	0
1651	3168	1
1651	3170	1
1651	3173	1
1651	2984	1
1651	3593	1
1651	618 1	

1651	3594	1
1651	44	1
1651	1548	1
1651	46	1
1651	463	1
1651	3595	1
1651	3596	1
1651	3597	1
1651	51	1
1651	2326	1
1651	3673	0
1651	978	0
1651	114	0
1651	898	0
1651	1734	0
1651	1692	0
1651	2280	0
1651	1051	0
1651	2512	0
1651	130	0
1651	1515	0
1651	3434	0
1651	1595	0
1651	2631	0
1651	1081	0
1651	644	0
1652	66	1
1652	131	1
1652	132	1
1652	230	1

1652	72	1
1652	267	1
1652	237	1
1652	1549	1
1652	80	1
1652	405	1
1652	22	1
1652	2071	1
1652	3516	1
1652	539	1
1652	956	1
1652	1103	0
1652	1913	0
1652	1429	0
1652	417	0
1652	3728	0
1652	1004	0
1652	3066	0
1652	2495	0
1652	865	0
1652	145	0
1652	2068	0
1652	239	0
1652	3248	0
1652	575	0
1652	3399	0
1653	135	1
1653	1258	1
1653	171	1
1653	363	1

1653	206	1
1653	79	1
1653	3598	1
1653	434	1
1653	115	1
1653	373	1
1653	184	1
1653	185	1
1653	923	1
1653	1938	0
1653	799	0
1653	967	0
1653	547	0
1653	1132	0
1653	2611	0
1653	1144	0
1653	2818	0
1653	1510	0
1653	2034	0
1653	3026	0
1653	3025	0
1653	1718	0
1654	548	1
1654	141	1
1654	3599	1
1654	126	1
1654	127	1
1654	1313	0
1654	569	0
1654	2812	0

1654	1162	0
1654	1475	0
1655	291 1	
1655	100 1	
1655	708 1	
1655	2949	1
1655	111 1	
1655	3600	1
1655	1586	1
1655	883 1	
1655	314 1	
1655	382 1	
1655	511 1	
1655	2821	0
1655	2473	0
1655	1367	0
1655	2986	0
1655	783 0	
1655	2408	0
1655	2902	0
1655	627 0	
1655	3151	0
1655	1217	0
1655	2206	0
1656	3601	1
1656	3602	1
1656	3603	1
1656	3604	1
1656	655 0	
1656	3560	0

1656	3813	0
1656	2962	0
1657	163 1	
1657	2020	1
1657	295 1	
1657	107 1	
1657	523 1	
1657	1103	1
1657	112 1	
1657	1396	1
1657	3605	1
1657	3606	1
1657	190 1	
1657	180 0	
1657	2028	0
1657	3578	0
1657	919 0	
1657	2114	0
1657	2199	0
1657	1815	0
1657	3490	0
1657	302 0	
1657	3384	0
1657	2054	0
1658	96 1	
1658	1412	1
1658	72 1	
1658	171 1	
1658	237 1	
1658	79 1	

1658	1199	1
1658	539 1	
1658	2723	0
1658	990 0	
1658	1517	0
1658	753 0	
1658	3447	0
1658	3670	0
1658	2614	0
1658	406 0	
1659	1986	1
1659	3607	1
1659	2469	1
1659	1126	1
1659	906 1	
1659	1130	1
1659	2797	1
1659	210 1	
1659	1012	1
1659	1015	1
1659	2015	1
1659	1502	1
1659	703 1	
1659	3344	0
1659	3403	0
1659	525 0	
1659	989 0	
1659	255 0	
1659	3186	0
1659	3576	0

1659	1131	0
1659	853	0
1659	2738	0
1659	3023	0
1659	3101	0
1659	2648	0
1660	2499	1
1660	241	1
1660	242	1
1660	726	1
1660	250	1
1660	1673	0
1660	348	0
1660	1446	0
1660	1642	0
1660	3686	0
1661	3608	1
1661	3609	1
1661	715	1
1661	3791	0
1661	2050	0
1661	217	0
1662	393	1
1662	394	1
1662	241	1
1662	1490	1
1662	3610	1
1662	250	1
1662	1115	1
1662	291	0

1662	2153	0
1662	821	0
1662	3391	0
1662	3653	0
1662	355	0
1662	3731	0
1663	3426	1
1663	676	1
1663	677	1
1663	939	1
1663	940	1
1663	2287	1
1663	306	1
1663	3032	1
1663	221	1
1663	795	1
1663	3611	1
1663	541	1
1663	2410	0
1663	3012	0
1663	265	0
1663	2566	0
1663	2894	0
1663	2340	0
1663	2205	0
1663	678	0
1663	930	0
1663	1333	0
1663	1699	0
1663	1275	0

1664	2120	1
1664	523	1
1664	3084	1
1664	46	1
1664	3069	1
1664	2905	1
1664	3612	1
1664	765	1
1664	190	1
1664	1644	0
1664	126	0
1664	2674	0
1664	2558	0
1664	220	0
1664	432	0
1664	313	0
1664	3033	0
1664	74	0
1665	135	1
1665	1258	1
1665	363	1
1665	76	1
1665	206	1
1665	79	1
1665	434	1
1665	184	1
1665	185	1
1665	538	1
1665	699	1
1665	63	1

1665	127	1
1665	474	0
1665	3135	0
1665	75	0
1665	3609	0
1665	2522	0
1665	2109	0
1665	2817	0
1665	505	0
1665	3416	0
1665	1483	0
1665	612	0
1665	3337	0
1665	1069	0
1666	608	1
1666	35	1
1666	2216	1
1666	141	1
1666	462	1
1666	752	1
1666	147	1
1666	1140	1
1666	2963	1
1666	3613	1
1666	3614	1
1666	2792	0
1666	1343	0
1666	3222	0
1666	3783	0
1666	1790	0

1666	1152	0
1666	3276	0
1666	21	0
1666	2663	0
1666	3735	0
1666	2696	0
1667	1604	1
1667	1254	1
1667	871	1
1667	1606	1
1667	2567	1
1667	971	1
1667	973	1
1667	2543	1
1667	114	1
1667	1523	1
1667	119	1
1667	1016	1
1667	441	1
1667	446	1
1667	3615	1
1667	1208	0
1667	1085	0
1667	3556	0
1667	3336	0
1667	1240	0
1667	2936	0
1667	1355	0
1667	1809	0
1667	776	0

1667	952	0
1667	1789	0
1667	676	0
1667	244	0
1667	3764	0
1667	2790	0
1668	96	1
1668	3616	1
1668	3815	0
1668	2783	0
1669	3617	1
1669	3618	1
1669	3619	1
1669	1124	1
1669	3620	1
1669	3621	1
1669	3622	1
1669	210	1
1669	1372	1
1669	2196	0
1669	1877	0
1669	3824	0
1669	392	0
1669	1822	0
1669	2921	0
1669	674	0
1669	1233	0
1669	3450	0
1670	241	1
1670	36	1

1670	1257	0
1670	1547	0
1671	897	1
1671	132	1
1671	484	1
1671	2279	1
1671	3623	1
1671	630	1
1671	3198	0
1671	2484	0
1671	1924	0
1671	3821	0
1671	2975	0
1671	2197	0
1672	128	1
1672	385	1
1672	674	1
1672	135	1
1672	2311	1
1672	41	1
1672	74	1
1672	75	1
1672	3624	1
1672	45	1
1672	3625	1
1672	81	1
1672	951	1
1672	1465	1
1672	58	1
1672	127	1

1672	585	0
1672	2183	0
1672	2589	0
1672	3345	0
1672	1796	0
1672	530	0
1672	2958	0
1672	1050	0
1672	1121	0
1672	176	0
1672	3031	0
1672	2031	0
1672	3477	0
1672	1781	0
1672	1075	0
1672	2656	0
1673	1153	1
1673	2338	1
1673	772	1
1673	776	1
1673	47	1
1673	437	1
1673	118	1
1673	55	1
1673	184	1
1673	1525	1
1673	859	1
1673	1176	0
1673	2984	0
1673	842	0

1673	3362	0
1673	1697	0
1673	1197	0
1673	2077	0
1673	2458	0
1673	2130	0
1673	1609	0
1673	2695	0
1674	96	1
1674	133	1
1674	357	1
1674	135	1
1674	74	1
1674	77	1
1674	87	1
1674	120	1
1674	127	1
1674	920	0
1674	2526	0
1674	3618	0
1674	1	0
1674	1176	0
1674	1075	0
1674	3124	0
1674	2803	0
1674	2349	0
1675	512	1
1675	2593	1
1675	2467	1
1675	196	1

1675	519	1
1675	1528	1
1675	3626	1
1675	460	1
1675	844	1
1675	1136	1
1675	1010	1
1675	2706	1
1675	727	1
1675	856	1
1675	601	1
1675	795	1
1675	508	1
1675	735	1
1675	3483	0
1675	2504	0
1675	2663	0
1675	3552	0
1675	2985	0
1675	2299	0
1675	1796	0
1675	1254	0
1675	2662	0
1675	3237	0
1675	2395	0
1675	258	0
1675	3465	0
1675	275	0
1675	955	0
1675	5	0

1675	2088	0
1675	2192	0
1676	2662	1
1676	551	1
1676	104	1
1676	776	1
1676	235	1
1676	2476	1
1676	334	1
1676	878	1
1676	114	1
1676	53	1
1676	90	1
1676	1228	0
1676	1564	0
1676	3542	0
1676	2462	0
1676	2432	0
1676	1691	0
1676	2102	0
1676	1852	0
1676	433	0
1676	840	0
1676	3738	0
1677	388	1
1677	1030	1
1677	528	1
1677	35	1
1677	36	1
1677	37	1

1677	3627	1
1677	3628	1
1677	2605	1
1677	46	1
1677	442	1
1677	572	1
1677	96	1
1677	1256	1
1677	107	1
1677	1389	1
1677	752	1
1677	118	1
1677	119	1
1677	3201	0
1677	184	0
1677	1234	0
1677	3290	0
1677	3448	0
1677	3726	0
1677	1471	0
1677	3123	0
1677	3361	0
1677	3547	0
1677	1524	0
1677	2302	0
1677	672	0
1677	3840	0
1677	1450	0
1677	1530	0
1677	198	0

1677	2760	0
1677	319	0
1678	419	1
1678	36	1
1678	1477	1
1678	42	1
1678	522	1
1678	1389	1
1678	111	1
1678	52	1
1678	475	1
1678	286	1
1678	2981	0
1678	1355	0
1678	2831	0
1678	3597	0
1678	2052	0
1678	936	0
1678	1200	0
1678	426	0
1678	1697	0
1678	2885	0
1679	135	1
1679	1258	1
1679	47	1
1679	1401	1
1679	538	1
1679	3691	0
1679	3126	0
1679	530	0

1679	1236	0
1679	32	0
1680	1411	1
1680	133	1
1680	74	1
1680	1293	1
1680	1294	1
1680	3629	1
1680	3630	1
1680	542	1
1680	1932	0
1680	3720	0
1680	1254	0
1680	646	0
1680	2353	0
1680	47	0
1680	588	0
1680	842	0
1681	2785	1
1681	2276	1
1681	3639	1
1681	551	1
1681	3631	1
1681	3632	1
1681	3633	1
1681	3634	1
1681	3635	1
1681	3636	1
1681	3637	1
1681	3638	1

1681	1367	1
1681	1307	0
1681	1053	0
1681	2528	0
1681	1003	0
1681	1469	0
1681	3026	0
1681	2664	0
1681	1096	0
1681	1249	0
1681	2295	0
1681	3426	0
1681	3338	0
1681	1021	0
1682	617 1	
1682	1545	1
1682	1677	1
1682	2065	1
1682	2353	1
1682	883 1	
1682	2609	1
1682	3640	1
1682	223 1	
1682	718 0	
1682	3484	0
1682	115 0	
1682	33 0	
1682	1995	0
1682	1060	0
1682	747 0	

1682	3039	0
1682	2908	0
1683	928	1
1683	353	1
1683	865	1
1683	1923	1
1683	196	1
1683	197	1
1683	3398	1
1683	1136	1
1683	1010	1
1683	438	1
1683	1528	1
1683	3641	1
1683	158	1
1683	3799	0
1683	322	0
1683	2156	0
1683	2403	0
1683	49	0
1683	3045	0
1683	3101	0
1683	1349	0
1683	2267	0
1683	937	0
1683	1609	0
1683	3445	0
1683	1481	0
1684	1217	1
1684	450	1

1684	36	1
1684	1225	1
1684	42	1
1684	3180	1
1684	655	1
1684	947	1
1684	3092	1
1684	1878	1
1684	3642	1
1684	285	1
1684	2109	0
1684	1155	0
1684	3093	0
1684	1593	0
1684	3205	0
1684	207	0
1684	333	0
1684	1070	0
1684	3562	0
1684	1411	0
1684	1252	0
1684	3453	0
1685	96	1
1685	738	1
1685	196	1
1685	998	1
1685	53	1
1685	119	1
1685	1528	1
1685	1530	1

1685	3643	1
1685	510 1	
1685	1535	1
1685	1627	0
1685	3610	0
1685	1449	0
1685	2193	0
1685	3192	0
1685	1599	0
1685	1588	0
1685	217 0	
1685	2147	0
1685	2337	0
1685	3572	0
1686	608 1	
1686	36 1	
1686	141 1	
1686	1140	1
1686	2647	1
1686	568 1	
1686	2429	1
1686	2205	0
1686	3389	0
1686	2228	0
1686	3765	0
1686	2885	0
1686	279 0	
1686	2481	0
1687	70 1	
1687	2289	1

1687	119 1	
1687	2073	1
1687	124 1	
1687	3645	1
1687	3644	1
1687	2511	0
1687	654 0	
1687	2797	0
1687	2514	0
1687	2581	0
1687	1294	0
1687	2827	0
1688	577 1	
1688	1873	1
1688	1236	1
1688	119 1	
1688	729 1	
1688	3646	1
1688	767 1	
1688	2590	0
1688	1294	0
1688	3372	0
1688	3635	0
1688	439 0	
1688	2438	0
1688	3511	0
1689	608 1	
1689	3201	1
1689	3553	1
1689	2371	1

1689	1988	1
1689	1071	1
1689	3570	1
1689	951	1
1689	2574	0
1689	1084	0
1689	155	0
1689	2926	0
1689	1030	0
1689	2168	0
1689	2292	0
1689	1257	0
1690	128	1
1690	255	1
1690	2311	1
1690	3624	1
1690	41	1
1690	74	1
1690	75	1
1690	77	1
1690	45	1
1690	3647	1
1690	951	1
1690	87	1
1690	377	1
1690	90	1
1690	127	1
1690	1815	0
1690	3837	0
1690	321	0

1690	129	0
1690	661	0
1690	1502	0
1690	2732	0
1690	97	0
1690	1569	0
1690	2398	0
1690	3400	0
1690	2414	0
1690	3217	0
1690	3358	0
1690	183	0
1691	133	1
1691	74	1
1691	77	1
1691	81	1
1691	1202	1
1691	863	1
1691	87	1
1691	127	1
1691	2116	0
1691	341	0
1691	3344	0
1691	2574	0
1691	1892	0
1691	868	0
1691	2552	0
1691	1376	0
1692	356	1
1692	197	1

1692	165	1
1692	199	1
1692	358	1
1692	776	1
1692	1098	1
1692	1503	1
1692	208	1
1692	989	1
1692	2013	1
1692	212	1
1692	54	1
1692	280	1
1692	1278	1
1692	253	1
1692	158	1
1692	159	1
1692	1650	0
1692	3306	0
1692	325	0
1692	2738	0
1692	2112	0
1692	502	0
1692	1843	0
1692	2698	0
1692	264	0
1692	2595	0
1692	2523	0
1692	3242	0
1692	2348	0
1692	1971	0

1692	1530	0
1692	1987	0
1692	1474	0
1692	1343	0
1693	128	1
1693	2689	1
1693	376	1
1693	102	1
1693	70	1
1693	136	1
1693	120	1
1693	1630	1
1693	1822	0
1693	3482	0
1693	945	0
1693	3202	0
1693	1035	0
1693	1945	0
1693	1888	0
1693	2831	0
1694	2336	1
1694	3648	1
1694	66	1
1694	134	1
1694	74	1
1694	363	1
1694	238	1
1694	79	1
1694	435	1
1694	1143	1

1694	2485	1
1694	254	1
1694	185	1
1694	90	1
1694	1787	1
1694	126	1
1694	2335	1
1694	1043	0
1694	2878	0
1694	997	0
1694	2573	0
1694	3167	0
1694	341	0
1694	2637	0
1694	1803	0
1694	3406	0
1694	2069	0
1694	2795	0
1694	891	0
1694	1708	0
1694	401	0
1694	3719	0
1694	2825	0
1694	1785	0
1695	673	1
1695	133	1
1695	134	1
1695	107	1
1695	109	1
1695	47	1

1695	187	1
1695	665	0
1695	892	0
1695	2704	0
1695	426	0
1695	282	0
1695	80	0
1695	3768	0
1696	257	1
1696	3649	1
1696	1677	1
1696	882	1
1696	883	1
1696	277	1
1696	22	1
1696	26	1
1696	221	1
1696	799	1
1696	2265	0
1696	3669	0
1696	3286	0
1696	3020	0
1696	600	0
1696	1177	0
1696	863	0
1696	2331	0
1696	878	0
1696	2538	0
1697	2003	1
1697	3622	0

1698	288	1
1698	131	1
1698	132	1
1698	133	1
1698	389	1
1698	137	1
1698	74	1
1698	237	1
1698	399	1
1698	22	1
1698	540	1
1698	957	1
1698	254	1
1698	3562	0
1698	599	0
1698	2264	0
1698	647	0
1698	955	0
1698	2359	0
1698	1780	0
1698	2651	0
1698	1025	0
1698	3094	0
1698	2625	0
1698	264	0
1698	1469	0
1699	608	1
1699	159	1
1699	3650	1
1699	45	1

1699	1807	1
1699	3282	1
1699	3347	1
1699	3580	1
1699	223	1
1699	132	0
1699	3235	0
1699	87	0
1699	1970	0
1699	1261	0
1699	3455	0
1699	3550	0
1699	1368	0
1699	2798	0
1700	1091	1
1700	3651	1
1700	3652	1
1700	1128	1
1700	297	1
1700	1003	1
1700	108	1
1700	878	1
1700	1650	1
1700	2322	1
1700	279	1
1700	1467	1
1700	252	1
1700	3423	1
1700	3472	0
1700	2182	0

1700	1611	0
1700	1993	0
1700	3597	0
1700	3252	0
1700	2094	0
1700	2976	0
1700	3442	0
1700	3486	0
1700	2607	0
1700	3412	0
1700	3047	0
1700	3184	0
1701	96	1
1701	133	1
1701	134	1
1701	40	1
1701	109	1
1701	302	1
1701	47	1
1701	560	1
1701	915	1
1701	52	1
1701	1589	1
1701	118	1
1701	1502	1
1701	863	1
1701	1530	0
1701	1288	0
1701	3025	0
1701	3650	0

1701	734	0
1701	3247	0
1701	2219	0
1701	3488	0
1701	3748	0
1701	3013	0
1701	1938	0
1701	1505	0
1701	1200	0
1701	236	0
1702	32	1
1702	161	1
1702	1333	1
1702	3517	1
1702	196	1
1702	3653	1
1702	3654	1
1702	3655	1
1702	3656	1
1702	3657	1
1702	3658	1
1702	727	1
1702	729	1
1702	474	1
1702	3292	1
1702	362	1
1702	2669	1
1702	2031	1
1702	883	1
1702	2791	0

1702	2087	0
1702	1675	0
1702	1923	0
1702	3220	0
1702	345 0	
1702	3771	0
1702	2297	0
1702	2244	0
1702	1619	0
1702	2592	0
1702	1002	0
1702	3839	0
1702	3050	0
1702	2209	0
1702	2340	0
1702	670 0	
1702	2718	0
1702	2330	0
1703	41 1	
1703	74 1	
1703	45 1	
1703	80 1	
1703	52 1	
1703	87 1	
1703	539 1	
1703	540 1	
1703	2058	0
1703	3182	0
1703	1062	0
1703	2298	0

1703	621	0
1703	3762	0
1703	2386	0
1703	2061	0
1704	96	1
1704	385	1
1704	132	1
1704	134	1
1704	1255	1
1704	79	1
1704	47	1
1704	114	1
1704	52	1
1704	116	1
1704	118	1
1704	184	1
1704	252	1
1704	1502	1
1704	863	1
1704	3563	0
1704	1187	0
1704	3377	0
1704	3668	0
1704	414	0
1704	2030	0
1704	2631	0
1704	2771	0
1704	3607	0
1704	2917	0
1704	1051	0

1704	634	0
1704	1763	0
1704	1602	0
1704	1885	0
1705	353	1
1705	131	1
1705	104	1
1705	137	1
1705	361	1
1705	1324	1
1705	1325	1
1705	957	1
1705	1487	1
1705	2065	1
1705	22	1
1705	1081	1
1705	187	1
1705	1308	1
1705	413	1
1705	1814	0
1705	3060	0
1705	2951	0
1705	740	0
1705	3173	0
1705	1834	0
1705	3664	0
1705	2741	0
1705	2525	0
1705	1648	0
1705	2119	0

1705	3359	0
1705	2671	0
1705	614	0
1705	850	0
1706	1183	1
1706	388	1
1706	1253	1
1706	391	1
1706	392	1
1706	105	1
1706	1416	1
1706	46	1
1706	112	1
1706	571	1
1706	187	1
1706	863	1
1706	925	0
1706	3696	0
1706	1669	0
1706	135	0
1706	795	0
1706	2153	0
1706	2254	0
1706	1631	0
1706	363	0
1706	1910	0
1706	3170	0
1706	3391	0
1707	1893	1
1707	70	1

1707	3659	1
1707	3660	1
1707	1623	1
1707	601	1
1707	2267	1
1707	2268	1
1707	2418	0
1707	2658	0
1707	2033	0
1707	3598	0
1707	2942	0
1707	3291	0
1707	3333	0
1707	2074	0
1708	36	1
1708	1126	1
1708	774	1
1708	3661	1
1708	782	1
1708	2063	1
1708	3279	1
1708	785	1
1708	2577	1
1708	3662	1
1708	2683	1
1708	3609	0
1708	2350	0
1708	340	0
1708	2380	0
1708	2021	0

1708	3757	0
1708	963	0
1708	2517	0
1708	1802	0
1708	1307	0
1708	2368	0
1709	132	1
1709	516	1
1709	295	1
1709	3151	1
1709	275	1
1709	1235	1
1709	117	1
1709	22	1
1709	989	1
1709	1029	0
1709	940	0
1709	2343	0
1709	1421	0
1709	112	0
1709	1107	0
1709	2837	0
1709	851	0
1709	1441	0
1710	1258	1
1710	77	1
1710	3663	1
1710	1202	1
1710	1306	1
1710	863	1

1710	1038	0
1710	2554	0
1710	1730	0
1710	106	0
1710	872	0
1710	828	0
1711	352	1
1711	608	1
1711	1091	1
1711	102	1
1711	141	1
1711	47	1
1711	2586	1
1711	55	1
1711	920	1
1711	1178	1
1711	2429	1
1711	3423	1
1711	2307	0
1711	136	0
1711	3268	0
1711	2722	0
1711	992	0
1711	560	0
1711	2965	0
1711	3367	0
1711	3392	0
1711	2893	0
1711	1173	0
1711	1839	0

1712	391	1
1712	392	1
1712	105	1
1712	1386	1
1712	107	1
1712	46	1
1712	1103	1
1712	112	1
1712	528	1
1712	3664	1
1712	1396	1
1712	55	1
1712	696	1
1712	764	1
1712	190	1
1712	3268	0
1712	2936	0
1712	3437	0
1712	862	0
1712	1710	0
1712	1511	0
1712	672	0
1712	885	0
1712	2360	0
1712	724	0
1712	1983	0
1712	2848	0
1712	3254	0
1712	3527	0
1712	2006	0

1713	133	1
1713	102	1
1713	42	1
1713	267	1
1713	652	1
1713	47	1
1713	431	1
1713	1589	1
1713	118	1
1713	55	1
1713	1517	0
1713	1583	0
1713	2369	0
1713	138	0
1713	2569	0
1713	621	0
1713	1584	0
1713	625	0
1713	3375	0
1713	2063	0
1714	96	1
1714	1888	1
1714	98	1
1714	1410	1
1714	376	1
1714	3142	1
1714	263	1
1714	714	1
1714	171	1
1714	269	1

1714	210	1
1714	3442	1
1714	184	1
1714	185	1
1714	2562	0
1714	590	0
1714	2891	0
1714	3578	0
1714	1896	0
1714	3096	0
1714	2251	0
1714	3070	0
1714	110	0
1714	2015	0
1714	1220	0
1714	3774	0
1714	666	0
1714	3304	0
1715	3298	1
1715	163	1
1715	100	1
1715	708	1
1715	518	1
1715	235	1
1715	2795	1
1715	46	1
1715	1103	1
1715	50	1
1715	882	1
1715	53	1

1715	821	1
1715	799	1
1715	2994	0
1715	3844	0
1715	1993	0
1715	3601	0
1715	2849	0
1715	1247	0
1715	2484	0
1715	671	0
1715	1898	0
1715	557	0
1715	2332	0
1715	351	0
1715	186	0
1715	3484	0
1716	1798	1
1716	1288	1
1716	42	1
1716	1836	1
1716	45	1
1716	3665	1
1716	211	1
1716	3261	1
1716	2998	1
1716	119	1
1716	284	1
1716	125	1
1716	286	1
1716	895	1

1716	1604	0
1716	952	0
1716	115	0
1716	2898	0
1716	983	0
1716	961	0
1716	3287	0
1716	140	0
1716	3743	0
1716	1784	0
1716	659	0
1716	2923	0
1716	748	0
1716	3184	0
1717	96	1
1717	802	1
1717	35	1
1717	1033	1
1717	1324	1
1717	110	1
1717	3666	1
1717	118	1
1717	824	1
1717	90	1
1717	187	1
1717	767	1
1717	925	0
1717	759	0
1717	1175	0
1717	1442	0

1717	2557	0
1717	2560	0
1717	956	0
1717	214	0
1717	758	0
1717	1590	0
1717	1457	0
1717	2340	0
1718	199	1
1718	776	1
1718	302	1
1718	47	1
1718	303	1
1718	494	1
1718	659	1
1718	118	1
1718	2839	1
1718	254	1
1718	567	0
1718	77	0
1718	2940	0
1718	3279	0
1718	3821	0
1718	1898	0
1718	2009	0
1718	3135	0
1718	3664	0
1718	133	0
1719	2498	1
1719	131	1

1719	483	1
1719	230	1
1719	231	1
1719	1416	1
1719	1257	1
1719	2985	1
1719	2353	1
1719	3667	1
1719	276	1
1719	22	1
1719	24	1
1719	891	1
1719	223	1
1719	1584	0
1719	1497	0
1719	675	0
1719	1762	0
1719	2759	0
1719	3353	0
1719	1427	0
1719	363	0
1719	2446	0
1719	847	0
1719	1232	0
1719	1065	0
1719	3137	0
1719	745	0
1719	3202	0
1720	2360	1
1720	1091	1

1720	645	1
1720	3434	1
1720	623	1
1720	145	1
1720	625	1
1720	3668	1
1720	3669	1
1720	2966	1
1720	3670	1
1720	24	1
1720	180	0
1720	2916	0
1720	3537	0
1720	587	0
1720	2233	0
1720	2794	0
1720	879	0
1720	1863	0
1720	2467	0
1720	3638	0
1720	2092	0
1720	613	0
1721	36	1
1721	332	1
1721	2892	1
1721	378	1
1721	879	1
1721	498	1
1721	307	1
1721	887	1

1721	1144	1
1721	442	1
1721	1020	1
1721	418	0
1721	1265	0
1721	1332	0
1721	63	0
1721	3068	0
1721	201	0
1721	2818	0
1721	2240	0
1721	1397	0
1721	968	0
1721	2633	0
1722	3672	1
1722	564	1
1722	3671	1
1722	468	0
1722	3568	0
1722	2754	0
1723	196	1
1723	136	1
1723	2161	1
1723	20	1
1723	538	1
1723	155	1
1723	3678	0
1723	414	0
1723	1614	0
1723	2232	0

1723	2738	0
1723	3845	0
1724	1089	1
1724	164	1
1724	36	1
1724	933	1
1724	680	1
1724	941	1
1724	887	1
1724	3673	1
1724	3674	1
1724	3675	1
1724	1308	1
1724	895	1
1724	838	0
1724	1353	0
1724	1269	0
1724	2265	0
1724	1659	0
1724	753	0
1724	3113	0
1724	432	0
1724	1713	0
1724	2840	0
1724	2625	0
1724	843	0
1725	2382	1
1725	563	1
1725	740	1
1725	2003	1

1725	126	0
1725	288	0
1725	2695	0
1725	1147	0
1726	96	1
1726	738	1
1726	802	1
1726	43	1
1726	301	1
1726	80	1
1726	210	1
1726	242	1
1726	118	1
1726	477	1
1726	201	0
1726	372	0
1726	679	0
1726	2125	0
1726	3300	0
1726	2207	0
1726	1822	0
1726	2394	0
1726	3412	0
1726	566	0
1727	2434	1
1727	105	1
1727	1260	1
1727	109	1
1727	46	1
1727	467	1

1727	52	1
1727	1461	1
1727	918	1
1727	252	1
1727	61	1
1727	1662	1
1727	3676	1
1727	2630	0
1727	3003	0
1727	2639	0
1727	1551	0
1727	3059	0
1727	3843	0
1727	3605	0
1727	1213	0
1727	2519	0
1727	2102	0
1727	3556	0
1727	2797	0
1727	2791	0
1728	96	1
1728	897	1
1728	194	1
1728	36	1
1728	171	1
1728	267	1
1728	1259	1
1728	2162	1
1728	1461	1
1728	118	1

1728	188	1
1728	2657	0
1728	2964	0
1728	819	0
1728	446	0
1728	2161	0
1728	2750	0
1728	3790	0
1728	1268	0
1728	5	0
1728	2085	0
1728	2375	0
1729	163	1
1729	1128	1
1729	105	1
1729	906	1
1729	3678	1
1729	878	1
1729	473	1
1729	3677	1
1729	670	1
1729	156	0
1729	1818	0
1729	1320	0
1729	1440	0
1729	1981	0
1729	201	0
1729	2952	0
1729	50	0
1729	194	0

1730	3680	1
1730	1901	1
1730	3679	1
1730	1862	0
1730	2473	0
1730	152 0	
1731	2401	1
1731	3682	1
1731	391 1	
1731	1255	1
1731	105 1	
1731	78 1	
1731	305 1	
1731	1589	1
1731	1974	1
1731	282 1	
1731	190 1	
1731	3237	0
1731	1287	0
1731	3684	0
1731	3566	0
1731	987 0	
1731	362 0	
1731	2087	0
1731	1813	0
1731	1614	0
1731	2153	0
1731	2216	0
1732	2498	1
1732	3683	1

1732	3684	1
1732	485	1
1732	3685	1
1732	487	1
1732	3686	1
1732	3687	1
1732	685	1
1732	1326	1
1732	3022	1
1732	22	1
1732	445	1
1732	1150	0
1732	1588	0
1732	101	0
1732	2668	0
1732	3682	0
1732	3197	0
1732	1179	0
1732	1016	0
1732	2924	0
1732	1243	0
1732	3435	0
1732	439	0
1732	1631	0
1733	288	1
1733	36	1
1733	7	1
1733	3688	1
1733	3689	1
1733	655	1

1733	240	1
1733	1329	1
1733	242	1
1733	217	1
1733	373	0
1733	760	0
1733	2099	0
1733	2608	0
1733	670	0
1733	230	0
1733	2103	0
1733	1996	0
1733	3538	0
1733	1339	0
1734	66	1
1734	1315	1
1734	72	1
1734	1448	1
1734	492	1
1734	399	1
1734	145	1
1734	433	1
1734	2835	1
1734	245	1
1734	22	1
1734	1110	1
1734	1754	1
1734	126	1
1734	825	0
1734	3510	0

1734	429	0
1734	1988	0
1734	2884	0
1734	2144	0
1734	2173	0
1734	3031	0
1734	2312	0
1734	3488	0
1734	3678	0
1734	643	0
1734	777	0
1734	3541	0
1735	439	1
1735	34	1
1735	131	1
1735	196	1
1735	1122	1
1735	210	1
1735	55	1
1735	24	1
1735	187	1
1735	190	1
1735	286	1
1735	1343	0
1735	1802	0
1735	309	0
1735	1780	0
1735	3472	0
1735	3608	0
1735	891	0

1735	2770	0
1735	521	0
1735	3356	0
1735	1534	0
1736	2696	1
1736	171	1
1736	55	1
1736	118	1
1736	254	1
1736	859	1
1736	830	1
1736	2675	0
1736	976	0
1736	1397	0
1736	826	0
1736	1004	0
1736	1174	0
1736	2416	0
1737	391	1
1737	392	1
1737	105	1
1737	3690	1
1737	107	1
1737	3691	1
1737	46	1
1737	1103	1
1737	2739	1
1737	52	1
1737	1396	1
1737	55	1

1737	648	0
1737	729	0
1737	1340	0
1737	112	0
1737	3317	0
1737	2145	0
1737	3023	0
1737	2135	0
1737	3454	0
1737	3380	0
1737	602	0
1737	2088	0
1738	1218	1
1738	134	1
1738	1545	1
1738	171	1
1738	235	1
1738	109	1
1738	858	1
1738	187	1
1738	254	1
1738	286	1
1738	2681	0
1738	2726	0
1738	1070	0
1738	1183	0
1738	258	0
1738	3146	0
1738	2498	0
1738	1128	0

1738	1743	0
1738	255	0
1739	2469	1
1739	102	1
1739	1255	1
1739	1128	1
1739	44	1
1739	3692	1
1739	878	1
1739	111	1
1739	1168	1
1739	3441	1
1739	55	1
1739	1175	1
1739	2910	1
1739	218	0
1739	994	0
1739	553	0
1739	1716	0
1739	3571	0
1739	656	0
1739	3748	0
1739	832	0
1739	1817	0
1739	3248	0
1739	1649	0
1739	745	0
1739	589	0
1740	105	1
1740	1545	1

1740	1259	1
1740	46	1
1740	1231	1
1740	2992	1
1740	114	1
1740	467	1
1740	2643	1
1740	1397	1
1740	918	1
1740	55	1
1740	764	1
1740	1662	1
1740	2482	0
1740	2881	0
1740	363	0
1740	288	0
1740	2249	0
1740	3364	0
1740	498	0
1740	576	0
1740	570	0
1740	3684	0
1740	2026	0
1740	654	0
1740	1379	0
1740	2041	0
1741	96	1
1741	70	1
1741	136	1
1741	808	1

1741	1448	1
1741	77	1
1741	46	1
1741	80	1
1741	117	1
1741	120	1
1741	2269	1
1741	127	1
1741	2496	0
1741	2828	0
1741	3056	0
1741	1871	0
1741	1862	0
1741	2194	0
1741	3020	0
1741	3535	0
1741	2939	0
1741	3406	0
1741	1542	0
1741	1611	0
1742	1248	1
1742	235	1
1742	876	1
1742	3693	1
1742	3694	1
1742	154	1
1742	1883	1
1742	1021	1
1742	159	1
1742	1261	0

1742	236	0
1742	1430	0
1742	1302	0
1742	1433	0
1742	3587	0
1742	1578	0
1742	2483	0
1742	2992	0
1743	296	1
1743	442	1
1743	254	1
1743	271	1
1743	3676	0
1743	1318	0
1743	1308	0
1743	2671	0
1744	128	1
1744	102	1
1744	232	1
1744	906	1
1744	3695	1
1744	979	1
1744	980	1
1744	1556	1
1744	282	1
1744	60	1
1744	907	0
1744	2620	0
1744	1020	0
1744	2140	0

1744	1010	0
1744	1632	0
1744	3042	0
1744	630	0
1744	3085	0
1744	2099	0
1745	257	1
1745	3365	1
1745	235	1
1745	3696	1
1745	2065	1
1745	3697	1
1745	307	1
1745	3698	1
1745	501	1
1745	1592	1
1745	799	1
1745	2422	0
1745	1315	0
1745	638	0
1745	274	0
1745	3794	0
1745	3029	0
1745	1014	0
1745	846	0
1745	912	0
1745	934	0
1745	2033	0
1746	171	1
1746	267	1

1746	364	1
1746	79	1
1746	47	1
1746	437	1
1746	1814	1
1746	184	1
1746	185	1
1746	863	1
1746	501	0
1746	2400	0
1746	1255	0
1746	2793	0
1746	2523	0
1746	2173	0
1746	697	0
1746	638	0
1746	1771	0
1746	2440	0
1747	832	1
1747	136	1
1747	377	1
1747	1228	1
1747	77	1
1747	270	1
1747	184	1
1747	185	1
1747	733	0
1747	1343	0
1747	898	0
1747	643	0

1747	2023	0
1747	3522	0
1747	2139	0
1747	1550	0
1748	3298	1
1748	107 1	
1748	1003	1
1748	2385	1
1748	3699	1
1748	3700	1
1748	3353	1
1748	2298	1
1748	1691	1
1748	764 1	
1748	1091	0
1748	2257	0
1748	2927	0
1748	11 0	
1748	1390	0
1748	346 0	
1748	1355	0
1748	2638	0
1748	626 0	
1748	2045	0
1749	673 1	
1749	66 1	
1749	98 1	
1749	131 1	
1749	102 1	
1749	72 1	

1749	232	1
1749	377	1
1749	87	1
1749	184	1
1749	185	1
1749	442	1
1749	1276	1
1749	1278	1
1749	127	1
1749	1059	0
1749	2416	0
1749	858	0
1749	3071	0
1749	2387	0
1749	3598	0
1749	943	0
1749	2820	0
1749	408	0
1749	3464	0
1749	2293	0
1749	165	0
1749	3212	0
1749	1351	0
1749	3703	0
1750	1095	1
1750	105	1
1750	2638	1
1750	3186	1
1750	2900	1
1750	55	1

1750	571	1
1750	3780	0
1750	3696	0
1750	3005	0
1750	1514	0
1750	3039	0
1750	2817	0
1750	1550	0
1751	196	1
1751	74	1
1751	1612	1
1751	1202	1
1751	3701	1
1751	22	1
1751	87	1
1751	3702	1
1751	185	1
1751	58	1
1751	539	1
1751	127	1
1751	2098	0
1751	2130	0
1751	1273	0
1751	1253	0
1751	234	0
1751	1599	0
1751	3078	0
1751	1596	0
1751	1005	0
1751	14	0

1751	1070	0
1751	3639	0
1752	102	1
1752	105	1
1752	47	1
1752	210	1
1752	52	1
1752	3703	1
1752	697	1
1752	190	1
1752	49	0
1752	2921	0
1752	2781	0
1752	1042	0
1752	1895	0
1752	2279	0
1752	975	0
1752	1679	0
1753	96	1
1753	771	1
1753	806	1
1753	1254	1
1753	241	1
1753	755	1
1753	52	1
1753	3579	1
1753	2723	0
1753	313	0
1753	42	0
1753	2495	0

1753	3412	0
1753	2235	0
1753	1434	0
1753	2609	0
1754	128	1
1754	515	1
1754	510	1
1754	136	1
1754	426	1
1754	791	1
1754	2028	1
1754	3704	1
1754	54	1
1754	790	1
1754	376	1
1754	3705	1
1754	3706	1
1754	795	1
1754	62	1
1754	415	1
1754	992	0
1754	3627	0
1754	2	0
1754	3072	0
1754	1789	0
1754	1745	0
1754	2205	0
1754	1775	0
1754	732	0
1754	1052	0

1754	645	0
1754	32	0
1754	1026	0
1754	1897	0
1754	3541	0
1754	2310	0
1755	827	1
1755	106	1
1755	940	1
1755	3707	1
1755	3708	1
1755	1438	1
1755	2567	0
1755	3127	0
1755	1121	0
1755	675	0
1755	2789	0
1755	161	0
1756	34	1
1756	195	1
1756	196	1
1756	483	1
1756	486	1
1756	3709	1
1756	200	1
1756	939	1
1756	1100	1
1756	52	1
1756	727	1
1756	314	1

1756	155	1
1756	763	1
1756	1309	1
1756	479	1
1756	2159	0
1756	3633	0
1756	3199	0
1756	1887	0
1756	3050	0
1756	1876	0
1756	3816	0
1756	3102	0
1756	3401	0
1756	3781	0
1756	510	0
1756	1142	0
1756	843	0
1756	797	0
1756	1433	0
1756	2984	0
1757	385	1
1757	66	1
1757	446	1
1757	74	1
1757	75	1
1757	334	1
1757	399	1
1757	276	1
1757	126	1
1757	2263	0

1757	2558	0
1757	3291	0
1757	516	0
1757	86	0
1757	1241	0
1757	3787	0
1757	2287	0
1757	1821	0
1758	105	1
1758	953	1
1758	36	1
1758	102	1
1758	2344	0
1758	1562	0
1758	3571	0
1758	3089	0
1759	3712	1
1759	513	1
1759	3235	1
1759	196	1
1759	519	1
1759	967	1
1759	3710	1
1759	462	1
1759	2030	1
1759	275	1
1759	2579	1
1759	2649	1
1759	314	1
1759	508	1

1759	510	1
1759	3711	1
1759	2504	0
1759	3719	0
1759	1932	0
1759	17	0
1759	2483	0
1759	3705	0
1759	2868	0
1759	2212	0
1759	1933	0
1759	3246	0
1759	2357	0
1759	248	0
1759	1257	0
1759	2895	0
1759	661	0
1759	2980	0
1760	3713	1
1760	3714	1
1760	35	1
1760	36	1
1760	293	1
1760	3597	1
1760	1720	1
1760	1583	1
1760	624	1
1760	50	1
1760	629	1
1760	2008	1

1760	632	1
1760	1721	1
1760	2142	1
1760	2143	1
1760	3774	0
1760	3208	0
1760	933	0
1760	2827	0
1760	588	0
1760	3745	0
1760	3617	0
1760	2119	0
1760	582	0
1760	3654	0
1760	1586	0
1760	3263	0
1760	1203	0
1760	3486	0
1760	1455	0
1760	1219	0
1761	33	1
1761	35	1
1761	3715	1
1761	197	1
1761	104	1
1761	105	1
1761	456	1
1761	1416	1
1761	109	1
1761	46	1

1761	57	1
1761	1051	1
1761	158	1
1761	2619	0
1761	2450	0
1761	3248	0
1761	2416	0
1761	2882	0
1761	916	0
1761	3090	0
1761	2217	0
1761	296	0
1761	3797	0
1761	2255	0
1761	3110	0
1761	724	0
1762	834	1
1762	3716	1
1762	805	1
1762	948	1
1762	52	1
1762	1291	0
1762	1971	0
1762	3408	0
1762	433	0
1762	1321	0
1763	98	1
1763	137	1
1763	74	1
1763	1324	1

1763	276	1
1763	22	1
1763	378	1
1763	923	1
1763	127	1
1763	3758	0
1763	711	0
1763	3060	0
1763	1532	0
1763	2633	0
1763	3083	0
1763	3380	0
1763	2685	0
1763	3769	0
1764	96	1
1764	1218	1
1764	389	1
1764	3717	1
1764	3718	1
1764	232	1
1764	2058	1
1764	459	1
1764	45	1
1764	141	1
1764	2703	1
1764	56	1
1764	154	1
1764	1883	1
1764	413	1
1764	2017	0

1764	2821	0
1764	1599	0
1764	736	0
1764	1490	0
1764	1145	0
1764	1274	0
1764	2138	0
1764	970	0
1764	1987	0
1764	2405	0
1764	3250	0
1764	2106	0
1764	890	0
1764	3554	0
1765	32	1
1765	353	1
1765	355	1
1765	1859	1
1765	1190	1
1765	1545	1
1765	2730	1
1765	2604	1
1765	950	1
1765	759	1
1765	282	1
1765	2462	1
1765	420	0
1765	86	0
1765	105	0
1765	2305	0

1765	3750	0
1765	413	0
1765	780	0
1765	2674	0
1765	3247	0
1765	3068	0
1765	974	0
1765	2905	0
1766	192	1
1766	827	1
1766	36	1
1766	197	1
1766	3719	1
1766	956	1
1766	107	1
1766	459	1
1766	401	1
1766	1426	1
1766	438	1
1766	1046	1
1766	1496	1
1766	442	1
1766	187	1
1766	220	1
1766	3831	0
1766	1368	0
1766	764	0
1766	547	0
1766	2204	0
1766	2242	0

1766	2694	0
1766	2009	0
1766	3417	0
1766	543	0
1766	2035	0
1766	743	0
1766	1527	0
1766	3245	0
1766	2098	0
1766	2254	0
1767	66	1
1767	130	1
1767	131	1
1767	136	1
1767	74	1
1767	202	1
1767	109	1
1767	80	1
1767	382	1
1767	127	1
1767	3051	0
1767	1384	0
1767	3811	0
1767	3122	0
1767	2012	0
1767	1293	0
1767	685	0
1767	2487	0
1767	3733	0
1767	1965	0

1768	96	1
1768	802	1
1768	580	1
1768	776	1
1768	42	1
1768	243	1
1768	504	1
1768	250	1
1768	125	1
1768	1104	0
1768	885	0
1768	819	0
1768	3829	0
1768	2823	0
1768	2265	0
1768	1490	0
1768	3367	0
1768	106	0
1769	97	1
1769	34	1
1769	36	1
1769	740	1
1769	836	1
1769	242	1
1769	726	1
1769	250	1
1769	347	1
1769	3790	0
1769	3425	0
1769	1796	0

1769	2817	0
1769	3074	0
1769	964	0
1769	3549	0
1769	2522	0
1769	1957	0
1770	993	1
1770	228	1
1770	36	1
1770	998	1
1770	2340	1
1770	2856	1
1770	44	1
1770	654	1
1770	879	1
1770	54	1
1770	1752	1
1770	221	1
1770	3782	0
1770	294	0
1770	3255	0
1770	3253	0
1770	2815	0
1770	3037	0
1770	1044	0
1770	1734	0
1770	820	0
1770	821	0
1770	3733	0
1770	3560	0

1771	923	1
1771	1051	1
1771	202	1
1771	363	1
1771	204	1
1771	80	1
1771	2160	1
1771	1143	1
1771	185	1
1771	859	1
1771	891	0
1771	845	0
1771	3476	0
1771	2683	0
1771	300	0
1771	1163	0
1771	2757	0
1771	1986	0
1771	479	0
1771	3837	0
1772	3720	1
1772	3721	1
1772	3722	1
1772	3723	1
1772	1036	1
1772	3724	1
1772	3386	1
1772	478	1
1772	3719	0
1772	117	0

1772	2280	0
1772	3715	0
1772	118	0
1772	1019	0
1772	155	0
1772	3784	0
1773	515	1
1773	422	1
1773	1513	1
1773	426	1
1773	3498	1
1773	3725	1
1773	1011	1
1773	3191	1
1773	601	1
1773	794	1
1773	508	1
1773	1628	1
1773	735	1
1773	2708	0
1773	2606	0
1773	1748	0
1773	1480	0
1773	3573	0
1773	99	0
1773	3160	0
1773	672	0
1773	2393	0
1773	268	0
1773	1345	0

1773	2687	0
1773	1230	0
1774	795	1
1774	769	1
1774	418	1
1774	67	1
1774	1092	1
1774	965	1
1774	422	1
1774	1482	1
1774	109	1
1774	3726	1
1774	3727	1
1774	3728	1
1774	531	1
1774	2579	1
1774	277	1
1774	2264	1
1774	601	1
1774	155	1
1774	2676	0
1774	2777	0
1774	302	0
1774	1414	0
1774	3840	0
1774	1562	0
1774	3654	0
1774	761	0
1774	1026	0
1774	3573	0

1774	1088	0
1774	1341	0
1774	985	0
1774	1403	0
1774	1102	0
1774	3619	0
1774	3698	0
1774	903	0
1775	864	1
1775	109	1
1775	941	1
1775	16	1
1775	1650	1
1775	54	1
1775	55	1
1775	990	1
1775	159	1
1775	1062	0
1775	1693	0
1775	2350	0
1775	39	0
1775	3429	0
1775	1533	0
1775	3471	0
1775	399	0
1775	2218	0
1776	770	1
1776	165	1
1776	1126	1
1776	104	1

1776	235 1	
1776	3407	1
1776	2065	1
1776	2193	1
1776	3729	1
1776	3730	1
1776	3731	1
1776	3732	1
1776	3733	1
1776	252 1	
1776	3677	1
1776	607 1	
1776	2235	0
1776	2218	0
1776	1608	0
1776	1595	0
1776	668 0	
1776	262 0	
1776	3028	0
1776	506 0	
1776	2802	0
1776	1255	0
1776	2813	0
1776	2698	0
1776	465 0	
1776	3133	0
1776	2993	0
1776	3011	0
1777	1542	1
1777	231 1	

1777	1257	1
1777	106	1
1777	107	1
1777	1385	1
1777	3245	1
1777	46	1
1777	3734	1
1777	858	1
1777	254	1
1777	863	1
1777	3038	0
1777	2384	0
1777	3578	0
1777	1456	0
1777	513	0
1777	3773	0
1777	1187	0
1777	1546	0
1777	378	0
1777	1184	0
1777	3583	0
1777	1946	0
1778	96	1
1778	998	1
1778	776	1
1778	3735	1
1778	235	1
1778	1869	1
1778	210	1
1778	52	1

1778	118 1	
1778	854 1	
1778	282 1	
1778	732 1	
1778	3782	0
1778	3178	0
1778	2792	0
1778	2887	0
1778	847 0	
1778	3181	0
1778	1855	0
1778	635 0	
1778	896 0	
1778	1952	0
1778	3498	0
1778	710 0	
1779	128 1	
1779	418 1	
1779	1956	1
1779	358 1	
1779	366 1	
1779	590 1	
1779	1711	1
1779	594 1	
1779	3736	1
1779	3737	1
1779	3738	1
1779	3739	1
1779	3295	1
1779	3133	0

1779	1375	0
1779	2401	0
1779	3751	0
1779	1490	0
1779	246	0
1779	3404	0
1779	578	0
1779	3711	0
1779	135	0
1779	3222	0
1779	1305	0
1779	1440	0
1780	608	1
1780	36	1
1780	102	1
1780	141	1
1780	52	1
1780	53	1
1780	54	1
1780	1108	1
1780	154	1
1780	2879	0
1780	3199	0
1780	3288	0
1780	1842	0
1780	2316	0
1780	3391	0
1780	2317	0
1780	1060	0
1780	519	0

1781	102	1
1781	105	1
1781	46	1
1781	52	1
1781	1589	1
1781	118	1
1781	3156	1
1781	282	1
1781	188	1
1781	863	1
1781	521	0
1781	2992	0
1781	3833	0
1781	743	0
1781	1358	0
1781	1599	0
1781	1593	0
1781	264	0
1781	2478	0
1781	1374	0
1782	3740	1
1782	163	1
1782	1315	1
1782	870	1
1782	105	1
1782	527	1
1782	1972	1
1782	2073	1
1782	472	1
1782	313	1

1782	476	1
1782	286	1
1782	1076	0
1782	2897	0
1782	2939	0
1782	3004	0
1782	1188	0
1782	2406	0
1782	1741	0
1782	1155	0
1782	1276	0
1782	34	0
1782	618	0
1782	2952	0
1783	128	1
1783	2311	1
1783	80	1
1783	1624	1
1783	796	1
1783	3741	1
1783	542	1
1783	863	0
1783	1500	0
1783	2300	0
1783	1303	0
1783	772	0
1783	3733	0
1783	383	0
1784	325	1
1784	241	1

1784	242	1
1784	1523	1
1784	213	1
1784	1980	1
1784	126	1
1784	1151	1
1784	3246	0
1784	2592	0
1784	2207	0
1784	2338	0
1784	1301	0
1784	1311	0
1784	2184	0
1784	788	0
1785	224	1
1785	3681	1
1785	2826	1
1785	971	1
1785	460	1
1785	1338	1
1785	1646	1
1785	1455	1
1785	2674	1
1785	1237	1
1785	438	1
1785	343	1
1785	24	1
1785	314	1
1785	732	1
1785	478	1

1785	1421	0
1785	721	0
1785	133	0
1785	2594	0
1785	389	0
1785	2076	0
1785	2427	0
1785	3392	0
1785	3423	0
1785	2378	0
1785	3503	0
1785	2864	0
1785	1610	0
1785	1292	0
1785	1968	0
1785	2082	0
1786	96	1
1786	385	1
1786	1315	1
1786	1604	1
1786	2820	1
1786	78	1
1786	1589	1
1786	1144	1
1786	2782	1
1786	1248	0
1786	114	0
1786	846	0
1786	397	0
1786	2631	0

1786	3841	0
1786	2130	0
1786	2746	0
1786	3458	0
1787	2969	1
1787	155 1	
1787	3742	1
1787	3743	1
1787	3744	1
1787	3745	1
1787	3746	1
1787	3747	1
1787	3748	1
1787	3749	1
1787	3750	1
1787	3751	1
1787	3752	1
1787	3753	1
1787	959 1	
1787	962 1	
1787	1512	1
1787	362 1	
1787	763 1	
1787	608 0	
1787	230 0	
1787	1202	0
1787	2358	0
1787	1165	0
1787	1265	0
1787	2465	0

1787	485	0
1787	195	0
1787	2365	0
1787	2892	0
1787	1917	0
1787	3061	0
1787	2371	0
1787	174	0
1787	1102	0
1787	718	0
1787	2947	0
1787	1151	0
1788	1304	1
1788	2594	1
1788	196	1
1788	517	1
1788	198	1
1788	486	1
1788	967	1
1788	460	1
1788	2452	1
1788	2453	1
1788	727	1
1788	24	1
1788	155	1
1788	509	1
1788	2264	1
1788	479	1
1788	2424	0
1788	1099	0

1788	167	0
1788	1922	0
1788	2876	0
1788	3759	0
1788	3016	0
1788	120	0
1788	2166	0
1788	3669	0
1788	488	0
1788	3779	0
1788	3802	0
1788	3704	0
1788	22	0
1788	1601	0
1789	3073	1
1789	358	1
1789	102	1
1789	2152	1
1789	3754	1
1789	395	1
1789	2736	1
1789	306	1
1789	2168	1
1789	3034	1
1789	1022	1
1789	315	0
1789	3058	0
1789	2826	0
1789	1666	0
1789	998	0

1789	2018	0
1789	2390	0
1789	2356	0
1789	841	0
1789	3841	0
1789	3718	0
1790	96	1
1790	1446	1
1790	391	1
1790	105	1
1790	46	1
1790	243	1
1790	571	1
1790	765	1
1790	1815	0
1790	2978	0
1790	3507	0
1790	497	0
1790	2543	0
1790	3316	0
1790	275	0
1790	3476	0
1791	163	1
1791	36	1
1791	105	1
1791	106	1
1791	233	1
1791	361	1
1791	1257	1
1791	46	1

1791	110	1
1791	111	1
1791	55	1
1791	413	1
1791	159	1
1791	2111	0
1791	3375	0
1791	1120	0
1791	1611	0
1791	1889	0
1791	1827	0
1791	740	0
1791	3520	0
1791	228	0
1791	1262	0
1791	3380	0
1791	1861	0
1791	169	0
1792	96	1
1792	1253	1
1792	295	1
1792	105	1
1792	1389	1
1792	46	1
1792	1615	1
1792	528	1
1792	722	1
1792	55	1
1792	764	1
1792	446	1

1792	727	0
1792	520	0
1792	1411	0
1792	567	0
1792	209	0
1792	2192	0
1792	103	0
1792	3019	0
1792	725	0
1792	155	0
1792	2169	0
1792	779	0
1793	353	1
1793	483	1
1793	389	1
1793	839	1
1793	3755	1
1793	45	1
1793	46	1
1793	399	1
1793	1197	1
1793	1043	1
1793	276	1
1793	52	1
1793	55	1
1793	2447	0
1793	1516	0
1793	418	0
1793	3467	0
1793	2942	0

1793	659	0
1793	1937	0
1793	697	0
1793	1826	0
1793	936	0
1793	1026	0
1793	3671	0
1793	470	0
1794	165	1
1794	391	1
1794	105	1
1794	107	1
1794	46	1
1794	462	1
1794	1039	1
1794	1298	1
1794	52	1
1794	118	1
1794	252	1
1794	1855	0
1794	3206	0
1794	1053	0
1794	878	0
1794	227	0
1794	1135	0
1794	2384	0
1794	2268	0
1794	212	0
1794	3444	0
1794	1579	0

1795	257	1
1795	109	1
1795	879	1
1795	1105	1
1795	498	1
1795	883	1
1795	277	1
1795	799	1
1795	1485	0
1795	993	0
1795	806	0
1795	364	0
1795	2377	0
1795	2586	0
1795	1880	0
1795	3246	0
1796	34	1
1796	578	1
1796	36	1
1796	803	1
1796	2089	1
1796	42	1
1796	3756	1
1796	16	1
1796	209	1
1796	210	1
1796	531	1
1796	657	1
1796	756	1
1796	1777	1

1796	286	1
1796	927	1
1796	1512	0
1796	1884	0
1796	1242	0
1796	2341	0
1796	1701	0
1796	309	0
1796	2183	0
1796	428	0
1796	279	0
1796	3128	0
1796	1963	0
1796	518	0
1796	2706	0
1796	121	0
1796	2902	0
1796	3211	0
1797	483	1
1797	3757	1
1797	3758	1
1797	3759	1
1797	22	1
1797	472	1
1797	1179	1
1797	1823	1
1797	1795	0
1797	1945	0
1797	3756	0
1797	2796	0

1797	3004	0
1797	1294	0
1797	747	0
1797	566	0
1798	356	1
1798	231	1
1798	360	1
1798	105	1
1798	361	1
1798	107	1
1798	2281	1
1798	1078	1
1798	863	1
1798	1521	0
1798	3819	0
1798	919	0
1798	584	0
1798	2781	0
1798	3683	0
1798	1486	0
1798	3232	0
1798	2601	0
1799	832	1
1799	1153	1
1799	98	1
1799	1412	1
1799	135	1
1799	72	1
1799	137	1
1799	75	1

1799	267	1
1799	206	1
1799	79	1
1799	3760	1
1799	115	1
1799	184	1
1799	1288	0
1799	2481	0
1799	2972	0
1799	1355	0
1799	1917	0
1799	2117	0
1799	3590	0
1799	1366	0
1799	3573	0
1799	2892	0
1799	1696	0
1799	3722	0
1799	1921	0
1799	2720	0
1800	353	1
1800	1122	1
1800	134	1
1800	3145	1
1800	236	1
1800	493	1
1800	3533	1
1800	1775	1
1800	2162	1
1800	1974	1

1800	55	1
1800	1240	1
1800	2072	1
1800	2523	0
1800	2458	0
1800	646	0
1800	474	0
1800	1732	0
1800	2514	0
1800	421	0
1800	2704	0
1800	2592	0
1800	1865	0
1800	3818	0
1800	1138	0
1800	3736	0
1801	104	1
1801	136	1
1801	137	1
1801	765	1
1801	524	1
1801	1324	1
1801	46	1
1801	111	1
1801	118	1
1801	55	1
1801	187	1
1801	413	1
1801	1714	0
1801	737	0

1801	434	0
1801	1820	0
1801	2032	0
1801	724	0
1801	3399	0
1801	335	0
1801	1168	0
1801	431	0
1801	3501	0
1801	3753	0
1802	98	1
1802	72	1
1802	137	1
1802	74	1
1802	377	1
1802	79	1
1802	80	1
1802	3761	1
1802	373	1
1802	184	1
1802	185	1
1802	538	1
1802	923	1
1802	858	0
1802	3213	0
1802	1534	0
1802	3557	0
1802	1365	0
1802	2995	0
1802	2570	0

1802	3162	0
1802	902	0
1802	3135	0
1802	2097	0
1802	3808	0
1802	3324	0
1803	96	1
1803	2696	1
1803	560	1
1803	3762	1
1803	52	1
1803	756	1
1803	118	1
1803	254	1
1803	126	1
1803	2315	0
1803	547	0
1803	348	0
1803	2018	0
1803	644	0
1803	3317	0
1803	1473	0
1803	3813	0
1803	3608	0
1804	135	1
1804	267	1
1804	363	1
1804	3213	1
1804	206	1
1804	78	1

1804	434	1
1804	3763	1
1804	374	1
1804	184	1
1804	185	1
1804	538	1
1804	413	1
1804	1983	0
1804	113	0
1804	2872	0
1804	321	0
1804	2142	0
1804	2244	0
1804	2132	0
1804	686	0
1804	1967	0
1804	547	0
1804	138	0
1804	2256	0
1804	2471	0
1805	1092	1
1805	293	1
1805	1541	1
1805	109	1
1805	112	1
1805	1104	1
1805	2353	1
1805	3764	1
1805	55	1
1805	57	1

1805	1341	1
1805	190 1	
1805	3539	0
1805	2460	0
1805	1030	0
1805	1368	0
1805	1530	0
1805	1071	0
1805	3730	0
1805	1860	0
1805	768 0	
1805	3617	0
1805	1025	0
1805	1137	0
1806	2946	1
1806	36 1	
1806	3767	1
1806	109 1	
1806	275 1	
1806	3769	1
1806	3765	1
1806	3766	1
1806	887 1	
1806	3768	1
1806	1209	1
1806	3770	1
1806	3814	0
1806	964 0	
1806	2282	0
1806	39 0	

1806	2025	0
1806	1015	0
1806	3344	0
1806	65	0
1806	1503	0
1806	1642	0
1806	1242	0
1806	467	0
1807	1122	1
1807	137	1
1807	75	1
1807	267	1
1807	1868	1
1807	78	1
1807	3533	1
1807	3506	1
1807	55	1
1807	121	1
1807	1239	1
1807	3771	1
1807	572	1
1807	2416	0
1807	848	0
1807	1526	0
1807	438	0
1807	1668	0
1807	2231	0
1807	1713	0
1807	2646	0
1807	3719	0

1807	2340	0
1807	2438	0
1807	3038	0
1807	113 0	
1808	3772	1
1808	517 1	
1808	198 1	
1808	2856	1
1808	460 1	
1808	1328	1
1808	474 1	
1808	1968	1
1808	1236	1
1808	2068	1
1808	22 1	
1808	727 1	
1808	854 1	
1808	729 1	
1808	314 1	
1808	155 1	
1808	732 1	
1808	3773	1
1808	3192	0
1808	714 0	
1808	172 0	
1808	1338	0
1808	148 0	
1808	209 0	
1808	2039	0
1808	3587	0

1808	268	0
1808	770	0
1808	867	0
1808	861	0
1808	3095	0
1808	3310	0
1808	1699	0
1808	2383	0
1808	1815	0
1808	212	0
1809	481	1
1809	34	1
1809	133	1
1809	231	1
1809	40	1
1809	457	1
1809	107	1
1809	652	1
1809	209	1
1809	1234	1
1809	1235	1
1809	1397	1
1809	858	1
1809	572	1
1809	3774	1
1809	1886	0
1809	1622	0
1809	3569	0
1809	179	0
1809	3533	0

1809	3642	0
1809	3656	0
1809	1867	0
1809	3080	0
1809	1515	0
1809	3331	0
1809	437	0
1809	821	0
1809	754	0
1809	150	0
1810	3713	1
1810	35	1
1810	644	1
1810	165	1
1810	70	1
1810	1671	1
1810	632	1
1810	844	1
1810	462	1
1810	622	1
1810	2476	1
1810	2703	1
1810	118	1
1810	54	1
1810	24	1
1810	3775	1
1810	2019	0
1810	3282	0
1810	2496	0
1810	1051	0

1810	3450	0
1810	872 0	
1810	1454	0
1810	295 0	
1810	1297	0
1810	2370	0
1810	2301	0
1810	2177	0
1810	1256	0
1810	3601	0
1810	3203	0
1810	1063	0
1811	72 1	
1811	74 1	
1811	1258	1
1811	1324	1
1811	80 1	
1811	916 1	
1811	919 1	
1811	1144	1
1811	857 1	
1811	923 1	
1811	254 1	
1811	127 1	
1811	498 0	
1811	3421	0
1811	3167	0
1811	3426	0
1811	3229	0
1811	3240	0

1811	2317	0
1811	1796	0
1811	568	0
1811	3112	0
1811	3103	0
1811	1643	0
1812	3776	1
1812	223	1
1812	165	1
1812	105	1
1812	235	1
1812	109	1
1812	111	1
1812	2065	1
1812	22	1
1812	571	1
1812	187	1
1812	252	1
1812	413	1
1812	159	1
1812	3382	0
1812	1985	0
1812	847	0
1812	1204	0
1812	1731	0
1812	1128	0
1812	3265	0
1812	3472	0
1812	71	0
1812	3774	0

1812	2504	0
1812	51	0
1812	1835	0
1812	2206	0
1813	923	1
1813	133	1
1813	105	1
1813	137	1
1813	1258	1
1813	1324	1
1813	78	1
1813	22	1
1813	55	1
1813	187	1
1813	572	1
1813	863	1
1813	3786	0
1813	2053	0
1813	1505	0
1813	226	0
1813	876	0
1813	2021	0
1813	3520	0
1813	3751	0
1813	1330	0
1813	3534	0
1813	1920	0
1813	3461	0
1814	354	1
1814	1858	1

1814	388	1
1814	105	1
1814	107	1
1814	46	1
1814	2478	1
1814	118	1
1814	55	1
1814	764	1
1814	1183	1
1814	1008	0
1814	2390	0
1814	3290	0
1814	2336	0
1814	2900	0
1814	2161	0
1814	3592	0
1814	2743	0
1814	1754	0
1814	3141	0
1814	1292	0
1815	3777	1
1815	3778	1
1815	516	1
1815	453	1
1815	1712	1
1815	2517	1
1815	191	1
1815	2991	0
1815	3831	0
1815	1240	0

1815	1185	0
1815	317	0
1815	1221	0
1815	1033	0
1816	1057	1
1816	290	1
1816	2531	1
1816	3779	1
1816	3780	1
1816	166	1
1816	1351	1
1816	3781	1
1816	20	1
1816	279	1
1816	1114	1
1816	1051	1
1816	1725	1
1816	1717	0
1816	2510	0
1816	3100	0
1816	1904	0
1816	732	0
1816	2348	0
1816	3491	0
1816	16	0
1816	2131	0
1816	3450	0
1816	3541	0
1816	3619	0
1816	2228	0

1817	132	1
1817	135	1
1817	72	1
1817	73	1
1817	74	1
1817	1258	1
1817	77	1
1817	81	1
1817	374	1
1817	957	1
1817	127	1
1817	2673	0
1817	847	0
1817	106	0
1817	2534	0
1817	2870	0
1817	382	0
1817	3212	0
1817	1242	0
1817	454	0
1817	489	0
1817	3033	0
1818	74	1
1818	399	1
1818	80	1
1818	55	1
1818	187	1
1818	79	0
1818	3808	0
1818	3357	0

1818	1802	0
1818	2467	0
1819	354	1
1819	356	1
1819	37	1
1819	133	1
1819	1092	1
1819	2020	1
1819	41	1
1819	74	1
1819	1783	1
1819	55	1
1819	1754	1
1819	1082	1
1819	27	1
1819	1310	1
1819	1206	0
1819	394	0
1819	1908	0
1819	3679	0
1819	543	0
1819	2411	0
1819	65	0
1819	893	0
1819	1759	0
1819	1550	0
1819	721	0
1819	1932	0
1819	1096	0
1819	2571	0

1820	131	1
1820	133	1
1820	230	1
1820	137	1
1820	237	1
1820	110	1
1820	1651	1
1820	1556	1
1820	55	1
1820	184	1
1820	185	1
1820	282	1
1820	923	1
1820	252	1
1820	159	1
1820	2578	0
1820	269	0
1820	2703	0
1820	902	0
1820	3405	0
1820	3552	0
1820	1056	0
1820	1046	0
1820	1934	0
1820	300	0
1820	3439	0
1820	2200	0
1820	235	0
1820	639	0
1820	3094	0

1821	96	1
1821	352	1
1821	2401	1
1821	772	1
1821	302	1
1821	47	1
1821	304	1
1821	52	1
1821	278	1
1821	254	1
1821	435	0
1821	1419	0
1821	1560	0
1821	493	0
1821	3526	0
1821	107	0
1821	740	0
1821	1771	0
1821	2344	0
1821	198	0
1822	97	1
1822	1122	1
1822	36	1
1822	1401	1
1822	1263	1
1822	756	1
1822	217	1
1822	250	1
1822	188	1
1822	2576	0

1822	1128	0
1822	566	0
1822	567	0
1822	2051	0
1822	1766	0
1822	2309	0
1822	3576	0
1822	2477	0
1823	1024	1
1823	611	1
1823	102	1
1823	776	1
1823	713	1
1823	3016	1
1823	302	1
1823	305	1
1823	53	1
1823	920	1
1823	280	1
1823	1594	1
1823	2652	1
1823	2013	1
1823	1502	1
1823	703	1
1823	3280	0
1823	2226	0
1823	1095	0
1823	2696	0
1823	2164	0
1823	233	0

1823	2179	0
1823	3086	0
1823	2204	0
1823	339	0
1823	408	0
1823	3610	0
1823	899	0
1823	1236	0
1823	1933	0
1823	802	0
1824	3008	1
1824	1114	1
1824	3782	1
1824	104	1
1824	143	1
1824	51	1
1824	1494	1
1824	154	1
1824	187	1
1824	158	1
1824	159	1
1824	2344	0
1824	2553	0
1824	2393	0
1824	1072	0
1824	3597	0
1824	2461	0
1824	2920	0
1824	1066	0
1824	2617	0

1824	3604	0
1824	2137	0
1825	36	1
1825	708	1
1825	3783	1
1825	105	1
1825	250	1
1825	46	1
1825	3605	1
1825	122	1
1825	476	1
1825	3714	0
1825	3021	0
1825	945	0
1825	39	0
1825	1330	0
1825	3678	0
1825	386	0
1825	347	0
1825	932	0
1826	4	1
1826	6	1
1826	13	1
1826	15	1
1826	1551	1
1826	285	1
1826	545	1
1826	1442	1
1826	1315	1
1826	1699	1

1826	555	1
1826	441	1
1826	446	1
1826	3784	1
1826	334	1
1826	857	1
1826	96	1
1826	871	1
1826	242	1
1826	126	1
1826	2516	0
1826	2340	0
1826	3648	0
1826	2927	0
1826	2441	0
1826	3075	0
1826	2955	0
1826	3744	0
1826	2609	0
1826	3766	0
1826	428	0
1826	1923	0
1826	3774	0
1826	3052	0
1826	1794	0
1826	3213	0
1826	1664	0
1826	2354	0
1826	2084	0
1826	3296	0

1827	3041	1
1827	483	1
1827	196	1
1827	1347	1
1827	200	1
1827	3785	1
1827	893	1
1827	735	1
1827	883	1
1827	20	1
1827	277	1
1827	278	1
1827	729	1
1827	155	1
1827	445	1
1827	479	1
1827	1530	0
1827	2188	0
1827	2061	0
1827	1699	0
1827	3144	0
1827	2919	0
1827	416	0
1827	3438	0
1827	3397	0
1827	1795	0
1827	2831	0
1827	1850	0
1827	673	0
1827	1493	0

1827	733	0
1827	2902	0
1828	1952	1
1828	66	1
1828	1798	1
1828	3786	1
1828	3787	1
1828	2060	1
1828	3788	1
1828	2094	1
1828	879	1
1828	1616	1
1828	3789	1
1828	3790	1
1828	3791	1
1828	2429	1
1828	3792	1
1828	1981	1
1828	3624	0
1828	631	0
1828	2071	0
1828	1231	0
1828	372	0
1828	1591	0
1828	3494	0
1828	1456	0
1828	3256	0
1828	536	0
1828	3282	0
1828	1656	0

1828	98	0
1828	1557	0
1828	724	0
1828	3396	0
1829	197	1
1829	165	1
1829	617	1
1829	48	1
1829	3793	1
1829	3794	1
1829	883	1
1829	2451	1
1829	3795	1
1829	1433	1
1829	3088	0
1829	441	0
1829	3789	0
1829	1376	0
1829	1901	0
1829	567	0
1829	1879	0
1829	1599	0
1829	2624	0
1829	2287	0
1830	867	1
1830	36	1
1830	2665	1
1830	2379	1
1830	590	1
1830	499	1

1830	3796	1
1830	3797	1
1830	30	1
1830	191	1
1830	2270	0
1830	1884	0
1830	2643	0
1830	1352	0
1830	469	0
1830	2234	0
1830	1256	0
1830	3660	0
1830	63	0
1830	934	0
1831	96	1
1831	655	1
1831	1167	1
1831	1745	1
1831	242	1
1831	245	1
1831	726	1
1831	254	1
1831	3798	1
1831	217	1
1831	126	1
1831	53	0
1831	2976	0
1831	2725	0
1831	2194	0
1831	1919	0

1831	3577	0
1831	1392	0
1831	2593	0
1831	2617	0
1831	854	0
1831	1920	0
1832	377	1
1832	1258	1
1832	76	1
1832	206	1
1832	79	1
1832	374	1
1832	3799	1
1832	184	1
1832	185	1
1832	538	1
1832	698	1
1832	815	0
1832	3339	0
1832	3804	0
1832	2138	0
1832	2595	0
1832	1064	0
1832	746	0
1832	2357	0
1832	704	0
1832	1437	0
1832	2501	0
1833	1315	1
1833	100	1

1833	4	1
1833	934	1
1833	8	1
1833	2158	1
1833	3470	1
1833	273	1
1833	498	1
1833	116	1
1833	277	1
1833	3800	1
1833	3801	1
1833	3802	1
1833	3803	1
1833	1725	0
1833	3558	0
1833	2793	0
1833	3378	0
1833	2328	0
1833	3775	0
1833	1378	0
1833	569	0
1833	435	0
1833	924	0
1833	1485	0
1833	875	0
1833	250	0
1833	2498	0
1833	1680	0
1834	1760	1
1834	2976	1

1834	931	1
1834	2787	1
1834	3804	1
1834	2279	1
1834	3805	1
1834	1161	1
1834	555	1
1834	1036	1
1834	1743	1
1834	3379	1
1834	245	1
1834	3388	1
1834	893	1
1834	126	1
1834	3773	0
1834	2887	0
1834	672	0
1834	3633	0
1834	2128	0
1834	1540	0
1834	1628	0
1834	2142	0
1834	3207	0
1834	3818	0
1834	371	0
1834	3771	0
1834	2572	0
1834	909	0
1834	1451	0
1834	615	0

1835	192	1
1835	738	1
1835	102	1
1835	1126	1
1835	235	1
1835	110	1
1835	78	1
1835	1015	1
1835	1171	1
1835	55	1
1835	920	1
1835	282	1
1835	91	1
1835	1153	0
1835	1708	0
1835	596	0
1835	1863	0
1835	2169	0
1835	892	0
1835	2925	0
1835	682	0
1835	2873	0
1835	2229	0
1835	1382	0
1835	824	0
1835	1378	0
1836	770	1
1836	1156	1
1836	261	1
1836	526	1

1836	276	1
1836	404	1
1836	3359	1
1836	673	1
1836	40	1
1836	957	1
1836	192	1
1836	80	1
1836	87	1
1836	91	1
1836	352	1
1836	102	1
1836	237	1
1836	117	1
1836	378	1
1836	127	1
1836	72	0
1836	1750	0
1836	1609	0
1836	3503	0
1836	1869	0
1836	936	0
1836	2229	0
1836	2421	0
1836	986	0
1836	2974	0
1836	284	0
1836	3252	0
1836	328	0
1836	2721	0

1836	2838	0
1836	93	0
1836	2740	0
1836	3555	0
1836	81	0
1836	2005	0
1837	418	1
1837	301	1
1837	77	1
1837	3569	1
1837	22	1
1837	87	1
1837	376	1
1837	127	1
1837	404	0
1837	867	0
1837	1317	0
1837	436	0
1837	3412	0
1837	3045	0
1837	3359	0
1837	1975	0
1838	803	1
1838	324	1
1838	1290	1
1838	655	1
1838	1554	1
1838	211	1
1838	1682	1
1838	1975	1

1838	2170	1
1838	3806	1
1838	3807	1
1838	2132	0
1838	931	0
1838	3321	0
1838	2862	0
1838	2071	0
1838	1188	0
1838	672	0
1838	174	0
1838	2599	0
1838	2254	0
1838	2386	0
1839	3808	1
1839	1857	1
1839	66	1
1839	98	1
1839	135	1
1839	73	1
1839	1452	1
1839	77	1
1839	237	1
1839	79	1
1839	1549	1
1839	1651	1
1839	2071	1
1839	87	1
1839	184	1
1839	377	1

1839	955	1
1839	2570	0
1839	2331	0
1839	2079	0
1839	1436	0
1839	1762	0
1839	3608	0
1839	2779	0
1839	480	0
1839	320	0
1839	2991	0
1839	472	0
1839	2468	0
1839	2513	0
1839	1391	0
1839	1466	0
1839	1200	0
1839	1304	0
1840	832	1
1840	3809	1
1840	292	1
1840	133	1
1840	165	1
1840	389	1
1840	41	1
1840	105	1
1840	1580	1
1840	45	1
1840	2028	1
1840	48	1

1840	1777	1
1840	1395	1
1840	55	1
1840	56	1
1840	442	1
1840	1277	1
1840	603	0
1840	1410	0
1840	2187	0
1840	3586	0
1840	3284	0
1840	1385	0
1840	614	0
1840	1689	0
1840	1634	0
1840	1469	0
1840	2052	0
1840	623	0
1840	3303	0
1840	43	0
1840	371	0
1840	1846	0
1840	2831	0
1840	44	0
1841	96	1
1841	802	1
1841	3810	1
1841	388	1
1841	293	1
1841	772	1

1841	1092	1
1841	137	1
1841	303	1
1841	55	1
1841	118	1
1841	54	1
1841	665	0
1841	810	0
1841	768	0
1841	3579	0
1841	1545	0
1841	2058	0
1841	1521	0
1841	2859	0
1841	317	0
1841	1923	0
1841	428	0
1841	3826	0
1842	96	1
1842	98	1
1842	99	1
1842	130	1
1842	101	1
1842	131	1
1842	3811	1
1842	1421	1
1842	79	1
1842	80	1
1842	1199	1
1842	88	1

1842	185	1
1842	126	1
1842	216	1
1842	3667	0
1842	1911	0
1842	3678	0
1842	2608	0
1842	3055	0
1842	820	0
1842	2070	0
1842	2511	0
1842	1579	0
1842	2943	0
1842	1317	0
1842	2776	0
1842	1709	0
1842	2934	0
1842	2962	0
1843	0	1
1843	655	1
1843	1427	1
1843	3228	1
1843	545	1
1843	1315	1
1843	441	1
1843	444	1
1843	334	1
1843	2255	1
1843	2256	1
1843	2005	1

1843	857	1
1843	1115	1
1843	1118	1
1843	3812	1
1843	3813	1
1843	3814	1
1843	242	1
1843	1275	1
1843	3736	0
1843	2103	0
1843	1120	0
1843	3816	0
1843	3232	0
1843	2716	0
1843	1102	0
1843	2777	0
1843	119	0
1843	1866	0
1843	26	0
1843	2141	0
1843	2677	0
1843	870	0
1843	1716	0
1843	1221	0
1843	3116	0
1843	1398	0
1843	2356	0
1843	700	0
1844	1446	1
1844	3815	1

1844	776	1
1844	3816	1
1844	3817	1
1844	3818	1
1844	589	1
1844	1869	1
1844	212	1
1844	1076	1
1844	214	1
1844	1690	1
1844	827	1
1844	2013	1
1844	3355	0
1844	655	0
1844	3401	0
1844	3698	0
1844	3439	0
1844	2737	0
1844	2271	0
1844	2079	0
1844	1867	0
1844	1694	0
1844	2221	0
1844	2560	0
1844	78	0
1844	128	0
1845	132	1
1845	3217	1
1845	406	1
1845	1047	1

1845	539	1
1845	798	1
1845	167	1
1845	2485	1
1845	951	1
1845	55	1
1845	1919	1
1845	66	1
1845	98	1
1845	1143	1
1845	253	1
1845	126	1
1845	893	1
1845	254	1
1845	2175	1
1845	1397	0
1845	232	0
1845	2416	0
1845	2326	0
1845	1149	0
1845	2283	0
1845	3586	0
1845	804	0
1845	1922	0
1845	483	0
1845	3833	0
1845	3139	0
1845	245	0
1845	958	0
1845	884	0

1845	2317	0
1845	1300	0
1845	3479	0
1845	3648	0
1846	832	1
1846	1538	1
1846	1315	1
1846	235	1
1846	3819	1
1846	46	1
1846	47	1
1846	114	1
1846	754	1
1846	1556	1
1846	118	1
1846	694	1
1846	1590	1
1846	282	1
1846	91	1
1846	830	1
1846	3551	1
1846	156	0
1846	1426	0
1846	2372	0
1846	1323	0
1846	223	0
1846	241	0
1846	208	0
1846	982	0
1846	1206	0

1846	3656	0
1846	1192	0
1846	1364	0
1846	2180	0
1846	3761	0
1846	2262	0
1846	262	0
1846	345	0
1847	96	1
1847	1156	1
1847	134	1
1847	102	1
1847	1261	1
1847	3569	1
1847	210	1
1847	52	1
1847	118	1
1847	376	1
1847	2243	0
1847	3294	0
1847	2170	0
1847	3190	0
1847	1562	0
1847	3609	0
1847	3029	0
1847	3512	0
1847	98	0
1847	860	0
1848	66	1
1848	132	1

1848	552	1
1848	74	1
1848	204	1
1848	365	1
1848	3820	1
1848	3821	1
1848	660	1
1848	185	1
1848	127	1
1848	2990	0
1848	3271	0
1848	931	0
1848	2103	0
1848	1612	0
1848	2238	0
1848	1723	0
1848	3028	0
1848	3416	0
1848	2483	0
1848	2820	0
1849	98	1
1849	263	1
1849	2696	1
1849	270	1
1849	79	1
1849	3822	1
1849	1395	1
1849	951	1
1849	473	1
1849	960	0

1849	3595	0
1849	3731	0
1849	3830	0
1849	1863	0
1849	995	0
1849	742	0
1849	1645	0
1849	2198	0
1850	132	1
1850	133	1
1850	237	1
1850	910	1
1850	975	1
1850	3823	1
1850	499	1
1850	22	1
1850	445	1
1850	2514	0
1850	3536	0
1850	653	0
1850	1744	0
1850	2465	0
1850	3272	0
1850	835	0
1850	1277	0
1850	433	0
1851	1315	1
1851	134	1
1851	46	1
1851	47	1

1851	1015	1
1851	1395	1
1851	1461	1
1851	87	1
1851	2427	1
1851	254	1
1851	863	1
1851	1048	0
1851	3725	0
1851	2773	0
1851	2360	0
1851	2852	0
1851	1435	0
1851	1616	0
1851	2883	0
1851	2431	0
1851	3022	0
1851	2377	0
1852	1315	1
1852	36	1
1852	446	1
1852	1092	1
1852	47	1
1852	754	1
1852	52	1
1852	118	1
1852	254	1
1852	1765	0
1852	3805	0
1852	3406	0

1852	2954	0
1852	753	0
1852	1114	0
1852	301	0
1852	3819	0
1852	3467	0
1853	2976	1
1853	325	1
1853	774	1
1853	1798	1
1853	40	1
1853	522	1
1853	3313	1
1853	188	1
1853	52	1
1853	252	1
1853	119	1
1853	856	1
1853	1401	1
1853	124	1
1853	1127	0
1853	3444	0
1853	2487	0
1853	2607	0
1853	2520	0
1853	249	0
1853	2521	0
1853	1510	0
1853	838	0
1853	2507	0

1853	2125	0
1853	1615	0
1853	1006	0
1853	79	0
1854	900	1
1854	377	1
1854	809	1
1854	78	1
1854	270	1
1854	3824	1
1854	305	1
1854	115	1
1854	1556	1
1854	1589	1
1854	185	1
1854	282	1
1854	671	1
1854	13	0
1854	3375	0
1854	2278	0
1854	3448	0
1854	1007	0
1854	1938	0
1854	1365	0
1854	2520	0
1854	595	0
1854	1153	0
1854	3237	0
1854	2062	0
1854	3804	0

1855	257	1
1855	227	1
1855	196	1
1855	485	1
1855	1476	1
1855	487	1
1855	3630	1
1855	3825	1
1855	1618	1
1855	275	1
1855	22	1
1855	887	1
1855	1526	1
1855	155	1
1855	156	1
1855	445	1
1855	735	1
1855	2552	0
1855	418	0
1855	1615	0
1855	2419	0
1855	2823	0
1855	3246	0
1855	1167	0
1855	2068	0
1855	2340	0
1855	3636	0
1855	3562	0
1855	2627	0
1855	2301	0

1855	2320	0
1855	3391	0
1855	753	0
1855	3090	0
1856	712	1
1856	106	1
1856	2058	1
1856	45	1
1856	495	1
1856	1393	1
1856	55	1
1856	56	1
1856	154	1
1856	413	1
1856	3151	0
1856	1916	0
1856	469	0
1856	2675	0
1856	952	0
1856	1984	0
1856	249	0
1856	1363	0
1856	3167	0
1856	247	0
1857	1540	1
1857	2661	1
1857	141	1
1857	271	1
1857	275	1
1857	499	1

1857	1140	1
1857	502	1
1857	441	1
1857	506	0
1857	219	0
1857	1044	0
1857	2397	0
1857	720	0
1857	2614	0
1857	58	0
1857	2805	0
1857	2545	0
1858	1183	1
1858	391	1
1858	392	1
1858	105	1
1858	46	1
1858	55	1
1858	863	1
1858	2479	0
1858	913	0
1858	609	0
1858	3595	0
1858	1641	0
1858	2823	0
1858	2187	0
1859	516	1
1859	295	1
1859	1095	1
1859	523	1

1859	107	1
1859	2927	1
1859	3826	1
1859	52	1
1859	765	1
1859	1170	0
1859	642	0
1859	1120	0
1859	2628	0
1859	975	0
1859	562	0
1859	2689	0
1859	3573	0
1859	422	0
1860	96	1
1860	66	1
1860	135	1
1860	1258	1
1860	47	1
1860	81	1
1860	118	1
1860	374	1
1860	1277	1
1860	846	0
1860	619	0
1860	2278	0
1860	3412	0
1860	917	0
1860	615	0
1860	2095	0

1860	3530	0
1860	185	0
1861	73	1
1861	1612	1
1861	77	1
1861	46	1
1861	141	1
1861	1232	1
1861	81	1
1861	1202	1
1861	87	1
1861	55	1
1861	3529	0
1861	3684	0
1861	3129	0
1861	3406	0
1861	1779	0
1861	585	0
1861	1302	0
1861	2185	0
1861	325	0
1861	3481	0
1862	225	1
1862	3201	1
1862	228	1
1862	677	1
1862	1444	1
1862	1992	1
1862	654	1
1862	944	1

1862	657	1
1862	306	1
1862	2975	1
1862	223	1
1862	984	0
1862	494	0
1862	2621	0
1862	1718	0
1862	1899	0
1862	3313	0
1862	1297	0
1862	6	0
1862	1878	0
1862	1314	0
1862	1247	0
1862	3827	0
1863	1955	1
1863	1956	1
1863	3428	1
1863	358	1
1863	998	1
1863	3528	1
1863	1322	1
1863	590	1
1863	366	1
1863	1487	1
1863	3827	1
1863	2293	1
1863	3453	1
1863	254	1

1863	119	0
1863	911	0
1863	2708	0
1863	1459	0
1863	3093	0
1863	1920	0
1863	2709	0
1863	2682	0
1863	879	0
1863	1026	0
1863	2371	0
1863	2072	0
1863	1710	0
1863	2765	0
1864	388	1
1864	391	1
1864	105	1
1864	109	1
1864	45	1
1864	46	1
1864	878	1
1864	1555	1
1864	53	1
1864	159	1
1864	2351	0
1864	217	0
1864	3021	0
1864	433	0
1864	1709	0
1864	482	0

1864	2782	0
1864	2046	0
1864	2721	0
1864	1707	0
1865	2429	1
1865	1947	1
1865	1140	1
1865	141 1	
1865	236 0	
1865	595 0	
1865	2585	0
1865	3193	0
1866	832 1	
1866	1441	1
1866	2210	1
1866	133 1	
1866	3831	1
1866	980 1	
1866	3828	1
1866	3829	1
1866	3830	1
1866	377 1	
1866	2076	1
1866	1439	1
1866	1532	0
1866	1566	0
1866	447 0	
1866	3241	0
1866	2500	0
1866	1165	0

1866	333 0	
1866	1001	0
1866	1050	0
1866	2331	0
1866	2798	0
1866	2288	0
1867	3714	1
1867	644 1	
1867	3834	1
1867	1291	1
1867	3832	1
1867	3833	1
1867	632 1	
1867	1721	1
1867	2234	1
1867	3835	1
1867	2686	1
1867	2847	1
1867	1730	0
1867	348 0	
1867	2415	0
1867	1037	0
1867	3356	0
1867	3199	0
1867	3255	0
1867	1418	0
1867	2990	0
1867	2648	0
1867	1350	0
1867	1448	0

1868	224	1
1868	1536	1
1868	354	1
1868	1664	1
1868	36	1
1868	197	1
1868	388	1
1868	1891	1
1868	3040	1
1868	109	1
1868	1103	1
1868	52	1
1868	57	1
1868	891	1
1868	956	1
1868	221	1
1868	1843	0
1868	1712	0
1868	3152	0
1868	2869	0
1868	461	0
1868	1165	0
1868	1916	0
1868	2636	0
1868	3499	0
1868	3808	0
1868	776	0
1868	871	0
1868	1405	0
1868	3328	0

1868	1631	0
1868	3447	0
1869	1347	1
1869	36	1
1869	293	1
1869	356	1
1869	388	1
1869	708	1
1869	105	1
1869	1092	1
1869	107	1
1869	46	1
1869	1328	1
1869	54	1
1869	55	1
1869	572	1
1869	765	1
1869	2910	1
1869	1955	0
1869	1345	0
1869	2625	0
1869	2474	0
1869	582	0
1869	2616	0
1869	2934	0
1869	1801	0
1869	464	0
1869	3278	0
1869	3227	0
1869	280	0

1869	358	0
1869	1075	0
1869	2087	0
1869	856	0
1870	3840	1
1870	509	1
1870	3841	1
1870	519	1
1870	1034	1
1870	908	1
1870	2617	1
1870	196	1
1870	844	1
1870	727	1
1870	100	1
1870	486	1
1870	1513	1
1870	3837	1
1870	508	1
1870	763	1
1870	3836	1
1870	3709	1
1870	3838	1
1870	3839	1
1870	2317	0
1870	2887	0
1870	3454	0
1870	630	0
1870	2579	0
1870	1019	0

1870	2965	0
1870	1528	0
1870	2171	0
1870	1892	0
1870	1569	0
1870	2125	0
1870	1838	0
1870	412	0
1870	864	0
1870	3190	0
1870	2464	0
1870	1976	0
1870	1825	0
1870	772	0
1871	1216	1
1871	1	1
1871	289	1
1871	3	1
1871	2532	1
1871	741	1
1871	1029	1
1871	1990	1
1871	1991	1
1871	1289	1
1871	1641	1
1871	3494	1
1871	12	1
1871	1906	1
1871	217	1
1871	284	1

1871	31	0
1871	3363	0
1871	2085	0
1871	3252	0
1871	355	0
1871	620	0
1871	1036	0
1871	983	0
1871	3114	0
1871	2844	0
1871	2805	0
1871	3057	0
1871	1630	0
1871	1394	0
1871	1410	0
1871	2122	0
