# Supplementary material for: Music recommendation algorithms based on knowledge graph and multi-task feature learning
Source: Sci Rep. 2024 Jan 24;14:2055. doi: 10.1038/s41598-024-52463-z (PMC10808181; doi:10.1038/s41598-024-52463-z)
Supplement: Supplementary file 4 — Supplementary Information 4. [file 41598_2024_52463_MOESM4_ESM.pdf]

userID	artistID	weight
2	51	13883
2	52	11690
2	53	11351
2	54	10300
2	55	8983
2	56	6152
2	57	5955
2	58	4616
2	59	4337
2	60	4147
2	61	3923
2	62	3782
2	63	3735
2	64	3644
2	65	3579
2	66	3312
2	67	3301
2	68	2927
2	69	2720
2	70	2686
2	71	2654
2	72	2619
2	73	2584
2	74	2547
2	75	2397
2	76	2382
2	77	2120
2	78	2119
2	79	1990

2	80	1972
2	81	1948
2	82	1868
2	83	1792
2	84	1740
2	85	1638
2	86	1594
2	87	1559
2	88	1553
2	89	1519
2	90	1471
2	91	1438
2	92	1411
2	93	1407
2	94	1373
2	95	1363
2	96	1342
2	97	1337
2	98	1332
2	99	1330
2	100	1315
3	101	13176
3	102	662
3	103	493
3	104	431
3	105	403
3	106	354
3	107	269
3	108	236
3	109	215

3	110	215
3	111	212
3	112	193
3	113	187
3	114	167
3	115	163
3	116	154
3	117	151
3	118	142
3	119	142
3	120	125
3	121	111
3	122	108
3	123	104
3	124	99
3	125	98
3	126	94
3	127	89
3	128	89
3	129	86
3	130	85
3	131	84
3	132	83
3	133	83
3	134	77
3	135	77
3	136	76
3	137	75
3	138	72
3	139	72

3	140	71
3	141	70
3	142	70
3	143	70
3	144	69
3	145	68
3	146	67
3	147	67
3	148	66
3	149	66
3	150	65
4	51	228
4	53	686
4	64	420
4	65	301
4	70	389
4	72	4983
4	77	396
4	151	1807
4	152	1208
4	153	903
4	154	826
4	155	777
4	156	743
4	157	732
4	158	729
4	159	724
4	160	712
4	161	632
4	162	524

4	163 465
4	164 455
4	165 446
4	166 445
4	167 407
4	168 404
4	169 384
4	170 366
4	171 359
4	172 309
4	173 292
4	174 287
4	175 285
4	176 284
4	177 284
4	178 279
4	179 264
4	180 256
4	181 255
4	182 254
4	183 253
4	184 247
4	185 240
4	186 236
4	187 223
4	188 221
4	189 210
4	190 208
4	191 202
4	192 197

4	193	193
5	53	181
5	154	884
5	173	241
5	182	222
5	190	826
5	194	777
5	195	673
5	196	620
5	197	452
5	198	451
5	199	374
5	200	337
5	201	304
5	202	298
5	203	287
5	204	283
5	205	275
5	206	265
5	207	259
5	208	255
5	209	252
5	210	229
5	211	222
5	212	210
5	213	209
5	214	207
5	215	196
5	216	192
5	217	185

5	218	184
5	219	178
5	220	170
5	221	161
5	222	161
5	223	149
5	224	143
5	225	142
5	226	139
5	227	138
5	228	138
5	229	138
5	230	137
5	231	134
5	232	133
5	233	130
5	234	129
5	235	129
5	236	127
5	237	118
5	238	115
6	77	16
6	239	41
6	240	41
6	241	40
6	242	37
6	243	34
6	244	33
6	245	31
6	246	31

6	247	27
6	248	26
6	249	24
6	250	24
6	251	24
6	252	24
6	253	23
6	254	20
6	255	20
6	256	19
6	257	18
6	258	18
6	259	18
6	260	18
6	261	18
6	262	17
6	263	17
6	264	17
6	265	17
6	266	16
6	267	16
6	268	16
6	269	16
6	270	16
6	271	15
6	272	15
6	273	15
6	274	15
6	275	14
6	276	14

6	277	14
6	278	14
6	279	14
6	280	14
6	281	14
6	282	14
6	283	14
6	284	13
6	285	13
6	286	13
6	287	13
7	55	3151
7	67	2540
7	89	5846
7	257	5188
7	288	43864
7	289	11112
7	290	6266
7	291	5379
7	292	4201
7	293	4059
7	294	3251
7	295	2852
7	296	2356
7	297	1848
7	298	1802
7	299	1780
7	300	1747
7	301	1553
7	302	1490

7 303 1426

7 304 1312

7 305 1169

7 306 1164

7 307 1162

7 308 1082

7 309 1027

7 310 962

7 311 960

7 312 926

7 313 900

7 314 868

7 315 834

7 316 809

7 317 773

7 318 753

7 319 741

7 320 719

7 321 674

7 322 667

7 323 646

7 324 605

7 325 599

7 326 593

7 327 587

7 328 571

7 329 534

7 330 527

7 331 516

7 332 510

7	333	498
8	71	181
8	89	921
8	97	354
8	251	235
8	257	479
8	265	581
8	288	428
8	289	2258
8	291	402
8	292	783
8	295	1425
8	296	1039
8	298	613
8	300	347
8	302	432
8	311	353
8	323	578
8	334	6291
8	335	2396
8	336	1735
8	337	1407
8	338	963
8	339	819
8	340	800
8	341	794
8	342	753
8	343	588
8	344	553
8	345	527

8	346	520
8	347	505
8	348	504
8	349	494
8	350	494
8	351	456
8	352	417
8	353	400
8	354	389
8	355	361
8	356	350
8	357	345
8	358	280
8	359	277
8	360	257
8	361	253
8	362	229
8	363	185
8	364	178
8	365	177
8	366	173
9	8	587
9	367	2396
9	368	1444
9	369	1158
9	370	1113
9	371	1103
9	372	1069
9	373	729
9	374	708

9	375 630
9	376 629
9	377 609
9	378 590
9	379 558
9	380 556
9	381 556
9	382 533
9	383 521
9	384 510
9	385 467
9	386 465
9	387 430
9	388 411
9	389 403
9	390 381
9	391 367
9	392 364
9	393 357
9	394 345
9	395 319
9	396 307
9	397 307
9	398 306
9	399 292
9	400 290
9	401 289
9	402 280
9	403 273
9	404 273

9 405 267

9 406 260

9 407 249

9 408 249

9 409 242

9 410 241

9 411 237

9 412 236

9 413 234

9 414 229

9 415 228

10 154 177

10 159 225

10 196 1137

10 199 1386

10 200 306

10 208 310

10 217 761

10 219 189

10 225 1297

10 228 279

10 229 992

10 416 3132

10 417 1584

10 418 1056

10 419 1029

10 420 1023

10 421 956

10 422 878

10 423 845

10	424 797
10	425 729
10	426 603
10	427 583
10	428 578
10	429 573
10	430 560
10	431 546
10	432 527
10	433 483
10	434 455
10	435 367
10	436 317
10	437 315
10	438 308
10	439 291
10	440 247
10	441 238
10	442 235
10	443 229
10	444 208
10	445 204
10	446 199
10	447 189
10	448 178
10	449 177
10	450 173
10	451 169
10	452 165
10	453 161

10	454	157
11	67	229
11	89	3384
11	157	1007
11	230	329
11	234	370
11	288	749
11	289	3851
11	292	555
11	294	264
11	295	521
11	300	347
11	301	306
11	304	1031
11	306	249
11	325	298
11	327	337
11	329	553
11	344	243
11	349	341
11	455	4416
11	456	1951
11	457	1788
11	458	1708
11	459	1567
11	460	1531
11	461	1500
11	462	1318
11	463	1235
11	464	1117

11	465	1006
11	466	956
11	467	793
11	468	719
11	469	552
11	470	548
11	471	537
11	472	514
11	473	441
11	474	425
11	475	424
11	476	337
11	477	336
11	478	329
11	479	321
11	480	314
11	481	293
11	482	251
11	483	247
11	484	244
11	485	238
12	72	2144
12	189	2976
12	191	4259
12	230	2110
12	288	3072
12	300	1064
12	333	23961
12	344	5489
12	377	59695

12	378	31617
12	405	1034
12	407	1600
12	466	1485
12	486	24097
12	487	7867
12	488	7425
12	489	7399
12	490	4527
12	491	3837
12	492	3823
12	493	3727
12	494	3648
12	495	3526
12	496	3407
12	497	3226
12	498	3134
12	499	3080
12	500	2471
12	501	2378
12	502	2303
12	503	1714
12	504	1646
12	505	1596
12	506	1591
12	507	1547
12	508	1495
12	509	1434
12	510	1341
12	511	1278

12	512	1260
12	513	1250
12	514	1227
12	515	1222
12	516	1183
12	517	1177
12	518	1001
12	519	982
12	520	953
12	521	939
12	522	927
13	55	6
13	67	9
13	89	3967
13	173	6
13	228	6
13	292	4
13	295	2
13	298	16
13	299	4
13	300	2
13	302	9
13	310	17
13	316	4
13	318	2
13	325	3
13	328	12
13	333	38
13	344	2
13	349	3

13	352	2
13	378	2
13	461	3
13	466	4
13	498	6
13	523	10
13	524	8
13	525	6
13	526	6
13	527	4
13	528	4
13	529	3
13	530	3
13	531	3
13	532	3
13	533	2
13	534	2
13	535	2
13	536	2
13	537	2
13	538	2
13	539	2
13	540	2
13	541	2
13	542	2
13	543	2
13	544	2
13	545	2
13	546	2
13	547	2

13 548 2

14 549 1259

14 550 908

14 551 850

14 552 849

14 553 843

14 554 834

14 555 831

14 556 812

14 557 809

14 558 772

14 559 768

14 560 765

14 561 724

14 562 710

14 563 706

14 564 676

14 565 674

14 566 671

14 567 670

14 568 665

14 569 656

14 570 655

14 571 654

14 572 653

14 573 653

14 574 650

14 575 648

14 576 646

14 577 645

14	578	638
14	579	635
14	580	634
14	581	634
14	582	633
14	583	632
14	584	632
14	585	631
14	586	627
14	587	621
14	588	619
14	589	619
14	590	618
14	591	612
14	592	610
14	593	610
14	594	609
14	595	607
14	596	602
14	597	601
14	598	601
15	69	242
15	154	751
15	159	297
15	197	599
15	203	352
15	205	477
15	212	242
15	215	253
15	218	246

15	222	235
15	227	747
15	233	464
15	266	247
15	429	366
15	511	740
15	599	1141
15	600	835
15	601	557
15	602	546
15	603	484
15	604	484
15	605	470
15	606	466
15	607	443
15	608	412
15	609	407
15	610	404
15	611	404
15	612	380
15	613	373
15	614	351
15	615	350
15	616	346
15	617	341
15	618	340
15	619	337
15	620	328
15	621	306
15	622	302

15	623	295
15	624	294
15	625	279
15	626	261
15	627	258
15	628	255
15	629	251
15	630	230
15	631	226
15	632	226
15	633	223
16	89	849
16	161	459
16	346	264
16	386	154
16	532	251
16	634	889
16	635	750
16	636	617
16	637	502
16	638	487
16	639	457
16	640	392
16	641	391
16	642	389
16	643	367
16	644	366
16	645	311
16	646	308
16	647	307

16	648	292
16	649	257
16	650	251
16	651	250
16	652	231
16	653	228
16	654	217
16	655	217
16	656	214
16	657	209
16	658	195
16	659	190
16	660	174
16	661	167
16	662	166
16	663	165
16	664	153
16	665	150
16	666	149
16	667	148
16	668	147
16	669	147
16	670	146
16	671	144
16	672	144
16	673	143
16	674	141
16	675	141
16	676	139
16	677	139

16 678 137
17 56 242
17 67 303
17 89 1255
17 229 610
17 230 120
17 288 129
17 289 118
17 292 110
17 298 215
17 300 1089
17 306 144
17 324 306
17 325 219
17 344 1804
17 403 101
17 461 984
17 464 266
17 466 925
17 498 1544
17 526 104
17 528 321
17 538 188
17 544 426
17 548 1446
17 679 14436
17 680 891
17 681 879
17 682 651
17 683 474

17	684	462
17	685	381
17	686	373
17	687	370
17	688	338
17	689	270
17	690	243
17	691	208
17	692	190
17	693	188
17	694	187
17	695	172
17	696	170
17	697	168
17	698	146
17	699	121
17	700	116
17	701	115
17	702	109
17	703	107
17	704	105
18	56	186
18	65	234
18	71	401
18	72	404
18	88	258
18	163	230
18	174	332
18	190	544
18	220	623

18 227 630

18 230 400

18 378 204

18 389 393

18 511 496

18 533 576

18 705 2187

18 706 853

18 707 838

18 708 682

18 709 660

18 710 646

18 711 603

18 712 518

18 713 500

18 714 427

18 715 426

18 716 409

18 717 363

18 718 359

18 719 359

18 720 318

18 721 298

18 722 283

18 723 277

18 724 246

18 725 245

18 726 241

18 727 240

18 728 236

18	729	232
18	730	222
18	731	219
18	732	216
18	733	215
18	734	197
18	735	192
18	736	181
18	737	171
18	738	171
18	739	170
20	56	108
20	88	111
20	141	987
20	163	119
20	201	118
20	504	154
20	512	290
20	743	483
20	744	375
20	745	304
20	746	285
20	747	274
20	748	268
20	749	261
20	750	245
20	751	242
20	752	239
20	753	202
20	754	185

20 755 174

20 756 171

20 757 167

20 758 162

20 759 160

20 760 156

20 761 149

20 762 141

20 763 137

20 764 133

20 765 130

20 766 129

20 767 124

20 768 122

20 769 120

20 770 116

20 771 113

20 772 108

20 773 108

20 774 103

20 775 102

20 776 100

20 777 99

20 778 99

20 779 99

20 780 98

20 781 98

20 782 97

20 783 96

20 784 96

20	785	95
21	55	4097
21	67	219
21	71	110
21	89	406
21	288	474
21	289	4620
21	292	437
21	293	599
21	295	152
21	298	149
21	299	516
21	300	252
21	302	118
21	306	138
21	310	187
21	318	215
21	325	202
21	352	116
21	461	436
21	465	122
21	466	222
21	476	432
21	523	186
21	531	188
21	535	1953
21	543	210
21	660	180
21	674	220
21	679	159

21	681 118
21	686 764
21	701 732
21	786 734
21	787 426
21	788 424
21	789 400
21	790 369
21	791 271
21	792 263
21	793 228
21	794 215
21	795 183
21	796 178
21	797 164
21	798 145
21	799 127
21	800 120
21	801 120
21	802 99
21	803 98
22	412 677
22	503 6257
22	514 939
22	804 3804
22	805 2548
22	806 2501
22	807 2352
22	808 2334
22	809 2301

22	810	2160
22	811	2153
22	812	1833
22	813	1742
22	814	1627
22	815	1451
22	816	1401
22	817	1284
22	818	1190
22	819	1190
22	820	1158
22	821	1044
22	822	1032
22	823	999
22	824	945
22	825	902
22	826	866
22	827	858
22	828	770
22	829	759
22	830	743
22	831	731
22	832	702
22	833	685
22	834	684
22	835	676
22	836	635
22	837	634
22	838	627
22	839	615

22	840	609
22	841	602
22	842	560
22	843	553
22	844	543
22	845	539
22	846	538
22	847	534
22	848	529
22	849	523
22	850	505
23	7	212
23	72	1752
23	152	330
23	159	401
23	163	124
23	181	297
23	220	112
23	226	292
23	227	143
23	233	379
23	420	117
23	436	242
23	511	752
23	599	652
23	615	131
23	707	176
23	851	2897
23	852	956
23	853	772

23	854 508
23	855 419
23	856 240
23	857 238
23	858 235
23	859 226
23	860 217
23	861 215
23	862 210
23	863 200
23	864 193
23	865 192
23	866 190
23	867 189
23	868 186
23	869 171
23	870 168
23	871 154
23	872 153
23	873 150
23	874 142
23	875 138
23	876 124
23	877 120
23	878 119
23	879 118
23	880 118
23	881 116
23	882 115
23	883 110

23	884	110
24	55	5956
24	67	6917
24	89	661
24	157	538
24	167	1412
24	229	2443
24	257	1203
24	258	531
24	285	586
24	288	677
24	289	1192
24	291	516
24	295	506
24	298	1226
24	300	1729
24	301	432
24	302	1037
24	310	677
24	316	1998
24	333	466
24	340	720
24	425	851
24	437	796
24	526	550
24	689	1720
24	690	475
24	709	875
24	786	1694
24	789	4466

24	885	2776
24	886	1999
24	887	1484
24	888	1114
24	889	1025
24	890	833
24	891	776
24	892	742
24	893	677
24	894	675
24	895	662
24	896	633
24	897	606
24	898	546
24	899	538
24	900	518
24	901	518
24	902	517
24	903	477
24	904	462
24	905	416
25	55	185
25	67	278
25	89	311
25	155	235
25	157	1694
25	288	208
25	289	1623
25	290	95
25	292	404

25	293	81
25	294	141
25	295	312
25	296	1765
25	302	120
25	308	205
25	309	239
25	310	77
25	311	76
25	314	160
25	318	431
25	325	244
25	328	191
25	329	425
25	331	153
25	349	231
25	350	1146
25	352	207
25	354	747
25	455	3009
25	459	96
25	462	82
25	469	355
25	476	1759
25	484	177
25	531	186
25	543	96
25	701	130
25	786	593
25	800	194

25	906	3004
25	907	271
25	908	222
25	909	173
25	910	150
25	911	133
25	912	97
25	913	89
25	914	81
25	915	80
25	916	78
26	6	550
26	25	177
26	511	1813
26	707	278
26	718	250
26	841	151
26	917	3967
26	918	2199
26	919	1818
26	920	1622
26	921	1356
26	922	1223
26	923	1176
26	924	1140
26	925	1088
26	926	812
26	927	794
26	928	715
26	929	682

26	930 633
26	931 522
26	932 503
26	933 493
26	934 433
26	935 433
26	936 376
26	937 334
26	938 328
26	939 316
26	940 314
26	941 313
26	942 284
26	943 279
26	944 269
26	945 264
26	946 263
26	947 247
26	948 226
26	949 218
26	950 205
26	951 198
26	952 194
26	953 184
26	954 175
26	955 170
26	956 165
26	957 142
26	958 127
26	959 123

26	960	115
27	51	85
27	67	117
27	152	200
27	172	106
27	183	156
27	328	86
27	348	86
27	423	408
27	463	159
27	548	144
27	614	102
27	641	89
27	644	71
27	689	80
27	701	214
27	703	226
27	726	153
27	861	72
27	917	81
27	961	226
27	962	198
27	963	184
27	964	172
27	965	157
27	966	137
27	967	131
27	968	114
27	969	108
27	970	103

27	971	102
27	972	100
27	973	99
27	974	97
27	975	96
27	976	92
27	977	81
27	978	73
27	979	72
27	980	70
27	981	67
27	982	66
27	983	66
27	984	65
27	985	64
27	986	62
27	987	62
27	988	61
27	989	60
27	990	60
27	991	58
28	51	10
28	59	8
28	61	8
28	72	8
28	85	14
28	91	11
28	100	14
28	193	27
28	366	10

28	905	7
28	992	18
28	993	16
28	994	15
28	995	15
28	996	14
28	997	13
28	998	13
28	999	12
28	1000	12
28	1001	11
28	1002	11
28	1003	11
28	1004	10
28	1005	10
28	1006	9
28	1007	9
28	1008	9
28	1009	9
28	1010	9
28	1011	9
28	1012	8
28	1013	8
28	1014	8
28	1015	8
28	1016	8
28	1017	8
28	1018	8
28	1019	7
28	1020	7

28	1021	7
28	1022	7
28	1023	7
28	1024	7
28	1025	7
28	1026	7
28	1027	7
28	1028	7
28	1029	7
28	1030	7
28	1031	7
29	55	404
29	89	153
29	161	30
29	209	23
29	288	42
29	289	79
29	292	34
29	300	68
29	302	121
29	317	33
29	318	24
29	328	62
29	333	337
29	378	27
29	455	26
29	461	40
29	464	32
29	466	501
29	472	63

29	481	57
29	498	445
29	500	56
29	528	131
29	531	32
29	534	23
29	537	201
29	543	53
29	679	293
29	686	144
29	687	2000
29	704	253
29	706	33
29	908	29
29	951	59
29	966	28
29	1032	300
29	1033	163
29	1034	150
29	1035	134
29	1036	106
29	1037	85
29	1038	77
29	1039	68
29	1040	63
29	1041	51
29	1042	49
29	1043	48
29	1044	42
29	1045	32

29 1046 23
30 89 648
30 190 120
30 228 209
30 229 152
30 230 259
30 234 165
30 288 203
30 289 1231
30 291 102
30 295 184
30 300 512
30 304 235
30 315 418
30 318 305
30 321 671
30 325 131
30 333 522
30 344 148
30 355 84
30 455 105
30 456 130
30 461 1375
30 465 184
30 471 89
30 481 192
30 498 1604
30 521 560
30 538 95
30 646 110

30	679	321
30	680	202
30	687	131
30	793	565
30	908	90
30	951	119
30	966	78
30	1043	255
30	1047	1725
30	1048	1433
30	1049	267
30	1050	212
30	1051	166
30	1052	158
30	1053	136
30	1054	136
30	1055	127
30	1056	109
30	1057	103
30	1058	81
30	1059	77
31	30	150
31	55	147
31	56	269
31	59	131
31	67	39
31	72	17561
31	77	99
31	159	48
31	227	126

31	238	108
31	289	395
31	515	36
31	534	266
31	603	56
31	757	178
31	875	193
31	880	440
31	959	68
31	972	463
31	1001	1360
31	1028	152
31	1060	887
31	1061	876
31	1062	522
31	1063	439
31	1064	422
31	1065	331
31	1066	262
31	1067	251
31	1068	188
31	1069	182
31	1070	182
31	1071	108
31	1072	93
31	1073	90
31	1074	88
31	1075	88
31	1076	69
31	1077	68

31	1078	67
31	1079	64
31	1080	63
31	1081	58
31	1082	58
31	1083	55
31	1084	52
31	1085	50
31	1086	49
31	1087	47
31	1088	40
32	72	236
32	88	859
32	154	435
32	155	261
32	159	307
32	170	410
32	173	5023
32	190	1837
32	196	143
32	200	246
32	203	3554
32	217	119
32	220	327
32	227	520
32	230	162
32	234	289
32	377	169
32	418	244
32	428	388

32	429	848
32	441	1180
32	447	329
32	493	208
32	533	278
32	599	194
32	620	216
32	808	123
32	851	5364
32	857	260
32	875	268
32	1089	2185
32	1090	1352
32	1091	659
32	1092	442
32	1093	431
32	1094	389
32	1095	310
32	1096	288
32	1097	287
32	1098	223
32	1099	221
32	1100	211
32	1101	201
32	1102	180
32	1103	158
32	1104	157
32	1105	144
32	1106	138
32	1107	128

32	1108	118
33	56	1129
33	152	940
33	183	637
33	188	1383
33	233	1535
33	418	1130
33	607	315
33	614	1113
33	632	318
33	964	759
33	968	354
33	977	374
33	1098	964
33	1109	3338
33	1110	3248
33	1111	2350
33	1112	2107
33	1113	1493
33	1114	1482
33	1115	1385
33	1116	1363
33	1117	1017
33	1118	996
33	1119	874
33	1120	858
33	1121	770
33	1122	689
33	1123	683
33	1124	681

33	1125	584
33	1126	576
33	1127	545
33	1128	467
33	1129	461
33	1130	438
33	1131	433
33	1132	386
33	1133	356
33	1134	347
33	1135	305
33	1136	296
33	1137	287
33	1138	246
33	1139	226
33	1140	219
33	1141	209
33	1142	209
33	1143	200
33	1144	196
33	1145	189
34	1	212
34	53	211
34	59	1455
34	70	341
34	72	1881
34	163	1416
34	173	775
34	196	578
34	199	1551

34	220	394
34	225	1075
34	226	366
34	227	908
34	229	235
34	390	2952
34	397	394
34	416	3801
34	418	454
34	430	185
34	441	2261
34	511	246
34	599	1387
34	622	412
34	717	1797
34	851	533
34	959	384
34	1109	208
34	1146	2101
34	1147	1761
34	1148	962
34	1149	771
34	1150	746
34	1151	658
34	1152	497
34	1153	490
34	1154	399
34	1155	354
34	1156	306
34	1157	297

34	1158	292
34	1159	285
34	1160	268
34	1161	265
34	1162	208
34	1163	201
34	1164	194
34	1165	194
34	1166	189
34	1167	182
34	1168	180
35	186 1	
35	212 4	
35	444 1	
35	920 1	
35	964 1	
35	1169	3
35	1170	2
35	1171	1
35	1172	1
35	1173	1
35	1174	1
35	1175	1
35	1176	1
35	1177	1
35	1178	1
36	45 321	
36	198 274	
36	209 262	
36	230 254	

36	333	318
36	430	448
36	464	292
36	470	345
36	481	308
36	488	358
36	493	611
36	497	782
36	500	497
36	537	258
36	540	629
36	687	1524
36	768	1417
36	779	709
36	805	952
36	808	1089
36	816	1420
36	966	358
36	1035	341
36	1043	305
36	1046	947
36	1047	1080
36	1091	361
36	1179	1126
36	1180	900
36	1181	765
36	1182	739
36	1183	718
36	1184	688
36	1185	675

36	1186	569
36	1187	535
36	1188	519
36	1189	494
36	1190	476
36	1191	424
36	1192	379
36	1193	352
36	1194	335
36	1195	315
36	1196	313
36	1197	301
36	1198	283
36	1199	275
36	1200	253
36	1201	252
37	157 434	
37	227 441	
37	233 434	
37	374 252	
37	565 274	
37	578 330	
37	599 308	
37	631 1395	
37	845 273	
37	875 238	
37	1098	276
37	1112	213
37	1150	278
37	1202	527

37	1203	474
37	1204	330
37	1205	313
37	1206	313
37	1207	312
37	1208	303
37	1209	298
37	1210	290
37	1211	288
37	1212	287
37	1213	285
37	1214	283
37	1215	281
37	1216	270
37	1217	268
37	1218	265
37	1219	262
37	1220	261
37	1221	261
37	1222	258
37	1223	258
37	1224	253
37	1225	252
37	1226	250
37	1227	247
37	1228	241
37	1229	235
37	1230	229
37	1231	223
37	1232	221

37 1233 219

37 1234 217

37 1235 215

37 1236 215

37 1237 213

37 1238 212

38 56 74

38 65 3008

38 88 792

38 89 20

38 176 25

38 182 507

38 187 21

38 190 134

38 203 180

38 227 118

38 228 153

38 229 967

38 288 7

38 298 24

38 300 95

38 306 33

38 333 74

38 342 56

38 344 122

38 366 42

38 421 100

38 466 49

38 486 9

38 489 9

38	498	1764
38	511	593
38	533	379
38	562	26
38	614	12
38	679	680
38	689	149
38	691	33
38	701	55
38	951	71
38	959	68
38	982	1235
38	1090	64
38	1151	15
38	1239	68
38	1240	68
38	1241	65
38	1242	40
38	1243	24
38	1244	19
38	1245	19
38	1246	15
38	1247	13
38	1248	12
38	1249	7
39	13	76
39	19	57
39	47	50
39	81	52
39	181	71

39	841 93	
39	843 54	
39	954 91	
39	1250	320
39	1251	124
39	1252	119
39	1253	109
39	1254	108
39	1255	106
39	1256	90
39	1257	90
39	1258	85
39	1259	80
39	1260	74
39	1261	74
39	1262	74
39	1263	72
39	1264	70
39	1265	67
39	1266	66
39	1267	66
39	1268	65
39	1269	63
39	1270	63
39	1271	60
39	1272	59
39	1273	59
39	1274	58
39	1275	58
39	1276	58

39	1277	57
39	1278	57
39	1279	57
39	1280	56
39	1281	55
39	1282	55
39	1283	55
39	1284	53
39	1285	53
39	1286	53
39	1287	53
39	1288	52
39	1289	52
39	1290	51
39	1291	51
40	32	411
40	34	1585
40	38	404
40	1020	196
40	1066	366
40	1292	1351
40	1293	1097
40	1294	1046
40	1295	969
40	1296	927
40	1297	845
40	1298	777
40	1299	730
40	1300	591
40	1301	543

40	1302	524
40	1303	481
40	1304	454
40	1305	450
40	1306	385
40	1307	337
40	1308	328
40	1309	317
40	1310	297
40	1311	296
40	1312	286
40	1313	275
40	1314	272
40	1315	272
40	1316	256
40	1317	255
40	1318	253
40	1319	251
40	1320	233
40	1321	228
40	1322	223
40	1323	216
40	1324	215
40	1325	210
40	1326	205
40	1327	200
40	1328	199
40	1329	196
40	1330	192
40	1331	187

40	1332	184
40	1333	178
40	1334	177
40	1335	176
40	1336	167
41	706 1118	
41	843 637	
41	917 7225	
41	918 2266	
41	922 1453	
41	928 845	
41	942 3522	
41	947 806	
41	954 580	
41	957 650	
41	1257	1085
41	1261	1055
41	1337	2498
41	1338	1955
41	1339	1888
41	1340	1460
41	1341	1216
41	1342	998
41	1343	860
41	1344	847
41	1345	832
41	1346	779
41	1347	773
41	1348	760
41	1349	705

41	1350	624
41	1351	607
41	1352	546
41	1353	535
41	1354	501
41	1355	485
41	1356	452
41	1357	451
41	1358	433
41	1359	430
41	1360	400
41	1361	395
41	1362	385
41	1363	379
41	1364	349
41	1365	341
41	1366	336
41	1367	305
41	1368	302
41	1369	299
41	1370	297
41	1371	296
41	1372	292
41	1373	292
41	1374	277
42	65	1897
42	89	122
42	154	1637
42	173	199
42	182	670

42	184	558
42	190	2336
42	191	161
42	204	704
42	221	707
42	225	192
42	228	150
42	229	1209
42	288	251
42	289	197
42	291	377
42	302	292
42	319	132
42	377	168
42	378	1050
42	425	213
42	472	164
42	511	130
42	534	136
42	538	704
42	689	271
42	962	131
42	966	527
42	1048	125
42	1052	146
42	1090	413
42	1375	1046
42	1376	568
42	1377	484
42	1378	330

42	1379	301
42	1380	294
42	1381	288
42	1382	277
42	1383	266
42	1384	226
42	1385	174
42	1386	153
42	1387	149
42	1388	147
42	1389	142
42	1390	135
42	1391	131
42	1392	118
42	1393	118
43	53	289
43	56	2691
43	58	162
43	64	6390
43	65	635
43	70	192
43	80	431
43	81	207
43	84	137
43	88	582
43	89	301
43	154	1561
43	190	409
43	207	214
43	227	323

43	229	231
43	230	133
43	238	314
43	278	121
43	306	234
43	328	1045
43	329	251
43	331	133
43	377	1028
43	475	175
43	486	227
43	603	156
43	748	3115
43	757	247
43	907	188
43	1009	171
43	1098	211
43	1099	1227
43	1394	1015
43	1395	548
43	1396	452
43	1397	380
43	1398	268
43	1399	255
43	1400	250
43	1401	225
43	1402	211
43	1403	187
43	1404	180
43	1405	171

43	1406	152
43	1407	129
43	1408	128
43	1409	123
43	1410	120
44	15	117
44	25	145
44	67	162
44	152	132
44	163	915
44	190	312
44	215	335
44	226	130
44	227	1493
44	234	156
44	418	628
44	436	331
44	562	389
44	609	186
44	614	555
44	615	184
44	630	289
44	706	629
44	707	469
44	717	178
44	718	181
44	735	463
44	854	772
44	868	136
44	917	425

44	918	177
44	957	109
44	964	468
44	982	138
44	1119	397
44	1131	120
44	1216	192
44	1369	168
44	1411	793
44	1412	667
44	1413	532
44	1414	348
44	1415	266
44	1416	255
44	1417	249
44	1418	235
44	1419	196
44	1420	194
44	1421	185
44	1422	161
44	1423	123
44	1424	118
44	1425	115
44	1426	112
44	1427	107
45	89	2319
45	161	443
45	288	6497
45	289	548
45	291	143

45 292 515

45 293 370

45 295 174

45 300 851

45 302 113

45 306 822

45 310 189

45 320 264

45 325 148

45 332 139

45 333 154

45 349 615

45 461 1916

45 466 307

45 543 418

45 641 276

45 646 4510

45 701 1171

45 787 180

45 790 318

45 792 178

45 795 219

45 802 11126

45 1428 4376

45 1429 862

45 1430 518

45 1431 351

45 1432 293

45 1433 263

45 1434 261

45 1435 258
45 1436 255
45 1437 223
45 1438 222
45 1439 205
45 1440 185
45 1441 174
45 1442 154
45 1443 129
45 1444 123
45 1445 122
45 1446 114
45 1447 112
45 1448 109
45 1449 109
46 89 2199
46 288 31427
46 289 1016
46 290 669
46 291 248
46 292 1052
46 295 3269
46 299 527
46 300 3579
46 301 668
46 302 284
46 306 1989
46 314 816
46 320 320
46 325 929

46	327	313
46	328	394
46	330	240
46	333	1165
46	344	278
46	349	1668
46	352	254
46	458	1562
46	461	973
46	464	3052
46	466	4521
46	475	220
46	484	256
46	498	688
46	542	417
46	646	671
46	679	252
46	681	559
46	686	746
46	701	993
46	802	927
46	908	234
46	1032	560
46	1037	1293
46	1042	401
46	1051	341
46	1428	1073
46	1450	668
46	1451	644
46	1452	555

46	1453	456
46	1454	268
46	1455	264
46	1456	234
46	1457	216
47	182 211	
47	230 7454	
47	257 346	
47	288 1194	
47	289 12197	
47	291 781	
47	292 1075	
47	293 451	
47	294 302	
47	298 642	
47	299 340	
47	300 545	
47	302 481	
47	304 589	
47	310 834	
47	317 243	
47	318 1344	
47	325 607	
47	333 1001	
47	344 998	
47	455 663	
47	456 1743	
47	461 1679	
47	464 231	
47	465 336	

47	466	2098
47	469	276
47	475	276
47	498	336
47	523	1044
47	525	331
47	540	440
47	680	247
47	681	1075
47	686	1223
47	701	422
47	790	252
47	794	233
47	903	356
47	908	1157
47	972	788
47	1037	275
47	1244	315
47	1456	218
47	1458	1392
47	1459	477
47	1460	404
47	1461	392
47	1462	228
47	1463	219
48	55	13
48	67	17476
48	89	112
48	157	60
48	176	33

48	180	586
48	220	240
48	227	15
48	230	18
48	246	19
48	258	9
48	265	17
48	283	122
48	288	13
48	289	23
48	295	6
48	299	15
48	300	38
48	340	406
48	378	78
48	475	4
48	511	483
48	533	17
48	575	43
48	679	5
48	701	14
48	733	19
48	889	230
48	945	64
48	951	92
48	959	14
48	972	9
48	1421	6
48	1464	4085
48	1465	3836

48	1466	486
48	1467	225
48	1468	78
48	1469	71
48	1470	52
48	1471	41
48	1472	35
48	1473	34
48	1474	31
48	1475	21
48	1476	20
48	1477	12
48	1478	8
48	1479	6
48	1480	6
49	40	79
49	89	156
49	166	87
49	223	80
49	288	124
49	289	137
49	299	102
49	306	108
49	308	86
49	310	117
49	312	282
49	314	144
49	324	176
49	448	107
49	461	106

49	701 240	
49	907 228	
49	913 85	
49	1106	197
49	1390	76
49	1400	112
49	1443	94
49	1481	502
49	1482	415
49	1483	393
49	1484	379
49	1485	276
49	1486	226
49	1487	204
49	1488	193
49	1489	179
49	1490	165
49	1491	161
49	1492	123
49	1493	116
49	1494	114
49	1495	112
49	1496	108
49	1497	105
49	1498	96
49	1499	92
49	1500	91
49	1501	85
49	1502	82
49	1503	82

49	1504	79
49	1505	78
49	1506	78
49	1507	78
49	1508	77
50	56	672
50	59	300
50	65	1984
50	159	790
50	163	759
50	171	419
50	173	278
50	207	3642
50	220	251
50	221	210
50	227	2135
50	228	3036
50	229	808
50	230	262
50	250	317
50	424	599
50	428	325
50	429	240
50	511	528
50	533	9355
50	604	616
50	703	298
50	706	251
50	709	231
50	718	269

50 733 204

50 757 379

50 868 249

50 959 359

50 969 960

50 982 364

50 1048 2689

50 1090 412

50 1106 277

50 1151 533

50 1376 281

50 1394 779

50 1412 228

50 1509 2122

50 1510 747

50 1511 647

50 1512 424

50 1513 342

50 1514 308

50 1515 302

50 1516 301

50 1517 279

50 1518 251

50 1519 216

50 1520 207

51 163 380

51 173 339

51 188 1074

51 198 708

51 225 531

51	503	1446
51	706	202
51	707	1231
51	726	139
51	806	1122
51	841	2490
51	917	1409
51	918	3549
51	923	1243
51	928	262
51	947	1180
51	949	423
51	954	327
51	959	308
51	1104	1010
51	1214	1074
51	1249	478
51	1257	1020
51	1261	328
51	1264	1214
51	1274	345
51	1337	228
51	1344	221
51	1356	157
51	1358	1346
51	1360	1044
51	1364	292
51	1369	267
51	1412	1110
51	1521	1084

51	1522	482
51	1523	431
51	1524	397
51	1525	362
51	1526	353
51	1527	293
51	1528	292
51	1529	221
51	1530	212
51	1531	182
51	1532	179
51	1533	167
51	1534	164
51	1535	163
51	1536	158
52	329	104
52	355	94
52	455	93
52	464	107
52	489	131
52	500	102
52	805	197
52	815	134
52	816	622
52	825	84
52	831	170
52	839	191
52	1044	112
52	1196	83
52	1274	103

52	1281	198
52	1537	1109
52	1538	384
52	1539	308
52	1540	291
52	1541	279
52	1542	279
52	1543	214
52	1544	203
52	1545	193
52	1546	191
52	1547	190
52	1548	185
52	1549	175
52	1550	164
52	1551	157
52	1552	151
52	1553	138
52	1554	135
52	1555	132
52	1556	125
52	1557	123
52	1558	121
52	1559	118
52	1560	110
52	1561	106
52	1562	103
52	1563	98
52	1564	94
52	1565	93

52	1566	90
52	1567	88
52	1568	82
52	1569	79
52	1570	79
53	55	33
53	67	64
53	89	1872
53	187	24
53	288	1620
53	289	267
53	292	332
53	293	207
53	296	360
53	300	4332
53	302	56
53	306	147
53	310	55
53	318	24
53	330	29
53	331	33
53	333	34
53	349	41
53	352	83
53	386	56
53	466	532
53	475	240
53	478	408
53	483	137
53	491	41

53	498	28
53	525	28
53	531	64
53	535	233
53	545	31
53	644	55
53	701	128
53	1571	585
53	1572	476
53	1573	305
53	1574	148
53	1575	93
53	1576	79
53	1577	78
53	1578	64
53	1579	63
53	1580	49
53	1581	42
53	1582	38
53	1583	36
53	1584	29
53	1585	29
53	1586	28
53	1587	28
53	1588	27
54	55	116
54	67	57
54	71	136
54	88	44
54	288	118

54	289	16391
54	291	201
54	292	233
54	296	61
54	298	73
54	299	214
54	310	68
54	318	1123
54	320	58
54	329	118
54	333	346
54	349	72
54	360	41
54	378	42
54	465	43
54	523	1160
54	525	139
54	535	78
54	538	68
54	701	230
54	793	329
54	798	263
54	800	80
54	906	152
54	911	46
54	913	161
54	1052	172
54	1377	43
54	1392	44
54	1589	247

54	1590	126
54	1591	112
54	1592	109
54	1593	69
54	1594	69
54	1595	63
54	1596	62
54	1597	59
54	1598	57
54	1599	51
54	1600	46
54	1601	39
54	1602	39
54	1603	38
54	1604	37
55	65	316
55	154	1485
55	190	568
55	220	374
55	227	468
55	267	358
55	276	761
55	278	481
55	331	648
55	425	757
55	429	419
55	569	559
55	1455	874
55	1605	1226
55	1606	1141

55	1607	829
55	1608	821
55	1609	786
55	1610	780
55	1611	728
55	1612	712
55	1613	700
55	1614	643
55	1615	624
55	1616	560
55	1617	548
55	1618	545
55	1619	533
55	1620	461
55	1621	458
55	1622	439
55	1623	390
55	1624	379
55	1625	371
55	1626	367
55	1627	354
55	1628	339
55	1629	336
55	1630	335
55	1631	328
55	1632	328
55	1633	322
55	1634	306
55	1635	305
55	1636	303

55	1637	298
55	1638	293
55	1639	285
55	1640	273
55	1641	273
56	72	400
56	159	497
56	171	822
56	173	404
56	207	484
56	228	1072
56	366	430
56	424	1934
56	436	351
56	438	550
56	488	676
56	604	693
56	683	1606
56	720	1702
56	857	642
56	932	674
56	982	379
56	1116	1350
56	1119	382
56	1513	2279
56	1515	346
56	1545	498
56	1642	2174
56	1643	1621
56	1644	1498

56	1645	1491
56	1646	887
56	1647	840
56	1648	754
56	1649	719
56	1650	704
56	1651	681
56	1652	675
56	1653	636
56	1654	593
56	1655	569
56	1656	557
56	1657	496
56	1658	477
56	1659	463
56	1660	456
56	1661	452
56	1662	447
56	1663	427
56	1664	403
56	1665	349
56	1666	342
56	1667	340
56	1668	339
56	1669	338
57	89	1428
57	207	141
57	234	50
57	288	179
57	289	53

57	292	28
57	295	23
57	300	279
57	333	1555
57	344	1600
57	377	32
57	378	325
57	424	68
57	464	23
57	466	39
57	470	3000
57	471	475
57	475	64
57	481	589
57	486	368
57	498	481
57	686	26
57	704	1531
57	779	10000
57	889	251
57	1034	24
57	1041	876
57	1043	777
57	1045	322
57	1180	6000
57	1185	161
57	1188	323
57	1190	366
57	1196	288
57	1246	37

57	1429	47
57	1541	138
57	1659	45
57	1670	13000
57	1671	1488
57	1672	796
57	1673	522
57	1674	490
57	1675	298
57	1676	241
57	1677	188
57	1678	155
57	1679	32
57	1680	31
57	1681	23
58	67	1225
58	89	1770
58	288	5
58	289	4474
58	291	4
58	292	1000
58	295	8
58	300	1412
58	302	224
58	318	7
58	321	236
58	325	171
58	333	455
58	344	416
58	386	10

58	461 8204
58	466 46
58	523 124
58	680 4
58	681 4269
58	686 4
58	797 3
58	835 43
58	889 5
58	892 275
58	908 158
58	1098	13
58	1244	6
58	1453	273
58	1458	47
58	1459	11
58	1597	525
58	1682	1023
58	1683	377
58	1684	178
58	1685	141
58	1686	109
58	1687	28
58	1688	27
58	1689	11
58	1690	9
58	1691	6
58	1692	6
58	1693	5
58	1694	4

58	1695	4
58	1696	3
58	1697	3
58	1698	3
58	1699	3
59	7	1410
59	27	1105
59	36	810
59	56	832
59	67	757
59	72	3445
59	190	3458
59	227	1472
59	233	16419
59	238	870
59	296	458
59	377	1779
59	407	1392
59	450	533
59	475	458
59	504	600
59	512	1023
59	517	551
59	599	587
59	603	447
59	707	3437
59	710	419
59	726	17216
59	1098	638
59	1104	3049

59	1114	670
59	1118	2900
59	1131	5156
59	1151	1060
59	1210	456
59	1358	688
59	1359	486
59	1534	574
59	1700	1802
59	1701	1778
59	1702	1592
59	1703	1217
59	1704	1129
59	1705	1055
59	1706	816
59	1707	713
59	1708	713
59	1709	699
59	1710	637
59	1711	633
59	1712	624
59	1713	610
59	1714	591
59	1715	559
59	1716	425
60	103 107	
60	113 40	
60	126 38	
60	204 43	
60	210 75	

60	416	113
60	418	158
60	621	55
60	755	42
60	1106	75
60	1163	40
60	1418	73
60	1717	258
60	1718	183
60	1719	96
60	1720	88
60	1721	84
60	1722	84
60	1723	83
60	1724	78
60	1725	77
60	1726	72
60	1727	69
60	1728	68
60	1729	68
60	1730	58
60	1731	57
60	1732	56
60	1733	53
60	1734	52
60	1735	52
60	1736	52
60	1737	51
60	1738	49
60	1739	49

60	1740	48
60	1741	48
60	1742	47
60	1743	47
60	1744	46
60	1745	45
60	1746	44
60	1747	44
60	1748	44
60	1749	43
60	1750	42
60	1751	40
60	1752	39
60	1753	39
60	1754	39
61	65	170
61	163	316
61	196	414
61	212	181
61	227	645
61	246	249
61	261	291
61	270	625
61	418	452
61	439	550
61	604	361
61	618	212
61	632	220
61	714	284
61	727	185

61	1109	1103
61	1112	208
61	1123	295
61	1244	431
61	1421	260
61	1424	438
61	1755	858
61	1756	696
61	1757	600
61	1758	471
61	1759	352
61	1760	343
61	1761	285
61	1762	267
61	1763	263
61	1764	240
61	1765	239
61	1766	238
61	1767	237
61	1768	232
61	1769	208
61	1770	201
61	1771	190
61	1772	189
61	1773	188
61	1774	187
61	1775	187
61	1776	182
61	1777	181
61	1778	179

61	1779	178
61	1780	171
61	1781	170
61	1782	170
61	1783	170
62	51	528
62	54	125
62	64	91
62	65	745
62	70	70
62	72	6918
62	75	59
62	81	76
62	154	272
62	173	443
62	182	1040
62	187	227
62	189	145
62	190	400
62	191	153
62	220	99
62	221	83
62	227	66
62	234	374
62	238	274
62	310	135
62	378	170
62	533	70
62	868	267
62	961	118

62	1072	281
62	1097	95
62	1306	172
62	1375	432
62	1384	139
62	1713	214
62	1784	542
62	1785	291
62	1786	146
62	1787	144
62	1788	112
62	1789	110
62	1790	101
62	1791	98
62	1792	95
62	1793	92
62	1794	89
62	1795	87
62	1796	77
62	1797	73
62	1798	71
62	1799	67
62	1800	64
62	1801	62
62	1802	58
63	51	563
63	159	132
63	163	147
63	183	73
63	190	172

63	193	79
63	203	65
63	212	127
63	227	170
63	234	151
63	554	103
63	562	141
63	614	438
63	704	113
63	706	219
63	707	136
63	735	71
63	841	100
63	854	403
63	856	70
63	857	385
63	868	81
63	917	94
63	918	1101
63	923	184
63	928	122
63	952	66
63	959	83
63	1109	173
63	1119	333
63	1343	79
63	1358	60
63	1400	110
63	1412	133
63	1527	148

63	1803	173
63	1804	104
63	1805	104
63	1806	100
63	1807	97
63	1808	97
63	1809	86
63	1810	82
63	1811	76
63	1812	66
63	1813	61
63	1814	61
63	1815	57
63	1816	57
63	1817	56
64	63	144
64	80	326
64	154	141
64	157	546
64	266	175
64	271	216
64	285	320
64	576	749
64	601	1679
64	987	615
64	1139	152
64	1156	154
64	1394	248
64	1427	402
64	1613	344

64	1641	267
64	1818	494
64	1819	398
64	1820	344
64	1821	286
64	1822	282
64	1823	270
64	1824	264
64	1825	252
64	1826	249
64	1827	245
64	1828	245
64	1829	239
64	1830	231
64	1831	225
64	1832	215
64	1833	213
64	1834	213
64	1835	186
64	1836	185
64	1837	181
64	1838	181
64	1839	174
64	1840	168
64	1841	167
64	1842	157
64	1843	156
64	1844	154
64	1845	154
64	1846	149

64 1847 145

64 1848 144

64 1849 144

64 1850 140

64 1851 138

65 173 87

65 209 355

65 333 183

65 371 2718

65 377 132

65 463 104

65 464 68

65 472 255

65 475 106

65 488 1189

65 489 88

65 497 453

65 498 490

65 540 272

65 805 74

65 808 136

65 816 1062

65 850 83

65 907 100

65 1145 110

65 1180 609

65 1195 248

65 1200 741

65 1678 112

65 1852 1731

65	1853	735
65	1854	471
65	1855	463
65	1856	450
65	1857	377
65	1858	254
65	1859	210
65	1860	201
65	1861	158
65	1862	150
65	1863	137
65	1864	133
65	1865	133
65	1866	113
65	1867	113
65	1868	99
65	1869	94
65	1870	92
65	1871	79
65	1872	77
65	1873	76
65	1874	75
65	1875	73
65	1876	70
65	1877	67
66	51	910
66	59	367
66	61	603
66	68	459
66	72	1303

66	85	888
66	98	310
66	99	497
66	100	417
66	874	427
66	880	313
66	993	611
66	994	256
66	997	688
66	998	350
66	1001	529
66	1006	455
66	1013	626
66	1014	478
66	1019	415
66	1027	337
66	1073	381
66	1122	399
66	1799	405
66	1836	370
66	1878	2190
66	1879	1179
66	1880	1097
66	1881	938
66	1882	792
66	1883	716
66	1884	691
66	1885	593
66	1886	572
66	1887	563

66	1888	544
66	1889	476
66	1890	438
66	1891	430
66	1892	384
66	1893	377
66	1894	347
66	1895	343
66	1896	337
66	1897	324
66	1898	320
66	1899	313
66	1900	303
66	1901	265
66	1902	255
68	65	171
68	67	624
68	89	916
68	239	63
68	257	682
68	283	63
68	285	96
68	288	603
68	289	591
68	290	137
68	292	105
68	294	379
68	295	4140
68	299	222
68	300	140

68	301	81
68	302	115
68	306	216
68	308	142
68	314	61
68	317	39
68	320	45
68	322	142
68	327	85
68	328	70
68	329	79
68	330	125
68	332	41
68	349	353
68	366	293
68	465	56
68	466	84
68	468	71
68	484	48
68	527	107
68	531	69
68	543	210
68	701	100
68	913	119
68	1032	321
68	1379	54
68	1934	233
68	1935	188
68	1936	185
68	1937	143

68	1938	130
68	1939	90
68	1940	89
68	1941	44
68	1942	40
69	107 225	
69	154 746	
69	195 521	
69	210 621	
69	267 238	
69	271 317	
69	331 290	
69	427 187	
69	429 232	
69	441 231	
69	527 203	
69	603 280	
69	609 400	
69	616 181	
69	618 226	
69	1098	238
69	1622	290
69	1627	849
69	1640	182
69	1943	1429
69	1944	578
69	1945	534
69	1946	520
69	1947	431
69	1948	415

69	1949	404
69	1950	396
69	1951	389
69	1952	352
69	1953	319
69	1954	319
69	1955	305
69	1956	301
69	1957	294
69	1958	283
69	1959	278
69	1960	273
69	1961	265
69	1962	260
69	1963	254
69	1964	237
69	1965	228
69	1966	226
69	1967	224
69	1968	217
69	1969	215
69	1970	209
69	1971	186
69	1972	177
69	1973	176
70	163 386	
70	173 595	
70	190 526	
70	207 419	
70	228 302	

70 229 232

70 234 581

70 245 321

70 428 372

70 432 196

70 475 695

70 497 252

70 512 295

70 517 212

70 536 204

70 614 194

70 707 287

70 709 962

70 757 695

70 854 191

70 856 272

70 868 2121

70 1048 343

70 1090 783

70 1104 170

70 1155 239

70 1376 169

70 1406 896

70 1412 1042

70 1426 195

70 1520 1257

70 1873 324

70 1974 1275

70 1975 1202

70 1976 811

70	1977	739
70	1978	722
70	1979	599
70	1980	478
70	1981	463
70	1982	369
70	1983	358
70	1984	314
70	1985	255
70	1986	247
70	1987	232
70	1988	210
70	1989	199
70	1990	198
70	1991	179
71	188 310	
71	707 285	
71	843 236	
71	917 562	
71	918 575	
71	920 1561	
71	922 148	
71	923 80	
71	930 243	
71	936 569	
71	944 394	
71	957 193	
71	978 81	
71	1104	111
71	1113	724

71	1128	95
71	1132	180
71	1249	274
71	1339	76
71	1348	241
71	1349	82
71	1350	138
71	1351	104
71	1535	296
71	1992	1174
71	1993	451
71	1994	398
71	1995	329
71	1996	262
71	1997	254
71	1998	236
71	1999	210
71	2000	203
71	2001	202
71	2002	165
71	2003	162
71	2004	135
71	2005	120
71	2006	113
71	2007	111
71	2008	104
71	2009	99
71	2010	93
71	2011	90
71	2012	87

71	2013	86
71	2014	79
71	2015	79
71	2016	78
71	2017	77
72	55	6
72	58	2
72	67	51
72	71	23
72	89	19
72	157	3
72	251	1
72	257	6
72	265	37
72	285	23
72	289	34
72	290	23
72	291	12
72	292	47
72	293	1
72	294	23
72	295	40
72	296	2
72	298	29
72	299	2
72	300	30
72	302	28
72	305	19
72	306	1
72	310	35

72	320	18
72	328	8
72	329	32
72	333	1
72	340	16
72	386	30
72	465	26
72	466	21
72	485	6
72	523	1
72	525	11
72	686	26
72	792	48
72	889	10
72	913	1
72	972	15
72	1820	4
72	2018	23
72	2019	17
72	2020	16
72	2021	5
72	2022	2
72	2023	1
72	2024	1
72	2025	1
73	19	374
73	30	782
73	36	294
73	51	1962
73	68	329

73	82	403
73	91	217
73	155	157
73	157	162
73	229	163
73	441	205
73	533	214
73	599	566
73	620	298
73	724	159
73	851	366
73	877	386
73	986	568
73	995	362
73	997	470
73	998	432
73	1001	2017
73	1073	557
73	1089	200
73	1297	206
73	1502	198
73	1704	149
73	1711	715
73	1714	323
73	1791	312
73	1890	428
73	1895	215
73	2026	1294
73	2027	625
73	2028	555

73	2029	528
73	2030	415
73	2031	374
73	2032	330
73	2033	326
73	2034	320
73	2035	319
73	2036	285
73	2037	271
73	2038	261
73	2039	241
73	2040	234
73	2041	201
73	2042	182
73	2043	169
74	157	3213
74	161	392
74	185	136
74	321	548
74	461	569
74	518	170
74	641	138
74	646	929
74	786	110
74	802	673
74	951	204
74	1114	177
74	1186	188
74	1189	302
74	2044	28397

74	2045	3961
74	2046	2562
74	2047	1166
74	2048	1042
74	2049	923
74	2050	724
74	2051	661
74	2052	459
74	2053	453
74	2054	449
74	2055	282
74	2056	255
74	2057	251
74	2058	220
74	2059	214
74	2060	210
74	2061	204
74	2062	204
74	2063	194
74	2064	183
74	2065	175
74	2066	174
74	2067	170
74	2068	163
74	2069	162
74	2070	155
74	2071	155
74	2072	146
74	2073	145
74	2074	135

74	2075	132
74	2076	128
74	2077	127
74	2078	121
74	2079	115
75	55	500
75	67	302
75	89	1342
75	157	102
75	161	195
75	209	126
75	230	517
75	288	848
75	289	1499
75	292	1553
75	295	725
75	298	71
75	299	66
75	300	712
75	302	414
75	306	1519
75	329	141
75	333	955
75	344	101
75	352	123
75	377	853
75	378	196
75	412	63
75	464	170
75	466	503

75	475	91
75	481	318
75	498	3085
75	531	82
75	538	210
75	543	143
75	544	535
75	646	373
75	679	2165
75	687	759
75	701	1749
75	972	69
75	1032	196
75	1037	611
75	1042	292
75	1246	116
75	1452	65
75	1455	119
75	1456	326
75	2080	407
75	2081	188
75	2082	152
75	2083	125
75	2084	107
75	2085	62
76	89	958
76	367	1011
76	369	562
76	375	1006
76	376	676

76 388 670
76 393 1276
76 397 3563
76 1857 746
76 1904 808
76 1910 1093
76 1915 782
76 1919 1842
76 1923 980
76 2000 3600
76 2086 4464
76 2087 3423
76 2088 2933
76 2089 2157
76 2090 2099
76 2091 2091
76 2092 1839
76 2093 1483
76 2094 1377
76 2095 1343
76 2096 1232
76 2097 1050
76 2098 998
76 2099 962
76 2100 900
76 2101 896
76 2102 854
76 2103 793
76 2104 786
76 2105 763

76 2106 754
76 2107 752
76 2108 731
76 2109 719
76 2110 713
76 2111 704
76 2112 699
76 2113 671
76 2114 668
76 2115 643
76 2116 631
76 2117 615
76 2118 614
76 2119 566
76 2120 556
77 88 269
77 155 163
77 183 128
77 190 193
77 207 10867
77 210 104
77 227 1367
77 229 555
77 230 203
77 424 1221
77 430 924
77 432 580
77 538 717
77 547 136
77 590 523

77	687 249	
77	689 142	
77	959 264	
77	962 433	
77	969 66	
77	982 602	
77	1239	238
77	1242	67
77	1390	2648
77	1512	1408
77	1515	494
77	1517	395
77	1988	78
77	2121	2356
77	2122	470
77	2123	457
77	2124	431
77	2125	254
77	2126	206
77	2127	196
77	2128	195
77	2129	191
77	2130	171
77	2131	169
77	2132	167
77	2133	157
77	2134	154
77	2135	144
77	2136	115
77	2137	90

77	2138	78
77	2139	70
77	2140	70
77	2141	68
77	2142	65
78	101	114
78	154	117
78	174	109
78	181	163
78	489	110
78	603	1202
78	744	779
78	757	255
78	759	92
78	778	132
78	785	108
78	818	224
78	842	101
78	845	117
78	1156	143
78	1222	154
78	1548	149
78	1745	363
78	2143	759
78	2144	361
78	2145	332
78	2146	323
78	2147	321
78	2148	304
78	2149	296

78	2150	262
78	2151	240
78	2152	233
78	2153	210
78	2154	201
78	2155	199
78	2156	180
78	2157	165
78	2158	157
78	2159	142
78	2160	136
78	2161	133
78	2162	132
78	2163	128
78	2164	123
78	2165	107
78	2166	103
78	2167	101
78	2168	101
78	2169	98
78	2170	97
78	2171	91
78	2172	85
78	2173	85
78	2174	83
79	89	89
79	157	30226
79	180	119
79	228	17
79	241	53

79 251 48

79 257 90

79 285 23

79 288 95

79 289 201

79 291 111

79 292 8052

79 294 196

79 295 182

79 304 40695

79 305 58

79 311 211

79 313 355

79 325 21

79 329 541

79 330 34

79 378 28

79 461 30

79 466 23

79 468 21

79 498 33

79 542 305

79 544 12384

79 683 85

79 786 24

79 797 48

79 889 15752

79 903 153

79 908 86

79 966 497

79	1043	48
79	1447	68
79	1475	41
79	1556	3498
79	1681	239
79	2175	155
79	2176	88
79	2177	76
79	2178	70
79	2179	54
79	2180	38
79	2181	21
79	2182	20
79	2183	14
79	2184	14
80	768	7
80	779	245
80	805	1170
80	813	281
80	815	7
80	816	1375
80	818	508
80	823	1353
80	825	27
80	830	4
80	831	147
80	849	138
80	1188	265
80	1196	4
80	1541	103

80	1542	3
80	1547	16
80	1861	29
80	2185	1608
80	2186	676
80	2187	575
80	2188	190
80	2189	180
80	2190	170
80	2191	149
80	2192	113
80	2193	107
80	2194	45
80	2195	18
80	2196	16
80	2197	13
80	2198	13
80	2199	13
80	2200	11
80	2201	10
80	2202	8
80	2203	6
80	2204	6
80	2205	6
80	2206	5
80	2207	5
80	2208	5
80	2209	4
80	2210	4
80	2211	4

80	2212	4
80	2213	3
80	2214	3
80	2215	3
80	2216	3
81	276 125	
81	278 147	
81	1641	115
81	1979	356
81	2217	859
81	2218	522
81	2219	477
81	2220	384
81	2221	359
81	2222	338
81	2223	338
81	2224	306
81	2225	302
81	2226	301
81	2227	301
81	2228	279
81	2229	272
81	2230	266
81	2231	262
81	2232	238
81	2233	213
81	2234	207
81	2235	205
81	2236	193
81	2237	191

81	2238	189
81	2239	188
81	2240	186
81	2241	185
81	2242	179
81	2243	175
81	2244	171
81	2245	157
81	2246	154
81	2247	150
81	2248	145
81	2249	144
81	2250	142
81	2251	141
81	2252	136
81	2253	128
81	2254	128
81	2255	128
81	2256	126
81	2257	123
81	2258	121
81	2259	118
81	2260	114
81	2261	113
81	2262	110
82	154 108	
82	412 140	
82	419 650	
82	426 162	
82	433 1617	

82	440 266	
82	445 107	
82	604 134	
82	622 909	
82	1106	130
82	1117	353
82	1139	125
82	1159	166
82	1633	186
82	1737	309
82	2263	2020
82	2264	1087
82	2265	771
82	2266	612
82	2267	521
82	2268	453
82	2269	385
82	2270	347
82	2271	297
82	2272	257
82	2273	256
82	2274	248
82	2275	246
82	2276	230
82	2277	230
82	2278	219
82	2279	212
82	2280	208
82	2281	198
82	2282	196

82 2283 161

82 2284 156

82 2285 146

82 2286 140

82 2287 135

82 2288 129

82 2289 127

82 2290 125

82 2291 125

82 2292 124

82 2293 115

82 2294 109

82 2295 104

82 2296 103

82 2297 102

83 154 1375

83 163 1881

83 196 724

83 200 532

83 384 931

83 959 910

83 1109 726

83 2298 4449

83 2299 1827

83 2300 1480

83 2301 1375

83 2302 1315

83 2303 1302

83 2304 1289

83 2305 1226

83	2306	1223
83	2307	1197
83	2308	1181
83	2309	1102
83	2310	1085
83	2311	1054
83	2312	969
83	2313	960
83	2314	917
83	2315	904
83	2316	862
83	2317	843
83	2318	839
83	2319	826
83	2320	817
83	2321	747
83	2322	732
83	2323	637
83	2324	597
83	2325	574
83	2326	562
83	2327	554
83	2328	545
83	2329	543
83	2330	539
83	2331	533
83	2332	526
83	2333	526
83	2334	515
83	2335	511

83 2336 497

83 2337 491

83 2338 489

83 2339 478

83 2340 467

84 25 77

84 163 215

84 176 117

84 198 217

84 220 88

84 517 147

84 570 90

84 706 507

84 707 502

84 716 129

84 724 1484

84 732 360

84 735 211

84 843 239

84 917 1831

84 918 152

84 923 1167

84 928 64

84 959 214

84 966 90

84 1044 173

84 1104 117

84 1180 79

84 1244 1359

84 1248 275

84	1249	945
84	1274	75
84	1338	1097
84	1351	176
84	1358	165
84	1360	424
84	1369	1648
84	1412	115
84	1513	129
84	1672	131
84	1803	210
84	1814	59
84	2341	3599
84	2342	1329
84	2343	675
84	2344	635
84	2345	318
84	2346	294
84	2347	215
84	2348	203
84	2349	102
84	2350	96
84	2351	76
84	2352	69
84	2353	54
85	7	185
85	152	87
85	181	277
85	190	95
85	226	174

85	233	121
85	238	109
85	504	228
85	512	212
85	517	128
85	599	170
85	720	108
85	808	96
85	851	119
85	854	201
85	860	103
85	861	89
85	923	99
85	962	112
85	982	94
85	1216	251
85	1240	89
85	1249	87
85	1410	208
85	1672	86
85	1787	175
85	2354	495
85	2355	384
85	2356	355
85	2357	292
85	2358	281
85	2359	217
85	2360	177
85	2361	160
85	2362	154

85	2363	153
85	2364	140
85	2365	128
85	2366	120
85	2367	119
85	2368	114
85	2369	113
85	2370	113
85	2371	113
85	2372	111
85	2373	111
85	2374	103
85	2375	98
85	2376	94
85	2377	92
86	190 539	
86	229 346	
86	371 235	
86	377 460	
86	450 259	
86	488 276	
86	500 614	
86	514 1275	
86	516 288	
86	517 511	
86	706 217	
86	707 672	
86	779 254	
86	804 304	
86	805 960	

86	809	1295
86	810	874
86	811	467
86	813	700
86	814	1459
86	815	598
86	823	597
86	825	270
86	826	297
86	830	345
86	839	386
86	841	310
86	842	402
86	978	608
86	1196	577
86	1540	253
86	1541	945
86	1557	519
86	1677	230
86	1678	249
86	1861	599
86	2143	278
86	2200	227
86	2378	646
86	2379	533
86	2380	492
86	2381	366
86	2382	341
86	2383	317
86	2384	311

86	2385	282
86	2386	257
86	2387	241
86	2388	240
86	2389	215
87	67	108
87	161	26
87	257	25
87	265	34
87	285	85
87	288	20
87	289	101
87	292	132
87	294	76
87	295	127
87	300	57
87	301	46
87	302	66
87	305	19
87	310	91
87	318	47
87	320	42
87	329	90
87	332	53
87	340	21
87	349	24
87	456	25
87	457	19
87	458	21
87	465	61

87	466	33
87	476	21
87	484	20
87	525	81
87	535	62
87	641	43
87	686	31
87	788	31
87	792	46
87	889	61
87	913	43
87	1441	38
87	1449	22
87	1936	26
87	1937	31
87	2019	19
87	2023	21
87	2390	72
87	2391	59
87	2392	56
87	2393	35
87	2394	32
87	2395	27
87	2396	22
87	2397	20
88	154	3
88	159	52
88	196	15
88	199	7
88	204	42

88	210	12
88	211	35
88	223	5
88	433	34
88	440	14
88	592	4
88	622	28
88	629	9
88	630	3
88	859	55
88	863	4
88	1242	7
88	1732	12
88	1944	4
88	1947	94
88	2277	22
88	2280	51
88	2398	68
88	2399	61
88	2400	53
88	2401	42
88	2402	38
88	2403	29
88	2404	28
88	2405	28
88	2406	24
88	2407	23
88	2408	21
88	2409	14
88	2410	12

88 2411 12
88 2412 9
88 2413 9
88 2414 9
88 2415 8
88 2416 7
88 2417 7
88 2418 6
88 2419 5
88 2420 5
88 2421 5
88 2422 5
88 2423 5
88 2424 4
88 2425 4
89 89 901
89 190 573
89 207 736
89 209 5627
89 228 818
89 289 421
89 291 1363
89 300 1089
89 302 760
89 318 229
89 321 238
89 333 1218
89 362 368
89 377 384
89 378 867

89	403	1867
89	455	407
89	464	745
89	486	1337
89	488	857
89	498	1106
89	525	537
89	680	3252
89	687	247
89	693	293
89	701	4363
89	704	1089
89	779	369
89	903	403
89	1035	249
89	1047	1441
89	1180	2360
89	1183	1039
89	1186	883
89	1188	749
89	1193	757
89	1246	236
89	1672	854
89	1673	616
89	1853	673
89	2426	1136
89	2427	738
89	2428	608
89	2429	605
89	2430	444

89	2431	393
89	2432	313
89	2433	284
89	2434	236
89	2435	232
90	172 91	
90	190 575	
90	227 119	
90	230 93	
90	356 198	
90	360 94	
90	389 119	
90	511 100	
90	538 84	
90	734 119	
90	735 102	
90	987 119	
90	1122	120
90	1244	142
90	1415	84
90	1427	91
90	2436	444
90	2437	342
90	2438	302
90	2439	281
90	2440	273
90	2441	245
90	2442	236
90	2443	231
90	2444	209

90	2445	189
90	2446	170
90	2447	163
90	2448	160
90	2449	153
90	2450	136
90	2451	132
90	2452	125
90	2453	123
90	2454	122
90	2455	120
90	2456	118
90	2457	106
90	2458	103
90	2459	99
90	2460	97
90	2461	97
90	2462	97
90	2463	96
90	2464	95
90	2465	94
90	2466	92
90	2467	90
90	2468	89
90	2469	87
91	197 1859	
91	441 1063	
91	475 418	
91	599 1066	
91	631 3481	

91	859 676	
91	867 1104	
91	1073	1178
91	1119	625
91	1167	519
91	1203	621
91	1221	678
91	1293	714
91	1504	1411
91	1534	830
91	1878	1136
91	1889	602
91	1890	3430
91	2026	7662
91	2030	2058
91	2037	1714
91	2042	632
91	2043	522
91	2470	3616
91	2471	1776
91	2472	1691
91	2473	1338
91	2474	1163
91	2475	1153
91	2476	1134
91	2477	1031
91	2478	1005
91	2479	939
91	2480	879
91	2481	855

91	2482	852
91	2483	823
91	2484	801
91	2485	765
91	2486	761
91	2487	701
91	2488	614
91	2489	562
91	2490	526
91	2491	514
91	2492	489
91	2493	475
91	2494	468
91	2495	437
91	2496	425
92	67	5
92	89	17
92	288	6
92	289	8
92	290	6
92	295	5
92	296	8
92	300	5
92	301	5
92	302	6
92	306	20
92	310	9
92	328	9
92	386	12
92	464	11

92	466	13
92	475	7
92	526	9
92	681	5
92	907	9
92	1243	13
92	1351	9
92	1358	28
92	1686	10
92	1689	5
92	2009	18
92	2086	14
92	2101	14
92	2497	306
92	2498	72
92	2499	35
92	2500	19
92	2501	19
92	2502	14
92	2503	10
92	2504	9
92	2505	9
92	2506	8
92	2507	8
92	2508	7
92	2509	7
92	2510	7
92	2511	7
92	2512	6
92	2513	5

92 2514 5
92 2515 5
92 2516 5
92 2517 5
92 2518 5
94 59 104
94 72 135
94 76 84
94 88 658
94 154 418
94 159 174
94 199 1283
94 203 1761
94 210 68
94 217 187
94 222 157
94 227 139
94 331 64
94 418 111
94 424 377
94 427 64
94 429 1227
94 433 223
94 440 91
94 441 86
94 533 564
94 599 120
94 612 313
94 620 267
94 858 164

94	859	159
94	1089	66
94	1098	282
94	1106	150
94	1107	80
94	1504	608
94	1954	74
94	1957	558
94	1958	73
94	1964	147
94	2139	298
94	2424	178
94	2530	175
94	2531	151
94	2532	119
94	2533	107
94	2534	95
94	2535	88
94	2536	87
94	2537	85
94	2538	81
94	2539	81
94	2540	76
94	2541	64
94	2542	60
95	55	127
95	67	120
95	89	309
95	157	24
95	257	99

95 288 587

95 289 402

95 291 89

95 292 86

95 295 367

95 298 85

95 299 22

95 300 176

95 302 144

95 306 23

95 310 35

95 314 46

95 317 22

95 321 134

95 329 282

95 333 64

95 344 290

95 349 50

95 461 208

95 466 474

95 486 32

95 498 108

95 525 57

95 528 28

95 542 47

95 543 116

95 679 1775

95 681 56

95 686 449

95 689 80

95	701	418
95	704	26
95	797	226
95	1032	30
95	1037	189
95	2137	26
95	2521	307
95	2543	257
95	2544	168
95	2545	125
95	2546	72
95	2547	66
95	2548	52
95	2549	32
95	2550	21
96	51	63
96	67	71
96	68	37
96	77	10
96	85	16
96	99	12
96	100	13
96	192	20
96	193	17
96	215	21
96	220	10
96	288	14
96	289	15
96	295	26
96	310	11

96	316	33
96	332	34
96	349	31
96	511	10
96	538	22
96	952	17
96	966	28
96	982	11
96	999	27
96	1076	14
96	1464	40
96	1556	17
96	1820	13
96	2183	11
96	2551	44
96	2552	36
96	2553	34
96	2554	31
96	2555	25
96	2556	20
96	2557	17
96	2558	16
96	2559	16
96	2560	14
96	2561	14
96	2562	14
96	2563	14
96	2564	14
96	2565	12
96	2566	12

96	2567	11
96	2568	11
96	2569	10
96	2570	10
96	2571	10
97	154 221
97	190 180
97	199 201
97	207 408
97	210 303
97	217 2021
97	225 500
97	418 193
97	419 542
97	432 209
97	492 346
97	632 188
97	755 1588
97	779 297
97	805 285
97	816 842
97	845 256
97	920 216
97	1090	234
97	1163	188
97	1858	284
97	1960	395
97	1977	197
97	2150	1551
97	2267	587

97	2273	452
97	2429	348
97	2572	2697
97	2573	2351
97	2574	2156
97	2575	2071
97	2576	1583
97	2577	1039
97	2578	926
97	2579	851
97	2580	848
97	2581	817
97	2582	681
97	2583	621
97	2584	453
97	2585	343
97	2586	319
97	2587	274
97	2588	239
97	2589	213
97	2590	212
97	2591	181
97	2592	179
97	2593	178
97	2594	170
98	40	87
98	55	99
98	65	30
98	67	114
98	88	56

98 89 223
98 155 34
98 157 66
98 161 37
98 227 284
98 230 35
98 288 177
98 289 2500
98 292 281
98 293 44
98 295 36
98 299 36
98 300 273
98 306 559
98 318 980
98 320 97
98 324 54
98 328 134
98 333 30
98 349 71
98 378 60
98 461 100
98 466 153
98 523 251
98 525 125
98 534 50
98 679 30
98 701 155
98 733 34
98 735 50

98	788	47
98	790	127
98	868	36
98	903	51
98	917	75
98	959	189
98	1249	51
98	1412	30
98	2020	60
98	2025	165
98	2595	130
98	2596	63
98	2597	58
98	2598	34
98	2599	32
99	65	443
99	66	209
99	70	415
99	72	5915
99	81	73
99	89	45
99	154	222
99	157	130
99	159	58
99	173	1612
99	190	156
99	204	50
99	210	161
99	228	323
99	229	1314

99	234	157
99	238	65
99	377	63
99	418	100
99	419	107
99	424	56
99	443	127
99	504	318
99	533	42
99	703	115
99	709	103
99	757	826
99	959	90
99	1072	224
99	1075	126
99	1099	82
99	1104	40
99	1379	78
99	1384	57
99	1390	46
99	1397	48
99	1400	269
99	1713	561
99	1983	69
99	2014	47
99	2121	72
99	2131	42
99	2137	177
99	2582	73
99	2600	188

99 2601 77
99 2602 75
99 2603 66
99 2604 60
99 2605 43
100 65 194
100 154 90
100 155 269
100 157 98
100 159 198
100 171 279
100 199 154
100 203 344
100 215 130
100 221 96
100 227 85
100 228 353
100 229 398
100 278 72
100 298 122
100 331 122
100 429 1449
100 511 163
100 538 136
100 618 435
100 712 124
100 715 130
100 851 261
100 857 147
100 859 75

100 863 98

100 1122 129

100 1375 164

100 1376 199

100 1384 97

100 1390 133

100 1633 91

100 1639 72

100 1958 137

100 2488 64

100 2490 77

100 2531 1384

100 2533 73

100 2580 113

100 2606 337

100 2607 325

100 2608 230

100 2609 152

100 2610 115

100 2611 107

100 2612 70

100 2613 68

100 2614 67

100 2615 66

100 2616 66

101 8 1513

101 56 430

101 209 1554

101 220 230

101 226 1663

101 227 611

101 233 555

101 234 737

101 324 92

101 377 1010

101 412 1888

101 470 272

101 481 81

101 493 257

101 498 802

101 500 1235

101 537 1397

101 540 166

101 681 75

101 687 87

101 707 17561

101 768 123

101 779 682

101 816 1496

101 830 118

101 982 459

101 1041 632

101 1045 602

101 1047 4218

101 1122 227

101 1180 1516

101 1186 248

101 1188 2492

101 1239 114

101 1246 75

101 1513 240

101 1670 499

101 1671 316

101 1672 3344

101 1853 4359

101 1910 1298

101 1929 94

101 2210 410

101 2617 1470

101 2618 268

101 2619 200

101 2620 140

101 2621 86

101 2622 85

101 2623 80

102 190 375

102 192 816

102 197 4658

102 198 415

102 199 602

102 202 1708

102 205 563

102 212 1015

102 227 3395

102 230 588

102 271 290

102 434 774

102 511 299

102 580 415

102 602 549

102	617	558
102	728	283
102	733	302
102	982	473
102	986	496
102	1378	323
102	1518	364
102	1633	468
102	1858	453
102	1881	518
102	1889	813
102	1901	3794
102	2265	295
102	2424	1027
102	2444	1294
102	2624	2797
102	2625	1587
102	2626	1553
102	2627	1086
102	2628	851
102	2629	565
102	2630	546
102	2631	538
102	2632	490
102	2633	474
102	2634	404
102	2635	374
102	2636	372
102	2637	355
102	2638	325

102 2639 319

102 2640 308

102 2641 292

102 2642 289

102 2643 286

103 53 36

103 64 56

103 65 53

103 72 1733

103 75 66

103 89 69

103 154 168

103 159 46

103 166 23

103 182 19

103 190 36

103 198 27

103 207 43

103 227 25

103 228 23

103 238 20

103 250 136

103 377 18

103 389 32

103 425 41

103 475 76

103 538 61

103 599 30

103 715 43

103 877 150

103 959 24
103 963 67
103 969 28
103 1001 59
103 1073 143
103 1104 44
103 1109 22
103 1121 69
103 1122 77
103 1124 144
103 1130 151
103 1388 36
103 1491 81
103 1713 23
103 1886 35
103 2247 21
103 2339 51
103 2644 121
103 2645 36
103 2646 35
103 2647 31
103 2648 28
103 2649 22
103 2650 20
103 2651 19
104 89 5748
104 188 3506
104 192 9999
104 229 1786
104 230 2673

104 234 2299

104 289 8544

104 324 2448

104 378 2192

104 383 2137

104 425 1763

104 464 2291

104 498 4264

104 500 1965

104 504 2430

104 680 1837

104 687 3274

104 693 3520

104 705 4800

104 928 2112

104 966 1955

104 1038 2642

104 1041 3396

104 1043 2428

104 1044 10082

104 1045 7325

104 1119 4421

104 1185 1849

104 1201 2224

104 1249 10102

104 1274 8004

104 1412 1940

104 1515 1835

104 1933 7127

104 2637 2986

104 2652 13426

104 2653 5895

104 2654 5539

104 2655 4869

104 2656 4143

104 2657 3978

104 2658 3939

104 2659 3374

104 2660 3213

104 2661 3158

104 2662 3132

104 2663 2912

104 2664 1879

104 2665 1864

104 2666 1754

105 603 360

105 616 285

105 744 293

105 748 285

105 753 113

105 755 116

105 756 158

105 772 171

105 778 189

105 1222 523

105 1423 91

105 1745 180

105 1945 177

105 1969 154

105 2146 242

105 2158	78
105 2161	227
105 2165	145
105 2174	197
105 2496	90
105 2667	432
105 2668	308
105 2669	283
105 2670	260
105 2671	253
105 2672	227
105 2673	207
105 2674	169
105 2675	165
105 2676	154
105 2677	128
105 2678	115
105 2679	115
105 2680	106
105 2681	105
105 2682	102
105 2683	99
105 2684	97
105 2685	93
105 2686	92
105 2687	91
105 2688	89
105 2689	89
105 2690	88
105 2691	86

105	2692	84
105	2693	84
105	2694	81
105	2695	80
105	2696	77
106	412	1095
106	460	655
106	603	851
106	969	916
106	1142	927
106	1191	1027
106	1481	984
106	1515	741
106	2679	1595
106	2680	554
106	2685	715
106	2693	985
106	2696	800
106	2697	2341
106	2698	2136
106	2699	1958
106	2700	1931
106	2701	1742
106	2702	1615
106	2703	1556
106	2704	1388
106	2705	1339
106	2706	1165
106	2707	1134
106	2708	1102

106 2709 1030

106 2710 1017

106 2711 994

106 2712 954

106 2713 949

106 2714 882

106 2715 868

106 2716 861

106 2717 798

106 2718 754

106 2719 748

106 2720 708

106 2721 708

106 2722 658

106 2723 638

106 2724 635

106 2725 624

106 2726 619

106 2727 614

106 2728 577

106 2729 558

106 2730 535

106 2731 509

106 2732 487

106 2733 483

107 174 3607

107 198 3089

107 201 7283

107 220 4108

107 234 1359

107 380 1698

107 460 2618

107 517 3398

107 592 20000

107 707 45407

107 724 4263

107 726 2064

107 841 4236

107 843 2683

107 917 14659

107 918 2946

107 923 3418

107 954 4268

107 957 4138

107 1044 6251

107 1099 2143

107 1103 1983

107 1274 2207

107 1348 2512

107 1353 2093

107 1360 1494

107 1369 9199

107 1406 10731

107 1535 15494

107 1789 2647

107 1992 3767

107 2038 8914

107 2379 2226

107 2620 1737

107 2704 10737

107 2734 13265

107 2735 7680

107 2736 5081

107 2737 4383

107 2738 3373

107 2739 3225

107 2740 2900

107 2741 2809

107 2742 2661

107 2743 2531

107 2744 2503

107 2745 2251

107 2746 2060

107 2747 1987

107 2748 1594

108 12 1019

108 15 278

108 28 675

108 47 506

108 152 345

108 188 785

108 517 488

108 841 492

108 1131 434

108 1214 393

108 1229 330

108 1251 484

108 1254 416

108 1259 358

108 1260 794

108 1274	267
108 1281	417
108 1521	455
108 2654	780
108 2749	1542
108 2750	825
108 2751	800
108 2752	768
108 2753	596
108 2754	566
108 2755	544
108 2756	526
108 2757	519
108 2758	513
108 2759	484
108 2760	479
108 2761	464
108 2762	451
108 2763	416
108 2764	402
108 2765	398
108 2766	391
108 2767	390
108 2768	386
108 2769	374
108 2770	369
108 2771	348
108 2772	346
108 2773	322
108 2774	315

108 2775 314

108 2776 297

108 2777 290

108 2778 275

108 2779 253

109 155 146

109 157 114

109 176 96

109 180 238

109 227 219

109 234 105

109 253 252

109 424 156

109 724 174

109 732 766

109 735 511

109 868 1461

109 951 378

109 952 148

109 959 342

109 1079 95

109 1249 2581

109 1360 102

109 1412 1351

109 1803 1177

109 1805 221

109 1807 239

109 1808 112

109 1810 104

109 1988 135

109 2343 949

109 2346 1577

109 2350 189

109 2351 299

109 2363 761

109 2372 350

109 2595 366

109 2780 711

109 2781 586

109 2782 526

109 2783 308

109 2784 284

109 2785 256

109 2786 222

109 2787 213

109 2788 173

109 2789 165

109 2790 143

109 2791 134

109 2792 129

109 2793 125

109 2794 115

109 2795 111

109 2796 109

109 2797 94

110 55 677

110 89 2770

110 257 977

110 265 796

110 288 6955

110 289 12705

110 291 812

110 292 1452

110 294 591

110 295 1084

110 300 1651

110 314 653

110 324 713

110 325 2279

110 333 570

110 349 1271

110 367 2773

110 369 591

110 377 559

110 388 3753

110 393 1579

110 400 4017

110 409 2013

110 413 1337

110 524 607

110 679 744

110 1904 841

110 1906 796

110 1918 753

110 2089 2668

110 2091 3539

110 2092 3511

110 2094 1302

110 2097 888

110 2098 1936

110 2102 3015

110 2106 1214

110 2117 696

110 2798 1373

110 2799 1151

110 2800 1048

110 2801 1006

110 2802 959

110 2803 847

110 2804 767

110 2805 745

110 2806 732

110 2807 574

110 2808 562

110 2809 559

111 88 431

111 173 435

111 197 503

111 207 719

111 226 1079

111 233 2965

111 238 834

111 407 354

111 432 213

111 441 406

111 533 1394

111 603 355

111 709 386

111 769 294

111 857 223

111	969	988
111	986	364
111	1048	213
111	1099	1188
111	1131	538
111	1191	231
111	1988	243
111	2271	217
111	2288	350
111	2380	206
111	2459	231
111	2605	417
111	2810	947
111	2811	943
111	2812	936
111	2813	798
111	2814	554
111	2815	486
111	2816	410
111	2817	409
111	2818	372
111	2819	359
111	2820	356
111	2821	322
111	2822	316
111	2823	291
111	2824	277
111	2825	277
111	2826	259
111	2827	242

111 2828 231

111 2829 230

111 2830 226

111 2831 225

111 2832 211

112 2833 10

113 176 2372

113 198 48199

113 220 1570

113 230 744

113 234 1908

113 377 3371

113 475 8289

113 498 2124

113 510 2469

113 519 1058

113 614 1803

113 707 7810

113 710 4807

113 815 3186

113 917 7391

113 920 5783

113 928 6680

113 936 2270

113 937 1431

113 942 1663

113 946 1082

113 957 5530

113 965 1981

113 982 3589

113	1113	1369
113	1216	2114
113	1249	2999
113	1339	3905
113	1349	1493
113	1350	6064
113	1369	3498
113	2643	1009
113	2834	6708
113	2835	6591
113	2836	5756
113	2837	3115
113	2838	3036
113	2839	2367
113	2840	1829
113	2841	1701
113	2842	1646
113	2843	1531
113	2844	1302
113	2845	1249
113	2846	1158
113	2847	1137
113	2848	832
113	2849	768
113	2850	756
113	2851	738
114	77	72
114	96	22
114	99	29
114	163	542

114 176 37

114 183 52

114 203 40

114 212 156

114 227 822

114 424 25

114 533 30

114 562 88

114 599 404

114 601 24

114 706 1047

114 717 250

114 728 79

114 733 24

114 735 46

114 857 65

114 868 941

114 918 22

114 951 72

114 959 236

114 1099 24

114 1249 825

114 1360 97

114 1369 44

114 1372 200

114 1412 1323

114 1426 265

114 1639 347

114 1803 94

114 1804 175

114 1810	203
114 1814	24
114 2176	24
114 2343	388
114 2346	201
114 2347	113
114 2852	73
114 2853	50
114 2854	44
114 2855	30
114 2856	30
114 2857	29
114 2858	27
114 2859	23
114 2860	23
114 2861	22
115 96 1	
115 683 1	
115 718 1	
115 728 2	
115 992 1	
115 1160	1
115 1244	1
115 2842	3
115 2855	1
115 2862	10
115 2863	4
115 2864	3
115 2865	3
115 2866	3

115 2867	3
115 2868	3
115 2869	2
115 2870	2
115 2871	2
115 2872	2
115 2873	2
115 2874	2
115 2875	2
115 2876	2
115 2877	2
115 2878	2
115 2879	2
115 2880	2
115 2881	2
115 2882	2
115 2883	2
115 2884	2
115 2885	2
115 2886	2
115 2887	2
115 2888	2
115 2889	2
115 2890	2
115 2891	2
115 2892	2
115 2893	1
115 2894	1
115 2895	1
115 2896	1

115 2897 1
115 2898 1
115 2899 1
115 2900 1
115 2901 1
115 2902 1
116 234 185
116 248 813
116 249 268
116 267 1851
116 276 145
116 475 162
116 527 333
116 619 321
116 1613 466
116 1620 321
116 1622 145
116 1629 1378
116 1640 226
116 1941 132
116 1952 275
116 1963 929
116 2179 266
116 2226 151
116 2252 390
116 2698 211
116 2903 1184
116 2904 966
116 2905 921
116 2906 771

116	2907	652
116	2908	569
116	2909	480
116	2910	469
116	2911	438
116	2912	434
116	2913	407
116	2914	400
116	2915	387
116	2916	383
116	2917	295
116	2918	294
116	2919	292
116	2920	267
116	2921	253
116	2922	237
116	2923	236
116	2924	222
116	2925	184
116	2926	180
116	2927	172
116	2928	170
116	2929	161
116	2930	155
116	2931	155
116	2932	134
117	51	1244
117	55	405
117	59	653
117	61	259

117 67 540

117 72 3365

117 91 641

117 96 234

117 99 192

117 100 232

117 157 430

117 159 309

117 180 385

117 187 568

117 192 180

117 193 327

117 227 243

117 253 262

117 268 693

117 294 184

117 377 223

117 429 256

117 467 196

117 717 1448

117 730 226

117 905 198

117 994 267

117 997 264

117 998 542

117 999 299

117 1001 894

117 1003 233

117 1004 488

117 1008 285

117 1014 754

117 1019 236

117 1026 203

117 1883 347

117 1892 477

117 2029 224

117 2031 357

117 2183 406

117 2554 538

117 2933 795

117 2934 365

117 2935 341

117 2936 319

117 2937 278

117 2938 267

117 2939 191

118 235 389

118 377 255

118 481 196

118 488 684

118 498 212

118 502 353

118 503 591

118 504 900

118 505 682

118 517 370

118 536 530

118 710 754

118 811 340

118 816 399

118 830 496

118 834 730

118 978 807

118	1145	432
118	1350	199
118	1406	322
118	1672	228
118	1797	645
118	1859	335
118	1870	890
118	1914	915
118	1925	257
118	2062	316
118	2744	1015
118	2940	906
118	2941	666
118	2942	653
118	2943	642
118	2944	621
118	2945	577
118	2946	380
118	2947	372
118	2948	364
118	2949	352
118	2950	346
118	2951	294
118	2952	282
118	2953	267
118	2954	265
118	2955	260

118 2956 244

118 2957 235

118 2958 229

118 2959 222

118 2960 218

118 2961 211

119 152 452

119 188 2356

119 370 304

119 503 262

119 517 139

119 724 377

119 806 122

119 812 486

119 813 224

119 820 130

119 821 178

119 824 200

119 835 140

119 841 129

119 843 159

119 917 142

119 918 398

119 920 957

119 923 594

119 930 283

119 936 621

119 943 235

119 953 641

119 1110 123

119 1112 140

119 1113 157

119 1134 247

119 1339 4920

119 1743 171

119 1994 3287

119 2172 187

119 2211 172

119 2370 138

119 2379 611

119 2654 157

119 2962 2086

119 2963 743

119 2964 431

119 2965 280

119 2966 271

119 2967 260

119 2968 213

119 2969 208

119 2970 189

119 2971 188

119 2972 182

119 2973 147

119 2974 135

119 2975 123

119 2976 123

120 65 48

120 157 76

120 191 134

120 257 441

120 288 102

120 289 1256

120 290 297

120 291 700

120 292 249

120 293 73

120 294 35

120 295 87

120 300 37

120 301 48

120 327 144

120 331 26

120 332 16

120 333 45

120 342 59

120 344 2047

120 352 20

120 377 503

120 378 14

120 403 16

120 461 35

120 475 33

120 486 23

120 498 409

120 543 19

120 548 16

120 687 43

120 693 41

120 701 47

120 704 18

120 915 30

120 1032 130

120 1059 126

120 1179 27

120 1380 22

120 1447 42

120 1459 475

120 1470 15

120 1556 320

120 1934 23

120 2977 248

120 2978 53

120 2979 24

120 2980 19

120 2981 19

120 2982 14

121 89 1040

121 161 109

121 257 54

121 283 62

121 288 381

121 289 1310

121 290 76

121 292 1430

121 295 530

121 300 581

121 301 32

121 302 116

121 306 38

121 321 16807

121 327 84

121 333 250

121 342 32

121 360 76

121 377 500

121 378 340

121 458 3170

121 461 650

121 475 116

121 518 200

121 542 49

121 641 28

121 646 57

121 683 74

121 701 260

121 802 260

121 908 48

121 966 47

121 1042 82

121 1247 48

121 1429 177

121 1447 76

121 1458 65

121 1470 73

121 2080 65

121 2176 80

121 2352 26

121 2655 79

121 2983 140

121 2984 78

121 2985 66
121 2986 60
121 2987 44
121 2988 29
121 2989 28
121 2990 27
122 212 16
122 586 14
122 588 19
122 599 16
122 610 12
122 613 16
122 625 13
122 735 16
122 863 24
122 1130 16
122 1232 22
122 1244 15
122 1422 14
122 1878 14
122 2288 17
122 2624 16
122 2991 33
122 2992 28
122 2993 23
122 2994 22
122 2995 22
122 2996 21
122 2997 20
122 2998 20

122 2999 19

122 3000 19

122 3001 18

122 3002 17

122 3003 16

122 3004 16

122 3005 16

122 3006 16

122 3007 16

122 3008 15

122 3009 15

122 3010 15

122 3011 14

122 3012 14

122 3013 14

122 3014 14

122 3015 14

122 3016 14

122 3017 14

122 3018 14

122 3019 13

122 3020 13

122 3021 13

122 3022 13

122 3023 13

122 3024 13

123 56 1266

123 64 324

123 88 16108

123 210 3566

123 225 301

123 366 1128

123 440 454

123 489 432

123 604 3642

123 691 374

123 703 960

123 768 2643

123 779 452

123 805 2439

123 808 446

123 816 820

123 1099 1160

123 1195 4991

123 1390 7877

123 1396 10944

123 1409 574

123 1546 1135

123 1858 1022

123 1988 388

123 2572 1198

123 2582 689

123 2605 377

123 2608 2287

123 2610 3731

123 2824 1223

123 3025 6756

123 3026 3406

123 3027 1339

123 3028 1193

123 3029 1102

123 3030 1096

123 3031 713

123 3032 683

123 3033 483

123 3034 475

123 3035 419

123 3036 409

123 3037 392

123 3038 365

123 3039 355

123 3040 335

123 3041 333

123 3042 323

123 3043 322

123 3044 321

124 7 102

124 63 784

124 65 191

124 70 119

124 72 16424

124 75 271

124 154 259

124 157 91

124 173 1722

124 182 215

124 187 424

124 190 892

124 198 189

124 201 98

124 229 373

124 230 156

124 377 764

124 407 1579

124 486 189

124 489 77

124 504 109

124 533 174

124 538 85

124 707 229

124 727 126

124 954 109

124 1044 89

124 1061 270

124 1072 683

124 1099 237

124 1104 166

124 1406 78

124 1713 1080

124 1788 91

124 1796 101

124 1859 557

124 2622 144

124 2747 74

124 3045 885

124 3046 374

124 3047 149

124 3048 143

124 3049 134

124 3050 129

124 3051 122

124 3052 118

124 3053 110

124 3054 88

124 3055 87

124 3056 76

125 56 132

125 65 206

125 67 102

125 88 318

125 89 74

125 166 126

125 176 321

125 227 23792

125 292 83

125 306 70

125 377 115

125 389 153

125 430 115

125 499 708

125 512 265

125 538 117

125 701 128

125 706 117

125 728 115

125 730 77

125 733 2230

125 788 76

125 959 279

125 1104 504

125 1239 152
125 1242 365
125 1249 159
125 1412 660
125 1414 150
125 1416 352
125 2014 65
125 2346 257
125 2786 115
125 3057 2237
125 3058 532
125 3059 529
125 3060 395
125 3061 255
125 3062 247
125 3063 208
125 3064 161
125 3065 142
125 3066 131
125 3067 125
125 3068 93
125 3069 80
125 3070 76
125 3071 75
125 3072 75
125 3073 71
126 55 1262
126 67 605
126 89 1366
126 159 194

126 182 748

126 184 281

126 229 795

126 256 133

126 288 432

126 289 738

126 292 672

126 295 146

126 298 253

126 300 258

126 316 163

126 321 285

126 325 282

126 328 99

126 333 193

126 342 440

126 403 704

126 456 121

126 466 114

126 481 160

126 486 263

126 498 1944

126 537 94

126 540 113

126 546 349

126 674 1868

126 680 229

126 705 99

126 903 241

126 908 134

126 982 103
126 1243 889
126 1246 105
126 1377 108
126 1400 104
126 1580 267
126 2277 278
126 2523 93
126 3074 837
126 3075 480
126 3076 440
126 3077 205
126 3078 204
126 3079 165
126 3080 157
126 3081 129
127 7 419
127 89 409
127 227 429
127 234 459
127 371 445
127 486 370
127 497 406
127 504 748
127 533 952
127 537 411
127 843 398
127 854 1140
127 918 812
127 920 560

127	951	2254
127	952	1307
127	965	690
127	1079	383
127	1246	626
127	1249	533
127	1672	2566
127	1803	831
127	1810	721
127	1927	1718
127	2062	774
127	2343	595
127	2346	588
127	2363	442
127	2372	638
127	2379	413
127	2785	526
127	2790	1574
127	2794	512
127	2797	386
127	3082	1597
127	3083	721
127	3084	689
127	3085	510
127	3086	466
127	3087	466
127	3088	460
127	3089	424
127	3090	413
127	3091	404

127 3092 403
127 3093 398
127 3094 382
127 3095 381
127 3096 381
127 3097 378
128 51 5254
128 54 470
128 72 1491
128 96 811
128 99 632
128 159 627
128 172 606
128 182 2569
128 193 1985
128 206 1117
128 211 1223
128 217 825
128 227 3369
128 453 3379
128 622 1517
128 962 532
128 1075 1554
128 1414 617
128 1416 467
128 1470 1419
128 1519 974
128 2031 752
128 2554 1735
128 2627 1003

128 2639 451

128 3098 3484

128 3099 2445

128 3100 2259

128 3101 1815

128 3102 1741

128 3103 1453

128 3104 1321

128 3105 1251

128 3106 1207

128 3107 1205

128 3108 1071

128 3109 969

128 3110 943

128 3111 836

128 3112 822

128 3113 783

128 3114 739

128 3115 638

128 3116 543

128 3117 482

128 3118 480

128 3119 443

128 3120 400

128 3121 356

128 3122 355

129 55 157

129 67 369

129 71 73

129 89 4111

129 257 484

129 265 90

129 288 953

129 289 440

129 292 267

129 293 366

129 294 96

129 295 159

129 296 608

129 299 125

129 300 693

129 302 225

129 304 122

129 323 103

129 325 195

129 333 444

129 344 704

129 352 156

129 362 1278

129 374 372

129 389 480

129 423 79

129 461 467

129 465 826

129 466 490

129 523 113

129 525 85

129 544 160

129 548 242

129 686 139

129 689 150

129 690 89

129 704 225

129 906 280

129 915 99

129 961 111

129 972 237

129 1052 80

129 1459 75

129 1463 884

129 1580 101

129 1971 176

129 3123 117

129 3124 92

129 3125 81

129 3126 78

130 55 385

130 67 519

130 71 445

130 89 610

130 157 98

130 161 132

130 241 64

130 251 87

130 257 1195

130 265 154

130 285 197

130 288 868

130 289 2835

130 290 87

130 292 402

130 293 722

130 294 145

130 295 918

130 298 218

130 299 137

130 300 96

130 311 73

130 326 88

130 333 170

130 344 101

130 349 120

130 362 855

130 378 264

130 389 420

130 461 368

130 465 273

130 523 313

130 525 101

130 532 248

130 535 141

130 680 73

130 701 156

130 906 82

130 961 78

130 972 700

130 1034 266

130 1441 108

130 1450 133

130 1463 678

130 1580 224
130 2094 206
130 2548 145
130 3127 272
130 3128 212
130 3129 181
131 154 179
131 250 106
131 418 74
131 441 158
131 757 108
131 1207 113
131 1946 84
131 2244 110
131 3003 131
131 3130 690
131 3131 353
131 3132 342
131 3133 301
131 3134 294
131 3135 286
131 3136 283
131 3137 213
131 3138 180
131 3139 179
131 3140 161
131 3141 152
131 3142 150
131 3143 145
131 3144 143

131 3145 140

131 3146 130

131 3147 128

131 3148 110

131 3149 108

131 3150 108

131 3151 108

131 3152 107

131 3153 106

131 3154 99

131 3155 97

131 3156 93

131 3157 93

131 3158 93

131 3159 93

131 3160 92

131 3161 90

131 3162 88

131 3163 87

131 3164 87

131 3165 85

131 3166 79

131 3167 78

131 3168 78

131 3169 76

131 3170 75

132 55 850

132 89 1267

132 182 1940

132 191 457

132 257 101

132 288 3310

132 289 495

132 294 113

132 295 1534

132 299 71

132 300 1027

132 301 646

132 302 170

132 306 333

132 310 10085

132 314 75

132 318 237

132 320 71

132 325 449

132 333 319

132 344 63

132 349 331

132 352 97

132 461 2260

132 464 158

132 466 248

132 498 221

132 525 98

132 528 156

132 531 66

132 532 81

132 542 181

132 543 112

132 544 66

132 545 60

132 681 228

132 686 2238

132 701 294

132 790 4162

132 797 457

132 907 337

132 1009 99

132 1455 64

132 1458 221

132 1470 109

132 2094 69

132 2176 290

132 2440 178

132 2521 116

132 3171 96

133 67 839

133 89 189

133 157 317

133 257 160

133 288 442

133 289 696

133 290 272

133 291 84

133 292 1213

133 295 115

133 300 603

133 302 80

133 306 88

133 311 116

133 325 146

133 329 117

133 332 75

133 333 212

133 340 343

133 349 377

133 461 323

133 464 74

133 466 526

133 486 105

133 498 321

133 523 337

133 528 136

133 531 223

133 543 193

133 544 135

133 646 272

133 679 509

133 681 178

133 701 285

133 704 79

133 797 198

133 802 311

133 889 778

133 972 118

133 1042 95

133 1043 89

133 1429 99

133 1451 172

133 1459 87

133 2548 156

133 2984 399

133 3172 154

133 3173 132

133 3174 105

133 3175 90

134 55 177

134 67 45

134 89 81

134 257 64

134 288 1600

134 289 588

134 292 355

134 299 200

134 308 575

134 312 96

134 317 156

134 325 77

134 326 173

134 334 38

134 344 25

134 349 36

134 386 65

134 464 41

134 466 257

134 468 33

134 528 96

134 543 143

134 544 74

134 686 662

134 701 32

134 790 93

134 915 53

134 972 27

134 1009 44

134 1032 1408

134 1034 208

134 1450 165

134 1451 131

134 1452 338

134 1596 41

134 1673 42

134 1860 25

134 2083 35

134 2094 501

134 2543 362

134 2548 287

134 2986 26

134 2990 60

134 3176 9399

134 3177 196

134 3178 121

134 3179 61

134 3180 28

134 3181 27

134 3182 27

135 2 1021

135 9 488

135 30 603

135 55 954

135 67 1040

135 72 520

135 89 5116

135 166 561

135 173 544

135 288 581

135 289 2350

135 292 856

135 296 559

135 300 711

135 352 705

135 386 594

135 466 1281

135 487 766

135 518 1736

135 544 619

135 686 493

135 790 1294

135 802 543

135 906 1059

135 930 526

135 970 677

135 972 1261

135 1034 494

135 1066 1128

135 1075 666

135 1263 494

135 1300 6766

135 1714 540

135 2014 523

135 3183 2855

135 3184 1683

135 3185 1186

135 3186 825

135 3187 746

135 3188 679

135 3189 660

135 3190 598

135 3191 582

135 3192 556

135 3193 555

135 3194 527

135 3195 526

135 3196 526

135 3197 514

135 3198 514

136 55 376

136 67 178

136 89 218

136 257 506

136 288 483

136 289 1665

136 290 150

136 291 339

136 292 1587

136 293 176

136 294 551

136 295 492

136 299 270

136 301 172

136 302 351

136 305 257

136 306 124

136 309 122

136 310 330

136 314 186

136 318 174

136 320 120

136 325 258

136 326 135

136 328 145

136 329 178

136 333 278

136 342 127

136 344 142

136 349 409

136 355 201

136 386 193

136 455 163

136 456 151

136 459 155

136 465 143

136 523 139

136 535 110

136 538 618

136 679 124

136 701 244

136 891 151

136 907 124

136 913 162

136 1457 118
136 1459 376
136 2022 113
136 2523 144
136 3173 287
136 3199 132
137 55 1108
137 56 1512
137 58 1569
137 64 409
137 65 536
137 67 1085
137 70 345
137 71 525
137 88 442
137 154 2178
137 167 897
137 227 499
137 246 2213
137 261 862
137 289 897
137 292 338
137 298 665
137 300 367
137 306 429
137 316 1944
137 418 320
137 525 529
137 534 529
137 701 991

137 714 295

137 757 516

137 913 306

137 980 906

137 1098 5708

137 1135 1694

137 1243 452

137 1390 399

137 1398 784

137 1424 477

137 1464 408

137 1556 320

137 1768 348

137 1781 1241

137 1934 451

137 1983 568

137 1990 653

137 2523 301

137 3057 1929

137 3200 1014

137 3201 860

137 3202 544

137 3203 502

137 3204 411

137 3205 324

137 3206 303

139 7 385

139 190 93

139 228 142

139 333 121

139 371 166

139 377 168

139 378 233

139 405 173

139 475 457

139 486 329

139 487 116

139 489 1090

139 493 117

139 498 2889

139 500 1136

139 518 158

139 519 318

139 536 869

139 704 150

139 706 91

139 707 94

139 726 183

139 923 816

139 930 1625

139 953 615

139 955 138

139 978 769

139 1044 1132

139 1131 215

139 1180 1086

139 1186 255

139 1379 97

139 1650 310

139 1672 3448

139 1870 193

139 2379 148

139 2384 95

139 2940 245

139 3236 1535

139 3237 751

139 3238 634

139 3239 384

139 3240 369

139 3241 367

139 3242 256

139 3243 186

139 3244 176

139 3245 134

139 3246 117

139 3247 110

140 65 622

140 126 1184

140 154 1559

140 170 486

140 190 2044

140 195 538

140 196 540

140 199 714

140 203 517

140 207 1058

140 211 400

140 216 1175

140 217 906

140 223 465

140 225 1625

140 237 504

140 416 1502

140 418 788

140 424 874

140 431 454

140 445 677

140 503 686

140 599 422

140 602 611

140 622 716

140 1090 498

140 1097 744

140 1151 751

140 1418 449

140 1633 643

140 1949 491

140 1964 436

140 2538 453

140 2632 427

140 3248 3376

140 3249 2358

140 3250 1272

140 3251 891

140 3252 801

140 3253 792

140 3254 719

140 3255 695

140 3256 562

140 3257 561

140 3258 523

140 3259 516

140 3260 480

140 3261 419

140 3262 415

140 3263 414

141 51 548

141 54 61

141 72 40

141 86 67

141 89 71

141 159 124

141 163 36

141 173 57

141 190 135

141 227 160

141 298 89

141 511 129

141 539 61

141 599 185

141 715 291

141 734 158

141 735 79

141 851 243

141 874 39

141 905 112

141 917 106

141 928 106

141 959 79

141 961 157

141	1075	102
141	1110	69
141	1122	47
141	1249	80
141	1366	79
141	1369	45
141	1413	95
141	1643	73
141	1726	194
141	2343	101
141	2444	136
141	2531	54
141	2945	58
141	3201	122
141	3264	544
141	3265	106
141	3266	76
141	3267	59
141	3268	57
141	3269	55
141	3270	47
141	3271	43
141	3272	43
141	3273	41
141	3274	40
141	3275	38
142	7	53
142	36	75
142	51	221
142	59	470

142 67 120

142 72 61

142 159 1533

142 429 384

142 441 69

142 466 75

142 599 93

142 627 128

142 868 59

142 880 122

142 997 178

142 1083 498

142 1203 173

142 1213 91

142 1504 94

142 1844 129

142 2289 59

142 2488 83

142 2531 217

142 2938 69

142 3201 108

142 3276 557

142 3277 252

142 3278 246

142 3279 237

142 3280 207

142 3281 179

142 3282 158

142 3283 146

142 3284 141

142 3285 114

142 3286 106

142 3287 104

142 3288 94

142 3289 87

142 3290 86

142 3291 68

142 3292 68

142 3293 67

142 3294 66

142 3295 57

142 3296 56

142 3297 55

142 3298 53

142 3299 52

142 3300 50

143 155 995

143 157 2598

143 161 2140

143 192 7007

143 227 2705

143 251 1156

143 258 1071

143 262 1140

143 292 926

143 359 1179

143 389 1336

143 511 2398

143 599 2539

143 614 1614

143 641 3681

143 717 2542

143 734 1210

143 959 1309

143 975 1311

143 986 9444

143 987 926

143 992 912

143 1150 2255

143 1244 2678

143 1412 1705

143 1414 982

143 1427 2160

143 1613 2123

143 1833 1556

143 2051 1089

143 2065 1182

143 2079 2625

143 2347 1886

143 2442 3766

143 2444 1500

143 2463 3662

143 2630 1637

143 2861 1849

143 3301 4202

143 3302 3382

143 3303 2746

143 3304 1638

143 3305 1634

143 3306 1456

143 3307 1308

143 3308 1166

143 3309 977

143 3310 937

143 3311 935

143 3312 934

144 55 82

144 67 100

144 89 381

144 157 1874

144 161 39

144 257 202

144 285 372

144 288 938

144 289 1508

144 292 640

144 294 27

144 295 768

144 299 55

144 300 224

144 301 139

144 302 80

144 306 62

144 312 25

144 314 160

144 317 49

144 322 24

144 327 41

144 328 39

144 332 25

144 333 26

144 349 77

144 455 28

144 458 45

144 466 234

144 475 32

144 525 25

144 528 28

144 644 60

144 652 23

144 701 102

144 907 26

144 913 71

144 1037 128

144 1444 36

144 1596 30

144 1825 101

144 2083 42

144 2094 42

144 2220 208

144 3313 49

144 3314 46

144 3315 34

144 3316 30

144 3317 25

144 3318 25

145 89 718

145 97 447

145 191 625

145 204 407

145 257 558

145 288 801

145 290 335

145 291 837

145 292 761

145 293 688

145 294 534

145 300 2042

145 301 416

145 302 622

145 304 1300

145 305 441

145 311 469

145 313 659

145 316 511

145 324 321

145 333 818

145 342 336

145 344 411

145 352 358

145 430 553

145 456 495

145 466 490

145 528 729

145 548 1351

145 674 391

145 689 666

145 701 520

145 704 896

145 903 479

145 1047 347

145 1106 451

145 1447 353

145 1511 539

145 1602 767

145 1934 1025

145 2130 481

145 2176 404

145 2852 1564

145 2977 2047

145 3081 1043

145 3171 385

145 3319 791

145 3320 726

145 3321 343

145 3322 334

146 67 476

146 89 2162

146 97 200

146 257 1640

146 288 23830

146 289 2426

146 290 1003

146 291 422

146 292 221

146 294 306

146 295 4291

146 298 457

146 300 5410

146 301 200

146 302 324

146 306 1364

146 311 187

146 314 229

146 318 820

146 320 207

146 325 312

146 330 229

146 333 587

146 344 2946

146 349 1437

146 386 919

146 461 1624

146 466 1876

146 484 269

146 498 639

146 528 414

146 543 223

146 545 235

146 546 193

146 701 1584

146 1444 176

146 1450 231

146 1613 261

146 1904 1937

146 2091 1006

146 2092 4480

146 2094 4506

146 2106 491

146 2799 782

146 2803 551

146 2990 2162

146 3323 974

146 3324 525

146 3325 395

146 3326 290

147 55 502

147 67 1165

147 155 3649

147 209 1105

147 257 496

147 283 164

147 288 971

147 289 20876

147 291 4725

147 292 2722

147 293 2126

147 295 1709

147 299 427

147 302 813

147 305 2107

147 306 189

147 310 313

147 316 1016

147 318 1254

147 320 888

147 325 1993

147 326 674

147 329 174

147 333 2410

147 349 677

147 378 1670

147 498 328

147 523 1596

147 525 424

147 537 208

147 538 172

147 540 352

147 542 947

147 679 859

147 701 706

147 790 203

147 906 226

147 972 228

147 1052 1335

147 1243 302

147 1580 2294

147 1684 902

147 2523 702

147 3327 1586

147 3328 1346

147 3329 1048

147 3330 801

147 3331 640

147 3332 524

147 3333 329

148 63 175

148 206 214

148 233 170

148 429 430

148 436 336

148 512 244

148 567 345

148 619 227

148 632 195

148 785 223

148 1118 214

148 1206 245

148 1409 282

148 1943 298

148 1986 202

148 2693 288

148 2818 182

148 3334 947

148 3335 833

148 3336 783

148 3337 381

148 3338 378

148 3339 352

148 3340 351

148 3341 280

148 3342 267

148 3343 259

148 3344 259

148 3345 255

148 3346 240

148 3347 236

148 3348 236

148 3349 223

148 3350 221

148 3351 212

148 3352 212

148 3353 210

148 3354 203

148 3355 200

148 3356 198

148 3357 192

148 3358 192

148 3359 183

148 3360 183

148 3361 174

148 3362 172

148 3363 166

148 3364 164

148 3365 164

148 3366 164

149 71 265

149 89 2178

149 159 606

149 173 1350

149 198 1300

149 230 337

149 291 183

149 300 233

149 302 499

149 306 558

149 342 339

149 366 501

149 378 1779

149 436 434

149 486 491

149 488 363

149 492 245

149 497 726

149 498 6610

149 518 260

149 536 615

149 546 221

149 572 622

149 680 796

149 687 182

149 697 178

149 966 196

149 972 1110

149 982 294

149 1041 233

149 1044 628

149 1052 710

149 1116 343

149 1130 317

149 1400 189

149 1470 456

149 1857 618

149 1934 201

149 2352 307

149 2545 358

149 2786 255

149 2839 878

149 3367 1559

149 3368 1108

149 3369 486

149 3370 313

149 3371 260

149 3372 210

149 3373 175

149 3374 164

150 89 187

150 155 114

150 157 123

150 161 54

150 190 58

150 227 161

150 251 47

150 257 843

150 265 190

150 283 40

150 288 61

150 289 111

150 291 60

150 294 45

150 295 506

150 299 185

150 301 60

150 302 73

150 306 46

150 320 78

150 321 55

150 325 76

150 329 68

150 331 73

150 333 49

150 340 51

150 344 82

150 349 52

150 378 52

150 455 42

150 468 52

150 472 46

150 528 79

150 646 55

150 701 115

150 966 40

150 986 50

150 1244 164

150 1445 49

150 1447 250

150 1459 77

150 1601 55

150 2176 56

150 2184 140

150 2439 79

150 2545 45

150 2861 40

150 3375 56

150 3376 40

150 3377 39

151 65 121

151 155 57

151 184 135

151 198 50

151 204 70

151 283 329

151 328 196

151 342 56

151 355 150

151 378 77

151 417 121

151 438 773

151 461 549

151 498 582

151 542 80

151 705 351

151 714 102

151 966 56

151 1681 343

151 1760 96

151 2080 148

151 2352 87

151 2545 184

151 2655 52

151 2983 104

151 2986 195

151 2988 279

151 3173 167

151 3205 123

151 3376 97

151 3377 174

151 3378 282

151 3379 163

151 3380 147

151 3381 132

151 3382 92

151 3383 92

151 3384 89

151 3385 84

151 3386 78

151 3387 71

151 3388 68

151 3389 65

151 3390 62

151 3391 61

151 3392 57

151 3393 54

151 3394 53

151 3395 53

151 3396 51

152 89 321

152 207 557

152 220 565

152 227 139

152 234 173

152 289 534

152 293 224

152 298 225

152 300 229

152 318 206

152 324 345

152 325 344

152 333 354

152 344 212

152 403 172

152 412 985

152 420 208

152 425 158

152 458 336

152 461 446

152 466 889

152 481 215

152 498 645

152 528 128

152 533 228

152 540 135

152 680 200

152 704 3138

152 735 133

152 797 128

152 859 358

152 903 208

152 1048 452

152 1244 108

152 1246 117

152 1400 629

152 1513 681

152 1684 142

152 1807 332

152 2435 320

152 2788 203

152 2839 139

152 3397 269

152 3398 225

152 3399 200
152 3400 196
152 3401 185
152 3402 140
152 3403 138
152 3404 122
153 90 159
153 154 98
153 159 479
153 162 268
153 174 236
153 188 151
153 189 1016
153 190 107
153 215 202
153 223 147
153 225 138
153 228 141
153 370 302
153 416 866
153 423 149
153 506 772
153 518 201
153 854 397
153 1094 283
153 1119 114
153 1151 495
153 1375 259
153 1376 217
153 1410 99

153 2006 136
153 2370 125
153 2374 196
153 2775 242
153 2794 113
153 2962 262
153 3405 609
153 3406 463
153 3407 235
153 3408 230
153 3409 227
153 3410 206
153 3411 199
153 3412 191
153 3413 183
153 3414 181
153 3415 153
153 3416 151
153 3417 148
153 3418 141
153 3419 131
153 3420 119
153 3421 111
153 3422 100
153 3423 100
153 3424 98
154 15 13
154 18 13
154 41 25
154 198 10

154 209 12

154 227 14

154 289 15

154 318 17

154 378 89

154 412 12

154 472 20

154 486 21

154 500 33

154 517 11

154 518 16

154 537 15

154 706 31

154 707 16

154 712 35

154 811 17

154 830 243

154 917 12

154 923 45

154 930 73

154 952 18

154 953 26

154 955 17

154 966 12

154 1104 25

154 1114 15

154 1116 22

154 1185 11

154 1339 16

154 1672 11

154 1689 21

154 1914 19

154 1917 15

154 2014 17

154 2100 23

154 2344 18

154 2940 36

154 2956 22

154 2983 18

154 3197 15

154 3239 13

154 3425 25

154 3426 19

154 3427 18

154 3428 13

154 3429 12

155 159 1073

155 206 720

155 441 642

155 599 1768

155 732 833

155 861 681

155 864 859

155 866 1252

155 870 573

155 875 3529

155 1083 2496

155 1206 3366

155 1217 889

155 1227 718

155 1244	678
155 1360	1691
155 1534	2074
155 1703	622
155 1878	1247
155 1889	1947
155 2481	1852
155 2624	707
155 3354	647
155 3430	4062
155 3431	3040
155 3432	2808
155 3433	2635
155 3434	2371
155 3435	1791
155 3436	1459
155 3437	1384
155 3438	1115
155 3439	1074
155 3440	1067
155 3441	1048
155 3442	893
155 3443	889
155 3444	879
155 3445	864
155 3446	841
155 3447	798
155 3448	786
155 3449	734
155 3450	723

155 3451 643

155 3452 638

155 3453 628

155 3454 626

155 3455 607

155 3456 592

156 65 104

156 88 26

156 89 18

156 154 748

156 157 31

156 159 5

156 163 530

156 170 185

156 171 7

156 190 31

156 196 5

156 207 2308

156 212 12

156 220 4

156 221 449

156 227 305

156 295 8

156 344 6

156 355 17

156 418 15

156 436 121

156 455 12

156 511 8

156 533 1218

156 538 6

156 604 11

156 735 8

156 961 21

156 1048 61

156 1377 5

156 1512 213

156 1515 10

156 1687 17

156 1806 76

156 1958 11

156 2855 17

156 3057 26

156 3457 24

156 3458 21

156 3459 11

156 3460 10

156 3461 9

156 3462 9

156 3463 9

156 3464 8

156 3465 8

156 3466 7

156 3467 7

156 3468 7

156 3469 6

157 163 92

157 176 109

157 228 445

157 300 255

157 466 73

157 607 73

157 630 84

157 706 509

157 710 58

157 712 88

157 868 161

157 917 241

157 918 421

157 928 55

157 947 66

157 952 151

157 959 100

157 1358 108

157 1360 64

157 1366 258

157 1369 65

157 1372 882

157 1412 469

157 1513 107

157 1639 72

157 1703 213

157 1807 67

157 1808 105

157 1810 98

157 2342 53

157 2343 125

157 2347 611

157 2782 68

157 2784 55

157 2787 258

157 2834 215

157 2973 69

157 3470 225

157 3471 139

157 3472 116

157 3473 107

157 3474 80

157 3475 73

157 3476 72

157 3477 71

157 3478 68

157 3479 64

157 3480 62

157 3481 55

157 3482 53

158 71 615

158 290 227

158 291 287

158 294 114

158 305 243

158 311 171

158 318 533

158 325 204

158 342 170

158 359 252

158 374 843

158 386 331

158 465 117

158 466 156

158 479 145

158 528 190

158 544 118

158 548 329

158 679 3779

158 681 155

158 686 212

158 797 346

158 909 477

158 915 151

158 1082 333

158 1450 176

158 1458 470

158 1601 359

158 1684 188

158 2102 325

158 2990 162

158 3081 265

158 3321 220

158 3483 2146

158 3484 931

158 3485 854

158 3486 836

158 3487 803

158 3488 478

158 3489 317

158 3490 317

158 3491 308

158 3492 230

158 3493 219

158 3494 203

158 3495 183

158 3496 143

158 3497 135

158 3498 128

158 3499 110

159 64 2016

159 65 541

159 67 4115

159 70 930

159 72 685

159 90 917

159 155 1226

159 173 4171

159 182 1154

159 189 1333

159 220 559

159 221 950

159 241 796

159 247 803

159 257 1416

159 265 769

159 279 3619

159 288 961

159 292 1176

159 294 719

159 296 2094

159 299 1103

159 301 1935

159 302 3275

159 310 3131

159 316 6307

159 332 496

159 354 582

159 377 1873

159 467 3304

159 504 1159

159 511 605

159 548 584

159 674 522

159 701 621

159 792 3105

159 906 458

159 1053 1841

159 1377 723

159 1379 1388

159 1380 1387

159 1470 580

159 1505 3529

159 1601 1301

159 1937 858

159 2521 463

159 3500 1596

159 3501 1433

159 3502 964

159 3503 465

161 41 1048

161 56 1754

161 63 1059

161 407 2169

161 487 34180

161 503 1682

161 519 1739

161 785 4020

161 812 2031

161 815 2196

161 953 1170

161 978 1150

161 1044 1113

161 1066 6370

161 1104 1454

161 1541 1753

161 2062 1077

161 2143 2135

161 2369 1131

161 2945 1029

161 3087 1208

161 3236 2368

161 3240 1066

161 3244 1127

161 3542 4657

161 3543 4133

161 3544 3039

161 3545 2590

161 3546 2294

161 3547 1530

161 3548 1505

161 3549 1337

161 3550 1296

161 3551 1248

161 3552 1248
161 3553 1213
161 3554 1164
161 3555 1127
161 3556 1110
161 3557 1097
161 3558 1073
161 3559 1068
161 3560 1068
161 3561 1044
161 3562 1031
161 3563 1026
161 3564 1024
161 3565 1022
161 3566 1021
161 3567 1015
162 7 215
162 67 278
162 89 486
162 172 186
162 227 446
162 289 835
162 293 538
162 295 323
162 297 264
162 302 383
162 304 208
162 325 180
162 333 414
162 378 414

162 455 4664

162 457 342

162 459 336

162 462 181

162 469 545

162 475 666

162 479 309

162 542 207

162 544 537

162 718 261

162 930 252

162 1034 174

162 1109 255

162 1114 660

162 1580 231

162 1781 199

162 2021 236

162 2094 273

162 2523 220

162 2645 694

162 2801 176

162 3200 251

162 3321 171

162 3568 646

162 3569 557

162 3570 324

162 3571 306

162 3572 303

162 3573 287

162 3574 287

162 3575 283

162 3576 241

162 3577 228

162 3578 197

162 3579 186

162 3580 182

163 344 368

163 356 845

163 360 416

163 892 214

163 1447 378

163 1459 541

163 2436 769

163 2439 377

163 2442 432

163 2447 792

163 2449 352

163 2451 246

163 2453 328

163 2454 235

163 2455 902

163 2456 339

163 2457 313

163 2467 414

163 2468 369

163 3581 1444

163 3582 876

163 3583 769

163 3584 704

163 3585 560

163	3586	528
163	3587	526
163	3588	489
163	3589	384
163	3590	373
163	3591	366
163	3592	365
163	3593	358
163	3594	342
163	3595	338
163	3596	336
163	3597	321
163	3598	312
163	3599	292
163	3600	291
163	3601	276
163	3602	270
163	3603	262
163	3604	258
163	3605	236
163	3606	232
163	3607	215
163	3608	213
163	3609	209
163	3610	209
163	3611	205
164	152	13706
164	154	9887
164	157	1357
164	159	1747

164 188 1917

164 190 2490

164 198 1517

164 204 8072

164 215 2021

164 217 2257

164 226 2254

164 316 5236

164 602 4141

164 614 14967

164 615 1018

164 622 980

164 683 4421

164 697 1230

164 920 5058

164 964 8744

164 1116 7700

164 1118 1806

164 1122 1925

164 1131 4687

164 1174 1230

164 1375 1837

164 1378 4354

164 1643 28151

164 1795 1054

164 1875 8850

164 1976 973

164 2006 2143

164 2206 1496

164 2352 1126

164 2775 1187
164 3105 2617
164 3106 1365
164 3272 965
164 3612 5719
164 3613 3996
164 3614 2030
164 3615 1957
164 3616 1954
164 3617 1678
164 3618 1671
164 3619 1515
164 3620 1482
164 3621 1376
164 3622 1254
164 3623 945
166 88 74
166 96 123
166 159 985
166 173 379
166 192 278
166 197 225
166 199 158
166 200 202
166 205 669
166 212 98
166 223 371
166 227 285
166 229 125
166 316 160

166 346 144

166 355 363

166 366 76

166 429 498

166 474 123

166 530 104

166 533 161

166 618 995

166 734 264

166 851 619

166 859 79

166 1249 325

166 1789 86

166 1843 72

166 1957 90

166 1985 74

166 1989 138

166 2277 77

166 2595 82

166 2629 236

166 2893 74

166 3264 961

166 3665 249

166 3666 204

166 3667 185

166 3668 172

166 3669 149

166 3670 128

166 3671 122

166 3672 117

166 3673 89

166 3674 88

166 3675 82

166 3676 75

166 3677 73

166 3678 72

168 88 573

168 107 575

168 154 234

168 440 2145

168 622 313

168 629 905

168 703 353

168 748 649

168 757 195

168 1097 236

168 1109 271

168 1163 1037

168 1236 292

168 1239 357

168 1627 265

168 1770 200

168 1943 1178

168 1945 1676

168 1953 318

168 1956 756

168 1957 245

168 1964 846

168 1965 281

168 1983 546

168 1990 304
168 2279 224
168 2398 280
168 2535 208
168 2542 332
168 2582 288
168 2605 376
168 3685 2070
168 3686 750
168 3687 532
168 3688 510
168 3689 481
168 3690 471
168 3691 439
168 3692 439
168 3693 397
168 3694 384
168 3695 338
168 3696 325
168 3697 287
168 3698 275
168 3699 271
168 3700 232
168 3701 225
168 3702 207
168 3703 198
170 154 909
170 234 1072
170 503 1292
170 504 455

170 512 1325

170 517 2491

170 614 731

170 707 1184

170 758 1541

170 768 679

170 815 509

170 841 1332

170 842 1407

170 843 2204

170 845 724

170 854 1355

170 917 1365

170 918 1695

170 923 1344

170 964 856

170 1044 643

170 1099 1058

170 1119 1955

170 1131 1127

170 1281 941

170 1369 569

170 1521 452

170 1561 1150

170 1812 849

170 1974 900

170 2148 837

170 2151 497

170 2369 573

170 2380 648

170 2594 1424

170 2744 734

170 2971 687

170 3717 1534

170 3718 1234

170 3719 1215

170 3720 1193

170 3721 1188

170 3722 1102

170 3723 848

170 3724 788

170 3725 718

170 3726 659

170 3727 608

170 3728 582

170 3729 478

171 65 121

171 88 115

171 154 1594

171 170 61

171 190 145

171 207 398

171 220 46

171 228 96

171 229 111

171 237 98

171 250 49

171 288 67

171 331 93

171 412 55

171 419 172

171 429 161

171 440 102

171 445 68

171 498 117

171 538 80

171 547 198

171 703 77

171 757 137

171 768 54

171 816 83

171 1179 74

171 1195 119

171 1400 113

171 1412 97

171 1512 52

171 1518 133

171 1609 66

171 1632 181

171 1639 56

171 1934 124

171 2125 92

171 2137 133

171 2271 139

171 2521 86

171 2582 195

171 2587 89

171 2602 106

171 2608 159

171 3030 141

171 3411 81
171 3707 94
171 3730 156
171 3731 69
171 3732 48
171 3733 48
172 7 249
172 80 456
172 154 272
172 163 929
172 167 188
172 173 1175
172 190 538
172 196 179
172 207 216
172 226 229
172 228 664
172 230 517
172 296 442
172 298 567
172 424 241
172 432 229
172 441 762
172 489 468
172 526 184
172 703 326
172 716 221
172 768 236
172 857 208
172 903 306

172 969 238

172 1014 234

172 1044 216

172 1075 420

172 1099 778

172 1206 619

172 1394 605

172 1397 194

172 1400 696

172 1406 276

172 1409 439

172 1410 240

172 1804 273

172 1806 324

172 1814 203

172 2265 388

172 2614 180

172 2821 318

172 3238 247

172 3333 659

172 3734 493

172 3735 324

172 3736 287

172 3737 198

172 3738 194

172 3739 184

173 154 363

173 159 272

173 163 248

173 173 578

173 190 174

173 207 291

173 210 295

173 215 212

173 225 196

173 227 206

173 412 179

173 418 515

173 422 368

173 424 970

173 428 214

173 429 1104

173 436 187

173 439 965

173 441 694

173 447 179

173 498 250

173 599 642

173 705 310

173 707 183

173 714 949

173 726 277

173 857 426

173 868 2401

173 903 3050

173 959 187

173 1185 271

173 1400 338

173 1412 366

173 1672 170

173 1755 211

173 1766 330

173 1814 219

173 2430 308

173 2608 578

173 2632 595

173 2786 319

173 3391 287

173 3740 863

173 3741 434

173 3742 311

173 3743 294

173 3744 218

173 3745 208

173 3746 207

173 3747 177

174 65 1098

174 184 137

174 191 209

174 204 195

174 206 146

174 217 876

174 220 234

174 229 182

174 329 95

174 403 132

174 463 93

174 511 179

174 683 3352

174 728 350

174 868 76

174 907 286

174 945 68

174 982 69

174 1043 111

174 1116 239

174 1375 497

174 1377 298

174 1415 101

174 1639 164

174 1643 868

174 1866 98

174 2051 100

174 2121 79

174 2436 98

174 2442 230

174 2453 137

174 2783 99

174 2834 180

174 3097 177

174 3171 133

174 3427 271

174 3466 119

174 3617 454

174 3748 1913

174 3749 335

174 3750 335

174 3751 193

174 3752 125

174 3753 114

174 3754 107
174 3755 94
174 3756 92
174 3757 90
174 3758 83
174 3759 80
175 257 797
175 265 2289
175 289 2160
175 344 5592
175 356 2167
175 360 3003
175 364 1333
175 461 620
175 463 831
175 465 820
175 544 763
175 679 5817
175 951 1787
175 1447 2413
175 1459 4494
175 1489 1565
175 1696 984
175 1782 917
175 2051 2099
175 2436 3200
175 2437 1328
175 2439 4363
175 2442 3255
175 2443 2064

175 2449 1824

175 2451 612

175 2453 7709

175 2454 4458

175 2455 767

175 2457 1195

175 2467 1360

175 2468 1903

175 3581 601

175 3583 745

175 3585 1236

175 3588 7458

175 3590 660

175 3592 1056

175 3593 575

175 3598 970

175 3600 2365

175 3603 624

175 3606 1127

175 3760 6180

175 3761 1498

175 3762 809

175 3763 782

175 3764 750

175 3765 680

175 3766 635

176 40 543

176 51 4266

176 59 521

176 72 2448

176 154 396

176 170 396

176 173 1168

176 223 1165

176 225 4364

176 227 2861

176 228 812

176 233 1040

176 424 205

176 429 638

176 432 185

176 440 247

176 441 239

176 450 487

176 612 241

176 748 459

176 965 357

176 969 313

176 1075 556

176 1090 599

176 1219 315

176 1242 190

176 1376 530

176 1390 270

176 1803 164

176 1878 171

176 1886 325

176 2541 184

176 2584 718

176 2611 254

176 2637 202

176 2938 229

176 3030 370

176 3110 616

176 3258 989

176 3281 201

176 3411 662

176 3576 457

176 3732 777

176 3754 320

176 3767 814

176 3768 755

176 3769 576

176 3770 517

176 3771 223

176 3772 222

177 152 935

177 154 593

177 163 2022

177 188 2208

177 206 496

177 220 615

177 227 4781

177 250 569

177 370 616

177 407 2026

177 429 767

177 436 2577

177 506 390

177 599 1284

177 607 633

177 610 460

177 724 1173

177 733 466

177 854 539

177 917 3496

177 928 402

177 936 465

177 943 422

177 957 672

177 959 444

177 1110 453

177 1113 654

177 1134 511

177 1353 523

177 1369 459

177 1411 652

177 1412 1134

177 1418 379

177 1426 2329

177 1427 1128

177 1639 657

177 1893 378

177 1941 970

177 2343 367

177 2369 449

177 2381 749

177 2624 8008

177 3421 861

177 3773 1163

177 3774 741

177 3775 734

177 3776 705

177 3777 650

177 3778 427

177 3779 368

178 188 1110

178 218 274

178 383 328

178 607 635

178 812 340

178 820 450

178 821 387

178 824 621

178 837 613

178 868 499

178 917 1227

178 949 600

178 1110 1131

178 1134 1283

178 1216 646

178 1264 325

178 1417 297

178 1425 418

178 1426 392

178 1528 575

178 1533 264

178 1639 524

178 1901 651

178 1992 356

178 1994 630
178 2370 397
178 2624 386
178 2746 803
178 2842 350
178 2964 292
178 2969 364
178 3264 482
178 3354 453
178 3444 459
178 3456 309
178 3660 388
178 3780 1132
178 3781 577
178 3782 571
178 3783 405
178 3784 362
178 3785 347
178 3786 345
178 3787 336
178 3788 322
178 3789 301
178 3790 299
178 3791 283
178 3792 282
178 3793 268
179 7 270
179 67 238
179 72 171
179 89 4263

179 157 895

179 159 702

179 173 1020

179 227 188

179 230 95

179 234 232

179 288 162

179 289 3853

179 291 237

179 292 1095

179 293 261

179 300 85

179 318 68

179 333 240

179 357 94

179 371 460

179 377 2271

179 378 86

179 420 221

179 461 109

179 464 109

179 466 257

179 475 458

179 481 97

179 486 58

179 498 70

179 499 97

179 523 1192

179 524 232

179 536 257

179 544 339

179 680 64

179 704 235

179 1034 321

179 1231 71

179 1406 176

179 1673 146

179 1988 180

179 2102 66

179 2432 2184

179 2821 181

179 2978 170

179 3794 492

179 3795 274

179 3796 257

179 3797 164

180 56 29

180 159 32

180 162 73

180 226 20

180 233 21

180 517 17

180 755 20

180 769 16

180 821 19

180 1046 20

180 1192 42

180 1205 29

180 1260 19

180 1376 20

180 1410	24
180 2196	26
180 2654	41
180 2763	19
180 3028	27
180 3417	18
180 3722	22
180 3798	114
180 3799	64
180 3800	56
180 3801	50
180 3802	43
180 3803	40
180 3804	39
180 3805	38
180 3806	33
180 3807	31
180 3808	29
180 3809	28
180 3810	24
180 3811	24
180 3812	22
180 3813	22
180 3814	20
180 3815	20
180 3816	20
180 3817	20
180 3818	19
180 3819	19
180 3820	18

180 3821 18

180 3822 18

180 3823 18

180 3824 17

180 3825 17

180 3826 16

181 173 3873

181 187 804

181 190 2253

181 227 95

181 234 565

181 245 620

181 498 284

181 517 1277

181 758 428

181 857 142

181 868 142

181 1091 666

181 1104 996

181 1406 601

181 1513 373

181 1786 83

181 1788 739

181 1796 80

181 1797 1810

181 2735 33

181 2743 33

181 2747 1776

181 3047 79

181 3827 3065

181 3828 1139

181 3829 751

181 3830 338

181 3831 206

181 3832 149

181 3833 143

181 3834 114

181 3835 105

181 3836 91

181 3837 90

181 3838 88

181 3839 78

181 3840 59

181 3841 48

181 3842 44

181 3843 42

181 3844 42

181 3845 41

181 3846 39

181 3847 39

181 3848 32

181 3849 30

181 3850 28

181 3851 28

181 3852 22

181 3853 17

182 69 427

182 258 220

182 297 361

182 333 814

182 344 470

182 474 1270

182 885 259

182 903 702

182 961 720

182 966 407

182 986 420

182 992 331

182 1220	235
182 1382	314
182 1432	221
182 1772	293
182 2047	424
182 2813	231
182 3046	660
182 3307	516
182 3854	1054
182 3855	1041
182 3856	710
182 3857	606
182 3858	594
182 3859	552
182 3860	548
182 3861	502
182 3862	487
182 3863	481
182 3864	451
182 3865	437
182 3866	409
182 3867	386

182 3868 381

182 3869 377

182 3870 375

182 3871 341

182 3872 339

182 3873 319

182 3874 317

182 3875 309

182 3876 299

182 3877 285

182 3878 263

182 3879 260

182 3880 237

182 3881 225

182 3882 225

182 3883 219

183 59 220

183 61 140

183 67 497

183 69 129

183 72 3571

183 77 123

183 99 290

183 154 585

183 157 213

183 159 844

183 163 374

183 172 156

183 193 166

183 215 564

183 220 261

183 221 159

183 420 184

183 429 426

183 511 125

183 533 212

183 562 281

183 575 118

183 962 134

183 1072 357

183 1083 182

183 1085 165

183 1098 169

183 1118 138

183 1122 135

183 1384 123

183 1412 541

183 1713 161

183 2531 360

183 2559 232

183 3201 254

183 3280 500

183 3364 154

183 3884 517

183 3885 419

183 3886 373

183 3887 356

183 3888 293

183 3889 274

183 3890 247

183 3891 145

183 3892 135

183 3893 132

183 3894 128

183 3895 126

183 3896 120

184 53 160

184 64 242

184 67 94

184 70 104

184 72 175

184 81 101

184 89 231

184 152 110

184 154 282

184 163 98

184 166 138

184 173 740

184 182 157

184 186 146

184 190 137

184 238 98

184 378 336

184 418 598

184 423 369

184 506 428

184 518 114

184 522 144

184 689 289

184 757 223

184 930 211

184 961 324

184 1097 185

184 1098 1176

184 1104 85

184 1109 236

184 1125 91

184 1167 97

184 1243 261

184 1400 86

184 1934 104

184 1983 251

184 1990 366

184 3057 249

184 3201 353

184 3259 277

184 3280 233

184 3897 442

184 3898 355

184 3899 298

184 3900 270

184 3901 252

184 3902 202

184 3903 144

184 3904 122

184 3905 85

185 51 49

185 59 73

185 61 123

185 64 24

185 72 100

185 159 163

185 187 69

185 193 27

185 486 42

185 504 53

185 851 94

185 861 111

185 864 129

185 877 122

185 997 30

185 998 36

185 1001 141

185 1013 25

185 1014 124

185 1064 45

185 1406 37

185 2018 27

185 2343 87

185 2358 127

185 2521 31

185 2937 69

185 3371 21

185 3469 45

185 3570 29

185 3906 289

185 3907 252

185 3908 172

185 3909 131

185 3910 127

185 3911 75

185 3912 67

185 3913 60

185 3914 58

185 3915 34

185 3916 32

185 3917 31

185 3918 28

185 3919 28

185 3920 27

185 3921 26

185 3922 25

185 3923 24

185 3924 23

185 3925 22

185 3926 22

186 89 11

186 190 9

186 198 19

186 220 9

186 230 31

186 288 46

186 289 77

186 291 562

186 292 15

186 293 93

186 300 451

186 302 250

186 306 17

186 333 6580

186 344 55

186 377 74

186 378 4974

186 412 9

186 464 12

186 466 99

186 481 147

186 498 1125

186 499 237

186 518 301

186 523 86

186 525 63

186 680 32

186 681 10

186 687 465

186 693 508

186 704 1017

186 917 13

186 966 33

186 1043 134

186 1052 52

186 1186 45

186 1455 46

186 1672 129

186 2352 32

186 2619 21

186 2839 27

186 3124 184

186 3794 34

186 3927 117

186 3928 67

186 3929 62

186 3930 20

186 3931 15

186 3932 14

186 3933 14

187 51 295

187 62 246

187 65 559

187 72 807

187 91 391

187 98 729

187 152 2114

187 159 218

187 163 1234

187 186 469

187 187 335

187 193 763

187 227 877

187 562 409

187 599 1308

187 601 192

187 717 721

187 730 187

187 852 223

187 959 231

187 993 1060

187 997 545

187 998 196

187 1008 399

187	1013	248
187	1062	189
187	1414	177
187	1425	182
187	1795	217
187	1824	245
187	1886	962
187	1891	241
187	2609	317
187	2616	430
187	3201	1146
187	3264	2838
187	3308	284
187	3469	185
187	3768	203
187	3777	366
187	3920	195
187	3934	1674
187	3935	1432
187	3936	676
187	3937	411
187	3938	309
187	3939	259
187	3940	228
187	3941	202
187	3942	190
188	2342	3
188	3470	1
189	7	553
189	173	602

189 190 944

189 198 418

189 226 375

189 230 679

189 233 840

189 234 306

189 377 1769

189 378 872

189 436 417

189 486 1575

189 489 258

189 492 620

189 493 430

189 494 462

189 496 241

189 498 1668

189 499 358

189 512 323

189 514 313

189 517 1122

189 540 698

189 614 990

189 707 446

189 808 588

189 851 399

189 856 435

189 965 304

189 978 1176

189 982 1199

189 1044 310

189 1116 1511

189 1118 888

189 1131 1066

189 1145 285

189 1854 298

189 1873 342

189 2014 524

189 2206 711

189 2612 620

189 2744 384

189 2843 412

189 2846 282

189 3240 267

189 3621 460

189 3943 485

189 3944 334

189 3945 314

189 3946 235

190 67 631

190 289 3199

190 292 454

190 455 2341

190 803 508

190 1081 21711

190 1460 986

190 2934 711

190 3173 573

190 3557 456

190 3762 1079

190 3947 9186

190 3948	3900
190 3949	3295
190 3950	2166
190 3951	1647
190 3952	1622
190 3953	1492
190 3954	1333
190 3955	1306
190 3956	1228
190 3957	1227
190 3958	1182
190 3959	1140
190 3960	1083
190 3961	1077
190 3962	889
190 3963	866
190 3964	865
190 3965	773
190 3966	742
190 3967	735
190 3968	720
190 3969	694
190 3970	687
190 3971	654
190 3972	651
190 3973	626
190 3974	594
190 3975	558
190 3976	553
190 3977	528

190 3978 522

190 3979 516

190 3980 488

190 3981 470

190 3982 455

190 3983 437

190 3984 429

190 3985 429

191 7 32

191 51 23

191 52 28

191 66 17

191 72 463

191 75 18

191 157 15

191 171 13

191 187 101

191 190 18

191 197 18

191 220 23

191 238 28

191 868 16

191 936 16

191 1072 13

191 1090 20

191 1104 18

191 1298 13

191 1369 18

191 1372 26

191 1379 29

191 1410	14
191 1412	27
191 1713	107
191 1787	16
191 1791	34
191 2469	28
191 2735	16
191 2743	22
191 2747	26
191 3019	18
191 3047	14
191 3049	67
191 3053	51
191 3197	26
191 3302	16
191 3501	16
191 3837	17
191 3986	36
191 3987	22
191 3988	18
191 3989	18
191 3990	16
191 3991	15
191 3992	14
191 3993	13
191 3994	13
191 3995	13
191 3996	13
192 162 86	
192 2579	48

192 2586	1407
192 2971	97
192 3727	39
192 3997	5427
192 3998	2360
192 3999	1006
192 4000	663
192 4001	155
192 4002	130
192 4003	122
192 4004	85
192 4005	60
192 4006	57
192 4007	46
192 4008	44
192 4009	31
192 4010	29
192 4011	29
192 4012	25
192 4013	22
192 4014	21
192 4015	16
192 4016	16
192 4017	16
192 4018	12
192 4019	11
192 4020	10
192 4021	8
193 56	84980
193 63	961

193 64 860

193 72 385

193 84 1034

193 89 384

193 157 409

193 163 5007

193 190 467

193 198 685

193 331 4691

193 511 334

193 512 511

193 532 337

193 548 469

193 707 2356

193 959 909

193 1001 375

193 1104 1125

193 1397 912

193 1398 1571

193 1983 1520

193 2271 770

193 2521 568

193 2706 511

193 2818 420

193 2923 436

193 3158 1076

193 4022 2919

193 4023 2285

193 4024 1761

193 4025 1541

193 4026 1308

193 4027 1087

193 4028 1067

193 4029 937

193 4030 805

193 4031 678

193 4032 527

193 4033 476

193 4034 433

193 4035 432

193 4036 405

193 4037 376

193 4038 376

193 4039 366

193 4040 344

193 4041 339

193 4042 316

193 4043 313

194 53 1284

194 58 597

194 70 3020

194 81 632

194 88 1918

194 154 3871

194 163 2138

194 173 700

194 197 1676

194 216 628

194 231 3367

194 238 1147

194 416 2098

194 418 5339

194 444 1429

194 454 578

194 858 2108

194 1098 7564

194 1130 510

194 1167 521

194 1226 571

194 1424 1881

194 1735 1144

194 1747 930

194 1751 1016

194 1787 627

194 1811 549

194 1971 1015

194 1990 806

194 3046 4836

194 3057 1095

194 3164 700

194 3259 1693

194 3861 520

194 3870 1571

194 3902 3755

194 4044 5547

194 4045 2037

194 4046 1610

194 4047 1052

194 4048 1037

194 4049 964

194 4050 847

194 4051 843

194 4052 586

194 4053 555

194 4054 544

194 4055 500

194 4056 500

194 4057 478

195 154 131

195 163 47

195 190 48

195 208 11

195 210 16

195 212 324

195 220 11

195 227 1434

195 418 33

195 424 31

195 428 63

195 533 63

195 554 71

195 562 32

195 592 36

195 709 31

195 733 101

195 735 30

195 859 30

195 863 31

195 868 78

195 969 157

195 986 42
195 1098 12
195 1242 23
195 1400 93
195 1412 10
195 1416 55
195 1426 56
195 1510 242
195 2542 9
195 2606 18
195 2857 15
195 3458 21
195 3767 14
195 4058 460
195 4059 113
195 4060 95
195 4061 78
195 4062 57
195 4063 53
195 4064 51
195 4065 51
195 4066 36
195 4067 35
195 4068 23
195 4069 21
195 4070 20
195 4071 17
195 4072 10
196 51 6015
196 59 323

196 65 976

196 154 1522

196 172 733

196 182 676

196 190 7133

196 206 332

196 207 3348

196 212 378

196 220 626

196 226 1242

196 227 29856

196 229 923

196 234 884

196 298 332

196 420 652

196 424 3523

196 425 427

196 436 711

196 437 503

196 486 653

196 498 2374

196 534 2183

196 538 668

196 599 528

196 683 395

196 709 3426

196 716 969

196 733 954

196 857 431

196 868 1384

196 965 834

196 969 357

196 982 581

196 1090 3050

196 1151 406

196 1239 2306

196 1412 1859

196 1414 774

196 1810 2163

196 1976 633

196 1977 1416

196 2637 553

196 2821 1246

196 3071 570

196 4073 1587

196 4074 627

196 4075 561

196 4076 430

198 207 582

198 492 641

198 496 496

198 498 654

198 503 692

198 510 493

198 512 6627

198 519 503

198 614 502

198 779 683

198 804 1240

198 808 485

198 813 1033

198 816 4209

198 823 1002

198 830 743

198 840 570

198 843 1209

198 846 1215

198 1044 659

198 1118 1276

198 1186 699

198 1195 2322

198 1216 590

198 1274 1407

198 1540 535

198 1541 1142

198 1672 497

198 1853 1405

198 1858 912

198 2143 716

198 2206 823

198 2576 561

198 2829 464

198 2850 1928

198 3029 580

198 3031 549

198 3399 547

198 4096 1736

198 4097 862

198 4098 824

198 4099 748

198 4100 719

198 4101 709

198 4102 706

198 4103 658

198 4104 554

198 4105 528

198 4106 501

198 4107 478

200 63 217

200 65 132

200 88 139

200 93 342

200 99 170

200 163 286

200 176 124

200 198 154

200 227 203

200 228 336

200 389 307

200 511 135

200 533 163

200 562 150

200 607 177

200 706 182

200 709 222

200 712 136

200 717 348

200 734 210

200 857 163

200 917 499

200 945 214

200 959 387

200 985 142

200 992 140

200 998 275

200 1122 134

200 1358 210

200 1397 197

200 1412 156

200 1415 170

200 1705 244

200 1802 186

200 1810 136

200 2013 124

200 2347 232

200 2787 371

200 2853 216

200 4123 1556

200 4124 404

200 4125 310

200 4126 293

200 4127 203

200 4128 197

200 4129 184

200 4130 164

200 4131 160

200 4132 128

200 4133 125

201 71 1056

201 72 4192

201	81	144
201	89	201
201	154	137
201	180	104
201	181	154
201	186	215
201	190	232
201	298	99
201	301	136
201	333	68
201	378	169
201	506	354
201	518	130
201	641	154
201	679	84
201	905	177
201	928	78
201	962	124
201	1104	87
201	1109	703
201	1177	117
201	1263	169
201	2014	89
201	2069	68
201	3197	105
201	3206	130
201	3422	1792
201	3901	495
201	4134	1545
201	4135	355

201 4136 257
201 4137 142
201 4138 131
201 4139 131
201 4140 106
201 4141 91
201 4142 82
201 4143 82
201 4144 81
201 4145 78
201 4146 76
201 4147 75
201 4148 74
201 4149 71
201 4150 71
201 4151 68
201 4152 66
201 4153 64
202 227 159
202 348 124
202 503 89
202 504 327
202 607 71
202 706 716
202 707 1416
202 710 159
202 732 76
202 821 168
202 837 88
202 840 465

202 843 87

202 917 427

202 918 1112

202 923 248

202 928 457

202 932 277

202 942 212

202 945 163

202 947 127

202 954 189

202 1104 66

202 1214 162

202 1249 113

202 1260 149

202 1276 124

202 1714 210

202 1914 154

202 1931 258

202 1992 267

202 2341 213

202 2342 119

202 2746 137

202 2970 126

202 3244 138

202 3416 380

202 3509 106

202 4154 554

202 4155 452

202 4156 329

202 4157 225

202 4158 167

202 4159 141

202 4160 122

202 4161 120

202 4162 111

202 4163 103

202 4164 92

202 4165 68

203 297 47

203 486 733

203 488 55

203 500 78

203 503 296

203 540 46

203 544 67

203 805 38

203 810 125

203 813 120

203 814 115

203 815 88

203 816 106

203 829 142

203 832 51

203 850 76

203 964 41

203 1180 133

203 1188 77

203 1193 66

203 1541 58

203 1854 40

203 2190	65
203 2192	136
203 2203	102
203 2209	50
203 2343	77
203 2586	84
203 2595	38
203 2621	39
203 2850	50
203 2940	235
203 3927	120
203 4166	193
203 4167	122
203 4168	112
203 4169	84
203 4170	79
203 4171	79
203 4172	67
203 4173	65
203 4174	56
203 4175	52
203 4176	51
203 4177	48
203 4178	48
203 4179	46
203 4180	44
203 4181	40
203 4182	37
204 734 148	
204 840 380	

204 1201	238
204 1239	123
204 2407	147
204 3714	174
204 3737	1652
204 4118	255
204 4183	1929
204 4184	1064
204 4185	922
204 4186	856
204 4187	837
204 4188	794
204 4189	778
204 4190	646
204 4191	491
204 4192	488
204 4193	487
204 4194	452
204 4195	436
204 4196	427
204 4197	349
204 4198	310
204 4199	271
204 4200	268
204 4201	240
204 4202	233
204 4203	225
204 4204	220
204 4205	195
204 4206	186

204 4207 183

204 4208 180

204 4209 176

204 4210 167

204 4211 163

204 4212 159

204 4213 153

204 4214 152

204 4215 149

204 4216 147

204 4217 143

204 4218 138

204 4219 138

204 4220 137

204 4221 135

204 4222 127

204 4223 123

204 4224 122

205 25 649

205 173 881

205 190 1187

205 209 1161

205 380 660

205 418 625

205 460 1666

205 486 939

205 498 1439

205 500 647

205 537 1508

205 757 1212

205 758 4295

205 759 1063

205 768 5440

205 774 1360

205 779 1033

205 814 1424

205 815 862

205 816 1100

205 972 1189

205 1091 599

205 1188 923

205 1263 1892

205 2154 843

205 2383 730

205 2820 1599

205 2829 577

205 2847 764

205 3362 1238

205 3411 992

205 4108 2159

205 4173 853

205 4225 5271

205 4226 2975

205 4227 1921

205 4228 1901

205 4229 1672

205 4230 1125

205 4231 1035

205 4232 1028

205 4233 913

205 4234 904
205 4235 898
205 4236 804
205 4237 781
205 4238 708
205 4239 644
205 4240 620
205 4241 615
206 7 70
206 154 85
206 173 83
206 207 468
206 208 310
206 226 47
206 227 101
206 229 46
206 295 51
206 302 225
206 428 145
206 441 94
206 533 58
206 696 56
206 703 74
206 709 156
206 735 45
206 757 71
206 857 50
206 858 51
206 868 116
206 962 44

206 969 129

206 1052 153

206 1103 130

206 1107 55

206 1512 48

206 1609 104

206 1786 62

206 1787 59

206 1795 60

206 1986 57

206 2158 48

206 2735 132

206 2747 102

206 3046 68

206 3200 46

206 3371 53

206 4047 55

206 4129 51

206 4242 147

206 4243 103

206 4244 91

206 4245 87

206 4246 74

206 4247 56

206 4248 51

206 4249 51

206 4250 50

206 4251 44

207 89 196

207 291 291

207 294 159

207 296 238

207 302 340

207 333 245

207 353 128

207 354 494

207 359 1327

207 403 243

207 425 175

207 430 168

207 466 134

207 499 146

207 524 134

207 539 342

207 544 439

207 674 1031

207 682 163

207 687 146

207 697 309

207 901 198

207 903 252

207 972 149

207 1034 522

207 1100 155

207 1185 193

207 1201 273

207 1463 1181

207 1486 258

207 2019 230

207 2210 129

207 2435 163
207 2521 426
207 2840 2780
207 3079 213
207 3341 227
207 4252 546
207 4253 526
207 4254 386
207 4255 361
207 4256 323
207 4257 213
207 4258 189
207 4259 174
207 4260 159
207 4261 140
207 4262 135
207 4263 128
207 4264 126
208 6 46
208 12 42
208 15 114
208 28 86
208 370 33
208 707 31
208 841 133
208 843 53
208 917 103
208 953 108
208 1254 129
208 1259 36

208 1260	165
208 1274	44
208 1276	34
208 1281	85
208 1358	118
208 1360	844
208 1369	31
208 1521	328
208 1522	128
208 1533	84
208 2038	37
208 3476	121
208 4265	320
208 4266	287
208 4267	227
208 4268	225
208 4269	195
208 4270	174
208 4271	155
208 4272	150
208 4273	98
208 4274	96
208 4275	92
208 4276	92
208 4277	56
208 4278	54
208 4279	53
208 4280	50
208 4281	49
208 4282	47

208 4283 44

208 4284 43

208 4285 40

208 4286 40

208 4287 36

208 4288 33

208 4289 30

208 4290 27

209 55 28

209 67 48

209 209 28

209 257 27

209 288 306

209 289 2041

209 290 79

209 291 197

209 292 46

209 294 26

209 295 240

209 299 45

209 300 77

209 302 23

209 306 25

209 310 217

209 311 21

209 318 476

209 320 189

209 321 87

209 325 237

209 326 60

209 329 156

209 332 55

209 333 464

209 342 29

209 349 74

209 377 49

209 378 98

209 456 63

209 461 52

209 486 154

209 498 21

209 523 68

209 525 69

209 538 82

209 646 78

209 793 155

209 908 175

209 1043 55

209 1045 25

209 1243 23

209 1740 37

209 2080 27

209 2598 53

209 2801 86

209 4291 106

209 4292 85

209 4293 57

209 4294 37

210 63 1067

210 67 130

210 72 56884

210 89 114

210 157 160

210 159 154

210 165 195

210 173 16895

210 187 194

210 190 311

210 220 107

210 288 111

210 289 352

210 340 180

210 405 153

210 463 510

210 466 111

210 498 127

210 516 304

210 544 131

210 722 266

210 972 407

210 1001 1333

210 1061 894

210 1062 130

210 1072 1660

210 1075 298

210 1091 111

210 1379 387

210 1673 135

210 1713 7898

210 1786 1565

210 1791 175

210 2506 99

210 3046 1602

210 4295 895

210 4296 348

210 4297 291

210 4298 274

210 4299 233

210 4300 205

210 4301 198

210 4302 163

210 4303 148

210 4304 137

210 4305 124

210 4306 120

210 4307 101

210 4308 101

210 4309 98

211 9 102

211 30 6713

211 51 128

211 65 1195

211 72 6825

211 89 100

211 153 359

211 154 1208

211 155 224

211 173 235

211 184 110

211 190 1682

211 191 133

211 200 149

211 203 288

211 220 369

211 229 1095

211 233 704

211 238 573

211 331 343

211 486 448

211 511 166

211 529 83

211 533 1069

211 606 83

211 969 2201

211 1048 92

211 1061 102

211 1062 842

211 1072 182

211 1090 652

211 1243 110

211 1304 109

211 1375 123

211 1410 91

211 1520 196

211 1713 709

211 1988 184

211 2129 95

211 3191 1667

211 4308 757

211 4310 554

211 4311 529

211 4312 402

211 4313 306

211 4314 186

211 4315 162

211 4316 142

211 4317 97

211 4318 89

212 917 31

212 920 25

212 923 25

212 924 87

212 928 103

212 930 47

212 936 112

212 937 101

212 942 58

212 944 38

212 946 77

212 957 104

212 1338 52

212 1339 106

212 1342 117

212 1349 239

212 1350 82

212 1363 55

212 1374 57

212 1993 51

212 1997 102

212 2009 61

212	2015	41
212	2838	46
212	2975	46
212	4319	160
212	4320	152
212	4321	89
212	4322	78
212	4323	64
212	4324	61
212	4325	49
212	4326	48
212	4327	45
212	4328	45
212	4329	43
212	4330	41
212	4331	40
212	4332	40
212	4333	40
212	4334	39
212	4335	37
212	4336	36
212	4337	36
212	4338	35
212	4339	34
212	4340	32
212	4341	27
212	4342	27
212	4343	23
213	7	429
213	163	390

213 190 1376

213 198 949

213 207 722

213 220 603

213 228 1523

213 229 838

213 234 1029

213 245 323

213 250 324

213 278 502

213 377 444

213 412 1447

213 475 484

213 489 822

213 512 341

213 517 997

213 707 1548

213 779 630

213 785 304

213 813 493

213 823 588

213 843 348

213 846 3401

213 850 689

213 868 417

213 978 327

213 1044 559

213 1099 382

213 1116 984

213 1216 337

213 1249 345

213 1351 407

213 1672 364

213 1974 551

213 1979 1564

213 2169 630

213 2179 318

213 2244 1034

213 2247 328

213 2384 292

213 2744 1387

213 3032 433

213 3637 434

213 3717 405

213 4344 634

213 4345 591

213 4346 465

213 4347 296

214 55 329

214 67 5755

214 89 189

214 157 7462

214 227 236

214 285 566

214 288 280

214 289 900

214 292 373

214 295 316

214 298 179

214 300 125

214 302 98

214 306 92

214 329 262

214 331 146

214 340 76

214 349 138

214 461 111

214 466 135

214 484 69

214 498 103

214 544 282

214 578 80

214 601 630

214 646 156

214 701 131

214 704 750

214 706 628

214 889 255

214 903 100

214 951 72

214 959 86

214 966 178

214 1244 125

214 1369 61

214 1412 90

214 1467 356

214 1807 469

214 1825 269

214 2020 75

214 2179 134

214 2788 256
214 3313 296
214 3743 71
214 4348 101
214 4349 71
214 4350 69
214 4351 67
214 4352 57
215 69 54
215 88 32
215 215 14
215 230 15
215 250 9
215 429 9
215 499 11
215 533 35
215 614 17
215 707 9
215 732 28
215 854 9
215 868 25
215 1426 9
215 1974 11
215 2531 38
215 2554 19
215 2637 15
215 3427 15
215 3475 13
215 3791 10
215 3992 11

215 4353 55

215 4354 39

215 4355 37

215 4356 32

215 4357 24

215 4358 24

215 4359 22

215 4360 22

215 4361 21

215 4362 21

215 4363 18

215 4364 17

215 4365 15

215 4366 14

215 4367 13

215 4368 12

215 4369 12

215 4370 12

215 4371 11

215 4372 11

215 4373 11

215 4374 11

215 4375 10

215 4376 10

215 4377 10

215 4378 9

215 4379 8

215 4380 8

216 56 265

216 72 2182

216 89 153

216 157 39

216 170 38

216 230 239

216 328 51

216 331 40

216 498 44

216 709 90

216 887 60

216 980 65

216 1014 42

216 1061 110

216 1062 73

216 1073 303

216 1075 995

216 1155 141

216 1243 63

216 1379 50

216 1398 103

216 1399 60

216 1400 63

216 1403 46

216 1449 43

216 1713 174

216 1886 52

216 1983 206

216 2824 43

216 3109 180

216 3266 166

216 3300 382

216 3502 43
216 3767 49
216 3781 87
216 4025 48
216 4381 198
216 4382 131
216 4383 128
216 4384 123
216 4385 110
216 4386 95
216 4387 81
216 4388 76
216 4389 55
216 4390 51
216 4391 44
216 4392 42
216 4393 41
216 4394 37
217 218 154
217 222 129
217 559 116
217 706 219
217 917 148
217 989 151
217 1132 112
217 1206 106
217 1360 100
217 1369 110
217 1504 100
217 1513 202

217 1901	230
217 2026	1805
217 2343	205
217 2375	123
217 2475	231
217 2493	127
217 2624	5563
217 3281	118
217 3430	148
217 4081	108
217 4395	796
217 4396	759
217 4397	450
217 4398	388
217 4399	294
217 4400	252
217 4401	234
217 4402	217
217 4403	163
217 4404	161
217 4405	158
217 4406	146
217 4407	142
217 4408	139
217 4409	129
217 4410	125
217 4411	123
217 4412	122
217 4413	120
217 4414	119

217 4415 119

217 4416 115

217 4417 112

217 4418 110

217 4419 108

217 4420 107

217 4421 101

217 4422 100

218 65 384

218 70 119

218 72 2806

218 88 192

218 163 183

218 197 85

218 198 119

218 221 75

218 227 856

218 250 94

218 441 91

218 488 66

218 706 207

218 707 192

218 814 58

218 867 309

218 868 1952

218 1044 109

218 1061 109

218 1072 661

218 1090 91

218 1104 61

218 1106	64
218 1112	83
218 1188	153
218 1410	75
218 1412	88
218 1414	89
218 1415	83
218 1416	63
218 1541	76
218 1639	314
218 1650	64
218 1713	193
218 2130	61
218 2186	93
218 2192	160
218 2200	173
218 2835	84
218 2902	193
218 3065	69
218 3934	63
218 4155	106
218 4166	179
218 4295	62
218 4423	127
218 4424	98
218 4425	96
218 4426	65
218 4427	55
219 184 1985	
219 190 1040	

219 300 1972

219 324 1438

219 325 915

219 333 803

219 357 972

219 376 1329

219 377 3486

219 408 883

219 453 2349

219 461 1508

219 486 11844

219 498 9499

219 499 2321

219 507 1050

219 674 1567

219 680 1517

219 966 1333

219 1043 1610

219 1116 2163

219 1118 1407

219 1182 1828

219 1246 2124

219 1378 963

219 1854 1994

219 1857 3507

219 1864 931

219 2150 5723

219 2206 2532

219 2619 3887

219 2785 1005

219 2839 2311

219 3084 1528

219 3094 1443

219 3753 1892

219 3859 1128

219 3945 4165

219 4428 3584

219 4429 1385

219 4430 1338

219 4431 1201

219 4432 1125

219 4433 1073

219 4434 998

219 4435 996

219 4436 977

219 4437 976

219 4438 910

219 4439 857

220 154 131

220 170 48

220 203 110

220 207 67

220 210 54

220 217 146

220 225 66

220 226 61

220 227 135

220 228 65

220 234 201

220 418 72

220 419 609

220 424 57

220 429 59

220 433 135

220 436 881

220 451 140

220 498 526

220 602 64

220 609 56

220 614 492

220 615 148

220 856 81

220 982 89

220 1116 437

220 1519 525

220 1721 46

220 2264 531

220 2277 66

220 2284 68

220 2425 56

220 3030 149

220 3252 58

220 4074 402

220 4440 4492

220 4441 154

220 4442 152

220 4443 149

220 4444 142

220 4445 116

220 4446 112

220 4447 101

220 4448 61

220 4449 58

220 4450 54

220 4451 53

220 4452 52

220 4453 46

220 4454 45

223 191 1394

223 288 2517

223 289 874

223 290 1258

223 291 10946

223 292 908

223 294 1193

223 295 1183

223 297 1411

223 300 1141

223 302 2238

223 304 2972

223 305 10908

223 311 1977

223 323 850

223 325 2720

223 326 1532

223 333 1988

223 342 2326

223 344 2882

223 349 1983

223 362 1476

223 461 1135

223 523 1582

223 528 3173

223 674 935

223 679 910

223 681 1676

223 906 1012

223 908 1563

223 966 1382

223 1375 1086

223 1377 927

223 1432 1173

223 1447 779

223 1470 1980

223 2019 781

223 2840 1061

223 3075 1714

223 3081 815

223 3489 877

223 3575 1145

223 3859 2063

223 3871 818

223 4487 3239

223 4488 2258

223 4489 1534

223 4490 1070

223 4491 913

223 4492 785

224 51 303

224 59 203

224 61 183

224 67 243

224 72 406

224 96 228

224 99 157

224 157 147

224 159 214

224 172 295

224 187 273

224 193 337

224 227 195

224 251 146

224 343 192

224 429 154

224 441 181

224 599 321

224 612 178

224 715 168

224 735 148

224 852 153

224 857 171

224 878 251

224 985 210

224 986 160

224 993 311

224 997 280

224 998 239

224 1004 443

224 1013 142

224 1017 174

224 1019 145

224 1027 193

224 1083 199

224 1122 167

224 1242 198

224 1413 178

224 1881 145

224 1886 150

224 1891 142

224 2265 258

224 2633 158

224 3119 189

224 3201 158

224 3280 195

224 3302 166

224 4493 197

224 4494 168

224 4495 159

225 300 833

225 503 2178

225 779 2728

225 785 1143

225 813 2545

225 814 1226

225 831 829

225 839 706

225 841 8065

225 842 2809

225 845 1292

225 949 1141

225 954 839	
225 1099	583
225 1196	1605
225 1260	491
225 1261	1800
225 1364	465
225 1522	491
225 1533	546
225 1541	2300
225 1547	5522
225 1557	825
225 2028	695
225 2062	823
225 2169	527
225 2744	501
225 2944	519
225 2961	1321
225 2970	1201
225 3028	1730
225 3549	997
225 4021	1487
225 4346	1156
225 4496	6193
225 4497	3947
225 4498	3835
225 4499	3506
225 4500	2332
225 4501	1320
225 4502	1319
225 4503	1272

225 4504 1124

225 4505 1085

225 4506 791

225 4507 589

225 4508 586

225 4509 573

225 4510 451

225 4511 451

226 67 200

226 89 3232

226 230 199

226 257 247

226 288 18539

226 289 117

226 291 190

226 292 673

226 295 849

226 300 1466

226 302 1361

226 306 176

226 310 545

226 318 133

226 321 125

226 325 1108

226 333 501

226 344 212

226 349 1163

226 378 131

226 461 550

226 466 894

226 475 260

226 498 165

226 523 695

226 534 135

226 542 1059

226 543 245

226 544 248

226 546 115

226 679 1176

226 681 187

226 686 1262

226 692 168

226 701 1736

226 790 106

226 802 194

226 907 174

226 972 154

226 1037 258

226 1453 1318

226 1455 108

226 1613 185

226 1673 922

226 2990 1075

226 3332 222

226 3571 504

226 4512 126

226 4513 125

226 4514 105

227 55 8119

227 67 1690

227 89 10178

227 257 26064

227 288 1575

227 289 8669

227 291 1118

227 292 55045

227 293 292

227 294 319

227 295 5110

227 299 119

227 300 1163

227 301 177

227 302 176

227 305 1412

227 326 110

227 333 243

227 344 128

227 349 427

227 352 295

227 386 79

227 393 127

227 400 1802

227 409 345

227 461 322

227 466 109

227 525 321

227 543 84

227 544 93

227 679 565

227 681 86

227 701 2459

227 1451 247

227 1459 666

227 1904 9945

227 1918 504

227 2089 451

227 2091 545

227 2092 1935

227 2094 1157

227 2097 371

227 2106 201

227 2392 349

227 2799 426

227 2803 2832

227 2806 141

227 4515 328

227 4516 178

227 4517 151

228 197 13

228 212 17

228 219 8

228 222 8

228 609 18

228 615 15

228 630 14

228 718 11

228 748 8

228 859 9

228 863 10

228 1150 13

228 1164	9
228 2226	10
228 2640	11
228 2641	7
228 3001	8
228 3145	8
228 3458	8
228 4079	10
228 4518	30
228 4519	26
228 4520	25
228 4521	18
228 4522	17
228 4523	15
228 4524	14
228 4525	14
228 4526	13
228 4527	13
228 4528	13
228 4529	12
228 4530	11
228 4531	11
228 4532	11
228 4533	11
228 4534	11
228 4535	10
228 4536	9
228 4537	9
228 4538	9
228 4539	9

228 4540 9

228 4541 8

228 4542 8

228 4543 8

228 4544 8

228 4545 8

228 4546 8

228 4547 8

229 7 1

229 38 2

229 89 5

229 161 2

229 209 3

229 234 2

229 241 1

229 288 9

229 289 1

229 291 1

229 294 2

229 299 1

229 301 1

229 306 2

229 317 3

229 318 6

229 329 1

229 330 3

229 344 65

229 349 29

229 465 2

229 499 58

229	518	2
229	527	3
229	830	1
229	858	1
229	913	1
229	930	6
229	964	1
229	1274	1
229	1595	3
229	1596	1
229	1601	1
229	1604	1
229	1641	2
229	1673	2
229	2024	1
229	2226	1
229	2840	1
229	2905	1
229	2959	18
229	2990	4
229	3542	3
229	3719	2
229	4548	21
229	4549	3
229	4550	2
229	4551	1
229	4552	1
229	4553	1
230	55	875
230	67	834

230 89 2596

230 257 5434

230 288 609

230 289 1667

230 291 565

230 292 1581

230 294 2801

230 299 2302

230 300 530

230 301 310

230 302 335

230 304 5248

230 306 316

230 318 650

230 321 466

230 324 443

230 333 405

230 344 832

230 349 328

230 355 488

230 374 1130

230 375 14993

230 378 834

230 394 443

230 456 16105

230 461 1676

230 465 373

230 466 878

230 518 335

230 544 570

230 701 539

230 793 336

230 908 330

230 1047 345

230 1602 1281

230 1934 417

230 2086 673

230 2097 297

230 2102 8034

230 3320 821

230 3324 1548

230 3330 326

230 4554 1311

230 4555 808

230 4556 433

230 4557 427

230 4558 353

230 4559 326

231 67 176

231 89 2833

231 161 119

231 257 189

231 288 1706

231 289 2873

231 292 181

231 295 248

231 296 104

231 298 111

231 299 79

231 300 465

231 306 154

231 317 196

231 318 130

231 323 522

231 327 279

231 328 126

231 331 155

231 332 85

231 349 87

231 352 423

231 386 77

231 458 116

231 461 162

231 466 454

231 468 87

231 484 79

231 528 92

231 535 242

231 543 317

231 545 81

231 644 1779

231 679 736

231 699 95

231 701 129

231 907 80

231 914 400

231 915 84

231 1037 353

231 1449 98

231 2025 271

231 2939 161

231 2990 131

231 3713 99

231 4560 421

231 4561 161

231 4562 142

231 4563 89

231 4564 82

232 7 1491

232 55 332

232 67 647

232 89 3984

232 241 422

232 251 1192

232 257 7046

232 266 238

232 285 26059

232 288 5062

232 289 11950

232 290 355

232 292 4432

232 293 325

232 294 10592

232 295 1967

232 299 3745

232 301 1022

232 306 833

232 308 778

232 309 3911

232 310 257

232 314 580

232 317 2229

232 318 467

232 320 345

232 332 432

232 349 341

232 352 598

232 465 1778

232 484 385

232 540 254

232 544 247

232 907 776

232 1104 462

232 1180 505

232 1513 264

232 1981 660

232 2515 1089

232 3237 2275

232 4565 4447

232 4566 2713

232 4567 2641

232 4568 1091

232 4569 1012

232 4570 723

232 4571 711

232 4572 502

232 4573 497

232 4574 306

233 55 65

233 67 74

233 89 5783

233 228 87

233 288 563

233 289 914

233 292 5531

233 298 27

233 300 1015

233 302 67

233 306 72

233 329 10

233 330 7

233 340 43

233 377 63

233 461 835

233 466 2038

233 475 40

233 498 379

233 525 59

233 526 267

233 534 10

233 679 49

233 680 18

233 687 34

233 706 185

233 714 16

233 903 13

233 972 143

233 1043 21

233 1047 73

233 1249 162

233 1453 26
233 1455 507
233 1456 11
233 1934 181
233 2085 210
233 2094 143
233 2543 22
233 3198 117
233 3662 6
233 3739 307
233 4575 56
233 4576 26
233 4577 24
233 4578 18
233 4579 14
233 4580 9
233 4581 8
233 4582 7
234 56 686
234 88 415
234 267 1449
234 271 832
234 276 1586
234 310 411
234 330 255
234 331 1284
234 377 517
234 432 362
234 517 392
234 605 1952

234 617 4734

234 731 256

234 1285	1599
234 1396	1028
234 1610	387
234 1636	660
234 1640	595
234 1952	1445
234 1974	379
234 1979	1739
234 2247	344
234 2252	1112
234 2698	727
234 2716	2240
234 2919	265
234 2931	300
234 3227	4218
234 4583	2323
234 4584	1230
234 4585	1142
234 4586	748
234 4587	560
234 4588	472
234 4589	462
234 4590	458
234 4591	426
234 4592	382
234 4593	364
234 4594	362
234 4595	347

234 4596 345

234 4597 328

234 4598 284

234 4599 283

234 4600 256

234 4601 247

234 4602 245

234 4603 232

235 15 601

235 152 163

235 163 194

235 188 2094

235 441 130

235 503 1019

235 707 347

235 769 547

235 812 1374

235 821 564

235 824 810

235 917 2778

235 918 373

235 920 525

235 930 335

235 942 1393

235 943 129

235 944 149

235 953 195

235 954 499

235 955 565

235 957 172

235	1131	259
235	1134	391
235	1216	139
235	1342	220
235	1358	1990
235	1368	200
235	1369	153
235	1992	130
235	2172	218
235	2370	369
235	2969	219
235	2975	175
235	3240	131
235	3415	143
235	3810	356
235	3940	206
235	4604	644
235	4605	491
235	4606	401
235	4607	303
235	4608	278
235	4609	174
235	4610	159
235	4611	159
235	4612	155
235	4613	140
235	4614	140
235	4615	129
236	65	1679
236	89	1435

236 154 812

236 190 7774

236 191 1721

236 204 406

236 220 623

236 228 618

236 229 390

236 230 503

236 234 927

236 257 1171

236 288 4769

236 289 698

236 292 3477

236 294 2496

236 295 9726

236 300 1134

236 301 2309

236 314 832

236 327 1764

236 329 1201

236 331 373

236 333 487

236 344 532

236 468 1102

236 475 618

236 518 414

236 547 1285

236 679 1115

236 691 606

236 701 472

236 1055 652
236 1243 463
236 1376 1217
236 1507 488
236 1511 1099
236 1613 571
236 1934 467
236 1980 524
236 2051 393
236 2176 644
236 2526 1575
236 3171 729
236 3317 387
236 3371 561
236 4616 3542
236 4617 2444
236 4618 772
236 4619 413
237 154 2605
237 163 350
237 190 99
237 195 70
237 196 101
237 203 1455
237 207 1135
237 208 543
237 221 547
237 222 70
237 225 72
237 227 100

237 234 165

237 237 162

237 416 187

237 424 4168

237 428 499

237 429 1781

237 436 342

237 440 114

237 441 227

237 442 122

237 511 101

237 533 1353

237 599 121

237 609 447

237 614 165

237 615 251

237 622 342

237 851 766

237 854 127

237 859 239

237 1089 198

237 1106 202

237 1122 109

237 1179 98

237 1384 204

237 1504 73

237 1512 84

237 2134 1066

237 2279 107

237 2531 125

237 2606 664

237 3481 91

237 3693 301

237 4620 227

237 4621 150

237 4622 82

237 4623 79

237 4624 72

238 7 1234

238 198 360

238 436 337

238 1411 1561

238 1726 412

238 1974 357

238 2375 618

238 3472 783

238 4625 10776

238 4626 8833

238 4627 8636

238 4628 5227

238 4629 5208

238 4630 4431

238 4631 3269

238 4632 3024

238 4633 3005

238 4634 2827

238 4635 2717

238 4636 2520

238 4637 2152

238 4638 1779

238	4639	1467
238	4640	996
238	4641	810
238	4642	683
238	4643	612
238	4644	590
238	4645	576
238	4646	541
238	4647	517
238	4648	507
238	4649	504
238	4650	478
238	4651	458
238	4652	454
238	4653	447
238	4654	425
238	4655	414
238	4656	410
238	4657	401
238	4658	390
238	4659	382
238	4660	379
238	4661	374
238	4662	355
238	4663	354
238	4664	345
238	4665	342
238	4666	336
240	55	97
240	67	94

240 89 1317

240 190 471

240 227 116

240 288 318

240 289 420

240 292 145

240 295 108

240 300 248

240 302 105

240 306 137

240 328 136

240 333 800

240 344 148

240 346 111

240 378 129

240 386 102

240 455 137

240 458 137

240 464 146

240 466 486

240 475 146

240 481 195

240 486 156

240 498 184

240 502 107

240 540 82

240 544 98

240 701 213

240 726 77

240 1042 104

240 1044 113

240 1104 184

240 1186 192

240 1449 90

240 1455 75

240 1464 76

240 1571 289

240 1576 176

240 1786 148

240 2498 202

240 3859 198

240 4242 127

240 4502 396

240 4685 503

240 4686 92

240 4687 89

240 4688 86

240 4689 80

241 6 1295

241 152 1301

241 181 1705

241 188 3787

241 198 870

241 227 2474

241 370 3435

241 436 780

241 439 1590

241 503 2466

241 504 1829

241 506 3220

241 533 822

241 706 673

241 707 688

241 710 1062

241 860 1831

241 920 1021

241 932 1778

241 943 612

241 982 1606

241 1119 576

241 1239 659

241 1244 664

241 1276 676

241 1412 589

241 1803 1356

241 2006 1749

241 2346 564

241 2350 1121

241 2369 548

241 2370 1705

241 2372 672

241 2654 1227

241 2789 1005

241 2961 1378

241 2970 1393

241 3405 1449

241 3416 1936

241 3800 651

241 3901 1169

241 4162 545

241 4270 752

241 4675 1112

241 4690 1751

241 4691 1400

241 4692 705

241 4693 600

241 4694 596

241 4695 568

242 67 81

242 89 761

242 257 17328

242 285 328

242 288 607

242 289 827

242 290 85

242 291 139

242 292 1044

242 294 717

242 295 1022

242 299 115

242 300 346

242 301 170

242 302 119

242 306 255

242 308 169

242 309 159

242 310 83

242 311 93

242 317 80

242 318 83

242 320 86

242 327 94

242 344 109

242 349 158

242 456 75

242 464 103

242 465 255

242 466 647

242 546 107

242 701 948

242 786 166

242 787 127

242 915 73

242 1037 130

242 1441 162

242 1446 92

242 2083 84

242 2176 116

242 2990 177

242 3498 78

242 4696 297

242 4697 179

242 4698 103

242 4699 92

242 4700 92

242 4701 77

242 4702 75

242 4703 75

243 53 957

243 55 2783

243 58 3234
243 64 1369
243 65 299
243 67 5180
243 70 422
243 71 498
243 72 1059
243 81 493
243 89 1133
243 97 913
243 155 350
243 167 672
243 172 2282
243 238 669
243 288 318
243 292 1052
243 295 463
243 296 2414
243 298 344
243 301 270
243 316 302
243 423 800
243 425 614
243 485 418
243 532 1323
243 534 626
243 546 982
243 548 591
243 689 1294
243 697 1238

243 906 2130

243 962 772

243 1001 801

243 1098 417

243 1379 485

243 1609 519

243 1824 781

243 1983 294

243 2083 461

243 2521 521

243 2544 326

243 3288 904

243 3460 984

243 3502 576

243 4704 677

243 4705 467

243 4706 343

243 4707 272

244 65 204

244 69 413

244 89 141

244 157 106

244 190 594

244 207 458

244 209 124

244 220 95

244 228 269

244 246 137

244 283 105

244 288 139

244 294 134

244 295 133

244 298 201

244 300 212

244 301 3654

244 304 365

244 306 100

244 378 347

244 424 177

244 430 210

244 481 146

244 486 240

244 498 263

244 514 141

244 533 112

244 538 117

244 540 177

244 683 246

244 693 111

244 715 95

244 716 246

244 903 177

244 1098 193

244 1180 155

244 1833 251

244 1934 228

244 2419 107

244 2544 546

244 3332 201

244 3398 208

244 3488 313

244 4291 141

244 4616 159

244 4708 385

244 4709 181

244 4710 145

244 4711 107

244 4712 102

247 179 104

247 403 88

247 488 320

247 700 128

247 966 152

247 982 101

247 1182 97

247 1377 84

247 1470 93

247 1852 223

247 1854 388

247 1864 136

247 1874 149

247 2150 105

247 2352 111

247 2843 102

247 2960 118

247 2978 130

247 3749 111

247 3873 120

247 4179 206

247 4439 237

247 4737 644
247 4738 452
247 4739 406
247 4740 352
247 4741 346
247 4742 317
247 4743 291
247 4744 285
247 4745 282
247 4746 247
247 4747 242
247 4748 231
247 4749 205
247 4750 204
247 4751 176
247 4752 163
247 4753 159
247 4754 148
247 4755 141
247 4756 139
247 4757 129
247 4758 124
247 4759 123
247 4760 114
247 4761 109
247 4762 104
247 4763 104
247 4764 88
248 89 511
248 161 129

248 288 1315

248 289 525

248 292 1141

248 295 181

248 296 91

248 300 122

248 306 1173

248 308 152

248 320 78

248 324 100

248 325 44

248 332 158

248 333 116

248 338 194

248 349 45

248 386 1143

248 455 370

248 458 92

248 461 269

248 464 2417

248 466 1116

248 498 94

248 532 87

248 541 34

248 543 382

248 544 114

248 644 165

248 686 120

248 701 149

248 907 38

248 908 40

248 915 143

248 1021 133

248 1071 53

248 1513 56

248 2020 167

248 2498 91

248 2543 124

248 3209 43

248 3397 47

248 4565 40

248 4765 334

248 4766 193

248 4767 140

248 4768 93

248 4769 53

248 4770 37

248 4771 34

249 7 230

249 209 287

249 291 198

249 293 214

249 325 325

249 333 424

249 371 197

249 377 246

249 403 521

249 464 522

249 486 1310

249 493 613

249 497 380

249 498 1372

249 500 990

249 503 271

249 536 208

249 537 2200

249 680 563

249 682 701

249 704 653

249 779 1084

249 808 630

249 811 223

249 816 453

249 968 462

249 1020 253

249 1029 206

249 1180 437

249 1184 312

249 1188 1151

249 1410 198

249 1853 463

249 1868 338

249 2619 313

249 2839 274

249 2850 291

249 2959 316

249 2978 241

249 3084 682

249 3213 200

249 3328 226

249 4036 253
249 4262 676
249 4772 932
249 4773 648
249 4774 634
249 4775 502
249 4776 315
249 4777 222
250 56 483
250 88 881
250 96 270
250 163 1132
250 188 334
250 212 338
250 222 2699
250 607 857
250 717 258
250 785 458
250 920 263
250 945 310
250 949 252
250 957 357
250 959 350
250 977 292
250 1154 397
250 1411 789
250 1412 326
250 1415 785
250 1631 278
250 1636 273

250 1901	257
250 1992	603
250 2151	307
250 2375	910
250 2624	2285
250 2642	1119
250 3057	345
250 3434	495
250 3444	988
250 3481	651
250 3711	369
250 3788	495
250 4214	260
250 4675	285
250 4678	294
250 4778	2336
250 4779	1633
250 4780	819
250 4781	635
250 4782	436
250 4783	399
250 4784	377
250 4785	353
250 4786	320
250 4787	279
250 4788	260
250 4789	257
250 4790	254
251 207 87	
251 226 76	

251	424	480
251	439	549
251	706	88
251	718	258
251	863	209
251	932	309
251	964	60
251	1090	120
251	1160	59
251	1249	96
251	1274	312
251	1360	230
251	1406	53
251	1512	233
251	1513	80
251	1528	165
251	1545	77
251	1814	83
251	2629	55
251	2899	102
251	3011	321
251	3058	56
251	3354	62
251	3745	622
251	4164	173
251	4209	151
251	4452	94
251	4459	159
251	4541	346
251	4791	435

251 4792 242

251 4793 223

251 4794 180

251 4795 170

251 4796 151

251 4797 151

251 4798 143

251 4799 135

251 4800 112

251 4801 88

251 4802 84

251 4803 73

251 4804 66

251 4805 61

251 4806 60

251 4807 58

251 4808 55

251 4809 54

252 55 1701

252 89 2078

252 161 203

252 288 1294

252 289 733

252 292 492

252 295 271

252 298 1375

252 300 6819

252 302 181

252 323 2972

252 325 481

252 333 444

252 344 2538

252 346 634

252 386 1152

252 430 254

252 458 438

252 461 2449

252 466 1043

252 498 217

252 528 685

252 530 274

252 532 177

252 535 465

252 548 595

252 679 2438

252 681 425

252 686 947

252 687 264

252 689 1096

252 690 684

252 915 282

252 1032 175

252 1409 503

252 1447 949

252 1505 1211

252 2076 412

252 2546 3787

252 2981 472

252 3057 180

252 3081 1933

252 3489 236
252 4618 199
252 4810 807
252 4811 582
252 4812 524
252 4813 312
252 4814 248
252 4815 194
253 55 1363
253 65 891
253 67 2111
253 89 2834
253 97 774
253 166 418
253 220 499
253 246 460
253 256 605
253 288 1411
253 289 5387
253 290 893
253 291 617
253 292 701
253 295 1913
253 298 509
253 302 1001
253 306 1027
253 310 930
253 333 531
253 340 400
253 349 788

253 350 347

253 362 510

253 423 1337

253 439 659

253 498 750

253 542 925

253 646 1053

253 689 348

253 701 3804

253 789 368

253 790 661

253 792 2031

253 802 15049

253 889 1592

253 903 857

253 906 509

253 1429 383

253 1771 334

253 2022 510

253 3187 392

253 3391 452

253 3466 704

253 3557 368

253 4716 1699

253 4816 472

253 4817 461

253 4818 352

253 4819 335

254 227 453

254 412 996

254 808 474

254 840 993

254 857 1112

254 1045 1490

254 1046 465

254 1186 408

254 1406 758

254 1513 1724

254 1645 706

254 1866 617

254 3398 436

254 4087 489

254 4157 769

254 4218 458

254 4375 2108

254 4434 552

254 4820 3817

254 4821 3744

254 4822 2743

254 4823 1742

254 4824 1594

254 4825 1508

254 4826 1414

254 4827 1240

254 4828 1231

254 4829 1021

254 4830 965

254 4831 943

254 4832 736

254 4833 690

254	4834	668
254	4835	660
254	4836	609
254	4837	555
254	4838	520
254	4839	517
254	4840	506
254	4841	504
254	4842	502
254	4843	479
254	4844	478
254	4845	471
254	4846	470
254	4847	429
254	4848	381
254	4849	369
254	4850	367
254	4851	359
255	81	294
255	101	211
255	120	130
255	198	188
255	220	498
255	238	967
255	512	594
255	603	288
255	757	156
255	772	833
255	778	785
255	831	185

255	845	431
255	1098	510
255	1156	617
255	1222	217
255	1745	891
255	1752	415
255	1789	128
255	1797	163
255	1818	245
255	1835	998
255	2145	303
255	2148	365
255	2156	484
255	2158	231
255	2164	147
255	2167	407
255	2173	194
255	2174	242
255	2576	167
255	2578	175
255	2668	648
255	2675	455
255	2775	155
255	4616	167
255	4852	1050
255	4853	750
255	4854	525
255	4855	348
255	4856	330
255	4857	313

255 4858 292

255 4859 230

255 4860 229

255 4861 147

255 4862 140

255 4863 137

255 4864 134

255 4865 131

256 55 1941

256 67 6368

256 89 7569

256 184 554

256 204 3490

256 246 1131

256 256 1175

256 257 1278

256 261 363

256 270 1223

256 283 8991

256 288 4882

256 289 38654

256 291 1132

256 292 7665

256 295 3735

256 300 1574

256 301 561

256 302 1187

256 306 1229

256 311 539

256 320 447

256 328 429

256 329 805

256 333 570

256 349 3130

256 352 390

256 439 1328

256 465 524

256 466 2431

256 476 503

256 701 766

256 708 726

256 1241 620

256 1771 1064

256 2082 411

256 2548 56650

256 2984 701

256 2988 499

256 3173 379

256 3376 468

256 3396 2396

256 4561 355

256 4818 503

256 4819 581

256 4866 1852

256 4867 1450

256 4868 1320

256 4869 396

256 4870 369

257 2 152

257 9 261

257 10 101

257 18 137

257 19 265

257 24 114

257 30 985

257 32 305

257 34 176

257 37 220

257 41 150

257 44 155

257 49 148

257 72 78

257 153 173

257 1066 84

257 1203 108

257 1270 101

257 1292 114

257 1293 192

257 1294 265

257 1295 245

257 1296 318

257 1297 231

257 1298 286

257 1299 136

257 1302 95

257 1303 326

257 1304 96

257 1312 177

257 1320 136

257 1321 123

257 1323 97

257 1328 172

257 1332 172

257 1334 101

257 1335 122

257 1336 92

257 3551 91

257 3988 101

257 4312 159

257 4317 121

257 4318 131

257 4871 231

257 4872 170

257 4873 105

257 4874 99

257 4875 87

257 4876 79

257 4877 77

258 89 16

258 288 26

258 289 333

258 292 18

258 293 2

258 295 2

258 299 9

258 300 21

258 302 16

258 306 3

258 308 9

258 310 2

258 314 76

258 320 2

258 325 13

258 329 2

258 333 16

258 349 10

258 352 3

258 455 35

258 461 179

258 466 16

258 523 3

258 528 4

258 538 3

258 680 16

258 681 13

258 686 7

258 701 11

258 793 7

258 797 2

258 907 2

258 913 2

258 972 2

258 1453 6

258 1698 5

258 2019 6

258 2025 2

258 2094 16

258 3367 3

258 3397 7

258 4878 14

258 4879 8

258 4880 3

258 4881 3

258 4882 3

258 4883 2

258 4884 2

258 4885 2

258 4886 2

259 154 260

259 173 2813

259 190 192

259 210 215

259 234 111

259 460 597

259 533 332

259 615 868

259 758 324

259 779 134

259 964 343

259 1090 201

259 1179 445

259 1784 354

259 1788 279

259 1790 505

259 1913 121

259 2735 575

259 2747 552

259 3046 292

259 3056 685

259 3828 313

259	4226	2392
259	4229	195
259	4244	273
259	4887	1686
259	4888	691
259	4889	658
259	4890	449
259	4891	416
259	4892	226
259	4893	200
259	4894	199
259	4895	192
259	4896	187
259	4897	147
259	4898	144
259	4899	142
259	4900	140
259	4901	137
259	4902	137
259	4903	137
259	4904	125
259	4905	124
259	4906	119
259	4907	118
259	4908	114
259	4909	111
259	4910	110
259	4911	109
260	67	278
260	89	707

260 157 5849

260 285 576

260 292 584

260 340 178

260 389 128

260 502 122

260 518 362

260 526 255

260 531 134

260 601 71

260 659 91

260 1042 381

260 1091 73

260 1379 82

260 1427 74

260 1601 125

260 1700 151

260 1705 474

260 1825 440

260 1834 359

260 1934 87

260 2075 59

260 2182 170

260 2446 296

260 3313 804

260 4348 287

260 4723 100

260 4912 366

260 4913 239

260 4914 207

260 4915 175

260 4916 168

260 4917 148

260 4918 116

260 4919 114

260 4920 111

260 4921 108

260 4922 101

260 4923 96

260 4924 85

260 4925 66

260 4926 65

260 4927 63

260 4928 62

260 4929 60

260 4930 60

260 4931 58

260 4932 57

261 230 553

261 377 330

261 403 379

261 412 847

261 475 854

261 547 1190

261 682 952

261 691 856

261 805 559

261 809 305

261 816 501

261 825 400

261 1188	375
261 1195	394
261 1196	332
261 1455	772
261 1541	416
261 1542	453
261 1552	389
261 1557	463
261 1568	299
261 1853	303
261 1863	761
261 2187	338
261 2203	558
261 2618	345
261 2923	571
261 3317	298
261 3341	319
261 4002	325
261 4103	307
261 4104	328
261 4497	463
261 4503	536
261 4933	995
261 4934	662
261 4935	640
261 4936	611
261 4937	538
261 4938	432
261 4939	419
261 4940	414

261 4941 405

261 4942 395

261 4943 385

261 4944 347

261 4945 335

261 4946 328

261 4947 321

261 4948 303

262 21 110

262 72 1308

262 81 131

262 186 156

262 190 110

262 370 505

262 429 119

262 506 950

262 591 148

262 718 122

262 953 166

262 1118 665

262 1726 779

262 2030 150

262 3267 1396

262 3405 202

262 3407 272

262 3472 111

262 3768 155

262 3806 112

262 3901 111

262 4269 135

262 4271 170

262 4649 220

262 4691 291

262 4949 427

262 4950 372

262 4951 350

262 4952 326

262 4953 321

262 4954 316

262 4955 315

262 4956 240

262 4957 238

262 4958 228

262 4959 198

262 4960 196

262 4961 186

262 4962 164

262 4963 163

262 4964 154

262 4965 152

262 4966 152

262 4967 149

262 4968 146

262 4969 127

262 4970 123

262 4971 118

262 4972 109

262 4973 108

263 51 386

263 61 159

263 67 474

263 78 162

263 85 585

263 91 446

263 157 930

263 161 177

263 176 205

263 187 6938

263 190 477

263 192 353

263 193 274

263 229 1783

263 253 180

263 316 223

263 377 262

263 679 190

263 877 2413

263 889 807

263 985 201

263 997 316

263 998 263

263 1001 510

263 1014 860

263 1081 250

263 1479 310

263 1705 371

263 1892 313

263 2031 160

263 2035 451

263 2130 1499

263 2183 273

263 2469 266

263 2558 432

263 3264 281

263 3302 335

263 3920 218

263 4130 394

263 4180 177

263 4923 168

263 4974 736

263 4975 493

263 4976 422

263 4977 380

263 4978 368

263 4979 356

263 4980 339

263 4981 301

263 4982 182

264 51 524

264 63 692

264 65 3921

264 67 429

264 69 431

264 72 4196

264 75 227

264 77 1963

264 100 211

264 157 741

264 166 654

264 174 273

264 175 280

264 176 752

264 182 2501

264 186 720

264 187 1060

264 366 534

264 384 732

264 405 816

264 486 353

264 501 1121

264 526 243

264 722 984

264 877 660

264 1001 594

264 1009 294

264 1012 214

264 1014 1131

264 1076 263

264 1292 331

264 1464 960

264 2176 246

264 2610 288

264 3280 464

264 3966 483

264 4024 277

264 4075 1863

264 4880 325

264 4983 3873

264 4984 3375

264 4985 1909

264 4986 1579

264 4987 902

264 4988 558

264 4989 480

264 4990 409

264 4991 343

264 4992 271

264 4993 201

265 154 261

265 198 278

265 227 173

265 229 152

265 233 739

265 234 1669

265 412 241

265 489 291

265 492 146

265 498 248

265 501 299

265 512 7586

265 517 3749

265 632 415

265 707 1203

265 779 1637

265 809 284

265 815 881

265 816 234

265 825 217

265 841 851

265 843 1238

265 845 527

265 949 433

265 977 132

265 1044 1587

265 1100 243

265 1116 311

265 1118 200

265 1145 617

265 1195 885

265 1196 309

265 1360 284

265 1513 285

265 1541 4500

265 1555 216

265 1557 308

265 2429 398

265 2608 231

265 2744 170

265 2850 623

265 3028 130

265 3718 2011

265 3721 584

265 4099 148

265 4166 241

265 4281 281

265 4947 133

265 4994 451

265 4995 142

266 51 55

266 65 157

266 71 217
266 155 65
266 159 88
266 171 149
266 173 85
266 182 295
266 184 146
266 185 77
266 187 79
266 190 87
266 203 234
266 221 311
266 229 227
266 360 80
266 511 110
266 533 167
266 538 100
266 620 130
266 712 59
266 716 96
266 734 54
266 851 107
266 905 58
266 962 87
266 969 62
266 1090 65
266 1375 170
266 1377 74
266 1381 55
266 1383 60

266 1384 101
266 1385 69
266 1505 107
266 1795 60
266 1892 53
266 2444 61
266 2614 105
266 3071 57
266 3119 66
266 3171 56
266 3255 62
266 3273 69
266 3466 70
266 3676 71
266 4996 291
266 4997 110
266 4998 74
266 4999 55
267 89 186
267 182 299
267 191 420
267 221 548
267 288 334
267 289 629
267 291 265
267 292 218
267 300 821
267 304 366
267 305 221
267 307 995

267 318 261

267 324 528

267 325 441

267 344 2688

267 352 207

267 362 701

267 378 254

267 403 574

267 437 265

267 456 1375

267 458 456

267 461 332

267 464 638

267 466 294

267 498 555

267 528 242

267 531 209

267 548 611

267 680 628

267 681 224

267 683 247

267 686 429

267 690 509

267 786 1632

267 908 207

267 1043 354

267 1375 223

267 1389 1212

267 1447 290

267 1459 1253

267 1462 216

267 1934 351

267 2392 460

267 3319 200

267 3756 440

267 5000 295

267 5001 276

267 5002 207

268 180 144

268 706 93

268 707 220

268 732 357

268 821 136

268 841 195

268 952 164

268 954 277

268 1104 151

268 1180 162

268 1249 313

268 1348 260

268 1360 235

268 1366 3361

268 1372 80

268 1412 101

268 1523 162

268 1803 3061

268 1808 81

268 1810 425

268 2031 191

268 2341 113

268 2343 567
268 2746 413
268 2781 509
268 2784 275
268 2787 85
268 2788 811
268 2790 2374
268 2973 99
268 3474 209
268 3480 346
268 3966 221
268 4165 148
268 4180 706
268 4880 135
268 5003 601
268 5004 340
268 5005 224
268 5006 223
268 5007 177
268 5008 149
268 5009 129
268 5010 128
268 5011 102
268 5012 100
268 5013 85
268 5014 85
268 5015 83
268 5016 78
269 7 18
269 51 26

269 65 23

269 70 539

269 72 81

269 88 27

269 155 52

269 161 65

269 163 54

269 173 15

269 184 602

269 190 37

269 203 612

269 229 589

269 230 12

269 245 74

269 250 64

269 377 42

269 403 78

269 460 67

269 483 63

269 511 14

269 599 80

269 706 151

269 951 386

269 959 40

269 968 24

269 986 15

269 1186 471

269 1464 17

269 1790 13

269 1795 23

269 2031 18
269 2383 29
269 2738 17
269 2747 40
269 3462 12
269 3827 43
269 3966 58
269 4241 78
269 4541 38
269 5017 1461
269 5018 165
269 5019 74
269 5020 41
269 5021 30
269 5022 22
269 5023 22
269 5024 21
269 5025 14
270 81 1
270 432 2
270 533 1
270 969 1
270 1186 2
270 2815 1
270 2818 1
270 2829 1
270 5026 2
270 5027 1
270 5028 1
270 5029 1

270 5030 1

270 5031 1

271 55 840

271 67 1215

271 89 5396

271 257 2019

271 288 2538

271 289 41112

271 290 2330

271 292 1648

271 293 824

271 294 833

271 295 10110

271 299 590

271 300 5365

271 302 1641

271 306 792

271 308 1463

271 314 829

271 325 1407

271 333 1811

271 344 891

271 349 3210

271 352 934

271 461 1563

271 466 790

271 523 3770

271 528 1028

271 543 741

271 646 1023

271 681 921

271 701 1022

271 797 506

271 908 822

271 913 755

271 1051 643

271 1428 677

271 1451 1003

271 1457 517

271 1459 920

271 1580 900

271 1937 544

271 2523 454

271 2548 8530

271 4563 2004

271 4566 705

271 4707 579

271 5032 1562

271 5033 1477

271 5034 957

271 5035 546

271 5036 485

273 45 498

273 198 723

273 289 347

273 331 272

273 371 495

273 377 917

273 378 269

273 489 281

273 498 347

273 499 352

273 500 371

273 516 439

273 527 752

273 680 355

273 779 676

273 815 480

273 816 301

273 818 296

273 830 682

273 930 284

273 1043 329

273 1185 412

273 1541 2356

273 1590 384

273 1853 358

273 1868 350

273 2214 293

273 2430 530

273 2618 399

273 2956 635

273 2959 419

273 3807 372

273 3812 435

273 4934 710

273 5060 1968

273 5061 1281

273 5062 757

273 5063 661

273 5064 637

273 5065 543

273 5066 535

273 5067 482

273 5068 470

273 5069 424

273 5070 347

273 5071 343

273 5072 332

273 5073 327

273 5074 292

273 5075 274

274 1 483

274 7 216

274 8 175

274 72 7386

274 157 1261

274 265 552

274 374 295

274 375 2046

274 385 128

274 390 746

274 395 118

274 396 373

274 397 169

274 401 99

274 877 137

274 1072 184

274 1713 164

274 1857 152

274 1910	462
274 1918	104
274 1930	210
274 2000	90
274 2101	109
274 2102	1024
274 2346	274
274 3267	118
274 3324	689
274 3494	165
274 4558	124
274 5076	1365
274 5077	910
274 5078	560
274 5079	487
274 5080	316
274 5081	264
274 5082	247
274 5083	241
274 5084	222
274 5085	212
274 5086	199
274 5087	197
274 5088	145
274 5089	145
274 5090	143
274 5091	126
274 5092	105
274 5093	95
274 5094	94

274 5095 94

274 5096 86

275 30 33

275 51 77

275 59 17

275 65 42

275 72 179

275 75 76

275 99 24

275 100 31

275 153 42

275 154 22

275 155 15

275 159 65

275 173 23

275 187 62

275 191 31

275 193 64

275 436 20

275 511 23

275 533 16

275 878 16

275 951 15

275 961 20

275 993 46

275 997 50

275 998 55

275 999 57

275 1001 79

275 1013 21

275	1014	112
275	1019	73
275	1027	22
275	1061	39
275	1072	101
275	1075	15
275	1375	16
275	1713	95
275	1772	17
275	1885	21
275	1886	31
275	1892	24
275	1895	21
275	3427	18
275	3617	30
275	3768	56
275	3988	78
275	4130	22
275	4308	25
275	5097	279
275	5098	24
275	5099	16
277	10	532
277	13	639
277	15	370
277	17	457
277	26	549
277	30	552
277	31	528
277	154	10470

277 159 4650

277 162 397

277 182 1550

277 190 20074

277 198 1991

277 220 3460

277 227 1180

277 229 3002

277 234 407

277 237 631

277 425 466

277 515 393

277 755 1053

277 758 1713

277 954 548

277 1090 366

277 1132 821

277 1277 554

277 1297 497

277 1299 432

277 1326 734

277 1632 1561

277 1788 768

277 1873 2828

277 1974 971

277 3184 519

277 3244 397

277 3432 629

277 3707 1178

277 4225 1045

277 4226 2734

277 4228 1036

277 4312 390

277 4887 1240

277 5111 1628

277 5112 1025

277 5113 794

277 5114 683

277 5115 643

277 5116 465

277 5117 451

277 5118 449

278 55 388

278 67 982

278 89 85

278 157 448

278 167 121

278 257 242

278 289 7104

278 291 405

278 292 877

278 293 319

278 298 111

278 300 47

278 306 57

278 316 41

278 318 124

278 329 112

278 331 54

278 333 312

278 377 60

278 378 48

278 386 722

278 461 38

278 466 58

278 468 270

278 475 90

278 481 109

278 498 84

278 523 58

278 544 125

278 679 633

278 701 51

278 789 42

278 951 158

278 966 101

278 970 148

278 972 38

278 1243 71

278 1450 74

278 1459 345

278 1556 70

278 1580 93

278 3795 105

278 4560 172

278 4704 73

278 5119 132

278 5120 85

278 5121 69

278 5122 59

278 5123 46

278 5124 44

279 89 236

279 306 307

279 486 533

279 494 283

279 500 222

279 779 687

279 809 149

279 823 2540

279 839 547

279 1181 307

279 1185 286

279 1188 215

279 1192 1378

279 1196 2406

279 1551 2098

279 1555 464

279 2192 441

279 2200 9796

279 2386 647

279 2387 262

279 2426 2216

279 2586 1011

279 4168 179

279 4375 870

279 4821 4183

279 4822 269

279 4830 716

279 5067 988

279 5125 2510

279 5126 2058

279 5127 1583

279 5128 1381

279 5129 929

279 5130 770

279 5131 633

279 5132 536

279 5133 501

279 5134 407

279 5135 356

279 5136 336

279 5137 208

279 5138 199

279 5139 199

279 5140 189

279 5141 183

279 5142 163

279 5143 161

279 5144 160

279 5145 155

279 5146 143

280 7 81

280 38 69

280 56 164

280 58 67

280 67 75

280 89 1054

280 97 248

280 163 77

280 167 950

280 190 133

280 292 156

280 295 121

280 300 86

280 331 223

280 420 709

280 425 473

280 437 192

280 450 110

280 526 153

280 539 217

280 599 78

280 689 181

280 701 79

280 703 173

280 704 66

280 709 85

280 757 201

280 1073 263

280 1098 99

280 1243 131

280 1360 115

280 1379 364

280 1398 79

280 1401 115

280 1537 102

280 1700 171

280 1934 429

280 1941 69

280 2083	574

280 3200	75

280 3739	280

280 3902	84

280 5147	959

280 5148	438

280 5149	395

280 5150	392

280 5151	244

280 5152	95

280 5153	77

280 5154	64

281 157 48

281 176 72

281 234 12

281 377 11

281 614 19

281 707 16

281 716 16

281 724 46

281 732 134

281 854 13

281 917 2420

281 918 14

281 922 169

281 928 10

281 951 34

281 952 16

281 956 17

281 1249	63

281 1343	16
281 1358	55
281 1369	85
281 1803	21
281 1808	166
281 2183	13
281 2342	26
281 2343	394
281 2558	89
281 2595	10
281 2646	36
281 2834	33
281 2936	10
281 2973	60
281 3471	41
281 4124	158
281 5155	96
281 5156	60
281 5157	32
281 5158	31
281 5159	30
281 5160	25
281 5161	21
281 5162	21
281 5163	15
281 5164	13
281 5165	11
281 5166	11
281 5167	11
281 5168	10

281 5169 10

281 5170 10

282 152 559

282 163 2504

282 220 630

282 226 248

282 227 2523

282 234 749

282 436 322

282 562 545

282 607 507

282 614 6458

282 706 219

282 707 972

282 724 331

282 728 203

282 854 1066

282 856 421

282 868 494

282 881 333

282 910 445

282 917 1933

282 959 842

282 1110 7467

282 1119 274

282 1122 1188

282 1125 335

282 1131 245

282 1249 506

282 1358 729

282	1360	319
282	1369	1490
282	1372	2077
282	1411	518
282	1412	2389
282	1413	308
282	1416	651
282	1639	267
282	2342	346
282	2343	331
282	2346	322
282	2375	606
282	2446	504
282	3481	622
282	4051	718
282	4782	343
282	4784	1080
282	5171	487
282	5172	450
282	5173	442
282	5174	326
282	5175	260
283	52	68
283	54	133
283	57	174
283	66	91
283	80	79
283	81	193
283	238	119
283	328	59

283 1009	58
283 1395	78
283 1407	64
283 1787	101
283 1790	61
283 1801	78
283 1824	95
283 2605	77
283 3109	119
283 3121	65
283 3132	148
283 3164	67
283 3868	95
283 3992	71
283 3995	57
283 4316	236
283 4863	63
283 5176	224
283 5177	135
283 5178	131
283 5179	128
283 5180	123
283 5181	118
283 5182	112
283 5183	98
283 5184	96
283 5185	94
283 5186	92
283 5187	86
283 5188	82

283 5189 80
283 5190 76
283 5191 74
283 5192 73
283 5193 73
283 5194 73
283 5195 72
283 5196 71
283 5197 69
283 5198 68
283 5199 61
283 5200 59
284 154 4
284 198 5
284 489 6
284 512 14
284 517 6
284 712 4
284 818 5
284 868 7
284 959 6
284 982 8
284 1098 4
284 1116 5
284 1118 7
284 1131 11
284 1184 8
284 1186 7
284 1248 6
284 1372 5

284 1415	7
284 1426	5
284 1806	4
284 1994	22
284 2150	16
284 2352	12
284 2580	27
284 2594	27
284 2794	17
284 2855	6
284 2964	5
284 3067	5
284 3944	13
284 4458	6
284 4994	9
284 5201	30
284 5202	26
284 5203	12
284 5204	12
284 5205	11
284 5206	9
284 5207	8
284 5208	7
284 5209	7
284 5210	7
284 5211	6
284 5212	5
284 5213	5
284 5214	5
284 5215	4

284 5216 4

284 5217 4

285 65 310

285 88 43

285 198 143

285 220 147

285 234 360

285 283 171

285 298 59

285 300 148

285 316 111

285 377 122

285 383 56

285 412 84

285 439 329

285 533 42

285 538 193

285 542 188

285 614 224

285 706 220

285 707 222

285 714 181

285 854 340

285 917 419

285 932 45

285 957 79

285 966 164

285 979 53

285 982 322

285 1206 1596

285 1286 126

285 1358 228

285 1360 408

285 1377 41

285 1513 137

285 1873 144

285 2346 42

285 3205 267

285 3540 65

285 3670 55

285 3780 58

285 3819 59

285 5218 735

285 5219 83

285 5220 80

285 5221 70

285 5222 59

285 5223 59

285 5224 57

285 5225 56

285 5226 49

285 5227 45

286 67 141

286 89 2146

286 257 1776

286 265 186

286 285 133

286 288 520

286 289 622

286 290 99

286 291 354

286 292 157

286 294 111

286 295 241

286 300 231

286 301 288

286 302 124

286 304 154

286 306 96

286 310 114

286 317 80

286 320 249

286 323 310

286 326 206

286 327 453

286 329 86

286 333 154

286 344 911

286 349 781

286 378 571

286 386 128

286 455 1030

286 456 83

286 461 122

286 468 110

286 484 162

286 485 80

286 498 376

286 525 180

286 538 98

286 545 124

286 546 154

286 679 240

286 701 148

286 980 162

286 1450 79

286 1556 194

286 2977 184

286 3193 87

286 4260 171

286 4817 97

286 5228 96

288 65 330

288 88 445

288 163 195

288 190 444

288 220 517

288 225 618

288 227 1545

288 229 164

288 230 917

288 412 199

288 436 209

288 475 173

288 497 176

288 517 325

288 533 211

288 707 2057

288 815 208

288 917 288

288 920 1111

288 982 263

288	1092	222
288	1106	239
288	1118	212
288	1398	491
288	1400	623
288	1406	293
288	1412	255
288	1513	349
288	1672	282
288	1816	941
288	1974	251
288	3892	1234
288	5235	2091
288	5236	1302
288	5237	1037
288	5238	915
288	5239	786
288	5240	723
288	5241	671
288	5242	498
288	5243	469
288	5244	462
288	5245	285
288	5246	247
288	5247	236
288	5248	219
288	5249	207
288	5250	202

288 5251 190

288 5252 165

289 65 823

289 89 267

289 154 639

289 163 340

289 190 7401

289 203 609

289 207 16566

289 220 286

289 227 835

289 228 968

289 229 637

289 230 509

289 300 2166

289 324 707

289 328 224

289 355 201

289 377 495

289 403 3021

289 412 4119

289 424 580

289 432 2289

289 464 673

289 470 4338

289 475 221

289 481 839

289 486 1698

289 498 4881

289 501 469

289 533 370

289 538 1214

289 540 295

289 547 1263

289 691 202

289 693 535

289 757 807

289 779 2219

289 816 3789

289 898 345

289 1043 262

289 1106 327

289 1186 280

289 1246 888

289 1390 431

289 1400 499

289 1512 239

289 1541 395

289 1672 301

289 3373 1089

289 3398 1018

289 5253 348

290 51 23

290 67 25

290 68 6

290 70 4

290 77 17

290 82 8

290 99 4

290 159 3

290 193 5

290 203 6

290 475 11

290 599 7

290 601 5

290 959 4

290 1001 11

290 1019 6

290 1031 4

290 1076 6

290 1079 22

290 1081 6

290 1090 4

290 1099 5

290 1227 5

290 1409 4

290 1479 4

290 1504 3

290 1686 4

290 1895 5

290 2342 5

290 2531 4

290 2571 4

290 2788 5

290 3281 4

290 3739 12

290 3768 8

290 3832 7

290 3920 7

290 4785 4

290 5254 18

290 5255 14

290 5256 8

290 5257 6

290 5258 6

290 5259 6

290 5260 5

290 5261 5

290 5262 5

290 5263 5

290 5264 4

290 5265 4

291 289 20

291 291 870

291 300 143

291 325 348

291 333 628

291 344 79144

291 461 266

291 466 452

291 498 562

291 679 376

291 681 337

291 686 290

291 975 1

291 1447 736

291 1459 2582

291 1486 4

291 1601 127

291 2020 75

291 3603	21
291 4706	27
291 4815	54
291 5266	273
291 5267	135
291 5268	88
291 5269	82
291 5270	76
291 5271	56
291 5272	54
291 5273	54
291 5274	44
291 5275	31
291 5276	31
291 5277	29
291 5278	29
291 5279	29
291 5280	28
291 5281	28
291 5282	27
291 5283	27
291 5284	27
291 5285	26
291 5286	26
291 5287	26
291 5288	25
291 5289	25
291 5290	25
291 5291	22
291 5292	22

291 5293 21

291 5294 2

292 61 412

292 152 875

292 154 1778

292 159 175

292 163 123065

292 199 539

292 212 175

292 225 165

292 227 2213

292 233 325

292 418 253

292 436 191

292 441 1132

292 607 236

292 610 440

292 613 329

292 707 169

292 735 191

292 863 402

292 868 833

292 879 3321

292 959 238

292 1110 195

292 1131 659

292 1169 150

292 1369 319

292 1411 2453

292 1412 463

292 1425 568

292 1426 225

292 1709 572

292 1806 360

292 2475 524

292 3481 1150

292 3511 468

292 4064 166

292 4675 242

292 4691 1300

292 4780 231

292 4784 148

292 4790 1693

292 5295 1008

292 5296 1005

292 5297 949

292 5298 438

292 5299 289

292 5300 203

292 5301 168

292 5302 154

292 5303 146

293 227 139

293 230 127

293 344 50

293 412 411

293 461 55

293 498 54

293 501 585

293 585 59

293 679 169

293 687 281

293 690 48

293 691 473

293 701 78

293 718 435

293 1039 148

293 1130 48

293 1145 48

293 1186 36

293 1351 38

293 1513 173

293 1672 110

293 1686 91

293 1689 4017

293 2467 90

293 2478 171

293 4385 117

293 4436 67

293 4559 105

293 4936 62

293 5304 922

293 5305 892

293 5306 684

293 5307 442

293 5308 350

293 5309 312

293 5310 218

293 5311 196

293 5312 155

293 5313 151

293 5314 140

293 5315 139

293 5316 120

293 5317 113

293 5318 96

293 5319 95

293 5320 72

293 5321 44

293 5322 43

293 5323 36

293 5324 34

294 220 67

294 286 178

294 300 93

294 397 144

294 706 51

294 782 166

294 1590 97

294 2055 83

294 2796 122

294 5173 64

294 5325 246

294 5326 228

294 5327 164

294 5328 161

294 5329 139

294 5330 136

294 5331 133

294 5332 127

294 5333	123
294 5334	121
294 5335	116
294 5336	110
294 5337	109
294 5338	109
294 5339	99
294 5340	98
294 5341	92
294 5342	90
294 5343	87
294 5344	87
294 5345	84
294 5346	83
294 5347	82
294 5348	79
294 5349	78
294 5350	76
294 5351	73
294 5352	70
294 5353	69
294 5354	61
294 5355	60
294 5356	60
294 5357	59
294 5358	58
294 5359	57
294 5360	55
294 5361	55
294 5362	55

294 5363 51

294 5364 51

295 163 725

295 198 332

295 220 291

295 503 841

295 706 518

295 707 391

295 732 322

295 735 308

295 841 614

295 843 672

295 917 958

295 918 459

295 922 361

295 923 391

295 942 329

295 949 355

295 953 251

295 954 859

295 959 343

295 1093 343

295 1261 641

295 1275 236

295 1338 303

295 1342 257

295 1352 238

295 1358 484

295 1360 1897

295 1364 402

295 1366 287

295 1369 553

295 1372 480

295 1412 1410

295 1513 284

295 1520 574

295 1522 313

295 1523 240

295 1527 1131

295 1531 313

295 1535 316

295 1803 334

295 1992 356

295 2342 249

295 2343 690

295 2838 275

295 3444 353

295 3476 620

295 3509 258

295 4273 258

295 5365 644

295 5366 311

296 563 2

296 570 3

296 718 2

296 1160 2

296 1415 2

296 2902 6

296 3896 4

296 5367 27

296 5368	10
296 5369	10
296 5370	7
296 5371	7
296 5372	6
296 5373	6
296 5374	6
296 5375	5
296 5376	5
296 5377	5
296 5378	5
296 5379	5
296 5380	5
296 5381	4
296 5382	4
296 5383	4
296 5384	4
296 5385	4
296 5386	4
296 5387	4
296 5388	4
296 5389	4
296 5390	4
296 5391	4
296 5392	3
296 5393	3
296 5394	3
296 5395	3
296 5396	3
296 5397	3

296 5398 3

296 5399 3

296 5400 3

296 5401 3

296 5402 3

296 5403 3

296 5404 3

296 5405 3

296 5406 3

296 5407 3

296 5408 3

296 5409 2

297 154 698

297 173 642

297 196 342

297 199 869

297 212 699

297 222 769

297 225 427

297 226 567

297 227 2118

297 231 419

297 425 1064

297 429 1075

297 439 1402

297 554 433

297 618 1951

297 622 346

297 629 1000

297 709 1799

297 858 550	
297 1090	418
297 1098	846
297 1109	330
297 1159	601
297 1231	1989
297 1424	2947
297 1504	585
297 1732	629
297 2281	652
297 2532	622
297 3057	951
297 3200	1002
297 3259	357
297 3469	472
297 3902	1167
297 4793	412
297 5410	761
297 5411	593
297 5412	518
297 5413	472
297 5414	435
297 5415	426
297 5416	424
297 5417	409
297 5418	405
297 5419	344
297 5420	342
297 5421	336
297 5422	334

297 5423 332

297 5424 329

298 207 316

298 208 598

298 209 608

298 212 531

298 227 25018

298 246 204

298 324 618

298 439 179

298 562 1274

298 733 2757

298 903 73771

298 1045 1048

298 1048 280

298 1100 1347

298 1116 391

298 1160 430

298 1195 340

298 1244 227

298 1390 207

298 1416 5726

298 1469 908

298 1763 1602

298 1766 356

298 1934 583

298 2176 353

298 2184 2601

298 2591 201

298 2608 177

298 2666 790

298 3200 199

298 3205 396

298 4104 682

298 4251 1930

298 4385 327

298 5425 3304

298 5426 974

298 5427 877

298 5428 791

298 5429 779

298 5430 712

298 5431 608

298 5432 471

298 5433 463

298 5434 447

298 5435 393

298 5436 378

298 5437 237

298 5438 204

298 5439 202

298 5440 193

299 55 448

299 65 377

299 67 221

299 89 215

299 182 131

299 184 236

299 207 799

299 210 563

299 221 144

299 229 290

299 289 427

299 295 148

299 298 453

299 331 521

299 342 526

299 366 230

299 421 266

299 424 377

299 430 201

299 439 157

299 498 115

299 538 257

299 546 170

299 716 223

299 903 488

299 1048 408

299 1090 103

299 1515 118

299 1556 351

299 1609 484

299 1763 110

299 1772 424

299 1775 342

299 2544 419

299 2858 271

299 3200 212

299 3204 195

299 3293 149

299 4716 164

299 4818 200

299 4828 112

299 5441 1097

299 5442 173

299 5443 166

299 5444 151

299 5445 128

299 5446 123

299 5447 108

299 5448 106

299 5449 101

300 176 158

300 187 371

300 189 445

300 198 280

300 212 617

300 438 1600

300 439 159

300 511 296

300 517 138

300 614 223

300 705 2804

300 706 390

300 707 253

300 708 519

300 714 572

300 716 409

300 718 415

300 730 596

300 932 2605

300 946 2227

300 1044	154
300 1132	180
300 1241	159
300 1249	374
300 1338	141
300 1350	191
300 1368	943
300 1415	2248
300 1545	225
300 1810	236
300 2346	454
300 2347	179
300 2660	511
300 2663	1144
300 2797	117
300 2986	610
300 3204	820
300 4087	435
300 4349	492
300 4473	126
300 4793	941
300 5441	132
300 5450	356
300 5451	302
300 5452	269
300 5453	247
300 5454	214
300 5455	193

300 5456 188

300 5457 130

301 67 374

301 89 608

301 97 167

301 173 896

301 228 164

301 283 256

301 288 286

301 289 590

301 292 241

301 295 276

301 298 456

301 300 132

301 302 332

301 316 398

301 333 490

301 430 121

301 461 286

301 498 345

301 523 441

301 525 149

301 539 359

301 599 294

301 646 135

301 704 285

301 705 161

301 706 288

301 735 849

301 739 149

301 802 164

301 898 154

301 903 250

301 959 122

301 982 254

301 985 225

301 1246 143

301 1249 694

301 1400 133

301 1464 184

301 1767 775

301 1775 308

301 1803 114

301 1934 232

301 1988 168

301 2184 126

301 2343 461

301 2346 366

301 2988 164

301 3200 276

301 3741 140

301 5458 132

302 55 436

302 89 21536

302 265 183

302 288 718

302 289 1038

302 292 7945

302 299 214

302 300 184

302	302	295
302	306	177
302	308	191
302	311	322
302	333	225
302	340	1991
302	352	598
302	360	1710
302	466	2260
302	518	7609
302	524	190
302	532	292
302	543	1056
302	669	1414
302	679	1736
302	701	1092
302	704	3375
302	930	568
302	1032	245
302	1034	1909
302	1037	924
302	1114	5362
302	1246	559
302	1263	335
302	1670	228
302	1934	201
302	2521	241
302	2981	727
302	3572	245
302	4137	434

302 4814 3781

302 5079 192

302 5459 1626

302 5460 1237

302 5461 1223

302 5462 406

302 5463 361

302 5464 333

302 5465 277

302 5466 204

302 5467 192

302 5468 184

303 56 422

303 88 228

303 159 924

303 163 318

303 198 155

303 226 325

303 227 253

303 228 1265

303 234 372

303 250 148

303 383 161

303 412 811

303 441 667

303 549 948

303 562 430

303 703 323

303 706 444

303 707 254

303 709 235

303 712 194

303 843 142

303 857 483

303 859 406

303 868 210

303 1206 175

303 1274 467

303 1369 603

303 1406 152

303 1412 421

303 1415 525

303 1426 198

303 1504 747

303 1513 1489

303 1804 383

303 1873 167

303 1974 642

303 1979 158

303 2347 263

303 2834 198

303 2853 215

303 2855 150

303 2901 581

303 3398 134

303 3792 153

303 4115 1048

303 4129 302

303 5172 196

303 5469 702

303 5470 324

303 5471 206

304 16 19

304 65 20

304 72 771

304 84 26

304 153 88

304 156 45

304 159 173

304 163 19

304 171 34

304 172 42

304 173 61

304 190 23

304 193 25

304 208 36

304 227 27

304 233 31

304 436 27

304 450 67

304 487 56

304 547 61

304 689 149

304 757 82

304 961 77

304 1048 62

304 1075 32

304 1083 53

304 1379 158

304 1384 29

304 1534 34

304 1713 49

304 1807 23

304 1824 136

304 2405 31

304 2605 43

304 2813 121

304 3183 22

304 3201 75

304 3258 65

304 3501 35

304 5079 58

304 5472 36

304 5473 32

304 5474 31

304 5475 29

304 5476 27

304 5477 21

304 5478 20

304 5479 20

304 5480 19

304 5481 19

305 55 116

305 65 42

305 89 2468

305 161 436

305 228 120

305 288 1176

305 289 600

305 292 352

305 294 2215

305 295 222

305 299 105

305 300 723

305 302 106

305 306 728

305 310 53

305 311 118

305 327 163

305 328 111

305 329 48

305 331 66

305 333 127

305 344 130

305 352 201

305 377 124

305 386 77

305 461 603

305 464 127

305 466 347

305 475 46

305 484 56

305 486 102

305 538 153

305 543 128

305 544 447

305 679 128

305 686 852

305 701 901

305 704 204

305 802 434

305 1032 294

305 1037 1201

305 1042 181

305 1443 41

305 1444 56

305 1447 380

305 1475 61

305 1672 520

305 1934 357

305 2990 176

305 4562 52

306 7 88

306 30 626

306 51 76

306 59 64

306 67 69

306 72 3706

306 75 293

306 99 328

306 153 102

306 159 175

306 187 143

306 234 110

306 250 49

306 377 125

306 441 311

306 455 73

306 463 452

306 467 346

306 486 113

306 497 105

306 511 66

306 521 77

306 707 61

306 868 69

306 877 189

306 880 87

306 978 59

306 1001 111

306 1014 59

306 1055 50

306 1061 71

306 1072 113

306 1075 74

306 1131 50

306 1298 97

306 1379 181

306 1713 611

306 2783 47

306 3683 96

306 3768 49

306 3988 89

306 5482 238

306 5483 165

306 5484 80

306 5485 74

306 5486 60

306 5487 58

306 5488 58

306 5489 51

306 5490 50

307 57 110

307 70 67

307 782 74

307 1226 183

307 1255 63

307 1269 74

307 1288 365

307 4725 134

307 5073 94

307 5327 83

307 5342 70

307 5346 79

307 5347 170

307 5348 67

307 5358 168

307 5360 79

307 5491 342

307 5492 244

307 5493 152

307 5494 146

307 5495 120

307 5496 115

307 5497 110

307 5498 104

307 5499 100

307 5500 97

307 5501 96

307 5502 93

307 5503 87

307 5504 85

307 5505 84

307 5506 84

307 5507 83

307 5508 79

307 5509 78

307 5510 77

307 5511 76

307 5512 74

307 5513 72

307 5514 72

307 5515 72

307 5516 70

307 5517 69

307 5518 66

307 5519 64

307 5520 64

307 5521 63

307 5522 63

307 5523 62

307 5524 62

308 163 511

308 190 1937

308 198 462

308 227 838

308 497 609

308 500 2145

308 706 1730

308 707 1204

308 713 950

308 724 1015

308 732 809

308 804 586

308 808 2694

308 810 682

308 813 1129

308 815 562

308 816 1448

308 830 1020

308 837 456

308 917 2444

308 918 922

308 920 479

308 923 908

308 924 739

308 946 786

308 951 3003

308 952 837

308 953 2221

308 982 466

308 1110 467

308 1180 1313

308 1249 1265

308 1350 544

308 1372 703

308 1412 1310

308 1672 4401

308 1905 1074

308 2343 1854

308 2346 1710

308 2347 659

308 2363 872

308 2379 1551

308 2576 1080

308 2834 1165

308 2941 1033

308 4180 1509

308 4460 910

308 4670 443

308 5525 518

308 5526 444

310 55 394

310 89 52

310 161 56

310 288 202

310 289 97

310 292 7099

310 294 153

310 296 91

310 300 1718

310 302 57

310 306 219

310 317 67

310 323 2962

310 327 78

310 328 55

310 330 106

310 352 1411

310 458 111

310	461	339
310	466	90
310	468	50
310	475	6259
310	527	55
310	528	169
310	531	106
310	543	60
310	548	63
310	906	1093
310	915	80
310	1037	1285
310	1051	54
310	1409	617
310	1451	109
310	1455	62
310	1463	86
310	2607	77
310	2939	421
310	4491	75
310	4562	69
310	4812	93
310	5528	153
310	5529	153
310	5530	93
310	5531	78
310	5532	76
310	5533	71
310	5534	71
310	5535	57

310 5536 55
310 5537 52
311 58 224
311 65 209
311 166 287
311 172 219
311 199 622
311 206 261
311 225 771
311 235 191
311 298 167
311 300 293
311 416 1286
311 534 304
311 604 236
311 679 2144
311 696 597
311 862 172
311 898 204
311 1001 194
311 1244 227
311 1632 406
311 1740 344
311 2137 221
311 3026 167
311 3108 237
311 3119 236
311 3391 1500
311 3742 268
311 4618 380

311 5538 857

311 5539 824

311 5540 670

311 5541 636

311 5542 633

311 5543 574

311 5544 535

311 5545 436

311 5546 393

311 5547 367

311 5548 342

311 5549 322

311 5550 307

311 5551 304

311 5552 255

311 5553 252

311 5554 229

311 5555 208

311 5556 195

311 5557 194

311 5558 161

311 5559 160

312 89 329

312 324 324

312 367 825

312 369 697

312 375 1497

312 388 274

312 393 442

312 394 314

312 395 344

312 399 1851

312 413 266

312 679 723

312 1906	815
312 1918	478
312 2086	412
312 2088	415
312 2092	784
312 2094	2449
312 2097	415
312 2098	628
312 2102	1355
312 2106	290
312 2799	327
312 2800	362
312 2808	348
312 3193	319
312 3324	953
312 3712	684
312 4558	311
312 5080	521
312 5081	397
312 5083	327
312 5089	312
312 5560	968
312 5561	756
312 5562	609
312 5563	603
312 5564	601

312 5565 574

312 5566 431

312 5567 402

312 5568 401

312 5569 368

312 5570 364

312 5571 321

312 5572 314

312 5573 313

312 5574 312

312 5575 298

312 5576 267

313 9 761

313 10 267

313 11 138

313 15 100

313 25 659

313 34 424

313 176 116

313 181 160

313 504 253

313 517 287

313 807 472

313 837 382

313 843 249

313 917 82773

313 918 107

313 922 4391

313 928 151

313 930 293

313	936	728
313	1079	133
313	1104	414
313	1249	169
313	1299	130
313	1339	382
313	1347	327
313	1361	93
313	1366	219
313	1369	280
313	1711	185
313	2787	99
313	2961	764
313	2975	350
313	3244	125
313	3470	403
313	3774	444
313	3798	107
313	3806	264
313	4161	383
313	4319	131
313	4326	134
313	5577	279
313	5578	259
313	5579	239
313	5580	169
313	5581	157
313	5582	122
313	5583	119
313	5584	106

313 5585 96

313 5586 93

314 89 221

314 166 120

314 176 535

314 184 171

314 220 118

314 257 209

314 300 453

314 302 122

314 316 196

314 333 139

314 344 257

314 355 499

314 378 235

314 458 207

314 461 781

314 466 139

314 472 165

314 486 225

314 498 963

314 499 318

314 681 260

314 683 400

314 714 137

314 966 298

314 1052 156

314 1249 149

314 1447 116

314 1681 150

314 2000 554

314 2352 249

314 2545 242

314 2549 137

314 2986 275

314 3378 592

314 3393 644

314 3859 278

314 4177 149

314 4463 339

314 4470 190

314 4471 157

314 4474 226

314 5587 691

314 5588 488

314 5589 308

314 5590 173

314 5591 172

314 5592 155

314 5593 141

314 5594 128

314 5595 126

315 53 308

315 56 1225

315 65 5367

315 88 371

315 89 289

315 154 1519

315 173 331

315 190 2890

315 199 718

315 203 206

315 207 762

315 209 234

315 220 291

315 227 1495

315 228 1047

315 229 868

315 230 602

315 298 251

315 300 385

315 302 266

315 306 338

315 316 314

315 328 718

315 331 167

315 377 483

315 424 243

315 475 220

315 486 285

315 511 172

315 525 365

315 526 247

315 533 622

315 534 500

315 546 390

315 614 209

315 679 410

315 689 845

315 701 208

315 709 198

315 903 519

315 982 357

315 1090 395

315 1375 181

315 1379 229

315 1398 703

315 1673 193

315 2083 315

315 2277 297

315 3200 283

315 4818 188

316 25 640

316 72 401

316 198 1241

316 207 192

316 377 932

316 390 167

316 488 529

316 489 986

316 514 187

316 517 528

316 707 833

316 726 293

316 770 292

316 1044 1034

316 1104 562

316 1116 216

316 1131 376

316 1145 733

316 1870	426
316 1914	378
316 1974	458
316 2032	223
316 2038	192
316 2370	272
316 2738	259
316 2835	757
316 2846	624
316 3032	1605
316 3244	470
316 3729	376
316 4457	187
316 4504	226
316 5596	2338
316 5597	1124
316 5598	528
316 5599	525
316 5600	519
316 5601	416
316 5602	415
316 5603	399
316 5604	380
316 5605	346
316 5606	296
316 5607	284
316 5608	264
316 5609	227
316 5610	196
316 5611	186

316 5612 167

316 5613 163

317 157 166

317 159 281

317 163 857

317 212 1693

317 220 137

317 227 2380

317 234 1456

317 441 144

317 511 370

317 562 378

317 599 2315

317 630 1920

317 706 214

317 707 209

317 734 1479

317 735 3280

317 857 157

317 868 2962

317 874 255

317 903 314

317 917 174

317 959 1053

317 1103 216

317 1244 2576

317 1249 1299

317 1369 199

317 1412 246

317 1513 247

317 1639 313

317 1804 146

317 1811 1007

317 2070 11492

317 2343 201

317 2346 1355

317 2786 1167

317 3472 203

317 3478 22370

317 4541 461

317 5242 132

317 5614 6735

317 5615 4179

317 5616 1104

317 5617 1040

317 5618 468

317 5619 306

317 5620 302

317 5621 260

317 5622 242

317 5623 170

317 5624 132

318 67 196

318 89 345

318 97 2613

318 167 2638

318 210 1264

318 230 6509

318 257 1000

318 289 522

318 293 1795

318 298 6768

318 299 259

318 300 35203

318 302 711

318 306 1103

318 314 600

318 318 164

318 331 300

318 349 504

318 360 7259

318 466 8779

318 475 800

318 525 7737

318 527 2121

318 534 330

318 540 1244

318 546 219

318 547 1205

318 701 60849

318 718 1136

318 898 2624

318 1032 260

318 1037 4881

318 1040 3014

318 1047 2340

318 1186 245

318 1394 411

318 1595 333

318 1613 1006

318 2081 606

318 2083 449

318 2179 242

318 2468 211

318 3583 670

318 4189 237

318 4811 324

318 5625 309

318 5626 292

318 5627 261

318 5628 260

318 5629 207

319 207 422

319 226 277

319 383 599

319 718 209

319 808 354

319 932 215

319 964 277

319 977 585

319 1192 228

319 1195 2362

319 1206 666

319 1645 2067

319 2574 388

319 2576 283

319 2608 579

319 2637 559

319 2723 377

319 3032 582

319 3041	707
319 3043	404
319 3729	572
319 4194	420
319 4208	1165
319 4211	645
319 4346	263
319 4797	510
319 4821	761
319 4830	1011
319 4833	662
319 5306	554
319 5600	359
319 5630	2341
319 5631	1284
319 5632	1013
319 5633	871
319 5634	834
319 5635	818
319 5636	745
319 5637	726
319 5638	684
319 5639	675
319 5640	514
319 5641	419
319 5642	379
319 5643	346
319 5644	344
319 5645	317
319 5646	281

319 5647 268
319 5648 266
320 18 416
320 45 774
320 57 463
320 58 409
320 88 357
320 159 1069
320 197 542
320 222 557
320 344 661
320 366 1421
320 437 817
320 583 294
320 681 302
320 696 758
320 697 394
320 986 269
320 1123 704
320 1203 325
320 1244 1287
320 1295 320
320 1298 280
320 1694 582
320 2407 490
320 2442 801
320 2478 383
320 3057 417
320 3285 473
320 3328 293

320 3440 432
320 3611 361
320 3748 9685
320 3902 467
320 5267 889
320 5649 1155
320 5650 919
320 5651 638
320 5652 569
320 5653 488
320 5654 442
320 5655 441
320 5656 431
320 5657 406
320 5658 383
320 5659 307
320 5660 301
320 5661 292
320 5662 284
320 5663 280
320 5664 261
320 5665 254
321 7 35
321 518 59
321 706 82
321 724 51
321 813 44
321 830 37
321 834 82
321 841 66

321 846 39

321 917 116

321 918 60

321 919 53

321 930 61

321 955 94

321 1119 36

321 1195 77

321 1249 50

321 1274 49

321 1358 109

321 1360 64

321 1366 45

321 1369 110

321 1547 67

321 1672 64

321 1803 80

321 1914 61

321 1917 30

321 2014 64

321 2062 74

321 2343 127

321 2347 135

321 2363 52

321 2379 67

321 3546 81

321 4135 39

321 4136 101

321 4137 35

321 4342 41

321 4343 41

321 4356 72

321 5100 37

321 5606 33

321 5666 93

321 5667 93

321 5668 67

321 5669 56

321 5670 52

321 5671 47

321 5672 30

321 5673 30

322 89 5853

322 288 15832

322 289 706

322 292 780

322 295 866

322 300 2972

322 306 3439

322 317 784

322 327 684

322 328 499

322 331 10723

322 458 687

322 461 1310

322 464 3684

322 466 3300

322 468 1066

322 475 2480

322 486 1079

322 527 2319

322 547 5475

322 548 810

322 701 735

322 797 420

322 808 687

322 907 806

322 1037 3577

322 1042 537

322 1104 862

322 1410 538

322 1455 2691

322 1613 522

322 2242 2842

322 3036 686

322 3317 2414

322 3713 458

322 4562 1088

322 4564 2519

322 5071 1316

322 5232 872

322 5536 4061

322 5674 4662

322 5675 3268

322 5676 3058

322 5677 2281

322 5678 2237

322 5679 2040

322 5680 1173

322 5681 951

322 5682 557

322 5683 504

324 163 283

324 188 275

324 706 453

324 707 1849

324 710 466

324 724 932

324 732 666

324 821 261

324 843 301

324 917 3908

324 918 805

324 922 1022

324 930 409

324 946 353

324 957 243

324 959 338

324 1103 232

324 1111 278

324 1126 958

324 1134 354

324 1343 765

324 1345 311

324 1358 642

324 1360 554

324 1369 4101

324 1372 288

324 1412 727

324 1873 236

324 2013 337
324 2342 3440
324 2757 266
324 2787 391
324 2838 458
324 3417 1698
324 3470 728
324 3476 277
324 4611 616
324 5526 320
324 5666 1162
324 5668 231
324 5688 1000
324 5689 504
324 5690 429
324 5691 343
324 5692 304
324 5693 248
324 5694 241
324 5695 239
324 5696 234
324 5697 233
325 2 3466
325 10 722
325 11 176
325 13 263
325 70 171
325 181 300
325 506 363
325 515 690

325 878 217	
325 1293	247
325 1297	198
325 1315	484
325 1320	320
325 1324	229
325 1329	530
325 1745	348
325 1788	190
325 1800	193
325 2028	196
325 2691	225
325 2747	1062
325 3191	206
325 4912	190
325 5053	430
325 5581	224
325 5698	928
325 5699	452
325 5700	381
325 5701	333
325 5702	331
325 5703	331
325 5704	284
325 5705	283
325 5706	262
325 5707	261
325 5708	256
325 5709	229
325 5710	213

325 5711 211

325 5712 208

325 5713 206

325 5714 204

325 5715 204

325 5716 203

325 5717 187

325 5718 182

325 5719 182

325 5720 182

325 5721 180

325 5722 179

326 65 133

326 67 106

326 89 89

326 230 77

326 257 290

326 283 183

326 288 283

326 291 113

326 292 525

326 294 216

326 295 537

326 299 78

326 306 144

326 316 112

326 318 76

326 320 84

326 325 135

326 327 245

326 329 191

326 333 562

326 349 153

326 377 245

326 378 159

326 455 1930

326 457 89

326 469 470

326 472 75

326 481 139

326 484 208

326 525 90

326 536 91

326 542 110

326 543 91

326 803 136

326 908 109

326 913 162

326 951 108

326 1247 84

326 1444 146

326 1556 76

326 2080 96

326 2523 180

326 2783 95

326 3173 93

326 3332 287

326 3376 269

326 3393 91

326 3568 104

326 3748 143

326 5723 216

327 55 510

327 67 111

327 71 152

327 89 1024

327 180 296

327 229 62

327 288 1291

327 289 9017

327 292 569

327 294 179

327 295 82

327 300 936

327 331 129

327 332 149

327 352 518

327 386 128

327 466 897

327 475 410

327 531 61

327 548 206

327 686 67

327 690 93

327 1053 283

327 1057 788

327 1059 225

327 1243 1707

327 1376 390

327 1379 551

327	1413	84
327	1934	308
327	2018	258
327	2025	81
327	2130	182
327	2179	88
327	2515	203
327	2521	76
327	3319	72
327	3500	369
327	4565	764
327	4568	126
327	4572	336
327	4765	115
327	4814	291
327	5487	62
327	5724	945
327	5725	299
327	5726	149
327	5727	79
327	5728	66
327	5729	64
328	65	163
328	89	1616
328	182	217
328	190	183
328	205	164
328	207	292
328	227	282
328	229	221

328 234 233

328 289 647

328 333 205

328 377 776

328 418 629

328 424 187

328 444 892

328 447 215

328 537 245

328 868 269

328 903 199

328 972 219

328 1048 163

328 1097 1104

328 1098 456

328 1100 316

328 1109 293

328 1246 279

328 1400 680

328 1866 190

328 1986 209

328 2398 176

328 2582 294

328 3187 179

328 3193 197

328 3332 334

328 3738 207

328 5730 533

328 5731 526

328 5732 491

328 5733 414

328 5734 334

328 5735 324

328 5736 285

328 5737 254

328 5738 221

328 5739 219

328 5740 202

328 5741 198

328 5742 184

328 5743 180

328 5744 163

329 55 4954

329 65 1012

329 67 2702

329 71 774

329 89 1382

329 97 580

329 155 1526

329 207 1006

329 220 1158

329 228 521

329 229 1504

329 246 474

329 285 2352

329 288 2247

329 289 1467

329 291 2086

329 292 2486

329 295 1103

329 296 680

329 298 754

329 301 594

329 302 3828

329 310 492

329 316 1222

329 331 538

329 352 528

329 425 569

329 485 906

329 525 660

329 534 546

329 535 602

329 689 2100

329 697 966

329 701 866

329 715 1976

329 899 1987

329 906 1813

329 961 586

329 962 649

329 1098 565

329 1505 551

329 1772 734

329 1934 1352

329 2544 650

329 3110 1129

329 3466 1422

329 3866 1228

329 4810 467

329 5000 536

329 5745 1502

330 67 178

330 89 659

330 157 160

330 190 649

330 230 165

330 257 112

330 289 781

330 295 423

330 300 2627

330 306 128

330 321 321

330 324 543

330 325 1031

330 333 283

330 344 116

330 349 258

330 458 665

330 461 533

330 464 1263

330 466 439

330 498 1811

330 540 172

330 646 293

330 679 4216

330 681 257

330 686 1205

330 692 185

330 701 420

330 704 2326

330 706 567

330 802 1349

330 903 140

330 1040 152

330 1054 314

330 1180 121

330 1245 163

330 1249 130

330 1453 511

330 1458 402

330 1468 280

330 1698 1681

330 1807 121

330 2020 1198

330 2801 264

330 3748 283

330 4558 320

330 5746 1326

330 5747 225

330 5748 151

330 5749 124

331 89 130

331 289 298

331 291 143

331 292 57

331 300 506

331 302 61

331 352 139

331 362 1270

331 386 47

331 466 87

331 475 58

331 498 96

331 524 201

331 526 177

331 528 214

331 544 62

331 679 189

331 682 90

331 690 601

331 704 47

331 972 70

331 1032 148

331 1047 96

331 1181 60

331 1441 54

331 1556 103

331 2025 186

331 2432 297

331 2433 89

331 2981 47

331 3373 53

331 3397 49

331 3404 77

331 4492 79

331 4708 180

331 5750 525

331 5751 360

331 5752 350

331 5753 175

331 5754 160

331 5755 129

331 5756 127

331 5757 116

331 5758 95

331 5759 88

331 5760 82

331 5761 81

331 5762 67

331 5763 58

331 5764 48

332 7 265

332 55 32

332 97 43

332 180 33

332 228 45

332 230 781

332 256 83

332 279 155

332 290 46

332 291 1277

332 292 55784

332 300 419

332 301 167

332 302 1814

332 305 231

332 307 109

332 311 45

332 316 287

332 317 61

332 333 33

332 378 51

332 466 142

332 534 57

332 542 62

332 689 82

332 697 69

332 704 41

332 714 43

332 720 110

332 889 90

332 1062 59

332 1249 71

332 1459 83

332 1470 169

332 1556 3235

332 2019 123

332 2082 313

332 2083 45

332 2392 405

332 2786 255

332 3200 64

332 3763 217

332 4489 80

332 4575 54

332 5765 2809

332 5766 2037

332 5767 373

332 5768 109

332 5769 98

332 5770 64

333 55 52

333 67 1436

333 97 35

333 191 34

333 265 121

333 288 139

333 289 9445

333 290 30

333 291 58

333 292 381

333 295 134

333 298 30

333 299 106

333 300 77

333 302 94

333 306 69

333 318 107

333 320 31

333 324 32

333 329 49

333 333 309

333 349 126

333 377 46

333 386 36

333 455 45

333 459 52

333 466 53

333 481 31

333 523 2351

333 679 55

333 701 85

333 907 30

333 1475 32

333 1580 295

333 2523 99

333 3332 149

333 3796 73

333 4350 126

333 4463 42

333 5771 193

333 5772 151

333 5773 57

333 5774 44

333 5775 42

333 5776 34

333 5777 33

333 5778 32

333 5779 31

333 5780 28

333 5781 28

334 88 451

334 166 268

334 190 1544

334 198 2279

334 203 1278

334 207 6138

334 220 1839

334 225 2654

334 226 5103

334 227 4351

334 234 1692

334 377 366

334 383 402

334 424 1274

334 507 415

334 716 820

334 854 543

334 857 401

334 868 1303

334 932 534

334 957 862

334 982 1683

334 1048 1469

334 1090 711

334 1206 408

334 1216 2865

334 1239 265

334 1369 317

334 1406 2175

334 1512 712

334 1513 1374

334 1520 8272

334 1545 2319

334 1672 776

334 1866 275

334 1974 764

334 1977 638

334 2364 2391

334 2524 329

334 2746 1710

334 3088 375

334 3467 656

334 4115 1079

334 4218 687

334 4459 2355

334 5639 563

334 5782 1686

334 5783 1160

334 5784 982

334 5785 514

335 55 490

335 65 5861

335 67 1823

335 89 2152

335 166 504

335 184 1288

335 204 11567

335 251 459

335 257 1645

335 261 461

335 270 1181

335 283 826

335 288 423

335 289 1355

335 292 4816

335 295 2335

335 300 423

335 316 578

335 323 1109

335 340 427

335 349 496

335 378 528

335 456 1854

335 458 708

335 481 622

335 486 474

335 518 1778

335 575 512

335 646 853

335 674 1127

335 679 1518

335 689 2842

335 704 1207

335 913 450

335 955 677

335 1662 548

335 1688 1281

335 1934 3249

335 2059 1699

335 2102 2420

335 2419 869

335 2786 2393

335 3324 2741

335 3874 1149

335 5464 4654

335 5786 2231

335 5787 1092

335 5788 830

335 5789 785

335 5790 510

336 64 359

336 70 465

336 162 5599

336 173 2479

336 190 1264

336 198 903

336 238 691

336 377 603

336 486 1048

336 512 686

336 517 1107

336 703 327

336 748 835

336 755 1618

336 757 357

336 774 1053

336 845 422

336 968 385

336 1044 443

336 1099 377

336 1632 332

336 1700 1683

336 1709 460

336 1787 1692

336 2193 371

336 2446 910

336 2681 1160

336 2771 1702

336 2831 643

336 2835 319

336 3411 564

336 5071 408

336 5791 3163

336 5792 1524

336 5793 1190

336 5794 822

336 5795 616

336 5796 590

336 5797 565

336 5798 485

336 5799 480

336 5800 419

336 5801 413

336 5802 388

336 5803 386

336 5804 385

336 5805 360

336 5806 327

336 5807 322

336 5808 310

337 6 336

337 841 106

337 917 466

337 954 424

337 956 137

337 1358 57

337 1360 73

337 1364 37

337 1369	58
337 1513	34
337 1524	1522
337 1528	33
337 1530	28
337 1531	38
337 1535	40
337 1913	75
337 2040	36
337 2055	144
337 2341	91
337 2838	436
337 3470	29
337 3636	32
337 4283	114
337 4791	31
337 4794	34
337 5157	39
337 5689	35
337 5809	251
337 5810	184
337 5811	138
337 5812	134
337 5813	92
337 5814	83
337 5815	69
337 5816	65
337 5817	64
337 5818	58
337 5819	54

337 5820 53

337 5821 48

337 5822 42

337 5823 31

337 5824 30

337 5825 28

337 5826 28

337 5827 27

337 5828 27

337 5829 27

337 5830 27

337 5831 26

338 51 2002

338 67 390

338 88 177

338 89 299

338 98 1399

338 157 2354

338 163 127

338 190 166

338 203 127

338 285 297

338 288 437

338 289 2407

338 378 380

338 400 293

338 429 169

338 455 555

338 599 150

338 732 979

338 757 183	
338 917 147	
338 1015	230
338 1098	297
338 1366	131
338 1456	120
338 1803	271
338 1808	107
338 2343	110
338 2787	118
338 2792	268
338 2923	114
338 2973	4868
338 3201	128
338 3427	159
338 3471	118
338 3474	112
338 4180	928
338 5003	170
338 5160	503
338 5832	812
338 5833	385
338 5834	318
338 5835	267
338 5836	220
338 5837	218
338 5838	202
338 5839	188
338 5840	168
338 5841	108

338 5842 108

338 5843 106

339 55 2463

339 67 1205

339 89 10170

339 161 350

339 257 1592

339 288 3591

339 289 13735

339 290 1718

339 291 1208

339 292 4459

339 293 304

339 294 385

339 295 3354

339 298 414

339 299 506

339 300 1811

339 302 1292

339 306 975

339 308 349

339 317 283

339 318 1094

339 320 276

339 325 1563

339 328 529

339 329 289

339 332 401

339 333 1146

339 349 2101

339 378 829

339 386 288

339 461 2019

339 466 558

339 498 294

339 523 964

339 531 318

339 538 272

339 542 343

339 543 536

339 544 1103

339 679 784

339 686 468

339 701 5376

339 704 412

339 903 251

339 915 259

339 1451 362

339 1934 538

339 2020 265

339 2548 382

339 4716 325

340 55 248

340 67 684

340 89 1284

340 227 268

340 234 614

340 250 2016

340 257 2570

340 286 243

340 288 631

340 289 468

340 292 536

340 295 578

340 299 204

340 300 1266

340 302 211

340 306 308

340 320 305

340 328 195

340 333 378

340 344 267

340 425 242

340 458 3288

340 461 439

340 466 420

340 518 251

340 525 297

340 534 216

340 614 350

340 689 383

340 701 588

340 868 490

340 903 882

340 907 259

340 1412 496

340 1503 250

340 1580 292

340 2902 216

340 3128 281

340 3464	214
340 3795	192
340 3796	239
340 4344	285
340 5474	197
340 5844	521
340 5845	435
340 5846	346
340 5847	276
340 5848	270
340 5849	268
340 5850	184
341 749 62	
341 977 97	
341 1222	91
341 1943	143
341 1946	178
341 1948	170
341 2030	131
341 2593	63
341 2811	65
341 3529	147
341 3703	230
341 4271	90
341 5851	218
341 5852	202
341 5853	168
341 5854	164
341 5855	148
341 5856	142

341 5857	139
341 5858	137
341 5859	120
341 5860	118
341 5861	117
341 5862	112
341 5863	112
341 5864	109
341 5865	104
341 5866	102
341 5867	96
341 5868	92
341 5869	92
341 5870	87
341 5871	85
341 5872	82
341 5873	82
341 5874	81
341 5875	80
341 5876	79
341 5877	78
341 5878	78
341 5879	76
341 5880	74
341 5881	73
341 5882	73
341 5883	68
341 5884	67
341 5885	67
341 5886	65

341 5887 63

341 5888 62

342 64 102

342 70 138

342 72 147

342 81 150

342 88 91

342 89 100

342 154 232

342 155 112

342 157 187

342 159 114

342 163 508

342 173 423

342 190 272

342 207 156

342 220 132

342 221 81

342 227 131

342 425 80

342 432 173

342 546 99

342 599 5450

342 706 78

342 716 74

342 757 156

342 851 157

342 859 94

342 903 264

342 910 64

342 1048 103

342 1075 263

342 1079 67

342 1090 445

342 1155 101

342 1244 197

342 1375 83

342 1398 74

342 1787 100

342 1986 105

342 2821 480

342 2855 178

342 3303 74

342 3501 116

342 3767 252

342 5844 153

342 5889 202

342 5890 117

342 5891 105

342 5892 77

342 5893 75

342 5894 65

343 227 60

343 234 140

343 706 256

343 707 403

343 724 244

343 732 203

343 808 160

343 843 63

343 857 69

343 917 1073

343 918 834

343 922 312

343 956 57

343 982 639

343 1110 451

343 1249 69

343 1343 125

343 1358 473

343 1360 395

343 1369 541

343 1372 638

343 1412 290

343 1426 270

343 1639 151

343 1810 63

343 1873 98

343 1974 287

343 2342 325

343 2343 95

343 2346 511

343 2347 113

343 2787 214

343 2853 587

343 3470 386

343 3475 305

343 4124 247

343 4458 77

343 4680 61

343 5157 142

343 5172 265

343 5365 64

343 5697 118

343 5895 299

343 5896 272

343 5897 109

343 5898 94

343 5899 75

343 5900 74

343 5901 65

343 5902 62

344 156 5

344 423 39

344 436 5

344 861 2

344 961 3

344 1013 1

344 1083 1

344 1098 3

344 1151 14

344 1167 1

344 1375 1

344 1376 2

344 1837 1

344 3201 1

344 4067 1

344 4366 12

344 5903 13

344 5904 11

344 5905 7

344 5906 6

344 5907 6

344 5908 6

344 5909 4

344 5910 3

344 5911 2

344 5912 2

344 5913 2

344 5914 2

344 5915 2

344 5916 2

344 5917 1

344 5918 1

344 5919 1

344 5920 1

344 5921 1

345 56 29

345 67 24

345 71 33

345 154 52

345 173 29

345 190 37

345 207 42

345 295 27

345 298 41

345 425 25

345 430 51

345 526 32

345 546 74

345 554 41

345 585 26

345 697 40

345 703 49

345 709 40

345 757 47

345 969 44

345 1048 227

345 1376 31

345 1398 27

345 1400 49

345 1403 29

345 1410 42

345 1512 26

345 1609 26

345 1632 30

345 2137 36

345 2158 78

345 2524 32

345 2582 27

345 2616 36

345 2721 26

345 2816 27

345 4228 100

345 4245 52

345 5922 129

345 5923 70

345 5924 64

345 5925 59

345 5926 56

345 5927 50

345 5928 49

345 5929 47

345 5930 37

345 5931 25

345 5932 25

345 5933 24

346 7 586

346 55 518

346 59 932

346 67 468

346 89 2173

346 97 1527

346 173 847

346 190 1217

346 207 11632

346 209 615

346 210 603

346 228 2568

346 229 3075

346 230 1008

346 234 644

346 289 414

346 300 866

346 412 1059

346 424 1342

346 425 1315

346 453 400

346 466 458

346 504 382

346 511 713

346 512 365

346 539 798

346 542 2497

346 704 687

346 709 389

346 859 1007

346 903 2893

346 982 544

346 1048 725

346 1090 524

346 1155 545

346 1160 345

346 1244 1868

346 1406 709

346 1415 388

346 1520 385

346 1648 795

346 1988 485

346 2428 1742

346 2524 390

346 2600 673

346 2941 523

346 3198 440

346 3200 920

346 4384 1483

346 5934 1320

347 65 56

347 230 270

347 371 224

347 377 1545

347 383 63

347 403 57

347 408 64

347 412 136

347 475 84

347 481 79

347 486 65

347 488 317

347 517 1102

347 726 212

347 834 244

347 966 808

347 978 2093

347 1044 80

347 1104 686

347 1145 91

347 1191 587

347 1200 76

347 1377 242

347 1410 1194

347 1455 64

347 1614 84

347 1645 174

347 1852 257

347 1854 1186

347 1867 315

347 1870 3197

347 1905 642

347 2843 408

347 2941 258

347 2956 1267

347 3928 95

347 4779 56

347 4821 942

347 5935 1811

347 5936 554

347 5937 375

347 5938 288

347 5939 287

347 5940 147

347 5941 106

347 5942 98

347 5943 91

347 5944 75

347 5945 64

347 5946 60

348 89 55

348 288 28

348 293 25

348 298 32

348 302 35

348 306 26

348 309 26

348 320 41

348 321 52

348 327 30

348 344 24

348 349 29

348 400 32

348 456 27

348 458 42

348 461 68

348 466 33

348 471 60

348 498 44

348 525 42

348 681 55

348 691 50

348 692 28

348 695 22

348 701 31

348 908 90

348 1040 45

348 1043 55

348 1198 30

348 1392 55

348 1458 33

348 1684 62

348 2083 21

348 2923 28

348 3200 59

348 3338 53

348 5036 22

348 5947 62

348 5948 45

348 5949 44

348 5950 44

348 5951 43

348 5952 40

348 5953 40

348 5954 29

348 5955 28

348 5956 27

348 5957 27

348 5958 26

348 5959 22

349 107 2220

349 210 404

349 230 652

349 425 349

349 440 529

349 757 5845

349 825 747

349 1035 4655

349 1188 3782

349 1204 414

349 1228 329

349 1390 407

349 1400 349

349 1550 366

349 1557 609

349 1745 322

349 1853 509

349 1983 379

349 2200 4539

349 2210 817

349 2214 422

349 2426 377

349 2429 619

349	2582	527
349	2586	387
349	2587	385
349	2593	403
349	3200	327
349	3362	425
349	3548	336
349	4096	469
349	4101	771
349	4387	365
349	4508	464
349	4550	323
349	5067	3947
349	5126	309
349	5960	5868
349	5961	4702
349	5962	4477
349	5963	632
349	5964	488
349	5965	410
349	5966	370
349	5967	367
349	5968	357
349	5969	356
349	5970	354
349	5971	329
349	5972	309
350	55	10439
350	67	12181
350	89	1094

350 161 550

350 257 1700

350 265 709

350 288 1269

350 289 2324

350 291 1974

350 292 1904

350 293 599

350 295 853

350 296 690

350 300 821

350 301 1272

350 306 863

350 310 1315

350 321 586

350 323 930

350 325 545

350 333 1756

350 334 1489

350 340 617

350 349 731

350 350 1459

350 352 2103

350 455 574

350 461 638

350 465 3418

350 466 695

350 544 675

350 646 980

350 701 1218

350 790 618

350 792 896

350 802 791

350 889 4329

350 906 2096

350 908 549

350 1453 1134

350 1505 948

350 2022 1157

350 2024 543

350 2523 1118

350 2548 3629

350 3176 1113

350 3332 964

350 4350 2411

350 5973 1366

350 5974 755

351 97 84

351 159 71

351 173 634

351 209 85

351 230 72

351 234 46

351 294 238

351 297 54

351 298 78

351 300 188

351 324 92

351 325 89

351 333 71

351 344 456

351 378 68

351 498 78

351 499 113

351 538 51

351 544 60

351 679 395

351 681 168

351 693 455

351 704 73

351 1032	221

351 1034	5897

351 1038	44

351 1045	206

351 1047	132

351 1098	404

351 1183	187

351 1390	173

351 1690	76

351 2121	150

351 2607	77

351 2619	197

351 2981	151

351 3319	128

351 3404	88

351 4491	94

351 4582	59

351 4773	85

351 4814	365

351 5462	110

351 5529 368

351 5958 57

351 5975 368

351 5976 114

351 5977 102

351 5978 56

351 5979 50

352 58 53

352 67 265

352 89 2840

352 229 97

352 288 302

352 289 541

352 292 515

352 293 166

352 295 144

352 300 409

352 302 295

352 306 68

352 328 51

352 333 111

352 344 47

352 436 50

352 466 53

352 471 75

352 498 73

352 524 69

352 532 63

352 536 64

352 538 68

352 543 103

352 544 65

352 679 43

352 680 354

352 681 40

352 689 45

352 701 85

352 704 930

352 909 60

352 966 121

352 982 112

352 1032 65

352 1121 83

352 1130 46

352 1186 244

352 1459 48

352 2020 112

352 2175 75

352 2595 39

352 2659 41

352 3201 73

352 5980 479

352 5981 125

352 5982 84

352 5983 81

352 5984 61

352 5985 37

353 89 61

353 257 3

353 258 7

353 285 3

353 288 43

353 289 49

353 292 4

353 295 490

353 298 4

353 300 16

353 306 30

353 314 8

353 325 11

353 327 11

353 331 10

353 332 2

353 333 24

353 349 4

353 378 4

353 461 35

353 466 59

353 468 36

353 481 2

353 484 58

353 498 30

353 528 4

353 542 8

353 547 3

353 681 41

353 686 3

353 701 25

353 913 193

353 1037 22

353 1042 13

353 1452 11

353 1453 7

353 1613 2

353 1935 3

353 1936 3

353 2939 9

353 3318 4

353 4576 3

353 5534 3

353 5986 18

353 5987 9

353 5988 6

353 5989 5

353 5990 4

353 5991 3

353 5992 3

354 89 11

354 190 20

354 257 8

354 288 13

354 289 281

354 292 19

354 295 33

354 299 35

354 300 5

354 301 11

354 312 10

354 314 3

354 317 3

354 318 5

354 325 12

354 333 58

354 346 4

354 349 16

354 377 57

354 378 8

354 386 10

354 461 6

354 465 4

354 466 11

354 475 4

354 525 3

354 528 17

354 644 16

354 652 6

354 655 7

354 686 5

354 701 46

354 1613 6

354 2507 6

354 2646 10

354 4561 12

354 5249 4

354 5993 19

354 5994 11

354 5995 8

354 5996 6

354 5997 6

354 5998 6

354 5999 5

354 6000 5

354 6001 4

354 6002 4

354 6003 4

354 6004 3

354 6005 3

355 65 201

355 89 334

355 173 207

355 190 147

355 191 637

355 228 294

355 288 861

355 289 335

355 292 144

355 300 336

355 307 182

355 326 151

355 328 135

355 344 180

355 371 146

355 377 252

355 403 168

355 408 147

355 461 346

355 464 149

355 466 308

355 486 286

355 496 382

355 503 168

355 537 211

355 544 161

355 548 301

355 679 318

355 680 204

355 689 517

355 704 298

355 907 275

355 966 334

355 1042 182

355 1375 174

355 1377 952

355 1378 157

355 1379 176

355 1470 232

355 1518 173

355 1852 244

355 1854 370

355 1905 202

355 2020 180

355 2607 196

355 2956 160

355 5002 192

355 6006 301

355 6007 169

355 6008 139

356 464 2470

356 703 1688

356 768 4220

356 779 3019

356 813 2755

356 832 2119

356 1181 1516

356 1188 1440

356 1191 1103

356 1194 877

356 1196 2496

356 1541 1954

356 1559 858

356 1678 1097

356 1853 2680

356 2192 1810

356 2200 849

356 2210 1970

356 2426 1145

356 2621 1203

356 4003 972

356 4898 2240

356 5126 1559

356 5960 2780

356 5962 1330

356 5963 994

356 5964 1425

356 5967 847

356 5969 2758

356 6009 2587

356 6010 2558

356 6011 2343

356 6012 1889

356 6013 1876

356 6014 1550

356 6015 1513

356 6016 1475

356 6017 1374

356 6018 1172

356 6019 1123

356 6020 1094

356 6021 1019

356 6022 1012

356 6023 945

356 6024 900

356 6025 893

356 6026 889

356 6027 859

356 6028 824

356 6029 805

357 55 2188

357 59 5777

357 72 2078

357 84 562

357 99 1584

357 154 2242

357 159 2002

357 206 501

357 229 2428

357 237 1589

357 295 1600

357 420 1576

357 423 810

357 429 1120

357 441 10625

357 599 869

357 603 720

357 703 2953

357 969 630

357 985 512

357 1106 1911

357 1151 1334

357 1155 1074

357 1376 3641

357 1400 1361

357 1745 1553

357 1883 802

357 1965 577

357 1983 2693

357 1990 1633

357 2602 993

357 3053 1741

357 3502 638

357 3686 698

357 3827 588

357 4245 792

357 4735 741

357 5547 864

357 6030 2161

357 6031 2135

357 6032 1474

357 6033 1148

357 6034 835

357 6035 791

357 6036 772

357 6037 736

357 6038 732

357 6039 669

357 6040 554

357 6041 487

358 53 140

358 56 245

358 70 118

358 88 264

358 162 115

358 196 137

358 207 259

358 238 92

358 418 295

358 475 76

358 603 113

358 617 83

358 621 117

358 709 84

358 969 78

358 1048 88

358 1106 99

358 1109 222

358 1396 134

358 1398 223

358 1400 213

358 1408 83

358 1418 106

358 1512 76

358 1705 102

358 1790 109

358 1835 106

358 2405 164

358 2582 116

358 2605 127

358 2711 98

358 3707 88

358 4039 165

358 4108 105

358 4230 88

358 5927 87

358 6042 261

358 6043 202

358 6044 192

358 6045 153

358 6046 135

358 6047 113

358 6048 100

358 6049 86

358 6050 79

358 6051 79

358 6052 78

358 6053 78

358 6054 75

358 6055 74

359 51 1317

359 59 267

359 61 326

359 68 357

359 72 929

359 75 826

359 93 7636

359 98 287

359 187 421

359 877 713

359 985 401

359 993 576

359 998 3799

359 1001 374

359 1013 292

359 1073 643

359 1464 560

359 1882 1824

359 1883 1246

359 1886 3119

359 1893 1046

359 1897 331

359 2936 431

359 2937 362

359 2938 807

359 4126 282

359 4920 367

359 4975 289

359 5159 409

359 5256 1084

359 6056 2348

359 6057 1906

359 6058 1087

359 6059	691
359 6060	650
359 6061	633
359 6062	473
359 6063	464
359 6064	448
359 6065	395
359 6066	375
359 6067	375
359 6068	361
359 6069	346
359 6070	344
359 6071	319
359 6072	300
359 6073	243
359 6074	243
359 6075	238
360 910 25	
360 1705	45
360 2060	228
360 2500	30
360 3956	50
360 4437	46
360 4928	87
360 6076	744
360 6077	142
360 6078	127
360 6079	92
360 6080	84
360 6081	59

360 6082	50
360 6083	42
360 6084	42
360 6085	40
360 6086	39
360 6087	39
360 6088	37
360 6089	35
360 6090	33
360 6091	32
360 6092	28
360 6093	27
360 6094	25
360 6095	25
360 6096	24
360 6097	23
360 6098	22
360 6099	22
360 6100	21
360 6101	17
360 6102	17
360 6103	16
360 6104	16
360 6105	16
360 6106	16
360 6107	15
360 6108	15
360 6109	14
360 6110	14
360 6111	14

360 6112 13

360 6113 13

360 6114 13

360 6115 13

360 6116 12

360 6117 12

360 6118 12

361 55 214

361 67 676

361 89 236

361 257 4772

361 279 210

361 283 653

361 285 335

361 288 823

361 289 2990

361 290 439

361 291 501

361 292 919

361 293 102

361 294 1138

361 295 811

361 299 392

361 302 197

361 304 274

361 305 432

361 308 170

361 309 182

361 310 399

361 318 166

361 320 135

361 322 323

361 333 254

361 340 186

361 342 102

361 344 937

361 349 841

361 360 116

361 461 468

361 511 97

361 523 165

361 525 199

361 538 261

361 542 148

361 641 1316

361 646 296

361 701 376

361 790 624

361 913 332

361 1243 131

361 1451 223

361 1459 333

361 1772 318

361 3173 146

361 4716 233

361 4883 131

361 6119 915

362 65 694

362 190 479

362 198 188

362 227 1818

362 228 283

362 229 182

362 230 943

362 306 227

362 324 847

362 412 422

362 421 576

362 439 1541

362 464 259

362 481 167

362 486 278

362 498 505

362 500 213

362 540 7608

362 679 651

362 687 264

362 693 244

362 714 232

362 959 198

362 1047 221

362 1090 403

362 1133 367

362 1199 200

362 1241 561

362 1246 517

362 1249 197

362 1406 263

362 1513 388

362 1545 183

362 1681 396

362 2080 373

362 2983 689

362 2986 216

362 3293 440

362 3378 272

362 3382 350

362 4087 842

362 4823 1093

362 4828 407

362 4834 334

362 4846 183

362 6120 1492

362 6121 553

362 6122 335

362 6123 291

362 6124 259

363 182 814

363 190 769

363 230 2067

363 234 633

363 488 784

363 494 879

363 497 1037

363 498 586

363 614 462

363 683 652

363 707 2992

363 805 794

363 808 959

363 810 508

363 830 1131

363 845 550

363 854 464

363 918 1836

363 920 620

363 982 690

363 1041 1524

363 1045 1793

363 1046 787

363 1179 736

363 1186 2474

363 1520 804

363 1546 514

363 1547 460

363 1646 453

363 1672 1324

363 1681 705

363 1858 3886

363 2206 739

363 2738 564

363 2956 697

363 3074 471

363 4189 539

363 5216 999

363 5784 450

363 6125 1053

363 6126 817

363 6127 780

363 6128 756

363 6129 655

363 6130 618

363 6131 598

363 6132 579

363 6133 571

363 6134 562

363 6135 536

364 154 2350

364 159 642

364 162 744

364 163 523

364 190 505

364 211 582

364 212 1845

364 227 1463

364 235 1152

364 418 1667

364 433 1128

364 438 751

364 554 803

364 615 458

364 618 1117

364 624 729

364 630 1396

364 715 3375

364 718 2767

364 737 459

364 1164 1731

364 1639 460

364 1709 911

364 1755	664
364 1779	515
364 1901	583
364 2265	604
364 2415	515
364 2460	518
364 3203	477
364 3347	1103
364 3693	556
364 3747	639
364 4532	997
364 4742	2523
364 4799	566
364 5416	594
364 6136	1133
364 6137	976
364 6138	973
364 6139	896
364 6140	881
364 6141	728
364 6142	655
364 6143	608
364 6144	593
364 6145	516
364 6146	494
364 6147	494
364 6148	467
365 93 9	
365 132 8	
365 141 6	

365 1633	7
365 1744	17
365 3216	5
365 3230	5
365 3504	9
365 3510	9
365 3511	7
365 3703	27
365 4123	30
365 4232	6
365 4248	23
365 5295	8
365 5358	5
365 6048	19
365 6149	70
365 6150	34
365 6151	31
365 6152	26
365 6153	21
365 6154	21
365 6155	17
365 6156	16
365 6157	16
365 6158	15
365 6159	15
365 6160	12
365 6161	10
365 6162	10
365 6163	9
365 6164	9

365 6165 9

365 6166 9

365 6167 9

365 6168 8

365 6169 8

365 6170 8

365 6171 8

365 6172 7

365 6173 7

365 6174 7

365 6175 7

365 6176 6

365 6177 6

365 6178 6

365 6179 5

365 6180 5

365 6181 5

366 53 1399

366 81 254

366 154 4853

366 163 3178

366 212 964

366 222 3569

366 227 708

366 234 374

366 418 1321

366 429 248

366 603 541

366 609 288

366 615 349

366 616 1333

366 715 429

366 748 5130

366 859 480

366 863 1128

366 1099 322

366 1121 287

366 1122 238

366 1404 295

366 1411 371

366 1412 305

366 1418 502

366 1709 493

366 1964 389

366 2281 869

366 2593 400

366 2676 385

366 2716 1308

366 3259 606

366 3294 230

366 3694 246

366 3700 262

366 4922 431

366 5549 380

366 5890 799

366 5925 562

366 6048 300

366 6108 347

366 6182 1770

366 6183 1025

366 6184 780

366 6185 396

366 6186 353

366 6187 311

366 6188 299

366 6189 287

366 6190 235

367 45 1087

367 159 552

367 173 2191

367 207 5086

367 208 486

367 209 13525

367 324 416

367 412 12209

367 428 1749

367 464 152

367 488 356

367 489 268

367 498 833

367 501 594

367 536 2169

367 540 1953

367 704 206

367 813 286

367 814 466

367 816 171

367 903 173

367 982 227

367 1048 2629

367 1180 260

367 1181 1094

367 1193 448

367 1206 438

367 1246 2385

367 1788 311

367 1988 198

367 2131 428

367 2343 388

367 3053 476

367 3078 146

367 3082 184

367 3341 1741

367 3373 251

367 3398 1068

367 4189 450

367 6191 1489

367 6192 1068

367 6193 598

367 6194 515

367 6195 395

367 6196 364

367 6197 252

367 6198 230

367 6199 207

367 6200 202

367 6201 198

368 170 1514

368 197 464

368 416 545

368 418 352

368 423 600

368 454 349

368 858 224

368 863 274

368 867 249

368 1075 295

368 1089 700

368 1101 225

368 1633 529

368 1707 651

368 1747 1254

368 1833 278

368 1837 3022

368 2139 1381

368 2538 246

368 2581 458

368 3280 598

368 3411 253

368 3690 426

368 3884 430

368 3890 227

368 4243 549

368 4384 592

368 5150 1283

368 5414 843

368 5890 228

368 6202 3658

368 6203 2877

368 6204 2042

368 6205 1510

368 6206 1095

368 6207 771

368 6208 508

368 6209 493

368 6210 447

368 6211 423

368 6212 330

368 6213 299

368 6214 298

368 6215 283

368 6216 275

368 6217 273

368 6218 235

368 6219 235

368 6220 231

368 6221 217

369 89 411

369 159 668

369 173 614

369 195 831

369 199 594

369 207 200

369 210 1457

369 217 1524

369 225 1439

369 229 857

369 424 1331

369 426 1107

369 429 965

369 450 296

369 540 171

369 615 329

369 618 797

369 689 169

369 961 523

369 1048 339

369 1090 227

369 1097 421

369 1231 272

369 1375 475

369 1960 179

369 2139 318

369 2407 189

369 2531 393

369 2580 369

369 2600 289

369 2601 489

369 2815 495

369 3057 287

369 3258 209

369 3692 519

369 4118 826

369 4262 262

369 4385 246

369 4810 282

369 5439 206

369 5546 204

369 6222 566

369 6223 285

369 6224 259

369 6225 227

369 6226 217

369 6227 202

369 6228 191

369 6229 185

369 6230 181

370 58 740

370 65 691

370 77 1357

370 89 734

370 154 1050

370 167 1338

370 171 662

370 172 731

370 173 1547

370 192 1161

370 195 976

370 215 681

370 221 1007

370 226 678

370 328 740

370 359 778

370 360 796

370 425 1054

370 486 750

370 504 758

370 511 743

370 533 884

370 538 876

370	599	1015
370	615	845
370	856	685
370	898	850
370	1062	777
370	1094	2076
370	1097	882
370	1116	770
370	1243	712
370	1244	1019
370	1375	883
370	1383	878
370	1464	924
370	1854	905
370	2524	1430
370	2600	847
370	2614	817
370	3411	981
370	3466	1035
370	3948	1305
370	4103	960
370	4257	1024
370	4620	675
370	5212	665
370	6231	977
370	6232	940
370	6233	871
371	39	647
371	72	5997
371	75	207

371 153 1244

371 159 94

371 250 165

371 504 543

371 758 118

371 759 960

371 855 96

371 959 139

371 992 88

371 1072 87

371 1104 253

371 1249 253

371 1366 186

371 1707 409

371 1713 343

371 1788 312

371 1796 89

371 1913 131

371 2154 532

371 2383 1550

371 2506 141

371 2785 177

371 2787 386

371 3049 276

371 3053 214

371 3827 116

371 3833 100

371 3837 150

371 3916 119

371 3988 92

371 3992 88
371 4173 94
371 4239 151
371 5254 289
371 6234 776
371 6235 470
371 6236 406
371 6237 193
371 6238 144
371 6239 141
371 6240 121
371 6241 111
371 6242 106
371 6243 101
371 6244 95
371 6245 94
371 6246 93
372 7 95
372 89 101
372 175 143
372 222 90
372 245 117
372 377 236
372 403 204
372 407 196
372 487 119
372 488 70
372 490 674
372 493 206
372 498 89

372 503 112

372 517 71

372 519 230

372 538 105

372 727 67

372 966 668

372 978 551

372 1062 55

372 1104 63

372 1378 111

372 1854 587

372 1873 57

372 1874 98

372 1931 157

372 2014 1030

372 2096 64

372 2661 85

372 2794 1220

372 2843 55

372 2851 366

372 2956 171

372 3197 181

372 3563 1040

372 3758 239

372 3946 75

372 4136 71

372 4342 84

372 5217 130

372 6247 354

372 6248 159

372 6249 126
372 6250 100
372 6251 92
372 6252 76
372 6253 71
372 6254 66
372 6255 58
374 51 71
374 85 1
374 2562 10
374 6261 1
375 58 33
375 67 77
375 71 92
375 89 90
375 97 48
375 167 53
375 229 136
375 288 144
375 289 364
375 291 175
375 292 371
375 295 243
375 300 199
375 302 74
375 306 32
375 316 44
375 318 32
375 325 64
375 329 38

375 340 109

375 344 95

375 352 77

375 528 32

375 534 59

375 679 113

375 686 64

375 696 149

375 704 171

375 892 114

375 913 35

375 1037 32

375 1379 60

375 1447 101

375 1459 76

375 1556 62

375 1934 259

375 2020 33

375 2083 33

375 2089 67

375 2092 40

375 2176 50

375 2454 54

375 2521 70

375 2607 45

375 2938 43

375 4037 91

375 4707 43

375 5460 67

375 5534 40

375 6262 36

376 163 8197

376 201 118

376 234 208

376 707 4547

376 841 1854

376 917 29001

376 922 1812

376 959 163

376 1257 74

376 1261 23

376 1274 404

376 1343 146

376 1344 1820

376 1358 970

376 1369 117

376 1425 2026

376 1522 539

376 1639 25

376 1796 299

376 1913 2536

376 2704 18

376 2744 427

376 3470 570

376 5157 108

376 5365 162

376 5503 31

376 6263 444

376 6264 196

376 6265 167

376 6266 163

376 6267 125

376 6268 124

376 6269 116

376 6270 99

376 6271 96

376 6272 71

376 6273 57

376 6274 26

376 6275 24

376 6276 23

376 6277 22

376 6278 22

376 6279 21

376 6280 19

376 6281 19

376 6282 19

376 6283 19

376 6284 18

376 6285 18

376 6286 17

377 15 949

377 25 3205

377 181 1939

377 188 8181

377 190 4574

377 226 1983

377 228 1712

377 503 5065

377 812 3070

377 821 2692

377 824 1968

377 841 1788

377 917 36052

377 918 947

377 920 1588

377 923 1581

377 936 5170

377 942 8565

377 943 924

377 953 2060

377 957 1907

377 1131 1309

377 1177 1848

377 1338 1641

377 1339 1438

377 1342 871

377 1349 900

377 1350 843

377 1374 1760

377 1412 2289

377 1527 1131

377 1613 865

377 1643 1235

377 1672 752

377 1710 1255

377 1974 1103

377 1976 4989

377 1992 1846

377 2009 872

377 2381 1027

377 2498 830

377 2654 1375

377 3621 1318

377 4387 913

377 4670 991

377 4779 894

377 6287 4241

377 6288 1970

377 6289 825

377 6290 769

378 67 267

378 77 151

378 157 12548

378 159 76

378 163 339

378 176 161

378 180 222

378 187 107

378 220 155

378 227 581

378 230 100

378 234 172

378 251 79

378 253 670

378 285 5143

378 377 73

378 486 124

378 533 157

378 562 247

378 575 166

378 578 152

378 601 360

378 614 157

378 706 813

378 735 312

378 951 3325

378 959 458

378 966 77

378 982 672

378 1042 233

378 1122 188

378 1244 403

378 1249 471

378 1412 114

378 1414 72

378 1415 141

378 1427 491

378 1691 86

378 1825 224

378 2031 79

378 2182 189

378 2346 1151

378 2347 237

378 2558 160

378 3302 195

378 3313 186

378 4180 139

378 4351 169

378 6291 96

378 6292 88

380 67 4161

380 89 3998

380 161 600

380 241 407

380 257 478

380 285 3089

380 288 4913

380 289 32135

380 291 608

380 292 2518

380 294 888

380 295 3229

380 296 1572

380 299 3244

380 308 1335

380 314 798

380 317 744

380 318 2511

380 320 464

380 323 882

380 325 1186

380 326 730

380 332 493

380 349 1755

380 352 532

380 456 1932

380 465 3217

380 466 1930

380 476 780

380 523 686

380 525 1131

380 543 623

380 908 1175

380 913 864

380 915 636

380 1052 2870

380 1596 613

380 1601 1704

380 1602 571

380 1937 580

380 2094 1072

380 2990 1123

380 4883 2107

380 6308 1592

380 6309 601

380 6310 585

380 6311 567

380 6312 485

380 6313 436

380 6314 436

382 52 924

382 276 685

382 331 598

382 605 1864

382 619 962

382 1397 539

382 1610 746

382 1611 704

382 1619 782

382 1621	546
382 1622	3084
382 1839	617
382 1970	1427
382 1972	618
382 1979	611
382 2236	3052
382 2251	550
382 2404	1994
382 2698	1263
382 2923	716
382 3227	791
382 3678	779
382 3776	586
382 5481	1501
382 6052	1156
382 6320	1859
382 6321	1516
382 6322	1459
382 6323	1444
382 6324	1407
382 6325	1119
382 6326	893
382 6327	836
382 6328	796
382 6329	792
382 6330	741
382 6331	723
382 6332	722
382 6333	654

382 6334 632

382 6335 600

382 6336 597

382 6337 592

382 6338 576

382 6339 554

382 6340 543

382 6341 538

382 6342 518

382 6343 518

382 6344 516

383 56 153

383 65 6494

383 88 3512

383 154 1383

383 171 701

383 172 236

383 182 127

383 184 128

383 190 279

383 191 146

383 203 1158

383 221 1120

383 229 1356

383 230 225

383 237 194

383 293 632

383 311 143

383 333 699

383 362 231

383 420 188

383 461 440

383 498 2687

383 499 134

383 538 695

383 546 132

383 680 1860

383 693 701

383 701 230

383 757 148

383 790 290

383 898 215

383 962 178

383 972 126

383 1059 258

383 1090 168

383 1239 156

383 1432 923

383 1433 127

383 1443 151

383 1520 192

383 1632 937

383 1768 156

383 1772 233

383 3187 187

383 3205 551

383 3255 238

383 5147 129

383 5149 389

383 6345 365

383 6346 134

385 56 539

385 65 10368

385 89 7877

385 173 806

385 182 630

385 190 1039

385 191 15465

385 217 513

385 228 1462

385 230 780

385 288 683

385 289 21902

385 291 6678

385 292 1432

385 300 3301

385 302 656

385 304 796

385 306 7751

385 316 1850

385 318 1180

385 321 1306

385 324 3065

385 325 2203

385 333 1665

385 344 39790

385 377 2342

385 386 653

385 461 52611

385 464 1115

385 466 3544

385 472 7489

385 486 2574

385 498 23970

385 518 641

385 544 636

385 679 922

385 680 2546

385 681 8547

385 686 1056

385 692 838

385 704 1778

385 907 1572

385 966 1061

385 1043 2801

385 1246 740

385 1377 1167

385 1470 2012

385 2071 842

385 2498 3205

385 2607 707

386 56 137

386 65 145

386 163 347

386 176 1338

386 230 776

386 344 124

386 412 111

386 504 170

386 707 926

386 726 204

386 856 648

386 899 158

386 959 332

386 982 323

386 1351 129

386 1406 408

386 1686 614

386 1740 118

386 1873 111

386 2346 200

386 2361 622

386 2838 124

386 4125 162

386 5666 400

386 6347 700

386 6348 645

386 6349 509

386 6350 427

386 6351 427

386 6352 354

386 6353 285

386 6354 276

386 6355 267

386 6356 261

386 6357 256

386 6358 234

386 6359 225

386 6360 208

386 6361 204

386 6362 193

386 6363 186

386 6364 185

386 6365 184

386 6366 173

386 6367 161

386 6368 136

386 6369 133

386 6370 125

386 6371 122

386 6372 117

387 67 10833

387 157 8329

387 173 6922

387 204 2740

387 227 11171

387 241 9943

387 251 3070

387 262 3640

387 266 3637

387 279 31594

387 287 4438

387 288 2985

387 301 3170

387 302 3156

387 316 8954

387 430 5393

387 437 2843

387 548 2876

387 907 10640

387 1155	2666
387 1409	3075
387 1595	5251
387 1613	2871
387 1620	4616
387 2083	6733
387 2220	3887
387 2226	3552
387 2521	12216
387 4732	2582
387 4883	6470
387 5048	13992
387 5186	4063
387 5789	2562
387 6373	30614
387 6374	13597
387 6375	12418
387 6376	10085
387 6377	5500
387 6378	4421
387 6379	3953
387 6380	3635
387 6381	3634
387 6382	3305
387 6383	3292
387 6384	3291
387 6385	3159
387 6386	2921
387 6387	2745
387 6388	2673

387 6389 2548

388 190 1891

388 228 1351

388 289 1662

388 377 8011

388 378 12882

388 407 2366

388 518 2345

388 706 2478

388 718 5867

388 726 4838

388 837 2805

388 910 1851

388 930 13860

388 953 1743

388 955 2679

388 982 1596

388 1104 1740

388 1686 1768

388 1705 8178

388 1710 3586

388 2014 1966

388 2035 4276

388 2446 2485

388 2746 2064

388 2838 4741

388 2944 3253

388 3425 2893

388 3667 2579

388 4105 1425

388 4543 1438

388 4917 2261

388 4920 1456

388 5807 1932

388 6056 9506

388 6077 11597

388 6252 1476

388 6390 5956

388 6391 5895

388 6392 3169

388 6393 1869

388 6394 1847

388 6395 1784

388 6396 1575

388 6397 1543

388 6398 1469

388 6399 1465

388 6400 1407

388 6401 1386

388 6402 1369

388 6403 1358

390 55 20118

390 67 4860

390 89 19376

390 257 4281

390 288 6750

390 289 7482

390 292 4172

390 294 491

390 295 1084

390 296 1002

390 299 1534

390 300 7014

390 302 448

390 306 1135

390 308 469

390 314 644

390 317 715

390 328 680

390 331 520

390 332 627

390 333 1044

390 340 395

390 386 532

390 400 1240

390 455 836

390 461 1242

390 464 440

390 466 4588

390 475 1031

390 523 577

390 543 1547

390 646 1188

390 679 2140

390 701 3331

390 802 700

390 907 887

390 1032 1301

390 1037 2269

390 1051 681

390 1243	1610
390 1444	381
390 1453	582
390 1934	1195
390 2021	1515
390 2523	1673
390 6426	3252
390 6427	1023
390 6428	707
390 6429	560
390 6430	424
391 191 235
391 245 258
391 377 336
391 460 570
391 472 318
391 486 506
391 488 214
391 493 640
391 498 316
391 687 199
391 779 445
391 816 782
391 1043	332
391 1180	913
391 1182	643
391 1186	252
391 1194	285
391 1670	208
391 1852	241

391 1854	218
391 2154	285
391 2435	516
391 2661	457
391 2940	557
391 3833	223
391 4234	1298
391 4237	357
391 4891	246
391 5310	355
391 6431	670
391 6432	539
391 6433	426
391 6434	397
391 6435	377
391 6436	332
391 6437	330
391 6438	298
391 6439	296
391 6440	292
391 6441	282
391 6442	281
391 6443	268
391 6444	254
391 6445	243
391 6446	234
391 6447	223
391 6448	222
391 6449	218
391 6450	218

391 6451 217
392 65 58
392 67 48
392 89 101
392 154 47
392 157 61
392 195 50
392 204 93
392 227 122
392 251 56
392 257 70
392 288 169
392 289 232
392 291 118
392 292 208
392 293 81
392 302 101
392 316 101
392 324 118
392 333 132
392 342 50
392 344 153
392 362 63
392 378 65
392 403 93
392 412 48
392 459 53
392 464 57
392 498 69
392 499 58

392 679 278

392 689 105

392 706 212

392 892 80

392 903 72

392 913 50

392 961 55

392 989 49

392 1081 65

392 1090 67

392 1091 70

392 1459 76

392 1464 73

392 1934 77

392 3070 61

392 6452 123

392 6453 79

392 6454 55

392 6455 48

392 6456 48

392 6457 48

393 56 82

393 65 49

393 190 534

393 203 161

393 206 47

393 207 80

393 209 244

393 227 11164

393 234 52

393 316 84

393 486 78

393 492 149

393 537 65

393 546 46

393 554 65

393 562 157

393 605 170

393 733 514

393 735 122

393 959 152

393 962 373

393 985 55

393 1081 47

393 1090 112

393 1100 257

393 1185 196

393 1197 53

393 1239 91

393 1242 87

393 1378 75

393 1414 97

393 1416 239

393 1464 61

393 2341 49

393 2343 111

393 2347 204

393 3065 73

393 3071 128

393 3470 60

393 3616	156
393 4089	45
393 5969	62
393 6458	260
393 6459	119
393 6460	114
393 6461	61
393 6462	55
393 6463	53
393 6464	50
393 6465	47
394 163 85	
394 245 49	
394 250 307	
394 1011	63
394 1110	51
394 1372	71
394 1412	210
394 1545	118
394 1681	76
394 1763	57
394 1764	54
394 1775	135
394 2346	59
394 2637	51
394 2786	51
394 3378	104
394 3392	116
394 3743	81
394 3746	396

394 4087	88
394 4463	84
394 4466	160
394 4467	76
394 4800	51
394 5119	46
394 5226	76
394 5443	56
394 5594	66
394 6466	240
394 6467	176
394 6468	154
394 6469	150
394 6470	140
394 6471	113
394 6472	105
394 6473	97
394 6474	85
394 6475	83
394 6476	80
394 6477	80
394 6478	72
394 6479	71
394 6480	71
394 6481	70
394 6482	67
394 6483	65
394 6484	64
394 6485	52
394 6486	51

394 6487 49

395 7 142

395 198 106

395 220 112

395 370 141

395 377 119

395 503 330

395 517 510

395 519 132

395 710 97

395 785 114

395 815 210

395 822 127

395 834 114

395 841 1196

395 843 356

395 846 236

395 917 146

395 918 194

395 954 200

395 978 297

395 1044 508

395 1104 1761

395 1131 91

395 1145 244

395 1274 1059

395 1281 88

395 1360 103

395 1521 157

395 1533 288

395 1547 461

395 1870 81

395 1974 304

395 2379 436

395 2380 1339

395 3032 127

395 3050 143

395 3719 251

395 4279 95

395 4500 193

395 4504 129

395 5105 96

395 6488 199

395 6489 178

395 6490 130

395 6491 113

395 6492 92

395 6493 88

395 6494 87

395 6495 83

395 6496 78

396 30 1331

396 56 216

396 67 715

396 70 473

396 72 2542

396 81 1017

396 238 690

396 356 690

396 722 143

396 734 580

396 880 1456

396 1073 965

396 1099 1188

396 1104 681

396 1394 670

396 1397 310

396 1711 1082

396 1793 450

396 1795 171

396 1842 192

396 2039 1134

396 2044 1651

396 2176 241

396 3280 362

396 4037 466

396 5249 2058

396 6497 1835

396 6498 1767

396 6499 1643

396 6500 1181

396 6501 1158

396 6502 1143

396 6503 1079

396 6504 913

396 6505 616

396 6506 489

396 6507 442

396 6508 431

396 6509 429

396 6510 358
396 6511 220
396 6512 218
396 6513 204
396 6514 185
396 6515 182
396 6516 166
396 6517 138
396 6518 129
396 6519 125
396 6520 119
397 2 56
397 7 128
397 9 78
397 30 221
397 51 60
397 68 23
397 72 1044
397 75 84
397 99 48
397 153 22
397 154 42
397 172 314
397 190 53
397 234 25
397 425 22
397 498 41
397 511 23
397 522 63
397 546 31

397 930 41

397 959 233

397 972 33

397 1001	32
397 1014	21
397 1062	56
397 1104	49
397 1249	50
397 1253	39
397 1300	67
397 1713	48
397 2369	27
397 3110	29
397 3184	54
397 3186	31
397 3196	43
397 3289	37
397 3462	36
397 3905	104
397 4137	71
397 4308	41
397 5020	32
397 5237	146
397 5238	236
397 6521	65
397 6522	62
397 6523	49
397 6524	43
397 6525	32
397 6526	32

397 6527 29
398 53 864
398 56 409
398 72 91
398 81 118
398 88 1989
398 154 1080
398 163 189
398 199 157
398 203 2466
398 206 153
398 207 207
398 212 126
398 215 210
398 225 85
398 226 110
398 227 707
398 233 141
398 237 99
398 238 1201
398 439 428
398 441 149
398 453 482
398 512 123
398 533 253
398 599 151
398 603 408
398 709 203
398 863 148
398 903 159

398 982 135
398 1044 83
398 1089 120
398 1095 138
398 1098 523
398 1107 132
398 1239 119
398 1394 109
398 1400 98
398 1414 111
398 1504 227
398 1620 96
398 1761 106
398 1763 99
398 1768 84
398 1806 122
398 1977 132
398 2535 294
398 3156 110
398 4316 140
398 4731 131
399 89 332
399 209 1545
399 257 833
399 288 11180
399 289 1517
399 290 363
399 291 1203
399 292 9452
399 294 2070

399 295 742

399 299 6092

399 300 183

399 301 218

399 302 498

399 310 365

399 311 1052

399 314 187

399 318 330

399 326 177

399 330 180

399 333 440

399 342 159

399 344 16375

399 352 432

399 405 218

399 456 319

399 461 174

399 465 188

399 466 543

399 481 313

399 525 201

399 542 1196

399 686 1022

399 701 726

399 915 1272

399 972 304

399 1037 321

399 1246 321

399 1428 661

399 1458 1781

399 1459 243

399 1580 195

399 1602 297

399 1690 277

399 2080 274

399 2130 610

399 2392 378

399 2990 574

399 3081 208

399 6528 973

400 55 45

400 67 63

400 89 301

400 161 74

400 288 427

400 289 1533

400 291 73

400 292 152

400 293 100

400 295 63

400 299 1004

400 300 596

400 302 80

400 306 386

400 310 62

400 316 62

400 318 615

400 325 61

400 333 669

400 378 47

400 461 267

400 464 120

400 466 12620

400 475 56

400 523 68

400 528 162

400 531 70

400 544 69

400 644 43

400 681 69

400 686 482

400 701 429

400 704 51

400 908 83

400 972 194

400 1032 117

400 1034 125

400 1037 350

400 1409 122

400 1428 182

400 1458 580

400 1690 202

400 2081 68

400 3187 72

400 3397 63

400 4260 128

400 4575 150

400 6527 88

400 6529 89

400 6530 64
401 89 157
401 220 66
401 230 51
401 234 775
401 289 90
401 349 54
401 412 455
401 466 70
401 475 68
401 492 96
401 497 229
401 498 1112
401 500 177
401 536 48
401 537 52
401 706 106
401 719 105
401 854 238
401 932 261
401 989 104
401 1041 83
401 1046 93
401 1116 276
401 1186 235
401 1189 89
401 1406 59
401 1513 557
401 1545 225
401 1681 77

401 1807 956

401 2850 126

401 2959 182

401 2987 127

401 3127 407

401 3333 1010

401 3428 152

401 3735 78

401 3739 75

401 3744 325

401 4828 62

401 5772 71

401 6531 466

401 6532 241

401 6533 238

401 6534 143

401 6535 130

401 6536 96

401 6537 72

401 6538 63

401 6539 51

402 7 1697

402 163 7550

402 212 1723

402 227 5083

402 230 679

402 234 2991

402 511 924

402 562 2409

402 706 5254

402 707 6761

402 724 845

402 732 1745

402 735 1157

402 841 2255

402 843 1181

402 846 1063

402 857 1527

402 868 13333

402 917 2942

402 918 3171

402 920 976

402 949 1735

402 951 944

402 959 10489

402 982 3151

402 1249 5842

402 1274 783

402 1281 794

402 1351 1164

402 1358 933

402 1360 827

402 1364 913

402 1369 2977

402 1372 926

402 1412 6295

402 1513 1728

402 1521 1763

402 1527 913

402 1639 4044

402 1804 829

402 1810 674

402 1814 975

402 2343 2317

402 2346 3164

402 2347 1201

402 2565 789

402 2797 686

402 4917 741

402 6540 743

402 6541 701

403 9 106

403 67 175

403 89 1939

403 209 102

403 288 515

403 289 817

403 291 114

403 292 167

403 300 1191

403 328 109

403 344 896

403 386 855

403 458 1015

403 461 986

403 464 131

403 466 3727

403 487 101

403 497 133

403 498 173

403 504 187

403 524 744

403 547 222

403 680 171

403 686 123

403 704 131

403 1035 249

403 1037 541

403 1066 681

403 1091 1350

403 1376 131

403 1682 198

403 2094 369

403 3319 376

403 3556 130

403 3558 2201

403 4262 217

403 5079 4458

403 5460 205

403 5670 108

403 6542 2405

403 6543 914

403 6544 476

403 6545 196

403 6546 195

403 6547 180

403 6548 168

403 6549 150

403 6550 110

403 6551 108

403 6552 106

404 209 41

404 230 1370

404 333 40

404 377 294

404 383 64

404 412 139

404 428 162

404 481 124

404 536 59

404 707 47

404 808 37

404 982 150

404 1046 53

404 1179 30

404 1181 34

404 1186 171

404 1192 52

404 1348 62

404 1351 96

404 1406 41

404 1672 57

404 1689 1100

404 1863 72

404 2343 212

404 3078 78

404 3373 84

404 4155 67

404 4156 191

404 4375 55

404 4820 30

404 4821 189

404 4822 121

404 4824 37

404 4826 121

404 4833 40

404 5065 31

404 5304 31

404 5310 84

404 5313 55

404 5939 50

404 6453 43

404 6553 990

404 6554 157

404 6555 70

404 6556 56

404 6557 54

404 6558 53

404 6559 45

404 6560 42

404 6561 37

405 55 3759

405 67 4706

405 69 610

405 89 4060

405 257 3043

405 262 1010

405 279 1162

405 285 11477

405 288 903

405 289 712

405 292 1215

405 295 1637

405 296 276

405 299 940

405 300 291

405 310 509

405 312 397

405 314 1547

405 317 710

405 322 1171

405 325 283

405 329 367

405 331 576

405 349 1454

405 350 446

405 466 489

405 478 486

405 485 692

405 525 412

405 527 460

405 543 576

405 638 339

405 913 547

405 1037 601

405 1451 1060

405 1596 313

405 2083 357

405 2220 846

405 4566 1262

405 4713 555

405 4814 908

405 5988 624

405 6311 463

405 6562 1893

405 6563 849

405 6564 357

405 6565 337

405 6566 324

405 6567 292

405 6568 282

406 152 2237

406 163 764

406 186 1007

406 188 1305

406 190 364

406 227 1759

406 370 1163

406 506 1831

406 609 474

406 920 1383

406 949 295

406 964 851

406 1110 728

406 1411 1271

406 1413 249

406 1425 277

406 1426 202

406 1837 366

406 1994 363

406 2006	1191
406 2016	280
406 2151	444
406 2475	503
406 2579	321
406 2624	540
406 2654	324
406 2962	345
406 3264	376
406 3272	582
406 3405	286
406 3421	400
406 3481	333
406 3788	856
406 3940	450
406 4615	277
406 4675	450
406 4787	744
406 4790	250
406 5833	470
406 6569	707
406 6570	569
406 6571	386
406 6572	330
406 6573	325
406 6574	301
406 6575	276
406 6576	237
406 6577	223
406 6578	217

406 6579 205

407 53 46

407 59 20

407 64 33

407 80 36

407 84 25

407 199 14

407 205 16

407 238 21

407 432 44

407 532 15

407 539 19

407 618 30

407 720 26

407 863 19

407 1073 31

407 1155 19

407 1394 18

407 1398 21

407 1405 25

407 1633 25

407 1709 14

407 1826 15

407 1833 14

407 1983 20

407 2043 18

407 2270 38

407 3158 16

407 3354 14

407 3673 56

407 3707 20

407 3993 15

407 4025 26

407 4027 17

407 4028 27

407 5192 17

407 5414 19

407 5925 25

407 6045 26

407 6053 15

407 6409 23

407 6580 31

407 6581 23

407 6582 18

407 6583 17

407 6584 17

407 6585 16

407 6586 15

407 6587 15

407 6588 14

407 6589 14

408 72 902

408 77 136

408 99 800

408 207 244

408 227 674

408 228 169

408 533 946

408 709 295

408 868 193

408 969 803

408 1090 119

408 1379 138

408 1510 216

408 1512 231

408 1976 182

408 1977 133

408 2122 96

408 2539 507

409 67 208

409 89 7682

409 157 119

409 159 35

409 167 169

409 184 422

409 187 11

409 190 10

409 227 18

409 228 98

409 230 47

409 292 747

409 295 805

409 298 69

409 301 10

409 302 94

409 331 34

409 403 25

409 468 86

409 485 47

409 486 171

409 518 41

409 525 126

409 541 118

409 544 188

409 547 27

409 599 849

409 706 25

409 734 251

409 913 30

409 959 99

409 1116 23

409 1249 11

409 1379 280

409 1412 25

409 1506 31

409 1511 103

409 1613 10

409 1810 41

409 2176 410

409 2923 15

409 3171 96

409 3576 26

409 3716 51

409 4543 26

409 5678 1305

409 5984 313

409 6590 31

409 6591 22

409 6592 11

410 333 26

410 403 83

410 464 40

410 471 225

410 501 86

410 687 66

410 691 326

410 1035 138

410 1036 47

410 1038 100

410 1040 232

410 1043 189

410 1183 136

410 1198 26

410 1201 93

410 2607 38

410 2653 75

410 3078 133

410 4264 78

410 4385 185

410 5314 52

410 5322 37

410 5950 205

410 5958 142

410 6552 48

410 6593 222

410 6594 210

410 6595 194

410 6596 179

410 6597 164

410 6598 164

410 6599 125

410 6600 118

410 6601 114

410 6602 76

410 6603 76

410 6604 73

410 6605 71

410 6606 63

410 6607 63

410 6608 50

410 6609 40

410 6610 39

410 6611 38

410 6612 35

410 6613 32

410 6614 30

410 6615 30

410 6616 29

410 6617 28

411 267 116

411 278 79

411 330 157

411 331 82

411 349 56

411 377 114

411 475 12128

411 491 66

411 527 52

411 907 106

411 1444 38

411 1613	176
411 1641	335
411 1860	32
411 1979	64
411 2179	1025
411 2226	771
411 2236	98
411 2241	32
411 2247	326
411 2251	66
411 2252	173
411 2916	186
411 2919	130
411 2924	309
411 3227	61
411 3357	86
411 4585	47
411 6376	112
411 6377	40
411 6618	1934
411 6619	458
411 6620	198
411 6621	164
411 6622	155
411 6623	131
411 6624	113
411 6625	86
411 6626	84
411 6627	72
411 6628	70

411 6629 62

411 6630 55

411 6631 49

411 6632 47

411 6633 45

411 6634 41

411 6635 41

411 6636 39

411 6637 37

412 67 50

412 89 119

412 97 76

412 157 23

412 196 58

412 207 48

412 227 31

412 292 17

412 295 46

412 298 43

412 300 20

412 306 16

412 320 22

412 327 18

412 439 57

412 455 158

412 464 17

412 525 20

412 542 18

412 546 34

412 585 21

412 679 97

412 701 20

412 857 26

412 903 54

412 1047 35

412 1244 102

412 1613 20

412 1691 28

412 1761 23

412 1763 41

412 1764 30

412 1768 27

412 1779 19

412 2176 54

412 2184 15

412 3200 35

412 3310 25

412 3747 31

412 4087 19

412 4708 16

412 4709 20

412 5745 32

412 6638 177

412 6639 73

412 6640 33

412 6641 22

412 6642 21

412 6643 16

412 6644 16

413 55 367

413 65 619

413 67 41216

413 69 293

413 88 192

413 157 112

413 190 203

413 203 295

413 220 685

413 229 365

413 230 1467

413 288 243

413 291 1106

413 292 2688

413 295 5257

413 298 888

413 301 670

413 302 4130

413 304 859

413 306 136

413 310 423

413 311 343

413 316 154

413 329 139

413 331 192

413 344 2472

413 377 152

413 461 124

413 466 456

413 525 483

413 526 336

413 547 137

413 641 276

413 679 423

413 689 115

413 701 4817

413 735 356

413 900 144

413 903 494

413 913 518

413 1447 164

413 1455 151

413 1458 276

413 1613 200

413 1936 144

413 2080 516

413 3460 248

413 3708 147

413 6645 195

413 6646 142

414 55 470

414 67 1541

414 157 781

414 256 185

414 257 3912

414 283 1562

414 288 3519

414 289 61440

414 292 2837

414 293 286

414 295 1785

414 298 1232

414 300 4543

414 302 998

414 306 691

414 313 389

414 314 664

414 325 259

414 333 1998

414 342 391

414 349 804

414 378 214

414 386 348

414 461 1535

414 464 283

414 466 4522

414 498 3877

414 526 265

414 538 573

414 542 1686

414 544 2207

414 546 380

414 646 512

414 679 199

414 686 1214

414 693 297

414 701 1279

414 903 3507

414 915 247

414 966 279

414 1032 522

414 1035 287
414 1045 272
414 1452 285
414 1771 224
414 2082 192
414 2988 193
414 3200 1014
414 5934 565
414 6647 262
415 55 1152
415 67 1473
415 89 6469
415 157 205
415 230 348
415 257 619
415 288 645
415 289 17582
415 291 336
415 292 9678
415 298 2261
415 300 4779
415 302 651
415 306 648
415 316 387
415 318 268
415 320 455
415 324 366
415 329 251
415 333 1258
415 352 3215

415 386 303

415 455 199

415 466 1040

415 498 3254

415 523 455

415 525 646

415 528 1418

415 532 491

415 534 202

415 542 342

415 546 290

415 646 506

415 674 1377

415 679 662

415 680 310

415 681 300

415 686 258

415 689 189

415 701 1079

415 704 1222

415 889 395

415 903 281

415 972 787

415 1034 704

415 1249 250

415 1451 350

415 1580 297

415 2020 3512

415 3404 948

416 162 63

416 603 92	
416 748 98	
416 1205	223
416 1709	71
416 2030	212
416 2043	80
416 2471	296
416 2668	78
416 2681	82
416 2775	78
416 2850	141
416 3417	83
416 3512	83
416 3703	85
416 4271	68
416 4959	291
416 4966	89
416 5718	69
416 5966	168
416 6159	61
416 6648	225
416 6649	191
416 6650	189
416 6651	178
416 6652	177
416 6653	162
416 6654	156
416 6655	117
416 6656	113
416 6657	102

416 6658 100
416 6659 96
416 6660 93
416 6661 93
416 6662 84
416 6663 81
416 6664 73
416 6665 73
416 6666 69
416 6667 69
416 6668 68
416 6669 68
416 6670 67
416 6671 64
416 6672 64
416 6673 63
416 6674 62
416 6675 61
416 6676 61
417 55 284
417 65 200
417 67 361
417 89 1222
417 155 231
417 157 498
417 171 204
417 257 691
417 279 216
417 285 373
417 288 780

417 289 1495

417 291 211

417 292 986

417 294 952

417 295 1358

417 296 269

417 298 564

417 299 627

417 300 290

417 301 469

417 302 744

417 306 461

417 311 287

417 323 248

417 333 281

417 349 352

417 426 227

417 430 358

417 461 346

417 475 408

417 546 198

417 701 206

417 906 390

417 913 190

417 915 212

417 1037 190

417 1051 276

417 1243 187

417 1383 216

417 1601 201

417 1718 224

417 1935 199

417 1936 193

417 2083 192

417 2544 258

417 3057 542

417 3201 413

417 3739 202

417 6677 254

418 56 223

418 89 1851

418 154 3141

418 167 805

418 211 306

418 217 345

418 229 343

418 374 1040

418 426 1629

418 428 1224

418 430 199

418 618 523

418 622 367

418 629 589

418 679 338

418 716 209

418 898 259

418 899 1004

418 903 233

418 1090 280

418 1105 612

418 1239	582
418 1243	314
418 1633	800
418 1944	4056
418 1947	1396
418 1958	738
418 2131	792
418 2398	267
418 2402	519
418 2407	400
418 2632	398
418 2893	448
418 3057	1894
418 3258	812
418 4118	827
418 4199	479
418 5810	348
418 6298	1052
418 6678	1196
418 6679	443
418 6680	425
418 6681	379
418 6682	359
418 6683	331
418 6684	287
418 6685	276
418 6686	260
418 6687	200
418 6688	198
419 63 7	

419 89 33

419 157 12

419 161 12

419 241 16

419 257 6

419 279 7

419 285 7

419 288 23

419 289 11

419 295 39

419 299 14

419 306 10

419 308 71

419 309 9

419 314 8

419 317 10

419 320 8

419 327 8

419 331 6

419 332 10

419 349 10

419 466 26

419 468 22

419 531 23

419 544 16

419 644 70

419 722 18

419 907 7

419 913 14

419 968 6

419 1042 45

419 1395 13

419 1450 8

419 1596 8

419 1937 12

419 2020 7

419 3491 12

419 3794 7

419 4562 11

419 4564 16

419 4566 9

419 4814 47

419 6689 40

419 6690 21

419 6691 17

419 6692 12

419 6693 9

419 6694 8

419 6695 8

420 55 203

420 63 284

420 67 1753

420 97 121

420 161 1828

420 171 349

420 180 850

420 257 382

420 333 186

420 340 443

420 457 437

420 641 114	
420 792 4294	
420 889 34272	
420 1015	110
420 1047	120
420 1464	1629
420 3067	163
420 3920	81
420 4880	12434
420 4981	101
420 6247	390
420 6640	207
420 6696	8511
420 6697	4206
420 6698	2255
420 6699	2017
420 6700	1967
420 6701	1678
420 6702	1442
420 6703	1372
420 6704	1318
420 6705	1196
420 6706	570
420 6707	555
420 6708	379
420 6709	280
420 6710	269
420 6711	247
420 6712	175
420 6713	168

420 6714 165

420 6715 154

420 6716 140

420 6717 113

420 6718 112

420 6719 106

420 6720 106

420 6721 94

420 6722 79

421 55 215

421 67 639

421 71 81

421 89 1048

421 157 221

421 180 85

421 250 64

421 257 145

421 288 2718

421 289 7886

421 291 209

421 292 560

421 293 451

421 295 1235

421 298 96

421 299 655

421 300 204

421 301 163

421 302 75

421 306 702

421 310 459

421 311 138

421 314 371

421 320 387

421 326 133

421 327 464

421 329 341

421 333 1813

421 349 523

421 360 74

421 378 169

421 461 103

421 466 354

421 468 136

421 475 293

421 484 75

421 525 1134

421 542 127

421 543 269

421 701 215

421 903 75

421 907 86

421 908 182

421 913 168

421 1505 69

421 1580 65

421 2179 87

421 2523 226

421 4069 105

421 6723 227

423 51 732

423 67 74

423 68 60

423 72 2338

423 85 350

423 96 62

423 99 86

423 154 63

423 187 2130

423 193 367

423 227 90

423 429 88

423 511 84

423 538 619

423 711 88

423 712 86

423 717 146

423 857 70

423 868 188

423 959 85

423 993 207

423 995 87

423 997 74

423 1001 157

423 1004 95

423 1013 216

423 1090 240

423 1464 194

423 1713 256

423 1891 79

423 2552 121

423 2554 109

423 2570 67

423 2571 86

423 3832 63

423 6732 1827

423 6733 933

423 6734 175

423 6735 174

423 6736 156

423 6737 138

423 6738 117

423 6739 111

423 6740 110

423 6741 109

423 6742 100

423 6743 90

423 6744 79

423 6745 76

423 6746 69

424 15 280

424 25 283

424 63 702

424 72 554

424 77 212

424 181 305

424 183 295

424 198 244

424 230 232

424 233 179

424 487 652

424 489 300

424 517 214

424 707 572

424 785 441

424 815 1107

424 826 210

424 841 321

424 843 225

424 917 410

424 920 1827

424 930 482

424 949 200

424 957 216

424 966 288

424 1104 871

424 1119 391

424 1274 337

424 1338 287

424 1349 388

424 1358 258

424 1368 198

424 1705 435

424 2014 459

424 2369 351

424 2744 359

424 2787 593

424 2794 332

424 2838 3811

424 3940 542

424 4608 219

424 4691 491

424 6747 459

424 6748 266

424 6749 260

424 6750 246

424 6751 233

424 6752 219

424 6753 185

424 6754 176

425 55 63

425 89 699

425 161 29

425 257 30

425 288 190

425 289 638

425 291 10664

425 292 578

425 295 89

425 299 36

425 300 12071

425 301 86

425 302 254

425 306 52

425 311 750

425 314 61

425 318 454

425 323 39

425 325 969

425 328 26

425 333 17115

425 342 275

425 344 4812

425 349 122

425 360 50

425 378 68

425 386 206

425 461 402

425 464 31

425 466 743

425 481 37

425 486 36

425 498 1319

425 528 107

425 544 35

425 646 42

425 680 493

425 681 552

425 686 635

425 701 180

425 704 50

425 797 50

425 802 190

425 966 36

425 1034 53

425 1052 38

425 1455 34

425 1456 39

425 1458 67

425 2020 64

426 53 721

426 63 368

426 64 490

426 70 419

426 81 483

426 103 408

426 107 364

426 138 327

426 162 642

426 175 443

426 238 466

426 418 967

426 444 821

426 603 393

426 621 388

426 632 351

426 697 396

426 727 388

426 747 680

426 748 526

426 756 431

426 772 331

426 782 410

426 1226 379

426 1383 381

426 1418 407

426 1423 402

426 1709 463

426 1731 971

426 1736 324

426 1747 693

426 1800 654

426 1824 342

426 2605 401

426 2676 510

426 3417 388

426 4230 334

426 6151 750

426 6159 768

426 6160 488

426 6755 1801

426 6756 537

426 6757 528

426 6758 482

426 6759 420

426 6760 401

426 6761 392

426 6762 380

426 6763 327

426 6764 323

427 65 397

427 331 1185

427 366 560

427 412 859

427 430 321

427 501 493

427 527 690

427 540 753

427 547 566

427 679 462

427 683 729

427 816 843

427 910 621

427	1043	348
427	1048	393
427	1096	2374
427	1100	978
427	1116	1393
427	1158	326
427	1179	338
427	1192	609
427	1195	541
427	1246	293
427	1723	754
427	1858	649
427	1941	346
427	2574	425
427	2608	399
427	3616	953
427	3754	1380
427	4100	323
427	4223	284
427	4262	3565
427	4762	338
427	5966	378
427	6225	319
427	6453	641
427	6600	735
427	6617	298
427	6765	1284
427	6766	853

427 6767 846

427 6768 842

427 6769 821

427 6770 624

427 6771 515

427 6772 455

427 6773 339

427 6774 320

427 6775 317

428 51 61012

428 55 2518

428 59 2592

428 67 13823

428 72 6337

428 76 1236

428 77 5423

428 85 1077

428 100 3033

428 157 1249

428 159 1309

428 167 5365

428 187 3296

428 627 1181

428 889 2954

428 900 1069

428 985 1573

428 993 1019

428 994 1878

428 997 3131

428 998 2939

428 999 4982

428 1001 12151

428 1004 1294

428 1014 4909

428 1017 1087

428 1019 1131

428 1028 1870

428 1062 1275

428 1076 1787

428 1885 2085

428 1892 3106

428 2531 1050

428 2552 2641

428 2559 2755

428 2562 7835

428 2571 1934

428 3281 2149

428 5255 4695

428 5258 1049

428 6065 995

428 6073 1271

428 6714 3232

428 6742 1008

428 6776 7831

428 6777 1217

428 6778 1138

428 6779 1105

428 6780 1063

428 6781 986

430 51 7488

430	59	538
430	68	200
430	72	5356
430	89	516
430	96	192
430	98	2220
430	99	1878
430	100	181
430	157	187
430	167	870
430	193	915
430	291	227
430	441	232
430	511	3443
430	599	456
430	612	194
430	683	309
430	715	190
430	874	278
430	905	2667
430	993	588
430	997	220
430	999	190
430	1122	615
430	1162	156
430	1795	421
430	1879	8240
430	1889	187
430	1890	196
430	2552	605

430 2556 195

430 2562 235

430 2938 396

430 3201 284

430 3777 150

430 4435 341

430 4704 210

430 5832 152

430 5833 1052

430 6782 2157

430 6783 716

430 6784 547

430 6785 517

430 6786 493

430 6787 474

430 6788 426

430 6789 282

430 6790 209

430 6791 180

431 65 3153

431 154 3033

431 157 1159

431 190 3325

431 207 3116

431 217 2287

431 220 1554

431 227 11443

431 228 2242

431 229 5200

431 230 1535

431 298 1195

431 321 1402

431 355 1816

431 377 5992

431 412 2925

431 424 2429

431 428 1957

431 439 4621

431 453 4877

431 501 1088

431 503 1141

431 511 1653

431 533 9453

431 614 4364

431 705 2725

431 707 1620

431 709 1118

431 843 1105

431 917 1710

431 923 1838

431 951 2386

431 952 1429

431 953 997

431 959 2086

431 966 985

431 982 4863

431 1047 1080

431 1048 1438

431 1104 1718

431 1116 1092

431 1249 4424

431 1772 2773

431 1854 1441

431 2343 1065

431 2346 1141

431 2787 1662

431 2794 1132

431 2797 5203

431 4115 1231

432 220 232

432 225 348

432 329 127

432 377 159

432 486 791

432 489 174

432 494 533

432 497 195

432 498 1626

432 500 1890

432 501 122

432 536 242

432 537 482

432 538 113

432 540 132

432 808 248

432 816 211

432 1041 1411

432 1091 279

432 1116 1511

432 1186 267

432 1200	1498
432 1645	223
432 1650	216
432 1672	595
432 1673	468
432 1866	416
432 1874	337
432 2661	123
432 2835	124
432 2940	334
432 2987	387
432 3097	124
432 3398	137
432 3428	637
432 4262	342
432 4821	154
432 4823	215
432 4832	510
432 4836	122
432 4850	741
432 5087	246
432 6196	328
432 6247	447
432 6536	315
432 6792	579
432 6793	434
432 6794	418
432 6795	238
432 6796	207
433 7	192

433	12	265
433	15	291
433	23	183
433	25	273
433	503	181
433	514	252
433	785	329
433	923	212
433	966	185
433	1044	259
433	1104	341
433	1281	427
433	1797	198
433	1803	338
433	2744	236
433	2963	183
433	2970	263
433	3244	405
433	3800	191
433	4268	224
433	4273	190
433	4275	234
433	6289	691
433	6797	1413
433	6798	1174
433	6799	1067
433	6800	791
433	6801	708
433	6802	603
433	6803	571

433 6804 571

433 6805 493

433 6806 454

433 6807 453

433 6808 295

433 6809 264

433 6810 262

433 6811 247

433 6812 228

433 6813 217

433 6814 210

433 6815 209

433 6816 196

433 6817 192

433 6818 189

433 6819 188

433 6820 186

433 6821 182

433 6822 181

434 53 43

434 56 5

434 61 15

434 81 10

434 206 10

434 219 28

434 233 70

434 234 5

434 434 14

434 578 5

434 599 22

434 605 7

434 611 11

434 612 11

434 629 14

434 748 28

434 1411 11

434 1508 21

434 1881 15

434 1970 26

434 2289 24

434 2470 7

434 3011 6

434 3285 7

434 3354 6

434 3364 31

434 3447 24

434 3666 12

434 3686 16

434 3987 7

434 4064 23

434 4079 6

434 5152 8

434 5434 16

434 5782 16

434 6205 45

434 6823 52

434 6824 31

434 6825 30

434 6826 28

434 6827 21

434 6828 14

434 6829 10

434 6830 9

434 6831 7

434 6832 7

434 6833 6

434 6834 6

434 6835 5

434 6836 5

435 173 1136

435 233 1335

435 492 1697

435 500 843

435 507 2553

435 755 781

435 768 1103

435 779 1199

435 780 874

435 813 1645

435 969 1188

435 1099 1205

435 1100 1483

435 1181 3853

435 1185 4653

435 1188 1562

435 1194 954

435 1195 1476

435 1197 2864

435 1541 3400

435 1853 2556

435 1863 1448

435 2582 1015

435 2810 1621

435 2847 1226

435 2850 1169

435 3027 1851

435 3028 1064

435 3200 1025

435 3729 1079

435 4550 1097

435 4774 1694

435 4898 1159

435 5208 882

435 5969 955

435 6440 1154

435 6837 2972

435 6838 2772

435 6839 2286

435 6840 2234

435 6841 1423

435 6842 1408

435 6843 1185

435 6844 1138

435 6845 1128

435 6846 1109

435 6847 1014

435 6848 971

435 6849 839

435 6850 803

436 51 2178

436 67 810

436 72 1434

436 99 771

436 172 609

436 212 681

436 228 4314

436 424 903

436 429 2891

436 441 694

436 511 2665

436 533 1608

436 562 662

436 683 649

436 706 784

436 715 635

436 734 781

436 735 10311

436 854 627

436 860 2897

436 863 684

436 868 1021

436 951 803

436 965 1430

436 969 1394

436 1089 1410

436 1122 941

436 1244 1417

436 1375 798

436 1384 736

436 1412 966

436 1424 1797

436 1652 2069

436 1816 657

436 2124 601

436 2531 4856

436 2606 1662

436 3097 2125

436 3403 819

436 3617 892

436 3676 766

436 4311 2278

436 5153 891

436 5235 1075

436 5249 734

436 6851 2693

436 6852 913

436 6853 837

436 6854 737

436 6855 667

437 89 147

437 257 191

437 288 141

437 289 418

437 290 174

437 294 175

437 302 143

437 325 655

437 333 133

437 335 185

437 347 128

437 350 232

437 354 606

437 375 341

437 386 127

437 455 179

437 461 252

437 466 118

437 498 129

437 536 181

437 544 816

437 669 192

437 674 318

437 909 186

437 915 137

437 2102 162

437 2646 308

437 2977 443

437 3367 151

437 3570 144

437 3859 179

437 4562 134

437 5465 474

437 5767 172

437 5977 197

437 6368 129

437 6856 722

437 6857 644

437 6858 624

437 6859 611

437 6860 379

437 6861 197
437 6862 156
437 6863 155
437 6864 141
437 6865 131
437 6866 131
437 6867 125
437 6868 123
437 6869 118
438 9 4
438 10 16
438 13 6
438 17 10
438 19 7
438 24 11
438 26 9
438 32 4
438 37 5
438 38 4
438 41 15
438 43 5
438 49 12
438 245 3
438 861 3
438 994 4
438 1270 17
438 1277 4
438 1299 10
438 1303 6
438 1313 3

438 1315	8
438 1332	5
438 1886	3
438 2938	3
438 3551	15
438 3768	3
438 4871	4
438 4873	7
438 4876	4
438 4877	9
438 5238	5
438 6237	4
438 6732	6
438 6736	4
438 6870	12
438 6871	10
438 6872	9
438 6873	9
438 6874	8
438 6875	7
438 6876	7
438 6877	6
438 6878	5
438 6879	5
438 6880	4
438 6881	4
438 6882	4
438 6883	3
438 6884	3
439 267 2265	

439 271 762

439 276 669

439 278 1226

439 475 5431

439 527 1506

439 1402 777

439 1613 1477

439 1636 916

439 1641 849

439 1839 1140

439 2179 789

439 2252 1635

439 2904 1257

439 2906 4197

439 2907 1493

439 2910 1329

439 2913 1007

439 2915 1327

439 2916 1339

439 2921 829

439 2923 1370

439 2927 1244

439 2930 1054

439 2931 714

439 6619 1972

439 6620 957

439 6626 718

439 6631 749

439 6885 4406

439 6886 3196

439 6887 2545

439 6888 1780

439 6889 1333

439 6890 1285

439 6891 1102

439 6892 1094

439 6893 1050

439 6894 1034

439 6895 1030

439 6896 981

439 6897 976

439 6898 972

439 6899 930

439 6900 921

439 6901 819

439 6902 778

439 6903 751

439 6904 712

439 6905 674

440 53 711

440 84 747

440 107 566

440 217 613

440 222 557

440 223 414

440 225 471

440 231 431

440 440 1442

440 441 943

440 445 413

440 602 467

440 603 660

440 606 1132

440 615 412

440 618 526

440 621 1115

440 748 5511

440 757 801

440 1155 491

440 1401 1225

440 1404 1629

440 1633 676

440 1943 585

440 1944 1002

440 1949 464

440 1957 799

440 1964 593

440 1965 1220

440 2285 1110

440 2415 340

440 2572 536

440 2573 402

440 2672 1137

440 2815 421

440 3258 465

440 3686 468

440 3691 357

440 3693 345

440 4058 674

440 6189 1838

440 6295 728

440 6409 1863

440 6824 426

440 6906 917

440 6907 877

440 6908 774

440 6909 729

440 6910 675

440 6911 483

441 54 115

441 58 159

441 59 114

441 64 651

441 65 302

441 72 109

441 84 338

441 225 90

441 226 127

441 228 148

441 229 308

441 233 173

441 238 77

441 331 90

441 425 91

441 532 70

441 533 195

441 715 170

441 851 90

441 964 77

441 982 219

441 1089	64
441 1104	136
441 1119	72
441 1151	159
441 1394	132
441 1398	142
441 1400	76
441 1633	145
441 1873	240
441 1941	322
441 2823	331
441 2923	76
441 3158	82
441 3252	283
441 3412	85
441 4734	193
441 5666	110
441 6365	86
441 6912	621
441 6913	206
441 6914	122
441 6915	90
441 6916	89
441 6917	88
441 6918	76
441 6919	68
441 6920	66
441 6921	65
441 6922	64
442 30	321

442	61	31
442	72	3138
442	75	861
442	77	23
442	182	88
442	187	112009
442	193	51
442	221	29
442	310	30
442	644	40
442	877	926
442	907	51
442	994	23
442	1001	293
442	1013	42
442	1014	113
442	1018	99
442	1059	22
442	1375	27
442	1713	31
442	1797	305
442	3832	3583
442	3982	56
442	4216	70
442	6524	64
442	6733	230
442	6739	74
442	6923	2936
442	6924	704
442	6925	348

442 6926 301

442 6927 289

442 6928 165

442 6929 156

442 6930 113

442 6931 93

442 6932 89

442 6933 89

442 6934 83

442 6935 77

442 6936 77

442 6937 71

442 6938 50

442 6939 43

442 6940 39

442 6941 36

442 6942 26

442 6943 24

442 6944 23

443 57 215

443 152 2227

443 163 3218

443 174 786

443 188 340

443 212 274

443 227 970

443 238 171

443 506 575

443 712 194

443 728 512

443 868 288

443 918 2218

443 920 1714

443 949 370

443 1094 303

443 1226 1282

443 1372 297

443 1639 320

443 1772 181

443 1778 521

443 2006 213

443 2300 9348

443 2304 353

443 2312 534

443 2317 409

443 2323 728

443 2338 500

443 2616 236

443 2786 1429

443 2787 265

443 4548 462

443 5844 2692

443 6945 2445

443 6946 1968

443 6947 1690

443 6948 1237

443 6949 1127

443 6950 1030

443 6951 635

443 6952 502

443 6953 397

443 6954 397

443 6955 336

443 6956 324

443 6957 308

443 6958 261

443 6959 223

443 6960 180

443 6961 168

444 9 49

444 22 58

444 744 124

444 1103 66

444 1229 111

444 1281 86

444 1521 60

444 2030 95

444 2470 51

444 2702 269

444 3528 74

444 3531 166

444 3536 168

444 4887 49

444 6654 188

444 6655 103

444 6659 75

444 6676 386

444 6962 620

444 6963 392

444 6964 335

444 6965	243
444 6966	232
444 6967	187
444 6968	175
444 6969	168
444 6970	125
444 6971	109
444 6972	99
444 6973	96
444 6974	87
444 6975	83
444 6976	82
444 6977	80
444 6978	80
444 6979	78
444 6980	73
444 6981	69
444 6982	67
444 6983	66
444 6984	62
444 6985	61
444 6986	60
444 6987	59
444 6988	59
444 6989	57
444 6990	50
444 6991	50
444 6992	50
444 6993	49
446 51	3669

446 59 1956

446 67 1904

446 72 107031

446 99 1574

446 153 1160

446 154 1384

446 159 1700

446 163 1541

446 166 2031

446 172 1855

446 173 6108

446 180 1556

446 187 3717

446 190 5447

446 212 1421

446 227 1890

446 234 2117

446 441 5662

446 511 8331

446 599 5456

446 735 1961

446 867 1114

446 868 1734

446 874 1347

446 900 1144

446 1001 6219

446 1014 1704

446 1061 5808

446 1072 6825

446 1085 1165

446 1103 1794

446 1109 2429

446 1384 1388

446 1412 1393

446 1700 2312

446 1707 2109

446 1709 1095

446 1713 10597

446 1795 1308

446 1890 1361

446 1892 1369

446 3201 1563

446 3885 1930

446 3886 5439

446 3988 1092

446 6439 5232

446 7007 2271

446 7008 1672

446 7009 1671

447 113 245

447 120 550

447 138 482

447 148 173

447 154 232

447 194 312

447 238 196

447 418 340

447 747 325

447 782 747

447 1736 397

447 1943	190
447 2605	699
447 3506	155
447 3520	381
447 3527	517
447 3541	297
447 3862	299
447 5043	172
447 6582	537
447 6762	965
447 6980	163
447 7010	2465
447 7011	2139
447 7012	1171
447 7013	1139
447 7014	778
447 7015	761
447 7016	596
447 7017	534
447 7018	521
447 7019	436
447 7020	420
447 7021	397
447 7022	376
447 7023	347
447 7024	261
447 7025	220
447 7026	216
447 7027	208
447 7028	200

447 7029 198

447 7030 191

447 7031 188

447 7032 182

447 7033 175

447 7034 175

447 7035 173

447 7036 172

447 7037 156

449 15 137

449 163 581

449 188 375

449 227 129

449 233 106

449 370 162

449 380 185

449 506 118

449 562 92

449 630 107

449 706 124

449 707 168

449 724 111

449 732 97

449 735 231

449 868 108

449 917 158

449 920 139

449 961 208

449 1110 128

449 1112 177

449 1143	126
449 1254	370
449 1259	139
449 1366	139
449 1369	307
449 1372	157
449 1412	602
449 1513	102
449 1531	111
449 1535	103
449 1806	109
449 2343	231
449 2347	129
449 2966	106
449 3417	228
449 3438	192
449 3509	106
449 4271	208
449 4272	194
449 4273	116
449 4605	92
449 5689	181
449 6258	143
449 7043	353
449 7044	131
449 7045	115
449 7046	104
449 7047	96
449 7048	92

450 152 5366

450 163 1435

450 186 585

450 188 2282

450 227 1070

450 370 713

450 506 319

450 562 1501

450 706 274

450 854 212

450 881 287

450 917 186

450 920 3179

450 959 1789

450 1216 280

450 1240 173

450 1249 290

450 1369 658

450 1372 504

450 1411 405

450 1412 20923

450 1418 212

450 1426 660

450 1513 495

450 1727 469

450 1998 565

450 2003 317

450 2006 229

450 2605 340

450 3418 163

450 3420 366

450 3888 196

450 3940 496

450 4251 1001

450 4675 606

450 6264 177

450 6765 290

450 6830 250

450 7049 559

450 7050 346

450 7051 311

450 7052 300

450 7053 300

450 7054 256

450 7055 251

450 7056 222

450 7057 207

450 7058 181

450 7059 159

450 7060 158

451 89 5857

451 190 688

451 230 2205

451 288 5028

451 289 1673

451 292 691

451 295 967

451 300 5324

451 306 6494

451 314 571

451 321 402

451 324 1800

451 325 920

451 327 437

451 328 1169

451 329 521

451 333 1320

451 344 1727

451 349 1988

451 355 397

451 377 914

451 458 54061

451 461 2635

451 464 6076

451 466 6017

451 470 1019

451 475 607

451 498 11269

451 524 1306

451 526 396

451 528 487

451 544 453

451 679 455

451 680 464

451 681 1878

451 686 5059

451 687 430

451 689 687

451 701 706

451 704 3684

451 802 434

451 1043 774

451 1047 8297

451 1449 403

451 1458 490

451 1678 522

451 2020 836

451 4621 423

451 5749 473

451 7061 623

452 51 172

452 67 123

452 72 142

452 85 39

452 93 36

452 100 31

452 163 50

452 187 86

452 511 24

452 554 41

452 599 16

452 712 24

452 717 100

452 877 27

452 889 58

452 951 24

452 959 121

452 997 23

452 998 144

452 999 33

452 1001 125

452 1008	19
452 1014	211
452 1019	35
452 1027	28
452 1081	58
452 1413	42
452 1414	30
452 1464	39
452 1686	26
452 2029	80
452 2444	27
452 2552	19
452 2554	29
452 2609	44
452 2901	18
452 2933	28
452 2936	26
452 3966	22
452 4975	22
452 6736	19
452 7062	819
452 7063	227
452 7064	194
452 7065	37
452 7066	27
452 7067	27
452 7068	20
452 7069	20
452 7070	18
453 51	235

453 59 365

453 65 375

453 72 641

453 84 185

453 99 111

453 154 251

453 157 459

453 159 129

453 172 238

453 173 219

453 190 364

453 208 180

453 225 214

453 227 186

453 229 238

453 233 100

453 429 338

453 436 272

453 441 1982

453 450 246

453 511 188

453 533 150

453 554 268

453 562 138

453 706 104

453 863 124

453 903 106

453 951 100

453 969 136

453 971 108

453 985 142

453 1090 99

453 1384 354

453 1400 123

453 1810 141

453 1814 404

453 2346 106

453 3200 119

453 3744 163

453 3892 97

453 4149 251

453 5926 101

453 7071 209

453 7072 133

453 7073 129

453 7074 124

453 7075 118

453 7076 113

453 7077 109

454 27 431

454 238 718

454 520 314

454 631 1512

454 861 279

454 864 601

454 867 499

454 876 998

454 880 989

454 976 284

454 1061 353

454 1098	343
454 1203	7273
454 1253	332
454 1394	473
454 1504	361
454 1534	288
454 1707	796
454 2026	2588
454 2030	441
454 2042	308
454 2470	275
454 2472	3260
454 3280	339
454 3289	1030
454 3431	589
454 3443	474
454 3513	569
454 4873	289
454 4875	409
454 5299	666
454 6871	371
454 10162	993
454 11107	280
454 12338	281
454 12505	268
454 12508	381
454 12509	351
454 13754	389
454 18734	1032
454 18735	895

454 18736 879

454 18737 560

454 18738 408

454 18739 379

454 18740 320

454 18741 301

454 18742 294

454 18743 287

454 18744 286

455 19 2496

455 27 2690

455 32 4675

455 36 3224

455 39 3564

455 45 2396

455 59 1780

455 64 2671

455 81 2239

455 84 1737

455 107 5449

455 153 1826

455 162 1786

455 190 2805

455 233 40946

455 238 1938

455 407 3697

455 450 2864

455 512 3608

455 515 1822

455 517 2429

455	519	2720
455	603	3588
455	748	2597
455	864	2652
455	1062	2942
455	1118	2835
455	1119	8340
455	1203	2850
455	1206	1871
455	1277	4322
455	1745	2677
455	1886	3397
455	1983	5959
455	2369	4633
455	2473	1845
455	2486	2562
455	2691	3678
455	3109	2458
455	3191	3041
455	3285	4108
455	3562	2426
455	4025	2044
455	5197	6709
455	6257	3000
455	7078	3019
455	7079	2786
455	7080	2671
455	7081	2108
455	7082	1811
456	65	83

456 89 56

456 230 121

456 257 68

456 288 44

456 295 254

456 321 43

456 333 994

456 475 47

456 542 798

456 646 59

456 707 47

456 966 116

456 1045 102

456 1249 73

456 1659 45

456 1681 240

456 2080 402

456 2984 54

456 3173 59

456 3378 273

456 3384 64

456 3387 678

456 3389 132

456 3392 175

456 4462 127

456 4463 266

456 4469 202

456 4470 51

456 4474 946

456 4514 46

456 4850 112

456 5022 59

456 5226 72

456 5594 782

456 5749 698

456 7061 548

456 7083 154

456 7084 98

456 7085 97

456 7086 95

456 7087 81

456 7088 75

456 7089 65

456 7090 64

456 7091 62

456 7092 51

456 7093 46

456 7094 45

456 7095 45

457 89 449

457 176 125

457 188 268

457 380 392

457 506 371

457 518 370

457 679 877

457 918 86

457 920 87

457 924 617

457 925 96

457 930 4704

457 937 117

457 946 787

457 955 274

457 957 247

457 1104	102
457 1114	920
457 1117	192
457 1339	154
457 1358	353
457 1360	200
457 1705	169
457 1993	223
457 2343	139
457 2346	211
457 2478	184
457 2787	349
457 2852	185
457 3901	785
457 5076	287
457 5585	92
457 6282	97
457 6284	421
457 7096	823
457 7097	586
457 7098	346
457 7099	342
457 7100	274
457 7101	227
457 7102	194

457 7103 144

457 7104 135

457 7105 131

457 7106 127

457 7107 124

457 7108 106

457 7109 93

457 7110 93

457 7111 90

458 55 54

458 65 69

458 67 75

458 69 43

458 72 136

458 97 67

458 157 50

458 163 206

458 187 242

458 190 337

458 207 73

458 209 125

458 227 181

458 229 123

458 233 79

458 288 64

458 295 153

458 329 46

458 333 56

458 371 85

458 378 75

458 432 47

458 453 144

458 486 905

458 493 93

458 498 168

458 504 44

458 511 59

458 512 94

458 517 129

458 518 69

458 540 45

458 544 597

458 599 53

458 603 46

458 706 98

458 707 177

458 709 43

458 959 125

458 1001 65

458 1098 64

458 1116 72

458 1121 71

458 1246 98

458 1382 113

458 1412 84

458 1413 60

458 1464 72

458 3621 140

458 5966 136

459 15 3124

459 25 626

459 176 878

459 198 110185

459 706 1217

459 707 831

459 710 400

459 724 1861

459 821 940

459 835 3899

459 917 1154

459 918 4437

459 923 15541

459 952 16947

459 956 1542

459 1249 45704

459 1259 2125

459 1358 1354

459 1360 853

459 1366 5487

459 1369 762

459 1412 429

459 1803 6271

459 1808 426

459 1810 473

459 2143 1575

459 2172 420

459 2341 956

459 2343 585

459 2654 602

459 2784 575

459 2787 2257

459 2835 5575

459 2901 497

459 2963 459

459 2970 781

459 2973 461

459 3474 440

459 4165 6220

459 4180 723

459 4270 360

459 4460 817

459 5004 627

459 7112 912

459 7113 756

459 7114 525

459 7115 524

459 7116 484

459 7117 467

459 7118 452

460 53 420

460 72 486

460 81 212

460 154 283

460 173 400

460 197 521

460 206 295

460 207 254

460 212 215

460 238 340

460 418 1875

460	436	207
460	444	194
460	533	130
460	591	569
460	603	207
460	748	221
460	753	142
460	986	262
460	1090	285
460	1098	379
460	1112	179
460	1121	1042
460	1123	1341
460	1130	1434
460	1146	340
460	1394	267
460	1515	127
460	1700	252
460	1736	307
460	1851	179
460	1859	129
460	2136	213
460	2346	427
460	2747	123
460	3057	362
460	3827	182
460	3863	381
460	4896	206
460	5073	179
460	5216	157

460 7119 901

460 7120 423

460 7121 379

460 7122 197

460 7123 182

460 7124 167

460 7125 162

460 7126 143

460 7127 127

461 52 18

461 65 1379

461 67 92

461 70 131

461 72 46

461 86 115

461 157 89

461 220 22

461 227 52

461 333 35

461 511 17403

461 521 63

461 538 141

461 715 69

461 734 253

461 913 32

461 962 20

461 986 24

461 993 123

461 1375 291

461 1410 61

461 1464	55
461 1772	43
461 1795	32
461 2018	19
461 2067	65
461 2277	72
461 2668	25
461 2742	23
461 2900	44
461 3302	253
461 3763	52
461 5447	21
461 6396	48
461 6789	40
461 7128	955
461 7129	92
461 7130	88
461 7131	67
461 7132	57
461 7133	52
461 7134	51
461 7135	46
461 7136	43
461 7137	41
461 7138	40
461 7139	23
461 7140	22
461 7141	21
461 7142	20
462 154 842	

462 159 1028

462 206 270

462 211 776

462 219 255

462 223 254

462 227 1331

462 416 354

462 429 345

462 511 253

462 599 252

462 610 289

462 612 256

462 618 431

462 715 1805

462 857 437

462 859 387

462 868 481

462 910 802

462 1414 262

462 1643 507

462 1881 602

462 1957 742

462 2071 314

462 2415 364

462 2424 379

462 2478 1990

462 2531 293

462 2624 395

462 2629 522

462 2633 367

462 2640 510

462 4079 776

462 5549 532

462 6229 290

462 6824 655

462 7143 1183

462 7144 857

462 7145 782

462 7146 726

462 7147 466

462 7148 431

462 7149 428

462 7150 416

462 7151 400

462 7152 357

462 7153 356

462 7154 299

462 7155 257

462 7156 253

463 46 10

463 63 129

463 157 97

463 230 9

463 245 11

463 253 13

463 378 13

463 389 16

463 390 12

463 726 14

463 747 22

463 910 112

463 930 72

463 1098	82
463 1226	9
463 1705	15
463 1707	24
463 1715	9
463 1800	11
463 1893	11
463 2068	22
463 2104	11
463 2419	10
463 2681	27
463 3310	40
463 4920	64
463 4922	80
463 4927	38
463 5605	22
463 6397	37
463 7157	83
463 7158	43
463 7159	26
463 7160	23
463 7161	21
463 7162	17
463 7163	16
463 7164	15
463 7165	15
463 7166	14
463 7167	14

463 7168 14

463 7169 14

463 7170 13

463 7171 12

463 7172 12

463 7173 12

463 7174 11

463 7175 11

463 7176 11

464 65 252

464 159 1524

464 207 144

464 217 412

464 225 129

464 228 238

464 229 759

464 419 539

464 424 3674

464 427 1788

464 533 1344

464 703 125

464 716 5151

464 735 412

464 857 305

464 868 272

464 969 612

464 1090 3318

464 1101 238

464 1376 376

464 1377 144

464 1390	762
464 1516	963
464 1517	206
464 2134	513
464 2539	232
464 2587	4012
464 2601	605
464 2666	112
464 2815	288
464 3030	102
464 3370	269
464 3767	620
464 5030	1145
464 6223	211
464 6920	117
464 7177	768
464 7178	432
464 7179	361
464 7180	320
464 7181	308
464 7182	306
464 7183	298
464 7184	266
464 7185	263
464 7186	263
464 7187	218
464 7188	157
464 7189	117
464 7190	112
465 56	133

465 65 477

465 67 122

465 72 117

465 88 143

465 89 399

465 154 647

465 155 142

465 190 982

465 199 474

465 203 431

465 207 512

465 220 121

465 223 146

465 226 567

465 228 340

465 229 172

465 230 823

465 233 1748

465 424 863

465 425 197

465 434 286

465 436 114

465 443 114

465 511 200

465 533 1781

465 709 503

465 716 373

465 757 125

465 969 1842

465 1090 1295

465 1099 125

465 1151 217

465 1376 147

465 1384 137

465 1390 239

465 1398 126

465 1400 261

465 1510 720

465 1511 150

465 1520 115

465 1777 155

465 1964 129

465 2405 158

465 2535 230

465 2587 165

465 2823 200

465 4115 146

465 6054 152

465 6464 120

466 67 138

466 89 838

466 288 376

466 289 142

466 292 364

466 295 115

466 298 210

466 300 365

466 302 211

466 306 169

466 328 112

466 333 384

466 349 131

466 378 298

466 386 213

466 389 108

466 453 229

466 461 215

466 466 537

466 504 142

466 518 945

466 526 207

466 542 345

466 544 468

466 641 121

466 679 461

466 680 233

466 690 552

466 704 259

466 915 332

466 930 171

466 972 178

466 1034 392

466 1098 598

466 1673 245

466 1775 116

466 1781 226

466 1905 134

466 2014 705

466 2022 193

466 2988 304

466 4135	181
466 4561	182
466 5459	1190
466 6201	292
466 7191	705
466 7192	266
466 7193	238
466 7194	124
466 7195	111
467 213 186	
467 1126	249
467 4722	140
467 7196	867
467 7197	381
467 7198	331
467 7199	287
467 7200	270
467 7201	259
467 7202	249
467 7203	234
467 7204	222
467 7205	219
467 7206	218
467 7207	218
467 7208	215
467 7209	211
467 7210	210
467 7211	207
467 7212	206
467 7213	195

467 7214	193
467 7215	191
467 7216	190
467 7217	189
467 7218	189
467 7219	186
467 7220	185
467 7221	183
467 7222	181
467 7223	174
467 7224	171
467 7225	171
467 7226	171
467 7227	169
467 7228	166
467 7229	165
467 7230	164
467 7231	160
467 7232	159
467 7233	159
467 7234	156
467 7235	154
467 7236	150
467 7237	149
467 7238	149
467 7239	148
467 7240	148
467 7241	140
467 7242	139
468 65	525

468 157 1088

468 289 238

468 316 424

468 333 339

468 599 978

468 707 1253

468 715 1097

468 889 489

468 900 260

468 1103 265

468 1121 345

468 1123 583

468 1242 1078

468 1383 1398

468 1412 687

468 1464 259

468 1468 422

468 1762 472

468 1772 6050

468 1846 270

468 1881 287

468 1889 754

468 2044 380

468 2068 485

468 2176 570

468 2265 886

468 2463 454

468 2478 867

468 2938 243

468 3110 1270

468 3675 391

468 4918 269

468 5425 292

468 6250 428

468 7243 4911

468 7244 1383

468 7245 801

468 7246 650

468 7247 528

468 7248 422

468 7249 385

468 7250 363

468 7251 305

468 7252 296

468 7253 289

468 7254 279

468 7255 278

468 7256 276

468 7257 266

469 63 176

469 163 379

469 173 191

469 181 175

469 188 167

469 206 277

469 378 419

469 425 1043

469 472 180

469 488 787

469 490 207

469 504 239

469 506 172

469 518 381

469 562 211

469 706 350

469 707 254

469 709 186

469 726 181

469 812 267

469 821 489

469 824 322

469 841 379

469 856 224

469 903 166

469 917 1355

469 955 514

469 957 311

469 966 184

469 1109 255

469 1113 1148

469 1118 403

469 1143 377

469 1249 226

469 1369 197

469 2206 358

469 2379 188

469 2954 493

469 3717 264

469 4143 181

469 4179 474

469 4341 378

469 5437 285

469 5768 325

469 7258 335

469 7259 228

469 7260 198

469 7261 167

469 7262 165

469 7263 163

470 55 1757

470 67 5814

470 89 2545

470 157 568

470 180 559

470 227 657

470 253 474

470 257 372

470 288 2063

470 289 8353

470 290 546

470 291 465

470 292 4942

470 293 789

470 295 1939

470 298 557

470 300 1478

470 302 381

470 306 1229

470 310 876

470 318 657

470 320 725

470 325 1249

470 329 406

470 333 1403

470 342 598

470 344 1665

470 352 462

470 461 1253

470 466 760

470 523 402

470 525 712

470 526 611

470 528 813

470 544 757

470 646 406

470 686 1176

470 701 709

470 704 584

470 802 542

470 889 1379

470 903 740

470 908 974

470 959 369

470 1459 1059

470 1464 590

470 1580 540

470 2031 538

470 3302 386

470 3397 466

471 154 120

471 157 78

471 163 324

471 197 87

471 198 70

471 199 76

471 212 352

471 227 1901

471 230 62

471 267 86

471 331 119

471 429 118

471 440 197

471 475 74

471 562 343

471 580 70

471 599 227

471 605 72

471 610 112

471 707 82

471 709 95

471 718 117

471 733 137

471 735 141

471 857 92

471 863 173

471 868 78

471 949 71

471 959 151

471 1130 61

471 1239 590

471 1246 117

471 1254 65

471 1369 133

471 1412 98

471 1422 73

471 2343 74

471 2375 65

471 2475 73

471 2624 373

471 3065 73

471 3217 178

471 3531 69

471 4538 131

471 4543 109

471 5660 84

471 7264 180

471 7265 175

471 7266 69

471 7267 69

472 55 196

472 56 224

472 67 105

472 89 1490

472 161 245

472 176 77

472 191 107

472 199 539

472 228 709

472 288 1087

472 289 573

472 295 87

472 299 289

472 300 403

472 306 245

472 323 79

472 328 88

472 330 135

472 333 427

472 352 79

472 386 151

472 403 167

472 461 135

472 466 782

472 475 198

472 486 285

472 498 306

472 531 132

472 541 153

472 543 769

472 544 153

472 546 293

472 686 178

472 802 207

472 907 107

472 1032 141

472 1379 1382

472 1400 879

472 1455 194

472 1456 322

472 1613 129

472 1672 280

472 2081 123

472 2085 250

472 2990 115

472 4463 85

472 4814 113

472 7268 205

472 7269 126

472 7270 95

473 89 324

473 288 312

473 289 382

473 292 192

473 295 245

473 298 271

473 300 257

473 311 332

473 316 1042

473 318 159

473 362 225

473 430 216

473 523 139

473 525 652

473 542 211

473 697 406

473 903 140

473 991 180

473 1505 188

473 2392 166

473 2544 155

473 2980	162
473 3102	265
473 3110	310
473 3288	139
473 5035	221
473 5555	259
473 6454	184
473 7271	506
473 7272	462
473 7273	352
473 7274	332
473 7275	301
473 7276	275
473 7277	246
473 7278	246
473 7279	221
473 7280	204
473 7281	197
473 7282	186
473 7283	169
473 7284	168
473 7285	163
473 7286	151
473 7287	151
473 7288	150
473 7289	149
473 7290	148
473 7291	141
473 7292	140
474 7	1150

474 39 1491

474 67 243

474 70 243

474 72 28153

474 190 1602

474 289 7773

474 295 268

474 328 697

474 377 183

474 407 203

474 907 142

474 968 159

474 1009 456

474 1099 2021

474 1104 171

474 1306 167

474 1394 125

474 1401 1806

474 2509 118

474 3288 154

474 4024 130

474 4725 200

474 4728 139

474 5074 155

474 7293 1311

474 7294 568

474 7295 523

474 7296 476

474 7297 428

474 7298 427

474 7299 412
474 7300 344
474 7301 303
474 7302 274
474 7303 246
474 7304 218
474 7305 204
474 7306 186
474 7307 173
474 7308 171
474 7309 166
474 7310 158
474 7311 154
474 7312 149
474 7313 144
474 7314 136
474 7315 129
474 7316 124
474 7317 112
475 65 173
475 182 177
475 203 182
475 204 189
475 207 181
475 212 126
475 225 144
475 227 461
475 228 283
475 229 1698
475 231 147

475 355 123

475 416 177

475 421 201

475 424 174

475 439 288

475 486 264

475 488 547

475 533 129

475 705 171

475 716 126

475 969 123

475 1048 2544

475 1090 150

475 1151 318

475 1239 347

475 1375 239

475 1376 241

475 1390 123

475 1470 163

475 1511 227

475 1515 397

475 1517 133

475 1518 277

475 1652 232

475 1934 128

475 1988 133

475 2130 286

475 2524 502

475 2612 166

475 2614 283

475 3255 244

475 3400 209

475 3617 124

475 7318 228

475 7319 193

475 7320 171

475 7321 167

475 7322 147

475 7323 122

476 72 123

476 88 3113

476 89 107

476 154 548

476 173 491

476 190 2861

476 203 5567

476 207 1133

476 208 156

476 209 134

476 225 174

476 227 270

476 228 587

476 229 267

476 234 252

476 238 422

476 377 1795

476 424 312

476 432 182

476 460 221

476 491 408

476 533 997

476 599 115

476 620 132

476 689 143

476 857 268

476 969 2097

476 1075 235

476 1090 1047

476 1106 93

476 1107 250

476 1151 323

476 1379 1300

476 1400 457

476 1512 318

476 2029 182

476 2122 158

476 2130 106

476 2134 141

476 2193 106

476 2537 1505

476 2738 97

476 5048 166

476 5071 199

476 5836 104

476 6439 130

476 7324 206

476 7325 142

476 7326 127

476 7327 104

477 55 1087

477 67 1110

477 71 116

477 89 472

477 157 98

477 161 643

477 180 159

477 209 122

477 257 352

477 265 67

477 285 269

477 288 898

477 289 4749

477 292 2722

477 295 150

477 298 151

477 299 191

477 300 663

477 302 243

477 306 340

477 314 65

477 317 167

477 320 68

477 328 240

477 329 93

477 331 191

477 333 60

477 340 132

477 349 134

477 378 185

477 461 79

477 466 406

477 475 102

477 518 337

477 523 81

477 525 165

477 526 248

477 532 143

477 689 74

477 701 1421

477 889 179

477 903 1013

477 1062 62

477 1098 191

477 1580 158

477 1688 145

477 1934 219

477 1990 346

477 2083 422

477 6448 54

478 230 1799

478 250 308

478 366 2645

478 412 1578

478 424 177

478 498 1664

478 706 154

478 840 1138

478 932 1106

478 982 134

478 1206 2437

478 1513	1566
478 1545	316
478 1645	2715
478 1654	1829
478 1669	326
478 1866	251
478 1974	142
478 2637	3653
478 3398	139
478 3444	240
478 4198	213
478 4211	2490
478 4216	1023
478 4218	454
478 4375	190
478 4793	261
478 4820	151
478 4821	313
478 4822	3866
478 4823	2478
478 4828	1077
478 4834	1584
478 4847	601
478 5450	224
478 5630	2460
478 5639	1335
478 6121	193
478 7328	2608
478 7329	831
478 7330	746

478 7331 447

478 7332 320

478 7333 280

478 7334 232

478 7335 201

478 7336 194

478 7337 190

478 7338 184

478 7339 179

479 55 130

479 67 95

479 89 517

479 157 7813

479 241 116

479 257 378

479 285 2246

479 288 3740

479 289 6112

479 291 1421

479 292 13091

479 293 243

479 294 205

479 295 2125

479 299 1130

479 300 2260

479 301 3459

479 302 36

479 306 628

479 314 583

479 318 23

479 320 84

479 329 1554

479 330 86

479 331 392

479 332 19

479 333 87

479 349 245

479 352 46

479 458 253

479 461 369

479 463 23

479 466 27

479 468 6095

479 475 2430

479 484 1397

479 486 1917

479 538 353

479 540 734

479 543 19

479 544 52

479 701 104

479 907 42

479 913 20

479 1379 30

479 1444 25

479 2179 44

479 2905 138

479 3313 22

479 7340 62

480 51 330

480 59 132

480 72 569

480 75 175

480 82 131

480 98 290

480 99 119

480 100 246

480 159 401

480 187 143

480 193 246

480 599 190

480 852 359

480 993 309

480 997 510

480 998 528

480 999 205

480 1001 226

480 1004 286

480 1006 166

480 1013 537

480 1014 334

480 1017 268

480 1018 251

480 1019 161

480 1027 331

480 1073 247

480 1878 218

480 1886 374

480 1890 182

480 1891 254

480 1895 137

480 1897 206

480 2488 194

480 2936 432

480 3768 348

480 4123 182

480 5254 119

480 6064 134

480 6158 116

480 6241 135

480 6732 181

480 6784 163

480 7341 296

480 7342 206

480 7343 183

480 7344 126

480 7345 125

480 7346 116

480 7347 115

481 55 824

481 65 86

481 67 577

481 89 5733

481 154 74

481 227 350

481 289 138

481 292 516

481 295 80

481 300 167

481 306 136

481 324 104

481 423 133

481 425 226

481 498 233

481 525 90

481 526 288

481 528 124

481 532 270

481 548 532

481 599 250

481 679 107

481 680 250

481 689 838

481 690 599

481 703 170

481 704 143

481 757 285

481 889 145

481 898 167

481 961 128

481 972 767

481 1001 98

481 1034 298

481 1037 126

481 1090 108

481 1098 1072

481 1243 85

481 1609 658

481 1767 201

481 1807 121

481 1983 97

481 2094 216

481 2521 135

481 2988 83

481 3200 377

481 3739 250

481 7348 749

481 7349 372

481 7350 79

482 56 108

482 67 311

482 89 497

482 157 70

482 173 806

482 182 50

482 190 72

482 225 189

482 227 161

482 228 131

482 229 261

482 295 53

482 300 316

482 306 57

482 378 114

482 412 128

482 439 92

482 486 230

482 498 161

482 526 188

482 615 1160

482 679 64

482 689 77

482 716 52

482 1104 126

482 1106 290

482 1126 75

482 1242 64

482 1243 51

482 1246 76

482 1377 123

482 1390 64

482 1400 50

482 1406 106

482 1416 207

482 1464 99

482 1492 54

482 1771 108

482 1782 52

482 2176 343

482 2607 81

482 2632 57

482 2638 140

482 3186 54

482 7324 168

482 7351 257

482 7352 155

482 7353 154

482 7354 76

482 7355 64

484 7 626

484	52	150
484	67	221
484	72	879
484	151	148
484	173	159
484	176	129
484	190	462
484	192	124
484	198	204
484	199	172
484	220	231
484	407	270
484	480	117
484	603	127
484	732	127
484	959	95
484	972	268
484	1104	759
484	1106	217
484	1127	183
484	1784	92
484	1788	177
484	1796	466
484	2193	121
484	2681	115
484	2738	176
484	2743	113
484	2747	253
484	2818	138
484	3047	153

484 3056 153

484 3057 229

484 3827 438

484 3830 183

484 4297 382

484 6245 682

484 6265 354

484 7362 268

484 7363 246

484 7364 231

484 7365 184

484 7366 168

484 7367 138

484 7368 136

484 7369 119

484 7370 106

484 7371 97

484 7372 95

484 7373 93

485 154 110

485 166 30

485 205 32

485 220 36

485 226 33

485 234 115

485 418 56

485 610 78

485 614 110

485 755 29

485 854 59

485 860 28

485 879 34

485 882 38

485 949 29

485 959 136

485 964 38

485 977 80

485 1274 37

485 1418 190

485 1423 62

485 1426 51

485 1491 37

485 1504 119

485 2473 96

485 2493 80

485 2542 43

485 2585 34

485 3364 68

485 3447 29

485 4092 47

485 5423 181

485 6048 67

485 6724 98

485 7374 160

485 7375 148

485 7376 126

485 7377 98

485 7378 74

485 7379 68

485 7380 55

485 7381 52

485 7382 38

485 7383 36

485 7384 32

485 7385 32

485 7386 32

485 7387 29

485 7388 28

485 7389 27

486 55 112

486 67 196

486 89 652

486 157 11000

486 234 60

486 251 3

486 257 154

486 258 34

486 265 56

486 285 125

486 288 254

486 289 4761

486 291 17

486 292 6400

486 294 12

486 295 74

486 299 656

486 300 945

486 302 57

486 306 118

486 333 42

486 346 28

486 349 40

486 352 25

486 475 24

486 523 50

486 528 13

486 543 12

486 701 6183

486 2182 702

486 4767 22

486 7390 62

487 89 252

487 257 117

487 265 301

487 293 168

487 298 541

487 300 637

487 318 122

487 321 817

487 326 121

487 333 866

487 344 1922

487 360 278

487 386 121

487 456 122

487 461 16592

487 463 170

487 466 280

487 471 133

487 498 352

487 540 228

487 681 1255

487 793 9932

487 1239	247
487 1242	176
487 1243	144
487 1244	203
487 1246	223
487 1453	477
487 1458	172
487 1459	932
487 1590	262
487 1684	1869
487 2176	118
487 2439	150
487 3489	440
487 3754	370
487 5767	186
487 7391	1447
487 7392	1354
487 7393	704
487 7394	467
487 7395	455
487 7396	361
487 7397	341
487 7398	236
487 7399	223
487 7400	195
487 7401	147
487 7402	135

487 7403 119

488 51 165

488 154 305

488 190 206

488 206 279

488 207 231

488 225 171

488 226 239

488 227 259

488 229 223

488 245 167

488 250 225

488 418 296

488 424 185

488 425 229

488 436 231

488 454 131

488 511 146

488 614 107

488 716 194

488 730 113

488 735 128

488 868 263

488 961 173

488 969 288

488 1048 105

488 1090 323

488 1118 161

488 1131 148

488 1239 175

488 1412 200

488 1415 128

488 1426 125

488 1512 129

488 1515 172

488 1639 186

488 1958 139

488 1977 135

488 2044 151

488 2265 165

488 2630 135

488 2786 166

488 2857 139

488 3110 111

488 3400 115

488 3475 184

488 3482 113

488 7265 186

488 7404 160

488 7405 117

488 7406 104

489 163 2268

489 190 755

489 196 495

489 203 479

489 212 337

489 226 840

489 227 73

489 229 83

489 234 946

489 424 217

489 472 50

489 511 135

489 533 528

489 554 71

489 562 2881

489 614 375

489 706 105

489 707 6108

489 709 900

489 735 2115

489 841 66

489 863 46

489 868 9502

489 959 297

489 982 1035

489 1240 1263

489 1351 88

489 1360 183

489 1372 411

489 1412 15223

489 1415 244

489 1426 470

489 1639 296

489 1805 69

489 1873 423

489 2352 110

489 2786 544

489 2834 230

489 3482 48

489 4051 168

489 4251 59

489 6581 1044

489 6950 1091

489 7056 211

489 7248 58

489 7407 168

489 7408 116

489 7409 80

489 7410 60

489 7411 60

490 7 233

490 53 427

490 89 1125

490 154 181

490 172 288

490 173 202

490 199 592

490 207 195

490 209 608

490 228 380

490 229 208

490 289 276

490 298 320

490 324 436

490 378 286

490 423 228

490 424 579

490 464 281

490 486 205

490 498 293

490 518 2460

490 533 200

490 541 1475

490 546 370

490 757 288

490 903 289

490 930 287

490 955 390

490 1034 1221

490 1041 236

490 1048 867

490 1114 213

490 1400 219

490 1406 211

490 2014 641

490 2038 237

490 3200 503

490 3367 582

490 3564 216

490 4136 933

490 4704 276

490 6548 504

490 6915 510

490 7412 1472

490 7413 784

490 7414 780

490 7415 261

490 7416 256

490 7417 228

490 7418 221

491 12 315

491 152 372

491 163 1767

491 226 258

491 533 273

491 583 242

491 706 1551

491 707 4842

491 710 2033

491 821 551

491 841 2768

491 843 902

491 846 612

491 917 808

491 918 5186

491 920 748

491 947 782

491 949 517

491 954 576

491 1206 722

491 1257 564

491 1260 305

491 1261 578

491 1276 307

491 1337 301

491 1351 464

491 1358 1096

491 1360 3579

491 1364 592

491 1369 454
491 1372 696
491 1412 582
491 1523 252
491 1527 681
491 1528 410
491 1531 219
491 1975 289
491 2360 443
491 2744 209
491 2765 1021
491 2834 497
491 2901 403
491 3509 526
491 3624 2339
491 3636 567
491 7419 479
491 7420 389
491 7421 374
491 7422 332
491 7423 226
492 159 344
492 167 230
492 192 250
492 227 388
492 230 284
492 231 504
492 544 230
492 609 324
492 610 337

492 903 303

492 959 349

492 1097	266
492 1112	951
492 1121	425
492 1167	267
492 1414	971
492 1772	415
492 1804	378
492 1901	350
492 1944	241
492 1947	452
492 1980	217
492 2136	311
492 2616	409
492 2627	356
492 2632	2487
492 3112	917
492 3371	215
492 5925	213
492 5982	515
492 6540	280
492 7250	371
492 7323	425
492 7424	822
492 7425	757
492 7426	755
492 7427	662
492 7428	457
492 7429	436

492 7430 411

492 7431 350

492 7432 342

492 7433 339

492 7434 302

492 7435 269

492 7436 265

492 7437 250

492 7438 239

492 7439 227

492 7440 211

493 53 18

493 59 19

493 65 1

493 67 130

493 72 4577

493 93 56

493 99 230

493 159 1

493 190 755

493 203 12

493 234 654

493 302 33

493 333 103

493 377 206

493 441 64

493 511 164

493 868 4

493 880 80

493 951 12

493 959 2
493 1072 829
493 1713 804
493 2786 3
493 2919 17
493 3627 4
493 5483 3
493 5486 7
493 7441 105
493 7442 48
493 7443 45
493 7444 27
493 7445 23
493 7446 3
494 55 152
494 89 580
494 234 153
494 289 3726
494 291 863
494 292 1396
494 293 766
494 296 280
494 298 293
494 310 159
494 316 224
494 318 583
494 325 290
494 333 374
494 344 1364
494 425 414

494 461 565

494 465 362

494 498 661

494 528 764

494 534 387

494 535 206

494 536 373

494 548 536

494 680 1011

494 681 1210

494 686 901

494 689 213

494 690 670

494 906 355

494 909 549

494 985 383

494 1590 1187

494 1684 1201

494 2019 823

494 2435 253

494 2546 331

494 3075 141

494 3171 219

494 3319 496

494 3489 1689

494 4704 208

494 4705 202

494 4707 368

494 4773 223

494 5767 223

494 6591 235

494 7447 245

494 7448 144

494 7449 129

495 55 74

495 157 34

495 257 157

495 258 40

495 265 33

495 283 66

495 285 31

495 288 188

495 289 1692

495 291 103

495 292 682

495 294 145

495 295 50

495 299 447

495 300 31

495 318 36

495 320 57

495 325 68

495 333 62

495 340 345

495 344 34

495 349 35

495 523 315

495 544 79

495 701 331

495 786 1251

495 790 100

495 792 653

495 797 33

495 903 157

495 1051 35

495 1052 30

495 2080 36

495 2091 327

495 3074 74

495 3176 56

495 3332 72

495 3393 34

495 4350 38

495 4489 42

495 4707 131

495 5036 50

495 6647 42

495 6701 442

495 6706 154

495 6715 40

495 7450 123

495 7451 62

495 7452 36

495 7453 35

496 6 95

496 9 161

496 16 88

496 30 205

496 39 89

496 46 50

496 56 242

496 65 237

496 154 107

496 159 1801

496 163 192

496 166 59

496 173 287

496 188 73

496 190 312

496 504 471

496 515 58

496 518 63

496 522 2245

496 726 57

496 868 94

496 875 210

496 920 59

496 930 485

496 959 867

496 1069 53

496 1083 51

496 1090 471

496 1109 1008

496 1114 238

496 1116 127

496 1132 61

496 1295 73

496 1298 92

496 1300 87

496 1303 94

496 1491 183

496 1914 328

496 2055 415

496 4317 87

496 4872 71

496 5020 56

496 6282 169

496 7454 274

496 7455 231

496 7456 201

496 7457 143

496 7458 129

496 7459 129

496 7460 56

497 55 65

497 67 33

497 71 60

497 89 430

497 97 64

497 257 63

497 288 4383

497 289 2560

497 292 111

497 295 168

497 298 72

497 300 838

497 302 67

497 306 33

497 310 310

497 314 29

497 325 44

497 326 64

497 333 165

497 352 35

497 362 36

497 378 26

497 464 62

497 466 84

497 475 51

497 498 241

497 526 182

497 528 60

497 532 47

497 542 30

497 646 29

497 679 33

497 680 34

497 701 238

497 704 45

497 790 59

497 802 64

497 903 68

497 1032 38

497 1034 137

497 1045 31

497 1392 33

497 1451 41

497 1613 29

497 1659 28

497 1934 50

497 2020 142

497 2083 33

497 2094 53

497 7461 37

498 55 743

498 67 3877

498 71 465

498 77 1624

498 89 1742

498 157 1481

498 180 1329

498 184 555

498 239 997

498 241 325

498 251 2505

498 257 28980

498 262 1279

498 279 984

498 285 1074

498 288 3314

498 289 6182

498 291 2311

498 292 2435

498 294 413

498 295 5836

498 299 2102

498 300 976

498 301 2390

498 302 527

498 306 1707

498 310 1441

498 311 755

498 314 638

498 317 337

498 328 323

498 329 1007

498 330 318

498 331 1841

498 332 610

498 349 1292

498 378 462

498 468 490

498 472 630

498 484 615

498 485 534

498 525 1052

498 701 392

498 907 829

498 913 1381

498 1052 312

498 2220 445

498 2222 325

498 3749 423

498 7462 703

499 7 11

499 51 7

499 55 2

499 67 3

499 72 177

499 157 27

499 172 2

499 173 8

499 198 1

499 233 18

499 234 2

499 436 4

499 518 4

499 522 14

499 525 1

499 706 5

499 726 1

499 732 1

499 843 1

499 854 30

499 856 1

499 863 1

499 868 5

499 918 1

499 936 5

499 1001 1

499 1034 74

499 1044 5

499 1098 1

499 1104 2

499 1114 8

499 1263 3

499 1358 3

499 1360 1

499 1412 31

499 1527 1

499 1703	2
499 1713	1
499 1714	25
499 1803	2
499 1817	2
499 1892	1
499 2343	4
499 3110	1
499 4349	1
499 4460	1
499 5079	10
499 5672	1
499 7463	13
499 7464	4
500 603 837	
500 616 76	
500 744 1140	
500 748 1991	
500 749 186	
500 757 103	
500 1222	52
500 1801	56
500 2146	432
500 2673	48
500 2688	14
500 2944	11
500 3521	61
500 5891	148
500 6189	272
500 7373	19

500 7465	478
500 7466	333
500 7467	109
500 7468	92
500 7469	88
500 7470	84
500 7471	70
500 7472	66
500 7473	64
500 7474	64
500 7475	54
500 7476	52
500 7477	52
500 7478	51
500 7479	45
500 7480	42
500 7481	41
500 7482	41
500 7483	33
500 7484	21
500 7485	17
500 7486	16
500 7487	15
500 7488	15
500 7489	14
500 7490	12
500 7491	12
500 7492	11
500 7493	10
500 7494	10

500 7495 10

500 7496 10

500 7497 9

500 7498 9

501 55 550

501 67 284

501 89 142

501 184 206

501 257 363

501 283 121

501 288 273

501 289 9541

501 290 98

501 291 183

501 292 467

501 294 357

501 295 275

501 299 166

501 301 149

501 302 149

501 305 251

501 318 412

501 321 138

501 325 337

501 333 1040

501 349 225

501 374 203

501 375 936

501 378 103

501 461 401

501	523	129
501	525	84
501	646	124
501	681	169
501	701	364
501	786	124
501	972	174
501	1045	101
501	1047	143
501	1052	83
501	1556	303
501	2082	239
501	2094	228
501	2102	433
501	2523	193
501	2801	253
501	3081	209
501	3171	105
501	3324	578
501	3332	367
501	4350	141
501	5745	187
501	7499	127
501	7500	110
502	55	593
502	89	717
502	97	658
502	157	300
502	161	356
502	191	241

502 251 284

502 257 795

502 265 946

502 288 1759

502 289 2215

502 291 2172

502 292 2103

502 294 1756

502 295 1502

502 296 1223

502 298 406

502 299 384

502 300 568

502 302 982

502 306 372

502 323 1211

502 326 421

502 340 254

502 349 314

502 352 607

502 378 381

502 466 399

502 528 332

502 543 241

502 544 1464

502 679 983

502 798 517

502 903 272

502 906 3654

502 915 310

502 1032 429

502 1037 239

502 1601 408

502 1602 327

502 1934 564

502 1936 1084

502 2021 331

502 2022 377

502 2523 1627

502 2939 426

502 3319 253

502 5533 610

502 6528 370

502 7501 424

503 55 1086

503 65 53

503 67 1036

503 89 432

503 157 70

503 161 69

503 182 1096

503 229 515

503 257 195

503 288 188

503 289 15235

503 292 852

503 294 57

503 295 460

503 298 66

503 299 121

503 300 300

503 306 137

503 318 705

503 324 47

503 325 109

503 349 91

503 352 1067

503 418 81

503 476 63

503 479 72

503 525 120

503 646 346

503 679 1499

503 689 425

503 691 448

503 701 82

503 802 54

503 972 75

503 1243 139

503 1246 73

503 1375 89

503 1377 229

503 1409 127

503 1455 54

503 1580 114

503 1705 80

503 2020 125

503 2130 626

503 2176 120

503 2607 480

503 2977 323

503 5533 232

503 7502 657

503 7503 70

504 7 128

504 45 106

504 89 161

504 163 214

504 192 166

504 209 840

504 234 120

504 288 94

504 289 1419

504 300 873

504 306 129

504 324 476

504 333 365

504 344 458

504 412 97

504 466 111

504 470 209

504 475 279

504 487 180

504 498 102

504 504 133

504 540 199

504 544 265

504 679 237

504 681 192

504 686 129

504 687 411

504 691 154

504 693 170

504 793 119

504 1037 114

504 1038 203

504 1040 126

504 1194 106

504 1239 219

504 1246 110

504 1455 199

504 1678 435

504 1680 259

504 1684 297

504 1803 203

504 2346 138

504 2521 164

504 3057 283

504 3712 179

504 4104 281

504 5067 199

504 5948 115

504 7504 106

504 7505 100

506 9 239

506 10 120

506 34 81

506 38 62

506 49 185

506 172 80

506 173 94

506 190 180

506 207 295

506 225 115

506 229 125

506 233 566

506 423 62

506 450 86

506 486 127

506 546 73

506 880 61

506 1066 71

506 1075 521

506 1099 61

506 1294 93

506 1332 2064

506 1376 138

506 1400 83

506 1497 89

506 1731 62

506 1983 147

506 2166 58

506 3109 110

506 3413 80

506 5581 96

506 6008 285

506 6054 64

506 6847 111

506 6875 146

506 6878 233

506 6881	127
506 6920	224
506 7511	806
506 7512	266
506 7513	239
506 7514	216
506 7515	193
506 7516	132
506 7517	114
506 7518	91
506 7519	91
506 7520	85
506 7521	61
506 7522	58
507 207 263	
507 227 588	
507 429 628	
507 533 268	
507 857 697	
507 1976	377
507 2029	907
507 3382	309
507 3892	247
507 3905	486
507 5235	1031
507 5236	260
507 5237	937
507 5238	288
507 5247	348
507 5645	270

507 5789	393
507 7523	838
507 7524	679
507 7525	678
507 7526	643
507 7527	584
507 7528	570
507 7529	546
507 7530	513
507 7531	506
507 7532	478
507 7533	467
507 7534	452
507 7535	452
507 7536	448
507 7537	407
507 7538	361
507 7539	335
507 7540	322
507 7541	303
507 7542	295
507 7543	292
507 7544	291
507 7545	288
507 7546	288
507 7547	276
507 7548	272
507 7549	264
507 7550	258
507 7551	257

507 7552	253
507 7553	246
507 7554	238
507 7555	234
508 1136	15
508 4062	64
508 7556	74
508 7557	70
508 7558	62
508 7559	60
508 7560	51
508 7561	48
508 7562	42
508 7563	42
508 7564	33
508 7565	31
508 7566	29
508 7567	28
508 7568	25
508 7569	25
508 7570	25
508 7571	25
508 7572	24
508 7573	21
508 7574	21
508 7575	20
508 7576	19
508 7577	19
508 7578	18
508 7579	18

508 7580	18
508 7581	16
508 7582	16
508 7583	15
508 7584	15
508 7585	15
508 7586	14
508 7587	14
508 7588	14
508 7589	13
508 7590	12
508 7591	12
508 7592	12
508 7593	12
508 7594	11
508 7595	11
508 7596	11
508 7597	11
508 7598	11
508 7599	10
508 7600	10
508 7601	10
508 7602	10
508 7603	9

509 190 28

509 220 1

509 306 3

509 377 6

509 378 8

509 472 41

509 475 8

509 486 9

509 488 75

509 493 12

509 497 48

509 498 12

509 503 3

509 504 59

509 507 2

509 536 19

509 540 14

509 545 4

509 562 1

509 663 2

509 676 4

509 681 4

509 718 1

509 806 6

509 808 66

509 830 25

509 930 7

509 966 114

509 986 1

509 1180 35

509 1351 19

509 1378 2

509 1672 16

509 1854 11

509 1905 177

509 2352 91

509 2661 9

509 2664 2

509 3859 10

509 4995 10

509 6257 2

509 7604 13

509 7605 8

509 7606 8

509 7607 7

509 7608 7

509 7609 3

509 7610 3

509 7611 3

509 7612 1

510 4 1

510 55 3

510 285 77

510 288 45

510 295 1

510 314 9

510 377 30

510 474 15

510 486 190

510 680 54

510 754 8

510 903 13

510 907 28

510 920 19

510 1167 1

510 1941 2

510 3462 7

510 4110 1

510 4988 1

510 5236 39

510 5565 11

510 7160 2

510 7613 49

510 7614 44

510 7615 30

510 7616 21

510 7617 19

510 7618 17

510 7619 12

510 7620 12

510 7621 10

510 7622 6

510 7623 2

510 7624 2

510 7625 1

510 7626 1

510 7627 1

510 7628 1

510 7629 1

510 7630 1

510 7631 1

510 7632 1

510 7633 1

511 65 84

511 154 802

511 163 54

511 199 347

511 206 28

511 226 281

511 228 71

511 229 28

511 234 281

511 237 64

511 424 260

511 436 359

511 440 53

511 441 9

511 511 37

511 533 22

511 562 40

511 612 28

511 614 96

511 709 72

511 808 73

511 854 7

511 856 110

511 857 25

511 859 80

511 863 17

511 864 8

511 868 8

511 881 9

511 964 17

511 982 52

511 1106 23

511 1131 17

511 1187 30

511 1400 25

511 1412 13

511 1504 85

511 1633 79

511 1705 43

511 1873 27

511 1974 110

511 1976 62

511 2268 6

511 2542 127

511 2574 23

511 2821 38

511 3700 9

511 4079 294

511 6187 109

511 6540 15

512 8 948

512 45 887

512 88 809

512 189 558

512 371 431

512 382 422

512 407 2673

512 472 981

512 490 908

512 503 1903

512 504 3105

512 516 1106

512 517 3190

512 519 1242

512 707 501

512 754 1468

512 785 669

512 808 1130

512 810 567

512 812 542

512 837 460

512 966 769

512 978 679

512 1044 1118

512 1145 1031

512 1179 477

512 1339 721

512 1410 530

512 1672 1153

512 1854 1931

512 1873 711

512 2143 690

512 2366 583

512 2944 1886

512 2953 744

512 2961 2146

512 3084 461

512 3196 447

512 3244 550

512 3563 1369

512 4170 1664

512 4833 554

512 5310 489

512 7634 1056
512 7635 798
512 7636 762
512 7637 530
512 7638 517
512 7639 499
512 7640 421
513 64 336
513 65 314
513 126 245
513 207 281
513 212 391
513 222 377
513 223 1201
513 225 618
513 226 876
513 228 650
513 229 263
513 430 431
513 486 245
513 604 237
513 614 293
513 618 568
513 703 287
513 982 429
513 1048 399
513 1093 835
513 1119 271
513 1351 255
513 1400 468

513 1510 348

513 1512 265

513 1609 290

513 1633 276

513 1958 329

513 1988 295

513 2524 348

513 2608 319

513 2708 242

513 2823 275

513 2824 276

513 3057 315

513 3253 511

513 7641 1304

513 7642 1155

513 7643 1012

513 7644 942

513 7645 877

513 7646 350

513 7647 348

513 7648 334

513 7649 310

513 7650 260

513 7651 251

513 7652 243

513 7653 242

513 7654 240

514 67 4602

514 89 1433

514 209 384

514 288 1036

514 291 158

514 292 176133

514 293 1482

514 299 238

514 300 895

514 302 869

514 318 215

514 321 663

514 325 1091

514 333 378

514 340 246

514 344 843

514 349 176

514 461 109889

514 466 409

514 470 267

514 471 260

514 486 1290

514 523 1595

514 544 3148

514 674 225

514 681 4542

514 686 343

514 701 697

514 704 1253

514 903 908

514 913 146

514 972 142

514 1463 363

514 1556 236

514 1580 1064

514 1590 569

514 1596 242

514 1782 365

514 2521 475

514 3196 184

514 3266 552

514 3375 884

514 3570 674

514 7391 1077

514 7655 3907

514 7656 1576

514 7657 1046

514 7658 296

514 7659 162

514 7660 154

515 51 274

515 62 1463

515 84 179

515 88 120

515 154 204

515 203 250

515 207 234

515 227 243

515 228 285

515 331 247

515 424 162

515 440 249

515 446 142

515 533 148

515 1106 312

515 1187 198

515 1239 117

515 1242 264

515 1390 226

515 1627 125

515 1721 145

515 1881 145

515 1943 141

515 1950 199

515 2532 113

515 2627 179

515 2639 139

515 3258 120

515 3691 120

515 4079 116

515 4616 118

515 5428 123

515 6297 123

515 6785 117

515 7661 458

515 7662 286

515 7663 223

515 7664 190

515 7665 177

515 7666 177

515 7667 172

515 7668 169

515 7669 166

515 7670 140

515 7671 128

515 7672 121

515 7673 119

515 7674 117

515 7675 116

515 7676 115

516 7 13270

516 39 5194

516 72 1904

516 89 572

516 172 2062

516 173 805

516 227 356

516 292 363

516 300 1144

516 378 383

516 412 239

516 481 1818

516 487 4151

516 497 4314

516 518 230

516 522 1418

516 524 2346

516 544 343

516 718 330

516 724 406

516 816 2190

516 903 1035

516 917 252

516 930 545

516 972 280

516 986 395

516 1034 203

516 1083 367

516 1091 572

516 1194 194

516 1206 276

516 1244 13781

516 1672 306

516 1673 24612

516 1708 650

516 2184 174

516 3189 169

516 3244 715

516 3328 164

516 3367 1431

516 3431 159

516 3896 185

516 5079 2287

516 5102 245

516 7464 271

516 7677 1458

516 7678 838

516 7679 545

516 7680 512

516 7681 340

517 107 247

517 121 149

517 154 346

517	196	288
517	231	187
517	434	184
517	604	176
517	629	133
517	703	219
517	981	254
517	1106	191
517	1155	207
517	1167	168
517	1424	139
517	1627	243
517	1717	240
517	1943	256
517	1946	330
517	2277	133
517	2292	137
517	2408	145
517	2535	242
517	2605	129
517	3026	280
517	3158	176
517	3693	155
517	4032	133
517	5925	198
517	6004	180
517	6915	164
517	7682	206
517	7683	203
517	7684	202

517 7685 194
517 7686 190
517 7687 182
517 7688 179
517 7689 170
517 7690 164
517 7691 162
517 7692 152
517 7693 140
517 7694 140
517 7695 139
517 7696 139
517 7697 135
517 7698 134
517 7699 132
517 7700 129
517 7701 129
518 55 548
518 67 4087
518 89 2794
518 257 46
518 265 51
518 288 1419
518 289 3080
518 292 1666
518 295 4180
518 298 49
518 300 634
518 306 59
518 320 68

518 321 153

518 333 275

518 344 349

518 349 155

518 378 45

518 461 1068

518 466 159

518 475 89

518 498 156

518 523 98

518 524 145

518 531 71

518 543 130

518 679 127

518 686 239

518 701 195

518 790 191

518 793 199

518 797 76

518 802 657

518 903 294

518 908 123

518 913 163

518 1032 48

518 1043 49

518 1054 274

518 1447 69

518 1659 49

518 2080 46

518 4350 56

518 5749 448

518 6646 343

518 7702 78

518 7703 64

518 7704 56

518 7705 49

518 7706 44

519 13 205

519 72 691

519 183 278

519 198 1701

519 234 274

519 370 835

519 377 336

519 487 243

519 490 220

519 706 1028

519 807 206

519 917 8130

519 918 7200

519 920 1254

519 922 511

519 928 1430

519 936 445

519 940 1870

519 944 215

519 946 231

519 954 428

519 959 259

519 1113 1345

519	1339	727
519	1344	436
519	1358	1534
519	1406	199
519	1788	931
519	1797	297
519	1803	424
519	1913	487
519	2004	263
519	2014	559
519	2342	802
519	2797	397
519	2972	750
519	3425	427
519	3739	332
519	3946	288
519	4342	320
519	4670	505
519	5115	240
519	5461	292
519	7707	4400
519	7708	1017
519	7709	619
519	7710	312
519	7711	310
519	7712	283
519	7713	231
520	30	1525
520	55	126
520	64	155

520	72	23304
520	89	202
520	159	354
520	181	4795
520	187	1298
520	189	344
520	190	365
520	198	1125
520	221	107
520	238	114
520	490	169
520	707	117
520	880	4635
520	1061	2642
520	1072	735
520	1406	209
520	1705	337
520	1713	2425
520	1817	607
520	1886	115
520	1913	1587
520	1914	398
520	2735	143
520	2835	317
520	2858	112
520	3045	88
520	3367	255
520	3557	256
520	3945	956
520	4295	80

520 4308 980

520 5215 77

520 6263 187

520 6265 535

520 6267 164

520 7429 804

520 7514 160

520 7516 278

520 7714 1494

520 7715 615

520 7716 454

520 7717 379

520 7718 308

520 7719 121

520 7720 101

520 7721 97

520 7722 87

521 89 512

521 204 167

521 207 659

521 209 210

521 226 148

521 230 290

521 300 181

521 333 340

521 377 578

521 403 3615

521 424 302

521 430 1291

521 486 799

521 492 143

521 498 9738

521 499 1679

521 536 1020

521 537 1085

521 687 369

521 840 1016

521 982 488

521 1043 426

521 1105 142

521 1183 228

521 1186 7098

521 1198 639

521 1200 166

521 1246 322

521 1541 779

521 1866 2381

521 2344 314

521 2619 720

521 2840 4708

521 2850 2146

521 3097 252

521 3373 596

521 3946 288

521 4170 676

521 4375 351

521 4434 163

521 4820 308

521 4821 2180

521 4834 436

521 4837	1664
521 6127	348
521 6196	1787
521 7329	764
521 7723	907
521 7724	270
521 7725	222
523 163 1	
523 207 2	
523 210 2	
523 225 1	
523 227 3	
523 424 1	
523 425 2	
523 443 2	
523 448 2	
523 554 1	
523 618 1	
523 735 1	
523 868 2	
523 898 3	
523 1376	3
523 1426	4
523 1427	1
523 1516	2
523 1883	1
523 1954	2
523 2265	3
523 2538	3
523 2600	4

523 2601	4
523 2815	2
523 2855	2
523 2899	1
523 3030	3
523 3732	1
523 4493	1
523 5844	2
523 7735	6
523 7736	6
523 7737	4
523 7738	3
523 7739	3
523 7740	2
523 7741	2
523 7742	2
523 7743	2
523 7744	2
523 7745	2
523 7746	2
523 7747	2
523 7748	2
523 7749	2
523 7750	2
523 7751	2
523 7752	1
523 7753	1
525 209 798	
525 230 806	
525 403 870	

525 481 1218

525 488 1103

525 490 332

525 493 587

525 497 707

525 498 719

525 500 1601

525 536 733

525 537 448

525 540 637

525 687 563

525 768 320

525 808 1865

525 816 3364

525 917 540

525 978 546

525 982 333

525 1038 390

525 1179 917

525 1180 2949

525 1181 2840

525 1182 801

525 1184 516

525 1185 746

525 1188 562

525 1190 860

525 1193 3015

525 1195 1344

525 1246 475

525 1249 786

525 1380 316

525 1672 1032

525 1853 533

525 1854 1609

525 1868 461

525 2618 590

525 2619 439

525 2794 479

525 2843 385

525 2847 576

525 2959 1843

525 3084 492

525 3427 739

525 4104 515

525 7775 708

525 7776 501

525 7777 335

526 7 16

526 198 361

526 220 607

526 366 8

526 383 5

526 412 456

526 424 2

526 464 110

526 488 43

526 517 10

526 527 5

526 536 72

526 706 135

526 707 126

526 724 28

526 732 11

526 816 661

526 830 67

526 843 33

526 920 15

526 979 1

526 982 22

526 1041 2

526 1043 4

526 1044 169

526 1046 97

526 1110 2

526 1186 1

526 1192 39

526 1196 2

526 1249 269

526 1369 2

526 1412 514

526 1639 3

526 1672 501

526 1681 20

526 2226 2

526 2352 45

526 2595 44

526 2789 15

526 2791 1

526 2835 3

526 3316 7

526 3670 17

526 4850 92

526 7331 32

526 7778 4

526 7779 3

526 7780 1

527 55 227

527 67 271

527 89 12679

527 265 113

527 288 810

527 289 1113

527 292 546

527 295 166

527 299 123

527 300 119

527 302 103

527 331 216

527 400 648

527 461 235

527 466 599

527 485 737

527 528 196

527 530 113

527 543 312

527 544 569

527 548 449

527 680 505

527 689 228

527 690 1288

527 697 342

527 704 173

527 896 100

527 903 254

527 959 312

527 1034 96

527 1037 2376

527 1379 147

527 1452 83

527 1934 580

527 2025 92

527 2091 274

527 2094 834

527 2098 165

527 2102 1645

527 2521 105

527 2543 7024

527 3404 142

527 3502 168

527 4704 243

527 4814 853

527 5150 127

527 5460 167

527 7349 248

527 7781 490

527 7782 87

529 163 365

529 607 341

529 707 331

529 917 264

529 959 445	
529 1073	311
529 1110	1415
529 1358	451
529 1369	674
529 1411	360
529 1413	1724
529 1468	519
529 1527	349
529 2463	329
529 2569	605
529 3264	258
529 3481	1005
529 3774	333
529 4124	343
529 4675	615
529 4784	328
529 4790	506
529 5297	697
529 5301	432
529 5901	388
529 5902	276
529 6172	257
529 6264	313
529 6959	264
529 7047	310
529 7057	423
529 7791	728
529 7792	617
529 7793	498

529 7794 457
529 7795 421
529 7796 387
529 7797 377
529 7798 366
529 7799 346
529 7800 338
529 7801 336
529 7802 319
529 7803 314
529 7804 307
529 7805 297
529 7806 292
529 7807 279
529 7808 276
529 7809 273
530 59 39
530 72 48
530 81 90
530 84 42
530 182 36
530 228 21
530 416 48
530 433 18
530 526 20
530 548 26
530 689 42
530 703 67
530 722 26
530 1106 34

530 1553	58
530 1763	22
530 1776	28
530 1790	56
530 2534	22
530 2616	19
530 3164	45
530 3391	19
530 3992	49
530 4717	37
530 5184	50
530 5412	20
530 6217	33
530 7007	19
530 7324	23
530 7452	27
530 7810	97
530 7811	53
530 7812	49
530 7813	44
530 7814	42
530 7815	37
530 7816	32
530 7817	31
530 7818	30
530 7819	25
530 7820	24
530 7821	23
530 7822	23
530 7823	22

530 7824 22

530 7825 21

530 7826 21

530 7827 20

530 7828 20

530 7829 19

531 55 206

531 67 291

531 89 553

531 288 877

531 289 2746

531 291 1126

531 292 1067

531 293 461

531 295 564

531 298 603

531 299 2518

531 300 645

531 301 1379

531 302 771

531 306 255

531 307 249

531 320 329

531 324 1009

531 325 3864

531 333 833

531 342 181

531 344 1462

531 378 220

531 458 190

531 461 1349

531 464 366

531 466 1049

531 498 327

531 523 426

531 524 238

531 525 180

531 528 220

531 543 232

531 679 1134

531 681 357

531 686 1732

531 701 335

531 704 355

531 797 188

531 908 578

531 1042 246

531 1047 203

531 1246 386

531 1458 735

531 1679 266

531 1934 236

531 2020 13006

531 2543 185

531 5149 247

531 7830 241

532 207 141

532 220 71

532 227 84

532 412 445

532 464 92

532 470 174

532 471 193

532 498 93

532 540 85

532 687 1451

532 693 92

532 779 106

532 816 1126

532 1036 195

532 1038 306

532 1040 149

532 1041 200

532 1045 541

532 1048 201

532 1180 782

532 1183 212

532 1185 147

532 1188 240

532 1192 173

532 1193 195

532 1198 193

532 1201 112

532 1246 147

532 1659 71

532 1670 127

532 1672 145

532 1853 133

532 2426 100

532 2608 163

532 2617 100

532 2618 84

532 2619 79

532 2653 183

532 3398 91

532 4166 104

532 4828 125

532 4995 78

532 5950 97

532 5958 77

532 6614 74

532 7831 256

532 7832 110

532 7833 95

532 7834 86

532 7835 85

533 67 116

533 161 279

533 180 215

533 192 221

533 258 118

533 265 121

533 340 361

533 641 387

533 727 178

533 1464 150

533 1494 522

533 2023 484

533 2044 7652

533 2046 126

533 2051	425
533 2059	342
533 2075	199
533 2394	366
533 2395	1667
533 2396	775
533 2441	166
533 3062	217
533 3063	137
533 3307	252
533 4253	123
533 5787	135
533 6522	464
533 6860	313
533 7506	135
533 7836	1949
533 7837	686
533 7838	434
533 7839	276
533 7840	214
533 7841	203
533 7842	152
533 7843	151
533 7844	137
533 7845	135
533 7846	128
533 7847	127
533 7848	123
533 7849	117
533 7850	108

533 7851 105
533 7852 94
533 7853 91
533 7854 90
533 7855 88
533 7856 86
534 7 932
534 63 247
534 67 714
534 70 241
534 72 2863
534 89 397
534 154 116
534 155 57
534 161 181
534 191 39
534 288 1069
534 289 1068
534 292 1015
534 294 189
534 295 808
534 296 59
534 298 122
534 299 328
534 300 91
534 306 616
534 308 105
534 316 132
534 320 85
534 328 590

534 329 131

534 330 43

534 331 428

534 349 155

534 352 186

534 405 545

534 466 49

534 468 39

534 475 712

534 535 141

534 543 166

534 679 792

534 701 601

534 907 92

534 1061 1143

534 1072 172

534 1099 39

534 1379 309

534 1449 78

534 1451 55

534 1713 223

534 1795 103

534 2500 271

534 2595 44

534 2634 56

534 3011 52

535 55 211

535 89 202

535 180 140

535 253 531

535 257 170

535 289 314

535 292 739

535 302 150

535 310 138

535 328 139

535 342 213

535 355 333

535 378 286

535 455 1799

535 472 146

535 488 269

535 511 132

535 518 149

535 544 148

535 683 277

535 701 198

535 708 354

535 951 483

535 966 422

535 1241 210

535 1247 354

535 1249 480

535 1459 1394

535 1470 225

535 1556 228

535 1698 338

535 2020 175

535 2067 208

535 2346 358

535 2392 169
535 2977 227
535 2986 151
535 3074 1204
535 3081 255
535 3097 1718
535 3376 130
535 3856 175
535 4708 353
535 4850 153
535 5441 304
535 7321 367
535 7857 230
535 7858 165
535 7859 146
535 7860 136
536 67 69
536 89 206
536 157 154
536 161 76
536 163 79
536 184 35
536 192 47
536 220 33
536 227 235
536 288 138
536 289 1310
536 292 1086
536 299 46
536 300 125

536 301 75

536 329 168

536 333 113

536 455 72

536 459 104

536 468 48

536 511 157

536 533 239

536 591 58

536 614 40

536 679 110

536 683 406

536 701 157

536 733 72

536 792 79

536 802 121

536 959 44

536 1042 79

536 1414 165

536 1429 37

536 1447 36

536 1475 48

536 1755 279

536 1934 194

536 2031 37

536 2044 274

536 2047 33

536 2176 98

536 2440 36

536 2521 86

536 3063 38

536 3071 49

536 3171 44

536 5249 98

536 7861 56

536 7862 47

537 88 37

537 89 113

537 220 53

537 234 64

537 257 58

537 288 516

537 289 426

537 292 303

537 300 144

537 301 29

537 306 53

537 323 26

537 325 97

537 344 42

537 400 126

537 461 209

537 464 222

537 466 149

537 498 44

537 539 48

537 544 41

537 614 31

537 679 1323

537 701 116

537 790 499

537 802 802

537 860 36

537 955 80

537 982 67

537 1034 230

537 1037 36

537 1114 130

537 1428 36

537 1447 22

537 1458 148

537 1673 107

537 1873 34

537 2091 104

537 2092 140

537 2094 237

537 2098 21

537 2543 25

537 2799 143

537 3124 36

537 4558 143

537 7863 85

537 7864 36

537 7865 27

537 7866 26

537 7867 24

538 55 120

538 89 1922

538 288 2327

538 289 2156

538 291 98

538 292 1191

538 293 93

538 294 162

538 295 363

538 299 620

538 300 1386

538 301 231

538 302 305

538 306 470

538 310 90

538 323 173

538 328 137

538 330 181

538 333 1001

538 344 2628

538 346 337

538 352 484

538 461 856

538 466 1215

538 468 130

538 475 193

538 531 111

538 543 481

538 545 100

538 679 115

538 686 172

538 690 185

538 701 288

538 907 105

538 915 88

538 1032 197

538 1037 97

538 1042 270

538 1051 120

538 1452 97

538 1455 349

538 1456 178

538 1459 94

538 1934 184

538 2020 379

538 4565 116

538 5267 119

538 5534 151

538 7868 109

538 7869 103

539 51 42

539 65 25

539 67 23

539 70 39

539 72 1384

539 75 35

539 89 55

539 99 144

539 153 185

539 159 27

539 165 22

539 166 40

539 182 95

539 183 53

539 190 54

539 209 108

539 233 1609

539 265 20

539 288 90

539 295 43

539 377 99

539 389 40

539 436 99

539 455 28

539 486 1439

539 491 41

539 493 54

539 511 805

539 599 130

539 718 57

539 961 24

539 1001 286

539 1061 48

539 1072 43

539 1079 28

539 1099 35

539 1104 126

539 1210 240

539 1379 23

539 1713 438

539 1787 34

539 3920 107

539 5000 57

539 5532 22

539 7870 137

539 7871 110

539 7872 86

539 7873 34

539 7874 32

539 7875 24

540 51 4753

540 53 1914

540 58 2929

540 59 2274

540 67 1511

540 72 4129

540 82 1802

540 84 1795

540 98 3184

540 159 3838

540 173 1339

540 225 1502

540 423 2485

540 429 1536

540 599 4890

540 998 3319

540 999 1955

540 1001 2964

540 1013 1752

540 1014 1572

540 1075 2214

540 1083 4824

540 1097 2456

540 1167 1214

540 1213 1170

540 1694 3040

540 1707 2683

540 1844 1965

540 1892 1570

540 1895 1815

540 2531 1478

540 2938 3310

540 3259 2716

540 3279 1399

540 3280 24958

540 3286 1594

540 3295 1318

540 3411 1555

540 3768 1237

540 3893 1477

540 3896 1601

540 4025 1404

540 4384 1317

540 5913 2055

540 6883 1192

540 7876 3815

540 7877 2981

540 7878 2014

540 7879 2013

540 7880 1334

541 52 894

541 152 4503

541 163 36310

541 186 3251

541 202 1784

541 212 1464

541 227 1474

541 562 8960

541 607 8362

541 614 11002

541 630 1668

541 706 2161

541 707 1891

541 712 1724

541 715 906

541 728 2692

541 735 1598

541 854 1342

541 863 854

541 868 2799

541 982 1064

541 1119 1141

541 1249 917

541 1369 1099

541 1372 1386

541 1411 1188

541 1412 22851

541 1415 1811

541 1425 3867

541 1639 2439

541 1643 1469

541 1778 1347

541 1806 6713

541 1813 2400

541 1873 1019

541 1974 968

541 2834 1809

541 2842 939

541 2855 1257

541 2898 2922

541 3620 4181

541 4349 1298

541 4675 3697

541 5172 2030

541 5175 8705

541 5416 1241

541 5441 1193

541 5844 3552

541 7881 3894

541 7882 2806

542 157 21

542 289 131733

542 544 512

542 669 175

542 679 792

542 2184 2

542 7864 1

543 58 1331

543 64 713

543 65 1249

543 70 992

543 72 910

543 89 924

543 154 1054

543 162 796

543 173 1443

543 174 554

543 182 1144

543 183 509

543 190 3884

543 199 1238

543 207 676

543 215 602

543 225 711

543 229 682

543 233 1919

543 303 433

543 389 673

543 418 774

543 423 662

543 425 418

543 453 1719

543 511 1455

543 537 597

543 683 1108

543 920 410

543 936 463

543 964 521

543 1062 648

543 1090 549

543 1098 484

543 1104 643

543 1116 601

543 1118 576

543 1151 468

543 1246 542

543 1376 631

543 1379 734

543 1381 470

543 1400 497

543 2006 541

543 3411 446

543 4600 468

543 7187 476

543 7883 699

543 7884 480

543 7885 472

544 65 249

544 84 470

544 170 693

544 210 276

544 217 364

544 403 380

544 418 1272

544 444 618

544 498 177

544 691 746

544 826 276

544 875 179

544 1046 249

544 1075 443

544 1385 484

544 1398 222

544 1400 459

544 1504	576
544 1736	254
544 2425	810
544 2580	191
544 3411	438
544 3414	169
544 3753	371
544 3769	260
544 3775	245
544 3817	206
544 4022	187
544 4174	189
544 4211	177
544 5547	223
544 5927	496
544 5950	379
544 6602	313
544 7071	271
544 7076	226
544 7194	241
544 7382	258
544 7886	365
544 7887	361
544 7888	341
544 7889	255
544 7890	244
544 7891	232
544 7892	211
544 7893	206
544 7894	200

544 7895 195

544 7896 192

544 7897 179

545 7 312

545 89 361

545 173 829

545 189 252

545 190 1009

545 191 880

545 204 340

545 209 13816

545 288 327

545 289 507

545 291 299

545 331 341

545 333 393

545 371 1017

545 377 1223

545 378 622

545 403 931

545 405 304

545 408 404

545 472 299

545 481 680

545 486 673

545 488 1057

545 490 239

545 497 268

545 498 421

545 518 491

545 537 1082

545 538 345

545 680 349

545 966 430

545 1091 495

545 1179 241

545 1186 281

545 1246 301

545 1377 375

545 1378 327

545 1470 518

545 1852 880

545 1854 365

545 1863 435

545 2941 650

545 2956 303

545 3094 316

545 3373 360

545 3859 1051

545 4177 287

545 6006 602

545 6693 246

545 7898 300

546 12 1746

546 23 1000

546 152 3775

546 163 2742

546 176 1460

546 188 8497

546 227 2449

546 533 1135

546 812 1581

546 841 922

546 917 2892

546 918 6423

546 923 12420

546 949 846

546 953 862

546 954 1054

546 1104 5786

546 1123 936

546 1131 2720

546 1358 2168

546 1369 6568

546 1643 2202

546 1710 1816

546 1712 2580

546 2006 2962

546 2151 1216

546 2469 1950

546 2576 2564

546 2586 1475

546 2654 1388

546 2746 2328

546 2772 970

546 2963 5657

546 3280 2723

546 3509 3649

546 3788 930

546 4163 1024

546 4669 1908

546 4971 3007

546 5157 2250

546 5605 1558

546 7713 1617

546 7899 2444

546 7900 2170

546 7901 1814

546 7902 1217

546 7903 1046

546 7904 964

546 7905 932

546 7906 892

547 163 138

547 226 141

547 227 165

547 233 103

547 424 85

547 436 131

547 562 86

547 599 154

547 614 89

547 615 148

547 617 139

547 619 75

547 709 143

547 854 132

547 856 129

547 857 125

547 858 146

547 881 121

547 917 106

547 918 130

547 959 128

547 964 74

547 1110 130

547 1119 112

547 1202 82

547 1369 88

547 1412 95

547 1416 80

547 1425 85

547 1510 73

547 1639 79

547 1709 155

547 1976 76

547 1979 77

547 2271 97

547 2821 87

547 3227 91

547 3364 94

547 3440 102

547 3705 134

547 3792 117

547 4123 88

547 6730 88

547 7407 125

547 7907 92

547 7908 82

547 7909 77

547 7910 76

547 7911 75

547 7912 74

548 3 37

548 6 221

548 12 57

548 20 60

548 23 39

548 28 48

548 47 41

548 48 47

548 188 210

548 383 359

548 707 159

548 841 82

548 918 244

548 947 58

548 949 89

548 1131 667

548 1214 122

548 1254 78

548 1258 59

548 1259 38

548 1276 305

548 1358 235

548 1710 349

548 2375 58

548 2761 164

548 2970 47

548 3438 100

548 3509 43

548 3660 39

548 3798 62

548 3806 132

548 4265 178

548 4266 45

548 4273 46

548 4280 59

548 4290 235

548 7913 387

548 7914 339

548 7915 251

548 7916 223

548 7917 211

548 7918 181

548 7919 162

548 7920 136

548 7921 127

548 7922 103

548 7923 82

548 7924 72

548 7925 47

548 7926 39

549 88 212

549 99 81

549 157 300

549 163 13309

549 176 86

549 198 151

549 203 680

549 227 657

549 389 74

549 536 76

549 572 80

549 585 81

549 707 526

549 724 148

549 868 499

549 917 170

549 959 169

549 1091 106

549 1249 1302

549 1415 66

549 1416 501

549 1469 854

549 2013 149

549 2136 11405

549 2265 115

549 2861 78

549 2899 92

549 2902 82

549 3230 1124

549 3471 421

549 4123 3493

549 4125 4578

549 4126 483

549 4605 97

549 6165 453

549 7054 472

549 7426 3111

549 7927 1985

549 7928 868

549 7929 786

549 7930 658

549 7931 613

549 7932 468

549 7933 226

549 7934 101

549 7935 92

549 7936 83

549 7937 75

549 7938 74

549 7939 72

550 188 173

550 198 283

550 200 386

550 226 247

550 614 185

550 706 162

550 707 1002

550 710 782

550 809 169

550 820 179

550 821 449

550 834 295

550 841 371

550 843 741

550 846 959

550 917 538

550 923 134

550 947 309	
550 952 134	
550 954 421	
550 1044	467
550 1104	419
550 1216	303
550 1257	432
550 1261	193
550 1274	505
550 1358	305
550 1360	220
550 1364	823
550 1369	291
550 1412	323
550 1522	221
550 1527	393
550 1528	296
550 2143	573
550 2342	315
550 2379	145
550 2744	436
550 2836	256
550 2838	580
550 2901	153
550 3719	747
550 4275	176
550 4949	149
550 5105	625
550 5666	298
550 7423	510

550 7924 162
550 7940 285
550 7941 201
551 65 19
551 89 77
551 190 106
551 209 22
551 228 23
551 229 99
551 288 42
551 295 39
551 300 20
551 306 15
551 356 86
551 468 35
551 486 90
551 498 16
551 500 81
551 501 71
551 512 62
551 602 20
551 808 42
551 966 44
551 989 15
551 1130 19
551 1181 25
551 1182 168
551 1189 247
551 1193 35
551 1200 28

551 1377	18
551 1613	15
551 1672	115
551 1854	208
551 2206	29
551 2637	14
551 3739	15
551 3769	33
551 4179	23
551 4262	16
551 4354	18
551 5475	17
551 5746	21
551 6389	22
551 7177	16
551 7260	28
551 7756	22
551 7942	125
551 7943	23
551 7944	16
551 7945	16
551 7946	14
551 7947	14
552 2153	17
552 5060	4
552 5063	3
552 5999	3
552 7948	18
552 7949	15
552 7950	15

552 7951	13
552 7952	13
552 7953	11
552 7954	10
552 7955	10
552 7956	10
552 7957	10
552 7958	9
552 7959	9
552 7960	7
552 7961	7
552 7962	6
552 7963	6
552 7964	6
552 7965	6
552 7966	5
552 7967	5
552 7968	5
552 7969	5
552 7970	5
552 7971	5
552 7972	5
552 7973	5
552 7974	5
552 7975	5
552 7976	4
552 7977	4
552 7978	4
552 7979	4
552 7980	4

552 7981 3

552 7982 3

552 7983 3

552 7984 3

552 7985 3

552 7986 3

552 7987 3

552 7988 3

552 7989 3

552 7990 3

552 7991 3

552 7992 3

552 7993 3

553 67 87

553 157 2310

553 161 684

553 265 72

553 288 38

553 289 1626

553 292 4486

553 295 39

553 300 56

553 306 38

553 340 44

553 346 144

553 349 107

553 478 293

553 535 48

553 644 47

553 660 84

553 908 38

553 913 34

553 1573 95

553 1576 148

553 1601 42

553 3053 40

553 3503 1399

553 4298 306

553 4688 38

553 5731 59

553 5767 94

553 6201 42

553 6245 62

553 7994 786

553 7995 567

553 7996 267

553 7997 252

553 7998 247

553 7999 217

553 8000 122

553 8001 84

553 8002 75

553 8003 72

553 8004 69

553 8005 57

553 8006 56

553 8007 56

553 8008 55

553 8009 48

555 89 238

555 161 252

555 251 111

555 288 226

555 289 396

555 290 83

555 292 91

555 295 327

555 296 220

555 298 102

555 300 122

555 302 90

555 314 205

555 323 343

555 332 174

555 333 99

555 350 193

555 386 94

555 464 95

555 530 123

555 676 89

555 679 294

555 906 101

555 913 76

555 915 111

555 1395 121

555 1449 88

555 1596 83

555 2018 149

555 2455 85

555 3491 486

555 3503 82

555 3557 128

555 3948 135

555 4705 121

555 4770 155

555 6232 208

555 6313 91

555 6429 184

555 6690 84

555 7340 233

555 7395 85

555 8017 431

555 8018 234

555 8019 148

555 8020 127

555 8021 122

555 8022 107

555 8023 99

555 8024 76

556 56 1

556 65 224

556 173 9

556 184 79

556 191 246

556 199 6

556 214 7

556 389 1

556 416 21

556 418 1088

556 444 18

556 454 1043

556 511 9

556 632 5

556 691 11

556 1009 58

556 1243 7

556 1375 222

556 1401 2

556 1700 6

556 1705 31

556 1731 61

556 2582 28

556 2840 16

556 3411 4

556 3413 15

556 3427 2

556 4553 5

556 5099 7

556 5547 21

556 5806 141

556 6229 128

556 6299 42

556 8025 90

556 8026 67

556 8027 59

556 8028 55

556 8029 50

556 8030 45

556 8031 38

556 8032 12

556 8033 10

556 8034 6

556 8035 4

556 8036 4

556 8037 4

556 8038 3

556 8039 3

556 8040 3

556 8041 2

557 5 260

557 17 197

557 18 720

557 32 164

557 34 164

557 72 203

557 156 635

557 159 1240

557 173 1045

557 181 813

557 450 345

557 487 424

557 498 332

557 506 573

557 518 384

557 614 647

557 871 574

557 1066 439

557 1292 323

557 1295 591

557 1298 201

557 1300	546
557 1301	474
557 1303	528
557 1534	256
557 1706	197
557 1707	251
557 1914	263
557 2013	163
557 2014	404
557 2465	171
557 3195	505
557 3556	288
557 3558	288
557 4059	253
557 4874	192
557 5073	362
557 5079	200
557 5358	170
557 6548	188
557 7775	186
557 8042	755
557 8043	733
557 8044	618
557 8045	480
557 8046	402
557 8047	400
557 8048	342
557 8049	271
557 8050	163
558 7	43

558 67 21730

558 157 51

558 251 2

558 340 114

558 535 5

558 701 10

558 889 45

558 2789 34

558 3304 2

558 8051 6

558 8052 1

559 70 244

559 88 269

559 163 24

559 166 25

559 190 23

559 208 241

559 220 1378

559 227 385

559 428 323

559 511 60

559 599 5385

559 706 21

559 733 54

559 735 63

559 863 66

559 959 52

559 969 27

559 985 61

559 1103 94

559 1122	46
559 1242	23
559 1372	17
559 1414	421
559 1416	40
559 1740	90
559 1804	60
559 1811	41
559 1889	18
559 2265	63
559 2742	22
559 3011	103
559 3071	25
559 3371	25
559 4541	89
559 4795	27
559 5693	112
559 5844	21
559 6724	81
559 8053	179
559 8054	152
559 8055	117
559 8056	106
559 8057	87
559 8058	84
559 8059	72
559 8060	71
559 8061	43
559 8062	29
559 8063	20

559 8064 18

560 2 134

560 7 213

560 9 866

560 56 81

560 59 111

560 72 723

560 159 599

560 181 72

560 187 65

560 203 113

560 204 115

560 227 799

560 370 195

560 429 127

560 432 214

560 448 84

560 512 1352

560 533 118

560 605 176

560 709 65

560 716 408

560 718 68

560 858 312

560 997 87

560 998 74

560 1061 330

560 1083 613

560 1098 246

560 1099 121

560 1104 333

560 1155 105

560 1394 70

560 1414 98

560 1707 112

560 1814 72

560 2531 123

560 2708 122

560 3280 137

560 3291 114

560 3767 86

560 4387 142

560 4994 93

560 7187 125

560 7639 89

560 8065 632

560 8066 328

560 8067 137

560 8068 95

560 8069 90

560 8070 77

561 239 3446

561 262 2390

561 267 3531

561 279 3899

561 309 1923

561 312 4172

561 314 1100

561 317 1840

561 322 4386

561 722 1652

561 1457	890
561 1622	1550
561 2220	918
561 2922	1548
561 4566	13061
561 4580	930
561 4696	2413
561 4713	1079
561 4980	1068
561 6385	1015
561 6566	2142
561 6903	1747
561 7766	890
561 8071	7009
561 8072	5011
561 8073	3893
561 8074	3372
561 8075	3360
561 8076	2862
561 8077	2421
561 8078	2200
561 8079	2025
561 8080	1997
561 8081	1974
561 8082	1899
561 8083	1579
561 8084	1413
561 8085	1392
561 8086	1391

561 8087 1377

561 8088 1233

561 8089 1189

561 8090 1064

561 8091 1055

561 8092 1032

561 8093 1030

561 8094 984

561 8095 937

561 8096 877

561 8097 874

562 199 206

562 257 241

562 288 212

562 292 411

562 302 255

562 344 473

562 356 239

562 360 324

562 364 422

562 511 341

562 712 271

562 718 286

562 734 213

562 891 336

562 892 1388

562 1934 338

562 2051 299

562 2392 315

562 2436 325

562 2437	519
562 2442	308
562 2443	199
562 2445	260
562 2449	213
562 2453	241
562 2455	253
562 2456	422
562 2467	347
562 2468	649
562 3460	809
562 3584	296
562 3585	333
562 3588	310
562 3589	323
562 3603	452
562 3765	280
562 5150	204
562 5267	740
562 5649	537
562 5767	438
562 8098	377
562 8099	345
562 8100	319
562 8101	301
562 8102	264
562 8103	224
562 8104	221
562 8105	212
562 8106	205

562 8107 196

563 154 759

563 206 504

563 227 1268

563 250 2620

563 366 641

563 565 2303

563 569 430

563 572 713

563 575 526

563 580 2032

563 581 2781

563 582 416

563 583 415

563 728 2069

563 1422 1025

563 1976 1984

563 2630 547

563 2637 721

563 2893 1043

563 2894 1305

563 2896 443

563 3024 765

563 4524 561

563 4543 668

563 5660 3435

563 6914 1011

563 7056 874

563 8108 3080

563 8109 3030

563 8110	2367
563 8111	1701
563 8112	1550
563 8113	1533
563 8114	1215
563 8115	1132
563 8116	963
563 8117	888
563 8118	851
563 8119	840
563 8120	831
563 8121	820
563 8122	781
563 8123	696
563 8124	592
563 8125	592
563 8126	581
563 8127	483
563 8128	476
563 8129	453
563 8130	411
565 58	80
565 59	175
565 64	124
565 67	152
565 72	1232
565 76	20
565 81	48
565 85	55
565 159	51

565 172 119

565 173 124

565 182 318

565 187 439

565 190 322

565 238 261

565 546 48

565 599 336

565 627 52

565 715 28

565 851 192

565 857 83

565 997 23

565 998 586

565 1013 37

565 1019 90

565 1073 56

565 1089 141

565 1099 71

565 1151 25

565 1512 129

565 1513 21

565 1713 43

565 1895 52

565 2616 107

565 3186 24

565 3768 143

565 4308 438

565 4382 116

565 4810 97

565 6222 44

565 6915 62

565 8140 108

565 8141 96

565 8142 88

565 8143 87

565 8144 55

565 8145 39

565 8146 27

565 8147 26

565 8148 25

566 18 90

566 32 207

566 59 69

566 72 4101

566 75 73

566 99 84

566 154 72

566 159 435

566 166 145

566 181 106

566 203 64

566 206 61

566 227 74

566 234 63

566 416 89

566 515 246

566 599 152

566 707 62

566 821 71

566 843 83

566 854 142

566 856 74

566 868 194

566 905 428

566 949 74

566 982 58

566 1116 289

566 1119 67

566 1131 81

566 1295 201

566 1302 59

566 1303 144

566 1335 69

566 1336 64

566 1384 183

566 1660 126

566 1778 102

566 2347 65

566 2783 83

566 2834 112

566 3441 61

566 3460 73

566 3988 260

566 4369 63

566 4871 90

566 4876 66

566 5913 80

566 7404 80

566 8149 65

566 8150 58

567 40 762

567 65 1423

567 154 18414

567 173 3277

567 190 3663

567 195 6711

567 196 1112

567 199 1190

567 210 1116

567 211 1833

567 217 5677

567 222 2680

567 225 3501

567 227 1814

567 234 695

567 418 682

567 426 2330

567 428 1970

567 429 1011

567 433 722

567 440 2895

567 615 1547

567 618 949

567 622 2231

567 683 1150

567 859 4257

567 1105 845

567 1375 1226

567 1378 1147

567	1389	947
567	1504	750
567	1632	621
567	1947	613
567	2398	637
567	2402	614
567	2407	1160
567	3258	1615
567	3259	1607
567	3616	1445
567	3691	773
567	3693	818
567	3714	582
567	4079	634
567	4118	1421
567	4262	800
567	5027	634
567	5781	1149
567	6294	1353
567	6298	1084
567	6687	712
568	51	450
568	59	808
568	67	143
568	72	817
568	76	132
568	81	126
568	89	152
568	187	154
568	199	308

568 207 304

568 220 108

568 228 403

568 257 108

568 288 267

568 289 219

568 296 127

568 301 123

568 310 315

568 366 111

568 441 188

568 511 1079

568 533 105

568 599 351

568 627 223

568 712 128

568 715 226

568 718 178

568 734 791

568 735 283

568 851 315

568 986 163

568 993 227

568 1001 153

568 1089 106

568 1090 131

568 1094 638

568 1244 134

568 1376 329

568 1383 159

568 1390 165

568 1633 206

568 1848 211

568 3488 107

568 5787 923

568 6369 126

568 6913 122

568 6920 180

568 8151 336

568 8152 303

568 8153 131

569 55 248

569 65 92

569 67 646

569 89 131

569 157 84

569 251 145

569 257 1285

569 265 105

569 283 155

569 288 487

569 289 285

569 292 259

569 295 335

569 297 82

569 298 140

569 300 189

569 301 148

569 302 98

569 304 112

569 306 200

569 314 120

569 328 377

569 329 108

569 349 165

569 355 189

569 378 85

569 461 83

569 466 88

569 468 94

569 538 80

569 641 102

569 679 641

569 701 160

569 903 138

569 1475 81

569 1761 95

569 1763 344

569 2988 89

569 3074 149

569 3171 188

569 3173 146

569 3396 131

569 3859 89

569 4709 669

569 4716 530

569 4818 95

569 7499 356

569 8154 548

569 8155 125

569 8156 99

570 157 51

570 180 261

570 267 195

570 278 254

570 288 187

570 295 442

570 299 31

570 306 55

570 328 242

570 330 236

570 331 428

570 475 3934

570 527 312

570 545 42

570 907 539

570 951 42

570 1122 353

570 1244 84

570 1455 185

570 1613 633

570 1641 308

570 2222 205

570 2226 88

570 2236 261

570 2247 1197

570 2346 41

570 2906 500

570 2976 1238

570 4562 153

570 5041 903
570 6618 387
570 6621 132
570 6627 125
570 6630 258
570 6631 291
570 7930 91
570 8157 702
570 8158 592
570 8159 482
570 8160 259
570 8161 211
570 8162 171
570 8163 159
570 8164 144
570 8165 138
570 8166 55
570 8167 51
570 8168 49
570 8169 33
570 8170 32
571 55 2948
571 56 362
571 58 2750
571 67 3423
571 89 4474
571 155 15861
571 167 2014
571 229 1089
571 288 1388

571 289 23931

571 292 455

571 295 457

571 298 324

571 299 444

571 300 852

571 306 424

571 310 438

571 318 367

571 320 493

571 329 1346

571 333 301

571 349 739

571 466 1034

571 467 4382

571 485 1957

571 525 1148

571 538 1113

571 679 5050

571 701 897

571 787 345

571 790 590

571 792 324

571 795 559

571 886 308

571 970 515

571 1243 536

571 1580 451

571 2025 556

571 2083 467

571 2130 463

571 2521 631

571 2977 510

571 3193 597

571 3198 534

571 3199 312

571 3502 425

571 3575 1005

571 3674 300

571 5000 435

571 5140 804

572 55 8222

572 67 8350

572 89 1650

572 97 514

572 157 134

572 161 208

572 167 565

572 182 326

572 229 392

572 257 264

572 288 271

572 289 6171

572 292 836

572 295 233

572 298 333

572 299 271

572 300 240

572 302 125

572 306 320

572	310	128
572	318	184
572	325	246
572	344	134
572	352	517
572	461	231
572	466	229
572	523	278
572	535	317
572	548	108
572	646	362
572	679	194
572	701	640
572	802	193
572	890	113
572	906	289
572	1001	218
572	1379	266
572	1409	111
572	1453	172
572	1820	226
572	1934	227
572	3397	120
572	3557	127
572	4560	114
572	4704	236
572	6119	247
572	6428	149
572	7324	114
572	8171	117

572 8172 113
573 69 68
573 241 102
573 278 58
573 292 120
573 301 66
573 306 84
573 330 73
573 378 42
573 475 84
573 903 72
573 1611 218
573 1613 107
573 1641 75
573 1936 52
573 1952 178
573 2179 291
573 2226 149
573 2253 38
573 2906 54
573 2909 45
573 2916 87
573 2921 41
573 2924 40
573 3047 46
573 3227 61
573 3357 176
573 3838 83
573 4586 47
573 6621 69

573 6626 100

573 6629 53

573 6895 69

573 8173 302

573 8174 244

573 8175 200

573 8176 141

573 8177 109

573 8178 105

573 8179 102

573 8180 101

573 8181 93

573 8182 85

573 8183 53

573 8184 49

573 8185 44

573 8186 44

573 8187 43

573 8188 42

573 8189 40

573 8190 39

574 220 137

574 227 205

574 230 745

574 234 722

574 333 131

574 366 195

574 377 1340

574 396 437

574 481 69

574 486 119

574 489 92

574 498 1515

574 501 97

574 533 64

574 536 1321

574 540 231

574 706 207

574 707 76

574 708 210

574 714 450

574 946 605

574 966 481

574 982 670

574 1041 72

574 1043 259

574 1045 1088

574 1249 98

574 1377 86

574 1673 199

574 1874 252

574 2080 240

574 2352 747

574 2661 328

574 2983 110

574 2987 687

574 4823 96

574 4828 422

574 4834 83

574 5630 366

574 6120 3001

574 6121 74

574 6132 424

574 6536 666

574 8191 261

574 8192 254

574 8193 201

574 8194 178

574 8195 91

574 8196 68

574 8197 64

575 7 232

575 63 93

575 67 396

575 70 688

575 72 8824

575 77 88

575 88 200

575 89 829

575 154 91

575 155 606

575 157 173

575 161 120

575 176 883

575 187 358

575 190 1439

575 191 100

575 192 136

575 207 135

575 288 932

575 289 682

575 292 1698

575 294 148

575 298 148

575 306 269

575 316 306

575 328 263

575 331 298

575 349 195

575 475 483

575 486 208

575 511 1648

575 538 829

575 701 171

575 951 285

575 959 149

575 1042 99

575 1061 514

575 1072 193

575 1090 852

575 1109 126

575 1243 176

575 1379 318

575 1400 101

575 1401 258

575 1410 89

575 1705 295

575 1713 722

575 1786 259

575 1795 226

575 8198 110

576 51 398

576 82 62

576 137 61

576 208 55

576 226 71

576 573 60

576 858 69

576 881 59

576 903 53

576 1787 153

576 1790 174

576 1849 137

576 2668 53

576 3875 55

576 3877 52

576 4247 54

576 4723 73

576 4734 60

576 5200 62

576 5348 80

576 5927 92

576 8199 126

576 8200 120

576 8201 100

576 8202 96

576 8203 88

576 8204 87

576 8205 78

576 8206 75

576 8207 74

576 8208 67

576 8209 65

576 8210 63

576 8211 61

576 8212 61

576 8213 61

576 8214 61

576 8215 59

576 8216 59

576 8217 58

576 8218 58

576 8219 56

576 8220 55

576 8221 54

576 8222 54

576 8223 54

576 8224 53

576 8225 53

576 8226 52

576 8227 52

577 51 71

577 59 34

577 72 854

577 81 108

577 153 40

577 154 104

577 159 102

577 163 40

577 182 44

577 187 50

577 190 39

577 203 28

577 221 101

577 228 38

577 234 35

577 238 158

577 429 62

577 441 37

577 533 34

577 868 26

577 880 35

577 1062 175

577 1072 55

577 1073 37

577 1075 188

577 1097 31

577 1098 56

577 1375 26

577 1713 137

577 1743 31

577 1787 26

577 1791 33

577 2330 249

577 3889 45

577 4316 63

577 7026 28

577 8228 176

577 8229 132

577 8230 80

577 8231 57

577 8232 54

577 8233 48

577 8234 45

577 8235 43

577 8236 42

577 8237 41

577 8238 37

577 8239 36

577 8240 32

577 8241 25

578 209 329

578 290 191

578 300 453

578 321 270

578 324 107

578 325 216

578 344 8345

578 458 1063

578 461 2517

578 464 129

578 466 292

578 470 555

578 471 152

578 488 401

578 498 1045

578 536 243

578 540 393

578 681 786

578 685 373

578 686 1513

578 687 3711

578 688 323

578 693 207

578 726 119

578 793 243

578 816 138

578 1035 377

578 1036 163

578 1043 104

578 1183 135

578 1193 823

578 1198 102

578 1455 101

578 1458 118

578 1462 118

578 1863 396

578 1866 102

578 1934 191

578 2617 410

578 2619 395

578 2982 159

578 3057 98

578 3902 131

578 6006 207

578 6595 167

578 7831 426

578 8242 370

578 8243 331

578 8244 286

578 8245 115

579 15 238

579 25 99

579 503 219

579 707 83

579 820 103

579 821 124

579 841 639

579 843 182

579 846 111

579 923 407

579 953 335

579 954 183

579 1214 130

579 1274 321

579 1281 508

579 1547 228

579 2654 244

579 2744 430

579 2765 88

579 2970 80

579 3240 68

579 3637 203

579 3717 130

579 3722 196

579 3820 145

579 3928 81

579 4178 89

579 4265 164

579 4275 73

579 5227 119

579 6289 123

579 6493 319

579 6816 101

579 7941 288

579 8246 351

579 8247 306

579 8248 266

579 8249 258

579 8250 258

579 8251 214

579 8252 170

579 8253 131

579 8254 114

579 8255 114

579 8256 94

579 8257 87

579 8258 87

579 8259 87

579 8260 86

579 8261 71

580 2 803

580 65 588

580 188 326

580 204 377

580 228 798

580 230 393

580 366 1206

580 512 1566

580 614 663

580 722 345

580 770 493

580 805 844

580 808 1277

580 1122 672

580 1216 523

580 1406 553

580 1470 393

580 2398 408

580 2612 397

580 2775 723

580 3670 564

580 3780 452

580 4222 332

580 4826 378

580 4830 340

580 5244 740

580 5444 1096

580 5605 1559

580 5631 430

580 6397 606

580 6602 421

580 8262 1253

580 8263 887

580 8264 732

580 8265 649

580 8266 626

580 8267 625

580 8268 623

580 8269 610

580 8270 603

580 8271 589

580 8272 538

580 8273 529

580 8274 520

580 8275 517

580 8276 419

580 8277 346

580 8278 343

580 8279 335

580 8280 324

581 7 938

581 55 2463

581 67 12062

581 86 16829

581 89 800

581 157 4868

581 161 1512

581 220 4040

581 251 2052

581 257 969

581 265 21338

581 288 735

581 289 782

581 292 693

581 294 4731

581 295 4725

581 299 924

581 310 648

581 333 5049

581 637 733

581 889 7991

581 917 1033

581 954 2865

581 961 5188

581 1130	3407
581 1274	1192
581 1406	666
581 1979	697
581 2179	5645
581 3227	4252
581 3500	795
581 3501	37419
581 3674	1556
581 3940	9792
581 4126	7202
581 4917	6151
581 6429	686
581 7232	605
581 7241	6140
581 8281	9615
581 8282	8471
581 8283	7701
581 8284	4890
581 8285	4611
581 8286	2764
581 8287	1077
581 8288	902
581 8289	871
581 8290	753

581 8291 676

582 768 1204

582 779 1274

582 805 2204

582 813 2275

582 815 2354

582 816 2692

582 823 1634

582 831 2514

582 832 1352

582 839 1658

582 842 2293

582 845 1749

582 846 1135

582 1044 1756

582 1196 984

582 1541 5071

582 1547 1100

582 1549 1028

582 1551 1560

582 1555 1088

582 1557 1334

582 1566 1393

582 1568 3664

582 2143 965

582 2192 1332

582 2210 1745

582 2574 1717

582 2576 2651

582 2586 3122

582 2971 1529

582 3028 1704

582 3548 1230

582 3997 1874

582 4003 7166

582 4004 1094

582 4498 1609

582 5135 1561

582 5961 1356

582 6014 1369

582 8292 13145

582 8293 2289

582 8294 2013

582 8295 1859

582 8296 1544

582 8297 1392

582 8298 1370

582 8299 1274

582 8300 1269

582 8301 1010

582 8302 990

583 162 983

583 173 1464

583 190 339

583 198 860

583 229 575

583 230 720

583 234 1605

583 333 528

583 486 1220

583 490 937

583 492 2140

583 493 388

583 498 1091

583 499 934

583 507 399

583 537 398

583 768 329

583 779 641

583 808 960

583 816 852

583 1044 533

583 1145 355

583 1180 330

583 1181 1159

583 1195 567

583 1197 389

583 1541 405

583 1700 1035

583 1853 591

583 2618 599

583 2661 485

583 2747 356

583 2959 594

583 3040 380

583 3053 820

583 3817 325

583 4228 375

583 4346 359

583 4550 360

583 4891 1002

583 4948 318

583 5926 869

583 6191 320

583 6453 387

583 8277 490

583 8303 610

583 8304 600

583 8305 459

583 8306 369

583 8307 359

584 55 1790

584 291 4820

584 298 6180

584 299 700

584 300 660

584 318 4540

584 344 6650

584 352 2710

584 356 4530

584 360 930

584 364 4140

584 465 6550

584 466 4120

584 570 1800

584 585 5900

584 701 3670

584 718 6290

584 734 3310

584 735 2440

584 985 3260

584 1244	11600
584 1459	9800
584 2436	3750
584 2448	4100
584 2449	3020
584 2450	610
584 2786	3100
584 2902	4550
584 3583	610
584 3598	4520
584 3603	570
584 3765	6450
584 5168	3320
584 5267	32200
584 5375	6700
584 5382	6460
584 5767	950
584 7239	620
584 7408	3450
584 8308	32990
584 8309	6600
584 8310	3010
584 8311	2470
584 8312	2190
584 8313	810
584 8314	690
584 8315	650
584 8316	605
584 8317	550

584 8318 540

585 7 277

585 65 990

585 86 330

585 97 462

585 173 1472

585 184 868

585 190 2713

585 204 298

585 207 2952

585 220 571

585 227 1501

585 228 1275

585 250 993

585 301 403

585 378 257

585 430 429

585 432 454

585 475 662

585 517 598

585 521 1551

585 689 288

585 1048 11004

585 1053 347

585 1512 1730

585 1515 551

585 1975 985

585 1980 621

585 2068 835

585 2515 265

585 2614	765
585 2783	248
585 3237	1519
585 3238	700
585 3683	1074
585 4567	318
585 4569	644
585 4570	608
585 5485	621
585 5488	258
585 5847	340
585 6762	337
585 7324	267
585 8830	407
585 10289	266
585 11394	398
585 13025	310
585 13715	704
585 15996	434
585 18458	310
585 18745	426
586 12	1570
586 28	77
586 706 44
586 712 44
586 821 28
586 923 17
586 954 35
586 1254	124
586 1260	185

586 1261	7
586 1276	765
586 1369	11
586 2765	157
586 2772	32
586 2835	15
586 2961	43
586 2970	277
586 3864	192
586 4269	7
586 4271	3625
586 6129	11
586 6258	37
586 8319	898
586 8320	859
586 8321	579
586 8322	508
586 8323	393
586 8324	286
586 8325	171
586 8326	133
586 8327	130
586 8328	128
586 8329	94
586 8330	78
586 8331	46
586 8332	45
586 8333	34
586 8334	27
586 8335	15

586 8336 12
586 8337 11
586 8338 10
586 8339 10
586 8340 9
586 8341 8
586 8342 8
586 8343 7
586 8344 6
586 8345 6
586 8346 5
587 65 1789
587 152 242
587 163 1726
587 170 335
587 188 2955
587 190 275
587 198 333
587 217 995
587 377 203
587 475 187
587 493 317
587 503 26642
587 510 382
587 514 1519
587 516 1329
587 517 1379
587 710 6225
587 724 379
587 785 765

587 805 391

587 806 2646

587 811 1554

587 815 5236

587 823 1394

587 843 1856

587 982 293

587 1116 675

587 1425 288

587 1854 1208

587 1974 294

587 2661 389

587 2843 469

587 3051 230

587 3240 1447

587 3549 972

587 3758 638

587 4179 666

587 5444 278

587 6724 231

587 7639 291

587 8254 1479

587 8347 1162

587 8348 1055

587 8349 897

587 8350 527

587 8351 391

587 8352 324

587 8353 270

587 8354 216

587 8355 190

589 89 125

589 161 37

589 283 13

589 288 89

589 289 1035

589 290 33

589 292 285

589 295 29

589 300 63

589 318 57

589 325 367

589 327 85

589 344 186

589 355 27

589 455 15

589 456 20

589 458 196

589 461 209

589 466 142

589 475 36

589 498 31

589 523 65

589 544 43

589 646 606

589 686 36

589 701 18

589 787 15

589 790 87

589 797 61

589 802 2410

589 1042 28

589 1428 14

589 1429 144

589 1430 133

589 1432 25

589 1447 20

589 1455 44

589 1775 21

589 2080 54

589 2179 22

589 2462 19

589 3060 16

589 3255 71

589 4474 24

589 5249 55

589 5746 96

589 5988 35

589 7865 40

589 8356 73

589 8357 16

590 89 3239

590 157 34

590 257 63

590 288 225

590 289 27

590 300 781

590 302 1823

590 304 20

590 306 1179

590 316 37

590 327 42

590 333 15

590 342 28

590 349 54

590 355 8171

590 377 47

590 458 27

590 461 177

590 464 435

590 466 1085

590 475 37

590 486 1211

590 498 50

590 523 27

590 531 28

590 545 21

590 679 162

590 685 50

590 691 116

590 701 45

590 797 20

590 907 26

590 1458 26

590 1470 26

590 1681 627

590 1775 16

590 2548 65

590 2591 49

590 3171 426

590 3384 20
590 4291 1491
590 5099 23
590 8358 296
590 8359 200
590 8360 125
590 8361 99
590 8362 26
590 8363 24
590 8364 19
590 8365 15
592 7 2900
592 15 388
592 51 16725
592 55 1081
592 67 389
592 72 515
592 89 561
592 155 656
592 157 3978
592 190 23085
592 230 411
592 234 503
592 292 13760
592 299 627
592 328 474
592 450 2074
592 463 544
592 638 5501
592 652 5973

592 659 718

592 724 3126

592 903 521

592 906 739

592 955 426

592 965 776

592 1044	634
592 1098	631
592 1099	3967
592 1104	2243
592 1123	619
592 1249	858
592 1475	5711
592 1491	779
592 1672	668
592 1788	11232
592 1795	1019
592 2668	2018
592 3019	869
592 3046	2993
592 3047	750
592 3674	6015
592 4316	539
592 4502	782
592 5706	864
592 6262	456
592 8014	908
592 8366	10574
592 8367	9097
592 8368	3152

592 8369 510

593 157 1893

593 230 346

593 288 260

593 295 216

593 300 148

593 302 167

593 304 2378

593 306 151

593 321 1722

593 333 1183

593 344 2220

593 355 209

593 456 141

593 458 118

593 461 655

593 466 219

593 468 136

593 484 141

593 498 1866

593 681 23284

593 685 675

593 686 128

593 691 198

593 701 1466

593 793 96

593 797 118

593 908 116

593 1198 114

593 1436 151

593 1438 1128

593 1698 89

593 2395 300

593 2440 431

593 2462 302

593 3073 330

593 3401 134

593 4260 88

593 4294 298

593 5249 192

593 8370 5022

593 8371 467

593 8372 377

593 8373 358

593 8374 260

593 8375 210

593 8376 165

593 8377 131

593 8378 118

593 8379 97

593 8380 93

594 157 13

594 163 12

594 176 12

594 183 27

594 190 12

594 198 17

594 209 13

594 220 233

594 228 30

594 234 10

594 250 12

594 377 40

594 475 13

594 488 12

594 494 27

594 511 307

594 537 12

594 706 38

594 707 311

594 712 21

594 768 283

594 816 31

594 830 47

594 918 204

594 1044 18

594 1116 57

594 1131 35

594 1132 11

594 1145 17

594 1180 35

594 1185 17

594 1194 21

594 1249 11

594 1369 11

594 1406 13

594 1412 152

594 1639 43

594 1672 521

594 1863 332

594 1974 65

594 2204 14

594 2797 27

594 2834 21

594 2850 23

594 3373 23

594 8381 318

594 8382 66

594 8383 52

594 8384 23

594 8385 13

595 25 1811

595 163 1086

595 506 2183

595 707 5139

595 724 1451

595 737 1056

595 821 1416

595 841 5923

595 843 1205

595 917 29849

595 918 4310

595 920 1487

595 922 5580

595 923 1108

595 924 1067

595 928 3539

595 930 2185

595 934 2135

595 936 2767

595 937 4115

595 942 4976

595 947 2332

595 952 1186

595 953 1396

595 954 2304

595 956 1470

595 957 4639

595 1206 972

595 1257 1459

595 1274 1931

595 1337 1124

595 1338 1335

595 1339 1255

595 1343 1778

595 1344 992

595 1349 1920

595 1355 990

595 1358 1090

595 1364 910

595 1369 3472

595 1374 949

595 1412 1181

595 1527 1924

595 1992 1698

595 2838 1093

595 3425 1497

595 4326 1231

595 4680 2581

595 8386 981

595 8387 953

596 30 586

596 51 536

596 59 1254

596 72 13228

596 82 1038

596 159 855

596 167 834

596 187 265

596 203 333

596 429 5863

596 441 16295

596 599 739

596 715 440

596 863 608

596 868 506

596 875 408

596 993 310

596 998 388

596 1001 1270

596 1014 1304

596 1019 240

596 1073 309

596 1083 1166

596 1103 245

596 1295 259

596 1513 297

596 1534 406

596 1811 230

596 1814 456

596 1886 291

596 2470 310

596 2531 4360

596 2552 244

596 2559 227

596 2637 577

596 2938 255

596 3281 233

596 3431 372

596 3768 3379

596 3781 284

596 3941 610

596 5258 314

596 5260 1535

596 5420 255

596 5785 483

596 6241 353

596 6722 314

596 7343 839

596 8388 35323

596 8389 243

598 55 478

598 89 4484

598 166 862

598 288 715

598 289 11718

598 292 687

598 299 379

598 300 806

598 301 412

598 308 296

598 316 432

598 323 623

598 324 470

598 328 465

598 333 519

598 342 646

598 349 669

598 461 328

598 466 614

598 498 288

598 546 300

598 644 786

598 679 297

598 686 348

598 704 354

598 903 391

598 907 353

598 1375 309

598 1470 739

598 1609 660

598 1781 1660

598 2018 386

598 2035 454

598 2071 460

598 3073 500

598 3897 406

598 3904 394

598 4260 307

598 4923 439

598 5806 459

598 7888 415

598 8409 1445

598 8410 687

598 8411 621

598 8412 542

598 8413 539

598 8414 501

598 8415 500

598 8416 349

598 8417 330

601 65 288

601 81 4031

601 196 871

601 238 826

601 384 298

601 723 325

601 726 907

601 1109 1074

601 1705 1004

601 1743 454

601 1772 572

601 2307 484

601 2312 631

601 3877 478

601 4365 541

601 5074 693

601 6961 424

601 8424 1729

601 8425 1558

601 8426	1432
601 8427	1311
601 8428	1008
601 8429	992
601 8430	985
601 8431	913
601 8432	891
601 8433	788
601 8434	740
601 8435	739
601 8436	717
601 8437	662
601 8438	565
601 8439	554
601 8440	553
601 8441	540
601 8442	520
601 8443	443
601 8444	425
601 8445	422
601 8446	410
601 8447	386
601 8448	386
601 8449	371
601 8450	366
601 8451	344
601 8452	339
601 8453	310
601 8454	300
601 8455	298

601	8456	292
602	152	497
602	162	257
602	276	211
602	412	221
602	436	343
602	500	172
602	770	315
602	953	169
602	1090	478
602	1122	213
602	1162	308
602	1228	302
602	1960	194
602	2196	282
602	2276	195
602	2381	191
602	2574	560
602	2580	933
602	2847	167
602	3028	174
602	3040	185
602	3041	182
602	3335	324
602	3685	233
602	4100	525
602	4356	167
602	5100	175
602	5423	423
602	8457	2377

602 8458 525

602 8459 431

602 8460 255

602 8461 249

602 8462 247

602 8463 246

602 8464 235

602 8465 231

602 8466 229

602 8467 226

602 8468 217

602 8469 210

602 8470 198

602 8471 197

602 8472 190

602 8473 188

602 8474 176

602 8475 169

602 8476 168

602 8477 166

602 8478 165

603 89 34

603 190 203

603 198 64

603 208 37

603 220 88

603 230 99

603 418 61

603 420 48

603 560 33

603 707 505	
603 709 66	
603 716 50	
603 862 44	
603 959 27	
603 1044	83
603 1145	31
603 1400	252
603 1510	65
603 1632	86
603 1700	252
603 1873	27
603 1988	47
603 2121	142
603 2605	64
603 2821	41
603 3259	35
603 3461	180
603 3475	120
603 4243	92
603 4889	94
603 5154	101
603 5657	45
603 6197	41
603 7665	29
603 8479	345
603 8480	144
603 8481	104
603 8482	90
603 8483	80

603 8484 79
603 8485 67
603 8486 61
603 8487 47
603 8488 39
603 8489 39
603 8490 38
603 8491 32
603 8492 30
603 8493 28
603 8494 27
604 55 1168
604 67 15146
604 71 1342
604 89 2707
604 155 1783
604 257 1165
604 265 2010
604 288 2156
604 289 3544
604 291 986
604 292 1431
604 293 1064
604 295 1154
604 296 1159
604 299 8339
604 300 1059
604 301 1165
604 302 1669
604 306 1261

604 310 2969

604 311 1431

604 316 770

604 333 4106

604 349 1164

604 360 2512

604 378 8434

604 389 1997

604 518 1363

604 525 2735

604 534 993

604 592 1281

604 701 1890

604 722 1000

604 891 1658

604 907 1666

604 913 1608

604 930 4694

604 959 1959

604 1729 962

604 2220 1440

604 2523 2115

604 4883 1410

604 4920 3036

604 5767 807

604 5768 798

604 7148 1312

604 8495 1145

604 8496 1088

604 8497 956

604 8498 786

605 56 454

605 154 670

605 159 756

605 163 392

605 203 536

605 207 889

605 208 461

605 210 403

605 215 354

605 217 523

605 220 452

605 227 1733

605 229 570

605 234 2260

605 245 358

605 412 831

605 428 1121

605 441 1798

605 533 698

605 534 384

605 599 3859

605 606 660

605 620 745

605 707 346

605 709 538

605 718 443

605 961 778

605 982 386

605 986 425

605 1089 370

605 1382 511

605 1504 439

605 1519 868

605 1983 1249

605 2361 463

605 3253 2103

605 4384 492

605 5048 659

605 6348 342

605 6917 345

605 8499 1488

605 8500 796

605 8501 659

605 8502 511

605 8503 497

605 8504 435

605 8505 415

605 8506 384

605 8507 367

605 8508 351

606 52 801

606 53 635

606 54 560

606 65 576

606 72 722

606 81 443

606 152 1823

606 154 5308

606 188 2402

606 190 582

606 203 569

606 226 586

606 233 771

606 237 605

606 238 604

606 370 522

606 418 1157

606 436 668

606 614 1982

606 629 618

606 813 439

606 815 703

606 842 486

606 843 498

606 846 1137

606 854 483

606 859 1070

606 953 428

606 964 861

606 1098 503

606 1131 1991

606 1372 1083

606 1781 659

606 1824 692

606 1992 1154

606 2006 885

606 2151 913

606 2576 522

606 2594 721

606 2654	478
606 3788	1589
606 4143	456
606 4285	712
606 5237	444
606 8509	853
606 8510	655
606 8511	603
606 8512	579
606 8513	469
606 8514	425
607 12 120	
607 841 615	
607 1124	59
607 1264	43
607 2749	149
607 2753	407
607 2774	39
607 2824	66
607 3809	46
607 4271	154
607 4272	136
607 4290	95
607 6983	341
607 7055	889
607 8322	303
607 8323	623
607 8324	422
607 8328	38
607 8329	53

607 8332	124
607 8336	551
607 8344	199
607 8515	1494
607 8516	1300
607 8517	1093
607 8518	549
607 8519	440
607 8520	325
607 8521	257
607 8522	213
607 8523	187
607 8524	126
607 8525	121
607 8526	118
607 8527	116
607 8528	105
607 8529	103
607 8530	83
607 8531	83
607 8532	77
607 8533	75
607 8534	66
607 8535	59
607 8536	57
607 8537	55
607 8538	53
607 8539	45
607 8540	43
607 8541	41

607 8542	39
608 717 24	
608 1242	58
608 1244	23
608 1802	20
608 2351	15
608 2463	15
608 2796	12
608 2834	56
608 3230	32
608 3458	17
608 3482	25
608 4785	43
608 5433	38
608 5436	14
608 7937	21
608 8314	22
608 8543	49
608 8544	47
608 8545	45
608 8546	45
608 8547	40
608 8548	30
608 8549	28
608 8550	28
608 8551	26
608 8552	22
608 8553	21
608 8554	20
608 8555	19

608 8556 19

608 8557 18

608 8558 18

608 8559 17

608 8560 17

608 8561 17

608 8562 17

608 8563 16

608 8564 15

608 8565 15

608 8566 15

608 8567 14

608 8568 14

608 8569 14

608 8570 14

608 8571 13

608 8572 13

608 8573 12

608 8574 12

608 8575 12

608 8576 12

609 55 358

609 67 460

609 89 3656

609 154 359

609 157 544

609 159 434

609 196 424

609 228 2554

609 229 373

609 234 577

609 288 1204

609 289 2531

609 292 724

609 295 992

609 298 392

609 300 497

609 301 447

609 318 1149

609 326 629

609 333 508

609 425 535

609 432 1603

609 456 1513

609 461 1495

609 466 1213

609 485 578

609 523 483

609 526 778

609 689 752

609 703 485

609 757 760

609 1037 540

609 1048 1219

609 1151 570

609 1243 365

609 1375 374

609 1390 1274

609 1400 5228

609 1609 770

609 1954 553
609 2137 565
609 2533 493
609 2614 1307
609 3371 529
609 3672 599
609 3693 452
609 5001 375
609 6374 1531
609 8577 567
609 8578 478
611 32 6
611 58 9
611 72 55
611 84 12
611 190 26
611 432 6
611 443 94
611 450 12
611 526 19
611 532 15
611 539 18
611 703 33
611 889 9
611 997 90
611 1001 7
611 1062 214
611 1151 34
611 1155 28
611 1167 7

611 1376	35
611 1396	7
611 1409	19
611 1609	9
611 1632	17
611 1738	48
611 2521	6
611 2535	17
611 2587	8
611 2602	8
611 2708	6
611 3288	6
611 4038	12
611 4245	6
611 4308	140
611 4435	6
611 4704	64
611 4725	8
611 4732	8
611 4735	10
611 5547	6
611 6374	58
611 8048	7
611 8579	242
611 8580	30
611 8581	21
611 8582	17
611 8583	17
611 8584	10
611 8585	10

611 8586 8

612 55 9084

612 56 202

612 58 238

612 64 102

612 65 115

612 89 552

612 190 170

612 207 135

612 228 343

612 229 153

612 288 587

612 289 218

612 298 127

612 300 159

612 306 115

612 352 571

612 424 79

612 446 137

612 461 234

612 526 124

612 532 399

612 535 582

612 546 103

612 679 250

612 689 152

612 701 117

612 886 345

612 906 1723

612 1090 137

612 1098 451

612 1106 123

612 1383 185

612 1390 311

612 1400 330

612 1409 126

612 1609 91

612 1934 259

612 2121 200

612 2130 203

612 2137 142

612 2139 208

612 3068 78

612 3288 221

612 3502 231

612 3706 114

612 4089 158

612 5480 129

612 8587 143

612 8588 125

612 8589 122

613 204 3

613 212 5

613 228 55

613 366 9

613 418 3

613 433 9

613 602 10

613 606 10

613 630 1

613 718 15

613 735 6

613 868 11

613 1375 6

613 1415 10

613 1426 10

613 1519 9

613 1718 12

613 1875 1

613 2277 3

613 2786 14

613 3619 13

613 4538 21

613 5446 6

613 5790 13

613 7039 9

613 7147 1

613 7411 7

613 8313 2

613 8590 17

613 8591 15

613 8592 13

613 8593 8

613 8594 5

613 8595 5

614 173 675

614 182 1895

614 198 638

614 207 725

614 209 1401

614 233 607

614 268 641

614 378 765

614 383 615

614 403 753

614 472 816

614 488 1012

614 492 1821

614 493 916

614 494 579

614 497 3280

614 500 861

614 507 1383

614 517 787

614 519 1061

614 536 771

614 537 644

614 540 650

614 602 1207

614 813 728

614 815 771

614 830 1513

614 966 1009

614 978 2316

614 1116 1751

614 1145 723

614 1375 1513

614 1378 746

614 1470 1245

614 1672 32460

614 1854 2123

614 1873 616

614 1874 686

614 2379 667

614 2380 951

614 2843 596

614 2850 769

614 3246 628

614 3428 563

614 3753 734

614 4103 682

614 4179 800

614 6249 706

614 7639 729

614 8596 1004

615 8597 7

616 12 131

616 472 100

616 503 129

616 707 48808

616 710 216

616 726 724

616 821 239

616 834 94

616 837 336

616 841 925

616 843 400

616 846 309

616 917 833

616 918 2172

616 920 383	
616 947 232	
616 954 251	
616 978 151	
616 1044	153
616 1131	154
616 1274	212
616 1344	288
616 1351	94
616 1356	149
616 1357	109
616 1360	226
616 1364	112
616 1522	100
616 1525	201
616 1526	192
616 1527	282
616 1672	324
616 1870	100
616 2379	237
616 2380	172
616 2595	178
616 2744	353
616 2746	169
616 2794	284
616 3238	871
616 3637	244
616 3717	202
616 3719	183
616 5485	182

616 7930 148

616 8254 144

616 8598 181

616 8599 111

616 8600 102

616 8601 95

617 55 655

617 67 317

617 72 450

617 89 301

617 172 164

617 173 173

617 239 358

617 257 770

617 260 161

617 263 239

617 266 379

617 288 286

617 292 532

617 295 2332

617 299 187

617 301 685

617 312 256

617 314 271

617 317 1121

617 318 190

617 322 233

617 332 165

617 349 301

617 468 357

617 484 182

617 543 320

617 757 243

617 903 321

617 913 380

617 1451 430

617 1596 186

617 1613 374

617 1935 187

617 2220 625

617 3109 198

617 3314 423

617 3317 184

617 3318 179

617 3488 234

617 4566 3281

617 4713 548

617 6226 186

617 6378 605

617 8074 262

617 8077 473

617 8602 320

617 8603 263

617 8604 250

617 8605 235

617 8606 188

618 383 187

618 418 183

618 460 77

618 762 109

618 763 380	
618 959 66	
618 1543	263
618 2585	60
618 3043	96
618 4597	63
618 4888	122
618 4893	664
618 5716	440
618 5936	174
618 6228	143
618 8607	878
618 8608	417
618 8609	383
618 8610	353
618 8611	338
618 8612	236
618 8613	182
618 8614	154
618 8615	149
618 8616	129
618 8617	125
618 8618	121
618 8619	111
618 8620	110
618 8621	105
618 8622	102
618 8623	94
618 8624	89
618 8625	84

618 8626 81

618 8627 75

618 8628 73

618 8629 69

618 8630 69

618 8631 68

618 8632 67

618 8633 66

618 8634 66

618 8635 64

618 8636 64

618 8637 61

618 8638 61

618 8639 60

618 8640 57

618 8641 56

619 88 321

619 154 502

619 190 1521

619 207 5879

619 208 442

619 217 1804

619 227 2130

619 228 578

619 229 399

619 298 552

619 412 434

619 424 2924

619 425 406

619 427 349

619 428 863

619 429 511

619 430 561

619 432 353

619 441 1293

619 448 605

619 599 321

619 618 682

619 629 774

619 689 624

619 1090 626

619 1109 401

619 1163 2368

619 1375 329

619 1376 373

619 1390 364

619 1504 486

619 1512 1161

619 1517 1079

619 1520 756

619 1971 511

619 1988 625

619 2083 345

619 2133 462

619 2591 343

619 2600 377

619 2815 1140

619 3213 369

619 3685 426

619 3692 2029

619 3797 842
619 5429 410
619 7738 757
619 8642 748
619 8643 734
619 8644 368
620 67 585
620 89 699
620 97 242
620 209 2141
620 288 836
620 289 1294
620 291 302
620 292 316
620 298 277
620 300 974
620 302 234
620 306 425
620 333 361
620 344 303
620 378 1125
620 380 301
620 461 222
620 486 255
620 498 808
620 518 3521
620 542 371
620 544 229
620 679 451
620 689 249

620 701 303

620 714 278

620 889 367

620 903 407

620 930 1404

620 955 730

620 957 231

620 961 1205

620 1114 842

620 1246 1250

620 1511 264

620 2014 427

620 2094 304

620 2478 251

620 2632 334

620 3057 739

620 3184 541

620 4135 1093

620 4136 261

620 4137 317

620 5459 1140

620 8645 1364

620 8646 637

620 8647 381

620 8648 343

620 8649 269

621 55 1978

621 89 15112

621 190 4361

621 191 2703

621 229 2243

621 279 3353

621 288 4464

621 289 45189

621 290 2696

621 291 8999

621 292 6777

621 294 3942

621 295 2470

621 300 2554

621 302 1431

621 311 1775

621 323 3557

621 344 3014

621 349 2671

621 369 1519

621 377 3018

621 378 5620

621 393 2027

621 486 1947

621 498 9504

621 518 9763

621 679 6019

621 680 5544

621 681 12006

621 906 13005

621 1383 1382

621 1684 4287

621 1705 3610

621 1918 1420

621 2035 4832

621 2092 4081

621 2097 1463

621 2619 1623

621 4487 1399

621 4918 2480

621 4920 1383

621 4922 1440

621 4923 2760

621 5459 1423

621 5746 1352

621 8650 2578

621 8651 2146

621 8652 2040

621 8653 1426

621 8654 1421

622 7 245

622 152 1143

622 154 3504

622 159 231

622 163 260

622 197 211

622 199 471

622 203 385

622 206 893

622 210 235

622 212 263

622 227 1152

622 230 202

622 233 881

622 235 312

622 429 288

622 434 219

622 436 354

622 440 1582

622 475 831

622 498 218

622 511 227

622 599 499

622 603 201

622 615 400

622 616 237

622 622 230

622 715 235

622 851 2037

622 854 224

622 857 456

622 859 523

622 964 393

622 1098 437

622 1119 385

622 1239 257

622 1858 219

622 1881 371

622 2006 215

622 2491 271

622 2493 713

622 2531 676

622 2624 283

622 3272 225

622 3505 1426

622 7081 214

622 8655 975

622 8656 713

622 8657 327

622 8658 199

623 81 425

623 152 4295

623 154 3418

623 156 452

623 163 3578

623 173 465

623 186 435

623 188 1218

623 190 1332

623 203 469

623 215 1110

623 226 1747

623 227 2809

623 234 772

623 436 549

623 453 951

623 506 1024

623 512 833

623 615 841

623 854 500

623 859 560

623 868 761

623 964 668

623 975 1041

623 1110 528

623 1118 373

623 1131 1288

623 1216 385

623 1372 1340

623 1411 3041

623 1413 909

623 1504 650

623 2624 526

623 3230 607

623 3272 513

623 3481 840

623 3774 494

623 3940 523

623 4366 1099

623 4675 1860

623 4784 552

623 4790 401

623 5172 433

623 5297 808

623 5416 1507

623 6137 675

623 7929 1166

623 8659 1606

623 8660 791

623 8661 567

624 25 114

624 72 259

624 96 736

624 154 92

624 159 150

624 163 220

624 192 131

624 198 278

624 199 139

624 204 93

624 229 112

624 230 196

624 238 785

624 352 105

624 377 929

624 378 116

624 418 621

624 599 119

624 679 614

624 712 234

624 718 365

624 959 464

624 982 322

624 1001 353

624 1249 201

624 1295 457

624 1343 115

624 1378 1205

624 1534 363

624 1700 500

624 1729 749

624 2614 123

624 3438 207

624 3886 156

624 3945 94

624 5074 290

624 5215 106

624 5781 189

624 7323 162

624 8422 124

624 8662 416

624 8663 217

624 8664 215

624 8665 159

624 8666 147

624 8667 135

624 8668 125

624 8669 102

624 8670 91

624 8671 87

625 59 212

625 65 388

625 89 216

625 154 327

625 203 159

625 225 113

625 229 117

625 416 183

625 418 176

625 430 501

625 440 309

625 632 301

625 748 585

625 757 288

625	898	210
625	1062	169
625	1163	111
625	1164	296
625	1239	331
625	1390	252
625	1394	158
625	1947	151
625	1954	111
625	1964	638
625	2139	119
625	2416	127
625	2534	133
625	2535	211
625	2538	323
625	2542	342
625	2582	432
625	3294	167
625	3411	386
625	3700	332
625	4384	324
625	4704	135
625	4736	167
625	5229	242
625	6210	125
625	6217	672
625	7074	355
625	8672	305
625	8673	223
625	8674	162

625 8675 147

625 8676 134

625 8677 128

625 8678 128

625 8679 112

625 8680 107

627 63 458

627 69 1106

627 163 374

627 166 271

627 246 2113

627 389 373

627 917 1781

627 920 1765

627 924 1294

627 928 3517

627 930 225

627 937 785

627 944 370

627 949 725

627 956 703

627 957 1195

627 959 561

627 1264 294

627 1342 604

627 1343 2726

627 1349 2406

627 1710 445

627 1740 434

627 1816 1311

627 1836 234

627 2055 565

627 2446 302

627 3063 483

627 3788 1467

627 4316 528

627 4679 326

627 4787 586

627 5236 2554

627 5456 397

627 6516 283

627 6522 1596

627 6575 316

627 7097 693

627 7409 286

627 8689 1614

627 8690 950

627 8691 535

627 8692 417

627 8693 417

627 8694 313

627 8695 311

627 8696 301

627 8697 300

627 8698 247

627 8699 237

628 53 273

628 59 111

628 64 144

628 65 230

628 72 211

628 84 99

628 88 164

628 154 147

628 159 171

628 182 117

628 184 97

628 190 177

628 207 182

628 210 187

628 225 92

628 229 154

628 418 105

628 424 177

628 432 293

628 436 78

628 441 105

628 444 137

628 447 80

628 450 97

628 533 205

628 604 352

628 606 129

628 757 108

628 1061 190

628 1094 124

628 1106 116

628 1109 141

628 1239 342

628 1242 144

628 1396 106
628 1964 401
628 1986 305
628 1988 121
628 2605 84
628 2610 90
628 3026 188
628 6074 86
628 6217 216
628 8700 202
628 8701 134
628 8702 92
628 8703 89
628 8704 85
628 8705 80
628 8706 78
629 65 339
629 81 430
629 154 597
629 163 1277
629 166 327
629 207 512
629 212 580
629 222 402
629 225 1183
629 226 1193
629 227 1572
629 228 586
629 422 1176
629 424 402

629 436 392

629 439 1080

629 554 442

629 599 437

629 705 359

629 706 498

629 735 594

629 863 1021

629 868 688

629 932 520

629 982 522

629 1090 589

629 1242 445

629 1244 329

629 1412 354

629 1416 570

629 1553 408

629 1764 445

629 1771 482

629 1977 374

629 2277 556

629 2364 422

629 2899 1200

629 3391 408

629 4793 957

629 5416 431

629 6147 752

629 7411 376

629 8707 1137

629 8708 608

629 8709 592

629 8710 535

629 8711 513

629 8712 506

629 8713 441

629 8714 323

631 65 4394

631 154 661

631 182 388

631 184 246

631 190 844

631 198 1241

631 207 1936

631 212 233

631 217 1209

631 225 674

631 227 645

631 228 1499

631 229 475

631 230 579

631 234 347

631 424 1519

631 439 211

631 511 263

631 533 316

631 614 217

631 706 1336

631 707 2627

631 708 291

631 709 317

631 716 687

631 726 230

631 917 1834

631 918 205

631 922 307

631 959 242

631 982 395

631 1048 1281

631 1090 329

631 1098 227

631 1338 372

631 1358 436

631 1372 288

631 1375 315

631 1406 304

631 1412 262

631 1415 398

631 1512 775

631 1515 805

631 1873 1308

631 1988 462

631 2009 340

631 2343 261

631 2344 366

631 2346 288

631 8710 4034

632 55 1623

632 67 3130

632 89 3426

632 207 604

632 257 607

632 285 464

632 288 1584

632 289 12746

632 291 2174

632 292 4072

632 293 2496

632 294 478

632 295 2741

632 296 554

632 298 1408

632 299 498

632 300 553

632 302 1384

632 306 1483

632 310 370

632 311 572

632 318 1329

632 325 450

632 326 380

632 333 835

632 344 1067

632 349 663

632 352 466

632 461 559

632 466 6952

632 498 1206

632 525 559

632 536 1188

632 538 1372

632 680 408

632 681 464

632 701 920

632 903 682

632 913 683

632 972 1112

632 980 623

632 1451 394

632 1684 630

632 2018 456

632 2083 609

632 2521 1082

632 3057 605

632 3200 382

632 3501 440

632 5934 371

633 65 68

633 88 63

633 182 207

633 209 125

633 220 575

633 230 121

633 377 271

633 424 104

633 498 124

633 510 328

633 536 145

633 705 183

633 808 112

633 810 145

633 813 247

633 815 323

633 818 88

633 823 329

633 846 58

633 982 149

633 1541	162
633 1662	101
633 1672	213
633 1681	435
633 1725	233
633 2346	260
633 2595	133
633 3032	207
633 3041	300
633 3376	97
633 3382	88
633 3383	49
633 4177	111
633 5119	55
633 5454	46
633 5630	425
633 6120	61
633 7756	83
633 8724	292
633 8725	204
633 8726	200
633 8727	188
633 8728	159
633 8729	92

633 8730	90

633 8731	82

633 8732	55

633 8733	53

633 8734	50

633 8735	49

634 65	193

634 89	83

634 157 450

634 184 350

634 187 97

634 227 100

634 229 956

634 257 89

634 300 142

634 311 399

634 333 816

634 344 6785

634 403 91

634 458 420

634 498 122

634 536 339

634 689 110

634 691 1285

634 712 244

634 734 120

634 951 222

634 959 108

634 1122	120

634 1246	108

634 1413 311

634 1447 451

634 1459 687

634 1462 121

634 1464 102

634 1489 102

634 1689 100

634 1934 131

634 2439 95

634 2525 87

634 2982 1025

634 3302 457

634 3373 202

634 3588 298

634 5267 1362

634 8736 679

634 8737 419

634 8738 325

634 8739 161

634 8740 152

634 8741 135

634 8742 107

634 8743 106

634 8744 104

634 8745 89

634 8746 88

635 53 66

635 65 173

635 159 47

635 190 632

635 203 47

635 207 63

635 212 44

635 227 1176

635 237 815

635 418 717

635 429 51

635 430 73

635 437 109

635 445 89

635 511 419

635 618 49

635 707 63

635 733 244

635 863 46

635 868 60

635 1092 94

635 1413 81

635 1415 49

635 1425 917

635 1426 74

635 1556 79

635 2055 210

635 2139 49

635 2842 46

635 2852 78

635 2855 45

635 3302 71

635 3693 187

635 4545 63

635 5175 452
635 5212 200
635 5241 154
635 6187 117
635 8027 103
635 8747 229
635 8748 206
635 8749 172
635 8750 170
635 8751 150
635 8752 68
635 8753 57
635 8754 52
635 8755 52
635 8756 48
635 8757 47
636 55 173
636 67 369
636 86 294
636 89 263
636 157 1424
636 161 299
636 180 169
636 190 336
636 251 244
636 257 528
636 265 3264
636 288 479
636 289 248
636 291 399

636 292 272

636 294 490

636 295 1024

636 296 250

636 299 220

636 301 224

636 310 335

636 349 277

636 354 271

636 360 172

636 461 966

636 465 207

636 701 565

636 909 200

636 959 787

636 1058 263

636 1463 229

636 1475 191

636 2067 734

636 2454 253

636 3500 1265

636 4565 364

636 4568 233

636 4573 164

636 4769 235

636 8758 496

636 8759 361

636 8760 286

636 8761 247

636 8762 234

636 8763	217

636 8764	211

636 8765	205

636 8766	199

636 8767	192

636 8768	169

637 159 67

637 176 35

637 228 36

637 234 68

637 511 39

637 614 205

637 630 32

637 706 35

637 710 37

637 715 36

637 724 38

637 735 418

637 854 60

637 856 89

637 857 74

637 858 43

637 917 117

637 918 155

637 951 41

637 959 36

637 982 52

637 1249	84

637 1360	80

637 1372	38

637 1415	39
637 1426	125
637 1513	91
637 1643	34
637 1813	102
637 1814	32
637 1976	77
637 2346	50
637 2347	47
637 2785	37
637 2787	44
637 2853	33
637 3400	53
637 3470	33
637 5926	50
637 6541	34
637 7214	65
637 7227	270
637 8769	317
637 8770	86
637 8771	58
637 8772	55
637 8773	44
637 8774	36
637 8775	35
637 8776	32
638 55	280
638 67	210
638 71	292
638 89	539

638 161 306

638 257 2226

638 285 191

638 288 1504

638 289 25118

638 290 292

638 291 945

638 292 641

638 294 886

638 295 1404

638 296 213

638 298 172

638 299 3434

638 300 2178

638 301 463

638 302 391

638 306 585

638 309 260

638 314 173

638 318 186

638 324 258

638 325 614

638 329 147

638 333 470

638 344 179

638 349 494

638 403 151

638 455 473

638 461 1572

638 465 773

638 466 331

638 475 119

638 523 140

638 538 438

638 543 229

638 544 298

638 679 627

638 701 6708

638 908 259

638 1243 705

638 1580 211

638 1601 632

638 1672 308

638 2801 132

638 4489 1168

638 4565 195

639 56 549

639 64 147

639 107 287

639 174 185

639 328 177

639 386 188

639 389 78

639 418 104

639 529 89

639 722 199

639 757 68

639 968 431

639 1009 580

639 1012 139

639 1021	115
639 1029	121
639 1306	123
639 1383	69
639 1398	88
639 1401	296
639 1410	86
639 2498	131
639 2500	204
639 2605	71
639 3573	151
639 3673	121
639 3707	120
639 3868	101
639 3982	218
639 4036	161
639 5063	116
639 5072	156
639 8390	72
639 8589	69
639 8777	210
639 8778	141
639 8779	138
639 8780	129
639 8781	126
639 8782	126
639 8783	117
639 8784	109
639 8785	91
639 8786	79

639 8787 79

639 8788 78

639 8789 72

639 8790 71

639 8791 69

639 8792 68

640 157 176

640 163 322

640 212 221

640 220 1150

640 227 2214

640 230 224

640 554 207

640 562 741

640 599 2074

640 614 183

640 705 259

640 706 1336

640 725 373

640 728 623

640 732 165

640 735 3367

640 868 1856

640 918 174

640 920 1022

640 959 175

640 1244 278

640 1369 1519

640 1372 1236

640 1411 202

640 1412 2209

640 1415 169

640 1426 266

640 1757 331

640 1803 231

640 1804 329

640 2047 570

640 2342 1418

640 2343 6955

640 2347 257

640 2786 1909

640 2787 186

640 2853 304

640 2855 342

640 2901 1705

640 3470 914

640 3478 1658

640 4251 167

640 4458 374

640 5172 181

640 5416 188

640 5660 815

640 7056 337

640 8793 223

640 8794 196

640 8795 178

641 170 1113

641 190 1486

641 209 2158

641 228 1061

641 229 1103

641 230 1617

641 289 877

641 297 864

641 321 2089

641 324 1276

641 333 864

641 377 4711

641 405 3882

641 455 3175

641 479 1066

641 481 2597

641 497 1422

641 504 1254

641 536 1145

641 544 3113

641 681 1368

641 912 1949

641 1043 1296

641 1044 1301

641 1047 15114

641 1186 4023

641 1246 1973

641 1375 3216

641 1377 1869

641 1380 861

641 1934 1498

641 2150 890

641 2358 1151

641 2526 831

641 3373 1081

641 3375 1061

641 3684 935

641 3919 2500

641 4762 852

641 4923 838

641 5729 1622

641 5746 3603

641 6453 1333

641 6859 1660

641 8796 2414

641 8797 1159

641 8798 1115

641 8799 1077

641 8800 1073

641 8801 906

642 265 252

642 511 239

642 564 223

642 565 190

642 572 627

642 574 254

642 575 161

642 580 575

642 581 550

642 583 425

642 584 429

642 717 216

642 1244 395

642 1469 295

642 2786	429
642 2842	280
642 2864	162
642 2894	360
642 2896	191
642 3019	297
642 3071	151
642 6958	192
642 6959	171
642 7056	709
642 8123	289
642 8282	172
642 8802	847
642 8803	593
642 8804	540
642 8805	519
642 8806	462
642 8807	393
642 8808	386
642 8809	345
642 8810	319
642 8811	302
642 8812	289
642 8813	271
642 8814	268
642 8815	258
642 8816	241
642 8817	214
642 8818	197
642 8819	195

642 8820 194
642 8821 193
642 8822 187
642 8823 171
642 8824 166
642 8825 162
643 51 940
643 53 489
643 89 289
643 96 275
643 98 675
643 157 307
643 173 394
643 199 283
643 221 325
643 288 128
643 429 128
643 436 308
643 437 635
643 554 167
643 599 147
643 615 265
643 679 205
643 689 543
643 690 221
643 905 163
643 971 358
643 1089 331
643 1097 859
643 1164 160

643 1242 955

643 1390 171

643 1514 324

643 1519 259

643 1633 145

643 2127 136

643 2541 498

643 3201 421

643 3279 365

643 3502 130

643 3890 202

643 4384 918

643 4622 137

643 5833 702

643 6203 896

643 8600 431

643 8656 251

643 8670 156

643 8715 190

643 8826 301

643 8827 208

643 8828 199

643 8829 173

643 8830 136

643 8831 135

643 8832 128

644 65 380

644 152 112

644 154 1593

644 163 109

644 182 383

644 190 143

644 227 430

644 229 192

644 418 4340

644 428 50

644 429 41

644 430 138

644 441 46

644 454 113

644 538 164

644 614 47

644 618 195

644 632 73

644 964 43

644 1098 558

644 1109 1463

644 1118 229

644 1122 163

644 1130 61

644 1135 289

644 1375 59

644 1767 214

644 2123 40

644 3057 348

644 3897 282

644 3898 54

644 3902 240

644 3905 356

644 4121 1264

644 4423 139

644 5185 87

644 5235 108

644 6756 119

644 7623 75

644 8229 68

644 8410 575

644 8833 1182

644 8834 1013

644 8835 675

644 8836 426

644 8837 71

644 8838 57

644 8839 47

644 8840 43

644 8841 41

645 63 37

645 97 41

645 227 63

645 265 81

645 294 57

645 575 43

645 727 53

645 903 252

645 959 44

645 1114 43

645 1427 39

645 1800 37

645 1833 37

645 2044 73

645 2046	42
645 2047	48
645 2050	88
645 2054	63
645 2059	38
645 2067	48
645 2079	46
645 3466	45
645 4616	52
645 4711	45
645 5624	45
645 5745	44
645 5787	42
645 7626	49
645 8152	53
645 8842	116
645 8843	76
645 8844	72
645 8845	60
645 8846	60
645 8847	59
645 8848	56
645 8849	55
645 8850	52
645 8851	51
645 8852	49
645 8853	46
645 8854	46
645 8855	46
645 8856	45

645 8857 44
645 8858 43
645 8859 42
645 8860 41
645 8861 38
645 8862 37
646 89 142
646 157 115
646 161 448
646 191 95
646 288 569
646 289 433
646 292 161
646 295 225
646 299 141
646 300 671
646 302 128
646 304 236
646 306 257
646 321 1531
646 325 208
646 327 1007
646 328 123
646 333 511
646 342 123
646 344 706
646 377 101
646 456 183
646 458 1706
646 461 727

646 466 506

646 468 189

646 475 101

646 498 235

646 528 205

646 538 94

646 679 124

646 681 792

646 686 952

646 691 214

646 692 224

646 701 351

646 790 116

646 797 343

646 907 104

646 1032 126

646 1042 887

646 1047 307

646 1452 193

646 1455 168

646 1458 220

646 2607 340

646 2939 123

646 4562 301

646 4811 330

646 8863 117

647 53 1026

647 58 720

647 81 433

647 154 1370

647 159 799

647 162 374

647 203 561

647 217 809

647 233 460

647 238 648

647 418 1225

647 429 654

647 436 414

647 441 895

647 444 808

647 604 409

647 618 469

647 621 431

647 632 467

647 763 532

647 962 628

647 1062 375

647 1098 487

647 1418 717

647 1504 639

647 1731 810

647 1745 362

647 2277 372

647 2289 408

647 2542 812

647 2616 680

647 2691 407

647 3280 1594

647 3411 854

647 3884	677
647 3893	704
647 4058	530
647 5414	1695
647 6217	739
647 6414	590
647 7878	372
647 8672	528
647 8864	1189
647 8865	695
647 8866	435
647 8867	434
647 8868	357
647 8869	357
647 8870	357
647 8871	352

649 190 29

649 198 76

649 220 28

649 377 93

649 490 75

649 500 26

649 503 1586

649 504 27

649 614 41

649 707 55

649 806 32

649 808 354

649 812 352

649 815 132

649 837 101

649 843 29

649 846 187

649 917 25

649 918 38

649 920 57

649 930 84

649 949 31

649 953 39

649 957 77

649 966 371

649 982 72

649 1206 50

649 1339 127

649 1350 23

649 1358 44

649 1372 21

649 1854 733

649 1905 121

649 2009 65

649 2143 28

649 2172 51

649 2379 39

649 2744 21

649 2794 1149

649 2843 25

649 2966 244

649 3051 33

649 3859 82

649 4178 36

649 4608 207

649 4612 42

649 4971 58

649 5673 253

649 8881 222

649 8882 32

650 67 304

650 72 5137

650 89 260

650 159 218

650 190 233

650 210 263

650 228 449

650 229 206

650 288 188

650 289 284

650 317 251

650 329 560

650 330 167

650 377 481

650 403 429

650 405 257

650 486 700

650 498 488

650 500 364

650 521 182

650 527 258

650 531 188

650 540 581

650 689 857

650 716 312

650 969 283

650 1056 400

650 1090 406

650 1098 174

650 1099 411

650 1180 315

650 1183 292

650 1184 183

650 1246 573

650 1376 355

650 1378 201

650 1705 420

650 1713 156

650 3238 806

650 5044 168

650 5482 161

650 5490 608

650 5847 1122

650 6008 472

650 7324 167

650 8883 1136

650 8884 484

650 8885 314

650 8886 184

650 8887 174

651 55 1682

651 64 584

651 67 21540

651 89 526

651 157 530

651 257 105

651 288 161

651 289 7871

651 295 222

651 310 562

651 329 371

651 389 853

651 525 624

651 701 691

651 789 126

651 889 178

651 903 197

651 972 192

651 980 116

651 1098 342

651 1464 671

651 1740 155

651 1942 271

651 2521 430

651 2523 168

651 3501 4368

651 3502 801

651 3674 473

651 4043 333

651 4126 118

651 4572 420

651 6429 1003

651 7445 1421

651 8586 435

651 8888	1669
651 8889	218
651 8890	217
651 8891	216
651 8892	192
651 8893	135
651 8894	127
651 8895	126
651 8896	118
651 8897	116
651 8898	111
651 8899	109
651 8900	107
651 8901	106
651 8902	106
651 8903	94
652 7	387
652 45	87
652 88	152
652 154 133
652 198 157
652 203 61
652 233 3716
652 238 71
652 377 175
652 503 76
652 512 343
652 517 752
652 1044	8
652 1104	37

652 1116	101
652 1118	364
652 1119	2089
652 1131	104
652 1203	100
652 1672	77
652 1701	21
652 1870	31
652 2151	21
652 2380	8
652 2484	162
652 2493	107
652 3238	5
652 3523	7
652 6345	43
652 6394	22
652 7081	211
652 7639	5
652 8347	5956
652 8904	603
652 8905	541
652 8906	524
652 8907	471
652 8908	467
652 8909	146
652 8910	132
652 8911	118
652 8912	99
652 8913	66
652 8914	34

652 8915 19

652 8916 18

652 8917 17

652 8918 13

652 8919 11

652 8920 6

654 46 104

654 65 105

654 163 56

654 181 41

654 186 256

654 188 110

654 190 57

654 230 84

654 370 347

654 497 125

654 506 105

654 511 159

654 518 150

654 592 88

654 707 100

654 715 48

654 726 125

654 910 65

654 959 60

654 969 61

654 978 73

654 1044 99

654 1109 266

654 1118 92

654 1130 53

654 1143 249

654 1150 45

654 1411 45

654 1700 157

654 1705 83

654 1781 74

654 2446 287

654 3084 67

654 3405 75

654 4917 94

654 5151 107

654 8853 49

654 8953 138

654 8954 128

654 8955 93

654 8956 89

654 8957 70

654 8958 69

654 8959 66

654 8960 61

654 8961 57

654 8962 56

654 8963 47

654 8964 45

654 8965 44

655 226 3

655 229 17

655 230 9

655 288 9

655 291 7

655 295 4

655 310 10

655 314 3

655 327 3

655 331 27

655 349 3

655 475 12

655 525 168

655 538 5

655 709 3

655 735 9

655 889 3

655 907 7

655 966 18

655 982 6

655 1470 5

655 1613 3

655 1834 3

655 2083 5

655 3079 4

655 3459 11

655 4566 4

655 6376 4

655 8966 176

655 8967 14

655 8968 10

655 8969 10

655 8970 10

655 8971 9

655 8972 9
655 8973 8
655 8974 7
655 8975 5
655 8976 5
655 8977 5
655 8978 5
655 8979 4
655 8980 4
655 8981 4
655 8982 4
655 8983 3
655 8984 3
655 8985 3
655 8986 3
655 8987 3
656 51 263
656 72 158
656 77 43
656 100 22
656 157 14
656 227 141
656 258 18
656 599 33
656 601 473
656 707 25
656 732 20
656 889 18
656 952 37
656 987 19

656 998 59	
656 1015	25
656 1064	268
656 1079	19
656 1416	16
656 1846	42
656 1889	24
656 2343	60
656 2356	43
656 2554	43
656 2556	16
656 2558	64
656 2562	18
656 2784	36
656 2936	12
656 3201	16
656 3501	53
656 3674	36
656 3856	20
656 3921	22
656 4130	49
656 4180	13
656 5156	22
656 5744	21
656 6776	13
656 8988	155
656 8989	80
656 8990	70
656 8991	43
656 8992	43

656 8993 24

656 8994 22

656 8995 21

656 8996 16

656 8997 15

656 8998 13

657 7 212

657 190 1003

657 207 753

657 227 300

657 233 263

657 367 405

657 377 727

657 381 297

657 388 281

657 399 346

657 425 1099

657 432 352

657 460 318

657 486 847

657 512 594

657 754 872

657 758 4001

657 759 617

657 972 208

657 1048 290

657 1700 396

657 1786 438

657 1789 213

657 1865 632

657 1876 236

657 1904 996

657 2106 226

657 2154 1233

657 2383 1133

657 2747 328

657 2806 230

657 2820 912

657 3046 627

657 3833 336

657 4118 456

657 4430 216

657 4435 475

657 4502 207

657 4896 772

657 6252 315

657 7637 1423

657 8298 319

657 8790 696

657 8999 458

657 9000 261

657 9001 259

657 9002 257

657 9003 247

657 9004 246

657 9005 224

658 51 220

658 56 212

658 58 196

658 64 150

658 72 1513

658 84 264

658 154 169

658 182 183

658 187 343

658 208 503

658 225 125

658 227 122

658 535 138

658 733 153

658 998 243

658 1001 273

658 1013 145

658 1072 150

658 1155 374

658 1379 138

658 1398 461

658 1409 143

658 1639 165

658 1713 133

658 1738 448

658 1895 165

658 1983 168

658 2134 128

658 2136 1284

658 2535 157

658 2818 256

658 3371 335

658 3768 119

658 4022 394

658 4027 199

658 4028 142

658 4245 139

658 4720 277

658 4727 123

658 4733 172

658 5154 228

658 6055 145

658 8141 131

658 9006 210

658 9007 190

658 9008 175

658 9009 131

658 9010 121

658 9011 121

658 9012 118

659 55 4473

659 67 4983

659 154 1197

659 207 652

659 257 613

659 285 1256

659 288 714

659 289 4653

659 291 613

659 292 6291

659 293 562

659 299 1643

659 311 568

659 314 1217

659 316 538

659 322 978

659 349 2693

659 354 1975

659 360 537

659 374 506

659 375 767

659 389 519

659 456 1883

659 465 4273

659 467 828

659 486 1044

659 523 939

659 543 553

659 646 737

659 787 2425

659 790 578

659 794 597

659 891 1204

659 906 581

659 1459 1054

659 1580 736

659 1596 678

659 1918 712

659 1929 521

659 1988 2030

659 2094 745

659 2102 3326

659 2376 670

659 2523 862

659 3324 1488

659 3570 4736

659 5574 6522

659 5767 1270

659 6397 504

659 9013 929

660 67 130

660 187 52

660 666 60

660 669 69

660 877 83

660 1057 126

660 1464 289

660 2515 174

660 3500 130

660 3570 268

660 3572 61

660 3966 85

660 4309 58

660 4565 180

660 4568 60

660 4880 105

660 6002 64

660 6697 109

660 7136 249

660 7734 86

660 8718 72

660 8765 70

660 8823 53

660 9014 282

660 9015	192
660 9016	174
660 9017	170
660 9018	138
660 9019	134
660 9020	130
660 9021	119
660 9022	111
660 9023	109
660 9024	103
660 9025	96
660 9026	94
660 9027	91
660 9028	88
660 9029	85
660 9030	84
660 9031	83
660 9032	81
660 9033	77
660 9034	72
660 9035	68
660 9036	66
660 9037	65
660 9038	64
660 9039	64
660 9040	58
661 196 210	
661 2314	130
661 2316	138
661 4675	123

661 7201	230
661 8425	269
661 8427	124
661 8436	99
661 8439	104
661 8445	112
661 8450	205
661 8454	172
661 9041	305
661 9042	287
661 9043	277
661 9044	259
661 9045	254
661 9046	241
661 9047	225
661 9048	213
661 9049	210
661 9050	184
661 9051	181
661 9052	173
661 9053	172
661 9054	159
661 9055	156
661 9056	153
661 9057	148
661 9058	147
661 9059	145
661 9060	144
661 9061	133
661 9062	131

661 9063 129

661 9064 129

661 9065 122

661 9066 122

661 9067 118

661 9068 117

661 9069 115

661 9070 114

661 9071 113

661 9072 111

661 9073 111

661 9074 107

661 9075 102

661 9076 101

661 9077 100

661 9078 100

662 65 698

662 171 1333

662 191 1286

662 210 432

662 291 728

662 300 654

662 307 466

662 333 636

662 342 760

662 344 5313

662 362 2196

662 403 472

662 501 824

662 533 493

662 679 1317

662 689 2488

662 697 568

662 704 6044

662 966 494

662 1375	1379
662 1377	6476
662 1378	2402
662 1470	2380
662 1852	449
662 1866	443
662 2840	1955
662 3255	549
662 3427	656
662 3466	533
662 3749	819
662 3753	1871
662 3756	514
662 3859	2104
662 4177	857
662 4223	456
662 4488	600
662 4756	784
662 6006	1173
662 6608	664
662 7898	739
662 8274	457
662 8356	423
662 9079	1788
662 9080	1470

662 9081 1449

662 9082 1416

662 9083 960

662 9084 650

662 9085 570

662 9086 492

663 374 180

663 375 432

663 379 269

663 396 321

663 518 379

663 930 388

663 936 158

663 945 276

663 955 147

663 1157 294

663 1857 213

663 2000 238

663 2014 242

663 2111 1392

663 2116 539

663 2498 187

663 2512 1190

663 5087 167

663 5093 199

663 5335 248

663 9087 1084

663 9088 859

663 9089 859

663 9090 679

663 9091	471
663 9092	463
663 9093	411
663 9094	399
663 9095	395
663 9096	300
663 9097	251
663 9098	242
663 9099	225
663 9100	212
663 9101	209
663 9102	203
663 9103	195
663 9104	185
663 9105	175
663 9106	175
663 9107	173
663 9108	172
663 9109	170
663 9110	159
663 9111	158
663 9112	155
663 9113	152
663 9114	148
663 9115	142
663 9116	141

664 154 752

664 196 2042

664 231 1318

664 276 442

664 418 777

664 425 441

664 433 623

664 440 353

664 444 970

664 618 2244

664 622 606

664 632 437

664 757 723

664 1100	353
664 1159	456
664 1732	2903
664 2269	614
664 2433	427
664 3227	321
664 3259	445
664 3685	2220
664 3688	1274
664 3693	504
664 3785	2164
664 3902	455
664 4058	864
664 4091	316
664 4779	1667
664 5439	515
664 5605	811
664 5936	1005
664 6416	389
664 6687	589
664 8657	1030

664 8689 477

664 9117 936

664 9118 824

664 9119 823

664 9120 625

664 9121 577

664 9122 544

664 9123 514

664 9124 504

664 9125 445

664 9126 422

664 9127 381

664 9128 377

664 9129 371

664 9130 342

664 9131 339

665 56 660

665 59 818

665 65 129

665 70 145

665 72 10099

665 99 31

665 154 302

665 159 207

665 173 3095

665 182 84

665 190 876

665 203 171

665 229 190

665 234 437

665 424 93

665 429 251

665 436 178

665 511 588

665 533 186

665 614 100

665 707 520

665 715 310

665 857 46

665 993 24

665 1001 1436

665 1014 119

665 1061 73

665 1072 44

665 1073 68

665 1075 662

665 1090 193

665 1249 33

665 1369 26

665 1381 41

665 1384 25

665 1394 231

665 1397 58

665 1398 87

665 1713 587

665 2149 33

665 2531 170

665 3413 185

665 3892 323

665 4306 29

665 5237 71

665 9132 129

665 9133 123

665 9134 104

665 9135 71

665 9136 26

666 7 265

666 61 257

666 72 1394

666 159 498

666 190 663

666 196 535

666 227 247

666 233 215

666 234 293

666 441 390

666 515 433

666 609 392

666 867 160

666 875 350

666 1098 210

666 1104 184

666 1121 255

666 1123 650

666 1222 206

666 1412 317

666 1534 1099

666 1707 1874

666 3201 1258

666 3280 902

666 3432 184

666 3438 431

666 3454 330

666 3893 1627

666 5079 371

666 5658 296

666 5913 165

666 5919 167

666 6582 433

666 8050 286

666 9137 446

666 9138 430

666 9139 412

666 9140 398

666 9141 383

666 9142 339

666 9143 303

666 9144 286

666 9145 240

666 9146 198

666 9147 190

666 9148 184

666 9149 172

666 9150 163

666 9151 155

666 9152 153

667 67 103

667 89 1492

667 157 133

667 257 131

667 288 779

667 289 5554

667 291 173

667 292 223

667 293 62

667 295 1008

667 300 596

667 302 239

667 306 1776

667 309 91

667 314 111

667 318 129

667 320 51

667 324 121

667 325 58

667 328 212

667 330 139

667 333 74

667 344 79

667 349 471

667 461 494

667 464 150

667 466 322

667 468 94

667 484 105

667 498 314

667 511 91

667 525 72

667 531 101

667 538 49

667 540 77

667 545 146

667 546 132

667 701 427

667 793 208

667 907 123

667 1037 122

667 1241 49

667 1444 56

667 1449 53

667 1453 134

667 1456 49

667 1613 105

667 5947 110

667 7340 104

667 7868 59

668 65 551

668 176 730

668 220 757

668 230 903

668 412 529

668 438 452

668 439 455

668 498 540

668 500 499

668 533 887

668 705 2157

668 706 1183

668 707 902

668 708 5069

668 714 623

668 724 491

668 917 738

668 918 458

668 924 675

668 932 1651

668 946 844

668 951 527

668 957 869

668 1045 854

668 1104 518

668 1186 918

668 1241 1087

668 1249 533

668 1350 414

668 1369 483

668 1513 671

668 1545 783

668 1662 536

668 1672 558

668 1681 1224

668 2032 436

668 2080 1142

668 2346 822

668 2987 561

668 3204 529

668 4087 600

668 4463 611

668 4793 826

668 4850 775

668 5630 908

668 5749 536

668 6120 929

668 6282 401

668 6792 464

668 9153 561

669 89 3879

669 288 1672

669 289 1586

669 290 325

669 293 451

669 300 123

669 318 407

669 320 239

669 321 132

669 325 152

669 333 3705

669 344 166

669 378 296

669 458 218

669 461 1625

669 466 4390

669 468 92

669 471 293

669 475 278

669 481 102

669 486 269

669 498 2612

669 540 121

669 542 292

669 790 547

669 972 1426

669 1035 403

669 1045 438

669 1659 417

669 1670 109

669 1673 1945

669 2025 232

669 2080 2405

669 2082 216

669 2435 356

669 2498 1815

669 2987 452

669 3074 278

669 3080 179

669 3187 1350

669 3392 69

669 3397 7043

669 3404 148

669 8364 487

669 9154 1745

669 9155 408

669 9156 311

669 9157 233

669 9158 174

669 9159 171

670 55 727

670 56 584

670 65 3017

670 84 1144

670 88 1012

670 154 1123

670 167 742

670 182 508

670 206 599

670 207 883

670 228 535

670 229 817

670 298 559

670 310 810

670 535 2392

670 546 783

670 716 485

670 903 905

670 969 535

670 1048 1620

670 1055 1187

670 1090 724

670 1106 633

670 1155 475

670 1376 621

670 1400 473

670 1409 760

670 1486 767

670 1934 511

670 1941 725

670 1954 916

670 1982 672

670 1986 606

670 2132 659

670 2137 1303

670 2521 753

670 2544 589

670 2582 500

670 3237 1523

670 3288 798

670 3730 1250

670 3731 1340

670 4706 993

670 5045 2197

670 5428 618

670 7445 596

670 9160 1454

670 9161 698

670 9162 624

670 9163 489

671 89 221

671 240 91

671 290 7595

671 299 448

671 300 186

671 318 244

671 327 80

671 333 270

671 393 177

671 455 96

671 458 1663

671 469 263

671 486 360

671 538 101

671 544 577

671 646 354

671 679 61947

671 1470	563
671 2080	201
671 2439	565
671 2498	414
671 2548	8018
671 2977	150
671 2986	77
671 3387	74
671 3392	132
671 3576	641
671 4463	172
671 4466	187
671 4474	407
671 5988	110
671 7061	1211
671 7083	100
671 9164	2028
671 9165	595
671 9166	336
671 9167	324
671 9168	299
671 9169	291
671 9170	216
671 9171	211
671 9172	183
671 9173	176
671 9174	159

671 9175 146

671 9176 140

671 9177 137

671 9178 92

671 9179 88

671 9180 85

672 56 140

672 220 241

672 227 117

672 412 254

672 536 130

672 540 166

672 691 79

672 706 101

672 707 452

672 754 83

672 808 471

672 816 243

672 977 94

672 978 86

672 1044 219

672 1046 107

672 1100 171

672 1116 102

672 1180 185

672 1185 154

672 1186 149

672 1188 72

672 1246 172

672 1401 124

672 1672 203

672 1866 88

672 1974 109

672 2214 76

672 2617 104

672 2621 82

672 2829 96

672 2845 260

672 2847 119

672 2959 161

672 4014 100

672 4166 100

672 4550 85

672 4774 97

672 5140 187

672 5239 98

672 5961 72

672 6461 135

672 7160 206

672 7540 166

672 8242 96

672 8371 107

672 8684 113

672 9181 151

672 9182 108

672 9183 80

673 65 507

673 88 232

673 366 321

673 378 281

673 412 206

673 472 230

673 488 401

673 614 195

673 706 362

673 707 182

673 708 168

673 726 2273

673 727 174

673 963 258

673 966 323

673 982 4197

673 1044 476

673 1470 2064

673 1556 525

673 1672 188

673 1681 254

673 2068 2060

673 3078 1297

673 3427 686

673 3459 270

673 3749 452

673 4439 691

673 4466 322

673 4828 332

673 5099 255

673 5425 416

673 5427 3017

673 5630 451

673 7756 987

673 8728 505

673 8810 220

673 9184 1066

673 9185 1043

673 9186 1042

673 9187 936

673 9188 442

673 9189 395

673 9190 394

673 9191 381

673 9192 300

673 9193 271

673 9194 227

673 9195 225

673 9196 194

673 9197 165

674 55 2954

674 89 23305

674 288 552

674 289 102

674 292 173

674 300 347

674 302 209

674 306 158

674 352 425

674 386 64

674 388 112

674 400 305

674 413 1622

674 461 166

674 464 71

674 466 562

674 524 66

674 525 93

674 526 2291

674 528 245

674 532 2724

674 543 877

674 544 2145

674 686 74

674 689 365

674 793 116

674 972 77

674 1032 3245

674 1444 397

674 1451 1606

674 1452 108

674 1456 200

674 1918 402

674 1934 3109

674 2020 113

674 2081 101

674 2091 2052

674 2092 926

674 2094 1725

674 2117 1005

674 2521 226

674 2799 1078

674 4561 563

674 4814 113

674 5537 1390

674 5574 75

674 9198 1263

674 9199 1176

674 9200 403

674 9201 139

675 55 2383

675 67 3641

675 89 2196

675 103 615

675 167 438

675 257 479

675 288 1041

675 289 3277

675 290 547

675 292 1178

675 294 603

675 295 618

675 296 2372

675 299 404

675 300 411

675 301 430

675 306 454

675 323 859

675 349 526

675 352 1043

675 374 1316

675 375 843

675 423 449

675 425 889

675 466 603

675 530 446

675 532 1601

675 535 646

675 544 743

675 548 605

675 674 519

675 679 466

675 689 561

675 697 548

675 703 459

675 789 538

675 886 411

675 906 2994

675 1001 1254

675 1009 472

675 1934 758

675 2094 520

675 2102 1108

675 2521 442

675 2543 665

675 3490 403

675 3491 649

675 7445 409

675 9202 1471

675 9203 490

676 198 169

676 234 112

676 250 76

676 383 90

676 436 71

676 503 69

676 562 165

676 614 800

676 630 69

676 706 142

676 707 71

676 709 130

676 724 122

676 728 101

676 732 162

676 735 94

676 841 198

676 854 283

676 856 127

676 859 168

676 917 212

676 918 224

676 949 86

676 959 81

676 982 126

676 1119 71

676 1131 89

676 1202 132

676 1206 159

676 1261 71

676 1274 83

676 1281 99

676 1358 249

676 1360 143

676 1369 309

676 1412 377

676 1533 76

676 2343 227

676 2347 75

676 2369 153

676 2970 95

676 3476 242

676 4155 127

676 4218 86

676 4359 193

676 5157 69

676 5785 104

676 9204 133

676 9205 81

676 9206 69

677 89 754

677 161 183

677 289 332

677 310 140

677 917 230

677 949 424

677 954 316

677 957 296

677 1134 500

677 1206 294

677 1261 303

677 1264 307

677 1274 306

677 1353 132

677 1360	177
677 1521	167
677 1522	137
677 1531	171
677 1533	137
677 1963	175
677 2746	223
677 3509	375
677 3722	145
677 4266	514
677 4277	282
677 6258	872
677 6658	212
677 7917	222
677 7918	226
677 9207	1355
677 9208	800
677 9209	423
677 9210	321
677 9211	317
677 9212	300
677 9213	271
677 9214	258
677 9215	232
677 9216	228
677 9217	214
677 9218	166
677 9219	166
677 9220	164
677 9221	159

677 9222 158

677 9223 157

677 9224 155

677 9225 147

677 9226 140

677 9227 131

678 65 127

678 157 171

678 190 67

678 220 70

678 227 138

678 234 185

678 251 59

678 256 92

678 283 62

678 412 53

678 472 64

678 511 219

678 533 86

678 614 48

678 641 150

678 679 528

678 705 309

678 706 121

678 707 216

678 708 87

678 714 1019

678 725 46

678 917 72

678 951 119

678 959 130

678 966 69

678 982 744

678 1241 57

678 1247 53

678 1249 523

678 1375 270

678 1406 46

678 1412 45

678 1432 52

678 1470 124

678 1760 56

678 1771 50

678 1775 97

678 2346 235

678 2352 400

678 2852 63

678 3741 119

678 3859 68

678 4087 291

678 4849 81

678 4941 274

678 5772 51

678 9228 380

678 9229 136

678 9230 47

679 55 1728

679 67 736

679 89 911

679 157 173

679 285 289

679 288 446

679 289 4866

679 292 378

679 295 314

679 299 255

679 300 241

679 306 466

679 310 189

679 318 501

679 325 331

679 349 458

679 352 179

679 386 301

679 455 261

679 461 289

679 466 317

679 474 117

679 485 239

679 523 144

679 525 118

679 531 150

679 535 313

679 541 172

679 638 949

679 674 685

679 679 173

679 689 242

679 791 516

679 793 384

679 794 540

679 1503 143

679 1505 118

679 1580 109

679 2083 245

679 2094 379

679 3948 328

679 4563 461

679 4576 116

679 4770 157

679 4814 107

679 5953 184

679 8017 115

679 8718 543

679 9231 268

679 9232 146

680 55 596

680 89 1198

680 97 1557

680 172 716

680 288 1619

680 289 4759

680 291 1038

680 292 1198

680 293 1502

680 300 1220

680 302 572

680 318 3043

680 325 34674

680 328 916

680 357 877

680 360 720

680 375 1740

680 377 841

680 461 16108

680 465 631

680 466 1438

680 475 522

680 523 696

680 536 548

680 686 930

680 701 1898

680 797 887

680 811 3970

680 816 532

680 906 744

680 907 2305

680 908 1607

680 930 628

680 951 1111

680 1021 841

680 1601 526

680 1797 975

680 2094 639

680 2102 1093

680 2500 1910

680 3324 1265

680 3763 547

680 4156 1527

680 4562 560

680 4767 535

680 7390 3068

680 7448 1046

680 9233 1240

680 9234 732

680 9235 709

681 4 562

681 7 440

681 8 245

681 154 162

681 159 235

681 170 170

681 173 725

681 178 112

681 206 195

681 209 936

681 229 157

681 333 303

681 374 266

681 378 128

681 486 613

681 487 223

681 599 118

681 715 114

681 768 120

681 1090 224

681 1104 271

681 1179 635

681 1180 110

681 1181 230

681 1195 135

681 1375 319

681 1797 139

681 2430 112

681 2591 184

681 2815 200

681 2850 139

681 3208 143

681 3213 125

681 4103 173

681 5089 468

681 5969 194

681 8827 227

681 9236 1114

681 9237 899

681 9238 890

681 9239 348

681 9240 329

681 9241 284

681 9242 266

681 9243 212

681 9244 209

681 9245 200

681 9246 194

681 9247 172

681 9248 172

682 55 359

682 89 429

682 190 241

682 256 198

682 288 494

682 289 355

682 295 169

682 300 1109

682 304 373

682 306 587

682 314 323

682 324 408

682 327 150

682 328 340

682 333 210

682 342 830

682 344 4126

682 456 202

682 458 192

682 461 313

682 464 306

682 466 691

682 468 116

682 486 291

682 498 471

682 531 114

682 538 513

682 544 373

682 679 334

682 681 173

682 683 500

682 686 183

682 691 398

682 704 331

682 797 152

682 1038 114

682 1198 179

682 1428 301

682 1447 120

682 1452 180

682 1470 125

682 2545 167

682 2977 171

682 2988 123

682 3378 139

682 5749 202

682 5950 264

682 8093 137

682 9249 1764

682 9250 333

683 51 3127

683 59 438

683 61 392

683 68 1683

683 72 411

683 85 478

683 98 3774

683 99 1186

683 159 676

683 599 651

683 874 1667

683 985 448

683 993 558

683 995 495

683 997 1481

683 998 514

683 1013	4237
683 1027	562
683 1079	881
683 1083	1135
683 1799	1352
683 1878	392
683 1882	452
683 1885	1076
683 1886	3627
683 1892	356
683 1893	774
683 1895	2312
683 1897	1944
683 2559	887
683 2936	1205
683 2937	467
683 3280	1061
683 3431	481
683 3920	2998
683 5260	471
683 5833	988
683 5917	575
683 5918	1847
683 6065	1833
683 6073	558
683 6940	722
683 7343	407
683 7347	499

683 9251 691

683 9252 594

683 9253 569

683 9254 518

683 9255 437

683 9256 433

684 67 4429

684 161 761

684 257 1269

684 288 31229

684 289 49889

684 290 294

684 291 613

684 292 15137

684 294 872

684 295 10213

684 296 935

684 299 2899

684 300 7044

684 301 453

684 302 1778

684 306 647

684 309 269

684 318 878

684 323 1126

684 325 638

684 327 370

684 328 485

684 331 317

684 332 459

684 333 2584

684 344 2086

684 349 1475

684 352 1229

684 461 5636

684 466 2759

684 468 562

684 475 749

684 498 372

684 523 3223

684 525 279

684 543 1367

684 544 449

684 686 264

684 701 535

684 704 267

684 972 818

684 1032 345

684 1037 2636

684 1451 3548

684 2020 874

684 3397 381

684 4814 426

684 9257 985

684 9258 809

684 9259 788

685 7 1643

685 67 647

685 89 1465

685 188 1125

685 195 583

685 198 2460

685 199 1013

685 206 733

685 209 1153

685 212 585

685 220 667

685 226 738

685 227 1389

685 233 2859

685 234 1014

685 278 1001

685 300 1122

685 333 1281

685 344 2491

685 466 548

685 512 1006

685 517 1005

685 554 1015

685 614 1011

685 706 1803

685 707 3434

685 718 2904

685 841 2938

685 843 1301

685 856 604

685 857 1652

685 917 1028

685 918 1085

685 964 1025

685 1261 1226

685 1369 1013

685 1412 1430

685 1513 1018

685 1547 1838

685 1613 592

685 1687 546

685 1974 2008

685 2083 574

685 2347 1036

685 2532 737

685 2546 1103

685 3239 1180

685 5667 1007

685 8945 1405

685 9260 660

686 9 1954

686 32 804

686 49 949

686 51 719

686 58 730

686 72 19417

686 91 3443

686 153 579

686 159 586

686 171 849

686 173 2563

686 190 8762

686 227 1362

686 233 1582

686 441 535

686 450 553

686 486 6697

686 599 1174

686 854 486

686 880 1568

686 959 1688

686 965 2840

686 982 2589

686 997 808

686 998 605

686 1001 668

686 1014 841

686 1019 833

686 1072 863

686 1075 1263

686 1079 711

686 1098 1394

686 1151 1034

686 1294 551

686 1295 1431

686 1332 503

686 1378 521

686 1379 1275

686 1713 3054

686 3191 2318

686 3201 1036

686 3988 2670

686 4312 824

686 6926 1029

686 8915	486

686 9261	1363

686 9262	1256

686 9263	769

686 9264	654

686 9265	489

687 46	23

687 599 112

687 735 59

687 867 12

687 868 4

687 875 49

687 945 2

687 959 1

687 1013	4

687 1079	28

687 1083	5

687 1123	1

687 1150	5

687 1205	10

687 1206	23

687 1358	3

687 1510	1

687 1534	48

687 1703	1

687 1804	7

687 1813	2

687 1886	1

687 2488	5

687 2784	3

687 2861 1

687 3279 25

687 3431 33

687 3432 2

687 3435 3

687 3436 194

687 4541 3

687 4807 5

687 5260 18

687 5787 5

687 5915 17

687 7348 3

687 8283 3

687 8291 74

687 8668 28

687 9266 13

687 9267 13

687 9268 12

687 9269 9

687 9270 4

687 9271 3

687 9272 3

687 9273 3

687 9274 2

687 9275 2

687 9276 2

688 55 132

688 65 1626

688 67 135

688 70 191

688 89 284

688 95 122

688 99 135

688 182 1521

688 184 147

688 190 1484

688 207 390

688 209 1082

688 220 158

688 227 1125

688 228 270

688 229 1998

688 230 172

688 288 134

688 292 210

688 316 407

688 329 374

688 333 435

688 344 502

688 349 160

688 362 301

688 377 216

688 378 167

688 412 127

688 424 473

688 428 135

688 455 155

688 466 147

688 498 164

688 511 1530

688 528 173

688 533 242

688 679 689

688 681 1213

688 689 134

688 885 544

688 1048 384

688 1090 179

688 1249 245

688 1377 138

688 1934 190

688 2130 243

688 4818 215

688 7194 1325

688 7888 246

688 9277 217

689 55 145

689 67 311

689 89 3154

689 161 24

689 180 140

689 288 70

689 289 484

689 292 75

689 295 16

689 299 25

689 300 76

689 304 34

689 306 108

689 311 17

689 317 30

689 325 18

689 326 42

689 328 38

689 333 1067

689 344 354

689 352 188

689 461 86

689 466 192

689 486 18

689 531 17

689 542 50

689 543 64

689 544 81

689 679 681

689 701 34

689 982 40

689 1452 10

689 1456 198

689 1795 56

689 2046 9

689 2067 10

689 2082 19

689 2548 186

689 3311 17

689 3393 51

689 3741 21

689 4912 43

689 5988 32

689 7864 12

689 9278 175

689 9279 32

689 9280 20

689 9281 16

689 9282 11

689 9283 10

690 55 3185

690 58 348

690 67 5390

690 89 1642

690 167 320

690 229 1141

690 257 1347

690 261 1097

690 285 739

690 288 1537

690 289 2134

690 291 199

690 292 3199

690 294 324

690 295 307

690 299 3623

690 300 1824

690 301 458

690 302 335

690 306 177

690 310 1055

690 333 183

690 340 183

690 344 209

690 352 1745

690 389 286

690 461 189

690 466 686

690 525 584

690 535 743

690 543 533

690 544 229

690 641 302

690 686 176

690 701 1622

690 787 1033

690 790 734

690 792 904

690 802 319

690 889 546

690 906 684

690 915 201

690 1037 384

690 1090 236

690 1098 2159

690 2083 1746

690 2521 852

690 4260 173

690 5533 169

690 8409 205

691 58 615

691 64 915

691 70 490

691 89 1811

691 97 404

691 173 2656

691 190 1421

691 204 465

691 228 557

691 238 526

691 257 400

691 288 2223

691 289 978

691 292 13499

691 295 582

691 298 485

691 300 1002

691 301 425

691 302 589

691 326 574

691 331 1005

691 333 1042

691 344 800

691 352 635

691 425 661

691 466 484

691 498 4716

691 499 812

691 506 620

691 548 1939

691 689 1746

691 701 1400

691 703 1110

691 704 723

691 722 675

691 930 602

691 1037 456

691 1075 669

691 1104 416

691 1379 449

691 1854 391

691 2521 587

691 2542 388

691 2608 425

691 3411 871

691 4153 530

691 5736 530

691 8701 784

691 9284 516

691 9285 486

692 100 401

692 157 6615

692 227 14154

692 585 693

692 997 386

692 1002 956

692 1073 460

692 1123 797

692 1242 5983

692 1244 12182

692 1414 1172

692 1416 636

692 1464 5046

692 1465 2495

692 1469	416
692 1686	656
692 1820	429
692 1825	756
692 2459	596
692 2523	447
692 2902	1127
692 2936	618
692 3065	872
692 3313	579
692 6076	498
692 6703	538
692 6708	2764
692 7935	5332
692 8545	734
692 8546	545
692 8552	1856
692 8812	412
692 9286	2395
692 9287	2229
692 9288	1891
692 9289	897
692 9290	891
692 9291	743
692 9292	689
692 9293	671
692 9294	655
692 9295	639
692 9296	636
692 9297	594

692 9298 566
692 9299 425
692 9300 414
692 9301 400
692 9302 399
692 9303 398
693 195 103
693 217 117
693 222 347
693 223 252
693 225 206
693 228 222
693 412 136
693 426 767
693 429 126
693 466 333
693 526 89
693 532 139
693 615 538
693 679 1047
693 886 267
693 898 564
693 1389 170
693 1481 255
693 1632 867
693 2264 221
693 2415 197
693 2534 91
693 2542 116
693 3279 165

693 3616 93

693 3884 156

693 4074 508

693 4079 112

693 4384 126

693 4704 160

693 5926 452

693 6229 415

693 6422 341

693 8274 470

693 9304 389

693 9305 377

693 9306 354

693 9307 326

693 9308 275

693 9309 260

693 9310 254

693 9311 187

693 9312 179

693 9313 174

693 9314 151

693 9315 120

693 9316 119

693 9317 102

693 9318 90

693 9319 88

694 30 264

694 51 332

694 59 92

694 72 559

694 99 190

694 159 100

694 382 303

694 429 129

694 709 145

694 857 180

694 859 332

694 865 115

694 868 180

694 1001 179

694 1067 1413

694 1104 260

694 1298 554

694 1412 168

694 1878 117

694 1884 101

694 2014 234

694 2531 137

694 2938 725

694 3281 153

694 3436 526

694 3451 119

694 3781 96

694 3988 548

694 4025 117

694 4129 124

694 4732 273

694 5254 105

694 5260 919

694 7069 95

694 7325 282

694 8014 202

694 8910 416

694 9320 343

694 9321 292

694 9322 280

694 9323 276

694 9324 228

694 9325 213

694 9326 166

694 9327 143

694 9328 138

694 9329 137

694 9330 110

694 9331 107

694 9332 106

695 65 19

695 154 69

695 162 55

695 163 47

695 171 21

695 221 23

695 533 34

695 607 19

695 621 96

695 632 72

695 748 98

695 756 55

695 851 32

695 860 20

695 1366	53
695 1384	21
695 1418	28
695 1655	22
695 1808	19
695 1812	24
695 2347	17
695 2572	17
695 2676	33
695 2677	29
695 2973	55
695 3095	22
695 3676	50
695 4232	34
695 5160	41
695 6159	41
695 6189	77
695 6582	18
695 6759	19
695 6762	92
695 7147	17
695 8423	50
695 9333	138
695 9334	39
695 9335	38
695 9336	35
695 9337	35
695 9338	32
695 9339	23
695 9340	21

695 9341 21

695 9342 20

695 9343 19

695 9344 18

695 9345 18

695 9346 17

696 96 636

696 176 67

696 212 81

696 220 197

696 227 412

696 234 77

696 562 157

696 607 147

696 706 206

696 717 98

696 728 142

696 730 201

696 734 77

696 735 143

696 868 145

696 945 79

696 951 590

696 959 414

696 966 107

696 982 86

696 1249 524

696 1372 192

696 1412 991

696 1415 122

696 1803 70

696 2013 76

696 2183 130

696 2346 119

696 2347 75

696 2525 161

696 2783 234

696 2789 102

696 2854 209

696 2855 94

696 3777 73

696 3888 593

696 4165 71

696 4369 105

696 5020 305

696 5844 68

696 6291 69

696 6292 97

696 8102 119

696 8743 78

696 9347 525

696 9348 444

696 9349 270

696 9350 154

696 9351 91

696 9352 84

697 52 443

697 53 365

697 56 591

697 57 636

697 63 947

697 64 569

697 80 666

697 151 421

697 174 736

697 175 502

697 238 549

697 329 358

697 377 505

697 497 403

697 744 489

697 747 1367

697 748 6886

697 782 1006

697 1099 680

697 1226 729

697 1620 431

697 1793 771

697 2030 387

697 2605 805

697 2676 1256

697 2677 475

697 2691 1300

697 2711 750

697 3411 705

697 3863 512

697 3992 493

697 4865 471

697 5072 366

697 5329 667

697 5346 479

697 5358 791

697 5360 678

697 5700 918

697 5803 445

697 8513 416

697 9353 853

697 9354 795

697 9355 792

697 9356 595

697 9357 581

697 9358 549

697 9359 468

697 9360 425

697 9361 372

697 9362 359

698 63 2677

698 67 1108

698 70 3118

698 81 967

698 157 4174

698 184 1113

698 241 935

698 257 4462

698 265 2641

698 279 1441

698 288 774

698 289 1028

698 291 921

698 292 1183

698 294 1244

698 295 1609

698 309 919

698 377 1541

698 378 1387

698 389 3642

698 485 1709

698 518 1853

698 727 3731

698 972 854

698 1098 2543

698 1114 1568

698 1244 1831

698 1705 965

698 1743 751

698 1921 1003

698 2044 1564

698 2045 3835

698 2047 1643

698 2051 4890

698 2052 1068

698 3067 2635

698 4912 3011

698 6052 2874

698 6250 1084

698 7256 1504

698 7862 1414

698 8854 7528

698 9363 12091

698 9364 2956

698 9365 2484

698 9366 1553

698 9367 1229

698 9368 1186

698 9369 1030

698 9370 822

699 51 1010

699 72 2354

699 98 292

699 99 649

699 159 1068

699 176 514

699 187 253

699 193 577

699 227 316

699 429 314

699 706 409

699 707 287

699 852 557

699 859 355

699 861 245

699 917 828

699 982 693

699 993 236

699 997 557

699 998 332

699 1008 430

699 1013 890

699 1027 229

699 1073 253

699 1132 855

699 1351 255

699 1358 414

699 1360 779

699 1369 416

699 1372 396

699 1415 368

699 1803 237

699 1878 249

699 1881 726

699 1891 297

699 1892 282

699 1899 279

699 2013 564

699 2029 547

699 2342 221

699 2343 417

699 2347 668

699 2624 358

699 2853 333

699 2901 715

699 3476 371

699 3777 736

699 6541 931

699 6957 492

699 6959 372

700 89 1357

700 161 493

700 191 492

700 228 269

700 257 1248

700 288 2565

700 289 1039

700 290 353

700 291 726

700 294 285

700 295 1695

700 300 1251

700 304 274

700 306 1814

700 324 423

700 325 399

700 327 801

700 328 465

700 329 290

700 330 445

700 331 495

700 333 571

700 344 1492

700 349 947

700 355 319

700 377 503

700 458 472

700 461 386

700 464 410

700 466 429

700 468 1139

700 474 312

700 475 1200

700 527 360

700 531 446

700 538 1167

700 545 506

700 691 897

700 907 536

700 1042 274

700 1449 433

700 1455 434

700 1602 342

700 1613 408

700 2176 538

700 3317 1243

700 3318 577

700 3859 468

700 4562 660

700 7340 782

701 89 3017

701 161 141

701 257 67

701 288 1408

701 289 720

701 292 101

701 295 62

701 300 570

701 306 128

701 329 474

701 466 97

702 67 801

702 89 745

702 157 964

702 161 548

702 257 1035

702 262 1621

702 285 685

702 288 5238

702 289 1137

702 290 458

702 292 1074

702 295 1326

702 299 1092

702 301 449

702 302 514

702 306 1369

702 310 709

702 314 4119

702 317 780

702 320 870

702 322 724

702 325 601

702 327 478

702 328 426

702 329 2069

702 332 792

702 333 1473

702 349 530

702 352 462

702 455 440

702 456 1000

702 466 619

702 475 1052

702 476 484

702 481 434

702 484 688

702 498 505

702 525 506

702 528 417

702 699 521

702 907 440

702 908 575

702 1457 906

702 2220 831

702 2977 1366

702 3498 1067

702 4560 618

702 5953 917

702 5988 998

702 9371 465

703 55 92

703 67 7

703 89 9670

703 246 12

703 283 6

703 288 1570

703 289 6109

703 292 1605

703 295 25

703 299 17

703 300 1091

703 302 29

703 306 22

703 320 31

703 328 11

703 333 154

703 349 6

703 355 9

703 458 15

703 461 114

703 466 1345

703 475 26

703 543 12

703 679 320

703 790 18

703 802 11

703 903 35

703 1032 50

703 1043 11

703 1246 8

703 1452 45

703 1455 19

703 2176 10

703 2543 121

703 4463 27

703 4464 13

703 4577 9

703 5268 13

703 6722 10

703 9169 23

703 9372 20

703 9373 16

703 9374 13

703 9375 12

703 9376 11

703 9377 10

703 9378 9

703 9379 8

703 9380 7

703 9381 7

704 89 8

704 278 35

704 300 73

704 302 39

704 327 7

704 330 36

704 331 58

704 333 7

704 466 28

704 475 22867

704 527 276

704 1037 84

704 1245 17

704 1444 35

704 1455 88

704 1613 69

704 1641 33

704 2179 36

704 2226 38

704 2236 20

704 2522 6

704 2906 34

704 3317 152

704 4617 21

704 4983 30

704 6501 9

704 6567 10

704 6618 264

704 6619 42

704 6620 10

704 6621 64

704 8160 33

704 8162 10

704 9382 1027

704 9383 532

704 9384 50

704 9385 20

704 9386 19

704 9387 18

704 9388 16

704 9389 16

704 9390 15

704 9391 14

704 9392 11

704 9393 10

704 9394 9

704 9395 9

704 9396 9

704 9397 8

704 9398 7

705 56 479

705 89 1135

705 190 655

705 257 1727

705 289 1199

705 290 601

705 291 993

705 292 1117

705 297 617

705 299 668

705 300 1535

705 302 1618

705 349 736

705 377 577

705 455 1242

705 481 1388

705 498 1056

705 528 515

705 679 855

705 682 1198

705 691 922

705 786 532

705 891 770

705 910 821

705 912 666

705 1201 707

705 1459 530

705 1705 1441

705 2071 547

705 2446 779

705 2478 2509

705 4747 485

705 4750 1293

705 4918 2857

705 4920 563

705 4923 1689

705 4927 668

705 5605 549

705 6397 727

705 7170 541

705 8361 682

705 8962 574

705 9399 1189

705 9400 780

705 9401 690

705 9402 670

705 9403 634

705 9404 571

705 9405 504

705 9406 498

706 7 1244

706 15 116

706 72 13

706 154 16

706 155 25

706 182 9

706 186 154

706 190 8

706 198 18

706 209 55

706 234 1054

706 370 122

706 486 9

706 497 68

706 504 46

706 506 32

706 517 8

706 707 26

706 813 79

706 841 40

706 843 12

706 903 14

706 918 11

706 957 16

706 965 58

706 1044 14

706 1052 8

706 1143 236

706 1274 19

706 1412 14

706 1814 9

706 2167 20

706 2370 32

706 2374 145

706 2794 36

706 2970 31

706 3405 35

706 3462 9

706 4675 21

706 8722 26

706 9407 22

706 9408 19

706 9409 19

706 9410 17

706 9411 16

706 9412 14

706 9413 14

706 9414 13

706 9415 12

706 9416 11

707 302 11

707 342 17

707 389 19

707 717 14

707 730 31

707 945 13

707 963 19

707 1005 14

707 1447 14

707 1459 14

707 1468 14

707 2054 13

707 2183 14

707 2463 25

707 2982 13

707 3081 15

707 3482 12

707 3488 14

707 3777 15

707 3921 12

707 5168 14

707 5452 11

707 5978 11

707 6920	14
707 7869	11
707 8312	17
707 8563	17
707 8853	11
707 9347	10
707 9417	24
707 9418	23
707 9419	23
707 9420	20
707 9421	19
707 9422	17
707 9423	15
707 9424	15
707 9425	15
707 9426	14
707 9427	14
707 9428	13
707 9429	13
707 9430	13
707 9431	13
707 9432	12
707 9433	11
707 9434	11
707 9435	11
707 9436	11
707 9437	10
708 226 2	
708 751 3	
708 752 6	

708 763 2	
708 841 5	
708 856 3	
708 917 8	
708 1119	11
708 1202	14
708 1221	3
708 1229	3
708 1369	5
708 1418	2
708 2473	14
708 2493	3
708 2593	7
708 2624	3
708 3351	9
708 3444	3
708 3505	10
708 3508	2
708 3531	2
708 3694	9
708 3809	2
708 4081	12
708 5299	3
708 6126	4
708 7081	8
708 7376	2
708 9438	8
708 9439	6
708 9440	6
708 9441	6

708 9442 4

708 9443 4

708 9444 3

708 9445 3

708 9446 3

708 9447 3

708 9448 3

708 9449 3

708 9450 3

708 9451 3

708 9452 3

708 9453 3

708 9454 3

708 9455 2

708 9456 2

708 9457 2

708 9458 2

709 176 69

709 582 144

709 706 3237

709 724 203

709 732 71

709 868 123

709 917 290

709 918 455

709 945 41

709 956 764

709 959 44

709 1079 40

709 1249 384

709 1358	122
709 1366	97
709 1369	219
709 1372	211
709 1412	83
709 1415	199
709 1639	43
709 1803	3059
709 2183	40
709 2341	389
709 2342	715
709 2343	1839
709 2347	716
709 2463	178
709 2558	45
709 2787	145
709 2790	53
709 2834	91
709 2838	1902
709 3993	83
709 4458	349
709 5008	52
709 5156	45
709 5820	844
709 5844	386
709 6959	144
709 8795	48
709 9274	139
709 9334	84
709 9459	631

709 9460 439

709 9461 288

709 9462 253

709 9463 224

709 9464 158

709 9465 102

709 9466 43

710 59 127

710 72 97

710 154 54

710 159 260

710 172 152

710 190 40

710 193 37

710 230 41

710 234 258

710 316 38

710 425 45

710 429 229

710 436 380

710 441 341

710 599 76

710 612 61

710 720 48

710 854 168

710 857 164

710 859 131

710 985 71

710 1017 38

710 1083 111

710 1206	47
710 1239	35
710 1504	178
710 1513	81
710 1645	40
710 1814	227
710 1817	81
710 2542	177
710 2938	77
710 3011	34
710 3109	43
710 3110	97
710 3279	38
710 3333	34
710 3444	119
710 3739	335
710 3744	136
710 4092	100
710 5306	38
710 7325	42
710 9332	87
710 9338	35
710 9344	57
710 9467	116
710 9468	84
710 9469	80
710 9470	52

711 163 73

711 176 39

711 250 46

711 377 62

711 378 43

711 489 40

711 500 211

711 504 507

711 517 416

711 706 77

711 707 238

711 841 72

711 917 1035

711 951 23

711 952 48

711 1044 256

711 1249 677

711 1254 30

711 1338 125

711 1360 37

711 1369 44

711 1412 60

711 1639 120

711 1803 48

711 1804 452

711 1808 242

711 1810 1736

711 2343 205

711 2346 26

711 2347 57

711 2380 25

711 2463 31

711 2595 65

711 2787	270
711 2790	372
711 2901	27
711 3083	238
711 3239	45
711 4165	235
711 4180	40
711 4280	24
711 4460	575
711 5015	226
711 6259	41
711 9334	319
711 9471	281
711 9472	97
711 9473	61
711 9474	23
711 9475	23
712 204 1	
712 212 1	
712 214 1	
712 221 2	
712 309 1	
712 682 1	
712 716 3	
712 851 1	
712 1048	1
712 1298	1
712 1515	1
712 1609	1
712 1633	1

712 2127	1
712 3255	1
712 3676	1
712 5471	1
712 5547	1
712 6229	1
712 6920	1
712 9476	5
712 9477	3
712 9478	2
712 9479	1
712 9480	1
712 9481	1
712 9482	1
712 9483	1
712 9484	1
712 9485	1
712 9486	1
712 9487	1
712 9488	1
712 9489	1
712 9490	1
712 9491	1
712 9492	1
712 9493	1
713 55	1522
713 67	428
713 89	447
713 161	850
713 257	608

713 279 196

713 285 1362

713 288 3268

713 289 8696

713 292 724

713 293 532

713 295 1354

713 299 930

713 300 270

713 301 1720

713 302 296

713 306 227

713 309 248

713 310 375

713 312 523

713 314 573

713 318 577

713 320 197

713 322 267

713 329 280

713 332 569

713 333 176

713 349 498

713 455 283

713 456 154

713 465 1130

713 468 423

713 476 3700

713 484 223

713 485 392

713 523 294

713 525 149

713 543 589

713 701 738

713 786 258

713 907 203

713 1451 182

713 1475 1185

713 1580 141

713 1596 518

713 4292 244

713 4487 497

713 6308 161

713 9494 248

713 9495 166

714 16 148

714 40 85

714 51 439

714 72 273

714 98 72

714 159 224

714 233 112

714 441 73

714 450 79

714 487 155

714 515 90

714 862 82

714 875 119

714 997 99

714 1075 433

714 1083	83
714 1294	72
714 1787	118
714 1886	178
714 1897	115
714 2938	103
714 3186	72
714 3196	94
714 3432	98
714 3769	86
714 4025	191
714 4038	127
714 4069	91
714 4382	102
714 4732	103
714 5586	97
714 5709	209
714 5917	76
714 6847	115
714 7516	98
714 8369	119
714 9256	98
714 9496	120
714 9497	115
714 9498	115
714 9499	106
714 9500	104
714 9501	96
714 9502	89
714 9503	88

714 9504 86

714 9505 85

714 9506 85

714 9507 81

714 9508 73

715 15 198

715 163 250

715 181 244

715 227 238

715 707 2753

715 710 199

715 724 239

715 841 222

715 917 14003

715 919 186

715 920 379

715 922 528

715 928 488

715 930 205

715 937 440

715 943 196

715 946 225

715 957 431

715 1257 183

715 1338 551

715 1339 214

715 1350 302

715 1358 2288

715 1369 473

715 1372 323

715 1415 324

715 1810 189

715 2342 520

715 2973 224

715 3476 316

715 3636 430

715 4331 260

715 4341 286

715 5016 266

715 5157 410

715 5485 434

715 5694 305

715 5724 186

715 5841 222

715 6273 184

715 6748 314

715 7042 504

715 7930 195

715 8163 192

715 9509 439

715 9510 386

715 9511 246

715 9512 229

715 9513 210

715 9514 186

716 65 1169

716 209 941

716 298 1355

716 377 1545

716 412 1233

716 486 1105

716 494 1345

716 498 1946

716 507 1305

716 512 1095

716 537 1848

716 540 1521

716 682 1465

716 687 1140

716 779 1633

716 805 1002

716 808 1804

716 813 1142

716 815 1359

716 816 3621

716 823 1806

716 830 1435

716 1046 4300

716 1116 1199

716 1181 1948

716 1187 2472

716 1192 2262

716 1195 1701

716 1197 2506

716 1200 1191

716 1541 2301

716 1853 1439

716 1858 1514

716 1868 1428

716 2608 2005

716 2619 1338

716 2723 2302

716 2847 2439

716 2850 2806

716 2959 1624

716 3616 1200

716 3817 1727

716 4099 1826

716 4262 1957

716 4385 1044

716 8274 1248

716 8464 1803

716 8473 957

716 9515 2451

716 9516 1006

717 55 3203

717 67 1820

717 89 448

717 157 1294

717 257 479

717 288 251

717 289 72776

717 291 239

717 292 6242

717 293 235

717 295 670

717 298 2184

717 299 767

717 300 1596

717 301 400

717 302 795

717 306 1300

717 310 325

717 311 287

717 314 258

717 318 242

717 320 242

717 325 1685

717 333 698

717 344 380

717 349 714

717 377 579

717 461 632

717 464 409

717 466 986

717 525 249

717 528 2787

717 542 1409

717 544 390

717 680 323

717 701 387

717 903 1746

717 906 240

717 908 708

717 972 4891

717 1032 243

717 1450 312

717 1451 430

717 1556 915

717 2025 622

717 2094 380

717 3177 326

717 9517 524

717 9518 344

717 9519 243

718 53 704

718 56 159

718 58 277

718 59 426

718 64 349

718 72 764

718 84 256

718 88 332

718 154 902

718 173 301

718 190 249

718 206 756

718 207 321

718 210 164

718 237 207

718 238 280

718 603 343

718 616 175

718 757 218

718 887 178

718 1062 1098

718 1098 577

718 1155 692

718 1400 622

718 1632 164

718 1745	166
718 1752	166
718 1954	183
718 1969	188
718 1983	1279
718 1990	293
718 2083	441
718 2271	177
718 2289	179
718 2535	155
718 2675	221
718 3186	212
718 3196	199
718 3200	708
718 3277	197
718 3693	479
718 3892	188
718 3995	645
718 4025	230
718 4731	213
718 4732	269
718 5926	238
718 7470	220
718 8215	223
718 9520	146

719 163 1740

719 227 125

719 562 142

719 607 633

719 706 652

719 724 67

719 728 64

719 730 285

719 868 70

719 917 200

719 918 61

719 954 60

719 956 41

719 1110 1390

719 1343 78

719 1358 2879

719 1369 3398

719 1372 1783

719 1394 877

719 1415 83

719 1513 4970

719 1545 715

719 1974 1311

719 2342 11022

719 2344 56

719 2346 1086

719 2347 728

719 2787 775

719 2834 130

719 3470 1987

719 3476 186

719 4782 50

719 5172 129

719 5688 1355

719 5692 40

719 6257 53

719 6363 211

719 7791 67

719 7792 119

719 7927 65

719 8795 62

719 9521 136

719 9522 134

719 9523 96

719 9524 69

719 9525 67

719 9526 45

719 9527 44

719 9528 43

719 9529 40

720 162 32

720 163 684

720 176 140

720 188 238

720 192 62

720 227 135

720 234 26

720 316 152

720 506 33

720 630 24

720 706 68

720 707 83

720 712 34

720 717 36

720 718 67

720 730 95

720 735 36

720 917 51

720 920 25

720 949 83

720 951 28

720 957 37

720 959 143

720 975 139

720 992 35

720 1106 168

720 1122 51

720 1134 41

720 1145 29

720 1249 73

720 1358 237

720 1369 31

720 1412 148

720 1415 49

720 1513 25

720 2013 45

720 2265 110

720 2444 94

720 2787 164

720 2935 55

720 3308 93

720 3482 76

720 4125 40

720 4620 64

720 7100 61

720 9530 134

720 9531 68

720 9532 39

720 9533 38

720 9534 29

721 195 1363

721 197 414

721 205 602

721 211 678

721 212 757

721 217 454

721 220 194

721 222 324

721 425 169

721 426 756

721 436 800

721 437 196

721 442 190

721 445 213

721 614 255

721 618 1265

721 622 315

721 630 428

721 709 161

721 1382 173

721 1687 565

721 1732 207

721 1944 2213

721 1947 174

721 2640 186

721 3057	397
721 3258	318
721 3619	171
721 4432	294
721 4532	570
721 6206	181
721 6224	183
721 6302	251
721 6305	402
721 6685	157
721 7406	156
721 8109	299
721 8594	265
721 8776	193
721 9535	654
721 9536	262
721 9537	260
721 9538	258
721 9539	225
721 9540	200
721 9541	178
721 9542	166
721 9543	162
721 9544	158
721 9545	153
722 55	856
722 67	647
722 89	522
722 172	766
722 187	131

722 203 234

722 220 150

722 230 514

722 257 235

722 288 953

722 289 4170

722 292 1601

722 293 1678

722 295 158

722 299 125

722 300 822

722 302 1080

722 306 530

722 310 819

722 318 376

722 324 191

722 325 303

722 326 174

722 329 209

722 333 2871

722 344 448

722 349 407

722 360 210

722 386 243

722 455 649

722 459 390

722 461 1672

722 464 192

722 466 1182

722 475 296

722 498 675

722 523 710

722 525 2637

722 534 9010

722 544 171

722 545 212

722 701 328

722 704 363

722 790 135

722 793 136

722 972 468

722 1034 173

722 1580 359

722 2523 210

722 5926 394

723 55 65

723 67 59

723 89 514

723 157 138

723 184 69

723 190 49

723 257 127

723 288 1353

723 289 1540

723 290 42

723 292 3643

723 294 71

723 295 463

723 298 137

723 299 149

723 300 290

723 302 51

723 309 51

723 310 78

723 313 324

723 318 87

723 324 44

723 328 51

723 332 41

723 333 247

723 342 560

723 344 1768

723 349 154

723 352 43

723 355 565

723 461 63

723 466 411

723 475 77

723 531 65

723 544 2313

723 679 82

723 691 69

723 701 14306

723 1032 48

723 1459 69

723 1475 146

723 2052 47

723 2805 49

723 3173 173

723 4618 72

723 5472 58
723 9546 70
723 9547 53
723 9548 53
723 9549 44
725 63 350
725 72 84
725 81 142
725 163 821
725 407 94
725 592 239
725 607 64
725 744 310
725 1073 263
725 1104 334
725 1121 4313
725 1123 357
725 1146 194
725 1154 111
725 1225 95
725 1705 82
725 1806 87
725 1909 121
725 2469 123
725 2478 201
725 2626 94
725 2634 70
725 3886 65
725 4126 77
725 4922 281

725 5073	213
725 7054	127
725 7124	199
725 7126	154
725 7133	85
725 7148	105
725 7431	63
725 7488	98
725 9289	95
725 9550	183
725 9551	178
725 9552	177
725 9553	130
725 9554	128
725 9555	124
725 9556	124
725 9557	106
725 9558	89
725 9559	87
725 9560	84
725 9561	82
725 9562	77
725 9563	75
725 9564	75
725 9565	73
726 288 5	
726 295 5	
726 296 3	
726 374 31	
726 375 348	

726 379 1

726 404 21

726 413 5

726 1148	1
726 1253	1
726 1918	1
726 1923	4
726 2083	1
726 2088	33
726 2094	6
726 2097	38
726 2101	1
726 2111	27
726 3324	6
726 3490	15
726 3501	114
726 3991	1
726 4558	2
726 5094	1
726 5572	9
726 5767	1
726 9092	2
726 9566	140
726 9567	39
726 9568	31
726 9569	31
726 9570	31
726 9571	8
726 9572	8
726 9573	6

726 9574 5

726 9575 3

726 9576 3

726 9577 2

726 9578 1

726 9579 1

726 9580 1

726 9581 1

726 9582 1

726 9583 1

726 9584 1

726 9585 1

726 9586 1

726 9587 1

726 9588 1

727 245 751

727 250 1230

727 718 324

727 857 384

727 1207 336

727 1645 365

727 3147 354

727 3657 845

727 4536 392

727 8121 870

727 9589 1282

727 9590 1231

727 9591 1225

727 9592 1221

727 9593 1041

727 9594	771
727 9595	749
727 9596	737
727 9597	695
727 9598	661
727 9599	656
727 9600	643
727 9601	619
727 9602	572
727 9603	535
727 9604	477
727 9605	444
727 9606	400
727 9607	392
727 9608	389
727 9609	370
727 9610	369
727 9611	364
727 9612	360
727 9613	350
727 9614	342
727 9615	340
727 9616	313
727 9617	302
727 9618	294
727 9619	291
727 9620	290
727 9621	289
727 9622	286
727 9623	283

727 9624 273

727 9625 271

727 9626 263

727 9627 249

727 9628 248

728 7 712

728 59 900

728 72 788

728 76 828

728 159 1564

728 207 588

728 225 948

728 226 1458

728 229 792

728 233 1479

728 425 680

728 436 1934

728 450 967

728 511 1876

728 517 2083

728 599 567

728 715 831

728 860 603

728 862 698

728 869 580

728 969 1496

728 1089 704

728 1151 626

728 1164 571

728 1240 680

728	1376	810
728	1510	1130
728	1787	1021
728	1886	673
728	1985	1203
728	2531	2166
728	3290	734
728	3941	887
728	4022	621
728	4058	1526
728	4069	3668
728	4316	608
728	4435	751
728	4451	675
728	5890	1448
728	6728	2739
728	6730	968
728	6920	899
728	7074	621
728	7128	571
728	9629	1291
728	9630	760
728	9631	758
728	9632	756
728	9633	600
729	18	59
729	30	232
729	50	24
729	65	267
729	72	888

729 89 36

729 159 503

729 173 1234

729 215 64

729 228 64

729 229 125

729 302 26

729 450 66

729 930 26

729 982 166

729 1052 34

729 1079 109

729 1104 36

729 1114 23

729 1239 17

729 1295 413

729 1298 1194

729 1299 16

729 1369 18

729 1463 22

729 1534 293

729 1704 298

729 1711 31

729 1816 20

729 2038 25

729 3057 22

729 3184 49

729 3310 27

729 3546 16

729 4872 19

729 5714	39
729 7457	19
729 9263	66
729 9634	2706
729 9635	120
729 9636	46
729 9637	26
729 9638	25
729 9639	22
729 9640	22
729 9641	21
729 9642	20
729 9643	19
729 9644	19
729 9645	17
730 55	148
730 67	10020
730 72	155
730 86	1152
730 89	3717
730 154	1846
730 163	175
730 185	1032
730 190	165
730 209	2355
730 227	16381
730 230	194
730 258	124
730 288	734
730 289	4376

730 292 4559

730 295 758

730 300 177

730 310 123

730 314 478

730 333 752

730 340 115

730 344 1018

730 352 120

730 357 716

730 461 4274

730 466 504

730 498 1072

730 511 738

730 562 496

730 563 114

730 704 1061

730 735 18841

730 868 171

730 874 1097

730 903 124

730 1104 327

730 1122 123

730 1412 578

730 1414 388

730 1415 185

730 1464 140

730 1793 664

730 2444 386

730 2526 506

730 3067 226

730 4123 284

730 7792 120

730 9646 475

730 9647 136

731 96 56

731 152 37

731 163 189

731 180 50

731 187 51

731 188 118

731 190 95

731 500 101

731 503 332

731 538 37

731 630 50

731 707 283

731 734 47

731 806 59

731 815 134

731 830 65

731 846 123

731 854 293

731 856 162

731 917 93

731 923 115

731 1145 93

731 1162 50

731 1216 125

731 1248 33

731	1378	37
731	1425	65
731	1705	70
731	1778	40
731	1854	151
731	2352	85
731	2366	79
731	2370	106
731	2379	106
731	2580	34
731	2595	72
731	2744	173
731	2794	211
731	2853	89
731	3088	46
731	3475	66
731	3940	40
731	6133	60
731	6540	121
731	8042	71
731	9260	58
731	9648	82
731	9649	69
731	9650	51
731	9651	36
732	55	13792
732	67	24619
732	71	7345
732	89	2319
732	167	2737

732 265 10946

732 288 839

732 289 18838

732 291 1092

732 292 2348

732 295 395

732 300 1310

732 329 5300

732 333 6232

732 458 371

732 464 304

732 466 496

732 486 8615

732 498 1522

732 524 289

732 525 1088

732 637 255

732 640 10504

732 677 1295

732 701 8417

732 962 1528

732 972 339

732 1243 455

732 1379 479

732 1601 3836

732 2044 1988

732 2054 295

732 2067 455

732 3198 1009

732 3310 490

732 3501 15576

732 3502 1348

732 3560 280

732 3674 355

732 3795 294

732 3991 1095

732 6866 2317

732 9652 3067

732 9653 1159

732 9654 789

732 9655 516

732 9656 312

732 9657 264

732 9658 258

732 9659 242

733 65 81

733 89 66

733 155 84

733 190 97

733 230 223

733 297 1442

733 304 659

733 321 182

733 324 64

733 333 67

733 355 207

733 403 134

733 464 83

733 466 117

733 470 150

733 472 71

733 479 65

733 498 60

733 544 669

733 679 1217

733 683 148

733 687 159

733 691 134

733 1038 159

733 1043 137

733 1047 3597

733 1189 100

733 1198 85

733 1689 149

733 1866 88

733 2176 103

733 2657 121

733 2977 540

733 3078 90

733 3373 124

733 3461 133

733 4375 68

733 4559 298

733 4708 739

733 5228 79

733 5313 147

733 5760 181

733 6608 226

733 7322 94

733 9250 516

733 9660 308

733 9661 253

733 9662 135

733 9663 129

733 9664 74

734 154 2361

734 162 2999

734 190 3154

734 198 10863

734 217 8171

734 220 3539

734 227 2676

734 412 2600

734 418 10370

734 622 2518

734 632 2605

734 691 2693

734 707 6627

734 770 2140

734 978 2551

734 1075 2565

734 1131 2054

734 1163 1986

734 1389 2334

734 1633 3531

734 2425 2431

734 2627 10017

734 3040 2223

734 3057 5773

734 3248 18698

734 3249 5959

734 3411 2017

734 3685 2295

734 3705 2677

734 4174 2528

734 4208 2354

734 4230 21686

734 4385 2011

734 6048 4124

734 6293 2711

734 6298 2054

734 6463 3971

734 6724 5071

734 6759 4232

734 8027 4238

734 8459 2635

734 9665 9975

734 9666 6785

734 9667 6574

734 9668 5489

734 9669 3683

734 9670 2643

734 9671 2577

734 9672 2120

734 9673 2113

735 25 55

735 55 66

735 67 55

735 89 427

735 157 523

735 180 64

735 220 88

735 283 75

735 289 46

735 295 75

735 306 42

735 378 69

735 512 188

735 518 45

735 706 139

735 707 53

735 710 72

735 718 58

735 724 47

735 792 53

735 843 110

735 903 68

735 917 555

735 918 74

735 922 70

735 930 102

735 932 301

735 940 81

735 957 37

735 959 207

735 966 38

735 1214 42

735 1249 47

735 1264 169

735 1281 42

735 1360 683

735 1369 109

735 1372 36

735 1412 124

735 1415 200

735 1513 70

735 2343 258

735 3462 44

735 4087 71

735 4990 276

735 6527 84

735 7927 48

735 9674 127

735 9675 79

735 9676 42

736 190 522

736 227 5608

736 278 299

736 288 3175

736 294 1796

736 295 416

736 302 4039

736 331 298

736 349 597

736 377 3875

736 475 36098

736 907 386

736 1456 285

736 1613 467

736 1677 751

736 2179	1028
736 2226	257
736 2526	409
736 2905	355
736 3034	1131
736 3667	1474
736 5058	2640
736 5254	15503
736 5988	691
736 6618	602
736 6627	645
736 7759	31944
736 7874	1080
736 8600	704
736 9677	8082
736 9678	5565
736 9679	3591
736 9680	3270
736 9681	2557
736 9682	2034
736 9683	1993
736 9684	1902
736 9685	1516
736 9686	1353
736 9687	1280
736 9688	1272
736 9689	1010
736 9690	826
736 9691	782
736 9692	744

736 9693 671

736 9694 312

736 9695 219

736 9696 215

736 9697 209

737 7 329

737 154 418

737 173 316

737 198 428

737 203 310

737 210 339

737 220 420

737 226 379

737 227 434

737 228 305

737 230 243

737 234 473

737 331 247

737 412 271

737 424 206

737 428 225

737 429 352

737 436 471

737 475 227

737 489 283

737 512 323

737 517 426

737 554 387

737 562 452

737 599 191

737 614 331

737 707 486

737 709 427

737 718 212

737 735 444

737 855 268

737 859 497

737 917 316

737 959 281

737 978 361

737 982 388

737 1044 397

737 1119 403

737 1131 275

737 1249 218

737 1400 217

737 1402 205

737 1412 218

737 1426 366

737 1814 319

737 1974 225

737 2612 278

737 2637 187

737 4092 285

737 5844 220

738 163 830

738 227 636

738 607 214

738 706 911

738 707 2724

738 735 255

738 841 589

738 843 329

738 868 348

738 910 138

738 917 3221

738 918 1945

738 928 472

738 947 574

738 949 258

738 954 617

738 955 263

738 957 340

738 1274 151

738 1276 135

738 1342 148

738 1348 298

738 1353 803

738 1357 142

738 1358 1535

738 1360 336

738 1366 134

738 1369 6369

738 1412 1386

738 1415 290

738 1527 175

738 1803 170

738 2009 643

738 2342 1281

738 2343 2246

738 2463 1253

738 2746 251

738 2834 150

738 3470 276

738 3476 177

738 3509 520

738 3660 175

738 5159 164

738 5689 478

738 6576 406

738 7047 199

738 7922 275

738 9222 137

738 9334 193

738 9698 414

739 261 279

739 270 179

739 283 125

739 295 135

739 300 95

739 316 279

739 333 165

739 542 127

739 599 119

739 705 163

739 892 105

739 903 179

739 1150 116

739 1421 110

739 1463 189

739 1464	168
739 1755	292
739 1756	449
739 1758	95
739 1763	225
739 1767	137
739 1772	133
739 3057	681
739 3070	180
739 3393	99
739 4260	2795
739 4618	180
739 4620	97
739 4701	109
739 5441	208
739 5745	184
739 6795	157
739 8594	263
739 8756	1275
739 9539	101
739 9699	5159
739 9700	1320
739 9701	300
739 9702	242
739 9703	216
739 9704	212
739 9705	208
739 9706	185
739 9707	177
739 9708	174

739 9709 140

739 9710 125

739 9711 124

739 9712 115

739 9713 113

741 7 397

741 56 891

741 65 610

741 154 260

741 157 253

741 161 427

741 182 321

741 190 457

741 191 395

741 209 413

741 226 457

741 229 1777

741 302 582

741 324 356

741 333 645

741 377 3278

741 403 615

741 471 400

741 481 292

741 486 656

741 491 326

741 497 325

741 498 611

741 507 285

741 511 462

741 536 356

741 540 559

741 706 687

741 808 247

741 1043 312

741 1091 749

741 1179 325

741 1186 2068

741 1198 805

741 1377 272

741 1385 241

741 1406 518

741 1410 697

741 1444 254

741 1866 345

741 2498 344

741 2661 921

741 3475 262

741 3753 468

741 4189 315

741 4196 783

741 4218 370

741 4435 1178

741 5446 259

741 6453 288

742 88 35

742 89 141

742 107 54

742 196 66

742 228 42

742 331 188

742 445 42

742 548 235

742 689 273

742 981 33

742 1098 208

742 1167 82

742 1379 143

742 1485 134

742 1486 38

742 1518 90

742 1609 256

742 1934 275

742 1971 34

742 2137 58

742 2376 74

742 2521 106

742 3317 64

742 3687 95

742 4035 39

742 4704 69

742 5071 34

742 5150 174

742 5233 97

742 6038 116

742 7781 79

742 7782 41

742 8674 39

742 9285 181

742 9718 138

742 9719 105

742 9720 79

742 9721 59

742 9722 53

742 9723 50

742 9724 49

742 9725 46

742 9726 41

742 9727 39

742 9728 39

742 9729 38

742 9730 37

742 9731 33

742 9732 32

742 9733 32

743 67 1951

743 89 4390

743 344 1546

743 367 11447

743 396 1314

743 428 1127

743 455 1383

743 641 1209

743 701 1204

743 898 2614

743 930 2005

743 1009 3366

743 1048 2631

743 1339 11828

743 1385 1767

743 1443	1918
743 1904	2158
743 2094	2070
743 2498	1984
743 3501	1633
743 3503	1207
743 3573	1559
743 3865	3000
743 4118	1180
743 4335	1350
743 4437	1141
743 4686	2035
743 5575	1244
743 5929	2253
743 6282	1602
743 6505	2446
743 7233	2291
743 7414	5771
743 7506	1316
743 8504	1403
743 9734	6192
743 9735	3399
743 9736	2923
743 9737	2223
743 9738	2174
743 9739	1958
743 9740	1777
743 9741	1602
743 9742	1510
743 9743	1467

743 9744 1334

743 9745 1301

743 9746 1292

743 9747 1184

743 9748 1175

744 89 90

744 157 89

744 230 67

744 288 112

744 289 258

744 291 51

744 299 54

744 300 112

744 308 58

744 377 272

744 378 74

744 405 108

744 466 170

744 475 832

744 481 300

744 486 279

744 498 1366

744 518 360

744 544 107

744 680 168

744 701 121

744 722 44

744 726 285

744 802 66

744 930 110

744 936 149

744 951 52

744 955 154

744 966 325

744 968 56

744 972 50

744 1057	107
744 1338	130
744 1339	184
744 1347	115
744 1379	52
744 1684	58
744 2075	73
744 2619	301
744 2941	82
744 3940	57
744 4309	64
744 5487	98
744 7243	46
744 8761	49
744 9749	82
744 9750	57
744 9751	54
744 9752	48
744 9753	48
745 1575	75
745 4687	615
745 6563	2871
745 9754	888
745 9755	603

745 9756 393

745 9757 288

745 9758 218

745 9759 203

745 9760 188

746 56 307

746 88 511

746 107 1637

746 190 2271

746 233 288

746 472 231

746 488 700

746 493 1072

746 497 308

746 498 791

746 503 2362

746 507 1448

746 514 232

746 516 843

746 533 224

746 748 541

746 806 3015

746 810 850

746 830 3993

746 978 320

746 1044 265

746 1180 202

746 1195 578

746 1236 207

746 1672 1495

746 1745	236
746 1874	912
746 1941	434
746 1961	367
746 2605	259
746 2940	1036
746 2956	243
746 2961	336
746 3246	234
746 3686	268
746 3687	378
746 4170	338
746 4171	1052
746 4734	305
746 5310	223
746 7079	341
746 7639	331
746 8471	207
746 9761	6030
746 9762	406
746 9763	315
746 9764	264
746 9765	258
746 9766	214
746 9767	203
747 464 464	
747 466 434	
747 507 626	
747 691 614	
747 768 951	

747 779 1009

747 813 364

747 816 1626

747 829 433

747 830 475

747 831 690

747 1091 501

747 1100 556

747 1181 815

747 1185 640

747 1187 611

747 1188 381

747 1192 378

747 1197 545

747 1383 341

747 1541 569

747 1853 384

747 1868 1159

747 2430 736

747 2576 341

747 2850 792

747 3621 1358

747 3721 379

747 3769 669

747 4100 386

747 4102 664

747 4169 515

747 4174 2739

747 4262 460

747 4948 376

747 5969 479

747 6773 593

747 7946 821

747 8264 1083

747 8464 385

747 8684 448

747 9768 739

747 9769 617

747 9770 588

747 9771 481

747 9772 473

747 9773 401

747 9774 399

747 9775 379

747 9776 366

749 55 590

749 65 775

749 67 4323

749 89 2653

749 191 2028

749 207 348

749 227 649

749 229 6032

749 257 2041

749 262 367

749 288 1047

749 289 12590

749 291 671

749 292 16904

749 294 1309

749 295 2724

749 298 1207

749 300 2264

749 301 652

749 306 595

749 320 784

749 324 888

749 325 1226

749 333 554

749 344 1206

749 349 2049

749 352 1406

749 375 379

749 461 26802

749 466 1151

749 498 354

749 523 735

749 528 1456

749 679 6381

749 688 490

749 691 1061

749 915 569

749 961 683

749 1243 1083

749 1246 350

749 1377 478

749 1459 1153

749 1602 3017

749 2025 558

749 2544 594

749 2584 530

749 2977 3072

749 3128 948

749 3397 2603

749 3488 417

750 81 95

750 154 192

750 197 173

750 207 138

750 226 186

750 229 154

750 233 140

750 234 81

750 238 136

750 424 125

750 425 211

750 441 72

750 512 168

750 562 98

750 603 162

750 748 118

750 757 190

750 769 209

750 770 70

750 851 94

750 854 79

750 964 430

750 977 111

750 1099 131

750 1116 107

750 1118	198

750 1119	76

750 1131	161

750 1203	146

750 1216	85

750 2472	78

750 2473	107

750 2582	155

750 3011	108

750 3259	203

750 3447	73

750 3767	92

750 8407	153

750 9452	221

750 9454	193

750 9587	83

750 9779	214

750 9780	190

750 9781	170

750 9782	136

750 9783	136

750 9784	118

750 9785	117

750 9786	75

750 9787	68

751 7	163

751 209 77

751 230 186

751 234 183

751 278 62

751 333 1371

751 377 78

751 378 47

751 412 100

751 436 81

751 475 92

751 480 68

751 481 57

751 486 89

751 498 135

751 501 609

751 508 123

751 536 89

751 614 47

751 724 141

751 808 100

751 863 45

751 930 243

751 1044 180

751 1145 83

751 1179 99

751 1186 187

751 1406 45

751 1613 46

751 1673 59

751 1689 44

751 1786 46

751 1854 150

751 1866 179

751 2226 47

751 2236 67

751 2380 765

751 3833 259

751 4173 76

751 4226 45

751 4375 114

751 4502 79

751 6196 95

751 7712 246

751 9788 168

751 9789 123

751 9790 84

751 9791 81

751 9792 56

751 9793 44

752 298 235

752 412 1355

752 498 384

752 805 1247

752 816 587

752 823 584

752 850 315

752 989 311

752 1046 219

752 1048 375

752 1100 533

752 1181 232

752 1192 552

752 1541 231

752 1552 375

752 1562	195
752 2187	325
752 2247	391
752 2502	188
752 2618	475
752 2665	187
752 3027	367
752 3029	1109
752 5759	252
752 8294	203
752 8681	202
752 8925	312
752 9794	1206
752 9795	1024
752 9796	874
752 9797	446
752 9798	446
752 9799	376
752 9800	310
752 9801	253
752 9802	251
752 9803	241
752 9804	239
752 9805	237
752 9806	236
752 9807	222
752 9808	221
752 9809	194
752 9810	187
752 9811	183

752 9812 183

752 9813 181

752 9814 181

752 9815 178

752 9816 177

753 67 447

753 157 587

753 192 538

753 253 1041

753 257 303

753 328 292

753 344 264

753 360 188

753 732 620

753 734 598

753 735 520

753 945 181

753 951 2337

753 952 495

753 959 1111

753 1248 778

753 1249 1149

753 1369 193

753 1412 301

753 1639 254

753 1803 1003

753 1810 1007

753 1813 382

753 2053 356

753 2346 799

753 2347 1064

753 2350 228

753 2467 225

753 2783 272

753 2787 403

753 2797 191

753 2859 608

753 3063 940

753 3473 171

753 3477 255

753 3576 162

753 3748 204

753 4024 185

753 4165 580

753 5841 225

753 6292 426

753 6319 192

753 8743 188

753 9334 365

753 9817 738

753 9818 347

753 9819 320

753 9820 291

753 9821 184

753 9822 170

754 96 275

754 197 648

754 220 311

754 227 1077

754 228 294

754 229 226

754 235 292

754 366 232

754 511 236

754 554 875

754 562 670

754 575 299

754 599 231

754 612 300

754 614 343

754 618 264

754 734 886

754 735 285

754 851 322

754 857 501

754 859 287

754 905 429

754 961 322

754 1110 252

754 1415 258

754 1513 333

754 1519 281

754 1772 236

754 1881 237

754 2629 240

754 2641 240

754 2853 227

754 2893 282

754 3201 351

754 3279 242

754 3458 227

754 3469 226

754 3777 278

754 4531 233

754 4999 596

754 5446 310

754 6728 277

754 7352 298

754 7661 229

754 9631 277

754 9823 449

754 9824 401

754 9825 292

754 9826 277

754 9827 237

756 53 18

756 65 27

756 81 17

756 154 159

756 190 32

756 199 33

756 202 21

756 203 23

756 207 34

756 210 28

756 220 21

756 222 106

756 227 116

756 228 25

756 229 18

756 420 24

756 424 19

756 425 27

756 429 22

756 441 29

756 554 27

756 562 43

756 599 32

756 689 28

756 709 22

756 716 22

756 859 17

756 863 22

756 868 17

756 985 27

756 1090 39

756 1106 19

756 1239 44

756 1504 24

756 1513 29

756 1791 28

756 1934 28

756 1958 22

756 1976 134

756 2029 29

756 2121 24

756 2277 32

756 2616 21

756 3030 20

756 3279 18

756 3475 28

756 3993 20

756 5890 23

756 7076 60

756 7869 18

757 89 3050

757 157 26148

757 161 26723

757 184 22

757 230 24

757 257 274

757 265 591

757 288 31800

757 289 30230

757 291 762

757 292 860

757 294 18

757 295 60877

757 300 10368

757 302 708

757 306 26767

757 327 1102

757 329 3767

757 330 484

757 331 10

757 352 471

757 456 123

757 461 20003

757 462 1337

757 466 23277

757 468 1408

757 475 26933

757 484 7

757 486 63

757 531 267

757 538 98

757 544 38

757 701 169596

757 903 21

757 907 3673

757 1037 238

757 1042 2039

757 1052 5

757 1452 77

757 1455 203

757 1613 1192

757 4562 11

757 9844 2955

757 9845 1336

757 9846 37

757 9847 19

757 9848 12

757 9849 6

757 9850 5

757 9851 4

758 7 556

758 65 264

758 159 214

758 173 771

758 190 232

758 198 206

758 214 1073

758 227 4471

758 298 267

758 301 475

758 344 440

758 371 386

758 416 592

758 423 335

758 425 184

758 429 1341

758 486 313

758 488 155

758 497 186

758 498 3128

758 499 297

758 521 185

758 523 455

758 533 1204

758 537 1053

758 548 629

758 681 152

758 703 1189

758 718 246

758 735 728

758 779 170

758 816 898

758 1009 332

758 1105 350

758 1200 562

758 1242 570
758 1244 391
758 1377 965
758 1378 175
758 1379 236
758 1424 651
758 1518 416
758 1609 195
758 1853 237
758 2210 760
758 2616 431
758 3258 171
758 4747 192
758 8047 645
758 9852 159
759 67 265
759 89 747
759 154 867
759 170 223
759 172 455
759 173 246
759 190 299
759 191 748
759 203 174
759 206 168
759 207 192
759 221 186
759 227 733
759 229 1809
759 237 188

759 297 189

759 298 132

759 302 126

759 329 179

759 420 118

759 423 216

759 425 318

759 429 608

759 437 181

759 441 475

759 511 201

759 526 160

759 535 158

759 538 281

759 544 3571

759 548 189

759 599 136

759 683 228

759 701 122

759 703 121

759 757 133

759 859 511

759 1075 186

759 1090 300

759 1106 335

759 1151 268

759 1243 236

759 1390 172

759 1632 608

759 1767 182

759 2083 127

759 2130 201

759 2977 208

759 7276 161

759 9853 133

760 209 228

760 220 829

760 227 8816

760 230 587

760 234 5017

760 283 332

760 377 247

760 378 158

760 389 74

760 481 98

760 498 1708

760 533 278

760 540 124

760 542 83

760 705 1960

760 706 3045

760 714 544

760 808 20233

760 868 132

760 932 44570

760 951 383

760 959 3777

760 966 739

760 982 1017

760 1241 97

760 1244 209

760 1246 452

760 1249 1345

760 1513 13319

760 1545 315

760 1775 300

760 1814 377

760 1854 987

760 2080 680

760 2344 89

760 2848 122

760 2983 2373

760 2988 90

760 3382 158

760 4087 399

760 4818 187

760 5772 239

760 6120 104

760 6123 143

760 7330 934

760 9229 1076

760 9854 1452

760 9855 286

760 9856 70

760 9857 68

761 36 881

761 51 3500

761 55 220

761 56 310

761 58 510

761 59 340

761 67 1431

761 70 231

761 72 590

761 159 1111

761 172 1540

761 302 321

761 599 480

761 777 669

761 864 290

761 875 600

761 985 1300

761 997 1985

761 999 250

761 1014 441

761 1019 450

761 1083 2229

761 1099 532

761 1394 430

761 1827 410

761 1878 269

761 1885 648

761 1886 544

761 1891 390

761 2027 240

761 2488 490

761 2535 359

761 2552 2082

761 2562 460

761 2936 330

761 3281 634

761 3285 469

761 3291 380

761 3562 1069

761 4732 280

761 5045 521

761 5258 610

761 8582 400

761 8790 350

761 9858 657

761 9859 569

761 9860 500

761 9861 420

761 9862 300

761 9863 260

762 30 287

762 65 121

762 72 3039

762 75 140

762 159 757

762 187 68

762 190 50

762 193 32

762 220 43

762 227 61

762 229 151

762 441 103

762 450 71

762 486 31

762 511 66

762 515 37

762 522 37

762 601 51

762 707 37

762 865 71

762 877 93

762 878 69

762 959 61

762 1019	38
762 1055	108
762 1072	194
762 1073	33
762 1075	34
762 1099	65
762 1104	103
762 1244	47
762 1376	300
762 1379	174
762 1400	38
762 1534	36
762 1713	105
762 1714	118
762 1799	56
762 2130	54
762 2783	38
762 4872	67
762 5483	32
762 7324	37
762 7426	32
762 9864	585

762 9865 107

762 9866 50

762 9867 42

762 9868 34

762 9869 32

763 7 178

763 65 236

763 173 224

763 181 235

763 190 151

763 233 263

763 328 236

763 371 287

763 377 641

763 378 139

763 403 177

763 481 154

763 486 197

763 488 654

763 489 449

763 493 308

763 497 256

763 498 165

763 500 592

763 503 182

763 504 208

763 517 383

763 518 160

763 726 142

763 808 158

763 966 144

763 978 183

763 1044 352

763 1091 139

763 1104 504

763 1145 150

763 1179 155

763 1186 289

763 1200 160

763 1797 131

763 1854 290

763 1859 171

763 1863 267

763 2014 519

763 2121 193

763 2379 200

763 2843 223

763 3247 166

763 3367 215

763 6750 306

763 6819 247

763 7775 328

763 8254 179

763 9870 200

763 9871 162

764 7 172

764 96 241

764 166 1134

764 193 258

764 207 215

764 222 189

764 234 242

764 238 511

764 316 225

764 360 164

764 366 347

764 370 428

764 416 6111

764 436 258

764 533 429

764 562 190

764 630 2334

764 859 1044

764 898 364

764 903 202

764 959 660

764 969 281

764 1103 163

764 1118 4312

764 1131 184

764 1242 252

764 1358 232

764 1415 1128

764 1537 177

764 1806 1004

764 1813 212

764 2265 188

764 2531 1164

764 2821 247

764 2834 2016

764 2853 2160

764 3201 280

764 3289 169

764 3302 1039

764 3707 186

764 3732 3516

764 4051 417

764 4111 421

764 4620 1258

764 5947 271

764 6140 1060

764 8313 322

764 9419 455

764 9872 1312

764 9873 317

765 53 356

765 154 521

765 170 337

765 196 420

765 199 909

765 210 898

765 212 328

765 217 456

765 225 295

765 227 341

765 331 306

765 416 250

765 418 1816

765 440 899

765 444 419

765 445 479

765 604 262

765 615 292

765 618 712

765 619 275

765 621 297

765 622 355

765 632 449

765 703 280

765 757 552

765 1155 348

765 1390 297

765 1627 557

765 1633 300

765 1735 586

765 1943 244

765 1960 392

765 1963 411

765 2268 373

765 2405 541

765 3026 253

765 3411 262

765 3685 517

765 3693 439

765 5927 254

765 6756 284

765 7071 341

765 8706 357

765 9874 601

765 9875 566

765 9876 305

765 9877 301

765 9878 280

765 9879 268

765 9880 262

767 30 6316

767 65 339

767 72 71852

767 153 383

767 173 3144

767 176 196

767 185 572

767 190 3257

767 209 163

767 220 124

767 310 222

767 328 217

767 349 154

767 377 232

767 378 1894

767 405 220

767 521 202

767 534 121

767 722 209

767 878 136

767 880 1284

767 930 329

767 1019 178

767 1053 277

767 1072 1274

767 1091 282

767 1109 254

767 1298 217

767 1330 159

767 1713 5741

767 2526 205

767 2638 129

767 3070 129

767 3237 872

767 3238 386

767 3768 248

767 3877 126

767 4156 166

767 4574 280

767 5485 242

767 5487 241

767 8284 125

767 9038 219

767 9886 998

767 9887 364

767 9888 202

767 9889 168

767 9890 161

767 9891 143

767 9892 136

768 166 1

768 230 2

768 289 7

768 325 1

768 333 25

768 342 3

768 344 158

768 458 1

768 461 2

768 466 3

768 475 5

768 498 16

768 523 23

768 635 7

768 681 7

768 685 1

768 686 3

768 691 1

768 722 2

768 735 1

768 797 10

768 1047 9

768 1455 12

768 1458 4

768 1459 1

768 1462 2

768 1684 1

768 1874 11

768 2439 1

768 3464 1

768 3557 3

768 4260 1

768 4294 1

768 4879 1

768 5099 5

768 5267 3

768 5958 2

768 7399 8

768 7500 6

768 7704 1

768 8245 1

768 9893 2

768 9894 1

768 9895 1

769 51 238

769 72 9876

769 157 646

769 162 531

769 172 181

769 173 1013

769 184 290

769 187 561

769 190 4322

769 207 2906

769 227 5265

769 233 1352

769 432 483

769 486 600

769 533 14793

769 599 1036

769 726 2896

769 969 1524

769 1001 175

769 1061 1443

769 1072 190

769 1090 3474

769 1121 358

769 1511 428

769 1512 1795

769 1791 1261

769 2128 162

769 3046 281

769 5000 650

769 6857 1745

769 7130 218

769 9896 180

769 9897 153

770 67 243

770 89 1255

770 161 400

770 191 216

770 229 252

770 230 148

770 288 1391

770 289 215

770 292 297

770 294 193

770 295 412

770 300 147

770 302 683

770 306 573

770 308 314

770 310 8746

770 320 156

770 328 484

770 329 193

770 377 619

770 386 662

770 403 159

770 471 239

770 498 544

770 525 353

770 538 227

770 545 149

770 641 219

770 701 1171

770 907 716

770 972 211

770 1009 165

770 1449 265

770 1678 176

770 1863 150

770 2083 353

770 2440 278

770 2498 311

770 3171 152

770 3316 195

770 3985 282

770 4024 295

770 4817 309

770 5731 169

770 5947 151

770 6309 177

770 6317 222

770 6376 255

770 9898	184

770 9899	163

771 65	18

771 228 43

771 245 17

771 249 41

771 250 9

771 266 40

771 267 51

771 271 53

771 278 46

771 301 81

771 330 73

771 331 281

771 468 98

771 527 17

771 547 12

771 617 12

771 1375	7

771 1402	7

771 1556	6

771 1613	40

771 1620	21

771 1641	16

771 1839	67

771 1841	70

771 2226	8

771 2910	12

771 2914	6

771 2921	8

771 2923	189
771 2931	13
771 3317	62
771 4696	31
771 7259	9
771 8071	8
771 8161	8
771 9900	3176
771 9901	28
771 9902	24
771 9903	21
771 9904	20
771 9905	20
771 9906	13
771 9907	12
771 9908	12
771 9909	11
771 9910	10
771 9911	8
771 9912	7
771 9913	7
771 9914	7

772 176 14

772 500 16

772 724 34

772 917 20

772 918 12

772 928 9

772 945 12

772 978 16

772 1358	58
772 1360	25
772 1366	19
772 1369	85
772 1372	14
772 1468	10
772 1535	9
772 1992	9
772 2013	16
772 2183	9
772 2341	16
772 2342	32
772 2347	8
772 2362	16
772 2463	20
772 2569	18
772 2838	15
772 3470	34
772 3471	10
772 3476	13
772 3921	8
772 4124	10
772 4680	9
772 5157	21
772 5365	9
772 5695	16
772 8258	10
772 8743	16
772 8994	13
772 9529	9

772 9915 32

772 9916 16

772 9917 14

772 9918 13

772 9919 12

772 9920 10

772 9921 10

772 9922 10

772 9923 10

772 9924 9

772 9925 9

772 9926 8

773 70 576

773 88 987

773 154 3172

773 159 639

773 163 1459

773 173 1115

773 197 2932

773 198 1715

773 202 1173

773 209 957

773 220 1698

773 227 7613

773 234 1021

773 250 997

773 436 7027

773 521 637

773 614 573

773 707 993

773 709 798

773 726 869

773 867 1192

773 868 2458

773 959 706

773 1146	1140
773 1351	1211
773 1372	957
773 1981	2543
773 1984	2683
773 2612	732
773 2637	612
773 2893	634
773 3213	866
773 3625	1252
773 3627	735
773 3635	1491
773 4118	570
773 5042	1015
773 5257	677
773 5485	924
773 7038	989
773 7426	1806
773 8772	1381
773 9886	1399
773 9927	3476
773 9928	3136
773 9929	1321
773 9930	996
773 9931	981

773 9932 790
773 9933 585
776 190 75
776 220 148
776 227 190
776 234 446
776 412 252
776 475 134
776 489 123
776 517 182
776 538 79
776 707 237
776 813 97
776 814 98
776 815 64
776 831 92
776 842 66
776 850 121
776 966 93
776 1091 421
776 1195 74
776 1196 103
776 1206 552
776 1249 67
776 1406 362
776 1547 118
776 1788 95
776 2211 66
776 2379 125
776 2384 54

776 2586	71
776 2738	70
776 3028	100
776 3354	54
776 3721	82
776 3729	140
776 4096	107
776 4216	63
776 4504	55
776 4723	158
776 4785	92
776 4821	164
776 9122	62
776 9286	99
776 9960	211
776 9961	146
776 9962	136
776 9963	98
776 9964	94
776 9965	84
776 9966	62
776 9967	60
777 7	492
777 11	136
777 17	500
777 19	126
777 30	1131
777 39	389
777 55	242
777 67	3382

777 70 730

777 72 188

777 75 135

777 157 284

777 172 225

777 194 914

777 506 364

777 534 224

777 535 272

777 599 1120

777 718 233

777 868 125

777 875 271

777 1067 2986

777 1069 1335

777 1075 198

777 1083 171

777 1297 430

777 1366 128

777 1394 124

777 1534 108

777 1707 1134

777 1885 131

777 2369 121

777 2681 356

777 3087 207

777 3198 197

777 3878 289

777 4038 273

777 5115 152

777 5257	124
777 6648	132
777 6667	119
777 7324	132
777 9968	836
777 9969	328
777 9970	267
777 9971	155
777 9972	138
777 9973	123
777 9974	119
777 9975	102
778 7	1168
778 55	3278
778 67	2089
778 89	3818
778 209	1895
778 251	1399
778 257	26974
778 265	2974
778 279	19385
778 285	4589
778 288	1850
778 289	23947
778 290	3287
778 291	3562
778 292	5213
778 293	1678
778 299	2005
778 300	6279

778 302 4837

778 304 1621

778 311 2933

778 314 1983

778 318 2870

778 325 2579

778 326 4239

778 342 7597

778 344 2299

778 349 1640

778 355 5549

778 461 3784

778 464 1435

778 466 7548

778 485 1696

778 498 6530

778 523 2191

778 538 2155

778 548 2554

778 602 2046

778 679 1249

778 682 4849

778 691 1881

778 701 4232

778 3081 2035

778 4566 6123

778 4619 2346

778 4710 2457

778 5746 1638

778 8155 1561

778 9976 2541

778 9977 1649

779 67 1487

779 72 6994

779 89 108

779 157 116

779 159 49

779 163 305

779 187 475

779 289 1441

779 475 620

779 511 519

779 518 71

779 539 49

779 706 54

779 711 46

779 722 179

779 877 141

779 917 235

779 919 434

779 930 247

779 957 73

779 959 47

779 1415 122

779 1463 51

779 1596 91

779 1704 10203

779 1793 116

779 1900 169

779 2500 142

779 2514 46

779 2533 66

779 3719 91

779 4258 103

779 4871 53

779 4912 56

779 5254 51

779 6200 2002

779 6626 536

779 6748 203

779 7414 333

779 7415 160

779 9978 442

779 9979 233

779 9980 193

779 9981 175

779 9982 113

779 9983 94

779 9984 88

779 9985 86

779 9986 79

779 9987 47

780 56 34

780 58 769

780 64 53

780 89 1264

780 91 91

780 96 86

780 100 65

780 163 46

780 167 41

780 193 34

780 227 526

780 229 1438

780 298 94

780 324 50

780 378 61

780 525 106

780 526 35

780 530 32

780 532 43

780 733 42

780 852 73

780 959 248

780 972 29

780 997 40

780 1001 3381

780 1027 33

780 1073 52

780 1379 157

780 1409 567

780 2014 93

780 2027 59

780 2029 54

780 2130 190

780 2134 41

780 2538 42

780 3201 46

780 3739 49

780 4389 55

780 4885 39

780 5256 66

780 7347 39

780 8583 114

780 8878 103

780 9647 46

780 9988 191

780 9989 63

780 9990 54

780 9991 34

780 9992 33

780 9993 29

781 89 315

781 257 60

781 288 769

781 289 1313

781 290 173

781 291 182

781 292 1222

781 294 162

781 295 570

781 296 208

781 299 228

781 300 420

781 301 110

781 302 55

781 306 80

781 308 149

781 317 74

781 320 160

781 323 78

781 325 112

781 327 76

781 328 62

781 329 80

781 333 65

781 344 101

781 349 479

781 352 54

781 386 59

781 458 79

781 466 211

781 468 58

781 484 101

781 528 106

781 538 63

781 543 75

781 679 218

781 680 54

781 681 234

781 686 225

781 699 118

781 701 130

781 797 107

781 913 62

781 1032 91

781 1042 67

781 1051 53

781 1458 67

781 2083 51

781 2392 89

781 7340 80

782 89 178

782 137 768

782 174 69

782 418 114

782 444 5168

782 603 1456

782 744 206

782 747 96

782 748 4049

782 749 127

782 757 215

782 767 139

782 1098 361

782 1222 859

782 1747 97

782 2667 749

782 2673 1058

782 2676 100

782 2702 518

782 4049 290

782 4271 99

782 4297 697

782 5927 398

782 6756 90

782 7021 662

782 7023 80

782 8390 74

782 9970 120

782 9994 760

782 9995 600

782 9996 384

782 9997 298

782 9998 278

782 9999 262

782 10000 203

782 10001 201

782 10002 179

782 10003 163

782 10004 144

782 10005 142

782 10006 141

782 10007 137

782 10008 121

782 10009 92

782 10010 89

782 10011 87

782 10012 77

782 10013 74

782 10014 74

782 10015 67

784 65 363

784 154 1895

784 197 1426

784 199 880

784 205 828

784 211 1154

784 217 654

784 227 3081

784 236 577

784 331 532

784 418 1494

784 433 819

784 440 566

784 445 578

784 599 439

784 602 733

784 622 1806

784 629 1693

784 703 724

784 748 544

784 959 493

784 1106 637

784 1122 809

784 1164 436

784 1242 382

784 1390 452

784 1686 367

784 1732 938

784 1944 3226

784 1947 1822

784 1958 738

784 2401 453

784 2424 1251

784 2535 450

784 2627 520

784 3057 827

784 3258 661

784 3411 563

784 3621 558

784 3666 1022

784 3685 1904

784 4079 1511

784 4118 505

784 4123 759

784 5434 554

784 6229 888

784 6910 410

784 10024 541

784 10025 489

784 10026 427

785 1 76

785 154 146

785 157 165

785 159 52

785 206 49

785 220 45

785 227 118

785 234 71

785 258 937

785 261 232

785 270 38

785 292 103

785 316 105

785 340 2707

785 390 314

785 396 264

785 406 173

785 424 76

785 436 45

785 439 50

785 599 2863

785 701 36

785 709 106

785 735 56

785 889 129

785 903 215

785 917 109

785 982 159

785 985 58

785 1098 386

785 1520 91

785 1768 1755

785 1771 3022

785 1775 64

785 1929 45

785 2121 58

785 2786 50

785 3469 96

785 4135 117

785 4793 35

785 4819 355

785 5416 501

785 5470 815

785 6721 46

785 6777 255

785 8707 1541

785 9528 280

785 9700 88

785 10027 104

785 10028 46

786 70 300

786 88 2673

786 154 176

786 191 268

786 220 555

786 234 163

786 300 164

786 371 342

786 492 274

786 526 243

786 547 330

786 757 709

786 759 311

786 968 347

786 969 230

786 1009 210

786 1099 445

786 1104 874

786 1196 194

786 1270 850

786 1400 188

786 1410 426

786 1552 202

786 1789 173

786 2158 355

786 2578 268

786 2702 1930

786 2747 561

786 3028 245

786 3046 231

786 3685 387

786 3827 1254

786 3828 188

786 4896 186

786 4944 500

786 5000 248

786 5018 1577

786 5472 608

786 5988 299

786 6014 376

786 8881 174

786 10029 554

786 10030 307

786 10031 273

786 10032 262

786 10033 235

786 10034 234

786 10035 225

786 10036 182

786 10037 169

787 182 61

787 190 261

787 199 55

787 207 103

787 229 56

787 377 229

787 424 191

787 432 189

787 486 64

787 492 105

787 493 123

787 512 110

787 536 55

787 687 363

787 691 67

787 768 142

787 779 74

787 808 102

787 816 282

787 1038 146

787 1104 55

787 1181 79

787 1183 330

787 1190 125

787 1195 100

787 1376 424

787 1410 184

787 1455 90

787 1517 105

787 1853 74

787 2130 196

787 2210 56

787 2533 1069

787 2580 81

787 2582 104

787 2601 74

787 2607 58

787 2608 68

787 3082 63

787 3769 425

787 4444 68

787 4995 197

787 5000 66

787 5001 201

787 6223 165

787 7776 70

787 7777 181

787 9936 511

787 10038 397

787 10039 176

788 7 106

788 51 726

788 67 154

788 77 95

788 157 111

788 192 73

788 227 194

788 230 121

788 234 175

788 245 225

788 517 92

788 533 146

788 601 102

788 707 106

788 715 77

788 724 76

788 733 141

788 857 72

788 860 81	
788 951 99	
788 1076	77
788 1079	88
788 1242	119
788 1249	132
788 1406	107
788 1412	70
788 1413	122
788 1414	79
788 1415	73
788 1803	81
788 1974	82
788 2346	102
788 2347	445
788 2444	90
788 2554	185
788 2556	82
788 2783	99
788 3097	67
788 3281	149
788 3302	110
788 3473	73
788 3675	83
788 3732	404
788 3777	91
788 5168	72
788 8991	73
788 8992	112
788 10040	85

788 10041 78

788 10042 73

789 220 331

789 267 591

789 278 2190

789 279 407

789 292 373

789 294 606

789 295 285

789 301 235

789 312 280

789 327 960

789 329 258

789 331 1039

789 377 806

789 468 767

789 475 449

789 484 429

789 707 484

789 913 262

789 966 241

789 1056 772

789 1369 548

789 1444 257

789 1641 1030

789 1975 1486

789 1979 1355

789 1989 325

789 2179 352

789 3227 1954

789 5038 1451

789 5041 283

789 5487 421

789 5488 502

789 5490 267

789 5847 1113

789 8887 204

789 9840 461

789 10043 1412

789 10044 1226

789 10045 1043

789 10046 1001

789 10047 929

789 10048 578

789 10049 497

789 10050 435

789 10051 372

789 10052 369

789 10053 330

789 10054 311

789 10055 309

789 10056 267

790 89 1004

790 190 290

790 230 236

790 288 430

790 289 2394

790 291 416

790 292 572

790 300 987

790 305 128

790 318 331

790 321 924

790 323 293

790 325 1362

790 344 2097

790 349 978

790 362 327

790 377 167

790 461 270

790 481 224

790 498 2024

790 525 495

790 635 544

790 681 1943

790 686 273

790 691 152

790 701 313

790 912 557

790 951 203

790 1438 124

790 1459 1616

790 1462 213

790 1475 306

790 1684 140

790 2395 359

790 2462 216

790 3063 1624

790 3905 189

790 4817 214

790 4923 392
790 5266 243
790 5746 373
790 8372 538
790 8756 149
790 9654 615
790 10057 423
790 10058 352
790 10059 254
790 10060 204
790 10061 168
790 10062 161
791 51 97
791 65 19
791 72 11
791 84 22
791 88 15
791 98 54
791 159 29
791 165 12
791 182 50
791 193 26
791 199 18
791 203 30
791 225 55
791 229 76
791 424 13
791 429 23
791 441 13
791 486 22

791	511	25
791	546	22
791	599	30
791	716	50
791	757	25
791	857	27
791	874	24
791	969	24
791	985	11
791	1048	29
791	1062	29
791	1090	51
791	1107	21
791	1151	47
791	1376	72
791	1379	53
791	1513	14
791	1886	11
791	1895	13
791	2137	14
791	2587	25
791	2938	18
791	2978	22
791	4704	15
791	5833	37
791	7187	15
791	10063	44
791	10064	14
791	10065	14
791	10066	13

791 10067 12

791 10068 12

792 45 428

792 190 157

792 198 773

792 209 1893

792 230 4033

792 377 532

792 378 1148

792 405 190

792 412 254

792 432 188

792 481 398

792 486 176

792 488 256

792 500 1068

792 517 195

792 536 942

792 540 1555

792 768 436

792 813 103

792 830 252

792 857 117

792 951 120

792 966 116

792 1044 405

792 1104 176

792 1180 134

792 1186 2705

792 1206 248

792 1246 1342

792 1585 288

792 1672 5181

792 1803 283

792 2062 187

792 2363 165

792 2430 1248

792 2835 395

792 2956 186

792 2967 143

792 3086 136

792 3333 148

792 3621 129

792 3744 281

792 3767 268

792 4394 278

792 6194 138

792 7906 136

792 10069 210

792 10070 126

792 10071 116

792 10072 107

793 55 1520

793 65 839

793 67 12089

793 89 2045

793 155 433

793 157 289

793 190 772

793 198 603

793 207 444

793 228 316

793 229 636

793 265 376

793 285 388

793 288 1047

793 289 4390

793 292 1806

793 295 886

793 296 383

793 300 364

793 306 1136

793 310 818

793 328 414

793 329 333

793 349 586

793 466 495

793 526 548

793 532 403

793 534 428

793 535 817

793 538 642

793 544 304

793 679 966

793 886 709

793 907 337

793 959 293

793 1109 327

793 1409 566

793 1464 533

793 2523 485

793 3046 1027

793 3058 537

793 3502 440

793 3674 300

793 4026 362

793 5000 382

793 5150 383

793 8962 407

793 10073 511

793 10074 323

793 10075 303

794 7 99

794 65 99

794 207 38

794 208 38

794 215 20

794 227 2333

794 228 48

794 229 226

794 378 23

794 424 36

794 511 134

794 533 128

794 599 19

794 706 44

794 707 171

794 716 65

794 728 19

794 735 62

794 857 21

794 920 123

794 959 60

794 969 59

794 975 30

794 982 49

794 1048 289

794 1249 145

794 1358 63

794 1384 20

794 1406 23

794 1412 41

794 1414 789

794 1416 365

794 1502 25

794 1512 21

794 1516 23

794 1556 39

794 2121 76

794 2176 40

794 2607 25

794 2783 67

794 2789 135

794 2852 24

794 3071 33

794 3240 64

794 5079 26

794 5446 28

794 10063 112

794 10076 54

794 10077 51

794 10078 32

795 55 11459

795 65 1079

795 67 2795

795 89 766

795 157 391

795 161 1148

795 191 311

795 241 151

795 257 366

795 258 251

795 285 236

795 288 1958

795 289 18049

795 292 2016

795 295 634

795 296 295

795 298 2311

795 300 1608

795 310 752

795 316 344

795 327 363

795 328 169

795 331 636

795 333 126

795 349 1580

795 352 547

795 377 476

795 405 173

795 455 1529

795 458 427

795 461 4357

795 465 210

795 466 1042

795 468 742

795 478 476

795 523 859

795 543 184

795 544 149

795 644 143

795 686 142

795 701 380

795 798 467

795 907 274

795 972 1023

795 1573 743

795 1574 148

795 3501 145

795 4688 2051

795 8002 127

795 8008 209

797 67 2

797 89 3115

797 289 4

797 292 52

797 300 2

797 333 554

797 344 53395

797 466 1

797 679 2

797 1458 19

797 1679 8

798 12 154

798 198 161

798 370 44

798 503 1932

798 710 234

798 820 282

798 821 834

798 837 907

798 843 61

798 846 1123

798 1099 89

798 1104 174

798 1214 105

798 1261 80

798 1274 95

798 1281 89

798 2151 121

798 2211 457

798 2446 81

798 2654 758

798 2749 70

798 2750 47

798 2765 63

798 2769 322

798 2964 47

798 2966 457

798 4290 100

798 4669 64

798 5807 95

798 6816 109

798 7722 70

798 10086 275

798 10087 270

798 10088 248

798 10089 224

798 10090 203

798 10091 178

798 10092 153

798 10093 139

798 10094 131

798 10095 127

798 10096 120

798 10097 92

798 10098 81

798 10099 70

798 10100 68

798 10101 66

798 10102 49

798 10103 45

798 10104 44

799 59 452

799 61 116

799 96 90

799 159 248

799 227 98

799 441 263

799 511 166

799 630 73

799 715 258

799 857 74

799 863 113

799 998 65

799 1017 121

799 1073 205

799 1083 286

799 1213 92

799 1709 83

799 2533 142

799 2534 111

799 3259 73

799 3280 306

799 3281 292

799 3294 619

799 3884 88

799 4078 142

799 5255 102

799 7155 130

799 8676 111

799 9286 86

799 9825 284

799 9864 72

799 10105 920

799 10106 297

799 10107 274

799 10108 135

799 10109 128

799 10110 116

799 10111 96

799 10112 86

799 10113 82

799 10114 81

799 10115 80

799 10116 80

799 10117 76

799 10118 71

799 10119 69

799 10120 68

799 10121 66

799 10122 65

799 10123 63

800 163 863

800 176 588

800 190 350

800 198 667

800 229 287

800 614 294

800 706 499

800 707 82951

800 710 296

800 724 969

800 841 301

800 843 292

800 917 701

800 918 1159

800 920 1058

800 922 368

800 924 1085

800 928 636

800 932 1122

800 937 370

800 952 283

800 956 283

800 957 307

800 1145 367

800 1339 2042

800 1342 324

800 1347 375

800 1350 818

800 1360 743

800 1545 779

800 1672 2859

800 1870 343

800 2343 1768

800 2344 770

800 2347 447

800 2797 318

800 2834 343

800 2838 391

800 2901 597

800 3204 679

800 3376 265

800 4342 450

800 4841 272

800 5306 303

800 5450 711

800 5451 355

800 10124 880

800 10125 359
800 10126 335
800 10127 321
801 67 11
801 95 5
801 157 28
801 262 14
801 285 12
801 340 47
801 575 30
801 576 2
801 599 6
801 1821 78
801 1834 534
801 1843 44
801 1844 119
801 1892 5
801 2419 6
801 3303 9
801 6699 13
801 8975 37
801 10128 529
801 10129 66
801 10130 58
801 10131 49
801 10132 33
801 10133 30
801 10134 25
801 10135 21
801 10136 20

801 10137 19

801 10138 19

801 10139 12

801 10140 11

801 10141 11

801 10142 9

801 10143 9

801 10144 8

801 10145 7

801 10146 5

801 10147 5

801 10148 5

801 10149 4

801 10150 3

801 10151 3

801 10152 3

801 10153 3

801 10154 3

801 10155 3

801 10156 2

801 10157 2

801 10158 2

801 10159 2

802 27 16

802 30 2

802 53 14

802 54 2

802 72 5

802 75 4

802 81 1

802 90 2

802 153 4

802 238 4

802 441 10

802 631 8

802 1014 3

802 1019 3

802 1061 3

802 1072 3

802 1073 3

802 1394 2

802 1534 12

802 1787 2

802 2026 3

802 2030 4

802 2037 5

802 2039 4

802 2042 13

802 2043 17

802 2470 7

802 2486 5

802 3508 2

802 3530 3

802 3532 9

802 3537 2

802 4316 1

802 6649 7

802 6962 2

802 10160 70

802 10161 17

802 10162 14

802 10163 13

802 10164 4

802 10165 4

802 10166 2

802 10167 2

802 10168 2

802 10169 2

802 10170 2

802 10171 2

802 10172 2

802 10173 2

802 10174 1

804 67 508

804 88 335

804 161 360

804 288 1468

804 289 9069

804 290 522

804 291 509

804 292 671

804 293 457

804 295 453

804 298 477

804 299 542

804 304 1570

804 306 1226

804 310 781

804 314 779

804 318 350

804 320 356

804 325 711

804 326 1051

804 327 1021

804 329 472

804 330 1061

804 333 685

804 349 381

804 455 1328

804 456 16018

804 457 931

804 458 730

804 459 560

804 465 347

804 473 684

804 474 909

804 525 384

804 906 435

804 907 784

804 908 1783

804 1052 346

804 3498 409

804 3603 1403

804 5735 608

804 6600 3955

804 8589 664

804 10185 6148

804 10186 1083

804 10187 496

804 10188 441

804 10189 440

804 10190 381

804 10191 335

805 65 200

805 154 191

805 163 474

805 227 32

805 228 99

805 366 156

805 418 72

805 511 199

805 562 30

805 616 45

805 621 50

805 683 128

805 744 95

805 748 704

805 756 116

805 772 130

805 1106 32

805 1122 83

805 1375 278

805 1377 98

805 1383 39

805 1412 109

805 1744 59

805 2673 77

805 2676 254

805 2677 51

805 5175 285

805 6151 76

805 6189 258

805 6762 420

805 9333 535

805 9335 41

805 9337 1448

805 9339 182

805 10192 309

805 10193 276

805 10194 223

805 10195 191

805 10196 169

805 10197 156

805 10198 153

805 10199 83

805 10200 68

805 10201 64

805 10202 47

805 10203 43

805 10204 41

805 10205 33

805 10206 32

805 10207 30

806 7 999

806 25 254

806 58 635

806 89 1849

806 172 985

806 173 2089

806 190 671

806 233 316

806 238 400

806 316 203

806 377 594

806 436 659

806 539 272

806 592 300

806 599 200

806 1118 423

806 1121 4516

806 1130 479

806 1191 473

806 1705 304

806 1799 564

806 1833 260

806 1886 2407

806 2025 221

806 2638 318

806 3198 253

806 3280 304

806 3310 203

806 4918 285

806 4922 235

806 6075 281

806 6540 192

806 10208 722

806 10209 660

806 10210 555

806 10211 518

806 10212 405

806 10213 364

806 10214 343

806 10215 343

806 10216 331

806 10217 322

806 10218 288

806 10219 270

806 10220 262

806 10221 247

806 10222 235

806 10223 213

806 10224 203

806 10225 179

807 8 151

807 374 846

807 375 3820

807 412 156

807 475 107

807 498 297

807 504 64

807 533 315

807 808 69

807 968 60

807 1104 153

807 1206 126

807 1857 481

807 1866 80

807 1919 957

807 2086 2234

807 2092 60

807 2095	63
807 2101	955
807 2102	250
807 2370	60
807 2512	829
807 3324	75
807 3490	542
807 5089	1338
807 5926	153
807 9103	57
807 9105	272
807 10226	398
807 10227	344
807 10228	294
807 10229	278
807 10230	254
807 10231	223
807 10232	201
807 10233	191
807 10234	156
807 10235	133
807 10236	126
807 10237	122
807 10238	115
807 10239	101
807 10240	96
807 10241	81
807 10242	75
807 10243	69
807 10244	61

807 10245 60

807 10246 59

807 10247 56

808 55 2004

808 67 5585

808 89 4158

808 182 142

808 229 413

808 288 652

808 292 368

808 300 661

808 301 624

808 302 161

808 320 136

808 328 175

808 333 244

808 352 195

808 425 375

808 437 133

808 466 131

808 523 302

808 525 3320

808 526 145

808 534 3098

808 542 187

808 546 528

808 690 256

808 701 198

808 714 161

808 962 398

808 1044 175

808 1047 181

808 1098 134

808 1106 864

808 1379 575

808 1632 229

808 2020 251

808 2521 269

808 3057 158

808 3164 544

808 3200 1604

808 3319 139

808 3322 396

808 3672 586

808 4303 344

808 4716 243

808 4828 203

808 8589 256

808 8710 328

808 9230 257

808 9729 365

808 10248 300

808 10249 148

809 67 12

809 89 7224

809 155 40

809 161 1539

809 190 19

809 288 1938

809 289 3403

809 295 1478

809 299 135

809 300 1385

809 301 19

809 302 131

809 306 337

809 314 23

809 320 21

809 325 15

809 328 22

809 333 15

809 349 428

809 377 17

809 378 29

809 466 1719

809 468 39

809 486 101

809 525 41

809 528 270

809 531 68

809 538 23

809 541 88

809 542 94

809 543 212

809 544 323

809 679 1049

809 701 195

809 790 21

809 802 73

809 1034 31

809 1037	65
809 1052	23
809 1104	16
809 1429	368
809 1444	54
809 1452	164
809 1453	120
809 1456	410
809 1475	2909
809 2025	19
809 3179	15
809 5463	100
809 7994	1550
810 333 99	
810 377 14	
810 378 98	
810 972 127	
810 1104	13
810 2180	23
810 2298	34
810 2305	167
810 2308	17
810 2309	53
810 2312	243
810 2320	14
810 2336	15
810 2338	97
810 3462	27
810 7157	20
810 7262	24

810	10250	462
810	10251	300
810	10252	284
810	10253	253
810	10254	156
810	10255	76
810	10256	73
810	10257	63
810	10258	55
810	10259	49
810	10260	36
810	10261	36
810	10262	36
810	10263	33
810	10264	32
810	10265	31
810	10266	29
810	10267	27
810	10268	27
810	10269	27
810	10270	26
810	10271	24
810	10272	24
810	10273	22
810	10274	20
810	10275	20
810	10276	19
810	10277	17
810	10278	16
810	10279	15

810 10280 15

810 10281 14

810 10282 13

812 198 482

812 209 476

812 377 453

812 500 457

812 707 690

812 768 442

812 770 365

812 785 326

812 810 369

812 814 555

812 815 654

812 818 278

812 823 325

812 832 362

812 839 495

812 842 346

812 843 369

812 846 387

812 850 351

812 913 288

812 918 449

812 953 277

812 956 331

812 978 319

812 1044 429

812 1104 540

812 1249 436

812 1372 429

812 1440 356

812 1672 649

812 2151 364

812 2209 304

812 2384 290

812 3234 280

812 3236 288

812 3717 492

812 4170 349

812 10299 1766

812 10300 519

812 10301 512

812 10302 485

812 10303 443

812 10304 413

812 10305 393

812 10306 329

812 10307 316

812 10308 300

812 10309 287

812 10310 286

812 10311 276

813 53 97

813 58 139

813 64 127

813 65 97

813 70 85

813 81 287

813 137 83

813 154 375

813 195 285

813 197 480

813 228 272

813 229 89

813 231 81

813 238 90

813 418 158

813 424 119

813 432 81

813 444 167

813 445 106

813 512 94

813 527 84

813 621 86

813 748 224

813 903 485

813 981 121

813 1098 250

813 1400 225

813 1486 91

813 1609 93

813 1735 91

813 1824 116

813 1983 149

813 2277 120

813 2375 85

813 2605 162

813 2923 143

813 3298 80

813 4045 125

813 4113 183

813 4118 87

813 4316 94

813 5927 131

813 6409 207

813 6582 232

813 6585 83

813 6756 92

813 10312 181

813 10313 169

813 10314 95

813 10315 88

814 89 509

814 182 836

814 228 511

814 229 281

814 230 472

814 288 411

814 300 478

814 318 340

814 321 3564

814 325 237

814 327 226

814 333 1506

814 412 501

814 455 254

814 461 1141

814 471 145

814 475 132

814 498 2034

814 528 317

814 540 588

814 646 184

814 681 3542

814 683 402

814 687 371

814 693 151

814 704 615

814 1035 245

814 1043 232

814 1045 307

814 1047 472

814 1048 155

814 1059 284

814 1193 183

814 1239 283

814 1246 1105

814 1444 152

814 1447 160

814 1453 152

814 2080 166

814 2607 233

814 2665 268

814 3079 196

814 3859 172

814 4562 174

814 4814 356

814 5958 464

814 6601 158

814 10316 254

814 10317 169

814 10318 138

815 89 181

815 251 1365

815 257 1400

815 288 28004

815 289 856

815 291 1046

815 292 879

815 293 1035

815 294 1541

815 295 1372

815 300 5765

815 302 1191

815 306 2215

815 310 2228

815 311 44

815 316 578

815 318 218

815 320 661

815 324 1088

815 325 2977

815 326 201

815 330 237

815 333 8380

815 344 2738

815 349 1060

815 352 138

815 355 811

815 458 78

815 464 2509

815 465 384

815 466 3323

815 498 1683

815 523 425

815 525 418

815 538 2101

815 680 602

815 691 76

815 701 2909

815 907 66

815 908 1158

815 1450 478

815 1580 526

815 1613 1605

815 2020 91

815 2544 302

815 2546 275

815 3393 35

815 4512 42

815 8360 65

815 10319 79

816 55 7

816 89 799

816 288 31

816 289 11

816 291 4

816 292 14

816 293 5

816 295 16

816 296 4

816 298 12

816 299 9

816 300 13

816 301 5

816 302 6

816 306 25

816 310 6

816 318 5

816 320 7

816 325 5

816 329 6

816 331 4

816 349 16

816 352 9

816 458 6

816 461 9

816 466 20

816 523 4

816 525 8

816 526 6

816 528 7

816 541 7

816 543 5

816 545 17

816 686 8

816 690 4

816 701 7

816 907 13

816 908 8

816 915 9

816 982 7

816 1034 4

816 1441 7

816 2543 5

816 3080 36

816 3316 5

816 3397 6

816 4562 8

816 4814 4

816 5984 4

816 10320 6

817 154 376

817 159 276

817 212 235

817 226 262

817 229 277

817 230 276

817 324 240

817 470 252

817 471 258

817 481 258

817 488 368

817 504 477

817 511 367

817 533 262

817 548 238

817 614 532

817 683 249

817 689 244

817 734 475

817 854 382

817 959 355

817 966 295

817 969 236

817	1375	278
817	1376	284
817	1377	250
817	1470	487
817	1515	400
817	1517	285
817	1905	256
817	2352	238
817	2361	414
817	2607	457
817	2815	294
817	2840	290
817	2941	404
817	3078	412
817	3252	314
817	3753	257
817	4177	382
817	4179	269
817	5666	241
817	6249	365
817	6348	560
817	7183	441
817	10318	260
817	10321	382

817 10322 324

817 10323 293

817 10324 254

818 7 399

818 65 1093

818 154 456

818 182 335

818 190 1616

818 207 645

818 209 472

818 210 472

818 220 465

818 229 1215

818 230 636

818 366 2984

818 377 482

818 412 783

818 475 428

818 489 397

818 498 364

818 614 916

818 707 564

818 716 290

818 808 292

818 917 501

818 959 480

818 1048 1366

818 1090 437

818 1151 646

818 1179 951

818 1186	2438

818 1198	450

818 1390	1057

818 1400	296

818 1406	1028

818 1639	471

818 1778	389

818 1966	289

818 2131	412

818 3945	685

818 4196	1651

818 4435	475

818 4616	727

818 4821	523

818 7260	368

818 7283	525

818 8262	595

818 8577	423

818 9831	330

818 10325	1818

818 10326	792

818 10327	359

818 10328	327

819 55	214

819 67	922

819 89	3113

819 251 325

819 257 1100

819 265 186

819 285 203

819 288 958

819 289 100761

819 291 303

819 292 1275

819 294 2455

819 295 373

819 298 160

819 299 163

819 300 450

819 318 298

819 320 171

819 325 352

819 326 130

819 329 147

819 333 755

819 349 213

819 352 202

819 374 167

819 377 162

819 466 267

819 523 121

819 525 127

819 530 242

819 908 121

819 1580 398

819 2092 179

819 2094 343

819 2523 192

819 3330 1380

819 5778 172

819 7393	1253
819 7864	262
819 10329	131
819 10330	116
820 5352	17
820 6588	8
820 7148	8
820 10331	32
820 10332	29
820 10333	28
820 10334	25
820 10335	22
820 10336	22
820 10337	20
820 10338	19
820 10339	19
820 10340	19
820 10341	19
820 10342	17
820 10343	17
820 10344	17
820 10345	17
820 10346	17
820 10347	16
820 10348	16
820 10349	14
820 10350	14
820 10351	13
820 10352	13
820 10353	13

820	10354	13
820	10355	12
820	10356	11
820	10357	11
820	10358	10
820	10359	10
820	10360	10
820	10361	10
820	10362	10
820	10363	9
820	10364	9
820	10365	9
820	10366	9
820	10367	9
820	10368	9
820	10369	8
820	10370	8
820	10371	8
820	10372	8
820	10373	8
820	10374	8
820	10375	8
820	10376	8
820	10377	8
821	65	55
821	72	70
821	154	72
821	159	44
821	190	51
821	199	50

821 207 55

821 220 48

821 228 81

821 233 885

821 378 43

821 418 139

821 436 670

821 441 92

821 486 63

821 599 112

821 689 94

821 843 67

821 969 61

821 982 47

821 1106 112

821 1116 84

821 1231 74

821 1377 66

821 1688 173

821 1787 62

821 1964 108

821 2139 43

821 3733 62

821 5423 450

821 7188 76

821 7650 211

821 8500 177

821 8587 111

821 8915 89

821 10116 48

821 10378 143

821 10379 129

821 10380 125

821 10381 90

821 10382 90

821 10383 77

821 10384 72

821 10385 70

821 10386 66

821 10387 58

821 10388 55

821 10389 52

821 10390 46

821 10391 45

822 227 1376

822 234 837

822 245 4000

822 250 771

822 266 1803

822 267 2157

822 271 1573

822 366 1453

822 447 734

822 475 1412

822 567 1274

822 617 5855

822 630 1495

822 718 783

822 1237 780

822 1543 3277

822 1613	1058
822 1614	5289
822 1620	863
822 1636	2504
822 1639	944
822 1640	1678
822 1833	743
822 1946	2765
822 1952	1052
822 1963	1088
822 1974	1191
822 2637	1764
822 2716	986
822 2893	922
822 2931	1704
822 3227	2477
822 3357	1245
822 3710	733
822 4108	990
822 4586	1097
822 4597	2351
822 4779	2693
822 6343	1475
822 6581	737
822 8657	829
822 10392	3462
822 10393	2083
822 10394	1721
822 10395	1212
822 10396	1113

822 10397 1051

822 10398 971

822 10399 908

822 10400 750

823 197 3121

823 209 1003

823 484 651

823 493 870

823 497 1048

823 498 681

823 504 896

823 540 1009

823 599 1285

823 768 738

823 815 752

823 818 913

823 867 2303

823 964 745

823 1091 777

823 1116 1086

823 1179 671

823 1206 2533

823 1650 959

823 2150 1475

823 2576 663

823 2580 732

823 3086 656

823 3242 919

823 3328 968

823 3341 856

823 3360 1995

823 3455 1476

823 3621 2692

823 4051 1394

823 4189 1833

823 4724 660

823 5202 750

823 6257 956

823 6391 1360

823 6773 1024

823 7650 875

823 7678 5151

823 8065 694

823 9960 2042

823 10401 1406

823 10402 1374

823 10403 862

823 10404 853

823 10405 814

823 10406 778

823 10407 716

823 10408 695

823 10409 676

823 10410 652

824 65 43

824 154 71

824 163 24

824 190 25

824 199 29

824 203 46

824 206 22

824 207 30

824 208 28

824 210 24

824 212 45

824 217 31

824 221 90

824 222 27

824 225 30

824 227 88

824 228 27

824 229 31

824 424 54

824 428 29

824 429 22

824 434 30

824 436 23

824 533 49

824 554 34

824 599 28

824 618 24

824 630 26

824 709 32

824 716 42

824 718 28

824 733 40

824 735 38

824 857 26

824 859 42

824 863 24

824 969 30

824 1048 22

824 1090 54

824 1239 25

824 1375 22

824 1515 22

824 1957 21

824 1958 25

824 2614 23

824 3258 25

824 3469 28

824 4079 73

824 10411 34

824 10412 22

825 154 113

825 183 217

825 190 107

825 203 50

825 207 97

825 210 70

825 227 124

825 421 259

825 422 225

825 438 103

825 439 364

825 447 225

825 612 68

825 618 216

825 629 422

825 1155 48

825 1207	87
825 1400	530
825 1755	61
825 1761	95
825 1764	62
825 1947	104
825 2401	118
825 2535	44
825 2637	50
825 3747	81
825 4796	45
825 4834	47
825 5416	457
825 6137	64
825 6146	67
825 7812	174
825 8583	68
825 9480	94
825 10413	320
825 10414	232
825 10415	212
825 10416	190
825 10417	180
825 10418	175
825 10419	141
825 10420	113
825 10421	100
825 10422	64
825 10423	56
825 10424	55

825 10425 50
825 10426 46
825 10427 41
825 10428 40
826 88 21
826 89 98
826 173 13
826 190 30
826 289 374
826 300 111
826 374 195
826 375 14
826 377 163
826 461 40
826 466 150
826 475 28
826 486 25
826 488 33
826 500 20
826 518 16
826 534 11
826 546 55
826 701 702
826 704 96
826 707 42
826 923 15
826 930 44
826 1081 14
826 1104 43
826 1338 39

826 1410	39
826 1452	24
826 1456	15
826 1463	30
826 1913	25
826 2009	18
826 2102	42
826 2963	15
826 3324	36
826 4575	84
826 5083	132
826 9462	38
826 10429	173
826 10430	137
826 10431	38
826 10432	31
826 10433	24
826 10434	24
826 10435	16
826 10436	14
826 10437	14
826 10438	14
826 10439	14
826 10440	10

827 176 20

827 706 79

827 707 45

827 720 45

827 724 22

827 732 142

827 841 19

827 918 91

827 952 264

827 1206	66
827 1249	777
827 1360	73
827 1369	364
827 1372	23
827 1513	18
827 1803	74
827 1814	27
827 2343	44
827 2360	106
827 2364	27
827 2372	546
827 2595	98
827 2781	295
827 2784	79
827 2790	71
827 2795	23
827 3445	18
827 3456	98
827 3471	134
827 3792	62
827 4457	268
827 4458	34
827 4794	43
827 4807	273
827 5010	62
827 5014	251

827 5105 19
827 7047 93
827 10441 267
827 10442 127
827 10443 89
827 10444 78
827 10445 69
827 10446 63
827 10447 36
827 10448 32
827 10449 25
827 10450 25
827 10451 20
827 10452 18
828 152 82
828 163 65
828 176 64
828 614 79
828 732 65
828 851 72
828 917 205
828 920 182
828 928 176
828 937 78
828 944 138
828 959 75
828 1110 110
828 1132 148
828 1338 84
828 1343 221

828 1358	95
828 1369	165
828 1372	171
828 1412	128
828 1413	146
828 1703	178
828 1810	82
828 1813	103
828 2787	109
828 2853	124
828 2901	65
828 3264	169
828 3476	79
828 3481	115
828 3774	218
828 3779	154
828 3921	103
828 4124	150
828 4280	74
828 4286	70
828 4784	97
828 5365	82
828 5605	150
828 5697	115
828 7423	103
828 7797	103
828 7900	110
828 9440	68
828 10453	163
828 10454	123

828 10455 92

828 10456 89

828 10457 72

828 10458 65

829 81 291

829 154 288

829 159 213

829 195 365

829 199 295

829 205 288

829 206 327

829 210 381

829 217 300

829 222 412

829 228 251

829 229 407

829 235 226

829 418 208

829 424 217

829 425 500

829 426 198

829 433 278

829 434 276

829 440 418

829 602 249

829 615 638

829 618 374

829 622 195

829 716 270

829 748 238

829 858 346

829 961 325

829 1048 296

829 1062 317

829 1090 259

829 1098 330

829 1167 239

829 1375 251

829 1383 275

829 1400 225

829 1424 370

829 1504 232

829 1947 235

829 1983 342

829 2277 311

829 2407 294

829 3110 262

829 3186 270

829 3258 356

829 3259 340

829 3902 197

829 4704 206

829 5914 237

829 6417 203

830 65 204

830 67 124

830 89 588

830 257 134

830 283 238

830 288 615

830 289 1817

830 295 188

830 300 261

830 302 202

830 306 244

830 327 110

830 328 147

830 329 88

830 333 189

830 349 361

830 377 238

830 439 141

830 455 344

830 458 97

830 459 105

830 461 381

830 466 100

830 484 315

830 498 90

830 533 87

830 542 93

830 646 2159

830 701 179

830 714 446

830 802 127

830 1241 86

830 1428 462

830 1429 414

830 1430 87

830 1453 90

830 1659 238

830 1681 392

830 2523 102

830 2983 548

830 3332 226

830 4350 125

830 4466 114

830 4561 159

830 4828 2031

830 4840 131

830 6120 462

830 6121 320

830 10459 138

830 10460 96

831 57 1752

831 63 3986

831 66 3409

831 67 2766

831 69 1618

831 72 2653

831 77 1481

831 86 1704

831 155 3411

831 157 2188

831 429 1585

831 511 1923

831 518 1632

831 533 1635

831 601 1696

831 706 1531

831 871 1733

831 903 2121

831 959 1597

831 975 1692

831 993 2723

831 1001 3503

831 1122 2140

831 1412 1533

831 1463 1620

831 1793 2048

831 2524 1638

831 3307 1771

831 3863 1755

831 4155 1634

831 4156 2614

831 5254 2609

831 6128 1792

831 7068 1727

831 7157 3656

831 7874 2854

831 8853 2623

831 9685 1566

831 9888 3205

831 10176 2866

831 10182 2237

831 10461 3622

831 10462 3265

831 10463 2671

831 10464 2350

831 10465 2314

831 10466 2088

831 10467 2017

831 10468 1904

831 10469 1872

832 51 167

832 72 164

832 81 87

832 152 207

832 181 72

832 187 187

832 188 171

832 190 147

832 518 121

832 607 102

832 851 72

832 917 83

832 920 141

832 928 93

832 930 80

832 936 207

832 942 360

832 943 96

832 944 82

832 955 104

832 1001 73

832 1089 84

832 1113 173

832 1343 147

832 1358 236

832 1369 116

832 1372 91

832 1464 122

832 1810 82

832 2003 122

832 2005 96

832 2013 82

832 2014 104

832 2343 148

832 2787 205

832 2975 79

832 3201 80

832 3425 86

832 3901 160

832 3940 117

832 4119 75

832 9125 115

832 10453 116

832 10470 113

832 10471 102

832 10472 102

832 10473 92

832 10474 92

832 10475 79

832 10476 72

834 378 142

834 412 189

834 768 264

834 781 178

834 808 469

834 1104 428

834 1186	142
834 1206	138
834 1547	137
834 1650	197
834 2033	177
834 2732	308
834 2735	145
834 3239	314
834 3543	188
834 3734	199
834 3834	225
834 3943	333
834 4218	132
834 4229	288
834 5600	129
834 6997	146
834 7008	157
834 7329	174
834 10482	372
834 10483	301
834 10484	232
834 10485	224
834 10486	221
834 10487	209
834 10488	200
834 10489	192
834 10490	172
834 10491	168
834 10492	163
834 10493	158

834 10494 155

834 10495 153

834 10496 143

834 10497 138

834 10498 136

834 10499 135

834 10500 133

834 10501 132

834 10502 130

834 10503 130

834 10504 129

834 10505 129

834 10506 125

834 10507 125

835 157 89

835 163 82

835 176 106

835 187 43

835 227 97

835 230 81

835 292 59

835 377 22

835 389 24

835 475 35

835 486 137

835 504 105

835 544 30

835 706 54

835 707 143

835 724 171

835 732 42

835 735 41

835 918 62

835 951 433

835 952 95

835 956 29

835 959 52

835 1076 25

835 1249 778

835 1366 24

835 1379 27

835 1412 162

835 1803 86

835 1804 86

835 1808 42

835 1810 354

835 1814 94

835 1892 37

835 2341 55

835 2343 166

835 2346 41

835 2347 1132

835 2661 38

835 2787 128

835 2790 87

835 2859 27

835 2861 25

835 4165 76

835 4180 265

835 4460 257

835 6064 29

835 6292 33

835 10508 48

835 10509 45

836 55 240

836 58 26

836 67 100

836 77 39

836 157 513

836 161 218

836 251 42

836 257 2043

836 262 79

836 265 39

836 266 22

836 272 34

836 285 63

836 292 3625

836 294 97

836 299 160

836 301 814

836 302 324

836 306 70

836 316 59

836 329 146

836 362 130

836 423 56

836 525 118

836 534 360

836 689 221

836 697 152

836 701 1607

836 1076 76

836 1475 52

836 1601 234

836 1772 59

836 2175 48

836 2220 181

836 3237 73

836 3672 59

836 4619 25

836 4704 25

836 4883 82

836 4920 31

836 5746 254

836 6317 23

836 6385 48

836 6426 27

836 8878 27

836 10510 105

836 10511 66

836 10512 34

836 10513 30

836 10514 29

837 53 209

837 81 341

837 126 237

837 154 816

837 162 222

837 237 225

837 238 300

837 418 624

837 444 258

837 603 329

837 616 393

837 621 329

837 632 197

837 748 447

837 755 341

837 756 114

837 868 205

837 1098 412

837 1109 224

837 1222 315

837 1418 405

837 1737 116

837 1745 324

837 1747 134

837 1781 327

837 1787 173

837 1790 670

837 1835 286

837 1965 228

837 2145 210

837 2605 331

837 2668 549

837 2716 155

837 2811 196

837 3411 159

837 3902 176

837 4113 157

837 4118 162

837 4852 130

837 5876 129

837 6043 193

837 7060 149

837 7382 129

837 9783 150

837 10515 226

837 10516 188

837 10517 181

837 10518 172

837 10519 132

837 10520 116

838 72 138

838 81 253

838 88 265

838 103 328

838 152 419

838 154 2096

838 156 130

838 173 196

838 203 163

838 206 160

838 233 1518

838 237 283

838 238 1133

838 407 333

838 418 1112

838 512 248

838 517 458

838 603 214

838 620 126

838 748 265

838 1085 129

838 1098 574

838 1118 250

838 1131 153

838 1146 510

838 1394 242

838 1418 208

838 1510 220

838 1705 139

838 1731 123

838 1738 247

838 1787 856

838 1790 247

838 1965 152

838 3884 494

838 4069 213

838 4316 592

838 4734 228

838 5545 332

838 5806 186

838 6585 217

838 6823 710

838 7152 356

838 7649 136

838 9414 126

838 10456 172

838 10521 234

838 10522 147

838 10523 133

838 10524 123

839 45 706

839 190 262

839 233 361

839 377 1275

839 408 2912

839 475 464

839 486 290

839 488 1311

839 493 949

839 497 308

839 503 1384

839 507 309

839 514 334

839 519 1310

839 537 746

839 707 346

839 806 1406

839 808 932

839 810 1079

839 811 786

839 830 1200

839 834 287

839 835 621

839 966 272

839 978 530

839 1044 1171

839 1116 435

839 1145 654

839 1179 553

839 1180 693

839 1181 386

839 1191 308

839 1195 853

839 1406 498

839 1410 665

839 1854 678

839 1859 607

839 1874 1379

839 2846 505

839 2851 265

839 2944 1448

839 2956 596

839 2961 983

839 3240 324

839 5972 848

839 7078 361

839 7113 1051

839 8254 364

839 9761 662

839 10525 413

840 154 2234

840 163 904

840 172 341

840 173 964

840 220 390

840 233 698

840 234 816

840 237 338

840 418 3381

840 562 369

840 614 63627

840 615 5362

840 706 1775

840 715 457

840 854 1074

840 856 776

840 860 375

840 868 1119

840 1092 909

840 1098 6011

840 1109 342

840 1118 576

840 1412 1372

840 1639 553

840 1655 1109

840 1660 539

840 1778 2430

840 2346 1394

840 2446 1228

840 2786 828

840 4092 455

840 4359 497

840 5236 534

840 7623 376

840 10526 8784

840 10527 6166

840 10528 3435

840 10529 3005

840 10530 2971

840 10531 2635

840 10532 2602

840 10533 2298

840 10534 1968

840 10535 1665

840 10536 1625

840 10537 1092

840 10538 981

840 10539 970

840 10540 384

840 10541 363

841 45 221

841 65 22

841 71 44

841 184 52

841 194 30

841 198 22

841 306 44

841 366 140

841 438 34

841 472 20

841 498 154

841 500 178

841 521 40

841 536 34

841 538 473

841 705 107

841 708 28

841 818 203

841 830 213

841 930 219

841 1044 71

841 1046 34

841 1048 164

841 1104 22

841 1180 178

841 1191 140

841 1378 170

841 1380 335

841 1406 57

841 1425 35

841 1672 14164

841 3057 52

841 3089 20

841 3384 26

841 3746 19

841 4473 27

841 4549 55

841 5082 51

841 6120 20

841 6480 39

841 7038 23

841 7650 22

841 8161 37

841 9766 66

841 9928 20

841 10542 52

841 10543 35

841 10544 31

841 10545 25

841 10546 20

842 107 268

842 267 182

842 331 196

842 603 235

842 617 259

842 619 209

842 1236 179

842 1613 211

842 1614 183

842 1622 283

842 1627 1074

842 1943 1155

842 1945 189

842 1946 584

842 1950 197

842 1953 181

842 1959 231

842 1963 240

842 1969 861

842 2086 265

842 2101 468

842 2132 178

842 2667 462

842 2668 207

842 2909 188

842 2921 275

842 2923 358

842 3132 187

842 4779 308

842 5150 210

842 5565 254

842 5868 197

842 6046 379

842 6336 274

842 10547 449

842 10548 444

842 10549 293

842 10550 286

842 10551 262

842 10552 260

842 10553 259

842 10554 247

842 10555 213

842 10556 205

842 10557 204

842 10558 202

842 10559 197

842 10560 194

842 10561 194

842 10562 178

843 230 23

843 412 136

843 605 42

843 1186 18

843 1406 55

843 1481 217

843 1528	42
843 1645	25
843 2637	24
843 3032	210
843 3043	30
843 3737	23
843 4097	13
843 4189	13
843 4194	38
843 4198	201
843 4204	76
843 4211	82
843 4212	14
843 4213	37
843 4504	13
843 4821	92
843 5305	55
843 5315	17
843 5600	29
843 8275	17
843 8925	63
843 8931	63
843 8932	67
843 8947	29
843 9801	16
843 10563	102
843 10564	50
843 10565	44
843 10566	41
843 10567	34

843 10568 33

843 10569 28

843 10570 27

843 10571 25

843 10572 24

843 10573 22

843 10574 21

843 10575 21

843 10576 20

843 10577 19

843 10578 19

843 10579 17

843 10580 14

843 10581 13

844 7 738

844 8 189

844 40 453

844 89 382

844 173 229

844 190 386

844 199 300

844 209 638

844 227 898

844 229 853

844 230 207

844 333 330

844 464 230

844 470 240

844 481 271

844 486 529

844 498 2030

844 536 428

844 542 213

844 548 223

844 768 797

844 779 2230

844 972 267

844 1035	327
844 1044	403
844 1180	241
844 1185	961
844 1188	184
844 1196	384
844 1673	341
844 1678	468
844 1853	1707
844 1910	1985
844 2080	241
844 2130	202
844 2210	664
844 2661	311
844 2847	464
844 2850	665
844 2959	559
844 3187	213
844 3367	197
844 5082	264
844 5149	266
844 5308	181
844 6543	474

844 7724 209

844 8398 260

844 10582 527

844 10583 323

845 188 152

845 503 180

845 533 6

845 593 6

845 607 25

845 707 605

845 726 1

845 812 402

845 841 1

845 917 773

845 918 381

845 920 110

845 949 68

845 1249 29

845 1274 1

845 1358 1

845 1360 238

845 1364 1

845 1411 20

845 2006 7

845 4787 172

845 6282 188

845 9552 7

845 10584 93

845 10585 62

845 10586 56

845 10587 3

845 10588 3

846 56 847

846 88 1047

846 173 771

846 234 11081

846 429 803

846 441 2217

846 537 1586

846 631 798

846 757 1092

846 854 706

846 866 534

846 875 2082

846 1202 742

846 1205 1457

846 1206 1042

846 1222 772

846 2026 2233

846 2030 1392

846 2043 1016

846 2470 924

846 2471 506

846 2475 989

846 2481 523

846 2486 963

846 2542 511

846 2624 760

846 2637 661

846 3431 1135

846 3432 894

846 3442 499

846 3456 612

846 3508 1834

846 3740 2906

846 4087 6419

846 4359 805

846 5113 655

846 5299 728

846 5641 743

846 7343 526

846 7389 735

846 10007 806

846 10164 740

846 10589 1089

846 10590 966

846 10591 755

846 10592 723

846 10593 619

846 10594 533

846 10595 517

846 10596 481

847 161 55

847 288 111

847 289 55

847 295 57

847 302 155

847 308 60

847 317 214

847 331 132

847 353 75

847 403 77

847 468 94

847 484 207

847 490 77

847 547 70

847 548 68

847 701 74

847 914 79

847 1032 112

847 1042 79

847 1377 239

847 1449 99

847 1507 53

847 1588 135

847 1648 157

847 1854 200

847 2242 60

847 2977 129

847 2990 62

847 3316 53

847 3404 61

847 4177 59

847 4814 123

847 5151 74

847 5228 107

847 5768 92

847 7340 390

847 7868 138

847 8155 158

847 10597 213
847 10598 118
847 10599 100
847 10600 99
847 10601 82
847 10602 81
847 10603 69
847 10604 68
847 10605 67
847 10606 63
847 10607 59
847 10608 55
848 197 648
848 199 341
848 218 525
848 222 731
848 236 334
848 416 1112
848 429 419
848 433 333
848 440 465
848 445 335
848 612 482
848 615 441
848 622 308
848 629 308
848 632 363
848 757 365
848 898 324
848 1418 621

848 1424	402
848 1732	425
848 1944	482
848 1958	323
848 2139	467
848 2402	652
848 2535	802
848 2541	370
848 3260	524
848 3347	358
848 3411	332
848 3430	387
848 3685	550
848 3694	377
848 3714	587
848 4053	483
848 6151	439
848 6159	1306
848 6910	407
848 6911	316
848 8705	300
848 9457	684
848 10384	642
848 10517	340
848 10609	591
848 10610	422
848 10611	376
848 10612	375
848 10613	340
848 10614	327

848 10615 324

848 10616 322

849 65 355

849 154 1066

849 170 233

849 190 658

849 199 766

849 206 269

849 207 1772

849 217 204

849 225 1718

849 226 276

849 227 487

849 228 207

849 229 1532

849 233 990

849 418 326

849 424 2000

849 432 203

849 439 501

849 441 385

849 533 270

849 599 647

849 615 729

849 716 210

849 881 212

849 964 679

849 977 291

849 1090 202

849 1206 209

849 1239 900

849 1378 1256

849 1520 1286

849 1929 205

849 2363 361

849 2364 1401

849 2372 1396

849 2373 246

849 2524 888

849 3469 216

849 4457 1136

849 4459 411

849 4793 311

849 5926 202

849 6539 202

849 6726 254

849 8470 224

849 8710 447

849 10617 564

849 10618 513

849 10619 307

849 10620 227

850 30 12464

850 32 1002

850 55 1165

850 72 3279

850 82 28945

850 153 948

850 194 2637

850 463 784

850 877 1301

850 986 2361

850 1001	5719
850 1014	4641
850 1104	1158
850 1244	978
850 2029	826
850 2030	2516
850 2459	1090
850 3501	736
850 3768	12350
850 3966	754
850 3988	689
850 4317	999
850 4872	1548
850 6232	1050
850 6241	4626
850 6245	2282
850 6930	1428
850 8314	799
850 9028	1198
850 9299	746
850 9869	685
850 10130	1232
850 10621	5399
850 10622	2826
850 10623	2320
850 10624	1893
850 10625	1605
850 10626	1488

850 10627 1063

850 10628 1053

850 10629 1042

850 10630 932

850 10631 896

850 10632 858

850 10633 818

850 10634 782

850 10635 751

850 10636 744

850 10637 714

850 10638 676

851 55 2573

851 67 1247

851 89 7775

851 288 5698

851 289 4124

851 290 2836

851 291 5552

851 292 2006

851 293 2862

851 294 2354

851 295 2239

851 296 2555

851 298 1814

851 299 6714

851 300 7559

851 302 3179

851 310 2737

851 311 1404

851 316 1279

851 317 1263

851 318 15706

851 324 2586

851 325 5712

851 333 2512

851 342 2898

851 344 6914

851 386 1556

851 461 4218

851 466 2043

851 498 4975

851 523 5128

851 524 1846

851 674 1382

851 676 1724

851 680 1447

851 681 2004

851 682 1981

851 686 2640

851 790 2426

851 908 1247

851 972 7340

851 1034 2409

851 1441 1311

851 1684 4775

851 4707 3605

851 4773 1836

851 8171 1511

851 10639 2093

851 10640 1386

851 10641 1298

852 55 517

852 67 109

852 71 94

852 89 345

852 230 136

852 257 130

852 288 341

852 289 1035

852 290 153

852 291 301

852 292 1327

852 294 107

852 295 282

852 296 192

852 298 95

852 300 790

852 301 170

852 306 113

852 311 715

852 317 144

852 318 454

852 323 560

852 325 171

852 333 97

852 349 379

852 352 237

852 374 315

852 375 147

852 455 897

852 461 220

852 466 303

852 479 104

852 525 485

852 528 185

852 543 119

852 686 122

852 689 276

852 906 3915

852 908 177

852 1034 144

852 1459 135

852 1519 212

852 1684 225

852 1694 100

852 2083 118

852 2521 1448

852 2607 113

852 3201 576

852 5533 154

852 8654 377

853 7 164

853 55 134

853 71 3612

853 86 175

853 95 158

853 166 175

853 184 1071

853 291 1544

853 292 282

853 294 183

853 298 487

853 300 385

853 302 332

853 305 209

853 311 255

853 316 1866

853 333 1433

853 378 549

853 465 149

853 517 135

853 518 144

853 535 248

853 592 255

853 680 171

853 701 526

853 707 351

853 786 346

853 891 597

853 909 999

853 930 248

853 974 137

853 991 596

853 1052 785

853 1114 131

853 1459 235

853 1463 465

853 1505 398

853 1772 527

853 1791 1175

853 2014 259

853 2022 260

853 2067 604

853 2454 353

853 2734 938

853 2741 598

853 3047 186

853 3460 135

853 3881 134

853 5459 494

853 9961 132

854 198 33

854 278 72

854 489 145

854 507 79

854 514 90

854 805 119

854 815 396

854 818 338

854 823 91

854 830 236

854 831 321

854 846 34

854 850 38

854 1188 83

854 1547 136

854 2151 46

854 2168 51

854 2187 93

854 2429	74
854 2565	36
854 2576	144
854 2586	100
854 2955	35
854 2956	93
854 3227	117
854 3245	44
854 3719	213
854 3720	220
854 3943	36
854 4003	49
854 4015	35
854 4179	32
854 4948	126
854 6017	32
854 6440	38
854 7639	337
854 8277	496
854 8350	40
854 8610	79
854 10491	63
854 10525	44
854 10642	136
854 10643	131
854 10644	120
854 10645	88
854 10646	61
854 10647	57
854 10648	40

854 10649 34

854 10650 33

855 59 1178

855 84 661

855 159 484

855 182 1016

855 207 28108

855 208 1392

855 248 1180

855 276 3661

855 428 6230

855 429 1909

855 430 2287

855 441 1550

855 443 5602

855 605 535

855 618 1018

855 716 468

855 857 2159

855 867 704

855 986 5581

855 1048 2482

855 1090 492

855 1512 1943

855 1514 743

855 1515 583

855 1946 854

855 1950 1084

855 1963 2392

855 2131 471

855 2133	4100
855 2582	1533
855 2818	500
855 3767	969
855 4129	3821
855 4246	1445
855 4250	1251
855 5040	1011
855 7324	408
855 7745	2457
855 8600	1169
855 10651	1596
855 10652	1584
855 10653	1340
855 10654	1262
855 10655	1101
855 10656	995
855 10657	989
855 10658	666
855 10659	541
855 10660	418
855 10661	411

856 154 1314

856 163 508

856 173 1118

856 199 168

856 207 275

856 208 128

856 220 312

856 226 666

856 227 636

856 230 136

856 233 272

856 234 990

856 240 132

856 424 302

856 428 221

856 429 128

856 436 3728

856 441 377

856 453 419

856 533 150

856 562 220

856 614 448

856 709 189

856 854 402

856 855 170

856 857 223

856 859 219

856 875 247

856 898 528

856 964 1102

856 1090 238

856 1118 279

856 1119 151

856 1400 228

856 1406 225

856 1412 326

856 1424 485

856 1504 1175

856 1513 289

856 1639 188

856 1974 186

856 1977 181

856 3354 145

856 3444 192

856 3744 176

856 4359 279

856 4848 367

856 10662 227

856 10663 161

856 10664 145

858 67 263

858 70 128

858 89 338

858 157 8987

858 190 170

858 237 238

858 285 552

858 288 412

858 289 505

858 292 284

858 295 202

858 298 992

858 299 195

858 300 179

858 306 791

858 318 275

858 327 175

858 329 197

858 333 220

858 455 139

858 464 406

858 466 262

858 468 309

858 484 297

858 488 155

858 531 333

858 538 228

858 544 1909

858 701 267

858 703 1227

858 704 175

858 722 127

858 1042 183

858 1244 141

858 1379 215

858 1401 130

858 1409 194

858 1410 127

858 1444 217

858 1455 123

858 1679 175

858 1791 389

858 2020 687

858 2025 128

858 4026 581

858 4242 179

858 5746 221

858 7868 266

858 10670 173

858 10671 120

859 55 575

859 59 366

859 66 176

859 67 733

859 70 301

859 72 4627

859 88 177

859 89 386

859 153 98

859 166 133

859 203 666

859 253 166

859 288 210

859 295 227

859 299 351

859 301 106

859 318 370

859 328 132

859 377 97

859 429 154

859 463 243

859 466 146

859 511 141

859 529 280

859 532 182

859 533 531

859 536 150

859 605 1476

859 661 113

859 701 163

859 986 160

859 1061 518

859 1099 1018

859 1104 506

859 1379 227

859 1520 101

859 1795 155

859 1817 185

859 1990 254

859 2020 97

859 2083 177

859 2220 131

859 2500 126

859 2539 100

859 3739 102

859 4043 134

859 5256 180

859 5683 102

859 6246 313

859 10523 96

860 152 662

860 592 313

860 605 368

860 751 508

860 752 862

860 769 1189

860 882 683

860 917 522

860 1110	1213
860 1131	1004
860 1202	1425
860 1223	484
860 1229	475
860 1369	509
860 1411	579
860 1413	647
860 2006	376
860 2749	393
860 2771	342
860 2772	450
860 2817	498
860 2828	325
860 3028	857
860 3364	558
860 3417	1190
860 3481	331
860 3511	355
860 3523	1027
860 3528	394
860 3703	456
860 3805	1327
860 3809	527
860 4675	522
860 4970	323
860 5299	406
860 5636	382
860 6151	380
860 6576	656

860 6830 760

860 7047 571

860 7265 433

860 9440 527

860 9445 1020

860 9448 395

860 9450 796

860 10672 749

860 10673 390

860 10674 380

860 10675 371

860 10676 336

861 89 1064

861 209 845

861 257 328

861 289 480

861 291 804

861 292 1469

861 295 4160

861 298 297

861 300 1862

861 302 863

861 307 749

861 311 1144

861 320 456

861 333 13205

861 344 285

861 403 243

861 412 166

861 430 163

861 498 254

861 523 551

861 542 843

861 687 194

861 693 397

861 903 397

861 913 555

861 974 223

861 1052 615

861 1182 363

861 1186 301

861 1198 185

861 1243 301

861 1463 161

861 2082 343

861 2094 698

861 2546 386

861 2839 171

861 3081 285

861 3489 4703

861 4436 345

861 4488 277

861 5036 248

861 6233 609

861 7448 177

861 7449 843

861 7835 209

861 9079 170

861 10677 1115

861 10678 276

861 10679 271

861 10680 245

862 89 595

862 190 660

862 288 242

862 289 465

862 298 545

862 300 576

862 302 348

862 333 416

862 349 335

862 430 564

862 461 229

862 466 3920

862 498 810

862 526 478

862 528 405

862 532 1367

862 535 235

862 544 223

862 548 590

862 689 546

862 690 346

862 691 437

862 697 1640

862 797 262

862 901 380

862 972 210

862 1034 10623

862 1098 389

862 1383 215

862 1485 462

862 1688 364

862 1990 368

862 2022 653

862 2024 400

862 2081 193

862 2523 327

862 3077 228

862 3288 213

862 3404 3428

862 4704 700

862 4706 214

862 5147 256

862 5149 212

862 5547 249

862 7660 1424

862 8589 630

862 10681 902

862 10682 439

862 10683 272

862 10684 204

863 56 70

863 65 116

863 89 130

863 154 274

863 173 105

863 182 77

863 190 177

863 207 171

863 226 100

863 227 200

863 228 237

863 229 174

863 300 87

863 420 197

863 424 104

863 425 79

863 432 117

863 498 121

863 533 180

863 546 196

863 716 86

863 757 225

863 887 135

863 903 125

863 969 115

863 980 81

863 1048 124

863 1090 235

863 1106 101

863 1116 79

863 1239 88

863 1246 84

863 1396 156

863 1398 106

863 1400 143

863 1405 82

863 1408 121

863 1632 119

863 2535 90

863 2708 72

863 3186 86

863 3200 135

863 3730 185

863 4025 71

863 4435 92

863 4704 78

863 4732 92

863 5544 80

863 7327 105

863 10685 130

864 51 182

864 65 583

864 67 125

864 89 139

864 154 687

864 166 212

864 180 92

864 190 445

864 193 172

864 198 222

864 203 154

864 206 143

864 207 547

864 220 420

864 227 471

864 228 718

864 234 246

864 289 343

864 316 121

864 333 128

864 424 213

864 425 104

864 436 225

864 439 102

864 453 2287

864 498 156

864 511 92

864 533 96

864 534 93

864 540 106

864 546 156

864 615 175

864 679 440

864 703 101

864 709 102

864 714 115

864 715 106

864 790 330

864 959 134

864 1048 163

864 1079 130

864 1098 1077

864 1400 100

864 2343 91

864 2346 255

864 3057 245

864 3200 135

864 3298 227

864 3400 95

864 3557 153

865 72 26

865 157 46

865 176 58

865 706 27

865 707 97

865 732 264

865 917 33

865 928 55

865 951 155

865 952 335

865 959 70

865 1249 67

865 1358 66

865 1366 793

865 1369 61

865 1527 236

865 1536 31

865 1803 1005

865 1808 295

865 1913 52

865 1914 71

865 2343 708

865 2785 20

865 2787 69

865 2790 70

865 2838 40

865 3470 84

865 3480 100

865 3966 32

865 4165 60

865 4180 113

865 4460 255

865 5004 285

865 5006 42

865 5010 55

865 5011 29

865 5157 244

865 5159 22

865 5900 43

865 6292 60

865 7117 58

865 9345 117

865 9460 111

865 9819 30

865 9822 74

865 10686 158

865 10687 126

865 10688 90

865 10689 60

865 10690 27

868 70 289

868 89 90

868 187 236

868 190 121

868 191 356

868 198 98

868 229 217

868 475 75

868 486 203

868 518 209

868 529 422

868 722 403

868 726 213

868 959 90

868 968 110

868 1009 4013

868 1029 89

868 1104 1048

868 1305 852

868 1796 81

868 2047 79

868 2735 743

868 2835 383

868 3863 499

868 4024 111

868 4036 1045

868 4242 90

868 6243 128

868 6267 92

868 7301 96

868 7316 83

868 8674 79

868 10694 269

868 10695 212

868 10696 190

868 10697 135

868 10698 129

868 10699 118

868 10700 111

868 10701 104

868 10702 100

868 10703 96

868 10704 96

868 10705 95

868 10706 94

868 10707 90

868 10708 88

868 10709 84

868 10710 83

868 10711 77

869 503 2093

869 707 47

869 917 830

869 918 563

869 923 996

869 949 116

869 1104 5

869 2009 19

869 3624 29

869 3806 1

870 159 69

870 163 81

870 220 141

870 227 580

870 230 64

870 234 32

870 412 169

870 500 65

870 533 77

870 540 100

870 542 37

870 687 138

870 705 209

870 706 365

870 708 246

870 714 50

870 724 37

870 917 45

870 932 51

870 966 49

870 1122 328

870 1246 78

870 1249 1694

870 1406 185

870 1412 39

870 1513 148

870 1545 104

870 1672 59

870 1681 48

870 2595 42

870 2789 85

870 2848 51

870 3382 55

870 4087 198

870 4170 33

870 4823 372

870 4828 74

870 4834 118

870 4846 198

870 4848 105

870 5630 40

870 5772 114

870 6120 87

870 6121 58

870 6122 340

870 8713 50

870 10712 226

870 10713 161

870 10714 43

870 10715 39

871 65 750

871 152 2597

871 159 677

871 163 839

871 173 556

871 188 2146

871 225 1954

871 226 1372

871 227 507

871 233 4304

871 316 963

871 436 833

871 441 729

871 512 881

871 689 515

871 707 2981

871 726 650

871 769 674

871 841 588

871 917 771

871 918 1366

871 920 557

871 971 887

871 982 855

871 1110 619

871 1116 749

871 1118 1124

871 1131 1138

871 1216 1839

871 1360 538

871 1384 1684

871 1412 1435

871 1426 681

871 1816 780

871 2369 1602

871 2582 563

871 3057 1567

871 3676 703

871 3892 1979

871 3905 811

871 5153 2334

871 5236 1557

871 5237 2859

871 5244 629

871 5245 1145

871 5247 803

871 6830 1133

871 10716 1177

871 10717 814

871 10718 629

873 55 668

873 67 4909

873 89 6611

873 97 165

873 157 198

873 257 4085

873 279 194

873 285 655

873 288 1860

873 289 39332

873 290 441

873 291 409

873 292 3134

873 294 562

873 295 5229

873 296 571

873 298 457

873 299 667

873 300 1150

873 302 871

873 306 252

873 308 257

873 310 415

873 311 234

873 314 1452

873 317 292

873 325 196

873 329 289

873 332 348

873 333 253

873 344 238

873 349 977

873 354 300

873 377 203

873 378 216

873 461 174

873 466 1215

873 523 192

873 543 405

873 701 1080

873 1037 182

873 1379 239

873 1580 184

873 2025 522

873 10730 453

873 10731 423

873 10732 387

873 10733 210

873 10734 200

873 10735 168

874 66 244

874 126 354

874 154 824

874 170 276

874 171 230

874 173 2582

874 190 1274

874 210 365

874 436 258

874 486 711

874 487 486

874 504 509

874 697 306

874 1066 236

874 1075 2447

874 1091 806

874 1097 605

874 1098 772

874 1126 276

874 1179 222

874 1191 226

874 1298 1949

874 1300 276

874 1375 564

874 1410 516

874 1609 536

874 1745 231

874 1799 282

874 1983 223

874 1988 487

874 2432 387

874 2521 281

874 2711 394

874 3087 223

874 3109 1048

874 3184 234

874 3797 279

874 4044 531

874 4162 332

874 4255 565

874 4734 297

874 5610 325

874 7078 630

874 8503 294

874 10736 705

874 10737 391

874 10738 384

874 10739 316

874 10740 312

874 10741 232

875 67 96

875 89 639

875 161 159

875 191 150

875 288 390

875 289 1053

875 292 1142

875 294 123

875 295 256

875 299 180

875 300 278

875 304 113

875 306 154

875 314 173

875 318 321

875 323 293

875 325 216

875 326 127

875 333 146

875 344 357

875 349 1262

875 352 153

875 458 118

875 461 612

875 466 428

875 486 111

875 528 150

875 531 98

875 543 14470

875 544 224

875 646 388

875 679 249

875 681 124

875 686 459

875 701 163

875 790 101

875 793 108

875 802 354

875 1047 197

875 1189 94

875 1428 174

875 1429 119

875 1451 520

875 1458 102

875 1597 107

875 2091 124

875 2548 316

875 5463 370

875 5537 117

875 5952 127

876 159 152

876 197 102

876 234 285

876 441 126

876 554 500

876 585 213

876 599 469

876 732 190

876 735 217

876 852 98

876 863 144

876 1083 166

876 1504 519

876 1513 406

876 1807 99

876 1878 118

876 1888 100

876 3430 204

876 3436 100

876 4194 125

876 4807 99

876 4809 107

876 6195 99

876 9597 148

876 10742 362

876 10743 255

876 10744 209

876 10745 178

876 10746 171

876 10747 159

876 10748 150

876 10749 131

876 10750 128

876 10751 126

876 10752 125

876 10753 123

876 10754 122

876 10755 114

876 10756 107

876 10757 105

876 10758 101

876 10759 100

876 10760 99

876 10761 97

876 10762 95

876 10763 94

876 10764 91

876 10765 90

876 10766 90

876 10767 89

877 227 242

877 841 282

877 917 415

877 918 334

877 947 428

877 949 245

877 954 559

877 1257 448

877 1261	1102
877 1337	774
877 1344	406
877 1352	450
877 1353	327
877 1358	233
877 1360	410
877 1364	336
877 1369	268
877 1372	291
877 1522	234
877 1523	397
877 1524	318
877 1525	290
877 1527	243
877 1529	370
877 1530	353
877 1531	389
877 1536	314
877 1804	403
877 3476	263
877 3540	256
877 3780	296
877 4791	249
877 4803	293
877 5818	374
877 6264	308
877 7331	335
877 7357	274
877 7924	305

877 8599 292

877 9212 216

877 10768 573

877 10769 461

877 10770 411

877 10771 353

877 10772 338

877 10773 303

877 10774 290

877 10775 271

877 10776 267

877 10777 236

878 89 607

878 288 324

878 289 638

878 290 44

878 291 198

878 292 37

878 295 22

878 299 73

878 300 680

878 302 116

878 308 22

878 309 211

878 314 29

878 317 53

878 318 109

878 321 31

878 325 144

878 333 111

878 344 95

878 349 106

878 378 68

878 461 284

878 466 498

878 468 56

878 498 47

878 531 31

878 681 4741

878 686 252

878 701 52

878 793 185

878 797 49

878 907 34

878 1021 22

878 1032 59

878 1037 47

878 1054 23

878 1198 83

878 1451 59

878 1452 46

878 1453 38

878 2247 44

878 2990 26

878 4879 102

878 8378 90

878 10778 144

878 10779 86

878 10780 69

878 10781 63

878 10782 60

878 10783 32

881 65 223

881 154 198

881 190 90

881 210 79

881 220 153

881 230 242

881 246 305

881 333 103

881 412 158

881 424 112

881 439 2140

881 540 167

881 691 608

881 959 77

881 1045 1139

881 1048 100

881 1193 217

881 1246 486

881 1398 90

881 1761 315

881 2346 388

881 2425 103

881 2608 482

881 2661 1165

881 3200 152

881 4087 90

881 4164 105

881 4189 584

881 4191 158

881 4207 78

881 4375 184

881 4434 127

881 4823 232

881 4828 112

881 5644 1449

881 6120 59

881 6132 342

881 6602 3315

881 8710 392

881 9230 173

881 10797 3468

881 10798 1424

881 10799 1213

881 10800 733

881 10801 521

881 10802 235

881 10803 119

881 10804 106

881 10805 64

881 10806 60

882 89 3286

882 157 145

882 257 116

882 288 33278

882 289 80

882 292 74

882 295 101

882 299 113

882 300 3181

882 302 206

882 367 206

882 374 1444

882 386 220

882 397 957

882 400 626

882 409 637

882 461 115

882 466 498

882 475 259

882 526 118

882 544 214

882 701 918

882 707 121

882 930 614

882 951 162

882 1032 3050

882 1034 2738

882 1037 496

882 1114 2217

882 1249 2651

882 1453 128

882 1613 114

882 1659 113

882 1691 105

882 1904 343

882 1910 270

882 1932 363

882 2094 1768

882 2102 631

882 2502 226

882 3324 166

882 3708 98

882 6690 158

882 7160 108

882 8392 89

882 10807 628

882 10808 358

882 10809 343

882 10810 194

882 10811 118

884 89 454

884 161 120

884 257 441

884 288 453

884 289 165

884 292 224

884 295 5851

884 299 137

884 300 655

884 301 138

884 302 256

884 310 145

884 314 323

884 317 679

884 325 273

884 327 232

884 332 186

884 333 259

884 349 140

884 456 565

884 458 405

884 461 214

884 464 151

884 466 478

884 475 338

884 481 131

884 484 177

884 498 1623

884 528 245

884 542 128

884 543 122

884 646 167

884 679 245

884 701 514

884 802 293

884 913 565

884 915 183

884 1042 307

884 1045 367

884 1246 306

884 1428 232

884 1613 144

884 2092 700

884 3393 119

884 3571 436

884 4850 165

884 6121 147

884 7832 161

884 9169 222

884 10821 155

885 55 1103

885 67 2566

885 89 1084

885 157 196

885 257 439

885 285 325

885 288 1525

885 289 8210

885 292 875

885 293 912

885 295 597

885 298 191

885 299 589

885 300 434

885 306 622

885 309 181

885 310 330

885 312 589

885 314 349

885 317 395

885 318 956

885 325 479

885 328 198

885 333 596

885 349 2095

885 455 572

885 456 326

885 466 1450

885 523 1089

885 525 307

885 528 359

885 543 291

885 680 693

885 701 332

885 704 661

885 794 268

885 907 241

885 908 224

885 1451 349

885 1589 202

885 1684 569

885 2018 198

885 2392 181

885 2523 281

885 3075 614

885 3397 260

885 3489 181

885 9401 600

885 10822 806

885 10823 191

886 72 274

886 89 28

886 163 38

886 209 107

886 228 32

886 230 164

886 234 83

886 253 38

886 265 37

886 306 39

886 344 40

886 374 276

886 377 112

886 378 2212

886 436 66

886 461 38

886 486 59

886 498 99

886 504 40

886 517 32

886 518 174

886 533 24

886 542 30

886 641 151

886 701 60

886 704 28

886 714 100

886 726 25

886 955 178

886 966 26

886 1247 28

886 1438 76

886 1513 78

886 1760 28

886 1857 53

886 2014 56

886 2050 199

886 2080 275

886 2352 56

886 2440 28

886 3124 232

886 3302 29

886 3393 63

886 3750 31

886 6006 84

886 6292 46

886 10824 276

886 10825 67

886 10826 42

886 10827 37

887 152 770

887 163 160

887 170 127

887 186 162

887 188 512

887 190 142

887 370 80

887 436 74

887 511 90

887 615 110

887 824 203

887 944 334

887 959 104

887 961 561

887 1113 107

887 1837 143

887 1994 1693

887 2172 91

887 2594	115
887 2757	103
887 2962	863
887 2964	391
887 2969	325
887 3272	86
887 3417	419
887 3421	74
887 3470	88
887 3723	134
887 3788	176
887 3805	70
887 3901	254
887 3940	226
887 4383	72
887 4675	389
887 6570	150
887 7428	80
887 7903	99
887 8658	134
887 9716	155
887 9717	159
887 10196	132
887 10828	662
887 10829	161
887 10830	154
887 10831	136
887 10832	122
887 10833	103
887 10834	100

887 10835 80

887 10836 74

888 63 494

888 151 223

888 154 297

888 162 710

888 171 238

888 175 120

888 189 285

888 229 143

888 238 407

888 378 354

888 407 344

888 486 121

888 488 115

888 497 179

888 498 437

888 518 1402

888 722 2465

888 930 182

888 968 1051

888 1009 856

888 1020 169

888 1306 531

888 1378 322

888 1379 1149

888 1394 411

888 1470 285

888 1707 596

888 1800 1996

888 1854 2726

888 2075 202

888 2509 143

888 2702 154

888 4036 478

888 4177 832

888 4181 167

888 5605 957

888 5939 2453

888 6051 215

888 6056 254

888 6257 147

888 6760 297

888 9363 151

888 10837 706

888 10838 476

888 10839 304

888 10840 255

888 10841 228

888 10842 224

888 10843 179

888 10844 162

889 56 232

889 88 284

889 154 414

889 163 129

889 197 292

889 199 159

889 206 153

889 212 373

889 222 198

889 227 457

889 416 450

889 424 109

889 533 157

889 612 165

889 629 285

889 735 117

889 752 492

889 851 237

889 857 302

889 859 252

889 977 293

889 1216 188

889 1239 143

889 1240 105

889 1504 173

889 1631 107

889 1947 110

889 1950 224

889 1957 597

889 1958 403

889 1976 137

889 2415 113

889 2542 163

889 3217 187

889 3299 105

889 3364 313

889 4092 144

889 4110 388

889 5205 117

889 5605 576

889 6108 832

889 6729 175

889 8403 1444

889 10554 900

889 10845 643

889 10846 419

889 10847 212

889 10848 208

889 10849 201

889 10850 151

890 77 13

890 99 16

890 159 32

890 163 18

890 176 29

890 227 77

890 234 24

890 429 26

890 706 63

890 707 16

890 724 70

890 728 30

890 732 34

890 733 46

890 735 28

890 868 195

890 917 18

890 918 52

890 951 78

890 952 325

890 959 1344

890 1079 16

890 1122 19

890 1249 752

890 1360 41

890 1366 67

890 1369 46

890 1412 157

890 1803 298

890 1810 72

890 2343 32

890 2346 15

890 2347 197

890 2351 19

890 2463 16

890 2595 34

890 2624 26

890 2783 17

890 2784 231

890 2785 25

890 2787 52

890 3011 24

890 4031 82

890 4130 24

890 4460 38

890 7117 56

890 8794 22

890 10851 49

890 10852 21

890 10853 20

891 7 379

891 36 225

891 190 1056

891 198 468

891 233 4565

891 509 166

891 516 324

891 519 282

891 707 162

891 726 380

891 748 181

891 785 227

891 864 362

891 918 456

891 920 386

891 978 214

891 1104 802

891 1113 503

891 1203 142

891 1295 444

891 1410 748

891 1417 533

891 1701 142

891 1714 224

891 1870 168

891 1992 241

891 2011 3564

891 2362 501

891 2363 194

891 2381 514

891 2954 195

891 2961 189

891 2964 141

891 3183 270

891 3244 162

891 3246 722

891 4872 361

891 5672 158

891 6130 203

891 6257 1005

891 6390 352

891 8014 143

891 10854 322

891 10855 281

891 10856 223

891 10857 191

891 10858 182

891 10859 175

891 10860 151

891 10861 142

893 7 129

893 53 135

893 65 2146

893 66 870

893 70 360

893 72 17137

893 153 189

893 198 2129

893 207 323

893 306 404

893 328 158

893 489 1691

893 529 861

893 707 119

893 907 126

893 1001 182

893 1072 1162

893 1090 477

893 1104 4709

893 1398 370

893 1713 2420

893 3039 274

893 3462 101

893 5074 2720

893 5677 206

893 5679 258

893 6819 133

893 7261 258

893 7326 891

893 7759 3996

893 9635 402

893 9679 1869

893 9681 4267

893 10433 114

893 10871 7666

893 10872 2780

893 10873 1533

893 10874 1431

893 10875 1176

893 10876 1061

893 10877 948

893 10878 937

893 10879 477

893 10880 344

893 10881 251

893 10882 210

893 10883 202

893 10884 195

893 10885 124

893 10886 116

895 6 358

895 7 441

895 152 577

895 163 1105

895 234 407

895 489 238

895 517 260

895 562 225

895 707 1345

895 709 403

895 712 786

895 726 347

895 735 248

895 868 1061

895 875 326

895 1099 241

895 1104 272

895 1146 1028

895 1351	274
895 1358	403
895 1360	249
895 1369	787
895 1372	212
895 1411	355
895 1412	1255
895 1415	314
895 1639	381
895 1707	341
895 1710	441
895 1908	233
895 1974	192
895 1977	216
895 1981	1504
895 1984	583
895 1989	269
895 2475	238
895 2616	332
895 2746	206
895 2901	205
895 4675	213
895 4790	241
895 5297	534
895 5485	324
895 5488	207
895 5845	390
895 7414	195
895 9927	396
895 10897	390

895 10898 286

895 10899 204

896 53 616

896 57 590

896 65 1332

896 66 516

896 81 664

896 107 722

896 154 637

896 173 470

896 198 1703

896 227 445

896 228 632

896 230 665

896 234 453

896 238 1032

896 407 405

896 424 479

896 475 468

896 492 631

896 498 753

896 500 586

896 540 401

896 603 530

896 605 551

896 816 889

896 830 531

896 982 528

896 1098 399

896 1185 506

896 1195 521

896 1197 451

896 1672 728

896 1790 508

896 1858 1118

896 2605 627

896 2716 955

896 3057 579

896 3132 2019

896 3428 419

896 4230 450

896 4720 432

896 5137 433

896 7530 430

896 7838 395

896 10784 447

896 10900 4708

896 10901 2863

896 10902 671

896 10903 472

896 10904 434

896 10905 428

897 72 34

897 152 219

897 163 63

897 176 31

897 183 68

897 188 205

897 212 148

897 234 98

897 506 43

897 562 20

897 632 32

897 712 69

897 728 140

897 730 64

897 885 111

897 983 44

897 986 73

897 1113 108

897 1126 33

897 1134 42

897 1249 30

897 1274 50

897 1400 58

897 1412 30

897 1418 45

897 1747 20

897 1795 50

897 1824 21

897 1974 53

897 2006 144

897 3750 95

897 4613 61

897 4616 348

897 5299 100

897 5787 35

897 8216 123

897 10325 49

897 10390 152

897 10906 157

897 10907 153

897 10908 127

897 10909 79

897 10910 71

897 10911 64

897 10912 61

897 10913 48

897 10914 47

897 10915 39

897 10916 34

897 10917 21

898 55 3147

898 64 239

898 67 15307

898 77 786

898 89 625

898 94 202

898 155 205

898 157 940

898 180 276

898 227 300

898 257 208

898 258 208

898 261 613

898 285 452

898 288 438

898 289 1752

898 292 760

898 295 1138

898 299 1101

898 300 342

898 306 404

898 310 279

898 316 393

898 328 535

898 329 282

898 332 221

898 340 690

898 349 253

898 355 389

898 523 478

898 525 474

898 535 369

898 701 487

898 792 240

898 889 1821

898 906 199

898 985 228

898 1098 1074

898 1399 274

898 1681 216

898 1771 438

898 1820 903

898 2523 224

898 3281 208

898 4868 893

898 5416 351

898 5775 593

898 5973 257

898 6721 400

898 10918 216

899 324 500

899 403 692

899 464 260

899 471 1104

899 481 287

899 498 408

899 537 765

899 540 537

899 687 1526

899 691 320

899 693 243

899 816 253

899 1035 1000

899 1038 1166

899 1040 1939

899 1043 607

899 1182 614

899 1183 255

899 1186 319

899 1198 834

899 1201 584

899 1246 375

899 1674 305

899 1678 322

899 1866 321

899 2653 275

899 2665 324

899 3369 245

899 4262 728

899 4264 253

899 4385 285

899 5067 392

899 5253 251

899 5311 315

899 5949 245

899 5950 404

899 5958 250

899 6453 635

899 6597 255

899 6603 313

899 6608 253

899 6610 258

899 10318 1236

899 10496 253

899 10919 314

899 10920 301

899 10921 256

899 10922 255

899 10923 254

899 10924 249

900 65 171

900 182 767

900 289 176

900 367 178

900 374 209

900 375 649

900 379 304

900 400 160

900 406 490

900 963 185

900 1130 215

900 1770 253

900 1904 541

900 2088 725

900 2089 159

900 2092 331

900 2094 473

900 2097 221

900 2104 249

900 2428 319

900 2799 314

900 3858 379

900 4437 1248

900 5192 173

900 5565 178

900 5566 211

900 5574 188

900 6046 199

900 6857 344

900 8398 561

900 9013 324

900 10548 709

900 10925 1367

900 10926 797

900 10927 568

900 10928 499

900 10929 476

900 10930 461

900 10931 349

900 10932 309

900 10933 263

900 10934 220

900 10935 216

900 10936 203

900 10937 192

900 10938 187

900 10939 175

900 10940 171

900 10941 160

900 10942 158

903 190 1619

903 198 1219

903 497 612

903 503 146411

903 707 1636

903 806 2870

903 808 3234

903 810 3628

903 812 3388

903 813 3130

903 815 4549

903 820 1029

903 821 14253

903 824 5860

903 835 1915

903 917 1011

903 923 5786

903 928 756

903 930 4526

903 953 714

903 978 1292

903 1044 956

903 1104 1777

903 1182 578

903 1186 990

903 1206 665

903 1347 3592

903 1348 806

903 1854 633

903 2206 4426

903 2379 2416

903 2381 595

903 2661 624

903 2738 1418

903 2961 1300

903 2963 1356

903 2974 1831

903 3543 1093

903 4178 1683

903 4971 3691

903 5306 1388

903 5666 1309

903 6289 1760

903 10952 2687

903 10953 2162

903 10954 1876

903 10955 1603

903 10956 1416

903 10957 928
903 10958 536
904 7 45
904 65 2477
904 71 334
904 72 930
904 173 764
904 190 7420
904 220 105
904 228 93
904 295 105
904 377 103
904 378 93
904 486 136
904 498 200
904 510 96
904 511 1839
904 518 220
904 689 180
904 715 57
904 722 91
904 867 173
904 910 1575
904 968 61
904 1034 68
904 1055 85
904 1075 300
904 1375 87
904 1379 72
904 1380 53

904 1406 61

904 1455 58

904 1632 51

904 1787 969

904 1793 152

904 1873 94

904 1874 51

904 1892 38

904 1905 41

904 1934 83

904 3320 155

904 4257 42

904 4918 117

904 4923 387

904 4927 97

904 5001 43

904 5153 39

904 6177 79

904 10959 75

904 10960 51

904 10961 48

904 10962 40

905 55 163

905 65 369

905 67 225

905 89 859

905 97 265

905 230 100

905 288 522

905 289 585

905 291 115

905 292 381

905 295 596

905 298 715

905 300 598

905 302 129

905 306 242

905 310 159

905 320 225

905 321 112

905 327 115

905 329 519

905 331 761

905 342 136

905 349 109

905 412 131

905 461 181

905 464 321

905 466 534

905 475 151

905 484 100

905 486 110

905 498 134

905 525 132

905 545 197

905 546 399

905 679 308

905 701 183

905 903 544

905 907 154

905 1032 152

905 1037 235

905 1047 101

905 1455 116

905 1934 110

905 2544 137

905 2598 103

905 3057 169

905 3200 233

905 3378 111

905 4837 106

905 10571 131

906 67 973

906 89 2247

906 198 327

906 230 536

906 289 4826

906 295 320

906 298 444

906 300 569

906 302 410

906 325 948

906 328 364

906 333 539

906 349 720

906 378 459

906 461 387

906 464 1569

906 466 667

906 481 4747

906 498 543

906 524 12011

906 536 1206

906 538 687

906 540 411

906 542 943

906 546 355

906 779 4569

906 1035 499

906 1041 562

906 1045 1489

906 1047 360

906 1246 3039

906 1672 601

906 1674 471

906 1678 4750

906 1689 618

906 1863 1090

906 2080 348

906 2801 588

906 3239 626

906 5307 1252

906 5759 862

906 5934 2778

906 5950 321

906 6448 1356

906 6543 3499

906 10963 1030

906 10964 1019

906 10965 510

906 10966 477

906 10967 341

907 72 2064

907 163 1407

907 190 1569

907 212 1896

907 227 1641

907 533 534

907 554 135

907 562 794

907 582 132

907 591 118

907 599 226

907 630 1119

907 706 116

907 718 427

907 728 143

907 733 314

907 734 505

907 735 399

907 863 435

907 868 2388

907 1242 354

907 1244 646

907 1249 191

907 1412 294

907 1413 115

907 1415 302

907 1464 274

907 1513 361

907 1639 563

907 1806 931

907 1811 618

907 2265 402

907 2786 284

907 2855 184

907 2857 270

907 3458 111

907 5088 750

907 5257 145

907 5375 258

907 5844 801

907 7145 193

907 8130 160

907 8594 305

907 9543 179

907 10412 227

907 10968 312

907 10969 174

907 10970 155

907 10971 152

907 10972 137

908 9 137

908 36 114

908 50 30

908 233 73

908 370 43

908 487 60

908 518 98

908 707 34

908 815 34

908 930 205

908 955 133

908 1066 77

908 1104 31

908 1114 32

908 1263 37

908 1295 132

908 1298 44

908 1300 143

908 1914 56

908 2014 87

908 2963 107

908 2967 31

908 3183 37

908 3184 181

908 3190 44

908 3196 47

908 3197 33

908 3244 178

908 3425 48

908 3429 97

908 3558 63

908 3901 51

908 4162 38

908 5079 700

908 5459 193

908 5670 49

908 6129 61

908 6257 42

908 6542 31

908 6548 36

908 10973 90

908 10974 75

908 10975 49

908 10976 36

908 10977 35

908 10978 34

908 10979 32

908 10980 31

908 10981 30

908 10982 28

909 55 41

909 65 61

909 67 3541

909 89 371

909 173 4288

909 190 1040

909 198 2005

909 209 4158

909 229 1092

909 230 45

909 234 122

909 289 5452

909 292 53

909 293 226

909 295 81

909 306 33

909 324 129

909 329 76

909 377 541

909 378 267

909 405 162

909 412 216

909 486 124

909 488 11350

909 497 527

909 536 303

909 537 197

909 540 503

909 726 408

909 1044 56

909 1104 36479

909 1179 341

909 1186 61

909 1246 39369

909 1406 62

909 1714 414

909 1810 42

909 1860 169

909 1914 61

909 2179 114

909 2526 80

909 2664 44

909 2835 619

909 2961 41

909 3367 556

909 5609 1140

909 10983 1643

909 10984 436

909 10985 207

909 10986 80

910 55 33529

910 58 2798

910 89 2975

910 241 1109

910 251 996

910 257 2147

910 265 1515

910 279 1616

910 285 791

910 288 2014

910 289 2311

910 291 681

910 292 8667

910 294 1972

910 295 4917

910 296 4548

910 299 1783

910 305 703

910 308 2206

910 309 934

910 311 899

910 314 2226

910 322 898

910 323 1962

910 328 676

910 332 800

910 349 6756

910 352 820

910 535 1935

910 543 3814

910 548 868

910 697 1608

910 701 5571

910 906 701

910 915 2288

910 1934 1141

910 1936 866

910 1937 953

910 3500 1352

910 4563 1277

910 4566 1366

910 4572 1471

910 7781 1511

910 8605 943

910 8765 1746

910 10987 1905

910 10988 1128

910 10989 1001

910 10990 974

910 10991 953

911 67 443

911 89 2616

911 161 161

911 191 445

911 238 222

911 288 759

911 289 24251

911 290 290

911 292 1221

911 295 619

911 299 172

911 300 1440

911 306 658

911 308 157

911 310 169

911 318 184

911 324 173

911 327 173

911 328 2141

911 329 948

911 333 237

911 349 222

911 459 151

911 464 244

911 466 1015

911 476 192

911 484 144

911 485 190

911 523 284

911 528 258

911 545 235

911 644 209

911 661 208

911 679 145

911 907 190

911 968 217

911 1009 180

911 1379 166

911 1444 171

911 1449 413

911 1456 537

911 1486 218

911 2020 1239

911 2081 204

911 2521 399

911 4309 207

911 4685 151

911 5478 148

911 7293 216

911 10992 179

912 163 2770

912 212 1384

912 705 1584

912 706 740

912 707 979

912 710 1073

912 724 734

912 732 997

912 734 857

912 735 1412

912 843 652

912 917 1384

912 918 1105

912 919 1008

912 928 1059

912 932 1815

912 937 764

912 942 864

912 951 1288

912 952 1636

912 1249 2768

912 1339 925

912 1350 1615

912 1360 1620

912 1372 676

912 1412 1363

912 1513 1795

912 1545 936

912 1654 1039

912 1803 3006

912 1810 978

912 2038 1155

912 2343 2591

912 2346 643

912 2372 705

912 2784 1015

912 2797 838

912 2834 2660

912 3427 786

912 3660 696

912 4165 721

912 6282 848

912 7097 791

912 7107 1515

912 7108 945

912 10126 846

912 10993 6406

912 10994 939

912 10995 686

912 10996 659

913 45 413

913 65 1626

913 88 270

913 154 1316

913 171 387

913 172 376

913 173 1246

913 182 680

913 190 10282

913 198 616

913 203 327

913 207 838

913 210 937

913 217 529

913 220 368

913 221 302

913 225 261

913 229 1029

913 234 469

913 333 379

913 377 598

913 425 445

913 436 371

913 486 561

913 489 305

913 497 484

913 517 393

913 525 364

913 533 377

913 709 389

913 716 852

913 851 294

913 966 954

913 969 366

913 982 471

913 1090 1585

913 1118 337

913 1151 303

913 1244 271

913 1375 365

913 1406 395

913 1515 324

913 1786 798

913 1870 356

913 2614 280

913 2815 362

913 3945 291

913 4889 489

913 7177 477

913 8659 313

914 10 1332

914 11 762

914 17 577

914 19 1302

914 31 1018

914 41 562

914 56 1752

914 64 804

914 89 376

914 171 2073

914 188 1709

914 226 1417

914 370 1526

914 488 459

914 503 2279

914 504 584

914 516 688

914 683 657

914 707 391

914 728 364

914 812 1051

914 815 358

914 917 560

914 923 1517

914 932 963

914 978 5248

914 1270 4507

914 1277 515

914 1292 870

914 1299 730

914 1654 830

914 1914 1134

914 2370 554

914 2961 514

914 2963 1159

914 3464 509

914 3558 363

914 3940 849

914 4171 523

914 4178 537

914 4326 421

914 4693 1301

914 5180 533

914 6870 792

914 6873 646

914 6957 366

914 8348 777

914 10993 1942

914 10997 487

914 10998 388

915 207 352

915 220 329

915 221 372

915 234 220

915 240 768

915 250 314

915 261 297

915 366 1225

915 383 606

915 439 401

915 453 203

915 507 218

915 605 440

915 830 320

915 840 466

915 932 522

915 1090 222

915 1195 709

915 1241	248
915 1274	297
915 1401	189
915 1548	407
915 1613	329
915 1681	547
915 1979	269
915 2179	726
915 2226	648
915 2850	249
915 3721	212
915 3743	379
915 3746	403
915 4157	347
915 4504	225
915 4800	408
915 5066	265
915 5639	1058
915 5641	203
915 6193	332
915 6470	684
915 8713	280
915 10449	516
915 10806	593
915 10999	656
915 11000	393
915 11001	315
915 11002	270
915 11003	251
915 11004	217

915 11005 203

915 11006 191

916 67 275

916 89 732

916 257 772

916 279 410

916 288 1414

916 289 4861

916 292 843

916 293 511

916 295 1326

916 299 416

916 300 707

916 301 2951

916 311 329

916 321 1413

916 325 890

916 333 960

916 349 419

916 456 1954

916 457 2185

916 458 1608

916 461 1070

916 465 773

916 466 541

916 479 231

916 484 1458

916 544 283

916 679 620

916 681 1635

916 686 915

916 793 830

916 908 1643

916 913 251

916 1054 541

916 1470 248

916 1556 338

916 1659 490

916 3173 250

916 4294 352

916 7398 324

916 11007 1719

916 11008 892

916 11009 788

916 11010 785

916 11011 452

916 11012 405

916 11013 344

916 11014 316

916 11015 268

916 11016 262

916 11017 239

917 65 1696

917 154 1314

917 159 2466

917 172 1689

917 173 1670

917 198 2442

917 203 1349

917 234 1917

917 383 1387

917 424 3870

917 428 1615

917 429 3252

917 436 3534

917 437 1895

917 441 2822

917 453 1366

917 499 4705

917 512 1702

917 533 1880

917 709 1598

917 718 1437

917 808 1962

917 859 1803

917 863 1785

917 1048 1874

917 1109 1315

917 1424 2200

917 1504 4892

917 1510 2154

917 1513 1531

917 1767 2000

917 1817 1334

917 2531 2442

917 2938 1616

917 3129 1444

917 3279 1774

917 3333 5318

917 3461 1312

917 3475 1401

917 3735 1435

917 3739 3882

917 4092 2082

917 4346 2111

917 5153 1458

917 5414 1638

917 5638 1724

917 6297 1397

917 8350 1369

917 9332 1348

917 11018 1316

919 58 322

919 65 583

919 81 932

919 154 5774

919 184 253

919 190 5858

919 207 213

919 210 206

919 227 383

919 237 500

919 238 759

919 298 347

919 418 5623

919 420 962

919 425 238

919 430 693

919 432 607

919 454 253

919 535 344

919 546 298

919 632 269

919 690 412

919 691 200

919 703 1294

919 755 552

919 757 1624

919 779 421

919 1090 199

919 1098 307

919 1181 229

919 1390 369

919 1400 298

919 1504 1562

919 1632 1077

919 1745 441

919 1752 348

919 2121 485

919 3057 450

919 3259 441

919 3501 311

919 3767 237

919 3797 246

919 4074 252

919 5754 243

919 5926 209

919 6187 904

919 7079 312

919 8027 264

919 11030 954

919 11031 734

920 198 86

920 209 288

920 230 139

920 377 223

920 378 98

920 412 217

920 481 255

920 488 132

920 490 124

920 497 169

920 499 94

920 500 241

920 504 211

920 537 371

920 540 117

920 687 87

920 691 119

920 810 101

920 815 98

920 816 316

920 830 269

920 834 180

920 978 122

920 1180 355

920 1182 118

920 1185 242

920 1186 92

920 1194 225

920 1197	242
920 1246	215
920 1406	83
920 1650	88
920 1670	106
920 1672	483
920 1675	129
920 1863	318
920 1866	244
920 1905	148
920 2210	158
920 2214	97
920 2430	137
920 2850	115
920 3084	104
920 3239	154
920 4189	243
920 4262	97
920 4428	222
920 5065	189
920 6773	142
920 11032	85

921 198 953

921 208 963

921 234 14299

921 250 1289

921 278 1612

921 428 609

921 441 813

921 475 3646

921	868	630
921	1202	1695
921	1513	2075
921	1520	580
921	1640	642
921	1946	501
921	1952	699
921	2222	764
921	2227	1191
921	2247	556
921	3227	4491
921	3333	526
921	3354	3071
921	3355	4061
921	3357	557
921	3780	508
921	3792	1223
921	4585	1100
921	4586	635
921	4603	732
921	6618	505
921	6637	628
921	6901	2379
921	6997	742
921	9799	725
921	10742	771
921	11004	1170
921	11033	1527
921	11034	1394
921	11035	1028

921 11036 1024

921 11037 944

921 11038 888

921 11039 730

921 11040 709

921 11041 626

921 11042 580

921 11043 549

921 11044 543

921 11045 526

921 11046 517

921 11047 495

922 57 166

922 58 40

922 65 107

922 72 89

922 88 77

922 95 38

922 137 43

922 154 295

922 166 159

922 173 67

922 182 136

922 183 38

922 237 38

922 238 87

922 418 48

922 444 46

922 511 50

922 858 48

922 1089	48
922 1098	80
922 1375	48
922 1735	53
922 1787	61
922 1790	72
922 1800	101
922 1983	39
922 2605	57
922 2616	49
922 2935	40
922 3164	61
922 3280	213
922 3290	162
922 3676	53
922 3681	199
922 3884	128
922 4049	47
922 4230	72
922 4865	39
922 5542	55
922 6231	71
922 6756	65
922 7382	54
922 7644	38
922 10479	196
922 11048	130
922 11049	78
922 11050	61
922 11051	49

922 11052	45
922 11053	41
924 239	134
924 241	121
924 247	111
924 252	124
924 257	123
924 262	159
924 269	188
924 272	125
924 273	243
924 279	117
924 287	164
924 314	130
924 322	111
924 327	135
924 468	348
924 484	158
924 913	135
924 1507	130
924 1544	194
924 2220	133
924 3318	126
924 6375	136
924 6384	260
924 8081	190
924 8991	140
924 9904	121
924 9907	144
924 10608	207

924 11062 336

924 11063 298

924 11064 265

924 11065 222

924 11066 196

924 11067 195

924 11068 170

924 11069 169

924 11070 166

924 11071 163

924 11072 160

924 11073 153

924 11074 129

924 11075 127

924 11076 126

924 11077 123

924 11078 117

924 11079 114

924 11080 112

924 11081 111

924 11082 110

924 11083 108

925 53 883

925 67 855

925 72 2900

925 154 863

925 173 780

925 204 749

925 215 3011

925 227 1599

925 229 1325

925 233 1737

925 238 803

925 316 836

925 533 954

925 599 1571

925 610 1426

925 614 1626

925 679 847

925 683 948

925 697 725

925 701 717

925 787 825

925 868 1688

925 961 1151

925 965 1436

925 986 1282

925 1092 1387

925 1098 1783

925 1118 783

925 1131 714

925 1244 2240

925 1643 765

925 1985 763

925 2053 778

925 2531 2887

925 3060 684

925 3063 1143

925 3110 2234

925 4037 1700

925 5186 807

925 5217 796

925 5235 1510

925 5236 2445

925 5237 1452

925 5245 733

925 5247 4855

925 5249 1066

925 6225 743

925 9720 832

925 11084 1807

925 11085 670

926 9 194

926 19 212

926 45 239

926 777 153

926 1394 192

926 3289 291

926 3527 252

926 3536 290

926 6542 294

926 6659 163

926 6876 156

926 6878 152

926 6962 356

926 6981 254

926 6988 180

926 7521 183

926 9997 165

926 11086 1211

926 11087	478
926 11088	414
926 11089	405
926 11090	372
926 11091	348
926 11092	295
926 11093	279
926 11094	262
926 11095	255
926 11096	247
926 11097	245
926 11098	219
926 11099	214
926 11100	214
926 11101	205
926 11102	205
926 11103	196
926 11104	189
926 11105	189
926 11106	186
926 11107	184
926 11108	180
926 11109	176
926 11110	175
926 11111	171
926 11112	164
926 11113	161
926 11114	157
926 11115	157
926 11116	154

926 11117 153

926 11118 149

927 7 391

927 45 2045

927 88 432

927 206 396

927 209 654

927 220 358

927 226 369

927 233 1608

927 488 779

927 492 355

927 493 783

927 507 450

927 512 750

927 517 378

927 537 768

927 785 349

927 808 450

927 813 480

927 815 679

927 841 385

927 845 660

927 854 530

927 923 403

927 1091 998

927 1097 643

927 1179 628

927 1195 443

927 1216 437

927 1239 417

927 1672 505

927 1706 388

927 1858 718

927 2211 366

927 2362 665

927 2744 387

927 2851 798

927 3769 490

927 4099 746

927 4346 368

927 6130 472

927 6382 445

927 6461 391

927 6547 551

927 7419 506

927 8350 626

927 8464 751

927 11119 498

927 11120 494

927 11121 443

927 11122 425

928 53 24

928 81 30

928 444 27

928 603 94

928 616 92

928 744 668

928 748 109

928 756 61

928 772 555

928 778 35

928 986 26

928 1156	23
928 1222	89
928 1716	136
928 1745	50
928 1772	49
928 1781	35
928 1835	27
928 1965	53
928 1969	56
928 2146	71
928 2161	42
928 2174	49
928 2667	63
928 2668	78
928 2669	80
928 2670	111
928 2671	60
928 2672	91
928 2673	89
928 2675	40
928 2676	113
928 2677	48
928 2681	46
928 2688	23
928 2691	63
928 3617	31
928 3681	32

928 4865 24

928 6582 30

928 10517 36

928 11123 95

928 11124 35

928 11125 32

928 11126 31

928 11127 27

928 11128 26

928 11129 25

928 11130 25

928 11131 24

929 65 173

929 157 73

929 184 79

929 198 192

929 234 62

929 328 213

929 366 250

929 447 190

929 518 535

929 602 115

929 707 59

929 726 165

929 923 85

929 930 163

929 983 495

929 1009 77

929 1238 307

929 1249 60

929 1444	58
929 2179	114
929 3462	129
929 6506	80
929 6678	191
929 7203	102
929 7301	87
929 8278	61
929 9892	56
929 10910	103
929 11132	892
929 11133	183
929 11134	178
929 11135	144
929 11136	111
929 11137	101
929 11138	88
929 11139	83
929 11140	82
929 11141	79
929 11142	76
929 11143	72
929 11144	69
929 11145	66
929 11146	65
929 11147	65
929 11148	64
929 11149	64
929 11150	63
929 11151	60

929 11152 60

929 11153 57

930 89 3653

930 159 515

930 257 885

930 288 495

930 289 1076

930 295 1335

930 300 2524

930 307 2139

930 333 3462

930 344 3300

930 461 1612

930 466 924

930 498 56134

930 523 1576

930 615 492

930 646 485

930 679 5463

930 680 882

930 681 849

930 683 944

930 686 696

930 691 523

930 693 885

930 701 1883

930 704 19718

930 714 781

930 802 456

930 1045 8020

930 1377	1718

930 1467	1732

930 1470	1282

930 1934	1137

930 2020	6091

930 2548	664

930 2839	530

930 2840	1691

930 3928	519

930 4514	924

930 4850	492

930 5749	3458

930 6121	527

930 6132	1185

930 6196	606

930 9079	1635

930 9249	576

930 11154	2109

930 11155	1405

930 11156	758

930 11157	739

930 11158	516

931 238 102

931 441 88

931 706 957

931 707 367

931 839 78

931 841 531

931 843 104

931 845 248

931 846 180

931 917 101

931 918 524

931 1206	174
931 1216	120
931 1249	294
931 1257	180
931 1261	77
931 1274	72
931 1281	91
931 1286	86
931 1337	83
931 1358	153
931 1360	425
931 1411	170
931 1412	169
931 1526	278
931 1527	123
931 1531	90
931 1533	169
931 1549	84
931 1555	74
931 1976	200
931 2624	229
931 2823	187
931 4271	79
931 4946	105
931 5040	72
931 5126	87
931 6048	94

931 6983 224

931 7047 202

931 7472 112

931 9212 90

931 9260 87

931 11159 202

931 11160 180

931 11161 128

931 11162 105

931 11163 85

931 11164 77

931 11165 76

932 63 41805

932 77 219

932 89 338

932 151 321

932 176 763

932 187 15117

932 221 317

932 227 319

932 333 225

932 405 310

932 478 231

932 479 1161

932 707 500

932 877 516

932 959 8695

932 972 1344

932 1001 417

932 1014 390

932 1021	375
932 1081	798
932 1243	285
932 1491	1960
932 1795	1170
932 1913	1023
932 2031	260
932 2075	301
932 2444	260
932 2558	216
932 3501	366
932 3832	1655
932 3914	1199
932 3955	212
932 3966	1659
932 3978	351
932 5923	214
932 6064	556
932 6246	216
932 7130	805
932 7708	2305
932 11166	1275
932 11167	1051
932 11168	735
932 11169	468
932 11170	417
932 11171	414
932 11172	402
932 11173	259
932 11174	249

932 11175 247

932 11176 232

933 154 1252

933 182 510

933 195 530

933 199 785

933 207 791

933 225 1519

933 226 310

933 227 462

933 228 880

933 229 773

933 416 354

933 424 1076

933 425 717

933 432 306

933 439 585

933 533 370

933 614 889

933 618 371

933 715 506

933 716 518

933 932 297

933 962 340

933 969 549

933 982 516

933 1090 361

933 1151 355

933 1246 332

933 1376 301

933 1400	425
933 1545	308
933 1632	767
933 1633	413
933 1768	362
933 1949	318
933 1976	402
933 2611	297
933 2815	699
933 4384	329
933 4448	432
933 4452	1129
933 4459	837
933 5630	833
933 10590	311
933 10617	779
933 11177	4023
933 11178	557
933 11179	385
933 11180	373
933 11181	366
933 11182	330

934 154 266

934 163 135

934 190 434

934 206 489

934 207 129

934 226 313

934 227 388

934 230 95

934 233 243

934 234 95

934 434 112

934 554 111

934 562 506

934 599 130

934 614 159

934 630 94

934 707 223

934 709 97

934 846 116

934 868 132

934 917 91

934 959 106

934 964 157

934 1110 141

934 1131 429

934 1216 446

934 1239 182

934 1369 118

934 1901 296

934 1976 101

934 2362 140

934 2381 102

934 2415 367

934 2424 274

934 2823 203

934 2855 147

934 3258 311

934 4079 109

934 4354 113
934 4356 205
934 6824 107
934 6836 302
934 7143 195
934 7147 166
934 7625 107
934 8012 255
934 9715 121
934 11183 174
934 11184 102
934 11185 95
935 2 428
935 9 108
935 13 122
935 30 364
935 72 185
935 153 353
935 487 108
935 510 193
935 515 392
935 878 93
935 1256 145
935 1277 107
935 1292 296
935 1293 113
935 1295 80
935 1300 176
935 1303 536
935 1312 70

935 1314	91
935 1327	79
935 1331	138
935 1401	148
935 1793	219
935 2166	91
935 2935	181
935 3188	177
935 4056	89
935 4317	255
935 4318	222
935 4871	205
935 4874	147
935 4876	152
935 5670	76
935 6750	95
935 7521	109
935 9499	100
935 11186	284
935 11187	188
935 11188	140
935 11189	99
935 11190	96
935 11191	91
935 11192	91
935 11193	87
935 11194	86
935 11195	80
935 11196	80
935 11197	69

935 11198 69

935 11199 69

936 603 1327

936 616 931

936 744 1463

936 748 1735

936 756 545

936 772 453

936 778 480

936 1222 625

936 1745 586

936 1943 972

936 1945 597

936 1965 405

936 1969 791

936 2146 745

936 2149 782

936 2161 392

936 2174 404

936 2605 373

936 2667 1236

936 2668 1146

936 2669 850

936 2670 702

936 2671 1114

936 2672 901

936 2673 2211

936 2674 365

936 2675 442

936 2677 603

936 2681 1454

936 2688 405

936 2691 385

936 4865 315

936 7470 315

936 7481 302

936 7493 434

936 9766 516

936 10518 722

936 11128 393

936 11200 1422

936 11201 1264

936 11202 682

936 11203 459

936 11204 450

936 11205 406

936 11206 404

936 11207 368

936 11208 350

936 11209 323

936 11210 296

936 11211 292

938 71 1746

938 89 595

938 166 1549

938 229 836

938 289 1300

938 291 3263

938 295 712

938 297 1588

938 299 488

938 300 1234

938 301 572

938 302 934

938 310 4119

938 316 503

938 317 498

938 344 1153

938 377 494

938 378 129595

938 466 5819

938 490 527

938 498 6425

938 499 1984

938 518 2402

938 680 12210

938 907 777

938 913 648

938 930 2253

938 972 956

938 1037 718

938 1377 1150

938 1455 605

938 2014 1239

938 2607 2832

938 3124 3518

938 3859 696

938 4761 704

938 4817 510

938 5459 769

938 6250 560

938 7191 599

938 9187 9358

938 9193 555

938 11217 3150

938 11218 1108

938 11219 1088

938 11220 711

938 11221 583

938 11222 576

938 11223 555

938 11224 490

939 157 341

939 209 1055

939 257 803

939 317 366

939 344 346

939 486 1646

939 492 1127

939 493 863

939 498 1184

939 500 1005

939 507 517

939 528 474

939 693 766

939 779 720

939 810 873

939 811 1439

939 813 1047

939 816 827

939 823 359

939 832 478

939 845 510

939 953 392

939 955 590

939 1044 495

939 1091 827

939 1116 717

939 1145 456

939 1180 450

939 1188 412

939 1190 938

939 1200 510

939 1672 10595

939 1866 497

939 1868 1603

939 2062 638

939 2352 515

939 2619 540

939 2940 1044

939 3093 472

939 3545 1125

939 3621 537

939 5100 923

939 5666 433

939 6249 630

939 9811 449

939 10038 738

939 11225 949

939 11226 595

939 11227 395

939 11228 370

940 59 275

940 97 777

940 154 328

940 190 295

940 203 242

940 212 385

940 221 282

940 227 576

940 228 1190

940 427 535

940 428 444

940 429 847

940 533 843

940 562 269

940 620 271

940 630 360

940 735 303

940 851 1076

940 1089 1114

940 1384 660

940 1416 327

940 1510 317

940 1511 300

940 1519 547

940 1934 259

940 2131 251

940 2537 389

940 2614 549

940 3293 281

940 3945 452

940 4064 524

940 4079 279

940 4442 283

940 4742 606

940 6202 279

940 7525 1117

940 8043 822

940 8656 306

940 8840 413

940 9632 252

940 9633 881

940 10080 567

940 10407 1244

940 11229 1410

940 11230 1289

940 11231 1025

940 11232 742

940 11233 388

940 11234 366

940 11235 276

941 65 106

941 89 459

941 166 2887

941 191 6943

941 208 4359

941 209 176

941 223 15200

941 228 1086

941 230 134

941 293 229

941 313 226

941 318 443

941 333 3153

941 425 105

941 544 12815

941 680 95

941 691 85

941 693 3311

941 696 4229

941 704 20118

941 757 3187

941 794 259

941 1097 738

941 1186 96

941 1383 106

941 1400 4360

941 1464 143

941 2083 1780

941 2407 351

941 2533 3381

941 2582 291

941 2982 3263

941 3399 3846

941 3769 120

941 4575 427

941 5412 103

941 5547 3001

941 7945 3911

941 8104 190

941 8674 532

941 9082 3347

941 11236 11720

941 11237 3584

941 11238 1263

941 11239 1096

941 11240 1041

941 11241 510

941 11242 363

941 11243 316

941 11244 288

942 55 1

942 89 3

942 229 2

942 288 1

942 291 1

942 295 1

942 306 1

942 314 1

942 323 2

942 360 1

942 525 1

942 528 1

942 532 1

942 543 1

942 1048 2

942 1451 1

942 1489 1

942 2018 1

942 3397 1

942 4467 1

942 4617 1

942 5767 1

942 7324 1

942 8161 1

942 10608 1

942 11069 1

942 11245 3

942 11246 3

942 11247 2

942 11248 1

942 11249 1

942 11250 1

942 11251 1

942 11252 1

943 56 41

943 88 117

943 163 87

943 166 34

943 207 35

943 212 45

943 217 51

943 220 142

943 223 91

943 227 327

943 229 25

943 238 26

943 300 33

943 412 102

943 424 52

943 501 52

943 527 29

943 533 33

943 537 105

943 562 32

943 599 37

943 709 86

943 733 28

943 735 48

943 859 23

943 959 30

943 1106 28

943 1237 24

943 1383 48

943 1400 109

943 1412 55

943 1424 26

943 1873 26

943 1958 94

943 1977 28

943 2346 79

943 2637 169

943 3341 106

943 3673 47

943 3823 23

943 4118 46

943 4262 47

943 4369 73

943 5547 85

943 7324 29
943 10564 32
943 11253 139
943 11254 35
943 11255 33
943 11256 25
946 230 1427
946 324 375
946 377 1178
946 412 297
946 470 196
946 471 4061
946 481 761
946 492 176
946 498 213
946 537 500
946 687 144559
946 816 1020
946 1038 198
946 1040 1745
946 1041 370
946 1043 1634
946 1045 7752
946 1047 138
946 1180 156
946 1182 774
946 1183 1635
946 1185 1048
946 1186 1187
946 1188 195

946 1193 170

946 1198 615

946 1201 168

946 1247 141

946 1541 216

946 1678 817

946 2653 1602

946 2987 314

946 4385 139

946 4822 132

946 5749 336

946 5950 1013

946 5958 141

946 6120 267

946 6132 1347

946 6601 238

946 6793 200

946 6794 263

946 7832 832

946 7834 126

946 8192 2918

946 10318 131

946 11273 157

946 11274 128

946 11275 123

946 11276 122

948 152 84

948 154 126

948 170 81

948 190 98

948 199 107

948 416 240

948 448 88

948 629 87

948 969 92

948 1164 134

948 1216 150

948 1413 129

948 1547 190

948 2524 214

948 2775 107

948 3028 114

948 3467 117

948 3689 216

948 3693 93

948 3805 98

948 3870 104

948 4199 121

948 4222 243

948 4820 93

948 5205 372

948 5423 98

948 5636 151

948 5638 93

948 5879 281

948 5925 93

948 6205 303

948 6295 130

948 6424 215

948 6570 309

948 6579 96
948 7639 113
948 8043 83
948 8659 91
948 9260 107
948 10515 325
948 10610 122
948 11282 209
948 11283 205
948 11284 169
948 11285 158
948 11286 132
948 11287 122
948 11288 106
948 11289 106
948 11290 85
949 687 383
949 779 2553
949 816 354
949 1035 2120
949 1100 607
949 1180 331
949 1185 490
949 1187 660
949 1188 1629
949 1541 582
949 1678 371
949 1853 510
949 1868 856
949 2192 475

949 2203	1623
949 2204	600
949 2210	880
949 2214	507
949 2426	945
949 2429	1880
949 2430	343
949 2617	1277
949 2618	406
949 2621	853
949 2850	357
949 4166	5719
949 4168	1958
949 4774	342
949 4938	442
949 5067	349
949 5130	561
949 6012	476
949 6016	416
949 6019	553
949 6448	841
949 7831	783
949 8384	319
949 8684	1033
949 9516	788
949 9934	310
949 11291	1015
949 11292	905
949 11293	835
949 11294	689

949 11295 575

949 11296 476

949 11297 459

949 11298 413

949 11299 332

949 11300 314

950 15 128

950 25 411

950 72 161

950 159 163

950 163 137

950 176 307

950 227 1091

950 599 157

950 607 92

950 732 836

950 733 94

950 841 176

950 917 3513

950 918 317

950 919 108

950 922 335

950 1069 205

950 1079 95

950 1259 114

950 1366 238

950 1414 205

950 1416 213

950 1803 148

950 1808 112

950 1809 144

950 1810 1772

950 2341 112

950 2343 864

950 2787 406

950 2838 336

950 2973 424

950 3471 325

950 4165 146

950 4180 362

950 4271 156

950 4458 132

950 4460 161

950 4680 167

950 5008 239

950 5155 632

950 5157 107

950 5161 129

950 5164 368

950 7100 184

950 8386 118

950 9465 120

950 10689 112

950 10776 99

950 11301 143

950 11302 121

951 19 814

951 45 447

951 107 2047

951 153 957

951 170 715

951 173 612

951 407 707

951 487 581

951 512 504

951 515 987

951 1148	903
951 1292	615
951 1295	540
951 1296	558
951 1297	1820
951 1301	631
951 1303	1528
951 1312	529
951 1314	621
951 1700	457
951 1716	881
951 1983	425
951 2112	471
951 2667	568
951 5929	509
951 5966	745
951 6016	1217
951 6582	403
951 10430	1103
951 10719	937
951 11303	3183
951 11304	2445
951 11305	1744
951 11306	1281

951 11307 976

951 11308 663

951 11309 639

951 11310 635

951 11311 588

951 11312 539

951 11313 533

951 11314 522

951 11315 509

951 11316 476

951 11317 450

951 11318 448

951 11319 444

951 11320 422

951 11321 409

951 11322 401

952 67 198

952 89 103

952 157 127

952 187 101

952 192 142

952 207 420

952 208 62

952 227 2962

952 241 62

952 289 104

952 295 156

952 298 240

952 417 71

952 428 62

952 429 67

952 439 873

952 466 59

952 470 268

952 525 56

952 533 191

952 538 63

952 618 136

952 733 307

952 898 54

952 903 625

952 1048 1392

952 1106 216

952 1249 62

952 1515 66

952 1755 122

952 1771 133

952 1775 54

952 2031 303

952 2121 87

952 2988 54

952 3741 61

952 3763 55

952 3795 74

952 5099 65

952 5416 207

952 6722 108

952 7348 56

952 9197 67

952 10969 75

952 11323 650

952 11324 78

952 11325 62

952 11326 62

952 11327 58

952 11328 55

953 89 428

953 196 142

953 207 74

953 227 717

953 228 75

953 229 100

953 230 169

953 288 46

953 289 89

953 295 65

953 298 74

953 300 55

953 306 110

953 324 64

953 329 57

953 366 66

953 403 103

953 421 61

953 425 103

953 439 42

953 464 293

953 466 51

953 470 133

953 498 162

953 526 43

953 533 79

953 545 42

953 546 331

953 629 112

953 679 236

953 691 64

953 1043 170

953 1047 149

953 1090 237

953 1155 124

953 1243 55

953 1390 657

953 1398 45

953 1400 92

953 1502 157

953 1513 48

953 1517 114

953 2121 267

953 2533 46

953 3200 468

953 3373 137

953 3712 37

953 4941 457

953 5934 84

953 11329 39

954 30 167

954 32 85

954 55 355

954 59 244

954 67 148

954 72 2096

954 75 117

954 76 51

954 89 185

954 153 1238

954 157 80

954 187 279

954 535 375

954 880 133

954 1001 1385

954 1014 186

954 1061 402

954 1062 273

954 1067 794

954 1072 134

954 1073 193

954 1074 367

954 1080 136

954 1099 66

954 1297 106

954 1298 80

954 1304 95

954 1379 165

954 1713 147

954 2935 789

954 3183 235

954 3191 156

954 3289 243

954 3501 105

954 3768 75

954 3988 136

954 4245 102

954 4296 688

954 4308 314

954 4310 998

954 4317 116

954 4704 107

954 8149 149

954 10625 75

954 11330 283

954 11331 215

954 11332 90

954 11333 83

954 11334 64

954 11335 54

955 55 2352

955 67 1404

955 72 480

955 157 433

955 167 354

955 172 364

955 173 708

955 226 452

955 230 2036

955 285 2380

955 369 315

955 388 230

955 468 473

955 511 666

955 536 233

955 715 378

955 716 340

955 789 598

955 969 262

955 1090 936

955 1104 345

955 1406 356

955 1427 246

955 1825 1505

955 1826 277

955 2783 322

955 3313 330

955 3503 833

955 4348 5700

955 4932 555

955 5568 502

955 8992 260

955 8998 404

955 10130 340

955 11336 1424

955 11337 1093

955 11338 757

955 11339 566

955 11340 485

955 11341 436

955 11342 433

955 11343 387

955 11344 372

955 11345 368

955 11346 270

955 11347 267

955 11348 250

955 11349 245

955 11350 242

955 11351 233

957 267 857

957 276 671

957 278 1698

957 475 3323

957 1037 535

957 1444 540

957 1613 355

957 1641 2145

957 1975 429

957 2179 64223

957 2226 1686

957 2227 993

957 2236 3918

957 2241 1284

957 2247 2611

957 2251 513

957 2252 8399

957 2916 9400

957 2924 16648

957 2930 342

957 3357 6354

957 3667 500

957 5039 580

957 5051 374

957 5988 1193

957 6618 294

957 6619 341

957 6620 973

957 6621 647

957 6622 9343

957 6626 374

957 6629 502

957 6631 334

957 6636 547

957 6905 339

957 8164 624

957 8600 364

957 11379 8215

957 11380 2650

957 11381 1165

957 11382 817

957 11383 747

957 11384 602

957 11385 487

957 11386 473

957 11387 386

957 11388 314

957 11389 303

957 11390 286

957 11391 267

958 239 46

958 288 81

958 291 152

958 295 184

958 301 114

958 302 94

958 307 54

958 317 60

958 333 57

958 344 602

958 364 84

958 475 72

958 497 53

958 498 63

958 680 49

958 693 125

958 816 68

958 1187 84

958 1193 87

958 1197 230

958 1447 47

958 1905 48

958 2439 64

958 2617 99

958 2839 160

958 2840 45

958 2847 263

958 2990 47

958 3033 46

958 3318 85

958 3584 66

958 4223 192

958 4259 252

958 4264 75

958 5267 56

958 5649 56

958 6298 45

958 7831 76

958 7947 208

958 8242 66

958 8737 97

958 8738 96

958 9799 215

958 10613 64

958 11291 45

958 11392 190

958 11393 74

958 11394 72

958 11395 60

958 11396 45

959 51 78

959 58 84

959 59 222

959 61 166

959 65 122

959 68 166

959 70 194

959 72 1782

959 84 86

959 89 329

959 98 328

959 99 156

959 154 81

959 155 125

959 159 421

959 187 91

959 221 70

959 429 170

959 511 83

959 599 589

959 644 114

959 858 165

959 878 383

959 959 505

959 972 501

959 993 169

959 1001 164

959 1013 277

959 1109 271

959 1384 516

959 1492 119

959 1708 90

959 1764 120

959 1886 306

959 1892 167

959 1895 132

959 1897 181

959 2059 71

959 2554 110

959 2783 87

959 3104 71

959 4423 77

959 4448 121

959 5480 72

959 7096 76

959 9632 106

959 11397 191

959 11398 124

959 11399 92

959 11400 75

960 56 2205

960 65 114

960 72 47

960 89 8

960 107 8

960 198 141

960 238 126

960 249 33

960 317 9

960 328 55

960 331 1187

960 377 453

960 407 410

960 475 430

960 526 5

960 547 589

960 617 25

960 661 17

960 903 80

960 914 7

960 968 40

960 1099 127

960 1130 7

960 1576 9

960 1613 5

960 1627 6

960 1795 5

960 1942 6

960 2180 13

960 2923 186

960 3106 7

960 3317 171

960 4309 8

960 5537 7

960 5680 5

960 6226 6

960 8161 10

960 9986 5

960 11365 142

960 11401 137

960 11402 20

960 11403 15

960 11404 11

960 11405 9

960 11406 9

960 11407 8

960 11408 6

960 11409 5

960 11410 5

960 11411 5

961 96 144

961 154 118

961 195 130

961 211 96

961 217 92

961 227 156

961 331 189

961 344 98

961 419 123

961 426 445

961 615 144

961 898 1768

961 1046 99

961 1164 160

961 1239 261

961 1632 214

961 1684 195

961 1947 203

961 2840 87

961 2923 237

961 3057 221

961 3078 131

961 3108 295

961 3206 86

961 3616 841

961 3619 340

961 4118 86

961 4203 97

961 5233 177

961 5652 111

961 5926 116

961 6422 91

961 7272 379

961 8109 342

961 9882 450

961 10214 103

961 10618 211

961 10955 88

961 11412 528

961 11413 245

961 11414 242

961 11415 202

961 11416 148

961 11417 122

961 11418 115

961 11419 114

961 11420 112

961 11421 108

961 11422 99

961 11423 97

962 163 335

962 212 331

962 227 257

962 563 640

962 607 288

962 609 250

962 613 228

962 625 172

962 1109 180

962 1110 572

962 1411 363

962 1412 169

962 1413 535

962 1422 182

962 2459	200
962 2624	266
962 2898	322
962 3481	180
962 4790	385
962 5297	1155
962 5302	162
962 5303	186
962 5416	809
962 5447	185
962 6176	335
962 7797	342
962 7799	195
962 9455	291
962 10209	227
962 10413	189
962 10969	256
962 11424	887
962 11425	484
962 11426	363
962 11427	299
962 11428	297
962 11429	290
962 11430	289
962 11431	273
962 11432	235
962 11433	211
962 11434	211
962 11435	208
962 11436	206

962 11437 198

962 11438 197

962 11439 194

962 11440 188

962 11441 172

962 11442 169

963 55 250

963 67 21425

963 89 167

963 157 75

963 187 120

963 283 31

963 289 51

963 306 36

963 340 72

963 679 22

963 725 31

963 794 265

963 877 36

963 903 38

963 985 808

963 1001 21

963 1014 209

963 1015 20

963 1062 55

963 1243 25

963 1464 18522

963 1465 555

963 1471 81

963 1480 58

963 1755 180

963 1892 94

963 1941 58

963 3281 109

963 5458 39

963 5775 160

963 6232 256

963 6703 536

963 6869 23

963 7452 22

963 8067 24

963 9745 36

963 10358 66

963 11171 52

963 11443 295

963 11444 162

963 11445 149

963 11446 96

963 11447 75

963 11448 38

963 11449 35

963 11450 31

963 11451 25

963 11452 22

963 11453 21

963 11454 20

964 89 64

964 155 65

964 159 64

964 163 95

964 190 85

964 196 56

964 207 520

964 209 151

964 227 465

964 228 72

964 229 135

964 230 180

964 265 74

964 298 132

964 300 120

964 344 68

964 412 260

964 424 132

964 461 57

964 464 93

964 466 64

964 470 430

964 486 82

964 498 545

964 533 905

964 537 115

964 540 113

964 683 81

964 687 94

964 714 205

964 816 286

964 1038 141

964 1043 92

964 1047 1082

964 1244 52

964 1246 289

964 1249 131

964 1512 132

964 1866 88

964 2047 63

964 2840 65

964 3067 70

964 3373 198

964 3400 55

964 3462 78

964 4262 52

964 4468 55

964 6196 97

964 6453 56

964 11455 98

965 55 970

965 67 2389

965 89 696

965 97 133

965 157 329

965 161 119

965 257 1031

965 285 1349

965 288 1401

965 289 8806

965 291 452

965 292 5047

965 293 132

965 294 150

965 295 302

965 298 574

965 299 1352

965 300 1287

965 302 777

965 306 281

965 314 415

965 318 478

965 320 123

965 325 691

965 329 167

965 333 1562

965 340 213

965 344 608

965 349 173

965 352 189

965 360 257

965 455 200

965 458 186

965 461 346

965 465 145

965 466 1267

965 523 712

965 525 545

965 528 128

965 544 224

965 679 2128

965 686 272

965 701 1116

965 889 1679

965 1451 140

965 1580 158

965 2020 218

965 2094 158

965 2523 192

965 2801 140

966 55 208

966 65 182

966 67 283

966 89 767

966 154 233

966 191 308

966 228 199

966 288 3422

966 289 1663

966 292 1290

966 295 992

966 300 401

966 301 341

966 302 340

966 306 1425

966 310 488

966 314 1490

966 317 314

966 320 557

966 327 484

966 328 2261

966 329 4423

966 330 214

966 331 624

966 333 1129

966 349 1026

966 424 201

966 458 186

966 464 235

966 466 2448

966 485 252

966 498 363

966 523 546

966 526 349

966 543 709

966 679 291

966 701 433

966 907 616

966 1378 293

966 1449 369

966 1451 390

966 1632 239

966 2020 1270

966 2025 525

966 2521 773

966 3404 267

966 3756 359

966 4432 245

966 8155 290

966 8721 182

967 65 103

967 182 119

967 221 78

967 225 110

967 422 251

967 439 173

967 492 316

967 493 62

967 498 106

967 501 158

967 507 637

967 510 285

967 533 52

967 618 901

967 706 509

967 808 77

967 816 370

967 840 97

967 1091 219

967 1180 321

967 1182 65

967 1186 76

967 1197 753

967 1513 178

967 1766 325

967 1817 131

967 1966 414

967 3097 93

967 3462 64

967 3740 323

967 3743 350

967 3745 1326

967 3746 103

967 3791 151

967 4189 137

967 4201 130

967 4828 175

967 5313 257

967 6120 82

967 10427 155

967 10693 217

967 11177 264

967 11182 72

967 11456 341

967 11457 334

967 11458 264

967 11459 186

967 11460 149

967 11461 131

967 11462 57

968 53 464

968 64 204

968 84 222

968 208 617

968 226 331

968 234 214

968 424 392

968 428 210

968 432 404

968 440 200

968 444 565

968 604 293

968 629 311

968 703 262

968 746 311

968 757 334

968 863 229

968 1090 191

968 1101 218

968 1109 451

968 1159 287

968 1206 181

968 1239 375

968 1242 451

968 1400 380

968 1504 636

968 1766 256

968 1964 1406

968 1986 443

968 2139 1471

968 2268 407

968 2402 399

968 2480 264

968 2530 281

968 2533 562

968 2542 365

968 6032 181

968 6155 190

968 6210 457

968 6217 805

968 7692 498

968 8026 470

968 8703 851

968 8705 213

968 11463 370
968 11464 313
968 11465 301
968 11466 246
968 11467 242
968 11468 194
969 67 160
969 71 770
969 163 135
969 166 1907
969 178 605
969 190 173
969 207 290
969 227 457
969 229 460
969 230 292
969 256 568
969 289 322
969 292 213
969 298 354
969 300 342
969 306 137
969 316 2364
969 333 1001
969 344 324
969 378 314
969 498 202
969 523 209
969 536 256
969 542 250

969 689 518

969 701 586

969 703 247

969 714 374

969 930 3258

969 972 554

969 1104 375

969 1114 811

969 1400 269

969 1406 233

969 1464 164

969 1469 400

969 1545 126

969 1590 140

969 1710 524

969 1775 159

969 2277 231

969 2446 132

969 3070 142

969 3200 326

969 3763 184

969 4155 144

969 4254 206

969 7100 347

969 10604 371

969 11469 348

971 65 767

971 159 42

971 198 93

971 209 155

971 220 49

971 229 104

971 230 96

971 306 124

971 333 175

971 344 57

971 377 412

971 412 1122

971 464 54

971 486 2309

971 498 120

971 540 170

971 687 141

971 705 233

971 708 230

971 966 99

971 1043 94

971 1044 205

971 1046 685

971 1047 989

971 1246 65

971 1375 60

971 1415 40

971 1470 245

971 1673 55

971 1681 528

971 2080 44

971 2352 54

971 2625 40

971 3378 282

971 3379 53

971 3741 54

971 4850 1402

971 5099 95

971 6120 200

971 6647 105

971 8728 70

971 8732 241

971 9183 55

971 9185 104

971 11154 59

971 11470 156

971 11471 151

971 11472 68

971 11473 51

971 11474 49

972 54 118

972 63 276

972 93 164

972 154 378

972 163 586

972 418 474

972 506 103

972 610 103

972 632 146

972 689 103

972 755 154

972 964 190

972 1098 140

972 1109 195

972 1117	938
972 1148	331
972 1385	105
972 1418	280
972 1423	584
972 1700	288
972 1708	141
972 1737	207
972 1770	164
972 1781	169
972 1783	140
972 1790	203
972 1835	343
972 2711	528
972 2775	325
972 3815	125
972 4087	133
972 4123	169
972 4437	1592
972 5441	182
972 5799	181
972 6048	220
972 6179	109
972 6187	141
972 7382	320
972 9555	198
972 11475	474
972 11476	425
972 11477	385
972 11478	144

972 11479 132

972 11480 121

972 11481 121

972 11482 116

972 11483 110

972 11484 102

975 65 1154

975 161 1347

975 182 734

975 190 711

975 209 818

975 234 691

975 288 925

975 291 801

975 310 715

975 316 1955

975 333 2276

975 377 833

975 378 2237

975 405 807

975 412 795

975 481 697

975 488 1276

975 498 2052

975 499 1436

975 523 691

975 536 828

975 537 1528

975 538 713

975 641 1204

975 701 1351

975 971 1357

975 972 839

975 1181 803

975 1186 1314

975 1375 655

975 1377 754

975 1378 979

975 1432 823

975 1438 864

975 1470 786

975 1656 783

975 1854 1297

975 1866 1318

975 3094 756

975 3187 1954

975 4179 831

975 4432 693

975 4817 1186

975 5140 1709

975 5249 883

975 6505 1104

975 7846 957

975 9183 1053

975 10900 1017

975 11498 726

976 166 80

976 171 175

976 173 93

976 190 65

976 193 38

976 333 126

976 378 48

976 396 34

976 472 60

976 488 54

976 498 93

976 499 286

976 567 45

976 599 125

976 697 41

976 710 249

976 714 138

976 843 220

976 868 44

976 951 304

976 952 466

976 961 37

976 966 35

976 1182 157

976 1502 487

976 1803 101

976 1859 36

976 2343 37

976 2346 259

976 2525 148

976 2780 227

976 2787 780

976 2797 41

976 2840 122

976 2893 128

976 3057 42

976 3333 274

976 3741 47

976 4165 56

976 4336 42

976 4747 78

976 6231 48

976 6235 35

976 7321 288

976 9737 79

976 10668 149

976 11499 122

976 11500 105

976 11501 40

976 11502 39

977 72 154

977 154 79

977 173 40

977 190 19

977 203 48

977 212 33

977 220 1214

977 222 31

977 227 263

977 228 32

977 230 95

977 316 68

977 377 44

977 511 157

977 534 26

977 603 20

977 605 33

977 614 55

977 630 33

977 715 900

977 733 19

977 734 67

977 857 34

977 858 44

977 862 26

977 951 37

977 961 18

977 969 20

977 982 28

977 1133 28

977 1464 33

977 1601 20

977 1977 41

977 2265 20

977 2475 20

977 2542 32

977 3364 21

977 4133 29

977 5890 25

977 6724 1327

977 7260 39

977 7525 41

977 10063 24

977 11503 82

977 11504 80

977 11505 57

977 11506 56

977 11507 26

977 11508 26

977 11509 23

978 152 2183

978 162 295

978 186 444

978 188 501

978 196 349

978 370 335

978 418 722

978 506 435

978 621 474

978 632 305

978 751 279

978 769 283

978 812 302

978 917 350

978 920 604

978 923 369

978 924 286

978 930 400

978 937 381

978 1113 305

978 1134 298

978 1295 462

978 1339 1322

978 1418 346

978 1709 336

978 1710 504

978 1743 292

978 1994 503

978 2006 1169

978 2370 395

978 2418 274

978 2573 721

978 2962 271

978 2965 573

978 3272 305

978 3417 325

978 3663 380

978 3788 397

978 4610 339

978 6657 287

978 6765 488

978 9715 367

978 10675 625

978 11510 1463

978 11511 877

978 11512 604

978 11513 508

978 11514 471

978 11515 413

978 11516 274

979 7 638

979 89 181

979 230 281

979 233 1168

979 302 332

979 377 190

979 496 219

979 497 266

979 517 439

979 538 244

979 540 164

979 544 307

979 707 1028

979 709 309

979 724 275

979 843 209

979 854 189

979 860 239

979 868 313

979 959 452

979 978 201

979 979 229

979 1104 171

979 1249 269

979 1360 391

979 1412 247

979 1803 234

979 1976 549

979 2346 206

979 2369 346

979 2380 327

979 2478 191

979 2523 155

979 2595 451

979 2783	220
979 2791	169
979 2851	234
979 3445	252
979 3475	362
979 5421	398
979 6257	340
979 7419	254
979 9144	348
979 10069	205
979 10857	239
979 10859	338
979 10977	273
979 11517	576
979 11518	381
979 11519	240
980 747 103	
980 782 84	
980 1226	65
980 5325	89
980 5327	96
980 5329	65
980 5342	69
980 5343	103
980 5346	109
980 5348	77
980 5350	131
980 5359	86
980 5360	92
980 5361	68

980 7017	88
980 7018	60
980 7021	65
980 7029	86
980 9353	110
980 9354	91
980 9356	139
980 9357	102
980 11193	129
980 11520	146
980 11521	125
980 11522	109
980 11523	105
980 11524	104
980 11525	103
980 11526	101
980 11527	96
980 11528	94
980 11529	91
980 11530	90
980 11531	89
980 11532	78
980 11533	75
980 11534	72
980 11535	68
980 11536	67
980 11537	66
980 11538	65
980 11539	65
980 11540	64

980 11541 64

980 11542 63

980 11543 62

980 11544 57

980 11545 56

980 11546 55

981 199 117

981 210 147

981 217 192

981 222 271

981 223 113

981 298 147

981 324 202

981 425 113

981 433 226

981 492 170

981 540 111

981 602 112

981 1096 363

981 1159 173

981 1378 122

981 1390 122

981 1645 126

981 1723 439

981 1858 203

981 1947 218

981 2121 164

981 2425 283

981 2661 256

981 3616 1675

981 3673 199

981 3705 112

981 4118 179

981 4223 119

981 4387 125

981 4837 112

981 6228 227

981 6293 481

981 6294 146

981 6298 145

981 6463 493

981 6773 510

981 7723 141

981 8304 148

981 8459 115

981 11412 919

981 11547 230

981 11548 229

981 11549 212

981 11550 201

981 11551 179

981 11552 172

981 11553 160

981 11554 150

981 11555 148

981 11556 128

982 56 63

982 66 61

982 67 124

982 89 1136

982 163 96

982 174 115

982 229 62

982 331 322

982 691 62

982 757 134

982 768 58

982 930 118

982 968 559

982 1410 94

982 1613 104

982 1705 135

982 1885 103

982 1970 79

982 2083 127

982 2247 81

982 2668 145

982 2906 58

982 3691 57

982 3703 87

982 3712 124

982 3882 118

982 4028 71

982 4706 107

982 4775 64

982 5071 99

982 5074 61

982 5348 91

982 6052 233

982 7340 369

982 9388 61

982 11193 82

982 11557 511

982 11558 247

982 11559 198

982 11560 145

982 11561 144

982 11562 126

982 11563 88

982 11564 72

982 11565 69

982 11566 65

982 11567 61

982 11568 59

982 11569 58

982 11570 57

984 67 5927

984 89 19919

984 161 4985

984 190 830

984 191 708

984 288 19833

984 289 17977

984 295 3070

984 300 6478

984 302 1835

984 306 2290

984 317 633

984 328 1569

984 329 905

984 331 3048

984 333 666

984 344 612

984 352 918

984 371 2501

984 377 2374

984 386 599

984 458 1252

984 464 11762

984 466 1248

984 475 3241

984 486 437

984 502 835

984 518 552

984 545 440

984 547 441

984 674 887

984 679 5971

984 701 4558

984 907 720

984 1441	1971
984 1449	3983
984 1452	5632
984 1455	2843
984 1470	4804
984 1576	2245
984 3123	626
984 4242	537
984 4688	445
984 4814	504

984 9684 2020
984 11574 4052
984 11575 3728
984 11576 2481
984 11577 1932
984 11578 802
985 55 532
985 67 39865
985 89 12693
985 157 23561
985 161 361
985 265 365
985 283 830
985 285 1703
985 288 6461
985 291 984
985 292 23174
985 295 6228
985 299 1925
985 300 1379
985 302 3244
985 306 1175
985 310 8546
985 314 1013
985 316 2082
985 320 850
985 325 596
985 328 393
985 329 3097
985 333 1094

985 349 2439

985 360 535

985 377 569

985 378 2521

985 484 366

985 498 498

985 525 2378

985 526 998

985 534 1674

985 538 541

985 542 1523

985 701 4647

985 714 1850

985 727 2507

985 903 315

985 907 1553

985 913 1008

985 972 1389

985 1032 382

985 1052 351

985 1243 2572

985 1464 729

985 3313 330

985 3992 707

985 6777 473

985 11579 337

986 198 787

986 209 58

986 226 56

986 230 59

986 324 57

986 377 3685

986 412 43

986 481 39

986 488 238

986 493 134

986 497 397

986 498 186

986 500 542

986 536 152

986 810 169

986 813 2779

986 816 982

986 830 3869

986 846 258

986 917 146

986 918 169

986 930 667

986 1044 703

986 1114 85

986 1145 853

986 1180 398

986 1195 33

986 1196 42

986 1200 68

986 1358 41

986 1470 566

986 1541 360

986 1672 12487

986 1854 155

986 1860 78
986 1861 288
986 1874 210
986 1974 76
986 2214 290
986 2346 299
986 2379 274
986 2576 83
986 2619 56
986 2794 379
986 3946 287
986 4170 82
986 6453 37
986 9941 70
986 11580 150
986 11581 60
987 67 205
987 89 2518
987 97 646
987 257 276
987 288 1301
987 289 582
987 291 135
987 292 7462
987 294 499
987 295 2932
987 298 284
987 299 356
987 300 519
987 301 185

987 308 3175

987 309 520

987 317 687

987 325 224

987 344 365

987 349 2601

987 466 754

987 525 402

987 528 1244

987 538 132

987 543 320

987 545 190

987 689 344

987 907 174

987 913 197

987 1032 473

987 1127 213

987 1243 203

987 1451 9839

987 1452 307

987 1456 126

987 1459 358

987 1486 1239

987 1934 1508

987 1936 663

987 2392 149

987 3194 344

987 3397 261

987 3399 296

987 4562 140

987 5150 151
987 7393 715
987 8364 178
987 11582 394
987 11583 301
987 11584 171
989 67 169
989 89 378
989 154 241
989 195 159
989 196 871
989 199 325
989 203 230
989 206 415
989 227 347
989 261 212
989 298 222
989 418 1149
989 424 184
989 429 142
989 439 290
989 440 202
989 618 669
989 622 207
989 629 591
989 858 724
989 981 263
989 1097 148
989 1098 4499
989 1106 296

989 1155 145

989 1159 527

989 1390 479

989 1424 260

989 1553 882

989 1730 383

989 1735 176

989 1954 158

989 2083 788

989 2268 167

989 2277 158

989 2287 172

989 2521 577

989 3697 183

989 4532 233

989 5150 407

989 6217 175

989 6756 388

989 7505 202

989 10014 156

989 10415 150

989 11587 412

989 11588 401

989 11589 173

989 11590 158

989 11591 156

990 53 652

990 59 587

990 65 535

990 70 509

990 88 555

990 154 3373

990 203 1765

990 204 248

990 206 244

990 225 1212

990 227 401

990 237 263

990 418 2408

990 429 246

990 433 2053

990 436 202

990 441 1462

990 444 585

990 599 462

990 603 237

990 604 619

990 620 447

990 851 165

990 971 278

990 1073 256

990 1098 854

990 1107 357

990 1151 261

990 1709 671

990 1958 586

990 2606 291

990 2691 533

990 3112 550

990 3208 505

990 3252 588

990 3411 342

990 3898 173

990 4444 887

990 5925 244

990 6217 8509

990 6410 536

990 6762 257

990 8027 530

990 8678 211

990 10111 585

990 10969 890

990 11084 259

990 11592 292

990 11593 267

990 11594 177

991 7 321

991 65 1331

991 152 1747

991 154 1706

991 163 14473

991 182 307

991 184 161

991 186 168

991 190 310

991 197 1435

991 203 959

991 212 672

991 221 588

991 227 380

991 230 149

991 234 222

991 238 386

991 333 133

991 377 478

991 432 298

991 506 390

991 511 214

991 533 4715

991 562 343

991 607 137

991 609 135

991 630 147

991 712 728

991 730 275

991 733 424

991 1090 841

991 1131 162

991 1150 196

991 1186 857

991 1244 332

991 1384 353

991 1412 332

991 1639 351

991 1778 226

991 1790 531

991 1852 227

991 4675 310

991 5202 154

991 7935 170

991 8958 155

991 8964 348

991 11595 703

991 11596 310

991 11597 182

991 11598 147

993 217 98

993 225 90

993 412 220

993 441 104

993 547 80

993 751 250

993 769 127

993 841 71

993 1195 74

993 2273 50

993 2582 49

993 2817 112

993 3028 47

993 3040 70

993 3826 46

993 4262 78

993 4273 129

993 4283 54

993 5135 54

993 6286 53

993 6410 56

993 6759 63

993 7665 55

993 8475 42

993 10612 84

993 10890 64

993 11604 196

993 11605 187

993 11606 174

993 11607 148

993 11608 141

993 11609 138

993 11610 124

993 11611 100

993 11612 88

993 11613 73

993 11614 72

993 11615 66

993 11616 64

993 11617 60

993 11618 59

993 11619 53

993 11620 53

993 11621 52

993 11622 48

993 11623 47

993 11624 47

993 11625 44

993 11626 43

993 11627 42

994 58 202

994 70 868

994 89 169

994 157 200

994 163 154

994 190 260

994 227 743

994 238 170

994 298 1743

994 300 232

994 348 188

994 349 288

994 375 688

994 418 3323

994 454 226

994 728 186

994 910 111

994 981 133

994 1073 743

994 1098 916

994 1104 445

994 1148 722

994 1726 432

994 2075 273

994 3324 324

994 3890 79

994 4520 137

994 4649 296

994 6140 1432

994 6150 10154

994 6582 122

994 7802 78

994 8027 99

994 8689 83

994 8802 827

994 11628 717

994 11629 575

994 11630 249

994 11631 185

994 11632 181

994 11633 177

994 11634 145

994 11635 138

994 11636 112

994 11637 107

994 11638 105

994 11639 95

994 11640 89

994 11641 86

994 11642 84

995 89 878

995 257 2

995 288 241

995 289 174

995 295 4

995 299 7

995 300 296

995 306 15

995 328 9

995 333 48

995 344 162

995 461 172

995 466 260

995 468 10

995 475 1

995 484 10

995 498 30

995 528 5

995 543 53

995 544 27

995 679 24

995 686 207

995 701 12

995 790 1

995 797 16

995 802 1

995 1032 8

995 1428 1

995 1447 7

995 1452 28

995 1453 3

995 1455 20

995 1456 9

995 1458 12

995 1690 17

995 6590 7

995 11643 7

995 11644 1

996 610 181

996 613 119

996 625 139

996 2292 82

996 3004 151

996 4253 71

996 7123	76
996 8110	72
996 8837	88
996 8843	79
996 10209	107
996 11645	208
996 11646	168
996 11647	151
996 11648	148
996 11649	145
996 11650	141
996 11651	137
996 11652	130
996 11653	130
996 11654	124
996 11655	123
996 11656	121
996 11657	114
996 11658	114
996 11659	111
996 11660	107
996 11661	100
996 11662	96
996 11663	95
996 11664	94
996 11665	94
996 11666	94
996 11667	93
996 11668	92
996 11669	91

996 11670 88

996 11671 88

996 11672 86

996 11673 85

996 11674 84

996 11675 83

996 11676 78

996 11677 72

996 11678 71

996 11679 70

996 11680 70

996 11681 70

996 11682 69

996 11683 68

997 53 627

997 154 1323

997 170 767

997 190 381

997 211 286

997 226 542

997 233 300

997 267 284

997 331 805

997 436 667

997 527 745

997 617 393

997 618 626

997 622 633

997 629 521

997 757 1325

997 816 689

997 831 349

997 1062 550

997 1100 996

997 1118 417

997 1131 441

997 1187 494

997 1216 467

997 1613 345

997 1614 386

997 1943 303

997 1948 357

997 1950 344

997 1958 293

997 1963 366

997 1964 393

997 2210 280

997 2405 297

997 2574 451

997 2847 312

997 2921 307

997 3277 458

997 3411 532

997 3673 327

997 3693 644

997 4497 578

997 5652 312

997 5782 588

997 6191 329

997 10549 329

997 10615 364

997 11684 360

997 11685 359

997 11686 296

998 89 339

998 97 108

998 209 245

998 262 139

998 288 132

998 289 120

998 300 350

998 306 378

998 333 73481

998 344 684

998 367 226

998 369 104

998 388 374

998 409 125

998 427 106

998 458 148

998 498 119

998 679 106

998 701 110

998 898 134

998 1857 189

998 1904 43001

998 1918 141

998 2040 127

998 2089 148

998 2106 119

998 2343 130

998 2544 113

998 2798 103

998 4923 167

998 5575 129

998 5948 104

998 9178 502

998 9199 234

998 11687 1501

998 11688 1458

998 11689 1218

998 11690 656

998 11691 289

998 11692 206

998 11693 167

998 11694 162

998 11695 156

998 11696 137

998 11697 130

998 11698 115

998 11699 114

998 11700 112

998 11701 111

998 11702 110

999 65 433

999 152 401

999 154 218

999 188 128

999 190 344

999 227 100

999 233 72

999 234 128

999 321 422

999 344 129

999 355 80

999 377 327

999 418 167

999 461 206

999 486 140

999 498 125

999 506 565

999 511 307

999 533 131

999 538 232

999 679 220

999 681 413

999 755 263

999 910 104

999 920 1409

999 964 281

999 1110 145

999 1116 181

999 1118 223

999 1132 215

999 1172 88

999 1413 171

999 1470 155

999 1705 298

999 2006 244

999 2014 281

999	2151	158
999	2574	143
999	2775	250
999	3417	166
999	3779	100
999	3900	184
999	3901	186
999	3940	149
999	3998	210
999	6957	128
999	8690	159
999	9716	80
999	10472	170
999	11703	143
1001	56	162
1001	67	245
1001	191	2232
1001	234	689
1001	289	453
1001	290	201
1001	291	21086
1001	292	4047
1001	293	213
1001	297	1807
1001	298	158
1001	302	242
1001	304	275
1001	333	276
1001	378	470
1001	464	297

1001	511	177
1001	518	532
1001	679	2745
1001	681	327
1001	683	271
1001	691	179
1001	700	253
1001	701	342
1001	704	1409
1001	951	241
1001	966	208
1001	1043	166
1001	1047	703
1001	1447	461
1001	1459	1252
1001	1470	709
1001	1807	240
1001	1934	972
1001	1976	346
1001	2051	337
1001	2150	443
1001	2439	247
1001	2977	1858
1001	3073	282
1001	3489	1452
1001	3739	371
1001	3781	359
1001	3859	310
1001	4559	224
1001	7318	175

1001	9250	204
1001	10967	200
1001	11717	1166
1001	11718	306
1002	55	114
1002	72	431
1002	89	148
1002	157	72
1002	175	70
1002	239	96
1002	252	73
1002	257	63
1002	279	85
1002	287	92
1002	288	165
1002	291	99
1002	293	65
1002	294	78
1002	295	79
1002	296	110
1002	299	110
1002	310	91
1002	314	118
1002	317	56
1002	322	114
1002	323	228
1002	328	77
1002	332	106
1002	333	61
1002	349	126

1002	535	94
1002	701	98
1002	722	76
1002	800	77
1002	906	808
1002	909	71
1002	911	189
1002	913	81
1002	1001	89
1002	1023	140
1002	1596	87
1002	1937	95
1002	2018	58
1002	3314	84
1002	3498	72
1002	3985	60
1002	4566	108
1002	6429	56
1002	8076	68
1002	11719	129
1002	11720	82
1002	11721	78
1002	11722	57
1002	11723	56
1003	51	919
1003	55	212
1003	58	213
1003	67	571
1003	77	196
1003	89	555

1003	96	554
1003	155	528
1003	157	399
1003	159	384
1003	167	256
1003	187	393
1003	192	259
1003	228	197
1003	229	272
1003	253	312
1003	289	250
1003	323	477
1003	352	206
1003	511	313
1003	548	221
1003	576	199
1003	599	214
1003	601	322
1003	734	279
1003	903	245
1003	906	527
1003	959	605
1003	997	212
1003	1001	261
1003	1014	240
1003	1427	428
1003	1485	262
1003	2444	222
1003	2607	194
1003	3306	255

1003	3313	233
1003	3576	326
1003	4130	337
1003	5000	3305
1003	6714	248
1003	9631	208
1003	11724	709
1003	11725	457
1003	11726	437
1003	11727	333
1003	11728	281
1003	11729	210
1003	11730	208
1003	11731	203
1004	159 9	
1004	197 4	
1004	554 1	
1004	601 1	
1004	859 1	
1004	917 1	
1004	1947	2
1004	1976	1
1004	2289	1
1004	2422	1
1004	2541	11
1004	3267	26
1004	3730	2
1004	4384	1
1004	4620	1
1004	4795	1

1004	5412	2
1004	6382	2
1004	6687	16
1004	7324	2
1004	9877	2
1004	11732	41
1004	11733	32
1004	11734	21
1004	11735	14
1004	11736	10
1004	11737	9
1004	11738	8
1004	11739	6
1004	11740	5
1004	11741	2
1004	11742	2
1004	11743	2
1004	11744	2
1004	11745	1
1004	11746	1
1004	11747	1
1004	11748	1
1004	11749	1
1004	11750	1
1004	11751	1
1005	207 402	
1005	249 345	
1005	271 895	
1005	276 307	
1005	306 286	

1005	331 1799	
1005	366 657	
1005	547 936	
1005	617 634	
1005	619 530	
1005	1155	300
1005	1605	476
1005	1613	1398
1005	1620	433
1005	1622	621
1005	1627	1932
1005	1941	852
1005	1943	458
1005	1946	1908
1005	1948	466
1005	1950	596
1005	1954	311
1005	1975	3982
1005	2132	341
1005	2605	706
1005	2716	319
1005	2923	882
1005	2931	701
1005	3707	404
1005	5041	1341
1005	5048	1205
1005	5883	554
1005	6226	707
1005	6581	498
1005	7665	655

1005	8474	366
1005	9912	615
1005	10398	677
1005	10914	692
1005	11752	1375
1005	11753	1086
1005	11754	1050
1005	11755	551
1005	11756	543
1005	11757	508
1005	11758	482
1005	11759	482
1005	11760	334
1005	11761	328
1005	11762	285
1006	53	285
1006	55	349
1006	65	605
1006	67	1202
1006	69	549
1006	154	528
1006	171	219
1006	203	246
1006	227	358
1006	230	324
1006	231	304
1006	246	228
1006	261	404
1006	266	471
1006	270	1188

1006	289	580
1006	295	225
1006	301	377
1006	316	540
1006	329	258
1006	436	422
1006	439	557
1006	618	393
1006	701	292
1006	709	221
1006	735	399
1006	903	298
1006	1239	289
1006	1394	424
1006	1424	219
1006	1553	297
1006	1755	2703
1006	1756	380
1006	1757	285
1006	1764	717
1006	1772	463
1006	1775	223
1006	1833	740
1006	2460	266
1006	2632	219
1006	5745	894
1006	5787	304
1006	6137	324
1006	9700	465
1006	10423	1002

1006	11763	596
1006	11764	360
1006	11765	276
1006	11766	252
1006	11767	239
1007	157 69	
1007	161 60	
1007	291 47	
1007	356 91	
1007	360 64	
1007	455 56	
1007	718 47	
1007	1244	126
1007	1459	48
1007	1489	124
1007	1694	83
1007	2442	124
1007	2445	75
1007	2449	80
1007	2454	53
1007	2455	165
1007	2466	69
1007	2467	231
1007	2468	157
1007	3306	92
1007	3581	172
1007	3584	55
1007	3588	111
1007	3590	51
1007	3593	53

1007	3601	55
1007	3602	85
1007	3609	56
1007	4520	46
1007	4535	54
1007	4542	159
1007	4543	45
1007	8364	49
1007	8557	101
1007	11640	73
1007	11768	189
1007	11769	152
1007	11770	83
1007	11771	77
1007	11772	74
1007	11773	72
1007	11774	70
1007	11775	66
1007	11776	66
1007	11777	64
1007	11778	53
1007	11779	50
1007	11780	49
1007	11781	47
1007	11782	43
1008	65 130	
1008	70 96	
1008	107 151	
1008	159 72	
1008	196 129	

1008	217 55	
1008	233 65	
1008	429 73	
1008	441 128	
1008	619 97	
1008	1090	61
1008	1239	54
1008	1700	76
1008	1717	104
1008	1747	88
1008	1947	90
1008	2582	137
1008	3357	109
1008	3417	1279
1008	3695	81
1008	3703	1046
1008	3718	388
1008	4096	109
1008	4271	87
1008	4970	117
1008	5299	69
1008	5807	289
1008	6163	153
1008	6582	99
1008	6764	160
1008	6773	155
1008	6887	135
1008	6906	154
1008	7152	97
1008	7665	123

1008	8459	1418
1008	8466	71
1008	8472	73
1008	8474	79
1008	10549	60
1008	11783	952
1008	11784	478
1008	11785	392
1008	11786	111
1008	11787	90
1008	11788	81
1008	11789	65
1008	11790	63
1008	11791	62
1008	11792	55
1009	173 110	
1009	181 6	
1009	190 52	
1009	209 79	
1009	229 141	
1009	230 9	
1009	377 55	
1009	378 296	
1009	403 82	
1009	412 25	
1009	424 90	
1009	472 28	
1009	481 148	
1009	486 376	
1009	493 117	

1009	497	15
1009	498	82
1009	501	106
1009	504	11
1009	536	122
1009	540	67
1009	951	5
1009	966	69
1009	969	51
1009	1043	98
1009	1046	12
1009	1048	84
1009	1075	74
1009	1090	46
1009	1091	52
1009	1179	13
1009	1186	10
1009	1246	76
1009	1298	9
1009	1375	38
1009	1381	43
1009	1406	9
1009	1874	9
1009	2131	9
1009	2614	28
1009	2840	6
1009	3078	27
1009	3400	26
1009	3427	10
1009	3753	15

1009	3945	12
1009	4177	33
1009	4742	10
1009	7187	20
1009	11793	8
1010	51	18
1010	59	2
1010	63	2
1010	68	8
1010	72	7
1010	75	3
1010	77	3
1010	85	10
1010	99	3
1010	157	5
1010	180	3
1010	187	6
1010	189	16
1010	193	11
1010	488	4
1010	877	5
1010	966	3
1010	993	8
1010	997	10
1010	998	4
1010	999	9
1010	1013	3
1010	1014	7
1010	1019	8
1010	1076	3

1010	1079	3
1010	1249	3
1010	1810	11
1010	1854	4
1010	1885	6
1010	1889	4
1010	1892	3
1010	1895	3
1010	2027	6
1010	2347	2
1010	2526	3
1010	2562	4
1010	2787	5
1010	2936	12
1010	2941	5
1010	2973	3
1010	3768	6
1010	3859	3
1010	5165	4
1010	5254	5
1010	5841	4
1010	6698	3
1010	6732	4
1010	7927	3
1010	11794	3
1011	7	449
1011	45	4204
1011	209	2446
1011	230	1745
1011	298	224

1011	324 1310	
1011	362 230	
1011	377 219	
1011	378 1983	
1011	464 572	
1011	466 265	
1011	470 201	
1011	471 337	
1011	487 1244	
1011	492 189	
1011	498 1914	
1011	499 1422	
1011	518 430	
1011	524 1049	
1011	537 1656	
1011	687 3456	
1011	691 589	
1011	704 217	
1011	779 476	
1011	816 957	
1011	1034	1299
1011	1035	453
1011	1044	1546
1011	1180	1108
1011	1185	483
1011	1191	287
1011	1194	262
1011	1246	235
1011	1590	396
1011	1670	203

1011	1678	623
1011	1680	252
1011	1863	1075
1011	2432	305
1011	2617	229
1011	2959	204
1011	3247	558
1011	4385	307
1011	5065	243
1011	5308	507
1011	5437	207
1011	6448	304
1011	6605	379
1011	11795	1248
1011	11796	215
1012	7	149
1012	45	547
1012	56	99
1012	173	134
1012	190	1442
1012	206	1361
1012	209	1003
1012	228	79
1012	230	198
1012	233	1300
1012	234	79
1012	331	56
1012	378	74
1012	403	77
1012	412	90

1012	425	93
1012	486	137
1012	498	61
1012	504	159
1012	537	999
1012	540	600
1012	709	137
1012	907	98
1012	913	54
1012	969	99
1012	975	69
1012	982	67
1012	1048	75
1012	1098	112
1012	1099	103
1012	1162	74
1012	1194	72
1012	1210	232
1012	1246	246
1012	1378	77
1012	1382	56
1012	1672	75
1012	1974	91
1012	1983	130
1012	2014	106
1012	2580	67
1012	2600	128
1012	2823	128
1012	2840	60
1012	2841	90

1012	3058	79
1012	5111	252
1012	5439	65
1012	6453	872
1012	11754	375
1013	4229	12
1014	65	1
1014	67	314
1014	288	1
1014	301	2
1014	306	6
1014	327	1
1014	328	14
1014	455	7
1014	458	54
1014	461	4
1014	466	10
1014	527	1
1014	545	1
1014	686	1
1014	903	1
1014	1037	3
1014	1042	2
1014	1444	2
1014	1449	7
1014	1452	8
1014	1455	1
1014	1475	7
1014	1673	18
1014	4811	3

1014	8375	2
1015	65	546
1015	196	394
1015	217	126
1015	310	123
1015	366	170
1015	602	185
1015	606	212
1015	622	1239
1015	701	123
1015	1375	201
1015	1377	108
1015	1383	200
1015	1725	174
1015	1772	217
1015	2277	155
1015	2425	543
1015	2440	201
1015	2458	116
1015	2693	178
1015	3078	149
1015	3258	277
1015	3617	419
1015	3670	202
1015	3706	128
1015	3750	149
1015	4121	375
1015	4177	437
1015	4361	238
1015	5480	132

1015	5972	161
1015	6298	104
1015	6602	141
1015	9707	118
1015	10381	143
1015	11223	144
1015	11267	256
1015	11797	863
1015	11798	245
1015	11799	207
1015	11800	164
1015	11801	160
1015	11802	149
1015	11803	143
1015	11804	142
1015	11805	142
1015	11806	134
1015	11807	129
1015	11808	122
1015	11809	120
1015	11810	102
1016	159 1066	
1016	195 1979	
1016	210 812	
1016	211 478	
1016	217 489	
1016	220 772	
1016	222 397	
1016	227 1501	
1016	412 416	

1016	416	1056
1016	425	774
1016	426	680
1016	433	494
1016	602	456
1016	604	847
1016	615	1392
1016	621	667
1016	622	1169
1016	632	854
1016	709	417
1016	1159	1019
1016	1231	532
1016	1375	887
1016	1424	860
1016	1632	466
1016	1944	619
1016	2277	492
1016	2281	700
1016	2407	1031
1016	2616	559
1016	2628	773
1016	2661	656
1016	2840	989
1016	3097	576
1016	3258	526
1016	3341	1291
1016	3398	555
1016	3616	1122
1016	4092	429

1016	4118	560
1016	4385	576
1016	6146	433
1016	6302	436
1016	6771	467
1016	6773	1217
1016	6836	1148
1016	7274	589
1016	9485	796
1016	11811	1091
1016	11812	613
1017	55	493
1017	67	211
1017	89	464
1017	161	381
1017	191	366
1017	257	684
1017	265	169
1017	288	516
1017	289	5479
1017	290	175
1017	291	733
1017	292	974
1017	293	257
1017	294	1397
1017	295	383
1017	296	423
1017	299	583
1017	300	229
1017	301	197

1017	310	170
1017	311	284
1017	318	447
1017	325	1035
1017	328	307
1017	333	264
1017	342	162
1017	344	197
1017	349	211
1017	352	1398
1017	386	173
1017	461	489
1017	465	858
1017	466	386
1017	476	246
1017	543	298
1017	637	207
1017	641	831
1017	644	314
1017	701	881
1017	909	633
1017	915	433
1017	966	179
1017	1025	168
1017	1051	240
1017	1459	376
1017	1591	221
1017	3397	390
1017	3491	1508
1017	6308	227

1017	11813	155
1018	12	375
1018	15	367
1018	154	352
1018	163	515
1018	186	2244
1018	188	723
1018	370	1295
1018	503	871
1018	769	468
1018	811	571
1018	812	1228
1018	813	3217
1018	815	690
1018	821	839
1018	834	444
1018	841	356
1018	846	1511
1018	943	574
1018	953	573
1018	1131	343
1018	1214	317
1018	1861	341
1018	2172	818
1018	2370	593
1018	2374	2467
1018	2378	315
1018	2379	490
1018	2384	319
1018	2765	902

1018	2963	360
1018	2970	582
1018	3221	299
1018	3717	339
1018	3722	541
1018	4004	464
1018	4612	585
1018	4669	291
1018	4678	515
1018	5100	281
1018	5667	375
1018	8661	775
1018	9410	682
1018	9412	305
1018	10091	873
1018	10895	504
1018	11814	945
1018	11815	420
1018	11816	418
1018	11817	302
1018	11818	295
1019	65	270
1019	154	261
1019	166	165
1019	190	194
1019	198	247
1019	202	423
1019	217	167
1019	227	636
1019	229	195

1019	289	233
1019	298	215
1019	412	162
1019	424	192
1019	439	330
1019	494	208
1019	517	469
1019	537	406
1019	540	243
1019	618	172
1019	701	146
1019	709	266
1019	989	205
1019	1048	158
1019	1093	338
1019	1181	179
1019	1228	168
1019	1231	151
1019	1241	160
1019	1243	182
1019	1246	196
1019	1755	237
1019	2276	145
1019	2430	150
1019	2523	197
1019	2573	160
1019	3200	270
1019	3341	343
1019	3382	151
1019	3391	179

1019	3468	382
1019	4201	184
1019	4262	190
1019	4434	157
1019	4941	247
1019	5027	282
1019	6602	325
1019	8467	254
1019	8710	174
1019	11550	148
1019	11819	170
1020	45	257
1020	88	236
1020	209	310
1020	227	906
1020	230	426
1020	234	843
1020	257	552
1020	291	583
1020	293	270
1020	298	372
1020	300	653
1020	325	232
1020	333	1134
1020	377	740
1020	378	711
1020	403	250
1020	458	275
1020	461	745
1020	466	432

1020	470 4342	
1020	472 296	
1020	475 247	
1020	498 2571	
1020	500 577	
1020	536 239	
1020	537 971	
1020	540 261	
1020	680 799	
1020	681 398	
1020	687 241	
1020	693 266	
1020	704 1669	
1020	707 318	
1020	951 305	
1020	982 279	
1020	1035	517
1020	1043	463
1020	1044	530
1020	1047	362
1020	1180	669
1020	1182	297
1020	1189	369
1020	1246	234
1020	1249	417
1020	1513	227
1020	1672	2693
1020	1853	224
1020	2959	431
1020	2987	330

1020	5253	679
1021	72	518
1021	96	258
1021	154	323
1021	159	208
1021	163	840
1021	199	417
1021	202	390
1021	203	405
1021	212	201
1021	220	246
1021	225	703
1021	226	308
1021	227	371
1021	228	574
1021	366	638
1021	436	306
1021	443	213
1021	511	332
1021	554	286
1021	562	790
1021	599	642
1021	706	317
1021	709	838
1021	715	353
1021	718	267
1021	728	366
1021	735	894
1021	858	342
1021	868	340

1021	910	317
1021	959	356
1021	975	303
1021	982	370
1021	1103	213
1021	1126	381
1021	1242	202
1021	1372	215
1021	1412	1189
1021	1413	242
1021	1415	254
1021	1426	295
1021	1772	355
1021	1795	214
1021	2136	215
1021	2786	205
1021	3308	203
1021	4922	208
1021	5605	597
1021	7126	405
1021	7227	201
1022	65	265
1022	89	561
1022	155	1426
1022	157	727
1022	167	269
1022	184	609
1022	209	651
1022	227	3267
1022	298	479

1022	300	292
1022	302	358
1022	321	2595
1022	333	486
1022	455	276
1022	464	554
1022	470	545
1022	524	432
1022	540	625
1022	544	5550
1022	599	491
1022	679	415
1022	735	372
1022	951	4854
1022	959	1319
1022	1042	524
1022	1048	407
1022	1183	637
1022	1243	819
1022	1244	295
1022	1351	382
1022	1415	372
1022	1648	394
1022	1803	713
1022	2050	574
1022	2176	547
1022	2653	282
1022	2823	1052
1022	3266	2094
1022	3712	418

1022	3906	484
1022	3915	560
1022	4165	505
1022	5000	887
1022	5306	418
1022	5926	815
1022	6291	478
1022	7044	449
1022	10582	370
1022	11820	734
1022	11821	310
1023	55	1449
1023	67	4252
1023	89	3487
1023	157	434
1023	220	73
1023	288	260
1023	289	15339
1023	292	5001
1023	295	152
1023	298	524
1023	300	1778
1023	302	80
1023	306	72
1023	310	133
1023	333	1081
1023	349	81
1023	350	186
1023	352	332
1023	375	649

1023	466	1160
1023	486	492
1023	525	134
1023	528	55
1023	532	76
1023	679	295
1023	689	182
1023	691	194
1023	701	172
1023	704	113
1023	708	228
1023	789	84
1023	797	75
1023	886	130
1023	906	149
1023	908	70
1023	970	145
1023	1034	112
1023	1456	72
1023	1505	255
1023	1705	195
1023	2102	210
1023	2523	118
1023	2543	123
1023	2801	116
1023	3194	133
1023	3200	107
1023	3324	191
1023	3391	72
1023	4704	236

1023	6262	66
1024	7	157
1024	45	932
1024	190	767
1024	198	3969
1024	289	302
1024	293	147
1024	324	731
1024	377	1831
1024	378	222
1024	486	81
1024	497	148
1024	498	179
1024	519	579
1024	536	733
1024	540	319
1024	543	77
1024	707	122
1024	726	126
1024	754	452
1024	978	2086
1024	1044	2131
1024	1099	840
1024	1104	2509
1024	1154	305
1024	1246	921
1024	1714	495
1024	1859	1005
1024	1860	632
1024	2841	139

1024	3240	183
1024	3367	147
1024	4161	116
1024	4430	153
1024	4502	857
1024	4980	328
1024	5988	145
1024	7639	312
1024	9576	171
1024	10583	1591
1024	11822	975
1024	11823	880
1024	11824	328
1024	11825	209
1024	11826	168
1024	11827	139
1024	11828	128
1024	11829	101
1024	11830	92
1024	11831	88
1024	11832	83
1025	154 111	
1025	163 650	
1025	196 587	
1025	207 131	
1025	212 89	
1025	220 661	
1025	227 630	
1025	234 84	
1025	245 67	

1025	250	849
1025	511	288
1025	607	151
1025	614	528
1025	630	242
1025	706	781
1025	727	148
1025	728	201
1025	733	210
1025	868	417
1025	917	150
1025	956	174
1025	959	260
1025	983	228
1025	1109	183
1025	1244	500
1025	1249	563
1025	1351	141
1025	1372	180
1025	1411	96
1025	1412	860
1025	1639	440
1025	1814	157
1025	1981	88
1025	2055	346
1025	2265	103
1025	2342	323
1025	2742	116
1025	3400	164
1025	3470	107

1025	3679	126
1025	4251	116
1025	5845	141
1025	6363	76
1025	6724	693
1025	7426	80
1025	9529	122
1025	11833	243
1025	11834	227
1025	11835	141
1025	11836	129
1026	23	477
1026	25	234
1026	67	471
1026	89	1080
1026	157	230
1026	198	594
1026	298	437
1026	378	269
1026	447	435
1026	517	827
1026	707	323
1026	785	845
1026	806	357
1026	843	1327
1026	854	539
1026	857	418
1026	903	270
1026	949	356
1026	959	311

1026	972 337	
1026	978 423	
1026	1079	403
1026	1104	240
1026	1206	806
1026	1249	244
1026	1281	253
1026	1400	341
1026	1463	222
1026	1513	219
1026	1534	274
1026	1547	1227
1026	1797	649
1026	2033	276
1026	2370	549
1026	2381	285
1026	2744	316
1026	2961	266
1026	2967	254
1026	2970	431
1026	3195	610
1026	3244	488
1026	4272	272
1026	5079	383
1026	5600	263
1026	6490	377
1026	10771	572
1026	11837	947
1026	11838	709
1026	11839	457

1026	11840	232
1027	55	6920
1027	67	1215
1027	89	6458
1027	157	1548
1027	239	1101
1027	241	474
1027	251	3206
1027	257	3260
1027	279	1815
1027	285	971
1027	288	2922
1027	289	3310
1027	292	720
1027	295	5753
1027	301	616
1027	306	583
1027	309	396
1027	314	2841
1027	317	702
1027	320	499
1027	322	551
1027	327	478
1027	331	678
1027	374	1638
1027	423	397
1027	456	483
1027	466	1310
1027	468	1110
1027	484	1166

1027	543	479
1027	601	468
1027	701	888
1027	789	694
1027	913	612
1027	1253	857
1027	1596	592
1027	2083	1679
1027	2094	1134
1027	2102	994
1027	3314	939
1027	3318	1289
1027	3324	1711
1027	3488	545
1027	3490	475
1027	4566	1977
1027	4696	463
1027	6226	806
1027	6562	448
1027	11841	458
1027	11842	408
1028	45	131
1028	89	99
1028	190	1147
1028	209	376
1028	230	521
1028	234	141
1028	297	38
1028	300	70
1028	377	439

1028 378 391

1028 405 39

1028 486 1641

1028 490 50

1028 498 332

1028 499 96

1028 518 474

1028 537 182

1028 540 151

1028 544 128

1028 704 83

1028 830 89

1028 930 251

1028 959 151

1028 966 45

1028 982 123

1028 1116 122

1028 1246 267

1028 1249 55

1028 1691 41

1028 1700 56

1028 1705 53

1028 1908 443

1028 1910 69

1028 2665 62

1028 3124 83

1028 4437 84

1028 4980 134

1028 5215 197

1028 5437 280

1028	5980	371
1028	8292	175
1028	9576	114
1028	9940	82
1028	10931	194
1028	11822	87
1028	11843	111
1028	11844	63
1028	11845	50
1028	11846	48
1028	11847	44
1029	51	330
1029	52	195
1029	59	174
1029	65	246
1029	72	287
1029	98	207
1029	154	195
1029	159	250
1029	163	173
1029	196	270
1029	203	464
1029	206	250
1029	215	209
1029	227	511
1029	245	445
1029	429	209
1029	447	237
1029	533	196
1029	576	199

1029 599 748

1029 601 565

1029 718 185

1029 851 218

1029 857 614

1029 959 1084

1029 985 290

1029 993 361

1029 997 200

1029 998 275

1029 1027 175

1029 1073 896

1029 1089 202

1029 1090 263

1029 1122 745

1029 1375 245

1029 1394 182

1029 1891 178

1029 1892 185

1029 2029 736

1029 2136 247

1029 2490 686

1029 2609 236

1029 3469 327

1029 3789 390

1029 3992 200

1029 4616 177

1029 9321 183

1029 11602 493

1029 11848 245

1029	11849	220
1030	383 493	
1030	785 697	
1030	805 607	
1030	808 768	
1030	809 1277	
1030	814 640	
1030	839 520	
1030	841 749	
1030	842 869	
1030	845 726	
1030	1360	872
1030	1364	504
1030	1406	732
1030	1528	805
1030	1547	723
1030	1975	1258
1030	2160	835
1030	2955	596
1030	3729	1341
1030	3799	500
1030	4346	534
1030	4504	1878
1030	5041	524
1030	5101	933
1030	5104	474
1030	5107	637
1030	5600	597
1030	5666	481
1030	8910	490

1030	8947	771
1030	9805	486
1030	9961	701
1030	10046	513
1030	10573	495
1030	11850	1310
1030	11851	1231
1030	11852	1202
1030	11853	1052
1030	11854	938
1030	11855	859
1030	11856	692
1030	11857	674
1030	11858	664
1030	11859	601
1030	11860	551
1030	11861	525
1030	11862	506
1030	11863	505
1030	11864	467
1030	11865	459
1032	96 26	
1032	192 200	
1032	227 253	
1032	562 61	
1032	565 64	
1032	567 73	
1032	572 191	
1032	576 48	
1032	580 169	

1032	581 63	
1032	583 121	
1032	585 35	
1032	599 31	
1032	728 237	
1032	730 56	
1032	735 83	
1032	868 47	
1032	959 27	
1032	1427	114
1032	1639	38
1032	2786	44
1032	2842	362
1032	2861	148
1032	2864	165
1032	2873	130
1032	2894	87
1032	2901	145
1032	2902	23
1032	3024	55
1032	3778	52
1032	4379	35
1032	5660	100
1032	7056	251
1032	7248	25
1032	8108	196
1032	8110	220
1032	8111	102
1032	8118	33
1032	8122	37

1032	8805	74
1032	9348	24
1032	9352	27
1032	11881	187
1032	11882	34
1032	11883	33
1032	11884	32
1032	11885	31
1032	11886	30
1032	11887	29
1032	11888	24
1033	55	1490
1033	67	33996
1033	89	225
1033	97	331
1033	155	3190
1033	180	748
1033	258	1050
1033	265	143
1033	288	6235
1033	289	247
1033	291	800
1033	292	10413
1033	294	1579
1033	295	155
1033	300	2941
1033	301	283
1033	302	9501
1033	306	1738
1033	310	2665

1033 329 709
1033 333 1294
1033 340 10366
1033 346 709
1033 349 155
1033 466 2837
1033 475 2401
1033 485 1715
1033 690 827
1033 691 106
1033 701 997
1033 959 595
1033 1042 491
1033 1052 224
1033 1379 326
1033 1455 551
1033 1463 2461
1033 1601 145
1033 1805 831
1033 1934 2721
1033 2024 455
1033 2521 175
1033 3491 189
1033 3795 145
1033 4767 630
1033 5000 345
1033 7340 197
1033 7868 106
1033 11452 248
1033 11889 491

1033	11890	115
1034	7	113
1034	191	133
1034	230	104
1034	288	175
1034	292	1668
1034	296	99
1034	300	145
1034	302	250
1034	306	214
1034	310	140
1034	314	128
1034	317	220
1034	326	100
1034	327	270
1034	367	184
1034	377	109
1034	378	139
1034	393	466
1034	400	131
1034	409	96
1034	410	197
1034	456	435
1034	466	200
1034	475	14651
1034	484	200
1034	486	484
1034	545	188
1034	680	632
1034	704	348

1034	706	296
1034	907	452
1034	913	138
1034	1042	94
1034	1451	365
1034	1455	190
1034	1456	124
1034	1613	595
1034	1641	218
1034	1918	174
1034	2020	176
1034	2092	126
1034	2094	2072
1034	2107	133
1034	2247	234
1034	2913	93
1034	3317	159
1034	6618	767
1034	6619	98
1034	6635	145
1034	11891	128
1035	52	15
1035	58	2
1035	59	2
1035	170	2
1035	418	2
1035	429	2
1035	515	9
1035	618	2
1035	632	2

1035	1083	2
1035	1131	3
1035	1510	4
1035	2289	16
1035	2542	6
1035	3201	3
1035	3280	4
1035	3282	3
1035	3290	4
1035	3411	4
1035	3451	10
1035	3884	6
1035	3890	2
1035	4064	2
1035	4138	2
1035	4222	2
1035	5658	12
1035	5932	35
1035	6205	2
1035	6414	2
1035	6582	2
1035	7878	2
1035	8672	3
1035	8680	2
1035	11006	2
1035	11892	1162
1035	11893	18
1035	11894	12
1035	11895	4
1035	11896	3

1035	11897	3
1035	11898	3
1035	11899	3
1035	11900	2
1035	11901	2
1035	11902	2
1035	11903	2
1035	11904	2
1035	11905	2
1035	11906	2
1035	11907	2
1036	59	371
1036	97	655
1036	154	199
1036	190	369
1036	203	849
1036	227	1305
1036	234	234
1036	429	950
1036	430	354
1036	533	363
1036	609	283
1036	615	313
1036	620	249
1036	630	353
1036	707	479
1036	715	294
1036	728	218
1036	735	631
1036	851	437

1036	920 229	
1036	1089	4028
1036	1249	897
1036	1384	1751
1036	1400	241
1036	1414	238
1036	1416	2418
1036	2606	345
1036	3030	176
1036	3676	260
1036	4541	329
1036	6202	257
1036	6215	211
1036	6222	438
1036	6743	746
1036	8656	249
1036	8840	346
1036	9629	270
1036	9823	773
1036	10080	1102
1036	10407	707
1036	11230	4196
1036	11232	1565
1036	11908	1147
1036	11909	580
1036	11910	352
1036	11911	235
1036	11912	229
1036	11913	202
1036	11914	198

1036	11915	187
1037	565 5	
1037	572 4	
1037	580 8	
1037	583 6	
1037	718 4	
1037	728 14	
1037	735 5	
1037	868 3	
1037	975 4	
1037	1412	6
1037	1415	4
1037	1639	11
1037	2175	4
1037	2343	4
1037	2419	3
1037	2786	4
1037	2834	5
1037	2842	9
1037	2864	6
1037	2893	3
1037	2894	10
1037	2896	7
1037	2901	10
1037	4125	4
1037	4251	5
1037	5446	3
1037	5660	4
1037	6363	4
1037	6541	8

1037	6958	7
1037	6959	6
1037	7056	6
1037	8111	10
1037	8120	4
1037	8126	5
1037	8808	4
1037	8821	5
1037	11885	5
1037	11916	7
1037	11917	5
1037	11918	5
1037	11919	4
1037	11920	4
1037	11921	4
1037	11922	4
1037	11923	4
1037	11924	4
1037	11925	4
1037	11926	3
1037	11927	3
1038	51	461
1038	68	190
1038	72	315
1038	82	192
1038	91	293
1038	98	220
1038	100	284
1038	187	277
1038	193	318

1038	877 310	
1038	993 242	
1038	994 164	
1038	995 257	
1038	997 266	
1038	998 326	
1038	999 381	
1038	1004	489
1038	1005	149
1038	1006	204
1038	1013	632
1038	1019	216
1038	1027	320
1038	1062	186
1038	1079	495
1038	1810	168
1038	1883	159
1038	1885	181
1038	1886	190
1038	1891	207
1038	1892	237
1038	1895	273
1038	1897	778
1038	2027	197
1038	2936	329
1038	2937	149
1038	3119	187
1038	3451	207
1038	3768	172
1038	6742	150

1038	7343	146
1038	7885	168
1038	8555	238
1038	9253	172
1038	9255	155
1038	11928	244
1038	11929	223
1038	11930	219
1038	11931	204
1038	11932	196
1038	11933	161
1039	89	1750
1039	157	123
1039	191	83
1039	198	46
1039	288	370
1039	327	376
1039	328	52
1039	329	166
1039	331	451
1039	371	187
1039	377	426
1039	464	147
1039	466	50
1039	468	81
1039	475	1564
1039	498	430
1039	500	233
1039	504	148
1039	517	57

1039	527 461	
1039	547 64	
1039	687 189	
1039	907 96	
1039	978 148	
1039	1037	75
1039	1044	1145
1039	1145	193
1039	1182	70
1039	1197	121
1039	1377	136
1039	1444	123
1039	1455	279
1039	1507	48
1039	1613	50
1039	1672	1364
1039	1863	233
1039	2595	48
1039	2976	156
1039	3084	59
1039	3317	922
1039	4562	161
1039	5947	53
1039	5948	129
1039	5972	107
1039	5988	112
1039	6384	68
1039	6633	50
1039	7340	152
1039	9384	55

1039	11934	48
1040	56	167
1040	209	716
1040	356	200
1040	403	161
1040	405	204
1040	412	233
1040	486	390
1040	492	336
1040	498	245
1040	500	622
1040	538	259
1040	720	667
1040	779	309
1040	808	301
1040	816	238
1040	830	911
1040	923	167
1040	1116	256
1040	1180	665
1040	1185	508
1040	1192	318
1040	1195	186
1040	1246	579
1040	1339	608
1040	1513	165
1040	1672	362
1040	1853	356
1040	1868	226
1040	2346	271

1040	2363	1087
1040	2364	549
1040	2372	1466
1040	2373	292
1040	2723	378
1040	2781	221
1040	2790	169
1040	2797	337
1040	2850	192
1040	2941	480
1040	3032	304
1040	3090	405
1040	4104	269
1040	4164	239
1040	4189	280
1040	4457	439
1040	4459	164
1040	4823	219
1040	5306	197
1040	5638	221
1040	11935	261
1041	7	51
1041	65	64
1041	173	746
1041	190	122
1041	198	196
1041	209	279
1041	220	111
1041	227	579
1041	333	406

1041	377	370
1041	405	257
1041	460	44
1041	461	92
1041	488	63
1041	498	71
1041	707	118
1041	735	53
1041	793	70
1041	917	42
1041	928	77
1041	930	92
1041	942	62
1041	946	77
1041	957	83
1041	959	158
1041	978	88
1041	982	55
1041	1090	148
1041	1104	379
1041	1186	107
1041	1338	650
1041	1348	46
1041	1350	90
1041	1406	44
1041	1796	458
1041	1854	51
1041	1913	140
1041	2836	42
1041	2956	45

1041	3054	50
1041	3833	187
1041	4679	98
1041	5609	84
1041	9263	132
1041	10430	173
1041	11170	70
1041	11936	271
1041	11937	79
1041	11938	66
1041	11939	48
1042	65	320
1042	67	189
1042	89	605
1042	155	280
1042	191	112
1042	228	1679
1042	289	927
1042	291	111
1042	292	102
1042	294	219
1042	295	833
1042	300	451
1042	302	153
1042	304	588
1042	318	83
1042	324	1630
1042	325	152
1042	326	72
1042	328	295

1042 329 3610
1042 333 96
1042 349 1081
1042 403 132
1042 461 70
1042 464 672
1042 465 215
1042 466 1351
1042 475 87
1042 498 128
1042 540 93
1042 686 103
1042 689 704
1042 699 326
1042 701 8248
1042 703 119
1042 907 81
1042 1043 592
1042 1246 339
1042 1455 82
1042 1873 468
1042 1985 760
1042 3739 88
1042 5746 182
1042 6308 245
1042 8372 222
1042 9079 110
1042 11940 395
1042 11941 299
1042 11942 142

1042	11943	123
1044	227 802	
1044	231 214	
1044	440 189	
1044	511 358	
1044	615 1957	
1044	630 495	
1044	718 214	
1044	735 195	
1044	757 194	
1044	964 337	
1044	1202	463
1044	1504	315
1044	1732	271
1044	1878	1076
1044	2281	750
1044	2470	168
1044	2471	434
1044	2475	716
1044	2624	1603
1044	3299	504
1044	3351	474
1044	3430	171
1044	3443	289
1044	4316	734
1044	5299	1111
1044	6098	181
1044	6146	693
1044	7264	547
1044	7265	198

1044	7375	290
1044	8866	520
1044	10812	236
1044	11953	1023
1044	11954	773
1044	11955	695
1044	11956	522
1044	11957	503
1044	11958	479
1044	11959	451
1044	11960	376
1044	11961	344
1044	11962	334
1044	11963	333
1044	11964	258
1044	11965	245
1044	11966	238
1044	11967	214
1044	11968	204
1044	11969	194
1044	11970	189
1046	55	103
1046	67	69
1046	89	1852
1046	257	172
1046	288	615
1046	289	594
1046	292	751
1046	295	288
1046	299	92

1046	300 862	
1046	301 65	
1046	302 207	
1046	306 163	
1046	314 53	
1046	317 164	
1046	325 139	
1046	333 142	
1046	344 146	
1046	349 118	
1046	352 105	
1046	458 392	
1046	461 2961	
1046	464 151	
1046	466 1617	
1046	498 69	
1046	524 465	
1046	528 105	
1046	531 76	
1046	544 88	
1046	679 1368	
1046	681 91	
1046	686 529	
1046	689 63	
1046	701 388	
1046	790 68	
1046	793 207	
1046	797 113	
1046	802 47	
1046	1037	446

1046	1054	88
1046	1452	47
1046	1456	48
1046	1458	69
1046	2020	54
1046	2084	49
1046	2548	111
1046	2984	73
1046	4561	54
1046	4562	79
1046	11976	115
1047	88	93
1047	154	149
1047	195	90
1047	206	146
1047	223	89
1047	540	94
1047	622	159
1047	630	79
1047	763	60
1047	859	155
1047	1046	102
1047	1216	106
1047	1378	176
1047	2166	79
1047	2415	90
1047	2643	93
1047	2817	74
1047	3028	335
1047	3041	127

1047	3616	66
1047	3714	120
1047	3737	133
1047	4189	344
1047	6048	140
1047	6305	78
1047	6834	62
1047	6906	149
1047	9649	201
1047	10309	97
1047	10611	61
1047	11000	110
1047	11413	64
1047	11977	477
1047	11978	180
1047	11979	160
1047	11980	157
1047	11981	153
1047	11982	152
1047	11983	150
1047	11984	124
1047	11985	119
1047	11986	107
1047	11987	97
1047	11988	92
1047	11989	84
1047	11990	80
1047	11991	77
1047	11992	71
1047	11993	70

1047	11994	69
1049	53	73
1049	56	23
1049	59	18
1049	72	51
1049	81	43
1049	154	47
1049	159	26
1049	166	19
1049	172	16
1049	173	252
1049	190	58
1049	196	126
1049	203	17
1049	207	26
1049	226	33
1049	227	17
1049	234	54
1049	425	38
1049	429	52
1049	436	65
1049	439	18
1049	441	455
1049	450	180
1049	599	71
1049	614	24
1049	618	34
1049	714	28
1049	715	23
1049	716	35

1049	718 20	
1049	858 44	
1049	859 17	
1049	860 21	
1049	863 21	
1049	964 49	
1049	1083	31
1049	1101	45
1049	1191	41
1049	1244	19
1049	1424	28
1049	1504	84
1049	1534	28
1049	1632	33
1049	1755	55
1049	1873	20
1049	1990	22
1049	3195	37
1049	6847	31
1049	10524	35
1049	11998	42
1050	53 1060	
1050	154 1083	
1050	199 931	
1050	217 793	
1050	225 1064	
1050	236 1251	
1050	418 719	
1050	425 668	
1050	433 1857	

1050	440 2526	
1050	622 1417	
1050	629 771	
1050	748 1788	
1050	1097	759
1050	1098	634
1050	1504	2003
1050	1888	765
1050	1947	701
1050	1957	1321
1050	1964	2410
1050	1969	798
1050	1983	749
1050	2083	708
1050	2139	836
1050	2281	1245
1050	2402	1031
1050	2416	1471
1050	2573	791
1050	3411	1530
1050	3504	955
1050	3691	774
1050	3694	771
1050	3700	1404
1050	3703	955
1050	3707	843
1050	4079	759
1050	5549	950
1050	6151	2249
1050	6155	994

1050	6217	1431
1050	6910	994
1050	9446	628
1050	11783	712
1050	11999	842
1050	12000	829
1050	12001	779
1050	12002	719
1050	12003	682
1050	12004	654
1050	12005	647
1051	154 857	
1051	174 787	
1051	190 959	
1051	198 1187	
1051	207 682	
1051	209 1004	
1051	220 495	
1051	226 1791	
1051	228 587	
1051	234 425	
1051	422 1042	
1051	424 455	
1051	439 834	
1051	493 398	
1051	512 407	
1051	706 769	
1051	707 813	
1051	710 1460	
1051	854 5199	

1051	917 1139	
1051	918 661	
1051	920 3434	
1051	924 1002	
1051	931 460	
1051	941 524	
1051	946 611	
1051	1041	1722
1051	1045	1418
1051	1099	699
1051	1110	547
1051	1131	2134
1051	1195	1718
1051	1343	1278
1051	1350	1391
1051	1369	437
1051	1412	1384
1051	1672	784
1051	1974	1080
1051	2006	786
1051	2789	485
1051	3745	727
1051	4828	2504
1051	4831	505
1051	5630	6360
1051	5644	590
1051	6121	495
1051	7328	1669
1051	10537	406
1051	10802	628

1051	12006	422
1052	56	136
1052	65	498
1052	107	143
1052	225	220
1052	227	127
1052	228	2150
1052	234	564
1052	407	179
1052	418	279
1052	427	482
1052	436	106
1052	454	500
1052	486	239
1052	501	107
1052	567	125
1052	585	103
1052	703	181
1052	709	568
1052	857	95
1052	1094	252
1052	1151	274
1052	1206	152
1052	1239	102
1052	1376	600
1052	1390	118
1052	1513	1018
1052	1731	115
1052	2611	642
1052	3279	130

1052	3467	106
1052	4155	458
1052	4384	1003
1052	5423	284
1052	5746	159
1052	5925	173
1052	6037	96
1052	6824	135
1052	7324	126
1052	7642	707
1052	8587	101
1052	12007	2089
1052	12008	282
1052	12009	262
1052	12010	205
1052	12011	194
1052	12012	187
1052	12013	134
1052	12014	111
1052	12015	105
1052	12016	90
1053	55	164
1053	56	144
1053	65	221
1053	67	135
1053	89	1040
1053	161	292
1053	199	68
1053	229	476
1053	288	1456

1053	289	15588
1053	292	350
1053	299	93
1053	300	853
1053	302	53
1053	320	72
1053	324	2247
1053	325	108
1053	327	180
1053	329	120
1053	331	448
1053	333	1627
1053	352	108
1053	386	90
1053	461	106
1053	464	423
1053	466	817
1053	468	1408
1053	531	70
1053	533	64
1053	538	1847
1053	679	161
1053	686	52
1053	691	103
1053	982	73
1053	1037	793
1053	1042	164
1053	1409	63
1053	1455	101
1053	1456	398

1053	2130	886
1053	2137	194
1053	2521	655
1053	4562	66
1053	4814	76
1053	5988	97
1053	7868	174
1053	8019	148
1053	10421	160
1053	12017	417
1053	12018	53
1054	103	3813
1054	154	5531
1054	217	2144
1054	237	1161
1054	246	4091
1054	418	10877
1054	444	1728
1054	454	1050
1054	537	1234
1054	548	1305
1054	703	855
1054	981	3436
1054	1155	769
1054	1396	3711
1054	1504	939
1054	1700	1469
1054	1727	4547
1054	1731	4584
1054	1745	1782

1054	1747	3150
1054	1964	1009
1054	2184	1362
1054	2264	1201
1054	2840	1846
1054	3414	1266
1054	4230	1100
1054	4495	3823
1054	4716	3261
1054	6137	2690
1054	6164	807
1054	6217	1489
1054	6594	1588
1054	7502	2053
1054	8027	2361
1054	8752	2819
1054	10517	1954
1054	10613	1986
1054	10719	913
1054	10918	808
1054	11464	1109
1054	11783	934
1054	12005	2331
1054	12019	4078
1054	12020	3314
1054	12021	1759
1054	12022	1153
1054	12023	1040
1054	12024	935
1054	12025	857

1054	12026	767
1055	283 58	
1055	333 105	
1055	371 97	
1055	439 75	
1055	498 150	
1055	499 97	
1055	542 59	
1055	679 161	
1055	1247	121
1055	1470	50
1055	1672	51
1055	1681	127
1055	1809	67
1055	1854	140
1055	1855	59
1055	1857	48
1055	1859	59
1055	2082	61
1055	2352	103
1055	2655	82
1055	2983	68
1055	2986	278
1055	2987	104
1055	3247	62
1055	3376	53
1055	3927	107
1055	3928	77
1055	4177	73
1055	4747	149

1055	4750	52
1055	5427	83
1055	5459	51
1055	6123	61
1055	7249	94
1055	7754	67
1055	8047	47
1055	8724	51
1055	8725	49
1055	8728	357
1055	8732	73
1055	12027	114
1055	12028	75
1055	12029	74
1055	12030	69
1055	12031	65
1055	12032	63
1055	12033	56
1055	12034	51
1055	12035	51
1055	12036	48
1056	65	667
1056	161	283
1056	257	255
1056	288	644
1056	289	1254
1056	292	1585
1056	295	220
1056	300	183
1056	302	334

1056	318 206	
1056	321 324	
1056	333 311	
1056	344 1534	
1056	352 820	
1056	360 159	
1056	455 627	
1056	461 1489	
1056	466 805	
1056	479 595	
1056	498 240	
1056	523 433	
1056	524 564	
1056	544 210	
1056	646 592	
1056	679 3933	
1056	681 633	
1056	686 1869	
1056	687 1325	
1056	701 409	
1056	704 172	
1056	793 147	
1056	802 637	
1056	908 587	
1056	1428	942
1056	1429	191
1056	1430	230
1056	1447	209
1056	1461	1108
1056	1659	246

1056	1672	370
1056	2025	163
1056	2091	1419
1056	2092	135
1056	2523	227
1056	2548	4373
1056	2977	169
1056	3187	312
1056	3393	144
1056	4474	321
1056	6528	222
1057	55	1295
1057	89	1656
1057	189	109
1057	209	365
1057	257	52
1057	261	127
1057	265	106
1057	288	703
1057	289	1146
1057	292	210
1057	295	108
1057	299	144
1057	300	152
1057	301	95
1057	302	195
1057	306	125
1057	310	69
1057	320	67
1057	330	111

1057	333 377	
1057	344 81	
1057	352 291	
1057	360 78	
1057	362 98	
1057	400 84	
1057	466 178	
1057	518 196	
1057	528 51	
1057	543 197	
1057	646 76	
1057	701 1164	
1057	790 146	
1057	792 6938	
1057	802 135	
1057	914 48	
1057	959 216	
1057	1032	113
1057	1244	139
1057	1438	273
1057	1441	117
1057	1459	120
1057	1775	65
1057	2019	75
1057	2082	103
1057	2392	68
1057	2548	64
1057	3081	256
1057	3125	227
1057	12037	262

1057	12038	136
1058	89	414
1058	161	109
1058	288	353
1058	289	695
1058	290	106
1058	292	476
1058	295	251
1058	300	555
1058	302	71
1058	304	94
1058	306	319
1058	308	90
1058	321	239
1058	323	109
1058	324	116
1058	328	223
1058	332	107
1058	344	131
1058	366	89
1058	458	644
1058	461	562
1058	464	91
1058	466	162
1058	484	92
1058	544	356
1058	669	167
1058	679	172
1058	680	189
1058	681	202

1058	685 162	
1058	686 119	
1058	701 93	
1058	793 73	
1058	802 578	
1058	1045	132
1058	1451	101
1058	1453	227
1058	1459	136
1058	1659	209
1058	2080	76
1058	2984	438
1058	3125	88
1058	3377	215
1058	3393	345
1058	4561	97
1058	5749	126
1058	6612	81
1058	7086	94
1058	12039	139
1058	12040	102
1060	162 476	
1060	163 1207	
1060	188 619	
1060	220 529	
1060	226 935	
1060	227 564	
1060	380 401	
1060	418 411	
1060	429 428	

1060	433 949	
1060	605 1428	
1060	632 622	
1060	769 985	
1060	881 536	
1060	964 516	
1060	1110	703
1060	1116	761
1060	1131	1250
1060	1237	435
1060	1404	764
1060	1412	1359
1060	1606	718
1060	1614	416
1060	1632	773
1060	1943	801
1060	1963	579
1060	2542	423
1060	2572	1323
1060	2574	726
1060	3258	494
1060	3346	1789
1060	3707	2822
1060	3732	749
1060	4108	566
1060	5605	502
1060	5966	399
1060	6046	2282
1060	6159	1214
1060	6182	986

1060	6321	454
1060	6336	674
1060	6828	425
1060	7054	590
1060	7900	514
1060	11378	415
1060	12044	1862
1060	12045	516
1060	12046	481
1060	12047	428
1060	12048	406
1061	184 197	
1061	220 591	
1061	447 416	
1061	565 242	
1061	580 272	
1061	581 192	
1061	582 327	
1061	583 292	
1061	728 190	
1061	917 339	
1061	966 502	
1061	1130	190
1061	1816	275
1061	2033	993
1061	2346	233
1061	2842	197
1061	2864	207
1061	2873	142
1061	2894	294

1061	2896	561
1061	3024	211
1061	3892	1094
1061	5660	181
1061	6958	173
1061	6959	217
1061	7056	873
1061	7260	214
1061	7532	1686
1061	7623	1616
1061	8114	174
1061	8123	475
1061	8126	249
1061	8371	644
1061	8805	158
1061	8808	168
1061	10026	228
1061	11885	147
1061	12049	1107
1061	12050	1029
1061	12051	978
1061	12052	717
1061	12053	400
1061	12054	356
1061	12055	241
1061	12056	240
1061	12057	230
1061	12058	217
1061	12059	215
1061	12060	181

1061	12061	159
1062	56	34
1062	57	33
1062	157	54
1062	220	120
1062	235	53
1062	238	33
1062	286	36
1062	567	35
1062	609	35
1062	614	57
1062	843	79
1062	859	34
1062	964	43
1062	1119	55
1062	1427	53
1062	1639	38
1062	1778	40
1062	1957	36
1062	1958	35
1062	2415	35
1062	2532	112
1062	2637	56
1062	3133	39
1062	3164	35
1062	3440	35
1062	3443	33
1062	3445	41
1062	3523	34
1062	3705	39

1062	3778	49
1062	4092	63
1062	4316	53
1062	6556	49
1062	6724	95
1062	6730	37
1062	7407	38
1062	8126	39
1062	10394	34
1062	10663	43
1062	10814	34
1062	10848	62
1062	11506	47
1062	12058	34
1062	12062	117
1062	12063	86
1062	12064	57
1062	12065	48
1062	12066	34
1062	12067	34
1062	12068	34
1063	39	76
1063	173	350
1063	195	1044
1063	210	54
1063	217	147
1063	225	708
1063	229	123
1063	329	49
1063	418	266

1063	428	135
1063	591	84
1063	599	91
1063	604	104
1063	615	143
1063	618	706
1063	930	47
1063	932	49
1063	980	46
1063	1073	141
1063	1098	240
1063	1104	75
1063	1155	111
1063	1167	418
1063	1424	157
1063	1464	127
1063	2277	75
1063	3200	105
1063	3298	96
1063	3333	56
1063	3741	82
1063	3744	161
1063	3902	480
1063	7356	69
1063	7389	54
1063	8584	136
1063	8788	162
1063	10969	103
1063	12026	94
1063	12069	181

1063	12070	143
1063	12071	137
1063	12072	111
1063	12073	87
1063	12074	77
1063	12075	74
1063	12076	67
1063	12077	60
1063	12078	59
1063	12079	57
1063	12080	53
1064	96	12
1064	163	97
1064	192	25
1064	234	15
1064	316	8
1064	378	18
1064	706	81
1064	707	142
1064	710	38
1064	717	36
1064	724	95
1064	730	16
1064	732	12
1064	868	18
1064	917	80
1064	918	11
1064	930	27
1064	956	27
1064	959	49

1064	982 7	
1064	1110	28
1064	1160	12
1064	1249	18
1064	1358	81
1064	1369	113
1064	1372	12
1064	1412	11
1064	1425	155
1064	1468	18
1064	1803	9
1064	2342	43
1064	2343	177
1064	2346	32
1064	2463	16
1064	2569	15
1064	2787	7
1064	2861	7
1064	2936	8
1064	3445	10
1064	3470	20
1064	3888	8
1064	4130	62
1064	5812	28
1064	8340	9
1064	8423	8
1064	8555	52
1064	8794	9
1064	12081	35
1064	12082	27

1064	12083	8
1065	58	5
1065	72	5
1065	89	662
1065	157	17
1065	257	53
1065	288	33
1065	289	30
1065	291	17
1065	292	165
1065	294	12
1065	295	107
1065	298	6
1065	300	28
1065	301	65
1065	302	5
1065	306	28
1065	311	20
1065	328	12
1065	329	9
1065	333	13
1065	344	22
1065	349	28
1065	377	6
1065	378	45
1065	403	8
1065	499	7
1065	506	6
1065	518	15
1065	525	11

1065	903	32
1065	1009	11
1065	1098	6
1065	1123	5
1065	1388	10
1065	1491	6
1065	1609	7
1065	1797	5
1065	2309	7472
1065	2317	16
1065	2498	7
1065	2646	6
1065	3502	7
1065	6930	5
1065	12084	47
1065	12085	17
1065	12086	16
1065	12087	10
1065	12088	6
1065	12089	6
1065	12090	6
1067	30	368
1067	59	390
1067	89	422
1067	154	386
1067	159	3480
1067	163	2309
1067	207	387
1067	227	3131
1067	250	690

1067	306	710
1067	331	445
1067	412	724
1067	424	904
1067	429	453
1067	441	383
1067	470	420
1067	714	541
1067	779	712
1067	875	540
1067	903	769
1067	1083	498
1067	1513	825
1067	1639	391
1067	1645	881
1067	1654	1408
1067	2080	602
1067	2637	390
1067	2938	371
1067	3430	371
1067	3444	450
1067	3456	585
1067	4087	425
1067	4385	555
1067	4459	382
1067	4791	947
1067	4800	929
1067	4821	833
1067	4825	659
1067	4831	647

1067	4834	833
1067	5416	748
1067	5630	438
1067	5639	795
1067	6120	718
1067	7330	478
1067	8713	427
1067	10124	560
1067	11276	495
1067	12092	549
1067	12093	473
1068	7	103
1068	159	329
1068	198	133
1068	209	930
1068	220	498
1068	230	1378
1068	234	525
1068	302	79
1068	383	324
1068	405	229
1068	412	3166
1068	475	202
1068	489	77
1068	497	458
1068	534	343
1068	540	509
1068	605	842
1068	614	132
1068	709	122

1068	808 155	
1068	840 131	
1068	857 1261	
1068	1090	255
1068	1186	268
1068	1246	83
1068	1406	4339
1068	1513	587
1068	1645	2254
1068	1650	4043
1068	1814	756
1068	2738	408
1068	3333	293
1068	3442	77
1068	3444	91
1068	3456	1111
1068	3734	87
1068	4194	176
1068	4218	89
1068	4820	119
1068	4821	183
1068	4822	111
1068	4833	82
1068	8945	566
1068	10126	386
1068	10127	180
1068	10753	102
1068	12094	344
1068	12095	179
1068	12096	89

1068	12097	77
1069	55	5070
1069	67	5502
1069	89	2009
1069	220	141
1069	283	203
1069	288	1226
1069	289	31600
1069	292	397
1069	295	1158
1069	299	1690
1069	300	621
1069	309	322
1069	310	403
1069	326	159
1069	328	156
1069	349	2625
1069	456	274
1069	525	306
1069	542	323
1069	701	4856
1069	708	3683
1069	1009	138
1069	1045	2694
1069	1241	312
1069	1432	358
1069	1436	360
1069	1681	282
1069	2080	451
1069	2440	1273

1069	2462	520
1069	2983	274
1069	3074	2252
1069	3173	242
1069	3198	668
1069	3376	381
1069	4716	1626
1069	4850	210
1069	5249	136
1069	6120	264
1069	6792	131
1069	7727	593
1069	8371	865
1069	8380	333
1069	11139	212
1069	12098	407
1069	12099	178
1069	12100	153
1069	12101	141
1069	12102	135
1069	12103	131
1070	65	300
1070	191	116
1070	207	275
1070	209	106
1070	227	98
1070	228	201
1070	229	1038
1070	230	450
1070	300	154

1070	333	323
1070	403	103
1070	412	1165
1070	486	91
1070	488	191
1070	497	425
1070	498	354
1070	501	156
1070	528	85
1070	533	279
1070	687	303
1070	691	145
1070	808	113
1070	816	421
1070	1040	96
1070	1043	124
1070	1045	119
1070	1186	716
1070	1192	175
1070	1195	228
1070	1672	179
1070	1689	259
1070	2130	438
1070	2176	170
1070	2608	117
1070	2661	170
1070	3333	357
1070	3398	87
1070	3735	153
1070	3747	108

1070	5630	131
1070	5979	109
1070	6132	100
1070	6605	191
1070	7328	190
1070	7835	100
1070	10798	114
1070	12104	340
1070	12105	106
1070	12106	91
1070	12107	86
1071	55	1056
1071	65	543
1071	67	1177
1071	89	3143
1071	161	251
1071	190	122
1071	228	255
1071	257	1238
1071	288	2355
1071	289	4421
1071	291	520
1071	292	20436
1071	293	156
1071	294	386
1071	295	1670
1071	298	367
1071	299	271
1071	300	1793
1071	301	218

1071	302	1126
1071	306	3738
1071	310	451
1071	311	190
1071	318	178
1071	320	1571
1071	325	213
1071	328	810
1071	329	155
1071	330	165
1071	333	790
1071	349	1232
1071	378	205
1071	386	114
1071	412	166
1071	461	276
1071	464	152
1071	466	680
1071	498	697
1071	525	166
1071	544	620
1071	546	145
1071	701	1017
1071	903	342
1071	972	535
1071	1034	158
1071	1042	211
1071	1062	154
1071	1449	114
1071	1613	145

1071	9766	116
1072	67	196
1072	89	523
1072	172	175
1072	257	316
1072	283	223
1072	288	574
1072	289	270
1072	291	855
1072	292	19165
1072	295	1039
1072	296	171
1072	298	381
1072	300	1459
1072	301	286
1072	302	744
1072	306	275
1072	307	417
1072	316	214
1072	325	540
1072	333	2645
1072	340	303
1072	342	231
1072	344	4454
1072	349	182
1072	360	251
1072	461	2515
1072	498	209
1072	542	514
1072	546	170

1072	680 1049	
1072	701 1308	
1072	708 242	
1072	802 173	
1072	890 192	
1072	903 447	
1072	907 185	
1072	1052	230
1072	1459	193
1072	2523	211
1072	2801	268
1072	2984	291
1072	3173	328
1072	3176	199
1072	3205	305
1072	3332	274
1072	3931	186
1072	5774	218
1072	12108	434
1072	12109	384
1072	12110	227
1073	154 605	
1073	206 563	
1073	212 667	
1073	223 266	
1073	227 1753	
1073	245 291	
1073	511 259	
1073	578 1450	
1073	580 352	

1073	585	283
1073	610	1011
1073	613	811
1073	617	252
1073	717	402
1073	718	419
1073	734	1347
1073	735	396
1073	1244	248
1073	1412	425
1073	1426	546
1073	1427	622
1073	1833	350
1073	2292	247
1073	3776	255
1073	5660	302
1073	8119	1736
1073	8126	498
1073	8311	293
1073	8837	285
1073	9433	551
1073	10891	249
1073	11477	721
1073	11652	420
1073	12111	1510
1073	12112	942
1073	12113	928
1073	12114	657
1073	12115	647
1073	12116	574

1073	12117	553
1073	12118	522
1073	12119	520
1073	12120	463
1073	12121	451
1073	12122	423
1073	12123	368
1073	12124	301
1073	12125	279
1073	12126	251
1073	12127	250
1074	51	1971
1074	67	323
1074	72	325
1074	77	125
1074	95	99
1074	96	93
1074	157	192
1074	159	533
1074	163	162
1074	180	739
1074	187	144
1074	193	92
1074	227	297
1074	229	259
1074	258	106
1074	429	451
1074	437	94
1074	511	102
1074	533	108

1074	599	1649
1074	601	150
1074	716	131
1074	735	397
1074	851	302
1074	875	613
1074	959	124
1074	982	143
1074	1079	100
1074	1122	88
1074	1414	285
1074	1416	1033
1074	1534	144
1074	1767	201
1074	1795	120
1074	1832	166
1074	1892	228
1074	2861	374
1074	2938	158
1074	3074	922
1074	3201	91
1074	3441	159
1074	3451	123
1074	3739	114
1074	4369	273
1074	5836	149
1074	6709	142
1074	6790	164
1074	9160	262
1074	12128	124

1074	12129	96
1075	53	737
1075	64	1474
1075	65	1383
1075	70	3460
1075	81	761
1075	89	1104
1075	154	2662
1075	155	747
1075	159	1108
1075	173	2047
1075	182	1101
1075	204	1419
1075	207	861
1075	228	1034
1075	229	891
1075	423	1027
1075	485	888
1075	526	1223
1075	618	2047
1075	703	2416
1075	715	1429
1075	757	3471
1075	1001	979
1075	1075	825
1075	1097	1327
1075	1098	4415
1075	1151	732
1075	1155	925
1075	1167	1424

1075	1400	1678
1075	1409	963
1075	1424	1014
1075	1481	1414
1075	1485	966
1075	1983	2960
1075	1990	771
1075	2521	1212
1075	2531	2928
1075	3057	827
1075	3200	804
1075	3206	1036
1075	3300	1207
1075	3738	882
1075	3902	1383
1075	4728	735
1075	5149	1152
1075	7324	978
1075	7885	967
1075	9877	1298
1075	12130	1190
1076	249 105	
1076	267 63	
1076	271 131	
1076	331 119	
1076	355 55	
1076	384 101	
1076	538 81	
1076	610 268	
1076	613 123	

1076	617 59	
1076	619 84	
1076	683 50	
1076	986 116	
1076	1553	70
1076	1627	84
1076	1976	110
1076	2176	71
1076	2247	56
1076	2842	55
1076	2923	174
1076	2931	72
1076	3004	76
1076	3171	83
1076	3710	60
1076	4984	105
1076	4987	59
1076	5191	147
1076	6389	86
1076	7056	110
1076	8071	67
1076	9912	87
1076	10399	117
1076	11652	50
1076	11655	141
1076	11682	95
1076	11753	75
1076	12131	257
1076	12132	193
1076	12133	171

1076	12134	138
1076	12135	133
1076	12136	102
1076	12137	98
1076	12138	70
1076	12139	66
1076	12140	63
1076	12141	63
1076	12142	60
1076	12143	56
1076	12144	52
1078	163 39	
1078	706 96	
1078	724 68	
1078	868 55	
1078	917 100	
1078	924 47	
1078	928 56	
1078	940 52	
1078	951 69	
1078	952 94	
1078	1119	119
1078	1249	113
1078	1360	61
1078	1369	67
1078	1412	76
1078	1415	51
1078	1803	104
1078	1808	115
1078	1914	63

1078	2346	53
1078	2558	48
1078	2784	69
1078	2787	108
1078	2797	76
1078	3474	101
1078	4165	72
1078	4460	94
1078	5010	44
1078	5011	61
1078	5157	39
1078	5165	158
1078	6493	51
1078	8423	89
1078	9819	53
1078	9822	40
1078	12149	127
1078	12150	76
1078	12151	76
1078	12152	56
1078	12153	55
1078	12154	55
1078	12155	52
1078	12156	51
1078	12157	43
1078	12158	42
1078	12159	40
1078	12160	39
1078	12161	39
1078	12162	38

1078	12163	38
1079	172	1753
1079	209	754
1079	221	680
1079	227	1294
1079	383	2605
1079	487	1218
1079	498	946
1079	504	4499
1079	537	730
1079	615	611
1079	810	641
1079	851	854
1079	898	4604
1079	930	605
1079	1062	2603
1079	1231	3732
1079	1249	1151
1079	1298	770
1079	1389	1593
1079	1632	1543
1079	1672	11830
1079	1718	1782
1079	1807	996
1079	1863	4871
1079	2062	990
1079	2083	689
1079	3086	748
1079	3186	798
1079	3328	1286

1079	3455	1050
1079	3734	1461
1079	4058	1654
1079	4069	709
1079	4189	2023
1079	4262	1144
1079	4384	850
1079	5100	1091
1079	5926	1260
1079	6214	616
1079	6235	761
1079	6453	1298
1079	6685	1159
1079	9747	655
1079	9960	1211
1079	10401	832
1079	10859	628
1079	12164	2305
1079	12165	988
1079	12166	757
1079	12167	664
1080	67	633
1080	89	185
1080	157	33
1080	176	33
1080	227	228
1080	257	303
1080	288	951
1080	289	2142
1080	292	1018

1080	295	1060
1080	300	414
1080	301	565
1080	306	58
1080	316	32
1080	318	75
1080	325	1417
1080	349	122
1080	377	35
1080	439	99
1080	461	1144
1080	498	469
1080	543	63
1080	635	1533
1080	646	1134
1080	681	1258
1080	683	126
1080	686	98
1080	701	232
1080	705	60
1080	790	51
1080	793	28
1080	908	68
1080	1047	263
1080	1241	97
1080	1246	55
1080	1428	8014
1080	1429	68
1080	1438	23
1080	1673	27

1080	1771	24
1080	1934	47
1080	2984	25
1080	3173	101
1080	3378	57
1080	3741	43
1080	3763	28
1080	4260	23
1080	4471	29
1080	4709	74
1080	6472	32
1081	190 1260	
1081	207 325	
1081	209 204	
1081	225 495	
1081	227 93	
1081	228 70	
1081	229 9	
1081	234 9	
1081	424 98	
1081	470 262	
1081	533 35	
1081	689 33	
1081	708 82	
1081	709 24	
1081	1000	21
1081	1048	40
1081	1090	128
1081	1106	75
1081	1116	65

1081	1375	11
1081	1400	3
1081	1511	14
1081	1512	23
1081	1513	71
1081	1515	158
1081	1516	51
1081	1669	9
1081	2137	65
1081	2524	1093
1081	2821	9
1081	3770	8
1081	3771	11
1081	5429	73
1081	7091	13
1081	7199	51
1081	7822	4143
1081	12168	606
1081	12169	218
1081	12170	88
1081	12171	38
1081	12172	24
1081	12173	13
1081	12174	12
1081	12175	6
1081	12176	6
1081	12177	6
1081	12178	4
1081	12179	4
1081	12180	3

1081	12181	3
1082	89	495
1082	205	292
1082	207	269
1082	217	219
1082	227	223
1082	271	392
1082	288	341
1082	374	1559
1082	425	417
1082	437	950
1082	446	449
1082	618	225
1082	679	302
1082	1109	452
1082	1121	714
1082	1390	731
1082	1857	426
1082	1919	523
1082	1922	510
1082	1929	511
1082	1934	349
1082	2000	427
1082	2083	274
1082	2086	1034
1082	2094	695
1082	2277	290
1082	2610	405
1082	3057	401
1082	3324	924

1082	3484	385
1082	3488	234
1082	4923	311
1082	5089	2489
1082	5412	237
1082	5605	1010
1082	7170	407
1082	8408	607
1082	9108	276
1082	9567	252
1082	10229	315
1082	10231	273
1082	12182	1674
1082	12183	1018
1082	12184	590
1082	12185	379
1082	12186	312
1082	12187	305
1082	12188	271
1082	12189	269
1082	12190	258
1083	65	289
1083	182	225
1083	184	765
1083	204	398
1083	209	968
1083	227	370
1083	229	217
1083	230	178
1083	291	227

1083	316	315
1083	333	412
1083	344	337
1083	376	237
1083	378	168
1083	396	278
1083	403	862
1083	481	286
1083	498	272
1083	533	243
1083	536	278
1083	540	376
1083	891	333
1083	898	501
1083	899	206
1083	961	236
1083	965	247
1083	1375	264
1083	1377	307
1083	1470	458
1083	1505	154
1083	1857	244
1083	1872	280
1083	1922	589
1083	2427	228
1083	2544	848
1083	3373	169
1083	3398	281
1083	3466	226
1083	3489	449

1083	4177	196
1083	4618	554
1083	5592	173
1083	6454	335
1083	7175	176
1083	10583	163
1083	10677	280
1083	12191	559
1083	12192	285
1083	12193	246
1083	12194	162
1084	56	1854
1084	64	1309
1084	65	537
1084	72	598
1084	81	503
1084	88	435
1084	154	853
1084	163	658
1084	170	2881
1084	173	755
1084	190	1007
1084	199	779
1084	207	586
1084	225	1192
1084	418	996
1084	423	350
1084	425	375
1084	429	371
1084	432	354

1084	441	428
1084	539	302
1084	620	403
1084	718	323
1084	757	463
1084	959	380
1084	969	506
1084	1062	470
1084	1073	1824
1084	1090	755
1084	1097	1542
1084	1098	527
1084	1109	717
1084	1390	371
1084	1394	600
1084	1398	692
1084	1400	325
1084	1983	1183
1084	1990	684
1084	2708	357
1084	3252	937
1084	3259	848
1084	3767	445
1084	4025	594
1084	4247	310
1084	4728	336
1084	4922	331
1084	7743	600
1084	8136	402
1084	12195	441

1084	12196	301
1085	195 382	
1085	215 410	
1085	418 2848	
1085	444 2187	
1085	445 468	
1085	602 502	
1085	604 546	
1085	621 414	
1085	622 749	
1085	632 1113	
1085	763 1411	
1085	1423	1230
1085	1717	537
1085	1731	377
1085	1747	460
1085	2276	389
1085	2285	698
1085	2572	1318
1085	2573	1677
1085	2575	758
1085	2608	1277
1085	3411	469
1085	4532	1324
1085	6159	485
1085	6345	389
1085	6756	541
1085	6759	423
1085	6761	985
1085	6906	3456

1085	7382	577
1085	8264	1148
1085	8461	393
1085	8467	1327
1085	8866	2113
1085	9666	1021
1085	11396	421
1085	11591	455
1085	11900	498
1085	11968	606
1085	12197	1348
1085	12198	1177
1085	12199	932
1085	12200	784
1085	12201	633
1085	12202	566
1085	12203	486
1085	12204	485
1085	12205	381
1085	12206	371
1085	12207	358
1086	192 119	
1086	212 133	
1086	227 1326	
1086	307 289	
1086	316 145	
1086	321 129	
1086	333 148	
1086	554 655	
1086	679 165902	

1086	680 382	
1086	683 15608	
1086	694 27058	
1086	728 1002	
1086	730 303	
1086	1105	128
1086	1242	755
1086	1244	118
1086	1383	167
1086	1415	127
1086	2265	174
1086	2444	213
1086	2456	479
1086	2629	305
1086	3097	126
1086	3110	118
1086	3375	378
1086	3748	355
1086	3749	515
1086	4557	5257
1086	4780	370
1086	5662	120
1086	6299	549
1086	6600	254
1086	6710	160
1086	7286	406
1086	8313	200
1086	9084	439
1086	9417	118
1086	9428	129

1086	10677	197
1086	12208	2658
1086	12209	1479
1086	12210	1449
1086	12211	1281
1086	12212	953
1086	12213	616
1086	12214	433
1086	12215	389
1086	12216	317
1086	12217	206
1087	72	153
1087	159	741
1087	207	237
1087	208	321
1087	210	458
1087	225	980
1087	227	1029
1087	228	137
1087	424	445
1087	428	1097
1087	429	1755
1087	432	136
1087	441	1677
1087	443	763
1087	562	284
1087	599	397
1087	612	176
1087	735	124
1087	863	575

1087	1048	222
1087	1090	141
1087	1376	129
1087	1390	140
1087	1412	137
1087	1517	113
1087	1811	161
1087	1888	363
1087	2531	127
1087	2591	428
1087	3371	134
1087	3797	166
1087	4129	289
1087	4369	152
1087	4541	507
1087	4649	186
1087	5088	112
1087	5429	578
1087	5785	150
1087	7076	393
1087	7179	211
1087	7663	159
1087	10105	248
1087	12218	717
1087	12219	466
1087	12220	455
1087	12221	338
1087	12222	279
1087	12223	155
1087	12224	139

1087 12225 121

1088 191 635

1088 217 532

1088 223 847

1088 230 1311

1088 333 1544

1088 403 398

1088 412 1254

1088 438 733

1088 470 1158

1088 481 1414

1088 498 3824

1088 499 725

1088 500 411

1088 507 424

1088 537 1082

1088 714 519

1088 716 978

1088 814 467

1088 816 2882

1088 898 640

1088 1041 10673

1088 1043 1062

1088 1045 5389

1088 1046 634

1088 1180 1066

1088 1185 401

1088 1195 395

1088 1200 2844

1088 1201 387

1088	1246	737
1088	1672	492
1088	1868	981
1088	2080	2736
1088	2430	534
1088	2608	1417
1088	2618	1482
1088	2847	1287
1088	3384	513
1088	3392	632
1088	4346	435
1088	4823	646
1088	4828	2303
1088	4831	1483
1088	4840	511
1088	4850	489
1088	5749	1267
1088	6121	570
1088	6132	575
1088	6536	1384
1088	12226	596
1089	9	114
1089	11	148
1089	13	134
1089	30	816
1089	31	197
1089	32	518
1089	49	188
1089	51	109
1089	59	198

1089	72	2130
1089	81	265
1089	153	318
1089	154	190
1089	159	418
1089	187	114
1089	233	288
1089	515	807
1089	599	128
1089	718	142
1089	858	320
1089	878	145
1089	961	686
1089	986	118
1089	1001	406
1089	1014	332
1089	1069	241
1089	1203	385
1089	1205	326
1089	1225	137
1089	1294	249
1089	1295	513
1089	1296	151
1089	1298	144
1089	1312	183
1089	1534	189
1089	1707	445
1089	1713	151
1089	1886	283
1089	1983	215

1089	2041	277
1089	2638	154
1089	3441	148
1089	3901	109
1089	4138	150
1089	4871	156
1089	4959	557
1089	5581	171
1089	10628	109
1089	12227	189
1089	12228	109
1090	2244	5
1090	4344	2
1090	5353	15
1090	9837	16
1090	9838	31
1090	11074	12
1090	12229	25
1090	12230	24
1090	12231	24
1090	12232	23
1090	12233	22
1090	12234	21
1090	12235	20
1090	12236	20
1090	12237	18
1090	12238	18
1090	12239	17
1090	12240	17
1090	12241	11

1090	12242	11
1090	12243	10
1090	12244	9
1090	12245	7
1090	12246	7
1090	12247	7
1090	12248	6
1090	12249	6
1090	12250	6
1090	12251	5
1090	12252	5
1090	12253	4
1090	12254	4
1090	12255	3
1090	12256	3
1090	12257	3
1090	12258	3
1090	12259	3
1090	12260	3
1090	12261	3
1090	12262	3
1090	12263	3
1090	12264	3
1090	12265	2
1090	12266	2
1090	12267	2
1090	12268	2
1090	12269	2
1090	12270	2
1090	12271	2

1090	12272	2
1091	173 742	
1091	180 690	
1091	210 363	
1091	291 370	
1091	302 378	
1091	307 507	
1091	310 549	
1091	371 978	
1091	377 1240	
1091	378 1925	
1091	426 561	
1091	486 564	
1091	488 401	
1091	490 657	
1091	498 1255	
1091	499 11224	
1091	510 2128	
1091	518 1103	
1091	562 380	
1091	898 953	
1091	930 626	
1091	974 404	
1091	1091	1804
1091	1145	373
1091	1470	453
1091	1971	373
1091	2014	659
1091	2661	3709
1091	2775	2728

1091	3427	702
1091	3557	507
1091	3757	1296
1091	3859	587
1091	4189	488
1091	4921	372
1091	5202	953
1091	5972	432
1091	6134	999
1091	7898	571
1091	8047	2100
1091	11729	853
1091	11796	428
1091	11806	707
1091	12273	1553
1091	12274	1328
1091	12275	1207
1091	12276	1132
1091	12277	626
1091	12278	514
1091	12279	377
1092	55	1005
1092	56	842
1092	64	1402
1092	65	874
1092	67	2955
1092	71	2201
1092	89	2828
1092	257	1549
1092	288	1652

1092	289 3216	
1092	291 856	
1092	292 819	
1092	295 914	
1092	298 771	
1092	300 1975	
1092	301 1248	
1092	302 1676	
1092	306 1120	
1092	311 662	
1092	317 925	
1092	374 9311	
1092	375 2808	
1092	485 763	
1092	679 784	
1092	701 899	
1092	1052	998
1092	1383	3200
1092	1857	1190
1092	1922	1068
1092	1934	897
1092	2086	2617
1092	2094	1779
1092	2101	2268
1092	2102	1095
1092	2111	873
1092	2521	2260
1092	2523	862
1092	2546	955
1092	2808	791

1092	3081	1255
1092	3324	6551
1092	3490	2300
1092	5093	1660
1092	5150	1013
1092	9567	1546
1092	9569	1031
1092	10226	890
1092	12280	829
1092	12281	768
1092	12282	669
1093	15	62
1093	65	65
1093	163	245
1093	189	70
1093	229	62
1093	503	116
1093	518	93
1093	718	75
1093	821	200
1093	824	285
1093	837	93
1093	883	112
1093	930	170
1093	946	289
1093	957	94
1093	959	81
1093	1104	102
1093	1106	94
1093	1113	204

1093	1124	97
1093	1177	60
1093	1338	86
1093	1339	326
1093	1340	61
1093	1710	130
1093	2012	67
1093	2013	63
1093	2055	235
1093	2068	105
1093	2359	147
1093	2446	78
1093	2746	233
1093	3197	63
1093	3660	176
1093	4069	61
1093	4285	75
1093	4608	67
1093	6258	293
1093	7100	138
1093	7922	83
1093	8420	190
1093	8741	62
1093	8962	82
1093	10585	137
1093	12283	124
1093	12284	83
1093	12285	78
1093	12286	68
1093	12287	65

1093	12288	62
1094	65	512
1094	154	540
1094	166	1569
1094	171	560
1094	182	2534
1094	210	227
1094	217	219
1094	220	385
1094	221	443
1094	225	3816
1094	227	792
1094	228	289
1094	418	788
1094	451	1932
1094	511	320725
1094	562	557
1094	599	221
1094	715	11248
1094	735	248
1094	851	337
1094	993	372
1094	1122	2085
1094	1151	2478
1094	1375	749
1094	1377	276
1094	1385	537
1094	1413	571
1094	1639	218
1094	1875	8582

1094	2077	1182
1094	2137	1286
1094	3302	529
1094	4444	262
1094	5099	1862
1094	5890	726
1094	6678	567
1094	6789	2787
1094	7404	227
1094	8479	226
1094	10197	474
1094	10515	395
1094	10910	296
1094	12289	1810
1094	12290	571
1094	12291	534
1094	12292	465
1094	12293	349
1094	12294	264
1094	12295	260
1094	12296	243
1097	58	2062
1097	67	3407
1097	71	1515
1097	89	1924
1097	97	698
1097	178	3474
1097	180	1403
1097	182	547
1097	288	896

1097 289 675

1097 294 1092

1097 295 832

1097 300 789

1097 342 1794

1097 344 549

1097 359 1362

1097 362 946

1097 423 2398

1097 518 1733

1097 644 552

1097 689 748

1097 697 1465

1097 722 1044

1097 903 572

1097 914 690

1097 972 667

1097 1098 31199

1097 1114 788

1097 1167 855

1097 1375 659

1097 1377 925

1097 1459 1486

1097 1463 582

1097 1470 707

1097 1934 1523

1097 2018 4054

1097 2044 1239

1097 3081 1948

1097 3200 643

1097	3495	951
1097	5032	688
1097	7137	711
1097	7157	1021
1097	7324	601
1097	7727	673
1097	7810	1350
1097	9234	636
1097	12309	710
1097	12310	600
1097	12311	585
1098	53	27
1098	54	44
1098	57	48
1098	58	70
1098	72	19
1098	81	26
1098	93	21
1098	233	33
1098	302	47
1098	316	18
1098	511	21
1098	544	18
1098	603	58
1098	614	20
1098	689	51
1098	756	20
1098	854	21
1098	903	17
1098	961	26

1098	1062	18
1098	1066	51
1098	1208	30
1098	1231	27
1098	1239	40
1098	1244	49
1098	1383	57
1098	1772	29
1098	1782	26
1098	1824	68
1098	2146	28
1098	2277	32
1098	3081	21
1098	3100	20
1098	3109	19
1098	3110	21
1098	3417	65
1098	3430	31
1098	3464	19
1098	4113	141
1098	4117	81
1098	4134	28
1098	5197	33
1098	6762	25
1098	7352	55
1098	9144	22
1098	10521	46
1098	12312	50
1098	12313	37
1098	12314	34

1098	12315	17
1099	56	28
1099	58	237
1099	59	38
1099	61	57
1099	72	1996
1099	154	22
1099	159	23
1099	163	42
1099	173	94
1099	180	22
1099	182	39
1099	183	110
1099	187	37
1099	190	76
1099	192	59
1099	227	36
1099	229	64
1099	298	24
1099	302	43
1099	425	29
1099	511	149
1099	533	24
1099	539	33
1099	690	43
1099	706	71
1099	709	23
1099	712	67
1099	715	29
1099	716	141

1099	998	26
1099	1001	77
1099	1014	47
1099	1052	33
1099	1131	148
1099	1243	23
1099	1244	40
1099	1713	292
1099	1790	35
1099	2027	37
1099	2031	24
1099	2901	25
1099	3158	30
1099	3308	24
1099	3992	46
1099	4982	39
1099	8069	152
1099	9745	67
1099	9926	114
1099	10017	67
1099	12316	25
1100	45	209
1100	81	179
1100	154	359
1100	206	471
1100	233	206
1100	235	452
1100	238	161
1100	615	381
1100	845	157

1100	859 189	
1100	1119	344
1100	1131	171
1100	1202	149
1100	1212	244
1100	1708	503
1100	1994	311
1100	2026	396
1100	2369	182
1100	2472	197
1100	2473	502
1100	2475	151
1100	2493	1660
1100	2624	332
1100	2964	940
1100	3110	171
1100	3417	257
1100	4081	528
1100	4655	146
1100	5299	150
1100	6730	168
1100	8866	365
1100	8904	216
1100	8910	172
1100	11958	150
1100	12317	510
1100	12318	393
1100	12319	376
1100	12320	285
1100	12321	261

1100	12322	239
1100	12323	235
1100	12324	235
1100	12325	198
1100	12326	186
1100	12327	176
1100	12328	155
1100	12329	153
1100	12330	149
1100	12331	149
1100	12332	146
1101	152 894	
1101	154 534	
1101	159 747	
1101	206 576	
1101	227 528	
1101	233 757	
1101	418 388	
1101	603 399	
1101	616 789	
1101	744 1051	
1101	748 944	
1101	767 749	
1101	772 435	
1101	1116	721
1101	1203	747
1101	1418	432
1101	1731	469
1101	1745	450
1101	1969	857

1101	2030	813
1101	2152	398
1101	2668	562
1101	2671	412
1101	2673	808
1101	2677	514
1101	2691	433
1101	3732	1317
1101	3995	431
1101	5043	433
1101	5360	807
1101	6160	542
1101	6581	463
1101	7013	863
1101	11019	388
1101	11131	407
1101	11191	424
1101	12333	864
1101	12334	834
1101	12335	710
1101	12336	628
1101	12337	515
1101	12338	493
1101	12339	451
1101	12340	433
1101	12341	432
1101	12342	429
1101	12343	413
1101	12344	410
1101	12345	405

1101	12346	405
1102	53	151
1102	55	4665
1102	58	380
1102	64	147
1102	84	78
1102	89	199
1102	97	165
1102	167	268
1102	229	93
1102	288	88
1102	289	170
1102	298	172
1102	310	481
1102	318	192
1102	328	114
1102	430	221
1102	467	82
1102	525	77
1102	526	147
1102	530	397
1102	532	913
1102	535	258
1102	538	81
1102	546	94
1102	690	73
1102	697	175
1102	701	239
1102	722	248
1102	886	305

1102	906	84
1102	980	281
1102	1097	904
1102	1155	97
1102	1243	284
1102	1394	80
1102	1405	96
1102	1409	628
1102	1486	179
1102	1767	359
1102	2018	93
1102	2020	87
1102	2083	166
1102	2277	143
1102	3193	120
1102	3198	143
1102	4025	193
1102	4028	121
1102	4725	232
1102	4732	104
1102	8589	109
1103	9	33
1103	64	29
1103	66	27
1103	70	36
1103	72	775
1103	75	41
1103	81	129
1103	153	75
1103	166	33

1103	173	43
1103	185	30
1103	191	376
1103	198	44
1103	210	40
1103	228	185
1103	238	29
1103	295	189
1103	306	58
1103	333	28
1103	357	26
1103	359	85
1103	362	31
1103	378	61
1103	599	82
1103	697	121
1103	715	33
1103	1044	40
1103	1075	213
1103	1116	61
1103	1295	209
1103	1296	43
1103	1300	26
1103	1301	44
1103	1704	182
1103	1713	72
1103	2824	34
1103	4316	56
1103	4317	46
1103	4876	38

1103	7514	34
1103	7759	53
1103	7874	86
1103	9869	29
1103	10182	34
1103	10462	38
1103	10622	27
1103	12347	139
1103	12348	66
1103	12349	39
1103	12350	35
1104	55	357
1104	63	254
1104	67	670
1104	77	221
1104	89	136
1104	157	501
1104	180	227
1104	192	239
1104	241	120
1104	251	265
1104	257	627
1104	258	220
1104	261	124
1104	265	367
1104	285	193
1104	289	163
1104	292	521
1104	295	220
1104	340	172

1104 455 156

1104 459 113

1104 479 971

1104 601 741

1104 889 171

1104 913 118

1104 959 323

1104 1000 124

1104 1001 207

1104 1081 193

1104 1098 851

1104 1414 453

1104 1427 544

1104 1464 239

1104 1602 97

1104 1764 478

1104 1820 433

1104 1850 166

1104 2183 370

1104 2346 131

1104 2783 110

1104 3878 162

1104 6376 145

1104 6722 414

1104 6959 129

1104 8422 406

1104 8802 135

1104 12129 141

1104 12351 222

1104 12352 119

1104 12353 95
1106 55 672
1106 65 406
1106 67 8651
1106 89 4749
1106 157 354
1106 191 1751
1106 251 911
1106 265 2062
1106 288 2002
1106 289 6051
1106 290 1881
1106 291 5417
1106 292 987
1106 294 2759
1106 295 1506
1106 298 1762
1106 300 5229
1106 301 2306
1106 302 2286
1106 304 5531
1106 306 547
1106 316 6769
1106 356 501
1106 360 747
1106 364 4456
1106 378 539
1106 455 1226
1106 461 7206
1106 464 4648

1106	466	6969
1106	475	1251
1106	531	786
1106	545	737
1106	679	655
1106	701	5378
1106	892	464
1106	1447	3485
1106	1456	1195
1106	1459	24266
1106	1489	1489
1106	1684	986
1106	1772	648
1106	1934	648
1106	2437	799
1106	2977	2313
1106	3460	4151
1106	3584	1306
1106	5267	1500
1106	12356	7566
1106	12357	393
1107	56	820
1107	209	2808
1107	220	1427
1107	331	639
1107	371	1205
1107	472	1116
1107	475	3371
1107	488	6291
1107	490	1333

1107	493	16522
1107	497	2695
1107	498	1231
1107	500	1722
1107	503	3857
1107	507	4228
1107	536	3310
1107	537	2182
1107	779	6379
1107	806	2075
1107	808	1339
1107	810	1373
1107	811	1313
1107	830	14008
1107	982	1116
1107	1038	1043
1107	1091	1572
1107	1145	1168
1107	1180	8989
1107	1181	1112
1107	1182	2237
1107	1183	3177
1107	1193	1412
1107	1195	1407
1107	1200	2277
1107	1249	1017
1107	1672	24607
1107	1714	1200
1107	1853	555
1107	1854	1094

1107	2350	1559
1107	2940	3003
1107	3082	1750
1107	3563	1021
1107	3859	1859
1107	4103	1268
1107	4741	1465
1107	7639	598
1107	8258	652
1107	9761	5128
1107	10784	421
1108	103 84	
1108	748 111	
1108	1098	179
1108	1135	56
1108	1161	53
1108	1619	77
1108	1640	111
1108	1732	197
1108	1943	62
1108	1965	109
1108	1969	168
1108	1983	59
1108	2672	119
1108	2677	208
1108	2691	81
1108	2811	108
1108	2921	59
1108	3417	108
1108	3534	136

1108	3686	67
1108	4852	93
1108	5411	288
1108	5415	59
1108	6407	135
1108	6408	190
1108	6910	121
1108	9458	237
1108	9585	92
1108	10556	60
1108	11515	70
1108	12334	52
1108	12358	373
1108	12359	142
1108	12360	106
1108	12361	100
1108	12362	97
1108	12363	88
1108	12364	71
1108	12365	68
1108	12366	66
1108	12367	66
1108	12368	64
1108	12369	64
1108	12370	62
1108	12371	60
1108	12372	57
1108	12373	57
1108	12374	55
1108	12375	53

1108	12376	53
1109	55	260
1109	67	323
1109	71	225
1109	76	181
1109	89	319
1109	265	108
1109	285	243
1109	288	1800
1109	289	6236
1109	291	354
1109	292	1403
1109	293	381
1109	294	202
1109	295	695
1109	299	1384
1109	302	267
1109	306	179
1109	310	454
1109	311	328
1109	318	1439
1109	320	435
1109	325	2179
1109	326	192
1109	333	944
1109	349	353
1109	359	627
1109	378	757
1109	405	103
1109	461	105

1109	465 402	
1109	485 197	
1109	523 2845	
1109	525 376	
1109	534 175	
1109	646 598	
1109	701 620	
1109	790 588	
1109	792 167	
1109	802 160	
1109	907 502	
1109	908 602	
1109	913 163	
1109	972 185	
1109	4181	94
1109	4560	189
1109	6376	234
1109	7846	134
1109	12377	108
1109	12378	96
1109	12379	85
1110	188 487	
1110	377 371	
1110	472 254	
1110	489 284	
1110	497 268	
1110	503 219	
1110	507 713	
1110	707 198	
1110	712 305	

1110	730 177	
1110	806 1966	
1110	812 360	
1110	843 876	
1110	846 1843	
1110	918 311	
1110	920 4205	
1110	930 441	
1110	937 473	
1110	943 574	
1110	944 1351	
1110	945 248	
1110	953 348	
1110	1110	2305
1110	1181	227
1110	1186	182
1110	1263	244
1110	1350	306
1110	1406	300
1110	1412	186
1110	1672	419
1110	2014	215
1110	2346	244
1110	2379	1189
1110	2654	830
1110	2661	241
1110	2850	227
1110	2966	407
1110	3779	236
1110	3940	197

1110	4670	1436
1110	4949	342
1110	5585	189
1110	5667	287
1110	6056	423
1110	7148	258
1110	10833	398
1110	12380	588
1110	12381	389
1110	12382	339
1110	12383	266
1111	65	1286
1111	300	1537
1111	470	10122
1111	498	2835
1111	682	6037
1111	683	8758
1111	687	5382
1111	779	2048
1111	816	15903
1111	823	1446
1111	825	2218
1111	1035	7632
1111	1038	2284
1111	1100	6866
1111	1180	9404
1111	1181	1401
1111	1185	2366
1111	1187	6144
1111	1188	5144

1111	1541	12663
1111	1853	4767
1111	1868	12719
1111	2192	3363
1111	2204	5509
1111	2210	11698
1111	2214	7516
1111	2242	9170
1111	2426	7156
1111	2617	3972
1111	2619	7012
1111	2621	1852
1111	2723	1302
1111	2847	4171
1111	2959	2373
1111	4166	1621
1111	4168	2223
1111	4436	2633
1111	4550	7251
1111	5547	1815
1111	5950	1521
1111	6611	1443
1111	6773	3555
1111	7831	2328
1111	8242	2654
1111	11294	4221
1111	11393	2453
1111	12384	7423
1111	12385	2973
1111	12386	2794

1111	12387	2160
1112	152 268	
1112	154 219	
1112	162 534	
1112	210 165	
1112	217 1386	
1112	220 179	
1112	222 189	
1112	233 274	
1112	503 189	
1112	507 316	
1112	512 358	
1112	540 226	
1112	632 467	
1112	755 157	
1112	845 149	
1112	979 183	
1112	1109	166
1112	1378	241
1112	1418	518
1112	1858	578
1112	1929	225
1112	2150	407
1112	2172	273
1112	2573	235
1112	2576	890
1112	2579	333
1112	2580	1148
1112	2594	151
1112	2608	186

1112	2775	154
1112	3616	1145
1112	3705	259
1112	3944	251
1112	4230	164
1112	4262	521
1112	5203	224
1112	5208	233
1112	6463	465
1112	6570	158
1112	6759	142
1112	6773	145
1112	7323	157
1112	10194	190
1112	11228	148
1112	12299	181
1112	12388	1001
1112	12389	497
1112	12390	264
1112	12391	156
1112	12392	149
1113	89	130
1113	239	124
1113	257	125
1113	273	100
1113	279	82
1113	288	279
1113	289	279
1113	292	129
1113	295	84

1113	299	144
1113	301	77
1113	312	96
1113	314	184
1113	317	164
1113	327	96
1113	332	154
1113	349	111
1113	468	145
1113	482	127
1113	484	88
1113	523	138
1113	907	84
1113	913	84
1113	1066	104
1113	1507	107
1113	1544	119
1113	1596	95
1113	3314	80
1113	3317	129
1113	3318	175
1113	4566	136
1113	4617	97
1113	4696	117
1113	6562	129
1113	7340	82
1113	8071	89
1113	8072	150
1113	8081	155
1113	8604	88

1113	10608	224
1113	11063	105
1113	11067	176
1113	11069	167
1113	11080	105
1113	12393	191
1113	12394	147
1113	12395	120
1113	12396	108
1113	12397	100
1113	12398	93
1114	65	364
1114	72	489
1114	154	807
1114	190	7759
1114	199	514
1114	207	976
1114	220	191
1114	226	343
1114	227	265
1114	229	1114
1114	230	329
1114	233	657
1114	300	311
1114	302	371
1114	306	299
1114	320	290
1114	344	252
1114	349	271
1114	366	643

1114	377	269
1114	378	229
1114	416	265
1114	418	245
1114	424	716
1114	436	1568
1114	471	256
1114	481	733
1114	498	3510
1114	499	327
1114	504	614
1114	511	399
1114	533	896
1114	534	205
1114	536	187
1114	679	710
1114	689	1721
1114	982	236
1114	1044	211
1114	1116	391
1114	1239	236
1114	1375	186
1114	1390	249
1114	1400	184
1114	1409	253
1114	2137	198
1114	3057	706
1114	3081	739
1114	4817	217
1114	7532	238

1114	12399	572
1115	51	4
1115	877	1
1115	12400	9
1115	12401	1
1115	12402	1
1116	239	13
1116	247	20
1116	260	24
1116	262	14
1116	269	15
1116	273	22
1116	295	14
1116	317	22
1116	331	14
1116	482	19
1116	1544	15
1116	1821	13
1116	1841	25
1116	2242	14
1116	3317	25
1116	3318	34
1116	4696	21
1116	6384	21
1116	6562	21
1116	8071	15
1116	8081	26
1116	9901	13
1116	9904	23
1116	9905	17

1116	10608	17
1116	10997	15
1116	11062	19
1116	11063	14
1116	11064	16
1116	11066	16
1116	11067	15
1116	11069	29
1116	11070	20
1116	11071	17
1116	11076	18
1116	11077	14
1116	11080	24
1116	11083	13
1116	11685	18
1116	12243	13
1116	12261	16
1116	12268	25
1116	12270	16
1116	12403	18
1116	12404	17
1116	12405	16
1116	12406	15
1116	12407	14
1116	12408	14
1116	12409	13
1118	72	673
1118	81	449
1118	154	1022
1118	173	575

1118	190 11403	
1118	198 486	
1118	220 683	
1118	226 333	
1118	229 410	
1118	233 397	
1118	238 426	
1118	377 515	
1118	425 557	
1118	460 1079	
1118	489 459	
1118	498 315	
1118	500 793	
1118	503 1238	
1118	517 561	
1118	758 2776	
1118	759 404	
1118	768 696	
1118	779 408	
1118	810 296	
1118	814 880	
1118	815 367	
1118	923 1149	
1118	1090	307
1118	1861	298
1118	1873	660
1118	1876	421
1118	1974	386
1118	2148	633
1118	2574	318

1118	2576	454
1118	2699	595
1118	2720	741
1118	2820	715
1118	2944	611
1118	3817	349
1118	4051	461
1118	4173	411
1118	4225	1163
1118	4226	1290
1118	4229	294
1118	4896	912
1118	5111	422
1118	10491	378
1118	12412	524
1118	12413	390
1119	166 10	
1119	184 24	
1119	202 15	
1119	220 13	
1119	230 34	
1119	298 23	
1119	344 64	
1119	498 287	
1119	499 114	
1119	528 13	
1119	683 14	
1119	691 49	
1119	727 7	
1119	819 40	

1119	1455	8
1119	1469	7
1119	1740	15
1119	2338	69
1119	2339	27
1119	2435	15
1119	2619	80
1119	2646	12
1119	2858	11
1119	3057	12
1119	3369	68
1119	4747	56
1119	4920	16
1119	6222	7
1119	7326	10
1119	7835	7
1119	8054	41
1119	10478	10
1119	12414	77
1119	12415	60
1119	12416	43
1119	12417	37
1119	12418	32
1119	12419	24
1119	12420	17
1119	12421	17
1119	12422	12
1119	12423	11
1119	12424	11
1119	12425	10

1119	12426	9
1119	12427	9
1119	12428	8
1119	12429	8
1119	12430	8
1119	12431	7
1120	55	230
1120	67	161
1120	89	53
1120	157	31
1120	257	83
1120	279	33
1120	285	1843
1120	288	891
1120	289	593
1120	292	103
1120	293	48
1120	295	259
1120	296	44
1120	299	179
1120	302	34
1120	306	68
1120	309	41
1120	310	28
1120	311	48
1120	314	91
1120	316	56
1120	318	53
1120	320	32
1120	331	46

1120	332	42
1120	349	44
1120	424	28
1120	455	32
1120	465	46
1120	485	43
1120	523	61
1120	525	43
1120	535	180
1120	701	46
1120	789	27
1120	886	147
1120	906	171
1120	911	26
1120	913	62
1120	972	38
1120	1409	74
1120	1937	37
1120	2083	100
1120	2094	52
1120	2220	28
1120	2523	92
1120	2548	26
1120	3288	29
1120	4566	27
1120	4883	26
1121	378	3
1121	681	15
1121	1226	20
1121	7142	3

1121	8670	5
1121	12432	437
1121	12433	260
1121	12434	244
1121	12435	180
1121	12436	170
1121	12437	115
1121	12438	90
1121	12439	79
1121	12440	66
1121	12441	41
1121	12442	31
1121	12443	31
1121	12444	26
1121	12445	24
1121	12446	18
1121	12447	16
1121	12448	16
1121	12449	11
1121	12450	10
1121	12451	9
1121	12452	8
1121	12453	8
1121	12454	8
1121	12455	8
1121	12456	7
1121	12457	7
1121	12458	7
1121	12459	7
1121	12460	7

1121	12461	6
1121	12462	6
1121	12463	5
1121	12464	5
1121	12465	5
1121	12466	5
1121	12467	5
1121	12468	5
1121	12469	5
1121	12470	4
1121	12471	4
1121	12472	4
1121	12473	4
1121	12474	4
1121	12475	4
1121	12476	4
1122	89	33
1122	288	39
1122	289	16
1122	300	52
1122	314	10
1122	318	11
1122	321	9
1122	324	28
1122	325	10
1122	333	27
1122	344	1449
1122	458	100
1122	461	102
1122	464	27

1122	466	155
1122	498	44
1122	527	24
1122	528	35
1122	531	10
1122	548	22
1122	679	29
1122	680	12
1122	681	82
1122	683	12
1122	686	68
1122	687	24
1122	691	50
1122	699	37
1122	704	305
1122	779	11
1122	793	65
1122	1037	55
1122	1042	18
1122	1043	54
1122	1377	12
1122	2498	9
1122	2841	10
1122	3317	18
1122	4562	36
1122	5035	137
1122	5953	9
1122	6346	10
1122	8649	20
1122	11949	18

1122	12477	40
1122	12478	17
1122	12479	15
1122	12480	13
1122	12481	12
1122	12482	11
1123	217 222	
1123	222 1099	
1123	412 217	
1123	429 275	
1123	440 420	
1123	632 208	
1123	1242	386
1123	1504	1107
1123	1732	217
1123	2267	480
1123	2279	283
1123	2294	716
1123	2415	370
1123	2542	217
1123	2572	800
1123	2585	206
1123	4053	469
1123	4092	579
1123	5549	392
1123	5876	471
1123	6294	258
1123	6906	1777
1123	7374	205
1123	8460	374

1123	8467	399
1123	8469	443
1123	10214	395
1123	10847	702
1123	10848	800
1123	12483	636
1123	12484	544
1123	12485	444
1123	12486	367
1123	12487	359
1123	12488	334
1123	12489	326
1123	12490	322
1123	12491	321
1123	12492	315
1123	12493	314
1123	12494	293
1123	12495	261
1123	12496	242
1123	12497	239
1123	12498	233
1123	12499	232
1123	12500	231
1123	12501	229
1123	12502	224
1123	12503	219
1124	67	2173
1124	89	5497
1124	288	2433
1124	289	4133

1124	292 833
1124	293 6558
1124	295 1326
1124	298 628
1124	299 557
1124	300 667
1124	304 792
1124	310 638
1124	311 801
1124	318 755
1124	325 1671
1124	333 780
1124	344 886
1124	349 748
1124	362 3942
1124	461 4550
1124	466 936
1124	475 1287
1124	498 1172
1124	518 2978
1124	523 725
1124	528 1317
1124	530 800
1124	532 579
1124	542 1211
1124	544 1167
1124	679 2602
1124	686 2789
1124	689 3363
1124	701 3262

1124	790	1058
1124	930	1643
1124	972	848
1124	1032	685
1124	1034	2746
1124	1098	2481
1124	1114	563
1124	1246	964
1124	2025	2798
1124	2083	1514
1124	3125	854
1124	3198	21621
1124	3200	533
1124	3332	984
1124	6430	652
1124	7389	547
1125	17	39
1125	19	81
1125	24	43
1125	27	33
1125	30	58
1125	34	48
1125	36	66
1125	37	58
1125	41	75
1125	49	37
1125	159	107
1125	515	36
1125	864	125
1125	876	60

1125	880 93	
1125	1066	58
1125	1083	122
1125	1203	166
1125	1253	83
1125	1294	43
1125	1295	34
1125	1300	39
1125	1711	39
1125	1886	103
1125	2043	51
1125	2165	40
1125	2667	36
1125	2684	42
1125	2686	52
1125	3244	33
1125	3289	98
1125	3530	38
1125	3551	34
1125	4871	42
1125	4875	52
1125	5670	65
1125	11087	48
1125	12338	50
1125	12504	145
1125	12505	105
1125	12506	78
1125	12507	65
1125	12508	60
1125	12509	56

1125	12510	52
1125	12511	45
1125	12512	42
1125	12513	41
1125	12514	39
1125	12515	33
1126	209 112	
1126	220 74	
1126	230 79	
1126	257 66	
1126	355 65	
1126	412 117	
1126	489 94	
1126	498 86	
1126	500 186	
1126	504 81	
1126	536 82	
1126	614 59	
1126	706 80	
1126	707 62	
1126	709 71	
1126	808 227	
1126	827 100	
1126	840 91	
1126	857 57	
1126	917 219	
1126	923 80	
1126	930 80	
1126	1046	102
1126	1244	127

1126	1339	99
1126	1406	135
1126	1645	64
1126	1672	454
1126	2343	153
1126	3242	73
1126	3919	119
1126	4218	95
1126	6120	59
1126	12516	598
1126	12517	266
1126	12518	247
1126	12519	151
1126	12520	126
1126	12521	126
1126	12522	118
1126	12523	115
1126	12524	108
1126	12525	92
1126	12526	86
1126	12527	75
1126	12528	71
1126	12529	69
1126	12530	68
1126	12531	61
1126	12532	60
1128	89	1681
1128	190	157
1128	217	67
1128	229	71

1128	288	1031
1128	289	9502
1128	291	235
1128	292	816
1128	293	354
1128	298	1871
1128	300	971
1128	302	2462
1128	307	410
1128	333	2595
1128	344	1407
1128	403	114
1128	461	160
1128	466	407
1128	486	1085
1128	498	4692
1128	525	383
1128	534	336
1128	536	634
1128	546	76
1128	680	864
1128	682	142
1128	693	151
1128	704	3803
1128	898	183
1128	989	233
1128	1052	843
1128	1245	108
1128	1375	62
1128	1455	103

1128	1590	322
1128	1609	89
1128	2020	373
1128	2142	74
1128	2435	122
1128	2839	91
1128	3369	710
1128	3399	61
1128	4254	126
1128	5747	162
1128	5781	304
1128	5926	565
1128	5984	198
1128	6456	130
1128	7655	3598
1128	9079	64
1130	53	289
1130	65	96
1130	70	86
1130	72	132
1130	81	120
1130	89	64
1130	154	62
1130	173	876
1130	182	77
1130	190	212
1130	210	73
1130	225	95
1130	228	252
1130	229	131

1130	234	83
1130	238	65
1130	378	120
1130	423	101
1130	429	54
1130	430	102
1130	441	204
1130	450	209
1130	486	251
1130	498	302
1130	697	53
1130	703	205
1130	722	67
1130	969	56
1130	1075	269
1130	1083	73
1130	1090	56
1130	1118	57
1130	1376	98
1130	1394	74
1130	1424	167
1130	1463	114
1130	1632	111
1130	1799	87
1130	2206	94
1130	3890	163
1130	5657	106
1130	6539	91
1130	7818	64
1130	11155	76

1130	12547	1253
1130	12548	176
1130	12549	95
1130	12550	73
1130	12551	71
1130	12552	57
1131	89	139
1131	161	53
1131	288	71
1131	289	130
1131	291	129
1131	292	145
1131	295	38
1131	300	133
1131	306	52
1131	318	85
1131	320	105
1131	321	107
1131	324	45
1131	333	270
1131	455	30
1131	456	40
1131	458	86
1131	461	258
1131	464	162
1131	498	51
1131	523	30
1131	646	48
1131	679	87
1131	681	810

1131	686	108
1131	701	102
1131	787	40
1131	790	225
1131	795	38
1131	797	44
1131	908	56
1131	1054	34
1131	1447	52
1131	1453	289
1131	1475	36
1131	1597	79
1131	4291	31
1131	4480	33
1131	7396	46
1131	8378	49
1131	12038	247
1131	12553	133
1131	12554	71
1131	12555	64
1131	12556	60
1131	12557	54
1131	12558	50
1131	12559	38
1131	12560	30
1131	12561	28
1132	55	1384
1132	65	502
1132	67	10698
1132	89	17827

1132	157 325
1132	257 618
1132	288 3532
1132	289 19348
1132	291 2975
1132	292 5321
1132	293 988
1132	294 658
1132	295 3520
1132	298 522
1132	300 2017
1132	302 2533
1132	306 344
1132	311 498
1132	316 1266
1132	318 373
1132	320 399
1132	324 371
1132	325 2495
1132	333 5306
1132	342 969
1132	344 743
1132	349 976
1132	378 358
1132	430 367
1132	461 1352
1132	464 1124
1132	466 423
1132	472 610
1132	498 1939

1132	528	508
1132	538	583
1132	679	1911
1132	680	461
1132	689	334
1132	701	1619
1132	903	467
1132	908	960
1132	966	287
1132	1042	308
1132	1043	362
1132	1047	366
1132	1450	263
1132	2977	348
1132	3756	295
1132	3859	606
1133	7	232
1133	53	284
1133	56	392
1133	70	132
1133	81	124
1133	88	1456
1133	89	219
1133	154	551
1133	163	382
1133	173	171
1133	190	219
1133	198	396
1133	207	632
1133	212	139

1133	221	202
1133	225	225
1133	226	283
1133	227	650
1133	233	68
1133	246	116
1133	295	306
1133	298	134
1133	418	153
1133	424	3111
1133	432	85
1133	439	880
1133	447	559
1133	599	542
1133	618	549
1133	701	71
1133	718	748
1133	903	106
1133	969	69
1133	1090	183
1133	1098	254
1133	1398	837
1133	1504	233
1133	1681	143
1133	1755	934
1133	1764	79
1133	1766	110
1133	1941	70
1133	2343	67
1133	2600	114

1133	3747	156
1133	4600	157
1133	4716	71
1133	5119	173
1133	8593	70
1133	12562	92
1134	65	33
1134	81	2
1134	154	3
1134	183	5
1134	190	4
1134	191	5
1134	234	2
1134	253	2
1134	300	20
1134	310	3
1134	321	9
1134	333	8
1134	471	12
1134	481	2
1134	497	4
1134	498	3
1134	510	5
1134	533	4
1134	567	5
1134	966	5
1134	1105	6
1134	1519	4
1134	1632	5
1134	1772	6

1134	2051	3
1134	2783	2
1134	3078	7
1134	3375	4
1134	3461	4
1134	3466	4
1134	3489	6
1134	3859	3
1134	3871	3
1134	4432	22
1134	5072	17
1134	6600	52
1134	8071	7
1134	9082	16
1134	10837	11
1134	12563	30
1134	12564	27
1134	12565	9
1134	12566	9
1134	12567	7
1134	12568	6
1134	12569	6
1134	12570	4
1134	12571	3
1134	12572	3
1134	12573	2
1135	55	1007
1135	67	1041
1135	89	114672
1135	157	43

1135	220 37
1135	265 383
1135	288 1879
1135	289 1048
1135	292 2560
1135	293 305
1135	295 1274
1135	296 15
1135	298 26
1135	299 60
1135	300 1073
1135	302 47
1135	306 30
1135	310 30
1135	325 46
1135	333 46
1135	340 165
1135	344 29
1135	461 238
1135	464 61
1135	466 1296
1135	470 401
1135	498 18
1135	524 230
1135	525 1079
1135	542 32
1135	544 2004
1135	646 30
1135	679 101
1135	689 222

1135	701	16
1135	802	189
1135	889	1075
1135	903	133
1135	1032	241
1135	1034	120
1135	1037	31
1135	1045	129
1135	1246	16
1135	1452	23
1135	1868	39
1135	2548	28
1135	4291	75
1135	4560	19
1135	6132	15
1135	12574	351
1136	55	180
1136	56	137
1136	89	878
1136	288	234
1136	289	972
1136	292	258
1136	293	117
1136	300	359
1136	306	128
1136	318	192
1136	323	447
1136	324	234
1136	333	126
1136	344	865

1136	352	133
1136	430	1607
1136	461	277
1136	464	802
1136	466	3092
1136	524	425
1136	526	152
1136	535	245
1136	618	106
1136	679	355
1136	686	263
1136	689	268
1136	690	775
1136	691	216
1136	704	183
1136	779	95
1136	906	230
1136	980	138
1136	1034	121
1136	1037	532
1136	1059	333
1136	1481	226
1136	1503	108
1136	2083	1504
1136	2392	137
1136	2521	465
1136	2543	658
1136	3322	378
1136	3404	840
1136	4814	725

1136	5147	160
1136	5149	252
1136	7706	138
1136	10684	334
1136	11335	103
1136	12575	127
1137	55	237
1137	67	452
1137	71	134
1137	155	115
1137	183	79
1137	241	91
1137	257	264
1137	265	234
1137	285	94
1137	288	226
1137	289	317
1137	291	70
1137	292	203
1137	295	122
1137	299	170
1137	306	118
1137	310	104
1137	316	193
1137	349	125
1137	360	108
1137	463	77
1137	525	97
1137	529	92
1137	535	68

1137	641 89	
1137	701 141	
1137	722 204	
1137	787 121	
1137	966 96	
1137	968 93	
1137	1009	141
1137	1399	84
1137	1505	120
1137	1601	121
1137	2064	79
1137	2390	211
1137	2391	199
1137	2783	126
1137	3063	136
1137	3316	100
1137	3865	78
1137	3873	78
1137	4024	196
1137	5072	129
1137	5249	88
1137	7846	161
1137	9234	80
1137	12576	113
1137	12577	95
1137	12578	81
1138	152 465	
1138	154 2591	
1138	163 2883	
1138	173 1041	

1138 188 655

1138 206 340

1138 215 498

1138 226 609

1138 227 542

1138 233 1070

1138 383 271

1138 418 1166

1138 506 3087

1138 599 539

1138 715 226

1138 733 249

1138 867 331

1138 920 229

1138 1104 449

1138 1110 1159

1138 1134 410

1138 1143 514

1138 1420 451

1138 1425 269

1138 1643 1099

1138 1790 245

1138 2632 781

1138 3308 752

1138 3405 690

1138 3417 1337

1138 3445 261

1138 4143 266

1138 4451 559

1138 5175 1253

1138	6075	210
1138	6730	1220
1138	7123	247
1138	8231	610
1138	8866	356
1138	9228	276
1138	9630	471
1138	10724	261
1138	10740	2129
1138	12201	314
1138	12579	906
1138	12580	530
1138	12581	502
1138	12582	460
1138	12583	443
1138	12584	407
1139	67	486
1139	89	872
1139	161	252
1139	173	3080
1139	288	949
1139	289	15773
1139	292	1232
1139	293	491
1139	295	378
1139	299	434
1139	301	261
1139	333	369
1139	344	370
1139	349	323

1139 374 831

1139 375 3921

1139 377 647

1139 455 355

1139 456 1956

1139 458 2310

1139 459 483

1139 465 441

1139 646 491

1139 701 327

1139 790 590

1139 794 617

1139 972 1573

1139 1023 253

1139 2094 1161

1139 3580 456

1139 6529 1706

1139 6856 371

1139 7545 312

1139 10967 425

1139 12555 1851

1139 12576 425

1139 12585 4493

1139 12586 2154

1139 12587 1554

1139 12588 1288

1139 12589 1167

1139 12590 627

1139 12591 577

1139 12592 427

1139	12593	312
1139	12594	312
1139	12595	297
1139	12596	283
1139	12597	280
1139	12598	246
1140	154 215	
1140	196 160	
1140	199 223	
1140	206 202	
1140	434 203	
1140	440 362	
1140	554 493	
1140	562 229	
1140	599 219	
1140	615 332	
1140	629 179	
1140	703 143	
1140	859 190	
1140	1155	128
1140	1242	299
1140	1414	190
1140	1745	138
1140	1946	423
1140	1957	478
1140	1964	149
1140	2139	282
1140	2289	177
1140	2408	443
1140	2417	244

1140	2422	266
1140	2541	145
1140	2629	178
1140	3686	165
1140	3691	299
1140	3707	242
1140	4079	152
1140	6182	159
1140	6409	270
1140	6824	462
1140	7145	303
1140	8587	180
1140	9206	148
1140	10196	274
1140	11738	298
1140	11742	127
1140	12599	241
1140	12600	232
1140	12601	186
1140	12602	175
1140	12603	153
1140	12604	141
1140	12605	134
1140	12606	132
1140	12607	130
1140	12608	127
1143	89	138
1143	257	79
1143	289	86
1143	291	79

1143	294	180
1143	300	96
1143	304	226
1143	326	61
1143	329	1533
1143	333	68
1143	355	184
1143	356	386
1143	366	84
1143	407	218
1143	455	798
1143	456	242
1143	459	133
1143	462	151
1143	464	238
1143	469	63
1143	479	613
1143	538	61
1143	541	99
1143	699	175
1143	701	57
1143	907	152
1143	1186	110
1143	1467	325
1143	1602	266
1143	2035	118
1143	2071	229
1143	2176	71
1143	2809	77
1143	2977	420

1143	3320	144
1143	3330	168
1143	3375	81
1143	3569	804
1143	3576	150
1143	4920	67
1143	6394	290
1143	7137	58
1143	7340	60
1143	8721	513
1143	11157	93
1143	12621	245
1143	12622	126
1143	12623	79
1143	12624	67
1143	12625	66
1144	212 134	
1144	583 89	
1144	728 316	
1144	2786	73
1144	2834	93
1144	2842	103
1144	2855	82
1144	2864	583
1144	2869	228
1144	2871	91
1144	2873	1142
1144	2894	331
1144	2901	279
1144	3620	73

1144	6541	111
1144	6959	158
1144	7056	96
1144	8111	200
1144	8128	92
1144	9352	116
1144	9926	77
1144	11885	181
1144	11918	94
1144	11923	159
1144	12083	195
1144	12626	507
1144	12627	298
1144	12628	236
1144	12629	224
1144	12630	184
1144	12631	176
1144	12632	168
1144	12633	158
1144	12634	124
1144	12635	119
1144	12636	118
1144	12637	116
1144	12638	110
1144	12639	108
1144	12640	98
1144	12641	94
1144	12642	90
1144	12643	88
1144	12644	84

1144	12645	81
1144	12646	81
1144	12647	78
1144	12648	74
1144	12649	74
1144	12650	73
1145	89	333
1145	288	88
1145	289	136
1145	291	276
1145	292	1151
1145	295	282
1145	298	175
1145	300	502
1145	302	128
1145	321	382
1145	325	353
1145	333	695
1145	342	238
1145	344	5199
1145	456	82
1145	458	4452
1145	461	945
1145	466	317
1145	471	129
1145	481	127
1145	484	71
1145	498	220
1145	528	345
1145	679	858

1145	680	405
1145	681	615
1145	685	126
1145	686	516
1145	693	134
1145	793	249
1145	797	237
1145	908	104
1145	1042	76
1145	1043	176
1145	1047	98
1145	1054	106
1145	1239	88
1145	1447	254
1145	1458	201
1145	1459	456
1145	1659	266
1145	1673	100
1145	1679	113
1145	1691	142
1145	2392	202
1145	2653	259
1145	2801	97
1145	5267	178
1145	5749	97
1145	6454	117
1146	65	247
1146	67	246
1146	81	409
1146	157	278

1146	187 339	
1146	190 1562	
1146	217 252	
1146	223 532	
1146	289 446	
1146	300 272	
1146	307 656	
1146	329 381	
1146	362 220	
1146	378 203165	
1146	436 1516	
1146	455 44119	
1146	469 655	
1146	475 379	
1146	498 2001	
1146	499 2142	
1146	518 483	
1146	542 707	
1146	708 261	
1146	814 274	
1146	907 220	
1146	930 312	
1146	1034	2516
1146	1098	652
1146	1118	425
1146	1375	362
1146	1378	879
1146	1459	317
1146	1672	249
1146	1983	400

1146	2840	260
1146	3081	372
1146	3255	298
1146	3378	576
1146	3568	8141
1146	4474	311
1146	7091	23188
1146	8707	788
1146	10827	2696
1146	12544	326
1146	12651	1045
1146	12652	808
1146	12653	582
1146	12654	346
1146	12655	299
1146	12656	257
1147	15	64
1147	56	78
1147	188	117
1147	198	289
1147	227	44
1147	234	58
1147	377	60
1147	378	76
1147	408	356
1147	503	231
1147	726	217
1147	754	69
1147	812	101
1147	818	98

1147	920 130	
1147	937 102	
1147	943 97	
1147	953 64	
1147	956 44	
1147	957 50	
1147	1044	87
1147	1104	135
1147	1121	65
1147	1339	449
1147	1673	79
1147	1710	82
1147	1854	64
1147	1920	116
1147	1997	84
1147	2006	132
1147	2944	661
1147	2975	71
1147	3217	97
1147	4866	88
1147	5939	79
1147	7101	94
1147	7159	268
1147	9013	65
1147	9828	73
1147	10460	49
1147	12657	142
1147	12658	97
1147	12659	97
1147	12660	92

1147	12661	76
1147	12662	66
1147	12663	58
1147	12664	50
1147	12665	49
1147	12666	43
1148	59	71
1148	72	230
1148	86	69
1148	93	146
1148	154	143
1148	159	82
1148	163	288
1148	172	73
1148	187	98
1148	229	62
1148	230	70
1148	498	75
1148	515	78
1148	717	232
1148	724	67
1148	885	75
1148	930	117
1148	963	109
1148	994	70
1148	998	155
1148	1004	165
1148	1013	114
1148	1073	89
1148	1300	184

1148	1358	88
1148	1372	64
1148	1412	73
1148	1705	75
1148	1707	136
1148	1715	74
1148	1797	73
1148	2075	75
1148	2469	102
1148	2936	67
1148	3264	73
1148	3280	84
1148	3295	76
1148	3302	72
1148	3501	110
1148	3674	73
1148	4126	117
1148	5485	78
1148	6150	288
1148	7068	86
1148	9540	250
1148	12667	101
1148	12668	94
1148	12669	83
1148	12670	66
1148	12671	65
1149	51	15
1149	59	20
1149	72	112
1149	77	12

1149	81	11
1149	159	45
1149	172	12
1149	173	55
1149	193	15
1149	225	22
1149	227	24
1149	429	11
1149	436	16
1149	441	13
1149	599	22
1149	715	18
1149	851	14
1149	866	9
1149	868	20
1149	875	30
1149	879	11
1149	1083	47
1149	1119	9
1149	1121	10
1149	1122	13
1149	1412	15
1149	1464	11
1149	1707	17
1149	1708	8
1149	1726	14
1149	1914	12
1149	1985	13
1149	2469	10
1149	3886	11

1149	4493	13
1149	4982	9
1149	5242	18
1149	6075	9
1149	6823	12
1149	7577	15
1149	9149	12
1149	9632	13
1149	12672	30
1149	12673	19
1149	12674	14
1149	12675	13
1149	12676	11
1149	12677	11
1149	12678	9
1149	12679	9
1150	289 574	
1150	291 1497	
1150	344 4133	
1150	356 1291	
1150	461 1143	
1150	528 777	
1150	681 634	
1150	718 1159	
1150	730 975	
1150	892 684	
1150	906 553	
1150	1459	7499
1150	1489	559
1150	1694	1141

1150	1696	785
1150	2076	691
1150	2436	659
1150	2437	577
1150	2439	1059
1150	2442	2108
1150	2449	633
1150	2455	675
1150	2467	10390
1150	2468	1543
1150	3127	1122
1150	3178	1206
1150	3583	872
1150	3588	1654
1150	3593	726
1150	3609	802
1150	4520	928
1150	4542	1741
1150	4543	1938
1150	5032	2008
1150	5266	1655
1150	5267	3471
1150	5767	626
1150	11236	606
1150	11769	801
1150	12680	2362
1150	12681	1812
1150	12682	859
1150	12683	850
1150	12684	815

1150	12685	792
1150	12686	723
1150	12687	702
1150	12688	668
1150	12689	645
1150	12690	602
1151	65	1235
1151	371	1876
1151	495	124
1151	499	2143
1151	510	1615
1151	511	263
1151	533	518
1151	813	377
1151	967	147
1151	1470	1040
1151	1725	1000
1151	1809	1008
1151	1852	391
1151	1855	521
1151	1859	192
1151	1867	1015
1151	2352	134
1151	2652	635
1151	2661	232
1151	3078	1833
1151	3127	492
1151	3247	255
1151	3927	697
1151	3928	318

1151	4170	574
1151	4177	6312
1151	4361	144
1151	4375	387
1151	4747	399
1151	5972	232
1151	7249	126
1151	7724	468
1151	7780	136
1151	8013	1289
1151	8244	123
1151	9187	140
1151	9279	308
1151	10737	136
1151	12030	121
1151	12691	1802
1151	12692	810
1151	12693	364
1151	12694	313
1151	12695	258
1151	12696	227
1151	12697	175
1151	12698	147
1151	12699	141
1151	12700	140
1151	12701	119
1152	59	132
1152	154	191
1152	212	535
1152	396	458

1152	418 498	
1152	599 108	
1152	615 227	
1152	763 191	
1152	851 169	
1152	1089	1747
1152	1510	161
1152	1806	172
1152	1930	258
1152	3290	150
1152	3884	478
1152	4082	162
1152	4108	181
1152	4230	121
1152	5076	749
1152	5087	377
1152	7175	121
1152	8672	226
1152	8680	756
1152	9633	165
1152	10080	370
1152	10194	113
1152	11230	238
1152	11475	182
1152	11712	121
1152	12702	305
1152	12703	263
1152	12704	237
1152	12705	225
1152	12706	216

1152	12707	212
1152	12708	198
1152	12709	183
1152	12710	172
1152	12711	170
1152	12712	162
1152	12713	162
1152	12714	161
1152	12715	160
1152	12716	159
1152	12717	134
1152	12718	132
1152	12719	124
1152	12720	119
1152	12721	119
1152	12722	112
1153	59	901
1153	65	978
1153	171	1099
1153	173	1307
1153	190	1107
1153	207	1120
1153	212	713
1153	227	1884
1153	228	2386
1153	229	1122
1153	230	857
1153	235	999
1153	475	1089
1153	511	1154

1153 533 928
1153 599 1705
1153 614 2225
1153 630 1610
1153 679 802
1153 706 837
1153 709 1447
1153 715 1002
1153 734 2066
1153 735 1490
1153 969 875
1153 1414 1254
1153 1519 1496
1153 1660 1069
1153 1703 928
1153 1813 2131
1153 2629 873
1153 2633 1056
1153 3071 1050
1153 3201 871
1153 3464 1049
1153 4079 740
1153 4129 1132
1153 4442 801
1153 4444 1084
1153 5890 925
1153 6728 1358
1153 7147 1509
1153 7525 2277
1153 9545 861

1153	9629	1227
1153	9826	813
1153	12723	1764
1153	12724	1389
1153	12725	1028
1153	12726	718
1154	93	741
1154	151	732
1154	175	777
1154	592	524
1154	747	661
1154	782	915
1154	910	512
1154	963	555
1154	1705	912
1154	1793	1180
1154	1800	559
1154	1900	737
1154	2044	531
1154	4123	491
1154	4126	1312
1154	4928	463
1154	4988	613
1154	5256	693
1154	5360	537
1154	5493	465
1154	6060	504
1154	6150	708
1154	6990	670
1154	7029	833

1154	7054	587
1154	7167	457
1154	8802	1190
1154	9146	1429
1154	11544	555
1154	12288	487
1154	12727	859
1154	12728	824
1154	12729	814
1154	12730	770
1154	12731	618
1154	12732	586
1154	12733	582
1154	12734	545
1154	12735	539
1154	12736	531
1154	12737	517
1154	12738	509
1154	12739	500
1154	12740	497
1154	12741	492
1154	12742	481
1154	12743	480
1154	12744	462
1154	12745	460
1154	12746	458
1155	56	133
1155	222	120
1155	249	121
1155	440	218

1155	442 270	
1155	605 143	
1155	618 298	
1155	1117	162
1155	1163	198
1155	1398	137
1155	1627	1293
1155	1721	363
1155	1943	309
1155	1950	143
1155	1954	170
1155	1957	237
1155	1960	123
1155	1963	144
1155	1964	145
1155	2294	198
1155	2407	120
1155	2554	133
1155	2921	339
1155	3280	128
1155	3335	486
1155	3673	196
1155	3695	238
1155	3700	202
1155	3702	395
1155	5048	121
1155	5414	153
1155	7665	120
1155	8927	170
1155	10558	117

1155	12492	603
1155	12618	330
1155	12747	278
1155	12748	273
1155	12749	218
1155	12750	192
1155	12751	177
1155	12752	161
1155	12753	158
1155	12754	146
1155	12755	145
1155	12756	141
1155	12757	134
1155	12758	127
1155	12759	121
1155	12760	120
1156	65	2002
1156	70	367
1156	72	4188
1156	88	580
1156	154	3310
1156	159	2179
1156	162	3175
1156	190	2480
1156	226	1034
1156	229	652
1156	374	899
1156	407	2051
1156	486	758
1156	512	1081

1156	621 825	
1156	632 1136	
1156	717 475	
1156	774 454	
1156	1009	413
1156	1020	500
1156	1306	653
1156	1713	652
1156	1731	1139
1156	1858	3156
1156	2605	386
1156	2775	4669
1156	3334	660
1156	3414	489
1156	4110	909
1156	4177	406
1156	5806	604
1156	5966	538
1156	6046	447
1156	7160	1638
1156	7170	1526
1156	11122	463
1156	11806	926
1156	11825	1844
1156	12761	2120
1156	12762	1132
1156	12763	694
1156	12764	692
1156	12765	611
1156	12766	594

1156	12767	554
1156	12768	456
1156	12769	414
1156	12770	399
1156	12771	396
1156	12772	395
1157	7	308
1157	198	360
1157	357	391
1157	378	278
1157	486	1113
1157	488	1884
1157	490	284
1157	493	919
1157	497	643
1157	499	345
1157	500	892
1157	505	518
1157	514	325
1157	517	703
1157	519	764
1157	537	301
1157	808	484
1157	811	402
1157	815	1195
1157	1179	340
1157	1547	358
1157	1704	429
1157	1711	502
1157	1714	659

1157	1852	357
1157	1854	730
1157	1855	275
1157	1859	1408
1157	1874	421
1157	2206	568
1157	2209	295
1157	2380	279
1157	2940	771
1157	2956	456
1157	3240	410
1157	3245	275
1157	3563	287
1157	7639	376
1157	8258	639
1157	8350	278
1157	8726	548
1157	9765	403
1157	10310	404
1157	11018	318
1157	12538	276
1157	12773	575
1157	12774	438
1157	12775	395
1157	12776	320
1157	12777	281
1158	209 203	
1158	377 548	
1158	498 305	
1158	500 232	

1158	503	505
1158	504	1714
1158	507	315
1158	536	149
1158	538	265
1158	737	165
1158	779	171
1158	804	210
1158	813	242
1158	816	254
1158	818	161
1158	830	223
1158	850	269
1158	1180	377
1158	1186	162
1158	1380	197
1158	1672	221
1158	1852	254
1158	1853	156
1158	1914	265
1158	1931	212
1158	2014	164
1158	2062	558
1158	2618	631
1158	2661	189
1158	2873	153
1158	2959	336
1158	4103	182
1158	4104	145
1158	4162	283

1158	4550	346
1158	5673	170
1158	5926	149
1158	6016	193
1158	6461	268
1158	8462	191
1158	10390	286
1158	10784	146
1158	11006	153
1158	11754	206
1158	12778	569
1158	12779	318
1158	12780	281
1158	12781	164
1158	12782	161
1158	12783	156
1159	65	386
1159	152	2003
1159	163	531
1159	182	298
1159	188	452
1159	230	360
1159	371	169
1159	377	503
1159	503	353
1159	506	179
1159	537	319
1159	687	186
1159	691	247
1159	707	430

1159	808 457	
1159	816 221	
1159	837 767	
1159	843 226	
1159	846 577	
1159	854 272	
1159	917 599	
1159	918 428	
1159	920 1012	
1159	943 174	
1159	947 248	
1159	965 197	
1159	1118	1412
1159	1131	1021
1159	1132	176
1159	1193	1148
1159	1380	523
1159	2002	176
1159	2379	438
1159	2661	215
1159	2971	178
1159	3561	180
1159	3563	244
1159	4177	2113
1159	4428	266
1159	4432	380
1159	4931	195
1159	4993	179
1159	6249	265
1159	6845	191

1159	10069	247
1159	12784	270
1159	12785	249
1159	12786	245
1159	12787	216
1159	12788	170
1160	209	307
1160	220	3185
1160	371	1081
1160	412	2111
1160	498	2919
1160	499	554
1160	500	1674
1160	540	1049
1160	818	665
1160	830	865
1160	1041	322
1160	1045	725
1160	1046	764
1160	1180	2387
1160	1185	270
1160	1192	565
1160	1201	916
1160	1672	1197
1160	1768	1090
1160	1853	1661
1160	1866	518
1160	2430	1467
1160	2576	1266
1160	2840	640

1160	2850	2806
1160	2983	356
1160	3200	294
1160	3333	314
1160	3392	400
1160	4486	274
1160	4828	2042
1160	4831	545
1160	5313	265
1160	5630	2512
1160	6120	2525
1160	6121	401
1160	6123	632
1160	6772	2188
1160	6793	584
1160	6794	303
1160	7835	1636
1160	10496	396
1160	10737	661
1160	10784	563
1160	12789	1647
1160	12790	1518
1160	12791	1431
1160	12792	440
1160	12793	437
1160	12794	379
1161	8	513
1161	89	1342
1161	171	1498
1161	300	320

1161	344	293
1161	376	314
1161	390	427
1161	396	310
1161	397	310
1161	466	323
1161	498	372
1161	1098	401
1161	1429	552
1161	1865	455
1161	1910	798
1161	1915	660
1161	1933	638
1161	2093	473
1161	2096	357
1161	2100	1289
1161	5082	777
1161	5110	1032
1161	6995	666
1161	7002	315
1161	8398	855
1161	8407	1862
1161	10582	838
1161	10807	1016
1161	12795	3546
1161	12796	1881
1161	12797	766
1161	12798	764
1161	12799	740
1161	12800	572

1161	12801	566
1161	12802	520
1161	12803	501
1161	12804	501
1161	12805	468
1161	12806	464
1161	12807	455
1161	12808	430
1161	12809	398
1161	12810	364
1161	12811	321
1161	12812	316
1161	12813	302
1161	12814	301
1161	12815	287
1161	12816	284
1163	30	23
1163	51	19
1163	59	4
1163	61	10
1163	65	110
1163	67	2
1163	72	1091
1163	75	39
1163	81	2
1163	84	16
1163	153	50
1163	154	81
1163	159	28
1163	163	2

1163 173 6

1163 180 3

1163 187 15

1163 190 254

1163 193 6

1163 268 3

1163 511 51

1163 599 91

1163 877 4

1163 880 4

1163 945 3

1163 959 13

1163 998 3

1163 1013 7

1163 1019 2

1163 1055 6

1163 1061 3

1163 1072 46

1163 1073 4

1163 1098 5

1163 1122 2

1163 1381 7

1163 1713 84

1163 1892 5

1163 2901 5

1163 2936 5

1163 3171 25

1163 3768 2

1163 4031 3

1163 4130 12

1163	7324	7
1163	8998	2
1163	9969	3
1163	12817	3
1163	12818	3
1163	12819	3
1164	64	735
1164	72	2873
1164	93	275
1164	173	276
1164	292	666
1164	296	246
1164	298	504
1164	424	589
1164	526	552
1164	627	449
1164	980	374
1164	1061	500
1164	1062	2336
1164	1065	371
1164	1098	373
1164	1099	266
1164	1709	335
1164	2086	1080
1164	2309	781
1164	2470	832
1164	2486	437
1164	2542	355
1164	3186	457
1164	3201	294

1164	3502	455
1164	4032	398
1164	4308	2151
1164	5149	1011
1164	6150	2862
1164	7885	355
1164	8140	295
1164	8390	279
1164	8582	660
1164	9498	655
1164	11331	258
1164	11630	330
1164	11638	316
1164	11783	291
1164	11879	340
1164	12820	3018
1164	12821	682
1164	12822	500
1164	12823	394
1164	12824	366
1164	12825	348
1164	12826	296
1164	12827	255
1164	12828	254
1164	12829	253
1164	12830	248
1165	65	199
1165	72	3157
1165	85	174
1165	154	205

1165	159	930
1165	173	320
1165	190	155
1165	212	269
1165	220	329
1165	227	157
1165	228	161
1165	229	227
1165	230	186
1165	355	168
1165	436	414
1165	511	693
1165	533	176
1165	562	434
1165	614	3591
1165	630	200
1165	683	240
1165	715	232
1165	734	338
1165	735	782
1165	951	196
1165	993	171
1165	1048	158
1165	1079	151
1165	1090	167
1165	1116	197
1165	1249	293
1165	1375	159
1165	1382	219
1165	1489	493

1165	1513	2266
1165	1519	259
1165	1713	252
1165	1778	292
1165	2439	255
1165	3302	204
1165	4179	161
1165	6724	179
1165	6728	189
1165	6926	209
1165	12831	437
1165	12832	266
1165	12833	260
1165	12834	230
1165	12835	190
1165	12836	171
1166	72	220
1166	220	35
1166	768	33
1166	1099	17
1166	1410	43
1166	2153	34
1166	2173	76
1166	2678	8
1166	2813	60
1166	3725	9
1166	3807	13
1166	4181	13
1166	5060	8
1166	5063	26

1166	5071	30
1166	7079	9
1166	7954	9
1166	7955	23
1166	7958	29
1166	7963	19
1166	7981	12
1166	12837	59
1166	12838	56
1166	12839	33
1166	12840	25
1166	12841	22
1166	12842	20
1166	12843	18
1166	12844	17
1166	12845	16
1166	12846	14
1166	12847	14
1166	12848	14
1166	12849	14
1166	12850	13
1166	12851	13
1166	12852	12
1166	12853	12
1166	12854	11
1166	12855	11
1166	12856	11
1166	12857	11
1166	12858	10
1166	12859	10

1166	12860	9
1166	12861	9
1166	12862	9
1166	12863	9
1166	12864	8
1166	12865	8
1167	266 145	
1167	424 95	
1167	604 35	
1167	606 74	
1167	697 83	
1167	747 47	
1167	748 138	
1167	782 646	
1167	857 39	
1167	961 29	
1167	1048	512
1167	1090	90
1167	1520	42
1167	1731	109
1167	2605	235
1167	2638	38
1167	3863	115
1167	6151	38
1167	6914	66
1167	7021	40
1167	7152	169
1167	7180	72
1167	7565	102
1167	8201	139

1167	11207	86
1167	12866	662
1167	12867	114
1167	12868	107
1167	12869	85
1167	12870	71
1167	12871	68
1167	12872	58
1167	12873	57
1167	12874	55
1167	12875	51
1167	12876	45
1167	12877	40
1167	12878	38
1167	12879	36
1167	12880	36
1167	12881	36
1167	12882	35
1167	12883	35
1167	12884	35
1167	12885	34
1167	12886	33
1167	12887	32
1167	12888	32
1167	12889	32
1167	12890	31
1170	89	298
1170	207	1270
1170	227	452
1170	228	263

1170	298 570	
1170	300 371	
1170	366 340	
1170	403 580	
1170	420 200	
1170	424 222	
1170	425 870	
1170	437 365	
1170	498 3899	
1170	546 285	
1170	680 211	
1170	689 369	
1170	690 154	
1170	709 164	
1170	898 166	
1170	980 161	
1170	1043	319
1170	1048	828
1170	1090	252
1170	1109	211
1170	1239	187
1170	1246	354
1170	1390	160
1170	1424	751
1170	1502	201
1170	1515	347
1170	1545	157
1170	1556	150
1170	1632	231
1170	1988	381

1170	2121	348
1170	2661	189
1170	2815	426
1170	3057	275
1170	3200	403
1170	3206	154
1170	3369	155
1170	3400	146
1170	3730	240
1170	3797	167
1170	4773	160
1170	5439	277
1170	7787	353
1170	12917	166
1170	12918	155
1170	12919	154
1171	7	59
1171	55	72
1171	67	126
1171	89	174
1171	157	102
1171	190	59
1171	251	67
1171	257	78
1171	288	183
1171	289	178
1171	292	258
1171	294	158
1171	295	131
1171	299	100

1171	301 74	
1171	306 83	
1171	498 63	
1171	641 171	
1171	701 283	
1171	787 77	
1171	792 60	
1171	1440	93
1171	1475	64
1171	2053	59
1171	2390	693
1171	2395	78
1171	2523	70
1171	3060	75
1171	3063	105
1171	3462	60
1171	4702	238
1171	4817	145
1171	5249	138
1171	6000	63
1171	6505	63
1171	7623	81
1171	7837	87
1171	7846	143
1171	8409	76
1171	9183	100
1171	9777	97
1171	12561	124
1171	12920	324
1171	12921	108

1171	12922	106
1171	12923	90
1171	12924	81
1171	12925	79
1171	12926	67
1171	12927	62
1172	67	250
1172	72	382
1172	163	281
1172	176	440
1172	289	162
1172	300	151
1172	328	191
1172	378	158
1172	506	292
1172	511	145
1172	518	707
1172	712	528
1172	726	321
1172	727	195
1172	903	196
1172	917	640
1172	920	1809
1172	924	435
1172	930	890
1172	936	874
1172	937	155
1172	946	364
1172	955	830
1172	1009	169

1172	1109	177
1172	1114	408
1172	1339	362
1172	1643	175
1172	1729	234
1172	2044	258
1172	2047	205
1172	2067	162
1172	2975	143
1172	4135	275
1172	4319	228
1172	4331	131
1172	4332	187
1172	4341	327
1172	4671	198
1172	4679	134
1172	6077	134
1172	6540	158
1172	7191	317
1172	10865	158
1172	11825	391
1172	12928	353
1172	12929	267
1172	12930	146
1172	12931	146
1172	12932	134
1173	53	1699
1173	59	3287
1173	61	380
1173	72	2091

1173	76	397
1173	84	307
1173	154	523
1173	199	504
1173	233	843
1173	238	995
1173	425	242
1173	429	546
1173	440	540
1173	441	823
1173	511	1222
1173	599	2202
1173	603	411
1173	612	1405
1173	627	1025
1173	630	1046
1173	715	590
1173	748	791
1173	757	235
1173	862	841
1173	879	379
1173	1001	4657
1173	1073	1338
1173	1155	508
1173	1709	2428
1173	1827	325
1173	1881	1514
1173	1964	304
1173	2043	322
1173	2083	782

1173	2136	1431
1173	2139	278
1173	2480	273
1173	2487	488
1173	2535	391
1173	2542	1261
1173	3202	444
1173	3279	663
1173	3280	326
1173	3411	255
1173	5503	240
1173	6075	468
1173	6151	299
1173	6189	229
1173	10847	243
1173	12933	250
1174	706 560	
1174	707 1394	
1174	724 1046	
1174	732 1085	
1174	841 449	
1174	843 460	
1174	917 646	
1174	918 730	
1174	951 634	
1174	952 1296	
1174	954 1066	
1174	1249	1220
1174	1353	440
1174	1358	1162

1174	1366	1197
1174	1369	557
1174	1513	598
1174	1522	1080
1174	1803	2448
1174	1808	523
1174	2343	1517
1174	2363	671
1174	2781	1210
1174	2782	1110
1174	2785	1015
1174	2790	594
1174	2797	543
1174	2838	627
1174	2859	659
1174	3471	449
1174	3474	629
1174	3480	542
1174	4165	1407
1174	4180	493
1174	4460	604
1174	4807	1025
1174	5004	1041
1174	5006	484
1174	5008	450
1174	5165	543
1174	5841	540
1174	7117	684
1174	9460	457
1174	12934	1060

1174	12935	602
1174	12936	532
1174	12937	499
1174	12938	488
1174	12939	472
1174	12940	447
1176	190 776	
1176	198 2827	
1176	209 949	
1176	230 3353	
1176	377 1827	
1176	386 740	
1176	412 528	
1176	466 423	
1176	475 1505	
1176	488 578	
1176	500 3770	
1176	503 2166	
1176	687 739	
1176	707 880	
1176	779 518	
1176	806 423	
1176	808 1240	
1176	809 1263	
1176	810 1808	
1176	813 2438	
1176	815 631	
1176	816 1245	
1176	823 381	
1176	830 456	

1176	834 373	
1176	841 494	
1176	843 655	
1176	923 547	
1176	937 907	
1176	951 407	
1176	978 517	
1176	1044	1280
1176	1104	479
1176	1145	448
1176	1180	1847
1176	1195	383
1176	1196	362
1176	1249	358
1176	1406	924
1176	1410	762
1176	1541	549
1176	1672	5141
1176	1866	687
1176	2379	965
1176	2940	525
1176	4774	483
1176	5100	522
1176	5666	336
1176	12943	446
1176	12944	381
1177	371 668	
1177	510 307	
1177	1470	311
1177	1852	272

1177	1855	795
1177	1859	577
1177	1867	525
1177	2652	281
1177	2661	185
1177	3092	452
1177	3127	293
1177	3927	1584
1177	3928	529
1177	4170	479
1177	4177	537
1177	4361	256
1177	4745	501
1177	4746	179
1177	4750	962
1177	7754	313
1177	7756	643
1177	8244	251
1177	8361	189
1177	8724	183
1177	8727	251
1177	8728	296
1177	8732	178
1177	9187	810
1177	9282	405
1177	9400	643
1177	10306	448
1177	11217	1232
1177	11796	656
1177	12027	232

1177	12034	645
1177	12692	273
1177	12693	276
1177	12945	757
1177	12946	494
1177	12947	478
1177	12948	476
1177	12949	461
1177	12950	348
1177	12951	335
1177	12952	300
1177	12953	258
1177	12954	256
1177	12955	227
1177	12956	208
1177	12957	194
1178	190 313	
1178	207 1165	
1178	289 1324	
1178	291 264	
1178	292 663	
1178	297 232	
1178	300 1741	
1178	316 228	
1178	333 4801	
1178	344 33200	
1178	349 349	
1178	355 500	
1178	366 1241	
1178	424 651	

1178	455 738	
1178	461 462	
1178	466 365	
1178	475 411	
1178	479 300	
1178	533 505	
1178	544 3213	
1178	679 1117	
1178	683 339	
1178	698 367	
1178	951 366	
1178	1043	492
1178	1047	354
1178	1090	420
1178	1406	433
1178	1511	226
1178	1515	242
1178	1766	333
1178	1767	362
1178	1934	1541
1178	2451	463
1178	2498	1252
1178	2523	313
1178	2606	345
1178	3393	607
1178	4474	2167
1178	4828	529
1178	5746	372
1178	6539	303
1178	6865	4854

1178	12958	1034
1178	12959	526
1178	12960	420
1178	12961	344
1178	12962	330
1178	12963	246
1179	65	1510
1179	89	390
1179	97	261
1179	220	814
1179	228	91
1179	288	285
1179	289	1762
1179	291	94
1179	292	206
1179	295	207
1179	297	135
1179	300	2090
1179	306	885
1179	308	99
1179	328	480
1179	430	97
1179	439	253
1179	461	139
1179	464	563
1179	466	987
1179	498	244
1179	530	113
1179	533	1393
1179	538	200

1179	545	87
1179	546	291
1179	683	140
1179	701	89
1179	704	191
1179	1042	934
1179	1241	351
1179	1377	172
1179	1449	145
1179	1456	117
1179	1509	163
1179	1613	110
1179	1681	105
1179	1776	377
1179	2080	89
1179	3171	106
1179	3462	183
1179	4716	93
1179	6528	457
1179	7868	130
1179	8155	330
1179	8161	109
1179	12964	524
1179	12965	139
1179	12966	129
1179	12967	84
1180	65	31
1180	67	126
1180	89	67
1180	157	141

1180	228	119
1180	279	34
1180	288	120
1180	295	40
1180	306	420
1180	310	50
1180	327	49
1180	328	86
1180	329	72
1180	330	41
1180	331	277
1180	468	74
1180	477	50
1180	484	42
1180	527	79
1180	531	37
1180	546	70
1180	903	206
1180	907	75
1180	959	50
1180	969	45
1180	1090	30
1180	1402	82
1180	1409	103
1180	1613	83
1180	2132	50
1180	2179	47
1180	2220	31
1180	2598	36
1180	2907	40

1180	2919	37
1180	3316	30
1180	3730	133
1180	6377	31
1180	6389	41
1180	6902	30
1180	7340	54
1180	8067	45
1180	8173	216
1180	9907	43
1180	11063	32
1180	12968	86
1180	12969	34
1180	12970	33
1180	12971	32
1180	12972	30
1181	416 366	
1181	599 368	
1181	631 422	
1181	748 362	
1181	757 360	
1181	858 818	
1181	861 523	
1181	962 292	
1181	1075	276
1181	1085	485
1181	1164	557
1181	1213	321
1181	1253	383
1181	1886	331

1181	1964	684
1181	2030	799
1181	2139	423
1181	2402	509
1181	2486	629
1181	2542	386
1181	3201	434
1181	3259	510
1181	3280	745
1181	3286	330
1181	3287	339
1181	3290	367
1181	3434	577
1181	3767	340
1181	3884	664
1181	3893	354
1181	4067	275
1181	5050	380
1181	5911	416
1181	6217	393
1181	6410	324
1181	6824	452
1181	6910	355
1181	8150	324
1181	8680	394
1181	10163	281
1181	10385	513
1181	11592	430
1181	12330	348
1181	12495	325

1181	12973	599
1181	12974	450
1181	12975	429
1181	12976	338
1181	12977	308
1181	12978	282
1182	154 1681	
1182	159 674	
1182	162 555	
1182	233 431	
1182	237 196	
1182	266 448	
1182	418 2081	
1182	437 239	
1182	454 140	
1182	512 1487	
1182	601 267	
1182	603 507	
1182	610 318	
1182	616 127	
1182	625 1545	
1182	632 2314	
1182	744 350	
1182	748 7966	
1182	769 1376	
1182	964 186	
1182	981 195	
1182	1098	752
1182	1118	361
1182	1131	448

1182	1176	398
1182	1400	187
1182	1401	346
1182	1418	908
1182	1716	581
1182	1943	727
1182	2174	656
1182	2419	1634
1182	2574	569
1182	2716	358
1182	4024	379
1182	5543	198
1182	5966	137
1182	6048	218
1182	6187	113
1182	6759	625
1182	8459	430
1182	8782	189
1182	8988	213
1182	12026	950
1182	12114	761
1182	12759	118
1182	12979	1588
1182	12980	383
1182	12981	289
1182	12982	129
1183	25	327
1183	234	4833
1183	383	369
1183	403	327

1183	412	766
1183	486	434
1183	492	355
1183	494	768
1183	500	1972
1183	501	349
1183	536	398
1183	537	347
1183	779	1665
1183	813	3149
1183	816	790
1183	818	552
1183	846	1812
1183	1041	890
1183	1044	623
1183	1180	1345
1183	1181	329
1183	1185	861
1183	1196	712
1183	1197	567
1183	1206	408
1183	1274	623
1183	1541	492
1183	1555	2203
1183	1678	498
1183	1853	1629
1183	2080	525
1183	2386	351
1183	2430	1117
1183	2850	21670

1183	2959	592
1183	3032	428
1183	3719	435
1183	3740	710
1183	4218	547
1183	4821	709
1183	4822	585
1183	4823	328
1183	4831	943
1183	4839	585
1183	5066	330
1183	5630	468
1183	5640	568
1183	5641	849
1183	7328	394
1183	12983	321
1184	55	787
1184	58	134
1184	67	3917
1184	89	10939
1184	155	81
1184	167	975
1184	230	69
1184	288	122
1184	289	3277
1184	292	360
1184	295	327
1184	299	71
1184	300	755
1184	302	119

1184	306	108
1184	310	95
1184	321	186
1184	328	553
1184	329	94
1184	331	140
1184	466	128
1184	485	202
1184	525	82
1184	532	213
1184	546	75
1184	701	674
1184	794	103
1184	886	137
1184	889	148
1184	903	434
1184	980	100
1184	1244	515
1184	1464	395
1184	1475	114
1184	2018	442
1184	2858	115
1184	3069	143
1184	3080	108
1184	3310	129
1184	3403	68
1184	3557	106
1184	3948	107
1184	4024	70
1184	5239	172

1184	6527	250
1184	8067	94
1184	12984	180
1184	12985	160
1184	12986	73
1184	12987	72
1185	52	684
1185	55	1321
1185	58	1576
1185	64	294
1185	65	570
1185	67	3756
1185	81	415
1185	97	296
1185	154	592
1185	182	471
1185	199	444
1185	228	283
1185	289	744
1185	295	267
1185	298	742
1185	389	1296
1185	423	420
1185	548	646
1185	689	674
1185	690	811
1185	697	518
1185	886	395
1185	895	447
1185	896	211

1185	900	246
1185	1382	596
1185	1609	304
1185	1632	284
1185	1934	1031
1185	1971	817
1185	2125	311
1185	2137	293
1185	2149	274
1185	2521	800
1185	2544	958
1185	3057	422
1185	3502	990
1185	3878	494
1185	4704	498
1185	7660	289
1185	7781	278
1185	8141	340
1185	11729	354
1185	12988	1725
1185	12989	514
1185	12990	494
1185	12991	461
1185	12992	248
1185	12993	238
1185	12994	212
1186	55	956
1186	67	660
1186	86	683
1186	89	1180

1186	97	466
1186	161	519
1186	257	616
1186	285	427
1186	288	1382
1186	289	2294
1186	290	509
1186	291	732
1186	292	664
1186	294	720
1186	295	814
1186	296	722
1186	298	943
1186	300	833
1186	302	810
1186	310	460
1186	311	523
1186	323	587
1186	349	468
1186	430	429
1186	466	815
1186	485	460
1186	531	590
1186	535	594
1186	544	486
1186	548	1752
1186	679	1713
1186	691	578
1186	701	537
1186	786	412

1186	889	471
1186	901	404
1186	903	677
1186	906	415
1186	915	468
1186	1243	419
1186	1934	978
1186	2521	844
1186	3319	442
1186	3617	668
1186	4252	615
1186	4707	640
1186	5754	414
1186	5767	405
1186	8589	405
1186	11728	414
1187	56	142
1187	88	157
1187	163	136
1187	227	484
1187	434	199
1187	440	175
1187	554	357
1187	608	126
1187	615	153
1187	618	191
1187	629	412
1187	715	162
1187	735	206
1187	863	126

1187	1098	153
1187	1242	555
1187	1416	177
1187	1709	156
1187	1901	147
1187	2265	188
1187	2276	120
1187	2280	340
1187	2289	201
1187	2401	150
1187	3112	159
1187	3208	153
1187	3458	178
1187	3461	120
1187	3469	188
1187	4058	138
1187	5549	229
1187	6409	136
1187	6824	155
1187	8546	128
1187	9825	275
1187	10367	134
1187	11420	236
1187	12002	168
1187	12995	306
1187	12996	273
1187	12997	198
1187	12998	172
1187	12999	169
1187	13000	161

1187	13001	155
1187	13002	137
1187	13003	134
1187	13004	128
1187	13005	127
1187	13006	119
1188	55	2558
1188	64	913
1188	89	3081
1188	184	784
1188	207	807
1188	229	966
1188	288	1643
1188	289	1524
1188	292	1010
1188	295	1199
1188	298	601
1188	300	1873
1188	423	623
1188	432	624
1188	526	2311
1188	530	896
1188	532	2521
1188	548	1826
1188	690	675
1188	697	917
1188	703	801
1188	895	1203
1188	1032	1683
1188	1062	630

1188	1097	636
1188	1098	1213
1188	1106	1702
1188	1398	795
1188	1400	1419
1188	1409	1465
1188	1609	934
1188	1983	1311
1188	2083	613
1188	2521	1685
1188	3110	1157
1188	3128	620
1188	3200	1476
1188	3288	1357
1188	3502	1070
1188	3730	1354
1188	4704	1303
1188	5149	636
1188	5547	1389
1188	6453	1107
1188	6615	667
1188	7071	759
1188	7324	1077
1188	8589	713
1188	13007	1221
1188	13008	1028
1189	55	2000
1189	67	1815
1189	89	1000
1189	230	264

1189	257 375
1189	288 1068
1189	289 4605
1189	292 923
1189	295 185
1189	299 452
1189	301 211
1189	302 276
1189	306 134
1189	310 404
1189	314 97
1189	325 178
1189	328 99
1189	333 605
1189	349 260
1189	352 106
1189	458 129
1189	461 650
1189	466 815
1189	485 277
1189	525 585
1189	531 93
1189	535 300
1189	542 189
1189	543 150
1189	544 230
1189	686 1050
1189	701 541
1189	790 202
1189	797 315

1189	903	184
1189	1032	340
1189	1034	516
1189	1042	151
1189	1047	193
1189	1050	126
1189	1243	111
1189	1451	114
1189	1452	86
1189	2091	125
1189	2521	284
1189	2801	100
1189	2984	265
1189	4707	120
1189	5150	376
1189	7830	101
1190	56	318
1190	70	211
1190	84	608
1190	154	657
1190	199	278
1190	210	307
1190	225	236
1190	231	644
1190	418	480
1190	425	324
1190	432	1233
1190	440	290
1190	441	470
1190	689	253

1190	757 1003	
1190	1090	503
1190	1101	435
1190	1155	543
1190	1396	1391
1190	1400	574
1190	1409	341
1190	1412	225
1190	1964	962
1190	1986	623
1190	2535	394
1190	2542	201
1190	2582	300
1190	2587	729
1190	2715	305
1190	3693	635
1190	3700	231
1190	3730	340
1190	3738	202
1190	3767	446
1190	4022	632
1190	4025	363
1190	4038	457
1190	5547	321
1190	5930	251
1190	6054	312
1190	7324	280
1190	7327	331
1190	7743	489
1190	8215	340

1190	8675	252
1190	9010	210
1190	9011	414
1190	13009	1163
1190	13010	397
1190	13011	370
1191	56	95
1191	67	128
1191	70	97
1191	72	75
1191	163	83
1191	166	119
1191	220	449
1191	227	107
1191	229	74
1191	302	107
1191	306	76
1191	333	72
1191	340	75
1191	366	71
1191	389	68
1191	562	89
1191	601	102
1191	630	110
1191	706	112
1191	732	87
1191	859	87
1191	951	104
1191	985	122
1191	1081	66

1191	1099	85
1191	1150	95
1191	1358	92
1191	1380	74
1191	1394	149
1191	1397	125
1191	1413	72
1191	1639	80
1191	1686	125
1191	1810	70
1191	1836	67
1191	1878	151
1191	1883	131
1191	2346	78
1191	2352	69
1191	2444	66
1191	2792	74
1191	2901	114
1191	4432	115
1191	5072	84
1191	6677	71
1191	11253	77
1191	13012	93
1191	13013	81
1191	13014	79
1191	13015	71
1192	51	618
1192	65	402
1192	81	443
1192	159	759

1192	163	1249
1192	166	757
1192	182	392
1192	203	393
1192	205	462
1192	220	1780
1192	226	546
1192	227	711
1192	234	976
1192	424	350
1192	436	931
1192	453	961
1192	511	531
1192	533	986
1192	610	500
1192	614	8017
1192	706	532
1192	707	666
1192	735	948
1192	854	2056
1192	856	1637
1192	859	503
1192	860	1198
1192	959	1381
1192	982	693
1192	1109	518
1192	1119	349
1192	1249	893
1192	1412	740
1192	1772	805

1192	1778	736
1192	1810	823
1192	1824	401
1192	1873	462
1192	1985	516
1192	2346	1125
1192	2347	406
1192	3310	521
1192	3892	731
1192	4179	947
1192	6724	1263
1192	9344	1404
1192	9346	385
1192	9818	394
1192	10040	456
1192	10537	405
1193	198 823	
1193	227 656	
1193	489 675	
1193	506 830	
1193	1055	740
1193	1369	905
1193	1406	1030
1193	1412	674
1193	1650	630
1193	1814	636
1193	1975	755
1193	1978	3915
1193	1980	626
1193	1981	2385

1193	1984	13534
1193	3237	1410
1193	3238	870
1193	3627	722
1193	3638	954
1193	3640	1134
1193	3646	3118
1193	3648	4613
1193	3650	2614
1193	3664	1493
1193	3679	1365
1193	4156	876
1193	4572	916
1193	4805	1716
1193	5041	635
1193	7038	1103
1193	7040	1472
1193	8286	2558
1193	9932	3606
1193	10390	1730
1193	10897	4443
1193	13016	1287
1193	13017	1257
1193	13018	1174
1193	13019	1103
1193	13020	904
1193	13021	834
1193	13022	833
1193	13023	830
1193	13024	813

1193	13025	805
1193	13026	696
1193	13027	689
1193	13028	629
1193	13029	625
1193	13030	607
1194	56	657
1194	64	783
1194	65	476
1194	67	505
1194	69	852
1194	72	1719
1194	107	3366
1194	154	2114
1194	228	567
1194	238	679
1194	423	602
1194	858	1035
1194	898	1027
1194	910	4043
1194	961	900
1194	1098	577
1194	1109	1016
1194	1116	693
1194	1131	471
1194	1148	2634
1194	1705	1020
1194	1708	2584
1194	1943	464
1194	1971	942

1194	2446	8200
1194	2478	4655
1194	2608	578
1194	2667	1053
1194	2673	1430
1194	3213	1466
1194	4051	922
1194	4734	531
1194	4920	843
1194	4922	693
1194	4923	2782
1194	5698	2421
1194	7152	5989
1194	8670	707
1194	8829	1068
1194	9285	889
1194	9547	471
1194	11309	980
1194	12288	494
1194	13031	1167
1194	13032	697
1194	13033	665
1194	13034	664
1194	13035	648
1194	13036	576
1194	13037	473
1195	154 3641	
1195	232 1995	
1195	416 2204	
1195	1613	2704

1195	1627	3936
1195	1633	2181
1195	1944	8167
1195	1964	7280
1195	2139	6795
1195	2541	2841
1195	2906	4640
1195	3260	6945
1195	3691	3650
1195	3693	5780
1195	3700	10284
1195	4736	6487
1195	6410	28722
1195	7476	2727
1195	7665	2646
1195	8705	6676
1195	8832	3186
1195	9955	2116
1195	10108	5009
1195	10615	5470
1195	11463	5442
1195	11959	2129
1195	11962	3275
1195	11999	9528
1195	12046	7351
1195	12491	2245
1195	13038	10568
1195	13039	7028
1195	13040	6110
1195	13041	5089

1195	13042	4607
1195	13043	4375
1195	13044	3614
1195	13045	3496
1195	13046	3160
1195	13047	2778
1195	13048	2723
1195	13049	2637
1195	13050	2608
1195	13051	2589
1195	13052	2440
1195	13053	2349
1195	13054	2347
1195	13055	2340
1195	13056	2061
1195	13057	1956

1196 159 156
1196 198 2392
1196 220 1040
1196 230 1056
1196 234 40100
1196 378 1045
1196 412 151
1196 453 2303
1196 489 1031
1196 498 1272
1196 510 1513
1196 534 153
1196 542 163
1196 614 1193

1196	704 837	
1196	705 5127	
1196	707 328	
1196	708 187	
1196	854 2858	
1196	856 4093	
1196	860 220	
1196	965 1453	
1196	966 1072	
1196	982 4202	
1196	1116	139
1196	1239	219
1196	1406	300
1196	1415	150
1196	1873	1018
1196	1874	1161
1196	1974	1050
1196	1985	162
1196	2206	241
1196	2655	117
1196	2794	1055
1196	3739	118
1196	3946	4607
1196	4359	316
1196	5169	1745
1196	5453	561
1196	5780	253
1196	6196	118
1196	7407	1000
1196	9261	3730

1196	9344	149
1196	13058	1141
1196	13059	201
1196	13060	156
1196	13061	141
1196	13062	117
1197	55	67
1197	89	264
1197	257	12472
1197	288	206
1197	289	1135
1197	291	271
1197	292	2892
1197	293	307
1197	299	547
1197	320	179
1197	325	744
1197	326	716
1197	333	698
1197	344	763
1197	456	2800
1197	461	3771
1197	465	676
1197	523	767
1197	679	239
1197	681	1586
1197	686	1273
1197	704	120
1197	786	1211
1197	797	130

1197	913	159
1197	1037	192
1197	1246	340
1197	1415	92
1197	1447	97
1197	1458	533
1197	1459	1091
1197	1556	70
1197	1590	900
1197	1683	133
1197	1684	873
1197	1698	331
1197	1934	140
1197	3327	1285
1197	3393	336
1197	3397	1128
1197	3497	82
1197	4254	570
1197	4258	190
1197	4488	1154
1197	4560	420
1197	5974	334
1197	9299	156
1197	10640	159
1197	13063	155
1197	13064	115
1198	55	503
1198	89	2732
1198	207	335
1198	209	182

1198	227 724
1198	288 184
1198	289 753
1198	291 327
1198	292 573
1198	298 192
1198	300 1057
1198	302 320
1198	306 429
1198	324 226
1198	325 166
1198	328 219
1198	333 172
1198	375 523
1198	386 226
1198	400 186
1198	461 197
1198	464 1630
1198	466 884
1198	485 255
1198	498 879
1198	523 173
1198	526 349
1198	528 396
1198	532 242
1198	544 252
1198	548 477
1198	679 709
1198	686 229
1198	689 187

1198	690	179
1198	704	1921
1198	903	460
1198	1034	568
1198	1400	502
1198	2025	183
1198	2083	281
1198	2089	163
1198	2091	167
1198	2092	457
1198	2094	401
1198	2102	528
1198	2137	379
1198	2521	374
1198	3324	158
1198	3404	311
1199	154	837
1199	163	367
1199	190	502
1199	192	881
1199	197	282
1199	202	518
1199	227	3450
1199	434	607
1199	554	1993
1199	572	394
1199	599	342
1199	615	435
1199	717	391
1199	728	489

1199	733 704	
1199	959 416	
1199	1130	312
1199	1158	363
1199	1414	1006
1199	1416	1509
1199	1515	300
1199	1901	467
1199	2424	366
1199	2444	441
1199	2624	1625
1199	2629	953
1199	2632	285
1199	2636	424
1199	2639	283
1199	3003	544
1199	3012	528
1199	3275	304
1199	3777	410
1199	3934	582
1199	4079	758
1199	7056	306
1199	7789	382
1199	8012	531
1199	8311	385
1199	8812	334
1199	11480	320
1199	11926	281
1199	12599	652
1199	13065	711

1199	13066	669
1199	13067	456
1199	13068	437
1199	13069	361
1199	13070	334
1199	13071	331
1200	51	1712
1200	65	300
1200	72	383
1200	88	154
1200	154	157
1200	157	196
1200	159	152
1200	163	199
1200	190	417
1200	203	221
1200	212	319
1200	220	429
1200	227	515
1200	228	173
1200	229	395
1200	380	208
1200	429	187
1200	511	350
1200	533	611
1200	599	185
1200	709	161
1200	711	276
1200	712	411
1200	718	334

1200	728	333
1200	734	307
1200	735	999
1200	851	538
1200	857	167
1200	1089	201
1200	1090	167
1200	1122	232
1200	1153	310
1200	1412	183
1200	1519	151
1200	1772	151
1200	1795	345
1200	2558	275
1200	6724	1435
1200	8995	235
1200	12833	195
1200	12836	491
1200	13072	654
1200	13073	554
1200	13074	528
1200	13075	304
1200	13076	224
1200	13077	170
1200	13078	168
1200	13079	145
1202	51	3261
1202	58	400
1202	59	1017
1202	70	1083

1202	72	2472
1202	77	540
1202	99	490
1202	100	250
1202	159	1299
1202	187	301
1202	193	368
1202	210	359
1202	229	377
1202	233	815
1202	429	408
1202	441	309
1202	511	1151
1202	599	1340
1202	601	564
1202	603	255
1202	612	257
1202	627	1509
1202	715	383
1202	905	299
1202	959	288
1202	982	296
1202	993	543
1202	998	427
1202	1000	273
1202	1001	1950
1202	1014	620
1202	1019	314
1202	1079	328
1202	1151	261

1202 1156 321

1202 1686 308

1202 1810 652

1202 2027 437

1202 2531 524

1202 2554 253

1202 3201 283

1202 3289 450

1202 3466 610

1202 4308 276

1202 4447 279

1202 6742 283

1202 6852 254

1202 13084 393

1202 13085 297

1202 13086 292

1204 154 284

1204 222 183

1204 227 435

1204 249 267

1204 250 219

1204 331 374

1204 567 240

1204 603 1109

1204 616 312

1204 748 361

1204 857 269

1204 1123 321

1204 1242 1177

1204 1620 188

1204	1622	430
1204	1627	516
1204	1645	454
1204	1946	1048
1204	1948	207
1204	1969	359
1204	2136	1131
1204	2146	270
1204	2164	319
1204	2288	358
1204	2673	410
1204	2921	343
1204	3131	205
1204	3145	199
1204	3147	792
1204	3677	241
1204	3778	210
1204	4821	199
1204	5353	215
1204	5434	312
1204	6151	318
1204	7493	268
1204	7527	301
1204	8705	228
1204	10111	327
1204	10374	312
1204	10392	609
1204	10394	232
1204	11204	218
1204	13089	473

1204	13090	382
1204	13091	291
1204	13092	290
1204	13093	254
1204	13094	227
1204	13095	205
1205	2139	1
1205	6206	1
1205	9948	1
1205	11741	1
1205	13096	2
1205	13097	2
1205	13098	2
1205	13099	2
1205	13100	2
1205	13101	2
1205	13102	1
1205	13103	1
1205	13104	1
1205	13105	1
1206	163 175	
1206	227 531	
1206	234 294	
1206	554 238	
1206	607 237	
1206	706 595	
1206	707 244	
1206	724 182	
1206	726 149	
1206	841 170	

1206	868 130	
1206	917 454	
1206	957 246	
1206	959 126	
1206	1202	118
1206	1358	121
1206	1369	1270
1206	1372	2249
1206	1412	625
1206	1639	250
1206	2013	164
1206	2075	168
1206	2265	252
1206	2343	236
1206	2787	211
1206	3302	139
1206	3470	334
1206	3774	1007
1206	5692	333
1206	9529	128
1206	13106	1116
1206	13107	785
1206	13108	596
1206	13109	511
1206	13110	460
1206	13111	437
1206	13112	422
1206	13113	353
1206	13114	318
1206	13115	309

1206	13116	304
1206	13117	277
1206	13118	249
1206	13119	235
1206	13120	211
1206	13121	209
1206	13122	192
1206	13123	187
1206	13124	156
1206	13125	124
1207	157 74	
1207	198 51	
1207	220 141	
1207	412 70	
1207	712 46	
1207	730 72	
1207	917 414	
1207	920 1117	
1207	924 54	
1207	945 112	
1207	963 674	
1207	1110	820
1207	1172	157
1207	1425	42
1207	1468	268
1207	1501	204
1207	1868	38
1207	1998	61
1207	2343	214
1207	2558	952

1207	2565	84
1207	2567	212
1207	2797	43
1207	3481	330
1207	3779	249
1207	3892	75
1207	5444	139
1207	7051	147
1207	8371	1494
1207	8372	65
1207	10717	196
1207	13126	559
1207	13127	369
1207	13128	361
1207	13129	323
1207	13130	188
1207	13131	183
1207	13132	132
1207	13133	122
1207	13134	93
1207	13135	63
1207	13136	58
1207	13137	57
1207	13138	50
1207	13139	47
1207	13140	45
1207	13141	44
1207	13142	41
1207	13143	37
1207	13144	37

1209	51	1073
1209	61	35
1209	68	55
1209	72	77
1209	85	44
1209	91	183
1209	98	79
1209	99	45
1209	183	34
1209	187	210
1209	227	139
1209	429	45
1209	599	64
1209	874	37
1209	905	64
1209	993	53
1209	994	46
1209	1005	40
1209	1013	54
1209	1014	82
1209	1018	44
1209	1073	47
1209	1414	153
1209	1416	60
1209	1899	43
1209	2029	140
1209	2490	45
1209	2556	62
1209	2562	46
1209	2937	42

1209	3201	36
1209	3832	84
1209	3920	40
1209	4616	37
1209	6732	123
1209	6740	40
1209	6784	124
1209	6790	149
1209	7347	40
1209	8995	40
1209	13151	322
1209	13152	132
1209	13153	74
1209	13154	71
1209	13155	63
1209	13156	55
1209	13157	53
1209	13158	47
1209	13159	37
1209	13160	35
1210	51	103150
1210	59	4561
1210	67	5553
1210	72	27229
1210	77	5057
1210	85	4810
1210	98	3505
1210	155	2082
1210	157	1535
1210	159	16739

1210	170	1948
1210	172	2210
1210	187	1777
1210	190	4055
1210	193	3633
1210	227	5005
1210	511	15662
1210	533	1944
1210	599	4628
1210	601	1284
1210	732	1500
1210	874	5444
1210	917	2472
1210	959	3028
1210	993	1400
1210	996	1287
1210	997	2710
1210	998	1615
1210	999	1289
1210	1001	2428
1210	1013	3624
1210	1014	5635
1210	1019	1300
1210	1076	1413
1210	1122	1787
1210	1413	1563
1210	1810	1530
1210	1892	2029
1210	2343	1506
1210	2531	1257

1210	2556	5255
1210	2559	1325
1210	2562	4583
1210	3280	1840
1210	3920	1521
1210	4313	10034
1210	5000	1585
1210	6350	2735
1210	8995	2411
1210	13161	1543
1211	59	176
1211	72	149
1211	96	118
1211	420	153
1211	599	212
1211	874	87
1211	985	126
1211	997	79
1211	1083	182
1211	1504	105
1211	1893	106
1211	2039	223
1211	2830	118
1211	3202	89
1211	3279	133
1211	3280	117
1211	3411	92
1211	3430	184
1211	3451	92
1211	3767	135

1211	3890	83
1211	4308	123
1211	5420	147
1211	5787	88
1211	6780	81
1211	9151	141
1211	9256	92
1211	9501	79
1211	9505	103
1211	9507	113
1211	10165	129
1211	10969	132
1211	11520	133
1211	13162	250
1211	13163	230
1211	13164	211
1211	13165	159
1211	13166	147
1211	13167	133
1211	13168	126
1211	13169	111
1211	13170	105
1211	13171	102
1211	13172	100
1211	13173	95
1211	13174	95
1211	13175	89
1211	13176	88
1211	13177	86
1211	13178	79

1212	56	1162
1212	58	864
1212	65	479
1212	88	293
1212	154	264
1212	163	291
1212	167	282
1212	172	374
1212	182	576
1212	199	517
1212	202	466
1212	206	249
1212	211	548
1212	217	1237
1212	220	2554
1212	222	1396
1212	227	354
1212	229	1692
1212	233	1673
1212	425	297
1212	431	349
1212	436	901
1212	512	345
1212	533	333
1212	614	312
1212	709	526
1212	898	493
1212	959	267
1212	964	744
1212	982	370

1212	1090	272
1212	1159	1303
1212	1398	274
1212	2346	265
1212	2407	1302
1212	2424	751
1212	2497	314
1212	3097	257
1212	3400	289
1212	3427	669
1212	3753	569
1212	4118	2482
1212	4255	325
1212	5798	453
1212	6186	331
1212	8786	494
1212	10554	797
1212	10849	726
1212	13179	387
1212	13180	312
1213	30	21
1213	32	25
1213	72	199
1213	153	449
1213	190	19
1213	238	29
1213	526	35
1213	717	28
1213	1028	20
1213	1062	36

1213	1067	21
1213	1075	23
1213	1084	174
1213	1295	34
1213	1298	24
1213	3988	36
1213	5484	28
1213	7810	24
1213	10720	29
1213	12552	45
1213	13181	268
1213	13182	248
1213	13183	205
1213	13184	85
1213	13185	70
1213	13186	70
1213	13187	46
1213	13188	43
1213	13189	42
1213	13190	42
1213	13191	41
1213	13192	41
1213	13193	37
1213	13194	33
1213	13195	32
1213	13196	32
1213	13197	30
1213	13198	29
1213	13199	29
1213	13200	26

1213	13201	25
1213	13202	25
1213	13203	25
1213	13204	24
1213	13205	24
1213	13206	23
1213	13207	22
1213	13208	21
1213	13209	20
1213	13210	19
1214	370	127
1214	378	330
1214	496	173
1214	499	372
1214	500	295
1214	536	136
1214	706	150
1214	707	167
1214	710	116
1214	726	321
1214	811	110
1214	834	715
1214	835	348
1214	932	606
1214	978	558
1214	1044	271
1214	1131	252
1214	1360	206
1214	1527	212
1214	1672	357

1214	1852	274
1214	1854	390
1214	1870	202
1214	2062	368
1214	2347	145
1214	2380	157
1214	2940	208
1214	2967	111
1214	3089	309
1214	3239	299
1214	4691	155
1214	4825	119
1214	5525	237
1214	5526	302
1214	6120	178
1214	6253	185
1214	6820	146
1214	7113	166
1214	8259	550
1214	8681	195
1214	8881	300
1214	9944	1023
1214	10693	190
1214	11581	209
1214	12780	170
1214	13211	1458
1214	13212	524
1214	13213	271
1214	13214	173
1214	13215	113

1215	65	305
1215	97	251
1215	157	416
1215	166	279
1215	247	371
1215	257	224
1215	300	357
1215	316	252
1215	355	316
1215	360	186
1215	426	212
1215	430	261
1215	466	201
1215	470	822
1215	683	265
1215	697	832
1215	701	442
1215	896	1034
1215	962	1427
1215	1243	376
1215	1400	205
1215	1511	676
1215	1772	706
1215	1934	2047
1215	2433	307
1215	2444	451
1215	2544	799
1215	2608	171
1215	3057	356
1215	3081	4331

1215	3102	217
1215	3488	2475
1215	3749	2284
1215	3763	4636
1215	4252	929
1215	4616	493
1215	4618	825
1215	4710	183
1215	5789	536
1215	6231	186
1215	8977	475
1215	9976	222
1215	11240	217
1215	12227	228
1215	13216	694
1215	13217	247
1215	13218	214
1215	13219	196
1215	13220	184
1215	13221	171
1216	45	186
1216	190	3371
1216	209	994
1216	228	615
1216	229	201
1216	230	158
1216	324	1717
1216	403	696
1216	412	216
1216	429	239

1216	486 8802	
1216	488 230	
1216	493 1184	
1216	494 310	
1216	498 458	
1216	536 162	
1216	537 496	
1216	540 1091	
1216	687 914	
1216	1043	277
1216	1044	141
1216	1046	138
1216	1091	516
1216	1105	197
1216	1179	1291
1216	1180	569
1216	1193	179
1216	1195	1770
1216	1246	298
1216	1378	197
1216	1672	330
1216	1854	197
1216	1858	1080
1216	2150	149
1216	2580	145
1216	2600	200
1216	2661	370
1216	2723	247
1216	3616	7246
1216	4255	1531

1216	4262	6450
1216	6453	669
1216	6463	820
1216	6766	1888
1216	6770	1367
1216	8884	1430
1216	10038	153
1216	11795	398
1216	12620	199
1216	13222	196
1217	362 217	
1217	604 171	
1217	618 282	
1217	898 295	
1217	984 294	
1217	1375	153
1217	1383	361
1217	1389	633
1217	1463	397
1217	1780	232
1217	1934	189
1217	1971	277
1217	2139	222
1217	2269	159
1217	2544	194
1217	3057	298
1217	3128	153
1217	3258	148
1217	3259	358
1217	3681	318

1217	4618	222
1217	4810	152
1217	5414	194
1217	5437	454
1217	5650	157
1217	6222	239
1217	6422	159
1217	7137	157
1217	7644	200
1217	8047	307
1217	8264	176
1217	8479	315
1217	8676	170
1217	8727	154
1217	9485	148
1217	9882	151
1217	11416	182
1217	11421	227
1217	11915	160
1217	12165	248
1217	12410	188
1217	13223	274
1217	13224	258
1217	13225	247
1217	13226	227
1217	13227	202
1217	13228	178
1217	13229	155
1217	13230	149
1217	13231	144

1219	58	1083
1219	65	1186
1219	67	990
1219	70	1126
1219	172	449
1219	180	723
1219	182	284
1219	184	699
1219	187	414
1219	190	306
1219	191	385
1219	199	309
1219	207	449
1219	221	1912
1219	222	923
1219	229	1120
1219	230	262
1219	289	263
1219	300	514
1219	302	572
1219	306	317
1219	316	1710
1219	318	190
1219	425	283
1219	511	309
1219	533	709
1219	535	200
1219	722	971
1219	962	405
1219	1062	1020

1219	1375	436
1219	1377	1215
1219	1470	1166
1219	1505	776
1219	1934	189
1219	2521	474
1219	2544	1664
1219	2783	271
1219	3057	249
1219	3676	524
1219	3706	375
1219	3753	874
1219	3763	400
1219	3871	1193
1219	4308	285
1219	4435	710
1219	5790	223
1219	7321	395
1219	8674	236
1219	13232	331
1220	58	969
1220	64	1049
1220	65	989
1220	67	2438
1220	70	831
1220	80	892
1220	84	697
1220	97	1199
1220	157	1408
1220	159	2539

1220	167 715	
1220	172 722	
1220	173 5299	
1220	183 1092	
1220	199 799	
1220	228 778	
1220	229 742	
1220	292 3969	
1220	301 1271	
1220	378 1081	
1220	416 709	
1220	425 1096	
1220	432 808	
1220	436 1168	
1220	437 880	
1220	533 1756	
1220	546 686	
1220	689 2397	
1220	701 1240	
1220	757 1177	
1220	886 1312	
1220	901 772	
1220	903 1212	
1220	1001	853
1220	1009	968
1220	1090	992
1220	1409	1523
1220	1424	739
1220	1556	1154
1220	1934	1186

1220	1954	775
1220	2083	869
1220	2277	872
1220	3502	826
1220	4384	756
1220	4435	1277
1220	5045	781
1220	5150	802
1220	5932	692
1220	8675	727
1221	412 25	
1221	1046	35
1221	1192	26
1221	4185	47
1221	4188	26
1221	4219	32
1221	4224	40
1221	6294	47
1221	8478	44
1221	8925	36
1221	8934	24
1221	9794	60
1221	9807	60
1221	9812	21
1221	11553	24
1221	12490	30
1221	12492	22
1221	12499	27
1221	13233	86
1221	13234	67

1221	13235	67
1221	13236	60
1221	13237	54
1221	13238	48
1221	13239	35
1221	13240	34
1221	13241	33
1221	13242	30
1221	13243	30
1221	13244	28
1221	13245	27
1221	13246	26
1221	13247	25
1221	13248	25
1221	13249	24
1221	13250	24
1221	13251	23
1221	13252	21
1221	13253	21
1221	13254	21
1221	13255	21
1221	13256	21
1221	13257	20
1221	13258	20
1221	13259	19
1221	13260	19
1221	13261	19
1221	13262	19
1221	13263	19
1221	13264	18

1222	67	1529
1222	89	630
1222	180	717
1222	187	648
1222	257	821
1222	279	558
1222	285	761
1222	289	1296
1222	291	682
1222	292	830
1222	295	852
1222	296	3622
1222	299	570
1222	302	905
1222	305	593
1222	323	1625
1222	328	549
1222	333	573
1222	342	553
1222	349	843
1222	350	843
1222	455	4136
1222	456	822
1222	459	761
1222	462	1382
1222	465	578
1222	467	1413
1222	472	653
1222	474	762
1222	479	1931

1222	786	635
1222	798	614
1222	800	1065
1222	891	1125
1222	906	1140
1222	951	895
1222	1081	823
1222	1459	701
1222	1602	1253
1222	2021	548
1222	2022	548
1222	3460	719
1222	3748	2345
1222	3763	727
1222	4257	680
1222	5000	701
1222	9231	752
1222	11725	558
1222	13265	1007
1222	13266	806
1223	59	602
1223	66	945
1223	69	753
1223	72	1160
1223	93	583
1223	157	894
1223	163	624
1223	199	647
1223	227	807
1223	228	901

1223	235 572	
1223	539 669	
1223	565 898	
1223	580 813	
1223	583 676	
1223	599 766	
1223	601 1681	
1223	728 748	
1223	734 870	
1223	735 984	
1223	903 609	
1223	992 957	
1223	1005	623
1223	1106	1039
1223	1123	589
1223	1412	779
1223	1427	899
1223	1519	710
1223	1752	1014
1223	1844	661
1223	2629	762
1223	2641	809
1223	2901	568
1223	4369	577
1223	5150	1211
1223	5659	688
1223	5790	601
1223	6742	683
1223	7056	698
1223	7525	940

1223	7643	975
1223	7650	915
1223	7685	866
1223	8267	636
1223	8312	731
1223	9888	603
1223	11124	813
1223	11927	634
1223	13267	855
1223	13268	737
1224	565 95	
1224	580 108	
1224	581 55	
1224	582 66	
1224	583 85	
1224	728 97	
1224	735 79	
1224	868 44	
1224	975 51	
1224	1412	48
1224	1415	40
1224	1426	54
1224	1639	91
1224	2786	49
1224	2834	56
1224	2842	111
1224	2855	63
1224	2864	100
1224	2869	45
1224	2871	81

1224	2894	150
1224	2896	60
1224	2901	77
1224	4251	46
1224	5660	40
1224	6165	58
1224	6363	42
1224	6541	47
1224	6958	44
1224	6959	83
1224	7056	119
1224	8111	83
1224	8114	84
1224	8123	50
1224	8126	57
1224	8805	59
1224	8808	50
1224	11885	100
1224	11917	43
1224	11918	43
1224	11923	52
1224	12057	60
1224	12059	40
1224	12123	77
1224	13269	45
1224	13270	44
1224	13271	44
1224	13272	41
1224	13273	38
1224	13274	38

1225	52	78
1225	53	59
1225	54	37
1225	64	23
1225	65	16
1225	66	14
1225	69	69
1225	72	565
1225	81	62
1225	86	21
1225	99	62
1225	153	16
1225	159	68
1225	173	223
1225	176	17
1225	180	26
1225	187	82
1225	190	33
1225	198	14
1225	229	18
1225	238	22
1225	441	21
1225	511	61
1225	518	20
1225	522	17
1225	735	23
1225	851	14
1225	877	18
1225	930	47
1225	959	15

1225	961	38
1225	1014	17
1225	1061	17
1225	1099	19
1225	1106	16
1225	1150	34
1225	1249	24
1225	1713	76
1225	3763	20
1225	4988	16
1225	6524	13
1225	6760	50
1225	11794	52
1225	12287	34
1225	13275	43
1225	13276	21
1225	13277	20
1225	13278	18
1225	13279	16
1225	13280	15
1226	55	2076
1226	67	423
1226	89	405
1226	261	305
1226	265	192
1226	270	423
1226	288	168
1226	289	768
1226	292	539
1226	295	1016

1226	299	12016
1226	300	136
1226	310	1567
1226	316	182
1226	333	867
1226	377	249
1226	378	1193
1226	466	162
1226	542	413
1226	652	481
1226	677	1105
1226	701	2163
1226	903	191
1226	1098	487
1226	1123	164
1226	1432	539
1226	1475	402
1226	1601	238
1226	1764	544
1226	2047	232
1226	2498	172
1226	2984	405
1226	3173	4131
1226	3332	443
1226	3387	203
1226	3393	597
1226	3503	1527
1226	3930	196
1226	4716	2425
1226	6505	649

1226	6721	266
1226	10691	843
1226	11445	158
1226	13281	1038
1226	13282	428
1226	13283	329
1226	13284	276
1226	13285	174
1226	13286	155
1226	13287	138
1228	107 479	
1228	152 2144	
1228	154 501	
1228	163 1158	
1228	220 266	
1228	226 348	
1228	227 272	
1228	230 333	
1228	233 258	
1228	234 319	
1228	407 341	
1228	441 421	
1228	562 229	
1228	603 741	
1228	614 406	
1228	707 359	
1228	747 343	
1228	769 834	
1228	845 269	
1228	881 392	

1228	920 274	
1228	959 595	
1228	982 228	
1228	1110	733
1228	1119	662
1228	1131	225
1228	1229	340
1228	1372	394
1228	1411	366
1228	1412	722
1228	1418	236
1228	1947	339
1228	1974	420
1228	2151	312
1228	2347	528
1228	2473	240
1228	2493	612
1228	2716	221
1228	2817	418
1228	3417	414
1228	3528	426
1228	3616	234
1228	3805	436
1228	4271	516
1228	6048	499
1228	6151	425
1228	6160	230
1228	6830	261
1228	9716	1035
1228	13291	289

1229	51	157
1229	67	373
1229	72	288
1229	89	114
1229	155	809
1229	157	155
1229	161	148
1229	257	125
1229	288	180
1229	295	203
1229	300	190
1229	306	353
1229	327	173
1229	378	130
1229	529	276
1229	534	270
1229	605	152
1229	641	110
1229	968	375
1229	972	888
1229	1009	609
1229	1014	111
1229	1433	1360
1229	1443	410
1229	1500	129
1229	1673	532
1229	2179	234
1229	3063	141
1229	3316	410
1229	3674	259

1229	3985	243
1229	5239	162
1229	6317	457
1229	6319	129
1229	7994	1020
1229	7995	496
1229	10717	192
1229	13292	402
1229	13293	244
1229	13294	235
1229	13295	228
1229	13296	223
1229	13297	184
1229	13298	174
1229	13299	165
1229	13300	142
1229	13301	134
1229	13302	122
1229	13303	116
1229	13304	113
1230	73	8
1230	219	9
1230	1709	7
1230	1743	9
1230	1762	8
1230	1783	9
1230	1790	5
1230	3462	7
1230	3505	8
1230	4379	6

1230	4928	5
1230	6161	6
1230	6176	9
1230	6764	6
1230	11146	6
1230	13231	7
1230	13305	83
1230	13306	72
1230	13307	13
1230	13308	12
1230	13309	11
1230	13310	11
1230	13311	11
1230	13312	10
1230	13313	9
1230	13314	9
1230	13315	9
1230	13316	8
1230	13317	8
1230	13318	8
1230	13319	8
1230	13320	8
1230	13321	8
1230	13322	8
1230	13323	8
1230	13324	7
1230	13325	7
1230	13326	7
1230	13327	7
1230	13328	7

1230	13329	7
1230	13330	7
1230	13331	6
1230	13332	6
1230	13333	6
1230	13334	6
1230	13335	6
1230	13336	6
1230	13337	5
1230	13338	5
1231	63	555
1231	67	339
1231	72	324
1231	93	783
1231	157	523
1231	163	170
1231	234	315
1231	511	434
1231	706	160
1231	712	646
1231	715	424
1231	724	495
1231	729	172
1231	733	211
1231	734	252
1231	735	242
1231	868	874
1231	917	428
1231	959	237
1231	1098	257

1231	1110	679
1231	1130	786
1231	1131	923
1231	1244	343
1231	1249	248
1231	1272	172
1231	1285	186
1231	1372	394
1231	1412	278
1231	1413	157
1231	1415	345
1231	1425	165
1231	1800	194
1231	2375	258
1231	2901	194
1231	4126	263
1231	4600	431
1231	5073	272
1231	5175	293
1231	5497	181
1231	5521	843
1231	7119	167
1231	7152	257
1231	8802	266
1231	9706	190
1231	11193	173
1231	12352	174
1231	12738	266
1231	13339	338
1231	13340	271

1232	30	66
1232	51	65
1232	59	144
1232	72	2793
1232	154	86
1232	159	1654
1232	173	75
1232	418	104
1232	429	62
1232	441	1125
1232	450	166
1232	515	530
1232	599	598
1232	858	95
1232	866	193
1232	875	455
1232	1072	69
1232	1083	631
1232	1089	96
1232	1151	107
1232	1534	296
1232	1707	85
1232	1713	148
1232	1886	121
1232	2531	276
1232	2938	142
1232	3280	397
1232	3441	217
1232	3472	56
1232	4649	66

1232	4973	132
1232	5260	73
1232	5906	188
1232	5908	56
1232	5913	350
1232	5918	176
1232	6883	72
1232	9256	343
1232	9501	104
1232	11489	115
1232	13341	585
1232	13342	440
1232	13343	422
1232	13344	317
1232	13345	241
1232	13346	185
1232	13347	149
1232	13348	83
1232	13349	68
1232	13350	58
1233	55	15252
1233	56	1589
1233	64	612
1233	67	14122
1233	89	398
1233	288	405
1233	289	5871
1233	299	2612
1233	310	1369
1233	323	393

1233	328 705	
1233	352 516	
1233	386 427	
1233	532 907	
1233	535 2274	
1233	701 687	
1233	786 397	
1233	789 9142	
1233	792 2250	
1233	886 686	
1233	906 5555	
1233	1001	1305
1233	1109	444
1233	1395	622
1233	1409	361
1233	1464	1737
1233	1729	4632
1233	2018	428
1233	2521	543
1233	3193	943
1233	3397	592
1233	3491	371
1233	3795	649
1233	4153	377
1233	4983	467
1233	5178	362
1233	7243	443
1233	7302	547
1233	7814	1047
1233	7821	355

1233	8586	428
1233	9134	451
1233	10691	736
1233	11084	1074
1233	13351	5202
1233	13352	1400
1233	13353	1060
1233	13354	764
1233	13355	590
1233	13356	376
1234	53	176
1234	55	769
1234	72	387
1234	77	430
1234	86	295
1234	89	874
1234	155	243
1234	157	211
1234	175	229
1234	187	388
1234	288	480
1234	289	712
1234	292	219
1234	301	226
1234	466	334
1234	486	192
1234	502	235
1234	544	1160
1234	690	196
1234	972	230

1234	1001	869
1234	1004	219
1234	1379	242
1234	1571	505
1234	1772	212
1234	1793	194
1234	1892	265
1234	2044	358
1234	2054	250
1234	2067	259
1234	2521	272
1234	3367	1048
1234	3501	1842
1234	3966	635
1234	4707	164
1234	4880	1068
1234	6705	249
1234	7643	234
1234	8002	395
1234	8283	189
1234	9656	260
1234	9742	216
1234	13357	402
1234	13358	375
1234	13359	257
1234	13360	249
1234	13361	247
1234	13362	213
1234	13363	181
1234	13364	166

1235	51	410
1235	65	221
1235	72	209
1235	163	235
1235	172	256
1235	230	507
1235	233	1011
1235	298	346
1235	377	433
1235	389	785
1235	540	248
1235	592	368
1235	599	282
1235	603	324
1235	707	310
1235	715	437
1235	978	779
1235	1110	326
1235	1122	277
1235	1246	227
1235	1390	278
1235	1707	237
1235	1718	480
1235	1772	263
1235	1795	352
1235	2469	842
1235	3119	273
1235	4126	428
1235	4928	516
1235	5258	208

1235	6060	1055
1235	6257	268
1235	7096	264
1235	7256	258
1235	7260	916
1235	7507	272
1235	8670	448
1235	11510	393
1235	12315	268
1235	12736	736
1235	13365	1019
1235	13366	456
1235	13367	360
1235	13368	356
1235	13369	314
1235	13370	254
1235	13371	243
1235	13372	235
1235	13373	227
1235	13374	213
1236	7	15239
1236	198	1158
1236	210	897
1236	377	990
1236	416	1032
1236	453	1758
1236	464	914
1236	472	3877
1236	475	10240
1236	490	2557

1236 517 1969

1236 547 881

1236 614 69301

1236 757 1070

1236 808 2088

1236 812 2955

1236 854 2674

1236 856 2495

1236 923 6015

1236 953 1988

1236 966 1970

1236 978 7651

1236 1104 7365

1236 1118 9050

1236 1131 18600

1236 1179 3653

1236 1401 700

1236 1409 874

1236 1410 3392

1236 1449 696

1236 2172 14820

1236 2352 1274

1236 2366 2559

1236 2380 2096

1236 2474 3749

1236 2479 10491

1236 2818 1251

1236 4365 2503

1236 5154 787

1236 5947 1396

1236	6257	7606
1236	6618	5743
1236	6678	928
1236	8347	803
1236	8930	1575
1236	10956	2390
1236	11021	776
1236	13375	1606
1236	13376	1500
1236	13377	778
1237	67	358
1237	89	1209
1237	154	365
1237	159	735
1237	173	520
1237	195	1416
1237	196	368
1237	207	1142
1237	208	318
1237	222	335
1237	227	1424
1237	233	338
1237	288	236
1237	295	335
1237	298	300
1237	310	240
1237	329	411
1237	425	430
1237	428	328
1237	572	1074

1237	604 547	
1237	615 488	
1237	689 841	
1237	757 278	
1237	868 413	
1237	907 454	
1237	1097	371
1237	1153	1561
1237	1155	553
1237	1400	295
1237	1511	807
1237	1648	233
1237	1976	378
1237	1983	503
1237	2277	247
1237	2711	542
1237	3019	230
1237	3057	370
1237	11838	321
1237	12791	370
1237	13079	633
1237	13378	846
1237	13379	482
1237	13380	456
1237	13381	441
1237	13382	423
1237	13383	291
1237	13384	281
1237	13385	256
1237	13386	255

1238	88	343
1238	154	390
1238	163	184
1238	197	463
1238	199	513
1238	203	156
1238	212	852
1238	216	211
1238	227	665
1238	228	211
1238	234	327
1238	238	234
1238	250	216
1238	418	424
1238	420	174
1238	425	570
1238	429	200
1238	436	500
1238	437	484
1238	573	644
1238	618	192
1238	709	225
1238	735	184
1238	859	446
1238	863	254
1238	1062	258
1238	1390	350
1238	1412	263
1238	1513	232
1238	1639	285

1238	1710	415
1238	1958	249
1238	2419	1527
1238	3310	189
1238	5235	684
1238	5237	439
1238	5414	334
1238	5660	419
1238	7505	269
1238	8649	170
1238	8824	163
1238	8837	265
1238	9590	176
1238	9595	244
1238	13387	924
1238	13388	664
1238	13389	338
1238	13390	207
1238	13391	197
1238	13392	185
1239	53	233
1239	65	339
1239	81	190
1239	152	841
1239	154	646
1239	159	222
1239	163	2334
1239	166	196
1239	171	369
1239	188	272

1239	203	178
1239	204	177
1239	220	490
1239	226	293
1239	227	1232
1239	228	725
1239	298	413
1239	424	333
1239	436	167
1239	439	401
1239	533	346
1239	609	357
1239	615	231
1239	630	280
1239	683	401
1239	868	346
1239	962	161
1239	964	274
1239	982	251
1239	1048	719
1239	1412	419
1239	1414	336
1239	1424	257
1239	1519	332
1239	1643	172
1239	1755	528
1239	1767	161
1239	2605	275
1239	2608	169
1239	2711	268

1239	2786	191
1239	2797	161
1239	2855	232
1239	4675	261
1239	6295	203
1239	6724	649
1239	10418	381
1239	13393	485
1239	13394	388
1239	13395	176
1240	154	135
1240	162	154
1240	163	557
1240	197	159
1240	199	245
1240	207	194
1240	216	114
1240	227	333
1240	416	118
1240	418	436
1240	425	161
1240	432	118
1240	441	130
1240	554	207
1240	632	246
1240	763	143
1240	862	158
1240	863	159
1240	871	133
1240	1011	127

1240	1418	1777
1240	1423	359
1240	1504	350
1240	1510	671
1240	1700	119
1240	1737	179
1240	2531	154
1240	2532	119
1240	2828	141
1240	3279	151
1240	3364	850
1240	5434	114
1240	6048	329
1240	6161	129
1240	6182	104
1240	6759	151
1240	7375	208
1240	7376	215
1240	7387	118
1240	8043	148
1240	9260	119
1240	9448	105
1240	11234	290
1240	11958	139
1240	13396	414
1240	13397	177
1240	13398	146
1240	13399	131
1240	13400	122
1240	13401	113

1242	154	380
1242	163	325
1242	174	153
1242	181	179
1242	198	1290
1242	202	746
1242	204	375
1242	220	259
1242	709	127
1242	726	407
1242	735	596
1242	785	430
1242	846	296
1242	883	208
1242	885	574
1242	919	589
1242	951	128
1242	956	129
1242	959	513
1242	965	185
1242	978	346
1242	1093	3279
1242	1104	581
1242	1118	783
1242	1119	172
1242	1131	159
1242	1145	680
1242	1249	263
1242	1358	322
1242	1372	171

1242	1412	215
1242	1510	132
1242	1700	180
1242	1714	184
1242	1810	140
1242	2342	392
1242	2369	235
1242	2835	246
1242	2836	740
1242	3470	220
1242	3877	377
1242	6678	131
1242	8042	266
1242	8333	156
1242	8910	385
1242	11510	228
1242	11587	140
1242	13409	326
1242	13410	207
1242	13411	200
1243	154 303	
1243	159 270	
1243	173 161	
1243	234 268	
1243	383 163	
1243	412 115	
1243	424 111	
1243	436 292	
1243	453 80	
1243	512 160	

1243	517 114	
1243	614 351	
1243	715 165	
1243	843 236	
1243	854 358	
1243	856 134	
1243	860 108	
1243	918 111	
1243	965 224	
1243	977 70	
1243	982 362	
1243	1044	84
1243	1116	154
1243	1118	76
1243	1145	88
1243	1239	257
1243	1406	124
1243	1513	209
1243	1527	97
1243	1797	83
1243	1873	81
1243	1957	135
1243	2206	73
1243	2352	83
1243	2637	122
1243	3732	150
1243	4179	76
1243	4216	83
1243	5216	70
1243	6130	72

1243	7423	103
1243	7532	82
1243	8747	273
1243	9344	103
1243	9467	144
1243	11517	70
1243	13412	209
1243	13413	120
1243	13414	95
1243	13415	83
1244	59	565
1244	76	179
1244	100	284
1244	155	284
1244	159	616
1244	203	960
1244	207	387
1244	227	415
1244	298	378
1244	429	1713
1244	441	310
1244	475	300
1244	539	309
1244	599	393
1244	618	295
1244	620	860
1244	715	2960
1244	851	421
1244	863	216
1244	1090	300

1244	1206	459
1244	1244	1043
1244	1351	213
1244	1890	295
1244	2531	3573
1244	2587	268
1244	4732	257
1244	5229	383
1244	6331	331
1244	10652	254
1244	10766	919
1244	11234	221
1244	11849	1652
1244	12165	528
1244	12724	479
1244	12726	188
1244	13416	2185
1244	13417	1651
1244	13418	978
1244	13419	787
1244	13420	650
1244	13421	397
1244	13422	273
1244	13423	254
1244	13424	239
1244	13425	228
1244	13426	211
1244	13427	208
1244	13428	190
1244	13429	170

1245	209 447	
1245	228 450	
1245	229 696	
1245	403 1111	
1245	481 820	
1245	486 4443	
1245	488 1274	
1245	492 1331	
1245	493 1735	
1245	497 671	
1245	498 905	
1245	504 828	
1245	507 768	
1245	540 3050	
1245	930 425	
1245	1043	746
1245	1105	1123
1245	1179	5074
1245	1182	990
1245	1193	724
1245	1197	1107
1245	1201	636
1245	1246	848
1245	1378	3230
1245	1517	524
1245	1854	1586
1245	1866	1502
1245	2600	468
1245	2661	5742
1245	2815	437

1245	3082	3480
1245	3341	421
1245	3373	1198
1245	3616	450
1245	3769	995
1245	4223	2196
1245	4255	3395
1245	4262	1489
1245	6008	1317
1245	6191	2479
1245	6247	903
1245	6298	1709
1245	6769	1247
1245	7776	1551
1245	7897	494
1245	8884	655
1245	13430	1201
1245	13431	661
1245	13432	626
1245	13433	552
1246	55	1173
1246	56	468
1246	64	285
1246	67	266
1246	151	242
1246	161	215
1246	184	177
1246	187	3416
1246	289	331
1246	292	351

1246	295	164
1246	389	975
1246	526	192
1246	535	261
1246	701	218
1246	727	588
1246	959	162
1246	1464	196
1246	2051	641
1246	2075	186
1246	2076	568
1246	2079	2512
1246	2441	705
1246	2446	266
1246	2521	643
1246	3288	175
1246	3674	170
1246	3832	1029
1246	3991	323
1246	4301	202
1246	6181	624
1246	6733	469
1246	6739	199
1246	7096	163
1246	8848	1267
1246	8853	875
1246	9422	201
1246	10358	188
1246	12351	160
1246	12734	228

1246	13434	815
1246	13435	775
1246	13436	323
1246	13437	265
1246	13438	251
1246	13439	219
1246	13440	209
1246	13441	189
1246	13442	167
1246	13443	166
1247	55	6788
1247	67	17130
1247	89	2530
1247	157	445
1247	191	235
1247	257	331
1247	265	669
1247	288	1244
1247	289	18779
1247	291	924
1247	292	7692
1247	295	784
1247	300	1144
1247	302	335
1247	305	208
1247	309	355
1247	310	247
1247	318	4145
1247	320	301
1247	325	1778

1247	328 237	
1247	329 165	
1247	331 165	
1247	333 348	
1247	349 405	
1247	352 155	
1247	386 710	
1247	461 1870	
1247	466 1674	
1247	475 166	
1247	498 155	
1247	523 615	
1247	531 151	
1247	679 1450	
1247	680 256	
1247	681 209	
1247	686 647	
1247	701 233	
1247	793 442	
1247	907 183	
1247	1037	181
1247	1379	189
1247	1580	239
1247	1684	205
1247	1700	1217
1247	2521	178
1247	3397	357
1247	4920	637
1247	13444	380
1247	13445	306

1248	69	38
1248	97	608
1248	241	169
1248	251	1090
1248	257	3629
1248	261	38
1248	263	229
1248	275	102
1248	283	283
1248	291	34
1248	292	764
1248	294	56
1248	301	787
1248	302	51
1248	311	47
1248	333	223
1248	342	76
1248	498	44
1248	528	81
1248	575	213
1248	683	61
1248	691	38
1248	701	37
1248	903	203
1248	1377	39
1248	1447	110
1248	1556	338
1248	1772	386
1248	1775	48
1248	1934	1036

1248	1936	234
1248	2067	114
1248	2220	45
1248	2546	50
1248	2988	77
1248	3081	111
1248	3110	84
1248	3321	39
1248	3488	90
1248	3796	53
1248	4616	166
1248	5789	70
1248	6914	66
1248	7781	119
1248	11240	193
1248	12406	67
1248	13446	763
1248	13447	64
1248	13448	51
1248	13449	37
1249	51	235
1249	72	259
1249	88	217
1249	157	245
1249	159	258
1249	163	278
1249	197	216
1249	212	216
1249	222	275
1249	227	429

1249 233 219

1249 245 392

1249 578 280

1249 607 490

1249 630 212

1249 764 219

1249 864 234

1249 880 402

1249 910 218

1249 1083 219

1249 1119 460

1249 1203 408

1249 1358 233

1249 1369 405

1249 1372 294

1249 1412 312

1249 1427 398

1249 1707 363

1249 1881 252

1249 2013 236

1249 2222 209

1249 2362 291

1249 2493 431

1249 2855 224

1249 3230 290

1249 3289 385

1249 3313 268

1249 3444 210

1249 3505 311

1249 9321 256

1249	12083	421
1249	12243	240
1249	12643	425
1249	13450	346
1249	13451	264
1249	13452	255
1249	13453	250
1249	13454	236
1249	13455	217
1249	13456	216
1253	55	232
1253	65	700
1253	89	1595
1253	161	334
1253	229	628
1253	283	262
1253	288	2630
1253	289	11302
1253	292	678
1253	295	937
1253	298	311
1253	299	2493
1253	300	1303
1253	306	644
1253	318	1236
1253	325	636
1253	329	769
1253	333	2999
1253	342	334
1253	349	1129

1253 352 363

1253 461 241

1253 464 397

1253 466 682

1253 498 4362

1253 540 223

1253 542 5479

1253 543 554

1253 646 235

1253 679 1162

1253 680 468

1253 687 206

1253 701 363

1253 704 413

1253 802 517

1253 903 359

1253 1032 222

1253 1042 564

1253 1045 2218

1253 1047 210

1253 1246 294

1253 2080 314

1253 2548 250

1253 2984 330

1253 3378 231

1253 4474 403

1253 4708 261

1253 5594 216

1253 7706 381

1253 13497 624

1254	152 1172	
1254	154 344	
1254	162 964	
1254	174 275	
1254	186 375	
1254	188 604	
1254	238 689	
1254	370 1405	
1254	418 337	
1254	503 406	
1254	506 290	
1254	512 310	
1254	707 249	
1254	748 580	
1254	812 517	
1254	918 329	
1254	1974	235
1254	2298	316
1254	2299	485
1254	2300	366
1254	2308	818
1254	2605	316
1254	2716	710
1254	3417	517
1254	3940	533
1254	4613	641
1254	6948	263
1254	6961	346
1254	7456	249
1254	8431	267

1254	8444	372
1254	9405	256
1254	10663	259
1254	10719	568
1254	12452	258
1254	13498	1478
1254	13499	762
1254	13500	728
1254	13501	683
1254	13502	629
1254	13503	550
1254	13504	508
1254	13505	324
1254	13506	311
1254	13507	274
1254	13508	271
1254	13509	263
1254	13510	249
1254	13511	237
1254	13512	236
1255	55	598
1255	59	1202
1255	67	573
1255	72	5380
1255	89	1153
1255	172	650
1255	173	1437
1255	190	335
1255	199	751
1255	204	381

1255	229 408	
1255	289 441	
1255	292 317	
1255	295 402	
1255	298 1028	
1255	300 279	
1255	316 398	
1255	418 292	
1255	421 271	
1255	423 591	
1255	430 563	
1255	432 1091	
1255	436 667	
1255	437 490	
1255	439 659	
1255	440 606	
1255	504 261	
1255	525 295	
1255	679 745	
1255	689 633	
1255	690 247	
1255	962 1314	
1255	985 448	
1255	1098	1449
1255	1106	407
1255	1243	358
1255	1379	606
1255	1400	296
1255	1755	406
1255	1986	330

1255	2277	276
1255	2531	781
1255	3057	296
1255	3200	1645
1255	4716	426
1255	6210	364
1255	6414	386
1255	8707	874
1255	10427	246
1255	13513	412
1256	65	364
1256	96	77
1256	159	351
1256	217	154
1256	229	178
1256	403	135
1256	441	192
1256	498	148
1256	511	26101
1256	715	160
1256	910	91
1256	967	373
1256	977	140
1256	982	474
1256	1122	106
1256	1249	74
1256	1378	2309
1256	1519	1732
1256	2276	123
1256	3335	111

1256	3621	99
1256	3754	132
1256	4079	146
1256	4201	137
1256	4207	67
1256	4442	300
1256	5650	69
1256	6294	93
1256	6602	258
1256	6906	125
1256	8461	84
1256	8467	273
1256	8469	293
1256	9852	220
1256	11512	459
1256	13514	952
1256	13515	619
1256	13516	607
1256	13517	326
1256	13518	191
1256	13519	149
1256	13520	143
1256	13521	121
1256	13522	109
1256	13523	83
1256	13524	82
1256	13525	78
1256	13526	75
1256	13527	74
1256	13528	67

1258	98	85
1258	159	69
1258	203	44
1258	234	53
1258	429	54
1258	441	89
1258	612	72
1258	631	159
1258	851	59
1258	852	47
1258	857	74
1258	863	35
1258	866	51
1258	867	168
1258	875	111
1258	985	148
1258	1017	60
1258	1083	109
1258	1513	67
1258	1534	141
1258	1740	63
1258	1811	41
1258	1814	544
1258	1879	79
1258	1881	53
1258	2029	52
1258	2488	61
1258	2938	53
1258	3430	37
1258	3436	51

1258	3444	219
1258	4129	89
1258	4155	142
1258	4156	146
1258	4250	72
1258	4278	53
1258	4795	38
1258	5260	75
1258	5785	48
1258	7076	44
1258	9469	158
1258	10127	46
1258	11933	35
1258	11963	46
1258	12314	80
1258	13529	63
1258	13530	49
1258	13531	46
1258	13532	45
1258	13533	37
1259	45	4589
1259	185	260
1259	209	26787
1259	472	486
1259	486	54600
1259	497	389
1259	498	1282
1259	500	1378
1259	501	1385
1259	503	405

1259	517	469
1259	536	342
1259	537	2559
1259	707	1571
1259	918	405
1259	930	256
1259	951	2103
1259	966	355
1259	982	404
1259	1044	490
1259	1145	337
1259	1179	601
1259	1180	1099
1259	1672	5103
1259	1854	707
1259	1914	420
1259	1927	653
1259	2014	495
1259	2044	299
1259	2352	316
1259	2436	884
1259	2524	405
1259	2634	354
1259	2794	409
1259	3427	378
1259	3567	285
1259	3859	351
1259	4262	568
1259	5437	286
1259	6194	2128

1259	6235	442
1259	6249	298
1259	6292	555
1259	11590	1181
1259	12168	1121
1259	13534	2297
1259	13535	387
1259	13536	346
1259	13537	301
1259	13538	291
1261	58	356
1261	65	389
1261	81	641
1261	154	3405
1261	196	185
1261	199	254
1261	202	240
1261	204	190
1261	206	203
1261	217	247
1261	225	643
1261	228	433
1261	231	184
1261	237	318
1261	246	276
1261	261	615
1261	418	965
1261	450	386
1261	533	221
1261	604	658

1261	615 236	
1261	622 240	
1261	689 293	
1261	703 323	
1261	1075	490
1261	1098	2028
1261	1109	350
1261	1389	218
1261	1400	397
1261	1424	1238
1261	1512	314
1261	1632	615
1261	1633	191
1261	1755	1375
1261	1764	331
1261	1767	222
1261	1776	215
1261	1934	259
1261	2277	264
1261	2638	297
1261	3057	647
1261	3110	202
1261	3259	194
1261	3468	246
1261	5414	184
1261	5745	180
1261	5925	837
1261	6222	350
1261	10729	504
1261	13540	225

1262	288	1
1262	295	1
1262	545	1
1262	793	1
1262	1698	1
1262	3316	3
1262	4879	1
1262	5353	1
1262	12243	1
1262	12244	2
1262	12254	1
1262	12261	1
1262	12268	1
1262	12270	1
1262	12407	1
1262	13541	2
1262	13542	2
1262	13543	1
1262	13544	1
1262	13545	1
1262	13546	1
1263	55	925
1263	67	411
1263	89	740
1263	157	601
1263	283	2130
1263	288	372
1263	289	15185
1263	290	372
1263	292	512

1263	295	601
1263	299	747
1263	300	378
1263	305	582
1263	315	1819
1263	318	692
1263	324	775
1263	325	675
1263	377	543
1263	461	410
1263	523	584
1263	536	628
1263	637	1159
1263	638	528
1263	641	530
1263	669	1192
1263	679	1605
1263	701	1763
1263	790	829
1263	791	525
1263	1246	388
1263	2018	487
1263	2645	835
1263	3173	1073
1263	3176	778
1263	3873	452
1263	4461	481
1263	4709	691
1263	6119	586
1263	7340	492

1263	7448	392
1263	7452	454
1263	7706	572
1263	8718	2093
1263	13547	2529
1263	13548	663
1263	13549	599
1263	13550	454
1263	13551	436
1263	13552	401
1263	13553	377
1264	65	169
1264	163	3163
1264	190	719
1264	192	205
1264	203	130
1264	207	108
1264	212	121
1264	220	158
1264	227	4514
1264	234	490
1264	511	158
1264	533	2621
1264	562	1525
1264	599	628
1264	614	88
1264	706	558
1264	712	78
1264	714	341
1264	717	67

1264	725 287	
1264	733 106	
1264	735 313	
1264	917 73	
1264	959 8943	
1264	982 460	
1264	1110	74
1264	1122	132
1264	1241	785
1264	1242	456
1264	1244	351
1264	1249	112
1264	1351	244
1264	1412	505
1264	1414	224
1264	1416	213
1264	1513	93
1264	1755	483
1264	1764	83
1264	2347	71
1264	2625	83
1264	2742	124
1264	3071	69
1264	3308	133
1264	3400	282
1264	3635	171
1264	4087	199
1264	4349	208
1264	5772	414
1264	13554	82

1264	13555	70
1265	53	127
1265	59	93
1265	64	231
1265	154	752
1265	162	389
1265	170	496
1265	203	703
1265	207	365
1265	418	380
1265	437	124
1265	441	319
1265	454	99
1265	533	841
1265	604	246
1265	618	284
1265	632	465
1265	755	525
1265	1089	254
1265	1159	175
1265	1418	119
1265	1504	240
1265	1747	387
1265	2137	131
1265	2425	114
1265	2542	452
1265	2582	72
1265	2585	287
1265	2691	181
1265	3411	198

1265	4153	146
1265	5414	137
1265	5547	65
1265	5799	92
1265	6217	396
1265	6414	149
1265	6756	83
1265	7377	79
1265	8215	76
1265	8672	409
1265	8864	199
1265	11571	71
1265	12612	249
1265	12716	92
1265	13556	508
1265	13557	150
1265	13558	111
1265	13559	95
1265	13560	91
1265	13561	91
1265	13562	67
1266	13563	92
1266	13564	29
1266	13565	23
1267	65	375
1267	154	457
1267	163	456
1267	188	676
1267	209	861
1267	227	376

1267	229 890	
1267	230 1545	
1267	234 668	
1267	378 593	
1267	412 4524	
1267	439 1840	
1267	475 2144	
1267	498 1469	
1267	501 745	
1267	540 776	
1267	706 1579	
1267	707 1969	
1267	857 531	
1267	917 3227	
1267	918 634	
1267	924 2509	
1267	930 488	
1267	932 1322	
1267	957 1142	
1267	1041	519
1267	1044	430
1267	1045	891
1267	1046	715
1267	1186	515
1267	1206	1120
1267	1249	515
1267	1339	2561
1267	1358	579
1267	1406	1440
1267	1545	785

1267	1672	404
1267	1755	780
1267	4164	396
1267	4468	747
1267	4821	541
1267	4828	1167
1267	4834	896
1267	4841	423
1267	4845	743
1267	4848	713
1267	6120	651
1267	13566	646
1267	13567	413
1267	13568	333
1270	65	77
1270	89	107
1270	184	212
1270	217	155
1270	227	610
1270	253	74
1270	356	78
1270	366	432
1270	412	93
1270	471	62
1270	475	71
1270	507	68
1270	527	368
1270	538	88
1270	687	179
1270	959	92

1270	1043	104
1270	1100	179
1270	1105	77
1270	1246	71
1270	1249	1791
1270	1383	79
1270	1803	133
1270	1866	119
1270	2346	577
1270	2661	62
1270	2840	103
1270	2850	128
1270	3033	180
1270	3616	143
1270	3621	65
1270	3670	72
1270	3754	107
1270	4118	76
1270	4262	343
1270	4837	203
1270	6247	70
1270	6301	80
1270	6453	289
1270	6543	550
1270	6600	116
1270	6769	63
1270	6773	79
1270	11056	99
1270	13539	71
1270	13579	140

1270	13580	118
1270	13581	71
1270	13582	65
1270	13583	62
1271	212 758	
1271	227 4247	
1271	562 295	
1271	618 698	
1271	721 348	
1271	733 377	
1271	735 531	
1271	1123	1498
1271	1150	300
1271	1175	574
1271	1242	400
1271	1414	396
1271	1416	290
1271	2265	519
1271	2419	278
1271	2638	297
1271	2857	308
1271	2902	317
1271	3119	365
1271	3458	619
1271	4123	333
1271	4620	499
1271	6181	563
1271	6756	378
1271	7157	473
1271	7644	941

1271	7877	309
1271	8843	363
1271	9699	417
1271	10969	264
1271	10970	297
1271	11268	594
1271	11646	316
1271	11676	504
1271	13584	826
1271	13585	732
1271	13586	610
1271	13587	602
1271	13588	529
1271	13589	378
1271	13590	364
1271	13591	345
1271	13592	343
1271	13593	332
1271	13594	323
1271	13595	286
1271	13596	270
1271	13597	265
1271	13598	263
1271	13599	261
1272	55	1123
1272	65	1746
1272	67	854
1272	89	14335
1272	199	531
1272	209	1270

1272	227 463
1272	228 1105
1272	229 2235
1272	230 7242
1272	288 683
1272	289 680
1272	291 1863
1272	292 2355
1272	298 2493
1272	300 8554
1272	302 2406
1272	324 263
1272	333 40024
1272	344 2455
1272	378 1401
1272	412 1198
1272	464 1108
1272	466 652
1272	481 2429
1272	486 1093
1272	498 10556
1272	528 2212
1272	538 1175
1272	544 1310
1272	646 488
1272	679 1070
1272	680 1147
1272	681 2358
1272	686 483
1272	687 1270

1272	689	1378
1272	691	1020
1272	701	265
1272	704	2823
1272	802	454
1272	1034	258
1272	1043	1494
1272	1047	11706
1272	1243	292
1272	1246	2339
1272	1390	1080
1272	1400	1019
1272	1673	257
1272	1934	440
1273	7	109
1273	9	275
1273	36	355
1273	56	109
1273	72	445
1273	81	172
1273	154	132
1273	159	298
1273	190	645
1273	225	188
1273	227	401
1273	228	123
1273	229	457
1273	233	125
1273	300	116
1273	407	1148

1273	441	129
1273	450	291
1273	522	149
1273	599	274
1273	707	172
1273	755	107
1273	930	111
1273	971	478
1273	972	236
1273	1090	104
1273	1098	260
1273	1104	1125
1273	1109	1035
1273	1300	833
1273	1385	110
1273	1494	129
1273	1710	338
1273	1714	144
1273	2038	155
1273	3184	176
1273	3186	111
1273	5605	110
1273	5609	109
1273	6397	224
1273	6876	179
1273	7100	145
1273	7415	237
1273	9979	193
1273	10063	157
1273	13600	341

1273	13601	249
1273	13602	220
1273	13603	133
1273	13604	108
1274	96	180
1274	163	190
1274	176	147
1274	192	223
1274	212	413
1274	220	157
1274	227	368
1274	230	145
1274	234	147
1274	302	235
1274	511	127
1274	607	151
1274	614	187
1274	630	182
1274	706	671
1274	707	143
1274	710	156
1274	724	179
1274	728	323
1274	730	187
1274	732	132
1274	734	423
1274	735	528
1274	868	146
1274	951	194
1274	1110	180

1274	1244	131
1274	1249	156
1274	1358	136
1274	1369	147
1274	1372	249
1274	1412	246
1274	1803	143
1274	2183	266
1274	2346	392
1274	2347	151
1274	2788	187
1274	2861	141
1274	2864	197
1274	2893	176
1274	2901	431
1274	4369	628
1274	5168	272
1274	5550	156
1274	8311	279
1274	8312	285
1274	8555	129
1274	9926	148
1274	13605	167
1274	13606	149
1275	58	621
1275	163	1867
1275	190	835
1275	199	794
1275	207	1028
1275	227	7044

1275	228 1016	
1275	229 838	
1275	380 522	
1275	423 549	
1275	424 843	
1275	425 776	
1275	429 864	
1275	437 899	
1275	554 651	
1275	562 8695	
1275	599 511	
1275	709 1004	
1275	733 1194	
1275	735 4299	
1275	868 2821	
1275	903 768	
1275	969 886	
1275	1090	802
1275	1098	556
1275	1122	590
1275	1244	744
1275	1414	730
1275	1424	639
1275	1512	600
1275	1639	931
1275	1772	605
1275	2277	644
1275	2786	711
1275	3057	538
1275	3156	591

1275	3201	1403
1275	3206	638
1275	4129	758
1275	4384	1276
1275	5416	505
1275	7266	1307
1275	8549	1123
1275	8594	753
1275	10971	728
1275	11739	517
1275	13607	1206
1275	13608	797
1275	13609	597
1275	13610	519
1276	412 172	
1276	429 103	
1276	441 133	
1276	1228	165
1276	1239	129
1276	1947	88
1276	1962	80
1276	2531	192
1276	2542	80
1276	2575	103
1276	3043	91
1276	3227	91
1276	3364	98
1276	4092	188
1276	4183	293
1276	6146	239

1276	6901	136
1276	8469	156
1276	8921	90
1276	8929	95
1276	8931	82
1276	8951	94
1276	9794	164
1276	9801	82
1276	10449	160
1276	11401	114
1276	11981	168
1276	11989	126
1276	11993	87
1276	13611	276
1276	13612	204
1276	13613	168
1276	13614	126
1276	13615	122
1276	13616	114
1276	13617	110
1276	13618	109
1276	13619	107
1276	13620	103
1276	13621	101
1276	13622	98
1276	13623	97
1276	13624	96
1276	13625	94
1276	13626	89
1276	13627	87

1276	13628	84
1276	13629	83
1276	13630	83
1276	13631	82
1277	65	2394
1277	154	7294
1277	159	3870
1277	182	1943
1277	184	1174
1277	190	5789
1277	198	1710
1277	207	2465
1277	217	4987
1277	220	1550
1277	221	2276
1277	222	3704
1277	227	3503
1277	229	2180
1277	333	1621
1277	342	2125
1277	366	1537
1277	424	3123
1277	429	1833
1277	439	2757
1277	453	1299
1277	472	1195
1277	498	1508
1277	533	20935
1277	538	1690
1277	540	1241

1277	604	2633
1277	614	3070
1277	615	1126
1277	898	2856
1277	899	1803
1277	1044	1185
1277	1047	1512
1277	1048	2610
1277	1116	1701
1277	1375	1581
1277	1377	1423
1277	1873	1182
1277	2277	1151
1277	2544	1532
1277	2607	1137
1277	3097	2013
1277	3258	2991
1277	3489	1304
1277	3749	1254
1277	4115	1800
1277	5217	7301
1277	6231	3859
1277	9193	1679
1277	10677	2762
1278	65	52
1278	89	104
1278	161	42
1278	191	56
1278	288	1052
1278	295	113

1278	300 483	
1278	302 66	
1278	306 316	
1278	312 1043	
1278	318 53	
1278	325 43	
1278	327 80	
1278	328 221	
1278	332 46	
1278	333 344	
1278	352 158	
1278	464 127	
1278	466 681	
1278	475 431	
1278	498 824	
1278	528 114	
1278	531 75	
1278	545 559	
1278	644 44	
1278	680 112	
1278	701 200	
1278	704 121	
1278	915 88	
1278	1009	49
1278	1042	145
1278	1057	1379
1278	1444	108
1278	1449	61
1278	1455	470
1278	1456	281

1278	1975	158
1278	2084	51
1278	2498	71
1278	2990	182
1278	4564	84
1278	4768	83
1278	5055	274
1278	5530	78
1278	5988	64
1278	7268	54
1278	7868	78
1278	8761	566
1278	9684	52
1278	10987	217
1279	65	68
1279	154	374
1279	170	200
1279	182	37
1279	184	64
1279	190	445
1279	191	60
1279	207	165
1279	208	349
1279	217	411
1279	227	132
1279	229	89
1279	234	52
1279	418	404
1279	425	35
1279	428	324

1279	430 41	
1279	444 266	
1279	632 68	
1279	689 177	
1279	690 48	
1279	697 131	
1279	704 188	
1279	755 207	
1279	757 49	
1279	763 157	
1279	903 49	
1279	1048	46
1279	1090	210
1279	1375	66
1279	1418	103
1279	1424	79
1279	1735	130
1279	1736	121
1279	1747	187
1279	1772	64
1279	2068	49
1279	2607	48
1279	3371	243
1279	3817	37
1279	4049	43
1279	4618	40
1279	5927	84
1279	6249	40
1279	6756	68
1279	8836	73

1279 11237 64

1279 13540 106

1279 13632 176

1279 13633 108

1280 40 322

1280 55 1773

1280 58 784

1280 59 615

1280 72 1057

1280 76 532

1280 89 590

1280 154 2790

1280 159 550

1280 167 1118

1280 172 1094

1280 173 709

1280 190 2684

1280 203 560

1280 221 665

1280 222 640

1280 229 878

1280 233 376

1280 288 542

1280 298 519

1280 430 304

1280 463 316

1280 467 370

1280 511 1422

1280 533 595

1280 535 1092

1280 689 1001
1280 701 676
1280 716 788
1280 789 421
1280 851 2190
1280 906 428
1280 911 469
1280 961 1530
1280 1075 1170
1280 1089 5979
1280 1090 354
1280 1231 350
1280 1591 1045
1280 2083 414
1280 3109 1300
1280 3754 455
1280 8656 2091
1280 9633 589
1280 10080 1020
1280 11230 2248
1280 11232 1295
1280 13634 569
1280 13635 523
1280 13636 403
1281 152 13
1281 188 8
1281 198 15
1281 370 9
1281 408 15
1281 472 9

1281	488	15
1281	490	19
1281	497	9
1281	500	10
1281	517	14
1281	519	11
1281	614	9
1281	735	8
1281	769	10
1281	854	12
1281	868	10
1281	920	9
1281	982	7
1281	1118	19
1281	1131	12
1281	1170	15
1281	1372	7
1281	1639	7
1281	1701	8
1281	1854	20
1281	1873	10
1281	1974	10
1281	1998	8
1281	2006	7
1281	2206	16
1281	2352	7
1281	2380	21
1281	2794	14
1281	2855	10
1281	2941	8

1281	3240	20
1281	3563	7
1281	3928	10
1281	3940	9
1281	3946	8
1281	4179	10
1281	4359	7
1281	7639	14
1281	7947	8
1281	8350	9
1281	9344	7
1281	10310	8
1281	13637	45
1281	13638	11
1282	54	190
1282	56	179
1282	65	533
1282	67	172
1282	72	4891
1282	107	260
1282	154	658
1282	157	1398
1282	159	520
1282	190	353
1282	225	505
1282	229	275
1282	233	210
1282	289	213
1282	306	653
1282	329	464

1282	331	247
1282	425	616
1282	450	265
1282	547	202
1282	683	411
1282	907	149
1282	1062	267
1282	1075	6045
1282	1098	270
1282	1116	166
1282	1383	187
1282	1449	205
1282	1486	441
1282	1713	381
1282	1886	147
1282	1934	251
1282	2083	263
1282	2220	163
1282	3109	2906
1282	3300	165
1282	3317	157
1282	3756	179
1282	5893	343
1282	5925	266
1282	6374	215
1282	8176	181
1282	8510	239
1282	11080	235
1282	13639	229
1282	13640	217

1282	13641	183
1282	13642	162
1282	13643	160
1282	13644	157
1283	89	3156
1283	182	801
1283	190	1082
1283	191	696
1283	217	2283
1283	288	903
1283	289	2881
1283	290	738
1283	291	2057
1283	292	912
1283	298	1095
1283	300	2719
1283	305	941
1283	307	1558
1283	311	1254
1283	318	2176
1283	321	6094
1283	324	2592
1283	325	1711
1283	326	1143
1283	333	1134
1283	344	10586
1283	349	1440
1283	362	2485
1283	456	988
1283	461	2349

1283	471	1081
1283	498	5366
1283	523	1227
1283	679	5238
1283	680	1569
1283	681	5346
1283	686	1165
1283	689	1032
1283	701	685
1283	908	1344
1283	1043	1507
1283	1054	1198
1283	1243	1832
1283	1459	5085
1283	1470	2149
1283	1590	1390
1283	1684	2998
1283	1685	1417
1283	4435	778
1283	5032	713
1283	5266	729
1283	6527	693
1283	10060	685
1283	13645	675
1284	227	218
1284	444	1062
1284	603	528
1284	616	1169
1284	744	1361
1284	748	852

1284	772 276	
1284	778 535	
1284	782 217	
1284	1073	314
1284	1098	296
1284	1099	382
1284	1222	257
1284	1410	220
1284	1969	561
1284	2146	210
1284	2161	255
1284	2500	209
1284	2504	588
1284	2667	2490
1284	2668	412
1284	2669	301
1284	2671	1277
1284	2673	458
1284	2691	204
1284	4865	1031
1284	5411	356
1284	6092	182
1284	6405	201
1284	7468	198
1284	7493	450
1284	7694	257
1284	8786	428
1284	8868	197
1284	11128	614
1284	11948	295

1284	12020	204
1284	12337	516
1284	13646	568
1284	13647	566
1284	13648	391
1284	13649	354
1284	13650	336
1284	13651	303
1284	13652	300
1284	13653	296
1284	13654	228
1284	13655	199
1284	13656	197
1284	13657	188
1286	81	386
1286	173	351
1286	207	283
1286	225	594
1286	229	341
1286	236	324
1286	238	362
1286	416	498
1286	418	507
1286	419	370
1286	433	260
1286	441	454
1286	863	405
1286	1130	268
1286	1151	437
1286	1163	323

1286	1390	386
1286	1396	477
1286	1412	270
1286	1504	427
1286	1964	779
1286	2139	559
1286	2402	518
1286	2510	274
1286	2536	867
1286	2538	315
1286	2542	486
1286	3279	419
1286	3280	365
1286	3294	606
1286	3299	461
1286	3411	383
1286	3884	345
1286	4092	545
1286	5414	408
1286	5420	271
1286	5549	495
1286	7071	268
1286	11181	341
1286	12051	362
1286	13039	662
1286	13054	457
1286	13665	859
1286	13666	816
1286	13667	432
1286	13668	400

1286	13669	377
1286	13670	340
1286	13671	328
1286	13672	288
1287	3	330
1287	6	205
1287	12	271
1287	15	77
1287	25	321
1287	47	40
1287	48	163
1287	825	79
1287	843	61
1287	923	117
1287	953	423
1287	1044	97
1287	1099	37
1287	1259	500
1287	1260	50
1287	1263	92
1287	1276	52
1287	1281	37
1287	1521	586
1287	2014	123
1287	2432	147
1287	2756	81
1287	2765	104
1287	2835	51
1287	2970	40
1287	3239	68

1287	4273	90
1287	5126	53
1287	5667	282
1287	6289	145
1287	7003	62
1287	7639	115
1287	7925	174
1287	9226	96
1287	9540	194
1287	11159	52
1287	11372	137
1287	13673	197
1287	13674	182
1287	13675	163
1287	13676	86
1287	13677	79
1287	13678	66
1287	13679	54
1287	13680	52
1287	13681	49
1287	13682	49
1287	13683	47
1287	13684	46
1287	13685	45
1288	67	95
1288	96	159
1288	184	289
1288	206	414
1288	207	232
1288	212	295

1288	223 131	
1288	227 143	
1288	230 215	
1288	245 399	
1288	306 157	
1288	475 91	
1288	605 177	
1288	715 102	
1288	718 352	
1288	1186	266
1288	1415	81
1288	1672	181
1288	1941	238
1288	1972	443
1288	2265	80
1288	3316	286
1288	3466	167
1288	3749	206
1288	3750	93
1288	4369	244
1288	5626	83
1288	5783	156
1288	8121	356
1288	11754	370
1288	11971	167
1288	12565	221
1288	13686	493
1288	13687	474
1288	13688	461
1288	13689	247

1288	13690	225
1288	13691	206
1288	13692	202
1288	13693	184
1288	13694	182
1288	13695	163
1288	13696	162
1288	13697	146
1288	13698	141
1288	13699	132
1288	13700	124
1288	13701	106
1288	13702	105
1288	13703	90
1289	121 384	
1289	138 320	
1289	154 2678	
1289	196 495	
1289	222 642	
1289	225 262	
1289	231 437	
1289	237 279	
1289	418 630	
1289	433 433	
1289	440 2224	
1289	444 484	
1289	615 352	
1289	621 727	
1289	622 1341	
1289	632 460	

1289	748 339	
1289	977 260	
1289	1109	859
1289	1418	302
1289	1504	331
1289	1947	624
1289	1950	329
1289	1964	447
1289	1965	410
1289	2139	841
1289	2267	383
1289	2279	964
1289	2282	299
1289	2593	383
1289	3673	295
1289	3691	466
1289	3693	1791
1289	3694	350
1289	3700	570
1289	3707	856
1289	6189	294
1289	6295	585
1289	6409	414
1289	6910	1058
1289	7742	623
1289	8706	261
1289	10108	345
1289	13045	409
1289	13145	416
1289	13704	528

1289	13705	428
1289	13706	360
1289	13707	275
1289	13708	260
1290	316 2	
1290	362 1	
1290	426 1	
1290	599 1	
1290	697 1	
1290	961 2	
1290	1383	2
1290	1687	2
1290	2277	4
1290	2632	1
1290	3081	1
1290	3110	4
1290	3119	1
1290	3460	1
1290	3466	1
1290	4447	1
1290	5790	1
1290	5914	1
1290	6214	1
1290	6417	1
1290	6454	1
1290	7287	1
1290	8592	2
1290	8711	2
1290	9470	2
1290	12194	1

1290	13709	2
1290	13710	2
1290	13711	1
1290	13712	1
1290	13713	1
1290	13714	1
1291	55	58
1291	67	339
1291	77	50
1291	95	317
1291	183	46
1291	192	95
1291	227	177
1291	250	49
1291	257	171
1291	261	194
1291	265	106
1291	266	56
1291	270	54
1291	285	92
1291	439	72
1291	701	50
1291	703	152
1291	913	48
1291	917	84
1291	924	56
1291	928	93
1291	962	174
1291	985	104
1291	1081	56

1291	1098	235
1291	1243	97
1291	1390	66
1291	1664	49
1291	1721	132
1291	1764	446
1291	1772	80
1291	1833	47
1291	1834	80
1291	1935	80
1291	2277	619
1291	2419	186
1291	2460	248
1291	2552	72
1291	3063	132
1291	4709	167
1291	5150	128
1291	5458	165
1291	5745	173
1291	11763	106
1291	11764	500
1291	13715	127
1291	13716	83
1291	13717	56
1291	13718	53
1291	13719	48
1292	65 9	
1292	299 5	
1292	300 21	
1292	307 8	

1292	318	18
1292	321	47
1292	325	7
1292	456	21
1292	461	53
1292	465	6
1292	523	12
1292	533	23
1292	546	55
1292	681	96
1292	735	5
1292	793	45
1292	908	81
1292	969	9
1292	980	7
1292	1048	37
1292	1054	30
1292	1059	5
1292	1400	116
1292	1426	6
1292	1453	39
1292	1590	21
1292	1597	33
1292	1639	16
1292	1684	16
1292	2606	16
1292	2801	23
1292	3065	7
1292	3071	6
1292	3293	46

1292	3327	25
1292	3497	17
1292	4291	7
1292	4294	14
1292	4704	60
1292	5036	7
1292	7838	6
1292	8555	5
1292	8675	6
1292	11010	19
1292	13063	14
1292	13720	30
1292	13721	17
1292	13722	10
1292	13723	8
1292	13724	5
1293	7	116
1293	166	65
1293	173	106
1293	198	112
1293	209	122
1293	228	60
1293	229	109
1293	234	32
1293	302	50
1293	333	39
1293	371	125
1293	378	144
1293	403	118
1293	405	74

1293	488	95
1293	493	84
1293	497	95
1293	498	82
1293	499	62
1293	503	93
1293	518	50
1293	536	120
1293	537	79
1293	540	44
1293	818	52
1293	854	60
1293	930	64
1293	966	35
1293	978	48
1293	1034	48
1293	1104	71
1293	1179	123
1293	1180	36
1293	1195	85
1293	1249	41
1293	1672	247
1293	1803	61
1293	1854	143
1293	1863	58
1293	2380	61
1293	2432	86
1293	2785	114
1293	2956	35
1293	2959	54

1293	3328	38
1293	3928	91
1293	4255	40
1293	4382	75
1293	5079	81
1293	9944	41
1294	71	28
1294	89	43
1294	97	23
1294	209	51
1294	289	33
1294	291	384
1294	292	30
1294	300	60
1294	301	34
1294	302	66
1294	304	1104
1294	313	17
1294	321	22
1294	324	114
1294	403	19
1294	458	36
1294	461	17
1294	498	24
1294	525	22
1294	669	38
1294	679	942
1294	681	198
1294	686	114
1294	690	19

1294	691	39
1294	701	24
1294	790	22
1294	1042	21
1294	1090	18
1294	1243	23
1294	1431	24
1294	1447	30
1294	1461	105
1294	1680	42
1294	1934	61
1294	2025	27
1294	2177	69
1294	2346	23
1294	2653	18
1294	2977	23
1294	4260	41
1294	4291	32
1294	4491	25
1294	4700	19
1294	4703	58
1294	5760	41
1294	7838	22
1294	7897	56
1294	9155	18
1294	11717	106
1295	56	262
1295	81	394
1295	154	3393
1295	159	404

1295	162 322	
1295	163 449	
1295	173 323	
1295	195 1565	
1295	207 2069	
1295	212 215	
1295	215 302	
1295	217 726	
1295	225 782	
1295	227 1052	
1295	230 957	
1295	412 247	
1295	418 1350	
1295	424 1477	
1295	428 369	
1295	429 441	
1295	430 326	
1295	439 3086	
1295	441 1752	
1295	533 916	
1295	609 526	
1295	615 3982	
1295	618 420	
1295	632 388	
1295	703 295	
1295	1048	630
1295	1090	378
1295	1239	244
1295	1390	358
1295	1418	364

1295	1515	389
1295	1755	424
1295	1766	693
1295	1773	417
1295	1957	799
1295	1966	355
1295	2265	341
1295	2271	253
1295	2542	424
1295	2608	343
1295	3400	278
1295	4164	511
1295	5310	338
1295	8713	227
1295	10418	357
1295	13725	517
1296	12	56
1296	15	339
1296	152	100
1296	154	92
1296	163	52
1296	186	1511
1296	391	153
1296	785	140
1296	809	50
1296	811	103
1296	812	687
1296	813	19372
1296	815	585
1296	821	385

1296	824 71	
1296	834 396	
1296	835 187	
1296	855 102	
1296	920 134	
1296	944 71	
1296	956 383	
1296	957 440	
1296	1118	99
1296	1131	106
1296	1281	54
1296	1338	375
1296	1411	47
1296	1541	69
1296	1790	162
1296	1861	79
1296	2151	75
1296	2360	94
1296	2370	494
1296	2374	386
1296	2378	432
1296	2379	370
1296	2385	250
1296	2744	182
1296	2963	1911
1296	2966	60
1296	2969	118
1296	4605	372
1296	4612	406
1296	4971	131

1296	6811	191
1296	9410	543
1296	11814	57
1296	13726	503
1296	13727	275
1296	13728	186
1298	67	1989
1298	89	577
1298	257	104
1298	288	548
1298	289	1555
1298	290	56
1298	291	415
1298	292	311
1298	299	109
1298	300	105
1298	302	63
1298	309	512
1298	310	100
1298	317	146
1298	318	161
1298	326	71
1298	327	96
1298	330	194
1298	344	435
1298	352	250
1298	360	1553
1298	386	77
1298	389	181
1298	455	182

1298	458	119
1298	461	487
1298	466	262
1298	468	57
1298	484	56
1298	686	90
1298	701	63
1298	793	62
1298	906	93
1298	907	237
1298	1444	108
1298	1447	144
1298	1459	176
1298	1489	3735
1298	1589	94
1298	1684	61
1298	2018	179
1298	2437	143
1298	3397	67
1298	4562	137
1298	6459	62
1298	6693	85
1298	7868	56
1298	9847	64
1298	10989	68
1298	13730	54
1299	55	176
1299	67	228
1299	89	1107
1299	97	144

1299	163 151	
1299	289 1878	
1299	291 1113	
1299	292 894	
1299	295 113	
1299	298 191	
1299	300 696	
1299	302 211	
1299	318 290	
1299	333 454	
1299	340 1590	
1299	342 722	
1299	344 340	
1299	378 1237	
1299	461 461	
1299	518 193	
1299	523 637	
1299	535 448	
1299	542 364	
1299	646 370	
1299	701 221	
1299	790 517	
1299	802 254	
1299	909 561	
1299	917 136	
1299	930 480	
1299	946 394	
1299	955 877	
1299	1114	2968
1299	1447	110

1299	1580	135
1299	2014	163
1299	2044	2209
1299	2544	222
1299	3460	146
1299	4135	137
1299	4286	132
1299	5461	181
1299	5768	158
1299	6539	153
1299	6862	1287
1299	8645	500
1299	13731	1852
1299	13732	1572
1299	13733	223
1299	13734	177
1300	55	5186
1300	67	9589
1300	71	517
1300	89	2017
1300	251	1750
1300	257	3918
1300	288	2398
1300	289	24283
1300	291	1007
1300	292	1506
1300	295	943
1300	296	472
1300	298	947
1300	300	2931

1300	302	1961
1300	306	1273
1300	318	3272
1300	329	1313
1300	333	481
1300	344	926
1300	349	755
1300	374	1278
1300	375	3564
1300	455	669
1300	464	1371
1300	466	6381
1300	523	991
1300	524	2127
1300	525	720
1300	528	927
1300	548	677
1300	679	1829
1300	798	473
1300	906	1011
1300	972	2005
1300	1556	917
1300	1601	2305
1300	2022	1314
1300	2024	761
1300	2094	1628
1300	2102	11473
1300	2521	1287
1300	2523	2517
1300	2977	639

1300	3324	6140
1300	3575	856
1300	4350	592
1300	6063	796
1300	13735	831
1300	13736	591
1301	55	3294
1301	67	29317
1301	89	16982
1301	157	1085
1301	251	494
1301	257	3293
1301	265	331
1301	288	1786
1301	289	60180
1301	291	1669
1301	292	1470
1301	293	433
1301	295	3652
1301	298	2932
1301	301	748
1301	302	3273
1301	304	3875
1301	306	1014
1301	325	313
1301	340	10362
1301	349	810
1301	375	2183
1301	389	1247
1301	430	540

1301	523	1881
1301	524	6330
1301	525	3240
1301	534	288
1301	535	393
1301	680	1712
1301	890	1935
1301	903	1874
1301	906	8591
1301	972	1640
1301	1098	545
1301	1580	271
1301	1601	1073
1301	2022	1050
1301	2024	2502
1301	2102	5972
1301	3057	944
1301	3200	267
1301	3206	265
1301	3324	654
1301	3488	1374
1301	3575	5488
1301	4350	371
1301	5036	4187
1301	8363	509
1301	13735	5559
1302	65	595
1302	97	105
1302	154	654
1302	190	169

1302	196	281
1302	204	106
1302	210	220
1302	212	259
1302	227	1458
1302	229	294
1302	316	148
1302	355	687
1302	366	162
1302	418	124
1302	424	662
1302	428	107
1302	430	91
1302	439	889
1302	447	112
1302	615	264
1302	632	87
1302	683	394
1302	709	119
1302	716	141
1302	718	130
1302	858	104
1302	903	181
1302	1048	172
1302	1116	259
1302	1242	142
1302	1375	135
1302	1400	199
1302	1424	545
1302	1511	121

1302	1515	192
1302	1556	464
1302	1755	545
1302	1761	431
1302	1766	509
1302	2277	403
1302	3057	86
1302	3171	105
1302	3386	256
1302	3488	252
1302	3750	79
1302	5416	210
1302	5439	255
1302	5446	118
1302	8155	448
1302	13737	137
1303	154 866	
1303	162 284	
1303	173 265	
1303	190 757	
1303	195 607	
1303	196 435	
1303	199 199	
1303	205 164	
1303	207 196	
1303	217 786	
1303	219 537	
1303	221 152	
1303	225 686	
1303	227 176	

1303	228	151
1303	229	325
1303	416	824
1303	418	261
1303	421	308
1303	424	223
1303	429	146
1303	433	159
1303	436	389
1303	439	448
1303	441	420
1303	450	187
1303	533	761
1303	602	199
1303	615	2884
1303	632	454
1303	964	238
1303	1106	280
1303	1109	481
1303	1163	282
1303	1418	227
1303	1423	146
1303	1504	266
1303	1510	252
1303	1515	179
1303	1779	171
1303	1957	444
1303	2531	165
1303	2542	181
1303	4384	167

1303	5979	213
1303	6146	214
1303	7219	164
1303	9955	475
1303	12583	350
1303	13738	425
1304	89	1766
1304	191	78
1304	288	1066
1304	289	1519
1304	292	154
1304	299	118
1304	300	625
1304	302	237
1304	327	128
1304	332	111
1304	333	1976
1304	342	272
1304	344	185
1304	378	135
1304	386	96
1304	458	113
1304	461	960
1304	464	150
1304	466	938
1304	469	112
1304	498	135
1304	528	301
1304	543	141
1304	646	2251

1304	686	581
1304	699	92
1304	704	480
1304	790	446
1304	793	200
1304	802	417
1304	1428	1889
1304	1429	185
1304	1430	256
1304	1452	161
1304	1458	1563
1304	2020	1696
1304	2082	149
1304	2498	94
1304	2548	79
1304	2990	147
1304	3073	129
1304	3462	95
1304	4562	261
1304	4811	2692
1304	7340	77
1304	13739	464
1304	13740	298
1304	13741	219
1304	13742	136
1304	13743	83
1305	55	520
1305	67	156
1305	89	14356
1305	257	340

1305	288 4842
1305	289 1798
1305	291 164
1305	292 1820
1305	295 620
1305	298 255
1305	299 1643
1305	300 1803
1305	302 246
1305	306 286
1305	308 627
1305	311 165
1305	314 664
1305	317 184
1305	331 149
1305	332 167
1305	333 918
1305	349 332
1305	352 302
1305	458 178
1305	461 334
1305	464 187
1305	466 1482
1305	475 201
1305	523 193
1305	524 729
1305	543 1065
1305	575 159
1305	679 1831
1305	686 326

1305 689 263
1305 701 720
1305 802 232
1305 903 179
1305 915 566
1305 1032 229
1305 1037 2555
1305 1042 238
1305 1452 379
1305 1934 628
1305 1936 603
1305 2091 154
1305 2548 358
1305 2990 744
1305 3179 414
1305 6543 187
1307 89 34328
1308 55 1294
1308 65 777
1308 67 38278
1308 89 677
1308 157 16324
1308 251 755
1308 253 723
1308 257 6596
1308 265 375
1308 285 20393
1308 288 1169
1308 289 14132
1308 290 426

1308	291 1899
1308	292 2473
1308	293 768
1308	294 1093
1308	295 2134
1308	296 677
1308	299 1969
1308	301 582
1308	302 482
1308	305 355
1308	311 344
1308	316 320
1308	318 1473
1308	325 907
1308	329 494
1308	333 945
1308	349 550
1308	362 315
1308	378 425
1308	455 974
1308	465 424
1308	466 322
1308	518 350
1308	523 1182
1308	525 380
1308	701 645
1308	889 312
1308	903 528
1308	913 1060
1308	966 839

1308	1459	870
1308	1602	825
1308	2024	654
1308	2523	1286
1308	3575	408
1308	4915	436
1308	13749	521
1309	7	557
1309	154	502
1309	163	504
1309	190	727
1309	206	503
1309	220	1098
1309	226	5093
1309	233	701
1309	436	880
1309	512	1165
1309	599	613
1309	614	1033
1309	706	527
1309	707	535
1309	854	522
1309	856	618
1309	859	614
1309	867	2153
1309	881	1860
1309	1083	1238
1309	1104	513
1309	1118	1088
1309	1119	1867

1309	1131	2280
1309	1202	850
1309	1240	1305
1309	1412	550
1309	1504	699
1309	1778	809
1309	1974	530
1309	1977	914
1309	2362	480
1309	2484	571
1309	2493	477
1309	2698	577
1309	2823	472
1309	3475	673
1309	4058	790
1309	4069	603
1309	4356	905
1309	5296	477
1309	5549	1252
1309	7081	785
1309	7908	689
1309	13750	818
1309	13751	649
1309	13752	629
1309	13753	501
1309	13754	481
1309	13755	465
1310	166 3	
1310	197 1	
1310	198 2	

1310	257	1
1310	261	1
1310	289	4
1310	310	2
1310	333	1
1310	344	1
1310	461	1
1310	492	1
1310	498	5
1310	525	1
1310	534	1
1310	681	3
1310	701	3
1310	892	1
1310	903	2
1310	908	2
1310	953	1
1310	986	1
1310	1038	2
1310	1116	1
1310	1428	1
1310	1580	1
1310	2127	3
1310	2265	1
1310	2276	1
1310	2442	1
1310	2467	4
1310	2786	6
1310	2815	1
1310	2834	1

1310	3110	1
1310	3431	1
1310	3765	1
1310	4025	1
1310	5314	2
1310	6293	1
1310	7736	2
1310	8152	1
1310	10184	4
1310	13756	20
1310	13757	3
1310	13758	2
1310	13759	2
1310	13760	1
1310	13761	1
1310	13762	1
1310	13763	1
1311	89	114
1311	97	148
1311	157	116
1311	247	108
1311	257	764
1311	258	165
1311	262	115
1311	266	97
1311	279	123
1311	287	208
1311	288	423
1311	292	451
1311	294	154

1311 295 740

1311 299 132

1311 301 315

1311 302 93

1311 314 155

1311 317 118

1311 322 109

1311 326 75

1311 332 874

1311 485 119

1311 701 104

1311 901 77

1311 913 252

1311 1934 95

1311 1935 112

1311 1936 169

1311 1937 310

1311 2393 444

1311 2515 69

1311 3500 219

1311 4560 171

1311 4566 518

1311 4572 84

1311 4574 189

1311 6562 137

1311 8188 117

1311 8604 97

1311 8765 240

1311 8768 71

1311 10988 214

1311	10989	184
1311	11082	100
1311	12403	139
1311	13446	155
1311	13764	113
1311	13765	89
1311	13766	74
1312	198	1412
1312	209	93
1312	331	43
1312	371	485
1312	405	94
1312	408	444
1312	475	221
1312	490	342
1312	491	122
1312	493	337
1312	497	107
1312	498	86
1312	499	68
1312	500	168
1312	707	171
1312	779	116
1312	808	410
1312	818	707
1312	830	676
1312	850	42
1312	978	292
1312	1044	2901
1312	1105	69

1312	1145	672
1312	1185	166
1312	1246	96
1312	1378	44
1312	1853	276
1312	1974	43
1312	2506	41
1312	2835	404
1312	2836	158
1312	2940	780
1312	3563	157
1312	3929	83
1312	4549	111
1312	5969	293
1312	7728	41
1312	9765	127
1312	13767	275
1312	13768	132
1312	13769	129
1312	13770	119
1312	13771	89
1312	13772	56
1312	13773	55
1312	13774	52
1312	13775	48
1312	13776	44
1312	13777	38
1313	64	244
1313	84	294
1313	152	558

1313	154	298
1313	162	390
1313	170	249
1313	211	286
1313	223	254
1313	416	302
1313	431	339
1313	434	353
1313	440	378
1313	622	277
1313	632	551
1313	748	327
1313	769	592
1313	858	377
1313	1418	370
1313	1633	255
1313	1813	264
1313	1949	295
1313	1954	256
1313	1964	406
1313	2139	293
1313	2151	368
1313	2281	323
1313	2408	242
1313	2424	333
1313	2582	344
1313	2583	313
1313	2640	487
1313	2771	397
1313	2828	556

1313	3693	244
1313	3700	267
1313	3810	390
1313	4079	333
1313	4448	325
1313	5423	521
1313	6830	296
1313	7147	341
1313	7665	278
1313	10196	244
1313	11181	310
1313	12723	495
1313	13050	261
1313	13398	544
1313	13454	447
1313	13778	271
1313	13779	257
1315	1845	1
1315	2144	16
1315	7259	1
1315	7472	1
1315	8975	2
1315	9117	1
1315	10041	1
1315	10364	1
1315	10394	1
1315	11370	9
1315	13790	7
1315	13791	7
1315	13792	6

1315	13793	6
1315	13794	6
1315	13795	6
1315	13796	6
1315	13797	6
1315	13798	5
1315	13799	5
1315	13800	5
1315	13801	4
1315	13802	3
1315	13803	3
1315	13804	3
1315	13805	3
1315	13806	3
1315	13807	3
1315	13808	2
1315	13809	2
1315	13810	2
1315	13811	2
1315	13812	1
1315	13813	1
1315	13814	1
1315	13815	1
1315	13816	1
1315	13817	1
1315	13818	1
1315	13819	1
1315	13820	1
1315	13821	1
1315	13822	1

1315	13823	1
1315	13824	1
1315	13825	1
1315	13826	1
1315	13827	1
1315	13828	1
1315	13829	1
1316	67	169
1316	89	267
1316	97	168
1316	157	279
1316	187	105
1316	192	203
1316	212	329
1316	227	244
1316	235	133
1316	289	229
1316	291	106
1316	300	193
1316	302	119
1316	333	244
1316	344	247
1316	461	292
1316	475	104
1316	498	104
1316	554	149
1316	599	107
1316	679	904
1316	681	130
1316	715	243

1316	718	267
1316	733	129
1316	734	310
1316	851	187
1316	906	120
1316	951	119
1316	959	141
1316	986	157
1316	998	120
1316	1242	115
1316	1244	176
1316	1414	144
1316	1633	116
1316	2029	106
1316	2444	142
1316	2531	151
1316	2902	160
1316	3306	140
1316	3319	286
1316	3889	111
1316	4620	147
1316	5000	183
1316	6784	131
1316	13069	116
1316	13830	136
1316	13831	122
1316	13832	108
1318	67	2170
1318	89	6550
1318	173	389

1318	209 2437
1318	227 661
1318	228 1757
1318	288 335
1318	289 523
1318	292 216
1318	295 1875
1318	298 729
1318	300 2858
1318	306 223
1318	324 1319
1318	333 198
1318	430 682
1318	464 627
1318	466 2395
1318	470 718
1318	498 2213
1318	526 1553
1318	528 1742
1318	532 203
1318	537 584
1318	542 1995
1318	544 262
1318	546 2507
1318	646 1530
1318	679 1303
1318	680 408
1318	693 1103
1318	701 477
1318	714 314

1318	980	249
1318	985	276
1318	1047	240
1318	1246	965
1318	1481	372
1318	1486	1119
1318	1760	345
1318	3033	451
1318	3205	789
1318	3247	310
1318	3741	1408
1318	3949	396
1318	4385	2372
1318	4576	208
1318	5926	617
1318	6121	264
1318	13838	243
1319	65	89
1319	154	129
1319	173	260
1319	190	157
1319	203	112
1319	207	386
1319	209	117
1319	215	117
1319	227	149
1319	228	96
1319	230	1638
1319	234	107
1319	362	107

1319 383 106

1319 405 118

1319 418 107

1319 475 74

1319 511 112

1319 521 115

1319 533 130

1319 546 143

1319 689 113

1319 690 79

1319 704 115

1319 707 67

1319 857 1465

1319 1055 129

1319 1179 331

1319 1249 63

1319 1390 148

1319 1400 1002

1319 1406 72

1319 1512 152

1319 1513 218

1319 1516 89

1319 1520 63

1319 1790 153

1319 1814 144

1319 3627 66

1319 3646 148

1319 3682 127

1319 3739 119

1319 3915 112

1319	4394	119
1319	5785	79
1319	6453	78
1319	7911	79
1319	13839	122
1319	13840	97
1319	13841	66
1321	56	83
1321	59	105
1321	207	784
1321	221	230
1321	227	175
1321	228	730
1321	229	573
1321	298	389
1321	424	506
1321	450	79
1321	533	177
1321	534	244
1321	538	147
1321	562	172
1321	585	170
1321	703	304
1321	706	1500
1321	709	235
1321	718	221
1321	724	99
1321	728	241
1321	735	262
1321	903	103

1321	969	212
1321	1048	121
1321	1369	123
1321	1412	384
1321	1510	405
1321	1520	595
1321	1556	81
1321	1873	120
1321	1988	218
1321	2142	481
1321	2176	242
1321	2280	100
1321	2364	149
1321	2789	91
1321	2821	144
1321	2855	376
1321	3400	85
1321	3469	78
1321	3475	472
1321	4115	178
1321	4251	382
1321	6678	96
1321	8309	79
1321	13842	605
1321	13843	173
1321	13844	84
1321	13845	84
1322	95	182
1322	159	343
1322	183	265

1322	188	276
1322	233	363
1322	238	308
1322	370	188
1322	605	294
1322	748	847
1322	769	203
1322	845	490
1322	854	158
1322	949	545
1322	1119	507
1322	1131	568
1322	1191	245
1322	1203	527
1322	1216	836
1322	1221	275
1322	1401	155
1322	1413	198
1322	2151	964
1322	2172	290
1322	2469	1147
1322	2962	305
1322	3132	193
1322	3417	174
1322	3528	2352
1322	3720	388
1322	3727	530
1322	3788	1276
1322	3810	248
1322	4615	173

1322	4787	539
1322	5061	180
1322	5071	246
1322	6575	627
1322	7326	263
1322	8696	200
1322	9354	452
1322	9783	163
1322	11477	220
1322	13846	1190
1322	13847	694
1322	13848	440
1322	13849	424
1322	13850	276
1322	13851	222
1322	13852	188
1322	13853	176
1323	7	258
1323	209	244
1323	230	147
1323	412	7182
1323	460	200
1323	486	1004
1323	498	1078
1323	501	1623
1323	537	549
1323	540	147
1323	687	465
1323	693	160
1323	755	306

1323	1040	179
1323	1186	1002
1323	1192	273
1323	1239	166
1323	1378	544
1323	1541	238
1323	1866	164
1323	2154	349
1323	2699	552
1323	3078	144
1323	3341	1032
1323	3398	426
1323	3827	233
1323	4896	271
1323	4917	270
1323	5310	169
1323	5922	225
1323	6010	298
1323	6196	465
1323	6769	203
1323	7329	149
1323	7743	155
1323	9986	171
1323	13237	176
1323	13854	1068
1323	13855	766
1323	13856	588
1323	13857	408
1323	13858	405
1323	13859	331

1323	13860	295
1323	13861	228
1323	13862	199
1323	13863	182
1323	13864	156
1324	47	367
1324	163	72
1324	227	117
1324	383	229
1324	429	311
1324	841	55
1324	917	157
1324	923	58
1324	949	167
1324	1254	58
1324	1521	115
1324	1528	103
1324	1533	55
1324	1535	77
1324	1992	75
1324	2029	82
1324	2490	164
1324	2749	257
1324	2772	68
1324	2962	91
1324	2969	60
1324	3417	90
1324	3509	83
1324	4271	179
1324	4285	66

1324	4335	57
1324	4605	125
1324	4611	65
1324	4965	447
1324	6258	94
1324	6658	71
1324	8341	94
1324	9122	122
1324	10676	59
1324	11372	246
1324	13478	175
1324	13865	468
1324	13866	323
1324	13867	206
1324	13868	195
1324	13869	149
1324	13870	132
1324	13871	132
1324	13872	118
1324	13873	98
1324	13874	81
1324	13875	79
1324	13876	75
1324	13877	70
1324	13878	64
1325	162 745	
1325	226 442	
1325	383 1479	
1325	632 294	
1325	763 434	

1325	853 675	
1325	861 374	
1325	867 361	
1325	871 2500	
1325	881 711	
1325	1131	323
1325	1202	544
1325	1418	661
1325	1513	288
1325	1703	305
1325	1974	324
1325	2362	583
1325	2835	250
1325	3364	256
1325	3438	278
1325	4080	269
1325	4356	326
1325	4359	467
1325	5911	325
1325	6730	697
1325	7074	288
1325	7319	274
1325	8663	677
1325	9344	487
1325	9916	255
1325	9920	448
1325	11283	544
1325	12325	354
1325	12583	328
1325	13396	259

1325	13753	345
1325	13879	1104
1325	13880	587
1325	13881	507
1325	13882	460
1325	13883	396
1325	13884	343
1325	13885	332
1325	13886	327
1325	13887	324
1325	13888	284
1325	13889	279
1325	13890	274
1325	13891	258
1325	13892	253
1327	51	69
1327	59	11
1327	61	10
1327	63	8
1327	68	11
1327	72	12
1327	91	9
1327	95	8
1327	98	19
1327	99	10
1327	100	9
1327	175	9
1327	187	19
1327	193	33
1327	253	18

1327	877 9	
1327	993 16	
1327	994 31	
1327	997 26	
1327	999 29	
1327	1000	25
1327	1001	17
1327	1006	28
1327	1014	14
1327	1015	17
1327	1019	17
1327	1027	9
1327	1122	15
1327	1884	8
1327	1886	9
1327	1889	15
1327	1891	21
1327	1892	13
1327	2027	11
1327	2469	28
1327	2556	14
1327	2559	26
1327	2562	46
1327	2567	18
1327	2936	14
1327	2937	18
1327	3201	8
1327	4978	12
1327	5258	10
1327	5260	10

1327	6073	11
1327	7927	9
1327	9255	10
1327	13900	15
1327	13901	9
1329	65	460
1329	180	125
1329	182	426
1329	202	799
1329	217	384
1329	219	977
1329	229	1001
1329	302	125
1329	348	335
1329	442	276
1329	511	1483
1329	533	252
1329	618	984
1329	1112	125
1329	1117	315
1329	1121	264
1329	1123	1138
1329	1130	491
1329	1160	164
1329	1163	98
1329	1375	198
1329	1515	415
1329	1755	426
1329	1770	147
1329	1772	445

1329	1944	593
1329	2419	166
1329	2524	385
1329	2628	914
1329	2815	2596
1329	3003	203
1329	3097	314
1329	3258	323
1329	5548	129
1329	6297	437
1329	7111	120
1329	7119	402
1329	7352	664
1329	7675	200
1329	11229	96
1329	11589	111
1329	11598	1132
1329	13417	209
1329	13905	870
1329	13906	373
1329	13907	227
1329	13908	218
1329	13909	181
1329	13910	137
1329	13911	120
1330	206 632	
1330	220 527	
1330	578 1642	
1330	610 934	
1330	1230	582

1330	1427	528
1330	1606	498
1330	1826	439
1330	1849	1191
1330	1946	438
1330	1953	454
1330	1963	839
1330	2698	706
1330	3776	474
1330	3778	513
1330	5119	627
1330	5169	938
1330	6831	964
1330	7073	714
1330	7259	416
1330	9361	866
1330	10201	706
1330	10333	1244
1330	10336	472
1330	10363	1019
1330	11935	809
1330	12137	1513
1330	13813	479
1330	13815	603
1330	13912	1152
1330	13913	1055
1330	13914	830
1330	13915	727
1330	13916	721
1330	13917	695

1330	13918	687
1330	13919	675
1330	13920	570
1330	13921	530
1330	13922	502
1330	13923	454
1330	13924	449
1330	13925	448
1330	13926	444
1330	13927	444
1330	13928	439
1330	13929	436
1330	13930	431
1330	13931	418
1330	13932	412
1331	152 1955	
1331	159 444	
1331	163 587	
1331	202 1491	
1331	212 430	
1331	227 1383	
1331	426 737	
1331	436 707	
1331	439 3273	
1331	562 584	
1331	889 515	
1331	920 1466	
1331	928 391	
1331	1119	1002
1331	1135	411

1331	1237	831
1331	1412	465
1331	1464	672
1331	1631	455
1331	1755	985
1331	1764	537
1331	3057	506
1331	3272	488
1331	3420	1320
1331	4801	703
1331	5079	487
1331	5150	517
1331	5416	781
1331	6046	694
1331	6224	467
1331	6529	470
1331	7051	424
1331	9700	398
1331	9713	406
1331	10418	511
1331	11434	384
1331	11485	556
1331	11511	527
1331	11935	377
1331	13933	2022
1331	13934	1006
1331	13935	854
1331	13936	779
1331	13937	614
1331	13938	610

1331	13939	503
1331	13940	478
1331	13941	473
1331	13942	424
1331	13943	404
1332	67	635
1332	89	15731
1332	97	1122
1332	191	801
1332	288	1880
1332	289	5528
1332	290	1568
1332	291	1742
1332	292	1707
1332	294	1264
1332	295	1127
1332	300	1253
1332	305	704
1332	306	683
1332	308	560
1332	314	495
1332	325	901
1332	328	624
1332	333	1543
1332	349	1094
1332	378	1057
1332	461	1283
1332	464	1049
1332	466	4164
1332	484	497

1332	485 664	
1332	526 777	
1332	528 504	
1332	544 1101	
1332	674 1584	
1332	679 569	
1332	686 657	
1332	1042	540
1332	1051	758
1332	1433	829
1332	1444	832
1332	1456	2708
1332	1457	532
1332	1602	605
1332	3316	528
1332	3491	716
1332	3575	647
1332	3576	649
1332	4562	593
1332	4814	532
1332	5684	631
1332	7340	784
1332	8002	551
1332	13944	1005
1332	13945	746
1333	65 567	
1333	154 334	
1333	182 204	
1333	195 323	
1333	203 370	

1333 207 412
1333 212 183
1333 217 2126
1333 221 742
1333 222 2368
1333 225 201
1333 227 1013
1333 228 171
1333 428 195
1333 432 331
1333 434 238
1333 436 170
1333 615 685
1333 618 1162
1333 622 329
1333 629 1337
1333 712 209
1333 716 220
1333 857 320
1333 898 265
1333 1048 396
1333 1090 363
1333 1155 341
1333 1195 206
1333 1239 859
1333 1242 175
1333 1375 336
1333 1502 249
1333 2277 222
1333 2537 235

1333	3206	180
1333	4114	330
1333	4115	314
1333	4444	369
1333	5439	238
1333	6202	171
1333	7190	227
1333	7675	174
1333	8493	258
1333	9823	280
1333	12154	215
1333	12787	357
1333	13516	569
1333	13939	277
1333	13946	224
1334	344 1	
1334	543 1	
1334	8390	1
1334	11818	2
1335	225 67	
1335	233 483	
1335	447 115	
1335	603 916	
1335	610 146	
1335	616 309	
1335	625 259	
1335	744 1069	
1335	748 177	
1335	756 54	
1335	859 58	

1335	1120	60
1335	1123	485
1335	1164	68
1335	1222	140
1335	1491	314
1335	1504	75
1335	1708	297
1335	1770	54
1335	1945	68
1335	2670	56
1335	2672	236
1335	3216	57
1335	3528	107
1335	6151	71
1335	6159	110
1335	6161	632
1335	6163	103
1335	6168	63
1335	7096	152
1335	7123	107
1335	7352	53
1335	7468	135
1335	9321	87
1335	10936	138
1335	11784	134
1335	13317	55
1335	13910	59
1335	13947	4669
1335	13948	244
1335	13949	201

1335	13950	182
1335	13951	115
1335	13952	108
1335	13953	98
1335	13954	79
1335	13955	60
1335	13956	58
1335	13957	53
1335	13958	53
1336	7	461
1336	51	276
1336	70	128
1336	72	2142
1336	88	425
1336	99	91
1336	154	199
1336	159	4440
1336	162	502
1336	163	491
1336	173	320
1336	198	122
1336	203	172
1336	220	406
1336	226	86
1336	233	590
1336	234	175
1336	235	147
1336	377	112
1336	441	280
1336	450	516

1336	511	171
1336	515	413
1336	599	164
1336	715	87
1336	755	144
1336	859	149
1336	865	130
1336	959	204
1336	969	875
1336	982	305
1336	1055	125
1336	1061	216
1336	1083	644
1336	1099	112
1336	1104	195
1336	1249	111
1336	1412	109
1336	1713	148
1336	1974	94
1336	1981	147
1336	1984	162
1336	3053	212
1336	3625	186
1336	3650	140
1336	3683	286
1336	4069	170
1336	7038	122
1336	13016	88
1336	13959	127
1337	67	565

1337	70	391
1337	72	918
1337	89	828
1337	173	1858
1337	190	422
1337	207	1010
1337	210	416
1337	217	975
1337	225	549
1337	227	374
1337	229	447
1337	289	999
1337	291	562
1337	293	597
1337	298	969
1337	306	491
1337	316	583
1337	424	1219
1337	425	489
1337	429	1324
1337	526	364
1337	618	434
1337	679	363
1337	701	368
1337	709	376
1337	716	639
1337	859	364
1337	903	346
1337	962	651
1337	969	377

1337	989	515
1337	1048	818
1337	1062	556
1337	1090	981
1337	1159	624
1337	1243	489
1337	1409	478
1337	1424	358
1337	1988	369
1337	2531	1550
1337	3186	413
1337	3200	2327
1337	3205	567
1337	3255	355
1337	3258	521
1337	3902	341
1337	4732	364
1337	5027	490
1337	6544	849
1339	65	78496
1339	89	296
1339	154	417
1339	182	1913
1339	190	1360
1339	207	4343
1339	217	434
1339	220	1005
1339	227	3349
1339	228	21342
1339	229	3159

1339	230 545	
1339	303 559	
1339	328 426	
1339	333 1870	
1339	366 1537	
1339	377 460	
1339	424 4233	
1339	498 82016	
1339	507 757	
1339	512 342	
1339	533 1178	
1339	538 590	
1339	540 1129	
1339	546 309	
1339	968 294	
1339	1045	4529
1339	1048	617
1339	1090	453
1339	1193	303
1339	1194	1973
1339	1195	377
1339	1200	373
1339	1239	1893
1339	1246	2066
1339	1375	518
1339	1384	460
1339	1400	959
1339	2544	465
1339	2608	1975
1339	2660	1054

1339	2661	2550
1339	2850	727
1339	2983	3251
1339	4466	299
1339	4747	347
1339	9766	528
1339	13964	1238
1339	13965	534
1339	13966	318
1340	383 617	
1340	429 414	
1340	605 1318	
1340	706 401	
1340	707 446	
1340	720 320	
1340	840 283	
1340	843 562	
1340	845 773	
1340	854 566	
1340	917 450	
1340	920 1766	
1340	977 827	
1340	1119	380
1340	1249	297
1340	1610	295
1340	1944	439
1340	1974	433
1340	2473	383
1340	2493	434
1340	2624	346

1340	2628	1109
1340	2637	502
1340	3333	538
1340	3364	837
1340	3444	349
1340	3739	286
1340	3744	328
1340	4183	411
1340	4184	347
1340	4188	1879
1340	4191	405
1340	4194	302
1340	4212	360
1340	4213	384
1340	4821	2998
1340	5600	450
1340	5636	2192
1340	8461	645
1340	8934	1176
1340	10214	509
1340	10662	380
1340	10848	404
1340	12325	296
1340	13523	311
1340	13967	1341
1340	13968	816
1340	13969	611
1340	13970	514
1340	13971	481
1342	163 31019	

1342	212	324
1342	447	594
1342	503	288
1342	707	301
1342	712	494
1342	806	336
1342	841	738
1342	952	829
1342	1132	673
1342	1369	317
1342	1411	770
1342	1412	1723
1342	1415	301
1342	1425	618
1342	1747	541
1342	1772	333
1342	1980	422
1342	2265	1435
1342	2312	289
1342	2339	507
1342	2471	459
1342	2558	718
1342	2853	1415
1342	2966	344
1342	3470	286
1342	4460	361
1342	4620	510
1342	4971	319
1342	5297	1214
1342	5550	545

1342	6961	1289
1342	8420	429
1342	8431	570
1342	8439	598
1342	9045	2166
1342	12992	282
1342	13981	6303
1342	13982	5732
1342	13983	2191
1342	13984	1436
1342	13985	718
1342	13986	700
1342	13987	552
1342	13988	425
1342	13989	418
1342	13990	384
1342	13991	356
1342	13992	316
1342	13993	277
1343	7	13
1343	51	14
1343	55	15
1343	72	160
1343	99	14
1343	159	13
1343	173	15
1343	203	12
1343	215	18
1343	226	14
1343	227	12

1343	233	22
1343	234	39
1343	289	18
1343	383	13
1343	436	13
1343	706	32
1343	707	29
1343	710	13
1343	724	12
1343	732	13
1343	854	80
1343	857	13
1343	903	13
1343	918	31
1343	936	26
1343	1034	68
1343	1104	20
1343	1249	20
1343	1360	12
1343	1369	13
1343	1412	92
1343	1513	20
1343	1534	13
1343	1703	24
1343	1713	14
1343	1714	61
1343	1807	14
1343	1817	21
1343	2343	15
1343	2789	13

1343	3741	13
1343	3744	14
1343	4165	13
1343	5550	12
1343	6532	13
1343	7464	13
1343	9468	23
1343	13994	17
1343	13995	16
1345	157 197	
1345	227 131	
1345	251 205	
1345	441 36	
1345	565 72	
1345	610 34	
1345	706 49	
1345	863 87	
1345	959 174	
1345	987 35	
1345	1112	294
1345	1117	39
1345	1121	81
1345	1123	236
1345	1124	36
1345	1130	401
1345	1227	56
1345	1242	653
1345	1244	67
1345	1249	112
1345	1411	51

1345	1412	49
1345	1464	127
1345	1802	866
1345	1844	36
1345	3444	74
1345	3481	53
1345	3777	141
1345	4784	54
1345	7119	80
1345	7427	59
1345	8594	34
1345	8843	548
1345	9056	61
1345	9271	116
1345	9453	55
1345	10142	99
1345	10225	49
1345	13999	1181
1345	14000	134
1345	14001	122
1345	14002	78
1345	14003	74
1345	14004	48
1345	14005	46
1345	14006	43
1345	14007	38
1345	14008	37
1345	14009	35
1345	14010	34
1346	191 153	

1346	291	134
1346	304	265
1346	313	184
1346	333	587
1346	344	451
1346	377	1574
1346	403	133
1346	461	930
1346	464	798
1346	466	217
1346	470	404
1346	481	808
1346	486	165
1346	531	254
1346	536	749
1346	537	168
1346	540	110
1346	544	290
1346	687	5288
1346	693	519
1346	695	130
1346	704	134
1346	816	2163
1346	914	144
1346	1043	403
1346	1180	624
1346	1183	156
1346	1186	2250
1346	1189	484
1346	1198	217

1346	1243	118
1346	1455	107
1346	1585	336
1346	1673	108
1346	1679	196
1346	1704	148
1346	1854	109
1346	2619	96
1346	2665	92
1346	2977	90
1346	3859	351
1346	4104	100
1346	4263	99
1346	4562	173
1346	6614	143
1346	9684	99
1346	10316	118
1346	14011	256
1346	14012	183
1347	205 4	
1347	565 5	
1347	577 7	
1347	580 6	
1347	582 4	
1347	583 8	
1347	683 13	
1347	1426	4
1347	1639	4
1347	1772	4
1347	2864	28

1347	2894	6
1347	2896	10
1347	3024	4
1347	5446	12
1347	6678	7
1347	6685	7
1347	7056	11
1347	7826	7
1347	8120	4
1347	8123	7
1347	8805	4
1347	11885	17
1347	12057	7
1347	12061	7
1347	12115	4
1347	12123	4
1347	12212	13
1347	14013	24
1347	14014	16
1347	14015	12
1347	14016	12
1347	14017	11
1347	14018	8
1347	14019	7
1347	14020	6
1347	14021	6
1347	14022	6
1347	14023	5
1347	14024	5
1347	14025	5

1347	14026	5
1347	14027	5
1347	14028	5
1347	14029	5
1347	14030	5
1347	14031	4
1347	14032	4
1347	14033	4
1347	14034	4
1348	89	94
1348	191	93
1348	288	121
1348	289	621
1348	290	89
1348	291	709
1348	294	221
1348	327	95
1348	333	3428
1348	342	64
1348	344	90
1348	356	63
1348	377	60
1348	386	674
1348	461	219
1348	466	517
1348	468	117
1348	475	395
1348	523	73
1348	681	74
1348	693	85

1348	701	108
1348	1025	100
1348	1043	105
1348	1444	244
1348	1447	253
1348	1459	642
1348	1684	164
1348	1685	66
1348	3078	97
1348	3127	158
1348	3489	120
1348	3859	171
1348	4562	61
1348	5032	149
1348	5267	463
1348	7340	327
1348	7756	421
1348	11223	139
1348	11890	87
1348	12683	62
1348	14035	569
1348	14036	410
1348	14037	102
1348	14038	96
1348	14039	84
1348	14040	84
1348	14041	82
1348	14042	69
1348	14043	63
1349	7	149

1349	70	198
1349	81	133
1349	154	1084
1349	173	1199
1349	190	166
1349	199	48
1349	220	178
1349	227	247
1349	234	542
1349	237	266
1349	238	51
1349	329	36
1349	377	150
1349	418	96
1349	450	249
1349	486	514
1349	489	78
1349	504	147
1349	533	73
1349	538	161
1349	617	112
1349	726	62
1349	757	104
1349	808	52
1349	978	220
1349	1048	291
1349	1090	34
1349	1091	147
1349	1145	86
1349	1244	56

1349	1424	78
1349	1700	328
1349	1786	221
1349	1852	40
1349	2193	63
1349	2699	377
1349	3094	249
1349	3850	90
1349	6187	64
1349	7183	115
1349	8750	52
1349	14044	138
1349	14045	64
1349	14046	58
1349	14047	43
1349	14048	42
1349	14049	41
1349	14050	39
1349	14051	38
1350	56	681
1350	96	606
1350	163	639
1350	176	383
1350	190	258
1350	220	261
1350	226	550
1350	227	1068
1350	233	362
1350	383	692
1350	607	934

1350	706 395	
1350	724 712	
1350	735 415	
1350	856 281	
1350	917 1386	
1350	918 334	
1350	920 309	
1350	922 467	
1350	924 381	
1350	940 358	
1350	942 456	
1350	959 258	
1350	1110	1015
1350	1122	416
1350	1132	273
1350	1249	408
1350	1338	275
1350	1343	670
1350	1350	249
1350	1358	531
1350	1369	462
1350	1372	469
1350	1394	368
1350	1412	996
1350	1639	722
1350	1703	373
1350	1883	380
1350	1993	287
1350	2346	508
1350	2362	256

1350	2565	260
1350	3774	280
1350	3777	373
1350	4130	492
1350	4675	583
1350	7052	366
1350	7054	1526
1350	14052	386
1350	14053	344
1351	89	215
1351	157	477
1351	288	73
1351	289	59
1351	298	25
1351	299	32
1351	300	68
1351	302	105
1351	323	27
1351	325	27
1351	333	64
1351	458	184
1351	461	26
1351	464	42
1351	466	92
1351	468	30
1351	470	331
1351	486	38
1351	498	185
1351	531	66
1351	541	42

1351	542	29
1351	544	49
1351	679	207
1351	687	31
1351	714	28
1351	1032	48
1351	1034	28
1351	1037	25
1351	1042	223
1351	1455	28
1351	1672	170
1351	1679	26
1351	2020	28
1351	2548	44
1351	2549	171
1351	3404	29
1351	4582	1623
1351	4811	39
1351	4814	203
1351	5950	43
1351	6124	59
1351	6543	168
1351	7269	47
1351	7705	45
1351	7868	292
1351	9249	50
1351	14054	83
1351	14055	49
1351	14056	37
1352	12	141

1352	25	103
1352	40	81
1352	70	100
1352	198	85
1352	222	389
1352	226	82
1352	228	161
1352	233	358
1352	370	167
1352	430	57
1352	503	111
1352	517	389
1352	716	106
1352	978	62
1352	1044	199
1352	1274	167
1352	1561	95
1352	1643	158
1352	1861	77
1352	1873	56
1352	1979	127
1352	2143	145
1352	2375	167
1352	2576	538
1352	2579	92
1352	2778	77
1352	2964	60
1352	3788	69
1352	3799	80
1352	3997	138

1352	4669	57
1352	4678	69
1352	5105	67
1352	5444	274
1352	6014	164
1352	7051	56
1352	7151	62
1352	7639	187
1352	7650	63
1352	8910	93
1352	8916	72
1352	9242	211
1352	9649	67
1352	14057	1082
1352	14058	144
1352	14059	84
1352	14060	77
1352	14061	70
1352	14062	63
1353	176 2100	
1353	198 306	
1353	233 1498	
1353	377 187	
1353	412 295	
1353	464 318	
1353	486 240	
1353	488 285	
1353	503 491	
1353	779 1575	
1353	808 462	

1353	810	4241
1353	813	1321
1353	816	1444
1353	830	253
1353	834	207
1353	837	1014
1353	917	427
1353	918	198
1353	946	760
1353	978	1334
1353	982	400
1353	1044	3735
1353	1145	409
1353	1180	198
1353	1186	577
1353	1188	495
1353	1195	546
1353	1200	347
1353	1513	756
1353	1672	420
1353	1852	222
1353	1853	1517
1353	1854	217
1353	1863	302
1353	1868	188
1353	1870	185
1353	1905	1117
1353	2191	325
1353	2210	226
1353	2214	2239

1353 2621 320
1353 2847 250
1353 2850 377
1353 2961 199
1353 2975 195
1353 4166 1616
1353 4550 371
1353 4947 472
1353 7389 197
1354 154 91
1354 190 140
1354 195 268
1354 199 129
1354 207 155
1354 217 72
1354 222 118
1354 223 77
1354 228 132
1354 229 92
1354 416 172
1354 419 146
1354 420 138
1354 424 226
1354 427 93
1354 430 109
1354 432 73
1354 441 130
1354 533 122
1354 554 82
1354 618 79

1354	757 85	
1354	860 87	
1354	982 93	
1354	1048	106
1354	1106	116
1354	1390	186
1354	1400	69
1354	1424	103
1354	1976	92
1354	2137	109
1354	2139	108
1354	2424	135
1354	2531	68
1354	2533	116
1354	2538	87
1354	2582	155
1354	2606	96
1354	2610	75
1354	2611	106
1354	2614	87
1354	3030	214
1354	3279	102
1354	4089	70
1354	4115	77
1354	5147	104
1354	7177	94
1354	8054	94
1354	8674	165
1354	11739	76
1355	196 402	

1355	199	315
1355	204	236
1355	205	610
1355	211	271
1355	223	157
1355	227	142
1355	229	137
1355	416	319
1355	434	195
1355	445	219
1355	602	144
1355	604	174
1355	606	1622
1355	622	329
1355	689	146
1355	709	134
1355	1481	433
1355	1633	292
1355	1944	155
1355	1976	134
1355	2269	142
1355	2424	557
1355	2584	526
1355	3158	126
1355	3249	455
1355	3258	250
1355	3681	183
1355	4058	295
1355	4069	158
1355	5925	145

1355	6204	244
1355	6205	157
1355	6229	355
1355	6390	187
1355	6681	126
1355	6836	127
1355	7274	127
1355	8234	369
1355	9277	176
1355	11589	486
1355	11812	135
1355	12546	197
1355	14063	374
1355	14064	318
1355	14065	253
1355	14066	173
1355	14067	163
1355	14068	162
1355	14069	144
1356	55	311
1356	67	313
1356	89	3818
1356	161	111
1356	257	337
1356	288	2245
1356	289	7211
1356	291	181
1356	292	1060
1356	294	336
1356	295	217

1356	298	240
1356	299	163
1356	300	816
1356	302	178
1356	308	2233
1356	311	134
1356	314	114
1356	323	477
1356	325	1235
1356	333	151
1356	349	263
1356	386	193
1356	400	299
1356	458	181
1356	461	1589
1356	466	1933
1356	498	243
1356	528	330
1356	543	104
1356	544	1242
1356	670	113
1356	680	212
1356	690	129
1356	701	860
1356	793	187
1356	915	132
1356	1037	144
1356	1243	309
1356	1441	461
1356	1451	791

1356	2091	174
1356	2092	291
1356	2094	847
1356	3397	1015
1356	3925	185
1356	4814	340
1356	7391	136
1356	13265	591
1356	14070	149
1357	227	167
1357	565	180
1357	570	185
1357	574	305
1357	582	126
1357	583	158
1357	585	288
1357	588	164
1357	592	187
1357	857	325
1357	985	147
1357	1220	331
1357	1227	136
1357	1244	313
1357	1513	633
1357	2490	378
1357	2999	141
1357	3439	154
1357	3877	126
1357	4520	460
1357	5382	489

1357	5627	193
1357	6458	296
1357	8121	217
1357	8126	128
1357	8812	274
1357	9287	160
1357	10358	166
1357	14020	229
1357	14071	5075
1357	14072	468
1357	14073	382
1357	14074	324
1357	14075	287
1357	14076	274
1357	14077	258
1357	14078	253
1357	14079	221
1357	14080	200
1357	14081	191
1357	14082	191
1357	14083	177
1357	14084	148
1357	14085	145
1357	14086	143
1357	14087	142
1357	14088	140
1357	14089	137
1357	14090	135
1357	14091	128
1358	48 218	

1358	152	1059
1358	162	616
1358	181	1955
1358	188	536
1358	190	392
1358	198	268
1358	370	1510
1358	378	411
1358	403	522
1358	407	324
1358	412	264
1358	504	374
1358	506	2333
1358	707	264
1358	813	263
1358	815	299
1358	841	327
1358	854	677
1358	883	217
1358	918	305
1358	920	234
1358	949	633
1358	1131	242
1358	1134	294
1358	1264	821
1358	1343	317
1358	1521	406
1358	1533	334
1358	1672	545
1358	1992	512

1358	2014	364
1358	2370	970
1358	2579	335
1358	2654	245
1358	4170	431
1358	4268	255
1358	4610	235
1358	5603	235
1358	6182	264
1358	8420	480
1358	10042	267
1358	10833	1128
1358	10956	682
1358	12283	267
1358	12662	273
1358	12783	1100
1358	14092	3269
1358	14093	532
1358	14094	378
1360	199	283
1360	207	134
1360	211	332
1360	217	437
1360	220	177
1360	222	243
1360	223	132
1360	424	262
1360	434	119
1360	440	219
1360	533	196

1360	618	117
1360	621	622
1360	622	160
1360	629	426
1360	632	127
1360	748	122
1360	778	289
1360	945	130
1360	1048	138
1360	1105	234
1360	1239	154
1360	1375	158
1360	1633	154
1360	1824	118
1360	1954	119
1360	2405	133
1360	2407	329
1360	2424	178
1360	2532	126
1360	2584	161
1360	2627	162
1360	3098	374
1360	3105	285
1360	3258	773
1360	3411	151
1360	3707	403
1360	3754	160
1360	4444	175
1360	5437	137
1360	5547	227

1360	5549	130
1360	6217	125
1360	6298	129
1360	6836	117
1360	8012	193
1360	11316	157
1360	11423	218
1360	11552	201
1360	14097	159
1362	198 112	
1362	227 154	
1362	230 214	
1362	234 121	
1362	377 121	
1362	383 3313	
1362	503 391	
1362	562 144	
1362	706 218	
1362	707 278	
1362	730 85	
1362	808 4438	
1362	857 88	
1362	859 81	
1362	917 120	
1362	936 121	
1362	1249	146
1362	1650	869
1362	1974	181
1362	2343	85
1362	2346	135

1362	3354	1197
1362	3434	848
1362	3442	1201
1362	3444	929
1362	3737	1176
1362	3792	647
1362	4218	935
1362	4821	2849
1362	5262	141
1362	5636	2836
1362	6495	264
1362	8945	420
1362	11042	556
1362	12096	637
1362	14106	1077
1362	14107	830
1362	14108	499
1362	14109	469
1362	14110	375
1362	14111	308
1362	14112	283
1362	14113	272
1362	14114	248
1362	14115	184
1362	14116	133
1362	14117	115
1362	14118	97
1362	14119	92
1362	14120	85
1363	159 738	

1363 173 899

1363 209 830

1363 210 538

1363 324 1432

1363 333 1169

1363 412 1349

1363 466 952

1363 486 861

1363 492 1239

1363 498 4370

1363 518 2021

1363 536 947

1363 537 2846

1363 540 1031

1363 548 683

1363 687 3444

1363 691 1172

1363 693 852

1363 703 831

1363 779 593

1363 816 930

1363 936 524

1363 968 833

1363 1035 759

1363 1040 1836

1363 1047 517

1363 1091 1116

1363 1182 820

1363 1183 2190

1363 1185 1683

1363	1186	2598
1363	1188	664
1363	1246	882
1363	1707	648
1363	1710	2914
1363	2210	702
1363	2430	733
1363	2441	1520
1363	2665	676
1363	2847	517
1363	3197	1233
1363	4136	583
1363	4262	1387
1363	4434	1633
1363	6453	699
1363	7831	734
1363	9216	678
1363	14121	1292
1363	14122	536
1364	227 254	
1364	234 58	
1364	554 44	
1364	570 35	
1364	585 62	
1364	607 34	
1364	728 74	
1364	732 54	
1364	733 40	
1364	735 78	
1364	868 106	

1364	959 41	
1364	986 79	
1364	1177	35
1364	1244	50
1364	1406	96
1364	2028	41
1364	2459	85
1364	2732	36
1364	2842	96
1364	2855	37
1364	2864	381
1364	2871	72
1364	2873	199
1364	3019	88
1364	3058	39
1364	4887	103
1364	4911	61
1364	7056	36
1364	7130	116
1364	8111	37
1364	8123	50
1364	8354	34
1364	11885	54
1364	11918	36
1364	11923	42
1364	12648	40
1364	14071	130
1364	14123	406
1364	14124	211
1364	14125	131

1364	14126	56
1364	14127	53
1364	14128	48
1364	14129	45
1364	14130	45
1364	14131	40
1364	14132	37
1364	14133	35
1364	14134	34
1365	55	1402
1365	67	843
1365	89	1021
1365	97	397
1365	257	813
1365	288	1841
1365	289	5874
1365	291	546
1365	292	1603
1365	293	548
1365	294	762
1365	295	615
1365	300	1341
1365	302	566
1365	306	955
1365	310	315
1365	311	702
1365	314	1220
1365	318	542
1365	323	2023
1365	325	832

1365	328 321	
1365	333 557	
1365	344 847	
1365	352 2674	
1365	461 687	
1365	464 486	
1365	466 484	
1365	485 711	
1365	498 670	
1365	525 612	
1365	528 351	
1365	543 1177	
1365	644 425	
1365	679 1925	
1365	680 386	
1365	686 1206	
1365	701 495	
1365	704 368	
1365	790 387	
1365	908 331	
1365	915 685	
1365	1034	1361
1365	1451	597
1365	1459	598
1365	2102	715
1365	2981	541
1365	2990	395
1365	3177	319
1365	5529	492
1366	89 59	

1366	155 203	
1366	191 243	
1366	229 79	
1366	300 661	
1366	306 111	
1366	324 74	
1366	328 207	
1366	344 79	
1366	346 215	
1366	355 80	
1366	377 119	
1366	403 245	
1366	466 238	
1366	471 77	
1366	477 155	
1366	486 2070	
1366	493 179	
1366	497 104	
1366	540 292	
1366	546 60	
1366	690 96	
1366	907 142	
1366	966 231	
1366	979 101	
1366	982 226	
1366	1106	210
1366	1239	78
1366	1375	1726
1366	1397	80
1366	1409	379

1366	1410	68
1366	1440	55
1366	1502	173
1366	1511	77
1366	1972	70
1366	2176	74
1366	2535	163
1366	2610	146
1366	3400	59
1366	3859	67
1366	4069	102
1366	5099	62
1366	5978	428
1366	7643	111
1366	7743	647
1366	7777	96
1366	8067	62
1366	10069	169
1366	14135	71
1367	89	729
1367	197	367
1367	466	634
1367	527	369
1367	592	322
1367	757	230
1367	898	351
1367	1037	705
1367	1123	255
1367	1833	293
1367	1952	215

1367	1963	237
1367	2026	855
1367	2083	289
1367	2289	353
1367	2402	303
1367	2416	815
1367	2419	318
1367	2470	202
1367	3227	316
1367	3299	2432
1367	4053	323
1367	4077	246
1367	4586	219
1367	4736	244
1367	5420	213
1367	6633	268
1367	6824	347
1367	7427	330
1367	8178	375
1367	8267	222
1367	9032	372
1367	10628	204
1367	10722	226
1367	10969	374
1367	11463	221
1367	13169	342
1367	14136	441
1367	14137	337
1367	14138	322
1367	14139	311

1367	14140	308
1367	14141	306
1367	14142	290
1367	14143	289
1367	14144	281
1367	14145	242
1367	14146	230
1367	14147	217
1367	14148	207
1368	7	198
1368	72	251
1368	89	3336
1368	154	341
1368	156	172
1368	157	2642
1368	159	232
1368	163	119
1368	173	143
1368	209	153
1368	229	120
1368	230	250
1368	233	1199
1368	288	580
1368	289	2967
1368	291	123
1368	292	385
1368	295	537
1368	300	379
1368	302	264
1368	331	533

1368	333 226	
1368	349 231	
1368	377 343	
1368	418 463	
1368	420 174	
1368	486 125471	
1368	498 432	
1368	523 274	
1368	537 272	
1368	540 141	
1368	707 453	
1368	907 177	
1368	917 118	
1368	966 124	
1368	978 277	
1368	1044	228
1368	1104	133
1368	1118	312
1368	1131	205
1368	1179	221
1368	1672	128
1368	1700	362
1368	1854	178
1368	2220	184
1368	2446	245
1368	3411	229
1368	14149	686
1368	14150	465
1368	14151	234
1369	288 87	

1369	290 76
1369	291 116
1369	298 86
1369	300 284
1369	318 61
1369	321 599
1369	324 51
1369	325 369
1369	333 186
1369	344 1711
1369	377 36
1369	403 38
1369	458 39
1369	461 371
1369	464 165
1369	466 337
1369	471 66
1369	481 59
1369	486 36
1369	498 159
1369	528 149
1369	538 57
1369	679 613
1369	680 48
1369	681 762
1369	685 90
1369	686 353
1369	688 35
1369	690 38
1369	907 41

1369	1043	140
1369	1377	53
1369	1447	96
1369	1453	306
1369	1470	69
1369	1934	35
1369	2020	250
1369	2607	90
1369	4562	41
1369	4767	47
1369	4879	56
1369	5001	159
1369	7777	181
1369	8378	64
1369	11010	57
1369	13458	34
1369	13720	64
1369	14152	163
1369	14153	68
1371	45	493
1371	81	143
1371	540	454
1371	546	231
1371	755	263
1371	768	195
1371	1099	193
1371	1191	408
1371	1192	145
1371	1237	143
1371	1568	388

1371	2704	178
1371	2771	143
1371	2820	223
1371	3227	426
1371	3428	265
1371	3703	226
1371	3737	211
1371	3812	637
1371	11265	454
1371	14154	1362
1371	14155	1290
1371	14156	922
1371	14157	913
1371	14158	594
1371	14159	557
1371	14160	464
1371	14161	435
1371	14162	359
1371	14163	332
1371	14164	292
1371	14165	287
1371	14166	280
1371	14167	263
1371	14168	253
1371	14169	244
1371	14170	242
1371	14171	222
1371	14172	221
1371	14173	217
1371	14174	197

1371	14175	188
1371	14176	181
1371	14177	181
1371	14178	172
1371	14179	169
1371	14180	160
1371	14181	153
1371	14182	146
1371	14183	144
1372	7	350
1372	65	554
1372	72	254
1372	89	172
1372	159	204
1372	173	1117
1372	182	212
1372	190	777
1372	207	1376
1372	208	274
1372	217	153
1372	227	668
1372	229	660
1372	230	451
1372	298	401
1372	424	351
1372	428	168
1372	429	173
1372	432	154
1372	441	187
1372	486	464

1372	488	252
1372	498	278
1372	511	153
1372	526	239
1372	540	1086
1372	716	137
1372	959	297
1372	986	169
1372	1048	250
1372	1075	709
1372	1090	138
1372	1091	137
1372	1097	163
1372	1104	145
1372	1246	309
1372	1398	326
1372	1512	584
1372	1714	192
1372	1789	269
1372	1874	269
1372	2131	575
1372	2137	169
1372	2665	194
1372	3053	127
1372	4905	229
1372	7008	215
1372	8830	182
1372	10208	145
1372	11170	164
1373	81	516

1373	190 349	
1373	203 336	
1373	217 258	
1373	230 878	
1373	378 413	
1373	477 173	
1373	498 925	
1373	540 303	
1373	716 1180	
1373	862 200	
1373	924 1385	
1373	925 226	
1373	926 239	
1373	930 217	
1373	946 241	
1373	955 975	
1373	1098	780
1373	1244	833
1373	1464	345
1373	1725	780
1373	1766	215
1373	2176	331
1373	2425	210
1373	3124	431
1373	3928	214
1373	4177	853
1373	4361	208
1373	5650	410
1373	5745	274
1373	5972	223

1373	7101	1367
1373	7194	195
1373	8594	185
1373	8727	177
1373	8728	550
1373	9185	183
1373	9187	208
1373	11217	455
1373	11222	246
1373	11323	346
1373	12947	723
1373	14184	538
1373	14185	529
1373	14186	399
1373	14187	322
1373	14188	237
1373	14189	217
1373	14190	206
1373	14191	181
1374	71 325	
1374	157 145	
1374	190 696	
1374	199 156	
1374	207 490	
1374	228 245	
1374	230 641	
1374	233 720	
1374	234 746	
1374	333 467	
1374	375 137	

1374	378 648	
1374	412 209	
1374	472 168	
1374	489 223	
1374	497 160	
1374	498 987	
1374	499 1730	
1374	512 384	
1374	517 181	
1374	518 1035	
1374	525 141	
1374	534 599	
1374	706 148	
1374	726 991	
1374	920 127	
1374	930 1364	
1374	955 289	
1374	959 1266	
1374	982 725	
1374	1091	362
1374	1104	116
1374	1116	1610
1374	1118	235
1374	1131	208
1374	1852	226
1374	1854	1413
1374	1974	254
1374	2014	491
1374	2094	127
1374	2346	882

1374	2843	571
1374	2959	123
1374	3763	257
1374	4262	1782
1374	4428	380
1374	7448	166
1374	7862	128
1374	14192	612
1374	14193	151
1375	152 453	
1375	162 238	
1375	163 3069	
1375	429 1300	
1375	607 180	
1375	707 122	
1375	805 383	
1375	810 256	
1375	813 564	
1375	815 1015	
1375	843 180	
1375	955 175	
1375	978 122	
1375	1104	162
1375	1195	608
1375	1239	110
1375	1244	122
1375	1369	349
1375	1858	492
1375	2006	164
1375	2531	242

1375	2572	580
1375	2575	531
1375	2579	109
1375	2608	109
1375	2637	161
1375	2711	186
1375	2864	118
1375	3197	195
1375	3308	158
1375	3405	256
1375	3482	127
1375	4087	144
1375	5317	167
1375	5667	241
1375	6906	186
1375	7804	540
1375	8278	226
1375	8467	131
1375	9716	793
1375	10613	233
1375	11032	217
1375	14194	297
1375	14195	213
1375	14196	203
1375	14197	166
1375	14198	157
1375	14199	144
1375	14200	130
1375	14201	112
1376	7	31

1376	72	26
1376	155	17
1376	159	16
1376	172	4
1376	207	15
1376	209	39
1376	220	7
1376	230	26
1376	234	39
1376	289	43
1376	333	12
1376	378	40
1376	405	9
1376	412	51
1376	486	81
1376	499	29
1376	500	17
1376	504	4
1376	518	16
1376	524	18
1376	536	15
1376	537	37
1376	538	35
1376	540	4
1376	607	6
1376	680	45
1376	861	7
1376	966	25
1376	1091	16
1376	1185	13

1376	1196	8
1376	1246	17
1376	1249	7
1376	1674	17
1376	2121	12
1376	2430	18
1376	2432	41
1376	3373	10
1376	3767	16
1376	4201	24
1376	4255	26
1376	4262	56
1376	4385	12
1376	5149	6
1376	5426	61
1376	6191	4
1376	6461	5
1376	9636	15
1376	14202	8
1377	190 763	
1377	195 80056	
1377	209 5809	
1377	289 726	
1377	412 987	
1377	537 903	
1377	687 926	
1377	779 2688	
1377	808 1066	
1377	816 4323	
1377	1038	796

1377	1100	2397
1377	1179	1080
1377	1187	920
1377	1195	1177
1377	1197	950
1377	1541	1218
1377	1632	784
1377	1650	666
1377	1858	2974
1377	2425	1300
1377	2430	1014
1377	2444	702
1377	2580	949
1377	2850	1905
1377	3057	980
1377	3616	5313
1377	3712	915
1377	3714	836
1377	3737	761
1377	4189	1561
1377	4262	849
1377	6463	1166
1377	6594	835
1377	6600	703
1377	6765	1324
1377	6771	770
1377	6773	2963
1377	7724	725
1377	8468	945
1377	9767	971

1377	11055	5515
1377	11412	4587
1377	13960	1038
1377	14203	1859
1377	14204	1227
1377	14205	1173
1377	14206	1155
1377	14207	763
1377	14208	683
1378	163 229	
1378	197 255	
1378	206 266	
1378	212 176	
1378	227 234	
1378	231 226	
1378	418 164	
1378	442 145	
1378	444 896	
1378	602 337	
1378	609 164	
1378	619 326	
1378	709 265	
1378	748 155	
1378	857 194	
1378	863 187	
1378	1242	355
1378	1423	170
1378	1631	210
1378	1807	162
1378	1969	244

1378	2285	195
1378	2478	276
1378	2541	369
1378	2716	148
1378	3019	187
1378	3279	161
1378	3310	220
1378	3456	176
1378	3686	168
1378	3694	537
1378	4079	199
1378	4779	294
1378	5434	201
1378	6335	205
1378	6409	343
1378	9457	237
1378	11791	391
1378	12002	356
1378	12605	150
1378	13289	250
1378	14209	447
1378	14210	274
1378	14211	252
1378	14212	243
1378	14213	243
1378	14214	228
1378	14215	170
1378	14216	164
1378	14217	154
1379	55 512	

1379 67 344
1379 89 561
1379 257 344
1379 289 2206
1379 291 855
1379 293 848
1379 294 512
1379 300 651
1379 305 390
1379 311 734
1379 318 1006
1379 321 337
1379 325 482
1379 333 1122
1379 342 1105
1379 344 4583
1379 377 374
1379 456 1676
1379 461 763
1379 466 518
1379 530 518
1379 535 313
1379 548 544
1379 679 1214
1379 680 384
1379 681 554
1379 686 360
1379 691 533
1379 701 1303
1379 786 583

1379	908	393
1379	972	357
1379	1242	349
1379	1459	536
1379	1470	494
1379	1857	3369
1379	2546	1281
1379	2801	319
1379	3081	608
1379	3327	478
1379	3489	458
1379	3754	324
1379	3763	790
1379	3871	383
1379	4488	311
1379	4867	330
1379	14218	1851
1379	14219	404
1379	14220	338
1380	65	88
1380	154	172
1380	159	185
1380	173	536
1380	182	159
1380	184	175
1380	207	343
1380	210	219
1380	220	485
1380	228	107
1380	229	718

1380	342	178
1380	366	103
1380	412	147
1380	424	127
1380	486	357
1380	498	134
1380	500	232
1380	501	101
1380	504	438
1380	533	261
1380	536	127
1380	540	227
1380	830	117
1380	930	111
1380	966	406
1380	982	418
1380	1044	160
1380	1145	96
1380	1180	233
1380	1181	139
1380	1185	292
1380	1188	175
1380	1244	204
1380	1246	185
1380	1247	411
1380	1377	191
1380	1470	349
1380	1672	429
1380	1905	222
1380	1914	358

1380	2150	89
1380	2352	175
1380	2785	128
1380	3027	221
1380	3427	494
1380	3464	255
1380	4439	270
1380	8371	130
1380	9156	345
1381	65	402
1381	103	60
1381	182	78
1381	229	65
1381	289	105
1381	292	60
1381	298	139
1381	362	258
1381	418	537
1381	423	80
1381	425	128
1381	444	152
1381	511	76
1381	602	69
1381	632	402
1381	703	73
1381	748	134
1381	757	387
1381	896	148
1381	1062	333
1381	1375	92

1381	1379	77
1381	1688	239
1381	1736	113
1381	1747	105
1381	1776	96
1381	1780	60
1381	2541	88
1381	2821	357
1381	3196	73
1381	3310	102
1381	3411	117
1381	3691	113
1381	3902	319
1381	4205	93
1381	5480	93
1381	5926	133
1381	5927	60
1381	6132	66
1381	6756	111
1381	6920	524
1381	10610	60
1381	10681	282
1381	11396	110
1381	11716	78
1381	11819	128
1381	13039	73
1381	14221	87
1381	14222	77
1381	14223	61
1382	65 889	

1382	229 306	
1382	249 1458	
1382	259 582	
1382	267 1416	
1382	276 338	
1382	278 383	
1382	331 785	
1382	547 800	
1382	683 2918	
1382	1613	568
1382	1620	556
1382	1622	628
1382	1627	4780
1382	1631	775
1382	1640	558
1382	1943	1198
1382	1946	6710
1382	1948	744
1382	1950	6291
1382	1959	1464
1382	1963	1479
1382	2252	725
1382	2921	1036
1382	2923	1144
1382	2930	285
1382	3227	350
1382	3355	679
1382	4585	284
1382	5605	599
1382	5883	1064

1382	6226	1412
1382	6321	613
1382	8929	378
1382	10552	557
1382	10555	664
1382	10556	406
1382	10893	455
1382	11360	447
1382	12755	554
1382	13094	461
1382	14224	1327
1382	14225	1177
1382	14226	896
1382	14227	598
1382	14228	561
1382	14229	529
1382	14230	496
1382	14231	411
1382	14232	361
1383	53	342
1383	58	219
1383	65	1254
1383	81	375
1383	154	6180
1383	163	249
1383	182	369
1383	190	680
1383	203	229
1383	207	402
1383	217	347

1383	227	2489
1383	228	371
1383	237	940
1383	418	636
1383	423	1512
1383	604	735
1383	703	452
1383	733	263
1383	868	439
1383	962	232
1383	986	231
1383	1062	384
1383	1089	224
1383	1098	3151
1383	1239	215
1383	1424	387
1383	1512	285
1383	1632	677
1383	1633	388
1383	2083	318
1383	2139	1023
1383	2277	1560
1383	2407	670
1383	3070	368
1383	3206	701
1383	3259	292
1383	4113	262
1383	5480	246
1383	8676	300
1383	8716	243

1383	10738	263
1383	11280	679
1383	13557	289
1383	13729	1048
1383	14233	331
1383	14234	292
1383	14235	278
1383	14236	229
1383	14237	197
1384	51	222
1384	53	194
1384	64	317
1384	65	336
1384	71	1345
1384	89	196
1384	172	700
1384	178	309
1384	182	1941
1384	203	1454
1384	220	981
1384	229	1197
1384	230	1403
1384	234	2025
1384	307	843
1384	497	3092
1384	525	1129
1384	533	204
1384	534	5476
1384	683	428
1384	728	388

1384	851 435	
1384	966 375	
1384	967 197	
1384	982 484	
1384	1239	291
1384	1375	431
1384	1470	841
1384	1505	2244
1384	1650	298
1384	1797	1648
1384	1814	409
1384	2176	532
1384	2454	269
1384	2544	998
1384	3200	313
1384	3333	1069
1384	4258	372
1384	4394	223
1384	4817	321
1384	5437	324
1384	7268	191
1384	11955	964
1384	12273	275
1384	13219	401
1384	13645	283
1384	14238	355
1384	14239	281
1384	14240	240
1384	14241	220
1385	89 168	

1385	288	180
1385	289	51
1385	291	57
1385	300	230
1385	313	57
1385	318	60
1385	321	72
1385	344	226
1385	367	107
1385	381	151
1385	388	319
1385	400	195
1385	410	155
1385	461	69
1385	464	84
1385	466	226
1385	523	59
1385	538	156
1385	540	73
1385	544	67
1385	548	126
1385	679	225
1385	681	152
1385	686	241
1385	793	113
1385	797	165
1385	1042	91
1385	1458	159
1385	1684	59
1385	1904	1117

1385	1934	47
1385	2089	220
1385	2092	140
1385	2094	127
1385	2523	60
1385	2546	60
1385	2807	74
1385	3081	113
1385	3375	61
1385	3404	48
1385	5565	102
1385	5978	48
1385	7898	104
1385	10809	285
1385	12275	133
1385	12280	65
1385	14242	112
1385	14243	109
1385	14244	51
1386	178 152	
1386	263 100	
1386	266 104	
1386	378 119	
1386	506 148	
1386	903 132	
1386	955 135	
1386	1150	193
1386	1167	269
1386	1772	306
1386	2124	129

1386	2136	128
1386	2298	203
1386	2324	103
1386	2332	908
1386	2338	130
1386	2339	232
1386	2616	128
1386	2786	100
1386	2952	237
1386	3259	212
1386	3902	177
1386	4143	139
1386	4151	107
1386	4548	171
1386	5914	404
1386	6930	229
1386	7810	546
1386	8230	117
1386	8427	114
1386	8454	311
1386	9050	111
1386	9056	139
1386	9061	109
1386	13164	129
1386	13502	171
1386	14245	375
1386	14246	289
1386	14247	199
1386	14248	185
1386	14249	165

1386	14250	154
1386	14251	136
1386	14252	135
1386	14253	130
1386	14254	117
1386	14255	114
1386	14256	108
1386	14257	101
1386	14258	100
1387	63	90
1387	65	250
1387	162	169
1387	191	93
1387	295	109
1387	423	347
1387	471	178
1387	472	87
1387	486	162
1387	501	210
1387	531	236
1387	548	101
1387	606	195
1387	689	89
1387	691	239
1387	972	243
1387	982	144
1387	1075	209
1387	1151	191
1387	1198	85
1387	1375	187

1387	1377	299
1387	1383	449
1387	2521	150
1387	2582	100
1387	2607	385
1387	2610	102
1387	2612	160
1387	3255	96
1387	3411	131
1387	3475	148
1387	4385	148
1387	5071	155
1387	5111	123
1387	5480	266
1387	6594	148
1387	6596	104
1387	6604	94
1387	7194	105
1387	9578	138
1387	9977	182
1387	10327	118
1387	11155	89
1387	11728	210
1387	12544	175
1387	14259	664
1387	14260	205
1387	14261	185
1387	14262	116
1387	14263	88
1388	81 23	

1388	89	198
1388	173	54
1388	207	20
1388	209	41
1388	230	22
1388	288	77
1388	289	59
1388	300	1282
1388	321	18
1388	324	19
1388	333	63
1388	344	31
1388	420	33
1388	425	53
1388	430	64
1388	461	48
1388	464	20
1388	466	24
1388	498	444
1388	528	17
1388	531	22
1388	543	22
1388	546	63
1388	681	74
1388	686	56
1388	704	796
1388	790	17
1388	859	46
1388	1032	199
1388	1045	25

1388	1048	16
1388	1424	16
1388	1452	26
1388	1455	21
1388	1456	31
1388	1673	17
1388	2020	17
1388	2080	41
1388	2084	18
1388	2137	23
1388	2277	30
1388	2549	18
1388	2788	29
1388	3200	44
1388	3373	18
1388	4028	29
1388	5149	37
1388	5180	45
1388	7834	47
1389	55	145
1389	65	317
1389	89	306
1389	154	566
1389	159	371
1389	190	381
1389	203	346
1389	207	124
1389	227	663
1389	229	544
1389	231	244

1389	298	182
1389	424	168
1389	425	178
1389	429	669
1389	432	400
1389	436	107
1389	440	397
1389	526	190
1389	530	109
1389	533	152
1389	599	396
1389	622	862
1389	690	113
1389	715	141
1389	757	353
1389	971	208
1389	1048	243
1389	1090	120
1389	1097	421
1389	1098	292
1389	1106	113
1389	1109	112
1389	1390	210
1389	1400	1725
1389	1632	169
1389	2083	129
1389	2531	201
1389	3057	561
1389	3902	127
1389	5235	167

1389	5237	134
1389	6726	164
1389	7324	150
1389	8756	478
1389	12915	524
1389	13668	125
1389	14264	153
1389	14265	144
1389	14266	109
1390	89	127
1390	154	168
1390	163	194
1390	173	747
1390	198	279
1390	207	398
1390	209	258
1390	227	1004
1390	234	266
1390	419	840
1390	421	286
1390	424	651
1390	430	425
1390	436	125
1390	437	353
1390	439	186
1390	441	171
1390	443	232
1390	533	657
1390	540	315
1390	562	295

1390	599	186
1390	689	139
1390	709	201
1390	859	196
1390	863	687
1390	868	257
1390	964	147
1390	1048	752
1390	1090	685
1390	1239	324
1390	1246	263
1390	1400	523
1390	1412	252
1390	1486	139
1390	1513	185
1390	1515	830
1390	1764	231
1390	1766	225
1390	2786	320
1390	2855	199
1390	3200	140
1390	3769	1188
1390	4124	165
1390	4802	380
1390	5414	124
1390	5416	256
1390	14267	265
1390	14268	178
1390	14269	165
1393	19	1458

1393	31	391
1393	37	346
1393	40	342
1393	41	480
1393	72	381
1393	487	775
1393	757	619
1393	1075	312
1393	1203	372
1393	1253	421
1393	1277	365
1393	1278	572
1393	1292	640
1393	1297	1266
1393	1315	493
1393	1320	579
1393	1326	331
1393	1793	365
1393	1983	484
1393	2667	675
1393	2935	316
1393	3087	371
1393	3183	1567
1393	3196	1985
1393	4308	338
1393	4874	980
1393	4875	360
1393	5715	495
1393	6873	686
1393	7078	594

1393	7512	1403
1393	7522	438
1393	9571	493
1393	11031	1311
1393	11112	341
1393	11331	350
1393	14284	664
1393	14285	598
1393	14286	507
1393	14287	501
1393	14288	453
1393	14289	441
1393	14290	421
1393	14291	403
1393	14292	388
1393	14293	385
1393	14294	343
1393	14295	315
1393	14296	312
1394	65	247
1394	67	1971
1394	88	805
1394	89	3682
1394	154	242
1394	167	449
1394	190	259
1394	207	618
1394	217	974
1394	227	289
1394	228	235

1394 230 331

1394 288 1158

1394 289 992

1394 291 368

1394 292 1107

1394 295 566

1394 298 198

1394 300 306

1394 302 172

1394 306 175

1394 310 375

1394 320 326

1394 344 518

1394 349 537

1394 353 310

1394 355 619

1394 416 457

1394 430 210

1394 466 524

1394 498 454

1394 545 234

1394 546 381

1394 679 1058

1394 689 204

1394 703 156

1394 735 262

1394 903 296

1394 980 267

1394 982 343

1394 1037 546

1394	1048	1197
1394	1243	416
1394	1400	295
1394	1463	287
1394	1613	158
1394	1772	1004
1394	1934	182
1394	2176	764
1394	13715	169
1395	56	254
1395	84	159
1395	107	423
1395	154	518
1395	197	415
1395	199	113
1395	203	517
1395	227	208
1395	331	387
1395	416	117
1395	429	122
1395	440	565
1395	608	123
1395	610	146
1395	629	145
1395	703	117
1395	715	407
1395	735	178
1395	757	314
1395	859	143
1395	1242	129

1395	1405	152
1395	1613	204
1395	1627	205
1395	1888	153
1395	1943	714
1395	1952	482
1395	1961	252
1395	1970	165
1395	2083	235
1395	2281	114
1395	2289	150
1395	2535	200
1395	2593	151
1395	2921	791
1395	3299	129
1395	3687	169
1395	3693	125
1395	3694	482
1395	5150	123
1395	5871	117
1395	7324	499
1395	12000	168
1395	14297	428
1395	14298	132
1395	14299	129
1395	14300	118
1395	14301	117
1395	14302	116
1395	14303	112
1396	163 509	

1396	227 147	
1396	234 289	
1396	436 156	
1396	614 146	
1396	706 198	
1396	707 268	
1396	724 77	
1396	732 69	
1396	735 162	
1396	841 240	
1396	843 220	
1396	854 1209	
1396	855 126	
1396	856 126	
1396	868 629	
1396	917 710	
1396	918 1680	
1396	947 93	
1396	949 105	
1396	1249	83
1396	1257	322
1396	1337	191
1396	1344	114
1396	1352	90
1396	1358	166
1396	1360	117
1396	1364	96
1396	1369	199
1396	1372	73
1396	1412	337

1396	1426	314
1396	1525	79
1396	1526	84
1396	1527	211
1396	1528	204
1396	1531	182
1396	1660	119
1396	2013	83
1396	2347	82
1396	2855	75
1396	3470	117
1396	3780	170
1396	7419	116
1396	7421	128
1396	7422	85
1396	9344	71
1396	9818	149
1396	14304	164
1396	14305	94
1397	1346	1
1397	1810	1
1397	2013	1
1397	2343	1
1397	2360	1
1397	3470	1
1397	3473	1
1397	4333	1
1397	4341	1
1397	10125	1
1397	14306	29

1397	14307	2
1397	14308	1
1397	14309	1
1398	371 548	
1398	377 512	
1398	378 723	
1398	394 559	
1398	395 1085	
1398	399 2092	
1398	463 698	
1398	472 1606	
1398	481 483	
1398	488 408	
1398	538 500	
1398	966 1193	
1398	1246	1295
1398	1377	490
1398	1686	518
1398	1854	628
1398	1866	395
1398	2453	347
1398	2467	638
1398	2840	524
1398	3057	450
1398	3078	821
1398	3097	820
1398	3616	368
1398	3856	361
1398	4177	414
1398	5080	1212

1398	7756	353
1398	10583	396
1398	11236	329
1398	14310	2138
1398	14311	1272
1398	14312	959
1398	14313	722
1398	14314	698
1398	14315	691
1398	14316	579
1398	14317	554
1398	14318	542
1398	14319	527
1398	14320	499
1398	14321	487
1398	14322	485
1398	14323	471
1398	14324	468
1398	14325	446
1398	14326	440
1398	14327	433
1398	14328	362
1398	14329	360
1399	55	177
1399	67	16
1399	89	3506
1399	288	278
1399	289	661
1399	292	514
1399	295	455

1399	299	41
1399	300	144
1399	302	92
1399	318	21
1399	321	26
1399	324	32
1399	325	57
1399	333	165
1399	340	79
1399	349	16
1399	461	192
1399	464	33
1399	466	471
1399	468	33
1399	523	84
1399	528	17
1399	531	35
1399	542	25
1399	543	18
1399	679	1418
1399	681	39
1399	686	1023
1399	687	45
1399	692	198
1399	701	20
1399	704	282
1399	793	236
1399	797	69
1399	913	39
1399	1054	68

1399	1450	27
1399	1453	21
1399	1458	109
1399	2020	69
1399	2543	568
1399	3953	30
1399	4814	54
1399	14330	32
1399	14331	30
1399	14332	29
1399	14333	20
1399	14334	19
1399	14335	16
1401	67	630
1401	157	694
1401	250	224
1401	282	668
1401	288	597
1401	291	195
1401	295	584
1401	306	410
1401	327	250
1401	328	391
1401	329	191
1401	330	262
1401	331	396
1401	349	996
1401	377	957
1401	485	279
1401	525	291

1401	526	295
1401	536	792
1401	544	959
1401	689	563
1401	706	2696
1401	917	280
1401	966	425
1401	1081	1423
1401	1241	1215
1401	1433	3302
1401	1443	1578
1401	1444	207
1401	1449	839
1401	1500	224
1401	1872	1212
1401	1873	1090
1401	1908	197
1401	2247	327
1401	2352	667
1401	2976	546
1401	3909	1726
1401	5073	215
1401	5239	248
1401	5772	197
1401	11139	290
1401	13292	2312
1401	13296	2646
1401	13303	870
1401	14339	288
1401	14340	275

1401	14341	256
1401	14342	197
1401	14343	191
1402	99	88
1402	159	35
1402	163	526
1402	172	56
1402	220	108
1402	227	250
1402	234	118
1402	429	9
1402	436	634
1402	441	33
1402	712	6
1402	733	11
1402	854	15
1402	855	33
1402	856	38
1402	863	6
1402	867	8
1402	868	49
1402	917	6
1402	982	23
1402	1110	27
1402	1369	26
1402	1412	60
1402	1413	187
1402	1425	115
1402	1426	7
1402	1504	79

1402	1639	8
1402	1806	74
1402	1884	10
1402	2469	65
1402	2481	38
1402	2487	12
1402	2542	113
1402	2606	6
1402	2637	12
1402	2792	68
1402	3481	99
1402	3739	133
1402	3846	7
1402	3938	6
1402	5175	41
1402	5844	9
1402	7800	14
1402	9332	72
1402	9818	48
1402	12219	6
1402	14344	16
1402	14345	12
1402	14346	9
1403	45	322
1403	190	237
1403	207	1743
1403	209	285
1403	229	1126
1403	230	440
1403	289	1113

1403	291 224	
1403	293 837	
1403	298 617	
1403	300 1078	
1403	329 256	
1403	333 1111	
1403	377 645	
1403	403 342	
1403	412 376	
1403	427 282	
1403	430 978	
1403	498 564	
1403	501 392	
1403	533 458	
1403	538 243	
1403	540 331	
1403	546 423	
1403	683 416	
1403	716 361	
1403	768 242	
1403	903 5563	
1403	982 1427	
1403	985 332	
1403	1043	643
1403	1046	226
1403	1047	1037
1403	1048	311
1403	1182	502
1403	1186	243
1403	1201	973

1403	1246	217
1403	1513	266
1403	1515	219
1403	2196	299
1403	2346	339
1403	2521	368
1403	3171	419
1403	3749	339
1403	5001	303
1403	5948	467
1403	7187	550
1403	9160	239
1403	10327	389
1404	154	2225
1404	197	2953
1404	199	1646
1404	211	594
1404	212	2126
1404	219	1737
1404	223	654
1404	227	508
1404	416	1780
1404	431	612
1404	434	1947
1404	440	1368
1404	554	866
1404	565	497
1404	602	2239
1404	609	590
1404	612	829

1404	615 624	
1404	622 854	
1404	626 568	
1404	632 551	
1404	709 714	
1404	715 545	
1404	859 529	
1404	863 650	
1404	867 665	
1404	1150	608
1404	1424	996
1404	1504	647
1404	1519	515
1404	1633	616
1404	1958	639
1404	1976	1454
1404	2277	708
1404	2281	1034
1404	2424	596
1404	2475	877
1404	2640	713
1404	2641	536
1404	3258	1749
1404	3691	612
1404	3693	1096
1404	4079	1743
1404	4532	786
1404	4538	497
1404	7147	692
1404	8109	718

1404	11927	672
1404	12220	549
1404	13000	593
1405	88	556
1405	167	1171
1405	195	727
1405	199	947
1405	205	1729
1405	207	676
1405	210	234
1405	211	1134
1405	217	360
1405	219	801
1405	222	319
1405	235	622
1405	236	684
1405	416	526
1405	418	269
1405	434	530
1405	440	543
1405	454	234
1405	602	385
1405	604	591
1405	618	1767
1405	622	1164
1405	629	538
1405	709	254
1405	716	384
1405	1090	330
1405	1097	252

1405	1106	602
1405	1164	712
1405	1390	225
1405	1777	218
1405	1941	577
1405	1949	817
1405	1976	247
1405	2405	313
1405	2424	2568
1405	2535	389
1405	2632	446
1405	2640	1590
1405	3258	885
1405	3666	355
1405	3690	503
1405	3693	695
1405	4118	638
1405	5150	238
1405	6224	855
1405	6297	396
1405	6915	394
1405	7147	722
1405	8030	553
1406	154 1477	
1406	190 237	
1406	217 470	
1406	225 380	
1406	226 293	
1406	228 250	
1406	234 324	

1406	237	381
1406	427	319
1406	703	192
1406	858	184
1406	859	174
1406	882	753
1406	977	433
1406	1167	167
1406	1171	194
1406	1974	329
1406	2398	399
1406	2580	967
1406	2582	372
1406	2627	468
1406	2632	487
1406	2633	400
1406	2639	161
1406	3299	448
1406	3354	212
1406	3357	272
1406	3364	281
1406	3689	227
1406	3690	219
1406	3714	1019
1406	3797	754
1406	6229	181
1406	8642	753
1406	8656	291
1406	9631	234
1406	9727	203

1406	9955	462
1406	14347	646
1406	14348	617
1406	14349	580
1406	14350	555
1406	14351	550
1406	14352	504
1406	14353	333
1406	14354	306
1406	14355	250
1406	14356	183
1406	14357	168
1406	14358	158
1407	65	977
1407	154	721
1407	157	156
1407	171	421
1407	182	1173
1407	184	204
1407	190	274
1407	203	126
1407	217	1050
1407	221	1026
1407	225	169
1407	227	1077
1407	228	285
1407	229	721
1407	298	404
1407	424	323
1407	441	269

1407	511 792	
1407	533 2972	
1407	554 140	
1407	715 228	
1407	734 246	
1407	851 425	
1407	863 116	
1407	1048	482
1407	1089	291
1407	1090	518
1407	1151	239
1407	1239	152
1407	1241	229
1407	1375	302
1407	1377	137
1407	1384	752
1407	1390	159
1407	1412	125
1407	1509	116
1407	1512	143
1407	2487	125
1407	2539	202
1407	2606	789
1407	2614	148
1407	3469	248
1407	3676	192
1407	4444	484
1407	4999	205
1407	6920	214
1407	7725	145

1407	8810	131
1407	9823	337
1407	14359	172
1408	172 1	
1408	187 1	
1408	267 1	
1408	289 7	
1408	475 1	
1408	961 1	
1408	989 1	
1408	1062	1
1408	1085	1
1408	1130	1
1408	1231	2
1408	1601	2
1408	1655	5
1408	2179	6
1408	2716	1
1408	2924	1
1408	3335	1
1408	3720	1
1408	3771	1
1408	4230	4
1408	4349	1
1408	6598	2
1408	6618	1
1408	6621	1
1408	6631	1
1408	6632	1
1408	6636	1

1408	7038	2
1408	8587	6
1408	9692	1
1408	9858	1
1408	10616	2
1408	14343	1
1408	14360	2
1408	14361	2
1408	14362	2
1408	14363	2
1408	14364	2
1408	14365	2
1408	14366	2
1408	14367	1
1408	14368	1
1408	14369	1
1408	14370	1
1408	14371	1
1408	14372	1
1408	14373	1
1408	14374	1
1408	14375	1
1408	14376	1
1410	89	157
1410	190	78
1410	191	55
1410	217	28
1410	229	25
1410	237	25
1410	288	36

1410	289	138
1410	300	1096
1410	329	1193
1410	333	252
1410	344	13113
1410	356	450
1410	377	26
1410	458	1027
1410	461	5893
1410	466	1033
1410	498	27
1410	681	21
1410	683	38
1410	685	84
1410	686	78
1410	691	23
1410	793	936
1410	797	294
1410	912	428
1410	1042	33
1410	1043	51
1410	1447	42
1410	1459	1067
1410	1602	28
1410	1609	44
1410	1696	68
1410	2436	23
1410	2439	107
1410	2446	92
1410	2982	38

1410	4575	28
1410	4923	217
1410	5266	645
1410	6217	106
1410	7276	21
1410	9271	46
1410	9849	73
1410	12918	34
1410	14386	444
1410	14387	135
1410	14388	91
1410	14389	90
1410	14390	36
1411	63	67
1411	65	824
1411	67	162
1411	154	2144
1411	155	93
1411	163	690
1411	173	70
1411	184	64
1411	190	887
1411	212	51
1411	234	180
1411	238	294
1411	316	96
1411	730	70
1411	748	752
1411	868	80
1411	959	136

1411	1150	55
1411	1412	45
1411	1418	379
1411	1425	80
1411	1464	38
1411	1743	329
1411	1800	75
1411	2070	42
1411	2176	91
1411	3265	285
1411	3267	132
1411	3302	78
1411	3478	55
1411	3821	72
1411	3861	63
1411	4649	246
1411	5624	146
1411	8229	142
1411	10968	62
1411	11765	379
1411	12119	69
1411	12137	96
1411	14391	403
1411	14392	385
1411	14393	240
1411	14394	182
1411	14395	158
1411	14396	152
1411	14397	111
1411	14398	99

1411	14399	51
1411	14400	51
1411	14401	44
1413	55	1163
1413	67	66
1413	89	2141
1413	161	2666
1413	209	72
1413	288	2225
1413	289	13694
1413	291	169
1413	292	1011
1413	295	165
1413	299	1161
1413	300	3485
1413	301	115
1413	302	1629
1413	306	2161
1413	326	45
1413	327	35
1413	328	84
1413	330	955
1413	332	107
1413	333	404
1413	344	135
1413	377	31
1413	378	44
1413	458	33
1413	461	202
1413	466	3175

1413	468	297
1413	475	575
1413	484	238
1413	525	335
1413	531	39
1413	534	141
1413	543	1230
1413	544	81
1413	545	367
1413	686	278
1413	701	397
1413	889	153
1413	907	39
1413	1037	140
1413	1042	72
1413	1243	52
1413	1377	40
1413	1452	1521
1413	1455	80
1413	2598	49
1413	4561	63
1413	7727	46
1413	14410	43
1414	65	1662
1414	190	697
1414	217	418
1414	220	199
1414	228	270
1414	229	407
1414	371	141

1414 377 345
1414 436 5078
1414 486 154
1414 490 164
1414 498 243
1414 499 275
1414 501 156
1414 533 223
1414 614 200
1414 715 149
1414 808 636
1414 978 157
1414 982 620
1414 1091 201
1414 1106 173
1414 1116 206
1414 1131 805
1414 1181 357
1414 1239 209
1414 1375 777
1414 1378 267
1414 1400 286
1414 1412 1155
1414 1632 829
1414 1854 522
1414 1866 510
1414 1905 272
1414 2206 317
1414 2610 208
1414 2661 193

1414	2829	225
1414	2840	311
1414	2850	599
1414	3084	153
1414	3621	448
1414	3769	289
1414	3946	218
1414	4074	3277
1414	4262	200
1414	5547	474
1414	7177	281
1414	7183	192
1414	7188	281
1416	171 292	
1416	196 331	
1416	199 334	
1416	207 1457	
1416	217 1318	
1416	225 1221	
1416	226 554	
1416	227 3722	
1416	228 2485	
1416	229 2317	
1416	231 1671	
1416	416 626	
1416	421 623	
1416	422 252	
1416	424 1021	
1416	425 452	
1416	427 336	

1416 428 605

1416 439 2046

1416 533 296

1416 554 263

1416 604 1095

1416 614 683

1416 622 561

1416 703 307

1416 982 428

1416 1048 1574

1416 1090 953

1416 1106 508

1416 1116 362

1416 1400 270

1416 1502 318

1416 1515 409

1416 1766 297

1416 1977 471

1416 2131 267

1416 2277 496

1416 2600 322

1416 2660 363

1416 3030 533

1416 3057 302

1416 3400 658

1416 4114 861

1416 4115 830

1416 4118 256

1416 5439 412

1416 6147 389

1416 6726 502

1416 8710 306

1416 8840 351

1418 89 75414

1418 173 423

1418 190 27211

1418 292 1283

1418 325 2242

1418 333 25575

1418 377 18012

1418 412 393

1418 464 4175

1418 466 11945

1418 475 917

1418 486 395

1418 498 31834

1418 500 652

1418 536 28859

1418 680 11594

1418 701 853

1418 768 24663

1418 779 631

1418 1035 4649

1418 1185 8861

1418 1187 13831

1418 1188 15147

1418 1191 30566

1418 1455 1239

1418 1541 39826

1418 1636 393

1418	2200	2689
1418	2203	400
1418	2210	6156
1418	4936	2099
1418	5125	795
1418	5308	2134
1418	5961	394
1418	5962	552
1418	5967	1404
1418	6012	615
1418	6448	1168
1418	6614	392
1418	7943	449
1418	8682	1704
1418	14441	3700
1418	14442	1816
1418	14443	1814
1418	14444	1716
1418	14445	1343
1418	14446	1284
1418	14447	948
1418	14448	654
1418	14449	540
1419	65	763
1419	70	212
1419	71	225
1419	88	223
1419	182	824
1419	189	505
1419	198	230

1419	229 467	
1419	298 269	
1419	302 639	
1419	331 290	
1419	362 257	
1419	377 348	
1419	389 328	
1419	475 679	
1419	489 384	
1419	503 254	
1419	517 773	
1419	518 283	
1419	538 234	
1419	691 993	
1419	707 195	
1419	1306	313
1419	1375	208
1419	1377	523
1419	1410	258
1419	1470	230
1419	1613	207
1419	1852	219
1419	1854	324
1419	2176	256
1419	2449	287
1419	2526	198
1419	2607	224
1419	2627	211
1419	2661	270
1419	3097	250

1419	3427	836
1419	3459	891
1419	3712	192
1419	3753	299
1419	3756	228
1419	3859	287
1419	5481	449
1419	6249	250
1419	9193	219
1419	10526	340
1419	11754	210
1419	14450	419
1419	14451	248
1420	81	376
1420	154	351
1420	173	120
1420	190	134
1420	207	196
1420	212	85
1420	215	107
1420	225	105
1420	227	623
1420	228	185
1420	236	130
1420	420	111
1420	425	415
1420	440	118
1420	441	164
1420	445	143
1420	447	139

1420 539 327

1420 714 92

1420 858 90

1420 868 332

1420 932 103

1420 1090 145

1420 1389 115

1420 1394 92

1420 1400 109

1420 1424 267

1420 1504 93

1420 1513 163

1420 1766 121

1420 1775 120

1420 3468 99

1420 3747 174

1420 4063 158

1420 4793 199

1420 4819 151

1420 5416 177

1420 5660 136

1420 5925 136

1420 7351 91

1420 8707 537

1420 8708 234

1420 8710 85

1420 12410 184

1420 13716 293

1420 14222 103

1420 14452 190

1420	14453	145
1420	14454	86
1420	14455	85
1421	155 102	
1421	197 194	
1421	200 38	
1421	202 62	
1421	227 38	
1421	235 205	
1421	428 47	
1421	436 85	
1421	599 400	
1421	609 65	
1421	622 39	
1421	869 110	
1421	961 54	
1421	1150	37
1421	1231	70
1421	1380	169
1421	1389	43
1421	1814	46
1421	1883	50
1421	2136	44
1421	2475	64
1421	2629	60
1421	2632	62
1421	3616	88
1421	4354	57
1421	4435	51
1421	4451	329

1421	5605	194
1421	6685	52
1421	6730	75
1421	7146	49
1421	7329	46
1421	10203	78
1421	11253	128
1421	13406	45
1421	14456	94
1421	14457	87
1421	14458	82
1421	14459	82
1421	14460	77
1421	14461	74
1421	14462	74
1421	14463	66
1421	14464	61
1421	14465	59
1421	14466	53
1421	14467	48
1421	14468	47
1421	14469	46
1421	14470	40
1422	56	64
1422	65	406
1422	157	108
1422	182	84
1422	190	1242
1422	198	68
1422	210	370

1422	220	69
1422	223	264
1422	228	197
1422	230	205
1422	234	84
1422	412	1825
1422	418	150
1422	436	89
1422	486	146
1422	493	250
1422	498	109
1422	501	4390
1422	511	272
1422	540	670
1422	546	75
1422	689	109
1422	691	115
1422	707	97
1422	768	199
1422	969	93
1422	982	548
1422	1116	148
1422	1131	76
1422	1183	103
1422	1375	185
1422	1384	75
1422	1398	140
1422	1410	93
1422	1470	171
1422	2150	459

1422	2580	1012
1422	2582	717
1422	2612	71
1422	2614	163
1422	3398	104
1422	3411	72
1422	4074	188
1422	5000	270
1422	6052	124
1422	6077	213
1422	6196	557
1422	14471	331
1422	14472	71
1423	67	331
1423	89	492
1423	157	161
1423	257	168
1423	288	657
1423	289	1831
1423	290	284
1423	291	157
1423	292	322
1423	294	497
1423	295	239
1423	300	643
1423	301	170
1423	302	579
1423	304	1592
1423	310	232
1423	318	1118

1423	321 447	
1423	325 440	
1423	327 1148	
1423	330 447	
1423	331 365	
1423	344 662	
1423	352 459	
1423	386 167	
1423	461 577	
1423	466 300	
1423	468 206	
1423	474 224	
1423	476 306	
1423	481 197	
1423	484 414	
1423	641 161	
1423	674 788	
1423	681 295	
1423	686 441	
1423	1042	1563
1423	1047	150
1423	1436	198
1423	1444	906
1423	2390	183
1423	2645	274
1423	3570	148
1423	4562	361
1423	4817	146
1423	5535	166
1423	6505	242

1423	7340	902
1423	9183	271
1423	10608	291
1424	65	1629
1424	154	1571
1424	159	1080
1424	163	288
1424	182	1135
1424	190	415
1424	199	405
1424	215	747
1424	220	303
1424	225	333
1424	227	1318
1424	229	597
1424	418	827
1424	424	630
1424	429	1066
1424	439	1559
1424	498	250
1424	511	502
1424	604	1284
1424	714	240
1424	813	523
1424	823	451
1424	986	593
1424	1045	660
1424	1151	493
1424	1185	333
1424	1239	243

1424	1242	439
1424	1377	341
1424	1414	243
1424	1541	587
1424	1755	478
1424	1761	352
1424	1766	242
1424	2531	363
1424	2608	270
1424	2661	389
1424	2850	243
1424	3026	262
1424	3411	276
1424	3468	311
1424	4170	291
1424	4828	353
1424	4831	238
1424	5444	425
1424	6132	317
1424	6772	480
1424	8710	310
1424	12957	375
1424	14473	339
1425	298 1504	
1425	423 1536	
1425	430 3062	
1425	485 1516	
1425	526 851	
1425	532 1113	
1425	539 1245	

1425	548	2505
1425	689	10366
1425	690	7195
1425	703	1086
1425	757	1069
1425	895	1504
1425	901	1493
1425	1097	356
1425	1109	609
1425	1159	321
1425	1167	2670
1425	1485	276
1425	1609	649
1425	1934	489
1425	1960	564
1425	1971	477
1425	2083	491
1425	2521	854
1425	3057	6195
1425	3319	295
1425	3399	288
1425	3404	1710
1425	3488	317
1425	4252	244
1425	5048	236
1425	5428	307
1425	5754	2008
1425	6374	271
1425	7660	2191
1425	7743	739

1425	7781	1095
1425	8141	464
1425	8589	524
1425	8649	500
1425	8676	305
1425	9307	1296
1425	9947	1281
1425	11849	252
1425	13997	2485
1425	14474	751
1425	14475	549
1425	14476	308
1425	14477	268
1426	295 60	
1426	892 138	
1426	1220	82
1426	1244	161
1426	2902	307
1426	3581	81
1426	3611	64
1426	5375	76
1426	5390	105
1426	5392	152
1426	7224	69
1426	7235	164
1426	7239	407
1426	7681	360
1426	8309	155
1426	8560	146
1426	10543	66

1426	10630	67
1426	14072	114
1426	14089	106
1426	14478	394
1426	14479	313
1426	14480	312
1426	14481	285
1426	14482	244
1426	14483	173
1426	14484	146
1426	14485	129
1426	14486	117
1426	14487	113
1426	14488	112
1426	14489	112
1426	14490	110
1426	14491	98
1426	14492	90
1426	14493	87
1426	14494	77
1426	14495	75
1426	14496	73
1426	14497	73
1426	14498	68
1426	14499	67
1426	14500	67
1426	14501	67
1426	14502	66
1426	14503	64
1426	14504	63

1426	14505	62
1426	14506	61
1426	14507	59
1427	19	635
1427	30	493
1427	36	533
1427	37	349
1427	51	2485
1427	72	6566
1427	75	473
1427	153	644
1427	159	729
1427	187	328
1427	429	468
1427	603	340
1427	631	343
1427	875	402
1427	877	699
1427	985	561
1427	986	573
1427	997	346
1427	998	591
1427	1001	1060
1427	1014	2128
1427	1019	1062
1427	1073	570
1427	1098	341
1427	1225	547
1427	1253	735
1427	1298	387

1427	1464	418
1427	1709	627
1427	1886	806
1427	1892	325
1427	2459	550
1427	2531	374
1427	2638	683
1427	3003	380
1427	3015	336
1427	3289	789
1427	3863	357
1427	3889	362
1427	7344	339
1427	8149	420
1427	9302	442
1427	10635	506
1427	11206	385
1427	13451	567
1427	14294	398
1427	14508	615
1427	14509	599
1427	14510	416
1427	14511	355
1428	159 1950	
1428	196 269	
1428	207 393	
1428	212 2006	
1428	220 416	
1428	234 767	
1428	316 1891	

1428	333 1110	
1428	383 352	
1428	428 356	
1428	498 674	
1428	533 464	
1428	542 400	
1428	562 624	
1428	706 740	
1428	707 718	
1428	714 2346	
1428	726 410	
1428	754 321	
1428	815 864	
1428	841 465	
1428	917 628	
1428	918 378	
1428	924 273	
1428	928 363	
1428	932 4703	
1428	937 316	
1428	982 510	
1428	1246	550
1428	1249	576
1428	1274	277
1428	1281	278
1428	1351	441
1428	1360	506
1428	1364	474
1428	1372	271
1428	1406	467

1428	1521	310
1428	1545	344
1428	1651	362
1428	1672	815
1428	2664	287
1428	2834	416
1428	2861	343
1428	2963	289
1428	3427	533
1428	4087	308
1428	4156	326
1428	4821	279
1428	7329	844
1429	89	1783
1429	191	467
1429	198	451
1429	288	1640
1429	289	1401
1429	291	1627
1429	292	3354
1429	293	451
1429	294	1854
1429	295	3121
1429	299	556
1429	300	1152
1429	302	882
1429	305	1024
1429	306	529
1429	311	793
1429	325	1155

1429	326 447	
1429	333 630	
1429	344 892	
1429	349 1142	
1429	377 465	
1429	458 605	
1429	461 552	
1429	466 450	
1429	498 1010	
1429	523 679	
1429	543 845	
1429	544 1514	
1429	679 2871	
1429	681 815	
1429	682 1108	
1429	701 1300	
1429	909 427	
1429	930 1612	
1429	966 827	
1429	1034	730
1429	1051	397
1429	1098	1025
1429	1114	16734
1429	1246	1146
1429	1451	570
1429	1458	1027
1429	1673	1145
1429	1684	857
1429	2094	455
1429	2990	482

1429	3393	708
1429	6690	415
1429	7864	762
1430	65	114
1430	154	106
1430	159	83
1430	199	96
1430	227	381
1430	228	114
1430	229	244
1430	234	86
1430	297	357
1430	307	69
1430	412	122
1430	498	575
1430	546	84
1430	706	77
1430	709	73
1430	716	83
1430	840	111
1430	982	169
1430	1048	136
1430	1097	108
1430	1105	639
1430	1375	279
1430	1377	82
1430	1400	222
1430	1502	406
1430	1512	83
1430	1520	123

1430	1866	467
1430	2346	79
1430	2398	160
1430	2629	129
1430	3078	112
1430	3321	244
1430	3749	197
1430	4432	99
1430	4756	383
1430	5099	79
1430	5310	85
1430	5423	102
1430	5437	462
1430	5440	494
1430	5925	127
1430	6247	296
1430	6298	670
1430	6554	69
1430	7723	108
1430	8307	84
1430	9662	156
1430	14512	110
1430	14513	107
1431	55	321
1431	67	1056
1431	71	1069
1431	89	138
1431	155	995
1431	161	428
1431	180	327

1431	246	166
1431	261	1620
1431	265	455
1431	270	95
1431	289	104
1431	298	147
1431	316	540
1431	389	1182
1431	641	3446
1431	701	98
1431	714	2360
1431	727	532
1431	791	1346
1431	794	2812
1431	891	343
1431	1243	156
1431	1464	100
1431	1505	2056
1431	1760	2304
1431	1771	309
1431	2024	92
1431	2044	60845
1431	2051	2628
1431	2054	1099
1431	2075	989
1431	2523	185
1431	3307	123
1431	3332	329
1431	5458	320
1431	6721	736

1431	7505	91
1431	10022	750
1431	11473	689
1431	11579	96
1431	12961	376
1431	14514	954
1431	14515	883
1431	14516	468
1431	14517	254
1431	14518	243
1431	14519	226
1431	14520	152
1431	14521	130
1432	57	1
1432	72	1
1432	182	1
1432	3768	1
1432	5072	1
1432	8140	1
1432	14522	1
1432	14523	1
1432	14524	1
1433	65	415
1433	67	527
1433	157	701
1433	207	796
1433	208	650
1433	257	797
1433	265	607
1433	289	812

1433	461	420
1433	466	782
1433	478	660
1433	530	460
1433	620	607
1433	1048	517
1433	1098	1020
1433	1394	529
1433	2500	989
1433	2514	554
1433	2544	478
1433	2615	405
1433	2664	1480
1433	3046	474
1433	3110	461
1433	3119	1123
1433	3266	612
1433	3466	916
1433	3947	444
1433	3991	513
1433	4126	606
1433	4260	613
1433	4999	419
1433	5000	495
1433	5440	437
1433	6232	746
1433	7395	573
1433	7655	438
1433	9751	1987
1433	10436	585

1433	11724	1121
1433	12987	427
1433	14525	718
1433	14526	712
1433	14527	705
1433	14528	642
1433	14529	529
1433	14530	504
1433	14531	494
1433	14532	465
1433	14533	437
1433	14534	403
1434	65	355
1434	67	1654
1434	89	605
1434	155	819
1434	161	459
1434	288	699
1434	289	1287
1434	290	348
1434	291	198
1434	292	23859
1434	294	241
1434	299	308
1434	300	1221
1434	301	183
1434	302	19898
1434	306	322
1434	310	489
1434	340	319

1434	386	304
1434	466	1588
1434	474	734
1434	475	253
1434	526	335
1434	544	4093
1434	641	3954
1434	679	3661
1434	701	1482
1434	903	281
1434	972	1125
1434	1009	1609
1434	1037	355
1434	1042	672
1434	1377	653
1434	1429	511
1434	1934	1034
1434	1936	281
1434	2440	217
1434	5758	464
1434	6426	172
1434	8372	396
1434	9907	478
1434	12959	674
1434	13735	171
1434	14535	641
1434	14536	283
1434	14537	226
1434	14538	200
1434	14539	197

1434	14540	182
1434	14541	177
1435	55	84
1435	67	62
1435	89	714
1435	157	195
1435	227	92
1435	251	534
1435	257	20987
1435	262	229
1435	265	113
1435	285	1083
1435	288	298
1435	289	282
1435	291	602
1435	292	414
1435	294	194
1435	295	608
1435	299	73
1435	301	612
1435	302	148
1435	309	254
1435	310	100
1435	318	1180
1435	326	88
1435	331	83
1435	461	117
1435	523	296
1435	525	91
1435	575	63

1435	680	175
1435	790	96
1435	903	154
1435	989	103
1435	1052	108
1435	1243	125
1435	1580	94
1435	1596	352
1435	2019	385
1435	2083	126
1435	2390	247
1435	4696	127
1435	4701	157
1435	4702	494
1435	11081	93
1435	14542	1324
1435	14543	266
1435	14544	160
1435	14545	149
1435	14546	111
1435	14547	67
1435	14548	63
1437	65	42
1437	89	58
1437	257	27
1437	288	72
1437	289	53
1437	295	64
1437	300	43
1437	306	32

1437	328	38
1437	349	52
1437	377	15
1437	461	43
1437	466	21
1437	468	54
1437	475	26
1437	481	44
1437	498	45
1437	542	125
1437	544	26
1437	686	33
1437	790	27
1437	802	23
1437	903	55
1437	1032	15
1437	1042	21
1437	1429	25
1437	1452	50
1437	1455	21
1437	1681	37
1437	1860	17
1437	2984	18
1437	3173	50
1437	3378	27
1437	3386	34
1437	4474	42
1437	4561	16
1437	4811	31
1437	5099	27

1437	8364	17
1437	10992	19
1437	14550	36
1437	14551	31
1437	14552	25
1437	14553	24
1437	14554	23
1437	14555	19
1437	14556	16
1437	14557	16
1437	14558	15
1437	14559	15
1438	81	956
1438	154	2813
1438	196	3448
1438	204	959
1438	212	1007
1438	227	7572
1438	228	1323
1438	229	1155
1438	231	856
1438	250	1258
1438	366	707
1438	422	551
1438	424	1173
1438	437	569
1438	438	1242
1438	439	772
1438	445	1485
1438	447	611

1438	452 655	
1438	689 525	
1438	703 1539	
1438	705 3290	
1438	714 985	
1438	1048	3272
1438	1090	1036
1438	1106	589
1438	1109	2873
1438	1116	907
1438	1135	748
1438	1400	1617
1438	1414	659
1438	1755	3571
1438	1763	813
1438	1764	676
1438	1776	521
1438	1778	755
1438	2786	954
1438	3741	1222
1438	3902	1590
1438	5180	652
1438	5441	719
1438	5453	657
1438	6726	633
1438	8264	623
1438	8716	605
1438	9831	604
1438	10427	882
1438	14560	1026

1438	14561	1011
1438	14562	540
1439	72	530
1439	152	312
1439	154	859
1439	159	1082
1439	166	513
1439	173	309
1439	190	256
1439	215	587
1439	227	670
1439	229	371
1439	233	533
1439	316	445
1439	366	711
1439	436	3800
1439	438	255
1439	689	1276
1439	892	1019
1439	967	358
1439	982	379
1439	986	248
1439	1116	1041
1439	1118	308
1439	1145	930
1439	1643	386
1439	1666	3277
1439	1672	299
1439	2006	319
1439	2265	643

1439	2346	351
1439	3057	559
1439	3084	849
1439	3110	456
1439	3119	336
1439	3171	295
1439	3193	429
1439	3373	456
1439	4616	263
1439	5184	484
1439	5216	256
1439	6678	349
1439	6724	265
1439	7140	293
1439	7777	525
1439	8043	319
1439	10416	267
1439	11177	1333
1439	12773	1087
1439	14563	867
1439	14564	435
1439	14565	264
1440	257	380
1440	261	228
1440	265	153
1440	283	620
1440	289	280
1440	316	192
1440	360	133
1440	378	137

1440	701 172	
1440	714 385	
1440	786 134	
1440	1755	131
1440	1763	280
1440	1775	319
1440	2545	307
1440	3182	230
1440	3332	517
1440	3376	218
1440	3380	240
1440	3383	212
1440	3386	143
1440	3393	284
1440	3741	136
1440	4349	313
1440	4473	128
1440	4869	214
1440	6706	341
1440	6713	134
1440	7094	275
1440	7452	352
1440	9170	245
1440	10459	255
1440	10460	129
1440	11249	245
1440	11764	233
1440	13553	126
1440	14566	1579
1440	14567	381

1440	14568	361
1440	14569	339
1440	14570	302
1440	14571	246
1440	14572	230
1440	14573	213
1440	14574	198
1440	14575	196
1440	14576	166
1440	14577	165
1440	14578	139
1440	14579	125
1441	56	127
1441	89	158
1441	154	233
1441	196	148
1441	199	2658
1441	207	1752
1441	217	210
1441	221	143
1441	225	721
1441	227	409
1441	229	299
1441	298	113
1441	403	227
1441	421	355
1441	424	2659
1441	425	121
1441	430	113
1441	432	89

1441	437 944	
1441	439 239	
1441	533 199	
1441	679 138	
1441	689 910	
1441	690 556	
1441	709 151	
1441	951 277	
1441	969 209	
1441	1048	98
1441	1090	231
1441	1390	431
1441	1398	105
1441	1400	790
1441	1412	270
1441	1512	181
1441	1515	353
1441	2123	106
1441	2134	101
1441	2265	153
1441	2433	120
1441	2611	127
1441	2821	106
1441	3057	182
1441	4247	250
1441	4731	351
1441	5547	603
1441	6726	115
1441	7177	119
1441	10657	360

1441	12753	103
1441	14580	106
1442	45	392
1442	370	369
1442	375	417
1442	486	523
1442	500	316
1442	503	380
1442	506	350
1442	519	558
1442	689	341
1442	768	784
1442	805	933
1442	806	1061
1442	808	577
1442	810	454
1442	813	803
1442	814	327
1442	815	674
1442	823	710
1442	827	497
1442	830	662
1442	832	549
1442	850	440
1442	964	335
1442	1091	349
1442	1179	1424
1442	1191	380
1442	1195	492
1442	1541	414

1442	1861	708
1442	1982	503
1442	2379	346
1442	2744	601
1442	2850	714
1442	3027	567
1442	3032	1917
1442	3428	413
1442	3646	582
1442	4100	776
1442	4102	316
1442	4103	762
1442	4167	374
1442	4948	327
1442	5631	380
1442	5966	360
1442	8462	559
1442	12779	375
1442	13523	614
1442	14581	622
1442	14582	500
1442	14583	328
1443	86	34
1443	157	28
1443	159	35
1443	163	133
1443	166	45
1443	176	28
1443	227	66
1443	705	42

1443	706	43
1443	707	36
1443	712	43
1443	714	122
1443	715	70
1443	725	34
1443	728	34
1443	868	110
1443	889	41
1443	917	34
1443	924	47
1443	951	70
1443	952	28
1443	959	322
1443	1244	35
1443	1249	48
1443	1349	40
1443	1412	72
1443	1513	43
1443	1768	33
1443	1775	29
1443	1803	42
1443	1807	55
1443	2265	37
1443	2786	55
1443	2838	34
1443	2848	29
1443	3204	64
1443	3741	29
1443	3742	28

1443	3745	54
1443	4031	43
1443	4165	90
1443	4180	60
1443	5455	29
1443	5772	81
1443	6722	65
1443	8649	41
1443	9822	66
1443	14584	36
1443	14585	32
1443	14586	32
1444	321 1024	
1444	333 685	
1444	412 1298	
1444	419 534	
1444	470 468	
1444	471 358	
1444	498 5073	
1444	499 451	
1444	500 464	
1444	507 576	
1444	536 368	
1444	540 778	
1444	687 1332	
1444	691 475	
1444	816 366	
1444	982 387	
1444	1040	2326
1444	1045	618

1444	1046	930
1444	1180	658
1444	1192	1108
1444	1198	1032
1444	1201	1273
1444	1246	507
1444	1866	714
1444	2080	1154
1444	2546	456
1444	2850	1073
1444	2987	619
1444	3078	857
1444	3373	530
1444	3398	397
1444	4828	1747
1444	4850	619
1444	5313	929
1444	6597	607
1444	6600	450
1444	6793	969
1444	7835	902
1444	8728	415
1444	8732	884
1444	11061	509
1444	11470	363
1444	12792	1296
1444	12793	1180
1444	13468	616
1444	14587	1475
1444	14588	1236

1444 14589 453
1444 14590 358
1445 55 496
1445 67 1205
1445 157 349
1445 209 418
1445 257 2215
1445 285 336
1445 288 932
1445 289 2861
1445 292 828
1445 295 624
1445 298 307
1445 299 546
1445 300 736
1445 318 590
1445 325 379
1445 333 683
1445 349 655
1445 369 1991
1445 375 604
1445 388 360
1445 393 328
1445 399 417
1445 400 732
1445 908 932
1445 1201 313
1445 1243 409
1445 1246 869
1445 1453 574

1445	1556	314
1445	1580	290
1445	1904	869
1445	1906	1878
1445	2089	355
1445	2091	1166
1445	2094	594
1445	2102	45544
1445	2803	392
1445	2808	367
1445	3199	1518
1445	4515	543
1445	4558	845
1445	5081	402
1445	5568	306
1445	7620	518
1445	12596	325
1445	14591	1650
1445	14592	620
1445	14593	373
1445	14594	373
1445	14595	276
1446	89	214
1446	180	118
1446	191	189
1446	220	860
1446	229	157
1446	230	333
1446	234	226
1446	288	636

1446	300	324
1446	302	128
1446	306	156
1446	321	534
1446	324	341
1446	333	792
1446	346	161
1446	461	1154
1446	466	289
1446	468	129
1446	475	585
1446	486	246
1446	531	163
1446	538	278
1446	680	207
1446	681	543
1446	686	584
1446	689	194
1446	697	273
1446	704	294
1446	757	524
1446	797	140
1446	907	171
1446	912	130
1446	1179	128
1446	1375	196
1446	1449	229
1446	1456	148
1446	2598	166
1446	3966	132

1446	4435	427
1446	5041	420
1446	5149	161
1446	8163	427
1446	8167	151
1446	10295	149
1446	14095	141
1446	14366	135
1446	14596	186
1446	14597	181
1446	14598	145
1446	14599	145
1447	55	68
1447	89	383
1447	288	857
1447	289	443
1447	291	1833
1447	295	23
1447	299	175
1447	300	37585
1447	302	621
1447	306	36
1447	325	33
1447	327	75
1447	333	53
1447	344	48
1447	437	580
1447	455	50
1447	458	66
1447	461	360

1447	466 329	
1447	484 42	
1447	531 112	
1447	679 121	
1447	691 1145	
1447	908 21	
1447	959 294	
1447	1032	373
1447	1042	305
1447	1934	44
1447	2020	104
1447	2067	49
1447	2080	50
1447	2084	85
1447	2184	1153
1447	2439	20
1447	3404	62
1447	3712	113
1447	3982	114
1447	4474	24
1447	5947	69
1447	14600	1103
1447	14601	509
1447	14602	113
1447	14603	53
1447	14604	45
1447	14605	33
1447	14606	31
1447	14607	29
1447	14608	29

1447	14609	27
1447	14610	24
1449	65	373
1449	154	89
1449	211	94
1449	212	271
1449	227	9629
1449	250	69
1449	464	75
1449	475	265
1449	614	83
1449	630	85
1449	709	62
1449	718	64
1449	728	572
1449	730	432
1449	733	773
1449	734	142
1449	735	94
1449	1150	977
1449	1249	112
1449	1384	67
1449	1412	392
1449	1414	571
1449	1415	133
1449	1416	300
1449	1422	304
1449	1426	77
1449	1518	85
1449	1613	71

1449	1643	143
1449	1976	64
1449	2179	57
1449	2265	2734
1449	2626	833
1449	2630	1240
1449	2855	130
1449	2893	344
1449	3458	290
1449	3670	102
1449	4079	936
1449	4538	98
1449	4780	259
1449	6140	60
1449	6853	159
1449	8122	174
1449	8312	57
1449	9689	123
1449	13962	189
1449	14618	592
1449	14619	82
1449	14620	58
1450	56	320
1450	65	231
1450	89	1518
1450	207	281
1450	324	245
1450	429	354
1450	464	294
1450	470	225

1450	537 3241	
1450	540 1163	
1450	683 399	
1450	770 324	
1450	779 561	
1450	1040	437
1450	1043	395
1450	1045	2690
1450	1099	295
1450	1182	235
1450	1185	908
1450	1191	1123
1450	1193	1137
1450	1201	701
1450	1239	306
1450	1246	950
1450	1390	194
1450	1541	235
1450	2080	438
1450	2425	550
1450	2430	957
1450	2587	297
1450	2661	689
1450	3040	436
1450	3057	287
1450	3373	885
1450	4039	278
1450	4756	288
1450	5426	237
1450	5969	514

1450	6132	788
1450	6453	1387
1450	6769	525
1450	7723	816
1450	7832	266
1450	8884	209
1450	12794	793
1450	13581	193
1450	14621	675
1450	14622	392
1450	14623	380
1450	14624	247
1451	89	358
1451	230	109
1451	241	83
1451	251	98
1451	252	130
1451	257	171
1451	288	166
1451	289	521
1451	290	177
1451	291	533
1451	292	757
1451	294	473
1451	295	140
1451	300	559
1451	301	185
1451	302	1100
1451	306	103
1451	307	145

1451	311 180	
1451	313 185	
1451	318 132	
1451	320 157	
1451	342 289	
1451	344 899	
1451	378 105	
1451	400 120	
1451	458 111	
1451	461 243	
1451	466 167	
1451	544 91	
1451	679 2389	
1451	691 311	
1451	903 108	
1451	966 116	
1451	1596	118
1451	1782	124
1451	1934	254
1451	2801	211
1451	3081	104
1451	3097	124
1451	3488	111
1451	3489	198
1451	4941	96
1451	5789	121
1451	7160	146
1451	8878	186
1451	9277	249
1451	14625	454

1451	14626	200
1451	14627	104
1452	159 43	
1452	190 27	
1452	209 23	
1452	230 24	
1452	293 54	
1452	294 151	
1452	305 51	
1452	313 124	
1452	324 90	
1452	325 2095	
1452	333 54	
1452	378 63	
1452	470 26	
1452	481 16	
1452	486 28	
1452	498 384	
1452	542 37	
1452	680 264	
1452	681 557	
1452	683 55	
1452	688 38	
1452	689 27	
1452	704 90	
1452	797 130	
1452	1034	1533
1452	1038	38
1452	1043	16
1452	1045	151

1452	1047	99
1452	1091	23
1452	1183	303
1452	1246	36
1452	2435	95
1452	2619	166
1452	2977	146
1452	2981	52
1452	3319	134
1452	3397	79
1452	4489	31
1452	4773	51
1452	5065	28
1452	5079	44
1452	5749	68
1452	5958	153
1452	5978	42
1452	5984	18
1452	10683	19
1452	14628	26
1452	14629	25
1452	14630	22
1453	56	194
1453	67	264
1453	89	1211
1453	285	144
1453	288	9545
1453	289	6859
1453	292	3404
1453	295	561

1453	296 304
1453	299 406
1453	300 948
1453	306 184
1453	308 351
1453	314 473
1453	318 330
1453	320 176
1453	324 124
1453	325 808
1453	328 134
1453	329 418
1453	331 157
1453	332 328
1453	333 2205
1453	349 727
1453	352 311
1453	386 554
1453	461 3523
1453	464 170
1453	466 2406
1453	523 1066
1453	535 241
1453	543 1320
1453	679 765
1453	681 309
1453	686 1913
1453	701 838
1453	704 1644
1453	793 323

1453	908	749
1453	1037	162
1453	1456	420
1453	2020	3023
1453	2515	127
1453	3123	152
1453	3397	209
1453	4565	286
1453	4765	1002
1453	4769	261
1453	4814	344
1453	10991	164
1454	51	332
1454	59	1557
1454	65	328
1454	68	1099
1454	72	2496
1454	75	2344
1454	76	184
1454	85	240
1454	93	182
1454	95	308
1454	99	597
1454	153	1297
1454	159	1857
1454	180	380
1454	182	343
1454	187	1429
1454	190	341
1454	193	313

1454	220	345
1454	229	251
1454	230	558
1454	253	232
1454	441	405
1454	511	2278
1454	599	350
1454	612	317
1454	715	884
1454	722	294
1454	863	699
1454	877	572
1454	993	3978
1454	996	496
1454	997	1476
1454	998	559
1454	1001	2677
1454	1005	283
1454	1013	1846
1454	1014	1102
1454	1072	305
1454	1073	559
1454	1413	218
1454	1464	192
1454	1707	424
1454	1816	392
1454	1892	180
1454	2469	225
1454	2936	491
1454	3302	266

1454	3988	412
1454	11794	2835
1455	67	729
1455	89	1734
1455	157	778
1455	230	789
1455	288	1677
1455	289	5274
1455	290	689
1455	291	13357
1455	292	694
1455	294	688
1455	295	802
1455	296	1561
1455	300	1248
1455	302	1867
1455	306	836
1455	310	870
1455	311	772
1455	314	566
1455	318	1038
1455	330	735
1455	333	5054
1455	342	735
1455	344	949
1455	349	1187
1455	377	1614
1455	407	870
1455	461	1518
1455	466	791

1455	475	972
1455	488	687
1455	491	564
1455	498	4115
1455	536	1922
1455	547	569
1455	679	1658
1455	680	2027
1455	908	591
1455	1057	587
1455	1459	577
1455	1684	999
1455	1687	570
1455	3491	582
1455	3575	1258
1455	3859	942
1455	4574	1093
1455	5310	3598
1455	5684	654
1455	5806	802
1455	6201	584
1455	6693	578
1456	51	1447
1456	55	1699
1456	64	1667
1456	65	409
1456	72	1154
1456	99	732
1456	173	478
1456	182	257

1456 187 20270

1456 190 241

1456 253 233

1456 418 609

1456 453 911

1456 471 825

1456 679 238

1456 904 567

1456 905 491

1456 909 3763

1456 912 865

1456 1376 439

1456 1383 237

1456 1505 3114

1456 1882 373

1456 1990 452

1456 2079 389

1456 2176 278

1456 3109 265

1456 3681 507

1456 3832 4901

1456 4025 506

1456 6297 761

1456 6308 434

1456 6733 1278

1456 6739 3341

1456 7651 2044

1456 8171 536

1456 8996 414

1456 13435 1302

1456	14631	6342
1456	14632	856
1456	14633	478
1456	14634	453
1456	14635	430
1456	14636	405
1456	14637	399
1456	14638	380
1456	14639	313
1456	14640	312
1456	14641	264
1456	14642	244
1457	89	749
1457	198	261
1457	230	1348
1457	288	1509
1457	289	8945
1457	291	601
1457	292	468
1457	293	5609
1457	295	680
1457	299	288
1457	300	1031
1457	302	844
1457	306	463
1457	309	380
1457	310	266
1457	313	313
1457	314	405
1457	317	402

1457	318 761	
1457	321 495	
1457	324 320	
1457	325 3843	
1457	333 1247	
1457	344 945	
1457	377 1320	
1457	458 484	
1457	461 7916	
1457	464 356	
1457	466 1901	
1457	475 1333	
1457	498 5398	
1457	499 276	
1457	523 441	
1457	528 382	
1457	531 268	
1457	540 288	
1457	679 1229	
1457	681 1543	
1457	686 400	
1457	693 530	
1457	701 476	
1457	704 260	
1457	908 388	
1457	1032	495
1457	1451	371
1457	1453	361
1457	1684	384
1457	1934	411

1457	2619	497
1457	2977	272
1458	67	56
1458	89	34
1458	161	13
1458	184	13
1458	239	14
1458	257	35
1458	288	37
1458	289	30
1458	291	21
1458	293	34
1458	295	54
1458	299	26
1458	301	13
1458	306	16
1458	316	45
1458	320	27
1458	329	27
1458	333	45
1458	342	18
1458	378	25
1458	455	16
1458	456	14
1458	461	51
1458	481	26
1458	486	48
1458	498	24
1458	536	17
1458	542	28

1458	679 32	
1458	903 54	
1458	1021	28
1458	1047	19
1458	1243	35
1458	1444	33
1458	1684	87
1458	1685	92
1458	1866	22
1458	2080	14
1458	2544	24
1458	3373	14
1458	3672	16
1458	4292	29
1458	4468	23
1458	5594	30
1458	6121	79
1458	7449	14
1458	8364	39
1458	8365	28
1458	14643	81
1458	14644	15
1459	64 71	
1459	163 134	
1459	166 163	
1459	196 279	
1459	197 77	
1459	214 57	
1459	223 67	
1459	227 95	

1459	429	174
1459	604	150
1459	618	61
1459	697	70
1459	706	67
1459	707	58
1459	852	58
1459	981	131
1459	1075	118
1459	1109	147
1459	1150	121
1459	1463	57
1459	2300	133
1459	2317	59
1459	2320	99
1459	2616	85
1459	3119	91
1459	3258	125
1459	3310	99
1459	6730	80
1459	6930	188
1459	6946	97
1459	7219	70
1459	7456	120
1459	8230	71
1459	8454	445
1459	9054	134
1459	12330	290
1459	13279	97
1459	13454	140

1459	13888	107
1459	13988	328
1459	14254	104
1459	14645	175
1459	14646	168
1459	14647	113
1459	14648	93
1459	14649	91
1459	14650	76
1459	14651	74
1459	14652	65
1459	14653	62
1461	89	387
1461	155	43
1461	209	166
1461	230	43
1461	288	40
1461	289	160
1461	291	629
1461	294	84
1461	298	220
1461	300	417
1461	302	75
1461	306	40
1461	318	150
1461	325	146
1461	333	901
1461	344	301
1461	377	277
1461	378	480

1461	386 46	
1461	403 47	
1461	412 103	
1461	458 72	
1461	461 41	
1461	466 348	
1461	471 65	
1461	475 211	
1461	481 268	
1461	498 154	
1461	525 108	
1461	540 94	
1461	679 1028	
1461	681 129	
1461	687 209	
1461	690 49	
1461	691 56	
1461	793 115	
1461	959 162	
1461	966 60	
1461	1047	155
1461	2176	160
1461	2939	39
1461	3033	53
1461	3859	46
1461	4559	52
1461	7183	69
1461	7431	53
1461	11590	244
1461	12967	37

1461	14661	64
1461	14662	47
1462	7	2064
1462	55	434
1462	67	219
1462	89	4249
1462	157	148
1462	207	172
1462	208	164
1462	209	2070
1462	227	163
1462	229	150
1462	230	204
1462	234	2071
1462	256	300
1462	288	2284
1462	289	2321
1462	292	1756
1462	295	679
1462	300	1093
1462	302	623
1462	306	163
1462	333	235
1462	378	173
1462	436	1238
1462	466	202
1462	470	1695
1462	481	232
1462	526	181
1462	539	499

1462	540	254
1462	599	1354
1462	679	1006
1462	701	651
1462	704	2391
1462	790	153
1462	903	242
1462	951	254
1462	959	1660
1462	1081	161
1462	1246	150
1462	1673	2596
1462	1904	231
1462	2091	164
1462	2988	176
1462	3129	396
1462	3186	318
1462	3739	7364
1462	5984	1029
1462	7237	170
1462	14663	387
1462	14664	383
1463	72	141
1463	173	114
1463	183	193
1463	214	102
1463	604	202
1463	728	98
1463	903	100
1463	1062	155

1463	1155	148
1463	1167	176
1463	1897	172
1463	1941	113
1463	2037	275
1463	2531	112
1463	3213	323
1463	3693	191
1463	3861	126
1463	4723	331
1463	4731	223
1463	4732	310
1463	4735	598
1463	5879	339
1463	7694	149
1463	7887	108
1463	8587	100
1463	8678	143
1463	9860	117
1463	10621	145
1463	11352	170
1463	14646	1404
1463	14665	430
1463	14666	369
1463	14667	313
1463	14668	168
1463	14669	161
1463	14670	153
1463	14671	148
1463	14672	145

1463	14673	142
1463	14674	142
1463	14675	141
1463	14676	139
1463	14677	133
1463	14678	123
1463	14679	123
1463	14680	113
1463	14681	113
1463	14682	111
1463	14683	104
1463	14684	100
1464	27	801
1464	64	314
1464	81	359
1464	161	294
1464	173	3366
1464	174	463
1464	190	1301
1464	198	2126
1464	233	465
1464	380	811
1464	486	1021
1464	517	368
1464	631	415
1464	755	396
1464	761	389
1464	1104	458
1464	1116	584
1464	1118	250

1464	1706	733
1464	1784	246
1464	1786	521
1464	1921	584
1464	2028	622
1464	2148	246
1464	2440	207
1464	2605	242
1464	2668	354
1464	2835	812
1464	3046	1963
1464	3870	419
1464	4306	1081
1464	4889	373
1464	5444	389
1464	8258	215
1464	10519	243
1464	13011	496
1464	13292	734
1464	14685	3332
1464	14686	3246
1464	14687	1068
1464	14688	750
1464	14689	719
1464	14690	699
1464	14691	557
1464	14692	511
1464	14693	309
1464	14694	255
1464	14695	249

1464 14696 213

1464 14697 202

1465 182 1724

1465 184 698

1465 288 795

1465 290 3341

1465 291 1547

1465 295 743

1465 297 717

1465 300 641

1465 302 598

1465 304 595

1465 310 782

1465 316 2277

1465 328 675

1465 355 4337

1465 377 1111

1465 378 652

1465 408 1181

1465 472 636

1465 497 1204

1465 538 2214

1465 540 711

1465 679 1070

1465 683 915

1465 701 999

1465 722 585

1465 808 927

1465 1021 754

1465 1186 584

1465	1378	541
1465	1470	3189
1465	1852	515
1465	1874	907
1465	2000	686
1465	2607	981
1465	2843	590
1465	3076	801
1465	3171	2806
1465	3373	1046
1465	3375	886
1465	3488	1441
1465	3859	625
1465	4487	650
1465	4616	512
1465	6614	1617
1465	6865	628
1465	8155	2440
1465	8589	527
1465	9234	655
1465	10069	1146
1465	14698	560
1466	55	324
1466	67	262
1466	89	366
1466	182	115
1466	257	217
1466	285	255
1466	288	620
1466	289	1310

1466	291 255
1466	292 394
1466	293 405
1466	294 184
1466	295 212
1466	296 289
1466	298 186
1466	299 510
1466	300 158
1466	302 169
1466	311 196
1466	314 128
1466	318 1213
1466	320 131
1466	321 423
1466	325 215
1466	326 176
1466	333 223
1466	342 144
1466	344 140
1466	349 321
1466	386 233
1466	456 136
1466	461 289
1466	465 266
1466	523 699
1466	525 214
1466	680 157
1466	681 228
1466	786 162

1466 903 374

1466 906 772

1466 908 373

1466 966 130

1466 1459 132

1466 1580 178

1466 1601 209

1466 1684 184

1466 2183 165

1466 2546 251

1466 3460 128

1466 4566 122

1467 173 502

1467 227 371

1467 289 311

1467 367 1780

1467 377 4412

1467 393 782

1467 399 231

1467 455 12258

1467 459 867

1467 462 1561

1467 468 301

1467 475 251

1467 479 702

1467 488 613

1467 491 570

1467 493 466

1467 497 253

1467 499 258

1467	510 808	
1467	830 2525	
1467	1133	395
1467	1247	230
1467	1681	690
1467	1859	1667
1467	2206	986
1467	2428	1042
1467	2923	367
1467	3097	280
1467	3568	247
1467	3576	605
1467	3928	2900
1467	4177	337
1467	7711	335
1467	8728	1650
1467	8991	679
1467	11796	1515
1467	11824	399
1467	12028	456
1467	12651	1404
1467	12698	490
1467	14699	3380
1467	14700	1057
1467	14701	688
1467	14702	584
1467	14703	487
1467	14704	456
1467	14705	438
1467	14706	428

1467	14707	296
1467	14708	253
1469	56	63
1469	65	261
1469	67	102
1469	88	42
1469	89	243
1469	154	81
1469	157	33
1469	163	109
1469	190	109
1469	191	20
1469	207	21
1469	209	116
1469	220	22
1469	227	108
1469	230	2284
1469	234	120
1469	300	81
1469	302	33
1469	306	24
1469	329	13
1469	377	110
1469	424	23
1469	425	35
1469	464	20
1469	466	22
1469	475	58
1469	486	14
1469	500	42

1469	511	34
1469	533	68
1469	538	19
1469	544	17
1469	687	37
1469	691	16
1469	706	401
1469	707	30
1469	730	20
1469	951	124
1469	982	13
1469	1042	28
1469	1044	23
1469	1186	34
1469	1243	49
1469	1249	136
1469	1400	53
1469	1412	14
1469	2346	60
1469	2834	42
1469	9856	12
1469	14722	16
1470	56	542
1470	81	1196
1470	154	6194
1470	196	1255
1470	197	884
1470	199	1286
1470	203	2505
1470	205	972

1470	211	1346
1470	227	1136
1470	231	2029
1470	232	1491
1470	237	1244
1470	418	2715
1470	423	584
1470	433	770
1470	435	597
1470	440	6544
1470	599	882
1470	612	590
1470	615	733
1470	618	571
1470	620	882
1470	622	2364
1470	709	1599
1470	1098	925
1470	1155	1070
1470	1426	664
1470	1732	1829
1470	1944	564
1470	1964	713
1470	1976	843
1470	2139	1813
1470	2268	567
1470	2535	551
1470	3469	838
1470	3686	1418
1470	3691	2000

1470	3693	2361
1470	6032	678
1470	6187	615
1470	6410	1435
1470	6417	1259
1470	7742	1019
1470	11589	850
1470	13053	1521
1470	13066	601
1470	13463	668
1470	14096	1001
1470	14723	579
1471	55	2814
1471	67	392
1471	157	125
1471	176	173
1471	191	109
1471	234	319
1471	256	144
1471	257	357
1471	283	349
1471	288	330
1471	289	43647
1471	292	1666
1471	295	275
1471	300	288
1471	302	85
1471	306	791
1471	316	122
1471	318	64

1471 328 69

1471 349 114

1471 355 65

1471 375 1184

1471 377 978

1471 461 189

1471 466 145

1471 468 66

1471 498 1095

1471 517 455

1471 523 111

1471 535 72

1471 538 79

1471 541 72

1471 542 73

1471 614 704

1471 646 77

1471 679 376

1471 686 432

1471 701 265

1471 802 91

1471 1044 451

1471 1246 235

1471 1369 504

1471 1453 160

1471 1854 526

1471 2014 219

1471 2094 3040

1471 3173 92

1471 3741 68

1471	6713	116
1471	14724	121
1472	40	84
1472	56	129
1472	72	593
1472	152	126
1472	154	147
1472	156	973
1472	172	139
1472	173	495
1472	190	747
1472	209	337
1472	220	238
1472	348	93
1472	377	193
1472	407	208
1472	518	974
1472	726	120
1472	768	253
1472	868	213
1472	930	291
1472	1118	177
1472	1151	110
1472	1339	92
1472	1375	102
1472	1793	440
1472	1854	155
1472	2014	153
1472	2524	137
1472	2745	89

1472	2775	743
1472	2835	159
1472	3197	217
1472	3425	143
1472	4045	232
1472	4136	287
1472	4155	147
1472	5459	129
1472	5972	108
1472	6847	102
1472	8503	170
1472	9978	177
1472	10181	86
1472	12394	111
1472	14725	481
1472	14726	481
1472	14727	280
1472	14728	276
1472	14729	151
1472	14730	148
1472	14731	130
1472	14732	91
1473	227 1026	
1473	321 51	
1473	324 89	
1473	328 264	
1473	374 56	
1473	423 134	
1473	464 598	
1473	466 80	

1473	470	1267
1473	498	71
1473	524	317
1473	527	65
1473	547	121
1473	687	102
1473	691	54
1473	779	186
1473	903	62
1473	1037	1813
1473	1162	169
1473	1402	84
1473	1455	92
1473	1481	197
1473	1486	137
1473	1609	274
1473	1678	336
1473	2132	392
1473	2841	448
1473	3293	48
1473	3317	242
1473	3338	182
1473	3708	828
1473	3712	349
1473	3716	180
1473	4706	943
1473	5067	630
1473	5305	540
1473	5308	119
1473	5947	151

1473	6448	96
1473	8155	211
1473	9384	157
1473	9684	263
1473	10964	219
1473	11213	66
1473	12477	967
1473	14368	96
1473	14733	300
1473	14734	232
1473	14735	90
1473	14736	63
1474	56	578
1474	57	219
1474	64	374
1474	70	358
1474	88	420
1474	107	324
1474	183	312
1474	196	358
1474	231	2222
1474	250	610
1474	445	336
1474	447	3156
1474	602	230
1474	606	345
1474	744	597
1474	748	2431
1474	1109	1815
1474	1126	199

1474	1745	473
1474	1965	447
1474	2030	207
1474	2149	264
1474	2174	316
1474	2605	256
1474	2616	378
1474	2676	213
1474	2691	1073
1474	2711	230
1474	3131	426
1474	3670	243
1474	3696	469
1474	5927	206
1474	7079	301
1474	8510	224
1474	9766	725
1474	10518	314
1474	11200	256
1474	13378	305
1474	13381	325
1474	13700	267
1474	14737	463
1474	14738	308
1474	14739	287
1474	14740	283
1474	14741	252
1474	14742	230
1474	14743	220
1474	14744	209

1474	14745	209
1474	14746	202
1475	77	21
1475	89	68
1475	157	1632
1475	234	44
1475	251	317
1475	257	22
1475	262	23
1475	285	16
1475	288	129
1475	292	34
1475	294	45
1475	295	1024
1475	300	31
1475	301	120
1475	302	67
1475	306	26
1475	310	16
1475	314	32
1475	328	16
1475	329	36
1475	330	76
1475	332	66
1475	333	36
1475	468	19
1475	484	23
1475	701	97
1475	913	282
1475	966	19

1475	1042	78
1475	1052	19
1475	1440	97
1475	1613	22
1475	1834	123
1475	1934	64
1475	1935	68
1475	1943	35
1475	2068	90
1475	3316	157
1475	3823	217
1475	4344	90
1475	5038	43
1475	5150	96
1475	5216	42
1475	5487	38
1475	5988	17
1475	7433	22
1475	14366	18
1475	14747	255
1475	14748	18
1475	14749	16
1476	89	2912
1476	157	2963
1476	161	493
1476	190	3751
1476	209	2977
1476	288	3972
1476	289	1959
1476	290	962

1476	291 1111
1476	294 922
1476	295 2116
1476	300 621
1476	321 1220
1476	324 636
1476	325 840
1476	327 540
1476	331 829
1476	333 473
1476	344 1696
1476	352 697
1476	355 1178
1476	377 1191
1476	405 599
1476	458 1116
1476	461 1561
1476	464 1244
1476	466 1354
1476	470 766
1476	481 4145
1476	486 9282
1476	493 614
1476	498 3863
1476	504 17809
1476	528 1016
1476	531 566
1476	544 1548
1476	681 2000
1476	685 559

1476	687 4867	
1476	1047	1460
1476	1379	2086
1476	1455	503
1476	1672	4256
1476	1679	472
1476	2020	701
1476	2051	554
1476	3317	601
1476	5678	2190
1476	6693	719
1476	10429	542
1477	2149	27
1477	2824	12
1477	4734	13
1477	5871	36
1477	7312	12
1477	7694	14
1477	9766	15
1477	11200	20
1477	11202	19
1477	11205	33
1477	12374	10
1477	14742	12
1477	14744	26
1477	14750	39
1477	14751	32
1477	14752	32
1477	14753	28
1477	14754	27

1477	14755	27
1477	14756	26
1477	14757	24
1477	14758	23
1477	14759	20
1477	14760	18
1477	14761	18
1477	14762	18
1477	14763	17
1477	14764	16
1477	14765	15
1477	14766	15
1477	14767	15
1477	14768	14
1477	14769	14
1477	14770	14
1477	14771	13
1477	14772	13
1477	14773	12
1477	14774	12
1477	14775	12
1477	14776	11
1477	14777	11
1477	14778	11
1477	14779	11
1477	14780	11
1477	14781	10
1477	14782	10
1477	14783	10
1477	14784	10

1477	14785	10
1477	14786	9
1478	55	1153
1478	97	587
1478	257	1354
1478	288	32080
1478	290	11531
1478	291	1510
1478	292	850
1478	294	1566
1478	298	1289
1478	300	985
1478	306	19705
1478	314	3255
1478	324	761
1478	325	4262
1478	332	466
1478	333	490
1478	352	2180
1478	386	2778
1478	423	1773
1478	461	369
1478	466	3578
1478	486	479
1478	498	370
1478	524	699
1478	526	1741
1478	528	7845
1478	532	2053
1478	544	813

1478	548	2590
1478	681	1814
1478	682	1094
1478	686	1095
1478	689	8531
1478	690	668
1478	691	404
1478	701	6211
1478	1032	2975
1478	1034	11214
1478	1037	2012
1478	1098	32942
1478	1449	486
1478	1458	469
1478	1659	558
1478	1772	4945
1478	2020	867
1478	2025	508
1478	3491	487
1478	6690	1160
1478	14787	1092
1478	14788	390
1479	45	239
1479	198	223
1479	217	144
1479	220	241
1479	412	660
1479	489	247
1479	517	130
1479	547	309

1479	754 119	
1479	757 105	
1479	779 111	
1479	805 347	
1479	814 142	
1479	816 102	
1479	823 133	
1479	826 142	
1479	831 164	
1479	1155	183
1479	1186	361
1479	1192	150
1479	1406	184
1479	1540	112
1479	1552	124
1479	2143	102
1479	2187	108
1479	2387	181
1479	2735	429
1479	3028	106
1479	4002	151
1479	4503	141
1479	4936	142
1479	4948	761
1479	5310	148
1479	6008	154
1479	7386	134
1479	8261	248
1479	8401	127
1479	8947	160

1479	9963	110
1479	10036	201
1479	11865	100
1479	14692	339
1479	14789	751
1479	14790	220
1479	14791	191
1479	14792	184
1479	14793	183
1479	14794	132
1479	14795	130
1479	14796	122
1480	56	653
1480	89	349
1480	154	260
1480	190	366
1480	191	310
1480	209	223
1480	298	452
1480	300	1884
1480	306	312
1480	324	1133
1480	333	724
1480	377	181
1480	429	451
1480	437	949
1480	536	178
1480	690	190
1480	907	253
1480	959	795

1480	972 220	
1480	1239	266
1480	1243	276
1480	1246	359
1480	1381	343
1480	1383	358
1480	1390	217
1480	1481	279
1480	1485	520
1480	1609	219
1480	1632	395
1480	1777	179
1480	1863	223
1480	2121	179
1480	2277	246
1480	2287	205
1480	2425	475
1480	2433	300
1480	2610	218
1480	2653	246
1480	3293	204
1480	3769	2225
1480	4241	227
1480	7276	412
1480	7818	232
1480	7868	206
1480	13714	344
1480	14645	361
1480	14797	372
1480	14798	300

1480	14799	206
1480	14800	189
1481	173	1187
1481	190	2003
1481	207	4153
1481	209	2032
1481	228	1008
1481	229	1456
1481	230	1525
1481	377	2393
1481	412	1436
1481	424	1060
1481	481	1154
1481	533	2233
1481	540	3251
1481	959	1066
1481	969	1145
1481	1048	1790
1481	1090	3671
1481	1104	1576
1481	1179	3149
1481	1186	4383
1481	1406	2918
1481	1515	3292
1481	1520	7157
1481	1650	2674
1481	1689	3902
1481	1988	4391
1481	2524	13459
1481	2580	1408

1481	2614	1252
1481	3400	3740
1481	3475	2050
1481	3797	1647
1481	4115	1116
1481	4155	3096
1481	4196	1513
1481	5440	1389
1481	6128	1333
1481	7354	2674
1481	7650	7689
1481	9233	5444
1481	9888	1007
1481	12168	4893
1481	13744	2038
1481	14801	4546
1481	14802	1828
1481	14803	1812
1481	14804	1576
1481	14805	1279
1481	14806	1054
1481	14807	954
1483	72	102
1483	89	394
1483	198	2085
1483	209	69
1483	230	83
1483	234	62
1483	289	376
1483	300	216

1483	333 42	
1483	377 56	
1483	378 80	
1483	464 68	
1483	466 219	
1483	486 139	
1483	498 145	
1483	517 220	
1483	524 240	
1483	537 83	
1483	538 61	
1483	646 72	
1483	714 63	
1483	930 1233	
1483	939 43	
1483	951 107	
1483	955 69	
1483	959 69	
1483	1034	231
1483	1041	315
1483	1044	445
1483	1047	185
1483	1104	81
1483	1114	119
1483	1180	171
1483	1188	145
1483	1246	52
1483	1249	90
1483	1456	127
1483	1513	238

1483	1670	57
1483	1672	1057
1483	1673	3456
1483	1681	61
1483	1807	175
1483	1853	128
1483	1974	114
1483	2081	56
1483	2653	70
1483	3929	53
1483	4850	225
1483	6253	158
1484	198	75
1484	377	198
1484	383	211
1484	805	302
1484	808	787
1484	840	625
1484	982	261
1484	1044	70
1484	1116	311
1484	1145	147
1484	1527	486
1484	1547	1395
1484	1873	1111
1484	2352	229
1484	2378	69
1484	3032	217
1484	3034	486
1484	3043	126

1484	3729	151
1484	4184	123
1484	4218	1097
1484	4346	1226
1484	4504	325
1484	4597	68
1484	5602	300
1484	5628	86
1484	5638	185
1484	7260	429
1484	8303	192
1484	8937	549
1484	8947	313
1484	9961	105
1484	10320	262
1484	10569	101
1484	11864	102
1484	14827	2690
1484	14828	896
1484	14829	479
1484	14830	474
1484	14831	447
1484	14832	336
1484	14833	248
1484	14834	242
1484	14835	230
1484	14836	214
1484	14837	159
1484	14838	92
1484	14839	89

1484	14840	86
1484	14841	76
1485	55	564
1485	56	214
1485	67	444
1485	72	211
1485	89	212
1485	97	318
1485	154	1454
1485	166	1116
1485	167	917
1485	173	316
1485	199	386
1485	207	861
1485	227	1322
1485	288	328
1485	289	444
1485	298	256
1485	306	335
1485	539	177
1485	618	3210
1485	679	1153
1485	689	660
1485	718	461
1485	859	209
1485	903	239
1485	1098	516
1485	1154	256
1485	1161	1100
1485	1400	463

1485	1556	407
1485	1663	184
1485	1929	498
1485	2137	332
1485	2277	1475
1485	2398	744
1485	2616	466
1485	3057	3281
1485	3310	173
1485	4709	449
1485	5416	245
1485	5441	669
1485	5790	560
1485	6297	1198
1485	9487	743
1485	11765	198
1485	12364	394
1485	14842	325
1485	14843	259
1485	14844	233
1485	14845	216
1485	14846	200
1486	65	146
1486	72	190
1486	85	75
1486	88	18
1486	152	71
1486	159	339
1486	203	267
1486	207	46

1486	220	39
1486	221	25
1486	230	80
1486	234	103
1486	329	21
1486	377	41
1486	424	54
1486	429	162
1486	432	23
1486	441	148
1486	493	32
1486	599	227
1486	709	53
1486	716	19
1486	851	109
1486	856	19
1486	868	150
1486	875	70
1486	969	86
1486	982	53
1486	1048	76
1486	1083	266
1486	1089	84
1486	1090	84
1486	1107	31
1486	1116	63
1486	1239	18
1486	1249	101
1486	1515	31
1486	1534	57

1486	2128	21
1486	2133	27
1486	2176	30
1486	2531	80
1486	2552	73
1486	2666	24
1486	3461	29
1486	3646	30
1486	4616	59
1486	5030	87
1486	6196	29
1486	14847	28
1488	163 6	
1488	188 44	
1488	198 20	
1488	517 5	
1488	841 9	
1488	843 22	
1488	846 25	
1488	854 7	
1488	917 25	
1488	918 71	
1488	920 31	
1488	944 40	
1488	947 11	
1488	950 10	
1488	956 14	
1488	1119	24
1488	1128	9
1488	1132	6

1488	1172	11
1488	1216	60
1488	1249	79
1488	1274	8
1488	1344	12
1488	1523	10
1488	1527	9
1488	1998	13
1488	2001	6
1488	2744	6
1488	5838	7
1488	6506	6
1488	7707	10
1488	7902	9
1488	13143	6
1488	13211	6
1488	14871	38
1488	14872	33
1488	14873	12
1488	14874	11
1488	14875	8
1488	14876	8
1488	14877	8
1488	14878	7
1488	14879	7
1488	14880	6
1488	14881	6
1488	14882	6
1488	14883	6
1488	14884	6

1488	14885	6
1488	14886	5
1489	55	354
1489	67	241
1489	190	203
1489	209	186
1489	265	303
1489	288	779
1489	289	634
1489	291	283
1489	292	11152
1489	294	262
1489	296	322
1489	299	305
1489	305	199
1489	310	257
1489	318	288
1489	326	357
1489	329	265
1489	349	188
1489	377	940
1489	378	465
1489	472	170
1489	486	3856
1489	488	1602
1489	491	182
1489	498	4357
1489	518	676
1489	536	292
1489	540	161

1489	670 200	
1489	701 182	
1489	786 243	
1489	907 724	
1489	909 425	
1489	1377	175
1489	1475	706
1489	1854	988
1489	1874	343
1489	2018	187
1489	2440	197
1489	2607	212
1489	3763	147
1489	3859	419
1489	3873	196
1489	4177	282
1489	5746	219
1489	6567	179
1489	7290	252
1489	12923	167
1489	14887	228
1489	14888	177
1490	198 641	
1490	371 874	
1490	377 928	
1490	497 643	
1490	500 1243	
1490	503 972	
1490	805 657	
1490	806 1129	

1490	808 1270	
1490	810 1251	
1490	811 2323	
1490	813 1102	
1490	815 865	
1490	823 715	
1490	834 1811	
1490	835 2508	
1490	850 807	
1490	917 915	
1490	923 1622	
1490	1338	861
1490	1347	1544
1490	1540	1405
1490	1672	8364
1490	2009	659
1490	2190	1966
1490	2379	3550
1490	2384	815
1490	2961	2218
1490	3416	662
1490	3543	638
1490	3628	3769
1490	3806	884
1490	4170	2266
1490	4171	1025
1490	5100	652
1490	7767	1364
1490	8471	797
1490	9511	668

1490	11852	3582
1490	12658	1606
1490	12776	914
1490	12780	1436
1490	14889	1984
1490	14890	1563
1490	14891	1372
1490	14892	1068
1490	14893	884
1490	14894	803
1490	14895	764
1490	14896	754
1491	53	196
1491	56	428
1491	65	187
1491	66	277
1491	89	612
1491	173	1291
1491	207	213
1491	210	309
1491	225	333
1491	228	247
1491	236	202
1491	289	233
1491	300	278
1491	333	255
1491	416	326
1491	432	520
1491	448	252
1491	524	651

1491	525 328	
1491	689 177	
1491	690 191	
1491	757 4096	
1491	1055	334
1491	1075	393
1491	1097	990
1491	1099	321
1491	1101	975
1491	1390	216
1491	1396	239
1491	1398	172
1491	1400	184
1491	1409	431
1491	1632	241
1491	1752	203
1491	1978	401
1491	1980	171
1491	1986	422
1491	2398	334
1491	2693	430
1491	2702	287
1491	2818	273
1491	2830	210
1491	3293	308
1491	3411	232
1491	4029	226
1491	5149	314
1491	6027	213
1491	6032	266

1491	7071	210
1491	14897	234
1492	65	47
1492	89	363
1492	190	83
1492	288	198
1492	289	70
1492	292	40
1492	300	103
1492	306	74
1492	362	79
1492	378	65
1492	461	50
1492	466	227
1492	475	34
1492	486	333
1492	498	437
1492	525	31
1492	689	34
1492	693	26
1492	697	105
1492	968	105
1492	1009	27
1492	1056	162
1492	1057	78
1492	1379	74
1492	1456	62
1492	2081	85
1492	2085	43
1492	2521	18

1492	3404	21
1492	5038	540
1492	5044	18
1492	5057	109
1492	5475	49
1492	5847	83
1492	8047	24
1492	8761	25
1492	8887	17
1492	10047	41
1492	10429	21
1492	10684	33
1492	10992	24
1492	14898	79
1492	14899	37
1492	14900	30
1492	14901	28
1492	14902	26
1492	14903	25
1492	14904	22
1492	14905	21
1492	14906	20
1493	12	145
1493	34	128
1493	89	221
1493	162	182
1493	188	257
1493	197	208
1493	883	133
1493	947	158

1493	985 280	
1493	1112	255
1493	1123	186
1493	1275	279
1493	1547	124
1493	1551	154
1493	1561	288
1493	1565	248
1493	2446	142
1493	2750	137
1493	2763	375
1493	3221	263
1493	3417	382
1493	3798	125
1493	4271	153
1493	4276	144
1493	4503	128
1493	4669	694
1493	4965	316
1493	5040	142
1493	5306	148
1493	5727	141
1493	6983	193
1493	9223	165
1493	9927	422
1493	10895	142
1493	11760	156
1493	11936	351
1493	14907	683
1493	14908	292

1493	14909	281
1493	14910	227
1493	14911	208
1493	14912	200
1493	14913	183
1493	14914	165
1493	14915	163
1493	14916	161
1493	14917	150
1493	14918	138
1493	14919	132
1493	14920	131
1494	65 9	
1494	155 19	
1494	157 29	
1494	159 9	
1494	195 4	
1494	207 21	
1494	209 18	
1494	221 4	
1494	227 15	
1494	229 16	
1494	298 4	
1494	329 5	
1494	333 17	
1494	349 4	
1494	403 3	
1494	424 11	
1494	525 7	
1494	545 6	

1494	641 4	
1494	716 6	
1494	907 16	
1494	975 5	
1494	1122	4
1494	1432	4
1494	1520	8
1494	2064	5
1494	2346	83
1494	2440	5
1494	3063	52
1494	5236	8
1494	5249	4
1494	7073	16
1494	7530	7
1494	7838	49
1494	7846	4
1494	8067	3
1494	8372	15
1494	8592	7
1494	10717	3
1494	13929	8
1494	14921	38
1494	14922	15
1494	14923	11
1494	14924	9
1494	14925	9
1494	14926	9
1494	14927	9
1494	14928	7

1494	14929	6
1494	14930	5
1495	162 27	
1495	632 18	
1495	769 10	
1495	1213	6
1495	1418	26
1495	1504	8
1495	1737	9
1495	2542	11
1495	2817	24
1495	2828	31
1495	3294	10
1495	3364	20
1495	3411	14
1495	3462	11
1495	3805	12
1495	3884	6
1495	5205	11
1495	5911	29
1495	6048	10
1495	6179	10
1495	6421	13
1495	7379	18
1495	8234	8
1495	8461	29
1495	8633	9
1495	8680	7
1495	10214	6
1495	10385	29

1495	10859	14
1495	10892	10
1495	11052	12
1495	11592	27
1495	11625	16
1495	11896	6
1495	12717	45
1495	13664	7
1495	13672	6
1495	14931	53
1495	14932	39
1495	14933	34
1495	14934	18
1495	14935	17
1495	14936	15
1495	14937	15
1495	14938	12
1495	14939	10
1495	14940	10
1495	14941	6
1495	14942	6
1495	14943	6
1496	53	458
1496	59	487
1496	72	8402
1496	81	75
1496	154	104
1496	155	110
1496	157	122
1496	199	159

1496	210	71
1496	220	361
1496	221	177
1496	229	795
1496	233	106
1496	238	451
1496	429	816
1496	436	70
1496	441	107
1496	443	63
1496	533	68
1496	703	247
1496	715	173
1496	716	65
1496	969	71
1496	1001	1813
1496	1014	79
1496	1072	176
1496	1073	58
1496	1075	111
1496	1089	181
1496	1094	156
1496	1107	147
1496	1116	166
1496	1151	218
1496	1155	159
1496	1384	102
1496	1390	66
1496	1713	260
1496	2136	125

1496	2531	211
1496	2542	196
1496	2691	99
1496	3250	387
1496	3676	102
1496	6728	167
1496	6852	60
1496	7324	126
1496	8656	302
1496	13834	60
1496	14944	67
1496	14945	63
1497	992 191	
1497	1112	266
1497	1125	191
1497	1411	643
1497	1833	680
1497	4675	1089
1497	4784	275
1497	4790	233
1497	5301	208
1497	7057	538
1497	7111	373
1497	7791	357
1497	7929	241
1497	8229	202
1497	8452	209
1497	11636	256
1497	14649	196
1497	14946	534

1497	14947	514
1497	14948	505
1497	14949	432
1497	14950	402
1497	14951	369
1497	14952	352
1497	14953	340
1497	14954	337
1497	14955	310
1497	14956	301
1497	14957	297
1497	14958	293
1497	14959	291
1497	14960	290
1497	14961	279
1497	14962	248
1497	14963	248
1497	14964	236
1497	14965	223
1497	14966	218
1497	14967	217
1497	14968	214
1497	14969	211
1497	14970	210
1497	14971	207
1497	14972	206
1497	14973	205
1497	14974	203
1497	14975	197
1497	14976	196

1497	14977	195
1497	14978	190
1498	51	525
1498	72	734
1498	159	2858
1498	166	453
1498	173	890
1498	185	306
1498	238	347
1498	389	656
1498	418	428
1498	429	1332
1498	441	467
1498	511	2178
1498	599	1762
1498	618	1570
1498	620	458
1498	715	1056
1498	867	1379
1498	871	748
1498	878	1321
1498	900	306
1498	1013	404
1498	1083	478
1498	1098	538
1498	1150	1310
1498	1707	984
1498	1739	640
1498	1884	362
1498	2469	520

1498	2531	1107
1498	3280	558
1498	3281	533
1498	3893	3032
1498	3920	675
1498	4144	455
1498	4609	1491
1498	6181	389
1498	7157	491
1498	7533	5003
1498	8831	1020
1498	9980	545
1498	11602	460
1498	12820	4988
1498	13888	411
1498	14979	1186
1498	14980	1096
1498	14981	681
1498	14982	546
1498	14983	528
1498	14984	528
1498	14985	318
1499	5367	538
1499	5391	15948
1499	11774	568
1499	14986	15345
1499	14987	14082
1499	14988	1253
1499	14989	1246
1499	14990	1232

1499	14991	1167
1499	14992	1143
1499	14993	1093
1499	14994	977
1499	14995	942
1499	14996	917
1499	14997	736
1499	14998	734
1499	14999	711
1499	15000	708
1499	15001	707
1499	15002	661
1499	15003	659
1499	15004	628
1499	15005	626
1499	15006	618
1499	15007	610
1499	15008	604
1499	15009	597
1499	15010	576
1499	15011	572
1499	15012	563
1499	15013	561
1499	15014	552
1499	15015	551
1499	15016	545
1499	15017	538
1499	15018	537
1499	15019	536
1499	15020	527

1499	15021	527
1499	15022	514
1499	15023	512
1499	15024	510
1499	15025	497
1499	15026	486
1499	15027	477
1499	15028	475
1499	15029	462
1499	15030	420
1499	15031	392
1499	15032	387
1500	9	142
1500	18	92
1500	32	149
1500	72	196
1500	156	530
1500	172	197
1500	175	78
1500	316	263
1500	389	183
1500	511	192
1500	683	87
1500	715	252
1500	728	149
1500	1121	139
1500	1123	270
1500	1295	2579
1500	1298	95
1500	1303	93

1500	1422	121
1500	1468	144
1500	1707	190
1500	1795	156
1500	2265	112
1500	2935	137
1500	3110	98
1500	3466	437
1500	3481	128
1500	3613	112
1500	6150	127
1500	6544	253
1500	6760	204
1500	7142	482
1500	7157	229
1500	9162	1303
1500	9433	403
1500	10310	103
1500	10902	125
1500	12668	185
1500	13882	94
1500	15033	626
1500	15034	622
1500	15035	431
1500	15036	315
1500	15037	313
1500	15038	146
1500	15039	137
1500	15040	101
1500	15041	97

1500	15042	86
1500	15043	86
1501	805 580	
1501	809 482	
1501	825 286	
1501	829 217	
1501	839 167	
1501	842 143	
1501	845 1032	
1501	849 152	
1501	1187	1165
1501	1552	301
1501	2186	220
1501	2187	160
1501	2192	151
1501	2382	218
1501	2429	666
1501	2579	883
1501	2586	222
1501	2643	318
1501	3028	1946
1501	3029	526
1501	3721	157
1501	3997	1546
1501	3999	861
1501	4003	169
1501	4004	196
1501	4096	477
1501	4937	172
1501	4938	184

1501	4942	288
1501	4944	247
1501	4948	378
1501	5968	531
1501	8277	1089
1501	8293	229
1501	10309	199
1501	13264	716
1501	13431	173
1501	15044	543
1501	15045	434
1501	15046	227
1501	15047	226
1501	15048	170
1501	15049	166
1501	15050	163
1501	15051	157
1501	15052	146
1501	15053	145
1501	15054	141
1501	15055	135
1501	15056	135
1502	57	394
1502	66	48
1502	72	105
1502	84	150
1502	88	130
1502	159	267
1502	172	27
1502	196	36

1502	234	25
1502	238	73
1502	298	25
1502	423	38
1502	703	58
1502	858	220
1502	961	23
1502	1099	30
1502	1155	33
1502	1707	24
1502	1970	48
1502	1986	189
1502	2572	90
1502	2605	60
1502	3502	24
1502	3877	66
1502	3902	49
1502	4113	61
1502	4153	35
1502	4316	24
1502	5074	54
1502	6042	25
1502	6583	166
1502	7324	50
1502	8230	34
1502	8582	25
1502	8689	31
1502	9860	26
1502	11754	60
1502	11826	38

1502	13882	49
1502	15057	92
1502	15058	66
1502	15059	65
1502	15060	53
1502	15061	41
1502	15062	34
1502	15063	31
1502	15064	29
1502	15065	28
1502	15066	26
1502	15067	24
1503	56	78
1503	65	152
1503	88	76
1503	89	449
1503	154	339
1503	155	210
1503	157	325
1503	190	260
1503	227	944
1503	229	64
1503	233	5978
1503	288	244
1503	292	92
1503	295	409
1503	306	137
1503	331	306
1503	468	90
1503	489	60

1503	510	197
1503	512	176
1503	517	149
1503	544	1022
1503	547	743
1503	599	171
1503	603	78
1503	707	71
1503	779	60
1503	969	129
1503	1044	402
1503	1118	75
1503	1145	72
1503	1416	135
1503	1434	138
1503	1613	155
1503	1974	87
1503	3317	148
1503	3795	102
1503	4316	259
1503	5073	68
1503	8915	145
1503	9931	165
1503	10389	128
1503	12897	142
1503	15068	481
1503	15069	103
1503	15070	89
1503	15071	81
1503	15072	78

1503	15073	70
1503	15074	68
1504	56	7933
1504	64	4635
1504	70	2775
1504	154	2400
1504	432	2276
1504	603	2325
1504	616	2126
1504	969	2194
1504	1099	3864
1504	1156	4377
1504	1394	6989
1504	1396	4828
1504	1397	2441
1504	1398	3564
1504	1399	3770
1504	1405	3035
1504	1409	2542
1504	1738	2242
1504	1824	3851
1504	1969	2529
1504	2101	2366
1504	2818	2812
1504	3200	2277
1504	3413	6284
1504	4029	2496
1504	6046	3390
1504	6585	2134
1504	8790	2324

1504	8868	2144
1504	12364	3167
1504	14846	2626
1504	15075	12520
1504	15076	3858
1504	15077	3681
1504	15078	3664
1504	15079	3532
1504	15080	3524
1504	15081	3074
1504	15082	2767
1504	15083	2670
1504	15084	2666
1504	15085	2619
1504	15086	2381
1504	15087	2376
1504	15088	2369
1504	15089	2345
1504	15090	2302
1504	15091	2294
1504	15092	2213
1504	15093	2118
1505	51	451
1505	53	271
1505	59	637
1505	63	911
1505	64	291
1505	65	629
1505	70	309
1505	72	13790

1505	75	591
1505	153	4964
1505	154	479
1505	155	398
1505	159	267
1505	173	313
1505	187	273
1505	193	1682
1505	233	456
1505	238	273
1505	377	752
1505	378	1390
1505	488	658
1505	489	292
1505	518	2603
1505	707	274
1505	878	337
1505	959	396
1505	999	292
1505	1001	814
1505	1014	1119
1505	1061	429
1505	1072	642
1505	1098	429
1505	1104	1016
1505	1379	282
1505	1713	1919
1505	1787	344
1505	1793	2206
1505	1854	390

1505	1892	288
1505	2027	315
1505	6524	1036
1505	10182	379
1505	11529	1290
1505	14760	866
1505	15094	1524
1505	15095	532
1505	15096	413
1505	15097	383
1505	15098	347
1505	15099	267
1506	81	195
1506	126	292
1506	154	2899
1506	173	505
1506	190	391
1506	195	347
1506	196	166
1506	199	1387
1506	203	253
1506	210	453
1506	222	312
1506	225	1559
1506	229	276
1506	236	340
1506	237	206
1506	425	1114
1506	429	518
1506	432	223

1506	440	267
1506	441	323
1506	445	201
1506	599	242
1506	606	412
1506	618	147
1506	851	1801
1506	858	609
1506	1075	492
1506	1089	1420
1506	1097	296
1506	1107	688
1506	1151	846
1506	1167	313
1506	1376	318
1506	1390	254
1506	1400	186
1506	1512	227
1506	1633	193
1506	2398	334
1506	2531	1041
1506	3109	151
1506	3707	528
1506	3767	147
1506	3771	1426
1506	5890	356
1506	5929	473
1506	6204	392
1506	8656	305
1506	9727	523

1506	14836	360
1506	15100	312
1507	88	92
1507	245	47
1507	267	11
1507	278	14
1507	475	22
1507	619	74
1507	1610	76
1507	1621	12
1507	1948	34
1507	1962	12
1507	1963	26
1507	1979	99
1507	2226	22
1507	2227	18
1507	2698	18
1507	2903	27
1507	2907	28
1507	2930	16
1507	3357	16
1507	4344	13
1507	4701	54
1507	5239	72
1507	5869	45
1507	6288	16
1507	6626	14
1507	6901	12
1507	12751	28
1507	13303	37

1507	14540	25
1507	15101	100
1507	15102	76
1507	15103	66
1507	15104	63
1507	15105	48
1507	15106	43
1507	15107	42
1507	15108	37
1507	15109	37
1507	15110	35
1507	15111	31
1507	15112	24
1507	15113	22
1507	15114	21
1507	15115	16
1507	15116	15
1507	15117	14
1507	15118	14
1507	15119	13
1507	15120	13
1507	15121	13
1508	7	533
1508	56	881
1508	65	408
1508	67	701
1508	88	676
1508	154	5182
1508	157	549
1508	163	372

1508	190 1673	
1508	199 708	
1508	203 668	
1508	206 1704	
1508	207 548	
1508	227 3515	
1508	229 2865	
1508	234 1232	
1508	298 270	
1508	333 182	
1508	424 1341	
1508	436 2178	
1508	439 3026	
1508	441 243	
1508	453 5223	
1508	511 191	
1508	533 452	
1508	538 214	
1508	544 245	
1508	599 675	
1508	614 202	
1508	714 276	
1508	715 584	
1508	733 741	
1508	854 197	
1508	903 420	
1508	1090	244
1508	1098	2921
1508	1239	248
1508	1244	812

1508	1400	450
1508	1414	601
1508	1755	271
1508	1766	348
1508	2130	192
1508	2184	230
1508	3071	236
1508	3112	242
1508	3116	726
1508	3200	206
1508	3739	360
1508	3889	222
1509	65	1881
1509	89	597
1509	190	742
1509	198	690
1509	207	850
1509	220	540
1509	229	1078
1509	230	1044
1509	234	535
1509	288	217
1509	289	325
1509	291	213
1509	295	437
1509	300	354
1509	306	492
1509	321	224
1509	328	337
1509	333	963

1509	377 709	
1509	378 267	
1509	461 219	
1509	481 227	
1509	498 1505	
1509	538 509	
1509	540 625	
1509	542 361	
1509	903 246	
1509	966 266	
1509	982 468	
1509	1045	200
1509	1116	466
1509	1241	377
1509	1246	418
1509	1377	423
1509	1406	319
1509	1673	318
1509	1681	218
1509	2080	725
1509	2835	302
1509	3378	1001
1509	3859	258
1509	4463	374
1509	4471	359
1509	4474	422
1509	4484	225
1509	4828	504
1509	4850	208
1509	5226	268

1509	5594	399
1509	6480	214
1511	67	134
1511	89	412
1511	190	152
1511	257	68
1511	288	570
1511	289	1734
1511	291	254
1511	292	6926
1511	295	279
1511	298	235
1511	299	57
1511	300	4571
1511	302	702
1511	304	112
1511	306	105
1511	333	1303
1511	340	91
1511	344	680
1511	349	1269
1511	378	499
1511	386	362
1511	456	369
1511	461	165
1511	464	707
1511	466	634
1511	475	89
1511	486	100
1511	498	4346

1511	523 148	
1511	528 570	
1511	538 64	
1511	540 213	
1511	542 1241	
1511	543 996	
1511	544 57	
1511	646 224	
1511	679 875	
1511	714 59	
1511	797 72	
1511	802 359	
1511	1032	119
1511	1037	153
1511	1246	125
1511	1452	77
1511	1459	1736
1511	2392	86
1511	2977	205
1511	3179	60
1511	4292	95
1511	11252	281
1513	72 205	
1513	159 388	
1513	173 489	
1513	190 241	
1513	198 310	
1513	229 1304	
1513	230 658	
1513	306 167	

1513	420	2578
1513	425	327
1513	437	656
1513	441	292
1513	460	568
1513	727	341
1513	851	2689
1513	1090	523
1513	1109	309
1513	1231	309
1513	1380	161
1513	1398	258
1513	1406	994
1513	1513	707
1513	1786	625
1513	1789	258
1513	2478	248
1513	2524	544
1513	2738	377
1513	3046	289
1513	3057	1552
1513	3300	384
1513	3870	855
1513	4047	395
1513	4609	183
1513	4802	570
1513	5922	2090
1513	5930	255
1513	10491	170
1513	12710	847

1513	14049	1500
1513	15135	833
1513	15136	502
1513	15137	363
1513	15138	338
1513	15139	310
1513	15140	300
1513	15141	271
1513	15142	224
1513	15143	178
1514	51	275
1514	54	309
1514	58	232
1514	59	666
1514	63	657
1514	67	172
1514	70	180
1514	72	1251
1514	75	300
1514	76	230
1514	77	167
1514	93	182
1514	153	392
1514	154	205
1514	163	259
1514	172	139
1514	175	234
1514	187	318
1514	193	276
1514	233	253

1514	238	248
1514	603	138
1514	851	169
1514	862	257
1514	877	170
1514	880	312
1514	900	147
1514	993	325
1514	997	211
1514	1001	478
1514	1014	259
1514	1061	271
1514	1075	260
1514	1156	163
1514	1836	290
1514	1886	225
1514	1892	196
1514	1900	249
1514	2469	375
1514	3109	232
1514	3289	139
1514	3413	161
1514	3768	176
1514	3988	224
1514	4113	148
1514	4126	327
1514	4311	174
1514	5073	155
1514	5503	266
1514	8069	207

1515	89	184
1515	155	124
1515	157	401
1515	251	389
1515	257	1352
1515	265	142
1515	288	265
1515	290	196
1515	291	203
1515	294	824
1515	295	396
1515	297	222
1515	304	813
1515	327	156
1515	344	323
1515	458	428
1515	479	663
1515	543	149
1515	679	473
1515	906	130
1515	915	571
1515	1602	819
1515	1936	246
1515	2939	1039
1515	2977	279
1515	3319	292
1515	3387	222
1515	4350	127
1515	4474	131
1515	4491	2913

1515	4869	203
1515	5425	154
1515	5535	563
1515	5752	421
1515	6713	252
1515	12958	192
1515	12963	142
1515	13741	157
1515	14573	177
1515	15144	396
1515	15145	377
1515	15146	348
1515	15147	324
1515	15148	322
1515	15149	314
1515	15150	278
1515	15151	202
1515	15152	190
1515	15153	136
1515	15154	133
1516	67	98
1516	89	789
1516	155	49
1516	157	32
1516	176	50
1516	184	37
1516	220	223
1516	228	32
1516	253	49
1516	288	396

1516	289	1177
1516	293	36
1516	300	414
1516	306	32
1516	316	34
1516	349	50
1516	366	205
1516	465	37
1516	486	33
1516	511	56
1516	533	88
1516	679	89
1516	701	94
1516	907	34
1516	951	67
1516	1241	42
1516	1243	593
1516	2018	42
1516	2031	143
1516	2988	36
1516	3074	397
1516	3173	56
1516	3382	43
1516	3462	53
1516	3741	38
1516	3934	34
1516	3981	33
1516	5772	51
1516	6123	785
1516	6721	32

1516	6723	81
1516	7452	36
1516	8371	61
1516	8810	79
1516	11070	37
1516	11474	250
1516	15155	119
1516	15156	33
1516	15157	32
1516	15158	31
1517	55	745
1517	182	1272
1517	228	743
1517	239	3736
1517	257	4804
1517	279	752
1517	288	2871
1517	290	1147
1517	291	2902
1517	292	1616
1517	293	1818
1517	294	2256
1517	295	1178
1517	298	2322
1517	299	783
1517	300	744
1517	301	6551
1517	302	2312
1517	305	1376
1517	310	1075

1517	311 922	
1517	318 1097	
1517	320 786	
1517	326 1321	
1517	329 2292	
1517	331 1019	
1517	332 1297	
1517	333 3533	
1517	344 1068	
1517	355 1318	
1517	359 1032	
1517	468 1192	
1517	485 1620	
1517	538 1084	
1517	548 906	
1517	683 1321	
1517	903 2321	
1517	1048	1119
1517	1377	877
1517	1459	1448
1517	1556	5290
1517	1934	1520
1517	2082	1611
1517	2546	896
1517	2607	1014
1517	3488	789
1517	4619	1182
1517	8067	1104
1517	8155	1065
1517	12406	841

1518	59	584
1518	154	520
1518	159	853
1518	195	1041
1518	212	1038
1518	215	1743
1518	222	350
1518	416	789
1518	429	987
1518	511	703
1518	599	1154
1518	609	2350
1518	615	2728
1518	618	621
1518	620	434
1518	715	747
1518	851	3014
1518	857	481
1518	859	498
1518	863	986
1518	867	520
1518	961	506
1518	985	463
1518	1089	1195
1518	1094	1493
1518	1167	364
1518	1213	535
1518	1633	399
1518	1947	933
1518	1957	486

1518	2265	361
1518	2531	963
1518	2606	556
1518	2629	1143
1518	2633	513
1518	3280	828
1518	3884	812
1518	4079	732
1518	4366	1033
1518	4541	1055
1518	6182	464
1518	6728	1246
1518	8672	352
1518	11598	910
1518	14523	756
1518	15100	404
1518	15130	367
1518	15159	651
1518	15160	490
1518	15161	339
1519	65	115
1519	154	104
1519	182	115
1519	190	521
1519	207	227
1519	209	82
1519	229	123
1519	288	57
1519	289	55
1519	323	83

1519	333	88
1519	344	160
1519	377	138
1519	397	57
1519	424	157
1519	466	57
1519	470	87
1519	486	59
1519	488	68
1519	511	264
1519	533	239
1519	544	77
1519	683	56
1519	687	79
1519	693	95
1519	704	122
1519	779	71
1519	816	64
1519	951	145
1519	966	59
1519	972	156
1519	1045	88
1519	1047	1085
1519	1048	60
1519	1185	73
1519	1191	164
1519	1384	62
1519	1470	162
1519	1673	73
1519	1854	88

1519	1910	91
1519	1923	376
1519	2386	99
1519	2608	76
1519	2959	72
1519	3367	72
1519	5001	57
1519	5950	68
1519	8155	86
1519	8398	57
1520	58	203
1520	65	525
1520	89	3681
1520	154	228
1520	166	119
1520	173	185
1520	190	313
1520	203	1266
1520	207	1570
1520	209	229
1520	227	534
1520	229	117
1520	230	2488
1520	298	595
1520	374	122
1520	379	218
1520	418	268
1520	425	139
1520	444	107
1520	532	402

1520	533 597	
1520	689 711	
1520	720 177	
1520	903 519	
1520	961 1911	
1520	1090	115
1520	1095	272
1520	1098	909
1520	1384	115
1520	1400	187
1520	1512	146
1520	1513	129
1520	1814	168
1520	2128	125
1520	2815	130
1520	3456	239
1520	3945	153
1520	4224	233
1520	4980	1670
1520	5615	284
1520	9569	120
1520	11492	251
1520	11822	227
1520	13529	106
1520	14337	116
1520	14459	325
1520	15162	296
1520	15163	206
1520	15164	137
1520	15165	134

1521	161 527
1521	191 358
1521	288 2976
1521	289 1441
1521	291 709
1521	292 576
1521	293 417
1521	295 740
1521	299 1328
1521	300 2895
1521	304 361
1521	306 583
1521	311 426
1521	321 544
1521	333 10128
1521	344 1357
1521	352 1001
1521	458 380
1521	461 809
1521	466 1286
1521	470 1389
1521	498 1057
1521	524 516
1521	528 1078
1521	544 1617
1521	669 1208
1521	679 2096
1521	680 451
1521	681 608
1521	691 391

1521	701	1339
1521	704	1098
1521	966	650
1521	1032	886
1521	1042	903
1521	1377	480
1521	1447	606
1521	1456	737
1521	1458	373
1521	1470	2423
1521	1673	540
1521	1680	572
1521	2020	631
1521	3078	362
1521	3871	361
1521	4814	3303
1521	10808	423
1521	15166	613
1521	15167	606
1521	15168	347
1522	7	42
1522	45	43
1522	56	51
1522	65	265
1522	88	65
1522	163	1546
1522	190	1498
1522	207	72
1522	220	251
1522	227	3753

1522	229	161
1522	230	3210
1522	234	40
1522	436	62
1522	440	3062
1522	493	69
1522	497	43
1522	733	49
1522	808	42
1522	951	51
1522	959	48
1522	1043	44
1522	1090	74
1522	1099	44
1522	1106	42
1522	1155	72
1522	1198	52
1522	1246	53
1522	1249	107
1522	1377	73
1522	1398	86
1522	1400	19619
1522	1412	44
1522	1738	54
1522	1954	198
1522	2343	43
1522	2600	46
1522	3686	150
1522	3738	157
1522	4028	53

1522	4029	62
1522	4387	271
1522	6303	140
1522	7324	168
1522	8678	1167
1522	15169	273
1522	15170	99
1522	15171	84
1522	15172	63
1522	15173	54
1524	40	654
1524	67	850
1524	89	444
1524	154	503
1524	163	970
1524	198	340
1524	199	351
1524	200	588
1524	220	1645
1524	222	388
1524	226	394
1524	227	2453
1524	233	1011
1524	412	538
1524	424	913
1524	511	327
1524	534	641
1524	562	432
1524	603	322
1524	757	345

1524	857 327	
1524	886 352	
1524	1090	420
1524	1091	2359
1524	1105	539
1524	1246	447
1524	1412	420
1524	1632	656
1524	2271	659
1524	2569	352
1524	2628	411
1524	2637	432
1524	3200	368
1524	3340	545
1524	3737	402
1524	4196	587
1524	4198	357
1524	4208	3463
1524	4833	1189
1524	7188	430
1524	8270	726
1524	8275	773
1524	9485	2269
1524	10564	728
1524	13221	348
1524	14203	735
1524	15179	628
1524	15180	532
1524	15181	483
1524	15182	333

1525	89	53
1525	191	26
1525	288	63
1525	289	74
1525	291	60
1525	292	64
1525	293	65
1525	295	32
1525	299	20
1525	300	54
1525	310	39
1525	311	27
1525	321	61
1525	325	116
1525	326	17
1525	333	55
1525	342	72
1525	344	59
1525	346	51
1525	352	22
1525	357	18
1525	386	58
1525	458	90
1525	461	122
1525	466	75
1525	502	50
1525	544	19
1525	681	111
1525	686	84
1525	692	42

1525	701	55
1525	793	112
1525	797	83
1525	908	37
1525	1054	38
1525	1453	47
1525	1458	54
1525	1683	28
1525	1684	52
1525	1698	40
1525	4767	26
1525	4879	33
1525	7449	28
1525	8960	27
1525	10317	27
1525	13720	55
1525	15183	49
1525	15184	38
1525	15185	28
1525	15186	21
1527	89	118
1527	190	3241
1527	196	288
1527	207	317
1527	223	127
1527	228	1106
1527	229	132
1527	342	98
1527	371	205
1527	419	1053

1527	432 334	
1527	497 166	
1527	533 90	
1527	704 93	
1527	1048	1488
1527	1182	174
1527	1194	104
1527	1198	181
1527	1377	99
1527	1400	1044
1527	1517	449
1527	1852	92
1527	1988	1150
1527	2137	1031
1527	2614	1097
1527	2652	390
1527	2661	258
1527	2666	125
1527	2815	104
1527	3247	394
1527	3797	151
1527	3859	70
1527	3927	290
1527	4436	79
1527	4747	196
1527	5029	105
1527	5439	85
1527	5950	70
1527	6223	215
1527	6608	151

1527	7663	123
1527	10327	126
1527	11796	135
1527	12918	657
1527	14352	120
1527	15191	155
1527	15192	126
1527	15193	124
1527	15194	117
1527	15195	99
1528	55	483
1528	67	104
1528	89	407
1528	161	119
1528	207	42
1528	288	726
1528	289	1808
1528	292	2406
1528	295	1027
1528	299	166
1528	300	505
1528	306	279
1528	314	1489
1528	317	240
1528	318	54
1528	327	87
1528	332	114
1528	333	137
1528	349	1100
1528	461	150

1528	466 933	
1528	468 199	
1528	485 53	
1528	524 137	
1528	528 145	
1528	531 41	
1528	542 86	
1528	543 2892	
1528	544 182	
1528	646 71	
1528	686 113	
1528	701 792	
1528	790 91	
1528	797 160	
1528	802 380	
1528	903 62	
1528	1032	73
1528	1034	122
1528	1243	124
1528	1429	46
1528	1451	197
1528	1452	169
1528	1456	91
1528	2094	52
1528	2521	239
1528	2543	128
1528	2548	1006
1528	2990	42
1528	11252	172
1528	15196	58

1530	65	304
1530	88	887
1530	163	3637
1530	176	266
1530	193	331
1530	198	2321
1530	220	1970
1530	245	351
1530	383	476
1530	412	265
1530	424	397
1530	429	661
1530	436	436
1530	511	386
1530	517	389
1530	706	358
1530	707	906
1530	720	321
1530	732	294
1530	857	1784
1530	982	1491
1530	1044	1072
1530	1103	259
1530	1104	842
1530	1206	3650
1530	1249	1420
1530	1360	265
1530	1369	576
1530	1406	750
1530	1412	283

1530	1513	1903
1530	1520	321
1530	1545	1821
1530	2343	353
1530	2346	966
1530	2595	407
1530	2738	303
1530	3360	267
1530	3382	291
1530	3444	601
1530	4087	555
1530	4155	373
1530	4800	754
1530	4805	850
1530	5515	252
1530	7324	398
1530	7333	652
1530	10403	468
1530	10793	343
1530	14657	251
1531	28	312
1531	159	541
1531	841	245
1531	918	272
1531	954	489
1531	1206	699
1531	1358	354
1531	1369	240
1531	1513	395
1531	1521	566

1531	1522	335
1531	1804	1119
1531	2853	534
1531	3011	238
1531	3354	238
1531	3417	280
1531	3435	267
1531	3445	384
1531	3453	418
1531	3509	297
1531	3540	339
1531	3790	287
1531	3792	302
1531	4265	331
1531	4272	654
1531	5421	435
1531	9212	376
1531	9213	285
1531	9215	869
1531	9872	390
1531	10370	851
1531	10672	250
1531	10742	272
1531	10768	723
1531	13971	256
1531	15198	580
1531	15199	491
1531	15200	480
1531	15201	450
1531	15202	399

1531	15203	392
1531	15204	387
1531	15205	377
1531	15206	342
1531	15207	337
1531	15208	292
1531	15209	278
1531	15210	267
1531	15211	243
1531	15212	242
1532	55	5034
1532	67	9630
1532	89	525
1532	97	232
1532	155	951
1532	190	623
1532	257	481
1532	288	2301
1532	289	1870
1532	292	1885
1532	294	826
1532	295	10130
1532	298	230
1532	300	1378
1532	301	500
1532	302	664
1532	310	629
1532	311	220
1532	329	850
1532	332	644

1532	333	375
1532	340	255
1532	349	609
1532	425	924
1532	523	1417
1532	525	979
1532	534	429
1532	535	508
1532	542	1182
1532	680	296
1532	701	2303
1532	903	1440
1532	913	688
1532	972	643
1532	1052	414
1532	1098	427
1532	1463	286
1532	1556	1470
1532	1580	719
1532	1601	1251
1532	1694	2383
1532	1833	426
1532	1934	293
1532	1935	417
1532	2521	2094
1532	3057	249
1532	3173	245
1532	3200	353
1532	5934	239
1532	6313	296

1533	55	5431
1533	67	2244
1533	69	3666
1533	88	1025
1533	89	2819
1533	97	2295
1533	239	7144
1533	251	4122
1533	257	25140
1533	262	4473
1533	265	4989
1533	287	3300
1533	288	6613
1533	289	1946
1533	291	1261
1533	292	2153
1533	294	1908
1533	295	1740
1533	300	4068
1533	301	10992
1533	332	2967
1533	342	2154
1533	344	2606
1533	352	1046
1533	360	4158
1533	364	1561
1533	468	3295
1533	538	2380
1533	575	1630
1533	683	1638

1533	691	1148
1533	892	1405
1533	1459	5546
1533	1772	1827
1533	1828	2405
1533	1934	4833
1533	1936	5801
1533	2176	2266
1533	2523	1331
1533	3488	3313
1533	4696	1768
1533	5150	1684
1533	5767	1245
1533	8991	1703
1533	10989	3416
1533	11076	1303
1533	12406	1991
1533	15213	3275
1533	15214	1389
1533	15215	1025
1534	45	1786
1534	58	308
1534	65	1153
1534	77	314
1534	96	891
1534	152	3106
1534	163	4296
1534	173	1716
1534	174	995
1534	189	246

1534	204 340	
1534	217 1370	
1534	245 473	
1534	377 644	
1534	429 873	
1534	445 479	
1534	486 506	
1534	487 665	
1534	492 474	
1534	562 261	
1534	607 330	
1534	689 522	
1534	757 1031	
1534	768 3044	
1534	917 548	
1534	1075	649
1534	1099	434
1534	1104	273
1534	1180	523
1534	1184	340
1534	1191	276
1534	1375	238
1534	1382	255
1534	1410	339
1534	1541	802
1534	1705	299
1534	2369	334
1534	2850	1342
1534	2855	253
1534	3030	283

1534	3411	374
1534	3616	511
1534	3621	1831
1534	4781	502
1534	6008	363
1534	7152	1261
1534	9716	433
1534	12288	397
1534	15216	739
1534	15217	540
1535	89	1978
1535	190	1217
1535	191	605
1535	209	1018
1535	227	1425
1535	230	1078
1535	291	451
1535	324	768
1535	333	3727
1535	344	1031
1535	412	664
1535	430	1109
1535	461	985
1535	464	426
1535	471	636
1535	481	548
1535	498	1049
1535	530	518
1535	532	551
1535	537	2243

1535	540 1341	
1535	686 666	
1535	687 560	
1535	691 1266	
1535	1043	1592
1535	1047	1702
1535	1189	414
1535	1242	397
1535	1390	759
1535	1590	1227
1535	1866	534
1535	2852	399
1535	3079	1306
1535	3081	585
1535	3112	542
1535	3273	588
1535	3373	839
1535	4385	512
1535	4559	1176
1535	4810	842
1535	5785	479
1535	5950	529
1535	6397	675
1535	7777	732
1535	10318	499
1535	10327	400
1535	15218	7919
1535	15219	873
1535	15220	627
1535	15221	475

1536	12	45
1536	15	53
1536	72	292
1536	190	22
1536	198	324
1536	220	18
1536	517	102
1536	706	51
1536	707	45
1536	824	19
1536	841	1008
1536	917	10011
1536	918	1153
1536	922	498
1536	947	103
1536	949	359
1536	954	74
1536	959	33
1536	1044	291
1536	1104	277
1536	1119	37
1536	1249	65
1536	1257	103
1536	1261	380
1536	1339	32
1536	1347	128
1536	1350	19
1536	1358	334
1536	1360	347
1536	1372	20

1536	1521	30
1536	1527	105
1536	1639	57
1536	1804	37
1536	1810	21
1536	2342	20
1536	2346	23
1536	2347	164
1536	2493	25
1536	2835	20
1536	3624	33
1536	4271	116
1536	4680	19
1536	5076	45
1536	5157	20
1536	6273	56
1536	7386	52
1536	10686	42
1536	15222	104
1536	15223	29
1537	88	139
1537	89	26
1537	184	40
1537	198	46
1537	234	232
1537	291	35
1537	293	159
1537	300	114
1537	302	65
1537	303	58

1537	304	42
1537	366	196
1537	412	71
1537	429	72
1537	464	87
1537	466	103
1537	498	27
1537	501	81
1537	538	44
1537	679	124
1537	680	32
1537	705	129
1537	1043	49
1537	1047	40
1537	1400	58
1537	1545	45
1537	1775	36
1537	2839	131
1537	2841	30
1537	2988	208
1537	3378	1343
1537	3475	37
1537	4465	24
1537	4471	416
1537	4709	27
1537	4818	97
1537	4828	1845
1537	5119	224
1537	5249	36
1537	5425	49

1537	5441	46
1537	6308	33
1537	6453	55
1537	7084	24
1537	9228	33
1537	12072	82
1537	15224	211
1537	15225	77
1537	15226	70
1537	15227	25
1538	65	38
1538	154	109
1538	159	13
1538	163	17
1538	166	36
1538	172	18
1538	173	107
1538	190	37
1538	196	22
1538	198	24
1538	203	15
1538	206	16
1538	215	20
1538	221	20
1538	227	64
1538	228	17
1538	229	25
1538	412	24
1538	424	15
1538	450	24

1538	486	14
1538	506	46
1538	533	16
1538	602	15
1538	607	12
1538	851	16
1538	858	15
1538	903	32
1538	920	16
1538	955	16
1538	969	29
1538	1098	56
1538	1109	63
1538	1118	40
1538	1151	16
1538	1372	31
1538	1512	25
1538	1778	41
1538	1947	17
1538	2006	17
1538	2277	22
1538	2304	22
1538	2323	17
1538	2775	26
1538	2836	19
1538	10381	30
1538	14632	14
1538	15228	18
1538	15229	16
1538	15230	13

1539	56	18
1539	65	29
1539	88	32
1539	182	22
1539	190	26
1539	207	69
1539	225	17
1539	227	23
1539	229	43
1539	298	22
1539	420	22
1539	424	190
1539	425	40
1539	437	31
1539	498	75
1539	499	22
1539	704	50
1539	709	40
1539	716	25
1539	868	16
1539	959	16
1539	985	24
1539	989	22
1539	1090	47
1539	1240	25
1539	1390	91
1539	1400	44
1539	1486	19
1539	1515	24
1539	1807	37

1539	1988	27
1539	2133	14
1539	2343	15
1539	2533	17
1539	2821	64
1539	3200	24
1539	3333	44
1539	3399	16
1539	3475	87
1539	3767	16
1539	4149	93
1539	4291	17
1539	5030	21
1539	6529	32
1539	13845	15
1539	15231	36
1539	15232	27
1539	15233	26
1539	15234	15
1539	15235	14
1540	55	749
1540	88	361
1540	89	10733
1540	209	625
1540	288	467
1540	289	1224
1540	298	751
1540	300	919
1540	302	1610
1540	306	1141

1540	320 296	
1540	324 631	
1540	333 289	
1540	400 575	
1540	518 790	
1540	532 431	
1540	535 1642	
1540	540 321	
1540	701 3139	
1540	787 526	
1540	790 428	
1540	792 569	
1540	798 692	
1540	886 897	
1540	906 756	
1540	970 1864	
1540	1392	348
1540	1406	335
1540	1410	812
1540	1432	309
1540	1904	387
1540	2018	779
1540	2091	737
1540	2440	745
1540	2521	961
1540	2944	280
1540	3193	1200
1540	3769	660
1540	4024	445
1540	4260	355

1540	4817	1440
1540	6054	352
1540	7268	354
1540	7506	1087
1540	8136	310
1540	11866	441
1540	11995	295
1540	12921	280
1540	15236	395
1540	15237	272
1541	32	29
1541	40	27
1541	51	148
1541	58	100
1541	59	44
1541	67	48
1541	72	56
1541	227	72
1541	440	26
1541	511	33
1541	526	30
1541	532	266
1541	627	43
1541	993	59
1541	997	31
1541	998	88
1541	1001	410
1541	1014	183
1541	1019	73
1541	1028	130

1541	1062	94
1541	1090	68
1541	1738	28
1541	1886	31
1541	1890	25
1541	2531	87
1541	2535	34
1541	2938	46
1541	3183	67
1541	3186	40
1541	3768	68
1541	4308	83
1541	4732	46
1541	6203	38
1541	7096	34
1541	7347	37
1541	7649	123
1541	7885	27
1541	8583	32
1541	8585	56
1541	9869	57
1541	11331	225
1541	11335	163
1541	14639	28
1541	15238	83
1541	15239	60
1541	15240	44
1541	15241	37
1541	15242	33
1541	15243	30

1542	56	404
1542	65	184
1542	67	449
1542	72	140
1542	96	349
1542	154	486
1542	157	459
1542	163	328
1542	167	119
1542	182	308
1542	187	2197
1542	193	451
1542	206	124
1542	222	160
1542	227	415
1542	234	123
1542	389	274
1542	599	1666
1542	604	390
1542	683	464
1542	691	370
1542	994	132
1542	997	378
1542	1003	186
1542	1048	162
1542	1090	309
1542	1122	330
1542	1414	392
1542	1464	294
1542	1643	161

1542 1820 243
1542 1899 377
1542 2031 315
1542 2439 152
1542 2554 140
1542 3266 153
1542 3302 120
1542 3481 139
1542 4126 711
1542 4620 336
1542 5452 152
1542 6733 258
1542 11477 173
1542 14632 179
1542 15244 384
1542 15245 317
1542 15246 291
1542 15247 257
1542 15248 173
1542 15249 145
1543 174 53
1543 439 5
1543 714 54
1543 731 31
1543 917 15
1543 920 1
1543 928 44
1543 936 201
1543 946 4
1543 1285 29

1543	1343	87
1543	1350	2
1543	1412	23
1543	4587	13
1543	4600	23
1543	5073	33
1543	12006	20
1543	15250	602
1543	15251	337
1543	15252	282
1543	15253	266
1543	15254	260
1543	15255	34
1543	15256	15
1543	15257	12
1543	15258	7
1543	15259	5
1544	67	127
1544	89	230
1544	172	312
1544	183	126
1544	190	223
1544	246	313
1544	289	498
1544	291	131
1544	292	316
1544	293	261
1544	316	131
1544	333	214
1544	377	142

1544	378	518
1544	405	179
1544	464	140
1544	466	121
1544	487	518
1544	488	176
1544	490	279
1544	494	173
1544	499	254
1544	524	122
1544	680	118
1544	816	516
1544	903	121
1544	972	200
1544	1045	330
1544	1104	140
1544	1179	272
1544	1188	206
1544	1190	136
1544	1195	293
1544	1672	114
1544	1775	148
1544	2014	286
1544	2184	148
1544	2432	192
1544	2608	189
1544	2661	272
1544	2956	120
1544	3074	132
1544	3124	244

1544	3367	331
1544	5079	306
1544	5633	116
1544	5759	144
1544	6812	200
1544	7356	116
1544	8847	248
1545	59	319
1545	65	832
1545	154	777
1545	159	679
1545	166	403
1545	187	314
1545	199	211
1545	200	240
1545	225	382
1545	227	258
1545	229	686
1545	234	184
1545	316	280
1545	383	172
1545	389	176
1545	424	297
1545	425	287
1545	429	201
1545	439	783
1545	441	627
1545	450	385
1545	604	168
1545	705	238

1545	706	229
1545	714	205
1545	716	186
1545	840	203
1545	851	276
1545	859	184
1545	903	489
1545	932	310
1545	969	191
1545	1109	622
1545	1154	277
1545	1231	713
1545	1512	218
1545	1513	263
1545	2600	385
1545	2616	270
1545	2938	249
1545	3213	188
1545	4116	170
1545	5079	467
1545	5306	705
1545	5421	225
1545	6137	620
1545	7260	2053
1545	7638	335
1545	15260	230
1545	15261	184
1546	65	569
1546	67	371
1546	72	843

1546	88	188
1546	89	180
1546	154	837
1546	159	481
1546	173	1894
1546	190	1302
1546	203	446
1546	207	207
1546	210	311
1546	217	1460
1546	223	169
1546	225	1188
1546	226	774
1546	229	319
1546	295	235
1546	298	706
1546	420	319
1546	424	364
1546	425	234
1546	429	403
1546	436	342
1546	439	213
1546	510	243
1546	537	440
1546	547	318
1546	680	191
1546	689	250
1546	716	469
1546	840	351
1546	903	171

1546	964	188
1546	972	279
1546	982	187
1546	989	157
1546	1090	183
1546	1116	626
1546	1195	738
1546	1988	156
1546	2544	359
1546	2815	206
1546	3475	180
1546	3739	204
1546	4435	643
1546	5307	270
1546	6295	538
1546	10963	313
1546	13431	433
1547	81	121
1547	206	217
1547	207	495
1547	221	717
1547	225	238
1547	227	677
1547	229	149
1547	377	139
1547	418	399
1547	420	115
1547	429	131
1547	432	292
1547	440	150

1547	441	158
1547	448	115
1547	498	160
1547	511	199
1547	530	120
1547	533	1020
1547	707	175
1547	757	136
1547	768	216
1547	863	138
1547	898	300
1547	971	424
1547	1048	232
1547	1097	164
1547	1151	114
1547	1384	172
1547	1400	231
1547	1510	138
1547	1517	124
1547	1633	149
1547	2433	245
1547	2600	175
1547	2606	123
1547	3676	157
1547	3753	240
1547	3892	271
1547	4069	160
1547	4115	206
1547	4616	272
1547	5237	409

1547	5966	176
1547	7077	209
1547	10839	525
1547	11155	140
1547	12555	114
1547	15262	124
1547	15263	122
1549	163 139	
1549	171 167	
1549	182 301	
1549	207 159	
1549	217 86	
1549	226 46	
1549	227 75	
1549	424 71	
1549	425 157	
1549	539 43	
1549	562 41	
1549	575 216	
1549	604 42	
1549	618 43	
1549	689 79	
1549	690 54	
1549	706 67	
1549	903 136	
1549	959 81	
1549	985 51	
1549	1048	55
1549	1090	76
1549	1106	50

1549	1242	52
1549	1377	50
1549	1515	49
1549	1520	73
1549	1811	59
1549	1976	48
1549	1991	47
1549	2121	41
1549	2131	51
1549	2137	61
1549	2277	77
1549	2524	43
1549	2614	51
1549	2788	52
1549	2823	108
1549	3200	45
1549	3308	55
1549	3400	93
1549	3475	60
1549	3877	83
1549	4115	102
1549	5027	75
1549	10133	70
1549	15267	162
1549	15268	77
1549	15269	68
1549	15270	63
1551	2	868
1551	5	653
1551	7	1025

1551	9	663
1551	18	808
1551	32	676
1551	36	663
1551	45	547
1551	153	505
1551	154	495
1551	159	911
1551	163	550
1551	206	1528
1551	220	653
1551	227	654
1551	230	596
1551	233	2842
1551	436	512
1551	450	965
1551	487	900
1551	707	583
1551	878	691
1551	1091	936
1551	1099	539
1551	1116	649
1551	1203	536
1551	1292	579
1551	1295	819
1551	1298	1620
1551	1300	1569
1551	1303	2236
1551	1312	593
1551	1321	545

1551	1406	982
1551	1886	900
1551	2369	944
1551	3244	526
1551	3413	492
1551	3562	574
1551	4871	570
1551	5949	574
1551	6547	775
1551	7078	721
1551	8149	542
1551	8904	659
1551	8910	591
1551	10857	664
1551	14286	795
1551	15289	916
1551	15290	700
1552	161	1
1552	288	1
1552	299	1
1552	455	3
1552	457	1
1552	468	1
1552	479	1
1552	484	1
1552	646	1
1552	802	2
1552	1475	1
1552	15291	1
1553	7	155

1553	39	894
1553	89	349
1553	157	157
1553	288	344
1553	289	530
1553	298	252
1553	333	297
1553	377	322
1553	378	1169
1553	423	287
1553	430	165
1553	466	179
1553	500	358
1553	528	399
1553	538	193
1553	830	157
1553	903	353
1553	930	175
1553	1104	349
1553	1109	181
1553	1133	168
1553	1179	189
1553	1300	487
1553	1463	146
1553	1700	246
1553	1705	167
1553	2478	171
1553	3184	191
1553	3616	223
1553	4044	1715

1553	4155	209
1553	4767	189
1553	5079	2639
1553	5098	149
1553	5151	154
1553	5437	149
1553	10917	1095
1553	11050	222
1553	12022	359
1553	14645	188
1553	15292	1492
1553	15293	461
1553	15294	274
1553	15295	212
1553	15296	209
1553	15297	208
1553	15298	191
1553	15299	151
1553	15300	135
1554	163 521	
1554	207 258	
1554	227 746	
1554	554 228	
1554	562 302	
1554	570 133	
1554	599 219	
1554	709 169	
1554	718 101	
1554	733 107	
1554	735 579	

1554	857 141	
1554	863 390	
1554	868 636	
1554	903 117	
1554	969 170	
1554	975 129	
1554	1048	207
1554	1225	166
1554	1242	362
1554	1244	175
1554	1369	119
1554	1412	130
1554	1415	125
1554	1426	431
1554	1469	122
1554	1513	359
1554	1639	324
1554	1806	407
1554	1814	110
1554	2458	103
1554	2624	246
1554	2742	229
1554	2786	101
1554	2796	112
1554	2855	204
1554	2857	115
1554	2899	166
1554	2902	119
1554	3156	403
1554	3635	142

1554	3993	99
1554	4251	132
1554	4541	223
1554	5844	411
1554	6137	450
1554	6950	142
1554	7348	240
1554	12674	107
1554	15301	154
1555	55	494
1555	67	572
1555	89	459
1555	257	20168
1555	279	274
1555	285	531
1555	288	982
1555	289	1699
1555	290	302
1555	291	665
1555	292	782
1555	294	1038
1555	295	604
1555	296	1157
1555	299	1192
1555	300	647
1555	302	353
1555	306	436
1555	310	648
1555	311	340
1555	316	361

1555	318 699	
1555	323 704	
1555	328 428	
1555	344 272	
1555	349 579	
1555	352 435	
1555	374 354	
1555	525 564	
1555	528 424	
1555	641 954	
1555	680 422	
1555	701 1319	
1555	786 506	
1555	792 587	
1555	800 376	
1555	972 405	
1555	1243	541
1555	1470	290
1555	2094	718
1555	2220	345
1555	2462	326
1555	2523	397
1555	3074	340
1555	3491	368
1555	4350	256
1555	4488	263
1555	4814	279
1555	5568	1642
1555	7619	1190
1556	152 4052	

1556	154 436	
1556	163 1243	
1556	181 2524	
1556	186 584	
1556	188 1802	
1556	190 882	
1556	226 435	
1556	227 2623	
1556	238 440	
1556	370 498	
1556	436 1208	
1556	506 440	
1556	737 794	
1556	854 439	
1556	856 439	
1556	869 673	
1556	920 1638	
1556	963 923	
1556	1113	652
1556	1131	548
1556	1134	2091
1556	1433	1008
1556	1992	489
1556	2003	528
1556	2006	5123
1556	2624	587
1556	2775	1510
1556	2962	424
1556	3264	800
1556	3417	596

1556	3788	502
1556	3798	430
1556	3940	447
1556	4613	547
1556	4693	2390
1556	7049	1094
1556	7530	1210
1556	7802	652
1556	8904	475
1556	10675	1692
1556	10717	3790
1556	10833	718
1556	12930	1167
1556	13128	794
1556	15302	1275
1556	15303	570
1556	15304	527
1556	15305	505
1556	15306	451
1557	154 763	
1557	418 357	
1557	444 295	
1557	603 2177	
1557	616 1506	
1557	744 2132	
1557	748 912	
1557	753 212	
1557	756 409	
1557	772 560	
1557	1222	1196

1557	1745	419
1557	1835	416
1557	1965	782
1557	2146	372
1557	2161	255
1557	2605	217
1557	2667	600
1557	2668	467
1557	2669	1175
1557	2670	1079
1557	2671	736
1557	2672	259
1557	2673	827
1557	2677	1096
1557	3520	232
1557	4865	339
1557	7468	390
1557	7493	393
1557	8510	177
1557	9766	196
1557	10518	199
1557	11126	197
1557	11206	297
1557	11209	644
1557	13092	311
1557	13650	709
1557	14744	210
1557	15307	973
1557	15308	539
1557	15309	474

1557	15310	443
1557	15311	373
1557	15312	268
1557	15313	250
1557	15314	243
1557	15315	188
1557	15316	187
1557	15317	178
1557	15318	172
1558	67	105
1558	77	15
1558	89	307
1558	157	32
1558	171	25
1558	190	18
1558	198	15
1558	228	15
1558	229	107
1558	230	31
1558	234	16
1558	288	54
1558	289	41
1558	295	105
1558	300	157
1558	377	50
1558	424	14
1558	455	14
1558	486	1366
1558	488	198
1558	498	282

1558	502	44
1558	510	24
1558	528	22
1558	544	48
1558	704	31
1558	706	60
1558	725	46
1558	733	25
1558	917	47
1558	959	26
1558	966	21
1558	972	13
1558	1034	18
1558	1038	72
1558	1180	15
1558	1186	21
1558	1198	23
1558	1672	14
1558	1673	18964
1558	1677	37
1558	2343	30
1558	2435	31
1558	2619	17
1558	2657	15
1558	3240	23
1558	3332	14
1558	4578	27
1558	15319	401
1558	15320	122
1559	97	127

1559	182 185	
1559	184 1205	
1559	207 990	
1559	220 325	
1559	227 2720	
1559	228 144	
1559	229 984	
1559	230 451	
1559	300 107	
1559	328 393	
1559	329 852	
1559	333 366	
1559	377 139	
1559	412 1137	
1559	424 743	
1559	458 82806	
1559	468 736	
1559	475 3191	
1559	481 145	
1559	507 283	
1559	533 217	
1559	538 210	
1559	540 468	
1559	545 186	
1559	691 123	
1559	705 73	
1559	706 74	
1559	714 112	
1559	715 73	
1559	1042	1674

1559	1045	1157
1559	1090	223
1559	1194	427
1559	1200	191
1559	1246	435
1559	1375	163
1559	1384	130
1559	1400	243
1559	1778	234
1559	1974	108
1559	2544	92
1559	2850	257
1559	4466	203
1559	4474	425
1559	6132	212
1559	7834	499
1559	13965	86
1559	14614	96
1559	15321	79
1560	67	2931
1560	89	1949
1560	157	122
1560	251	2
1560	256	73
1560	288	428
1560	289	6988
1560	292	200
1560	295	82
1560	300	700
1560	306	1003

1560	321 13	
1560	329 22	
1560	355 7	
1560	439 10	
1560	458 47	
1560	466 100	
1560	468 83	
1560	481 162	
1560	531 88	
1560	542 14	
1560	545 20	
1560	622 985	
1560	679 226	
1560	701 110	
1560	708 50	
1560	725 40	
1560	889 35	
1560	903 5	
1560	907 356	
1560	1045	99
1560	1247	143
1560	1444	137
1560	1455	29
1560	1464	464
1560	1832	2
1560	2025	225
1560	2080	366
1560	3080	3062
1560	3231	858
1560	3317	1845

1560	3332	4
1560	3743	220
1560	3769	45
1560	7340	4674
1560	8364	69
1560	8707	18
1560	15322	12
1560	15323	12
1560	15324	10
1562	56	389
1562	89	673
1562	154	80
1562	167	63
1562	173	389
1562	207	130
1562	220	3565
1562	230	490
1562	234	1024
1562	292	66
1562	300	116
1562	333	77
1562	371	134
1562	377	173
1562	458	97
1562	466	173
1562	498	253
1562	500	64
1562	533	219
1562	546	224
1562	599	121

1562	679	115
1562	707	120
1562	908	63
1562	917	81
1562	952	63
1562	982	153
1562	1041	61
1562	1044	483
1562	1048	434
1562	1249	1868
1562	1400	510
1562	1513	632
1562	1632	124
1562	1659	59
1562	1672	221
1562	1673	64
1562	1803	158
1562	1807	381
1562	2343	643
1562	2346	248
1562	2595	270
1562	2788	184
1562	3011	110
1562	3200	83
1562	5226	151
1562	7324	73
1562	15327	97
1562	15328	85
1562	15329	81
1563	55	25

1563	67	9
1563	251	4
1563	257	6
1563	289	10
1563	299	25
1563	320	22
1563	701	26
1563	787	4
1563	1098	17
1563	1121	4
1563	1123	4
1563	1436	7
1563	1438	5
1563	1440	6
1563	1446	5
1563	1475	5
1563	2394	4
1563	2395	7
1563	2440	21
1563	2462	13
1563	3060	7
1563	3311	4
1563	3466	4
1563	3892	5
1563	4260	5
1563	4817	38
1563	5238	5
1563	7838	10
1563	7846	8
1563	8372	8

1563	9820	18
1563	10717	10
1563	12555	6
1563	13669	5
1563	14929	4
1563	15330	39
1563	15331	10
1563	15332	7
1563	15333	7
1563	15334	7
1563	15335	6
1563	15336	6
1563	15337	6
1563	15338	6
1563	15339	6
1563	15340	5
1563	15341	5
1563	15342	5
1563	15343	5
1564	55	308
1564	67	1306
1564	97	110
1564	157	198
1564	161	87
1564	257	360
1564	288	2513
1564	289	15282
1564	291	232
1564	292	875
1564	294	303

1564	295 815
1564	298 101
1564	299 769
1564	300 874
1564	302 202
1564	306 482
1564	314 410
1564	321 93
1564	325 273
1564	328 120
1564	331 148
1564	333 310
1564	344 580
1564	349 248
1564	352 196
1564	458 178
1564	461 621
1564	466 531
1564	475 249
1564	523 106
1564	528 188
1564	531 114
1564	543 248
1564	646 104
1564	679 868
1564	681 251
1564	686 364
1564	701 840
1564	802 269
1564	1042 97

1564	1246	86
1564	1428	89
1564	1453	95
1564	1613	131
1564	1934	433
1564	2020	157
1564	2548	91
1564	2984	289
1564	3173	139
1565	56	254
1565	81	299
1565	154	703
1565	190	353
1565	199	264
1565	207	750
1565	210	694
1565	217	170
1565	225	1139
1565	226	675
1565	228	618
1565	229	261
1565	236	196
1565	298	219
1565	416	217
1565	418	476
1565	421	212
1565	424	400
1565	439	442
1565	443	210
1565	450	263

1565	511	171
1565	517	287
1565	614	2786
1565	629	400
1565	709	572
1565	715	536
1565	982	224
1565	1062	197
1565	1090	249
1565	1098	287
1565	1116	1140
1565	1151	508
1565	1375	430
1565	1400	314
1565	1510	345
1565	1512	219
1565	1515	380
1565	1974	455
1565	2840	536
1565	3030	196
1565	3057	291
1565	4064	184
1565	4079	340
1565	4089	341
1565	6424	171
1565	11177	219
1565	15344	229
1565	15345	191
1565	15346	172
1566	88	244

1566	89	416
1566	154	339
1566	190	349
1566	196	364
1566	199	226
1566	206	255
1566	207	1222
1566	217	391
1566	225	408
1566	226	144
1566	227	483
1566	228	399
1566	229	898
1566	298	197
1566	412	329
1566	419	167
1566	424	1142
1566	425	479
1566	428	271
1566	430	160
1566	432	373
1566	440	172
1566	526	181
1566	533	337
1566	539	149
1566	546	363
1566	547	141
1566	689	330
1566	690	147
1566	703	173

1566	709 174	
1566	903 214	
1566	969 270	
1566	1048	478
1566	1090	624
1566	1098	177
1566	1106	204
1566	1390	340
1566	1400	513
1566	1424	228
1566	1512	199
1566	1976	364
1566	2134	229
1566	2137	220
1566	2582	343
1566	3200	412
1566	3730	233
1566	4435	141
1566	4704	153
1567	161 13322	
1567	190 3161	
1567	198 2252	
1567	229 690	
1567	307 840	
1567	349 1004	
1567	378 845	
1567	386 1690	
1567	455 876	
1567	461 1136	
1567	467 750	

1567	479 3817	
1567	490 2434	
1567	498 786	
1567	518 2074	
1567	535 719	
1567	657 1596	
1567	660 1712	
1567	669 1589	
1567	679 16305	
1567	680 1334	
1567	686 780	
1567	701 3649	
1567	966 2441	
1567	1057	1047
1567	1447	1042
1567	1464	817
1567	1470	7463
1567	1475	852
1567	1602	9742
1567	1980	842
1567	2645	6969
1567	2977	873
1567	3427	790
1567	3859	17310
1567	4565	1041
1567	5228	3465
1567	5487	4190
1567	5845	747
1567	6313	803
1567	12947	1629

1567 13549 893

1567 15347 9453

1567 15348 2324

1567 15349 2214

1567 15350 1977

1567 15351 1278

1567 15352 1114

1567 15353 1087

1567 15354 1045

1568 89 29701

1568 161 398

1568 209 108

1568 227 700

1568 230 1756

1568 288 1438

1568 289 1391

1568 292 642

1568 295 4040

1568 300 2564

1568 306 380

1568 325 146

1568 333 570

1568 378 110

1568 458 2670

1568 461 470

1568 466 4108

1568 470 1176

1568 486 1076

1568 498 516

1568 542 342

1568	646 349	
1568	701 150	
1568	704 800	
1568	1032	608
1568	1034	406
1568	1037	116
1568	1039	165
1568	1043	232
1568	1045	209
1568	1430	149
1568	1475	1388
1568	2025	2388
1568	2080	284
1568	2984	164
1568	3373	115
1568	3378	1632
1568	4309	292
1568	4350	115
1568	4471	152
1568	4474	754
1568	5314	242
1568	5749	12770
1568	5750	141
1568	7088	1080
1568	7091	216
1568	7706	249
1568	15355	377
1568	15356	168
1568	15357	127
1570	71 358	

1570	163	273
1570	234	143
1570	298	315
1570	300	714
1570	311	167
1570	333	739
1570	342	150
1570	430	653
1570	437	154
1570	445	323
1570	498	238
1570	548	192
1570	680	197
1570	681	463
1570	703	334
1570	704	168
1570	757	145
1570	898	621
1570	991	140
1570	1098	536
1570	1383	444
1570	1556	300
1570	1609	182
1570	1762	211
1570	1971	474
1570	2277	1399
1570	2521	169
1570	3057	360
1570	3202	291
1570	3206	283

1570	3310	160
1570	4704	253
1570	5079	162
1570	7283	312
1570	8592	327
1570	8715	2122
1570	9401	426
1570	11155	389
1570	14445	143
1570	15366	887
1570	15367	511
1570	15368	419
1570	15369	362
1570	15370	318
1570	15371	285
1570	15372	282
1570	15373	268
1570	15374	221
1570	15375	217
1572	55	649
1572	67	2100
1572	89	1343
1572	257	974
1572	279	363
1572	285	1611
1572	288	1747
1572	289	8633
1572	291	775
1572	292	1751
1572	293	1292

1572	294 594
1572	295 623
1572	296 1349
1572	299 1552
1572	300 519
1572	301 516
1572	302 1167
1572	309 472
1572	310 609
1572	312 572
1572	314 425
1572	318 1043
1572	325 607
1572	326 435
1572	333 2778
1572	344 751
1572	349 861
1572	378 446
1572	455 389
1572	461 559
1572	465 372
1572	466 1338
1572	498 579
1572	523 869
1572	646 788
1572	679 615
1572	680 830
1572	690 384
1572	701 441
1572	793 494

1572	906 980	
1572	908 395	
1572	1601	562
1572	1684	726
1572	2021	349
1572	2523	508
1572	2801	722
1572	6383	467
1572	6543	580
1573	157 105	
1573	173 83	
1573	190 826	
1573	202 185	
1573	207 1263	
1573	217 206	
1573	220 79	
1573	227 751	
1573	229 692	
1573	298 368	
1573	424 182	
1573	425 102	
1573	427 114	
1573	428 400	
1573	429 149	
1573	437 223	
1573	439 266	
1573	498 430	
1573	533 787	
1573	546 138	
1573	615 111	

1573	618 191	
1573	716 111	
1573	718 282	
1573	903 79	
1573	980 128	
1573	989 194	
1573	1090	269
1573	1186	283
1573	1244	165
1573	1390	141
1573	1400	254
1573	1515	120
1573	1517	79
1573	1520	110
1573	1663	451
1573	1764	123
1573	1814	195
1573	2131	117
1573	2788	128
1573	3128	160
1573	3293	103
1573	3333	931
1573	3370	109
1573	3767	131
1573	5304	150
1573	7355	130
1573	12105	169
1573	15378	113
1573	15379	82
1574	154 774	

1574	207	558
1574	210	488
1574	217	487
1574	220	1018
1574	226	596
1574	234	1385
1574	250	194
1574	377	296
1574	420	684
1574	441	743
1574	447	523
1574	475	237
1574	489	341
1574	497	408
1574	511	1338
1574	709	1537
1574	716	214
1574	757	659
1574	868	1851
1574	969	727
1574	978	349
1574	1048	1427
1574	1090	831
1574	1116	213
1574	1406	2404
1574	1412	357
1574	1520	1430
1574	1873	623
1574	1974	2376
1574	1975	2690

1574	1978	1372
1574	1979	607
1574	1982	234
1574	1987	340
1574	1989	772
1574	2738	297
1574	3640	190
1574	3769	236
1574	4089	223
1574	5488	345
1574	5845	286
1574	7040	224
1574	10050	831
1574	10897	1025
1574	11379	526
1574	15380	652
1574	15381	365
1574	15382	274
1574	15383	263
1575	154 341	
1575	227 355	
1575	554 164	
1575	562 505	
1575	576 182	
1575	599 180	
1575	601 152	
1575	706 267	
1575	735 642	
1575	857 179	
1575	959 144	

1575	1242	410
1575	1369	195
1575	1412	177
1575	1427	264
1575	1464	157
1575	1881	377
1575	1944	180
1575	2039	168
1575	2043	344
1575	2289	209
1575	2532	344
1575	3436	149
1575	3458	290
1575	3481	151
1575	3511	180
1575	3768	159
1575	4094	221
1575	4095	228
1575	4129	226
1575	5452	293
1575	6295	225
1575	6362	151
1575	7374	177
1575	8583	162
1575	9585	168
1575	9862	256
1575	10428	480
1575	14303	439
1575	15384	837
1575	15385	595

1575	15386	432
1575	15387	402
1575	15388	279
1575	15389	237
1575	15390	175
1575	15391	170
1575	15392	151
1575	15393	148
1575	15394	146
1576	7	367
1576	96	421
1576	190	198
1576	289	179
1576	389	229
1576	412	422
1576	538	219
1576	679	490
1576	680	192
1576	737	393
1576	889	5618
1576	985	213
1576	1083	431
1576	1243	310
1576	1244	287
1576	1375	316
1576	1464	731
1576	1694	2857
1576	1782	1103
1576	1934	179
1576	2044	3309

1576	2183	334
1576	3760	542
1576	4815	497
1576	5285	594
1576	5810	361
1576	6700	234
1576	8422	225
1576	9348	296
1576	11956	297
1576	13588	183
1576	15395	989
1576	15396	602
1576	15397	597
1576	15398	582
1576	15399	479
1576	15400	472
1576	15401	438
1576	15402	383
1576	15403	366
1576	15404	361
1576	15405	350
1576	15406	341
1576	15407	333
1576	15408	302
1576	15409	249
1576	15410	245
1576	15411	217
1576	15412	205
1576	15413	198
1577	67 647	

1577	89	3457
1577	161	516
1577	257	7000
1577	288	3906
1577	289	1447
1577	290	1102
1577	294	1178
1577	295	860
1577	299	282
1577	300	315
1577	304	458
1577	330	440
1577	344	568
1577	349	245
1577	352	345
1577	377	797
1577	386	1807
1577	396	1799
1577	455	895
1577	459	3305
1577	461	634
1577	466	805
1577	475	309
1577	479	3457
1577	525	208
1577	531	210
1577	660	1608
1577	663	374
1577	915	347
1577	972	1265

1577	1673	312
1577	2977	546
1577	3080	447
1577	3128	253
1577	3576	1225
1577	3586	318
1577	4257	199
1577	5076	1170
1577	7340	270
1577	7656	695
1577	7868	297
1577	9660	203
1577	10992	249
1577	15414	479
1577	15415	232
1577	15416	227
1577	15417	202
1577	15418	201
1577	15419	200
1578	67	191
1578	89	333
1578	279	77
1578	288	1872
1578	289	332
1578	290	239
1578	291	61
1578	292	523
1578	294	195
1578	295	3149
1578	296	160

1578 299 734

1578 300 294

1578 301 132

1578 306 862

1578 308 105

1578 314 264

1578 323 1219

1578 332 147

1578 349 51

1578 352 1775

1578 386 120

1578 461 144

1578 466 114

1578 528 168

1578 535 314

1578 543 286

1578 544 101

1578 690 81

1578 699 102

1578 701 114

1578 906 120

1578 913 491

1578 915 581

1578 1042 84

1578 1051 276

1578 1601 97

1578 1602 495

1578 1604 95

1578 1934 70

1578 1935 70

1578	1936	252
1578	2018	124
1578	2607	148
1578	2939	589
1578	3491	51
1578	4562	98
1578	4812	186
1578	5534	781
1578	11067	155
1579	56	380
1579	81	637
1579	154	1110
1579	173	780
1579	206	413
1579	207	1755
1579	226	1060
1579	227	2388
1579	228	887
1579	233	566
1579	271	435
1579	416	314
1579	424	591
1579	425	464
1579	430	316
1579	434	656
1579	439	782
1579	533	336
1579	547	393
1579	735	331
1579	903	577

1579	982 543	
1579	1090	643
1579	1106	385
1579	1109	1591
1579	1390	430
1579	1424	493
1579	1515	317
1579	1517	410
1579	1609	331
1579	1976	515
1579	2277	729
1579	2572	680
1579	2708	304
1579	2823	433
1579	3200	401
1579	3333	363
1579	4079	305
1579	4260	546
1579	4802	305
1579	5515	303
1579	6539	580
1579	6785	353
1579	7077	637
1579	7211	332
1579	8710	366
1579	11765	502
1579	13832	331
1579	15119	581
1579	15420	383
1580	89 1001	

1580	155 334
1580	157 1767
1580	279 170
1580	285 241
1580	288 290
1580	289 4281
1580	290 1251
1580	291 3271
1580	296 213
1580	300 150
1580	302 215
1580	305 343
1580	309 151
1580	310 547
1580	311 172
1580	318 532
1580	323 1862
1580	325 1103
1580	326 496
1580	349 1247
1580	377 230
1580	455 2081
1580	461 200
1580	466 532
1580	467 153
1580	468 137
1580	528 415
1580	532 444
1580	534 134
1580	538 326

1580	680 1375	
1580	681 195	
1580	682 426	
1580	686 264	
1580	786 737	
1580	791 268	
1580	794 234	
1580	906 293	
1580	908 522	
1580	911 121	
1580	1459	305
1580	1613	119
1580	1684	454
1580	1860	179
1580	2094	177
1580	2523	559
1580	14516	456
1580	15421	1755
1580	15422	151
1581	173 803	
1581	198 787	
1581	199 742	
1581	217 942	
1581	220 735	
1581	222 1430	
1581	228 2824	
1581	421 933	
1581	430 873	
1581	439 1075	
1581	615 1393	

1581	840 1158	
1581	858 813	
1581	898 10145	
1581	1043	1241
1581	1048	1193
1581	1239	764
1581	1375	1543
1581	1378	900
1581	1383	687
1581	1470	897
1581	1645	1022
1581	1958	663
1581	2271	759
1581	2660	1327
1581	2661	1373
1581	2840	2081
1581	3057	1290
1581	3186	1179
1581	3200	709
1581	3333	1155
1581	4079	1122
1581	4750	1081
1581	5027	1180
1581	5217	1074
1581	5439	1032
1581	5549	1117
1581	7322	709
1581	7832	684
1581	8479	1189
1581	10617	1161

1581	11215	1049
1581	11484	1106
1581	12104	714
1581	12154	1397
1581	15423	1553
1581	15424	1306
1581	15425	886
1581	15426	876
1581	15427	717
1582	154 714	
1582	198 364	
1582	209 125	
1582	229 90	
1582	498 671	
1582	512 83	
1582	537 637	
1582	718 92	
1582	977 73	
1582	1045	114
1582	1105	95
1582	1239	92
1582	1378	177
1582	1510	74
1582	2840	1056
1582	3205	536
1582	3468	133
1582	4191	220
1582	4201	1024
1582	4262	424
1582	4434	89

1582	4821	127
1582	4822	75
1582	4834	131
1582	4837	598
1582	4898	105
1582	5423	110
1582	5966	218
1582	6293	1136
1582	6792	329
1582	10214	727
1582	10799	428
1582	11548	202
1582	11803	266
1582	13515	137
1582	15428	513
1582	15429	293
1582	15430	270
1582	15431	253
1582	15432	203
1582	15433	167
1582	15434	153
1582	15435	110
1582	15436	98
1582	15437	84
1582	15438	82
1582	15439	80
1582	15440	71
1582	15441	71
1582	15442	69
1583	65 524	

1583	154	1013
1583	166	220
1583	172	1404
1583	173	2157
1583	190	3702
1583	210	1473
1583	215	1880
1583	225	2862
1583	228	622
1583	229	1019
1583	235	1026
1583	237	261
1583	316	766
1583	378	245
1583	429	452
1583	441	370
1583	445	438
1583	486	462
1583	511	231
1583	521	2246
1583	526	301
1583	546	503
1583	614	250
1583	632	1451
1583	851	290
1583	859	608
1583	961	1027
1583	1116	396
1583	1151	1831
1583	1239	641

1583	1375	5859
1583	1381	250
1583	1418	389
1583	1736	468
1583	1781	366
1583	1980	263
1583	1984	403
1583	2352	1088
1583	2836	415
1583	3057	1874
1583	3238	1373
1583	3627	473
1583	5215	1558
1583	5768	583
1583	8711	216
1583	13711	340
1583	13748	349
1583	15443	909
1583	15444	266
1584	190 32	
1584	209 200	
1584	230 120	
1584	234 34	
1584	298 33	
1584	300 42	
1584	302 41	
1584	316 47	
1584	333 228	
1584	377 42	
1584	403 143	

1584	412	111
1584	430	44
1584	481	49
1584	486	46
1584	500	36
1584	512	30
1584	526	39
1584	536	129
1584	540	41
1584	687	51
1584	689	42
1584	704	46
1584	716	35
1584	808	66
1584	1043	69
1584	1180	34
1584	1183	182
1584	1184	96
1584	1186	80
1584	1190	36
1584	1194	81
1584	1197	264
1584	1246	47
1584	1406	85
1584	1689	43
1584	1866	44
1584	2129	58
1584	2580	36
1584	3082	77
1584	3333	65

1584	3737	48
1584	4196	157
1584	4255	61
1584	4262	46
1584	6770	32
1584	15445	60
1584	15446	41
1584	15447	35
1584	15448	35
1585	51	24657
1585	56	1295
1585	61	4147
1585	65	1918
1585	67	5741
1585	69	1379
1585	72	1956
1585	80	1546
1585	99	1481
1585	157	1970
1585	159	1552
1585	182	2520
1585	187	3223
1585	193	1302
1585	253	2801
1585	285	2222
1585	291	1879
1585	511	8839
1585	748	1460
1585	777	1399
1585	945	1726

1585	993 9381	
1585	1001	2418
1585	1009	1374
1585	1013	1766
1585	1116	2883
1585	1122	5181
1585	1306	4038
1585	1405	1379
1585	1413	1641
1585	1810	1768
1585	2018	1621
1585	2469	1268
1585	2554	2192
1585	2608	1283
1585	3104	4192
1585	3302	1367
1585	3481	1469
1585	3768	1657
1585	3921	3587
1585	4130	2745
1585	5072	1303
1585	5452	1621
1585	7054	2545
1585	15449	2570
1585	15450	1826
1585	15451	1732
1585	15452	1658
1585	15453	1601
1585	15454	1324
1586	176 53	

1586	198 32	
1586	220 94	
1586	234 28	
1586	472 9	
1586	521 32	
1586	706 39	
1586	707 785	
1586	732 97	
1586	808 20	
1586	821 8	
1586	841 72	
1586	843 38	
1586	854 144	
1586	917 636	
1586	918 1356	
1586	928 11	
1586	930 21	
1586	947 14	
1586	951 35	
1586	952 216	
1586	1104	34
1586	1249	98
1586	1257	24
1586	1337	161
1586	1358	82
1586	1360	66
1586	1366	61
1586	1369	46
1586	1372	9
1586	1521	8

1586	1527	68
1586	1810	134
1586	1811	23
1586	1981	34
1586	2342	8
1586	2360	21
1586	2744	35
1586	2838	12
1586	3238	32
1586	3624	47
1586	3628	64
1586	3636	208
1586	3640	32
1586	5157	36
1586	5485	10
1586	6259	12
1586	10774	22
1586	10897	33
1586	15455	16
1587	52	2019
1587	53	577
1587	54	375
1587	55	660
1587	67	1505
1587	70	2306
1587	152	1812
1587	154	721
1587	163	1182
1587	165	523
1587	171	361

1587 172 1374
1587 173 613
1587 182 1198
1587 183 902
1587 186 509
1587 217 920
1587 221 3274
1587 223 588
1587 225 771
1587 229 918
1587 538 663
1587 614 1103
1587 968 1666
1587 1048 513
1587 1118 1575
1587 1151 594
1587 1385 1315
1587 1394 575
1587 1395 387
1587 1632 683
1587 1728 834
1587 1873 454
1587 1958 419
1587 2289 488
1587 2407 1222
1587 2641 917
1587 3046 1759
1587 3164 918
1587 3501 908
1587 3670 778

1587	3945	416
1587	4649	358
1587	6252	756
1587	7871	422
1587	11491	1379
1587	15456	672
1587	15457	644
1587	15458	642
1587	15459	354
1588	176	238
1588	198	96
1588	227	9
1588	344	1511
1588	356	1571
1588	360	6
1588	461	597
1588	562	309
1588	679	183
1588	706	443
1588	733	1
1588	868	2
1588	959	352
1588	1244	7
1588	1249	19
1588	1412	67
1588	1445	2
1588	1459	510
1588	1468	6
1588	1694	32
1588	1696	3488

1588	2439	405
1588	2442	259
1588	2444	32
1588	2445	244
1588	2447	1302
1588	2449	2
1588	2453	85
1588	2455	27
1588	2467	402
1588	2468	1160
1588	2834	108
1588	2901	43
1588	3460	3
1588	3470	4
1588	3583	301
1588	3585	25
1588	3588	1
1588	3598	58
1588	4031	38
1588	4543	2
1588	5267	251
1588	5649	23
1588	6367	23
1588	7041	3
1588	7704	4
1588	9849	116
1588	15460	561
1588	15461	378
1588	15462	18
1589	65 105	

1589	154 127	
1589	182 105	
1589	190 161	
1589	217 365	
1589	418 165	
1589	498 106	
1589	500 125	
1589	503 103	
1589	507 118	
1589	518 121	
1589	632 155	
1589	687 219	
1589	707 161	
1589	763 207	
1589	808 138	
1589	823 105	
1589	830 245	
1589	834 191	
1589	839 135	
1589	846 219	
1589	917 123	
1589	920 196	
1589	953 114	
1589	968 247	
1589	1177	114
1589	1418	101
1589	1533	110
1589	1547	113
1589	1551	170
1589	1672	545

1589	2379	122
1589	2576	142
1589	2579	137
1589	2586	152
1589	3997	305
1589	4002	125
1589	4611	116
1589	8293	169
1589	9260	111
1589	10675	223
1589	11264	253
1589	11900	117
1589	11936	142
1589	14908	143
1589	15463	205
1589	15464	186
1589	15465	171
1589	15466	103
1589	15467	103
1590	55	9864
1590	64	292
1590	67	12308
1590	72	333
1590	89	11117
1590	97	554
1590	157	245
1590	285	1818
1590	288	1997
1590	289	8953
1590	292	539

1590	295 867	
1590	296 288	
1590	298 759	
1590	306 202	
1590	310 433	
1590	317 202	
1590	349 639	
1590	350 704	
1590	354 366	
1590	425 387	
1590	466 482	
1590	485 331	
1590	523 1003	
1590	525 345	
1590	526 2669	
1590	532 672	
1590	541 204	
1590	680 374	
1590	701 642	
1590	789 437	
1590	889 1391	
1590	906 380	
1590	1098	226
1590	1392	238
1590	1503	235
1590	1609	240
1590	2083	818
1590	2094	739
1590	2521	575
1590	3186	225

1590	3198	1375
1590	3288	204
1590	3502	2603
1590	3902	238
1590	5735	207
1590	7292	663
1590	14222	217
1590	15468	214
1590	15469	211
1591	56	73
1591	89	550
1591	288	3576
1591	289	877
1591	290	335
1591	291	736
1591	292	330
1591	293	177
1591	294	298
1591	295	977
1591	300	82
1591	301	94
1591	302	218
1591	310	91
1591	318	283
1591	325	262
1591	327	73
1591	328	352
1591	329	71
1591	330	86
1591	333	680

1591 342 103

1591 349 694

1591 403 153

1591 461 192

1591 465 209

1591 484 466

1591 498 2211

1591 523 185

1591 525 181

1591 536 140

1591 680 2318

1591 701 122

1591 907 187

1591 908 151

1591 1043 95

1591 1377 143

1591 1441 167

1591 1580 72

1591 1613 191

1591 1684 173

1591 2801 111

1591 4385 150

1591 4716 116

1591 6346 126

1591 6567 143

1591 8364 108

1591 13292 77

1591 15470 168

1591 15471 78

1592 176 118

1592	220	194
1592	228	648
1592	229	192
1592	230	451
1592	234	162
1592	316	113
1592	371	129
1592	377	125
1592	378	219
1592	383	489
1592	403	150
1592	472	444
1592	489	163
1592	493	175
1592	497	253
1592	511	109
1592	533	229
1592	614	114
1592	706	965
1592	707	362
1592	714	114
1592	735	131
1592	854	139
1592	917	122
1592	932	112
1592	952	261
1592	959	175
1592	966	199
1592	982	683
1592	1104	445

1592	1116	243
1592	1186	232
1592	1239	123
1592	1248	147
1592	1249	932
1592	1406	502
1592	1672	138
1592	1866	471
1592	1873	253
1592	1974	115
1592	2344	109
1592	2346	465
1592	2347	270
1592	2364	351
1592	3400	249
1592	3873	175
1592	4179	224
1592	5772	124
1592	15472	246
1593	157 402	
1593	239 1221	
1593	240 234	
1593	257 459	
1593	261 119	
1593	279 502	
1593	285 306	
1593	288 239	
1593	289 2592	
1593	292 12320	
1593	295 20312	

1593	312 1827	
1593	314 11048	
1593	317 111	
1593	322 2478	
1593	327 216	
1593	330 4387	
1593	331 278	
1593	332 106	
1593	352 110	
1593	468 159	
1593	482 124	
1593	484 279	
1593	527 243	
1593	913 208	
1593	1037	302
1593	1042	219
1593	1451	299
1593	1537	488
1593	1544	134
1593	1596	894
1593	2083	100
1593	2220	639
1593	2548	766
1593	3314	2619
1593	3317	200
1593	3318	490
1593	4617	303
1593	4713	1598
1593	4814	213
1593	6346	128

1593	8081	189
1593	8605	120
1593	11069	101
1593	12535	396
1593	12545	119
1593	14734	267
1593	15473	1364
1593	15474	181
1593	15475	105
1594	65	537
1594	72	1703
1594	154	777
1594	172	750
1594	173	1397
1594	187	919
1594	190	3469
1594	207	864
1594	217	485
1594	225	540
1594	226	3661
1594	227	20712
1594	229	737
1594	234	400
1594	362	1637
1594	377	351
1594	412	579
1594	424	605
1594	429	1451
1594	439	619
1594	441	776

1594	453	778
1594	498	660
1594	585	969
1594	614	410
1594	615	392
1594	705	666
1594	706	391
1594	707	449
1594	709	1245
1594	728	918
1594	733	2153
1594	857	518
1594	859	403
1594	982	1565
1594	1118	543
1594	1239	479
1594	1240	500
1594	1414	5775
1594	1416	972
1594	1807	1430
1594	1974	1304
1594	1977	535
1594	2531	478
1594	3071	1707
1594	3938	460
1594	4793	507
1594	8710	966
1594	10969	409
1594	15476	492
1595	59	104

1595	72	5234
1595	77	302
1595	89	291
1595	99	125
1595	159	589
1595	163	1786
1595	193	202
1595	227	1177
1595	228	157
1595	234	126
1595	418	107
1595	441	1016
1595	511	385
1595	562	133
1595	599	918
1595	631	198
1595	706	487
1595	717	116
1595	733	158
1595	735	158
1595	757	115
1595	863	184
1595	865	938
1595	868	3411
1595	874	168
1595	959	640
1595	1013	458
1595	1079	164
1595	1122	125
1595	1242	129

1595	1244	152
1595	1249	241
1595	1369	173
1595	1412	470
1595	1534	170
1595	1892	133
1595	1895	348
1595	1897	573
1595	3264	262
1595	4130	115
1595	6950	104
1595	8291	106
1595	9253	255
1595	9513	122
1595	9969	121
1595	15472	122
1595	15477	314
1595	15478	183
1595	15479	141
1596	163 602	
1596	176 1049	
1596	220 655	
1596	227 2034	
1596	234 32	
1596	533 78	
1596	554 169	
1596	562 509	
1596	706 2135	
1596	709 477	
1596	712 96	

1596 730 42

1596 733 432

1596 735 1628

1596 857 47

1596 868 138

1596 951 171

1596 959 715

1596 1242 24

1596 1249 438

1596 1369 1146

1596 1372 1502

1596 1412 2188

1596 1414 29

1596 1415 249

1596 1468 59

1596 1469 143

1596 1639 425

1596 1977 109

1596 2127 36

1596 2341 61

1596 2346 1972

1596 2351 133

1596 2463 401

1596 2786 114

1596 2788 40

1596 2899 56

1596 3774 1142

1596 4087 77

1596 4130 24

1596 4180 36

1596	5452	36
1596	7039	25
1596	7935	68
1596	8559	25
1596	15480	45
1596	15481	32
1596	15482	32
1596	15483	23
1596	15484	23
1597	46	177
1597	56	286
1597	65	459
1597	70	213
1597	81	132
1597	96	141
1597	154	690
1597	159	146
1597	163	619
1597	172	216
1597	182	712
1597	197	396
1597	212	289
1597	217	415
1597	225	131
1597	227	1578
1597	229	350
1597	298	577
1597	418	246
1597	421	214
1597	424	159

1597 425 190
1597 525 143
1597 599 614
1597 706 158
1597 715 230
1597 733 132
1597 735 856
1597 868 406
1597 1062 211
1597 1090 276
1597 1098 497
1597 1243 166
1597 1412 909
1597 1416 177
1597 1427 215
1597 1633 287
1597 1639 564
1597 2478 139
1597 3308 268
1597 3469 253
1597 3739 227
1597 3747 561
1597 5079 176
1597 7056 462
1597 8712 159
1597 12026 171
1597 15485 1169
1597 15486 609
1597 15487 352
1599 229 171

1599	412 167	
1599	500 168	
1599	813 220	
1599	816 1335	
1599	823 940	
1599	840 147	
1599	1192	736
1599	1194	177
1599	1197	234
1599	1868	176
1599	2190	144
1599	2200	835
1599	2576	513
1599	2621	188
1599	4003	386
1599	4821	227
1599	4824	403
1599	4826	384
1599	4837	202
1599	5127	684
1599	5129	202
1599	5130	668
1599	5133	192
1599	5134	497
1599	5135	195
1599	5138	486
1599	5631	283
1599	5730	249
1599	6120	329
1599	6199	230

1599	6554	359
1599	10955	188
1599	14853	518
1599	14863	196
1599	15495	520
1599	15496	516
1599	15497	435
1599	15498	374
1599	15499	362
1599	15500	257
1599	15501	198
1599	15502	196
1599	15503	191
1599	15504	173
1599	15505	172
1599	15506	172
1599	15507	168
1599	15508	153
1599	15509	143
1600	55	15849
1600	67	2236
1600	89	6260
1600	230	252
1600	257	1007
1600	288	1370
1600	289	2787
1600	292	3325
1600	293	978
1600	295	161
1600	300	583

1600 302 1035

1600 318 521

1600 325 861

1600 328 1599

1600 332 783

1600 333 2493

1600 349 256

1600 466 1815

1600 485 1023

1600 523 543

1600 524 843

1600 526 1592

1600 532 1166

1600 535 995

1600 536 840

1600 544 437

1600 545 452

1600 548 1571

1600 701 537

1600 798 156

1600 886 476

1600 903 162

1600 915 180

1600 1062 192

1600 1451 185

1600 1456 374

1600 1580 160

1600 2018 407

1600 2083 161

1600 2521 349

1600	3397	397
1600	3502	161
1600	5926	365
1600	7301	428
1600	12550	284
1600	15168	255
1600	15510	330
1600	15511	289
1600	15512	153
1601	2	385
1601	16	236
1601	39	1016
1601	72	167
1601	159	743
1601	173	299
1601	429	362
1601	441	279
1601	450	142
1601	487	217
1601	515	442
1601	599	239
1601	866	374
1601	867	237
1601	870	457
1601	875	572
1601	1062	257
1601	1069	150
1601	1083	2237
1601	1217	385
1601	1376	157

1601	1534	776
1601	1703	187
1601	1814	457
1601	3431	745
1601	3432	1602
1601	3433	731
1601	3435	169
1601	3438	304
1601	3448	316
1601	3449	294
1601	5113	215
1601	8369	808
1601	8668	677
1601	9239	530
1601	10794	502
1601	12668	342
1601	15513	598
1601	15514	428
1601	15515	388
1601	15516	374
1601	15517	282
1601	15518	267
1601	15519	253
1601	15520	205
1601	15521	199
1601	15522	162
1601	15523	152
1601	15524	144
1601	15525	140
1603	15529	6220

1604	2	455
1604	10	366
1604	13	229
1604	25	178
1604	70	354
1604	176	218
1604	189	347
1604	486	253
1604	487	266
1604	488	435
1604	500	186
1604	503	758
1604	506	276
1604	515	176
1604	806	174
1604	968	184
1604	1081	331
1604	1104	410
1604	1145	332
1604	1277	403
1604	1292	588
1604	1300	1041
1604	1303	267
1604	1534	386
1604	1711	311
1604	1714	470
1604	1917	237
1604	2370	236
1604	3087	228
1604	3197	510

1604	3244	296
1604	3416	262
1604	4036	200
1604	4137	255
1604	4162	394
1604	4297	313
1604	4876	427
1604	4985	179
1604	5484	222
1604	8420	248
1604	9637	424
1604	10864	250
1604	10867	449
1604	10870	397
1604	10973	500
1604	15530	332
1604	15531	298
1604	15532	210
1604	15533	197
1604	15534	196
1605	55	108
1605	89	364
1605	161	124
1605	199	142
1605	288	542
1605	289	115
1605	295	140
1605	298	242
1605	300	160
1605	306	239

1605	310	114
1605	317	107
1605	328	161
1605	352	177
1605	432	107
1605	466	200
1605	468	204
1605	486	125
1605	531	242
1605	546	145
1605	548	265
1605	644	167
1605	689	149
1605	690	114
1605	701	190
1605	915	116
1605	968	163
1605	1037	307
1605	1042	214
1605	1379	184
1605	1410	108
1605	1456	116
1605	1463	167
1605	1632	156
1605	1934	156
1605	2521	144
1605	3128	246
1605	4561	112
1605	4564	276
1605	4707	1405

1605	5001	386
1605	5232	118
1605	5534	309
1605	5536	113
1605	7326	138
1605	7782	139
1605	8674	132
1605	9718	117
1605	13460	275
1605	15535	109
1606	55	441
1606	67	2420
1606	89	12350
1606	97	897
1606	157	1075
1606	190	418
1606	217	664
1606	228	572
1606	257	2607
1606	288	1479
1606	289	22170
1606	290	394
1606	291	1270
1606	292	2000
1606	293	402
1606	294	2187
1606	295	1209
1606	298	2640
1606	300	1872
1606	304	409

1606	306 518	
1606	314 415	
1606	321 766	
1606	322 386	
1606	325 762	
1606	333 1084	
1606	344 2370	
1606	349 1201	
1606	352 421	
1606	355 698	
1606	461 1988	
1606	464 497	
1606	466 3504	
1606	498 3301	
1606	524 438	
1606	528 734	
1606	544 625	
1606	548 826	
1606	646 1847	
1606	679 1177	
1606	681 466	
1606	686 887	
1606	689 2563	
1606	701 383	
1606	802 1245	
1606	1052	542
1606	1767	388
1606	1934	458
1606	2801	546
1606	3171	580

1607	7	250
1607	55	185
1607	161	178
1607	288	173
1607	289	1037
1607	292	1008
1607	300	332
1607	316	72
1607	318	69
1607	324	70
1607	325	289
1607	326	226
1607	329	126
1607	333	207
1607	360	75
1607	378	374
1607	386	119
1607	461	75
1607	465	220
1607	466	357
1607	468	73
1607	475	112
1607	486	167
1607	498	521
1607	523	1852
1607	531	103
1607	543	73
1607	544	114
1607	679	312
1607	680	205

1607	681	113
1607	686	225
1607	701	159
1607	704	1030
1607	797	132
1607	903	96
1607	1034	163
1607	1044	115
1607	1045	64
1607	1249	120
1607	1428	100
1607	1455	126
1607	1580	239
1607	1673	119
1607	2020	103
1607	2521	174
1607	12035	73
1607	12925	82
1607	13740	74
1607	15536	98
1608	55	77
1608	56	45
1608	65	63
1608	67	365
1608	89	134
1608	190	122
1608	207	59
1608	217	50
1608	228	85
1608	229	327

1608	288 50	
1608	289 792	
1608	292 190	
1608	293 76	
1608	295 76	
1608	300 105	
1608	333 63	
1608	420 82	
1608	424 52	
1608	432 81	
1608	439 123	
1608	453 56	
1608	455 57	
1608	456 58	
1608	459 58	
1608	528 55	
1608	538 58	
1608	546 94	
1608	679 176	
1608	689 45	
1608	716 47	
1608	841 50	
1608	886 63	
1608	903 103	
1608	980 56	
1608	1098	153
1608	1104	60
1608	1400	218
1608	1515	47
1608	1934	54

1608	2083	75
1608	2523	142
1608	3200	191
1608	3741	58
1608	4028	74
1608	4706	77
1608	5446	57
1608	5934	52
1608	15537	204
1608	15538	51
1611	72	1984
1611	998	499
1611	2500	651
1611	6245	987
1613	45	446
1613	209	870
1613	333	835
1613	371	1139
1613	486	3120
1613	488	465
1613	490	721
1613	493	6061
1613	498	1511
1613	499	743
1613	507	559
1613	510	592
1613	516	581
1613	536	479
1613	537	8373
1613	704	475

1613	808 3265	
1613	830 675	
1613	966 1718	
1613	978 500	
1613	1043	808
1613	1180	490
1613	1182	3184
1613	1191	2374
1613	1200	4312
1613	1401	1301
1613	1410	2521
1613	1852	3556
1613	1863	594
1613	1867	2342
1613	1870	973
1613	1874	484
1613	1905	2147
1613	2843	3814
1613	2940	934
1613	3084	1012
1613	3092	652
1613	3094	1092
1613	3240	3565
1613	3928	400
1613	3946	411
1613	4179	393
1613	5475	1107
1613	5937	842
1613	8254	397
1613	10069	935

1613	11796	578
1613	12788	1090
1613	15564	747
1613	15565	548
1614	7	2468
1614	65	640
1614	89	639
1614	157	1026
1614	190	659
1614	199	896
1614	250	3757
1614	306	1156
1614	328	984
1614	331	1211
1614	377	10092
1614	432	1885
1614	499	1420
1614	516	2268
1614	532	1515
1614	689	935
1614	690	775
1614	724	577
1614	1090	676
1614	1099	1684
1614	1118	2387
1614	1351	879
1614	1613	694
1614	1787	546
1614	1875	840
1614	2708	952

1614	2978	6918
1614	3200	851
1614	3461	802
1614	3475	680
1614	3735	591
1614	5119	569
1614	5474	556
1614	10639	584
1614	10836	831
1614	11038	880
1614	12909	975
1614	15566	1176
1614	15567	942
1614	15568	923
1614	15569	917
1614	15570	909
1614	15571	883
1614	15572	766
1614	15573	638
1614	15574	625
1614	15575	600
1614	15576	585
1614	15577	573
1614	15578	569
1615	198 77	
1615	206 77	
1615	209 75	
1615	217 56	
1615	220 775	
1615	227 638	

1615	229 36	
1615	230 57	
1615	233 56	
1615	234 64	
1615	250 35	
1615	316 46	
1615	412 40	
1615	436 50	
1615	486 40	
1615	534 49	
1615	536 114	
1615	562 62	
1615	709 61	
1615	735 37	
1615	808 52	
1615	982 119	
1615	1091	79
1615	1239	60
1615	1244	99
1615	1249	37
1615	1378	40
1615	1392	76
1615	1645	36
1615	1672	37
1615	1686	37
1615	1689	38
1615	1873	69
1615	1974	79
1615	2121	49
1615	2346	39

1615	2455	60
1615	2625	40
1615	2637	47
1615	2738	37
1615	3078	105
1615	3341	37
1615	3621	59
1615	3732	50
1615	4189	100
1615	4262	85
1615	5306	45
1615	6376	43
1615	6997	37
1615	7470	36
1616	54	88
1616	55	970
1616	65	154
1616	67	461
1616	97	187
1616	251	95
1616	257	1631
1616	265	203
1616	279	110
1616	287	181
1616	288	224
1616	289	876
1616	292	2527
1616	295	137
1616	296	155
1616	302	89

1616	316 790	
1616	322 175	
1616	333 76	
1616	340 83	
1616	349 75	
1616	360 156	
1616	378 215	
1616	389 1276	
1616	455 488	
1616	466 206	
1616	490 173	
1616	498 244	
1616	518 103	
1616	680 187	
1616	701 215	
1616	704 127	
1616	967 195	
1616	972 317	
1616	1112	362
1616	1377	88
1616	1464	296
1616	1934	186
1616	2022	185
1616	2067	212
1616	2220	95
1616	2523	176
1616	4024	72
1616	4705	186
1616	8290	127
1616	8292	439

1616	8991	78
1616	14516	515
1616	15579	107
1616	15580	102
1617	157 306	
1617	192 266	
1617	227 506	
1617	554 260	
1617	599 380	
1617	733 131	
1617	863 353	
1617	874 318	
1617	889 421	
1617	959 183	
1617	1242	550
1617	1513	248
1617	1709	709
1617	2277	209
1617	2629	380
1617	2861	281
1617	2902	285
1617	2938	187
1617	3279	134
1617	3677	284
1617	3778	253
1617	3920	387
1617	4495	353
1617	4541	262
1617	4620	230
1617	5434	497

1617	6458	135
1617	7407	132
1617	8359	157
1617	10394	133
1617	10454	196
1617	11124	129
1617	11589	127
1617	11733	248
1617	12219	145
1617	12358	141
1617	13156	276
1617	15391	138
1617	15581	452
1617	15582	350
1617	15583	292
1617	15584	259
1617	15585	217
1617	15586	209
1617	15587	194
1617	15588	184
1617	15589	160
1617	15590	159
1617	15591	153
1617	15592	152
1618	156 117	
1618	852 311	
1618	862 103	
1618	1085	279
1618	1112	105
1618	1132	222

1618	1205	101
1618	1253	124
1618	1837	252
1618	1897	108
1618	1900	176
1618	1978	88
1618	1985	124
1618	2043	82
1618	2060	101
1618	2561	321
1618	3158	118
1618	3289	96
1618	3886	130
1618	4928	274
1618	5913	196
1618	5917	181
1618	6150	175
1618	6650	293
1618	6724	538
1618	8767	560
1618	9330	173
1618	9506	100
1618	10596	77
1618	11931	82
1618	13168	135
1618	13702	139
1618	14511	2651
1618	15593	306
1618	15594	290
1618	15595	267

1618	15596	266
1618	15597	173
1618	15598	150
1618	15599	126
1618	15600	121
1618	15601	121
1618	15602	108
1618	15603	105
1618	15604	99
1618	15605	95
1618	15606	92
1618	15607	83
1618	15608	76
1618	15609	76
1619	6	1570
1619	7	1312
1619	15	338
1619	25	1907
1619	45	1360
1619	72	305
1619	152	1646
1619	159	502
1619	188	2152
1619	198	1566
1619	226	458
1619	227	736
1619	233	3046
1619	377	300
1619	378	598
1619	436	436

1619	487 1207	
1619	504 9275	
1619	512 257	
1619	602 326	
1619	605 227	
1619	718 308	
1619	785 368	
1619	806 390	
1619	818 1427	
1619	832 261	
1619	846 224	
1619	1044	1082
1619	1099	795
1619	1104	4411
1619	1131	425
1619	1145	912
1619	1216	530
1619	1369	227
1619	1645	351
1619	1714	437
1619	1811	220
1619	1934	368
1619	1974	343
1619	2014	496
1619	2379	274
1619	2945	825
1619	3328	356
1619	3455	902
1619	4105	3100
1619	6257	1173

1619	8711	251
1619	15610	524
1619	15611	418
1619	15612	243
1620	56	268
1620	89	335
1620	196	220
1620	212	317
1620	227	202
1620	233	231
1620	331	652
1620	344	303
1620	412	286
1620	429	216
1620	527	211
1620	548	405
1620	632	745
1620	748	1006
1620	755	7902
1620	770	620
1620	779	446
1620	1198	312
1620	1239	330
1620	1348	422
1620	1410	197
1620	1481	233
1620	1743	247
1620	1846	237
1620	1889	305
1620	1905	269

1620	2500	246
1620	2667	284
1620	3222	1888
1620	3705	365
1620	3817	213
1620	4108	671
1620	4208	275
1620	4720	208
1620	5304	535
1620	5318	260
1620	6346	607
1620	8136	236
1620	8781	778
1620	9718	294
1620	10612	417
1620	15564	306
1620	15613	396
1620	15614	275
1620	15615	275
1620	15616	270
1620	15617	251
1620	15618	241
1620	15619	215
1620	15620	213
1621	55	46
1621	58	57
1621	64	51
1621	67	34
1621	96	50
1621	175	47

1621	193 33	
1621	285 83	
1621	375 107	
1621	389 71	
1621	767 1279	
1621	961 517	
1621	1066	133
1621	1255	99
1621	1256	43
1621	1383	151
1621	1752	50
1621	1983	33
1621	2670	45
1621	3413	66
1621	4871	43
1621	5299	37
1621	6544	97
1621	6760	86
1621	9141	52
1621	10521	908
1621	11088	76
1621	11094	34
1621	11103	50
1621	11186	42
1621	12315	557
1621	12845	38
1621	13185	42
1621	13647	117
1621	15621	239
1621	15622	174

1621	15623	121
1621	15624	71
1621	15625	63
1621	15626	58
1621	15627	48
1621	15628	47
1621	15629	47
1621	15630	44
1621	15631	43
1621	15632	40
1621	15633	37
1621	15634	33
1621	15635	32
1621	15636	32
1622	212	149
1622	441	256
1622	527	159
1622	554	111
1622	565	140
1622	599	432
1622	733	124
1622	752	288
1622	863	253
1622	874	616
1622	879	361
1622	1160	158
1622	1627	156
1622	1837	644
1622	1888	365
1622	1896	253

1622	1983	144
1622	2475	185
1622	2481	129
1622	2668	106
1622	2938	328
1622	3216	122
1622	3280	135
1622	3295	169
1622	3458	121
1622	3778	195
1622	4058	362
1622	4541	109
1622	5295	246
1622	5913	144
1622	7259	103
1622	8673	104
1622	9825	186
1622	9997	184
1622	10105	579
1622	10333	119
1622	11206	138
1622	11631	121
1622	11736	163
1622	12220	183
1622	14378	327
1622	14385	249
1622	15160	142
1622	15637	402
1622	15638	322
1622	15639	201

1622	15640	152
1622	15641	134
1622	15642	114
1622	15643	106
1623	152	830
1623	154	1040
1623	188	1748
1623	190	1070
1623	196	1290
1623	359	817
1623	370	1226
1623	506	1497
1623	606	1428
1623	610	827
1623	812	1589
1623	815	1071
1623	843	869
1623	846	1293
1623	854	1006
1623	918	1224
1623	919	744
1623	920	2149
1623	931	1001
1623	943	889
1623	947	1443
1623	1110	1161
1623	1113	1187
1623	1116	1547
1623	1118	786
1623	1119	1552

1623	1131	1438
1623	1179	764
1623	1216	786
1623	1222	790
1623	1643	1143
1623	1755	1944
1623	1861	853
1623	1941	915
1623	2006	5007
1623	2458	829
1623	2778	1027
1623	2794	1017
1623	3084	1011
1623	3420	2264
1623	3788	919
1623	4616	816
1623	5169	952
1623	5441	1014
1623	6289	811
1623	6678	1223
1623	6724	1043
1623	11516	1256
1623	11979	779
1623	13935	1039
1624	64 55	
1624	154 187	
1624	231 112	
1624	604 108	
1624	618 177	
1624	622 60	

1624	689 75	
1624	858 77	
1624	1089	69
1624	1101	103
1624	1609	80
1624	2540	208
1624	3202	60
1624	3690	74
1624	3706	57
1624	5152	63
1624	6215	57
1624	6221	86
1624	6296	63
1624	6930	101
1624	8701	115
1624	8706	182
1624	9631	93
1624	9727	61
1624	10720	57
1624	10738	71
1624	11465	166
1624	11494	140
1624	12993	68
1624	13170	58
1624	14362	96
1624	15644	360
1624	15645	309
1624	15646	232
1624	15647	212
1624	15648	155

1624	15649	155
1624	15650	109
1624	15651	108
1624	15652	103
1624	15653	83
1624	15654	69
1624	15655	67
1624	15656	66
1624	15657	66
1624	15658	62
1624	15659	61
1624	15660	59
1624	15661	58
1624	15662	57
1625	63	4
1625	73	6
1625	157	6
1625	641	4
1625	972	4
1625	998	4
1625	1464	4
1625	1800	5
1625	1907	6
1625	2048	5
1625	3270	6
1625	3307	12
1625	3966	5
1625	4649	5
1625	4976	10
1625	5088	19

1625	5614	5
1625	5617	12
1625	5985	5
1625	6708	5
1625	7626	4
1625	8308	5
1625	8816	21
1625	10017	5
1625	11455	7
1625	14395	23
1625	15663	26
1625	15664	18
1625	15665	17
1625	15666	17
1625	15667	16
1625	15668	10
1625	15669	10
1625	15670	10
1625	15671	9
1625	15672	8
1625	15673	8
1625	15674	8
1625	15675	8
1625	15676	8
1625	15677	6
1625	15678	6
1625	15679	6
1625	15680	5
1625	15681	5
1625	15682	5

1625	15683	5
1625	15684	5
1625	15685	5
1625	15686	5
1626	57	126
1626	72	709
1626	73	563
1626	154	459
1626	159	957
1626	163	22659
1626	182	123
1626	227	157
1626	237	389
1626	245	142
1626	253	124
1626	366	159
1626	418	297
1626	511	294
1626	730	363
1626	885	94
1626	910	92
1626	963	201
1626	986	199
1626	1130	72
1626	1219	322
1626	1403	90
1626	1412	179
1626	1715	116
1626	1772	73
1626	2040	96

1626	2842	82
1626	2901	74
1626	3107	160
1626	3400	76
1626	3884	105
1626	3892	99
1626	4037	112
1626	4734	155
1626	6137	159
1626	6150	161
1626	6516	123
1626	7054	300
1626	9423	180
1626	10224	303
1626	12050	199
1626	15687	2444
1626	15688	493
1626	15689	477
1626	15690	244
1626	15691	126
1626	15692	117
1626	15693	99
1626	15694	84
1626	15695	73
1627	163 12800	
1627	227 9421	
1627	234 201	
1627	599 8189	
1627	607 285	
1627	706 1440	

1627	707 1462	
1627	733 1025	
1627	735 650	
1627	841 329	
1627	857 196	
1627	868 6336	
1627	917 548	
1627	918 269	
1627	952 265	
1627	959 1090	
1627	1206	187
1627	1244	444
1627	1249	1719
1627	1257	411
1627	1261	527
1627	1337	215
1627	1364	230
1627	1369	2066
1627	1372	410
1627	1412	22809
1627	1415	349
1627	1426	175
1627	1464	265
1627	1513	1607
1627	1806	317
1627	2013	173
1627	2265	278
1627	2343	666
1627	2347	408
1627	2784	930

1627	2790	201
1627	2854	280
1627	2871	3247
1627	3458	267
1627	3648	303
1627	3774	292
1627	4251	447
1627	5175	209
1627	5485	432
1627	5844	1457
1627	6165	242
1627	7048	254
1627	8794	231
1627	9928	179
1628	55	568
1628	89	1411
1628	288	3296
1628	289	11552
1628	291	197
1628	292	3097
1628	299	970
1628	300	1663
1628	302	396
1628	306	511
1628	314	398
1628	317	402
1628	323	355
1628	327	645
1628	328	203
1628	333	641

1628	344 776	
1628	349 584	
1628	352 258	
1628	458 186	
1628	461 950	
1628	464 383	
1628	466 3042	
1628	485 325	
1628	543 18907	
1628	548 771	
1628	679 490	
1628	686 427	
1628	690 349	
1628	697 254	
1628	701 238	
1628	704 374	
1628	797 403	
1628	1032	272
1628	1034	390
1628	1037	588
1628	1458	749
1628	1461	7607
1628	1934	357
1628	2083	329
1628	2521	1032
1628	2543	512
1628	3319	537
1628	3404	381
1628	4814	248
1628	5032	261

1628	5149	254
1628	7781	219
1628	11572	292
1628	15696	1054
1629	63	10156
1629	65	207
1629	154	228
1629	157	345
1629	161	316
1629	163	5204
1629	176	178
1629	190	262
1629	229	160
1629	377	1186
1629	378	3006
1629	506	4262
1629	511	1305
1629	592	712
1629	727	339
1629	729	454
1629	952	227
1629	963	387
1629	978	181
1629	1109	729
1629	1112	572
1629	1132	893
1629	1601	204
1629	1705	1370
1629	1793	1358
1629	1854	631

1629	2745	1349
1629	3876	177
1629	4037	411
1629	4126	16990
1629	4351	169
1629	4880	361
1629	4988	1918
1629	4990	567
1629	8853	1023
1629	8854	194
1629	15697	2565
1629	15698	1374
1629	15699	891
1629	15700	765
1629	15701	486
1629	15702	451
1629	15703	385
1629	15704	380
1629	15705	248
1629	15706	237
1629	15707	203
1629	15708	183
1629	15709	182
1629	15710	164
1630	64	378
1630	81	163
1630	88	283
1630	155	169
1630	170	138
1630	316	203

1630	423 218	
1630	445 142	
1630	511 229	
1630	604 831	
1630	614 296	
1630	703 143	
1630	854 491	
1630	858 277	
1630	986 138	
1630	1131	142
1630	1504	344
1630	1510	670
1630	1633	188
1630	1643	1759
1630	1718	198
1630	1974	152
1630	2277	160
1630	2544	265
1630	2610	365
1630	3257	224
1630	3884	141
1630	6348	197
1630	7079	331
1630	7129	144
1630	13709	160
1630	14124	181
1630	15711	702
1630	15712	391
1630	15713	347
1630	15714	296

1630	15715	292
1630	15716	261
1630	15717	235
1630	15718	221
1630	15719	189
1630	15720	178
1630	15721	178
1630	15722	174
1630	15723	174
1630	15724	168
1630	15725	164
1630	15726	159
1630	15727	158
1630	15728	141
1632	89	1987
1632	161	3566
1632	265	150
1632	288	1327
1632	289	497
1632	290	415
1632	300	846
1632	304	985
1632	306	183
1632	308	86
1632	325	136
1632	327	351
1632	328	98
1632	333	92
1632	386	140
1632	388	595

1632	455 152	
1632	458 4392	
1632	461 2097	
1632	466 852	
1632	468 82	
1632	531 81	
1632	543 240	
1632	802 841	
1632	1042	1069
1632	1045	1002
1632	1429	405
1632	1455	77
1632	1659	713
1632	2080	697
1632	2545	117
1632	2548	213
1632	2939	2050
1632	3378	204
1632	4474	231
1632	4562	1870
1632	4811	73
1632	4850	275
1632	5749	167
1632	5988	217
1632	6308	258
1632	9165	244
1632	9169	1120
1632	11249	84
1632	11890	302
1632	15737	205

1632	15738	179
1632	15739	148
1632	15740	138
1632	15741	77
1633	55	7953
1633	67	7947
1633	89	629
1633	258	4301
1633	288	338
1633	289	590
1633	292	451
1633	293	61
1633	295	1401
1633	298	238
1633	299	517
1633	300	102
1633	302	126
1633	310	8355
1633	316	185
1633	328	120
1633	340	2273
1633	342	234
1633	349	136
1633	466	640
1633	525	124
1633	535	86
1633	543	98
1633	546	386
1633	644	101
1633	701	393

1633	889	1003
1633	903	100
1633	913	254
1633	1052	66
1633	1464	82
1633	1767	74
1633	1771	89
1633	2083	948
1633	2919	61
1633	3067	86
1633	3316	57
1633	4087	128
1633	5416	136
1633	6376	4288
1633	6721	68
1633	8287	66
1633	9528	92
1633	12044	64
1633	15742	565
1633	15743	190
1633	15744	190
1633	15745	65
1633	15746	65
1633	15747	59
1634	609	2
1634	5665	1
1634	15748	6
1634	15749	1
1635	65	170
1635	70	468

1635	72	168
1635	154	342
1635	163	442
1635	172	191
1635	186	146
1635	190	175
1635	206	1358
1635	220	480
1635	225	330
1635	227	163
1635	228	1366
1635	235	382
1635	424	808
1635	436	141
1635	486	139
1635	511	1083
1635	533	328
1635	614	1242
1635	709	158
1635	734	706
1635	852	209
1635	969	105
1635	1118	406
1635	1164	325
1635	1377	108
1635	1384	147
1635	1510	484
1635	1519	144
1635	1790	576
1635	1795	165

1635	1873	204
1635	1884	327
1635	2469	99
1635	2524	584
1635	2572	362
1635	2580	121
1635	2607	225
1635	2626	149
1635	2775	1235
1635	2783	155
1635	3413	170
1635	3475	656
1635	5423	212
1635	5439	295
1635	9632	127
1635	13888	214
1635	15750	251
1635	15751	119
1636	58	23
1636	65	28
1636	67	40
1636	89	73
1636	184	25
1636	199	52
1636	223	33
1636	230	22
1636	288	56
1636	289	51
1636	295	32
1636	298	21

1636	300 54	
1636	306 127	
1636	310 29	
1636	314 22	
1636	321 28	
1636	327 36	
1636	328 42	
1636	333 31	
1636	344 22	
1636	366 24	
1636	377 39	
1636	455 20	
1636	458 27	
1636	461 41	
1636	464 49	
1636	466 64	
1636	498 31	
1636	532 28	
1636	542 40	
1636	545 39	
1636	546 48	
1636	701 25	
1636	708 27	
1636	1042	23
1636	1045	74
1636	1047	21
1636	1241	36
1636	1246	31
1636	1394	35
1636	1456	23

1636	1485	62
1636	1659	322
1636	2080	100
1636	3200	20
1636	4850	20
1636	5749	28
1636	7833	95
1636	7834	29
1637	58	1083
1637	154	2798
1637	190	1100
1637	195	1214
1637	199	2452
1637	212	910
1637	221	1115
1637	223	2150
1637	225	929
1637	228	1279
1637	416	3858
1637	423	1356
1637	426	1187
1637	433	1641
1637	434	1423
1637	437	1958
1637	440	1466
1637	441	3125
1637	533	1735
1637	614	1476
1637	615	1738
1637	618	1420

1637	962 3605	
1637	1106	2293
1637	1375	910
1637	1390	900
1637	1400	1312
1637	1424	1581
1637	1504	1440
1637	1510	3741
1637	1519	1811
1637	1633	1477
1637	1772	1215
1637	1976	1537
1637	2083	928
1637	2524	2096
1637	2544	2307
1637	2606	1862
1637	3057	1051
1637	3110	1178
1637	3250	1095
1637	3617	1033
1637	3681	1544
1637	4442	1333
1637	5414	893
1637	8592	2908
1637	8830	976
1637	9709	1143
1637	9823	1217
1637	15752	1017
1638	103 77	
1638	632 59	

1638	763 42	
1638	1229	46
1638	1411	76
1638	1418	65
1638	1423	60
1638	1504	47
1638	1708	80
1638	1709	94
1638	1717	58
1638	1736	57
1638	1744	48
1638	2542	51
1638	3417	67
1638	3703	82
1638	3724	57
1638	3805	68
1638	4121	62
1638	4271	44
1638	4790	57
1638	4959	57
1638	6048	133
1638	6151	144
1638	6159	100
1638	6167	55
1638	6759	56
1638	7380	41
1638	9342	48
1638	11482	41
1638	11604	68
1638	11611	44

1638	11784	40
1638	12317	48
1638	13332	68
1638	14931	65
1638	14960	41
1638	15362	51
1638	15753	91
1638	15754	82
1638	15755	68
1638	15756	52
1638	15757	51
1638	15758	50
1638	15759	49
1638	15760	45
1638	15761	43
1638	15762	42
1638	15763	42
1638	15764	41
1639	71	391
1639	155	980
1639	163	5681
1639	176	1814
1639	189	959
1639	192	3084
1639	198	759
1639	221	640
1639	227	3594
1639	230	586
1639	333	641
1639	380	1702

1639	405 1470	
1639	447 695	
1639	462 545	
1639	481 433	
1639	536 1165	
1639	707 1523	
1639	711 969	
1639	712 1101	
1639	717 389	
1639	728 388	
1639	732 387	
1639	917 422	
1639	951 1370	
1639	959 738	
1639	966 467	
1639	1243	1061
1639	1339	422
1639	1358	1656
1639	1372	1565
1639	1464	597
1639	1686	577
1639	1689	1157
1639	1866	372
1639	2023	374
1639	2265	771
1639	2343	631
1639	2440	516
1639	3470	1329
1639	3763	562
1639	3774	3020

1639	4130	535
1639	6282	361
1639	6959	401
1639	7054	935
1639	15765	921
1639	15766	816
1639	15767	533
1639	15768	396
1640	176 32	
1640	181 94	
1640	198 533	
1640	233 38	
1640	234 207	
1640	370 142	
1640	486 66	
1640	488 230	
1640	497 31	
1640	503 43	
1640	504 59	
1640	706 200	
1640	710 89	
1640	724 76	
1640	726 111	
1640	735 76	
1640	841 79	
1640	917 1005	
1640	918 492	
1640	922 465	
1640	923 279	
1640	982 191	

1640	1044	150
1640	1179	64
1640	1249	131
1640	1347	30
1640	1351	546
1640	1358	145
1640	1360	30
1640	1369	180
1640	1372	46
1640	1412	66
1640	1672	69
1640	1984	118
1640	2342	163
1640	2372	39
1640	2595	152
1640	2664	93
1640	2765	46
1640	2794	42
1640	3238	42
1640	3624	48
1640	3628	133
1640	3646	36
1640	4286	48
1640	6259	39
1640	8422	29
1640	15769	121
1640	15770	102
1640	15771	45
1641	57	177
1641	70	215

1641	159	160
1641	227	324
1641	340	174
1641	511	158
1641	572	159
1641	575	428
1641	599	184
1641	627	299
1641	629	212
1641	715	171
1641	718	202
1641	735	326
1641	961	157
1641	986	544
1641	1001	293
1641	1161	295
1641	1244	346
1641	1826	222
1641	1829	180
1641	1833	378
1641	1845	171
1641	1848	177
1641	1892	246
1641	2458	280
1641	2535	168
1641	2629	271
1641	2638	264
1641	3896	261
1641	3992	225
1641	4495	230

1641	5192	169
1641	7123	212
1641	7148	161
1641	7877	249
1641	10134	177
1641	10351	165
1641	10358	290
1641	10363	287
1641	11849	186
1641	13913	378
1641	13915	160
1641	13928	279
1641	15772	242
1641	15773	229
1641	15774	221
1641	15775	219
1641	15776	191
1641	15777	164
1642	56	107
1642	61	120
1642	72	352698
1642	75	142
1642	159	174
1642	187	111
1642	511	318
1642	529	109
1642	1009	207
1642	1072	10947
1642	1079	162
1642	1713	294

1642	2149	155
1642	2824	1982
1642	5871	117
1642	9980	498
1642	11520	212
1642	14675	213
1642	14739	135
1642	14744	307
1642	14750	6747
1642	14752	139
1642	14757	304
1642	14774	259
1642	14780	233
1642	15778	3621
1642	15779	2024
1642	15780	841
1642	15781	827
1642	15782	449
1642	15783	440
1642	15784	349
1642	15785	281
1642	15786	239
1642	15787	231
1642	15788	229
1642	15789	212
1642	15790	201
1642	15791	178
1642	15792	169
1642	15793	158
1642	15794	144

1642	15795	128
1642	15796	127
1642	15797	125
1642	15798	123
1642	15799	122
1642	15800	115
1642	15801	114
1642	15802	114
1643	51	21572
1643	67	399
1643	77	4916
1643	95	1215
1643	155	1923
1643	187	2407
1643	190	304
1643	227	2702
1643	296	498
1643	418	566
1643	679	1834
1643	694	537
1643	728	1163
1643	801	384
1643	906	655
1643	910	615
1643	959	1057
1643	972	491
1643	1001	667
1643	1047	632
1643	1076	988
1643	1414	286

1643	2071	571
1643	2523	438
1643	2556	946
1643	2562	992
1643	3070	664
1643	3217	363
1643	3866	311
1643	4068	338
1643	5000	4558
1643	6790	2918
1643	8676	545
1643	8995	1010
1643	11724	1082
1643	11727	311
1643	12129	3193
1643	13266	380
1643	13415	432
1643	14712	293
1643	15803	2716
1643	15804	1443
1643	15805	1177
1643	15806	643
1643	15807	436
1643	15808	404
1643	15809	354
1643	15810	338
1643	15811	315
1643	15812	310
1644	96 16	
1644	163 6	

1644	212 22	
1644	340 5	
1644	447 14	
1644	575 18	
1644	706 15	
1644	712 7	
1644	732 14	
1644	868 5	
1644	905 7	
1644	930 22	
1644	959 6	
1644	1079	8
1644	1081	22
1644	1244	50
1644	1369	7
1644	1415	19
1644	1639	5
1644	1810	5
1644	2346	27
1644	2624	19
1644	2746	62
1644	2855	9
1644	2857	13
1644	3119	6
1644	3919	34
1644	3934	16
1644	4161	27
1644	5375	31
1644	5403	6
1644	5583	37

1644	7044	34
1644	7101	8
1644	8559	10
1644	8564	7
1644	8807	22
1644	8816	6
1644	10358	5
1644	10626	17
1644	10627	13
1644	13265	6
1644	13995	14
1644	15544	7
1644	15803	9
1644	15813	132
1644	15814	39
1644	15815	10
1644	15816	9
1644	15817	6
1646	86	376
1646	89	765
1646	97	1086
1646	228	943
1646	229	1277
1646	288	1332
1646	289	460
1646	292	526
1646	294	374
1646	295	877
1646	298	330
1646	300	467

1646	301	1146
1646	306	1212
1646	310	373
1646	423	389
1646	430	411
1646	535	774
1646	548	380
1646	575	453
1646	689	1391
1646	690	903
1646	697	1297
1646	889	522
1646	896	483
1646	903	10581
1646	961	1267
1646	1463	725
1646	1511	605
1646	1556	746
1646	1688	406
1646	1767	462
1646	1772	842
1646	1934	2020
1646	1971	1009
1646	2521	411
1646	2544	476
1646	2601	352
1646	2786	792
1646	3057	941
1646	3110	1366
1646	3126	392

1646	3201	1577
1646	3322	350
1646	3371	547
1646	3488	394
1646	3502	677
1646	4252	425
1646	8020	540
1646	12990	383
1647	65	295
1647	89	469
1647	157	116
1647	161	295
1647	190	132
1647	257	123
1647	288	335
1647	289	1589
1647	292	637
1647	300	227
1647	318	157
1647	321	759
1647	325	864
1647	328	483
1647	333	137
1647	344	329
1647	349	305
1647	377	355
1647	456	290
1647	458	186
1647	461	1580
1647	464	482

1647	486	258
1647	491	248
1647	493	123
1647	498	148
1647	681	134
1647	701	143
1647	793	432
1647	907	341
1647	908	328
1647	912	999
1647	966	169
1647	1054	209
1647	1075	716
1647	1186	379
1647	1388	150
1647	1459	116
1647	1597	107
1647	1854	147
1647	1975	161
1647	2179	103
1647	2801	718
1647	2813	106
1647	3171	313
1647	3570	126
1647	4923	216
1647	5746	151
1647	13147	110
1647	13546	800
1648	55	1991
1648	56	275

1648	67	6428
1648	89	1568
1648	155	237
1648	227	427
1648	257	76
1648	265	59
1648	288	141
1648	289	60
1648	292	346
1648	295	118
1648	299	24
1648	300	103
1648	306	57
1648	418	194
1648	437	343
1648	441	29
1648	444	46
1648	454	24
1648	466	61
1648	485	78
1648	511	9
1648	679	816
1648	959	45
1648	1034	15
1648	1098	84
1648	1244	294
1648	1384	15
1648	1456	20
1648	1464	160
1648	2533	9

1648	2634	252
1648	3209	14
1648	3411	138
1648	3763	77
1648	4575	16
1648	5150	64
1648	5427	45
1648	6756	17
1648	8027	25
1648	12013	13
1648	15823	38
1648	15824	13
1648	15825	10
1648	15826	10
1648	15827	10
1648	15828	10
1648	15829	9
1648	15830	8
1649	7	1582
1649	89	2628
1649	173	642
1649	189	1005
1649	288	808
1649	289	1002
1649	291	638
1649	292	1213
1649	294	644
1649	295	1597
1649	300	3142
1649	302	2489

1649	310 917	
1649	326 574	
1649	352 457	
1649	377 600	
1649	378 4631	
1649	405 691	
1649	466 1472	
1649	486 7218	
1649	488 1034	
1649	497 480	
1649	498 606	
1649	499 474	
1649	500 1040	
1649	504 1155	
1649	518 514	
1649	544 2104	
1649	680 1146	
1649	701 423	
1649	704 1180	
1649	779 439	
1649	1032	919
1649	1034	891
1649	1042	453
1649	1185	655
1649	1186	534
1649	1189	511
1649	1678	1565
1649	1854	1937
1649	2956	425
1649	8356	439

1649	10733	718
1649	10735	2610
1649	15831	1166
1649	15832	621
1649	15833	593
1649	15834	592
1649	15835	508
1649	15836	447
1650	159 1585	
1650	227 1535	
1650	234 898	
1650	333 3420	
1650	383 1393	
1650	418 823	
1650	424 1110	
1650	441 3651	
1650	859 798	
1650	863 1368	
1650	868 710	
1650	1121	851
1650	1412	1169
1650	1418	1479
1650	1504	3852
1650	1513	4074
1650	1796	1982
1650	1888	805
1650	1957	696
1650	2034	817
1650	2542	711
1650	3019	2149

1650	3053	1222
1650	3364	842
1650	3791	734
1650	4092	1991
1650	4188	2239
1650	4194	1072
1650	4225	993
1650	4821	5062
1650	5549	2226
1650	5785	918
1650	6146	955
1650	6824	938
1650	7008	1239
1650	7260	680
1650	7374	1802
1650	7375	914
1650	8933	2637
1650	8945	931
1650	10111	918
1650	10565	1085
1650	10572	753
1650	10753	1378
1650	12145	878
1650	13854	1945
1650	15837	1406
1650	15838	1056
1650	15839	811
1650	15840	725
1652	67	299
1652	89	112

1652	155 140
1652	157 233
1652	190 70
1652	207 157
1652	220 116
1652	230 92
1652	250 117
1652	257 243
1652	289 2274
1652	292 180
1652	295 198
1652	300 104
1652	321 277
1652	329 276
1652	331 121
1652	349 181
1652	355 81
1652	377 220
1652	455 4141
1652	456 1201
1652	457 183
1652	459 181
1652	462 60
1652	469 339
1652	486 97
1652	489 88
1652	498 139
1652	511 70
1652	517 90
1652	910 156

1652	913	111
1652	951	69
1652	1044	109
1652	1099	118
1652	1244	61
1652	1377	108
1652	1406	252
1652	1464	134
1652	2179	67
1652	2523	72
1652	3568	158
1652	3712	250
1652	4920	64
1652	4927	63
1652	5746	78
1652	12651	133
1652	15847	88
1652	15848	79
1653	30	148
1653	67	715
1653	72	3315
1653	77	171
1653	89	521
1653	99	195
1653	159	528
1653	172	354
1653	173	191
1653	229	178
1653	233	704
1653	295	151

1653	423 264	
1653	425 373	
1653	429 429	
1653	441 475	
1653	599 2789	
1653	679 455	
1653	689 554	
1653	690 256	
1653	707 186	
1653	858 155	
1653	868 174	
1653	910 175	
1653	959 197	
1653	1001	163
1653	1014	147
1653	1061	238
1653	1062	187
1653	1072	856
1653	1073	154
1653	1075	494
1653	1079	196
1653	1083	227
1653	1097	1905
1653	1098	1107
1653	1103	202
1653	1119	689
1653	1167	170
1653	2478	161
1653	2491	141
1653	2493	197

1653	2521	569
1653	2531	990
1653	3291	188
1653	4923	184
1653	5237	248
1653	6527	145
1653	8229	199
1653	8231	226
1654	166 149	
1654	203 204	
1654	429 96	
1654	434 171	
1654	615 164	
1654	618 635	
1654	859 225	
1654	1622	183
1654	1901	282
1654	1947	164
1654	2416	305
1654	2424	511
1654	2640	127
1654	4324	1166
1654	5027	119
1654	5229	427
1654	5414	260
1654	5790	144
1654	6833	229
1654	9487	103
1654	12220	104
1654	12496	92

1654	14385	93
1654	15124	782
1654	15365	523
1654	15849	582
1654	15850	554
1654	15851	382
1654	15852	377
1654	15853	369
1654	15854	305
1654	15855	294
1654	15856	285
1654	15857	270
1654	15858	263
1654	15859	228
1654	15860	191
1654	15861	188
1654	15862	180
1654	15863	175
1654	15864	164
1654	15865	151
1654	15866	145
1654	15867	126
1654	15868	125
1654	15869	124
1654	15870	108
1654	15871	106
1654	15872	101
1654	15873	93
1655	81	283
1655	152	1093

1655	154 466	
1655	163 366	
1655	183 529	
1655	186 197	
1655	188 1644	
1655	189 193	
1655	204 251	
1655	215 410	
1655	418 419	
1655	506 863	
1655	706 1206	
1655	854 288	
1655	920 328	
1655	931 293	
1655	944 165	
1655	952 427	
1655	957 181	
1655	964 342	
1655	1134	214
1655	1170	288
1655	1343	170
1655	1412	381
1655	1522	184
1655	1660	247
1655	2003	281
1655	2005	167
1655	2787	354
1655	2797	351
1655	2834	230
1655	2837	494

1655	3405	284
1655	3620	211
1655	4690	274
1655	6765	703
1655	8658	917
1655	9334	312
1655	9822	566
1655	11283	364
1655	11502	444
1655	12909	347
1655	13129	244
1655	14135	197
1655	15874	254
1655	15875	252
1655	15876	221
1655	15877	212
1655	15878	206
1655	15879	190
1656	56	75
1656	65	139
1656	88	521
1656	154	674
1656	166	1157
1656	170	104
1656	173	356
1656	180	167
1656	182	379
1656	199	156
1656	203	2199
1656	227	1155

1656	293	75
1656	298	507
1656	418	76
1656	425	337
1656	429	521
1656	441	425
1656	526	202
1656	530	89
1656	533	1901
1656	546	148
1656	548	163
1656	689	557
1656	691	546
1656	703	876
1656	716	169
1656	757	513
1656	903	390
1656	980	226
1656	1048	238
1656	1090	268
1656	1195	70
1656	1206	78
1656	1398	429
1656	1400	756
1656	1509	81
1656	1513	387
1656	1672	70
1656	2137	113
1656	2537	129
1656	2618	85

1656	2786	249
1656	2839	168
1656	3057	366
1656	3898	193
1656	4118	131
1656	7077	144
1656	7505	195
1656	9701	793
1657	107 287	
1657	163 417	
1657	206 454	
1657	238 224	
1657	511 318	
1657	602 205	
1657	605 316	
1657	606 264	
1657	715 195	
1657	748 246	
1657	756 348	
1657	1098	361
1657	1412	249
1657	1424	243
1657	1835	234
1657	1969	168
1657	2605	310
1657	2668	220
1657	2688	177
1657	3225	227
1657	3413	390
1657	3440	297

1657	3619	180
1657	3992	171
1657	3995	335
1657	5312	242
1657	6581	350
1657	6762	171
1657	7146	285
1657	7700	176
1657	8042	194
1657	9361	194
1657	9882	170
1657	11801	219
1657	13033	207
1657	13087	168
1657	13891	179
1657	14279	220
1657	15880	352
1657	15881	301
1657	15882	277
1657	15883	245
1657	15884	235
1657	15885	228
1657	15886	206
1657	15887	191
1657	15888	184
1657	15889	183
1657	15890	179
1657	15891	172
1658	56	200
1658	190	193

1658	228 251	
1658	229 171	
1658	318 1601	
1658	333 2366	
1658	344 811	
1658	481 358	
1658	530 202	
1658	646 494	
1658	680 199	
1658	681 314	
1658	701 266	
1658	757 214	
1658	790 226	
1658	971 984	
1658	980 399	
1658	1048	2424
1658	1116	202
1658	1185	458
1658	1400	245
1658	1670	169
1658	2462	309
1658	3187	188
1658	3193	468
1658	3200	220
1658	3290	159
1658	4291	335
1658	5137	481
1658	5140	479
1658	6529	219
1658	10900	475

1658	10901	228
1658	11084	555
1658	12553	726
1658	12555	475
1658	13739	1631
1658	15892	1146
1658	15893	536
1658	15894	407
1658	15895	293
1658	15896	292
1658	15897	291
1658	15898	283
1658	15899	266
1658	15900	211
1658	15901	202
1658	15902	201
1658	15903	176
1658	15904	159
1659	55	212
1659	67	821
1659	89	5220
1659	289	333
1659	292	1219
1659	293	181
1659	295	100
1659	298	226
1659	300	199
1659	325	198
1659	328	134
1659	352	303

1659	423	1329
1659	466	1002
1659	470	106
1659	528	476
1659	637	583
1659	652	163
1659	701	232
1659	787	185
1659	788	635
1659	790	512
1659	792	144
1659	795	399
1659	802	159
1659	903	115
1659	1001	107
1659	1392	109
1659	1446	154
1659	1609	220
1659	2390	177
1659	2396	258
1659	4260	297
1659	4702	151
1659	6318	485
1659	6856	248
1659	7502	255
1659	12576	230
1659	14474	124
1659	15288	108
1659	15905	639
1659	15906	406

1659	15907	324
1659	15908	287
1659	15909	248
1659	15910	195
1659	15911	164
1659	15912	115
1659	15913	110
1659	15914	109
1660	163	159
1660	220	112
1660	234	145
1660	512	164
1660	559	118
1660	562	143
1660	605	113
1660	631	182
1660	735	315
1660	841	127
1660	859	136
1660	868	188
1660	882	123
1660	917	180
1660	959	123
1660	961	134
1660	1119	119
1660	1202	119
1660	1221	148
1660	1360	259
1660	1364	114
1660	1369	181

1660	1412	150
1660	1513	182
1660	1533	118
1660	1889	143
1660	1979	156
1660	2026	165
1660	2343	122
1660	2375	271
1660	2473	112
1660	2475	207
1660	2491	195
1660	2493	188
1660	2698	170
1660	3417	163
1660	3430	113
1660	3443	171
1660	3780	118
1660	4356	131
1660	4359	147
1660	4520	110
1660	4662	138
1660	6126	116
1660	10672	145
1660	11042	133
1660	15915	187
1660	15916	139
1660	15917	122
1660	15918	114
1661	190 341	
1661	199 228	

1661	207 366	
1661	217 359	
1661	227 232	
1661	228 449	
1661	229 158	
1661	291 270	
1661	293 251	
1661	298 214	
1661	300 608	
1661	316 322	
1661	333 611	
1661	344 923	
1661	403 502	
1661	420 211	
1661	464 355	
1661	498 1349	
1661	538 202	
1661	542 155	
1661	546 173	
1661	548 558	
1661	679 159	
1661	681 2196	
1661	683 628	
1661	687 185	
1661	691 183	
1661	696 155	
1661	704 1209	
1661	1047	339
1661	1048	202
1661	1090	144

1661	1106	208
1661	1246	146
1661	1400	201
1661	1470	226
1661	1492	161
1661	1502	159
1661	1632	376
1661	1976	369
1661	2121	238
1661	2277	222
1661	2407	166
1661	2977	321
1661	3373	149
1661	3400	211
1661	3461	145
1661	3730	138
1661	5978	156
1661	9079	151
1662	67	168
1662	69	838
1662	157	464
1662	227	233
1662	241	220
1662	249	244
1662	251	317
1662	257	794
1662	260	248
1662	267	275
1662	278	780
1662	285	187

1662	301	234
1662	331	856
1662	475	278
1662	576	899
1662	617	593
1662	701	181
1662	868	223
1662	903	342
1662	1076	164
1662	1122	168
1662	1402	169
1662	1427	824
1662	1613	285
1662	1620	646
1662	2083	166
1662	2220	197
1662	2222	745
1662	2226	242
1662	2907	365
1662	2919	261
1662	3317	210
1662	3488	320
1662	4710	604
1662	6376	195
1662	6389	709
1662	8173	216
1662	8991	549
1662	9907	704
1662	10997	172
1662	11070	532

1662	11078	547
1662	12406	185
1662	15919	580
1662	15920	500
1662	15921	336
1662	15922	322
1662	15923	164
1662	15924	155
1663	55	503
1663	67	845
1663	89	281
1663	157	1625
1663	180	332
1663	251	647
1663	257	1738
1663	265	5864
1663	288	222
1663	289	1485
1663	291	292
1663	292	566
1663	294	582
1663	295	1107
1663	296	147
1663	300	225
1663	306	119
1663	318	261
1663	321	159
1663	325	391
1663	333	1125
1663	344	97

1663	349 160	
1663	360 116	
1663	455 213	
1663	461 224	
1663	466 120	
1663	479 1001	
1663	641 93	
1663	701 321	
1663	793 177	
1663	798 126	
1663	908 312	
1663	909 359	
1663	913 363	
1663	951 115	
1663	1936	125
1663	2021	478
1663	2022	1371
1663	2024	754
1663	2523	1300
1663	2801	99
1663	3171	402
1663	3173	174
1663	3332	428
1663	3393	420
1663	4941	146
1663	5425	158
1663	15925	234
1663	15926	191
1664	157 115	
1664	198 386	

1664	207 199	
1664	295 214	
1664	300 114	
1664	371 609	
1664	377 160	
1664	486 146	
1664	498 227829	
1664	499 776	
1664	537 199	
1664	687 236	
1664	693 129	
1664	834 342	
1664	920 117	
1664	924 255	
1664	1040	118
1664	1041	220
1664	1045	110
1664	1180	703
1664	1201	117
1664	1389	152
1664	1657	531
1664	1659	2164
1664	1681	795
1664	1809	384
1664	1855	158
1664	2618	147
1664	2661	362
1664	2666	111
1664	2839	243
1664	2987	461

1664	3369	190
1664	4177	600
1664	4828	450
1664	5427	210
1664	6000	115
1664	7252	205
1664	8732	274
1664	9153	151
1664	12104	324
1664	12661	161
1664	13431	110
1664	13782	246
1664	15927	8320
1664	15928	784
1664	15929	310
1664	15930	229
1664	15931	161
1664	15932	118
1665	190 1	
1665	317 1	
1665	371 1	
1665	377 1	
1665	472 1	
1665	481 1	
1665	488 2	
1665	510 1	
1665	978 1	
1665	1450	1
1665	1586	1
1665	1854	1

1665	2580	1
1665	2602	1
1665	2610	1
1665	2613	1
1665	3570	2
1665	3758	1
1665	4491	1
1665	5731	1
1665	5734	1
1665	6618	1
1665	13275	1
1665	15933	2
1665	15934	1
1665	15935	1
1665	15936	1
1665	15937	1
1665	15938	1
1665	15939	1
1665	15940	1
1665	15941	1
1665	15942	1
1665	15943	1
1665	15944	1
1665	15945	1
1665	15946	1
1666	55	4315
1666	67	1240
1666	89	2942
1666	161	1370
1666	257	1811

1666	288 6633
1666	289 29821
1666	290 815
1666	291 2658
1666	292 2386
1666	293 12195
1666	294 987
1666	296 2962
1666	297 2318
1666	298 3799
1666	299 1877
1666	300 3972
1666	305 1031
1666	306 764
1666	307 1070
1666	308 1273
1666	311 899
1666	318 1907
1666	320 1693
1666	321 914
1666	323 2268
1666	324 835
1666	325 778
1666	326 1356
1666	332 780
1666	333 2188
1666	349 882
1666	403 997
1666	461 2044
1666	462 1499

1666	466	7185
1666	471	977
1666	498	2024
1666	525	1239
1666	528	789
1666	536	817
1666	540	935
1666	543	824
1666	701	1160
1666	908	1753
1666	1059	779
1666	1246	12920
1666	1684	1921
1666	3319	850
1666	3320	1953
1667	65	10
1667	71	587
1667	154	11
1667	203	9
1667	221	9
1667	604	16
1667	715	13
1667	972	11
1667	1090	9
1667	1106	17
1667	1798	13
1667	2524	9
1667	3046	333
1667	3047	11
1667	3056	10

1667	3057	23
1667	4037	66
1667	4297	14
1667	4298	22
1667	4299	10
1667	4385	34
1667	4609	29
1667	5030	14
1667	5923	11
1667	7390	9
1667	8002	13
1667	8015	12
1667	9777	14
1667	11147	9
1667	11722	10
1667	12773	10
1667	13154	9
1667	15947	46
1667	15948	21
1667	15949	20
1667	15950	17
1667	15951	13
1667	15952	12
1667	15953	12
1667	15954	11
1667	15955	11
1667	15956	11
1667	15957	11
1667	15958	10
1667	15959	10

1667	15960	10
1667	15961	9
1667	15962	9
1667	15963	9
1667	15964	9
1669	64	71
1669	65	76
1669	70	84
1669	71	59
1669	72	97
1669	162	309
1669	186	284
1669	234	120
1669	378	56
1669	504	89
1669	506	84
1669	517	63
1669	533	39
1669	602	56
1669	707	150
1669	726	143
1669	930	50
1669	937	47
1669	957	53
1669	1104	111
1669	1109	45
1669	1178	100
1669	1226	42
1669	2014	73
1669	2337	79

1669	2338	56
1669	2339	75
1669	2644	77
1669	3418	45
1669	4025	49
1669	4113	43
1669	4365	89
1669	7047	42
1669	7261	41
1669	7509	46
1669	8420	65
1669	8512	40
1669	13633	74
1669	14248	48
1669	14945	39
1669	15814	86
1669	15965	212
1669	15966	170
1669	15967	81
1669	15968	78
1669	15969	57
1669	15970	48
1669	15971	45
1669	15972	41
1669	15973	40
1670	377	71
1670	408	212
1670	472	122
1670	488	153
1670	490	109

1670	496 113	
1670	497 64	
1670	966 84	
1670	1854	184
1670	1874	67
1670	1905	105
1670	1985	89
1670	2206	78
1670	2352	127
1670	2794	157
1670	2843	128
1670	2941	205
1670	2956	83
1670	3051	78
1670	3084	62
1670	3085	180
1670	3094	54
1670	3563	97
1670	3758	182
1670	3859	111
1670	3928	191
1670	3946	71
1670	4179	108
1670	4368	69
1670	4428	55
1670	4737	69
1670	4760	59
1670	5217	168
1670	6249	181
1670	6251	133

1670	7775	123
1670	7947	158
1670	10069	87
1670	10597	112
1670	11018	93
1670	12788	64
1670	15974	612
1670	15975	152
1670	15976	144
1670	15977	94
1670	15978	90
1670	15979	84
1670	15980	81
1670	15981	73
1670	15982	70
1671	176 201	
1671	562 199	
1671	706 448	
1671	707 1018	
1671	732 690	
1671	841 1654	
1671	918 497	
1671	947 571	
1671	954 635	
1671	1206	387
1671	1261	703
1671	1274	216
1671	1358	429
1671	1360	1021
1671	1369	36365

1671	1372	1589
1671	1412	223
1671	1522	201
1671	1527	739
1671	1531	442
1671	1533	208
1671	1814	201
1671	2013	277
1671	2834	1351
1671	3011	2032
1671	3470	416
1671	3774	598
1671	4124	979
1671	4273	198
1671	5172	291
1671	5296	458
1671	5600	407
1671	6362	235
1671	7047	1130
1671	7331	389
1671	7795	374
1671	7796	229
1671	7798	275
1671	7800	259
1671	10620	1017
1671	10764	265
1671	13123	383
1671	15983	467
1671	15984	415
1671	15985	408

1671	15986	343
1671	15987	254
1671	15988	222
1671	15989	196
1671	15990	195
1672	72	4540
1672	81	234
1672	154	201
1672	173	244
1672	196	249
1672	197	398
1672	206	323
1672	208	114
1672	212	157
1672	215	557
1672	228	168
1672	231	177
1672	238	160
1672	416	104
1672	420	604
1672	428	105
1672	441	969
1672	533	129
1672	703	176
1672	735	196
1672	858	640
1672	863	137
1672	867	221
1672	868	889
1672	875	146

1672	1072	118
1672	1075	447
1672	1097	150
1672	1098	118
1672	1164	173
1672	1167	168
1672	1504	184
1672	1976	162
1672	2821	225
1672	3280	105
1672	3371	106
1672	3886	462
1672	4051	175
1672	5879	149
1672	5913	119
1672	6374	131
1672	7324	127
1672	8678	184
1672	10105	237
1672	13167	106
1672	13345	99
1672	14435	156
1672	15991	186
1672	15992	132
1672	15993	99
1674	53	116
1674	55	64
1674	65	77
1674	72	80
1674	81	78

1674	88	79
1674	89	104
1674	173	338
1674	174	159
1674	190	648
1674	196	64
1674	198	57
1674	207	260
1674	209	124
1674	226	94
1674	229	247
1674	256	117
1674	289	240
1674	298	240
1674	324	246
1674	333	75
1674	421	71
1674	424	276
1674	425	184
1674	432	213
1674	453	42
1674	461	112
1674	472	65
1674	532	214
1674	533	72
1674	538	68
1674	540	548
1674	591	55
1674	680	177
1674	701	93

1674	903 78	
1674	1104	97
1674	1116	173
1674	1246	196
1674	1512	364
1674	1673	106
1674	1873	47
1674	2018	58
1674	3057	91
1674	3200	113
1674	3462	54
1674	3739	73
1674	11249	44
1674	15994	189
1674	15995	157
1675	428 16	
1675	461 5236	
1675	498 115	
1675	687 118	
1675	793 2880	
1675	1040	127
1675	1043	110
1675	1046	108
1675	1191	29
1675	1193	106
1675	1866	117
1675	6773	121
1675	7704	1
1676	52 125	
1676	65 240	

1676	66	475
1676	88	126
1676	154	238
1676	198	210
1676	207	223
1676	220	995
1676	227	140
1676	233	238
1676	234	287
1676	425	138
1676	441	326
1676	604	342
1676	614	124
1676	755	598
1676	858	402
1676	865	192
1676	868	464
1676	959	177
1676	964	331
1676	977	171
1676	1055	489
1676	1090	131
1676	1249	273
1676	1790	440
1676	1978	454
1676	1984	308
1676	1987	352
1676	2616	455
1676	3057	127
1676	3237	915

1676	3627	293
1676	3640	377
1676	3648	256
1676	3685	207
1676	4569	240
1676	4570	154
1676	5847	159
1676	6724	1450
1676	7446	124
1676	8068	181
1676	11756	139
1676	11760	294
1676	13026	377
1676	15996	330
1676	15997	206
1676	15998	195
1676	15999	134
1676	16000	127
1677	71	245
1677	170	276
1677	182	342
1677	217	1862
1677	291	423
1677	293	1333
1677	331	471
1677	377	703
1677	496	1380
1677	499	281
1677	510	629
1677	512	3813

1677	537 267	
1677	605 1471	
1677	1200	550
1677	1418	245
1677	1505	401
1677	1613	557
1677	1863	486
1677	1874	624
1677	2608	1683
1677	2820	264
1677	2829	284
1677	2832	353
1677	2923	334
1677	3462	413
1677	4179	1262
1677	4225	283
1677	4233	455
1677	4617	272
1677	4888	450
1677	4947	507
1677	4994	1376
1677	5716	328
1677	6191	523
1677	6436	368
1677	9672	441
1677	9788	307
1677	9963	452
1677	10035	290
1677	11265	321
1677	11358	392

1677	16001	952
1677	16002	834
1677	16003	691
1677	16004	406
1677	16005	372
1677	16006	321
1677	16007	318
1677	16008	273
1678	67	61
1678	89	626
1678	234	60
1678	288	134
1678	289	890
1678	292	382
1678	295	252
1678	300	142
1678	302	72
1678	306	61
1678	318	46
1678	325	58
1678	328	60
1678	333	204
1678	344	58
1678	349	53
1678	371	74
1678	377	119
1678	461	96
1678	464	121
1678	466	113
1678	475	51

1678	486	73
1678	498	92
1678	523	111
1678	542	66
1678	646	110
1678	679	1239
1678	681	78
1678	686	117
1678	701	49
1678	704	59
1678	790	46
1678	802	71
1678	1045	299
1678	1429	53
1678	2020	48
1678	2080	232
1678	2548	100
1678	2984	47
1678	2987	66
1678	3074	54
1678	3332	64
1678	3393	46
1678	5749	108
1678	6132	102
1678	7832	133
1678	7834	72
1678	11158	52
1678	16009	90
1679	2	161
1679	16	131

1679	18	830
1679	173	272
1679	190	828
1679	229	148
1679	377	227
1679	486	451
1679	504	198
1679	516	706
1679	768	728
1679	959	187
1679	1075	422
1679	1090	178
1679	1091	121
1679	1191	139
1679	1298	316
1679	1303	277
1679	2747	313
1679	3087	805
1679	3190	137
1679	3367	373
1679	3546	274
1679	3769	242
1679	4502	615
1679	4609	278
1679	5079	177
1679	6008	142
1679	6439	182
1679	7078	1290
1679	9636	1236
1679	10430	577

1679	10859	1511
1679	10870	190
1679	11938	458
1679	13180	168
1679	15569	268
1679	15570	355
1679	16010	915
1679	16011	304
1679	16012	276
1679	16013	259
1679	16014	210
1679	16015	159
1679	16016	146
1679	16017	136
1679	16018	134
1679	16019	128
1679	16020	125
1679	16021	122
1680	71	850
1680	159	227
1680	163	409
1680	196	792
1680	221	384
1680	227	998
1680	506	789
1680	538	229
1680	604	1953
1680	697	420
1680	961	502
1680	1001	244

1680	1424	331
1680	1908	250
1680	2006	414
1680	2277	449
1680	2300	3670
1680	2320	266
1680	2330	290
1680	2338	281
1680	2339	439
1680	2616	574
1680	3405	401
1680	3417	246
1680	4447	439
1680	4620	362
1680	6930	323
1680	6945	1058
1680	6947	427
1680	6948	648
1680	7157	309
1680	7261	400
1680	7352	334
1680	8444	769
1680	8454	234
1680	8716	753
1680	8962	276
1680	11438	330
1680	12085	267
1680	13888	279
1680	15537	585
1680	16022	580

1680	16023	470
1680	16024	445
1680	16025	415
1680	16026	372
1680	16027	306
1680	16028	293
1680	16029	271
1680	16030	226
1682	65	2972
1682	154	1035
1682	182	1126
1682	190	625
1682	191	884
1682	217	2596
1682	228	1074
1682	229	666
1682	289	690
1682	291	1680
1682	300	877
1682	333	2094
1682	342	686
1682	344	952
1682	378	5435
1682	430	1125
1682	439	1598
1682	466	818
1682	470	2321
1682	486	694
1682	498	15423
1682	499	1604

1682	680 1492	
1682	687 702	
1682	898 1180	
1682	1038	2126
1682	1043	1089
1682	1045	1928
1682	1183	1642
1682	1189	630
1682	1193	875
1682	1195	857
1682	1201	1241
1682	1375	1464
1682	1377	1481
1682	1378	779
1682	2425	626
1682	2608	2788
1682	2653	918
1682	2839	1751
1682	2840	2670
1682	3369	668
1682	4177	752
1682	4223	630
1682	6612	1273
1682	7723	1169
1682	7832	981
1682	7834	1042
1682	8710	806
1682	16002	639
1683	154 461	
1683	163 327	

1683	173 380	
1683	190 354	
1683	195 360	
1683	204 306	
1683	207 327	
1683	217 1547	
1683	227 835	
1683	229 289	
1683	261 434	
1683	288 341	
1683	333 425	
1683	366 495	
1683	418 1572	
1683	438 1458	
1683	439 1240	
1683	536 326	
1683	599 361	
1683	632 653	
1683	680 277	
1683	683 697	
1683	714 2145	
1683	903 320	
1683	1045	305
1683	1109	932
1683	1116	418
1683	1189	288
1683	1244	327
1683	1246	331
1683	1775	611
1683	1866	924

1683	3110	293
1683	3200	313
1683	3384	860
1683	3743	411
1683	4466	552
1683	4616	313
1683	4709	773
1683	4716	562
1683	4818	494
1683	4828	485
1683	6600	542
1683	8504	602
1683	10418	445
1683	11460	505
1683	14645	395
1683	16039	717
1683	16040	576
1683	16041	441
1684	65	46
1684	89	19
1684	182	22
1684	184	4
1684	190	2
1684	288	41
1684	289	413
1684	295	9
1684	300	24
1684	321	7
1684	329	4
1684	331	5

1684	342 4	
1684	344 1018	
1684	349 26	
1684	412 2	
1684	455 27287	
1684	457 32	
1684	465 6	
1684	469 203	
1684	481 51	
1684	498 768	
1684	519 4	
1684	538 8	
1684	646 5	
1684	790 5	
1684	966 28	
1684	1045	4
1684	1243	3
1684	1246	30
1684	1249	2
1684	1375	7
1684	1673	67
1684	2080	46
1684	2523	2
1684	2986	36
1684	3176	2
1684	3217	4
1684	3373	5
1684	3482	13
1684	3568	549
1684	4753	2

1684	9514	8
1684	16042	27
1684	16043	8
1684	16044	5
1684	16045	4
1684	16046	4
1684	16047	3
1684	16048	2
1685	56	80
1685	67	72
1685	89	96
1685	154	87
1685	159	56
1685	173	1803
1685	190	125
1685	207	41
1685	217	136
1685	220	768
1685	225	41
1685	226	120
1685	227	57
1685	228	64
1685	234	42
1685	289	135
1685	316	63
1685	329	38
1685	424	62
1685	425	180
1685	429	71
1685	432	68

1685	436 52	
1685	453 51	
1685	455 549	
1685	469 62	
1685	539 43	
1685	599 38	
1685	683 34	
1685	854 33	
1685	1044	63
1685	1047	36
1685	1062	34
1685	1090	57
1685	1104	44
1685	1116	1524
1685	1383	41
1685	1410	40
1685	1632	106
1685	1799	41
1685	2531	36
1685	3200	100
1685	3732	47
1685	4732	53
1685	5439	39
1685	6724	99
1685	6869	43
1685	12651	58
1685	16049	59
1685	16050	46
1686	67 1122	
1686	89 457	

1686	172 1824
1686	182 2549
1686	207 720
1686	209 659
1686	227 1803
1686	229 4263
1686	298 343
1686	300 2416
1686	302 371
1686	324 695
1686	329 1126
1686	331 549
1686	342 366
1686	344 487
1686	377 839
1686	412 3088
1686	424 347
1686	438 1551
1686	464 367
1686	486 1157
1686	498 1016
1686	501 821
1686	536 451
1686	538 428
1686	540 341
1686	679 1899
1686	683 719
1686	689 397
1686	693 467
1686	714 1260

1686	716	1387
1686	969	1300
1686	989	1910
1686	1043	948
1686	1046	455
1686	1047	6788
1686	1052	749
1686	1116	394
1686	1201	367
1686	1400	1208
1686	2986	435
1686	3097	1194
1686	3171	457
1686	3859	779
1686	4514	472
1686	4941	363
1686	5099	412
1686	7750	998
1687	89	2837
1687	161	124
1687	288	36970
1687	289	537
1687	292	3572
1687	295	166
1687	300	8171
1687	302	413
1687	306	612
1687	328	369
1687	331	430
1687	333	175

1687	344 232	
1687	349 265	
1687	355 131	
1687	438 288	
1687	439 233	
1687	458 154	
1687	461 450	
1687	464 519	
1687	466 6161	
1687	475 1334	
1687	498 313	
1687	528 1094	
1687	531 141	
1687	540 183	
1687	544 97	
1687	546 676	
1687	680 162	
1687	689 166	
1687	701 339	
1687	907 426	
1687	1032	375
1687	1246	260
1687	1441	95
1687	1452	276
1687	1455	346
1687	1456	103
1687	1613	181
1687	1681	102
1687	1934	245
1687	3378	475

1687	3392	143
1687	4466	103
1687	4474	210
1687	4512	134
1687	5685	115
1687	6121	403
1687	6483	227
1687	11326	109
1688	52	142
1688	65	1911
1688	81	301
1688	154	2231
1688	173	160
1688	204	135
1688	237	516
1688	238	110
1688	246	189
1688	261	93
1688	316	140
1688	362	149
1688	423	2048
1688	445	110
1688	526	77
1688	548	119
1688	622	133
1688	689	1184
1688	690	89
1688	697	815
1688	703	693
1688	757	272

1688	961 529	
1688	1098	1020
1688	1116	142
1688	1135	461
1688	1383	397
1688	1486	126
1688	1609	335
1688	1781	114
1688	1934	81
1688	1983	101
1688	1990	392
1688	2277	679
1688	2433	83
1688	2544	101
1688	2605	144
1688	2988	95
1688	3057	109
1688	3110	1033
1688	4153	269
1688	5184	100
1688	5914	357
1688	5942	98
1688	6032	116
1688	7349	259
1688	9484	324
1688	12023	286
1688	16051	164
1688	16052	69
1689	59 283	
1689	88 327	

1689	154	604
1689	433	622
1689	601	178
1689	603	294
1689	608	362
1689	609	293
1689	611	336
1689	703	233
1689	748	672
1689	749	209
1689	1155	222
1689	1627	297
1689	1737	499
1689	1804	729
1689	1943	396
1689	1957	294
1689	1964	302
1689	1969	294
1689	2297	292
1689	2521	176
1689	2530	218
1689	2533	223
1689	2542	559
1689	3068	215
1689	3294	191
1689	3411	224
1689	3696	200
1689	3697	175
1689	3700	537
1689	4064	244

1689	4092	243
1689	5043	188
1689	6217	204
1689	7741	255
1689	10202	1248
1689	11204	242
1689	12000	200
1689	13659	230
1689	14937	267
1689	15798	185
1689	16053	547
1689	16054	348
1689	16055	295
1689	16056	281
1689	16057	269
1689	16058	256
1689	16059	246
1689	16060	226
1690	55	1079
1690	58	479
1690	67	594
1690	89	1204
1690	227	283
1690	288	351
1690	289	796
1690	291	251
1690	292	416
1690	298	797
1690	300	719
1690	323	434

1690	333 455	
1690	344 262	
1690	352 505	
1690	430 855	
1690	437 278	
1690	461 274	
1690	466 634	
1690	498 310	
1690	525 458	
1690	526 415	
1690	528 305	
1690	530 300	
1690	532 626	
1690	535 231	
1690	546 614	
1690	548 270	
1690	679 535	
1690	689 605	
1690	690 387	
1690	691 324	
1690	703 435	
1690	886 295	
1690	906 264	
1690	980 464	
1690	1243	309
1690	1400	238
1690	1781	355
1690	1934	338
1690	2521	376
1690	3057	409

1690	3198	237
1690	3288	370
1690	4704	835
1690	5533	236
1690	8589	428
1690	9737	1440
1690	12105	525
1690	16061	236
1691	53	205
1691	56	1014
1691	64	440
1691	65	179
1691	70	205
1691	80	173
1691	84	173
1691	88	138
1691	154	478
1691	199	151
1691	203	149
1691	207	145
1691	210	221
1691	220	199
1691	228	322
1691	424	198
1691	432	235
1691	511	177
1691	709	158
1691	735	173
1691	969	259
1691	982	459

1691	1073	186
1691	1099	294
1691	1106	461
1691	1394	1139
1691	1396	209
1691	1397	263
1691	1398	449
1691	1408	172
1691	1410	215
1691	1738	273
1691	1983	165
1691	2137	192
1691	2587	166
1691	2602	177
1691	2605	184
1691	2672	133
1691	3413	167
1691	4027	394
1691	4029	255
1691	4725	284
1691	4728	322
1691	5045	375
1691	6047	198
1691	6053	159
1691	6054	158
1691	15079	239
1691	16062	204
1691	16063	161
1692	56	275
1692	65	427

1692	67	182
1692	89	614
1692	190	220
1692	227	821
1692	228	402
1692	288	359
1692	289	196
1692	306	417
1692	310	252
1692	324	455
1692	329	259
1692	366	256
1692	389	361
1692	463	1018
1692	467	1118
1692	511	717
1692	538	307
1692	604	707
1692	679	1001
1692	689	308
1692	707	230
1692	898	673
1692	991	282
1692	1081	834
1692	1243	264
1692	1375	262
1692	1377	216
1692	1383	315
1692	1412	592
1692	1415	187

1692	2380	314
1692	2544	403
1692	4118	176
1692	5338	572
1692	5437	762
1692	6203	616
1692	7157	504
1692	7183	250
1692	9347	185
1692	9982	248
1692	10042	439
1692	14317	318
1692	15822	187
1692	16064	395
1692	16065	365
1692	16066	304
1692	16067	179
1692	16068	178
1693	67	2
1693	89	14
1693	288	153
1693	289	7226
1693	292	6
1693	295	1
1693	299	27
1693	300	74
1693	306	1
1693	324	1
1693	325	7
1693	329	9

1693	333 65	
1693	344 57	
1693	349 2	
1693	377 1	
1693	378 6	
1693	468 24	
1693	484 2	
1693	524 2	
1693	525 2	
1693	543 1	
1693	544 10	
1693	679 1	
1693	680 30	
1693	686 1	
1693	701 3	
1693	913 3	
1693	972 2	
1693	1042	15
1693	1449	1
1693	1451	1
1693	2548	1
1693	3404	6
1693	4350	188
1693	4486	2
1693	4576	17
1693	11247	1
1693	12625	2
1693	16069	7
1693	16070	5
1693	16071	4

1693	16072	2
1693	16073	2
1694	229 779	
1694	267 4672	
1694	271 464	
1694	278 1278	
1694	284 460	
1694	329 402	
1694	331 477	
1694	527 1321	
1694	547 945	
1694	569 410	
1694	697 440	
1694	1377	485
1694	1613	619
1694	1976	805
1694	2135	1611
1694	2179	657
1694	2237	546
1694	3317	606
1694	3318	446
1694	3346	583
1694	3716	890
1694	4024	508
1694	4562	623
1694	6329	1190
1694	6887	1552
1694	6905	648
1694	9350	587
1694	9388	428

1694	10399	993
1694	11401	570
1694	12134	493
1694	12902	607
1694	13902	1479
1694	14436	927
1694	16074	1673
1694	16075	1568
1694	16076	1383
1694	16077	1337
1694	16078	958
1694	16079	821
1694	16080	698
1694	16081	641
1694	16082	575
1694	16083	558
1694	16084	494
1694	16085	436
1694	16086	424
1694	16087	423
1694	16088	422
1694	16089	409
1695	154 750	
1695	163 164	
1695	172 53	
1695	190 124	
1695	203 85	
1695	207 1624	
1695	226 163	
1695	227 318	

1695	228 95	
1695	234 61	
1695	424 1413	
1695	428 102	
1695	429 96	
1695	436 203	
1695	439 363	
1695	533 199	
1695	562 89	
1695	604 79	
1695	618 92	
1695	857 54	
1695	863 52	
1695	1048	283
1695	1090	333
1695	1163	44
1695	1239	119
1695	1412	45
1695	1504	218
1695	1509	53
1695	1512	245
1695	1515	215
1695	1517	179
1695	1766	43
1695	1977	46
1695	1988	71
1695	2123	51
1695	2128	94
1695	2137	104
1695	3026	71

1695	3400	42
1695	4115	728
1695	5030	61
1695	6217	45
1695	6726	74
1695	8053	70
1695	8479	48
1695	8710	137
1695	16090	245
1695	16091	231
1695	16092	60
1695	16093	54
1696	152 79	
1696	163 1958	
1696	188 61	
1696	227 172	
1696	366 36	
1696	599 15	
1696	607 67	
1696	707 60	
1696	710 27	
1696	718 28	
1696	728 44	
1696	841 42	
1696	917 100	
1696	920 83	
1696	922 26	
1696	931 28	
1696	947 14	
1696	959 44	

1696	1110	60
1696	1216	18
1696	1274	36
1696	1358	23
1696	1360	24
1696	1372	41
1696	1411	26
1696	1412	45
1696	1413	56
1696	1425	47
1696	1468	19
1696	2006	41
1696	2016	14
1696	2346	18
1696	2347	45
1696	2624	56
1696	2787	30
1696	2797	18
1696	2864	42
1696	3071	20
1696	3481	62
1696	3675	15
1696	3774	17
1696	4369	16
1696	4500	18
1696	4784	41
1696	5175	15
1696	5212	28
1696	6957	24
1696	16094	30

1696	16095	17
1696	16096	15
1697	163	197
1697	227	462
1697	245	1387
1697	250	240
1697	286	534
1697	735	190
1697	1207	257
1697	1413	221
1697	2609	587
1697	2893	344
1697	3131	331
1697	3133	364
1697	3134	286
1697	3138	633
1697	3146	408
1697	3147	240
1697	3149	213
1697	3163	373
1697	4062	236
1697	4344	285
1697	5353	217
1697	5848	214
1697	5850	560
1697	7556	197
1697	8121	376
1697	9596	275
1697	9598	408
1697	9829	308

1697	9833	283
1697	9834	267
1697	9836	626
1697	9839	534
1697	9843	231
1697	10663	336
1697	12243	205
1697	12255	199
1697	12257	198
1697	12258	206
1697	16097	471
1697	16098	381
1697	16099	368
1697	16100	312
1697	16101	278
1697	16102	238
1697	16103	215
1697	16104	207
1697	16105	199
1697	16106	199
1697	16107	192
1697	16108	188
1699	89	489
1699	157	479
1699	278	103
1699	288	969
1699	289	101
1699	291	79
1699	295	206
1699	296	85

1699	298 91	
1699	299 110	
1699	302 86	
1699	306 162	
1699	308 76	
1699	330 103	
1699	333 312	
1699	349 296	
1699	378 161	
1699	475 16374	
1699	499 88	
1699	525 243	
1699	543 102	
1699	545 85	
1699	907 78	
1699	1051	81
1699	1052	126
1699	1104	225
1699	1351	75
1699	1463	80
1699	1613	98
1699	2179	845
1699	2247	218
1699	2252	446
1699	2976	465
1699	4292	173
1699	6133	101
1699	6346	83
1699	6618	555
1699	6619	439

1699	13025	520
1699	16111	1395
1699	16112	709
1699	16113	197
1699	16114	168
1699	16115	130
1699	16116	111
1699	16117	91
1699	16118	79
1699	16119	72
1699	16120	71
1699	16121	70
1700	52	93
1700	81	53
1700	154	441
1700	159	153
1700	162	56
1700	163	154
1700	215	163
1700	220	252
1700	599	263
1700	604	263
1700	609	303
1700	610	561
1700	613	381
1700	632	148
1700	962	60
1700	1098	75
1700	1123	67
1700	1783	213

1700	1835	253
1700	2593	72
1700	2711	243
1700	2716	56
1700	2864	323
1700	3861	101
1700	4366	64
1700	4734	255
1700	6137	113
1700	6169	70
1700	7123	76
1700	7206	71
1700	8837	75
1700	10891	67
1700	11477	197
1700	11635	58
1700	11652	175
1700	11667	157
1700	13318	84
1700	13956	112
1700	16122	429
1700	16123	268
1700	16124	232
1700	16125	224
1700	16126	151
1700	16127	145
1700	16128	132
1700	16129	111
1700	16130	104
1700	16131	85

1700	16132	57
1700	16133	54
1701	45	71
1701	195	62
1701	222	1352
1701	227	67
1701	412	184
1701	418	263
1701	436	71
1701	486	66
1701	501	89
1701	562	243
1701	735	70
1701	755	59
1701	859	171
1701	1116	117
1701	1131	57
1701	1351	118
1701	1378	423
1701	1700	56
1701	1944	92
1701	2269	66
1701	2415	220
1701	2625	115
1701	2775	93
1701	3105	118
1701	3248	88
1701	3341	68
1701	3616	76
1701	3754	1922

1701	4114	81
1701	4191	147
1701	4196	68
1701	4262	98
1701	4432	201
1701	6229	263
1701	6452	91
1701	6773	641
1701	6824	57
1701	8280	140
1701	8461	60
1701	11985	80
1701	12143	684
1701	13260	167
1701	13525	122
1701	13621	119
1701	13960	64
1701	14318	299
1701	16134	153
1701	16135	88
1701	16136	63
1701	16137	60
1702	163 586	
1702	212 135	
1702	227 190	
1702	554 276	
1702	562 300	
1702	599 67	
1702	607 305	
1702	728 105	

1702	730 267	
1702	735 283	
1702	863 112	
1702	868 102	
1702	1369	475
1702	1372	794
1702	1411	89
1702	1412	1466
1702	1413	103
1702	1415	87
1702	1416	139
1702	1425	141
1702	2127	116
1702	2265	224
1702	2342	66
1702	2343	115
1702	2787	416
1702	2834	126
1702	2855	229
1702	2899	113
1702	3308	84
1702	3470	227
1702	3774	727
1702	3888	231
1702	3921	164
1702	4124	259
1702	4180	384
1702	4251	76
1702	4460	151
1702	4675	165

1702	4784	71
1702	5172	235
1702	5844	113
1702	6165	207
1702	6267	116
1702	6362	118
1702	8546	93
1702	8794	106
1702	9347	234
1702	11501	227
1702	16138	78
1702	16139	74
1703	89	86
1703	176	120
1703	189	168
1703	191	130
1703	209	139
1703	220	145
1703	230	172
1703	291	293
1703	311	147
1703	355	96
1703	371	180
1703	377	182
1703	378	114
1703	475	91
1703	486	286
1703	488	1036
1703	497	1825
1703	499	126

1703	517	181
1703	518	106
1703	707	574
1703	726	515
1703	732	118
1703	918	139
1703	928	275
1703	930	231
1703	951	271
1703	966	461
1703	975	119
1703	982	143
1703	1104	82
1703	1249	83
1703	1339	102
1703	1377	99
1703	1406	92
1703	1672	343
1703	1803	109
1703	1852	105
1703	1854	4479
1703	1859	117
1703	1905	247
1703	2346	98
1703	2350	107
1703	2380	79
1703	2790	121
1703	2941	159
1703	3083	183
1703	3928	81

1703	4180	93
1703	16140	104
1704	77	11
1704	89	25
1704	257	25
1704	293	32
1704	296	29
1704	299	14
1704	318	13
1704	323	19
1704	326	39
1704	334	34
1704	462	14
1704	465	29
1704	479	272
1704	800	67
1704	803	27
1704	906	254
1704	909	17
1704	911	13
1704	951	52
1704	972	31
1704	986	29
1704	1249	44
1704	1673	55
1704	1795	17
1704	2797	15
1704	3302	33
1704	3311	13
1704	3320	12

1704	3367	99
1704	3576	22
1704	3966	37
1704	5000	14
1704	5260	14
1704	7390	87
1704	7394	34
1704	11024	38
1704	11719	29
1704	13266	15
1704	16141	61
1704	16142	60
1704	16143	58
1704	16144	41
1704	16145	24
1704	16146	19
1704	16147	15
1704	16148	15
1704	16149	15
1704	16150	13
1704	16151	13
1704	16152	12
1705	65 498	
1705	199 348	
1705	207 1029	
1705	215 724	
1705	225 890	
1705	226 692	
1705	227 1950	
1705	228 1416	

1705 420 811

1705 421 884

1705 424 459

1705 428 400

1705 438 518

1705 439 1645

1705 562 1385

1705 604 727

1705 964 1852

1705 969 721

1705 977 332

1705 1048 785

1705 1090 388

1705 1376 465

1705 1412 342

1705 1481 664

1705 1517 422

1705 1520 555

1705 1658 449

1705 1755 494

1705 1778 308

1705 1988 466

1705 2131 516

1705 2582 405

1705 2591 1749

1705 2608 311

1705 2815 514

1705 3030 426

1705 3747 916

1705 4793 347

1705	5423	337
1705	5429	843
1705	6534	436
1705	7177	306
1705	7663	428
1705	11421	303
1705	11460	420
1705	11819	349
1705	14455	428
1705	16153	343
1705	16154	314
1705	16155	302
1706	56	1416
1706	65	529
1706	80	1203
1706	89	878
1706	174	1036
1706	238	1376
1706	306	821
1706	328	1565
1706	400	1093
1706	404	624
1706	968	1525
1706	1009	1358
1706	1099	783
1706	1306	1231
1706	1396	563
1706	1397	1025
1706	1398	1244
1706	1401	1232

1706	1405	1208
1706	1409	1124
1706	2094	1467
1706	2097	608
1706	2101	3261
1706	2102	1590
1706	2115	514
1706	2655	487
1706	2804	697
1706	3200	1283
1706	3324	843
1706	4036	716
1706	5045	420
1706	5332	461
1706	6497	435
1706	7615	479
1706	8390	2084
1706	10247	590
1706	14840	6506
1706	16156	2017
1706	16157	1158
1706	16158	1148
1706	16159	1024
1706	16160	680
1706	16161	615
1706	16162	603
1706	16163	593
1706	16164	487
1706	16165	469
1706	16166	436

1706	16167	404
1706	16168	395
1707	154 41	
1707	166 34	
1707	207 236	
1707	212 113	
1707	221 51	
1707	227 1835	
1707	234 413	
1707	429 27	
1707	436 41	
1707	533 247	
1707	554 142	
1707	570 87	
1707	585 46	
1707	614 77	
1707	679 60	
1707	706 154	
1707	707 251	
1707	715 31	
1707	728 52	
1707	733 74	
1707	735 193	
1707	854 87	
1707	857 37	
1707	868 101	
1707	917 121	
1707	918 114	
1707	959 45	
1707	982 122	

1707	1048	41
1707	1126	30
1707	1150	33
1707	1244	67
1707	1249	76
1707	1358	47
1707	1369	32
1707	1390	44
1707	1412	30
1707	1414	174
1707	1416	119
1707	1468	62
1707	1512	40
1707	1807	47
1707	1974	28
1707	1985	43
1707	2029	35
1707	2339	33
1707	2786	76
1707	4251	42
1707	4821	41
1707	5844	96
1708	65	413
1708	70	600
1708	71	203
1708	88	850
1708	89	179
1708	154	398
1708	155	667
1708	167	166

1708	172 269	
1708	199 166	
1708	203 1993	
1708	206 1901	
1708	209 147	
1708	217 228	
1708	227 147	
1708	416 1911	
1708	426 224	
1708	429 416	
1708	434 343	
1708	511 3470	
1708	533 328	
1708	599 301	
1708	615 248	
1708	620 1360	
1708	709 683	
1708	715 146	
1708	718 190	
1708	857 247	
1708	863 211	
1708	982 339	
1708	1106	135
1708	1107	167
1708	1146	147
1708	1155	188
1708	1239	990
1708	1375	138
1708	1376	168
1708	1513	165

1708	1632	247
1708	1705	316
1708	1888	383
1708	1957	372
1708	2537	1030
1708	2584	178
1708	4069	148
1708	4079	599
1708	7194	628
1708	10610	342
1708	11234	285
1708	16169	178
1709	72	262
1709	163	492
1709	227	3886
1709	572	724
1709	607	125
1709	633	163
1709	717	215
1709	733	260
1709	735	839
1709	959	204
1709	975	455
1709	1244	1013
1709	1414	1201
1709	1415	149
1709	1416	439
1709	1426	120
1709	1464	319
1709	1639	148

1709	2026	121
1709	2346	540
1709	2394	380
1709	2475	377
1709	2624	3581
1709	2642	1321
1709	3065	410
1709	3892	1950
1709	4369	185
1709	4405	126
1709	4616	306
1709	4620	449
1709	4790	145
1709	5020	157
1709	5237	1613
1709	5241	418
1709	5302	171
1709	6959	261
1709	8802	665
1709	15134	140
1709	15675	134
1709	16170	914
1709	16171	492
1709	16172	351
1709	16173	323
1709	16174	320
1709	16175	220
1709	16176	207
1709	16177	176
1709	16178	170

1709	16179	166
1709	16180	134
1710	7	273
1710	407	187
1710	420	106
1710	986	100
1710	1112	245
1710	1124	92
1710	1491	498
1710	1700	204
1710	2756	80
1710	2832	122
1710	3528	75
1710	3531	75
1710	3886	79
1710	4887	617
1710	4895	140
1710	5066	88
1710	5791	79
1710	5970	228
1710	6967	127
1710	7977	126
1710	8625	124
1710	9783	118
1710	13857	181
1710	14169	270
1710	14624	209
1710	16181	570
1710	16182	513
1710	16183	367

1710	16184	352
1710	16185	206
1710	16186	170
1710	16187	163
1710	16188	139
1710	16189	136
1710	16190	126
1710	16191	116
1710	16192	114
1710	16193	108
1710	16194	105
1710	16195	101
1710	16196	99
1710	16197	98
1710	16198	94
1710	16199	91
1710	16200	89
1710	16201	88
1710	16202	86
1710	16203	85
1710	16204	78
1710	16205	74
1711	89	1157
1711	197	3725
1711	206	900
1711	289	931
1711	428	2350
1711	429	1371
1711	569	2732
1711	599	2643

1711	620	1553
1711	629	1654
1711	857	1403
1711	886	1054
1711	981	1150
1711	1001	798
1711	1075	3585
1711	1089	5086
1711	1164	2291
1711	1206	1599
1711	1513	824
1711	1837	1310
1711	1888	2187
1711	2131	2434
1711	2346	1014
1711	3057	1207
1711	3185	909
1711	3673	1257
1711	4495	979
1711	4810	2909
1711	5079	1304
1711	5785	1151
1711	6452	956
1711	6852	1004
1711	7076	1255
1711	7325	2286
1711	7661	6859
1711	8223	1054
1711	8659	3925
1711	9629	4211

1711	9931	1085
1711	10041	3279
1711	10761	841
1711	11230	2361
1711	11232	1298
1711	12724	2061
1711	12752	976
1711	13371	1202
1711	13416	7917
1711	16206	3195
1711	16207	1299
1711	16208	766
1712	7	264
1712	65	531
1712	163	4686
1712	166	1214
1712	176	363
1712	190	260
1712	198	253
1712	212	346
1712	226	473
1712	227	260
1712	228	520
1712	230	1014
1712	234	354
1712	436	1304
1712	614	4607
1712	707	873
1712	712	1486
1712	734	421

1712	854 5668	
1712	856 1245	
1712	860 1067	
1712	868 325	
1712	917 394	
1712	920 707	
1712	965 453	
1712	978 762	
1712	982 736	
1712	1118	1527
1712	1119	537
1712	1131	4734
1712	1369	1529
1712	1372	905
1712	1384	758
1712	1412	868
1712	1778	405
1712	1812	481
1712	2352	348
1712	2380	731
1712	3475	595
1712	4179	399
1712	6724	264
1712	6730	2102
1712	9344	1803
1712	9346	736
1712	9818	286
1712	10537	922
1712	13379	499
1712	13890	426

1712	16209	294
1712	16210	263
1715	55	238
1715	71	150
1715	81	127
1715	89	1299
1715	97	188
1715	172	1243
1715	173	2097
1715	288	637
1715	289	358
1715	295	129
1715	302	1860
1715	316	4037
1715	378	419
1715	423	564
1715	438	136
1715	439	632
1715	526	498
1715	532	1388
1715	538	130
1715	542	2413
1715	548	1804
1715	689	1681
1715	704	398
1715	714	308
1715	895	533
1715	896	336
1715	903	389
1715	915	135

1715	961	5599
1715	1098	696
1715	1167	713
1715	1243	255
1715	1375	209
1715	1379	868
1715	1382	205
1715	1424	629
1715	1452	175
1715	1934	199
1715	2130	748
1715	2521	1607
1715	3110	205
1715	3125	460
1715	4709	217
1715	4941	302
1715	5977	208
1715	6647	409
1715	8621	948
1715	10220	147
1715	10693	119
1715	12311	264
1717	55	523
1717	65	444
1717	67	956
1717	89	998
1717	257	724
1717	288	2220
1717	289	21382
1717	291	915

1717	292 1411
1717	293 468
1717	294 937
1717	295 1094
1717	296 1539
1717	299 731
1717	300 1681
1717	301 495
1717	306 457
1717	310 837
1717	311 467
1717	318 1756
1717	320 486
1717	326 588
1717	329 519
1717	333 4639
1717	344 1231
1717	374 1569
1717	375 7229
1717	377 2200
1717	378 2119
1717	379 2989
1717	456 560
1717	465 441
1717	466 506
1717	468 719
1717	474 444
1717	498 899
1717	525 1262
1717	531 603

1717	538	1947
1717	540	576
1717	546	595
1717	679	505
1717	906	932
1717	1098	527
1717	3081	718
1717	3324	876
1717	3466	466
1717	3489	1049
1717	3495	436
1717	7619	1216
1718	89	347
1718	97	220
1718	190	167
1718	295	318
1718	298	219
1718	300	198
1718	302	520
1718	306	192
1718	355	201
1718	366	202
1718	430	281
1718	437	794
1718	442	240
1718	538	267
1718	567	207
1718	575	405
1718	599	197
1718	896	344

1718	903 298	
1718	1390	185
1718	1427	190
1718	1772	525
1718	1833	1572
1718	1934	787
1718	1971	262
1718	2125	227
1718	2175	390
1718	2176	303
1718	2419	219
1718	2458	320
1718	2612	213
1718	2632	175
1718	2638	169
1718	3019	287
1718	3057	631
1718	3126	175
1718	3488	226
1718	3757	336
1718	4495	309
1718	4616	436
1718	4710	211
1718	5789	272
1718	7743	170
1718	7753	251
1718	8103	245
1718	8594	187
1718	11729	327
1718	11887	314

1718	12141	316
1718	12480	236
1719	616 113	
1719	1548	199
1719	2101	219
1719	2289	164
1719	4636	114
1719	5256	150
1719	8136	555
1719	8390	137
1719	9453	252
1719	9585	444
1719	9587	199
1719	10547	114
1719	10672	134
1719	11874	116
1719	11877	154
1719	11878	666
1719	12491	113
1719	14324	175
1719	15845	231
1719	16229	333
1719	16230	299
1719	16231	299
1719	16232	230
1719	16233	217
1719	16234	205
1719	16235	192
1719	16236	189
1719	16237	188

1719	16238	185
1719	16239	177
1719	16240	175
1719	16241	174
1719	16242	174
1719	16243	173
1719	16244	169
1719	16245	166
1719	16246	164
1719	16247	163
1719	16248	151
1719	16249	145
1719	16250	144
1719	16251	140
1719	16252	139
1719	16253	136
1719	16254	126
1719	16255	122
1719	16256	119
1719	16257	116
1719	16258	114
1719	16259	114
1720	72	1035
1720	159	604
1720	212	275
1720	222	404
1720	250	296
1720	412	438
1720	429	4363
1720	433	317

1720	547 290	
1720	703 304	
1720	859 448	
1720	1424	1213
1720	2541	652
1720	2542	2441
1720	3317	254
1720	3616	519
1720	3729	2602
1720	3792	542
1720	3884	675
1720	4197	636
1720	4736	953
1720	5600	375
1720	6997	260
1720	8932	1032
1720	8945	1135
1720	8948	388
1720	9457	290
1720	10750	341
1720	10753	1152
1720	10804	494
1720	11042	780
1720	11850	930
1720	11859	660
1720	11865	1222
1720	13616	381
1720	14108	734
1720	16260	1995
1720	16261	792

1720	16262	551
1720	16263	539
1720	16264	498
1720	16265	495
1720	16266	455
1720	16267	424
1720	16268	365
1720	16269	304
1720	16270	295
1720	16271	293
1720	16272	277
1720	16273	251
1721	585 77	
1721	2902	162
1721	4722	97
1721	4724	145
1721	5388	323
1721	5417	108
1721	7224	116
1721	7239	142
1721	8553	69
1721	9591	51
1721	14084	49
1721	16274	351
1721	16275	289
1721	16276	283
1721	16277	197
1721	16278	176
1721	16279	168
1721	16280	149

1721	16281	145
1721	16282	126
1721	16283	125
1721	16284	118
1721	16285	112
1721	16286	110
1721	16287	82
1721	16288	82
1721	16289	70
1721	16290	66
1721	16291	66
1721	16292	64
1721	16293	62
1721	16294	61
1721	16295	60
1721	16296	59
1721	16297	52
1721	16298	52
1721	16299	50
1721	16300	50
1721	16301	50
1721	16302	49
1721	16303	49
1721	16304	48
1721	16305	48
1721	16306	48
1721	16307	47
1721	16308	47
1721	16309	47
1721	16310	46

1721	16311	45
1721	16312	44
1722	51	100
1722	55	312
1722	65	336
1722	67	2041
1722	89	686
1722	97	51
1722	155	122
1722	161	60
1722	288	302
1722	289	448
1722	294	247
1722	295	224
1722	299	167
1722	300	130
1722	301	66
1722	302	49
1722	306	49
1722	310	201
1722	316	84
1722	327	69
1722	328	145
1722	329	122
1722	332	60
1722	352	97
1722	466	50
1722	485	56
1722	533	50
1722	535	53

1722	538	106
1722	641	103
1722	701	126
1722	907	91
1722	972	98
1722	1243	108
1722	1432	99
1722	1505	53
1722	3674	117
1722	4707	101
1722	5000	78
1722	5615	151
1722	5617	85
1722	5985	109
1722	7187	72
1722	8020	112
1722	10076	146
1722	15665	173
1722	16313	65
1722	16314	53
1722	16315	52
1722	16316	50
1723	57	44
1723	103	35
1723	238	42
1723	418	51
1723	748	44
1723	1121	67
1723	1123	78
1723	1130	50

1723	1175	58
1723	1491	55
1723	1731	37
1723	1747	38
1723	2605	30
1723	3539	29
1723	5193	29
1723	6151	51
1723	6159	37
1723	6173	37
1723	7119	35
1723	7700	50
1723	9143	103
1723	9271	37
1723	9362	64
1723	10889	79
1723	13587	37
1723	13590	74
1723	13595	94
1723	13597	68
1723	13953	89
1723	14361	109
1723	15338	83
1723	16317	111
1723	16318	76
1723	16319	75
1723	16320	74
1723	16321	73
1723	16322	69
1723	16323	66

1723	16324	64
1723	16325	58
1723	16326	57
1723	16327	57
1723	16328	53
1723	16329	53
1723	16330	52
1723	16331	41
1723	16332	39
1723	16333	33
1723	16334	32
1723	16335	30
1724	51	111
1724	52	20
1724	56	117
1724	65	39
1724	67	38
1724	72	481
1724	88	20
1724	172	114
1724	173	527
1724	183	170
1724	207	317
1724	229	34
1724	234	26
1724	238	29
1724	425	48
1724	432	36
1724	534	26
1724	748	37

1724	1048	22
1724	1512	99
1724	1991	67
1724	2122	25
1724	2601	52
1724	2716	71
1724	2747	24
1724	3501	22
1724	3992	55
1724	4913	27
1724	5890	32
1724	6246	33
1724	8174	29
1724	10722	82
1724	11753	59
1724	12674	39
1724	13090	72
1724	16336	94
1724	16337	85
1724	16338	69
1724	16339	61
1724	16340	49
1724	16341	46
1724	16342	42
1724	16343	37
1724	16344	28
1724	16345	25
1724	16346	25
1724	16347	24
1724	16348	24

1724	16349	24
1724	16350	22
1725	89	260
1725	97	110
1725	157	1790
1725	228	60
1725	266	42
1725	288	280
1725	289	47
1725	291	99
1725	294	88
1725	295	416
1725	296	81
1725	300	114
1725	301	94
1725	307	53
1725	311	42
1725	314	50
1725	317	77
1725	323	122
1725	324	198
1725	325	42
1725	344	82
1725	350	78
1725	352	390
1725	355	40
1725	461	97
1725	464	36
1725	466	53
1725	476	138

1725	521	52
1725	546	71
1725	548	74
1725	679	124
1725	689	72
1725	690	47
1725	698	66
1725	699	118
1725	701	150
1725	906	492
1725	1051	36
1725	1377	60
1725	2020	719
1725	2025	39
1725	4704	34
1725	4707	46
1725	5278	63
1725	5746	38
1725	7288	150
1725	8479	41
1725	10640	34
1725	11241	150
1726	55	93
1726	67	392
1726	89	517
1726	157	132
1726	161	110
1726	257	169
1726	285	252
1726	288	1056

1726	289 13804
1726	291 190
1726	292 1396
1726	293 164
1726	295 544
1726	299 191
1726	300 651
1726	302 146
1726	306 217
1726	309 239
1726	314 284
1726	327 150
1726	328 466
1726	330 87
1726	331 364
1726	349 224
1726	352 478
1726	459 101
1726	461 112
1726	466 446
1726	468 420
1726	475 294
1726	523 81
1726	525 145
1726	527 127
1726	531 95
1726	538 131
1726	544 163
1726	701 1027
1726	907 194

1726	913 170	
1726	1032	95
1726	1037	443
1726	1402	85
1726	1456	86
1726	2020	94
1726	2025	138
1726	2083	300
1726	2521	117
1726	4560	142
1726	6376	112
1726	6389	125
1727	65 326	
1727	173 411	
1727	182 328	
1727	190 2016	
1727	199 1035	
1727	207 710	
1727	210 1405	
1727	222 559	
1727	225 414	
1727	228 709	
1727	229 957	
1727	233 929	
1727	236 337	
1727	377 445	
1727	425 393	
1727	432 706	
1727	453 445	
1727	517 359	

1727	716 602	
1727	969 894	
1727	1090	442
1727	1151	685
1727	1155	383
1727	1375	953
1727	1515	523
1727	1988	515
1727	2535	478
1727	2580	365
1727	2614	624
1727	2815	640
1727	3084	367
1727	3258	397
1727	3273	701
1727	3475	520
1727	3681	657
1727	3945	555
1727	4025	423
1727	4089	354
1727	4118	341
1727	5030	326
1727	6222	487
1727	6391	341
1727	7663	382
1727	9648	511
1727	11244	322
1727	13376	574
1727	13377	691
1727	16351	714

1727	16352	703
1727	16353	350
1728	52	118
1728	54	92
1728	59	79
1728	65	290
1728	72	7877
1728	81	336
1728	154	348
1728	172	103
1728	173	331
1728	174	135
1728	182	418
1728	187	182
1728	190	1303
1728	234	218
1728	418	77
1728	423	96
1728	441	1023
1728	533	137
1728	858	112
1728	863	209
1728	880	199
1728	961	148
1728	969	80
1728	997	123
1728	1061	1390
1728	1072	651
1728	1073	90
1728	1085	97

1728	1090	266
1728	1097	206
1728	1098	150
1728	1213	106
1728	1384	76
1728	1424	96
1728	1534	103
1728	1707	204
1728	1713	238
1728	1790	462
1728	1794	89
1728	1836	81
1728	2030	577
1728	2542	420
1728	2681	187
1728	3280	170
1728	4316	150
1728	6585	81
1728	7934	100
1728	9245	80
1728	16354	83
1728	16355	76
1729	72 81	
1729	154 561	
1729	159 162	
1729	210 90	
1729	225 164	
1729	226 94	
1729	234 139	
1729	416 150	

1729	418	235
1729	427	117
1729	436	79
1729	441	138
1729	512	74
1729	615	525
1729	620	77
1729	715	95
1729	859	209
1729	1046	91
1729	1149	153
1729	1239	136
1729	1376	78
1729	1378	195
1729	1418	143
1729	1947	105
1729	1957	125
1729	1964	226
1729	2271	82
1729	2538	126
1729	2542	136
1729	2600	164
1729	2611	141
1729	3411	96
1729	3616	103
1729	3730	76
1729	4058	84
1729	4069	109
1729	6182	200
1729	6206	85

1729	8043	216
1729	8705	100
1729	9952	116
1729	9955	84
1729	14352	119
1729	15172	77
1729	15548	102
1729	16356	565
1729	16357	118
1729	16358	112
1729	16359	102
1729	16360	96
1730	7	432
1730	8	1581
1730	55	705
1730	58	475
1730	67	422
1730	84	412
1730	89	1198
1730	157	840
1730	229	745
1730	288	539
1730	289	709
1730	292	400
1730	295	485
1730	300	499
1730	371	499
1730	390	432
1730	423	850
1730	538	535

1730	542 466	
1730	544 444	
1730	546 402	
1730	674 454	
1730	811 787	
1730	816 467	
1730	1041	654
1730	1044	635
1730	1091	487
1730	1098	419
1730	1104	429
1730	1180	402
1730	1195	603
1730	1797	782
1730	1910	1396
1730	1919	591
1730	1934	822
1730	2100	3607
1730	2101	669
1730	2103	461
1730	2521	394
1730	2655	499
1730	3092	610
1730	3367	800
1730	4706	493
1730	6792	390
1730	6996	460
1730	8404	985
1730	8589	503
1730	16361	536

1730	16362	478
1730	16363	395
1731	16364	293
1732	52	6
1732	58	42
1732	80	6
1732	159	21
1732	206	147
1732	227	73
1732	429	23
1732	603	9
1732	612	8
1732	1156	11
1732	1801	19
1732	1958	7
1732	1971	55
1732	2161	12
1732	2265	103
1732	2277	18
1732	2419	7
1732	2531	112
1732	2535	10
1732	2630	9
1732	4444	8
1732	5192	28
1732	5414	40
1732	5890	90
1732	6004	15
1732	6151	20
1732	6167	9

1732	6169	7
1732	6229	62
1732	6764	6
1732	7123	6
1732	7742	19
1732	8513	23
1732	10663	10
1732	11737	7
1732	11919	7
1732	12868	8
1732	16365	72
1732	16366	65
1732	16367	54
1732	16368	47
1732	16369	37
1732	16370	35
1732	16371	17
1732	16372	13
1732	16373	9
1732	16374	9
1732	16375	9
1732	16376	8
1732	16377	7
1733	198 169	
1733	226 469	
1733	230 575	
1733	234 610	
1733	348 206	
1733	383 1291	
1733	500 249	

1733	707 141	
1733	726 201	
1733	806 151	
1733	808 266	
1733	821 346	
1733	840 159	
1733	841 790	
1733	843 499	
1733	917 415	
1733	923 736	
1733	942 293	
1733	953 1009	
1733	957 214	
1733	978 166	
1733	982 484	
1733	1091	239
1733	1099	195
1733	1116	261
1733	1119	184
1733	1179	404
1733	1360	135
1733	1369	135
1733	1527	217
1733	1650	288
1733	1974	138
1733	2032	333
1733	2352	169
1733	2373	459
1733	3717	292
1733	4155	316

1733	4156	469
1733	4375	167
1733	4821	870
1733	7113	365
1733	7650	455
1733	8246	194
1733	8249	198
1733	8259	165
1733	8788	139
1733	9635	282
1733	16378	280
1733	16379	232
1733	16380	214
1734	69	388
1734	89	5498
1734	157	303
1734	190	556
1734	228	550
1734	257	1916
1734	285	540
1734	288	7768
1734	289	1448
1734	292	475
1734	294	5721
1734	295	21648
1734	314	696
1734	321	268
1734	327	321
1734	331	1410
1734	332	434

1734	349 307	
1734	461 687	
1734	462 7137	
1734	464 266	
1734	467 1479	
1734	468 4682	
1734	472 854	
1734	482 1988	
1734	488 523	
1734	493 925	
1734	498 426	
1734	507 263	
1734	537 740	
1734	544 1209	
1734	646 425	
1734	681 319	
1734	686 371	
1734	830 255	
1734	912 1579	
1734	913 1176	
1734	1180	4593
1734	1457	281
1734	1596	569
1734	1613	774
1734	1672	406
1734	2783	624
1734	2990	380
1734	3320	2987
1734	5615	331
1734	16381	821

1734	16382	673
1734	16383	373
1734	16384	263
1736	55	1190
1736	67	602
1736	89	286
1736	97	434
1736	161	303
1736	227	1163
1736	256	233
1736	288	648
1736	292	1247
1736	324	251
1736	329	374
1736	333	944
1736	464	341
1736	466	720
1736	531	591
1736	541	604
1736	575	335
1736	679	2427
1736	689	267
1736	701	1218
1736	706	289
1736	715	261
1736	962	638
1736	1081	1376
1736	1456	250
1736	1505	1494
1736	1519	294

1736	1721	280
1736	2025	249
1736	2176	262
1736	2498	685
1736	2834	278
1736	3101	268
1736	3460	233
1736	3575	585
1736	3763	958
1736	4435	236
1736	4442	517
1736	4489	291
1736	5257	572
1736	5268	1124
1736	5270	238
1736	6389	258
1736	7868	233
1736	8718	1878
1736	8742	395
1736	13219	492
1736	16385	629
1736	16386	251
1736	16387	232
1737	162 929	
1737	183 392	
1737	198 300	
1737	210 232	
1737	220 484	
1737	233 968	
1737	512 321	

1737	517 485	
1737	537 287	
1737	755 480	
1737	761 590	
1737	777 227	
1737	1100	238
1737	1116	1340
1737	1118	818
1737	1131	650
1737	1170	276
1737	1701	372
1737	1706	2661
1737	1929	247
1737	2148	531
1737	2206	900
1737	2366	269
1737	2574	276
1737	2668	776
1737	2699	477
1737	2775	455
1737	3817	230
1737	4852	321
1737	5063	310
1737	5069	372
1737	5153	244
1737	5653	374
1737	5966	409
1737	6439	245
1737	8258	580
1737	8383	277

1737	9783	261
1737	10026	298
1737	13904	323
1737	14687	684
1737	14688	826
1737	14692	277
1737	16388	482
1737	16389	310
1737	16390	284
1737	16391	283
1737	16392	257
1737	16393	252
1737	16394	228
1740	56	856
1740	101	1382
1740	154	2005
1740	157	1076
1740	159	992
1740	207	1000
1740	278	1109
1740	418	1239
1740	430	561
1740	546	729
1740	578	705
1740	603	964
1740	604	570
1740	629	542
1740	757	653
1740	980	756
1740	1109	3258

1740	1122	935
1740	1131	496
1740	1222	491
1740	1394	441
1740	1396	1237
1740	1400	455
1740	1409	643
1740	1410	1181
1740	1969	658
1740	1975	3315
1740	2174	494
1740	2602	800
1740	2605	1499
1740	2667	1691
1740	2783	462
1740	2811	456
1740	3132	691
1740	3164	558
1740	3248	441
1740	3502	9516
1740	4153	598
1740	5045	655
1740	6581	650
1740	7079	3249
1740	8068	2067
1740	11852	524
1740	12868	1122
1740	15999	1170
1740	16404	1568
1740	16405	729

1740	16406	519
1740	16407	474
1740	16408	474
1741	55	247
1741	56	161
1741	157	1603
1741	190	290
1741	207	704
1741	210	1061
1741	424	1042
1741	432	629
1741	458	1209
1741	471	185
1741	486	176
1741	527	221
1741	547	372
1741	689	1142
1741	690	239
1741	716	186
1741	757	338
1741	768	252
1741	779	1661
1741	813	207
1741	815	510
1741	830	178
1741	923	318
1741	953	174
1741	1037	225
1741	1048	1018
1741	1097	3746

1741	1109	174
1741	1191	1496
1741	1398	403
1741	1400	246
1741	1409	492
1741	1511	223
1741	2083	163
1741	2092	194
1741	2134	439
1741	2242	1572
1741	2379	487
1741	2815	270
1741	2850	175
1741	3200	272
1741	4704	184
1741	4923	255
1741	5149	202
1741	6397	283
1741	7643	270
1741	7743	636
1741	8589	599
1741	11695	263
1741	12621	758
1742	55	1867
1742	65	245
1742	67	4814
1742	89	10666
1742	167	435
1742	227	318
1742	229	493

1742	288	1114
1742	289	4585
1742	292	2595
1742	295	1097
1742	298	206
1742	300	1205
1742	325	290
1742	333	1112
1742	340	326
1742	352	4416
1742	424	430
1742	464	167
1742	466	1564
1742	524	1198
1742	526	446
1742	530	165
1742	544	418
1742	548	1299
1742	599	495
1742	646	340
1742	679	1931
1742	689	501
1742	889	193
1742	959	1278
1742	980	596
1742	1037	365
1742	1045	270
1742	1243	1895
1742	1379	361
1742	1429	544

1742	1464	176
1742	1934	1384
1742	2083	328
1742	2521	174
1742	2543	2358
1742	3200	1502
1742	3319	212
1742	5529	1059
1742	5535	282
1742	5975	689
1742	9079	216
1742	16409	203
1742	16410	189
1743	209 580	
1743	466 920	
1743	498 1931	
1743	693 2602	
1743	768 1009	
1743	779 5235	
1743	805 1046	
1743	808 1208	
1743	809 961	
1743	816 2460	
1743	825 915	
1743	1035	1860
1743	1044	1491
1743	1180	3321
1743	1181	3333
1743	1182	863
1743	1184	1213

1743	1185	2436
1743	1188	3329
1743	1196	2939
1743	1281	1070
1743	1541	1524
1743	1551	795
1743	1557	1862
1743	1670	1838
1743	1678	538
1743	1853	2506
1743	1868	4222
1743	2200	1490
1743	2203	916
1743	2204	724
1743	2210	1892
1743	2214	4227
1743	2617	1520
1743	2618	2434
1743	2619	857
1743	2621	1191
1743	2850	563
1743	2959	676
1743	2967	719
1743	4168	1625
1743	4774	1224
1743	4936	827
1743	4995	729
1743	5126	1379
1743	5969	2955
1743	6016	2489

1743	14443	1259
1743	14444	3472
1743	16411	550
1744	65	7
1744	182	2
1744	366	4
1744	511	2
1744	533	7
1744	1375	2
1744	3112	15
1744	4109	3
1744	4446	3
1744	7194	3
1744	10969	1
1744	12726	7
1744	13102	1
1744	16412	11
1744	16413	1
1745	204	154
1745	217	313
1745	223	156
1745	227	364
1745	228	172
1745	342	166
1745	429	329
1745	430	215
1745	454	194
1745	682	210
1745	683	231
1745	816	254

1745	823 385	
1745	1181	153
1745	1183	439
1745	1187	193
1745	1192	156
1745	1195	318
1745	1447	180
1745	1485	143
1745	1541	143
1745	1552	612
1745	2051	199
1745	2204	203
1745	2439	378
1745	2572	168
1745	2608	413
1745	2621	366
1745	2840	249
1745	2847	350
1745	3411	220
1745	4096	325
1745	4102	158
1745	4166	238
1745	4177	140
1745	4232	198
1745	5547	207
1745	5972	159
1745	6906	474
1745	8264	564
1745	8274	232
1745	8459	228

1745	8464	136
1745	9943	453
1745	11393	247
1745	12686	204
1745	13430	208
1745	15975	155
1745	16414	249
1745	16415	217
1746	58	971
1746	59	835
1746	61	610
1746	65	713
1746	66	483
1746	67	761
1746	72	1364
1746	99	648
1746	154	2077
1746	182	434
1746	187	614
1746	199	1049
1746	206	1200
1746	207	413
1746	210	683
1746	220	491
1746	225	852
1746	228	701
1746	229	647
1746	238	618
1746	416	2366
1746	418	710

1746	429 440	
1746	511 2026	
1746	533 625	
1746	709 490	
1746	715 939	
1746	716 474	
1746	862 611	
1746	905 946	
1746	969 818	
1746	1384	409
1746	1977	468
1746	2469	504
1746	2531	1053
1746	2535	522
1746	2606	512
1746	3201	469
1746	3502	598
1746	3945	1328
1746	4064	465
1746	4311	994
1746	4444	957
1746	5419	395
1746	5890	1416
1746	7525	521
1746	9823	707
1746	11229	702
1746	12298	586
1746	16416	596
1747	53 28	
1747	81 31	

1747	88	34
1747	154	153
1747	227	104
1747	233	52
1747	237	30
1747	418	65
1747	444	47
1747	599	30
1747	601	32
1747	603	42
1747	610	51
1747	615	43
1747	621	25
1747	631	29
1747	735	29
1747	744	42
1747	1098	51
1747	1620	45
1747	1720	26
1747	1752	26
1747	2030	44
1747	2277	28
1747	2470	25
1747	2578	36
1747	2624	31
1747	3003	84
1747	3505	70
1747	3778	30
1747	4064	40
1747	4079	35

1747	4532	73
1747	5052	38
1747	5503	82
1747	6756	29
1747	6762	84
1747	8510	26
1747	8594	59
1747	9206	34
1747	9862	36
1747	9997	25
1747	12343	31
1747	15247	35
1747	15881	120
1747	16417	46
1747	16418	41
1747	16419	36
1747	16420	32
1747	16421	30
1748	230 157	
1748	288 262	
1748	291 144	
1748	300 184	
1748	371 145	
1748	377 192	
1748	466 141	
1748	475 190	
1748	487 184	
1748	493 149	
1748	497 137	
1748	498 434	

1748	499	388
1748	518	794
1748	548	149
1748	681	276
1748	686	155
1748	689	214
1748	690	162
1748	917	139
1748	930	435
1748	974	140
1748	1183	162
1748	1339	210
1748	1613	150
1748	2014	483
1748	2062	172
1748	2951	199
1748	3124	386
1748	3422	197
1748	3901	161
1748	5459	421
1748	5683	150
1748	7191	185
1748	10865	248
1748	10978	153
1748	11581	168
1748	13214	243
1748	14121	144
1748	14628	390
1748	16422	312
1748	16423	174

1748	16424	173
1748	16425	171
1748	16426	165
1748	16427	163
1748	16428	152
1748	16429	149
1748	16430	143
1748	16431	140
1749	7	947
1749	212	3391
1749	220	1282
1749	227	3841
1749	234	1256
1749	383	745
1749	497	1136
1749	599	1006
1749	709	1213
1749	718	1484
1749	732	1923
1749	733	946
1749	808	1372
1749	857	1524
1749	889	738
1749	898	901
1749	917	1056
1749	951	864
1749	959	1722
1749	985	1216
1749	1239	2235
1749	1248	989

1749	1513	2924
1749	1803	1671
1749	1810	763
1749	1974	1040
1749	2346	1832
1749	2350	1115
1749	2788	1387
1749	2902	2868
1749	2967	1036
1749	3129	1025
1749	3242	1337
1749	3328	912
1749	3365	1093
1749	3430	1212
1749	3444	1092
1749	3456	1967
1749	3739	1195
1749	5375	2256
1749	6195	1005
1749	6452	3769
1749	6677	2228
1749	8614	1762
1749	8649	1985
1749	10127	977
1749	10404	989
1749	10565	776
1749	12573	899
1749	16432	1005
1751	154 18	
1751	162 25	

1751	418 33	
1751	445 21	
1751	632 38	
1751	751 94	
1751	752 26	
1751	763 86	
1751	769 27	
1751	1098	41
1751	1134	41
1751	1418	38
1751	2429	71
1751	2542	42
1751	2573	34
1751	2579	18
1751	2771	25
1751	3028	152
1751	3040	26
1751	3805	38
1751	3826	56
1751	4086	56
1751	4611	59
1751	5880	38
1751	6048	37
1751	6759	37
1751	8458	43
1751	8475	21
1751	10449	43
1751	10676	55
1751	10831	63
1751	10890	41

1751	11604	133
1751	11608	56
1751	11609	81
1751	11611	76
1751	11612	22
1751	11613	25
1751	11614	77
1751	11625	26
1751	11712	20
1751	12324	35
1751	12487	67
1751	13478	32
1751	15876	47
1751	16437	56
1751	16438	37
1751	16439	26
1751	16440	22
1751	16441	19
1752	56	3729
1752	58	611
1752	65	198
1752	72	146
1752	154	374
1752	170	155
1752	190	437
1752	532	357
1752	604	1598
1752	689	397
1752	697	138
1752	703	1635

1752	912 557	
1752	1075	348
1752	1098	217
1752	1379	492
1752	1688	387
1752	1705	200
1752	1781	210
1752	2086	4346
1752	2101	3814
1752	2118	130
1752	3026	223
1752	3077	195
1752	3109	1003
1752	3858	2067
1752	3868	471
1752	4706	422
1752	4728	139
1752	4731	174
1752	4923	532
1752	5605	553
1752	5678	342
1752	6930	738
1752	7170	1003
1752	7324	721
1752	7665	1442
1752	8136	405
1752	8390	4463
1752	8589	1738
1752	9002	626
1752	9007	418

1752	9732	174
1752	10695	182
1752	10738	131
1752	12013	564
1752	16251	649
1752	16442	153
1752	16443	150
1752	16444	148
1753	55	315
1753	65	66
1753	67	2020
1753	89	7126
1753	157	248
1753	228	47
1753	229	42
1753	257	92
1753	288	1521
1753	289	4442
1753	292	472
1753	295	100
1753	299	114
1753	300	347
1753	302	54
1753	306	2382
1753	310	119
1753	328	2910
1753	329	89
1753	331	552
1753	332	79
1753	333	61

1753	352	201
1753	377	220
1753	386	80
1753	461	257
1753	464	226
1753	466	1148
1753	468	80
1753	475	55
1753	485	1092
1753	486	223
1753	526	68
1753	531	3039
1753	543	197
1753	545	65
1753	679	470
1753	689	589
1753	701	59
1753	889	117
1753	1009	81
1753	1032	114
1753	1042	55
1753	1047	64
1753	1456	162
1753	1934	90
1753	3397	129
1753	4024	39
1753	4814	792
1753	7868	62
1754	86	573
1754	154	387

1754	157 424	
1754	163 867	
1754	188 1185	
1754	198 434	
1754	506 1008	
1754	707 3764	
1754	712 342	
1754	812 277	
1754	841 441	
1754	854 295	
1754	917 827	
1754	918 693	
1754	949 546	
1754	954 333	
1754	1110	702
1754	1118	808
1754	1123	282
1754	1131	712
1754	1216	300
1754	1261	502
1754	1274	720
1754	1360	354
1754	1369	292
1754	1639	391
1754	1705	334
1754	1974	324
1754	2251	295
1754	2312	605
1754	2624	750
1754	2836	294

1754	3416	495
1754	3470	267
1754	3940	298
1754	4365	2819
1754	4675	333
1754	4690	286
1754	5157	357
1754	5216	357
1754	5446	339
1754	7791	382
1754	8431	301
1754	8439	495
1754	13991	532
1754	16445	1190
1754	16446	741
1754	16447	630
1754	16448	277
1754	16449	273
1755	56 148	
1755	154 141	
1755	766 173	
1755	1209	135
1755	1221	106
1755	1404	136
1755	1533	146
1755	1950	178
1755	1969	196
1755	1970	107
1755	2026	283
1755	2281	182

1755	2289	202
1755	3443	146
1755	3703	195
1755	4027	107
1755	6409	158
1755	7037	109
1755	9446	134
1755	9457	134
1755	9458	177
1755	10161	134
1755	10164	162
1755	10359	164
1755	10395	183
1755	10672	169
1755	12365	162
1755	14301	152
1755	14670	145
1755	15641	200
1755	16450	280
1755	16451	262
1755	16452	239
1755	16453	178
1755	16454	173
1755	16455	169
1755	16456	167
1755	16457	158
1755	16458	157
1755	16459	151
1755	16460	140
1755	16461	122

1755	16462	121
1755	16463	121
1755	16464	120
1755	16465	115
1755	16466	113
1755	16467	105
1755	16468	105
1755	16469	105
1757	55	77
1757	65	58
1757	80	39
1757	89	1910
1757	288	93
1757	289	61
1757	291	130
1757	292	191
1757	296	54
1757	306	97
1757	314	58
1757	318	40
1757	328	375
1757	329	34
1757	349	151
1757	374	78
1757	377	313
1757	378	42
1757	405	61
1757	430	62
1757	461	38
1757	464	1481

1757	475 67	
1757	498 48	
1757	525 62	
1757	532 612	
1757	680 42	
1757	704 1155	
1757	907 42	
1757	914 563	
1757	1026	130
1757	1179	353
1757	2526	100
1757	4024	259
1757	4561	64
1757	4814	509
1757	5149	1170
1757	5307	191
1757	5537	83
1757	5947	600
1757	6567	42
1757	10684	39
1757	14802	55
1757	16490	321
1757	16491	321
1757	16492	59
1757	16493	50
1757	16494	45
1757	16495	44
1757	16496	35
1758	16497	3
1759	7 587	

1759 198 814
1759 267 391
1759 377 2731
1759 489 1181
1759 491 657
1759 494 359
1759 496 351
1759 497 455
1759 499 357
1759 500 478
1759 510 967
1759 512 1381
1759 514 1332
1759 517 1548
1759 605 3523
1759 779 746
1759 816 356
1759 818 816
1759 1044 340
1759 1099 385
1759 1119 462
1759 1547 588
1759 1613 531
1759 1853 496
1759 1870 1210
1759 1974 539
1759 1979 1613
1759 2252 346
1759 2716 522
1759 2850 357

1759	2919	690
1759	2923	335
1759	2956	426
1759	2976	535
1759	3240	615
1759	3719	564
1759	5040	318
1759	6621	411
1759	7639	1094
1759	8182	377
1759	9821	809
1759	10508	393
1759	14439	431
1759	14692	1017
1759	15103	681
1759	16498	571
1759	16499	432
1759	16500	373
1759	16501	329
1760	56	194
1760	72	128
1760	85	106
1760	98	228
1760	154	106
1760	159	115
1760	163	234
1760	173	609
1760	198	409
1760	233	1162
1760	234	140

1760 310 112

1760 377 363

1760 498 250

1760 519 124

1760 614 114

1760 757 457

1760 768 795

1760 808 116

1760 841 176

1760 854 301

1760 856 226

1760 857 488

1760 868 246

1760 917 339

1760 923 189

1760 993 487

1760 1083 164

1760 1099 303

1760 1104 318

1760 1122 98

1760 1142 128

1760 1179 482

1760 1369 114

1760 1398 256

1760 1409 337

1760 1531 127

1760 1974 274

1760 3444 516

1760 3943 129

1760 4022 130

1760	5890	247
1760	6950	99
1760	7634	126
1760	8069	103
1760	8215	148
1760	10859	144
1760	11042	230
1760	16502	277
1760	16503	130
1761	60	47
1761	69	132
1761	157	86
1761	227	50
1761	263	207
1761	285	68
1761	475	65
1761	511	45
1761	576	51
1761	610	57
1761	613	77
1761	987	230
1761	1427	142
1761	1795	125
1761	1828	245
1761	1829	95
1761	1830	58
1761	1847	54
1761	1848	110
1761	4710	56
1761	10142	52

1761	10145	117
1761	10468	48
1761	10918	54
1761	12115	145
1761	12137	66
1761	13923	74
1761	14004	54
1761	15729	60
1761	15920	232
1761	16504	460
1761	16505	203
1761	16506	198
1761	16507	105
1761	16508	102
1761	16509	100
1761	16510	93
1761	16511	87
1761	16512	83
1761	16513	82
1761	16514	77
1761	16515	71
1761	16516	68
1761	16517	66
1761	16518	56
1761	16519	54
1761	16520	51
1761	16521	48
1761	16522	48
1761	16523	46
1762	67 3260	

1762	89	1200
1762	157	348
1762	161	89
1762	184	1127
1762	207	187
1762	288	339
1762	289	1039
1762	292	252
1762	293	1064
1762	294	670
1762	295	206
1762	300	4795
1762	302	114
1762	306	218
1762	324	586
1762	329	10854
1762	330	489
1762	333	86
1762	344	463
1762	349	812
1762	378	100
1762	424	108
1762	455	127
1762	459	480
1762	464	130
1762	466	478
1762	498	208
1762	528	296
1762	532	161
1762	540	1132

1762 641 86

1762 693 114

1762 699 3128

1762 701 2526

1762 907 623

1762 1032 195

1762 1246 144

1762 1433 180

1762 1985 206

1762 3322 256

1762 3427 103

1762 3712 117

1762 4184 109

1762 5099 193

1762 5235 122

1762 8371 96

1762 13292 106

1762 14600 116

1762 16524 430

1764 154 155

1764 206 114

1764 227 300

1764 231 125

1764 331 180

1764 423 154

1764 435 108

1764 440 124

1764 444 300

1764 445 297

1764 527 144

1764	602 140	
1764	604 131	
1764	609 140	
1764	618 757	
1764	622 141	
1764	703 217	
1764	1164	256
1764	1220	105
1764	1242	241
1764	1369	222
1764	1633	217
1764	1718	146
1764	1804	129
1764	1953	335
1764	2083	109
1764	2139	279
1764	3227	111
1764	3317	135
1764	3693	387
1764	3702	150
1764	3902	110
1764	4450	113
1764	5652	163
1764	6182	124
1764	6374	127
1764	6910	347
1764	8649	136
1764	8701	115
1764	9709	160
1764	11487	235

1764	12183	193
1764	12753	159
1764	14096	164
1764	14435	119
1764	16529	359
1764	16530	169
1764	16531	143
1764	16532	113
1764	16533	110
1765	55	121
1765	56	67
1765	65	62
1765	88	55
1765	89	257
1765	157	45
1765	159	46
1765	199	101
1765	228	134
1765	288	123
1765	289	118
1765	295	68
1765	300	59
1765	306	44
1765	331	71
1765	333	105
1765	377	52
1765	432	54
1765	441	50
1765	445	59
1765	458	91

1765	475	193
1765	548	73
1765	689	142
1765	703	72
1765	757	231
1765	857	69
1765	903	54
1765	906	71
1765	1032	59
1765	1037	277
1765	1090	60
1765	1155	45
1765	1375	90
1765	1398	67
1765	1409	51
1765	1613	75
1765	1934	147
1765	2137	144
1765	2242	152
1765	2582	54
1765	2829	45
1765	3030	218
1765	3201	52
1765	3730	54
1765	4181	54
1765	5071	46
1765	5547	50
1765	9285	155
1765	10615	49
1766	55	151

1766	67	477
1766	88	236
1766	89	1936
1766	285	80
1766	288	558
1766	289	3841
1766	291	391
1766	292	7920
1766	293	112
1766	295	347
1766	300	353
1766	302	715
1766	307	78
1766	310	188
1766	311	86
1766	320	82
1766	323	221
1766	328	164
1766	331	80
1766	333	294
1766	349	149
1766	378	257
1766	461	239
1766	466	315
1766	498	417
1766	502	329
1766	525	313
1766	644	301
1766	680	228
1766	681	148

1766	699	102
1766	701	612
1766	793	403
1766	886	768
1766	906	311
1766	961	103
1766	1034	245
1766	1051	155
1766	1104	102
1766	1450	159
1766	1503	195
1766	1684	125
1766	2018	74
1766	2094	565
1766	3769	78
1766	4707	465
1766	8605	123
1766	16534	106
1766	16535	81
1768	7	2753
1768	89	22867
1768	154	194
1768	207	120
1768	229	299
1768	288	280
1768	289	651
1768	292	170
1768	302	564
1768	306	292
1768	318	93

1768	324 195	
1768	325 116	
1768	328 681	
1768	333 101	
1768	461 296	
1768	466 3746	
1768	486 119	
1768	524 6593	
1768	526 371	
1768	542 153	
1768	544 788	
1768	646 239	
1768	680 1474	
1768	790 126	
1768	797 392	
1768	802 897	
1768	1034	998
1768	1037	326
1768	1041	118
1768	1045	1762
1768	1429	153
1768	1452	123
1768	1475	187
1768	1678	699
1768	2025	129
1768	3200	234
1768	5763	147
1768	6448	2724
1768	7834	351
1768	7994	246

1768	8589	4828
1768	10967	203
1768	12545	3343
1768	16559	676
1768	16560	440
1768	16561	199
1768	16562	183
1768	16563	153
1768	16564	95
1769	97	202
1769	209	3268
1769	230	115
1769	234	210
1769	288	67
1769	289	5713
1769	291	181
1769	292	3469
1769	293	236
1769	298	677
1769	300	2887
1769	302	106
1769	333	64
1769	349	94
1769	377	124
1769	378	241
1769	412	716
1769	424	46
1769	461	266
1769	466	190
1769	497	218

1769	498 318	
1769	523 227	
1769	528 925	
1769	532 174	
1769	540 78	
1769	646 374	
1769	679 60	
1769	680 53	
1769	704 325	
1769	709 63	
1769	790 122	
1769	802 277	
1769	966 57	
1769	972 192	
1769	1034	42
1769	1047	2930
1769	1246	232
1769	1673	4502
1769	1814	503
1769	2546	41
1769	3200	167
1769	5149	57
1769	9684	162
1769	14601	141
1769	15821	104
1769	16565	397
1769	16566	294
1769	16567	54
1769	16568	42
1770	234 390	

1770	306 625	
1770	466 208	
1770	605 925	
1770	619 150	
1770	661 128	
1770	691 260	
1770	777 323	
1770	1099	919
1770	1142	541
1770	1396	236
1770	1398	143
1770	1636	795
1770	2135	292
1770	2179	120
1770	2919	165
1770	2921	344
1770	3355	522
1770	3812	130
1770	4029	419
1770	4181	371
1770	4562	659
1770	4564	133
1770	5071	1597
1770	5115	133
1770	5679	619
1770	5874	132
1770	6387	264
1770	7324	390
1770	7759	441
1770	7963	135

1770	8782	274
1770	9677	183
1770	12839	185
1770	16569	387
1770	16570	314
1770	16571	280
1770	16572	243
1770	16573	228
1770	16574	224
1770	16575	205
1770	16576	200
1770	16577	199
1770	16578	180
1770	16579	145
1770	16580	136
1770	16581	136
1770	16582	133
1770	16583	132
1770	16584	129
1771	55	92
1771	67	730
1771	89	712
1771	157	284
1771	161	113
1771	191	96
1771	257	615
1771	288	304
1771	289	4850
1771	291	134
1771	293	108

1771	300	285
1771	303	146
1771	314	98
1771	318	252
1771	329	104
1771	330	253
1771	331	201
1771	344	232
1771	349	203
1771	360	154
1771	403	129
1771	412	470
1771	455	221
1771	456	95
1771	459	224
1771	461	157
1771	466	568
1771	471	96
1771	488	401
1771	498	186
1771	523	225
1771	527	99
1771	534	99
1771	536	118
1771	538	142
1771	540	1166
1771	913	101
1771	966	482
1771	1043	137
1771	1182	190

1771	1684	156
1771	1866	310
1771	1905	144
1771	2018	98
1771	2523	178
1771	3097	175
1771	4291	189
1771	4762	463
1771	16585	91
1772	56	7094
1772	154	2944
1772	198	519
1772	202	1978
1772	206	580
1772	220	3588
1772	228	2708
1772	230	533
1772	234	2275
1772	250	540
1772	316	644
1772	412	1173
1772	424	2341
1772	433	682
1772	453	1947
1772	534	1631
1772	614	608
1772	898	2492
1772	965	12855
1772	982	1985
1772	1116	5820

1772	1242	525
1772	1406	8883
1772	1643	1072
1772	1833	1032
1772	1974	865
1772	1977	657
1772	2055	638
1772	2346	849
1772	2478	980
1772	2627	1458
1772	2639	2905
1772	2738	720
1772	2840	1505
1772	3475	561
1772	3707	986
1772	4027	2380
1772	4432	3061
1772	5216	979
1772	9261	2782
1772	9262	2822
1772	9585	799
1772	11204	553
1772	13713	3350
1772	14634	2825
1772	16586	1450
1772	16587	824
1772	16588	627
1772	16589	603
1772	16590	598
1773	55 1	

1773	89	1
1773	288	1
1773	292	1
1773	310	1
1773	318	1
1773	328	1
1773	405	1
1773	523	1
1773	538	1
1774	9	111
1774	16	77
1774	173	80
1774	195	57
1774	209	54
1774	378	66
1774	536	33
1774	603	33
1774	615	46
1774	631	36
1774	779	48
1774	835	38
1774	867	37
1774	923	51
1774	1048	36
1774	1075	103
1774	1091	260
1774	1098	30
1774	1100	54
1774	1182	37
1774	1186	32

1774	1201	72
1774	1303	40
1774	1650	48
1774	1672	32
1774	1735	37
1774	2744	41
1774	2840	158
1774	3239	51
1774	3416	34
1774	3551	61
1774	4290	37
1774	4827	32
1774	4833	32
1774	4863	36
1774	5065	50
1774	8065	33
1774	9530	46
1774	10579	42
1774	11186	35
1774	12283	31
1774	12505	35
1774	16591	92
1774	16592	75
1774	16593	67
1774	16594	67
1774	16595	36
1774	16596	34
1774	16597	34
1774	16598	31
1775	51 104	

1775	59	130
1775	72	244
1775	187	286
1775	193	103
1775	230	49
1775	268	63
1775	377	108
1775	405	122
1775	486	131
1775	504	2104
1775	877	133
1775	997	47
1775	998	59
1775	1001	248
1775	1014	428
1775	1064	116
1775	1249	48
1775	1379	47
1775	1406	388
1775	1672	56
1775	1803	106
1775	1914	55
1775	1931	53
1775	2343	437
1775	2358	1444
1775	2359	175
1775	2785	112
1775	2937	62
1775	2940	118
1775	3570	60

1775	3906	584
1775	3907	103
1775	3909	812
1775	3910	93
1775	3911	165
1775	3912	70
1775	3916	119
1775	3919	163
1775	3922	61
1775	5729	58
1775	6235	124
1775	9869	106
1775	16599	165
1775	16600	142
1775	16601	103
1775	16602	95
1775	16603	78
1775	16604	74
1775	16605	51
1776	67	257
1776	89	6250
1776	230	240
1776	288	198
1776	289	1208
1776	291	104
1776	292	2363
1776	300	216
1776	317	80
1776	318	135
1776	325	1000

1776	333	1488
1776	344	3800
1776	352	141
1776	375	145
1776	377	1550
1776	400	94
1776	461	1071
1776	466	1026
1776	487	523
1776	498	126
1776	528	230
1776	532	71
1776	544	222
1776	548	156
1776	635	1135
1776	679	301
1776	686	1142
1776	704	2102
1776	790	2015
1776	797	117
1776	802	130
1776	966	172
1776	1034	205
1776	1047	84
1776	1186	69
1776	1428	112
1776	1673	157
1776	1688	614
1776	1934	79
1776	2020	1006

1776	2091	1084
1776	2094	109
1776	2432	434
1776	2978	69
1776	3200	68
1776	3404	295
1776	4814	77
1776	9199	159
1776	16606	72
1777	72	3455
1777	153	542
1777	190	287
1777	450	686
1777	526	337
1777	1028	338
1777	1062	256
1777	1075	1206
1777	1084	4388
1777	1486	336
1777	3988	951
1777	5484	247
1777	7810	718
1777	9869	240
1777	11333	254
1777	12218	510
1777	12552	939
1777	13181	652
1777	13182	15087
1777	13183	1409
1777	13184	819

1777	13185	817
1777	13186	1000
1777	13187	723
1777	13189	359
1777	13190	353
1777	13192	227
1777	13200	342
1777	13203	666
1777	13206	560
1777	13209	1589
1777	13637	330
1777	16607	1320
1777	16608	1162
1777	16609	1052
1777	16610	966
1777	16611	861
1777	16612	733
1777	16613	604
1777	16614	521
1777	16615	401
1777	16616	373
1777	16617	341
1777	16618	326
1777	16619	287
1777	16620	282
1777	16621	274
1777	16622	262
1777	16623	235
1777	16624	234
1778	65	10453

1778	72	722
1778	152	1329
1778	154	8189
1778	171	1415
1778	173	716
1778	182	549
1778	190	3648
1778	198	1064
1778	207	1626
1778	208	467
1778	210	609
1778	220	1269
1778	221	640
1778	229	777
1778	234	383
1778	237	499
1778	298	692
1778	301	268
1778	377	337
1778	424	405
1778	428	705
1778	432	453
1778	475	593
1778	533	2984
1778	615	1981
1778	709	798
1778	716	1836
1778	851	985
1778	959	277
1778	969	1167

1778	1048	688
1778	1090	1212
1778	1098	832
1778	1131	512
1778	1151	508
1778	1243	397
1778	1375	953
1778	1381	598
1778	1515	693
1778	1520	635
1778	2600	479
1778	2614	406
1778	2815	626
1778	3676	271
1778	5020	1079
1778	7187	477
1778	7323	579
1778	9160	379
1778	16625	295
1779	407 779	
1779	506 135	
1779	755 247	
1779	977 176	
1779	1134	310
1779	1143	84
1779	1206	195
1779	1344	141
1779	1362	126
1779	2573	112
1779	3417	169

1779	3456	166
1779	3780	393
1779	3788	119
1779	3792	80
1779	4182	90
1779	4611	122
1779	4693	121
1779	4970	78
1779	7331	441
1779	7895	145
1779	10672	135
1779	10820	118
1779	10831	172
1779	11475	95
1779	11483	79
1779	12094	90
1779	13478	79
1779	13491	109
1779	16437	129
1779	16626	481
1779	16627	299
1779	16628	255
1779	16629	232
1779	16630	215
1779	16631	203
1779	16632	174
1779	16633	173
1779	16634	169
1779	16635	166
1779	16636	140

1779	16637	139
1779	16638	121
1779	16639	112
1779	16640	109
1779	16641	108
1779	16642	105
1779	16643	103
1779	16644	93
1779	16645	82
1780	55	361
1780	67	368
1780	89	20265
1780	257	509
1780	288	3169
1780	289	8083
1780	290	292
1780	291	496
1780	292	5249
1780	295	1583
1780	299	351
1780	300	1712
1780	302	606
1780	306	526
1780	317	437
1780	318	600
1780	323	4273
1780	325	7429
1780	333	300
1780	344	1757
1780	349	1027

1780	352	574
1780	461	1694
1780	466	3869
1780	485	1100
1780	498	1858
1780	499	177
1780	523	490
1780	524	593
1780	528	1126
1780	532	231
1780	543	799
1780	544	1966
1780	679	2055
1780	680	553
1780	681	5466
1780	686	2079
1780	693	1096
1780	701	168
1780	797	214
1780	908	405
1780	1037	653
1780	1450	1137
1780	1684	1994
1780	2094	543
1780	2102	1864
1780	2392	344
1780	2521	284
1780	2801	639
1780	3397	1442
1781	65	240

1781	99	351
1781	193	155
1781	220	35
1781	227	300
1781	302	44
1781	366	274
1781	511	755
1781	565	35
1781	572	138
1781	583	87
1781	599	53
1781	735	42
1781	868	61
1781	959	41
1781	982	52
1781	1242	59
1781	1244	80
1781	1382	101
1781	1415	74
1781	2544	37
1781	2627	118
1781	2842	61
1781	2893	64
1781	3889	116
1781	4251	60
1781	4928	52
1781	6400	149
1781	6678	481
1781	7857	220
1781	8936	35

1781	9321	105
1781	11023	47
1781	12726	63
1781	13517	153
1781	16646	308
1781	16647	133
1781	16648	121
1781	16649	115
1781	16650	101
1781	16651	83
1781	16652	75
1781	16653	71
1781	16654	67
1781	16655	67
1781	16656	63
1781	16657	52
1781	16658	43
1781	16659	41
1781	16660	36
1782	15	36
1782	224	48
1782	234	84
1782	238	26
1782	256	27
1782	302	94
1782	333	209
1782	370	67
1782	475	45
1782	529	49
1782	535	32

1782	680	107
1782	734	44
1782	806	58
1782	821	37
1782	949	48
1782	953	47
1782	1065	71
1782	1274	28
1782	1276	110
1782	1339	39
1782	1710	93
1782	1732	32
1782	1858	103
1782	1941	38
1782	1957	28
1782	2521	97
1782	2716	44
1782	2745	34
1782	2830	37
1782	2970	185
1782	3288	51
1782	3502	115
1782	3800	67
1782	3806	98
1782	3825	40
1782	3901	50
1782	3991	300
1782	4180	27
1782	4611	31
1782	5421	30

1782	5689	42
1782	8251	51
1782	9234	37
1782	12051	39
1782	12707	56
1782	16661	102
1782	16662	39
1782	16663	35
1782	16664	34
1783	56	357
1783	59	489
1783	159	432
1783	163	1497
1783	170	446
1783	176	468
1783	190	240
1783	207	1956
1783	212	1129
1783	220	212
1783	226	266
1783	227	3844
1783	228	399
1783	229	281
1783	234	1184
1783	366	390
1783	421	1244
1783	424	450
1783	453	165
1783	498	678
1783	533	218

1783	562	1290
1783	599	445
1783	614	5605
1783	705	897
1783	706	1086
1783	707	280
1783	714	763
1783	854	242
1783	857	166
1783	868	180
1783	924	555
1783	982	782
1783	1090	169
1783	1110	496
1783	1119	168
1783	1412	1060
1783	1426	255
1783	1639	200
1783	1873	322
1783	1905	175
1783	2183	248
1783	2347	195
1783	2606	633
1783	2834	362
1783	3797	896
1783	4349	327
1783	5226	152
1783	12911	235
1783	16665	237
1784	65	403

1784	207 3007	
1784	227 521	
1784	229 430	
1784	246 629	
1784	261 313	
1784	289 499	
1784	298 1437	
1784	324 883	
1784	333 965	
1784	342 384	
1784	355 381	
1784	366 779	
1784	403 330	
1784	424 582	
1784	438 562	
1784	439 1321	
1784	470 490	
1784	472 349	
1784	498 3596	
1784	533 314	
1784	538 545	
1784	683 991	
1784	687 447	
1784	903 758	
1784	1038	355
1784	1040	618
1784	1046	299
1784	1048	586
1784	1105	356
1784	1106	353

1784	1193	861
1784	1201	1245
1784	1241	371
1784	1247	626
1784	1377	585
1784	1775	778
1784	1866	500
1784	3078	417
1784	3488	380
1784	3743	1108
1784	3747	379
1784	3748	542
1784	4223	625
1784	4716	1033
1784	4818	876
1784	6453	384
1784	6600	4443
1784	10918	381
1784	11550	1754
1785	53	986
1785	57	4318
1785	59	881
1785	64	953
1785	65	2361
1785	69	1495
1785	70	817
1785	84	862
1785	154	16312
1785	159	804
1785	163	809

1785	173 4328	
1785	174 1889	
1785	190 3046	
1785	199 1992	
1785	203 1272	
1785	206 1624	
1785	207 2505	
1785	210 880	
1785	221 1546	
1785	225 2855	
1785	226 1069	
1785	228 2190	
1785	229 2236	
1785	234 1280	
1785	237 2278	
1785	424 1193	
1785	425 1296	
1785	603 858	
1785	619 2388	
1785	703 986	
1785	706 2163	
1785	709 1068	
1785	715 3551	
1785	851 982	
1785	969 1098	
1785	1089	841
1785	1376	1084
1785	1518	881
1785	1622	1413
1785	1738	835

1785	1824	1226
1785	2137	982
1785	3334	1016
1785	4720	1577
1785	5358	3805
1785	11598	983
1785	16666	1077
1785	16667	977
1785	16668	825
1786	89	339
1786	191	270
1786	257	606
1786	288	159
1786	289	214
1786	292	84
1786	294	53
1786	300	100
1786	301	111
1786	317	110
1786	344	48
1786	374	262
1786	470	91
1786	679	327
1786	691	70
1786	693	57
1786	1035	269
1786	1037	81
1786	1038	240
1786	1042	81
1786	1043	211

1786	1239	59
1786	1389	137
1786	1447	61
1786	1481	255
1786	1680	166
1786	1936	141
1786	2068	150
1786	2176	92
1786	2607	2192
1786	2653	100
1786	2939	128
1786	2982	136
1786	3404	48
1786	3459	76
1786	4741	99
1786	5925	108
1786	5958	323
1786	5988	101
1786	6769	114
1786	7324	54
1786	8674	56
1786	9081	145
1786	11731	99
1786	16669	133
1786	16670	93
1786	16671	81
1786	16672	71
1786	16673	51
1786	16674	50
1787	99 157	

1787	154 232	
1787	166 488	
1787	173 468	
1787	190 390	
1787	198 864	
1787	203 263	
1787	220 199	
1787	226 423	
1787	227 741	
1787	234 2238	
1787	436 806	
1787	441 383	
1787	504 153	
1787	511 304	
1787	512 203	
1787	517 210	
1787	533 171	
1787	614 191	
1787	715 755	
1787	733 159	
1787	853 442	
1787	854 1222	
1787	856 150	
1787	859 158	
1787	868 686	
1787	917 194	
1787	952 161	
1787	965 166	
1787	982 912	
1787	1044	276

1787	1089	684
1787	1122	167
1787	1202	245
1787	1249	444
1787	1412	213
1787	1504	675
1787	1513	146
1787	1817	269
1787	2495	148
1787	3279	188
1787	3739	309
1787	4115	302
1787	4359	265
1787	8042	169
1787	12496	366
1787	15128	153
1787	16675	399
1787	16676	175
1787	16677	141
1788	89	1990
1788	288	1130
1788	289	4020
1788	291	739
1788	292	268
1788	293	237
1788	295	330
1788	299	227
1788	300	1365
1788	306	449
1788	318	542

1788	325 493	
1788	333 791	
1788	344 470	
1788	349 185	
1788	458 502	
1788	461 1617	
1788	464 252	
1788	466 844	
1788	481 164	
1788	486 215	
1788	498 353	
1788	523 483	
1788	528 248	
1788	544 719	
1788	646 873	
1788	679 3202	
1788	680 263	
1788	681 426	
1788	686 290	
1788	701 710	
1788	704 195	
1788	790 170	
1788	802 811	
1788	972 618	
1788	1043	229
1788	1045	149
1788	1047	408
1788	1428	217
1788	1453	191
1788	1934	208

1788	2020	364
1788	2080	284
1788	2548	266
1788	2801	255
1788	4350	263
1788	4474	170
1788	4870	283
1788	5749	641
1788	5752	457
1789	65	1044
1789	84	817
1789	182	2143
1789	205	752
1789	217	900
1789	221	1108
1789	416	1098
1789	433	807
1789	446	988
1789	604	882
1789	1400	877
1789	1633	1184
1789	2139	1941
1789	2280	1059
1789	2533	1018
1789	2538	852
1789	2601	1241
1789	2938	984
1789	3258	1578
1789	3468	804
1789	3706	1405

1789	5414	951
1789	6205	3196
1789	6210	1133
1789	6217	2744
1789	6728	1215
1789	7667	888
1789	8493	1155
1789	8591	1456
1789	8830	976
1789	8864	940
1789	8867	1014
1789	9477	924
1789	9823	1336
1789	11229	1902
1789	13419	2160
1789	13516	2250
1789	13610	1123
1789	16678	1766
1789	16679	1439
1789	16680	1271
1789	16681	1175
1789	16682	1113
1789	16683	903
1789	16684	868
1789	16685	821
1789	16686	797
1789	16687	794
1789	16688	776
1789	16689	772
1790	55 148	

1790	67	256
1790	97	204
1790	157	163
1790	204	150
1790	227	274
1790	266	258
1790	270	219
1790	289	169
1790	300	255
1790	301	146
1790	331	261
1790	424	157
1790	425	146
1790	437	191
1790	445	140
1790	484	140
1790	602	279
1790	604	273
1790	622	269
1790	689	148
1790	697	250
1790	858	202
1790	898	145
1790	903	181
1790	985	137
1790	989	172
1790	1090	155
1790	1106	178
1790	1400	265
1790	1764	159

1790	1772	150
1790	1934	200
1790	2083	251
1790	2277	271
1790	2460	266
1790	2638	259
1790	3293	144
1790	3488	177
1790	4447	154
1790	4704	156
1790	5789	184
1790	5790	309
1790	6137	137
1790	7123	186
1790	8594	241
1790	11764	285
1790	16690	228
1790	16691	193
1790	16692	147
1791	55	453
1791	67	97
1791	239	181
1791	241	177
1791	252	175
1791	257	5886
1791	265	105
1791	279	331
1791	285	200
1791	288	1472
1791	289	731

1791	291	1279
1791	292	124
1791	293	823
1791	294	442
1791	295	164
1791	299	1178
1791	301	125
1791	305	103
1791	308	111
1791	310	1494
1791	311	242
1791	314	123
1791	318	507
1791	320	128
1791	332	377
1791	344	441
1791	349	109
1791	465	237
1791	523	128
1791	525	212
1791	534	130
1791	701	103
1791	891	304
1791	892	108
1791	913	179
1791	1052	287
1791	1459	879
1791	1556	113
1791	1934	180
1791	1936	224

1791	2220	180
1791	2454	240
1791	3460	227
1791	3489	578
1791	4566	803
1791	8076	388
1791	10677	348
1791	11082	123
1791	16693	242
1792	163 630	
1792	176 421	
1792	212 578	
1792	227 1602	
1792	562 1078	
1792	565 507	
1792	580 502	
1792	583 359	
1792	585 273	
1792	599 537	
1792	607 615	
1792	707 366	
1792	728 344	
1792	732 213	
1792	735 319	
1792	863 643	
1792	868 636	
1792	959 720	
1792	1358	420
1792	1369	1142
1792	1372	862

1792	1412	2596
1792	1413	686
1792	1415	295
1792	1426	281
1792	1639	359
1792	1757	597
1792	1806	319
1792	2342	292
1792	2786	391
1792	2787	348
1792	2855	1036
1792	2864	287
1792	3470	306
1792	3475	215
1792	3481	1186
1792	3776	220
1792	4251	417
1792	4675	372
1792	4784	515
1792	4790	297
1792	5416	1166
1792	5660	245
1792	5844	232
1792	7047	1055
1792	7056	288
1792	8111	254
1792	8126	500
1792	8815	492
1792	13123	474
1793	54 689	

1793	56	396
1793	81	1069
1793	230	668
1793	238	2398
1793	250	768
1793	298	352
1793	330	1095
1793	439	1073
1793	475	455
1793	547	740
1793	972	1190
1793	1075	2464
1793	1241	391
1793	1402	909
1793	1433	794
1793	1449	451
1793	1613	765
1793	2222	1927
1793	2226	609
1793	2236	531
1793	2916	533
1793	2919	339
1793	2923	349
1793	2976	523
1793	3109	4340
1793	3227	457
1793	3316	379
1793	3384	586
1793	4316	2419
1793	4344	800

1793	4586	970
1793	5119	462
1793	5184	843
1793	5891	528
1793	6288	2148
1793	6626	1437
1793	7766	592
1793	8159	352
1793	8160	698
1793	14840	427
1793	15102	1239
1793	15923	1064
1793	16498	653
1793	16694	729
1793	16695	689
1793	16696	517
1793	16697	481
1793	16698	449
1793	16699	423
1794	173 68	
1794	234 46	
1794	377 67	
1794	492 43	
1794	493 1508	
1794	494 81	
1794	500 53	
1794	507 106	
1794	1182	37
1794	1184	129
1794	1200	168

1794	1379	70
1794	1470	41
1794	1874	90
1794	2366	40
1794	2845	216
1794	2847	74
1794	3375	394
1794	3428	432
1794	4196	94
1794	6450	43
1794	7187	61
1794	7776	193
1794	7777	66
1794	8876	82
1794	8884	150
1794	9934	88
1794	9936	1607
1794	10038	2499
1794	10784	242
1794	12620	552
1794	13377	39
1794	15218	46
1794	15448	142
1794	16352	97
1794	16700	782
1794	16701	410
1794	16702	364
1794	16703	360
1794	16704	234
1794	16705	188

1794	16706	168
1794	16707	110
1794	16708	107
1794	16709	84
1794	16710	62
1794	16711	51
1794	16712	47
1794	16713	39
1794	16714	36
1795	55	265
1795	65	388
1795	67	642
1795	89	308
1795	97	153
1795	154	755
1795	155	293
1795	157	591
1795	190	186
1795	199	374
1795	229	274
1795	257	174
1795	265	192
1795	285	345
1795	288	411
1795	289	594
1795	292	202
1795	295	171
1795	296	257
1795	298	216
1795	299	160

1795	300	334
1795	301	213
1795	302	348
1795	306	160
1795	310	197
1795	352	250
1795	377	182
1795	389	216
1795	418	329
1795	458	137
1795	461	190
1795	465	227
1795	466	225
1795	468	145
1795	475	236
1795	485	300
1795	532	263
1795	548	174
1795	686	212
1795	689	187
1795	690	141
1795	906	440
1795	1098	563
1795	1390	200
1795	1400	349
1795	2083	277
1795	2521	185
1795	4566	168
1795	4710	189
1796	89	2326

1796	207	522
1796	209	664
1796	225	101
1796	228	807
1796	229	569
1796	230	695
1796	306	112
1796	324	300
1796	378	288
1796	425	112
1796	472	385
1796	481	126
1796	498	718
1796	526	149
1796	533	299
1796	536	250
1796	540	109
1796	546	1103
1796	980	136
1796	1036	152
1796	1043	135
1796	1090	189
1796	1106	823
1796	1155	403
1796	1189	130
1796	1246	6936
1796	1375	224
1796	1376	847
1796	1400	123
1796	1613	154

1796	2206	267
1796	2602	107
1796	2610	956
1796	2840	357
1796	3427	121
1796	3730	561
1796	4074	1017
1796	5027	154
1796	5544	379
1796	5547	105
1796	5926	1189
1796	6197	838
1796	6453	2602
1796	14736	257
1796	16715	373
1796	16716	198
1796	16717	148
1796	16718	145
1796	16719	119
1797	65	1408
1797	154	1194
1797	163	291
1797	173	1004
1797	182	171
1797	190	3524
1797	191	606
1797	199	244
1797	212	196
1797	217	448
1797	225	491

1797	226	238
1797	227	624
1797	228	1486
1797	229	2031
1797	355	129
1797	377	851
1797	418	248
1797	429	265
1797	441	354
1797	472	312
1797	486	510
1797	488	267
1797	622	189
1797	683	639
1797	703	170
1797	706	134
1797	707	155
1797	716	145
1797	718	135
1797	859	153
1797	964	376
1797	966	227
1797	982	199
1797	1116	266
1797	1375	570
1797	1376	490
1797	1377	359
1797	1414	144
1797	1518	141
1797	1854	696

1797	1873	171
1797	1974	168
1797	1988	216
1797	2535	204
1797	2607	276
1797	3255	162
1797	3400	388
1797	6231	169
1797	8155	218
1798	53	27
1798	56	192
1798	59	26
1798	63	92
1798	65	45
1798	66	20
1798	72	1869
1798	75	35
1798	84	27
1798	154	90
1798	157	354
1798	163	36
1798	173	409
1798	190	159
1798	227	137
1798	228	39
1798	237	34
1798	238	670
1798	377	19
1798	418	32
1798	526	76

1798	562	36
1798	930	51
1798	936	157
1798	961	127
1798	1001	269
1798	1061	99
1798	1072	124
1798	1099	83
1798	1104	27
1798	1121	29
1798	1151	20
1798	1394	20
1798	1412	54
1798	1504	32
1798	1505	19
1798	1510	27
1798	1713	153
1798	1787	130
1798	1892	19
1798	2681	139
1798	3502	36
1798	4913	46
1798	5098	61
1798	5475	32
1798	9783	37
1798	12114	30
1798	16720	33
1798	16721	32
1798	16722	26
1799	55	134

1799	89	470
1799	157	260
1799	239	134
1799	251	141
1799	257	1002
1799	262	174
1799	285	434
1799	288	823
1799	289	509
1799	290	297
1799	292	406
1799	294	224
1799	295	290
1799	299	276
1799	300	246
1799	306	547
1799	308	276
1799	314	492
1799	317	261
1799	327	156
1799	328	243
1799	349	184
1799	367	187
1799	400	273
1799	466	427
1799	468	331
1799	485	168
1799	531	174
1799	545	137
1799	1037	343

1799	1449	155
1799	2083	141
1799	2091	165
1799	2092	184
1799	3318	162
1799	3330	188
1799	4562	147
1799	5947	195
1799	6562	174
1799	16670	325
1799	16723	217
1799	16724	134
1800	72	38
1800	174	7
1800	190	5
1800	238	10
1800	298	7
1800	349	4
1800	532	13
1800	539	8
1800	968	9
1800	1020	24
1800	1029	34
1800	1075	9
1800	1326	11
1800	1379	6
1800	1401	150
1800	1405	10
1800	1713	12
1800	2130	17

1800	3868	93
1800	4024	6
1800	4025	11
1800	4036	4
1800	4038	6
1800	5072	4
1800	5149	4
1800	5534	10
1800	5978	5
1800	6547	5
1800	6785	4
1800	6878	4
1800	8144	12
1800	8784	4
1800	10703	7
1800	11194	7
1800	13187	16
1800	16725	14
1800	16726	7
1800	16727	7
1800	16728	6
1800	16729	5
1800	16730	5
1800	16731	5
1800	16732	5
1800	16733	5
1800	16734	5
1800	16735	4
1800	16736	4
1800	16737	4

1800	16738	4
1800	16739	4
1801	53 48	
1801	603 62	
1801	1824	32
1801	1835	57
1801	1945	44
1801	1965	56
1801	2161	30
1801	2667	70
1801	2668	31
1801	2671	56
1801	2673	109
1801	2674	58
1801	2688	56
1801	2690	45
1801	2691	36
1801	3695	52
1801	3707	36
1801	4029	45
1801	4113	42
1801	4230	48
1801	5043	70
1801	6185	76
1801	6404	40
1801	6582	71
1801	6762	32
1801	6763	57
1801	7466	41
1801	10523	31

1801	11206	44
1801	15247	39
1801	16057	37
1801	16367	65
1801	16740	98
1801	16741	85
1801	16742	67
1801	16743	66
1801	16744	63
1801	16745	55
1801	16746	52
1801	16747	48
1801	16748	44
1801	16749	44
1801	16750	39
1801	16751	38
1801	16752	35
1801	16753	35
1801	16754	33
1801	16755	33
1801	16756	32
1801	16757	31
1802	65	391
1802	217	1380
1802	220	405
1802	229	409
1802	330	424
1802	366	867
1802	412	582
1802	498	453

1802	540 873	
1802	615 466	
1802	622 471	
1802	683 379	
1802	1046	560
1802	1105	441
1802	1116	506
1802	1246	759
1802	1375	1210
1802	1377	797
1802	1378	875
1802	1643	343
1802	1866	375
1802	2425	333
1802	2439	453
1802	2627	733
1802	2639	563
1802	2840	1625
1802	3258	334
1802	3341	3374
1802	3373	414
1802	3460	339
1802	3464	583
1802	3616	635
1802	3754	2186
1802	4114	345
1802	4118	516
1802	4262	760
1802	4385	476
1802	4762	433

1802	4837	691
1802	5437	774
1802	6298	631
1802	6452	656
1802	6600	606
1802	6602	1125
1802	6769	390
1802	6773	1193
1802	9423	337
1802	11550	1163
1802	16758	653
1802	16759	331
1803	318 106	
1803	326 112	
1803	344 155	
1803	352 132	
1803	354 191	
1803	360 754	
1803	465 442	
1803	677 275	
1803	679 399	
1803	681 114	
1803	686 486	
1803	790 196	
1803	791 501	
1803	792 686	
1803	794 115	
1803	798 130	
1803	1464	278
1803	2024	713

1803	2076	176
1803	2454	99
1803	2800	107
1803	3177	315
1803	3199	127
1803	3397	394
1803	3497	207
1803	4481	113
1803	5775	219
1803	6308	129
1803	6713	144
1803	8844	218
1803	8850	114
1803	14515	934
1803	15941	148
1803	16760	4077
1803	16761	455
1803	16762	385
1803	16763	341
1803	16764	274
1803	16765	257
1803	16766	222
1803	16767	165
1803	16768	157
1803	16769	133
1803	16770	131
1803	16771	119
1803	16772	116
1803	16773	109
1803	16774	103

1803	16775	101
1803	16776	100
1804	154 127	
1804	173 738	
1804	190 98	
1804	207 178	
1804	209 141	
1804	220 104	
1804	227 129	
1804	228 88	
1804	229 270	
1804	377 83	
1804	403 256	
1804	412 164	
1804	424 148	
1804	439 85	
1804	472 105	
1804	481 116	
1804	486 491	
1804	492 167	
1804	493 108	
1804	494 174	
1804	498 155	
1804	533 293	
1804	536 100	
1804	537 438	
1804	540 205	
1804	708 201	
1804	714 171	
1804	1011	104

1804	1043	123
1804	1048	84
1804	1116	79
1804	1186	207
1804	1195	80
1804	1239	222
1804	1246	107
1804	1375	136
1804	1377	115
1804	1681	181
1804	1775	83
1804	1866	622
1804	1874	234
1804	2080	109
1804	2346	108
1804	3200	93
1804	3382	118
1804	3741	187
1804	6120	103
1804	6127	82
1804	8873	117
1804	16777	81
1805	126 526	
1805	154 1965	
1805	163 751	
1805	190 365	
1805	204 252	
1805	206 168	
1805	209 298	
1805	212 1818	

1805	216	1795
1805	222	1623
1805	227	5617
1805	231	418
1805	234	229
1805	235	494
1805	237	571
1805	238	134
1805	331	213
1805	383	436
1805	418	154
1805	436	514
1805	554	696
1805	605	147
1805	615	1367
1805	706	148
1805	733	127
1805	858	391
1805	903	278
1805	964	304
1805	1091	638
1805	1414	151
1805	1957	243
1805	1958	153
1805	2276	137
1805	2542	365
1805	3225	302
1805	3259	747
1805	4092	232
1805	5205	136

1805	5636	220
1805	6182	193
1805	7375	171
1805	8656	987
1805	11598	276
1805	16778	1211
1805	16779	540
1805	16780	538
1805	16781	229
1805	16782	206
1805	16783	158
1805	16784	148
1806	81	493
1806	89	280
1806	154	1330
1806	171	1185
1806	173	254
1806	210	529
1806	217	528
1806	227	504
1806	237	212
1806	331	252
1806	416	1526
1806	418	3104
1806	429	209
1806	444	441
1806	454	1332
1806	486	220
1806	527	230
1806	533	694

1806	547 202	
1806	618 350	
1806	622 1310	
1806	734 259	
1806	744 362	
1806	757 409	
1806	898 8465	
1806	1097	298
1806	1098	338
1806	1163	595
1806	1412	315
1806	1732	678
1806	1747	217
1806	2026	298
1806	2135	249
1806	2591	267
1806	3030	1947
1806	3046	236
1806	3317	785
1806	3411	1368
1806	6151	901
1806	6229	213
1806	6296	232
1806	6756	268
1806	7643	224
1806	8027	298
1806	8418	510
1806	9955	194
1806	11416	304
1806	16785	695

1806	16786	259
1806	16787	249
1807	154 2	
1807	195 10	
1807	217 12	
1807	418 21	
1807	632 9	
1807	751 11	
1807	763 12	
1807	1398	3
1807	1418	6
1807	1541	2
1807	2276	5
1807	2429	6
1807	2582	6
1807	3028	8
1807	3686	5
1807	4086	36
1807	4271	5
1807	5966	4
1807	6307	6
1807	6759	44
1807	6906	7
1807	7665	3
1807	8480	54
1807	9666	13
1807	10449	22
1807	11604	75
1807	11606	5
1807	11609	2

1807	11612	2
1807	11613	17
1807	11614	23
1807	12487	24
1807	12980	3
1807	13629	8
1807	14208	2
1807	16788	19
1807	16789	11
1807	16790	9
1807	16791	9
1807	16792	8
1807	16793	7
1807	16794	6
1807	16795	6
1807	16796	6
1807	16797	5
1807	16798	5
1807	16799	4
1807	16800	4
1807	16801	4
1807	16802	3
1808	55	322
1808	65	365
1808	67	843
1808	89	883
1808	157	567
1808	182	357
1808	191	342
1808	257	1318

1808	288 1840
1808	289 2292
1808	290 382
1808	291 427
1808	292 486
1808	293 413
1808	294 426
1808	295 1280
1808	298 467
1808	299 466
1808	300 704
1808	302 490
1808	304 542
1808	306 2091
1808	311 842
1808	314 433
1808	320 685
1808	322 496
1808	325 494
1808	327 488
1808	328 340
1808	330 538
1808	333 1464
1808	342 338
1808	349 1029
1808	377 354
1808	456 330
1808	461 417
1808	468 383
1808	472 579

1808 479 429

1808 538 412

1808 543 818

1808 545 824

1808 701 525

1808 908 321

1808 966 355

1808 1444 388

1808 1459 362

1808 1470 946

1808 2941 348

1808 5988 424

1809 229 284

1809 230 5246

1809 377 540

1809 383 1100

1809 405 870

1809 412 213

1809 481 241

1809 488 319

1809 489 235

1809 497 590

1809 498 351

1809 501 253

1809 510 247

1809 518 220

1809 536 256

1809 708 400

1809 724 194

1809 857 217

1809	917 880	
1809	924 1230	
1809	925 217	
1809	930 329	
1809	932 263	
1809	939 200	
1809	978 307	
1809	1041	228
1809	1044	690
1809	1206	1064
1809	1274	324
1809	1339	388
1809	1351	307
1809	1360	325
1809	1369	371
1809	1406	1810
1809	1513	255
1809	1650	964
1809	1672	700
1809	1854	1156
1809	1874	205
1809	1974	229
1809	2343	445
1809	2352	432
1809	2435	580
1809	3205	206
1809	3360	394
1809	4218	650
1809	5310	360
1809	6006	257

1809	10404	343
1809	16803	283
1810	89	160
1810	268	1
1810	288	27486
1810	289	7
1810	300	1850
1810	306	1
1810	330	1
1810	349	1
1810	355	1
1810	466	8
1810	475	358
1810	701	162
1810	891	136
1810	907	1
1810	913	1
1810	1441	2
1810	1760	12
1810	2047	1
1810	3461	1
1810	5790	1
1810	9417	1
1810	16804	1
1811	67	1550
1811	89	1769
1811	161	2266
1811	257	579
1811	262	403
1811	283	221

1811	288 820	
1811	289 2784	
1811	293 815	
1811	295 34243	
1811	298 399	
1811	299 1130	
1811	300 373	
1811	301 491	
1811	332 3739	
1811	333 362	
1811	334 520	
1811	349 533	
1811	350 5525	
1811	378 515	
1811	542 6214	
1811	543 1028	
1811	646 3377	
1811	701 1024	
1811	792 614	
1811	802 231	
1811	903 1669	
1811	913 8217	
1811	972 2202	
1811	1613	330
1811	1937	275
1811	2094	931
1811	2523	734
1811	2984	4642
1811	3173	251
1811	4350	1310

1811	4719	224
1811	5036	217
1811	5468	208
1811	5774	424
1811	6308	326
1811	8360	528
1811	13164	353
1811	16564	2209
1811	16805	2467
1811	16806	1906
1811	16807	1383
1811	16808	302
1811	16809	237
1811	16810	234
1813	55	449
1813	65	185
1813	67	304
1813	89	2662
1813	288	2110
1813	289	491
1813	291	455
1813	292	6574
1813	295	592
1813	298	202
1813	300	6371
1813	301	114
1813	302	586
1813	323	169
1813	332	237
1813	333	414

1813	344 766	
1813	349 134	
1813	352 1235	
1813	386 152	
1813	461 508	
1813	464 270	
1813	523 172	
1813	528 652	
1813	532 145	
1813	543 102	
1813	544 1076	
1813	548 317	
1813	679 2411	
1813	681 118	
1813	686 406	
1813	689 1072	
1813	697 189	
1813	802 217	
1813	1098	151
1813	1243	475
1813	1400	126
1813	1451	128
1813	1455	281
1813	1464	446
1813	1684	116
1813	1934	523
1813	2543	305
1813	2657	202
1813	2981	120
1813	2990	432

1813	3171	160
1813	5150	695
1813	5472	208
1813	16832	147
1814	65	99
1814	72	400
1814	154	149
1814	159	464
1814	182	209
1814	190	1687
1814	207	455
1814	210	178
1814	225	405
1814	226	204
1814	227	110
1814	229	3037
1814	234	177
1814	424	445
1814	533	826
1814	562	271
1814	614	182
1814	683	274
1814	716	155
1814	860	123
1814	969	619
1814	982	153
1814	1090	141
1814	1098	235
1814	1122	118
1814	1155	351

1814	1246	101
1814	1249	199
1814	1360	220
1814	1369	132
1814	1513	278
1814	1515	182
1814	1520	142
1814	1669	267
1814	2121	192
1814	2343	2096
1814	2347	100
1814	2600	143
1814	2789	210
1814	2938	211
1814	3427	96
1814	4435	927
1814	4455	95
1814	4793	98
1814	7187	225
1814	13844	107
1814	16833	376
1814	16834	266
1814	16835	250
1814	16836	120
1815	65	314
1815	154	670
1815	163	283
1815	192	471
1815	195	300
1815	196	314

1815	199	445
1815	215	498
1815	221	267
1815	223	301
1815	227	1024
1815	265	487
1815	270	546
1815	288	318
1815	316	396
1815	418	477
1815	429	1311
1815	439	649
1815	615	413
1815	618	767
1815	714	504
1815	715	378
1815	900	330
1815	903	347
1815	982	419
1815	1089	338
1815	1116	359
1815	1167	431
1815	1384	339
1815	1421	315
1815	1519	1060
1815	1934	294
1815	1958	275
1815	2531	735
1815	2632	938
1815	3676	384

1815	4079	424
1815	4442	455
1815	4531	470
1815	5790	436
1815	7525	463
1815	8591	359
1815	8864	329
1815	11229	411
1815	11900	557
1815	13517	267
1815	16385	493
1815	16837	667
1815	16838	282
1815	16839	280
1816	72	176
1816	166	275
1816	220	170
1816	250	166
1816	261	259
1816	366	234
1816	422	229
1816	429	283
1816	430	389
1816	432	211
1816	452	440
1816	510	207
1816	683	242
1816	712	254
1816	715	563
1816	903	218

1816	1009	263
1816	1099	175
1816	1116	233
1816	1394	162
1816	1463	243
1816	1553	412
1816	1755	481
1816	1768	271
1816	1795	176
1816	1818	180
1816	2133	218
1816	2616	341
1816	3467	228
1816	3670	425
1816	3745	250
1816	4208	226
1816	4361	229
1816	4750	236
1816	5441	488
1816	6137	231
1816	6466	215
1816	6472	166
1816	7338	287
1816	7857	326
1816	8224	170
1816	9829	164
1816	11182	408
1816	11484	319
1816	12021	233
1816	16840	414

1816	16841	322
1816	16842	282
1816	16843	258
1816	16844	211
1817	64	354
1817	67	510
1817	151	1836
1817	157	1095
1817	182	597
1817	183	269
1817	375	717
1817	377	208
1817	390	576
1817	423	498
1817	425	3024
1817	546	363
1817	689	408
1817	894	1139
1817	1081	499
1817	1090	3507
1817	1098	1126
1817	1135	1137
1817	1609	219
1817	1688	400
1817	1781	228
1817	1983	1691
1817	1990	1096
1817	2083	218
1817	2521	204
1817	3324	299

1817	3502	1313
1817	3882	535
1817	3902	288
1817	8054	272
1817	8068	1858
1817	8697	244
1817	9566	520
1817	10535	3077
1817	11875	177
1817	12190	1112
1817	12370	164
1817	15614	308
1817	16845	1967
1817	16846	785
1817	16847	538
1817	16848	461
1817	16849	249
1817	16850	236
1817	16851	204
1817	16852	197
1817	16853	194
1817	16854	175
1817	16855	170
1817	16856	163
1818	51	1
1818	65	15
1818	72	25
1818	76	1
1818	85	4
1818	155	2

1818	173 5	
1818	180 3	
1818	187 4	
1818	190 12	
1818	193 1	
1818	203 1	
1818	221 2	
1818	229 62	
1818	253 3	
1818	306 1	
1818	424 1	
1818	429 8	
1818	533 1	
1818	716 2	
1818	734 28	
1818	877 2	
1818	993 14	
1818	1001	31
1818	1008	2
1818	1122	3
1818	1375	3
1818	1464	1
1818	1696	1
1818	2027	2
1818	2463	1
1818	3302	1
1818	5780	1
1818	5988	1
1818	8038	2
1818	8312	1

1818	8313	1
1818	10625	2
1818	13416	2
1818	16857	129
1818	16858	6
1818	16859	6
1818	16860	4
1818	16861	4
1818	16862	3
1818	16863	2
1818	16864	2
1818	16865	2
1818	16866	2
1818	16867	2
1819	89 25	
1819	157 66	
1819	161 77	
1819	230 252	
1819	247 32	
1819	251 34	
1819	257 26	
1819	292 23	
1819	301 138	
1819	321 32	
1819	326 32	
1819	327 28	
1819	329 134	
1819	330 54	
1819	455 48	
1819	459 88	

1819	468 2046	
1819	479 31	
1819	481 26	
1819	484 123	
1819	1037	54
1819	1059	72
1819	1427	26
1819	1433	39
1819	1443	29
1819	2523	25
1819	3078	44
1819	3317	106
1819	3318	65
1819	3498	22
1819	4557	25
1819	4710	76
1819	5948	33
1819	6385	27
1819	6389	277
1819	8081	43
1819	8991	30
1819	9799	28
1819	10608	44
1819	10656	27
1819	11085	22
1819	12254	41
1819	13292	58
1819	13542	38
1819	13579	32
1819	16723	24

1819	16868	68
1819	16869	32
1819	16870	25
1819	16871	23
1820	55	153
1820	67	104
1820	89	1169
1820	157	664
1820	161	120
1820	227	302
1820	234	64
1820	257	89
1820	288	922
1820	289	16076
1820	291	189
1820	292	1425
1820	295	249
1820	298	169
1820	299	148
1820	300	1781
1820	302	378
1820	306	1034
1820	316	138
1820	324	90
1820	327	360
1820	328	257
1820	329	66
1820	333	3161
1820	342	87
1820	344	69

1820	352	326
1820	458	61
1820	461	121
1820	466	1090
1820	468	255
1820	475	175
1820	526	302
1820	528	74
1820	531	586
1820	544	76
1820	545	96
1820	674	358
1820	679	601
1820	691	138
1820	701	431
1820	706	159
1820	903	152
1820	1042	355
1820	1244	242
1820	1249	1034
1820	1456	61
1820	2346	175
1820	3397	306
1820	3713	210
1821	55	493
1821	67	340
1821	89	798
1821	161	107
1821	257	174
1821	288	772

1821	289 4377
1821	291 79
1821	292 1535
1821	295 191
1821	298 314
1821	299 94
1821	300 876
1821	302 291
1821	306 85
1821	318 714
1821	323 122
1821	324 89
1821	325 110
1821	328 276
1821	333 210
1821	344 133
1821	352 550
1821	360 106
1821	458 102
1821	461 340
1821	464 359
1821	466 515
1821	475 132
1821	498 221
1821	528 1606
1821	531 76
1821	532 345
1821	535 628
1821	544 383
1821	679 1943

1821	686 173	
1821	701 613	
1821	704 73	
1821	903 119	
1821	972 220	
1821	1032	217
1821	1090	95
1821	1243	500
1821	1441	72
1821	2020	171
1821	2025	280
1821	2091	103
1821	2498	86
1821	8589	309
1822	383 33	
1822	720 30	
1822	852 21	
1822	853 16	
1822	857 38	
1822	1206	21
1822	1513	64
1822	1520	23
1822	1645	29
1822	1814	15
1822	3436	34
1822	3455	16
1822	4129	20
1822	4155	16
1822	4184	20
1822	4194	48

1822	4197	18
1822	4218	21
1822	4224	23
1822	4457	18
1822	4821	36
1822	5785	25
1822	6391	24
1822	6560	14
1822	8934	37
1822	9960	16
1822	10402	31
1822	10565	25
1822	10579	29
1822	13247	26
1822	13253	27
1822	13757	16
1822	16435	19
1822	16872	44
1822	16873	44
1822	16874	43
1822	16875	41
1822	16876	39
1822	16877	36
1822	16878	33
1822	16879	31
1822	16880	28
1822	16881	26
1822	16882	25
1822	16883	22
1822	16884	22

1822	16885	21
1822	16886	20
1822	16887	19
1822	16888	16
1823	65	1077
1823	154	200
1823	166	211
1823	182	196
1823	190	225
1823	227	431
1823	229	348
1823	300	320
1823	302	217
1823	316	631
1823	333	142
1823	366	173
1823	371	142
1823	377	260
1823	378	190
1823	389	170
1823	439	162
1823	464	322
1823	498	1180
1823	499	244
1823	501	221
1823	511	489
1823	517	134
1823	542	417
1823	679	261
1823	967	174

1823	982 168	
1823	1045	179
1823	1116	276
1823	1375	266
1823	1768	247
1823	1852	253
1823	2425	175
1823	2661	272
1823	3078	219
1823	4177	261
1823	4773	189
1823	5972	240
1823	6120	437
1823	6534	252
1823	6602	141
1823	7157	216
1823	8728	139
1823	8735	131
1823	9187	153
1823	11155	321
1823	12027	140
1823	16889	487
1823	16890	292
1823	16891	138
1824	89 131	
1824	154 559	
1824	190 532	
1824	212 112	
1824	222 69	
1824	227 1047	

1824	230 151	
1824	316 58	
1824	418 219	
1824	424 109	
1824	533 426	
1824	562 81	
1824	602 161	
1824	735 149	
1824	857 134	
1824	868 97	
1824	945 54	
1824	1239	148
1824	1240	94
1824	1398	128
1824	1400	289
1824	1412	423
1824	1414	77
1824	1415	64
1824	1520	76
1824	1639	58
1824	2121	80
1824	2463	88
1824	2818	140
1824	2855	113
1824	2899	59
1824	3110	187
1824	3258	688
1824	3300	218
1824	3739	78
1824	4022	122

1824	4115	52
1824	4205	125
1824	4251	57
1824	4618	95
1824	5320	129
1824	6298	104
1824	7140	108
1824	9467	92
1824	11500	203
1824	12273	84
1824	15994	286
1824	16892	127
1824	16893	100
1824	16894	66
1825	89 64	
1825	198 156	
1825	288 67	
1825	344 132	
1825	356 88	
1825	378 45	
1825	412 60	
1825	466 55	
1825	475 51	
1825	488 117	
1825	497 60	
1825	498 83	
1825	500 146	
1825	504 95	
1825	531 45	
1825	687 157	

1825	706 61	
1825	718 64	
1825	808 130	
1825	816 319	
1825	834 47	
1825	857 68	
1825	978 74	
1825	1037	69
1825	1044	60
1825	1180	47
1825	1185	44
1825	1186	46
1825	1188	109
1825	1206	104
1825	1459	81
1825	1513	76
1825	1645	46
1825	1650	133
1825	1672	454
1825	1914	117
1825	2381	56
1825	2436	112
1825	2439	56
1825	3239	48
1825	3317	71
1825	3328	101
1825	3333	188
1825	3444	102
1825	3589	61
1825	3946	44

1825	4821	75
1825	7634	67
1825	11042	45
1825	16895	45
1826	72	397
1826	89	649
1826	97	416
1826	167	203
1826	173	620
1826	228	316
1826	229	547
1826	233	414
1826	288	447
1826	289	511
1826	300	553
1826	425	252
1826	439	230
1826	453	368
1826	466	289
1826	486	454
1826	498	345
1826	504	1272
1826	506	700
1826	518	262
1826	525	253
1826	539	207
1826	542	195
1826	599	197
1826	680	235
1826	903	208

1826	918	258
1826	930	274
1826	1097	976
1826	1098	214
1826	1116	304
1826	1167	620
1826	1243	215
1826	1246	456
1826	1295	207
1826	1400	351
1826	1988	191
1826	2660	307
1826	3186	319
1826	3198	213
1826	3200	653
1826	3205	203
1826	3769	343
1826	5441	237
1826	5735	476
1826	6539	227
1826	8592	223
1826	11812	187
1826	12295	237
1826	16896	302
1827	9	662
1827	10	611
1827	11	632
1827	19	858
1827	43	544
1827	45	632

1827	1066	721
1827	1203	1029
1827	1222	518
1827	1253	616
1827	1277	564
1827	1299	1127
1827	1315	757
1827	2165	1256
1827	2472	762
1827	2678	759
1827	3551	485
1827	3988	568
1827	4875	796
1827	4876	631
1827	6542	560
1827	6548	594
1827	6878	567
1827	6971	522
1827	7080	1057
1827	9582	575
1827	10162	1573
1827	11093	523
1827	11186	579
1827	12845	489
1827	14287	937
1827	14292	1211
1827	16897	1509
1827	16898	1374
1827	16899	1352
1827	16900	1275

1827	16901	1101
1827	16902	813
1827	16903	799
1827	16904	747
1827	16905	660
1827	16906	609
1827	16907	601
1827	16908	600
1827	16909	600
1827	16910	597
1827	16911	573
1827	16912	573
1827	16913	528
1827	16914	487
1828	55	1235
1828	67	892
1828	89	1107
1828	155	521
1828	161	496
1828	229	1552
1828	257	621
1828	285	782
1828	288	991
1828	289	8738
1828	290	421
1828	291	833
1828	292	1122
1828	294	513
1828	295	894
1828	298	363

1828	299 679	
1828	300 969	
1828	302 472	
1828	310 410	
1828	311 665	
1828	318 992	
1828	320 593	
1828	321 696	
1828	325 361	
1828	328 851	
1828	329 374	
1828	333 911	
1828	349 469	
1828	455 2672	
1828	459 444	
1828	466 736	
1828	525 363	
1828	538 711	
1828	540 361	
1828	544 408	
1828	674 603	
1828	701 429	
1828	908 603	
1828	1090	445
1828	1246	776
1828	1375	378
1828	1409	407
1828	1580	409
1828	1684	438
1828	2083	495

1828	2521	640
1828	2523	500
1828	2801	652
1828	3575	381
1829	7	1132
1829	55	1247
1829	67	354
1829	209	1117
1829	288	1161
1829	289	1335
1829	291	541
1829	292	3062
1829	293	755
1829	295	338
1829	298	5590
1829	300	1434
1829	302	358
1829	306	1475
1829	316	12193
1829	318	1068
1829	320	347
1829	333	319
1829	340	296
1829	344	2007
1829	375	1574
1829	377	1313
1829	378	639
1829	423	4773
1829	430	1131
1829	458	5718

1829	461	598
1829	466	2176
1829	486	2404
1829	498	413
1829	528	420
1829	532	1336
1829	542	448
1829	679	1219
1829	682	414
1829	686	494
1829	701	784
1829	889	646
1829	903	331
1829	908	467
1829	1032	319
1829	1034	996
1829	1047	465
1829	1609	870
1829	1688	1117
1829	2025	287
1829	2521	398
1829	2546	470
1829	3173	1022
1829	3377	314
1830	65	1618
1830	97	1072
1830	154	4172
1830	173	1084
1830	182	1609
1830	190	1013

1830	196	1162
1830	198	3795
1830	207	2977
1830	220	1190
1830	221	1053
1830	228	1180
1830	234	1018
1830	316	1219
1830	333	1210
1830	362	1445
1830	424	2396
1830	436	1092
1830	439	21375
1830	498	2453
1830	500	731
1830	517	1331
1830	538	1559
1830	614	1694
1830	706	2611
1830	903	4834
1830	932	1918
1830	967	1898
1830	1044	4349
1830	1048	2530
1830	1145	1060
1830	1375	3314
1830	1424	1617
1830	1512	1152
1830	1513	705
1830	1545	1884

1830	1672	998
1830	1772	1179
1830	1775	2451
1830	1934	1440
1830	2346	1018
1830	2544	6779
1830	3081	1257
1830	3110	1053
1830	3373	1067
1830	3488	1321
1830	3747	1434
1830	4802	1026
1830	10418	1010
1830	12311	2314
1831	65 261	
1831	154 1181	
1831	173 373	
1831	190 1079	
1831	198 651	
1831	207 1292	
1831	217 414	
1831	225 1706	
1831	226 837	
1831	227 2274	
1831	228 253	
1831	229 394	
1831	234 552	
1831	419 351	
1831	424 2325	
1831	425 502	

1831	427	1447
1831	428	739
1831	429	650
1831	436	270
1831	439	767
1831	441	414
1831	533	1516
1831	618	226
1831	629	464
1831	707	934
1831	709	368
1831	714	310
1831	898	309
1831	917	884
1831	1048	365
1831	1089	314
1831	1239	247
1831	1412	388
1831	1424	308
1831	1513	408
1831	1515	275
1831	1766	753
1831	1957	220
1831	2428	214
1831	3200	252
1831	3693	214
1831	4541	221
1831	4793	445
1831	4802	258
1831	6726	239

1831	7351	276
1831	8710	457
1831	14271	460
1831	16915	414
1832	53	972
1832	65	2546
1832	70	898
1832	154	1968
1832	163	2275
1832	173	1009
1832	182	1822
1832	190	1483
1832	196	1141
1832	203	958
1832	206	1252
1832	207	1667
1832	217	1438
1832	220	1090
1832	222	882
1832	227	7718
1832	228	952
1832	229	1574
1832	234	947
1832	366	1569
1832	423	1162
1832	424	1077
1832	425	1431
1832	429	885
1832	437	1188
1832	439	1497

1832	602	1074
1832	615	1270
1832	618	1000
1832	622	1266
1832	689	1068
1832	709	1918
1832	757	925
1832	858	1404
1832	898	1531
1832	903	44472
1832	1048	1033
1832	1090	864
1832	1097	1141
1832	1098	2935
1832	1244	944
1832	1390	1258
1832	1400	2462
1832	1424	2262
1832	1486	1356
1832	2083	962
1832	2524	975
1832	3057	4301
1832	3902	973
1832	8710	920
1833	71	123
1833	86	108
1833	89	648
1833	161	568
1833	184	109
1833	252	138

1833	288	199
1833	289	293
1833	292	373
1833	295	586
1833	299	254
1833	308	216
1833	311	118
1833	320	105
1833	326	111
1833	328	100
1833	349	160
1833	352	125
1833	430	127
1833	468	112
1833	525	127
1833	528	185
1833	543	111
1833	701	2888
1833	907	187
1833	981	215
1833	1037	98
1833	1433	99
1833	1440	277
1833	1443	389
1833	1448	146
1833	1449	388
1833	1450	117
1833	1475	493
1833	1500	182
1833	1596	174

1833	2023	144
1833	2051	127
1833	2074	122
1833	2395	793
1833	4252	377
1833	5249	231
1833	6317	150
1833	7290	116
1833	7869	170
1833	10600	102
1833	11085	285
1833	16916	394
1833	16917	154
1833	16918	102
1834	288 1	
1834	295 1	
1834	310 4	
1834	321 2	
1834	325 86	
1834	333 30	
1834	344 170	
1834	349 1	
1834	461 81	
1834	465 1	
1834	528 2	
1834	680 2375	
1834	681 331	
1834	686 90	
1834	692 16	
1834	793 28	

1834	797 3	
1834	908 1	
1834	1051	2
1834	1054	8
1834	1059	8
1834	1453	18
1834	1590	413
1834	1597	11
1834	1684	336
1834	1740	18
1834	2801	1
1834	4879	7
1834	6865	2
1834	7397	4
1834	9849	7
1834	10641	4
1834	11010	5
1834	13546	2
1834	16771	6
1834	16919	173
1834	16920	94
1834	16921	16
1834	16922	11
1834	16923	10
1834	16924	8
1834	16925	5
1834	16926	5
1834	16927	3
1834	16928	3
1834	16929	2

1834	16930	2
1834	16931	2
1834	16932	2
1834	16933	2
1835	56	165
1835	67	41
1835	72	4551
1835	75	154
1835	88	29
1835	89	30
1835	90	167
1835	153	58
1835	161	31
1835	190	44
1835	206	88
1835	229	38
1835	233	77
1835	238	1071
1835	292	130
1835	441	42
1835	450	282
1835	539	30
1835	703	53
1835	868	58
1835	880	339
1835	1013	35
1835	1061	193
1835	1072	109
1835	1075	298
1835	1151	134

1835	1379	259
1835	1700	95
1835	1707	34
1835	1713	450
1835	1739	148
1835	1786	51
1835	1787	759
1835	1833	75
1835	1897	49
1835	2071	49
1835	2821	88
1835	3046	131
1835	3047	38
1835	3057	44
1835	3191	278
1835	3693	28
1835	3988	37
1835	4997	92
1835	5545	86
1835	7369	67
1835	16934	47
1835	16935	44
1835	16936	38
1835	16937	34
1836	53	3
1836	88	7
1836	154	5
1836	198	12
1836	207	13
1836	217	3

1836	318 4	
1836	355 4	
1836	419 4	
1836	424 5	
1836	446 4	
1836	453 5	
1836	576 179	
1836	622 4	
1836	683 5	
1836	687 9	
1836	718 186	
1836	986 3	
1836	1038	4
1836	1040	7
1836	1183	4
1836	1198	4
1836	1201	4
1836	1389	4
1836	1390	14
1836	1886	5
1836	1974	5
1836	1979	3
1836	2612	4
1836	2665	4
1836	3171	4
1836	3206	6
1836	3749	10
1836	4089	6
1836	6374	3
1836	6601	4

1836	6609	43
1836	6614	4
1836	7194	4
1836	7735	131
1836	7736	45
1836	7739	72
1836	7742	6
1836	7827	4
1836	8504	4
1836	10924	4
1836	11849	5
1836	16938	35
1836	16939	17
1836	16940	9
1838	55	1043
1838	57	190
1838	58	477
1838	65	304
1838	67	656
1838	81	249
1838	88	186
1838	89	303
1838	154	659
1838	167	368
1838	172	615
1838	195	291
1838	217	228
1838	227	201
1838	229	275
1838	285	1279

1838	288 237	
1838	292 336	
1838	298 195	
1838	310 301	
1838	316 816	
1838	511 195	
1838	614 244	
1838	622 282	
1838	697 502	
1838	888 284	
1838	889 461	
1838	891 361	
1838	900 455	
1838	903 294	
1838	961 729	
1838	1098	680
1838	1375	209
1838	1424	230
1838	1519	354
1838	1824	331
1838	2083	189
1838	2277	206
1838	2523	234
1838	3057	457
1838	3110	395
1838	3201	530
1838	3460	187
1838	3466	245
1838	4432	193
1838	4442	216

1838	4531	279
1838	4705	202
1838	4731	234
1838	9470	271
1839	227 324	
1839	245 186	
1839	562 181	
1839	724 377	
1839	728 435	
1839	732 304	
1839	881 277	
1839	917 512	
1839	922 179	
1839	942 162	
1839	957 91	
1839	1104	197
1839	1338	80
1839	1348	278
1839	1358	455
1839	1369	3377
1839	1372	368
1839	1412	190
1839	1426	632
1839	1810	233
1839	2004	85
1839	2342	1230
1839	2343	173
1839	2347	113
1839	2362	396
1839	2380	81

1839	2463	87
1839	2787	330
1839	2838	137
1839	2873	409
1839	2901	212
1839	3470	543
1839	3774	228
1839	4124	113
1839	4251	377
1839	5672	114
1839	5688	303
1839	6748	155
1839	6959	286
1839	7050	193
1839	8386	124
1839	9334	92
1839	12081	406
1839	13074	152
1839	15817	209
1839	16947	299
1839	16948	250
1839	16949	151
1839	16950	90
1839	16951	89
1840	65	399
1840	67	297
1840	166	181
1840	227	666
1840	265	273
1840	289	485

1840	293	261
1840	340	171
1840	511	190
1840	523	600
1840	891	253
1840	910	648
1840	961	1597
1840	986	303
1840	1147	622
1840	1705	195
1840	2031	1147
1840	2044	649
1840	2051	495
1840	2277	206
1840	2407	278
1840	2465	327
1840	3081	172
1840	3097	175
1840	3304	195
1840	3466	1104
1840	3888	322
1840	4918	695
1840	5662	1089
1840	6390	1201
1840	6397	246
1840	7142	726
1840	7287	262
1840	8047	241
1840	8102	229
1840	8844	1587

1840	9348	320
1840	11281	614
1840	12026	273
1840	13709	293
1840	15776	367
1840	16076	530
1840	16952	1708
1840	16953	587
1840	16954	381
1840	16955	321
1840	16956	236
1840	16957	233
1840	16958	179
1840	16959	172
1841	13	163
1841	16	461
1841	24	122
1841	34	126
1841	39	278
1841	43	114
1841	50	278
1841	75	193
1841	504	130
1841	515	464
1841	522	207
1841	1069	424
1841	1086	207
1841	1297	310
1841	1299	130
1841	1300	440

1841	2044	110
1841	2790	99
1841	2952	126
1841	3244	409
1841	3432	156
1841	3542	385
1841	3551	112
1841	4165	119
1841	5015	120
1841	5584	136
1841	6875	479
1841	7454	109
1841	7457	195
1841	9144	190
1841	9540	205
1841	9636	103
1841	10430	155
1841	10974	132
1841	11192	336
1841	16013	109
1841	16960	393
1841	16961	210
1841	16962	156
1841	16963	142
1841	16964	138
1841	16965	137
1841	16966	135
1841	16967	120
1841	16968	118
1841	16969	117

1841	16970	115
1841	16971	110
1841	16972	107
1841	16973	100
1842	7	101
1842	45	357
1842	56	138
1842	154	111
1842	159	147
1842	173	149
1842	198	159
1842	227	1197
1842	377	245
1842	487	109
1842	488	247
1842	512	234
1842	524	125
1842	537	103
1842	540	145
1842	707	167
1842	785	294
1842	830	430
1842	843	163
1842	907	367
1842	917	155
1842	945	209
1842	978	193
1842	1044	124
1842	1180	105
1842	1197	97

1842	1206	190
1842	1351	119
1842	1358	256
1842	1513	167
1842	1672	244
1842	2014	127
1842	2150	400
1842	2196	98
1842	2342	273
1842	2352	151
1842	2366	139
1842	2794	112
1842	3196	261
1842	3247	134
1842	3717	320
1842	4104	152
1842	5421	100
1842	6225	104
1842	6594	144
1842	6600	122
1842	8589	177
1842	16974	224
1842	16975	117
1842	16976	99
1844	65	188
1844	67	1623
1844	89	157
1844	154	704
1844	173	222
1844	196	262

1844	203	219
1844	221	763
1844	225	449
1844	229	443
1844	261	212
1844	288	214
1844	289	218
1844	295	193
1844	298	298
1844	300	342
1844	316	1240
1844	418	395
1844	424	471
1844	429	245
1844	430	198
1844	444	149
1844	450	265
1844	511	286
1844	546	204
1844	679	265
1844	689	374
1844	701	472
1844	715	388
1844	858	1797
1844	889	196
1844	961	5918
1844	1089	207
1844	1090	561
1844	1098	2022
1844	1239	203

1844	1375	148
1844	1424	210
1844	1811	176
1844	2277	159
1844	3057	1101
1844	3200	146
1844	3201	981
1844	3281	522
1844	3763	597
1844	4716	185
1844	6374	168
1844	6539	181
1844	7663	286
1844	17007	198
1845	51	2303
1845	56	131
1845	77	662
1845	85	288
1845	89	172
1845	96	186
1845	152	523
1845	157	146
1845	180	421
1845	227	1411
1845	228	171
1845	294	147
1845	329	181
1845	386	218
1845	479	193
1845	599	887

1845	601 165	
1845	689 580	
1845	706 170	
1845	707 579	
1845	724 259	
1845	874 1095	
1845	959 5278	
1845	1001	316
1845	1013	157
1845	1019	169
1845	1076	221
1845	1099	130
1845	1358	130
1845	1366	314
1845	1369	151
1845	1412	385
1845	1464	379
1845	1518	287
1845	2342	136
1845	2498	217
1845	2580	1099
1845	2609	145
1845	2787	140
1845	2853	294
1845	3470	146
1845	3920	724
1845	4031	344
1845	5020	651
1845	9347	168
1845	10820	146

1845	11590	230
1845	11849	135
1845	17008	679
1845	17009	160
1846	89	485
1846	157	197
1846	209	195
1846	212	300
1846	227	8891
1846	234	293
1846	288	194
1846	289	372
1846	298	347
1846	300	212
1846	321	740
1846	324	379
1846	326	247
1846	344	590
1846	386	489
1846	437	232
1846	455	186
1846	461	2359
1846	466	324
1846	475	320
1846	540	320
1846	679	2425
1846	681	544
1846	691	445
1846	694	467
1846	717	350

1846	733	484
1846	735	209
1846	793	1103
1846	868	187
1846	945	495
1846	959	314
1846	1242	234
1846	1243	324
1846	1246	638
1846	1414	746
1846	1416	398
1846	1459	224
1846	1782	521
1846	2055	453
1846	2176	256
1846	2265	621
1846	2801	227
1846	3071	524
1846	3097	235
1846	3712	447
1846	4920	212
1846	8552	248
1846	12209	431
1846	17010	201
1847	1888	4
1847	5503	4
1847	8127	12
1847	11542	5
1847	11963	4
1847	12966	9

1847	13033	22
1847	16449	5
1847	17011	162
1847	17012	63
1847	17013	58
1847	17014	32
1847	17015	29
1847	17016	28
1847	17017	28
1847	17018	27
1847	17019	25
1847	17020	24
1847	17021	23
1847	17022	22
1847	17023	22
1847	17024	21
1847	17025	21
1847	17026	20
1847	17027	19
1847	17028	19
1847	17029	17
1847	17030	16
1847	17031	16
1847	17032	15
1847	17033	12
1847	17034	12
1847	17035	11
1847	17036	10
1847	17037	9
1847	17038	9

1847	17039	9
1847	17040	8
1847	17041	8
1847	17042	8
1847	17043	7
1847	17044	6
1847	17045	5
1847	17046	5
1847	17047	5
1847	17048	4
1847	17049	4
1847	17050	4
1847	17051	4
1847	17052	4
1848	154 363	
1848	200 369	
1848	207 329	
1848	212 3274	
1848	219 342	
1848	227 463	
1848	433 304	
1848	859 322	
1848	1748	293
1848	2290	961
1848	2475	311
1848	5210	287
1848	5478	340
1848	6230	775
1848	8642	582
1848	9545	294

1848	9727	1228
1848	9971	508
1848	10407	584
1848	11900	431
1848	12583	1046
1848	13387	373
1848	14347	504
1848	14350	454
1848	14353	326
1848	14355	600
1848	14356	348
1848	15655	397
1848	17053	836
1848	17054	743
1848	17055	633
1848	17056	580
1848	17057	531
1848	17058	503
1848	17059	459
1848	17060	452
1848	17061	428
1848	17062	403
1848	17063	401
1848	17064	384
1848	17065	354
1848	17066	346
1848	17067	340
1848	17068	330
1848	17069	321
1848	17070	317

1848	17071	315
1848	17072	309
1848	17073	306
1848	17074	305
1850	55	760
1850	58	140
1850	65	265
1850	67	721
1850	89	2503
1850	167	145
1850	265	666
1850	285	167
1850	288	328
1850	289	1106
1850	294	469
1850	295	292
1850	296	1111
1850	298	251
1850	318	146
1850	323	734
1850	328	147
1850	352	612
1850	530	408
1850	532	274
1850	535	160
1850	669	146
1850	674	582
1850	679	500
1850	886	517
1850	906	6512

1850	911 958	
1850	915 201	
1850	1001	338
1850	1047	352
1850	1464	467
1850	1589	236
1850	2022	357
1850	2521	309
1850	2523	486
1850	3288	754
1850	3491	172
1850	3501	214
1850	3570	805
1850	3576	174
1850	3674	208
1850	3948	274
1850	4704	203
1850	6232	300
1850	8589	205
1850	8718	817
1850	16662	148
1850	17077	306
1850	17078	295
1850	17079	262
1851	89 3	
1851	288 71	
1851	289 1775	
1851	291 128	
1851	292 62	
1851	293 1	

1851	299 35	
1851	300 51	
1851	302 1	
1851	318 380	
1851	325 220	
1851	333 622	
1851	344 2676	
1851	456 2	
1851	461 3104	
1851	466 54	
1851	475 4	
1851	528 20	
1851	543 14	
1851	646 92	
1851	679 1108	
1851	680 27	
1851	681 204	
1851	686 2160	
1851	692 1	
1851	790 13	
1851	793 9	
1851	797 18	
1851	802 122	
1851	908 4245	
1851	1428	95
1851	1450	11
1851	1458	257
1851	1684	48
1851	1690	1
1851	1934	29

1851	2801	5
1851	3404	49
1851	3427	15
1851	4814	4
1851	4879	1
1851	16923	49
1851	17080	1
1852	89	314
1852	155	122
1852	161	115
1852	166	251
1852	227	155
1852	306	378
1852	378	188
1852	518	168
1852	525	132
1852	534	250
1852	554	191
1852	641	168
1852	701	193
1852	788	178
1852	917	522
1852	930	614
1852	1244	789
1852	1414	197
1852	1432	849
1852	1470	681
1852	2044	9974
1852	2047	344
1852	2343	162

1852	2396	177
1852	2531	303
1852	3060	134
1852	3073	116
1852	3874	201
1852	4149	1747
1852	4495	207
1852	4700	125
1852	5249	745
1852	7530	166
1852	7837	138
1852	7846	636
1852	8372	266
1852	10628	144
1852	11084	137
1852	15457	749
1852	15903	124
1852	17081	1315
1852	17082	558
1852	17083	539
1852	17084	432
1852	17085	332
1852	17086	314
1852	17087	310
1852	17088	293
1852	17089	279
1852	17090	157
1853	51	344
1853	89	269
1853	196	482

1853	217 641	
1853	226 920	
1853	383 245	
1853	407 233	
1853	429 370	
1853	440 270	
1853	450 312	
1853	743 224	
1853	746 476	
1853	757 958	
1853	769 199	
1853	1142	308
1853	1162	548
1853	1191	257
1853	1195	482
1853	1216	1038
1853	1222	446
1853	1390	270
1853	1398	485
1853	1400	231
1853	1486	259
1853	1947	189
1853	1954	497
1853	1958	603
1853	2402	714
1853	2582	226
1853	3280	284
1853	3517	189
1853	3685	226
1853	3809	234

1853	4118	238
1853	5475	220
1853	5746	227
1853	6032	260
1853	6409	183
1853	7730	185
1853	9260	221
1853	9649	500
1853	10831	197
1853	11004	276
1853	11320	500
1853	11352	181
1853	13179	418
1853	17091	374
1853	17092	369
1853	17093	296
1853	17094	190
1854	250 82	
1854	7558	149
1854	9053	95
1854	9550	536
1854	12434	241
1854	12439	79
1854	12447	213
1854	12454	231
1854	17095	402
1854	17096	319
1854	17097	260
1854	17098	257
1854	17099	226

1854	17100	224
1854	17101	216
1854	17102	183
1854	17103	147
1854	17104	140
1854	17105	134
1854	17106	130
1854	17107	121
1854	17108	117
1854	17109	116
1854	17110	115
1854	17111	113
1854	17112	112
1854	17113	112
1854	17114	107
1854	17115	102
1854	17116	100
1854	17117	96
1854	17118	93
1854	17119	91
1854	17120	90
1854	17121	90
1854	17122	90
1854	17123	89
1854	17124	88
1854	17125	83
1854	17126	82
1854	17127	81
1854	17128	81
1854	17129	80

1854	17130	80
1854	17131	80
1854	17132	80
1854	17133	79
1854	17134	78
1854	17135	76
1854	17136	76
1855	59	131
1855	173	117
1855	182	98
1855	190	112
1855	196	178
1855	206	222
1855	210	347
1855	217	135
1855	229	1027
1855	428	79
1855	431	194
1855	432	293
1855	434	494
1855	443	84
1855	540	207
1855	620	150
1855	629	76
1855	716	281
1855	757	106
1855	1048	655
1855	1155	174
1855	1398	156
1855	1400	88

1855	1492	209
1855	1502	520
1855	1977	172
1855	1983	248
1855	1986	147
1855	2133	171
1855	2587	98
1855	2614	187
1855	2715	142
1855	3079	118
1855	3105	88
1855	3266	104
1855	3373	145
1855	4115	158
1855	4435	267
1855	5439	266
1855	6045	129
1855	7071	79
1855	8479	91
1855	10535	182
1855	14359	118
1855	17137	195
1855	17138	175
1855	17139	134
1855	17140	83
1855	17141	79
1855	17142	79
1856	7	707
1856	55	440
1856	67	1242

1856	88	161
1856	157	1212
1856	234	177
1856	289	1026
1856	292	1562
1856	296	1075
1856	299	682
1856	318	289
1856	323	700
1856	325	249
1856	352	200
1856	377	175
1856	378	245
1856	405	406
1856	455	584
1856	459	287
1856	475	185
1856	523	262
1856	524	243
1856	544	577
1856	646	272
1856	701	208
1856	792	173
1856	798	199
1856	803	178
1856	906	1137
1856	913	529
1856	1044	170
1856	2021	565
1856	2022	419

1856	2091	562
1856	2094	405
1856	2098	180
1856	2523	550
1856	2548	761
1856	3176	610
1856	3332	203
1856	3961	196
1856	4350	732
1856	4558	635
1856	5533	180
1856	9231	257
1856	17143	486
1856	17144	420
1856	17145	286
1856	17146	276
1856	17147	172
1857	154 391	
1857	199 382	
1857	217 545	
1857	230 480	
1857	233 403	
1857	383 595	
1857	507 393	
1857	808 497	
1857	982 401	
1857	1239	1255
1857	1378	785
1857	1858	508
1857	2294	440

1857	2524	370
1857	2572	996
1857	2643	492
1857	3341	590
1857	3364	448
1857	3616	586
1857	3666	578
1857	3714	488
1857	3718	381
1857	3737	1169
1857	4185	478
1857	4189	849
1857	4191	646
1857	4193	477
1857	4196	644
1857	4198	441
1857	4201	680
1857	4207	413
1857	4821	561
1857	5631	722
1857	5966	432
1857	6294	695
1857	6773	580
1857	6836	705
1857	8280	560
1857	8464	399
1857	9773	369
1857	10564	493
1857	10955	546
1857	11413	547

1857	11988	433
1857	12205	454
1857	17148	464
1857	17149	447
1857	17150	418
1857	17151	403
1857	17152	382
1858	7	124
1858	176	131
1858	198	169
1858	227	114
1858	500	96
1858	517	130
1858	724	428
1858	732	160
1858	841	316
1858	843	169
1858	846	162
1858	917	479
1858	918	322
1858	922	187
1858	923	108
1858	928	282
1858	942	133
1858	947	150
1858	957	137
1858	1342	120
1858	1343	108
1858	1346	159
1858	1353	247

1858	1358	537
1858	1360	387
1858	1366	284
1858	1369	566
1858	1372	219
1858	1527	210
1858	1531	115
1858	1535	103
1858	1803	120
1858	1992	127
1858	2341	175
1858	2342	392
1858	2343	128
1858	2347	94
1858	2380	112
1858	2664	212
1858	2787	155
1858	2838	221
1858	3445	166
1858	3470	236
1858	3471	103
1858	3476	246
1858	4286	119
1858	5157	336
1858	5668	133
1858	6748	122
1858	10686	185
1859	196 1	
1859	202 1	
1859	429 1	

1859	441 1	
1859	562 1	
1859	697 1	
1859	859 1	
1859	1040	1
1859	1046	1
1859	1091	1
1859	1889	1
1859	2375	1
1859	2627	1
1859	2633	1
1859	4538	1
1859	10847	1
1859	13066	1
1859	17153	142
1859	17154	14
1859	17155	3
1859	17156	3
1859	17157	2
1859	17158	2
1859	17159	2
1859	17160	2
1859	17161	2
1859	17162	2
1859	17163	2
1859	17164	1
1859	17165	1
1859	17166	1
1859	17167	1
1859	17168	1

1859	17169	1
1859	17170	1
1859	17171	1
1859	17172	1
1860	706 904	
1860	707 2166	
1860	724 587	
1860	841 712	
1860	843 250	
1860	917 2974	
1860	918 1985	
1860	922 305	
1860	928 609	
1860	937 470	
1860	942 461	
1860	946 1503	
1860	947 478	
1860	949 1404	
1860	953 319	
1860	956 324	
1860	957 791	
1860	1110	351
1860	1257	851
1860	1339	616
1860	1349	424
1860	1350	1026
1860	1352	555
1860	1358	1700
1860	1360	1241
1860	1364	274

1860	1369	2045
1860	1372	441
1860	1412	232
1860	1521	421
1860	1522	408
1860	1526	272
1860	1527	638
1860	2004	355
1860	2342	2003
1860	3470	488
1860	3476	420
1860	4156	1235
1860	5585	360
1860	5688	584
1860	5689	329
1860	5899	379
1860	8696	316
1860	17173	845
1860	17174	809
1860	17175	784
1860	17176	607
1860	17177	572
1860	17178	288
1860	17179	270
1861	197 203	
1861	199 272	
1861	512 204	
1861	841 393	
1861	851 708	
1861	1202	183

1861	1206	258
1861	1239	230
1861	1264	181
1861	2026	193
1861	2470	228
1861	2471	619
1861	2531	202
1861	2921	191
1861	3354	320
1861	3364	220
1861	3440	337
1861	3442	240
1861	3523	211
1861	3694	210
1861	3792	353
1861	3805	226
1861	3809	244
1861	4107	185
1861	4185	203
1861	4520	191
1861	8945	178
1861	9260	227
1861	9445	224
1861	9805	186
1861	10674	322
1861	11604	215
1861	11624	256
1861	11788	352
1861	13611	184
1861	13612	207

1861	13616	300
1861	15918	198
1861	17180	426
1861	17181	413
1861	17182	348
1861	17183	297
1861	17184	288
1861	17185	224
1861	17186	218
1861	17187	195
1861	17188	193
1861	17189	190
1861	17190	185
1861	17191	178
1862	7	257
1862	187	93
1862	207	1436
1862	420	117
1862	425	111
1862	428	705
1862	430	180
1862	441	366
1862	475	123
1862	546	173
1862	709	294
1862	715	99
1862	716	248
1862	757	157
1862	1048	207
1862	1191	223

1862	1369	139
1862	1977	141
1862	2702	2114
1862	2816	129
1862	2818	131
1862	2821	267
1862	3475	545
1862	3946	364
1862	4382	222
1862	4890	164
1862	5716	407
1862	7324	118
1862	8586	533
1862	8618	476
1862	8910	166
1862	9718	102
1862	11743	275
1862	12838	110
1862	13827	731
1862	13902	394
1862	16527	135
1862	17192	417
1862	17193	318
1862	17194	316
1862	17195	301
1862	17196	227
1862	17197	216
1862	17198	215
1862	17199	176
1862	17200	142

1862	17201	122
1862	17202	102
1862	17203	93
1862	17204	93
1863	89	235
1863	256	69
1863	288	399
1863	289	448
1863	291	100
1863	292	89
1863	295	101
1863	298	81
1863	300	237
1863	304	375
1863	313	71
1863	321	57
1863	325	87
1863	333	252
1863	344	82
1863	352	68
1863	458	133
1863	461	552
1863	464	69
1863	466	221
1863	498	199
1863	523	96
1863	525	87
1863	543	96
1863	544	69
1863	679	688

1863	681 103

1863	683 52

1863	686 2086

1863	701 58

1863	797 291

1863	802 97

1863	908 119

1863	1032	369

1863	1034	66

1863	1037	112

1863	1428	516

1863	1451	57

1863	1453	734

1863	1458	151

1863	1690	56

1863	1934	54

1863	2020	54

1863	2025	161

1863	2545	53

1863	3332	211

1863	4814	127

1863	8721	311

1863	17205	62

1863	17206	62

1864	162 23

1864	253 22

1864	554 21

1864	580 24

1864	583 27

1864	730 29

1864	734 64	
1864	1418	27
1864	1426	25
1864	1468	23
1864	1747	30
1864	2265	29
1864	2570	30
1864	2771	28
1864	2895	40
1864	2896	34
1864	4251	34
1864	5168	28
1864	5806	23
1864	7056	21
1864	7137	25
1864	9351	21
1864	9428	21
1864	9437	38
1864	11871	24
1864	11954	24
1864	12057	29
1864	13272	27
1864	13605	21
1864	13621	24
1864	14740	43
1864	15268	34
1864	17207	28
1864	17208	28
1864	17209	27
1864	17210	27

1864	17211	25
1864	17212	25
1864	17213	25
1864	17214	24
1864	17215	23
1864	17216	23
1864	17217	22
1864	17218	22
1864	17219	22
1864	17220	22
1864	17221	22
1864	17222	21
1864	17223	21
1864	17224	21
1865	7	792
1865	36	205
1865	173	336
1865	233	1131
1865	234	283
1865	453	569
1865	487	773
1865	489	163
1865	497	221
1865	512	8093
1865	517	198
1865	605	240
1865	1066	1791
1865	1099	1087
1865	1270	484
1865	1545	1021

1865	1704	545
1865	1714	224
1865	1974	989
1865	2062	295
1865	3239	723
1865	3551	923
1865	3558	3823
1865	3719	329
1865	3743	248
1865	3746	591
1865	4800	1633
1865	5670	2161
1865	6120	219
1865	6542	1067
1865	6546	278
1865	6870	414
1865	6872	842
1865	7463	262
1865	9242	164
1865	9872	330
1865	10977	243
1865	10998	744
1865	11087	2986
1865	11865	663
1865	15570	1180
1865	17225	2098
1865	17226	1490
1865	17227	613
1865	17228	359
1865	17229	344

1865	17230	285
1865	17231	278
1865	17232	237
1865	17233	231
1866	7	226
1866	45	372
1866	51	19190
1866	65	1776
1866	67	386
1866	89	240
1866	190	395
1866	192	1337
1866	193	163
1866	227	736
1866	233	792
1866	234	205
1866	377	386
1866	490	163
1866	496	249
1866	512	211
1866	534	752
1866	540	929
1866	614	212
1866	854	289
1866	859	506
1866	860	535
1866	982	290
1866	1025	229
1866	1104	288
1866	1119	508

1866	1246	1509
1866	1413	400
1866	1795	165
1866	2444	1512
1866	2493	487
1866	2552	353
1866	2554	1105
1866	3070	183
1866	3213	170
1866	3302	225
1866	3308	317
1866	7054	840
1866	8042	1103
1866	8347	271
1866	9421	2291
1866	9470	228
1866	10069	814
1866	10310	436
1866	10626	194
1866	17234	679
1866	17235	397
1866	17236	271
1866	17237	214
1866	17238	213
1867	230 346	
1867	383 2392	
1867	808 239	
1867	1513	2
1867	1645	339
1867	2735	45

1867	4187	8
1867	4193	8
1867	4218	35
1867	4821	93
1867	4827	1
1867	5021	7
1867	5353	1
1867	6560	8
1867	7120	102
1867	8934	1
1867	9856	4
1867	12145	1
1867	13244	1
1867	17239	1379
1867	17240	1196
1867	17241	231
1867	17242	120
1867	17243	68
1867	17244	22
1867	17245	15
1867	17246	10
1867	17247	9
1867	17248	8
1867	17249	8
1867	17250	8
1867	17251	8
1867	17252	8
1867	17253	7
1867	17254	7
1867	17255	6

1867	17256	5
1867	17257	5
1867	17258	4
1867	17259	2
1867	17260	2
1867	17261	2
1867	17262	1
1867	17263	1
1867	17264	1
1867	17265	1
1867	17266	1
1867	17267	1
1867	17268	1
1867	17269	1
1868	157 23	
1868	163 1062	
1868	383 19	
1868	533 6	
1868	614 56	
1868	706 674	
1868	728 38	
1868	917 4563	
1868	922 2849	
1868	959 429	
1868	966 13	
1868	975 521	
1868	1104	222
1868	1249	179
1868	1347	96
1868	1358	379

1868	1369	1945
1868	1372	220
1868	1425	134
1868	1426	367
1868	1639	8
1868	2342	1530
1868	2347	15
1868	2763	2
1868	2855	142
1868	3470	82
1868	5155	3
1868	5164	48
1868	5485	9
1868	5688	110
1868	6259	356
1868	17270	57
1868	17271	13
1868	17272	1
1868	17273	1
1868	17274	1
1869	7	157
1869	55	2327
1869	67	3349
1869	69	402
1869	72	488
1869	157	1258
1869	172	257
1869	173	1169
1869	226	217
1869	257	231

1869	285 4776	
1869	288 269	
1869	289 788	
1869	295 141	
1869	299 148	
1869	468 151	
1869	535 120	
1869	576 1132	
1869	578 122	
1869	599 262	
1869	601 166	
1869	789 433	
1869	913 131	
1869	1104	477
1869	1122	368
1869	1406	257
1869	1427	124
1869	1475	124
1869	1795	187
1869	1820	723
1869	1825	264
1869	1826	579
1869	1843	221
1869	2179	207
1869	2308	242
1869	3313	349
1869	3503	382
1869	4348	214
1869	4919	165
1869	9734	307

1869	10130	111
1869	11338	164
1869	11339	400
1869	17275	662
1869	17276	590
1869	17277	498
1869	17278	491
1869	17279	203
1869	17280	119
1869	17281	115
1871	67	36
1871	184	27
1871	204	188
1871	239	130
1871	257	26
1871	288	253
1871	289	1084
1871	291	211
1871	292	166
1871	293	30
1871	295	165
1871	301	90
1871	310	70
1871	311	37
1871	318	230
1871	320	56
1871	322	51
1871	331	110
1871	332	62
1871	333	205

1871	455 38	
1871	465 45	
1871	475 28	
1871	523 265	
1871	525 94	
1871	527 99	
1871	534 40	
1871	543 32	
1871	683 28	
1871	903 180	
1871	907 123	
1871	913 57	
1871	1505	31
1871	1580	29
1871	1613	28
1871	2179	65
1871	2454	31
1871	3466	26
1871	3488	29
1871	3489	45
1871	5778	32
1871	8161	44
1871	8178	44
1871	9902	34
1871	9905	44
1871	9907	36
1871	17283	294
1871	17284	91
1871	17285	57
1871	17286	57

1872 209 919

1872 228 224

1872 230 3574

1872 300 262

1872 321 1037

1872 333 983

1872 344 403

1872 458 696

1872 461 344

1872 471 500

1872 481 1499

1872 484 223

1872 486 1192

1872 498 3636

1872 499 643

1872 540 8398

1872 680 305

1872 681 319

1872 687 13810

1872 691 587

1872 693 2140

1872 1043 2192

1872 1054 292

1872 1186 290

1872 1193 380

1872 1198 606

1872 1246 289

1872 1247 565

1872 1698 309

1872 2839 431

1872	2850	461
1872	3187	873
1872	4291	570
1872	4582	276
1872	4773	1759
1872	4811	305
1872	5140	301
1872	5749	439
1872	6124	296
1872	6614	333
1872	7396	273
1872	7704	273
1872	9158	244
1872	10023	681
1872	10924	366
1872	15900	511
1872	17287	1477
1872	17288	409
1872	17289	266
1872	17290	232
1873	55	67
1873	67	712
1873	89	88
1873	198	117
1873	257	65
1873	288	539
1873	289	3134
1873	291	134
1873	292	143
1873	295	712

1873	299 324
1873	306 197
1873	310 239
1873	316 307
1873	317 76
1873	320 293
1873	326 84
1873	327 247
1873	329 1001
1873	331 99
1873	333 501
1873	349 480
1873	377 596
1873	378 569
1873	405 218
1873	455 3086
1873	459 183
1873	468 182
1873	469 208
1873	484 275
1873	491 214
1873	498 353
1873	525 118
1873	538 1007
1873	540 293
1873	543 141
1873	903 1124
1873	907 692
1873	913 286
1873	966 276

1873	982 210	
1873	1246	78
1873	1247	72
1873	1397	84
1873	1444	149
1873	2598	88
1873	3392	208
1873	17291	568
1873	17292	105
1873	17293	73
1874	206 182	
1874	267 172	
1874	744 182	
1874	748 155	
1874	767 206	
1874	1731	367
1874	1745	226
1874	1943	372
1874	1950	198
1874	1963	367
1874	1969	350
1874	2165	454
1874	2252	251
1874	2574	241
1874	2668	375
1874	2716	387
1874	2921	176
1874	3132	512
1874	3169	184
1874	3317	190

1874	3703	225
1874	3767	195
1874	4230	250
1874	4586	170
1874	5191	241
1874	5628	166
1874	5883	212
1874	6151	214
1874	6159	319
1874	6757	191
1874	7013	308
1874	7683	211
1874	9117	219
1874	10561	342
1874	11204	382
1874	11762	182
1874	12979	181
1874	13654	162
1874	15778	190
1874	17189	255
1874	17294	472
1874	17295	363
1874	17296	316
1874	17297	286
1874	17298	184
1874	17299	179
1874	17300	178
1874	17301	159
1874	17302	159
1874	17303	159

1875	65	715
1875	81	353
1875	154	1287
1875	173	711
1875	195	1309
1875	199	511
1875	217	877
1875	222	302
1875	225	267
1875	333	420
1875	359	904
1875	420	617
1875	423	433
1875	429	277
1875	450	329
1875	453	584
1875	599	341
1875	615	1511
1875	697	346
1875	706	359
1875	757	439
1875	779	340
1875	898	2034
1875	1300	286
1875	1378	304
1875	1389	553
1875	1854	340
1875	1873	366
1875	1983	266
1875	1990	761

1875	2014	365
1875	2059	538
1875	2850	290
1875	3258	278
1875	3616	335
1875	3714	450
1875	3739	303
1875	3902	420
1875	4205	308
1875	4436	346
1875	7264	245
1875	8274	380
1875	10613	337
1875	11236	645
1875	11416	546
1875	11603	428
1875	17304	1430
1875	17305	351
1875	17306	307
1875	17307	248
1877	56 86	
1877	65 58	
1877	162 229	
1877	163 73	
1877	171 99	
1877	207 70	
1877	377 69	
1877	418 118	
1877	432 107	
1877	470 73	

1877	486 76	
1877	489 60	
1877	504 108	
1877	517 155	
1877	533 118	
1877	690 60	
1877	706 163	
1877	779 55	
1877	903 61	
1877	1048	208
1877	1191	95
1877	1196	69
1877	1246	96
1877	1390	56
1877	1418	61
1877	1510	53
1877	1705	245
1877	1833	66
1877	1988	82
1877	2137	78
1877	2346	67
1877	2429	62
1877	2771	100
1877	3411	55
1877	3767	94
1877	3812	62
1877	4805	74
1877	5423	167
1877	6396	102
1877	7071	72

1877	7177	89
1877	9688	56
1877	11031	53
1877	11272	300
1877	13448	54
1877	17320	192
1877	17321	98
1877	17322	65
1877	17323	64
1877	17324	53
1879	154 257	
1879	163 484	
1879	188 256	
1879	190 1672	
1879	198 649	
1879	220 806	
1879	226 289	
1879	227 749	
1879	230 1064	
1879	233 311	
1879	238 309	
1879	250 226	
1879	440 266	
1879	447 299	
1879	512 310	
1879	599 264	
1879	614 961	
1879	707 1697	
1879	854 310	
1879	959 1218	

1879	1116	261
1879	1118	559
1879	1119	1020
1879	1131	1355
1879	1186	410
1879	1351	241
1879	1412	412
1879	1413	284
1879	1622	239
1879	1789	230
1879	1790	267
1879	1873	220
1879	1974	639
1879	1975	311
1879	1980	271
1879	2624	222
1879	3238	317
1879	3627	1410
1879	3655	435
1879	3940	1060
1879	5423	274
1879	5490	755
1879	5845	455
1879	5847	321
1879	10046	257
1879	11760	321
1879	17326	557
1879	17327	315
1879	17328	250
1879	17329	237

1880	55	158
1880	67	1151
1880	89	347
1880	161	1985
1880	180	145
1880	190	246
1880	209	167
1880	234	1224
1880	288	3139
1880	289	2620
1880	291	145
1880	292	1864
1880	300	1494
1880	302	357
1880	316	4870
1880	333	1557
1880	342	360
1880	344	1776
1880	366	152
1880	378	1331
1880	464	103
1880	466	470
1880	472	142
1880	475	103
1880	498	12021
1880	543	478
1880	679	1988
1880	689	5174
1880	694	1549
1880	704	2742

1880	714	81
1880	790	448
1880	802	431
1880	903	318
1880	930	417
1880	1114	256
1880	1246	1765
1880	1377	93
1880	1447	311
1880	1768	176
1880	1775	86
1880	1934	426
1880	2020	30605
1880	2941	76
1880	3332	113
1880	3393	16439
1880	4814	405
1880	5453	279
1880	7750	1211
1880	7781	558
1881	51	2182
1881	67	555
1881	71	230
1881	72	238
1881	75	549
1881	155	346
1881	157	302
1881	180	1071
1881	187	305
1881	193	313

1881	227 225	
1881	253 322	
1881	257 338	
1881	288 354	
1881	289 551	
1881	301 240	
1881	306 255	
1881	310 325	
1881	316 235	
1881	351 283	
1881	455 805	
1881	459 442	
1881	468 376	
1881	474 234	
1881	511 454	
1881	538 375	
1881	889 526	
1881	905 211	
1881	913 207	
1881	951 245	
1881	1000	276
1881	1001	429
1881	1014	405
1881	1122	221
1881	1444	236
1881	2031	251
1881	2183	239
1881	2523	395
1881	2562	231
1881	3097	232

1881	3763	265
1881	4439	252
1881	4975	381
1881	4978	229
1881	5264	257
1881	5532	277
1881	13266	257
1881	17330	321
1881	17331	224
1881	17332	215
1882	266 138	
1882	1238	169
1882	2059	210
1882	2952	139
1882	5326	261
1882	6326	92
1882	9397	130
1882	9720	85
1882	10912	163
1882	12135	578
1882	17120	105
1882	17333	895
1882	17334	881
1882	17335	570
1882	17336	538
1882	17337	429
1882	17338	386
1882	17339	326
1882	17340	316
1882	17341	288

1882	17342	220
1882	17343	213
1882	17344	202
1882	17345	191
1882	17346	184
1882	17347	182
1882	17348	174
1882	17349	169
1882	17350	157
1882	17351	151
1882	17352	144
1882	17353	138
1882	17354	129
1882	17355	128
1882	17356	126
1882	17357	124
1882	17358	124
1882	17359	118
1882	17360	117
1882	17361	114
1882	17362	113
1882	17363	111
1882	17364	103
1882	17365	97
1882	17366	90
1882	17367	90
1882	17368	87
1882	17369	86
1882	17370	84
1882	17371	83

1883	159	1234
1883	333	1041
1883	599	1466
1883	609	885
1883	618	1324
1883	735	1392
1883	757	754
1883	858	789
1883	874	953
1883	959	1216
1883	985	863
1883	986	882
1883	1062	1986
1883	1213	1142
1883	1504	743
1883	1639	1145
1883	1888	816
1883	2289	2462
1883	2542	1357
1883	2616	716
1883	3053	1109
1883	3280	1128
1883	3281	737
1883	3286	738
1883	3831	1759
1883	3884	1592
1883	5413	920
1883	6155	1028
1883	6159	829
1883	6217	1429

1883	6780	905
1883	7878	787
1883	8672	951
1883	8676	949
1883	8703	820
1883	10110	1020
1883	10969	2405
1883	11289	1006
1883	11903	865
1883	12002	1440
1883	12717	728
1883	13167	851
1883	13888	1115
1883	14941	985
1883	15128	736
1883	17372	1096
1883	17373	828
1883	17374	795
1883	17375	747
1883	17376	742
1885	53	55
1885	70	109
1885	81	70
1885	103	172
1885	238	61
1885	418	487
1885	454	65
1885	621	136
1885	748	107
1885	755	48

1885	1383	117
1885	1418	98
1885	1700	90
1885	1709	146
1885	1717	68
1885	1731	95
1885	1739	93
1885	2407	100
1885	3208	53
1885	3417	54
1885	3703	140
1885	4230	179
1885	4232	53
1885	4994	58
1885	5805	77
1885	6151	93
1885	6159	336
1885	6179	91
1885	8027	116
1885	8870	139
1885	11562	52
1885	15760	73
1885	17313	89
1885	17379	877
1885	17380	275
1885	17381	167
1885	17382	150
1885	17383	119
1885	17384	108
1885	17385	93

1885	17386	71
1885	17387	68
1885	17388	62
1885	17389	61
1885	17390	61
1885	17391	57
1885	17392	52
1885	17393	52
1885	17394	51
1885	17395	47
1887	154 87	
1887	163 668	
1887	188 149	
1887	198 153	
1887	199 157	
1887	396 315	
1887	614 138	
1887	707 102	
1887	920 153	
1887	944 358	
1887	947 175	
1887	959 469	
1887	964 109	
1887	1113	171
1887	1116	173
1887	1119	441
1887	1343	100
1887	1468	105
1887	1726	2368
1887	2003	868

1887	2005	98
1887	2006	1392
1887	2370	571
1887	2558	79
1887	3472	1093
1887	3779	74
1887	3940	342
1887	4613	752
1887	4671	279
1887	4681	84
1887	4690	75
1887	7802	105
1887	7902	268
1887	8695	389
1887	11455	161
1887	13935	2294
1887	14391	195
1887	17398	1010
1887	17399	608
1887	17400	345
1887	17401	324
1887	17402	167
1887	17403	159
1887	17404	131
1887	17405	121
1887	17406	111
1887	17407	108
1887	17408	104
1887	17409	75
1887	17410	74

1888	65	323
1888	88	163
1888	203	187
1888	222	1549
1888	225	88
1888	227	1104
1888	234	1634
1888	377	299
1888	489	1330
1888	533	87
1888	572	203
1888	585	115
1888	706	116
1888	709	83
1888	715	102
1888	733	109
1888	748	886
1888	749	82
1888	859	85
1888	986	119
1888	1099	189
1888	1351	311
1888	1406	96
1888	1946	132
1888	2591	149
1888	2699	83
1888	2738	163
1888	2818	113
1888	2832	234
1888	3019	145

1888	3520	233
1888	3670	88
1888	6151	426
1888	6160	270
1888	6186	374
1888	11033	84
1888	16205	173
1888	17411	355
1888	17412	249
1888	17413	226
1888	17414	158
1888	17415	152
1888	17416	131
1888	17417	126
1888	17418	123
1888	17419	113
1888	17420	113
1888	17421	105
1888	17422	98
1888	17423	89
1889	163 3092	
1889	212 304	
1889	227 1313	
1889	554 1092	
1889	562 2030	
1889	563 184	
1889	599 581	
1889	607 2015	
1889	630 479	
1889	730 2493	

1889	735	1636
1889	863	1569
1889	868	1020
1889	1103	213
1889	1369	2660
1889	1372	4371
1889	1412	12759
1889	1413	256
1889	1415	673
1889	1416	266
1889	1425	216
1889	1639	374
1889	1806	349
1889	2127	186
1889	2265	694
1889	2343	259
1889	2787	1116
1889	2834	755
1889	2855	1670
1889	2871	678
1889	3458	334
1889	3470	162
1889	3481	230
1889	3774	4967
1889	3888	1453
1889	4180	508
1889	4251	439
1889	4460	205
1889	4538	176
1889	4675	925

1889	4784	304
1889	5172	347
1889	5844	470
1889	6165	191
1889	6267	205
1889	6362	219
1889	8794	302
1889	10412	326
1889	11501	644
1889	16138	345
1890	198 305	
1890	234 198	
1890	278 130	
1890	333 292	
1890	475 126	
1890	480 123	
1890	483 150	
1890	489 586	
1890	497 221	
1890	510 190	
1890	517 368	
1890	1249	119
1890	2380	180
1890	2713	145
1890	2731	141
1890	2747	220
1890	2922	129
1890	3563	130
1890	4244	154
1890	4892	151

1890	4893	227
1890	6193	199
1890	7639	343
1890	8350	142
1890	8619	158
1890	8624	152
1890	8726	126
1890	9765	181
1890	9788	185
1890	9789	262
1890	9791	143
1890	9821	153
1890	10508	183
1890	12775	273
1890	14692	147
1890	17230	118
1890	17424	3843
1890	17425	865
1890	17426	402
1890	17427	268
1890	17428	255
1890	17429	196
1890	17430	190
1890	17431	180
1890	17432	164
1890	17433	159
1890	17434	155
1890	17435	140
1890	17436	135
1890	17437	115

1891	81	42
1891	101	24
1891	206	18
1891	489	19
1891	744	89
1891	762	105
1891	2154	46
1891	2158	204
1891	2193	98
1891	2667	87
1891	2671	103
1891	2681	36
1891	2699	49
1891	2713	28
1891	2824	45
1891	3827	23
1891	4316	24
1891	4734	54
1891	4852	24
1891	4905	54
1891	5052	65
1891	7733	44
1891	10491	27
1891	16748	56
1891	17194	21
1891	17438	104
1891	17439	86
1891	17440	77
1891	17441	61
1891	17442	55

1891	17443	53
1891	17444	48
1891	17445	42
1891	17446	42
1891	17447	39
1891	17448	34
1891	17449	31
1891	17450	27
1891	17451	27
1891	17452	26
1891	17453	25
1891	17454	25
1891	17455	24
1891	17456	24
1891	17457	24
1891	17458	22
1891	17459	22
1891	17460	21
1891	17461	21
1891	17462	19
1892	65 91	
1892	86 125	
1892	182 109	
1892	227 190	
1892	229 239	
1892	230 123	
1892	291 134	
1892	294 92	
1892	333 162	
1892	348 104	

1892	403 132	
1892	447 160	
1892	481 167	
1892	533 124	
1892	540 180	
1892	585 173	
1892	728 283	
1892	928 94	
1892	1048	118
1892	1091	118
1892	1242	156
1892	1244	131
1892	1378	119
1892	1469	97
1892	1689	96
1892	1772	140
1892	2265	91
1892	2524	176
1892	2600	89
1892	2614	93
1892	2783	90
1892	3373	121
1892	3460	107
1892	3489	98
1892	3983	118
1892	4155	89
1892	4616	138
1892	5000	100
1892	7180	87
1892	8307	99

1892	8830	171
1892	9749	144
1892	10182	198
1892	12145	118
1892	12773	130
1892	14501	128
1892	16840	130
1892	16892	165
1892	17463	257
1892	17464	197
1893	17465	1
1893	17466	1
1893	17467	1
1893	17468	1
1894	67	357
1894	89	1049
1894	97	367
1894	184	511
1894	204	451
1894	257	393
1894	288	1362
1894	289	3892
1894	291	580
1894	292	638
1894	293	559
1894	294	166
1894	295	1133
1894	300	325
1894	302	780
1894	309	105

1894	310 124	
1894	311 109	
1894	317 184	
1894	329 608	
1894	330 305	
1894	331 486	
1894	333 595	
1894	349 201	
1894	359 227	
1894	461 187	
1894	465 615	
1894	468 229	
1894	475 635	
1894	523 1277	
1894	525 143	
1894	527 433	
1894	891 201	
1894	903 427	
1894	908 199	
1894	974 223	
1894	1037	883
1894	1451	550
1894	1459	103
1894	1934	158
1894	2083	116
1894	3489	245
1894	8178	127
1894	9902	268
1894	9905	207
1894	17283	219

1894	17284	265
1894	17285	489
1894	17469	297
1894	17470	112
1895	55	1245
1895	65	344
1895	67	667
1895	89	3666
1895	229	1232
1895	257	1121
1895	285	367
1895	288	2247
1895	289	1591
1895	292	1460
1895	293	387
1895	295	15309
1895	299	592
1895	300	633
1895	301	716
1895	302	605
1895	306	649
1895	310	1303
1895	314	1194
1895	317	382
1895	320	590
1895	329	650
1895	331	665
1895	332	1858
1895	349	928
1895	410	383

1895	455 319	
1895	468 1221	
1895	475 406	
1895	498 363	
1895	525 463	
1895	538 787	
1895	546 369	
1895	701 2481	
1895	903 535	
1895	907 539	
1895	908 492	
1895	913 3576	
1895	1400	310
1895	1935	449
1895	1937	546
1895	2089	850
1895	2091	440
1895	2092	1805
1895	2521	992
1895	2523	563
1895	3200	324
1895	4566	751
1895	17471	564
1895	17472	506
1896	154 788	
1896	170 597	
1896	173 406	
1896	199 210	
1896	210 861	
1896	217 1316	

1896	223 183	
1896	418 1202	
1896	444 969	
1896	621 893	
1896	622 314	
1896	632 240	
1896	755 369	
1896	768 278	
1896	779 244	
1896	805 196	
1896	809 527	
1896	813 490	
1896	815 400	
1896	823 183	
1896	964 155	
1896	968 162	
1896	977 372	
1896	1100	688
1896	1195	416
1896	1226	148
1896	1394	151
1896	1398	147
1896	1401	213
1896	1541	244
1896	2579	192
1896	2850	442
1896	3411	914
1896	3621	297
1896	4103	144
1896	4118	352

1896	4923	1227
1896	4934	413
1896	5065	236
1896	5753	176
1896	5969	403
1896	8459	1204
1896	8468	457
1896	10650	163
1896	14208	565
1896	15055	199
1896	17473	206
1896	17474	195
1896	17475	159
1896	17476	153
1897	190 160	
1897	278 572	
1897	306 169	
1897	330 760	
1897	331 159	
1897	412 299	
1897	466 75	
1897	475 9532	
1897	491 190	
1897	512 76	
1897	527 627	
1897	547 2273	
1897	562 429	
1897	614 455	
1897	706 142	
1897	707 1347	

1897	724 91	
1897	843 221	
1897	856 277	
1897	907 95	
1897	918 647	
1897	982 375	
1897	1358	100
1897	1369	467
1897	1402	201
1897	1412	695
1897	1449	93
1897	1455	140
1897	1613	80
1897	1672	1707
1897	1873	533
1897	1974	196
1897	2179	1623
1897	2242	718
1897	2347	480
1897	2924	303
1897	3084	327
1897	3317	113
1897	3754	713
1897	4130	453
1897	5988	65
1897	6633	152
1897	7338	231
1897	7766	99
1897	9384	133
1897	11685	137

1897	11695	432
1897	14280	90
1897	16697	228
1897	16995	67
1898	65	1643
1898	67	664
1898	71	1186
1898	89	896
1898	265	3278
1898	289	1206
1898	291	2346
1898	292	3523
1898	294	1169
1898	297	1041
1898	300	1048
1898	302	11318
1898	310	716
1898	318	1429
1898	325	3096
1898	333	3110
1898	344	810
1898	359	769
1898	378	1180
1898	461	16573
1898	466	677
1898	518	854
1898	523	1381
1898	542	1061
1898	544	917
1898	674	824

1898	679	1056
1898	686	760
1898	697	1243
1898	701	821
1898	704	1943
1898	790	1696
1898	802	873
1898	908	683
1898	909	7420
1898	955	738
1898	972	769
1898	1034	2847
1898	1428	793
1898	1691	680
1898	2392	651
1898	4254	706
1898	6456	737
1898	7289	3673
1898	9567	1193
1898	10016	5090
1898	10017	1510
1898	10020	1430
1898	10640	2089
1898	17477	1107
1899	7	2481
1899	72	4545
1899	81	1738
1899	89	580
1899	154	314
1899	173	3066

1899	190 463
1899	206 279
1899	209 149
1899	226 396
1899	233 1283
1899	288 234
1899	289 202
1899	291 1066
1899	292 381
1899	295 161
1899	298 276
1899	301 94
1899	302 270
1899	306 89
1899	316 884
1899	378 4318
1899	425 262
1899	432 80
1899	436 106
1899	490 1283
1899	496 101
1899	499 432
1899	517 438
1899	526 252
1899	537 960
1899	538 88
1899	542 185
1899	689 3409
1899	697 223
1899	701 5267

1899	703 924	
1899	757 109	
1899	843 101	
1899	903 341	
1899	961 6318	
1899	1098	5118
1899	1145	223
1899	1375	847
1899	2083	870
1899	2277	130
1899	2446	80
1899	2521	274
1899	3110	2818
1899	4716	130
1900	51 721	
1900	58 112	
1900	59 129	
1900	67 171	
1900	72 263	
1900	85 106	
1900	159 286	
1900	203 182	
1900	227 320	
1900	429 192	
1900	441 143	
1900	599 160	
1900	620 149	
1900	851 303	
1900	852 174	
1900	861 219	

1900	874	139
1900	875	124
1900	985	218
1900	986	425
1900	993	290
1900	997	112
1900	1004	111
1900	1013	123
1900	1017	113
1900	1083	694
1900	1089	228
1900	1804	141
1900	1811	118
1900	2938	223
1900	3201	143
1900	3279	124
1900	3280	200
1900	3287	183
1900	3296	104
1900	3436	143
1900	3941	190
1900	4129	219
1900	4495	127
1900	5260	216
1900	6780	118
1900	8555	158
1900	8656	171
1900	9338	102
1900	9469	147
1900	10105	106

1900	13575	104
1900	15361	285
1900	15637	110
1900	17478	127
1902	89	4277
1902	288	417
1902	289	336
1902	291	484
1902	300	344
1902	374	1538
1902	375	2005
1902	378	834
1902	379	1367
1902	498	1518
1902	499	825
1902	679	446
1902	1857	1590
1902	1919	715
1902	1922	482
1902	2086	736
1902	2088	2129
1902	2094	1499
1902	2099	372
1902	2102	3829
1902	2104	1058
1902	2111	1060
1902	2116	395
1902	3324	2297
1902	3485	499
1902	3487	707

1902	3490	499
1902	4980	328
1902	5089	1658
1902	5335	310
1902	7175	557
1902	8392	1599
1902	9087	1091
1902	9089	843
1902	9566	745
1902	9580	511
1902	9585	873
1902	10226	301
1902	10244	300
1902	10931	472
1902	12182	311
1902	16034	481
1902	17481	513
1902	17482	479
1902	17483	442
1902	17484	389
1902	17485	368
1902	17486	353
1902	17487	330
1902	17488	292
1903	55	3518
1903	58	217
1903	67	656
1903	89	511
1903	97	2799
1903	157	161

1903	251	195
1903	285	139
1903	288	392
1903	289	538
1903	292	428
1903	295	288
1903	299	129
1903	301	121
1903	310	221
1903	323	165
1903	352	130
1903	466	276
1903	530	149
1903	532	215
1903	535	406
1903	538	215
1903	548	98
1903	690	425
1903	701	1025
1903	789	145
1903	901	127
1903	903	435
1903	906	815
1903	970	109
1903	972	107
1903	1097	128
1903	1098	1198
1903	1127	103
1903	1243	99
1903	1244	197

1903	1464	150
1903	1511	96
1903	1556	102
1903	1844	215
1903	1934	418
1903	2024	112
1903	2094	249
1903	2521	237
1903	3201	99
1903	3288	114
1903	3502	189
1903	4260	95
1903	14369	243
1903	17489	171
1904	154 210	
1904	202 177	
1904	204 1637	
1904	205 1630	
1904	355 2542	
1904	366 818	
1904	609 132	
1904	620 199	
1904	622 372	
1904	715 539	
1904	734 130	
1904	858 126	
1904	1123	139
1904	1150	300
1904	1175	190
1904	1652	195

1904	1718	2912
1904	1740	129
1904	2265	299
1904	2277	612
1904	2625	237
1904	3464	337
1904	3466	330
1904	3613	392
1904	3619	260
1904	7281	1543
1904	7404	194
1904	8657	133
1904	9433	183
1904	9470	114
1904	9539	2050
1904	12194	200
1904	13065	1276
1904	14052	326
1904	15038	278
1904	15527	220
1904	16068	158
1904	16217	136
1904	16952	138
1904	17490	747
1904	17491	684
1904	17492	394
1904	17493	305
1904	17494	212
1904	17495	197
1904	17496	175

1904	17497	146
1904	17498	128
1904	17499	125
1904	17500	123
1905	51	745
1905	53	88
1905	67	689
1905	88	571
1905	182	83
1905	196	98
1905	203	257978
1905	229	387
1905	425	59
1905	429	1283
1905	435	195
1905	533	299
1905	599	128
1905	612	105
1905	615	98
1905	618	4762
1905	709	63
1905	735	84
1905	959	141
1905	962	131
1905	1090	113
1905	1095	157
1905	1162	214
1905	1164	585
1905	1171	155
1905	1213	100

1905	2407	618
1905	2415	61
1905	2531	818
1905	2584	140
1905	3258	79
1905	3673	62
1905	3689	99
1905	4069	78
1905	4199	220
1905	5782	1899
1905	6421	314
1905	6687	69
1905	9032	891
1905	9959	193
1905	11812	206
1905	15386	70
1905	15889	66
1905	17501	281
1905	17502	239
1905	17503	235
1905	17504	101
1905	17505	96
1905	17506	80
1905	17507	69
1906	65	166
1906	67	160
1906	89	171
1906	159	509
1906	183	111
1906	187	112

1906	190 261	
1906	209 5087	
1906	220 70	
1906	221 70	
1906	230 80	
1906	234 111	
1906	283 136	
1906	289 83	
1906	300 597	
1906	316 282	
1906	333 1064	
1906	377 952	
1906	422 99	
1906	436 74	
1906	439 393	
1906	486 887	
1906	498 353	
1906	536 428	
1906	538 90	
1906	540 316	
1906	542 857	
1906	714 525	
1906	903 190	
1906	959 113	
1906	1041	75
1906	1045	745
1906	1185	123
1906	1246	985
1906	1672	356
1906	1681	316

1906	2080	730
1906	2524	281
1906	2983	126
1906	3200	173
1906	3367	395
1906	3741	146
1906	3747	203
1906	5966	174
1906	6453	72
1906	7636	319
1906	7835	75
1906	8707	94
1906	13264	449
1906	15485	84
1907	95	196
1907	154	175
1907	627	243
1907	748	158
1907	987	219
1907	1156	313
1907	1411	263
1907	1787	306
1907	1829	199
1907	1832	186
1907	1844	241
1907	2668	148
1907	2673	192
1907	2681	352
1907	3280	241
1907	3426	219

1907	3992	238
1907	5173	347
1907	5803	162
1907	6585	161
1907	8513	179
1907	10468	226
1907	11638	185
1907	15083	173
1907	15086	188
1907	16666	214
1907	17508	325
1907	17509	266
1907	17510	237
1907	17511	214
1907	17512	207
1907	17513	207
1907	17514	205
1907	17515	194
1907	17516	193
1907	17517	193
1907	17518	192
1907	17519	184
1907	17520	177
1907	17521	174
1907	17522	170
1907	17523	170
1907	17524	164
1907	17525	163
1907	17526	162
1907	17527	159

1907	17528	155
1907	17529	153
1907	17530	151
1907	17531	144
1908	55	342
1908	65	774
1908	67	15544
1908	89	474
1908	99	166
1908	155	657
1908	157	2176
1908	184	153
1908	187	171
1908	204	201
1908	220	145
1908	227	730
1908	253	163
1908	261	228
1908	288	154
1908	289	3146
1908	292	4739
1908	295	943
1908	301	171
1908	302	217
1908	306	445
1908	310	301
1908	316	578
1908	328	261
1908	329	446
1908	349	201

1908	366	503
1908	511	629
1908	679	156
1908	683	146
1908	701	427
1908	714	150
1908	798	243
1908	900	240
1908	903	568
1908	913	160
1908	959	309
1908	1243	469
1908	1249	274
1908	1475	297
1908	2022	5630
1908	2024	355
1908	2346	383
1908	2523	3178
1908	2783	163
1908	3097	176
1908	3171	424
1908	3748	185
1908	4705	253
1908	6504	166
1909	7	309
1909	55	9198
1909	67	6498
1909	89	3133
1909	172	4689
1909	173	18117

1909 209 554

1909 220 655

1909 227 1370

1909 257 266

1909 289 20251

1909 295 1639

1909 300 405

1909 302 475

1909 306 239

1909 316 585

1909 325 486

1909 333 4222

1909 377 2391

1909 378 827

1909 453 485

1909 455 577

1909 459 665

1909 461 673

1909 467 271

1909 486 639

1909 498 5099

1909 499 12449

1909 511 293

1909 530 909

1909 535 551

1909 542 717

1909 680 239

1909 681 250

1909 962 9038

1909 1045 3129

1909	1453	260
1909	1505	29924
1909	1601	3800
1909	1874	371
1909	2346	248
1909	2352	396
1909	2523	605
1909	3200	1211
1909	3205	352
1909	3739	2079
1909	4802	391
1909	5966	3733
1909	17532	1766
1909	17533	497
1910	55	2122
1910	58	2573
1910	67	2974
1910	70	1289
1910	89	3291
1910	173	1965
1910	217	1461
1910	229	1182
1910	298	968
1910	316	6603
1910	423	3655
1910	425	1492
1910	430	1222
1910	532	899
1910	548	1975
1910	615	1025

1910	689 2245	
1910	697 1500	
1910	703 2645	
1910	961 2573	
1910	1075	823
1910	1097	1661
1910	1098	4831
1910	1243	2310
1910	1379	1704
1910	1383	1751
1910	1390	845
1910	1409	1450
1910	1609	1162
1910	1632	1889
1910	1934	2158
1910	1971	2536
1910	1990	812
1910	2277	1004
1910	2521	2864
1910	2591	832
1910	3502	2739
1910	4032	858
1910	4704	996
1910	4706	1120
1910	5045	849
1910	5149	1025
1910	5150	2655
1910	5836	803
1910	5891	1188
1910	5925	905

1910	7660	1467
1910	8589	914
1910	8676	1581
1910	15752	1171
1911	65	480
1911	154	1062
1911	207	1437
1911	226	1172
1911	227	787
1911	229	1766
1911	298	661
1911	420	1283
1911	424	3146
1911	428	667
1911	439	874
1911	443	364
1911	526	418
1911	539	866
1911	615	525
1911	618	614
1911	703	461
1911	863	1349
1911	1048	363
1911	1090	536
1911	1400	636
1911	1510	2613
1911	1515	387
1911	1520	403
1911	1649	362
1911	1669	560

1911	1766	621
1911	2134	730
1911	2660	1157
1911	3057	893
1911	3200	453
1911	3205	375
1911	3742	442
1911	3747	979
1911	4452	1074
1911	4793	812
1911	5544	548
1911	5549	1004
1911	7334	497
1911	8707	894
1911	8710	1280
1911	8713	1046
1911	11460	709
1911	16668	1355
1911	17534	1703
1911	17535	1415
1911	17536	955
1911	17537	690
1911	17538	482
1911	17539	343
1912	198 88	
1912	278 162	
1912	475 1540	
1912	1614	66
1912	1641	254
1912	2179	464

1912	2233	265
1912	2236	59
1912	2247	164
1912	2250	152
1912	2252	219
1912	5607	214
1912	5674	447
1912	5675	50
1912	5676	310
1912	5677	182
1912	6618	257
1912	6619	80
1912	7759	515
1912	7874	43
1912	9677	400
1912	9679	241
1912	9681	328
1912	9694	421
1912	10873	79
1912	10876	94
1912	10878	58
1912	10879	105
1912	10883	669
1912	10885	330
1912	17540	462
1912	17541	233
1912	17542	218
1912	17543	169
1912	17544	163
1912	17545	148

1912	17546	112
1912	17547	105
1912	17548	101
1912	17549	97
1912	17550	86
1912	17551	82
1912	17552	64
1912	17553	64
1912	17554	50
1912	17555	48
1912	17556	48
1912	17557	47
1912	17558	40
1912	17559	39
1914	163 1012	
1914	197 566	
1914	227 488	
1914	230 436	
1914	235 1334	
1914	250 546	
1914	511 353	
1914	562 961	
1914	630 524	
1914	715 688	
1914	728 1064	
1914	734 680	
1914	975 425	
1914	1239	426
1914	1425	470
1914	1639	308

1914	2629	551
1914	2633	381
1914	2641	313
1914	2842	497
1914	2864	472
1914	2871	762
1914	2873	415
1914	2894	471
1914	2902	385
1914	4073	826
1914	4196	443
1914	4369	383
1914	6824	509
1914	7266	571
1914	8111	301
1914	8673	442
1914	8837	296
1914	9709	296
1914	9824	420
1914	9825	345
1914	10847	1321
1914	11124	297
1914	11667	352
1914	13621	423
1914	15027	1005
1914	17562	1407
1914	17563	853
1914	17564	792
1914	17565	677
1914	17566	336

1914	17567	320
1914	17568	316
1914	17569	316
1914	17570	306
1915	55	24
1915	58	51
1915	80	28
1915	84	25
1915	89	11
1915	167	31
1915	298	32
1915	314	28
1915	352	13
1915	475	44
1915	485	25
1915	526	177
1915	530	20
1915	535	109
1915	691	32
1915	757	27
1915	1073	14
1915	1155	63
1915	1844	10
1915	1964	23
1915	3621	22
1915	4385	63
1915	4448	16
1915	4732	42
1915	6209	16
1915	6690	59

1915	10658	31
1915	12599	23
1915	14222	246
1915	16786	11
1915	17093	21
1915	17571	277
1915	17572	131
1915	17573	112
1915	17574	53
1915	17575	39
1915	17576	37
1915	17577	32
1915	17578	28
1915	17579	28
1915	17580	27
1915	17581	25
1915	17582	25
1915	17583	23
1915	17584	22
1915	17585	20
1915	17586	18
1915	17587	17
1915	17588	12
1915	17589	12
1917	89	197
1917	257	4
1917	288	21
1917	289	9
1917	290	44
1917	292	3

1917	294 7	
1917	295 8	
1917	302 2	
1917	309 3	
1917	317 12	
1917	318 15	
1917	326 13	
1917	327 7	
1917	458 2	
1917	461 4	
1917	465 5	
1917	478 2	
1917	498 6	
1917	679 11	
1917	786 9	
1917	793 3	
1917	1032	17
1917	1034	3
1917	1037	7
1917	1573	2
1917	1936	6
1917	2990	2
1917	3200	3
1917	4566	2
1917	4814	30
1917	5039	11
1917	5041	7
1917	5049	6
1917	5276	6
1917	5534	10

1917	5988	19
1917	6226	10
1917	7390	5
1917	11379	55
1917	11385	13
1917	11755	28
1917	17605	29
1917	17606	25
1917	17607	11
1917	17608	6
1917	17609	5
1917	17610	3
1917	17611	3
1917	17612	2
1918	65	26
1918	70	43
1918	154	58
1918	203	13
1918	209	59
1918	225	27
1918	226	34
1918	228	14
1918	230	71
1918	234	39
1918	235	22
1918	288	19
1918	333	16
1918	412	17
1918	475	37
1918	536	18

1918	554 31	
1918	715 37	
1918	718 16	
1918	982 14	
1918	1158	33
1918	1186	27
1918	1380	29
1918	1382	30
1918	2265	86
1918	2630	59
1918	2857	17
1918	3254	23
1918	3398	14
1918	4069	32
1918	4189	24
1918	4394	13
1918	7185	13
1918	7265	26
1918	7406	24
1918	10412	16
1918	11179	30
1918	12143	24
1918	12297	47
1918	13070	16
1918	14632	14
1918	15481	53
1918	15812	28
1918	15889	50
1918	17613	80
1918	17614	19

1918 17615 18

1918 17616 17

1918 17617 16

1918 17618 13

1919 207 466

1919 208 92

1919 210 131

1919 225 138

1919 227 35

1919 228 220

1919 229 36

1919 419 110

1919 424 378

1919 428 276

1919 431 45

1919 432 88

1919 441 211

1919 443 66

1919 716 184

1919 903 36

1919 969 47

1919 1048 272

1919 1090 94

1919 1400 60

1919 1515 344

1919 1520 43

1919 1988 106

1919 2128 40

1919 2131 120

1919 2133 150

1919	2271	174
1919	2524	139
1919	2537	34
1919	2600	50
1919	2614	26
1919	2815	71
1919	3469	32
1919	3767	67
1919	3797	48
1919	4115	27
1919	4742	48
1919	5030	190
1919	5417	63
1919	5429	85
1919	5439	53
1919	6223	30
1919	7180	37
1919	7190	39
1919	7663	27
1919	7744	27
1919	8830	128
1919	12165	111
1919	14359	43
1919	17619	73
1920	56	310
1920	65	474
1920	70	844
1920	89	243
1920	157	378
1920	159	523

1920	188 620	
1920	209 555	
1920	227 362	
1920	228 634	
1920	230 336	
1920	300 335	
1920	333 341	
1920	378 230	
1920	412 401	
1920	470 209	
1920	481 3470	
1920	486 294	
1920	498 1071	
1920	511 249	
1920	536 264	
1920	538 222	
1920	540 1082	
1920	679 1609	
1920	683 285	
1920	959 202	
1920	982 217	
1920	1033	291
1920	1035	618
1920	1040	956
1920	1043	193
1920	1047	1710
1920	1048	698
1920	1198	381
1920	1241	241
1920	1243	255

1920	1358	423
1920	1400	374
1920	1415	207
1920	1674	205
1920	1689	209
1920	2176	302
1920	2364	207
1920	2608	199
1920	3247	233
1920	6453	262
1920	9343	633
1920	12104	264
1920	15423	201
1920	15486	402
1921	230 4304	
1921	288 1620	
1921	290 509	
1921	321 642	
1921	371 2869	
1921	377 326	
1921	386 491	
1921	466 1099	
1921	475 7691	
1921	488 2046	
1921	497 318	
1921	498 3927	
1921	500 1312	
1921	536 2830	
1921	544 648	
1921	644 817	

1921	646 3938	
1921	680 504	
1921	681 5804	
1921	686 505	
1921	693 4102	
1921	808 3784	
1921	1021	413
1921	1057	1361
1921	1179	389
1921	1186	2888
1921	1200	519
1921	1406	292
1921	1453	3239
1921	1455	434
1921	1458	399
1921	1684	3042
1921	1854	967
1921	1874	2520
1921	2435	750
1921	2619	7495
1921	2843	324
1921	2940	16616
1921	3084	454
1921	4324	340
1921	5530	373
1921	6257	291
1921	7865	695
1921	10316	977
1921	11018	2475
1921	14153	746

1921	15995	1098
1921	16727	1105
1921	17620	622
1921	17621	271
1922	65	37
1922	70	47
1922	154	30
1922	159	108
1922	173	521
1922	190	95
1922	196	107
1922	206	167
1922	207	38
1922	214	37
1922	215	26
1922	217	125
1922	222	75
1922	225	30
1922	407	26
1922	421	39
1922	429	77
1922	615	38
1922	618	107
1922	629	35
1922	757	60
1922	859	56
1922	898	107
1922	1094	35
1922	1244	38
1922	1389	28

1922	1400	29
1922	1773	45
1922	1966	57
1922	2277	32
1922	2531	32
1922	2582	27
1922	2938	34
1922	3026	29
1922	3279	49
1922	3903	31
1922	4115	28
1922	4118	26
1922	4311	27
1922	5414	101
1922	6680	27
1922	9032	43
1922	12201	73
1922	13102	32
1922	13516	32
1922	13939	51
1922	15122	495
1922	17622	30
1922	17623	30
1922	17624	28
1923	7	758
1923	25	920
1923	188	1139
1923	204	608
1923	475	1188
1923	489	853

1923	514	574
1923	517	1035
1923	614	594
1923	707	1513
1923	719	809
1923	812	591
1923	843	677
1923	846	666
1923	868	641
1923	955	955
1923	1044	1178
1923	1098	3617
1923	1111	754
1923	1112	1420
1923	1118	1358
1923	1123	682
1923	1130	953
1923	1135	649
1923	1143	2615
1923	1249	716
1923	1406	654
1923	1770	1385
1923	1772	1903
1923	2419	1069
1923	3057	849
1923	3310	867
1923	4271	757
1923	4344	641
1923	4616	750
1923	4965	681

1923	5787	1111
1923	6626	909
1923	7352	868
1923	7540	572
1923	8372	818
1923	10691	1008
1923	12141	706
1923	13436	772
1923	14926	557
1923	17625	689
1923	17626	670
1923	17627	623
1923	17628	554
1923	17629	545
1924	12	1737
1924	25	1346
1924	65	3497
1924	89	1609
1924	154	8154
1924	162	2269
1924	195	1426
1924	198	2039
1924	227	2317
1924	288	2706
1924	359	5897
1924	370	22143
1924	377	1616
1924	378	4757
1924	418	1461
1924	430	5292

1924	437	2498
1924	466	1721
1924	486	8886
1924	487	4918
1924	498	66761
1924	499	5955
1924	503	2056
1924	689	4580
1924	703	2662
1924	757	2288
1924	779	1780
1924	830	2725
1924	1181	1338
1924	1196	2913
1924	1260	3516
1924	1276	1378
1924	1398	1652
1924	1861	1586
1924	2429	1636
1924	2824	1635
1924	2959	1610
1924	3034	2942
1924	3637	1694
1924	3822	2539
1924	4155	1488
1924	5149	2219
1924	6818	1936
1924	7147	4139
1924	7759	1443
1924	8331	1610

1924	17540	2293
1924	17630	4603
1924	17631	2519
1924	17632	2010
1925	209 114	
1925	333 100	
1925	460 123	
1925	486 147	
1925	492 122	
1925	498 214	
1925	691 176	
1925	758 168	
1925	759 100	
1925	768 127	
1925	779 257	
1925	814 307	
1925	816 229	
1925	972 151	
1925	1098	100
1925	1185	123
1925	1188	688
1925	1190	178
1925	1541	139
1925	1678	100
1925	1863	157
1925	1866	109
1925	1868	171
1925	2204	142
1925	2210	125
1925	2383	194

1925	2850	111
1925	2940	360
1925	4173	144
1925	4229	127
1925	4231	172
1925	4239	109
1925	4774	224
1925	4891	121
1925	5129	221
1925	5963	125
1925	6414	104
1925	6445	254
1925	6850	102
1925	7760	174
1925	8274	141
1925	8730	283
1925	9934	258
1925	11393	103
1925	12386	130
1925	16017	100
1925	17633	200
1925	17634	182
1925	17635	155
1925	17636	137
1926	53	438
1926	54	2219
1926	58	452
1926	64	840
1926	166	642
1926	172	464

1926	238	462
1926	526	488
1926	532	448
1926	858	469
1926	862	449
1926	886	500
1926	887	603
1926	1062	711
1926	1075	2551
1926	1081	398
1926	1085	2306
1926	1213	798
1926	1405	349
1926	1824	544
1926	1983	511
1926	2616	708
1926	2830	418
1926	3109	2777
1926	3206	1268
1926	3259	692
1926	3300	602
1926	3902	386
1926	4308	418
1926	4382	459
1926	4810	456
1926	5027	433
1926	5549	463
1926	5893	394
1926	9470	350
1926	10110	403

1926	10214	418
1926	10685	652
1926	10738	743
1926	12002	2011
1926	12319	411
1926	13008	448
1926	13393	710
1926	16361	813
1926	17637	929
1926	17638	792
1926	17639	648
1926	17640	642
1926	17641	624
1926	17642	381
1928	55	641
1928	64	674
1928	65	162
1928	67	1974
1928	72	115
1928	174	191
1928	199	392
1928	238	133
1928	251	242
1928	299	238
1928	340	183
1928	384	259
1928	416	382
1928	575	232
1928	985	1362
1928	1167	586

1928	1243	340
1928	1400	556
1928	1464	454
1928	1694	513
1928	1820	166
1928	1834	289
1928	1983	374
1928	2054	121
1928	2299	127
1928	2301	8131
1928	2324	189
1928	2332	153
1928	2616	167
1928	2632	982
1928	3304	235
1928	3465	152
1928	3902	210
1928	4025	916
1928	4069	271
1928	7261	184
1928	7626	182
1928	8983	130
1928	10142	136
1928	13281	117
1928	15973	221
1928	16765	807
1928	17644	1087
1928	17645	751
1928	17646	654
1928	17647	578

1928	17648	280
1928	17649	203
1928	17650	145
1928	17651	128
1929	72	908
1929	89	1480
1929	163	2052
1929	173	2111
1929	193	937
1929	229	849
1929	316	930
1929	475	978
1929	599	838
1929	706	1020
1929	857	1421
1929	864	1118
1929	877	2431
1929	910	1336
1929	917	1282
1929	959	1502
1929	1001	1098
1929	1014	846
1929	1017	869
1929	1110	2197
1929	1203	1031
1929	1298	975
1929	1343	1089
1929	1358	1149
1929	1413	1146
1929	1463	1258

1929	1513	1060
1929	1705	1241
1929	1814	857
1929	1886	980
1929	1890	1126
1929	2030	986
1929	2044	2344
1929	2236	3112
1929	2251	846
1929	2347	838
1929	2444	862
1929	2531	1221
1929	3264	2516
1929	3289	1431
1929	3481	878
1929	3769	2064
1929	3988	1927
1929	4126	1166
1929	4255	1533
1929	5175	2262
1929	8422	2069
1929	12733	1206
1929	17616	1086
1929	17652	1013
1930	390 513	
1930	401 812	
1930	1910	779
1930	1923	1392
1930	2100	1719
1930	2103	785

1930	6748	849
1930	6994	715
1930	6995	470
1930	8394	543
1930	8396	1740
1930	8397	416
1930	10937	482
1930	12801	1362
1930	12805	1292
1930	12816	595
1930	13763	955
1930	15290	358
1930	17653	4799
1930	17654	2287
1930	17655	2172
1930	17656	2167
1930	17657	1799
1930	17658	1787
1930	17659	1338
1930	17660	1165
1930	17661	962
1930	17662	886
1930	17663	853
1930	17664	841
1930	17665	738
1930	17666	723
1930	17667	680
1930	17668	613
1930	17669	564
1930	17670	558

1930	17671	499
1930	17672	492
1930	17673	433
1930	17674	421
1930	17675	408
1930	17676	408
1930	17677	385
1930	17678	378
1930	17679	375
1930	17680	373
1930	17681	344
1930	17682	332
1930	17683	317
1930	17684	316
1931	52	24
1931	65	13
1931	107	21
1931	167	14
1931	182	52
1931	206	33
1931	208	36
1931	227	577
1931	301	17
1931	355	57
1931	474	16
1931	526	22
1931	547	14
1931	603	44
1931	691	61
1931	733	29

1931	748 104	
1931	757 33	
1931	857 15	
1931	868 35	
1931	1042	18
1931	1106	43
1931	1122	13
1931	1181	15
1931	1242	60
1931	2020	18
1931	2667	73
1931	2675	28
1931	2818	28
1931	3030	52
1931	3065	59
1931	3373	57
1931	4299	17
1931	4728	21
1931	5027	14
1931	5925	42
1931	5950	17
1931	5953	23
1931	7224	23
1931	7324	14
1931	9160	25
1931	12013	26
1931	13362	24
1931	15143	17
1931	16525	35
1931	17685	41

1931	17686	19
1931	17687	16
1931	17688	16
1931	17689	15
1933	56	101
1933	163	57
1933	366	52
1933	566	64
1933	599	60
1933	605	63
1933	614	57
1933	631	79
1933	718	128
1933	750	40
1933	761	66
1933	859	136
1933	875	63
1933	879	44
1933	1639	137
1933	2277	44
1933	2477	49
1933	2641	46
1933	3289	108
1933	3299	48
1933	3310	39
1933	3431	57
1933	3444	72
1933	4062	102
1933	4597	51
1933	5045	56

1933	5862	45
1933	7265	105
1933	9839	86
1933	10394	75
1933	11485	50
1933	11826	41
1933	12338	60
1933	13416	45
1933	13879	66
1933	14620	39
1933	15639	59
1933	17699	129
1933	17700	103
1933	17701	94
1933	17702	90
1933	17703	71
1933	17704	67
1933	17705	59
1933	17706	53
1933	17707	53
1933	17708	52
1933	17709	49
1933	17710	41
1933	17711	40
1934	374 57	
1934	375 328	
1934	379 114	
1934	1857	57
1934	1912	39
1934	1922	81

1934	2086	76
1934	2092	70
1934	2094	135
1934	2095	108
1934	2097	52
1934	2098	34
1934	2099	43
1934	2102	248
1934	2115	28
1934	2746	41
1934	3324	145
1934	3483	93
1934	3552	133
1934	3554	207
1934	4980	39
1934	5083	350
1934	5089	66
1934	5563	116
1934	8392	123
1934	9089	45
1934	10226	32
1934	10228	32
1934	10233	91
1934	10237	52
1934	10244	51
1934	14324	41
1934	15325	191
1934	17485	29
1934	17712	120
1934	17713	79

1934	17714	57
1934	17715	49
1934	17716	47
1934	17717	45
1934	17718	43
1934	17719	40
1934	17720	40
1934	17721	37
1934	17722	34
1934	17723	32
1934	17724	32
1934	17725	32
1934	17726	31
1934	17727	27
1935	189 546	
1935	230 198	
1935	328 1252	
1935	377 299	
1935	378 1067	
1935	486 359	
1935	488 1977	
1935	497 227	
1935	498 3153	
1935	500 848	
1935	503 659	
1935	519 3177	
1935	540 1497	
1935	704 385	
1935	706 194	
1935	707 590	

1935	779 2176	
1935	808 1074	
1935	810 379	
1935	816 825	
1935	923 436	
1935	1044	332
1935	1099	406
1935	1180	1347
1935	1183	266
1935	1184	336
1935	1200	1379
1935	1246	642
1935	1672	2767
1935	1797	292
1935	1803	356
1935	1853	580
1935	1854	791
1935	1874	539
1935	2380	380
1935	2435	493
1935	2738	315
1935	2940	310
1935	2956	393
1935	2959	2289
1935	5459	289
1935	5947	489
1935	7639	609
1935	8350	1633
1935	9828	245
1935	10731	218

1935	16382	2031
1935	17728	421
1935	17729	394
1935	17730	213
1936	154 331	
1936	207 3035	
1936	220 103	
1936	227 108	
1936	246 360	
1936	261 208	
1936	316 73	
1936	424 3016	
1936	436 147	
1936	439 5596	
1936	472 77	
1936	498 96	
1936	533 223	
1936	683 185	
1936	708 243	
1936	714 207	
1936	962 230	
1936	964 161	
1936	1241	70
1936	1651	868
1936	1755	1577
1936	1761	416
1936	1764	132
1936	1766	1200
1936	1768	499
1936	1775	226

1936	1776	364
1936	2988	635
1936	3205	863
1936	3391	163
1936	3741	253
1936	3747	497
1936	4819	168
1936	5745	180
1936	6472	133
1936	6724	1122
1936	8716	88
1936	10418	289
1936	15197	913
1936	15260	329
1936	16041	87
1936	17731	880
1936	17732	315
1936	17733	204
1936	17734	108
1936	17735	100
1936	17736	82
1936	17737	80
1936	17738	79
1936	17739	71
1937	97	559
1937	159	2512
1937	163	1078
1937	212	773
1937	227	23670
1937	234	723

1937	261	1522
1937	289	956
1937	292	370
1937	377	487
1937	378	366
1937	486	5136
1937	498	35144
1937	499	562
1937	562	2509
1937	646	512
1937	679	18135
1937	680	3547
1937	705	827
1937	706	2546
1937	712	530
1937	739	654
1937	966	746
1937	971	386
1937	1041	1876
1937	1047	1811
1937	1048	529
1937	1412	5766
1937	1438	567
1937	1513	1243
1937	1775	673
1937	1807	3239
1937	2176	979
1937	3127	930
1937	3308	1163
1937	3313	793

1937	3384	365
1937	3859	612
1937	4818	1722
1937	4823	714
1937	5745	1774
1937	5754	1352
1937	6472	923
1937	6534	1185
1937	6794	498
1937	8732	409
1937	9187	641
1937	11796	1386
1937	17740	759
1937	17741	547
1938	199 1118	
1938	210 1066	
1938	217 1601	
1938	229 1285	
1938	419 555	
1938	424 1966	
1938	433 319	
1938	434 298	
1938	615 3994	
1938	629 228	
1938	683 667	
1938	730 219	
1938	959 233	
1938	982 4576	
1938	1090	484
1938	1632	2858

1938	1672	267
1938	1782	303
1938	2121	978
1938	2407	2614
1938	2422	654
1938	2424	2707
1938	2463	441
1938	2622	292
1938	2625	612
1938	3258	217
1938	3341	1425
1938	3712	397
1938	4079	5567
1938	4199	778
1938	4223	299
1938	4369	2174
1938	4444	314
1938	5452	2111
1938	5546	908
1938	6836	10547
1938	8016	303
1938	8210	1929
1938	11442	275
1938	17742	1422
1938	17743	1063
1938	17744	899
1938	17745	628
1938	17746	610
1938	17747	396
1938	17748	330

1938	17749	309
1938	17750	259
1938	17751	256
1938	17752	250
1939	154 401	
1939	227 499	
1939	1121	929
1939	1123	232
1939	1130	6113
1939	1412	475
1939	1491	3413
1939	2507	457
1939	5605	236
1939	6558	272
1939	7119	237
1939	7900	1397
1939	8400	1882
1939	10228	487
1939	15277	330
1939	17715	382
1939	17753	3491
1939	17754	3019
1939	17755	1977
1939	17756	1174
1939	17757	1037
1939	17758	770
1939	17759	636
1939	17760	620
1939	17761	538
1939	17762	386

1939	17763	380
1939	17764	373
1939	17765	350
1939	17766	294
1939	17767	272
1939	17768	272
1939	17769	263
1939	17770	246
1939	17771	234
1939	17772	234
1939	17773	227
1939	17774	224
1939	17775	220
1939	17776	211
1941	300 215	
1941	324 201	
1941	424 318	
1941	464 229	
1941	470 157	
1941	481 335	
1941	497 198	
1941	687 763	
1941	716 249	
1941	830 285	
1941	1043	365
1941	1045	199
1941	1048	932
1941	1105	224
1941	1186	222
1941	1191	207

1941	1198	273
1941	1201	566
1941	1502	381
1941	1515	233
1941	1672	289
1941	1689	170
1941	2121	207
1941	3370	162
1941	3373	368
1941	4802	190
1941	5322	171
1941	5950	147
1941	6453	304
1941	6534	145
1941	6554	174
1941	6558	175
1941	6769	149
1941	7787	418
1941	7832	166
1941	8244	428
1941	10318	426
1941	11460	189
1941	12104	544
1941	12791	329
1941	13468	2347
1941	14223	200
1941	16049	183
1941	16567	202
1941	17786	509
1941	17787	216

1941	17788	200
1941	17789	195
1941	17790	184
1941	17791	162
1942	56	5195
1942	58	4211
1942	59	4874
1942	67	5804
1942	72	88079
1942	76	4512
1942	89	2545
1942	167	16812
1942	533	3996
1942	601	7022
1942	1001	4474
1942	1014	47632
1942	1019	5974
1942	1062	6513
1942	1072	7482
1942	1122	2351
1942	1243	4703
1942	1713	2333
1942	1804	4727
1942	1827	2738
1942	1832	2372
1942	1836	5873
1942	1878	2401
1942	1890	4574
1942	2487	2184
1942	2535	3265

1942	3281	2767
1942	4308	6014
1942	5152	2431
1942	6742	2077
1942	7442	5958
1942	10625	2037
1942	10685	5299
1942	13558	5418
1942	17792	6823
1942	17793	6704
1942	17794	6085
1942	17795	5601
1942	17796	4871
1942	17797	4661
1942	17798	4042
1942	17799	3967
1942	17800	3702
1942	17801	2905
1942	17802	2727
1942	17803	2577
1942	17804	2488
1942	17805	2356
1942	17806	2271
1942	17807	2100
1944	15 185	
1944	163 952	
1944	227 195	
1944	405 504	
1944	439 318	
1944	500 615	

1944	614	539
1944	707	711
1944	710	898
1944	724	483
1944	729	422
1944	779	182
1944	837	194
1944	917	242
1944	918	833
1944	921	379
1944	923	519
1944	927	663
1944	953	513
1944	954	595
1944	963	1473
1944	1127	257
1944	1132	1043
1944	1264	947
1944	1522	166
1944	1669	414
1944	1778	653
1944	1974	222
1944	2342	424
1944	2370	153
1944	2379	4187
1944	2569	508
1944	2844	408
1944	3088	429
1944	3637	451
1944	3719	287

1944	4087	345
1944	4277	155
1944	4680	508
1944	5158	1113
1944	7100	327
1944	10209	394
1944	17811	1030
1944	17812	495
1944	17813	440
1944	17814	359
1944	17815	199
1944	17816	198
1944	17817	155
1944	17818	149
1946	55	707
1946	58	1767
1946	65	401
1946	70	372
1946	191	433
1946	257	1346
1946	265	382
1946	279	529
1946	288	684
1946	291	371
1946	292	1844
1946	294	1333
1946	295	488
1946	296	398
1946	300	381
1946	301	485

1946	302 546	
1946	326 553	
1946	389 533	
1946	406 620	
1946	518 1033	
1946	575 366	
1946	727 400	
1946	903 780	
1946	930 1672	
1946	936 719	
1946	955 571	
1946	1098	424
1946	1114	445
1946	1379	1283
1946	1556	483
1946	1688	1927
1946	1772	379
1946	1833	394
1946	1848	381
1946	2044	12953
1946	2051	1334
1946	2054	418
1946	3197	516
1946	3466	407
1946	4136	1539
1946	4147	1371
1946	4554	700
1946	5079	529
1946	5678	382
1946	7157	624

1946	8853	811
1946	12370	380
1946	17833	681
1946	17834	405
1947	45	243
1947	64	127
1947	70	101
1947	72	48
1947	88	228
1947	152	112
1947	190	77
1947	210	146
1947	225	82
1947	226	284
1947	228	187
1947	233	170
1947	234	141
1947	238	74
1947	298	153
1947	488	1277
1947	498	139
1947	526	64
1947	532	68
1947	614	156
1947	703	1369
1947	707	60
1947	716	59
1947	748	269
1947	808	98
1947	810	225

1947	859 337	
1947	967 110	
1947	969 438	
1947	1075	327
1947	1091	114
1947	1099	134
1947	1104	866
1947	1106	98
1947	1116	99
1947	1118	2022
1947	1131	295
1947	1239	1522
1947	1376	720
1947	1377	103
1947	1400	63
1947	1701	148
1947	2014	66
1947	2542	204
1947	3109	316
1947	4177	132
1947	4316	56
1947	4821	150
1947	6414	144
1947	16390	93
1948	89 269	
1948	191 110	
1948	228 331	
1948	230 72	
1948	300 436	
1948	302 83	

1948	321 120	
1948	374 146	
1948	464 117	
1948	471 203	
1948	486 243	
1948	498 666	
1948	646 69	
1948	674 84	
1948	680 1541	
1948	687 131	
1948	790 131	
1948	972 83	
1948	1035	109
1948	1040	268
1948	1045	72
1948	1246	133
1948	1438	190
1948	1444	80
1948	1656	140
1948	1673	628
1948	1684	353
1948	2462	481
1948	2498	344
1948	2653	74
1948	3074	855
1948	3187	584
1948	3193	238
1948	3204	104
1948	3377	115
1948	3738	112

1948	4260	283
1948	4716	150
1948	5465	179
1948	9183	117
1948	11718	83
1948	13645	186
1948	13739	184
1948	15892	1350
1948	15903	261
1948	16920	80
1948	17835	118
1948	17836	101
1948	17837	87
1948	17838	78
1949	72	898
1949	88	1703
1949	154	398
1949	203	615
1949	207	10617
1949	208	3483
1949	217	315
1949	220	2030
1949	227	1613
1949	228	185
1949	229	216
1949	234	366
1949	424	739
1949	427	275
1949	428	2257
1949	432	1133

1949	533	1646
1949	969	5583
1949	1048	1718
1949	1090	1113
1949	1099	677
1949	1179	411
1949	1239	335
1949	1249	417
1949	1512	349
1949	1515	274
1949	1520	1201
1949	1639	355
1949	2128	433
1949	2131	320
1949	2524	4706
1949	3019	472
1949	3371	508
1949	3400	190
1949	4115	265
1949	5030	353
1949	5439	474
1949	5928	5125
1949	7187	530
1949	7190	356
1949	9953	190
1949	11950	351
1949	15233	234
1949	17839	2804
1949	17840	727
1949	17841	353

1949	17842	346
1949	17843	331
1949	17844	239
1949	17845	199
1950	59	412
1950	81	442
1950	154	577
1950	166	1082
1950	172	291
1950	173	1110
1950	190	336
1950	199	426
1950	217	749
1950	221	303
1950	225	780
1950	233	2311
1950	378	610
1950	383	634
1950	403	382
1950	425	766
1950	429	933
1950	430	327
1950	436	593
1950	441	483
1950	450	346
1950	498	1216
1950	499	458
1950	517	421
1950	542	333
1950	615	852

1950	618	291
1950	646	444
1950	679	661
1950	680	307
1950	698	463
1950	703	332
1950	898	343
1950	972	316
1950	1062	932
1950	1089	293
1950	1118	499
1950	1206	480
1950	1424	1272
1950	2014	310
1950	3057	365
1950	3186	376
1950	3744	402
1950	4218	366
1950	4618	319
1950	4918	620
1950	5027	443
1950	6217	305
1950	9404	1035
1950	10214	579
1951	88	599
1951	200	297
1951	228	254
1951	230	308
1951	298	242
1951	412	395

1951	425 341	
1951	440 649	
1951	982 508	
1951	1186	261
1951	1398	230
1951	1405	213
1951	1645	253
1951	1944	601
1951	1976	308
1951	2415	420
1951	2637	760
1951	3057	437
1951	3258	278
1951	3333	560
1951	3673	312
1951	3685	307
1951	3744	236
1951	4184	697
1951	4199	235
1951	4217	355
1951	4354	319
1951	4387	237
1951	4802	502
1951	4821	481
1951	5305	456
1951	5628	228
1951	6452	411
1951	6773	246
1951	7743	258
1951	8125	893

1951	8270	424
1951	10564	654
1951	10955	247
1951	12114	259
1951	12918	210
1951	15994	581
1951	17846	1135
1951	17847	716
1951	17848	436
1951	17849	412
1951	17850	324
1951	17851	278
1951	17852	250
1951	17853	212
1952	7	128
1952	154	140
1952	187	100
1952	199	142
1952	207	250
1952	221	254
1952	225	358
1952	227	937
1952	228	101
1952	417	198
1952	424	312
1952	439	155
1952	450	184
1952	453	141
1952	714	201
1952	715	105

1952	719 121	
1952	903 117	
1952	982 146	
1952	1011	160
1952	1090	171
1952	1109	105
1952	1249	217
1952	1376	100
1952	1755	695
1952	1761	176
1952	1766	124
1952	1775	466
1952	2460	191
1952	2786	128
1952	2815	187
1952	3386	111
1952	3391	160
1952	3475	120
1952	3741	117
1952	4709	127
1952	4818	101
1952	5180	277
1952	5414	145
1952	5441	109
1952	6217	119
1952	6724	101
1952	6726	100
1952	9132	147
1952	11484	408
1952	13417	184

1952	14317	136
1952	17854	364
1952	17855	214
1952	17856	120
1954	65	576
1954	207	183
1954	230	333
1954	257	172
1954	278	101
1954	288	238
1954	290	55
1954	292	12312
1954	295	1248
1954	298	500
1954	300	219
1954	301	84
1954	302	143
1954	312	66
1954	317	54
1954	320	117
1954	327	303
1954	328	55
1954	330	948
1954	331	1915
1954	377	1282
1954	466	148
1954	468	246
1954	475	245
1954	482	94
1954	485	388

1954	491 368	
1954	525 91	
1954	528 83	
1954	547 5896	
1954	701 2222	
1954	735 54	
1954	841 44	
1954	1037	45
1954	1044	319
1954	1145	212
1954	1243	64
1954	1402	62
1954	1613	1016
1954	1860	43
1954	2083	158
1954	2226	124
1954	2978	46
1954	3317	49
1954	3318	1009
1954	3712	121
1954	5678	113
1954	6226	50
1954	9684	108
1954	14278	129
1955	65 33	
1955	154 26	
1955	170 81	
1955	199 50	
1955	210 41	
1955	217 93	

1955	225	27
1955	227	40
1955	229	29
1955	416	33
1955	425	35
1955	434	32
1955	445	35
1955	602	40
1955	606	46
1955	622	29
1955	626	44
1955	630	31
1955	898	32
1955	1091	28
1955	1518	39
1955	1614	26
1955	1633	32
1955	1717	29
1955	1954	29
1955	2425	43
1955	3078	28
1955	3250	47
1955	3775	43
1955	4779	26
1955	6213	91
1955	6343	48
1955	7015	28
1955	7826	35
1955	9478	49
1955	10042	26

1955	14065	39
1955	17865	69
1955	17866	52
1955	17867	49
1955	17868	38
1955	17869	38
1955	17870	33
1955	17871	32
1955	17872	32
1955	17873	28
1955	17874	28
1955	17875	27
1955	17876	27
1955	17877	25
1956	51	817
1956	59	384
1956	63	1262
1956	67	634
1956	89	1149
1956	157	206
1956	187	5845
1956	289	162
1956	295	189
1956	374	359
1956	375	569
1956	379	161
1956	389	1337
1956	511	420
1956	877	4244
1956	972	434

1956	1001	1462
1956	1064	5321
1956	1098	255
1956	1161	176
1956	1705	339
1956	1883	172
1956	2086	186
1956	2104	1022
1956	2546	185
1956	3202	472
1956	3486	140
1956	3487	971
1956	3674	1206
1956	3832	605
1956	4493	140
1956	4880	2085
1956	5000	380
1956	5089	2174
1956	5094	466
1956	6064	457
1956	6497	384
1956	6519	348
1956	6733	1561
1956	6739	185
1956	6939	194
1956	9087	1969
1956	9090	238
1956	9116	128
1956	13126	220
1956	17878	346

1956	17879	321
1956	17880	197
1956	17881	149
1956	17882	145
1957	217	444
1957	227	1040
1957	245	362
1957	257	341
1957	267	468
1957	289	764
1957	292	325
1957	300	279
1957	314	328
1957	330	429
1957	344	624
1957	356	727
1957	366	274
1957	412	378
1957	527	428
1957	537	313
1957	540	1803
1957	718	271
1957	892	401
1957	1105	837
1957	1378	552
1957	1447	241
1957	1613	454
1957	1866	273
1957	2179	233
1957	2439	362

1957	2442	792
1957	2453	264
1957	2468	342
1957	2637	898
1957	2840	266
1957	3097	410
1957	3317	454
1957	3341	376
1957	3583	324
1957	3584	440
1957	3587	351
1957	3588	444
1957	3616	941
1957	3748	745
1957	4118	581
1957	4262	479
1957	5267	996
1957	6247	297
1957	6294	279
1957	6298	624
1957	11550	627
1957	12573	513
1957	17883	1096
1957	17884	329
1958	51	37
1958	53	47
1958	59	33
1958	72	41
1958	96	36
1958	159	68

1958	167	53
1958	199	37
1958	227	38
1958	233	55
1958	429	68
1958	433	34
1958	434	67
1958	441	76
1958	487	60
1958	599	42
1958	618	41
1958	757	80
1958	863	41
1958	898	132
1958	981	34
1958	989	33
1958	997	49
1958	1062	58
1958	1097	58
1958	1110	50
1958	1203	62
1958	1504	46
1958	1899	34
1958	2407	70
1958	2433	42
1958	2531	39
1958	2834	35
1958	2938	49
1958	3201	71
1958	3411	59

1958	3739	55
1958	5452	33
1958	5785	48
1958	6458	41
1958	8676	43
1958	13012	34
1958	13085	634
1958	15593	33
1958	17885	210
1958	17886	159
1958	17887	126
1958	17888	64
1958	17889	56
1958	17890	52
1959	55	135
1959	89	4130
1959	161	467
1959	209	419
1959	288	398
1959	289	620
1959	292	721
1959	295	272
1959	300	460
1959	302	206
1959	304	68
1959	306	69
1959	321	167
1959	325	152
1959	333	172
1959	344	228

1959	458	1738
1959	464	595
1959	466	1094
1959	471	96
1959	498	90
1959	524	440
1959	526	344
1959	541	166
1959	544	92
1959	646	436
1959	679	1308
1959	680	50
1959	681	1114
1959	686	68
1959	689	577
1959	692	201
1959	704	287
1959	903	207
1959	1037	91
1959	1042	93
1959	1045	56
1959	1047	177
1959	1054	334
1959	1243	1331
1959	1429	59
1959	1475	60
1959	1698	106
1959	2801	187
1959	3200	235
1959	7434	217

1959	8360	239
1959	13743	128
1959	17891	203
1959	17892	49
1961	89	3983
1961	191	5104
1961	257	3502
1961	288	2922
1961	289	2433
1961	291	1627
1961	294	38765
1961	300	1665
1961	301	3107
1961	302	1327
1961	307	2374
1961	314	3591
1961	317	2902
1961	323	4628
1961	327	10778
1961	344	21139
1961	352	1712
1961	461	1756
1961	482	1712
1961	498	1175
1961	547	2003
1961	680	2658
1961	681	2401
1961	686	2125
1961	701	2127
1961	786	1350

1961	908 3245	
1961	915 5638	
1961	1037	3403
1961	1059	2047
1961	1459	6037
1961	1596	1123
1961	1602	1594
1961	1613	2330
1961	1934	2282
1961	2977	3637
1961	3081	2538
1961	3317	3100
1961	3318	7805
1961	3322	2832
1961	3716	1448
1961	4487	2587
1961	4696	1972
1961	6389	2232
1961	6631	3207
1961	7340	1951
1961	8071	2812
1961	10608	2297
1961	12535	1163
1961	17899	2505
1962	59 623	
1962	84 930	
1962	203 611	
1962	210 504	
1962	225 268	
1962	432 520	

1962	620	334
1962	757	319
1962	1062	691
1962	1101	261
1962	1171	491
1962	1405	728
1962	1888	312
1962	1954	309
1962	2271	286
1962	2587	271
1962	2815	220
1962	3281	281
1962	3689	246
1962	4025	611
1962	4763	435
1962	5027	234
1962	5429	221
1962	6032	369
1962	6847	297
1962	7663	387
1962	7665	284
1962	8675	493
1962	8705	382
1962	9860	232
1962	10033	629
1962	10248	460
1962	10615	306
1962	12165	297
1962	12491	295
1962	13660	424

1962	15122	224
1962	15551	444
1962	17798	261
1962	17900	619
1962	17901	484
1962	17902	434
1962	17903	427
1962	17904	337
1962	17905	327
1962	17906	311
1962	17907	279
1962	17908	236
1962	17909	224
1962	17910	219
1963	67	532
1963	89	3021
1963	97	189
1963	257	430
1963	265	466
1963	288	582
1963	289	2168
1963	290	1039
1963	291	2403
1963	292	390
1963	294	1431
1963	295	1186
1963	296	388
1963	298	431
1963	300	1713
1963	302	279

1963 323 2987

1963 344 10590

1963 349 210

1963 352 269

1963 386 329

1963 389 350

1963 412 209

1963 461 4429

1963 466 1807

1963 525 271

1963 528 613

1963 532 445

1963 679 274

1963 680 271

1963 681 1361

1963 686 855

1963 793 742

1963 892 724

1963 907 180

1963 959 219

1963 1121 1363

1963 1459 1735

1963 1464 202

1963 1694 1130

1963 1705 1609

1963 2446 970

1963 2626 213

1963 3763 203

1963 4917 962

1963 4918 172

1963	4923	1146
1963	7777	523
1963	8422	178
1963	17911	212
1964	59	152
1964	65	104
1964	72	1876
1964	159	270
1964	199	84
1964	225	1128
1964	228	2531
1964	229	1219
1964	420	400
1964	424	4549
1964	425	409
1964	429	443
1964	441	82
1964	599	357
1964	612	137
1964	757	130
1964	863	199
1964	874	129
1964	1090	3425
1964	1097	715
1964	1112	81
1964	1117	146
1964	1130	113
1964	1390	79
1964	1777	118
1964	1976	2149

1964	1986	148
1964	2130	92
1964	2134	1405
1964	2139	93
1964	2268	79
1964	2282	118
1964	2416	274
1964	2535	171
1964	2611	94
1964	3770	172
1964	4089	154
1964	4443	134
1964	5147	156
1964	6726	188
1964	6824	162
1964	7177	202
1964	7674	81
1964	9727	133
1964	11463	145
1964	12821	82
1964	13406	110
1964	14435	229
1964	16668	104
1964	17912	100
1965	55	366
1965	58	312
1965	67	396
1965	89	254
1965	157	302
1965	239	363

1965	241	225
1965	257	473
1965	262	178
1965	285	549
1965	287	234
1965	288	326
1965	289	359
1965	290	176
1965	292	356
1965	294	183
1965	295	369
1965	300	352
1965	301	362
1965	302	422
1965	306	212
1965	310	185
1965	314	446
1965	320	220
1965	322	326
1965	330	245
1965	349	232
1965	466	194
1965	525	261
1965	601	215
1965	680	197
1965	686	240
1965	707	592
1965	748	297
1965	1098	222
1965	1444	224

1965	1844	182
1965	1935	185
1965	2018	243
1965	2022	183
1965	2392	179
1965	3490	185
1965	4566	460
1965	6374	350
1965	6562	338
1965	8188	199
1965	11079	177
1965	17913	1030
1965	17914	460
1965	17915	416
1966	65	934
1966	154	186
1966	166	151
1966	190	1133
1966	198	314
1966	209	264
1966	220	482
1966	221	164
1966	229	1188
1966	230	5781
1966	234	861
1966	316	222
1966	366	405
1966	371	540
1966	378	261
1966	412	624

1966	424	414
1966	472	1256
1966	488	232
1966	490	152
1966	533	1868
1966	536	1064
1966	614	282
1966	683	259
1966	689	309
1966	716	212
1966	720	159
1966	808	301
1966	966	357
1966	982	1066
1966	1048	442
1966	1126	150
1966	1375	225
1966	1377	192
1966	1406	219
1966	1470	158
1966	1639	232
1966	1672	463
1966	1854	184
1966	1866	269
1966	1874	161
1966	2539	165
1966	3097	585
1966	3754	432
1966	3859	554
1966	6391	399

1966	6678	171
1966	7406	1223
1966	17916	374
1966	17917	157
1968	7	264
1968	176	106
1968	198	479
1968	220	215
1968	226	162
1968	227	145
1968	234	638
1968	377	343
1968	614	1243
1968	706	663
1968	707	1487
1968	710	1058
1968	724	3386
1968	735	99
1968	854	359
1968	856	180
1968	860	213
1968	917	557
1968	922	117
1968	952	113
1968	1044	121
1968	1104	405
1968	1119	189
1968	1249	1284
1968	1358	131
1968	1360	405

1968	1369	2383
1968	1372	567
1968	1412	148
1968	1778	97
1968	1873	232
1968	2342	101
1968	2346	633
1968	2379	122
1968	2595	135
1968	2789	327
1968	2791	131
1968	2835	168
1968	2836	143
1968	2975	106
1968	3475	110
1968	4105	660
1968	4342	164
1968	4800	129
1968	5105	115
1968	5688	171
1968	8351	162
1968	9716	215
1968	16950	109
1968	17925	86
1969	65	342
1969	217	967
1969	223	317
1969	248	394
1969	249	407
1969	267	1921

1969	331 308	
1969	475 590	
1969	527 306	
1969	547 501	
1969	617 346	
1969	1455	336
1969	1613	649
1969	1614	401
1969	1616	544
1969	1636	461
1969	1640	494
1969	1839	370
1969	1946	308
1969	1952	1042
1969	1959	371
1969	2242	711
1969	2906	788
1969	2909	548
1969	2913	1427
1969	2916	272
1969	2921	454
1969	2923	642
1969	3227	356
1969	3317	364
1969	3357	251
1969	3716	330
1969	6343	462
1969	6887	431
1969	6900	306
1969	6901	302

1969	8504	272
1969	10399	495
1969	10552	302
1969	10555	717
1969	11401	427
1969	11758	603
1969	12902	514
1969	14424	469
1969	17926	586
1969	17927	299
1969	17928	288
1969	17929	282
1969	17930	266
1969	17931	256
1971	426 4266	
1971	804 1785	
1971	918 2808	
1971	1528	1997
1971	1992	3084
1971	3028	3141
1971	3043	2297
1971	3442	2155
1971	3729	2665
1971	4107	2335
1971	4204	1967
1971	4504	1766
1971	4781	1902
1971	4934	10362
1971	5227	3393
1971	5632	3199

1971	5638	2868
1971	5879	2813
1971	8927	3185
1971	8932	1799
1971	8951	2848
1971	9452	3600
1971	9796	4015
1971	9801	1803
1971	10449	3217
1971	10759	2526
1971	11607	1814
1971	11859	4081
1971	13692	2091
1971	16272	1734
1971	17094	2088
1971	17935	7500
1971	17936	7342
1971	17937	3982
1971	17938	3719
1971	17939	3430
1971	17940	3372
1971	17941	3301
1971	17942	2715
1971	17943	2640
1971	17944	2408
1971	17945	2373
1971	17946	2371
1971	17947	2280
1971	17948	2144
1971	17949	2090

1971	17950	2040
1971	17951	1984
1971	17952	1889
1971	17953	1805
1972	152 135	
1972	198 79	
1972	203 148	
1972	220 87	
1972	235 96	
1972	418 100	
1972	603 163	
1972	747 170	
1972	748 66	
1972	782 77	
1972	864 109	
1972	964 285	
1972	977 90	
1972	1222	698
1972	1226	118
1972	1418	263
1972	1731	137
1972	1801	73
1972	1824	85
1972	1835	93
1972	2146	116
1972	2165	107
1972	2167	74
1972	2473	96
1972	2493	80
1972	2605	176

1972	2667	251
1972	2668	121
1972	2679	94
1972	2700	95
1972	2811	132
1972	3440	316
1972	3520	74
1972	3995	105
1972	4108	121
1972	5069	78
1972	5346	112
1972	6159	66
1972	7060	125
1972	7081	73
1972	9779	139
1972	12837	78
1972	12854	95
1972	12868	221
1972	16324	109
1972	17221	106
1972	17954	178
1972	17955	125
1972	17956	121
1972	17957	73
1973	55	2149
1973	65	919
1973	67	3332
1973	89	1491
1973	97	949
1973	157	3627

1973	161	1716
1973	166	2199
1973	180	2730
1973	257	2532
1973	265	2114
1973	285	1151
1973	288	1268
1973	289	3280
1973	291	1290
1973	292	2284
1973	294	1438
1973	295	1304
1973	299	1092
1973	300	1433
1973	301	927
1973	302	1747
1973	310	1053
1973	311	1038
1973	316	2238
1973	333	995
1973	378	903
1973	455	971
1973	465	1359
1973	475	1319
1973	518	1148
1973	534	1164
1973	641	1418
1973	680	963
1973	697	1335
1973	701	1798

1973	714	951
1973	903	1040
1973	930	2091
1973	1001	1148
1973	1081	2227
1973	1098	1736
1973	1104	1320
1973	1114	1139
1973	1300	1045
1973	1505	1362
1973	1556	889
1973	1601	1343
1973	1714	889
1973	3177	1056
1974	56	876
1974	67	252
1974	88	189
1974	157	198
1974	163	236
1974	173	224
1974	198	251
1974	227	676
1974	230	245
1974	233	201
1974	234	591
1974	375	227
1974	396	290
1974	412	183
1974	424	242
1974	537	262

1974	707 266	
1974	917 208	
1974	986 455	
1974	1044	248
1974	1121	184
1974	1130	188
1974	1154	492
1974	1206	253
1974	1246	271
1974	1249	247
1974	1349	352
1974	1406	197
1974	1491	212
1974	1501	199
1974	1929	311
1974	2044	261
1974	2086	487
1974	2101	249
1974	2458	225
1974	2459	208
1974	3063	329
1974	3193	317
1974	3324	238
1974	3462	298
1974	3494	292
1974	5605	200
1974	6282	194
1974	7530	412
1974	10929	184
1974	11084	223

1974	15119	273
1974	15325	223
1974	17958	245
1974	17959	217
1975	88	284
1975	154	335
1975	610	289
1975	748	360
1975	1073	317
1975	1167	257
1975	1222	581
1975	1708	329
1975	1747	669
1975	1943	319
1975	1946	335
1975	1953	734
1975	1961	434
1975	1967	401
1975	1969	456
1975	2616	346
1975	2671	362
1975	2673	441
1975	3520	578
1975	3541	374
1975	4726	371
1975	5883	450
1975	7493	355
1975	7695	659
1975	8510	572
1975	10002	429

1975	10516	701
1975	11128	301
1975	11520	754
1975	12195	471
1975	14760	609
1975	16742	325
1975	17960	809
1975	17961	670
1975	17962	560
1975	17963	461
1975	17964	422
1975	17965	421
1975	17966	419
1975	17967	414
1975	17968	395
1975	17969	388
1975	17970	384
1975	17971	375
1975	17972	358
1975	17973	322
1975	17974	302
1975	17975	297
1975	17976	266
1975	17977	256
1976	162 402	
1976	163 1453	
1976	196 1786	
1976	197 1124	
1976	214 417	
1976	447 1065	

1976	610 684	
1976	857 434	
1976	868 442	
1976	871 400	
1976	1109	1476
1976	1151	357
1976	1412	691
1976	1512	535
1976	2300	1363
1976	2307	381
1976	2312	706
1976	2314	424
1976	2330	521
1976	5844	712
1976	6397	504
1976	6945	431
1976	6947	764
1976	6951	384
1976	6952	487
1976	6960	350
1976	6961	940
1976	8230	580
1976	8239	403
1976	8425	883
1976	8429	533
1976	8439	479
1976	8454	351
1976	9045	1111
1976	9048	926
1976	9064	397

1976	13498	365
1976	13503	1012
1976	14646	497
1976	15562	625
1976	17067	452
1976	17978	559
1976	17979	519
1976	17980	437
1976	17981	420
1976	17982	400
1976	17983	394
1976	17984	369
1976	17985	359
1976	17986	356
1977	65	573
1977	81	241
1977	103	303
1977	113	283
1977	154	2002
1977	162	377
1977	190	264
1977	196	386
1977	207	253
1977	227	633
1977	237	224
1977	238	275
1977	418	2206
1977	423	295
1977	424	205
1977	436	291

1977	454 498	
1977	604 349	
1977	606 396	
1977	609 420	
1977	610 372	
1977	621 435	
1977	755 248	
1977	757 465	
1977	1098	890
1977	1109	860
1977	1121	233
1977	1123	206
1977	1130	292
1977	1390	346
1977	1418	532
1977	1424	297
1977	1708	480
1977	1735	325
1977	1736	494
1977	1766	245
1977	1770	582
1977	3057	306
1977	3902	374
1977	3905	421
1977	5549	238
1977	6756	287
1977	7505	386
1977	7895	266
1977	15499	262
1977	15722	296

1977	17987	415
1977	17988	356
1977	17989	307
1977	17990	193
1978	154 86	
1978	176 140	
1978	220 55	
1978	234 52	
1978	511 63	
1978	706 182	
1978	707 3266	
1978	724 702	
1978	732 448	
1978	841 85	
1978	843 49	
1978	854 69	
1978	917 677	
1978	918 3173	
1978	920 79	
1978	951 1914	
1978	952 466	
1978	959 57	
1978	1249	815
1978	1358	866
1978	1360	106
1978	1364	94
1978	1369	337
1978	1372	51
1978	1412	51
1978	1803	930

1978	1808	123
1978	1810	871
1978	2342	74
1978	2343	491
1978	2346	211
1978	2347	508
1978	2360	56
1978	2565	137
1978	2787	162
1978	2790	252
1978	2859	115
1978	2973	55
1978	3083	157
1978	3473	70
1978	3474	49
1978	4165	444
1978	4180	89
1978	4460	82
1978	5165	100
1978	6292	57
1978	9462	91
1978	9822	53
1978	17991	133
1978	17992	79
1979	55	1194
1979	58	68
1979	67	2835
1979	89	841
1979	97	528
1979	157	524

1979	192 357	
1979	257 273	
1979	258 70	
1979	288 491	
1979	289 850	
1979	292 589	
1979	295 161	
1979	298 166	
1979	300 590	
1979	301 123	
1979	306 58	
1979	310 113	
1979	320 73	
1979	333 179	
1979	340 1028	
1979	386 65	
1979	461 88	
1979	475 68	
1979	523 109	
1979	525 73	
1979	534 60	
1979	548 159	
1979	679 128	
1979	689 58	
1979	697 54	
1979	704 85	
1979	889 717	
1979	903 282	
1979	985 174	
1979	1081	146

1979	1098	84
1979	1379	86
1979	1463	167
1979	1464	191
1979	1580	132
1979	1934	1890
1979	2521	136
1979	3129	111
1979	3322	311
1979	3404	168
1979	5149	81
1979	6710	91
1979	7348	158
1979	17993	61
1980	63	20
1980	65	75
1980	190	98
1980	204	76
1980	217	55
1980	227	143
1980	231	40
1980	316	40
1980	344	20
1980	355	133
1980	366	133
1980	377	31
1980	498	21
1980	511	22
1980	554	46
1980	602	103

1980	614	47
1980	618	151
1980	683	263
1980	689	24
1980	706	25
1980	733	34
1980	892	60
1980	963	27
1980	982	19
1980	1390	45
1980	1511	34
1980	1609	27
1980	1743	113
1980	1772	40
1980	1778	89
1980	3771	20
1980	4618	87
1980	5432	94
1980	5754	26
1980	6301	29
1980	7194	35
1980	7826	36
1980	8649	45
1980	11739	49
1980	12481	20
1980	17994	83
1980	17995	65
1980	17996	54
1980	17997	49
1980	17998	33

1980	17999	26
1980	18000	23
1980	18001	23
1980	18002	22
1981	65	605
1981	95	88
1981	182	909
1981	190	229
1981	221	104
1981	477	94
1981	526	178
1981	530	978
1981	689	126
1981	1001	95
1981	1105	139
1981	1106	206
1981	1109	319
1981	1112	95
1981	1378	140
1981	1383	166
1981	1385	102
1981	2051	226
1981	2614	119
1981	2803	97
1981	3117	154
1981	3171	430
1981	3484	115
1981	3491	90
1981	3676	90
1981	3945	330

1981	4073	108
1981	4515	108
1981	4762	91
1981	6217	158
1981	7122	2749
1981	7187	191
1981	7194	150
1981	7667	244
1981	8589	685
1981	9485	253
1981	10327	123
1981	10548	139
1981	10961	529
1981	11253	214
1981	11277	143
1981	11910	390
1981	14359	195
1981	18003	275
1981	18004	187
1981	18005	142
1981	18006	130
1981	18007	96
1981	18008	94
1981	18009	94
1982	65	200
1982	70	236
1982	72	282
1982	81	206
1982	163	170
1982	172	162

1982 173 368

1982 182 201

1982 187 248

1982 196 419

1982 229 150

1982 233 178

1982 238 211

1982 355 247

1982 432 320

1982 492 176

1982 703 167

1982 722 220

1982 755 156

1982 809 181

1982 842 322

1982 845 221

1982 1075 149

1982 1504 239

1982 1561 405

1982 1700 240

1982 1705 165

1982 1743 205

1982 2473 185

1982 3028 819

1982 3043 193

1982 3501 247

1982 3548 158

1982 3886 759

1982 4113 299

1982 4316 221

1982	4504	679
1982	4940	148
1982	4997	190
1982	5604	261
1982	5927	152
1982	10500	528
1982	11049	200
1982	14560	238
1982	16595	236
1982	18010	377
1982	18011	366
1982	18012	229
1982	18013	201
1982	18014	178
1983	55	127
1983	67	77
1983	172	78
1983	257	298
1983	265	52
1983	285	198
1983	288	365
1983	289	356
1983	291	153
1983	295	381
1983	300	114
1983	302	101
1983	306	93
1983	310	95
1983	314	147
1983	320	48

1983	328 63	
1983	418 68	
1983	425 89	
1983	436 105	
1983	466 112	
1983	525 131	
1983	526 171	
1983	531 47	
1983	532 64	
1983	534 598	
1983	545 63	
1983	547 48	
1983	701 172496	
1983	787 184	
1983	1037	48
1983	1042	122
1983	1098	544
1983	1106	113
1983	1116	117
1983	1449	58
1983	1486	64
1983	1613	46
1983	1983	169
1983	1990	55
1983	3110	98
1983	4562	55
1983	5547	209
1983	5790	192
1983	5947	72
1983	6374	182

1983 7340 107

1983 7530 157

1983 8589 55

1983 18015 64

1984 8 4206

1984 188 707

1984 300 434

1984 377 1005

1984 396 3158

1984 498 721

1984 500 2355

1984 504 2790

1984 514 669

1984 537 751

1984 813 968

1984 816 1837

1984 818 2201

1984 830 618

1984 843 1136

1984 917 681

1984 918 749

1984 920 620

1984 924 437

1984 928 1103

1984 930 525

1984 932 665

1984 953 494

1984 957 1205

1984 978 2184

1984 1180 664

1984	1339	3825
1984	1350	1129
1984	1528	451
1984	1672	960
1984	1910	600
1984	1919	1266
1984	1923	2730
1984	1927	642
1984	1933	9942
1984	2014	550
1984	2062	1392
1984	2143	711
1984	2168	426
1984	2850	903
1984	2940	3510
1984	2959	1551
1984	4137	514
1984	4171	460
1984	4502	474
1984	5082	1489
1984	5103	581
1984	5202	1680
1984	5963	943
1984	7835	581
1986	152 209	
1986	163 971	
1986	176 157	
1986	227 362	
1986	234 180	
1986	380 168	

1986	498 507	
1986	607 761	
1986	706 1482	
1986	707 728	
1986	724 1377	
1986	841 2134	
1986	843 819	
1986	917 12951	
1986	918 2646	
1986	923 138	
1986	928 472	
1986	932 1224	
1986	942 892	
1986	946 622	
1986	953 222	
1986	954 430	
1986	957 236	
1986	1338	204
1986	1358	815
1986	1360	572
1986	1364	416
1986	1369	1158
1986	1412	1499
1986	1415	261
1986	1672	1221
1986	1854	168
1986	2341	140
1986	2342	380
1986	2343	171
1986	2347	310

1986	2664	183
1986	2746	1566
1986	2765	202
1986	2797	663
1986	2834	305
1986	2844	2520
1986	3093	159
1986	5451	650
1986	5772	197
1986	6282	257
1986	8042	137
1986	8351	210
1986	8386	283
1986	18022	578
1987	8	138
1987	65	417
1987	72	503
1987	159	1595
1987	163	396
1987	173	546
1987	190	236
1987	197	2211
1987	200	811
1987	201	145
1987	205	1389
1987	223	1545
1987	355	173
1987	366	205
1987	599	416
1987	707	721

1987	734	307
1987	851	1257
1987	861	176
1987	881	162
1987	964	204
1987	1376	391
1987	1413	284
1987	1790	447
1987	1981	267
1987	2226	168
1987	2343	150
1987	2369	594
1987	2463	138
1987	2595	188
1987	3264	3025
1987	3269	419
1987	3462	277
1987	3645	150
1987	3665	234
1987	3670	403
1987	3707	246
1987	3712	143
1987	4675	409
1987	5175	1081
1987	5306	266
1987	6823	251
1987	7140	137
1987	10026	149
1987	11756	151
1987	12495	418

1987 14439 336
1987 18023 591
1987 18024 272
1987 18025 147
1988 55 83
1988 89 474
1988 190 165
1988 238 52
1988 288 442
1988 289 1470
1988 292 317
1988 298 74
1988 300 323
1988 306 48
1988 328 63
1988 333 72
1988 355 106
1988 461 117
1988 466 625
1988 471 83
1988 544 80
1988 679 71
1988 686 130
1988 701 589
1988 787 46
1988 788 389
1988 790 2385
1988 792 70
1988 795 85
1988 802 289

1988	903 51	
1988	1034	55
1988	1400	91
1988	1428	104
1988	1431	64
1988	1432	76
1988	2596	235
1988	3187	120
1988	3193	182
1988	3404	97
1988	3905	58
1988	4254	74
1988	4703	67
1988	4817	108
1988	6529	148
1988	7395	54
1988	9157	311
1988	9158	62
1988	10967	103
1988	11221	91
1988	13603	57
1988	15696	77
1988	15900	67
1988	18026	107
1989	69 142	
1989	73 105	
1989	1800	174
1989	2044	87
1989	3863	102
1989	6250	103

1989	6755	163
1989	7015	203
1989	9353	134
1989	11642	90
1989	12890	397
1989	13563	79
1989	15667	126
1989	15671	89
1989	15675	278
1989	15676	184
1989	15679	1722
1989	18027	1286
1989	18028	932
1989	18029	648
1989	18030	497
1989	18031	421
1989	18032	357
1989	18033	340
1989	18034	335
1989	18035	282
1989	18036	254
1989	18037	192
1989	18038	184
1989	18039	173
1989	18040	146
1989	18041	140
1989	18042	130
1989	18043	124
1989	18044	122
1989	18045	121

1989	18046	119
1989	18047	117
1989	18048	116
1989	18049	115
1989	18050	109
1989	18051	109
1989	18052	106
1989	18053	104
1989	18054	102
1989	18055	95
1989	18056	94
1989	18057	90
1989	18058	85
1989	18059	78
1990	267 35	
1990	276 37	
1990	331 25	
1990	617 31	
1990	1099	21
1990	1605	20
1990	1610	19
1990	1618	22
1990	1621	24
1990	1627	34
1990	1636	21
1990	1943	19
1990	1948	26
1990	1952	19
1990	1959	39
1990	1963	47

1990	1967	46
1990	1979	35
1990	2226	18
1990	2252	28
1990	2605	24
1990	2698	23
1990	2716	22
1990	2915	31
1990	2923	28
1990	2930	18
1990	3227	30
1990	3357	35
1990	3708	24
1990	4585	26
1990	5048	27
1990	6046	21
1990	6629	18
1990	6887	18
1990	6901	22
1990	12902	24
1990	14226	31
1990	14451	23
1990	16501	23
1990	18060	43
1990	18061	35
1990	18062	31
1990	18063	31
1990	18064	30
1990	18065	28
1990	18066	27

1990	18067	24
1990	18068	22
1990	18069	20
1990	18070	20
1991	59	45
1991	72	302
1991	81	66
1991	233	143
1991	271	51
1991	436	306
1991	512	153
1991	603	100
1991	751	76
1991	769	73
1991	843	44
1991	854	67
1991	1083	67
1991	1098	126
1991	1210	70
1991	1216	51
1991	1620	63
1991	1627	142
1991	1640	89
1991	1709	123
1991	1952	211
1991	1963	54
1991	1994	100
1991	2252	100
1991	2542	165
1991	2817	89

1991	2828	37
1991	2921	184
1991	3133	533
1991	3227	126
1991	3357	47
1991	3411	164
1991	3532	91
1991	4074	49
1991	4585	55
1991	5867	53
1991	9672	160
1991	9843	178
1991	10370	152
1991	10555	81
1991	11128	53
1991	15919	61
1991	17189	187
1991	18071	95
1991	18072	85
1991	18073	80
1991	18074	54
1991	18075	47
1991	18076	43
1991	18077	39
1992	40	478
1992	53	540
1992	65	651
1992	154	903
1992	159	823
1992	195	2167

1992	196	1044
1992	199	656
1992	217	803
1992	223	568
1992	228	976
1992	429	2433
1992	433	1922
1992	434	668
1992	436	802
1992	612	1614
1992	615	2437
1992	622	3596
1992	857	1046
1992	859	679
1992	863	565
1992	961	606
1992	1097	939
1992	1239	1005
1992	1513	600
1992	1632	882
1992	1878	707
1992	1957	2471
1992	2271	776
1992	2398	785
1992	2405	550
1992	2531	1171
1992	2541	513
1992	2629	646
1992	3057	626
1992	3110	586

1992	3203	514
1992	3281	924
1992	3444	489
1992	3616	520
1992	3622	586
1992	3693	1377
1992	3744	525
1992	3781	570
1992	6417	570
1992	6687	871
1992	7151	536
1992	9485	1846
1992	18078	968
1992	18079	631
1993	56	10
1993	89	10
1993	423	58
1993	425	21
1993	446	19
1993	526	19
1993	632	11
1993	697	9
1993	757	16
1993	898	32
1993	914	45
1993	961	8
1993	962	9
1993	985	10
1993	1632	40
1993	1971	62

1993	2264	14
1993	2295	21
1993	2407	63
1993	2608	9
1993	2829	35
1993	2830	34
1993	3057	36
1993	3411	8
1993	3797	14
1993	4196	13
1993	4704	23
1993	8676	17
1993	8965	10
1993	9307	17
1993	9319	28
1993	12410	17
1993	12548	24
1993	14221	21
1993	15345	11
1993	15819	23
1993	17685	12
1993	17744	35
1993	17898	17
1993	18080	23
1993	18081	19
1993	18082	18
1993	18083	16
1993	18084	16
1993	18085	15
1993	18086	12

1993	18087	12
1993	18088	12
1993	18089	10
1993	18090	9
1994	59	47
1994	84	73
1994	89	80
1994	173	143
1994	190	51
1994	203	130
1994	207	120
1994	227	356
1994	229	94
1994	298	129
1994	425	134
1994	426	53
1994	429	115
1994	430	79
1994	432	93
1994	441	58
1994	599	402
1994	620	375
1994	757	105
1994	874	116
1994	887	66
1994	903	130
1994	920	83
1994	961	48
1994	972	167
1994	980	100

1994	1103	60
1994	1239	114
1994	1400	62
1994	1512	46
1994	1632	155
1994	1669	156
1994	1755	76
1994	1804	74
1994	3186	97
1994	3200	100
1994	3293	111
1994	4620	63
1994	4704	168
1994	4810	138
1994	5027	57
1994	5412	76
1994	5926	85
1994	6203	51
1994	6245	58
1994	7071	78
1994	10943	74
1994	13716	139
1994	14845	62
1994	18091	48
1995	71	667
1995	217	1464
1995	220	848
1995	221	539
1995	229	421
1995	333	405

1995	355 2306	
1995	366 627	
1995	377 434	
1995	418 5099	
1995	460 398	
1995	486 475	
1995	498 634	
1995	499 1420	
1995	501 4428	
1995	512 1933	
1995	808 343	
1995	898 750	
1995	961 1269	
1995	1044	414
1995	1105	391
1995	1116	1373
1995	1377	1479
1995	1731	5199
1995	1866	410
1995	2608	1798
1995	2676	5227
1995	2691	7883
1995	2813	792
1995	2850	2166
1995	3164	1016
1995	3670	582
1995	4225	3288
1995	4228	510
1995	4237	420
1995	4994	4842

1995	5698	2996
1995	6130	397
1995	6439	573
1995	7724	636
1995	7887	376
1995	10194	770
1995	11393	375
1995	15370	4322
1995	18092	1669
1995	18093	1340
1995	18094	622
1995	18095	594
1995	18096	504
1995	18097	357
1996	56	213
1996	238	236
1996	330	339
1996	331	162
1996	378	342
1996	475	139
1996	527	634
1996	569	266
1996	572	208
1996	718	449
1996	1020	248
1996	1401	827
1996	1402	165
1996	1433	190
1996	1448	295
1996	1613	449

1996	1620	340
1996	1934	153
1996	2222	466
1996	2232	336
1996	2667	144
1996	2923	293
1996	5605	400
1996	6626	142
1996	6903	165
1996	8160	175
1996	9910	233
1996	12854	147
1996	13292	483
1996	13303	379
1996	15101	591
1996	15113	1104
1996	18098	455
1996	18099	445
1996	18100	437
1996	18101	320
1996	18102	280
1996	18103	265
1996	18104	235
1996	18105	229
1996	18106	228
1996	18107	219
1996	18108	171
1996	18109	160
1996	18110	158
1996	18111	153

1996	18112	151
1996	18113	144
1996	18114	143
1996	18115	142
1997	65	120
1997	96	562
1997	163	720
1997	212	7692
1997	220	157
1997	222	312
1997	223	193
1997	225	367
1997	227	16116
1997	298	155
1997	412	379
1997	416	467
1997	526	139
1997	554	216
1997	562	701
1997	599	315
1997	602	197
1997	709	284
1997	718	334
1997	733	1253
1997	735	557
1997	859	152
1997	868	426
1997	1160	181
1997	1242	654
1997	1390	186

1997	1400	130
1997	1412	990
1997	1414	129
1997	1416	348
1997	1422	137
1997	1469	206
1997	1639	313
1997	2265	2028
1997	2444	197
1997	2855	884
1997	3458	288
1997	3754	2063
1997	3938	628
1997	4118	139
1997	4133	156
1997	4369	212
1997	4620	382
1997	8593	864
1997	9433	393
1997	11623	189
1997	12753	419
1997	13000	221
1997	18116	135
1997	18117	122
1998	65	755
1998	154	158
1998	166	321
1998	173	582
1998	190	380
1998	195	882

1998	207 483	
1998	217 1578	
1998	220 104	
1998	227 1068	
1998	229 101	
1998	230 237	
1998	234 124	
1998	289 140	
1998	318 78	
1998	320 110	
1998	333 321	
1998	412 116	
1998	424 759	
1998	425 76	
1998	429 296	
1998	439 239	
1998	486 228	
1998	511 6718	
1998	533 1235	
1998	537 2243	
1998	679 101	
1998	680 316	
1998	705 110	
1998	706 114	
1998	951 76	
1998	1105	111
1998	1186	97
1998	1241	110
1998	1389	106
1998	1672	352

1998	2840	91
1998	3200	255
1998	3332	126
1998	3373	371
1998	3616	2599
1998	3763	295
1998	4262	1311
1998	4832	134
1998	6228	751
1998	6293	723
1998	6453	344
1998	6602	95
1998	8878	192
1998	14239	86
1999	7	410
1999	8	545
1999	72	967
1999	157	728
1999	159	390
1999	173	1024
1999	176	596
1999	196	823
1999	198	516
1999	227	1415
1999	424	799
1999	429	557
1999	439	1734
1999	487	1663
1999	504	1012
1999	534	1391

1999	629 434	
1999	726 636	
1999	757 1075	
1999	955 392	
1999	1044	1553
1999	1106	511
1999	1116	700
1999	1118	371
1999	1145	484
1999	1180	1677
1999	1218	473
1999	1678	724
1999	1781	1018
1999	1796	680
1999	1910	930
1999	1985	427
1999	2014	642
1999	2060	510
1999	2379	445
1999	2428	642
1999	3197	363
1999	3236	4847
1999	3767	3213
1999	3901	735
1999	4143	527
1999	5079	1188
1999	5605	357
1999	8223	434
1999	8726	546
1999	9230	603

1999	10931	369
1999	18118	488
1999	18119	435
1999	18120	366
2000	67	12782
2000	161	3054
2000	288	24774
2000	289	4189
2000	294	3062
2000	328	8441
2000	329	13128
2000	330	7752
2000	331	25901
2000	468	4309
2000	475	47147
2000	527	33305
2000	545	18728
2000	644	6661
2000	701	8359
2000	907	10846
2000	1444	50559
2000	1449	5068
2000	2018	2662
2000	2031	1945
2000	2179	16027
2000	2226	3475
2000	2236	2680
2000	2247	2911
2000	2500	3803
2000	2919	25763

2000	2927	2032
2000	3713	1713
2000	5988	10866
2000	6621	2028
2000	6626	8331
2000	7340	2678
2000	8364	2631
2000	9902	3819
2000	10429	3899
2000	11139	8769
2000	18121	23462
2000	18122	10726
2000	18123	8131
2000	18124	6962
2000	18125	4415
2000	18126	3771
2000	18127	3307
2000	18128	2597
2000	18129	2081
2000	18130	1993
2000	18131	1938
2000	18132	1841
2000	18133	1558
2000	18134	1530
2001	166 486	
2001	198 421	
2001	488 443	
2001	494 351	
2001	497 390	
2001	517 1965	

2001	518 396	
2001	533 445	
2001	614 1034	
2001	706 470	
2001	707 4985	
2001	710 631	
2001	724 1102	
2001	779 484	
2001	785 1561	
2001	816 510	
2001	826 435	
2001	841 396	
2001	843 28851	
2001	854 900	
2001	922 426	
2001	923 435	
2001	930 412	
2001	937 684	
2001	949 604	
2001	954 527	
2001	978 605	
2001	1044	714
2001	1104	804
2001	1119	441
2001	1145	748
2001	1249	382
2001	1274	536
2001	1342	396
2001	1527	940
2001	1545	1415

2001	1672	1991
2001	1817	493
2001	2143	497
2001	2379	405
2001	2380	949
2001	3240	1916
2001	3637	483
2001	3722	337
2001	4284	389
2001	6120	414
2001	8259	759
2001	18135	804
2001	18136	629
2001	18137	385
2003	64	260
2003	72	102
2003	84	171
2003	233	137
2003	697	280
2003	767	273
2003	867	493
2003	1062	129
2003	1075	91
2003	1121	148
2003	1222	91
2003	1292	93
2003	1332	157
2003	1376	111
2003	1700	107
2003	1745	328

2003	1800	91
2003	2030	130
2003	2605	114
2003	2667	2262
2003	2691	323
2003	3411	94
2003	3536	148
2003	3551	860
2003	3617	151
2003	4735	312
2003	5197	156
2003	5475	98
2003	5709	119
2003	6582	107
2003	6667	123
2003	6676	179
2003	7079	162
2003	8291	206
2003	11652	127
2003	12330	105
2003	12964	100
2003	13651	878
2003	15037	331
2003	17783	190
2003	18145	1006
2003	18146	496
2003	18147	166
2003	18148	161
2003	18149	153
2003	18150	138

2003	18151	134
2003	18152	118
2003	18153	103
2003	18154	90
2004	7	219
2004	39	178
2004	89	532
2004	154	772
2004	172	282
2004	173	131
2004	190	48016
2004	198	202
2004	289	5192
2004	291	148
2004	292	634
2004	295	168
2004	300	111
2004	316	96
2004	318	293
2004	324	85
2004	377	171
2004	487	93
2004	497	153
2004	517	335
2004	687	134
2004	701	165
2004	758	101
2004	1090	562
2004	1104	117
2004	1179	87

2004	1470	97
2004	1505	90
2004	1786	210
2004	1788	174
2004	1901	84
2004	2206	89
2004	3049	87
2004	3827	183
2004	4226	568
2004	4255	128
2004	5079	106
2004	6033	218
2004	6245	275
2004	6247	146
2004	6439	91
2004	6453	137
2004	6542	114
2004	10430	124
2004	12130	129
2004	12291	155
2004	14930	127
2004	18155	336
2004	18156	281
2004	18157	121
2005	81	835
2005	197	302
2005	603	310
2005	617	287
2005	1098	963
2005	1411	852

2005	1708	782
2005	2473	511
2005	2811	282
2005	3505	1144
2005	3529	999
2005	6043	642
2005	6161	1351
2005	6345	733
2005	9783	322
2005	10516	532
2005	11211	430
2005	11927	618
2005	13318	628
2005	16123	512
2005	16127	1535
2005	18158	1825
2005	18159	1796
2005	18160	1230
2005	18161	1029
2005	18162	944
2005	18163	768
2005	18164	750
2005	18165	741
2005	18166	731
2005	18167	585
2005	18168	518
2005	18169	442
2005	18170	402
2005	18171	396
2005	18172	395

2005	18173	372
2005	18174	371
2005	18175	364
2005	18176	360
2005	18177	356
2005	18178	341
2005	18179	337
2005	18180	329
2005	18181	327
2005	18182	315
2005	18183	312
2005	18184	282
2005	18185	281
2005	18186	263
2006	64	233
2006	603	1977
2006	744	303
2006	748	5679
2006	756	781
2006	772	286
2006	778	731
2006	1745	267
2006	1801	365
2006	1833	280
2006	1835	560
2006	1945	274
2006	1965	2303
2006	2146	386
2006	2158	647
2006	2605	2667

2006	2668	372
2006	2669	186
2006	2671	468
2006	2695	410
2006	2831	1056
2006	3541	316
2006	3993	223
2006	4865	594
2006	5028	1479
2006	7685	286
2006	9672	277
2006	10517	190
2006	10519	189
2006	10522	3229
2006	11208	844
2006	17962	368
2006	18187	647
2006	18188	615
2006	18189	562
2006	18190	558
2006	18191	557
2006	18192	524
2006	18193	493
2006	18194	436
2006	18195	407
2006	18196	371
2006	18197	371
2006	18198	341
2006	18199	328
2006	18200	287

2006 18201 266

2006 18202 256

2006 18203 227

2006 18204 208

2007 154 1744

2007 227 2786

2007 234 4615

2007 235 1921

2007 383 2241

2007 412 1472

2007 475 3488

2007 605 1625

2007 614 4057

2007 707 2999

2007 710 1608

2007 841 1553

2007 854 2265

2007 856 1610

2007 918 2265

2007 1119 1511

2007 1123 2144

2007 1202 2063

2007 1204 2365

2007 1239 1739

2007 1369 2741

2007 1401 3290

2007 1410 1992

2007 1418 2552

2007 1527 2208

2007 1533 2373

2007	2473	3887
2007	2491	4392
2007	3445	1539
2007	3780	2294
2007	4781	14053
2007	4821	3932
2007	7331	1897
2007	7419	1963
2007	9122	2462
2007	9125	1708
2007	9128	2640
2007	9208	2473
2007	9225	1992
2007	9452	2493
2007	9716	8620
2007	11936	6383
2007	11986	2414
2007	13622	1695
2007	13846	3210
2007	14200	3221
2007	14692	1524
2007	18205	7494
2007	18206	5279
2007	18207	1413
2008	7	709
2008	56	267
2008	65	412
2008	198	303
2008	203	289
2008	207	14909

2008	225 614	
2008	226 400	
2008	227 2728	
2008	229 1505	
2008	230 1623	
2008	234 2310	
2008	300 793	
2008	377 324	
2008	378 412	
2008	424 1586	
2008	439 613	
2008	498 392	
2008	704 695	
2008	706 2314	
2008	709 1360	
2008	735 318	
2008	857 491	
2008	903 373	
2008	917 1570	
2008	918 2210	
2008	924 290	
2008	953 450	
2008	982 453	
2008	1044	715
2008	1090	1065
2008	1106	363
2008	1369	274
2008	1376	342
2008	1400	1039
2008	1414	391

2008	1502	467
2008	1512	7056
2008	1513	629
2008	1515	926
2008	1672	779
2008	1976	1025
2008	2123	461
2008	2134	353
2008	2343	421
2008	2788	683
2008	2821	744
2008	3088	691
2008	4941	552
2008	5667	796
2009	154 160	
2009	163 158	
2009	167 181	
2009	182 233	
2009	190 147	
2009	196 166	
2009	199 155	
2009	207 511	
2009	215 133	
2009	227 317	
2009	228 192	
2009	234 128	
2009	418 172	
2009	424 171	
2009	426 156	
2009	429 119	

2009	430 234	
2009	437 162	
2009	440 241	
2009	599 326	
2009	622 138	
2009	629 592	
2009	689 151	
2009	690 673	
2009	857 160	
2009	896 140	
2009	903 255	
2009	1048	339
2009	1090	321
2009	1097	158
2009	1243	11720
2009	1390	200
2009	1400	1422
2009	1409	125
2009	1424	125
2009	1609	225
2009	1633	150
2009	1934	160
2009	2083	198
2009	2277	218
2009	2524	148
2009	2601	215
2009	2614	130
2009	2632	220
2009	3057	176
2009	3201	203

2009	3693	179
2009	4704	152
2009	4810	132
2009	9285	212
2010	7	884
2010	65	669
2010	88	607
2010	154	1534
2010	173	707
2010	186	858
2010	190	1205
2010	191	613
2010	199	1212
2010	206	916
2010	207	2230
2010	225	817
2010	226	1523
2010	227	1518
2010	228	1029
2010	229	724
2010	236	682
2010	377	815
2010	421	858
2010	424	1344
2010	425	599
2010	434	685
2010	439	1520
2010	533	786
2010	540	1041
2010	614	614

2010	709	649
2010	969	896
2010	1155	1030
2010	1186	618
2010	1246	649
2010	1375	611
2010	1406	650
2010	1470	634
2010	1537	975
2010	1714	717
2010	1768	618
2010	1988	605
2010	2131	633
2010	2600	1149
2010	2652	825
2010	2815	635
2010	3378	709
2010	3383	672
2010	4793	801
2010	5226	808
2010	5423	619
2010	6570	797
2010	8710	1096
2010	18208	624
2011	63	54
2011	204	27
2011	207	26
2011	210	24
2011	217	117
2011	220	227

2011	228	112
2011	229	27
2011	234	30
2011	355	80
2011	366	462
2011	433	29
2011	544	31
2011	546	31
2011	606	74
2011	683	451
2011	715	58
2011	750	61
2011	891	216
2011	1081	26
2011	1383	249
2011	1511	48
2011	1519	130
2011	1556	71
2011	1730	226
2011	1772	39
2011	1824	96
2011	1873	47
2011	1954	71
2011	2176	93
2011	2544	165
2011	2632	75
2011	2783	43
2011	3057	24
2011	3104	35
2011	3114	485

2011	3881	31
2011	4402	33
2011	4553	215
2011	4616	655
2011	6297	45
2011	6351	808
2011	18209	138
2011	18210	129
2011	18211	64
2011	18212	59
2011	18213	51
2011	18214	40
2011	18215	29
2011	18216	24
2013	65	1603
2013	154	539
2013	173	329
2013	182	1285
2013	190	7213
2013	203	642
2013	207	5735
2013	208	203
2013	217	276
2013	220	269
2013	221	2832
2013	227	1004
2013	229	1357
2013	238	381
2013	298	698
2013	302	163

2013	424 659	
2013	432 313	
2013	486 167	
2013	533 499	
2013	604 216	
2013	632 219	
2013	962 224	
2013	969 727	
2013	1048	857
2013	1052	163
2013	1090	930
2013	1098	254
2013	1109	384
2013	1414	202
2013	1512	1643
2013	1515	505
2013	1517	331
2013	1747	211
2013	1772	507
2013	1786	656
2013	1795	280
2013	2133	195
2013	2632	170
2013	3046	1824
2013	3057	543
2013	3870	687
2013	4444	664
2013	4998	269
2013	5030	229
2013	7888	391

2013	8662	255
2013	9977	229
2013	14459	192
2013	18223	266
2014	51	265
2014	59	183
2014	61	351
2014	67	251
2014	72	421
2014	96	245
2014	100	179
2014	157	181
2014	159	970
2014	187	209
2014	193	163
2014	429	285
2014	436	241
2014	441	302
2014	511	256
2014	599	615
2014	601	189
2014	712	191
2014	852	281
2014	874	188
2014	875	368
2014	877	154
2014	992	206
2014	993	313
2014	997	230
2014	998	277

2014	1001	193
2014	1004	322
2014	1013	408
2014	1083	366
2014	1249	157
2014	1464	208
2014	1844	219
2014	1881	237
2014	1886	259
2014	2029	211
2014	2531	152
2014	2861	160
2014	2936	211
2014	2938	192
2014	3201	258
2014	3264	215
2014	3280	157
2014	3464	169
2014	4123	207
2014	5929	313
2014	7054	251
2014	18224	175
2014	18225	161
2014	18226	157
2015	40	564
2015	152	1004
2015	163	4203
2015	174	1507
2015	188	5260
2015	198	2656

2015	206	1560
2015	212	2656
2015	503	1651
2015	707	1130
2015	731	519
2015	806	719
2015	808	1281
2015	821	2610
2015	824	2310
2015	846	592
2015	917	607
2015	930	1774
2015	942	528
2015	943	2939
2015	978	1093
2015	1118	825
2015	1226	793
2015	1267	527
2015	1398	574
2015	1410	437
2015	1412	1113
2015	1994	2183
2015	2155	937
2015	2172	501
2015	2381	680
2015	2418	690
2015	2763	480
2015	2794	489
2015	2836	490
2015	2962	1065

2015	2966	2086
2015	2969	699
2015	3415	779
2015	3707	870
2015	3712	654
2015	3782	847
2015	4608	2518
2015	6098	4022
2015	7260	666
2015	7922	559
2015	8782	1533
2015	10585	1843
2015	18227	1148
2015	18228	482
2016	58	43
2016	81	41
2016	154	44
2016	238	63
2016	1098	139
2016	1781	37
2016	1824	64
2016	2419	60
2016	2605	27
2016	2711	49
2016	3902	28
2016	4113	34
2016	4316	45
2016	4401	38
2016	4404	34
2016	6930	409

2016	6949	37
2016	7492	34
2016	7810	33
2016	12834	67
2016	15058	35
2016	15671	77
2016	16324	31
2016	18229	570
2016	18230	213
2016	18231	82
2016	18232	82
2016	18233	56
2016	18234	51
2016	18235	42
2016	18236	41
2016	18237	40
2016	18238	40
2016	18239	38
2016	18240	38
2016	18241	37
2016	18242	36
2016	18243	36
2016	18244	36
2016	18245	35
2016	18246	35
2016	18247	35
2016	18248	34
2016	18249	32
2016	18250	30
2016	18251	29

2016	18252	28
2016	18253	27
2016	18254	27
2016	18255	27
2017	56	1606
2017	72	57
2017	84	554
2017	174	91
2017	236	80
2017	298	37
2017	424	163
2017	432	576
2017	498	51
2017	539	45
2017	546	115
2017	757	312
2017	887	55
2017	903	43
2017	969	147
2017	1098	119
2017	1191	122
2017	1285	40
2017	1394	439
2017	1396	330
2017	1398	362
2017	1400	120
2017	1405	105
2017	1408	637
2017	1409	38
2017	2083	184

2017	3200	2690
2017	3738	226
2017	4022	84
2017	4025	71
2017	4029	268
2017	4435	80
2017	4595	74
2017	4600	46
2017	4601	121
2017	4728	109
2017	5492	264
2017	5934	947
2017	6374	163
2017	7324	216
2017	7884	119
2017	9766	62
2017	16486	37
2017	18256	159
2017	18257	103
2017	18258	98
2017	18259	85
2017	18260	78
2017	18261	61
2017	18262	41
2018	55	330
2018	67	68
2018	97	250
2018	288	177
2018	289	1690
2018	291	383

2018 292 2636

2018 293 448

2018 294 190

2018 296 2239

2018 299 90

2018 300 69

2018 302 81

2018 304 547

2018 311 419

2018 314 79

2018 318 352

2018 323 1005

2018 325 99

2018 326 163

2018 333 1506

2018 344 142

2018 352 322

2018 386 343

2018 456 248

2018 461 161

2018 465 86

2018 466 283

2018 523 84

2018 528 333

2018 543 83

2018 548 130

2018 674 100

2018 679 175

2018 681 637

2018 786 143

2018	797	429
2018	800	320
2018	889	105
2018	891	349
2018	1047	153
2018	1451	301
2018	1453	130
2018	1458	534
2018	1934	95
2018	3081	130
2018	3319	131
2018	4291	105
2018	5533	162
2018	18263	165
2019	65	1155
2019	228	772
2019	333	1055
2019	498	2235
2019	499	1017
2019	542	1009
2019	683	6308
2019	689	2727
2019	899	1950
2019	991	1753
2019	1243	702
2019	1375	730
2019	1383	3543
2019	1470	1221
2019	1518	666
2019	1553	954

2019	1725	2232
2019	1768	1726
2019	1776	1297
2019	1852	1047
2019	1859	1007
2019	3078	2688
2019	3127	1085
2019	3171	634
2019	4177	3891
2019	4380	1666
2019	4487	1048
2019	4750	1939
2019	5425	1582
2019	7252	628
2019	8047	4382
2019	8244	1402
2019	8264	2789
2019	8592	1166
2019	8724	700
2019	8729	1011
2019	9187	3597
2019	9195	1423
2019	9724	754
2019	11217	2588
2019	12680	761
2019	12957	648
2019	14185	16702
2019	16709	806
2019	18264	1689
2019	18265	1297

2019	18266	1163
2019	18267	986
2019	18268	893
2019	18269	594
2020	7	107
2020	89	547
2020	157	141
2020	190	561
2020	198	118
2020	207	485
2020	208	123
2020	228	304
2020	234	363
2020	288	472
2020	295	657
2020	300	425
2020	331	139
2020	333	118
2020	420	149
2020	423	127
2020	425	351
2020	440	100
2020	441	137
2020	486	218
2020	498	822
2020	526	116
2020	539	402
2020	547	181
2020	548	160
2020	599	256

2020	689	573
2020	703	129
2020	704	240
2020	709	166
2020	779	110
2020	969	128
2020	982	105
2020	1098	184
2020	1390	109
2020	1400	212
2020	1512	95
2020	1609	124
2020	1807	646
2020	1814	130
2020	1978	169
2020	1990	181
2020	2083	155
2020	2128	105
2020	2351	94
2020	2788	118
2020	2821	337
2020	5149	99
2020	7660	228
2020	12013	130
2021	55	1269
2021	89	2842
2021	257	770
2021	288	2937
2021	289	2543
2021	290	610

2021	291 751
2021	292 1056
2021	294 563
2021	295 1020
2021	296 919
2021	299 1096
2021	300 2964
2021	302 546
2021	306 714
2021	310 635
2021	323 1136
2021	325 571
2021	326 600
2021	328 1007
2021	333 1237
2021	344 1483
2021	352 807
2021	386 537
2021	458 726
2021	461 1411
2021	464 597
2021	466 2448
2021	523 786
2021	528 638
2021	548 1057
2021	644 888
2021	679 2314
2021	680 640
2021	681 629
2021	686 1336

2021	691	755
2021	701	1589
2021	790	570
2021	907	609
2021	915	626
2021	1032	871
2021	1450	539
2021	1451	648
2021	1459	785
2021	2020	540
2021	3319	736
2021	3404	755
2021	7781	765
2021	18270	717
2022	89	748
2022	191	1901
2022	220	275
2022	289	257
2022	291	161
2022	295	206
2022	300	469
2022	304	1406
2022	333	3437
2022	377	2254
2022	461	207
2022	486	3763
2022	488	297
2022	498	225
2022	518	376
2022	700	164

2022	707 550	
2022	726 209	
2022	966 491	
2022	972 291	
2022	1377	239
2022	1447	162
2022	1470	17283
2022	1672	358
2022	1854	6440
2022	1872	187
2022	1905	147
2022	2080	797
2022	2352	247
2022	2794	517
2022	2840	156
2022	2940	223
2022	2941	1670
2022	2983	1135
2022	2987	132
2022	3427	1343
2022	3859	4279
2022	3927	133
2022	3928	461
2022	4439	166
2022	4473	138
2022	5217	339
2022	6006	3224
2022	6480	397
2022	6796	412
2022	8304	262

2022	9228	240
2022	11718	637
2022	18271	246
2022	18272	178
2023	65	128
2023	89	167
2023	173	326
2023	288	21
2023	289	1227
2023	292	1121
2023	300	1055
2023	306	64
2023	318	1259
2023	321	253
2023	324	65
2023	333	902
2023	340	25
2023	344	481
2023	430	27
2023	461	44
2023	464	292
2023	498	33
2023	523	432
2023	543	30
2023	679	2557
2023	680	30
2023	681	216
2023	686	140
2023	689	80
2023	704	5296

2023	797	107
2023	1034	27
2023	1042	23
2023	1047	560
2023	1428	31
2023	1452	31
2023	1456	248
2023	1580	30
2023	1609	29
2023	1679	63
2023	1690	171
2023	2020	1707
2023	5836	17
2023	5975	18
2023	6598	110
2023	9249	30
2023	14599	50
2023	18273	145
2023	18274	39
2023	18275	28
2023	18276	25
2023	18277	22
2023	18278	21
2023	18279	18
2024	65	4
2024	72	77
2024	154	25
2024	173	4
2024	182	3
2024	190	13

2024	199 9	
2024	206 4	
2024	210 7	
2024	221 6	
2024	225 12	
2024	228 8	
2024	229 10	
2024	233 12	
2024	237 4	
2024	238 10	
2024	418 12	
2024	425 8	
2024	436 3	
2024	443 4	
2024	450 4	
2024	511 14	
2024	716 4	
2024	969 4	
2024	1001	3
2024	1090	3
2024	1151	45
2024	1375	3
2024	2591	3
2024	2600	3
2024	3259	4
2024	4074	5
2024	4116	42
2024	4435	5
2024	5215	11
2024	5615	13

2024	5890	3
2024	5931	4
2024	6202	4
2024	6227	4
2024	7663	4
2024	8659	3
2024	9132	10
2024	9502	3
2024	10110	4
2024	10535	4
2024	15162	4
2024	15443	3
2024	18280	14
2024	18281	4
2025	51	647
2025	59	1053
2025	67	1741
2025	72	4477
2025	157	734
2025	159	846
2025	163	1555
2025	233	531
2025	436	909
2025	511	1684
2025	517	1253
2025	707	2110
2025	864	720
2025	889	630
2025	917	1062
2025	1000	1775

2025	1001	2249
2025	1014	691
2025	1019	1028
2025	1073	935
2025	1131	2277
2025	1330	696
2025	2183	527
2025	2347	866
2025	3289	931
2025	3675	537
2025	4590	537
2025	13176	580
2025	14343	783
2025	18282	1781
2025	18283	1485
2025	18284	1397
2025	18285	1386
2025	18286	1131
2025	18287	1100
2025	18288	978
2025	18289	926
2025	18290	878
2025	18291	848
2025	18292	800
2025	18293	758
2025	18294	746
2025	18295	746
2025	18296	728
2025	18297	702
2025	18298	690

2025	18299	611
2025	18300	602
2025	18301	552
2025	18302	545
2027	55	226
2027	161	153
2027	228	160
2027	289	781
2027	292	180
2027	293	226
2027	300	340
2027	306	160
2027	311	514
2027	318	469
2027	324	148
2027	333	161
2027	461	295
2027	464	144
2027	466	575
2027	471	163
2027	495	403
2027	498	258
2027	528	865
2027	548	345
2027	680	149
2027	691	319
2027	1034	196
2027	1038	639
2027	1043	155
2027	1047	376

2027	1470	356
2027	2137	162
2027	2977	139
2027	3927	143
2027	4177	252
2027	4264	516
2027	4746	508
2027	4773	296
2027	5032	187
2027	5472	210
2027	5684	139
2027	6600	257
2027	7723	286
2027	16568	165
2027	16709	249
2027	18305	961
2027	18306	390
2027	18307	285
2027	18308	230
2027	18309	223
2027	18310	213
2027	18311	181
2027	18312	168
2027	18313	154
2028	58	207
2028	72	181
2028	154	107
2028	238	182
2028	444	98
2028	603	641

2028	744 1180	
2028	756 399	
2028	757 162	
2028	772 224	
2028	1090	107
2028	1098	316
2028	1099	368
2028	1222	193
2028	1398	200
2028	1745	557
2028	1787	153
2028	1965	111
2028	1983	279
2028	2146	102
2028	2668	209
2028	2670	179
2028	2672	745
2028	2673	258
2028	2677	157
2028	2681	98
2028	2691	106
2028	3164	164
2028	3707	115
2028	4316	242
2028	4734	114
2028	4860	129
2028	4863	129
2028	4865	183
2028	5154	143
2028	6277	176

2028	8214	130
2028	8702	116
2028	10612	174
2028	10659	106
2028	11316	137
2028	12495	154
2028	16751	99
2028	17903	119
2028	18314	172
2028	18315	120
2028	18316	116
2028	18317	111
2028	18318	102
2028	18319	98
2029	36	384
2029	56	281
2029	72	458
2029	152	196
2029	172	153
2029	183	592
2029	226	1111
2029	233	1909
2029	306	242
2029	420	167
2029	422	306
2029	425	138
2029	432	268
2029	439	435
2029	516	170
2029	526	238

2029	538 552	
2029	615 231	
2029	777 954	
2029	806 255	
2029	862 155	
2029	887 132	
2029	982 148	
2029	1044	431
2029	1062	327
2029	1106	1458
2029	1131	190
2029	1398	150
2029	1738	389
2029	1812	169
2029	2121	200
2029	2137	378
2029	2364	411
2029	2535	353
2029	2601	133
2029	2708	240
2029	3186	351
2029	4725	302
2029	4732	314
2029	5441	232
2029	8582	259
2029	8915	199
2029	9498	235
2029	10220	181
2029	18320	315
2029	18321	234

2029	18322	198
2029	18323	195
2029	18324	185
2029	18325	151
2030	64	200
2030	65	130
2030	71	157
2030	73	132
2030	154	246
2030	162	281
2030	223	560
2030	238	272
2030	278	212
2030	418	219
2030	444	103
2030	475	125
2030	486	120
2030	632	294
2030	697	272
2030	1109	245
2030	1424	95
2030	1736	206
2030	1747	178
2030	1790	250
2030	1795	106
2030	2298	219
2030	2312	351
2030	2331	207
2030	2605	180
2030	3132	152

2030	4232	106
2030	4931	167
2030	6756	103
2030	6758	129
2030	7456	85
2030	10613	116
2030	11020	97
2030	11263	98
2030	11739	128
2030	12452	200
2030	12718	95
2030	13498	501
2030	13503	94
2030	14245	101
2030	15457	94
2030	17981	292
2030	18326	462
2030	18327	224
2030	18328	144
2030	18329	102
2030	18330	99
2030	18331	97
2030	18332	89
2030	18333	86
2031	7	3835
2031	55	7204
2031	67	4127
2031	71	1475
2031	89	12541
2031	173	3208

2031	195	1387
2031	288	8767
2031	289	128654
2031	292	5779
2031	293	2563
2031	295	2141
2031	296	1720
2031	300	4660
2031	310	1488
2031	312	4239
2031	314	2339
2031	318	10598
2031	323	1537
2031	328	1414
2031	333	1846
2031	378	7201
2031	466	11237
2031	512	1573
2031	523	7968
2031	525	2097
2031	704	10759
2031	911	7162
2031	972	2638
2031	1075	4937
2031	1098	5150
2031	1503	2182
2031	2025	2571
2031	2083	1736
2031	2094	4284
2031	2521	4913

2031	2546	1474
2031	3184	4776
2031	3375	2790
2031	3397	1693
2031	3404	1653
2031	4255	1836
2031	4814	2006
2031	4926	1673
2031	5149	2927
2031	6374	1487
2031	6428	6919
2031	6835	1672
2031	11030	9180
2031	18334	1964
2032	53	1399
2032	55	1245
2032	56	1549
2032	58	523
2032	65	1147
2032	67	1659
2032	81	972
2032	88	1406
2032	154	2146
2032	196	1313
2032	199	942
2032	206	1178
2032	227	564
2032	233	929
2032	331	1672
2032	418	5694

2032	424 628	
2032	425 557	
2032	433 1572	
2032	436 1034	
2032	440 1720	
2032	620 807	
2032	622 626	
2032	632 632	
2032	703 723	
2032	757 1603	
2032	858 1066	
2032	898 740	
2032	962 951	
2032	1098	2627
2032	1106	573
2032	1167	621
2032	1390	976
2032	1609	1114
2032	1632	753
2032	2083	2724
2032	2134	685
2032	2139	2550
2032	2277	1518
2032	2398	1615
2032	2521	2123
2032	2632	2723
2032	3110	1533
2032	3259	890
2032	3693	541
2032	5048	822

2032	5147	502
2032	8676	1104
2032	9951	854
2032	18335	2577
2033	55	345
2033	58	157
2033	89	12582
2033	97	150
2033	227	1246
2033	228	1279
2033	229	711
2033	288	8887
2033	289	11686
2033	292	1978
2033	295	551
2033	298	616
2033	300	1736
2033	302	180
2033	306	228
2033	324	163
2033	333	1159
2033	424	459
2033	466	272
2033	498	3083
2033	526	607
2033	530	697
2033	538	262
2033	548	159
2033	599	199
2033	689	205

2033	690	856
2033	703	234
2033	704	419
2033	889	347
2033	901	243
2033	903	1116
2033	980	326
2033	985	148
2033	1246	295
2033	1379	2345
2033	1632	1093
2033	1934	1234
2033	2137	197
2033	2521	1804
2033	2602	213
2033	3200	175
2033	3404	607
2033	4814	1156
2033	5147	253
2033	5149	385
2033	5926	220
2033	7781	310
2033	7787	177
2033	8589	243
2034	67	213
2034	72	623
2034	89	359
2034	154	606
2034	173	299
2034	190	765

2034	196	174
2034	207	303
2034	217	159
2034	225	715
2034	227	776
2034	228	377
2034	229	907
2034	298	131
2034	300	293
2034	416	1104
2034	421	321
2034	424	505
2034	428	292
2034	432	358
2034	439	1085
2034	441	230
2034	486	326
2034	533	155
2034	689	208
2034	857	161
2034	863	131
2034	903	170
2034	1048	466
2034	1090	170
2034	1243	283
2034	1384	358
2034	1400	225
2034	1424	648
2034	1510	1614
2034	1632	228

2034	1755	225
2034	1766	503
2034	2014	155
2034	2277	218
2034	2524	164
2034	2815	250
2034	3057	304
2034	3400	271
2034	3676	247
2034	3770	157
2034	4452	197
2034	4459	134
2034	7657	135
2034	10618	142
2035	96	107
2035	212	120
2035	612	108
2035	717	131
2035	733	83
2035	863	64
2035	1242	71
2035	1414	79
2035	1732	66
2035	1804	70
2035	1964	202
2035	2282	87
2035	2541	77
2035	2605	130
2035	3700	74
2035	5416	63

2035	5879	68
2035	6910	150
2035	7935	260
2035	8314	74
2035	9424	61
2035	9542	65
2035	9545	119
2035	10113	117
2035	10612	84
2035	10766	95
2035	12599	141
2035	13038	79
2035	13052	71
2035	13053	124
2035	13950	82
2035	14385	82
2035	14674	80
2035	18336	586
2035	18337	472
2035	18338	358
2035	18339	258
2035	18340	174
2035	18341	114
2035	18342	114
2035	18343	105
2035	18344	97
2035	18345	94
2035	18346	93
2035	18347	90
2035	18348	89

2035	18349	74
2035	18350	70
2035	18351	69
2035	18352	67
2036	52	95
2036	88	127
2036	206	104
2036	227	104
2036	263	222
2036	599	212
2036	601	311
2036	610	99
2036	633	134
2036	1095	265
2036	1136	170
2036	1729	119
2036	1755	290
2036	2330	253
2036	2419	242
2036	2711	108
2036	3057	293
2036	3130	192
2036	3462	341
2036	3776	136
2036	3854	126
2036	3905	181
2036	3920	451
2036	4153	556
2036	5250	397
2036	6462	206

2036	7007	247
2036	7124	226
2036	7623	101
2036	8383	140
2036	10891	260
2036	10912	196
2036	11477	100
2036	15556	135
2036	17048	314
2036	17962	150
2036	18353	354
2036	18354	317
2036	18355	298
2036	18356	294
2036	18357	288
2036	18358	226
2036	18359	180
2036	18360	145
2036	18361	133
2036	18362	128
2036	18363	123
2036	18364	109
2036	18365	99
2036	18366	98
2037	67	2536
2037	69	335
2037	97	732
2037	155	106
2037	157	60
2037	190	71

2037	241 85
2037	257 821
2037	262 934
2037	285 239
2037	288 1129
2037	292 411
2037	294 262
2037	295 316
2037	296 122
2037	298 80
2037	299 1057
2037	300 715
2037	301 111
2037	302 548
2037	306 130
2037	308 54
2037	310 193
2037	314 93
2037	317 58
2037	331 161
2037	332 51
2037	344 61
2037	352 166
2037	466 94
2037	468 56
2037	475 108
2037	525 147
2037	535 289
2037	543 135
2037	701 327

2037	900	108
2037	903	52
2037	1032	141
2037	1037	191
2037	1379	293
2037	1452	70
2037	1601	317
2037	1613	42
2037	1934	1457
2037	1936	91
2037	3046	44
2037	3502	44
2037	4814	128
2037	6226	50
2038	56	1272
2038	89	1443
2038	190	123
2038	228	199
2038	229	184
2038	230	177
2038	234	249
2038	288	1485
2038	289	461
2038	292	227
2038	295	199
2038	298	266
2038	300	2654
2038	302	296
2038	306	134
2038	310	166

2038	328	125
2038	333	382
2038	352	157
2038	430	172
2038	461	250
2038	464	318
2038	466	497
2038	470	1042
2038	498	853
2038	526	220
2038	528	351
2038	539	130
2038	544	188
2038	679	1745
2038	689	514
2038	691	137
2038	701	308
2038	703	387
2038	704	250
2038	706	121
2038	757	167
2038	1032	454
2038	1042	192
2038	1243	124
2038	1246	492
2038	1390	206
2038	1398	125
2038	1400	221
2038	1470	267
2038	1518	148

2038	1556	147
2038	1934	125
2038	1976	231
2038	2025	209
2039	65	940
2039	67	2031
2039	72	279
2039	90	1085
2039	154	382
2039	171	547
2039	173	772
2039	182	528
2039	184	359
2039	228	428
2039	238	268
2039	301	812
2039	310	296
2039	316	2536
2039	498	338
2039	511	475
2039	538	429
2039	614	605
2039	689	633
2039	697	999
2039	715	843
2039	777	346
2039	885	1792
2039	900	481
2039	913	351
2039	969	465

2039	1098	306
2039	1099	336
2039	1375	387
2039	1384	430
2039	1394	979
2039	3101	268
2039	3104	568
2039	3460	476
2039	3466	333
2039	3676	311
2039	3748	329
2039	3992	375
2039	4036	529
2039	4074	641
2039	5072	299
2039	5790	796
2039	7157	691
2039	7279	529
2039	8479	460
2039	8642	551
2039	10069	607
2039	12352	322
2039	18367	362
2039	18368	281
2040	55	96
2040	65	203
2040	89	599
2040	157	365
2040	172	652
2040	190	271

2040	217	78
2040	227	119
2040	228	145
2040	289	439
2040	291	97
2040	293	80
2040	300	103
2040	344	1975
2040	425	75
2040	437	70
2040	461	556
2040	466	164
2040	498	152
2040	532	142
2040	538	160
2040	548	152
2040	622	75
2040	679	292
2040	680	80
2040	681	328
2040	682	105
2040	686	369
2040	689	168
2040	704	161
2040	886	103
2040	906	128
2040	962	159
2040	1105	178
2040	1375	236
2040	1378	267

2040	1470	72
2040	1632	138
2040	2977	182
2040	3110	177
2040	3206	70
2040	3404	64
2040	5035	92
2040	5926	86
2040	6298	444
2040	6693	68
2040	7194	86
2040	9578	80
2040	11155	74
2040	18369	159
2041	67	464
2041	89	467
2041	154	279
2041	159	183
2041	190	213
2041	209	1026
2041	220	198
2041	230	178
2041	333	317
2041	377	473
2041	378	422
2041	396	428
2041	403	3832
2041	405	384
2041	488	777
2041	498	975

2041	511 3826	
2041	540 177	
2041	687 341	
2041	972 297	
2041	982 209	
2041	1046	541
2041	1092	252
2041	1180	988
2041	1181	566
2041	1185	259
2041	1186	327
2041	1189	812
2041	1194	329
2041	1198	199
2041	1246	281
2041	1380	175
2041	1502	310
2041	1866	212
2041	2839	1078
2041	2840	210
2041	3084	196
2041	3302	375
2041	3427	329
2041	4262	225
2041	4741	190
2041	4756	219
2041	5140	285
2041	5313	2430
2041	6558	181
2041	7837	210

2041	10318	597
2041	13600	447
2041	15218	302
2041	18370	221
2042	65	191
2042	163	176
2042	176	238
2042	378	272
2042	380	184
2042	503	429
2042	533	2314
2042	614	1368
2042	705	1336
2042	706	656
2042	707	1503
2042	714	290
2042	715	252
2042	726	221
2042	810	228
2042	837	1280
2042	860	231
2042	930	937
2042	957	562
2042	966	443
2042	1145	261
2042	1241	607
2042	1339	386
2042	1415	231
2042	1470	903
2042	1639	211

2042	1660	783
2042	1662	711
2042	1778	1048
2042	1905	452
2042	2014	377
2042	2062	236
2042	2346	299
2042	2352	379
2042	2794	249
2042	2941	475
2042	3051	188
2042	3427	1283
2042	3758	235
2042	3859	280
2042	3946	279
2042	4177	306
2042	4179	579
2042	4439	307
2042	4849	203
2042	5453	1042
2042	5454	343
2042	6249	360
2042	7322	193
2042	10851	235
2043	217 509	
2043	222 687	
2043	412 458	
2043	471 595	
2043	507 458	
2043	691 383	

2043	770 384	
2043	1035	366
2043	1046	367
2043	1096	423
2043	1100	797
2043	1171	420
2043	1181	547
2043	1185	284
2043	1187	906
2043	1197	748
2043	1201	306
2043	1541	404
2043	1868	470
2043	2270	274
2043	2572	3392
2043	2574	352
2043	2617	489
2043	2643	1335
2043	2850	952
2043	3398	426
2043	3616	6123
2043	3705	858
2043	3707	425
2043	3714	283
2043	3721	289
2043	3793	270
2043	4104	533
2043	4262	423
2043	4427	496
2043	5966	1854

2043	6191	258
2043	6294	347
2043	6463	596
2043	6769	358
2043	6772	625
2043	6773	691
2043	8459	752
2043	11412	441
2043	11548	311
2043	11623	1153
2043	11979	2100
2043	12980	484
2043	13526	447
2043	15055	833
2044	65	43
2044	154	134
2044	197	401
2044	199	39
2044	206	40
2044	215	104
2044	217	50
2044	219	142
2044	233	27
2044	418	38
2044	423	625
2044	433	60
2044	445	208
2044	565	23
2044	573	104
2044	602	28

2044	615	36
2044	621	318
2044	622	62
2044	697	39
2044	703	26
2044	869	36
2044	961	32
2044	982	31
2044	1098	37
2044	1424	53
2044	1633	41
2044	1718	255
2044	1833	29
2044	2408	31
2044	2469	25
2044	3117	40
2044	4051	26
2044	4079	95
2044	4153	292
2044	4532	27
2044	5890	102
2044	5914	52
2044	6204	304
2044	6213	31
2044	8706	96
2044	9825	65
2044	13454	29
2044	18371	570
2044	18372	148
2044	18373	58

2044	18374	40
2044	18375	36
2044	18376	36
2044	18377	26
2045	163	639
2045	166	41
2045	207	322
2045	208	48
2045	234	181
2045	424	430
2045	428	42
2045	460	519
2045	508	58
2045	757	39
2045	868	246
2045	957	90
2045	1090	262
2045	1400	62
2045	1786	202
2045	1788	293
2045	1796	153
2045	2055	179
2045	2131	44
2045	2582	41
2045	2743	55
2045	3046	1728
2045	3053	235
2045	3056	944
2045	3830	195
2045	3833	660

2045	3840	165
2045	4821	68
2045	4887	255
2045	4901	89
2045	4906	64
2045	6242	165
2045	6245	453
2045	7008	645
2045	10435	424
2045	11170	139
2045	13854	2138
2045	14128	460
2045	14693	1685
2045	17138	39
2045	18378	706
2045	18379	471
2045	18380	337
2045	18381	236
2045	18382	166
2045	18383	127
2045	18384	105
2045	18385	102
2045	18386	64
2045	18387	45
2046	7	1752
2046	9	1941
2046	13	539
2046	25	113
2046	30	1096
2046	34	155

2046	36	741
2046	190	1109
2046	198	401
2046	233	870
2046	234	138
2046	377	220
2046	450	225
2046	504	712
2046	517	207
2046	518	375
2046	522	2914
2046	707	528
2046	726	666
2046	923	121
2046	930	1288
2046	953	610
2046	955	269
2046	1044	1540
2046	1091	307
2046	1104	11504
2046	1109	233
2046	1113	438
2046	1131	239
2046	1145	185
2046	1300	564
2046	1672	242
2046	1704	396
2046	1711	115
2046	1714	2881
2046	2014	690

2046	2038	141
2046	2069	1486
2046	2972	190
2046	3184	188
2046	3239	203
2046	3244	1661
2046	5079	193
2046	5653	110
2046	6252	250
2046	6257	730
2046	6875	480
2046	18388	378
2046	18389	167
2046	18390	113
2047	52	157
2047	53	134
2047	63	1044
2047	66	122
2047	70	219
2047	72	1072
2047	73	224
2047	151	101
2047	193	202
2047	233	334
2047	238	284
2047	433	107
2047	447	247
2047	599	101
2047	603	278
2047	744	459

2047	748 503	
2047	782 275	
2047	920 313	
2047	961 633	
2047	1001	136
2047	1083	170
2047	1092	107
2047	1098	165
2047	1156	180
2047	1534	187
2047	1703	187
2047	1713	212
2047	1801	112
2047	1824	242
2047	2605	164
2047	2669	159
2047	2681	454
2047	2716	106
2047	3280	530
2047	3307	135
2047	3413	371
2047	3892	313
2047	4113	145
2047	5237	192
2047	8042	107
2047	9146	114
2047	11897	253
2047	12002	114
2047	13669	246
2047	13700	106

2047	18391	506
2047	18392	186
2047	18393	138
2047	18394	107
2048	69	15
2048	193	17
2048	265	16
2048	599	10
2048	1178	11
2048	1447	14
2048	1462	17
2048	1772	20
2048	2044	25
2048	3264	12
2048	3777	12
2048	3859	14
2048	4379	16
2048	4620	16
2048	5452	17
2048	5787	31
2048	6571	11
2048	6710	10
2048	7059	13
2048	7352	14
2048	8152	22
2048	8266	18
2048	8658	24
2048	8803	11
2048	9190	12
2048	9716	22

2048	10412	21
2048	12351	16
2048	16333	11
2048	17646	12
2048	18395	22
2048	18396	20
2048	18397	18
2048	18398	18
2048	18399	17
2048	18400	17
2048	18401	16
2048	18402	16
2048	18403	15
2048	18404	14
2048	18405	14
2048	18406	12
2048	18407	12
2048	18408	11
2048	18409	11
2048	18410	11
2048	18411	11
2048	18412	11
2048	18413	11
2048	18414	11
2051	154 24	
2051	159 26	
2051	211 2	
2051	216 1	
2051	233 1	
2051	234 1	

2051	238 2	
2051	441 1	
2051	599 2	
2051	632 2	
2051	755 2	
2051	879 1	
2051	885 1	
2051	1098	1
2051	1131	1
2051	1424	2
2051	1504	1
2051	1718	1
2051	1888	1
2051	1957	1
2051	2289	1
2051	2542	1
2051	2616	2
2051	2629	1
2051	3119	1
2051	3259	2
2051	3280	2
2051	3290	2
2051	3291	2
2051	3292	2
2051	3299	1
2051	5620	2
2051	6407	1
2051	6708	2
2051	7626	1
2051	9324	1

2051	10515	2
2051	11489	2
2051	11492	2
2051	11598	1
2051	18424	72
2051	18425	6
2051	18426	4
2051	18427	3
2051	18428	3
2051	18429	3
2051	18430	2
2051	18431	2
2051	18432	1
2051	18433	1
2052	68	1747
2052	72	2113
2052	93	1696
2052	96	966
2052	159	2729
2052	163	13002
2052	187	1047
2052	192	4288
2052	227	9301
2052	599	2345
2052	712	1055
2052	717	26284
2052	959	1012
2052	994	982
2052	998	1560
2052	1358	3151

2052	1372	1233
2052	1415	1517
2052	1891	906
2052	1892	1530
2052	1893	1807
2052	2013	4817
2052	2222	926
2052	2347	1074
2052	2444	1665
2052	2554	2860
2052	2861	999
2052	2936	879
2052	3230	1435
2052	3304	1098
2052	3308	3535
2052	3777	1461
2052	3921	5641
2052	4126	4807
2052	4131	1118
2052	4675	10455
2052	5365	827
2052	6150	29947
2052	7054	1333
2052	8802	1336
2052	9990	775
2052	10219	1356
2052	11630	758
2052	12599	941
2052	18434	3518
2052	18435	3206

2052	18436	1735
2052	18437	1132
2052	18438	868
2052	18439	842
2053	7	248
2053	25	239
2053	67	219
2053	89	2205
2053	172	365
2053	377	268
2053	378	614
2053	475	379
2053	503	1352
2053	504	233
2053	518	1417
2053	811	593
2053	824	520
2053	837	352
2053	841	369
2053	917	780
2053	923	2841
2053	930	6619
2053	936	1403
2053	953	527
2053	955	1344
2053	972	253
2053	978	237
2053	1044	308
2053	1104	333
2053	1114	1959

2053	1339	2708
2053	1359	1047
2053	1710	365
2053	2014	414
2053	2370	226
2053	2664	710
2053	2961	410
2053	2963	256
2053	3425	557
2053	4135	429
2053	4136	593
2053	4137	393
2053	4286	382
2053	4319	431
2053	4343	399
2053	5459	455
2053	6282	843
2053	7100	235
2053	7103	358
2053	8646	356
2053	9746	238
2053	10865	292
2053	16427	472
2053	18440	215
2054	89	929
2054	288	702
2054	289	1950
2054	292	1068
2054	295	169
2054	299	97

2054	300 1130	
2054	302 138	
2054	313 181	
2054	318 105	
2054	325 1259	
2054	333 1557	
2054	342 129	
2054	344 1102	
2054	352 1058	
2054	400 97	
2054	461 1366	
2054	466 1470	
2054	498 1109	
2054	528 360	
2054	679 199	
2054	680 55	
2054	681 1019	
2054	682 202	
2054	686 28219	
2054	693 73	
2054	699 89	
2054	701 252	
2054	790 194	
2054	793 274	
2054	797 80	
2054	1032	125
2054	1034	152
2054	1037	245
2054	1452	99
2054	1453	636

2054	1458	211
2054	1459	93
2054	1673	292
2054	2020	352
2054	2081	84
2054	2091	872
2054	2092	316
2054	2543	56
2054	2802	147
2054	3326	129
2054	4814	263
2054	4879	383
2054	7864	170
2054	18441	100
2055	9	172
2055	16	221
2055	18	655
2055	32	196
2055	51	1716
2055	72	208
2055	89	1343
2055	157	301
2055	159	381
2055	172	174
2055	300	386
2055	407	999
2055	463	863
2055	467	176
2055	487	2784
2055	504	621

2055	515 425	
2055	875 1074	
2055	1000	740
2055	1001	267
2055	1066	464
2055	1069	726
2055	1083	264
2055	1534	349
2055	1810	343
2055	1886	736
2055	1914	373
2055	3183	2508
2055	3217	399
2055	3432	148
2055	3454	242
2055	4980	155
2055	5079	567
2055	5114	241
2055	5670	236
2055	6068	296
2055	9144	583
2055	15524	235
2055	16206	638
2055	16238	235
2055	18442	820
2055	18443	382
2055	18444	298
2055	18445	255
2055	18446	212
2055	18447	195

2055	18448	187
2055	18449	175
2055	18450	152
2055	18451	151
2056	55	7
2056	251	8
2056	289	7
2056	360	8
2056	378	8
2056	455	7
2056	518	28
2056	657	9
2056	701	10
2056	726	7
2056	930	15
2056	936	10
2056	945	7
2056	955	11
2056	1114	22
2056	1793	9
2056	2014	12
2056	2069	9
2056	2523	8
2056	3307	16
2056	3429	14
2056	3863	7
2056	4135	16
2056	4136	24
2056	4137	10
2056	4331	19

2056	4817	12
2056	4983	13
2056	4987	11
2056	5767	7
2056	7099	17
2056	7191	9
2056	8646	10
2056	9734	8
2056	10076	8
2056	10865	8
2056	12138	11
2056	13351	9
2056	15665	12
2056	15685	11
2056	16427	24
2056	16431	12
2056	16970	8
2056	18038	9
2056	18452	14
2056	18453	11
2056	18454	10
2056	18455	9
2056	18456	8
2056	18457	8
2057	56	524
2057	228	386
2057	229	735
2057	300	484
2057	324	283
2057	333	204

2057	344 275	
2057	362 331	
2057	412 547	
2057	437 170	
2057	466 199	
2057	471 177	
2057	488 277	
2057	499 393	
2057	536 293	
2057	722 438	
2057	728 274	
2057	730 185	
2057	734 603	
2057	757 263	
2057	891 299	
2057	898 400	
2057	1105	539
2057	1186	278
2057	1375	579
2057	1376	442
2057	1378	704
2057	1400	171
2057	1406	303
2057	1519	201
2057	1632	331
2057	1929	677
2057	1949	280
2057	2738	308
2057	3398	381
2057	3427	651

2057	3466	301
2057	3614	259
2057	3737	1223
2057	3753	806
2057	3754	1366
2057	4025	201
2057	4189	713
2057	4196	466
2057	4371	330
2057	6452	238
2057	14074	217
2057	14318	466
2057	18458	215
2057	18459	191
2058	55	320
2058	67	2622
2058	69	184
2058	71	77
2058	89	414
2058	183	62
2058	288	91
2058	289	322
2058	299	74
2058	300	191
2058	302	576
2058	306	59
2058	328	32
2058	333	41
2058	342	23
2058	349	39

2058	362 225	
2058	412 28	
2058	485 61	
2058	525 40	
2058	536 21	
2058	715 174	
2058	722 37	
2058	903 73	
2058	906 80	
2058	1009	28
2058	1076	22
2058	1081	116
2058	1380	59
2058	1406	59
2058	1463	31
2058	1576	26
2058	1604	31
2058	1613	19
2058	1984	55
2058	2446	18
2058	2829	24
2058	3500	23
2058	3502	26
2058	3861	56
2058	3951	21
2058	4257	25
2058	4309	50
2058	4565	121
2058	5787	91
2058	9932	29

2058	15574	19
2058	18460	28
2058	18461	21
2058	18462	19
2059	7	230
2059	58	552
2059	67	805
2059	89	3806
2059	157	491
2059	288	3025
2059	289	1306
2059	291	195
2059	292	47630
2059	295	626
2059	300	499
2059	301	2184
2059	302	2767
2059	306	546
2059	317	196
2059	323	437
2059	329	1831
2059	333	5392
2059	340	286
2059	349	824
2059	352	472
2059	360	3632
2059	458	571
2059	459	331
2059	461	338
2059	464	393

2059	466	1061
2059	475	4870
2059	498	10151
2059	525	1418
2059	544	682
2059	690	510
2059	697	904
2059	704	521
2059	907	218
2059	915	399
2059	1032	259
2059	1037	3921
2059	1042	165
2059	1052	1076
2059	1183	218
2059	1455	200
2059	1601	185
2059	1620	569
2059	2226	248
2059	9384	269
2059	9913	334
2059	16891	410
2059	18463	343
2059	18464	169
2060	12	122
2060	198	144
2060	370	545
2060	506	161
2060	707	166
2060	726	543

2060	812	243
2060	820	119
2060	821	131
2060	824	120
2060	835	172
2060	917	183
2060	918	136
2060	949	243
2060	954	121
2060	1131	183
2060	1271	139
2060	1276	134
2060	1356	156
2060	1358	118
2060	2143	160
2060	2370	473
2060	2374	352
2060	2765	176
2060	2961	132
2060	2963	228
2060	2966	295
2060	2970	212
2060	3416	157
2060	4615	211
2060	4971	190
2060	5689	129
2060	6289	156
2060	6798	155
2060	7044	141
2060	7115	122

2060	7941	176
2060	8247	163
2060	9222	178
2060	10091	168
2060	10956	137
2060	14892	122
2060	15814	244
2060	18465	462
2060	18466	297
2060	18467	206
2060	18468	202
2060	18469	181
2060	18470	175
2060	18471	132
2061	89	26
2061	161	24
2061	257	12
2061	265	12
2061	279	11
2061	288	995
2061	289	21
2061	291	303
2061	293	11
2061	295	348
2061	302	13
2061	308	19
2061	314	23
2061	317	46
2061	325	738
2061	326	12

2061	349	37
2061	386	1189
2061	461	21
2061	465	11
2061	484	17
2061	523	13
2061	543	12
2061	674	14
2061	681	18
2061	906	11
2061	908	22
2061	1444	29
2061	1449	12
2061	1459	12
2061	1684	12
2061	2018	16
2061	2498	28
2061	2801	17
2061	3491	40
2061	3499	16
2061	3570	227
2061	3572	164
2061	4560	902
2061	4769	16
2061	5683	44
2061	6232	13
2061	6429	20
2061	6693	14
2061	7340	19
2061	7656	22

2061	7734	12
2061	8017	29
2061	8718	24
2061	18472	32
2062	65	18544
2062	184	1472
2062	190	10673
2062	192	764
2062	204	918
2062	211	1414
2062	227	1153
2062	228	1004
2062	229	625
2062	231	789
2062	355	1431
2062	366	2665
2062	424	858
2062	538	1125
2062	540	626
2062	632	1359
2062	679	917
2062	715	866
2062	945	856
2062	1090	1890
2062	1147	1612
2062	1243	1558
2062	1246	642
2062	1351	666
2062	1377	855
2062	1464	740

2062	1491	1445
2062	1511	698
2062	1705	2094
2062	1708	1353
2062	2051	1526
2062	2176	647
2062	2632	877
2062	3081	846
2062	3105	2054
2062	3171	1331
2062	3617	2008
2062	4920	870
2062	5306	2137
2062	5437	1706
2062	5836	965
2062	6297	600
2062	6390	699
2062	7260	2064
2062	8504	1235
2062	9404	593
2062	12164	689
2062	13624	608
2062	13832	1351
2062	18473	1004
2064	88	23
2064	190	28
2064	217	68
2064	223	17
2064	225	18
2064	228	477

2064	229	41
2064	230	74
2064	302	116
2064	333	24
2064	377	203
2064	471	17
2064	486	25
2064	488	16
2064	497	120
2064	498	1130
2064	499	1458
2064	500	139
2064	540	406
2064	548	24
2064	679	141
2064	689	101
2064	690	26
2064	697	19
2064	704	243
2064	779	145
2064	830	40
2064	1043	25
2064	1048	19
2064	1052	73
2064	1151	21
2064	1188	265
2064	1510	26
2064	1632	17
2064	1854	177
2064	2062	127

2064	2200	214
2064	2839	18
2064	2959	312
2064	4074	30
2064	4435	23
2064	10132	17
2064	13147	19
2064	14889	47
2064	18511	291
2064	18512	49
2064	18513	49
2064	18514	29
2064	18515	19
2064	18516	17
2065	89	2346
2065	191	376
2065	230	1288
2065	288	1303
2065	289	2347
2065	300	851
2065	325	755
2065	328	400
2065	333	1787
2065	344	789
2065	350	639
2065	352	396
2065	367	4130
2065	371	1460
2065	374	506
2065	377	1389

2065	378 677	
2065	386 615	
2065	393 458	
2065	409 1138	
2065	461 616	
2065	464 1166	
2065	466 1650	
2065	475 581	
2065	518 303	
2065	531 1035	
2065	532 444	
2065	544 1508	
2065	545 459	
2065	682 349	
2065	686 381	
2065	691 827	
2065	701 976	
2065	834 463	
2065	1042	358
2065	1179	1018
2065	1447	364
2065	1852	355
2065	1904	1082
2065	2091	532
2065	2092	518
2065	2107	345
2065	2940	1286
2065	3859	504
2065	4558	656
2065	4562	433

2065	5575	618
2065	8047	1356
2065	13780	588
2065	18517	807
2066	2	83
2066	198	120
2066	234	130
2066	487	182
2066	488	135
2066	500	88
2066	504	963
2066	707	120
2066	726	208
2066	923	106
2066	1104	222
2066	1145	155
2066	1179	98
2066	1249	100
2066	1300	606
2066	1339	211
2066	1351	89
2066	1704	717
2066	1711	166
2066	1712	137
2066	1914	267
2066	2038	314
2066	2370	104
2066	3184	313
2066	3188	143
2066	3244	82

2066	4155	197
2066	4156	508
2066	4162	116
2066	4912	193
2066	5079	690
2066	5610	314
2066	8405	229
2066	9263	994
2066	9634	133
2066	9637	122
2066	9978	155
2066	9979	80
2066	14728	96
2066	15457	94
2066	18518	368
2066	18519	207
2066	18520	169
2066	18521	156
2066	18522	124
2066	18523	119
2066	18524	115
2066	18525	114
2066	18526	109
2066	18527	104
2067	507 175	
2067	763 230	
2067	815 187	
2067	817 139	
2067	823 210	
2067	829 143	

2067	1541	114
2067	1550	208
2067	1552	97
2067	2214	365
2067	2608	148
2067	2850	163
2067	3043	138
2067	3999	473
2067	4002	135
2067	4004	274
2067	4170	202
2067	4937	122
2067	5667	103
2067	5799	253
2067	5968	127
2067	6837	261
2067	7724	93
2067	8459	407
2067	8462	122
2067	9804	234
2067	10650	348
2067	13847	179
2067	14208	632
2067	15054	96
2067	18528	508
2067	18529	414
2067	18530	393
2067	18531	392
2067	18532	325
2067	18533	243

2067	18534	222
2067	18535	168
2067	18536	159
2067	18537	151
2067	18538	147
2067	18539	139
2067	18540	137
2067	18541	134
2067	18542	130
2067	18543	117
2067	18544	103
2067	18545	102
2067	18546	99
2067	18547	97
2068	55	1068
2068	89	1217
2068	161	580
2068	180	422
2068	228	696
2068	265	1156
2068	300	1669
2068	302	457
2068	378	434
2068	386	1275
2068	389	578
2068	518	1118
2068	535	1055
2068	637	784
2068	641	852
2068	676	799

2068	680	465
2068	696	431
2068	701	484
2068	967	546
2068	968	485
2068	972	2239
2068	1424	539
2068	1590	502
2068	2022	444
2068	2067	455
2068	2934	417
2068	3307	452
2068	3466	520
2068	3489	777
2068	3763	516
2068	3890	562
2068	4143	478
2068	4254	701
2068	6429	1728
2068	6517	553
2068	8002	416
2068	8021	418
2068	8042	543
2068	8290	563
2068	9234	576
2068	10074	642
2068	10640	697
2068	10677	559
2068	11581	627
2068	14628	497

2068	15236	480
2068	18548	1200
2068	18549	525
2068	18550	486
2069	97	116
2069	190	127
2069	207	186
2069	209	298
2069	233	260
2069	234	173
2069	292	162
2069	316	165
2069	362	964
2069	378	303
2069	428	215
2069	430	221
2069	453	150
2069	475	127
2069	486	109484
2069	499	133
2069	500	207
2069	504	147
2069	540	140
2069	680	521
2069	689	262
2069	726	151
2069	898	413
2069	1034	265
2069	1043	287
2069	1044	134

2069	1045	313
2069	1094	689
2069	1105	167
2069	1180	124
2069	1231	230
2069	1246	233
2069	1375	150
2069	1672	336
2069	2526	165
2069	2785	142
2069	3074	117
2069	3081	150
2069	3672	253
2069	3753	224
2069	5079	689
2069	6194	281
2069	6197	151
2069	6298	347
2069	6454	215
2069	11256	445
2069	16823	183
2069	18551	336
2069	18552	219
2069	18553	125
2070	7	399
2070	176	77
2070	190	142
2070	220	60
2070	227	463
2070	229	139

2070	230	216
2070	333	62
2070	377	836
2070	378	233
2070	412	140
2070	472	122
2070	486	1025
2070	488	1346
2070	498	5757
2070	501	122
2070	524	412
2070	536	58
2070	542	621
2070	724	185
2070	779	165
2070	808	76
2070	816	389
2070	951	145
2070	982	92
2070	1046	90
2070	1091	112
2070	1186	233
2070	1239	156
2070	1249	208
2070	1406	115
2070	1412	147
2070	1455	137
2070	1672	147
2070	1673	203
2070	1689	392

2070	1857	502
2070	2346	3242
2070	2347	129
2070	2595	161
2070	2788	521
2070	3333	247
2070	3735	72
2070	4309	256
2070	4822	71
2070	5934	111
2070	7328	487
2070	16891	59
2070	18554	302
2070	18555	186
2071	67	1325
2071	71	279
2071	89	268
2071	157	182
2071	161	154
2071	257	69
2071	265	304
2071	285	281
2071	289	540
2071	291	86
2071	292	2441
2071	294	296
2071	295	1212
2071	300	321
2071	301	135
2071	302	286

2071	310 861	
2071	320 83	
2071	329 295	
2071	465 91	
2071	466 74	
2071	525 286	
2071	535 308	
2071	641 72	
2071	686 134	
2071	788 256	
2071	792 324663	
2071	795 171	
2071	913 65	
2071	1430	56
2071	1453	89
2071	1475	127
2071	2023	105
2071	2064	101
2071	2390	854
2071	2391	254
2071	2395	131
2071	2396	80
2071	2462	59
2071	3498	99
2071	3503	54
2071	4183	113
2071	4697	76
2071	5249	55
2071	7846	54
2071	11084	64

2071	11219	51
2071	13743	236
2071	18556	110
2071	18557	94
2072	89	2040
2072	210	1522
2072	257	1702
2072	288	1767
2072	289	44236
2072	291	4018
2072	293	4559
2072	304	1371
2072	307	1778
2072	318	7366
2072	325	6933
2072	333	8337
2072	342	2720
2072	344	11498
2072	386	3353
2072	403	3776
2072	455	2166
2072	461	2004
2072	498	3753
2072	523	2330
2072	679	2644
2072	680	15907
2072	786	2776
2072	793	2120
2072	1021	2523
2072	1059	3561

2072	1198	1393
2072	1684	18103
2072	1782	4013
2072	1866	2119
2072	2019	3177
2072	2094	3166
2072	2546	2853
2072	3373	1523
2072	3489	12842
2072	3497	3036
2072	3575	2264
2072	5437	1445
2072	5948	2980
2072	7449	2548
2072	11718	3926
2072	13645	7964
2072	13991	1799
2072	18558	4393
2072	18559	3517
2072	18560	2712
2072	18561	2336
2072	18562	2176
2072	18563	1754
2072	18564	1374
2075	51	166
2075	55	228
2075	58	115
2075	59	159
2075	67	398
2075	72	1994

2075	77	225
2075	89	203
2075	154	141
2075	155	101
2075	157	176
2075	159	185
2075	167	351
2075	193	118
2075	202	324
2075	227	212
2075	288	105
2075	289	206
2075	291	123
2075	292	135
2075	329	101
2075	429	129
2075	441	104
2075	511	122
2075	535	139
2075	538	105
2075	599	104
2075	605	108
2075	612	120
2075	860	146
2075	889	157
2075	906	151
2075	985	148
2075	1001	138
2075	1052	99
2075	1099	107

2075	1121	171
2075	1122	120
2075	1243	164
2075	1244	261
2075	1464	112
2075	1513	218
2075	1713	184
2075	1892	130
2075	2183	138
2075	2444	108
2075	3349	153
2075	3562	239
2075	18571	142
2075	18572	115
2076	67	108
2076	89	673
2076	190	216
2076	207	88
2076	209	87
2076	227	157
2076	283	582
2076	289	348
2076	295	111
2076	298	164
2076	300	163
2076	302	117
2076	333	289
2076	349	173
2076	378	248
2076	430	94

2076	498	922
2076	542	498
2076	701	129
2076	708	113
2076	930	152
2076	955	102
2076	972	164
2076	989	137
2076	1243	120
2076	1246	186
2076	1681	393
2076	1763	166
2076	1771	177
2076	1807	89
2076	2788	155
2076	2988	91
2076	3125	173
2076	3200	203
2076	3205	84
2076	3737	99
2076	3744	181
2076	4346	85
2076	4716	91
2076	5027	207
2076	5079	338
2076	5437	100
2076	5745	147
2076	6535	321
2076	7835	102
2076	10401	151

2076	11155	93
2076	14629	97
2076	18573	126
2076	18574	118
2077	72	285
2077	89	195
2077	159	163
2077	163	1532
2077	173	511
2077	182	200
2077	198	204
2077	209	183
2077	220	158
2077	229	128
2077	234	465
2077	429	485
2077	486	299
2077	487	150
2077	498	374
2077	533	135
2077	599	586
2077	679	137
2077	706	648
2077	707	204
2077	724	208
2077	732	397
2077	854	204
2077	855	185
2077	863	195
2077	868	162

2077	918	121
2077	951	484
2077	952	360
2077	959	257
2077	972	181
2077	1104	455
2077	1180	207
2077	1246	302
2077	1249	1176
2077	1366	150
2077	1369	182
2077	1376	156
2077	1400	384
2077	1412	1022
2077	1468	152
2077	1714	578
2077	1803	1263
2077	2346	240
2077	2784	392
2077	2785	208
2077	2790	301
2077	4074	193
2077	4460	215
2077	5079	531
2078	70	617
2078	97	1253
2078	172	1061
2078	183	674
2078	220	2556
2078	226	601

2078	234 972	
2078	405 939	
2078	421 668	
2078	439 2225	
2078	489 1303	
2078	510 796	
2078	517 611	
2078	533 4657	
2078	705 2052	
2078	710 594	
2078	959 1153	
2078	962 1436	
2078	1062	825
2078	1098	2252
2078	1104	1381
2078	1274	682
2078	1632	1384
2078	1681	1201
2078	1975	1024
2078	1979	753
2078	2372	1426
2078	2398	1230
2078	2842	693
2078	3169	916
2078	3719	859
2078	3763	1025
2078	4260	775
2078	5446	1478
2078	5453	759
2078	7352	738

2078	8707	1301
2078	8708	1148
2078	13291	606
2078	13351	6628
2078	15215	1431
2078	15915	1016
2078	16096	706
2078	18575	4444
2078	18576	2249
2078	18577	1799
2078	18578	1715
2078	18579	1251
2078	18580	820
2078	18581	680
2079	45	339
2079	89	474
2079	288	210
2079	289	1709
2079	293	231
2079	300	519
2079	324	142
2079	333	568
2079	344	3556
2079	360	136
2079	456	821
2079	458	1500
2079	461	257
2079	465	139
2079	466	173
2079	471	282

2079 498 188

2079 536 154

2079 686 380

2079 692 281

2079 704 255

2079 786 380

2079 793 296

2079 1038 219

2079 1043 266

2079 1059 273

2079 1105 454

2079 1201 207

2079 1453 191

2079 1459 259

2079 1462 263

2079 2094 254

2079 2392 535

2079 2427 140

2079 2432 144

2079 2977 633

2079 2980 145

2079 2982 152

2079 3321 173

2079 3577 274

2079 3584 421

2079 4223 444

2079 5266 141

2079 6298 793

2079 6453 245

2079 6597 160

2079	6869	902
2079	18582	298
2079	18583	210
2079	18584	160
2080	53	2087
2080	58	448
2080	59	1782
2080	72	80721
2080	75	840
2080	81	4869
2080	98	358
2080	238	840
2080	429	531
2080	441	968
2080	603	559
2080	616	719
2080	748	3384
2080	880	1226
2080	997	749
2080	1014	265
2080	1019	303
2080	1061	915
2080	1067	314
2080	1072	628
2080	1073	1115
2080	1156	277
2080	1376	580
2080	1707	1174
2080	1713	6559
2080	1827	321

2080	1836	787
2080	1888	496
2080	1893	1627
2080	2039	630
2080	2043	408
2080	2673	569
2080	2716	608
2080	3289	770
2080	4308	344
2080	5833	704
2080	6137	273
2080	6150	1313
2080	6583	618
2080	7470	1918
2080	11794	604
2080	17331	430
2080	18585	744
2080	18586	473
2080	18587	431
2080	18588	308
2080	18589	292
2080	18590	289
2080	18591	267
2080	18592	265
2081	204 4640	
2081	212 338	
2081	215 746	
2081	228 238	
2081	229 554	
2081	231 526	

2081	234	353
2081	301	186
2081	355	220
2081	366	1214
2081	426	205
2081	430	952
2081	445	258
2081	602	211
2081	609	396
2081	683	173
2081	689	590
2081	733	174
2081	981	411
2081	982	510
2081	1048	1481
2081	1249	175
2081	1390	207
2081	1400	3834
2081	1412	409
2081	1424	2181
2081	1609	185
2081	1772	822
2081	1875	618
2081	2612	642
2081	2614	470
2081	2786	277
2081	3468	259
2081	3618	359
2081	4064	258
2081	4082	202

2081	4448	486
2081	5428	256
2081	6374	248
2081	8053	385
2081	8054	258
2081	8649	411
2081	8716	362
2081	10748	205
2081	12989	169
2081	13000	645
2081	14645	277
2081	18593	189
2081	18594	176
2081	18595	174
2083	72	16
2083	81	11
2083	154	12
2083	155	36
2083	157	36
2083	159	16
2083	227	38
2083	234	12
2083	306	13
2083	554	17
2083	562	27
2083	706	17
2083	715	46
2083	857	41
2083	959	13
2083	1001	94

2083	1014	17
2083	1019	12
2083	1064	18
2083	1073	12
2083	1104	17
2083	1249	17
2083	1704	13
2083	1802	12
2083	1892	20
2083	1900	13
2083	2524	31
2083	4155	23
2083	4251	19
2083	4620	26
2083	5559	13
2083	6200	59
2083	7324	18
2083	11505	160
2083	11602	13
2083	13279	12
2083	13709	14
2083	15722	20
2083	18599	81
2083	18600	20
2083	18601	20
2083	18602	18
2083	18603	17
2083	18604	16
2083	18605	16
2083	18606	13

2083	18607	13
2083	18608	13
2083	18609	12
2083	18610	11
2084	65	279
2084	154	810
2084	173	686
2084	190	2703
2084	199	308
2084	207	12498
2084	208	1228
2084	210	278
2084	225	2140
2084	226	664
2084	227	438
2084	228	722
2084	229	920
2084	230	228
2084	424	2751
2084	425	267
2084	428	1116
2084	439	1293
2084	441	514
2084	533	261
2084	932	316
2084	982	361
2084	1048	944
2084	1090	1519
2084	1239	255
2084	1512	267

2084	1513	373
2084	1515	976
2084	1517	253
2084	1520	327
2084	1664	512
2084	1669	830
2084	1766	284
2084	3200	288
2084	3400	319
2084	3747	392
2084	4452	248
2084	4459	603
2084	5030	249
2084	6726	236
2084	8708	253
2084	8710	2015
2084	8713	2793
2084	9230	237
2084	10617	1363
2084	14271	371
2084	18611	503
2084	18612	423
2084	18613	266
2084	18614	253
2085	18615	4
2086	154 357	
2086	170 177	
2086	173 271	
2086	210 100	
2086	220 92	

2086	227	124
2086	420	140
2086	425	192
2086	432	129
2086	539	164
2086	709	390
2086	757	331
2086	964	125
2086	969	320
2086	1075	556
2086	1097	99
2086	1101	151
2086	1109	105
2086	1155	117
2086	1191	352
2086	1379	149
2086	1400	112
2086	1964	179
2086	1977	251
2086	2587	141
2086	2600	109
2086	2821	479
2086	3109	84
2086	3370	130
2086	3475	89
2086	3692	1061
2086	3700	117
2086	3767	86
2086	4089	98
2086	4997	84

2086	5984	100
2086	6216	164
2086	7194	91
2086	8642	195
2086	11819	133
2086	12712	370
2086	13007	162
2086	14640	161
2086	18087	151
2086	18616	195
2086	18617	125
2086	18618	123
2086	18619	118
2086	18620	117
2086	18621	90
2087	55	161
2087	65	63
2087	67	174
2087	89	1042
2087	180	176
2087	209	138
2087	257	445
2087	265	288
2087	288	725
2087	289	1356
2087	291	203
2087	292	1949
2087	294	72
2087	295	4977
2087	299	362

2087	300 337	
2087	301 126	
2087	302 527	
2087	305 54	
2087	306 62	
2087	314 127	
2087	332 82	
2087	333 532	
2087	342 268	
2087	344 216	
2087	377 625	
2087	378 492	
2087	461 105	
2087	466 158	
2087	486 1026	
2087	498 145	
2087	543 582	
2087	544 212	
2087	679 508	
2087	701 499	
2087	913 423	
2087	915 233	
2087	1032	66
2087	1034	331
2087	1042	64
2087	1246	141
2087	1379	254
2087	1452	59
2087	1456	95
2087	1470	126

2087	1934	218
2087	1936	61
2087	2977	146
2087	4814	284
2087	4920	79
2088	45	6
2088	55	8
2088	89	12
2088	157	21
2088	176	5
2088	207	15
2088	228	14
2088	234	9
2088	262	6
2088	279	6
2088	285	9
2088	288	14
2088	295	9
2088	299	12
2088	300	9
2088	308	10
2088	309	9
2088	312	6
2088	314	12
2088	317	6
2088	320	10
2088	326	15
2088	332	9
2088	349	14
2088	516	5

2088	517 5	
2088	554 5	
2088	630 6	
2088	913 5	
2088	959 5	
2088	1426	5
2088	1451	10
2088	1580	11
2088	1639	9
2088	1935	11
2088	2569	6
2088	2786	13
2088	2834	8
2088	2899	5
2088	3314	6
2088	4566	7
2088	4713	7
2088	5031	10
2088	6288	9
2088	6541	6
2088	6562	6
2088	7639	6
2088	9426	6
2088	9636	6
2088	18622	12
2090	233 432	
2090	234 356	
2090	370 803	
2090	441 446	
2090	450 532	

2090	707	775
2090	858	503
2090	868	650
2090	872	561
2090	875	445
2090	881	484
2090	883	431
2090	1099	579
2090	1202	492
2090	1231	356
2090	1534	424
2090	1888	699
2090	2369	732
2090	2381	382
2090	2471	491
2090	3333	407
2090	3455	391
2090	3809	504
2090	5658	532
2090	8065	482
2090	8333	438
2090	8492	635
2090	8555	342
2090	9242	378
2090	9783	328
2090	9921	603
2090	10403	416
2090	10785	369
2090	11754	637
2090	13347	1062

2090	13757	595
2090	13819	529
2090	14501	435
2090	15606	417
2090	18444	637
2090	18630	1521
2090	18631	932
2090	18632	827
2090	18633	514
2090	18634	464
2090	18635	458
2090	18636	414
2090	18637	351
2090	18638	350
2090	18639	339
2091	56	50
2091	81	146
2091	330	66
2091	468	26
2091	972	58
2091	1401	41
2091	1433	108
2091	1449	39
2091	1457	20
2091	2222	47
2091	2236	33
2091	3670	30
2091	3985	26
2091	4316	235
2091	4344	56

2091	4586	72
2091	5119	13
2091	5891	101
2091	6288	162
2091	6626	69
2091	7766	15
2091	8159	15
2091	8160	28
2091	9830	29
2091	12653	61
2091	13303	34
2091	14739	27
2091	14773	30
2091	15102	37
2091	15923	63
2091	16083	24
2091	16694	47
2091	16695	28
2091	16696	28
2091	16697	33
2091	18640	49
2091	18641	46
2091	18642	35
2091	18643	31
2091	18644	24
2091	18645	23
2091	18646	22
2091	18647	21
2091	18648	20
2091	18649	20

2091	18650	19
2091	18651	18
2091	18652	15
2091	18653	14
2091	18654	13
2092	173 917	
2092	195 429	
2092	196 1671	
2092	199 764	
2092	204 279	
2092	207 10370	
2092	217 636	
2092	222 1313	
2092	225 1337	
2092	227 344	
2092	230 154	
2092	234 849	
2092	424 563	
2092	425 839	
2092	427 445	
2092	428 154	
2092	436 326	
2092	439 259	
2092	609 158	
2092	622 280	
2092	709 183	
2092	714 258	
2092	1048	1437
2092	1239	249
2092	1375	923

2092	1390	218
2092	1424	205
2092	1510	538
2092	1512	595
2092	1515	1636
2092	1766	147
2092	1971	762
2092	2131	487
2092	2279	237
2092	2600	161
2092	3057	271
2092	3106	583
2092	3205	376
2092	3258	1084
2092	3259	210
2092	3475	207
2092	3693	490
2092	5233	406
2092	5429	236
2092	6296	189
2092	7190	212
2092	7663	410
2092	9545	223
2092	11550	734
2092	18655	172
2093	96	131
2093	176	164
2093	554	89
2093	607	135
2093	706	178

2093	718 97	
2093	724 274	
2093	728 86	
2093	730 88	
2093	733 90	
2093	735 273	
2093	868 118	
2093	917 110	
2093	959 99	
2093	1244	214
2093	1360	246
2093	1369	310
2093	1372	431
2093	1415	241
2093	1426	136
2093	1639	264
2093	2342	137
2093	2343	89
2093	2346	93
2093	2742	120
2093	2786	192
2093	2787	144
2093	2834	177
2093	2853	248
2093	2855	140
2093	2871	102
2093	2893	153
2093	2899	146
2093	3470	107
2093	3774	196

2093	3888	212
2093	4251	136
2093	4369	90
2093	4541	111
2093	5168	93
2093	5844	114
2093	6165	249
2093	8111	163
2093	8122	91
2093	8794	153
2093	9347	93
2093	9926	93
2093	10412	108
2093	18656	121
2093	18657	87
2094	89	951
2094	289	897
2094	291	1159
2094	300	1528
2094	342	1343
2094	344	3902
2094	356	2061
2094	364	3417
2094	371	2726
2094	458	1304
2094	461	1217
2094	464	1528
2094	466	1064
2094	538	1130
2094	679	1067

2094	691 1519	
2094	1447	4283
2094	1459	26090
2094	1462	1469
2094	2436	1245
2094	2439	1433
2094	2443	1914
2094	2451	1046
2094	2607	1580
2094	2661	1032
2094	2977	967
2094	3081	1706
2094	3584	1216
2094	3587	1095
2094	3859	911
2094	5032	4786
2094	5099	1728
2094	5266	1197
2094	5649	1113
2094	5651	1609
2094	8738	1038
2094	9400	1236
2094	12683	875
2094	12686	1782
2094	14035	1451
2094	14043	1424
2094	17875	902
2094	18658	2013
2094	18659	2008
2094	18660	1360

2094	18661	1332
2094	18662	1191
2094	18663	1186
2094	18664	1161
2094	18665	1146
2095	1109	241
2095	1126	85
2095	2312	158
2095	2331	46
2095	2952	193
2095	4972	148
2095	6961	548
2095	8425	51
2095	8436	45
2095	8439	114
2095	8445	285
2095	8452	113
2095	8699	58
2095	9528	32
2095	11676	43
2095	12414	40
2095	16122	142
2095	17982	76
2095	18666	263
2095	18667	161
2095	18668	161
2095	18669	144
2095	18670	116
2095	18671	110
2095	18672	110

2095	18673	97
2095	18674	95
2095	18675	85
2095	18676	83
2095	18677	82
2095	18678	75
2095	18679	73
2095	18680	70
2095	18681	69
2095	18682	62
2095	18683	58
2095	18684	57
2095	18685	56
2095	18686	47
2095	18687	46
2095	18688	43
2095	18689	38
2095	18690	37
2095	18691	37
2095	18692	34
2095	18693	33
2095	18694	32
2095	18695	31
2095	18696	31
2095	18697	31
2096	88	336
2096	154	2056
2096	163	982
2096	166	391
2096	172	578

2096	173 5190	
2096	187 494	
2096	190 3605	
2096	197 1928	
2096	198 412	
2096	203 1572	
2096	208 1079	
2096	220 427	
2096	234 735	
2096	237 481	
2096	436 14690	
2096	441 469	
2096	504 915	
2096	517 386	
2096	533 1304	
2096	620 762	
2096	715 355	
2096	755 709	
2096	851 546	
2096	859 992	
2096	867 1441	
2096	1075	2131
2096	1090	911
2096	1097	917
2096	1384	422
2096	1504	977
2096	1510	1671
2096	2346	1332
2096	3109	1884
2096	3300	374

2096	3501	479
2096	3739	756
2096	3767	337
2096	3902	443
2096	4051	374
2096	4115	732
2096	4311	999
2096	6187	738
2096	6556	376
2096	6730	510
2096	7871	815
2096	13664	489
2096	17934	437
2096	18303	487
2096	18698	1288
2097	56	127
2097	59	142
2097	72	2628
2097	81	181
2097	154	346
2097	159	337
2097	163	126
2097	166	119
2097	199	137
2097	203	110
2097	206	267
2097	207	914
2097	217	108
2097	221	107
2097	223	113

2097	225 631	
2097	226 224	
2097	228 107	
2097	238 154	
2097	416 120	
2097	424 144	
2097	432 201	
2097	434 281	
2097	441 139	
2097	511 109	
2097	533 145	
2097	614 125	
2097	620 134	
2097	709 601	
2097	859 145	
2097	982 225	
2097	1106	343
2097	1151	182
2097	1376	123
2097	1394	152
2097	1412	535
2097	1510	136
2097	1976	153
2097	2137	110
2097	2582	190
2097	2821	391
2097	3475	430
2097	3892	220
2097	4074	142
2097	4435	128

2097	5154	163
2097	5237	841
2097	10385	127
2097	12918	109
2097	18026	108
2099	138 104	
2099	407 193	
2099	748 72	
2099	1835	446
2099	1943	410
2099	1945	288
2099	1969	290
2099	2605	397
2099	2668	521
2099	2811	269
2099	3132	188
2099	3346	250
2099	3352	109
2099	3823	331
2099	3995	110
2099	4122	89
2099	4852	100
2099	4853	115
2099	5191	219
2099	6004	107
2099	6046	770
2099	6336	314
2099	7060	95
2099	8232	110
2099	11377	113

2099	12868	118
2099	13816	102
2099	14230	122
2099	16215	97
2099	17294	150
2099	17956	123
2099	18706	679
2099	18707	256
2099	18708	240
2099	18709	231
2099	18710	192
2099	18711	191
2099	18712	182
2099	18713	171
2099	18714	163
2099	18715	139
2099	18716	133
2099	18717	132
2099	18718	132
2099	18719	118
2099	18720	112
2099	18721	108
2099	18722	107
2099	18723	87
2099	18724	84
2100	3	408
2100	6	404
2100	12	1253
2100	28	911
2100	47	305

2100	837 762	
2100	841 553	
2100	1109	1333
2100	1111	1062
2100	1134	1213
2100	1260	1459
2100	1276	1133
2100	1281	573
2100	2749	532
2100	2765	457
2100	2772	485
2100	2774	436
2100	3806	389
2100	4182	258
2100	4271	2026
2100	4611	360
2100	4970	614
2100	6258	403
2100	6658	731
2100	7918	289
2100	8320	284
2100	8322	650
2100	8323	456
2100	8324	1068
2100	8326	626
2100	8327	613
2100	8332	655
2100	8344	640
2100	8525	232
2100	8529	429

2100	8531	607
2100	8533	724
2100	9783	793
2100	10008	228
2100	10894	705
2100	13677	278
2100	13679	346
2100	13978	535
2100	16437	443
2100	18725	758
2100	18726	337
2100	18727	297
2100	18728	281
2100	18729	280
2100	18730	263
